# Anti-inflammatories in Alzheimer’s disease—potential therapy or spurious correlate?

**DOI:** 10.1093/braincomms/fcaa109

**Published:** 2020-07-24

**Authors:** Jack Rivers-Auty, Alison E Mather, Ruth Peters, Catherine B Lawrence, David Brough

**Affiliations:** f1 Division of Neuroscience and Experimental Psychology, Faculty of Biology, Medicine and Health, School of Biological Sciences, Manchester Academic Health Science Centre, University of Manchester, Manchester M13 9PT, UK; f2 Lydia Becker Institute of Immunology and Inflammation, University of Manchester, Manchester M13 9PT, UK; f3 Medical Sciences, Tasmanian School of Medicine, College of Health and Medicine, University of Tasmania, Hobart 7000, Australia; f4 Quadram Institute Bioscience, Norwich, Norfolk NR4 7UA, UK; f5 University of East Anglia, Norwich, Norfolk NR4 7TJ, UK; f6 School of Psychology, University of New South Wales, Sydney, Australia; f7 Neuroscience Research Australia, Sydney 2031, Australia

**Keywords:** Alzheimer’s disease, NSAID, inflammation, progression, anti-inflammatory

## Abstract

Epidemiological evidence suggests non-steroidal anti-inflammatory drugs reduce the risk of Alzheimer’s disease. However, clinical trials have found no evidence of non-steroidal anti-inflammatory drug efficacy. This incongruence may be due to the wrong non-steroidal anti-inflammatory drugs being tested in robust clinical trials or the epidemiological findings being caused by confounding factors. Therefore, this study used logistic regression and the innovative approach of negative binomial generalized linear mixed modelling to investigate both prevalence and cognitive decline, respectively, in the Alzheimer’s Disease Neuroimaging dataset for each commonly used non-steroidal anti-inflammatory drug and paracetamol. Use of most non-steroidal anti-inflammatories was associated with reduced Alzheimer’s disease prevalence yet no effect on cognitive decline was observed. Paracetamol had a similar effect on prevalence to these non-steroidal anti-inflammatory drugs suggesting this association is independent of the anti-inflammatory effects and that previous results may be due to spurious associations. Interestingly, diclofenac use was significantly associated with both reduce incidence and slower cognitive decline warranting further research into the potential therapeutic effects of diclofenac in Alzheimer’s disease.

## Teaser

The therapeutic potential of non-steroidal anti-inflammatory drugs in Alzheimer’s disease is a contentious issue due to the incongruence among epidemiological, preclinical and clinical research. Using innovative statistical methods, we demonstrate that while most non-steroidal anti-inflammatory drugs did not slow the progression of cognitive impairment, diclofenac dramatically improved cognitive outcomes.

## Graphical Abstract

**Graphical Abstract:**
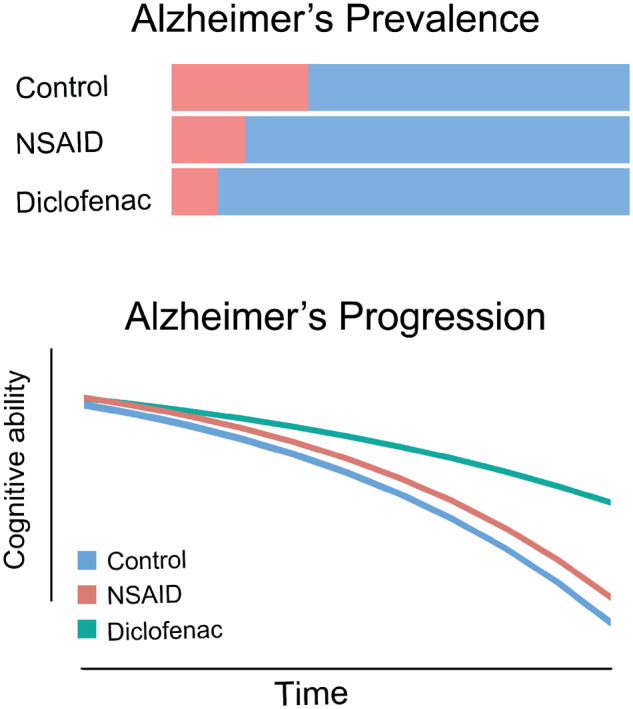

## Introduction

Alzheimer’s disease is a debilitating age-related dementia which is typified by initial loss of short-term memory and spatial awareness, followed by mid- and long-term memory loss, confusion, personality changes, frailty, loss of motor function and death normally 5–7 years following initial diagnosis (Wattmo *et al.*, 2014). Alzheimer disease is the most prevalent form of dementia, constituting 60–80% of dementia cases affecting an estimated 26 million people globally (Alzheimers Association, 2015). Because of the severe social and economic costs, Alzheimer disease has been the focus of extensive research yet the pathophysiology of Alzheimer disease remains poorly understood and disease modifying treatments continue to be elusive. However, the role of neuroinflammation as a key etiological feature is now widely accepted due the consensus of epidemiological, neuroimaging, preclinical and genetic evidence. Because of this, anti-inflammatories have been thoroughly researched as putative disease modifying agents.

Non-steroidal anti-inflammatory drugs (NSAIDs), which inhibit cyclooxygenase (COX) enzymes and subsequent prostanoid production, are the most commonly used anti-inflammatory drugs with over 110 million prescriptions annually in the US alone (Conaghan, 2012). The prevalence of NSAIDs use makes them an ideal candidate for epidemiological investigations into the potential therapeutic effects of anti-inflammatories in Alzheimer disease. Numerous epidemiological studies in a range of ethnodemographic populations have identified that NSAID use is associated with a lower risk of developing Alzheimer disease (Breitner *et al.*, 1995; Stewart *et al.*, 1997; in'T Veld *et al.*, 2001; Landi *et al.*, 2003; Fischer *et al.*, 2008; Szekely *et al.*, 2008; Vlad *et al.*, 2008; Cote *et al.*, 2012), and this association was suggested to be causal by numerous intervention-based studies in animal models (Lim *et al.*, 2000; Weggen *et al.*, 2001; Yan *et al.*, 2003). This led to a number of clinical trials of varying quality on NSAIDs and Alzheimer disease progression. Many of these trials were short term (6–12 months) and had low numbers of patients due to the lack of private funding available for existing drugs. Of the non-selective traditional NSAID clinical trials, Pasqualetti *et al.* performed the most extensive trial with 132 patients followed for 1 year. A total of 51 and 46 patients from the ibuprofen and placebo group completed the trial, respectively, and no differences in Alzheimer Disease Assessment Scale (ADAS) or the mini-mental state examination (MMSE) were observed between treatment groups (Pasqualetti *et al.*, 2009). This is still a relatively small group of patients given the variability of the disease and relatively short period of observation. Larger trials have been performed with patented novel NSAIDs including trials of celecoxib with 425 subjects (Soininen *et al.*, 2007), rofecoxib with 692 subjects (Reines *et al.*, 2004) and tarenflurbil with 1684 subjects (Green *et al.*, 2009), all were conducted for at least 1 year and all demonstrated no significant effect of the NSAIDs on Alzheimer disease progression (Imbimbo *et al.*, 2010). Several potential explanations for the discrepancy between the efficacy of NSAIDs in the epidemiological and clinical research fields have been put forward: (i) the NSAIDs effects seen in epidemiological research could be indirect through a hidden variable not investigated by the research. (ii) NSAIDs may require a long period of administration before they can provide a protective effect. This hypothesis is supported by epidemiological evidence; Stewart *et al.* (1997) analysed a longitudinal cohort study of 1686 elderly individuals and found that the risk of Alzheimer disease was only significantly decreased after more than 2 years of NSAID usage. (iii) The clinical trials methodology were not optimized for the treatments in that they should have: only included early Alzheimer disease or mild cognitively impaired (MCI) individuals with confirmed amyloid positivity and neuroinflammation through positron emission tomography imaging, had larger numbers and a longer period of treatment (Stewart *et al.*, 1997; Wyss-Coray, 2006; Szekely and Zandi, 2010). (iv) The NSAIDs selected for the high-quality clinical trials were chosen for the novelty and patentability of the drugs; as these drugs were not the focus of the epidemiological or preclinical research it possible that there are different therapeutic profiles of traditional NSAIDs and potentially the dominant mechanisms of action is independent of COX inhibition (Stewart *et al.*, 1997; Wyss-Coray, 2006; Szekely and Zandi, 2010). (v) While NSAIDs may reduce the risk of developing Alzheimer disease, they do not slow the progression of the disease; suggesting that NSAIDs act on initiating pathological processes of the disease not the downstream cascade of propagating mechanisms. The latter two of these explanations (iv and v) are addressed in this study by investigating the association of individual NSAID use and cognitive decline (as opposed to incidence/prevalence) in MCI and Alzheimer disease subjects, as measured by the MMSE and ADAS scores, in the Alzheimer’s disease Neuroimaging Initiative (ADNI) case-controlled longitudinal study dataset. From this it was found that most NSAIDs were not associated with any change in cognitive decline including celecoxib, aspirin, ibuprofen and naproxen. However, there was evidence that diclofenac was associated with slower cognitive decline. Though not technically an NSAID, paracetamol (acetaminophen) was included in the analysis as a common pain reliever which has indication overlap with NSAIDs and little mechanistic overlap. Interestingly, paracetamol use was associated with accelerated cognitive decline. Collectively this study concludes that the majority of NSAIDs do not affect the propagating mechanisms of Alzheimer disease and that the therapeutic potential of a subset of NSAIDs including diclofenac is likely to be independent of COX inhibition.

## Materials and methods

### Data acquisition

Data used in the preparation of this article were obtained from the ADNI database (adni.loni.usc.edu). The ADNI was launched in 2003 as a public–private partnership, led by Principal Investigator Michael W. Weiner, MD. The primary goal of ADNI has been to test whether serial magnetic resonance imaging, positron emission tomography, other biological markers and clinical and neuropsychological assessment can be combined to measure the progression of MCI and early Alzheimer’s disease. The subjects were recruited from over 50 sites across the USA and Canada. ADNI has undergone three stages of recruitment each with differences in the imaging and biomarker analyses, these have been named ADNI-1, ADNI-GO and ADNI-2. Collectively these protocols have recruited 1631 adults into the study consisting of age appropriate cognitively normal (CN) individuals, people with early or late MCI, and people with early Alzheimer disease. The follow-up duration of each group is specified in the protocols for ADNI-1, ADNI-2 and ADNI-GO with 120 months as the maximum. Subjects were evaluated upon entry into the study, then at the 6- and 12-month time points, and yearly after this. For up-to-date information, see www.adni-info.org.

### Data cleaning

The dataset of the medical history (RECMHIST.csv), recurrent medicines (RECCMEDS.csv) and patient summary data (ADNIMERGE.csv) were downloaded on 3 May 2018. To allow for adjustment for relevant nuisance variables string search methods were applied to generate the explanatory variables of headaches, arthritis, smoking, cardiovascular pathology and diabetes, where necessary (Supplementary 5.4). Cardiovascular pathology was defined as a subject diagnosed with hypertension or high cholesterol (Supplementary 5.4.2). Terms varied widely and spelling errors were present and so manual confirmation of correct identification was required. A similar process was required to identify recurrent oral administration of NSAIDs (Supplementary 5.2). Only oral administration was included because topical applications are not likely to reach relevant concentrations. The search terms required a mixture of pharmacological and brand names. While a range of NSAIDs were searched for only aspirin, ibuprofen, diclofenac, celecoxib and naproxen had sufficient numbers for analysis (Supplementary 5.2) and paracetamol was included as a mechanistically distinct pain-reliever with similar potencies and indications as NSAIDs. Individuals diagnosed as CN, MCI or Alzheimer disease were included in the study, those with no diagnosis but reported subjective memory concerns were removed as the subjective nature of self-diagnosis may be a source of variability.

### Distribution selection

To investigate cognitive decline over time generalized linear mixed modelling (GLMM) was applied. The selection of distribution was thoroughly performed both prior and following model construction investigating numerous distribution families (Supplementary 7.4, 7.5, 8.4, 8.5). Using graphical evaluation and Akaike information criterion (AIC) to evaluate the appropriateness of the distribution family, the negative binomial model was found to be the optimal distribution for both the MMSE and ADAS scores. For the simplicity of the model, the MMSE score was converted from a count of correct answers with a maximum of 30 into a count of incorrect answers (Supplementary 7.3), this has the advantage of now having the same directional relationship with disease severity as the ADAS score, with higher values correlating with worse cognitive performance and greater disease severity. Negative binomial models are optimal for overdispersed Poisson (count) data, suggesting that MMSE and ADAS scores can be modelled as a count of errors (Supplementary 7.5 and 8.5).

### Model construction

#### Selecting parameterization method

From the initial distribution analyses it was found that the variance was greater than the mean indicating that the data were over-dispersed as a Poisson model supporting the use of negative binomial models (Supplementary 7.5 and 8.5) (Hardin *et al.*, 2007; Bolker *et al*., 2011). There are several parameterization methods which describe the relationship between the mean and the variance in the negative binomial model (over-dispersion). The two most common [and the only methods available in the glmmADMB package on R version 3.5.1 (R Core Team, 2019) with  RStudio (2020) version 1.1.453 (Fournier *et al.*, 2012; Skaug *et al.*, 2013)] are the ‘nbinom1’ method, which assumes the variance = *k* × mean, and ‘nbinom2’ method, which assumes the variance = mean (1 + mean/*k*). The latter is most commonly used, particularly in count datasets, and is derived from a Gamma/Poisson model of a heterogeneous relationship between variance and mean (Hardin *et al.*, 2007). The former describes a simple proportional relationship between variance and mean and is less commonly used due to its inflexibility (Hardin *et al.*, 2007). Optimal parameterization method was investigated using AIC and log-likelihood both prior to, and following, the construction of the full models and the ‘nbinom1’ parameterization was selected (Supplementary 7.6 and 8.6).

#### Building of initial main effect model

Building of the GLMMs followed the protocol outlined by Hosmer and Lemeshow (2005). To construct the negative binomial GLMMs the package glmmADMB was used on R version 3.5.1 with RStudio (2020) version 1.1.453 (Fournier *et al.*, 2012; Skaug *et al.*, 2013). This package estimates parameters using the maximum likelihood method with the Laplace approximation to assess the marginal likelihood and provides coefficient summaries based on Wald approximations. The minimal model used had the explanatory variable of time (month) included as well as subject identification as a random effect (Supplementary 7.8 and 8.8). Then each biologically relevant explanatory variable was individually added to the minimal model and then compared against the minimal model using the log-likelihood ratio test with *P*-value estimated using the Chi-squared distribution and AIC to investigate if the model was significantly improved by the inclusion of the variable (Supplementary 7.8 and 8.8). Relevant nuisance explanatory variables were first investigated, followed by pain-reliever use. These included apolipoprotein E4 (ApoE4) genotype, age, diagnosis (control, MCI or Alzheimer’s disease), gender, education level, vascular pathology, smoking, headaches, arthritis, diabetes and drug use (naproxen, celecoxib, diclofenac, aspirin, ibuprofen or paracetamol). All significant variables were then included in the model and their continued input into the model in the presence of the other explanatory variables was evaluated using the Wald approximation statistics, log-likelihood ratio tests and AICs (Supplementary 7.9–7.12 and 8.9–8.12). Variables that ceased to contribute significantly to the model were dropped and the final main effect model of all significant variables was then constructed with *P* < 0.05 considered as statistically significant (Supplementary 7.12.8 and 8.12.8). Biologically relevant interaction terms were then investigated within the main effect model.

#### Inclusion of biologically relevant two-way interaction terms

The inference of multivariable interaction terms becomes difficult, therefore, a common approach is to investigate only biologically relevant two-way interaction terms (Hosmer and Lemeshow, 2005). Similar to the main effect analyses, each two-way interaction term was added to the main effect model in isolation (Supplementary 7.13 and 8.13). Model was again assessed with the log-likelihood ratio test and AIC values (Supplementary 7.13 and 8.13). A final model was constructed including all significant interactions (*P* < 0.05) and Wald approximation statistics were scrutinized for non-significant coefficients (Supplementary 7.14 and 8.14). Each interaction was then dropped in isolation from the model and compared against the full model including the variables that were dropped as main effect terms. The worsening of the model was assessed with the log-likelihood ratio test and AIC values (Supplementary 7.14–7.17 and 8.14–8.17). Covariance matrices were constructed of the final model and no substantial multicollinearity was found between included explanatory variables (Supplementary 7.22 and 8.22). The full model was then tested with time (month) treated as a factor (as opposed to a continuous numerical variable) and years in education treated as a continuous numeric variable (rather than being grouped into education levels of early, middle, tertiary and post-graduate) (Supplementary 7.18–7.19 and 8.18–8.19). Treating month as a factor introduced a substantial increase in the degrees of freedom into the model (Supplementary 7.18 and 8.18), which resulted in levels of the models with insufficient data to stabilize the model resulting in issues of model convergence, even when more simple models with fewer explanatory variables were attempted. Treating years in education as a numeric variable worsened model fit; this is probably due to the lack of correlation between numerical years and the (log) dependent variable (Supplementary 7.19 and 8.19). Coefficient plots were generated with Laplace approximated confidence intervals to allow a quick visualization of the effects of significant explanatory variables on the modelled decline over time (Supplementary 7.15 and 8.15). The variance/covariance matrices were used to generate Laplace plots of modelled decline given different combinations of the significant explanatory variables (Supplementary 9). To address issues of drop-out from the study for any reason, the analyses were repeated over the timeframe of 48 months (which has limited drop-out). The results were largely unaffected, suggesting drop-out was not having a strong bias effect on the full timeframe analyses (Supplementary 7.18 and 8.18). We used uncorrected *P*-values in the results to not limit the sensitivity of the study. The corrected *P*-values (Holm–Šídák) are reported in the Supplementary. Example cognitive decline plots are displayed in the Results section, these relationships can be fully explored at this interactive website depicting cognitive decline: https://braininflammationgroup-universityofmanchester.shinyapps.io/Rivers-Auty-ADNI/.

#### Final models for MMSE and ADNI

Below are the final models selected by the methods above for both MMSE and ADAS score.

*MMSE decline ∼ Time + Age (of participant) + Gender + Education level + Diagnosis (cognitive) + ApoE4 genotype + Diclofenac use + Aspirin use + Education level* × *Time + Diagnosis* × *Time + ApoE4 genotype* × *Time + Gender* × *Time + Paracetamol use* × *Time + Diclofenac use* × *Time + (Patient ID as a random variable).*

*ADAS decline ∼ Time + Age (of participant) + Gender + Education level + Diagnosis (cognitive) + ApoE4 genotype + Headache + Diclofenac use + Ibuprofen use + Education level* × *Time + Diagnosis* × *Time + ApoE4 genotype* × *Time + Gender* × *Time + Paracetamol use* × *Time + (Patient ID as a random variable).*

### Prevalence analysis

Baseline prevalence of Alzheimer disease was analysed with Chi-squared statistics and adjusted logistic regression. Stepwise logistic regression with AIC as the selection criteria on the confounding variables was performed to generate the base model. Each pain reliever was added to the model and the improvement was evaluated based on log-likelihood ratio tests. *P*-values were adjusted using Holm–Šídák multiple comparison adjustment method (Supplementary 6.4.4).

### Assumption check of residuals

The Pearson residuals were extracted and plotted against the explanatory variables grouped by individual identification. The ungrouped Pearson residuals were also plotted. No trends were observed for any explanatory variable, indicating the appropriateness of the negative binomial models (Supplementary 7.21 and 8.21).

### Data availability

For transparency and repeatability, the code and results of the complete analyses summarized in the manuscript are included in full in the Supplementary material. The data for the analyses presented here are available through application to the ADNI data repository at www.adni-info.org.

## Results

### Baseline statistics

**Figure 1:**
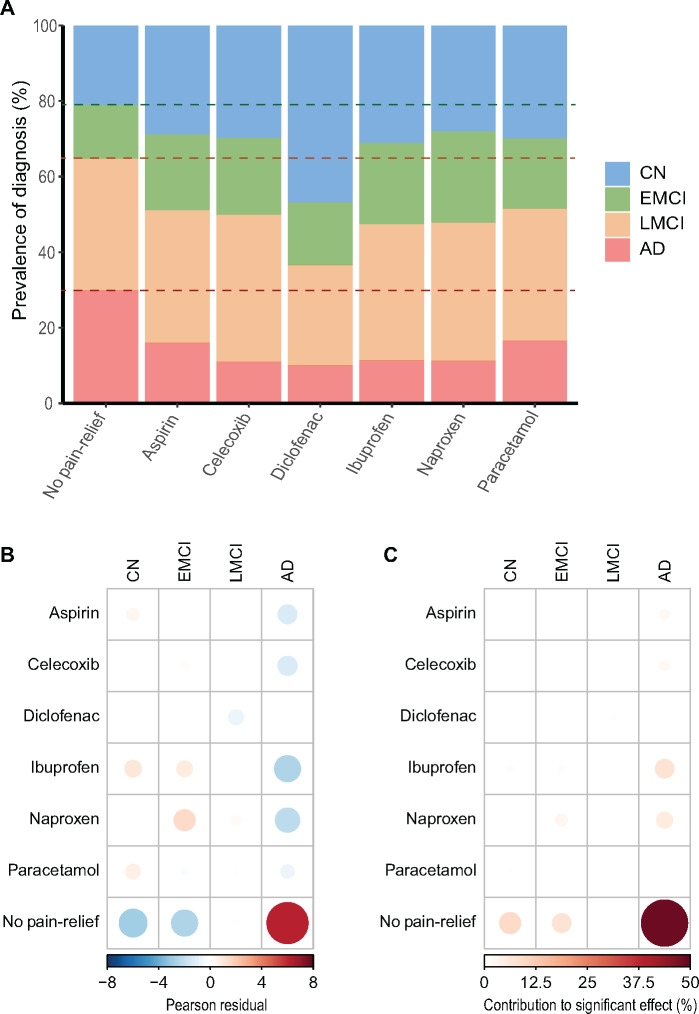
**At baseline, use of any pain-reliever was associated with lower prevalence of Alzheimer’s disease and a corresponding higher prevalence of CN diagnoses.** (**A**) Proportion of cognitive diagnosis between pain-reliever groups. Dotted lines show proportion divisions of No pain-reliever group. (**B**) Pearson residuals demonstrate the size and direction of the effect of pain-reliever use on cognitive diagnosis. (**C**) Contribution analysis of the significant association of pain-reliever subgroup and cognitive diagnosis reveals little difference between pain-reliever and the largest contribution to the significant effect is the No pain-relief group having a higher prevalence of Alzheimer disease and a lower prevalence of CN, compared to the other groups. CN: cognitively normal; EMCI and LMCI: early and late mild cognitive impairment.

This study included 1619 individuals of whom 338 (21%) had Alzheimer disease, 560 (35%) had late mild cognitive impairment, 306 (19%) had early mild cognitive impairment and 415 (26%) were CN. The proportions of each cognitive diagnosis did significantly differ with pain-reliever use (Supplementary 6.4.4). Celecoxib, diclofenac, ibuprofen, paracetamol, aspirin and naproxen use were all associated with a substantially reduced Alzheimer disease prevalence compared to the no pain-reliever group (Table 1, Fig. 1 and Supplementary 6.4), this effect remained after adjusting for confounding variables (Supplementary 6.4.4). This corresponded to significantly different mean baseline ADAS and MMSE scores (Table 1 and Supplementary 6.4). Other baseline metrics were nominally similar including gender proportions, mean age, ApoE4 status, educational attainment and diabetes prevalence (Table 1 and Supplementary 6). Headache, arthritis and cardiovascular risk factors were elevated in the pain-reliever groups (Table 1 and Supplementary 6). This is unsurprising as these pain-relievers are indicated for these conditions. Arthritis prevalence was highest in more potent pain-reliever groups such as celecoxib and diclofenac (73% and 77%, respectively), compared to 28% prevalence in the no pain-reliever group (Table 1 and Supplementary 6.4.10).

**Table 1:** Baseline statistics of ADNI cohort by pain-reliever use

	Aspirin	Celecoxib	Diclofenac	Ibuprofen	Naproxen	Paracetamol	No analgesic	Statistics
Diagnosis								*χ* ^2^ (18) = 78.1 *P* < 0.0001
CN	247 (29%)	19 (30%)	14 (47%)	79 (31%)	52 (28%)	119 (30%)	99 (21%)	
EMCI	174 (20%)	13 (20%)	5 (17%)	55 (22%)	45 (24%)	75 (19%)	67 (14%)	
LMCI	302 (35%)	25 (39%)	8 (27%)	92 (36%)	68 (37%)	140 (35%)	165 (35%)	
AD	138 (16%)	7 (10%)	3 (10%)	29 (11%)	21 (11%)	66 (17%)	141 (30%)	
ADAS	16.4 (9.0)***	15.8 (8.3)^ns^	12.3 (8.5)*	15.0 (8.5)***	15.4 (8.9)^ns^	16.2 (8.8)^ns^	19.7 (10.3)	*χ* ^2^ (6) = 57.5 *P* < 0.0001
MMSE	27.3 (2.6)***	27.2 (2.6)^ns^	28.0 (2.4)^ns^	27.7 (2.2)***	27.6 (2.4)^ns^	27.4 (2.5)^ns^	26.4 (2.8)	*χ* ^2^ (6) = 48.7 *P* <0.0001
Gender								*χ* ^2^ (6)=29.5 *P* < 0.0001
Male	323 (38%)	31 (48%)	15 (50%)	117 (46%)	95 (51%)	204 (51%)	223 (47%)	
Female	538 (62%)	33 (52%)	15 (50%)	138 (54%)	91 (49%)	196 (49%)	249 (53%)	
Age	74.3 (6.7)^ns^	73.4 (6.9)^ns^	75.22 (6.4)^ns^	72.7 (6.8)*	72.9 (7.1)^ns^	74.3 (7.2)^ns^	73.9 (8.1)	*F* (6) = 3.7 *P* = 0.001
ApoE4								*χ* ^2^ (12) = 7.5 *P* = 0.822
−/−	467 (54%)	35 (54%)	18 (60%)	145 (57%)	102 (55%)	231 (58%)	236 (50%)	
+/−	305 (35%)	24 (38%)	10 (33%)	88 (35%)	67 (36%)	133 (33%)	184 (39%)	
+/+	89 (10%)	5 (8%)	2 (7%)	22 (9%)	17 (9%)	36 (9%)	52 (11%)	
Education								*χ* ^2^ (18) = 15.7 *P* = 0.613
Primary	318 (37%)	21 (33%)	10 (33%)	83 (33%)	56 (30%)	128 (32%)	161 (34%)	
Secondary	251 (29%)	18 (28%)	11 (37%)	77 (30%)	59 (32%)	119 (30%)	126 (27%)	
Tertiary	166 (19%)	12 (19%)	3 (19%)	50 (20%)	40 (22%)	92 (23%)	92 (19%)	
Post-grad	126 (15%)	13 (20%)	6 (20%)	45 (18%)	31 (17%)	61 (15%)	93 (19%)	
Headache	67 (8%)	11 (17%)	4 (13%)	31 (12%)	20 (11%)	52 (13%)	33 (7%)	*χ* ^2^ (6) = 19.2 *P* = 0.004
Arthritis	342 (40%)	47 (73%)	23 (77%)	127 (50%)	98 (53%)	215 (54%)	132 (28%)	*χ* ^2^ (6) = 19.2 *P* < 0.001
Diabetes	92 (7%)	7 (7%)	4 (9%)	26 (7%)	21 (8%)	46 (7%)	33 (4.7%)	*χ* ^2^ (6) = 6.9 *P* = 0.330
Smoker	205 (24%)	17 (27%)	8 (27%)	71 (28%)	40 (22%)	107 (27%)	127 (27%)	*χ* ^2^ (6) = 4.5 *P* = 0.614
Cardiovascular risk factors	555 (64%)	44 (69%)	16 (53%)	158 (62%)	114 (61%)	273 (68%)	261 (55%)	*χ* ^2^ (6) = 19.8 *P* = 0.003
Total	861	64	30	255	186	400	472	

All data are in *N* (%) except for ADAS, MMSE or age which are expressed as mean (standard deviation). Statistical analyses are Chi-squared test for proportions, maximum likelihood generalized linear modelling for score data or general linear modelling for parametric data.

ADAS: Alzheimer’s disease assessment scale; CN: cognitively normal; EMCI and LMCI: early and late mild cognitive impairment; MMSE: mini-mental state examination. *P <0.05, **P <0.01, ***P <0.001.

### The effect of NSAIDs on cognitive scores

**Figure 2:**
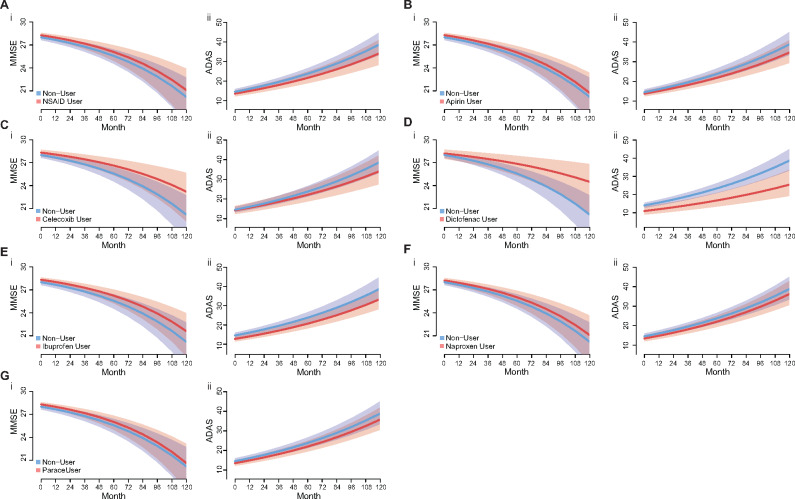
**The effect of pain-reliever use on predicted cognitive decline as measure by MMSE (i) and ADAS (ii) scores.** These models show the predicted decline of a late mild cognitive impairment, Female 70 years old, and how this decline changes when pain-relief use is included in the model. Any NSAID use (**A**); Aspirin (**B**); Celecoxib (**C**); Diclofenac (**D**); Ibuprofen (**E**); Naproxen (**F**); Paracetamol (**G**); Lines are predicted value, shaded area are 95% confidence interval. ADAS: Alzheimer’s disease assessment scale; MMSE: mini-mental state examination.

Aspirin, ibuprofen, naproxen and celecoxib were not found to be associated with any significant change in cognitive decline as measure by MMSE or ADAS (Tables 2 and 3, Fig. 2 and Supplementary 7.17 and 8.17). Paracetamol use was associated with significantly accelerated decline in both MMSE and ADAS scores, however, the effect size is of limited clinical relevance (Tables 2 and 3, Fig. 2). Diclofenac was found to be the only NSAID which was associated with reduced cognitive decline as measured by the MMSE score (Tables 2 and 3, Fig. 2), and this effect approached significance for the ADAS score (Supplementary 8.17) with clinically meaningful effect sizes (Fig. 2). The effect of diclofenac on MMSE decline remained significant (*P* = 0.039) after correcting for multiple comparisons (Supplementary 7.17).

**Table 2:** Summary of the final negative binomial GLMM with MMSE failures as the dependent variable

MMSE	Estimate	Stand error	*Z*-value	*P*-value	Statistics of inclusion
Intercept	−0.588	0.063	−9.33	*P* < 0.00001	
Fixed effects					
Diagnosis					*χ* ^2^ (3) = 1147.2, *P* < 0.00001
CN					
EMCI	0.708	0.066	12	*P* < 0.00001	
LMCI	1.242	0.056	22.38	*P* < 0.00001	
AD	2.11	0.062	33.9	*P* < 0.00001	
Gender					*χ* ^2^ (1) = 2.2, *P* = 0.138010
Female					
Male	0.096	0.040	2.36	*P* = 0.01806	
Age	0.018	0.003	6.96	*P* < 0.00001	*χ* ^2^ (1) = 48.2, *P* < 0.00001
ApoE4					*χ* ^2^ (2) = 60.6, *P* < 0.00001
−/−					
+/−	0.121	0.042	2.84	*P* = 0.00446	
+/+	0.226	0.065	3.49	*P* = 0.00048	
Education					*χ* ^2^ (3) = 47.4, *P* < 0.00001
Primary	0.352	0.057	6.14	*P* < 0.00001	
Secondary	0.362	0.056	6.5	*P* < 0.00001	
Tertiary	0.2	0.050	4.04	*P* = 0.00005	
Post-grad					
Aspirin	−0.075	0.037	−2.02	*P* = 0.04345	*χ* ^2^ (1) = 4.0, *P* = 0.045500
Paracetamol	−0.093	0.045	−2.07	*P* = 0.03882	*χ* ^2^ (1) = 1.8, *P* = 0.179712
Diclofenac	0.023	0.146	0.015	*P* = 0.87712	*χ* ^2^ (1) = 1.0, *P* = 0.317311
Interaction with time (months)					
Diagnosis					*χ* ^2^ (3) = 79.8, *P* < 0.00001
CN					
EMCI	−0.00332	0.0010	−3.18	*P* =0.00145	
LMCI	0.00286	0.0007	3.95	*P* =0.00008	
AD	0.00880	0.0014	6.12	*P* <0.00001	
Gender					*χ* ^2^ (2) = 8.4, *P* = 0.003752
Female					
Male	−0.00172	0.0006	−2.92	*P* =0.00355	
ApoE4					*χ* ^2^ (2) = 120.6, *P* < 0.00001
−/−					
+/−	0.00588	0.0006	9.52	*P* <0.00001	
+/+	0.00777	0.0009	8.32	*P* <0.00001	
Education					*χ* ^2^ (3) = 26.6, *P* < 0.00001
Primary	−0.00250	0.0008	−3.06	*P* =0.00224	
Secondary	−0.00226	0.0008	−2.79	*P* =0.00532	
Tertiary	−0.00375	0.0007	−5.05	*P* <0.00001	
Post-grad					
Paracetamol	0.00129	0.0006	2.18	*P* =0.02928	*χ* ^2^ (1) = 4.8, *P* = 0.0284597
Diclofenac	−0.00468	0.0016	−2.89	*P* =0.00380	*χ* ^2^ (1) = 8.4, *P* = 0.0037522

Shown are the maximum likelihood estimates with Laplace estimates of the standard error, *Z*-value and *P*-value from the Wald approximation, as well as, the significance of inclusion of the variable in the model evaluated using the log-likelihood ratio test Chi squared.

CN: cognitively normal; EMCI and LMCI: early and late mild cognitive impairment; MMSE: mini-mental state examination.

**Table 3:** Summary of the final negative binomial GLMM with ADAS score as the dependent variable

ADAS	Estimate	Stand error	Z-value	*P*-value	Statistics of inclusion
Intercept	2.991	0.032	92.09	*P* < 0.00001	
Fixed effects					
Diagnosis					*χ* ^2^ (3) = 1124.4, *P* < 0.00001
CN					
EMCI	0.357	0.035	10.18	*P* < 0.00001	
LMCI	0.771	0.03	25.69	*P* < 0.00001	
AD	1.248	0.035	35.84	*P* < 0.00001	
Gender					*χ* ^2^ (1) = 7.4, *P* = 0.006524
Female					
Male	0.106	0.023	4.6	*P* < 0.00001	
Age	0.0133	0.003	8.52	*P* < 0.00001	*χ* ^2^ (1) = 71.4, *P*<0.00001
ApoE4					*χ* ^2^ (2) = 63.6, *P* < 0.00001
−/−					
+/−	0.096	0.024	3.92	*P* < 0.00001	
+/+	0.149	0.039	3.86	*P* = 0.0011	
Education					*χ* ^2^ (3) = 26.2, *P* = 0.00009
Primary	0.181	0.033	5.41	*P* < 0.00001	
Secondary	0.148	0.032	4.66	*P* < 0.00001	
Tertiary	0.105	0.028	3.72	*P* =0.00020	
Post-grad					
Headache	−0.087	0.039	−2.23	*P* = 0.02563	*χ* ^2^ (1) = 5.0, *P* = 0.025347
Paracetamol	−0.035	0.026	−1.35	*P* = 0.17759	*χ* ^2^ (1) = 0.8, *P* = 0.371093
Ibuprofen	−0.093	0.030	−3.14	*P* = 0.00170	*χ* ^2^ (1) = 9.8, *P* = 0.0017451
Diclofenac	−0.224	0.080	−2.8	*P* = 0.00518	*χ* ^2^ (1) = 7.8, *P* = 0.0052246
Interaction with time (months)					
Diagnosis					χ^2^ (3) = 65.4, *P* < 0.00001
CN					
EMCI	−0.00162	0.0004	−3.98	*P* = 0.00007	
LMCI	0.00021	0.0003	0.74	*P* = 0.46177	
AD	0.00448	0.0007	6.17	*P* < 0.00001	
Gender					*χ* ^2^ (2) = 67.0, *P* < 0.00001
Female					
Male	−0.00210	0.0003	−8.22	*P* < 0.00001	
ApoE4					*χ* ^2^ (2) = 186.2, *P* < 0.00001
−/−					
+/−	0.00298	0.0003	11.31	*P* < 0.00001	
+/+	0.00476	0.0004	10.61	*P* < 0.00001	
Education					χ^2^ (3) = 50.6, *P* < 0.00001
Primary	−0.00135	0.0004	−3.74	*P* = 0.00019	
Secondary	−0.00149	0.0003	−4.29	*P* = 0.00002	
Tertiary	−0.00214	0.0003	−6.91	*P* < 0.00001	
Post-grad					
Paracetamol	0.00056	0.0003	−2.21	*P* < 0.00001	*χ* ^2^ (1) = 4.8, *P* = 0.0284597

Shown are the maximum likelihood estimates with Laplace estimates of the standard error, *Z*-value and *P*-value from the Wald approximation, as well as, the significance of inclusion of the variable in the model evaluated using the log-likelihood ratio test Chi squared.

ADAS: Alzheimer’s disease assessment scale; CN: cognitively normal; EMCI and LMCI: early and late mild cognitive impairment; MMSE: mini-mental state examination.

There was some evidence of a main effect of aspirin and ibuprofen being associated with slightly improved MMSE and ADAS scores, respectively (Tables 2 and 3, Fig. 2 and Supplementary 7.17 and 8.17). This suggests that their use is associated with a mild fixed positive effect on cognitive scores, but they were not associated with altered progression of cognitive decline (Tables 2 and 3, Fig. 2 and Supplementary 7.17 and 8.17). Diclofenac was associated with a significant positive effect on ADAS scores when included in the model only as a main effect (Tables 2 and 3, Fig. 2 and Supplementary 7.17 and 8.17), however, this main effect was not significant once the interaction term with time was included, suggesting the predominate effect of diclofenac is on cognitive decline.

**Figure 3:**
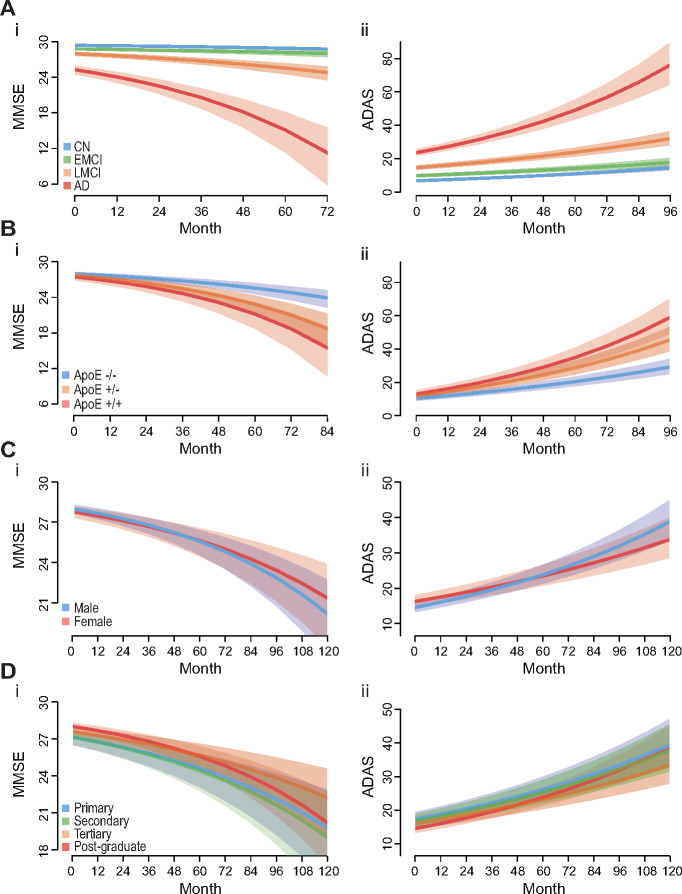
**The effect of significant explanatory variables on predicted cognitive decline as measure by MMSE (i) and ADAS (ii) scores.** These models show the predicted decline of a late mild cognitive impairment (except A), Female (except C), 70 years old, and how this decline changes when additional variables are included. Cognitive diagnosis was predictably the most influential variable, with Alzheimer’s disease and late mild cognitive impairment (LMCI) associated with rapid cognitive decline; early mild cognitive impairment (EMCI) and cognitively normal (CN) did not have appreciatively different rates of decline (**A**). ApoE4 showed a very strong gene dose association with accelerated cognitive decline (**B**). Gender showed mild differences with males having faster cognitive decline (**C**). Education had variable effects on cognitive decline with tertiary level education associated with the slowest decline (**D**). Lines are predicted value, shaded area are 95% confidence interval.

As expected, there were significant main effects and effects on progression conferred by cognitive diagnosis with Alzheimer disease and late mild cognitive impairment both having worse MMSE and ADAS values and accelerated decline (Tables 2 and 3, Fig. 3 and Supplementary 7.16 and 8.16). However, early mild cognitive impairment was not associated with accelerated progression of cognitive decline compared to the CN diagnosis, suggesting early mild cognitive impairment has limited prognostic utility (Tables 2 and 3, Fig. 3 and Supplementary 7.16 and 8.16).

Education level had a complex relationship with cognitive decline. Post-graduate level study was set as the reference level. All other education levels had worse cognitive performance as main effects, however, their progression slopes were less severe, with tertiary level education associating with the slowest progression (Tables 2 and 3, Fig. 3 and Supplementary 7.16 and 8.16). A simplified inference of this is that post-graduate level studies was associated with initial good performance in the cognitive tasks but faster decline compared to tertiary, secondary and early education levels (Tables 2 and 3, Fig. 3 and Supplementary 7.16 and 8.16).

Age did have a significant main effect on MMSE and ADAS scores associating with worse cognitive scores (Tables 2 and 3, Fig. 3 and Supplementary 7.16 and 8.16). However, there was no evidence that age was associated with altered progression (Supplementary 7.16 and 8.16).

ApoE4 genotype had a substantial gene dose main effect on MMSE and ADAS score associating with worse cognitive performance (Tables 2 and 3, Fig. 3 and Supplementary 7.16 and 8.16), as well as an association with substantially accelerated cognitive decline (Tables 2 and 3, Fig. 3 and Supplementary 7.16 and 8.16).

There was no discernible significant association of cardiovascular risk factors, smoking or diabetes on MMSE or ADAS score as a main effect or altering progression, therefore, for model parsimony they were not included in the final models (Supplementary 7.16 and 8.16).

## Discussion

Here, we used the innovative approach of negative binomial generalized linear modelling to analyse the association between pain-reliever use and cognitive decline in CN, MCI and Alzheimer disease individuals in the ADNI dataset. From this we found that, while pain-reliever use was associated with a lower prevalence of Alzheimer disease, there were no similarly positive associations with delayed cognitive decline, with the exception of diclofenac use. This is congruent with the decades of epidemiological evidence which suggests that NSAID use lowers the prevalence of Alzheimer disease (Breitner *et al.*, 1995; Stewart *et al.*, 1997; in'T Veld *et al.*, 2001; Landi *et al.*, 2003; Fischer *et al.*, 2008; Szekely *et al.*, 2008; Vlad *et al.*, 2008; Cote *et al.*, 2012) and the limited number of clinical trials which have found no effect of NSAIDs on disease progression (Scharf *et al.*, 1999; Lyketsos *et al.*, 2007; Green *et al.*, 2009; Pasqualetti *et al.*, 2009; Alzheimer's Disease Anti-inflammatory Prevention Trial Research Group, 2013). This suggests that either the therapeutic window of pain-relievers of Alzheimer disease is pre-symptomatic, acting on the initiating mechanisms of cognitive decline and not the propagating mechanisms of Alzheimer disease, or there is a hidden variable which explains the lower prevalence of Alzheimer disease in pain-reliever users, for example healthy user bias (Shrank *et al.*, 2011). Our analysis supports the latter. Healthy user bias is common in epidemiological research, it is caused by the effect of healthier individuals seeking and using therapies such as pain-relievers, resulting in spurious associations of therapy use and reduced disease prevalence. Our prevalence analysis shows similar reductions in the proportion of Alzheimer disease diagnoses between all pain-relievers, even structurally and functionally dissimilar compounds such as aspirin (weak COX1 and COX2 inhibitor), celecoxib (potent COX2 inhibitor) and paracetamol [endocannabinoid modulator, unlikely to inhibit COX1 and COX2 at physiological concentrations (Klinger-Gratz *et al.*, 2018)]. The similarity in effects on Alzheimer disease incidence, despite structural and functional difference supports the existence of a hidden variable such as the healthy user bias. Furthermore, individuals taking any of the investigated drugs at the beginning of the study were less likely to drop-out (due to very poor health, death or other). This again supports the healthy user bias conclusion, as healthier subjects were more likely to remain in the study. This alternative explanation of the epidemiological Alzheimer disease prevalence literature, is further supported by the placebo-controlled Alzheimer disease Anti-inflammatory Prevention Trial (ADAPT) trial (2013), which investigated the effects of celecoxib or naproxen on Alzheimer disease incidence in 2528 CN elderly people and found no positive effects, suggesting that COX inhibition is ineffective even in the presymptomatic stages of the disease.

Unlike the other pain-relievers, diclofenac use was associated with a slower cognitive decline as measured by MMSE scores and approached significance with ADAS scores. Though not thoroughly researched, there is evidence that diclofenac is a promising avenue of therapeutic development for Alzheimer disease. Landi *et al.* (2003) performed a cross-sectional study of 2708 community dwelling elderly people. They utilized logistic regression on the proportions of those diagnosed with Alzheimer disease in each NSAID category and found that diclofenac had the greatest effect on risk of Alzheimer disease diagnosis with an odds ratio of 0.21 (95% confidence interval of 0.05–0.90). Similarly, in this study, diclofenac had the lowest prevalence of Alzheimer disease of all pain-relievers tested, suggesting a potential prophylactic effect, as well as the reported potential therapeutic effect on disease progression. Furthermore, a small underpowered clinical trial was performed by Scharf *et al.* (1999). This was a single centre trial recruiting mild to moderate Alzheimer disease patients defined by an MMSE of 11–25. A total of 41 patients were recruited and 24 were randomly allocated to the placebo group and 17 to the daily diclofenac treatment group and the patients were followed up for cognitive assessment after 6 months of treatment (Scharf *et al.*, 1999). Due to the lack of power of the study and short time span, no strong inferences should be made, however, the trends largely concur with the Landi *et al.* (2003) study and this study; the placebo group declined cognitively with a mean MMSE score decline of −0.86 (standard deviation 3.21) and an ADAS increase in 1.93 (standard deviation 5.55), while the diclofenac MMSE score improved on average 0.41 (standard deviation 2.69) and the ADAS stayed relatively stable with a slight increase in 0.25 (standard deviation 4.5) (Scharf *et al.*, 1999). Given that the other NSAIDs tested in clinical trials did not slow the progression of Alzheimer disease and were not associated with slower decline in this study despite also being potent inhibitors of the COX enzymes, it is fair to hypothesize that any potential effects of diclofenac on Alzheimer disease are likely not through this mechanism of action. Unlike the other pain-relievers investigated in this research, diclofenac also inhibits the release of the inflammatory cytokine interleukine-1β (26, Supplementary 1) by inhibiting the activation of the intracellular receptor NLRP3 (NOD-like receptor family, pyrin domain containing 3). The NOD-like receptor family, pyrin domain containing 3 receptor in microglia has been shown to be central to the neuroinflammatory response observed in mouse models of Alzheimer disease (Daniels *et al.*, 2016) and inhibition of the NOD-like receptor family, pyrin domain containing 3 receptor with similar compounds has been found to be therapeutic in several animal models of Alzheimer disease (Daniels *et al.*, 2016; Dempsey *et al.*, 2017). Furthermore, studies have found that the genetic deletion of this receptor completely abated the Alzheimer disease phenotype in mouse models (Dostert *et al.*, 2008; Heneka *et al.*, 2013). Therefore, NOD-like receptor family, pyrin domain containing 3 inhibition may be the defining feature of diclofenac. However, it should be noted that only 30 subjects were diclofenac consumers with sufficient data for inclusion in the analysis. Therefore, any strong inference of efficacy should be avoided as future research is needed on this promising NSAID.

The results of the analyses presented here found substantial evidence for ApoE4 causing accelerated cognitive decline. The lipoprotein ApoE4 is a well-established risk factor for the development of Alzheimer disease (Notkola *et al.*, 1998; Cornelius *et al.*, 2004; Szekely *et al.*, 2008). Several studies have also found that ApoE4 alleles are associated with accelerated cognitive decline and accelerated cortical tissue atrophy (Kanai *et al.*, 1999; Tilvis *et al.*, 2004; Bartzokis *et al.*, 2006; Morra *et al.*, 2009; Whitehair *et al.*, 2010; Lo *et al.*, 2011; Mielke *et al.*, 2011; Young *et al.*, 2014; Vijayaraghavan *et al.*, 2016; Kim *et al.*, 2017; Rawle *et al.*, 2018). However, a limitation common in the existing literature is the use of multi-level linear modelling for non-Gaussian discrete cognitive scores (Kanai *et al.*, 1999; West *et al.*, 2008; Whitehair *et al.*, 2010; Lo *et al.*, 2011; Mielke *et al.*, 2011; Vemuri *et al.*, 2014; Kim *et al.*, 2017; Rawle *et al.*, 2018), therefore, this study, with 744 individuals with at least one ApoE4 gene, investigating both ADAS and MMSE measures of cognitive decline and applying discrete distribution GLMM analyses with the negative binomial models, represents a robust and important contribution to the field.

Previous research has reported that NSAIDs only alter Alzheimer disease incidence in ApoE4 carriers, suggesting an interaction between the potential therapeutic mechanism of NSAIDs and the pathological mechanisms of ApoE4. This was first reported in a thorough study by Szekely *et al.* (2008) who looked at Alzheimer disease incidence in 3229 elderly people during a 10-year period. They found that NSAID use was associated with a hazard ratio of 0.88 in non-ApoE4 carriers and 0.34 in ApoE4 carriers (compared to matched individuals). This study investigated a three-way interaction of NSAIDs, ApoE4 genotype and time (month), and no significant effects were observed (Supplementary 7.20 and 8.20). This suggests that if the Szekely *et al.* finding is due to NSAID-ApoE4 interactions, and not hidden nuisance variables present in the analysis, then this therapeutic effect may only be useful for the prophylactic treatment to prevent Alzheimer disease in ApoE4 individuals and not effective in altering the progression of the disease.

## Conclusion

This study is a thorough investigation into the effects of NSAID and paracetamol use on Alzheimer disease and MCI cognitive decline. Also investigated were the effects of gender, smoking status, headaches, arthritis, diabetes, age, vascular pathology, ApoE4 genotype and education level. Due to the discrete nature of the dependent variables MMSE and ADAS scores, GLMMs were investigated and the negative binomial distribution was found to be a robust approach which outperformed other models. Ibuprofen and aspirin use were associated with improved cognitive performance at baseline, however, neither were associated with an altered cognitive decline. Naproxen and celecoxib use were not associated with any significant alterations in cognitive performance and paracetamol use was associated with accelerated cognitive decline although this effect size was negligible. This suggests that NSAIDs and paracetamol are not promising therapeutics for altering the progression of cognitive decline in MCI and Alzheimer disease individuals. However, diclofenac use was associated with slower cognitive decline, and as this was the only NSAID to do so, this suggests that COX inhibition is not the likely mechanism of action. Therefore, the full interactome of diclofenac should be investigated for potential therapeutic avenues. Collectively, this study found interesting future avenues of research particularly the effects of paracetamol and diclofenac on Alzheimer disease progression and improved the evidence for our existing understanding of factors which effect Alzheimer disease such as the ApoE4 genotype by applying innovative statistical methods.

## Funding

Data collection and sharing for this project was funded by the Alzheimer's Disease Neuroimaging Initiative (ADNI) (National Institutes of Health Grant U01 AG024904) and DOD ADNI (Department of Defense award number W81XWH-12-2-0012). ADNI is funded by the National Institute on Aging, the National Institute of Biomedical Imaging and Bioengineering, and through generous contributions from the following: AbbVie, Alzheimer’s Association; Alzheimer’s Drug Discovery Foundation; Araclon Biotech; BioClinica, Inc.; Biogen; Bristol-Myers Squibb Company; CereSpir, Inc.; Cogstate; Eisai, Inc.; Elan Pharmaceuticals, Inc.; Eli Lilly and Company; EuroImmun; F. Hoffmann-La Roche Ltd. and its affiliated company Genentech, Inc.; Fujirebio; GE Healthcare; IXICO Ltd.; Janssen Alzheimer Immunotherapy Research & Development, LLC.; Johnson & Johnson Pharmaceutical Research & Development LLC.; Lumosity; Lundbeck; Merck & Co., Inc.; Meso Scale Diagnostics, LLC.; NeuroRx Research; Neurotrack Technologies; Novartis Pharmaceuticals Corporation; Pfizer, Inc.; Piramal Imaging; Servier; Takeda Pharmaceutical Company and Transition Therapeutics. The Canadian Institutes of Health Research is providing funds to support ADNI clinical sites in Canada. Private sector contributions are facilitated by the Foundation for the National Institutes of Health (www.fnih.org). The grantee organization is the Northern California Institute for Research and Education, and the study is coordinated by the Alzheimer’s Therapeutic Research Institute at the University of Southern California. ADNI data are disseminated by the Laboratory for Neuro Imaging at the University of Southern California. A.E.M. is a Food Standards Agency Fellow and is supported by the Biotechnology and Biological Sciences Research Council (BBSRC) Institute Strategic Programme Microbes in the Food Chain BB/R012504/1 and its constituent projects BBS/E/F/000PR10348 (Theme 1, Epidemiology and Evolution of Pathogens in the Food Chain) and BBS/E/F/000PR10351 (Theme 3, Microbial Communities in the Food Chain). J.R.-A. was a Future Leader Fellow supported by the BBSRC fellowship grant titled Understanding how dietary zinc and inflammation impact healthy ageing in the brain (BB/P01061X/1).

## Competing interests

The authors report no competing interests.

## Supplementary Material

fcaa109_Supplementary_Data

## Notes

Data used in preparation of this article were obtained from the Alzheimer’s Disease Neuroimaging Initiative (ADNI) database (adni.loni.usc.edu). As such, the investigators within the ADNI contributed to the design and implementation of ADNI and/or provided data but did not participate in analysis or writing of this report. A complete listing of ADNI investigators can be found at: http://adni.loni.usc.edu/wp-content/uploads/how_to_apply/ADNI_Acknowledgement_List.pdf.

## Abbreviations

ADAS =: Alzheimer Disease Assessment Scale
ADNI =: Alzheimer's disease neuroimaging initiative
AIC =: Akaike information criterion
ApoE4 =: apolipoprotein E4
CN =: cognitively normal
COX =: cyclooxygenase
GLMM =: generalized linear mixed model
ID =: identification
MCI =: mild cognitive impairment
MMSE =: mini-mental state examination
NSAID =: non-steroidal anti-inflammatory drug
