# Supplementary material for: Anti-inflammatories in Alzheimer’s disease—potential therapy or spurious correlate?
Source: Brain Commun. 2020 Jul 24;2(2):fcaa109. doi: 10.1093/braincomms/fcaa109 (PMC7585697; doi:10.1093/braincomms/fcaa109)
Supplement: fcaa109_Supplementary_Data [file fcaa109_supplementary_data.zip › Original Submission.pdf]

OXFORD UNIVERSITY PRESS | Brain Communications

Anti-inflammatories in Alzheimer's disease - potential therapy or spurious correlate?

Journal:	*Brain Communications*
Manuscript ID	BRAINCOM-2020-049
Manuscript Type:	Original Article
Date Submitted by the Author:	17-Feb-2020
Complete List of Authors:	Rivers-Auty, Jack; University of Tasmania, Medical Sciences; The University of Manchester, Department of Neuroscience and Experimental Psychology<br>Mather, Alison; Quadram Institute Bioscience; University of East Anglia<br>Peters, Ruth; University of New South Wales, School of Psychology; Neuroscience Research Australia<br>Lawrence, Catherine; The University of Manchester, Division of Neuroscience and Experimental Psychology; The University of Manchester, Lydia Becker Institute of Immunology and Inflammation<br>Brough, David; The University of Manchester, Division of Neuroscience & Experimental Psychology; The University of Manchester, Lydia Becker Institute of Immunology and Inflammation
Keywords:	

SCHOLARONE™ Manuscripts

Anti-inflammatories in Alzheimer's disease - potential therapy or spurious correlate?

Jack Rivers-Auty[1,2,3†], Alison E. Mather[4,5], Ruth Peters[6,7], Catherine B. Lawrence[1,2], David Brough[1,2].

*for the Alzheimer's Disease Neuroimaging Initiative

*Data used in preparation of this article were obtained from the Alzheimer's Disease Neuroimaging Initiative (ADNI) database (adni.loni.usc.edu). As such, the investigators within the ADNI contributed to the design and implementation of ADNI and/or provided data but did not participate in analysis or writing of this report. A complete listing of ADNI investigators can be found at: http://adni.loni.usc.edu/wp-content/uploads/how_to_apply/ADNI_Acknowledgement_List.pdf

[1]Division of Neuroscience and Experimental Psychology, School of Biological Sciences, Faculty of Biology, Medicine and Health, Manchester Academic Health Science Centre, University of Manchester, AV Hill Building, Oxford Road, Manchester, M13 9PT, U.K.

[2]Lydia Becker Institute of Immunology and Inflammation, University of Manchester, Manchester M13 9PT, UK

[3]Medical Sciences, School of Medicine, College of Health and Medicine, University of Tasmania, Hobart, Tasmania, Australia

[4]Quadram Institute Bioscience, Norwich Research Park, Norwich, Norfolk, NR4 7UA, UK.

[5]University of East Anglia, Norwich Research Park, Norwich, Norfolk, NR4 7TJ, UK.

[6]School of Psychology, University of New South Wales, Sydney, NSW, Australia

[7]Neuroscience Research Australia, Barker Street, Sydney, New South Wales 2031, Australia

[†]to whom correspondence should be addressed: Jack Rivers-Auty. Tel: +61 (0)475 924 722; Fax: +44 161 275 5948; Email: jack.auty@UTAS.edu.au

Search terms: Alzheimer's disease, NSAID, inflammation, Cognitive decline

Title Character Counts: 86

Number of Tables: 3

Number of Figures: 3

Word count of Abstract: 148

Word count of Paper: 4200

For Review Only

Financial Disclosures: No disclosures

Study funded by: See acknowledgment section

Statistical analysis conducted by JRA and AEM

For Review Only

Abstract

Epidemiological evidence suggests non-steroidal anti-inflammatories (NSAIDs) reduce the risk of Alzheimer's disease (AD). However, clinical trials have found no evidence of NSAID efficacy. This incongruence may be due to the wrong NSAIDs being tested in robust clinical trials or the epidemiological findings being caused by confounding factors. Therefore, this study utilized innovative statistical methods to investigate both prevalence and cognitive decline in the Alzheimer's Disease NeuroImaging (ADNI) dataset for each commonly used NSAID and paracetamol.

Use of most NSAIDs were associated with reduced AD prevalence yet no effect on cognitive decline was observed. Paracetamol had a similar effect on prevalence to these NSAIDs suggesting this association is independent of the anti-inflammatory effects and that previous results may be due to spurious associations. Interestingly, diclofenac use was significantly associated with both reduce incidence and slower cognitive decline warranting further research into the potential therapeutic effects of diclofenac in AD.

For Review Only

Introduction

Alzheimer's disease (AD) is a debilitating age related dementia which is typified by initial loss of short term memory and spatial awareness, followed by mid and long term memory loss, confusion, personality changes, frailty, loss of motor function and death normally 5-7 years following initial diagnosis [1]. AD is the most prevalent form of dementia, constituting 60-80% of dementia cases affecting an estimated 26 million people globally [2]. Because of the severe social and economic costs, AD has been the focus of extensive research yet the pathophysiology of AD remains poorly understood and disease modifying treatments continue to be elusive. However, the role of neuroinflammation as a key etiological feature is now widely accepted due the consensus of epidemiological, neuroimaging, preclinical and genetic evidence. Because of this, anti-inflammatories have been thoroughly researched as putative disease modifying agents.

Non-steroidal anti-inflammatory drugs (NSAIDs), which inhibit cyclooxygenase (COX) enzymes and subsequent prostanoid production, are the most commonly used anti-inflammatory drugs with over 110 million prescriptions annually in the USA alone [3]. The prevalence of NSAIDs use makes them an ideal candidate for epidemiological investigations into the potential therapeutic effects of anti-inflammatories in AD. Numerous epidemiological studies in a range of ethnodemographic populations have identified that NSAID use is associated with a lower risk of developing AD [4-11], and this association was suggested to be causal by numerous intervention based studies in animal models [12-14]. This led to a number of clinical trials of varying quality on NSAIDs and AD progression. Many of these trials were short term (6-12 months) and had low numbers of patients due to the lack of private funding available for existing drugs. Of the non-selective traditional NSAID clinical trials, Pasqualetti *et al.* performed the most extensive trial with 132 patients followed for 1 year. A total of 51 and 46 patients from the ibuprofen and placebo group completed the trial, respectively, and no differences in AD Assessment Scale (ADAS) or the Mini-Mental State Examination (MMSE) were observed between treatment groups [15]. This is still a relatively small group of patients given the variability of the disease and relatively short period of observation. Larger trials have been performed with patented novel NSAIDs including trials of celecoxib with 425 subjects [16], rofecoxib with 692 subjects [17]; and tarenfluribil with 1684 subjects [18], all were conducted for at least one year and all demonstrated no significant effect of the NSAIDs on AD progression [19]. Several potential explanations for the discrepancy between the efficacy of NSAIDs in the epidemiological and clinical research fields have been put forward: (i) The NSAIDs effects seen in epidemiological research could be indirect through a hidden variable not investigated by the research. (ii) NSAIDs may require a long period of administration before they can provide a protective effect. This hypothesis is supported by epidemiological evidence; Stewart *et al.* [9] analysed a longitudinal cohort study of 1686 elderly individuals and found that the risk of AD was only significantly decreased after more than two years of NSAID usage. (iii) The clinical trials methodology were not optimized for the treatments in that they should have: only included early AD or Mild Cognitively Impaired (MCI) individuals with confirmed amyloid positivity and neuroinflammation through PET imaging, had larger numbers, and a longer period of treatment [9,20,21]. (iv) The NSAIDs selected for the high quality clinical trials were chosen for the novelty and patentability of the drugs; as these drugs were not the focus of the epidemiological or preclinical research it possible that there are different therapeutic profiles of traditional NSAIDs and potentially the dominant mechanisms of action is independent of COX inhibition [9,20,21]. (v) While NSAIDs may reduce the risk of developing AD, they do not slow the progression of the disease; suggesting that NSAIDs act on initiating pathological processes of the disease not the downstream cascade of propagating mechanisms. The latter two of these explanations (iv & v) are

For Review Only

addressed in the present study by investigating the association of individual NSAID use and cognitive decline (as opposed to incidence/prevalence) in MCI and AD subjects, as measured by the MMSE and ADAS scores, in the Alzheimer's disease Neuroimaging Initiative (ADNI) case controlled longitudinal study dataset. From this it was found the most NSAIDs were not associated with any change in cognitive decline including celecoxib, aspirin, ibuprofen and naproxen. However, there was evidence that diclofenac was associated with slower cognitive decline. Though not technically an NSAID, paracetamol (acetaminophen) was included in the analysis as a common pain reliever which has indication overlap with NSAIDs and little mechanistic overlap. Interestingly, paracetamol use was associated with accelerated cognitive decline. Collectively this study concludes that the majority of NSAIDs do not affect the propagating mechanisms of AD and that the therapeutic potential of a subset of NSAIDs including diclofenac is likely to be independent of COX inhibition.

Methods

Data acquisition

Data used in the preparation of this article were obtained from the Alzheimer's Disease Neuroimaging Initiative (ADNI) database (adni.loni.usc.edu). The ADNI was launched in 2003 as a public-private partnership, led by Principal Investigator Michael W. Weiner, MD. The primary goal of ADNI has been to test whether serial magnetic resonance imaging (MRI), positron emission tomography (PET), other biological markers, and clinical and neuropsychological assessment can be combined to measure the progression of mild cognitive impairment (MCI) and early Alzheimer's disease (AD).The subjects were recruited from over 50 sites across the U.S. and Canada. ADNI has undergone three stages of recruitment each with differences in the imaging and biomarker analyses, these have been named ADNI-1, ADNI-GO and ADNI-2. Collectively these protocols have recruited 1631 adults into the study consisting of age appropriate cognitively normal individuals, people with early or late MCI, and people with early AD. The follow up duration of each group is specified in the protocols for ADNI-1, ADNI-2 and ADNI-GO with 120 months as the maximum. Subjects were evaluated upon entry into the study, then at the 6 and 12 month time points, and yearly after this. For up-to-date information, see www.adni-info.org.

Data cleaning

The dataset of the medical history (RECMHIST.csv), recurrent medicines (RECCMEDS.csv) and patient summary data (ADNIMERGE.csv) were downloaded on the 3rd of May 2018. To allow for adjustment for relevant nuisance variables string search methods were applied to generate the explanatory variables of headaches, arthritis, smoking, cardiovascular pathology and diabetes, where necessary (supplement 5.4). Cardiovascular pathology was defined as a subject diagnosed with hypertension or high cholesterol (supplement 5.4.2). Terms varied widely and spelling errors were present and so manual confirmation of correct identification was required. A similar process was required to identify recurrent oral administration of NSAIDs (supplement 5.2). Only oral administration was included because topical applications are not likely to reach relevant concentrations. The search terms required a mixture of pharmacological and brand names. While a range of NSAIDs were searched for only aspirin, ibuprofen, diclofenac, celecoxib and naproxen had sufficient numbers for analysis (supplement 5.2) and paracetamol was included as a mechanistically distinct pain-reliever with similar potencies and indications as NSAIDs.

Distribution selection

For Review Only

To investigate cognitive decline over time generalised linear mixed modelling (GLMM) was applied. The selection of distribution was thoroughly performed both prior and following model construction investigating numerous distribution families (supplement 7.5, 7.23, 8.5, 8.23). Using graphical evaluation and Akaike information criterion (AIC) to evaluate the appropriateness of the distribution family, the negative binomial model was found to be the optimal distribution for both the MMSE and ADAS scores. For the simplicity of the model, the MMSE score was converted from a count of correct answers with a maximum of 30 into a count of incorrect answers (supplement 7.3), this has the advantage of now having the same directional relationship with disease severity as the ADAS score, with higher values correlating with worse cognitive performance and greater disease severity. Negative binomial models are optimal for overdispersed Poisson (count) data, suggesting that MMSE and ADAS scores can be modelled as a count of errors (supplement 7.5 & 8.5).

Model construction

Selecting parameterisation method

From the initial distribution analyses it was found that the variance was greater than the mean indicating that the data were over-dispersed as a Poisson model supporting the use of negative binomial models (supplement 7.5 & 8.5)[22,23]. There are several parameterisation methods which describe the relationship between the mean and the variance in the negative binomial model (over-dispersion). The two most common (and the only methods available in the glmmadmb package on R version 3.5.1[24] with RStudio version 1.1.453 [25-27]) are the 'nbinom1' method, which assumes the variance = k × mean, and 'nbinom2' method, which assumes the variance = mean(1 + mean/k). The latter is most commonly used, particularly in count datasets, and is derived from a Gamma/Poisson model of a heterogeneous relationship between variance and mean [23]. The former describes a simple proportional relationship between variance and mean and is less commonly used due to its inflexibility[23]. Optimal parameterisation method was investigated using AIC and log-likelihood both prior to, and following, the construction of the full models and the 'nbinom1' parameterisation was selected (supplement 7.6 & 8.6).

Building of initial main effect model

Building of the GLMMs followed the protocol outlined by Hosmer et al. [28]. To construct the negative binomial GLMM models the package glmmadmb was used on R version 3.5.1[24] with RStudio version 1.1.453 [25-27]. This package estimates parameters using the maximum likelihood method with the Laplace approximation to assess the marginal likelihood and provides coefficient summaries based on Wald approximations. The minimal model used had the explanatory variable of time (month) included as well as subject ID as a random effect (supplement 7.8 & 8.8). Then each biologically relevant explanatory variable was individually added to the minimal model and then compared against the minimal model using the log likelihood ratio test with p-value estimated using the Chi-squared distribution and AIC to investigate if the model was significantly improved by the inclusion of the variable (supplement 7.8 & 8.8). Relevant nuisance explanatory variables were first investigated, followed by pain-reliever use. These included APOE4 genotype, age, diagnosis (control, MCI or Alzheimer's disease), gender, education level, vascular pathology, smoking, headaches, arthritis, diabetes and drug use (naproxen, celecoxib, diclofenac, aspirin, ibuprofen or paracetamol). All significant variables were then included in the model and their continued input into the model in the presence of the other explanatory variables was evaluated using the Wald approximation statistics, Log likelihood ratio tests and AICs (supplement 7.9-7.12 & 8.9-8.12). Variables that ceased to contribute significantly to the model were dropped and the final main effect model of all significant variables was then constructed with $p<0.05$ considered as

For Review Only

statistically significant (supplement 7.12.8 & 8.12.8). Biologically relevant interaction terms were then investigated within the main effect model.

Inclusion of biologically relevant two-way interaction terms

The inference of multivariable interaction terms becomes difficult, therefore, a common approach is to investigate only biologically relevant two-way interaction terms[28]. Similar to the main effect analyses, each two-way interaction term was added to the main effect model in isolation (supplement 7.13 & 8.13). Model was again assessed with the log likelihood ratio test and AIC values (supplement 7.13 & 8.13). A final model was constructed including all significant interactions ($p<0.05$) and Wald approximation statistics were scrutinized for non-significant coefficients (supplement 7.14 & 8.14). Each interaction was then dropped in isolation from the model and compared against the full model including the variables that were dropped as main effect terms. The worsening of the model was assessed with the log likelihood ratio test and AIC values (supplement 7.14-7.17 & 8.14-8.17). Covariance matrices were constructed of the final model and no substantial multicollinearity was found between included explanatory variables (supplement 7.22 & 8.22). The full model was then tested with time (month) treated as a factor (as opposed to a continuous numerical variable) and years in education treated as a continuous numeric variable (rather than being grouped into education levels of early, middle, tertiary and post-graduate) (supplement 7.18-7.19 & 8.18-8.19). Treating month as a factor introduced a substantial increase in the degrees of freedom into the model (supplement 7.18 & 8.18), which resulted in levels of the models with insufficient data to stabilize the model resulting in issues of model convergence, even when more simple models with fewer explanatory variables were attempted. Treating years in education as a numeric variable worsened model fit; this is probably due to the lack of correlation between numerical years and the (log) dependent variable (supplement 7.19 & 8.19). Coefficient plots were generated with Laplace approximated confidence intervals to allow a quick visualisation of the effects of significant explanatory variables on the modelled decline over time (supplement 7.15 & 8.15). The variance/covariance matrices were used to generate Laplace plots of modelled decline given different combinations of the significant explanatory variables (supplement 9). Example cognitive decline plots are displayed in the results section, these relationships can be fully explored at this interactive website depicting cognitive decline:

https://braininflammationgroup-universityofmanchester.shinyapps.io/Rivers-Auty-ADNI/

Assumption check of residuals

The Pearson residuals were extracted and plotted against the explanatory variables grouped by individual ID. The ungrouped Pearson residuals were also plotted. No trends were observed for any explanatory variable, indicating the appropriateness of the negative binomial models (supplement 7.21 & 8.21).

Data availability

For transparency and repeatability, the code and results of the complete analyses summarised in the manuscript are included in full in the supplemental material. The data for the analyses presented here are available through application to the ADNI data repository at www.adni-info.org.

Results

For Review Only

Baseline statistics

This study included 1619 individuals of whom 338 (21%) had AD, 560 (35%) had late mild cognitive impairment (LMCI), 306 (19%) had early mild cognitive impairment (EMCI) and 415 (26%) were cognitively normal (CN). The proportions of each cognitive diagnosis did significantly differ with pain-reliever use. Celecoxib, diclofenac, ibuprofen and naproxen use were all associated with a substantially reduced AD prevalence, and aspirin and paracetamol use was associated with a moderately reduced AD prevalence (table 1, fig. 1, supplement 6.4). This corresponded to significantly different mean baseline ADAS and MMSE scores (table 1, supplement 6.4). Other baseline metrics were nominally similar including gender proportions, mean age, APOE4 status, educational attainment and diabetes prevalence (table 1, supplement 6). Headache, arthritis and cardiovascular risk factors were elevated in the pain-reliever groups (table 1, supplement 6). This is unsurprising as these pain-relievers are indicated for these conditions. Arthritis prevalence was highest in more potent pain-reliever groups such as celecoxib and diclofenac (73% and 77%, respectively), compared to 28% prevalence in the no pain-reliever group (table 1, supplement 6.4.10).

The Effect of NSAIDs on Cognitive Scores

Aspirin, ibuprofen, naproxen and celecoxib were not found to be associated with any significant change in cognitive decline as measure by MMSE or ADAS (table 2 & 3, fig 3, supplement 7.17 & 8.17). Paracetamol use was associated with significantly accelerated decline in both MMSE and ADAS scores, however, the effect size is of limited clinical relevance (table 2 & 3, fig. 3). Diclofenac was found to be the only NSAIDs which was associated with reduced cognitive decline as measured by the MMSE score (table 2 & 3, fig. 3), and this effect approached significance for the ADAS score (supplement 8.17) with clinically meaningful effect sizes (fig. 3).

There was some evidence of a main effect of aspirin and ibuprofen being associated with slightly improved MMSE and ADAS scores, respectively (table 2 & 3, fig. 3, supplements 7.17 & 8.17). This suggests that their use is associated with a mild fixed positive effect on cognitive scores, but they were not associated with altered progression of cognitive decline (table 2 & 3, fig. 3, supplements 7.17 & 8.17). Diclofenac was associated with a significant positive effect on MMSE and ADAS scores when included in the model only as a main effect (table 2 & 3, fig. 3, supplement 7.17 & 8.17).

As expected there were significant main effects and effects on progression conferred by cognitive diagnosis with AD and LMCI both having worse MMSE and ADAS values and accelerated decline (table 2 & 3, fig. 2, supplements 7.16 & 8.16). However, EMCI was not associated with accelerated progression of cognitive decline compared to the CN diagnosis, suggesting EMCI has limited prognostic utility (table 2 & 3, fig., supplements 7.16 & 8.16).

Education level had a complex relationship with cognitive decline. Post-graduate level study was set as the reference level. All other education levels had worse cognitive performance as main effects, however, their progression slopes were less severe, with tertiary level education associating with the slowest progression (table 2 & 3, fig. 2, supplements 7.16 & 8.16). A simplified inference of this is that post-graduate level studies was associated with initial good performance in the cognitive tasks but faster decline compared to tertiary, secondary and early education levels (table 2 & 3, fig. 2, supplements 7.16 & 8.16).

For Review Only

Age did have a significant main effect on MMSE and ADAS scores associating with worse cognitive scores (table 2 & 3, fig. 2, supplements 7.16 & 8.16). However, there was no evidence that age was associated with altered progression (supplements 7.16 & 8.16).

APOE4 genotype had a substantial gene dose main effect on MMSE and ADAS score associating with worse cognitive performance (table 2 & 3, fig. 2, supplements 7.16 & 8.16), as well as an association with substantially accelerated cognitive decline (table 2 & 3, fig. 2, supplements 7.16 & 8.16).

There was no discernible significant association of cardiovascular risk factors, smoking or diabetes on MMSE or ADAS score as a main effect or altering progression, therefore, for model parsimony they were not included in the final models (supplements 7.16 & 8.16).

Discussion

Here we used the innovative approach of negative binomial generalized linear modelling to analyse the association between pain-reliever use and cognitive decline in CN, MCI and AD individuals in the ADNI dataset. From this we found that, while pain-reliever use was associated with a lower prevalence of AD, there were no similarly positive associations with delayed cognitive decline, with the exception of diclofenac use. This is congruent with the decades of epidemiological evidence which suggests that NSAID use lowers the prevalence of AD[4-11] and the limited number of clinical trials which have found no effect of NSAIDs on disease progression [15,18,29-31]. This suggests that either the therapeutic window of pain-relievers of AD is pre-symptomatic, acting on the initiating mechanisms of cognitive decline and not the propagating mechanisms of AD, or there is a hidden variable which explains the lower prevalence of AD in pain-reliever users, for example healthy user bias[32]. Our analysis supports the latter. Healthy user bias is common in epidemiological research, it is caused by the effect of healthier individuals seeking and using therapies such as pain-relievers, resulting in spurious associations of therapy use and reduced disease prevalence. Our prevalence analysis shows similar reductions in the proportion of AD diagnoses between all pain-relievers, even structurally and functionally dissimilar compounds such as aspirin (weak COX1 and COX2 inhibitor), celecoxib (potent COX2 inhibitor) and paracetamol (endocannabinoid modulator, unlikely to inhibit COX1 and 2 at physiological concentrations). The similarity in effects on AD incidence, despite structural and functional difference supports the existence of a hidden variable such as the healthy user bias. This alternative explanation of the epidemiological AD prevalence literature, is further supported by the placebo controlled AD Anti-inflammatory Prevention Trial (ADAPT) trial[29], which investigated the effects of celecoxib or naproxen on AD incidence in 2528 cognitively normal elderly people and found no positive effects, suggesting that COX inhibition is ineffective even in the presymptomatic stages of the disease.

Unlike the other pain-relievers, diclofenac use was associated with a slower cognitive decline as measured by MMSE scores and approached significance with ADAS scores. Though not thoroughly researched, there is evidence that diclofenac is a promising avenue of therapeutic development for AD. Landi et al. 2003[8] performed a cross-sectional study of 2708 community dwelling elderly people. They utilized logistic regression on the proportions of those diagnosed with AD in each NSAID category and found that diclofenac had the greatest effect on risk of AD diagnosis with an odds ratio of 0.21 (95%CI of 0.05 – 0.90). Furthermore, a small underpowered clinical trial was performed by Scharf et al. 1999[31]. This was a single centre trial recruiting mild to moderate AD patients defined by an MMSE of 11 to 25. A

For Review Only

total of 41 patients were recruited and 24 were randomly allocated to the placebo group and 17 to the daily diclofenac treatment group and the patients were followed up for cognitive assessment after six months of treatment [31]. Due to the lack of power of the study and short time span, no strong inferences should be made, however, the trends largely concur with the Landi et al. 2003 study and the present study; the placebo group declined cognitively with a mean MMSE score decline of -0.86 (SD 3.21) and an ADAS increase of 1.93 (SD 5.55), while the diclofenac MMSE score improved on average 0.41 (SD 2.69) and the ADAS stayed relatively stable with a slight increase of 0.25 (SD 4.5)[31]. Given that the other NSAIDs tested in clinical trials did not slow the progression of AD and were not associated with slower decline in the present study despite also being potent inhibitors of the COX enzymes, it is fair to hypothesise that any potential effects of diclofenac on AD are likely not through this mechanism of action. Unlike the other pain-relievers investigated in this research, diclofenac also inhibits the release of the inflammatory cytokine interleukine-1β (IL-1β) (26, supplement 1) by inhibiting the activation of the intracellular receptor NLRP3. The NLRP3 receptor in microglia has been shown to be central to the neuroinflammatory response observed in mouse models of AD[33] and inhibition of the NLRP3 receptor with similar compounds has been found to be therapeutic in several animal models of AD [33,34]. Furthermore, studies have found that the genetic deletion of this receptor completely abated the AD phenotype in mouse models [35,36]. Therefore, NLRP3 inhibition may be the defining feature of diclofenac. However, it should be noted that only 30 subjects were diclofenac consumers with sufficient data for inclusion in the analysis. Therefore, any strong inference of efficacy should be avoided as future research is needed on this promising NSAID.

The results of the analyses presented here found substantial evidence for APOE4 causing accelerated cognitive decline. The lipoprotein APOE4 is a well-established risk factor for the development of AD [10,37,38]. Several studies have also found that APOE4 alleles are associated with accelerated cognitive decline and accelerated cortical tissue atrophy[39-49]. However, a limitation common in the existing literature is the use of multi-level linear modelling for non-Gaussian discrete cognitive scores [40,41,43,45,47,48,50], therefore, the present study, with 744 individuals with at least one APOE4 gene, investigating both ADAS and MMSE measures of cognitive decline and applying discrete distribution GLMM analyses with the negative binomial models, represents a robust and important contribution to the field.

Previous research has reported that NSAIDs only alter AD incidence in APOE4 carriers, suggesting an interaction between the potential therapeutic mechanism of NSAIDs and the pathological mechanisms of APOE4. This was first reported in a thorough study by Szekely *et al.* 2008[10] who looked at AD incidence in 3229 elderly people during a 10 year period. They found that NSAID use was associated with a hazard ratio of 0.88 in non-APOE4 carriers and 0.34 in APOE4 carriers (compared to matched individuals). The present study investigated a three-way interaction of NSAIDs, APOE4 genotype and time (month), and no significant effects were observed (supplement 7.20 & 8.20). This suggests that if the Szekely et al. finding is due to NSAID-APOE4 interactions, and not hidden nuisance variables present in the analysis, then this therapeutic effect may only be useful for the prophylactic treatment to prevent AD in APOE4 individuals and not effective in altering the progression of the disease.

Conclusion

The present study is a thorough investigation into the effects of NSAID and paracetamol use on AD and MCI cognitive decline. Also investigated were the effects of gender, smoking status, headaches, arthritis, diabetes, age, vascular pathology, APOE4 genotype and education level. Due to the discrete nature of

For Review Only

the dependent variables MMSE and ADAS scores, GLMMs were investigated and the negative binomial distribution was found to be a robust approach which outperformed other models. Ibuprofen and aspirin use were associated with improved cognitive performance at baseline, however, neither were associated with an altered cognitive decline. Naproxen and celecoxib use were not associated with any significant alterations in cognitive performance and paracetamol use was associated with accelerated cognitive decline although this effect size was negligible. This suggests that NSAIDs and paracetamol are not promising therapeutics for altering the progression of cognitive decline in MCI and AD individuals. However, diclofenac use was associated with slower cognitive decline, and as this was the only NSAID to do so, this suggests that COX inhibition is not the likely mechanism of action. Therefore, the full interactome of diclofenac should be investigated for potential therapeutic avenues. Collectively, the present study found interesting future avenues of research particularly the effects of paracetamol and diclofenac on AD progression and improved the evidence for our existing understanding of factors which effect AD such as the APOE4 genotype by applying innovative statistical methods.

Acknowledgements Section

Data collection and sharing for this project was funded by the Alzheimer's Disease Neuroimaging Initiative (ADNI) (National Institutes of Health Grant U01 AG024904) and DOD ADNI (Department of Defense award number W81XWH-12-2-0012). ADNI is funded by the National Institute on Aging, the National Institute of Biomedical Imaging and Bioengineering, and through generous contributions from the following: AbbVie, Alzheimer’s Association; Alzheimer’s Drug Discovery Foundation; Araclon Biotech; BioClinica, Inc.; Biogen; Bristol-Myers Squibb Company; CereSpir, Inc.; Cogstate; Eisai Inc.; Elan Pharmaceuticals, Inc.; Eli Lilly and Company; EuroImmun; F. Hoffmann-La Roche Ltd and its affiliated company Genentech, Inc.; Fujirebio; GE Healthcare; IXICO Ltd.; Janssen Alzheimer Immunotherapy Research & Development, LLC.; Johnson & Johnson Pharmaceutical Research & Development LLC.; Lumosity; Lundbeck; Merck & Co., Inc.; Meso Scale Diagnostics, LLC.; NeuroRx Research; Neurotrack Technologies; Novartis Pharmaceuticals Corporation; Pfizer Inc.; Piramal Imaging; Servier; Takeda Pharmaceutical Company; and Transition Therapeutics. The Canadian Institutes of Health Research is providing funds to support ADNI clinical sites in Canada. Private sector contributions are facilitated by the Foundation for the National Institutes of Health (www.fnih.org). The grantee organization is the Northern California Institute for Research and Education, and the study is coordinated by the Alzheimer’s Therapeutic Research Institute at the University of Southern California. ADNI data are disseminated by the Laboratory for Neuro Imaging at the University of Southern California. AEM is a Food Standards Agency Fellow and is supported by the Biotechnology and Biological Sciences Research Council (BBSRC) Institute Strategic Programme Microbes in the Food Chain BB/R012504/1 and its constituent projects BBS/E/F/000PR10348 (Theme 1, Epidemiology and Evolution of Pathogens in the Food Chain) and BBS/E/F/000PR10351 (Theme 3, Microbial Communities in the Food Chain). JRA was a Future Leader Fellow supported by the BBSRC fellowship grant titled Understanding how dietary zinc and inflammation impact healthy ageing in the brain (BB/P01061X/1).

References

1. Wattmo C, Londos E, Minthon L. Risk Factors That Affect Life Expectancy in Alzheimer's Disease: A 15-Year Follow-Up. *Dementia and Geriatric Cognitive Disorders* 2014; **38**(5-6): 286-99.

2. Alzheimers A. Alzheimer's Association Report 2015 Alzheimer's disease facts and figures. *Alzheimers & Dementia* 2015; **11**(3): 332-84.

3. Conaghan PG. A turbulent decade for NSAIDs: update on current concepts of classification, epidemiology, comparative efficacy, and toxicity. *Rheumatology International* 2012; **32**(6): 1491-502.

For Review Only

4. Breitner JCS, Welsh KA, Helms MJ, et al. DELAYED-ONSET OF ALZHEIMERS-DISEASE WITH NONSTEROIDAL ANTIINFLAMMATORY AND HISTAMINE-H2 BLOCKING-DRUGS. *Neurobiology of Aging* 1995; **16**(4): 523-30.

5. Cote S, Carmichael PH, Verreault R, Lindsay J, Lefebvre J, Laurin D. Nonsteroidal anti-inflammatory drug use and the risk of cognitive impairment and Alzheimer's disease. *Alzheimers Dement* 2012; **8**(3): 219-26.

6. Fischer P, Zehetmayer S, Jungwirth S, et al. Risk factors for Alzheimer dementia in a community-based birth cohort at the age of 75 years. *Dement Geriatr Cogn Disord* 2008; **25**(6): 501-

7. in 't Veld BA, Ruitenberg A, Hofman A, et al. Nonsteroidal antiinflammatory drugs and the risk of Alzheimer's disease. *New England Journal of Medicine* 2001; **345**(21): 1515-21.

8. Landi F, Cesari M, Onder G, Russo A, Torre S, Bernabei R. Non-Steroidal Anti-Inflammatory Drug (NSAID) Use and Alzheimer Disease in Community-Dwelling Elderly Patients. *The American Journal of Geriatric Psychiatry* 2003; **11**(2): 179-85.

9. Stewart WF, Kawas C, Corrada M, Metter EJ. Risk of Alzheimer's disease and duration of NSAID use. *Neurology* 1997; **48**(3): 626-32.

10. Szekely CA, Breitner JC, Fitzpatrick AL, et al. NSAID use and dementia risk in the Cardiovascular Health Study: role of APOE and NSAID type. *Neurology* 2008; **70**(1): 17-24.

11. Vlad SC, Miller DR, Kowall NW, Felson DT. Protective effects of NSAIDs on the development of Alzheimer disease. *Neurology* 2008; **70**(19): 1672-7.

12. Yan Q, Zhang JH, Liu HT, et al. Anti-inflammatory drug therapy alters beta-amyloid processing and deposition in an animal model of Alzheimer's disease. *Journal of Neuroscience* 2003; **23**(20): 7504-9.

13. Lim GP, Yang F, Chu T, et al. Ibuprofen suppresses plaque pathology and inflammation in a mouse model for Alzheimer's disease. *Journal of Neuroscience* 2000; **20**(15): 5709-14.

14. Weggen S, Eriksen JL, Das P, et al. A subset of NSAIDs lower amyloidogenic A beta 42 independently of cyclooxygenase activity. *Nature* 2001; **414**(6860): 212-6.

15. Pasqualetti P, Bonomini C, Dal Forno G, et al. A randomized controlled study on effects of ibuprofen on cognitive progression of Alzheimer's disease. *Aging Clin Exp Res* 2009; **21**(2): 102-10.

16. Soininen H, West C, Robbins J, Niculescu L. Long-term efficacy and safety of celecoxib in Alzheimer's disease. *Dementia and Geriatric Cognitive Disorders* 2007; **23**(1): 8-21.

17. Reines SA, Block GA, Morris JC, et al. Rofecoxib - No effect on Alzheimer's disease in a 1-year, randomized, blinded, controlled study. *Neurology* 2004; **62**(1): 66-71.

For Review Only

18. Green RC, Schneider LS, Amato DA, et al. Effect of Tarenflurbil on Cognitive Decline and Activities of Daily Living in Patients With Mild Alzheimer Disease A Randomized Controlled Trial. *Jama-Journal of the American Medical Association* 2009; **302**(23): 2557-64.

19. Imbimbo BP, Solfrizzi V, Panza F. Are NSAIDs useful to treat Alzheimer's disease or mild cognitive impairment? *Frontiers in Aging Neuroscience* 2010; **2**.

20. Szekely CA, Zandi PP. Non-Steroidal Anti-Inflammatory Drugs and Alzheimer's Disease: The Epidemiological Evidence. *Cns & Neurological Disorders-Drug Targets* 2010; **9**(2): 132-9.

21. Wyss-Coray T. Inflammation in Alzheimer disease: driving force, bystander or beneficial response? *Nature Medicine* 2006; **12**(9): 1005-15.

22. Bolker BM, Brooks ME, Clark CJ, et al. GLMMs in action: gene-by-environment interaction in total fruit production of wild populations of Arabidopsis thaliana Revised version, part 2. *See http://glmm wdfiles com/local-files/examples/Banta_2011_part1 pdf* 2011.

23. Hardin JW, Hilbe JM, Hilbe J. Generalized linear models and extensions: Stata press; 2007.

24. R Core Team (2018). R: A language and environment for statistical computing. R Foundation for Statistical Computing, Vienna, Austria. URL https://www.R-project.org/.

25. Fournier DA, Skaug HJ, Ancheta J, et al. AD Model Builder: usingautomatic differentiation for statistical inference of highly parameterized complex nonlinear models. *Optim Methods Softw* 2012; **27**: 233-49.

26. Skaug H, Fournier D, Nielsen A, Magnusson A, Bolker B. Generalized Linear Mixed Models using AD Model Builder. *R package version 075* 2013.

27. RStudio Team. (2016) RStudio: Integrated Development for R. *RStudio, Inc, Boston, MA URL http://wwwrstudiocom*.

28. Hosmer JDW, Lemeshow S, Sturdivant RX. Model-Building Strategies and Methods for Logistic Regression. Applied Logistic Regression: John Wiley & Sons, Inc.; 2013: 89-151.

29. Results of a follow-up study to the randomized Alzheimer's Disease Anti-inflammatory Prevention Trial (ADAPT). *Alzheimers Dement* 2013; **9**(6): 714-23.

30. Lyketsos CG, Breitner JC, Green RC, et al. Naproxen and celecoxib do not prevent AD in early results from a randomized controlled trial. *Neurology* 2007; **68**(21): 1800-8.

31. Scharf S, Mander A, Ugoni A, Vajda F, Christophidis N. A double-blind, placebo-controlled trial of diclofenac/misoprostol in Alzheimer's disease. *Neurology* 1999; **53**(1): 197-201.

For Review Only

32. Shrank WH, Patrick AR, Brookhart MA. Healthy user and related biases in observational studies of preventive interventions: a primer for physicians. *Journal of general internal medicine* 2011; **26**(5): 546-50.

33. Daniels MJD, Rivers-Auty J, Schilling T, et al. Fenamate NSAIDs inhibit the NLRP3 inflammasome and protect against Alzheimer's disease in rodent models. *Nature Communications* 2016; **7**.

34. Dempsey C, Rubio Araiz A, Bryson KJ, et al. Inhibiting the NLRP3 inflammasome with MCC950 promotes non-phlogistic clearance of amyloid-beta and cognitive function in APP/PS1 mice. *Brain, behavior, and immunity* 2017; **61**: 306-16.

35. Dostert C, Petrilli V, Van Bruggen R, Steele C, Mossman BT, Tschopp J. Innate immune activation through Nalp3 inflammasome sensing of asbestos and silica. *Science* 2008; **320**(5876): 674-7.

36. Heneka MT, Kummer MP, Stutz A, et al. NLRP3 is activated in Alzheimer's disease and contributes to pathology in APP/PS1 mice. *Nature* 2013; **493**(7434): 674-+.

37. Notkola IL, Sulkava R, Pekkanen J, et al. Serum total cholesterol, apolipoprotein E epsilon 4 allele, and Alzheimer's disease. *Neuroepidemiology* 1998; **17**(1): 14-20.

38. Cornelius C, Fastbom J, Winblad B, Viitanen M. Aspirin, NSAIDs, risk of dementia, and influence of the apolipoprotein E epsilon 4 allele in an elderly population. *Neuroepidemiology* 2004; **23**(3): 135-43.

39. Bartzokis G, Lu PH, Geschwind DH, Edwards N, Mintz J, Cummings JL. Apolipoprotein E genotype and age-related myelin breakdown in healthy individuals - Implications for cognitive decline, and dementia. *Archives of General Psychiatry* 2006; **63**(1): 63-72.

40. Lo RY, Hubbard AE, Shaw LM, et al. Longitudinal Change of Biomarkers in Cognitive Decline. *Archives of Neurology* 2011; **68**(10): 1257-66.

41. Mielke MM, Leoutsakos J-M, Tschanz JT, et al. Interaction between vascular factors and the APOE ε4 allele in predicting rate of progression in Alzheimer's disease. *Journal of Alzheimer's disease : JAD* 2011; **26**(1): 127-34.

42. Morra JH, Tu Z, Apostolova LG, et al. Automated mapping of hippocampal atrophy in 1-year repeat MRI data from 490 subjects with Alzheimer's disease, mild cognitive impairment, and elderly controls. *Neuroimage* 2009; **45**(1): S3-S15.

43. Rawle MJ, Davis D, Bendayan R, Wong A, Kuh D, Richards M. Apolipoprotein-E (Apoe) ε4 and cognitive decline over the adult life course. *Translational Psychiatry* 2018; **8**(1): 18.

44. Tilvis RS, Kahonen-Vare MH, Jolkkonen J, Valvanne J, Pitkala KH, Strandberg TE. Predictors of cognitive decline and mortality of aged people over a 10-year period. *Journals of Gerontology Series a-Biological Sciences and Medical Sciences* 2004; **59**(3): 268-74.

For Review Only

45. Whitehair DC, Sherzai A, Emond J, et al. Influence of apolipoprotein E varepsilon4 on rates of cognitive and functional decline in mild cognitive impairment. *Alzheimer's & dementia : the journal of the Alzheimer's Association* 2010; **6**(5): 412-9.

46. Young AL, Oxtoby NP, Daga P, et al. A data-driven model of biomarker changes in sporadic Alzheimer's disease. *Brain* 2014; **137**: 2564-77.

47. Kim YJ, Seo SW, Park SB, et al. Protective effects of APOE e2 against disease progression in subcortical vascular mild cognitive impairment patients: A three-year longitudinal study. *Sci Rep* 2017; **7**(1): 1910.

48. Kanai M, Shizuka M, Urakami K, et al. Apolipoprotein E4 accelerates dementia and increases cerebrospinal fluid tau levels in Alzheimer's disease. *Neuroscience Letters* 1999; **267**(1): 65-8.

49. Vijayaraghavan S, Darreh-Shori T, Rongve A, et al. Association of Butyrylcholinesterase-K Allele and Apolipoprotein E varepsilon4 Allele with Cognitive Decline in Dementia with Lewy Bodies and Alzheimer's Disease. *Journal of Alzheimer's disease : JAD* 2016; **50**(2): 567-76.

50. Vemuri P, Lesnick TG, Przybelski SA, et al. Association of Lifetime Intellectual Enrichment With Cognitive Decline in the Older Population. *Jama Neurology* 2014; **71**(8): 1017-24.

For Review Only

Table 1: Baseline statistics of ADNI cohort by pain-reliever use.

	Aspirin	Celecoxib	Diclofenac	Ibuprofen	Naproxen	Paracetamol	No analgesic	Statistics
Diagnosis								
CN	247 (29%)	19 (30%)	14 (47%)	79 (31%)	52 (28%)	119 (30%)	99 (21%)	
EMCI	174 (20%)	13 (20%)	5 (17%)	55 (22%)	45 (24%)	75 (19%)	67 (14%)	X^2(18)=78.1 p<0.0001
LMCI	302 (35%)	25 (39%)	8 (27%)	92 (36%)	68 (37%)	140 (35%)	165 (35%)	
AD	138 (16%)	7 (10%)	3 (10%)	29 (11%)	21 (11%)	66 (17%)	141 (30%)	
ADAS	16.4 (9.0)***	15.8 (8.3) ^{ns}	12.3 (8.5)*	15.0 (8.5)***	15.4 (8.9) ^{ns}	16.2 (8.8)^{ns}	19.7 (10.3)	X^2(6)=57.5 p<0.0001
MMSE	27.3 (2.6)***	27.2 (2.6) ^{ns}	28.0 (2.4) ^{ns}	27.7 (2.2)***	27.6 (2.4) ^{ns}	27.4 (2.5)^{ns}	26.4 (2.8)	X^2(6)=48.7 p<0.0001
Gender								
Male	323 (38%)	31 (48%)	15 (50%)	117 (46%)	95 (51%)	204 (51%)	223 (47%)	X^2(6)=29.5 p<0.0001
Female	538 (62%)	33 (52%)	15 (50%)	138 (54%)	91 (49%)	196 (49%)	249 (53%)	
Age	74.3 (6.7) ^{ns}	73.4 (6.9)^{ns}	75.22 (6.4)^{ns}	72.7 (6.8)*	72.9 (7.1) ^{ns}	74.3 (7.2) ^{ns}	73.9 (8.1)	F(6)=3.7 P=0.001
APOE4								
-/-	467 (54%)	35 (54%)	18 (60%)	145 (57%)	102 (55%)	231 (58%)	236 (50%)	X^2(12)=7.5 p=0.822
+/-	305 (35%)	24 (38%)	10 (33%)	88 (35%)	67 (36%)	133 (33%)	184 (39%)	
+/+	89 (10%)	5 (8%)	2 (7%)	22 (9%)	17 (9%)	36 (9%)	52 (11%)	
Education								
Primary	318 (37%)	21 (33%)	10 (33%)	83 (33%)	56 (30%)	128 (32%)	161 (34%)	
Secondary	251 (29%)	18 (28%)	11 (37%)	77 (30%)	59 (32%)	119 (30%)	126 (27%)	X^2(18)=15.7 p=0.613
Tertiary	166 (19%)	12 (19%)	3 (19%)	50 (20%)	40 (22%)	92 (23%)	92 (19%)	
Post-grad	126 (15%)	13 (20%)	6 (20%)	45 (18%)	31 (17%)	61 (15%)	93 (19%)	
Headache	67 (8%)	11 (17%)	4 (13%)	31 (12%)	20 (11%)	52 (13%)	33 (7%)	X^2(6)=19.2 p=0.004
Arthritis	342 (40%)	47 (73%)	23 (77%)	127 (50%)	98 (53%)	215 (54%)	132 (28%)	X^2(6)=19.2 P<0.001
Diabetes	92 (7%)	7 (7%)	4 (9%)	26 (7%)	21 (8%)	46 (7%)	33 (4.7%)	X^2(6)=6.9 p=0.330
Smoker	205 (24%)	17 (27%)	8 (27%)	71 (28%)	40 (22%)	107 (27%)	127 (27%)	X^2(6)=4.5 p=0.614
Cardiovascular risk factors	555 (64%)	44 (69%)	16 (53%)	158 (62%)	114 (61%)	273 (68%)	261 (55%)	X^2(6)=19.8 p=0.003
Total	861	64	30	255	186	400	472	

All data are in N(%) except for ADAS, MMSE or Age which are expressed as mean (SD). CN=cognitively normal, EMCI and LMCI = early and late mild cognitive impairment, AD=Alzheimer's disease, ADAS= Alzheimer's disease assessment scale, MMSE=mini-mental state examination. Statistical analyses are Chi-squared test for proportions, maximum likelihood generalised linear modelling for score data, or general linear modelling for parametric data.

For Review Only

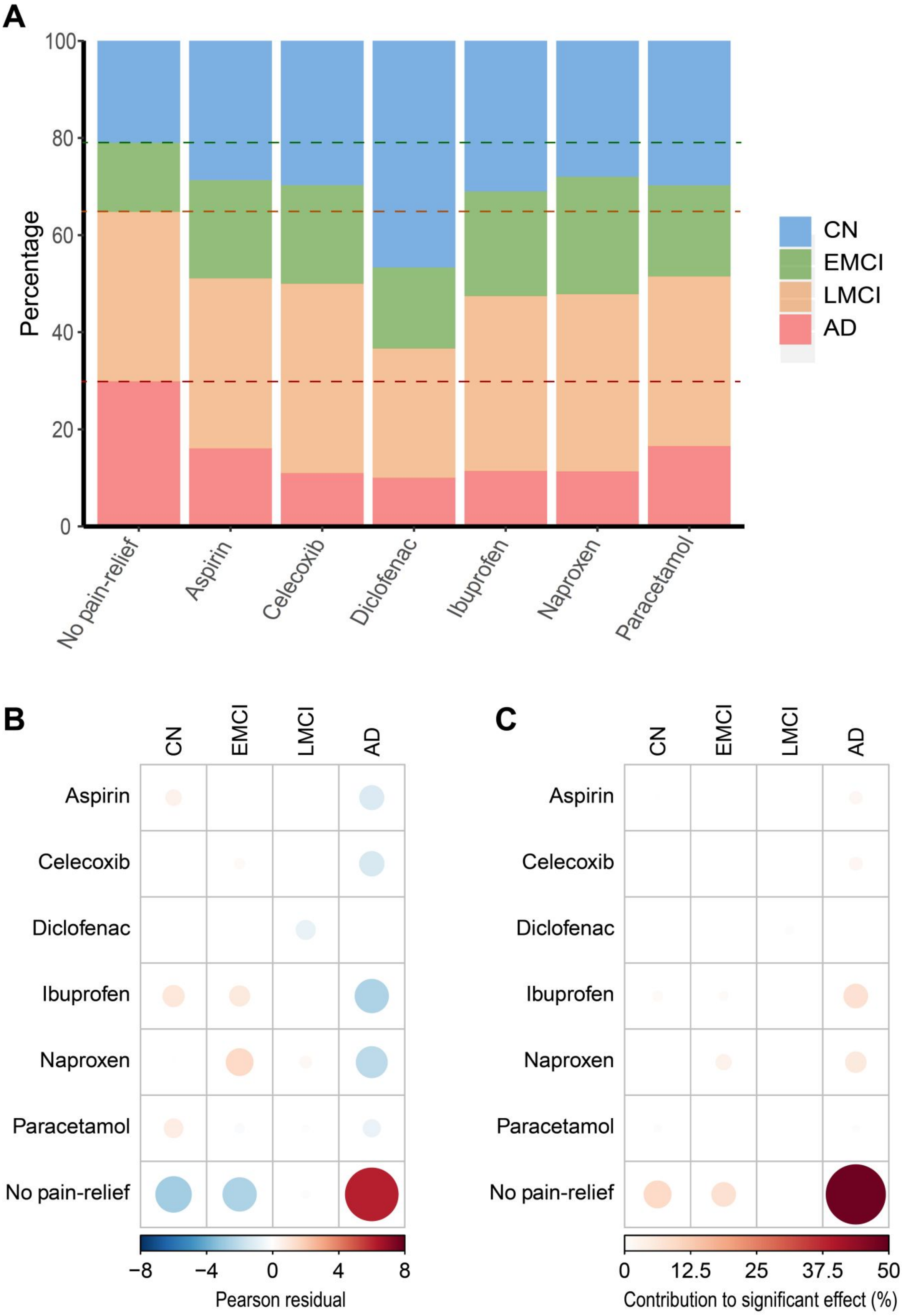

*Figure 1: At baseline, use of any pain-reliever was associated with lower prevalence of Alzheimer's disease (AD) and a corresponding higher prevalence of cognitively normal (CN). **A**) Proportion of cognitive diagnosis between pain reliever groups. Dotted lines show proportion divisions of no pain-reliever group. **B**) Pearson residuals demonstrate the size and direction of the effect of pain-reliever use on cognitive diagnosis. **C**) Contribution analysis of the significant association of pain-reliever subgroup and cognitive diagnosis reveals little difference between pain-reliever and the largest contribution to the significant effect is the No pain-relief group having a higher prevalence of AD and a lower prevalence of CN, compared to the other groups. CN=cognitively normal, EMCI and LMCI = early and late mild cognitive impairment, AD=Alzheimer's disease, ADAS= Alzheimer's disease assessment scale, MMSE=mini-mental state examination.*

Table 2: Summary of the final negative binomial GLMM with MMSE failures as the dependent variable.

MMSE	Estimate	Stand Error	Z value	p-value	Statistics of inclusion
Intercept	-0.588	0.063	-9.33	p <0.00001	-
**Fixed effects**					
Diagnosis					
CN	-	-	-	-	$\chi^2(3)=1147.2$, p<0.00001
EMCI	0.708	0.066	12	p <0.00001	
LMCI	1.242	0.056	22.38	p <0.00001	
AD	2.11	0.062	33.9	p <0.00001	
Gender					
Female	-	-	-	-	$\chi^2(1)=2.2$, p=0.138010
Male	0.096	0.040	2.36	p =0.01806	
Age	0.018	0.003	6.96	p <0.00001	$\chi^2(1)=48.2$, p<0.00001
APOE4					
-/-	-	-	-	-	$\chi^2(2)=60.6$, p<0.00001
+/-	0.121	0.042	2.84	p =0.00446	
+/+	0.226	0.065	3.49	p =0.00048	
Education					
Primary	0.352	0.057	6.14	p <0.00001	
Secondary	0.362	0.056	6.5	p <0.00001	$\chi^2(3)=47.4$, p<0.00001
Tertiary	0.2	0.050	4.04	p =0.00005	
Post-grad	-	-	-	-	
Aspirin	-0.075	0.037	-2.02	p =0.04345	$\chi^2(1)=4.0$, p=0.045500
Paracetamol	-0.093	0.045	-2.07	p =0.03882	$\chi^2(1)=1.8$, p=0.179712
Diclofenac	0.023	0.146	0.015	p =0.87712	$\chi^2(1)=1.0$, p=0.317311
**Interaction with time (months)**					
Diagnosis					
CN	-	-	-	-	$\chi^2(3)=79.8$, p<0.00001
EMCI	-0.00332	0.0010	-3.18	p =0.00145	
LMCI	0.00286	0.0007	3.95	p =0.00008	
AD	0.00880	0.0014	6.12	p <0.00001	
Gender					
Female	-	-	-	-	$\chi^2(2)=8.4$, p=0.003752
Male	-0.00172	0.0006	-2.92	p =0.00355	
APOE4					
-/-	-	-	-	-	$\chi^2(2)=120.6$, p<0.00001
+/-	0.00588	0.0006	9.52	p <0.00001	
+/+	0.00777	0.0009	8.32	p <0.00001	
Education					
Primary	-0.00250	0.0008	-3.06	p =0.00224	
Secondary	-0.00226	0.0008	-2.79	p =0.00532	$\chi^2(3)=26.6$, p<0.00001
Tertiary	-0.00375	0.0007	-5.05	p <0.00001	
Post-grad	-	-	-	-	
Paracetamol	0.00129	0.0006	2.18	p =0.02928	$\chi^2(1)=4.8$, p=0.0284597
Diclofenac	-0.00468	0.0016	-2.89	p =0.00380	$\chi^2(1)=8.4$, p=0.0037522

Shown are the maximum likelihood estimates with Laplace estimates of the standard error, Z- value and p-value from the Wald approximation, as well as, the significance of inclusion of the variable in the model evaluated using the log-likelihood ratio test chi squared. CN=cognitively normal, EMCI and LMCI = early and late mild cognitive impairment, AD=Alzheimer's disease, ADAS= Alzheimer's disease assessment scale, MMSE=mini-mental state examination.

For Review Only

Table 3: Summary of the final negative binomial GLMM with ADAS score as the dependent variable.

ADAS	Estimate	Stand Error	Z value	p-value	Statistics of inclusion
Intercept	2.991	0.032	92.09	p <0.00001	-
Fixed effects					
Diagnosis					
CN	-	-	-	-	$\chi^2(3)$=1124.4, p<0.00001
EMCI	0.357	0.035	10.18	p <0.00001	
LMCI	0.771	0.03	25.69	p <0.00001	
AD	1.248	0.035	35.84	p <0.00001	
Gender					
Female	-	-	-	-	$\chi^2(1)$=7.4, p=0.006524
Male	0.106	0.023	4.6	p <0.00001	
Age	0.0133	0.003	8.52	p <0.00001	$\chi^2(1)$=71.4, p<0.00001
APOE4					
-/-	-	-	-	-	$\chi^2(2)$=63.6, p<0.00001
+/-	0.096	0.024	3.92	p <0.00001	
+/+	0.149	0.039	3.86	p = 0.0011	
Education					
Primary	0.181	0.033	5.41	p <0.00001	$\chi^2(3)$=26.2, p=0.00009
Secondary	0.148	0.032	4.66	p <0.00001	
Tertiary	0.105	0.028	3.72	p =0.00020	
Post-grad	-	-	-	-	
Headache	-0.087	0.039	-2.23	p =0.02563	$\chi^2(1)$=5.0, p=0.025347
Paracetamol	-0.035	0.026	-1.35	p =0.17759	$\chi^2(1)$=0.8, p=0.371093
Ibuprofen	-0.093	0.030	-3.14	p =0.00170	$\chi^2(1)$=9.8, p=0.0017451
Diclofenac	-0.224	0.080	-2.8	p =0.00518	$\chi^2(1)$=7.8, p=0.0052246
Interaction with time (months)					
Diagnosis					
CN	-	-	-	-	$\chi^2(3)$=65.4, p<0.00001
EMCI	-0.00162	0.0004	-3.98	p =0.00007	
LMCI	0.00021	0.0003	0.74	p =0.46177	
AD	0.00448	0.0007	6.17	p <0.00001	
Gender					
Female	-	-	-	-	$\chi^2(2)$=67.0, p<0.00001
Male	-0.00210	0.0003	-8.22	p <0.00001	
APOE4					
-/-	-	-	-	-	$\chi^2(2)$=186.2, p<0.00001
+/-	0.00298	0.0003	11.31	p <0.00001	
+/+	0.00476	0.0004	10.61	p <0.00001	
Education					
Primary	-0.00135	0.0004	-3.74	p =0.00019	$\chi^2(3)$=50.6, p<0.00001
Secondary	-0.00149	0.0003	-4.29	p =0.00002	
Tertiary	-0.00214	0.0003	-6.91	p <0.00001	
Post-grad	-	-	-	-	
Paracetamol	0.00056	0.0003	-2.21	p <0.00001	$\chi^2(1)$=4.8, p=0.0284597

Shown are the maximum likelihood estimates with Laplace estimates of the standard error, Z- value and p-value from the Wald approximation, as well as, the significance of inclusion of the variable in the model evaluated using the log-likelihood ratio test chi squared. CN=cognitively normal, EMCI and LMCI = early and late mild cognitive impairment, AD=Alzheimer's disease, ADAS= Alzheimer's disease assessment scale, MMSE=mini-mental state examination.

For Review Only

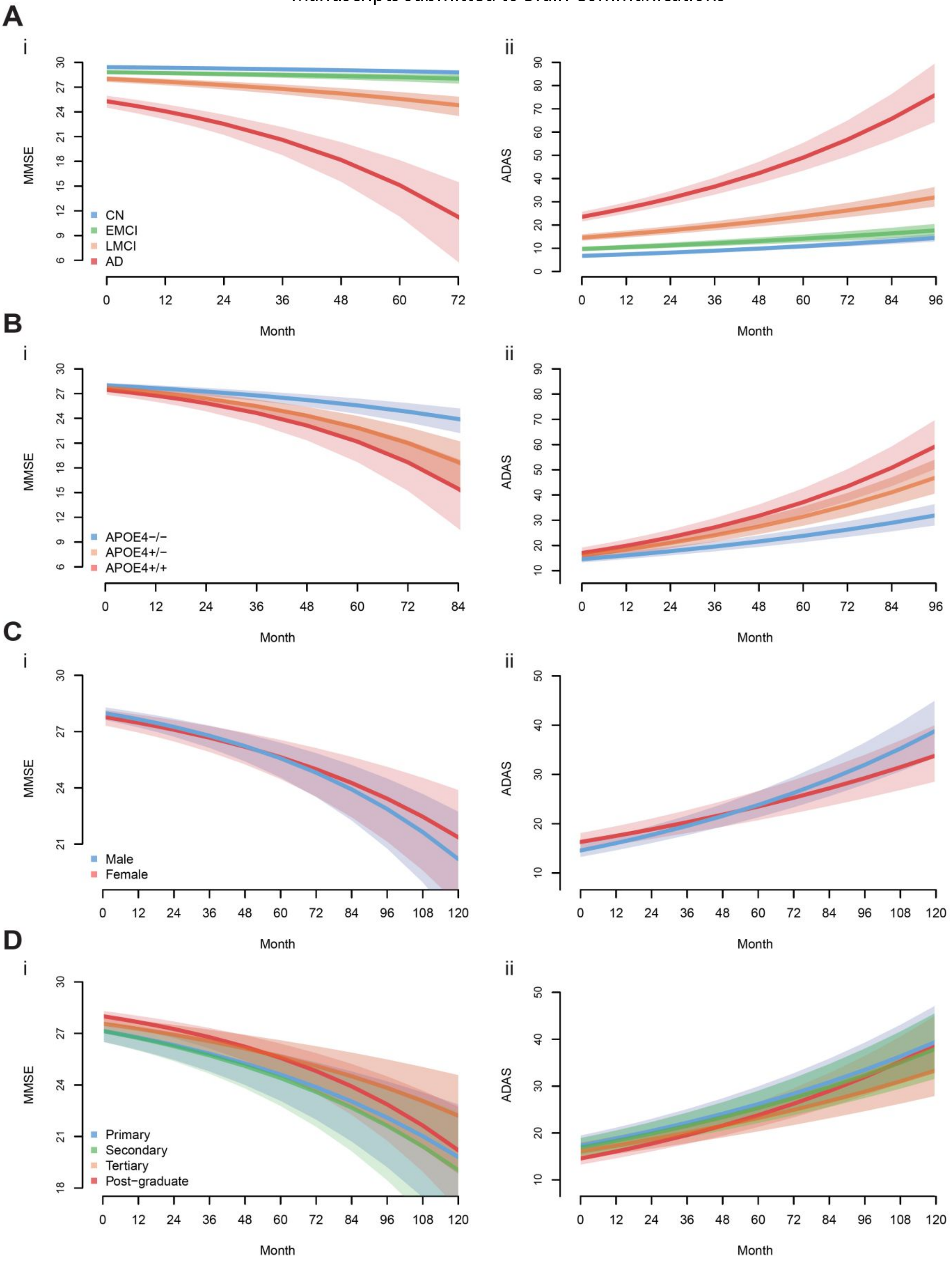

Figure 2: ***The effect of significant explanatory variables on predicted cognitive decline as measure by MMSE (i) and ADAS (ii) scores.*** *These models show the predicted decline of a LMCI (except A), Female (except C), 70 year old, and how this decline changes when additional variables are included. Cognitive diagnosis was predictably the most influential variable, with Alzheimer's disease (AD) and late mild cognitive impairment (LMCI) associated with rapid cognitive decline; early MCI (EMCI) and cognitively normal (CN) did not have appreciatively different rates of decline (**A**). APOE4 showed a very strong gene dose association with accelerated cognitive decline (**B**). Gender showed mild differences with males having faster cognitive decline (**C**). Education had variable effects on cognitive decline with tertiary level education associated with the slowest decline (**D**).Lines are predicted value, shaded area are 95% CI.*

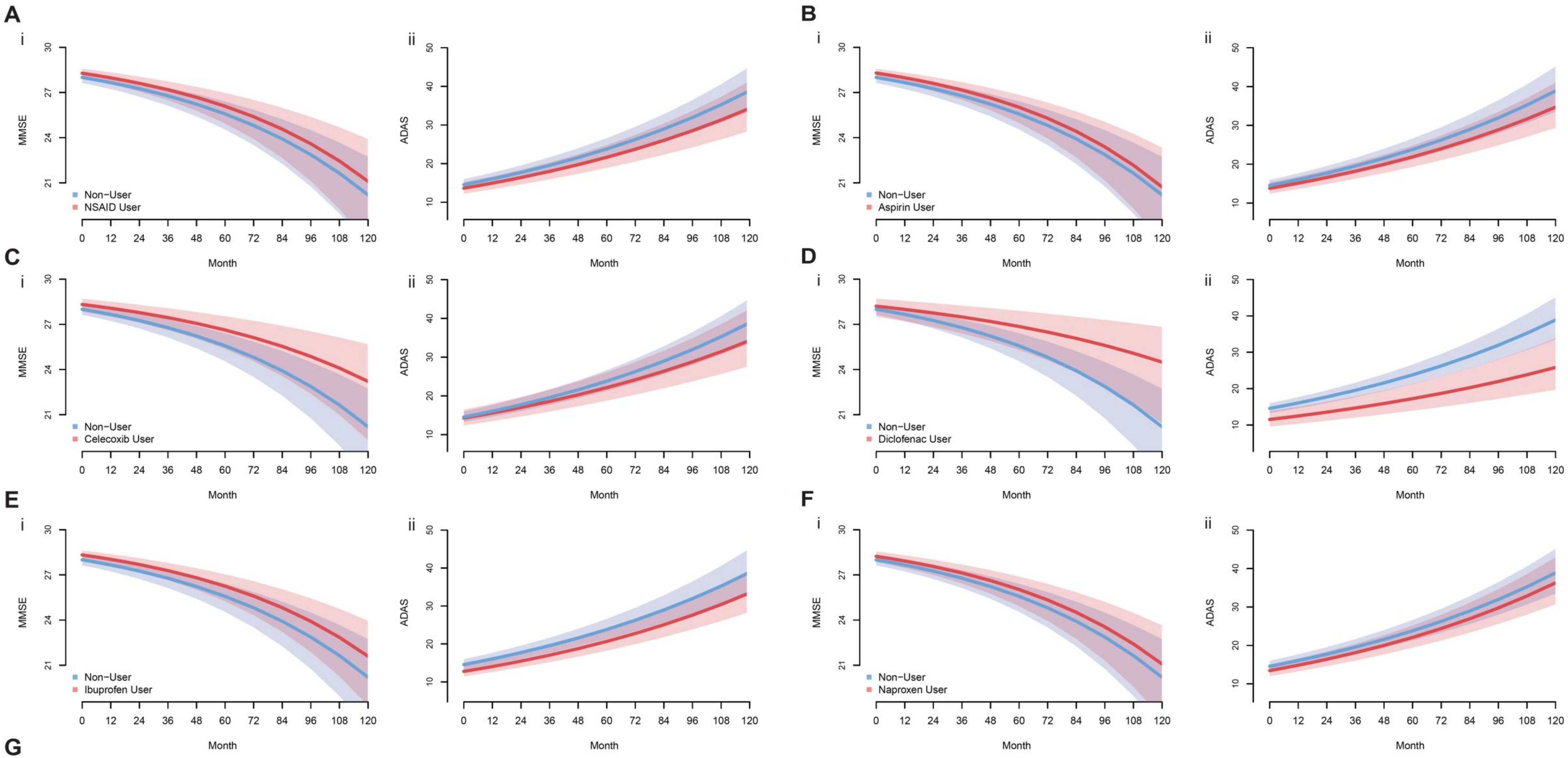

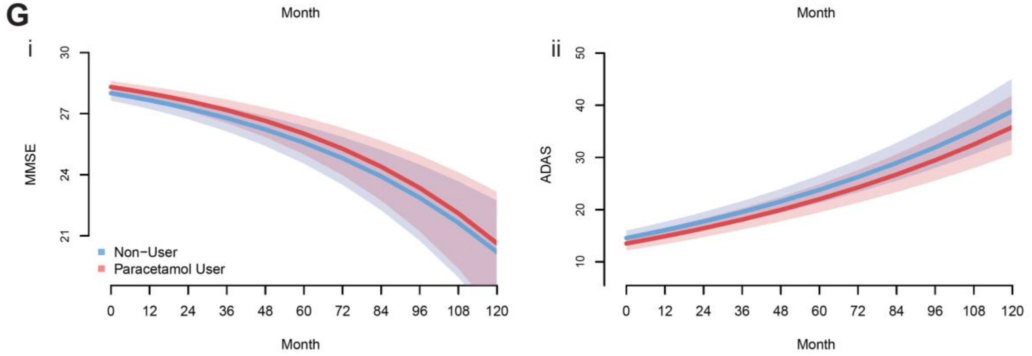

Figure 3: ***The effect of pain-reliever use on predicted cognitive decline as measure by MMSE (i) and ADAS (ii) scores.*** *These models show the predicted decline of a LMCI, Female 70 year old, and how this decline changes when pain-relief use is included in the model. Any NSAID use (**A**); Aspirin (**B**); Celecoxib (**C**); Diclofenac (**D**); Ibuprofen (**E**); Naproxen (**F**); Paracetamol (**G**); Lines are predicted value, shaded area are 95% CI. LMCI = late mild cognitive impairment, ADAS= Alzheimer's disease assessment scale, MMSE=mini-mental state examination.*

For Review Only

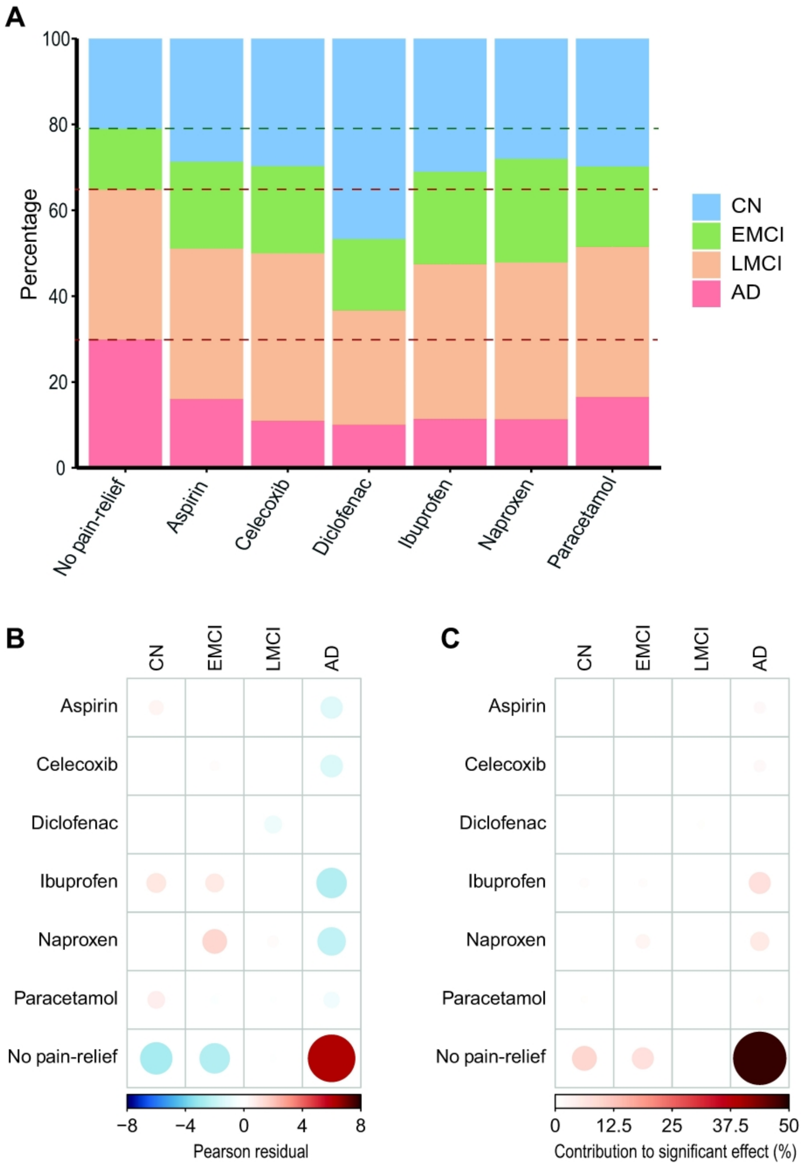

Figure 1: At baseline, use of any pain-reliever was associated with lower prevalence of Alzheimer's disease (AD) and a corresponding higher prevalence of cognitively normal (CN). A) Proportion of cognitive diagnosis between pain reliever groups. Dotted lines show proportion divisions of no pain-reliever group. B) Pearson residuals demonstrate the size and direction of the effect of pain-reliever use on cognitive diagnosis. C) Contribution analysis of the significant association of pain-reliever subgroup and cognitive diagnosis reveals little difference between pain-reliever and the largest contribution to the significant effect is the No pain-relief group having a higher prevalence of AD and a lower prevalence of CN, compared to the other groups. CN=cognitively normal, EMCI and LMCI = early and late mild cognitive impairment, AD=Alzheimer's disease, ADAS= Alzheimer's disease assessment scale, MMSE=mini-mental state examination.

187x270mm (600 x 600 DPI)

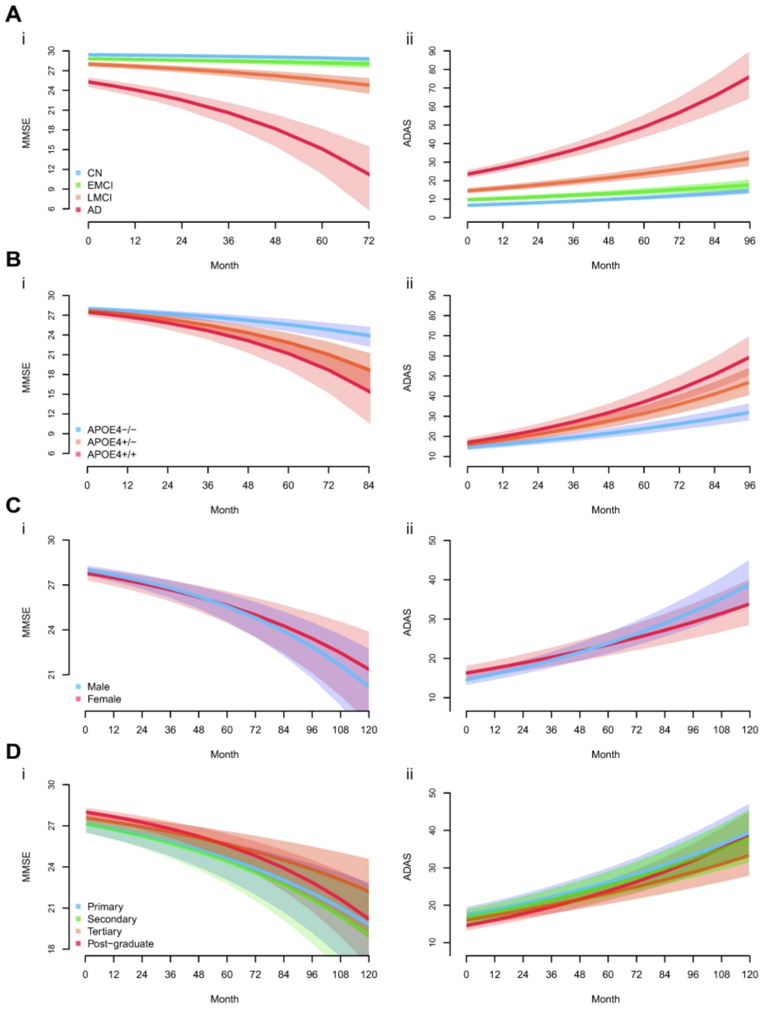

Figure 2: The effect of significant explanatory variables on predicted cognitive decline as measure by MMSE (i) and ADAS (ii) scores. These models show the predicted decline of a LMCI (except A), Female (except C), 70 year old, and how this decline changes when additional variables are included. Cognitive diagnosis was predictably the most influential variable, with Alzheimer's disease (AD) and late mild cognitive impairment (LMCI) associated with rapid cognitive decline; early MCI (EMCI) and cognitively normal (CN) did not have appreciatively different rates of decline (A). APOE4 showed a very strong gene dose association with accelerated cognitive decline (B). Gender showed mild differences with males having faster cognitive decline (C). Education had variable effects on cognitive decline with tertiary level education associated with the slowest decline (D).Lines are predicted value, shaded area are 95% CI.

210x297mm (600 x 600 DPI)

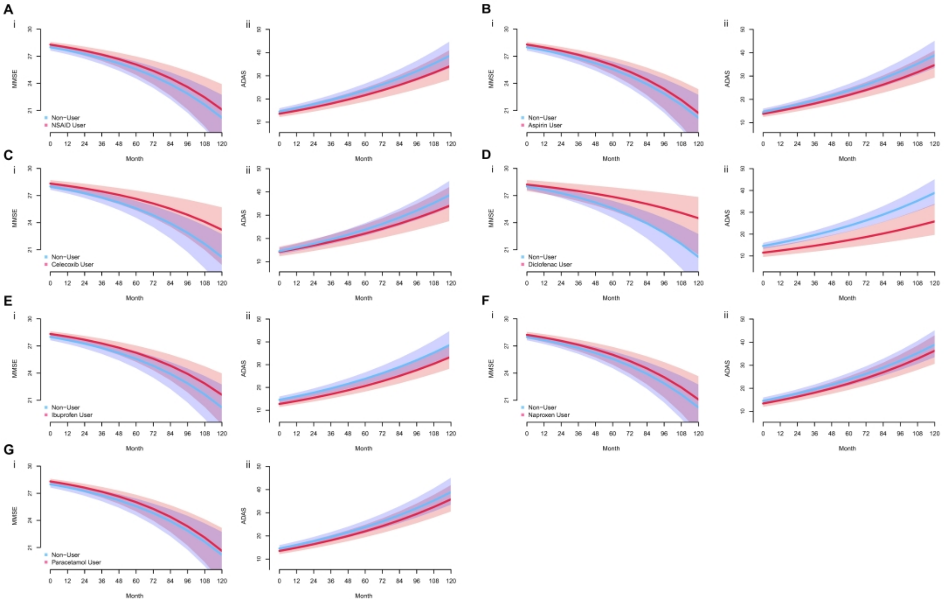

Figure 3: The effect of pain-reliever use on predicted cognitive decline as measure by MMSE (i) and ADAS (ii) scores. These models show the predicted decline of a LMCI, Female 70 year old, and how this decline changes when pain-relief use is included in the model. Any NSAID use (A); Aspirin (B); Celecoxib (C); Diclofenac (D); Ibuprofen (E); Naproxen (F); Paracetamol (G); Lines are predicted value, shaded area are 95% CI. LMCI = late mild cognitive impairment, ADAS= Alzheimer's disease assessment scale, MMSE=mini-mental state examination.

409x303mm (600 x 600 DPI)

Supplement

Contents

1. Cell culture experiment demonstrating differential effects of pain-relievers on IL-1β release 9
2. Additional methods outline 10
   - 2.1. Brief outline of the methods 10
   - 2.2. Preliminary distribution analysis 11
   - 2.3. Model construction 12
     - 2.3.1. Selecting parameterisation method 12
     - 2.3.2. Building of initial main effect model 12
     - 2.3.3. Inclusion of biologically relevant two-way interaction terms 13
   - 2.4. Assumption check of residuals 13
   - 2.5. Building final model with different distribution families 13
3. Additional results summary 14
4. Packages used 14
5. Data Cleaning 15
   - 5.2. Recurrent medicine data set 15
     - 5.2.1. Data Cleaning Oral Route only 15
     - 5.2.2. Data Cleaning Diclofenac Spelling 15
     - 5.2.3. Data Cleaning Aspirin Spelling 16
     - 5.2.4. Data Cleaning Paracetamol Spelling 16
     - 5.2.5. Data Cleaning Ibuprofen Spelling 17
     - 5.2.6. Data Cleaning Naproxen Spelling 17
     - 5.2.7. Data Cleaning Indomethacin Spelling 18
     - 5.2.8. Data Cleaning Celecoxib Spelling 18
   - 5.3. Combing drug data frame with outcome variable data frame 19
   - 5.4. Searching for confounding preexisting condition 19
     - 5.4.1. Diabetes 19
     - 5.4.2. Cardiovascular 20
     - 5.4.3. Arthritis 20
     - 5.4.4. Migraine headache 20
     - 5.4.5. Smoker 21
   - 5.5. Finaldrug table with disease summary 21
   - 5.6. Renaming columns and data clean 21

For Review Only

5.7. Centering data 22
5.8. Removing missing data from explanitory variables 22
6. Constructing tables of participants at the begining of the study 22
6.2. Explantory variables information by cogntive diagnosis 22
6.3. Mean age by eduction level 24
6.4. Explantory variables information by pain-releiver use 25
6.4.1. MMSE 25
6.4.2. ADAS 27
6.4.3. Education 30
6.4.4. Diagnosis 30
6.4.5. APOE 34
6.4.6. Gender 35
6.4.7. AGE 37
6.4.8. Smoking status 39
6.4.9. Arthritis 40
6.4.10. Headache 43
6.4.11. Cardiovascular disease 45
6.4.12. Diabetes 47
7. Analysis of cognitive decline using the MMSE score 49
7.2. Dependent variable check 49
7.3. Generation of dependent variables appropriate for different distributions 49
7.4. Transformation to obtain normal approximation 49
7.5. Selecting non-gaussian model 53
7.6. Build base negative binomial model and comparing the different parameterization methods. 84
7.7. Observing the distribution of the residuals in for the initial model. 84
7.8. Analysing all variables in isolation 98
7.8.1. Main effect of gender 98
7.8.2. Main effect of age 98
7.8.3. Main effect of education level 99
7.8.4. Main effect of diagnosis 100
7.8.5. Main effect of APOE status 101
7.8.6. Main effect of cardiovascular pathology 102
7.8.7. Main effect of diabetes 102

For Review Only

7.8.8. Main effect of smoking ...103
7.8.9. Main effect of headache ...104
7.8.10. Main effect of arthritis ...105
7.8.11. Main effect of diclofenac ...105
7.8.12. Main effect of paracetamol ...106
7.8.13. Main effect celecoxib ...107
7.8.14. Main effect of naproxen ...108
7.8.15. Main effect of aspirin ...108
7.8.16. Main effect of ibuprofen ...109
7.9. Building combined main effect model ...110
7.10. Dropping non-significant terms ...111
7.10.1. Narpoxen ...111
7.10.2. Diclofenac ...112
7.10.3. Headache ...113
7.10.4. Paracetamol ...114
7.10.5. Gender ...115
7.10.6. Arthritis ...116
7.10.7. Ibuprofen ...117
7.10.8. Aspirin ...118
7.11. Building combined main effect model ...119
7.12. Removing each explanitory variable in isolation ...120
7.12.1. Main effect of age at the start of the study ...120
7.12.2. Main effect of APOE4 genotype ...120
7.12.3. Main effect of education level ...121
7.12.4. Main effect of initial Alzhiemer's diagnosis ...121
7.12.5. Main effect of initial Arthritis ...121
7.12.6. Main effect of initial Ibuprofen ...121
7.12.7. Main effect of initial Aspirin ...122
7.12.8. AIC summary of main effect models ...122
7.13. Investigating interaction terms ...122
7.13.1. The effects of diagnosis on cognitive decline progression ...122
7.13.2. The effects of smoking on cognitive decline progression ...122
7.13.3. The effects of arthritis on cognitive decline progression ...123
7.13.4. The effects of cardiovascular disease on cognitive decline progression ...123

For Review Only

7.13.5. The effect of headaches on cognitive decline progression 123
7.13.6. The effect of diabetes on cognitive decline progression 124
7.13.7. The effect of AGE on cognitive decline progression 125
7.13.8. The effect of APOE status on cognitive decline progression 126
7.13.9. The effect of education status on cognitive decline progression 127
7.13.10. The effect of diabetes on cognitive decline progression 128
7.13.11. The effect of Gender on cognitive decline progression 129
7.13.12. The effect of aspirin on cognitive decline progression 130
7.13.13. The effect of paracetamol on cognitive decline progression......131
7.13.14. The effect of diclofenac on cognitive decline progression 132
7.13.15. The effect of ibuprofen on cognitive decline progression 133
7.13.16. The effect of naproxin on cognitive decline progression......134
7.13.17. The effect of celecoxib on cognitive decline progression 135
7.14. Combined interaction model 136
7.14.1. Dropping non-significant terms 137
7.15. Final full model and plots of the coeffecients 140
7.15.1. Coeffecient plot 142
7.15.2. Coeffecient plot of interaction terms 143
7.16. Dropping terms of the model to evaluate the significance of each variable in the ful model. 144
7.17. Evaluating the progession and main-effects of each pain medication 146
7.18. Month as a factor 147
7.19. Years education as a numerical variable 148
7.20. APOE4 and NSAIDs......148
7.21. looking at the distribution of the residuals in the final model, for each variable separately 154
7.22. Checking for multicolinearity......162
7.23. Checking other distributions......168
8. Analysis of cognitive decline using the ADAS score......184
8.2. Dependent variable check 184
8.3. Generation of dependent variables appropriate for different distributions 184
8.4. Transformation to obtain normal approximation 184
8.5. Selecting non-gaussian model 188
8.6. Build base negative binomial model and comparing the different paramaterization methods......219

For Review Only

8.7. Observing the distribution of the residuals in for the initial model. 219
8.8. Analysing all variables in isolation 233
8.8.1. Main effect of gender 233
8.8.2. Main effect of age....233
8.8.3. Main effect of education level....234
8.8.4. Main effect of diagnosis 235
8.8.5. Main effect of APOE status....236
8.8.6. Main effect of cardiovascular pathology 237
8.8.7. Main effect of diabetes....237
8.8.8. Main effect of smoking 238
8.8.9. Main effect of headache....239
8.8.10. Main effect of arthritis....240
8.8.11. Main effect of diclofenac 240
8.8.12. Main effect of paracetamol 241
8.8.13. Main effect celecoxib....242
8.8.14. Main effect of naproxen 243
8.8.15. Main effect of aspirin....243
8.8.16. Main effect of ibuprofen....244
8.9. Building combined main effect model....245
8.10. Dropping non-significant terms 246
8.10.1. Narpoxen 246
8.10.2. Paracetamol....247
8.10.3. Arthritis....248
8.10.4. Aspirin....249
8.11. Building combined main effect model....250
8.12. Removing each explanitory variable in isolation 251
8.12.1. Main effect of age at the start of the study....251
8.12.2. Main effect of APOE4 genotype 252
8.12.3. Main effect of education level....252
8.12.4. Main effect of initial Alzhiemer's diagnosis....252
8.12.5. Main effect of headaches 252
8.12.6. Main effect of Gender 253
8.12.7. Main effect of Ibuprofen 253
8.12.8. Main effect of Diclofenac....253

For Review Only

8.12.9. AIC summary of main effect models 254
8.13. Investigating interaction terms 254
8.13.1. The effects of diagnosis on cognitive decline progression 254
8.13.2. The effects of smoking on cognitive decline progression 254
8.13.3. The effects of arthritis on cognitive decline progression 254
8.13.4. The effects of cardiovascular disease on cognitive decline progression 254
8.13.5. The effect of headaches on cognitive decline progression 254
8.13.6. The effect of diabetes on cognitive decline progression 255
8.13.7. The effect of AGE on cognitive decline progression 256
8.13.8. The effect of APOE status on cognitive decline progression 257
8.13.9. The effect of education status on cognitive decline progression 258
8.13.10. The effect of diabetes on cognitive decline progression 259
8.13.11. The effect of Gender on cognitive decline progression 260
8.13.12. The effect of aspirin on cognitive decline progression 261
8.13.13. The effect of paracetamol on cognitive decline progression 262
8.13.14. The effect of diclofenac on cognitive decline progression 263
8.13.15. The effect of ibuprofen on cognitive decline progression 264
8.13.16. The effect of naproxin on cognitive decline progression 265
8.13.17. The effect of celecoxib on cognitive decline progression 266
8.14. Combined interaction model 267
8.14.1. Dropping non-significant interactions 269
8.15. Final full model and plots of the coeffecients 271
8.15.1. Coeffecient plot 272
8.15.2. Coeffecient plot of interaction terms 273
8.16. Dropping terms of the model to evaluate the significance of each variable in the ful model. 274
8.17. Evaluating the progession and main-effects of each pain medication 277
8.18. APOE4 and NSAIDs 279
8.18.1. General NSAID use is not associated with slower cognitive decline 285
8.18.2. General NSAID use is associated with a fixed effect on cognitive performance 285
8.19. Month as a factor 285
8.20. Years education as a numerical variable 285
8.21. looking at the distribution of the residuals in the final model, for each variable separately 286

For Review Only

8.22. Checking for multicollinearity 294
8.23. Checking other distributions 300
9. Plotting predicted decline for ADAS and MMSE 318
9.2. Generating model matrix for each pain medications MMSE 318
9.2.1. Building the models for pain medications use 318
9.2.2. Generating the model matrices for each pain medication 320
9.2.2.1. Naproxen 320
9.2.2.2. Aspirin 321
9.2.2.3. Paracetamol 321
9.2.2.4. Celecoxib 322
9.2.2.5. Ibuprofen 322
9.2.2.6. Diclofenac 323
9.2.2.7. NSAID 323
9.2.2.8. No.Painrelief 323
9.2.3. Combining the model matrix 324
9.3. Generating model matrix for each pain medications ADAS 324
9.3.1. Building models 324
9.3.2. Building the model matrix with 95% Laplance confidence interavals 327
9.3.2.1. Naproxen 327
9.3.2.2. Aspirin 327
9.3.2.3. Paracetamol 328
9.3.2.4. Celecoxib 328
9.3.2.5. Ibuprofen 329
9.3.2.6. Diclofenac 329
9.3.2.7. NSAID 330
9.3.2.8. No Pain Medication 330
9.3.2.9. Combining the model matrix 330
9.4. Cleaning model matrices 331
9.5. Graphing LMCI with and without diclofenac use 331
9.5.1. MMSE 331
9.5.2. ADAS 332
9.6. Graphing LMCI with and without Ibuprofen use 333
9.6.1. MMSE 333
9.6.2. ADAS 334

For Review Only

9.7. Graphing LMCI with and without Naproxen use 335
9.7.1. MMSE....335
9.7.2. ADAS 336
9.8. Graphing LMCI with and without Aspirin use 337
9.8.1. MMSE....337
9.8.2. ADAS 338
9.9. Graphing LMCI with and without Celecoxib use 339
9.9.1. MMSE....339
9.9.2. ADAS 340
9.10. Graphing LMCI with and without Paracetamol use 341
9.10.1. MMSE....341
9.10.2. ADAS 342
9.11. Graphing LMCI with and without NSAID use 344
9.11.1. MMSE....344
9.11.2. ADAS 345
9.12. Graphing LMCI with and without APOE genotypes 346
9.12.1. MMSE....346
9.12.2. ADAS 347
9.13. Graphing LMCI between Genders....348
9.13.1. MMSE....348
9.13.2. ADAS 349
9.14. Graphing Cogntive decline of diagnosis....350
9.14.1. MMSE....350
9.14.2. ADAS 352
9.15. Graphing Education level....353
9.15.1. MMSE....353
9.15.2. ADAS 354
10. Supplement references 356

For Review Only

1. Cell culture experiment demonstrating differential effects of pain-relievers on IL-1β release

As a putative mechanism of action a series of pain-relievers were screened for the additional anti-inflammatory mechanism of action of NLRP3 inhibition (Sup. Fig. 1.1). The NLRP3 receptor has been shown in animal models to be integral in the pathophysiology of Alzheimer's disease. Here we show that diclofenac, the only pain-reliever associated with slowed cognitive decline, is also the only pain-reliever present in the ADNI dataset that inhibits NLRP3. Mefenamic acid is more potent NLRP3 inhibitor and has been found to be therapeutic in animal models of AD, however, was not used in the ADNI dataset due to its most common indication being dysmenorrhoea (period pain). This suggests that if diclofenac is validated in other epidemiological dataset and NLRP3 is found to be a key mechanism of action of diclofenac in preclinical animal models, then mefenamic acid should also be investigated as putative therapy due to greater potency at the NLRP3 receptor and reduced side effects.

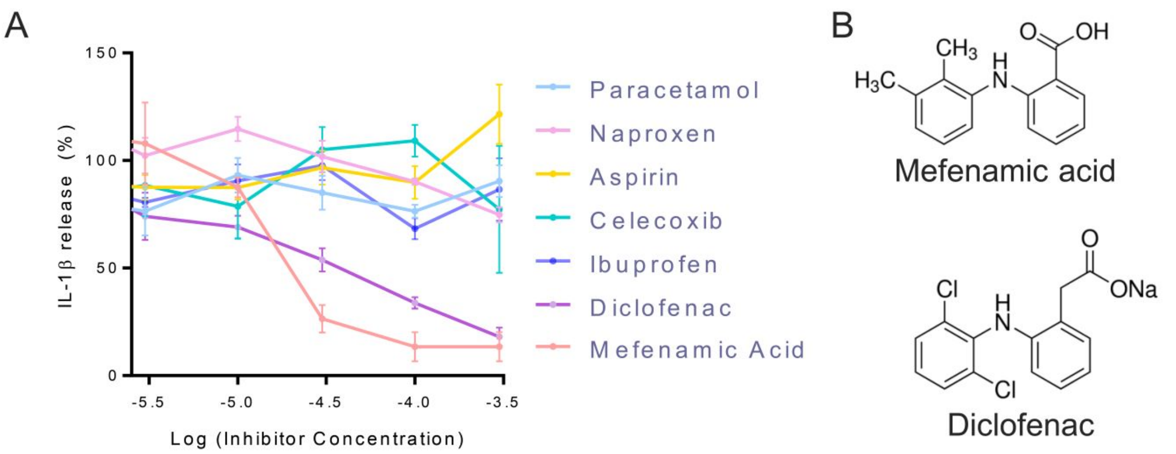

Supplemental figure 1.1: Effects of common pain-relievers on IL-1β production from mouse bone marrow derived macrophages (BMDMs). A) Bone marrow was extracted from four 12 week old C57/B6j male mice, these were differentiated into primary BMDMs through growth in 70% DMEM (containing 10% FBS, PenStrep) supplemented with 30% L929 mouse fibroblast-conditioned media for 7-10 days. Before experiments, cells were seeded overnight at 1 x 10^6 ml^{-1} in 96-well plates. The BMDMs were then treated with lipopolysaccharide (1μg ml^{-1}) for 4 hours in complete DMEM, the media was then replaced with serum free media and the drugs added at a range of concentrations (vehicle DSMO). Following a 15 minute incubation, the cells were stimulated with the NLRP3 activator nigericin (10μg ml^{-1}). After 1 hour the supernatant was removed an analysed for IL-1β concentration using ELISA assays (DY 401 DuoSet, R&D systems). B) The chemical structures of the two effective NLRP3 inhibiting pain-relievers.

2. Additional methods outline

2.1. *Brief outline of the methods*

1. Application for access to the Alzheimer's Disease Neuroimaging Initiative (ADNI) data was applied for on 21st October 2016, this was approve and the datasets were download on the 3rd of May 2018.
2. The datasets containing patient summaries, medical history, drug use and cognitive summaries were extensively cleaned dealing with inputting and spelling errors, and then merged by patient ID to create a final dataset for analysis. For the dependent variables of MMSE and ADAS score, a similar method of analysis was followed.
3. For each time point an initial assessment of data distribution was applied with negative binomial model selected as the optimal model distribution.
4. To select the parameterization method to initially construct the putative models, a simple main effect model with biologically relevant explanatory variables was applied using both available parameterization methods, these were then compared using the akaike information criterion (AIC).
5. Model construction consisted of investigating the main effect of each explanatory variable in isolation utilizing the negative binomial GLMM (all models included the additional variable of time (month -"M")). These were compared against the null model using a Chi-squared distribution of the log likelihood statistic to calculate the corresponding p value, with $p<0.05$ set at the threshold of significance.
6. All the significant main effects were then included in the model and then the coefficients were evaluated for continued input into the model in the presence of the other explanatory variables based of the Wald statistic approximation and log likelihood/chi squared method. Non-significant variables were dropped to create the final main effect model
7. Interaction terms were then investigated using the same method as the main effects with initial investigation to biologically relevant interaction terms added to the final main effect model in isolation, then all significant interaction terms were included and re-evaluated using the log likelihood/chi squared method and non-significant variables dropped.
8. Appropriate explanatory variables were then altered to numerical or factor variables and improvement in the model was evaluated as above.
9. The final model was selected base of Chi-square/log likelihood comparisons and AIC values.
10. The Pearson residuals were investigated ensuring no trends when plotted against any explanatory variables.
11. Covariance matrix was constructed and no substantial multicollinearity was observed.

For Review Only

12. Each interaction term was then dropped and compared against the final model using Chi-square/log likelihood to establish the significance of each interaction term.
13. Using the same explanatory variable model design as the final model, other GLMM families, as well as negative binomial variants, were attempted and compared against the final negative binomial model using AIC values.
14. Finally, the biologically relevant three-way interaction of APOE4, NSAID and Time was investigated and found to not significantly improve the model as determined by the Chi-square/log likelihood method [1].

2.2. *Preliminary distribution analysis*

Several possible distributions could be applied to the data, for simplicity each were tested within each time point. Normal distributions, with and without transformation, were first investigated. Previously it has been reported that a Box-Cox transformation can be utilized in multi-level linear models (MLM)(supplement 7.3-7.4 & 8.3-8.4) [2]. To do this total errors are generated by modelling the dependent variable using a simple linear model with the random effect (subject ID) as the explanatory variable. The Box-Cox method is then applied and the data transformed using the predicted lambda. If normality and heteroscedasticity of the total errors is achieved, it has been demonstrated that the Pearson residuals generated by the MLM will also adhere to the assumptions of the model [2]. Because of the upper and lower constrains of the cognitive scores, reduced variance at higher values of the MMSE score was observed and a stretching transformation was selected with a lambda of 4.79, the converse was true for the ADAS score and a lambda of 0.24 was applied (supplement 7.3-7.4 & 8.3-8.4). MLMs were then applied on the transformed scores using the LMER package with main biologically relevant explanatory variables included as well as the random effect of subject ID [3](supplement 4.2 & 5.2). Normality and heteroscedasticity was greatly improved in the models with the transformed dependent variables, however, the high numbers of cognitively normal subjects who received identical scores and the categorical nature of the scoring systems were still evident in the residual plots and so other modelling approaches were pursued (supplement 7.3-7.5 & 8.3-8.5). Similarly, GLMMs utilizing the Gamma distribution were relatively accurate models but were considered suboptimal because of the categorical nature of the scoring systems. While the exponential distribution is also a continuous distribution, and thus not appropriate for the score datasets, it was also a poor fit for both cognitive scores.

Models for discrete datasets were then investigated (supplement 7.5 & 8.5). Both MMSE and ADAS could be described as an aggregate of a series of Bernoulli trials and thus could be analysed as proportional data using logistic regression. Although this is an approximation as each trial (a point on the scale) is not identical because they correspond to different tasks in the assessments. Regardless, the numerous proportions of zero of data prevented these models from converging and so logistic regression was not appropriate (supplement 7.5 & 8.5). Poisson models were investigated as failures in the examinations could be counted and the rate of failures modelled (supplement 7.5 & 8.5). For this the MMSE score had to be converted into number of failures (30-MMSE), this has the advantage of now having the same directional relationship with disease

For Review Only

severity as the ADAS score, with higher values correlating with worse cognitive performance and greater disease severity. The Poisson model did not perform optimally as there was substantial overdispersal and so negative binomial models were explored on the same converted variables and found to be a good model to continue the analyses with (supplement 7.5 & 8.5).

2.3. *Model construction*

2.3.1. *Selecting parameterisation method*

From the initial distribution analyses it was found that the variance was greater than the mean indicating that the data is overdispersed supporting the use of negative binomial models (supplement 7.6 & 8.6)[4,5]. There are several parameterisation methods which describe the relationship between the mean and the variance in the negative binomial model. The two most common (and the only methods available in the glmmadmb package [6,7]) are the 'nbinom1' method, which assumes the variance = k × mean, and 'nbinom2' method, which assumes the variance = mean(1 + mean/k). The latter is most commonly used, particularly in count datasets, and is derived from a Gamma/Poisson model of a heterogeneous relationship between variance and mean [5]. The former describes a simple proportional relationship between variance and mean and is less commonly used due to its inflexibility [5]. Prior to the model construction, the parameterisation method must be selected. For this two negative binomial GLMMs were constructed with biologically relevant explanatory variables utilizing each of the parameterisation methods. The method that provided the lowest AIC score and maximized the log likelihood was selected. For both ADAS and MMSE scores the 'nbinom1' parameterisation was selected (supplement 7.6 & 8.6). The parameterisation method was then re-evaluated at the end of the analyses using the final models and 'nbinom1' remain the optimal parameterisation method (supplement 7.6 & 8.6). This is interesting as it is the less flexible method and not commonly used on count datasets [5]. Indicating these scores may have differences from traditional count distributions [5]. Initial residual diagnostics were run on the biologically relevant hypothesized models to ensure future analyses using the negative binomial GLMM were prudent (supplement 7.6 & 8.6).

2.3.2. *Building of initial main effect model*

Building of the GLMMs followed the protocol outlined by Hosmer et al. [8]. To construct the negative binomial GLMM models the package glmmadmb was used [6,7]. This package estimates parameters using the maximum likelihood method with the Laplace approximation to assess the marginal likelihood and provides coefficient summaries based on Wald approximations. As the aim of this study was to investigate disease progression, the baseline model was one with the explanatory variable of time (month) included as well as subject ID as a random effect (supplement 7.8 & 8.8). Then each biologically relevant explanatory variable was, in isolation, added to the baseline model and then compared against the baseline model using the Chi-squared/log likelihood method and AIC to investigate if the model was significantly improved by the inclusion of the variable (supplement 7.8 & 8.8). All significant variables were then included in the model and their continued input into the model in the presence of the other explanatory variables was evaluated using the Wald approximation statistics, Chi-squared/log likelihood method and AIC (supplement 7.10 & 8.10). Variables that ceased to significantly contribute to the model were dropped and the

For Review Only

final main effect model of all significant variables was then constructed (supplement 7.11 & 8.11). This main effect model was then added to with biologically relevant interaction terms.

2.3.3. Inclusion of biologically relevant two-way interaction terms

The inference of multivariable interaction terms becomes difficult, therefore, a common approach is to exclusively investigate biologically relevant two-way interaction terms [8]. Similar to the main effect analyses, each two-way interaction term was added to the main effect model in isolation (supplement 7.13 & 8.13). Significant improvement in the model was assessed with the Chi-squared/log likelihood method and AIC values (supplement 7.13 & 8.13). A final model was constructed including all significant interactions and Wald approximation statistics were scrutinized for non-significant coefficients (supplement 7.14-7.16 & 8.14-8.16). Each interaction was then dropped in isolation from the model and compared against the full model including the variables that were dropped as main effect terms. The worsening of the model was assessed with the Chi-squared/log likelihood method and AIC values (supplement 7.14-7.16 & 8.14-8.16). Covariance matrices were constructed of the final model and no substantial multicollinearity was found between explanatory variables (supplement 7.22 & 8.22). The full model was then tested with time (month) treated as a factor and years in education treated as a numeric variable (rather than being grouped into education levels of early, middle, tertiary and post-graduate) (supplement 7.18-7.19 & 8.18-8.19). From this it was found that treating month as a factor introduced substantially more degrees of freedom into the model (supplement 7.18 & 8.18). This caused levels of the models to not have sufficient data to stabilize the model causing issues of model convergence, even when more simple models with fewer explanatory variables were attempted. Treating years in education as a numeric variable worsened the model; this is probably due to the lack of correlation between years and the (log) dependent variable (supplement 7.19 &8.19). Indicating the model benefited from the more flexible relationship allowed by treating education as factor variable. Finally, coefficient plots were generated with Laplace approximated confidence intervals to allow a quick visualisation of significant effects with direction (supplement 7.15 & 8.15).

2.4. *Assumption check of residuals*

The Pearson residuals were extracted plotted against the explanatory variables grouped by ID. The ungrouped Pearson residuals were also plotted. No trends were observed for any explanatory variable indicating the appropriateness of the negative binomial models (supplement 7.21 & 8.21).

2.5. *Building final model with different distribution families*

To confirm the appropriateness of the negative binomial model further models were run using the same explanatory variable model design. Normal MLMs were run as well as GLMMs utilising Poisson, binomial (logistic), zero inflated negative binomials and negative binomial with alternative parameterization methods (supplement 7.23 & 8.23). All available link functions were analysed for the Poisson and binomial (logistic) models (supplement 7.23 & 8.23). Centred explanatory variables were also applied for these models. All of these models were compared using AIC and the original negative binomial models were confirmed as the preferred models (supplement 7.23 & 8.23). Interestingly, the Wald approximate statistics were very similar using many of the models

For Review Only

confirming the significances of the model terms (supplement 7.23 & 8.23). This provides greater support to the inferences of this study.

3. Additional results summary

In the present study a range of models were attempted. The negative binomial model was not expected to be the selected model; however, every evaluation of the models found that negative binomial GLMM outperformed models based on other distributions. While the canonical form of a negative binomial model is the number of successes in a series of Bernoulli trials before a specified number of failures, this does not appear appropriate for the cognitive scores investigated here, however, Hardin *et al.* [5] noted “the negative binomial is rarely used in canonical form. Its primary use is to serve as an overdispersed Poisson regression model”. Interestingly, the Poisson models appeared to be the next best GLMMs as measured by AIC (supplement 7.23 & 8.23), however, over-dispersion was clearly evident (supplement 7.5 & 8.5). Hence, negative binomial models were pursued in the present study. This suggests that the MMSE and ADAS scores can be model as a *count* of the number of mistakes which occur during testing. This concept is support by previous research which has successfully used negative binomial GLMMs for similar score based systems and further analyses in the present study confirmed the use of the negative binomial GLM (supplement 7.23 & 8.23)[9-11].

The results of the present study found that education had a capricious but significant relationship with cognitive decline. Here it was seen that those with the longest time spent in education had the fastest cognitive decline. This effect has previously been reported by Musicco *et al.* 2009 and Scarmeas *et al.* 2006. The proposed mechanism is cognitive reserve, those with greater intelligence have a delayed diagnosis as a greater loss of brain function is required before substantial symptoms set in. However, in the present study the slowest progressing group was those educated to the tertiary level, while those with secondary and early education levels declined at a rate in the middle. Therefore, this study neither supports nor refutes the cognitive reserve hypothesis [12-14]. The effect seen possibly suggests hidden variables are influence cognitive decline, for example perhaps those with post-graduate level education represent an atypical group with a number of factors exterior to their education that could alter disease progression. Therefore, while it is useful to include the education variable in the model to reduce the level of unexplained variation, no clear inferences can be made without further research.

4. Packages used

```
require(knitr)

require(lme4)

require(LMERConvenienceFunctions)

require(lmerTest)

require(MASS)

require(bbmle)

require(R2admb)
```


For Review Only

```
require(glmmADMB)

require(ggplot2)

require(coefplot)

require(car)

require(corrplot)

require(reshape2)
```

5. Data Cleaning

5.2. *Recurrent medicine data set*

The RECCMEDs data set contains all regularly taken medicines.

```
setwd("C:\\users\\mqbssjrn\\Dropbox\\Science\\Projects\\Epidemiology
ADNI\\Analysis")
#setwd("C:\\Users\\jackr\\Dropbox\\Science\\Projects\\Epidemiology
ADNI\\Analysis")
Meds<-read.csv("RECCMEDSnew.csv", header=T)
```

5.2.1. *Data Cleaning Oral Route only*

There are multiple routes of administration of pain medications, however, topical skin application will fail to reach biological relavent concentrations in the plasma in the patient and will only have action at the site of application. Futhermore, intravenous administration will unlikely be taken at a frequency that will be relavent for the slow and progressive nature of AD. Therefore, oral administration was selected as the administration route of interest and a dataframe containing only drugs administered orally was created. Then patient IDs were extracted from this data frame for each of the pain medications of interest.

```
Route<-c("po","PO","P.O.","P.O","p.o","p.o.","6","oral","Oral","ORAL","orally")
Meds$route<-ifelse(Meds$CMROUTE %in% Route,1,0)
Meds<-Meds[Meds$route==1,]
```

5.2.2. *Data Cleaning Diclofenac Spelling*

```
diclofenac<-c("Diclofemac Sodium","diclofen
sodium","diclofenac","Diclofenac","diclofenac NA","DICLOFENAC NA","Diclofenac
SOD","diclofenac sodium","Diclofenac sodium","Diclofenac Sodium Solution
1.5%","Diclofenac 0.01%","Diclofenac Sodium", "diclofen", "Diclofen","voltaren",
"Voltaren", "VOLTAREN","Volteren")
Meds$diclofenac[Meds$CMMED %in% diclofenac | Meds$CMMEDO %in% diclofenac] <- 1
dicloIDS<-Meds[Meds$diclofenac==1,3]
dicloIDS<-sort(unique(dicloIDS))


drugrow<-c()
for(i in 1:27){
  drugrow<-append(drugrow,grep(diclofenac[i],Meds$CMMED ))
  drugrow<-append(drugrow,grep(diclofenac[i],Meds$CMMEDO ))
}

dicloIDS<-Meds[drugrow,3]
```

```
dicloIDS<-sort(unique(dicloIDS))


Meds$diclofenac<-c()
Meds$diclofenac<-(Meds$RID %in% dicloIDS)*Meds$CMCONT
dicloIDS<-unique(Meds[Meds$diclofenac==1,3])
length(dicloIDS)

[1] 46
```

5.2.3. Data Cleaning Aspirin Spelling

```
aspirin<-c("Aspirin","aspirin","ASPIRIN","Baby aspirin","baby
aspirin","Apirin","asprin","Asprin","ASA (baby Asprin)","Aspirn","Aspirin
(e.c.)","Aspirin bayer","Aspirin enteric coated k.p.","apirin","apirin","Baby
Asprin","aspririn","ASA-baby asprin","Baby asprin","Enteric coated
aspirin","Baby Aspirin","Aspirin (e.c.)","Aspirin buffered325","Aspirin,
ibuprofen", "naproxen","Aspirn","aspirine")
Meds$aspirin<-numeric(length(Meds$ID))
Meds$aspirin[Meds$CMMED %in% aspirin | Meds$CMMEDO %in% aspirin] <- 1
AspIDS<-Meds[Meds$aspirin==1,3]
AspIDS<-unique(AspIDS)


drugrow<-c()
for(i in 1:27){
  drugrow<-append(drugrow,grep(aspirin[i],Meds$CMMED ))
  drugrow<-append(drugrow,grep(aspirin[i],Meds$CMMEDO ))
}

AspIDS<-Meds[drugrow,3]
AspIDS<-sort(unique(AspIDS))


Meds$aspir<-c()
Meds$aspir<-(Meds$RID %in% AspIDS)*Meds$CMCONT
AspIDS<-unique(Meds[Meds$aspir==1,3])
length(AspIDS)

[1] 1253
```

5.2.4. Data Cleaning Paracetamol Spelling

```
parac<-
c("paracetamol","panadol","Paracetamol","Panadol","acetaminophen","Acetaminophen
","Acetamin","acetamin",
"acetomenophen","Acetomenophen","ACETOMINOPHEN","acetominophen","Acetominophen",
"ACETAMINOPHEN","tylenol","Tylenol","TYLENOL","Tylox","Tyenol",
"Vicodin","vicodin","Vicodan","Vicodan","VICODIN")
Meds$parac<-numeric(length(Meds$ID))
Meds$parac[Meds$CMMED %in% parac | Meds$CMMEDO %in% parac] <- 1
paracIDS<-Meds[Meds$parac==1,3]
paracIDS<-unique(paracIDS)

drugrow<-c()
for(i in 1:24){
  drugrow<-append(drugrow,grep(parac[i],Meds$CMMED ))
  drugrow<-append(drugrow,grep(parac[i],Meds$CMMEDO ))
}
```

```
paracIDS<-Meds[drugrow,3]
paracIDS<-sort(unique(paracIDS))


Meds$parac<-c()
Meds$parac<-(Meds$RID %in% paracIDS)*Meds$CMCONT
paracIDS<-unique(Meds[Meds$parac==1,3])
length(paracIDS)

[1] 503
```

5.2.5. Data Cleaning Ibuprofen Spelling

```
Ibu<-c("Ibuprofen", "ibuprofen", "IBUPROFEN","Advil", "advil","ADVIL",
"Motrin","motrin","MOTRIN")
Meds$Ibu<-numeric(length(Meds$ID))
Meds$Ibu[Meds$CMMED %in% Ibu | Meds$CMMEDO %in% Ibu] <- 1
IbuIDS<-Meds[Meds$Ibu==1,3]
IbuIDS<-unique(IbuIDS)

drugrow<-c()
for(i in 1:9){
  drugrow<-append(drugrow,grep(Ibu[i],Meds$CMMED ))
  drugrow<-append(drugrow,grep(Ibu[i],Meds$CMMEDO ))
}

IbuIDS<-Meds[drugrow,3]
IbuIDS<-sort(unique(IbuIDS))


Meds$Ibu<-c()
Meds$Ibu<-(Meds$RID %in% IbuIDS)*Meds$CMCONT
IbuIDS<-unique(Meds[Meds$Ibu==1,3])
length(IbuIDS)

[1] 324
```

5.2.6. Data Cleaning Naproxen Spelling

```
naprox<-c("Naproxen", "naproxen", "NAPROXEN","Aleve", "aleve","ALEVE", "Accord",
"Anaprox", "Antalgin", "Apranax", "Feminax Ultra", "Flanax", "Inza", "Maxidol",
"Midol Extended Relief", "Nalgesin", "Naposin", "Naprelan", "Naprogesic",
"Naprosyn", "Narocin", "Pronaxen", "Proxen", "Soproxen", "Synflex", "MotriMax",
"Xenobid","naprox","Naprox")
Meds$naprox<-numeric(length(Meds$ID))
Meds$naprox[Meds$CMMED %in% naprox | Meds$CMMEDO %in% naprox] <- 1
naproxIDS<-Meds[Meds$naprox==1,3]
naproxIDS<-unique(naproxIDS)

drugrow<-c()
for(i in 1:29){
  drugrow<-append(drugrow,grep(naprox[i],Meds$CMMED ))
  drugrow<-append(drugrow,grep(naprox[i],Meds$CMMEDO ))
}

naproxIDS<-Meds[drugrow,3]
naproxIDS<-sort(unique(naproxIDS))
```

```
Meds$naprox<-c()
Meds$naprox<-(Meds$RID %in% naproxIDS)*Meds$CMCONT
naproxIDS<-unique(Meds[Meds$naprox==1,3])
length(naproxIDS)

[1] 241
```

5.2.7. Data Cleaning Indomethacin Spelling

Not enough subjects to include in future analysis.

```
indo<-c("Indomethacin", "indomethacin", "INDOMETHACIN","Indometacin",
"indometacin","INDOMETACIN", "Indocin", "indocin", "INDOCIN", "Tiverbex",
"tiverbex")
Meds$indo<-numeric(length(Meds$ID))
Meds$indo[Meds$CMMED %in% indo | Meds$CMMEDO %in% indo] <- 1
indoIDS<-Meds[Meds$indo==1,3]
indoIDS<-unique(indoIDS)

drugrow<-c()
for(i in 1:11){
  drugrow<-append(drugrow,grep(indo[i],Meds$CMMED ))
  drugrow<-append(drugrow,grep(indo[i],Meds$CMMEDO ))
}

indoIDS<-Meds[drugrow,3]
indoIDS<-sort(unique(indoIDS))


Meds$indo<-c()
Meds$indo<-(Meds$RID %in% indoIDS)*Meds$CMCONT
indoIDS<-unique(Meds[Meds$indo==1,3])
length(indoIDS)

[1] 16
```

5.2.8. Data Cleaning Celecoxib Spelling

```
celex<-c("Celecoxib", "celecoxib", "CELECOX","Celebrex",
"celebrex","CELEBREX","CELEBREX")

Meds$celex<-numeric(length(Meds$ID))
Meds$celex[Meds$CMMED %in% celex | Meds$CMMEDO %in% celex] <- 1
celexIDS<-Meds[Meds$celex==1,3]
celexIDS<-unique(celexIDS)

drugrow<-c()
for(i in 1:7){
  drugrow<-append(drugrow,grep(celex[i],Meds$CMMED ))
  drugrow<-append(drugrow,grep(celex[i],Meds$CMMEDO ))
}

celexIDS<-Meds[drugrow,3]
celexIDS<-sort(unique(celexIDS))


Meds$celex<-c()
Meds$celex<-(Meds$RID %in% celexIDS)*Meds$CMCONT
```

```
celexIDS<-unique(Meds[Meds$celex==1,3])
length(celexIDS)

[1] 87
```

5.3. *Combing drug data frame with outcome variable data frame*

The ADNIMERGE dataset is a complied data set of key variables from the ADNI study. Information about pain medication use was merged with the ADNIMERGE dataset.

```
setwd("C:\\users\\mqbssjrn\\Dropbox\\Science\\Projects\\Epidemiology
ADNI\\Analysis")
#setwd("C:\\Users\\jackr\\Dropbox\\Science\\Projects\\Epidemiology
ADNI\\Analysis")
summary<-read.csv("ADNIMERGEnew.csv", header=T)
Finaldrug<-summary[,which(names(summary) %in%
c("RID","DX_bl","AGE","PTGENDER","PTEDUCAT","APOE4","CDRSB","ADAS11","ADAS13","M
MSE","Hippocampus","WholeBrain","CDRSB_bl","ADAS11_bl","ADAS13_bl","MMSE_bl","M"
))]
Finaldrug$aspirin<-(Finaldrug$RID %in% AspIDS)*1
Finaldrug$parac<-(Finaldrug$RID %in% paracIDS)*1
Finaldrug$diclo<-(Finaldrug$RID %in% dicloIDS)*1
Finaldrug$Ibu<-(Finaldrug$RID %in% IbuIDS)*1
Finaldrug$naprox<-(Finaldrug$RID %in% naproxIDS)*1
Finaldrug$celex<-(Finaldrug$RID %in% celexIDS)*1

length(unique(Finaldrug$RID[Finaldrug$diclo==1]))

[1] 35
```

5.4. *Searching for confounding preexisting condition*

The RECMHIST is a dataset of preexisting condition. Diabetes and vascular diseases have been linked to changes in AD incidence and progression. Additionally, arthritis and headaches were the leading indications of pain medication use. Therefore, patient IDs where extracted and merged with the cognitive scoring dataset to include these variables in the analyses.

5.4.1. *Diabetes*

```
medhist<-read.csv("RECMHISTnew.csv", header=T)
diseases<-data.frame(table(medhist$MHDESC))
diabetes<-c("Disbetes", "diabetes","Diabetes", "DIABETES",
"diabetic","Diabetic","DIABETIC","diabetes", "diabetic","DIABETIC","DIABETES")

diseaserow<-c()
for(i in 1:11){
  diseaserow<-append(diseaserow,grep(diabetes[i],medhist$MHDESC ))
  diseaserow<-append(diseaserow,grep(diabetes[i],medhist$MHDESC ))
}

diabetesIDS<-medhist[diseaserow,3]
diabetesIDS<-sort(unique(diabetesIDS))


medhist$diabetes<-c()
medhist$diabetes<-(medhist$RID %in% diabetesIDS)*1
```


eview Only

```
diabetesIDS<-unique(medhist[medhist$diabetes==1,3])
length(diabetesIDS)

[1] 234
```

5.4.2. Cardiovascular

```
cardiovasc<-c("cholest", "pressure","hypertension", "Cholest",
"Pressure","Hypertension","stroke","Stroke","Heart attack","heart
attack","Hyoertension", "blood pressure", "cholesterol", "Cholesterol",
"PRESSURE",
"hypercholesterolemia","Hypercholesterolemia","pressure","CHOLESTEROL")

diseaserow<-c()
for(i in 1:19){
  diseaserow<-append(diseaserow,grep(cardiovasc[i],medhist$MHDESC ))
  diseaserow<-append(diseaserow,grep(cardiovasc[i],medhist$MHDESC ))
}

cardiovascIDS<-medhist[diseaserow,3]
cardiovascIDS<-sort(unique(cardiovascIDS))


medhist$cardiovasc<-c()
medhist$cardiovasc<-(medhist$RID %in% cardiovascIDS)*1
cardiovascIDS<-unique(medhist[medhist$cardiovasc==1,3])
length(cardiovascIDS)

[1] 1441
```

5.4.3. Arthritis

```
arthrit<-c("Arthritis", "arthritis","ARTHRITIS", "arthritic",
"Arthritic","Arthitis","osteoarthritis","Osteoarthritis",
"OSTEOARTHRITIS","ARTHRITIC","Arthritric")

diseaserow<-c()
for(i in 1:11){
  diseaserow<-append(diseaserow,grep(arthrit[i],medhist$MHDESC ))
}

arthritIDS<-medhist[diseaserow,3]
arthritIDS<-sort(unique(arthritIDS))


medhist$arthrit<-c()
medhist$arthrit<-(medhist$RID %in% arthritIDS)*1
arthritIDS<-unique(medhist[medhist$arthrit==1,3])
length(arthritIDS)

[1] 896
```

5.4.4. Migraine headache

```
headache<-c("headache", "Headache","HEADACHE", "Migraine",
"migraine","MIGRAINE")


diseaserow<-c()
```

```
for(i in 1:11){
  diseaserow<-append(diseaserow,grep(headache[i],medhist$MHDESC ))
}

headacheIDS<-medhist[diseaserow,3]
headacheIDS<-sort(unique(headacheIDS))


medhist$headache<-c()
medhist$headache<-(medhist$RID %in% headacheIDS)*1
headacheIDS<-unique(medhist[medhist$headache==1,3])
length(headacheIDS)

[1] 205
```

5.4.5. *Smoker*

```
smoke<-c("Smoker", "smoker","SMOKER", "SMOKE",
"Smoke","smoke","Smoking","smoking","SMOKING")

diseaserow<-c()
for(i in 1:9){
  diseaserow<-append(diseaserow,grep(smoke[i],medhist$MHDESC ))
}

smokeIDS<-medhist[diseaserow,3]
smokeIDS<-sort(unique(smokeIDS))


medhist$smoke<-c()
medhist$smoke<-(medhist$RID %in% smokeIDS)*1
smokeIDS<-unique(medhist[medhist$smoke==1,3])
length(smokeIDS)

[1] 579
```

5.5. *Finaldrug table with disease summary*

```
Finaldrug$diab<-(Finaldrug$RID %in% diabetesIDS)*1
Finaldrug$arthrit<-(Finaldrug$RID %in% arthritIDS)*1
Finaldrug$vasc<-(Finaldrug$RID %in% cardiovascIDS)*1
Finaldrug$smoke<-(Finaldrug$RID %in% smokeIDS)*1
Finaldrug$headache<-(Finaldrug$RID %in% headacheIDS)*1
```

5.6. *Renaming columns and data clean*

```
names(Finaldrug)[1]<-"ID"
names(Finaldrug)[2]<-"diagn"
names(Finaldrug)[4]<-"Gender"
names(Finaldrug)[5]<-"Yrs.edu"
names(Finaldrug)[11]<-"Hippo"
names(Finaldrug)[12]<-"Brain"
names(Finaldrug)[13]<-"CDRSB.bl"
names(Finaldrug)[14]<-"ADAS11.bl"
names(Finaldrug)[15]<-"ADAS13.bl"
names(Finaldrug)[16]<-"MMSE.bl"
names(Finaldrug)[17]<-"M"
Finaldrug$diagn<-as.character(Finaldrug$diagn)
Finaldrug$diagn[Finaldrug$diagn=="CN"]<-"1CN"
```

```
Finaldrug$diagn[Finaldrug$diagn=="EMCI"]<-"2EMCI"
Finaldrug$diagn[Finaldrug$diagn=="LMCI"]<-"3LMCI"
Finaldrug$diagn[Finaldrug$diagn=="AD"]<-"4AD"
Finaldrug<-Finaldrug[Finaldrug$diagn!="SMC",]
Finaldrug$diagn<-as.factor(Finaldrug$diagn)
```

5.7. *Centering data*

Future analyses required the centering of data to aid convergence of the model by adjusting the explanatory variables to comparable values.

```
Finaldrug$Yrs.ed.Z<-(Finaldrug$Yrs.edu-
mean(Finaldrug$Yrs.edu))/sd(Finaldrug$Yrs.edu)
Finaldrug$AGE.Z<-(Finaldrug$AGE-mean(Finaldrug$AGE))/sd(Finaldrug$AGE)
Finaldrug$AGEraw<-Finaldrug$AGE
Finaldrug$AGE<-Finaldrug$AGEraw-mean(Finaldrug$AGEraw)

Finaldrug$M.Z<-(Finaldrug$M-mean(Finaldrug$M))/sd(Finaldrug$M)
Finaldrug$x<-
Finaldrug$diclo+Finaldrug$naprox+Finaldrug$celex+Finaldrug$parac+Finaldrug$aspir
in+Finaldrug$Ibu
Finaldrug$Painrelief<-1*(Finaldrug$x>0)
Finaldrug$No.Painrelief<-(Finaldrug$Painrelief-1)^2
Finaldrug$x<-
Finaldrug$diclo+Finaldrug$naprox+Finaldrug$celex+Finaldrug$aspirin+Finaldrug$Ibu
Finaldrug$NSAID<-1*(Finaldrug$x>0)

write.csv(Finaldrug, file="Finaldrug.csv")
```

5.8. *Removing missing data from explanitory variables*

```
setwd("C:\\users\\mqbssjrn\\Dropbox\\Science\\Projects\\Epidemiology
ADNI\\Analysis")
#setwd("C:\\Users\\jackr\\Dropbox\\Science\\Projects\\Epidemiology
ADNI\\Analysis")
Fulldata<-read.csv("Finaldrug.csv", header=T)

data<-Fulldata[!is.na(Fulldata$APOE4),]
data<-data[!is.na(data$Gender),]
data<-data[!is.na(data$AGE),]
data<-data[!is.na(data$Yrs.edu),]
data<-data[!is.na(data$diagn),]
data$APOE4<-as.factor(data$APOE4)
data$ID<-as.factor(data$ID)
data$edu.cat[data$Yrs.edu<13]<-"4early"
data$edu.cat[data$Yrs.edu>=13 & data$Yrs.edu<16]<-"3mid"
data$edu.cat[data$Yrs.edu>=16& data$Yrs.edu<18]<-"2tertiary"
data$edu.cat[data$Yrs.edu>=18]<-"1post"
fulldata<-data
write.csv(data, file="CleanedFinalData.csv")
```

6. Constructing tables of participants at the begining of the study

6.2. *Explantory variables information by cogntive diagnosis*

```
initialdata<-fulldata[fulldata$M==0,]
```

```
Particpants<-length(initialdata$ID)
Particpants

[1] 1619

Gender<-table(initialdata$diagn, by=initialdata$Gender)

Mean.Age<-
round(c(0,mean(initialdata$AGE[initialdata$diagn=="1CN"]),mean(initialdata$AGE[initialdata$diagn=="2EMCI"]),mean(initialdata$AGE[initialdata$diagn=="3LMCI"]),mean(initialdata$AGE[initialdata$diagn=="4AD"])),2)
SD.AGE<-
round(c(0,sd(initialdata$AGE[initialdata$diagn=="1CN"]),sd(initialdata$AGE[initialdata$diagn=="2EMCI"]),sd(initialdata$AGE[initialdata$diagn=="3LMCI"]),sd(initialdata$AGE[initialdata$diagn=="4AD"])),2)
Mean.Age.total<-round(mean(initialdata$AGE,2))
SD.AGE.total<-round(sd(initialdata$AGE),2)

Aspirin<-table(initialdata$diagn,by=initialdata$aspirin)[,2]
Celecoxib<-table(initialdata$diagn,by=initialdata$celex)[,2]
Diclofenac<-table(initialdata$diagn,by=initialdata$diclo)[,2]
Ibuprofen<-table(initialdata$diagn,by=initialdata$Ibu)[,2]
Naproxen<-table(initialdata$diagn,by=initialdata$naprox)[,2]
Paracetamol<-table(initialdata$diagn,by=initialdata$parac)[,2]
No.Painrelief<-table(initialdata$diagn,by=initialdata$Painrelief)[,1]

Cardiovascular.Pathology<-table(initialdata$diagn,by=initialdata$vasc)[,2]
Diabetes<-table(initialdata$diagn,by=initialdata$diab)[,2]
Smoker<-table(initialdata$diagn,by=initialdata$smoke)[,2]
Headache<-table(initialdata$diagn,by=initialdata$headache)[,2]
Arthritis<-table(initialdata$diagn,by=initialdata$arthrit)[,2]

APOE4.1<-table(initialdata$diagn,by=initialdata$APOE4)[,2]
APOE4.2<-table(initialdata$diagn,by=initialdata$APOE4)[,3]
Early.Education<-table(initialdata$diagn,by=initialdata$edu.cat)[,4]
Mid.Education<-table(initialdata$diagn,by=initialdata$edu.cat)[,3]
Tertiary.Education<-table(initialdata$diagn,by=initialdata$edu.cat)[,2]
Postgraduate.Education<-table(initialdata$diagn,by=initialdata$edu.cat)[,1]


Mean.MMSE<-
round(c(0,mean(initialdata$MMSE[initialdata$diagn=="1CN"],na.rm=TRUE),mean(initialdata$MMSE[initialdata$diagn=="2EMCI"],na.rm=TRUE),mean(initialdata$MMSE[initialdata$diagn=="3LMCI"],na.rm=TRUE),mean(initialdata$MMSE[initialdata$diagn=="4AD"],na.rm=TRUE)),2)
SD.MMSE<-
round(c(0,sd(initialdata$MMSE[initialdata$diagn=="1CN"],na.rm=TRUE),sd(initialdata$MMSE[initialdata$diagn=="2EMCI"],na.rm=TRUE),sd(initialdata$MMSE[initialdata$diagn=="3LMCI"],na.rm=TRUE),sd(initialdata$MMSE[initialdata$diagn=="4AD"],na.rm=TRUE)),2)

Mean.MMSE.total<-round(mean(initialdata$MMSE,2))
SD.MMSE.total<-round(sd(initialdata$MMSE),2)

Mean.ADAS13<-
round(c(0,mean(initialdata$ADAS13[initialdata$diagn=="1CN"],na.rm=TRUE),mean(initialdata$ADAS13[initialdata$diagn=="2EMCI"],na.rm=TRUE),mean(initialdata$ADAS13[initialdata$diagn=="3LMCI"],na.rm=TRUE),mean(initialdata$ADAS13[initialdata$diagn=="4AD"],na.rm=TRUE)),2)
```

```
SD.ADAS13<-
round(c(0,sd(initialdata$ADAS13[initialdata$diagn=="1CN"],na.rm=TRUE),sd(initial
data$ADAS13[initialdata$diagn=="2EMCI"],na.rm=TRUE),sd(initialdata$ADAS13[initia
ldata$diagn=="3LMCI"],na.rm=TRUE),sd(initialdata$ADAS13[initialdata$diagn=="4AD"
],na.rm=TRUE)),2)

Mean.ADAS.total<-round(mean(initialdata$ADAS13,na.rm=TRUE),2)
SD.ADAS.total<-round(sd(initialdata$ADAS13,na.rm=TRUE),2)

table<-t(cbind(Gender, Mean.Age,
SD.AGE,Aspirin,Celecoxib,Diclofenac,Ibuprofen,Naproxen,Paracetamol,No.Painrelief
,Cardiovascular.Pathology,Diabetes,Smoker, Arthritis, Headache,
APOE4.1,APOE4.2,Early.Education,Mid.Education,Tertiary.Education,Postgraduate.Ed
ucation, Mean.MMSE,SD.MMSE,Mean.ADAS13,SD.ADAS13))[,2:5]
table

                            1CN  2EMCI  3LMCI    4AD
Female                   206.00 136.00 219.00 149.00
Male                     209.00 170.00 345.00 185.00
Mean.Age                   1.00  -2.54   0.23   1.15
SD.AGE                     5.73   7.40   7.50   7.82
Aspirin                  247.00 174.00 302.00 138.00
Celecoxib                 19.00  13.00  25.00   7.00
Diclofenac                14.00   5.00   8.00   3.00
Ibuprofen                 79.00  55.00  92.00  29.00
Naproxen                  52.00  45.00  68.00  21.00
Paracetamol              119.00  75.00 140.00  66.00
No.Painrelief             99.00  67.00 165.00 141.00
Cardiovascular.Pathology 271.00 182.00 348.00 195.00
Diabetes                  37.00  37.00  48.00  28.00
Smoker                   109.00  69.00 150.00  84.00
Arthritis                183.00 126.00 208.00 109.00
Headache                  36.00  30.00  49.00  23.00
APOE4.1                  103.00 110.00 234.00 157.00
APOE4.2                   11.00  21.00  73.00  65.00
Early.Education           44.00  45.00 102.00  82.00
Mid.Education             89.00  74.00  88.00  68.00
Tertiary.Education       120.00  76.00 167.00 100.00
Postgraduate.Education   162.00 111.00 207.00  84.00
Mean.MMSE                 29.07  28.33  27.18  23.18
SD.MMSE                    1.12   1.57   1.81   2.06
Mean.ADAS13                9.33  12.63  18.69  29.96
SD.ADAS13                  4.33   5.40   6.52   8.05

t(cbind(Mean.Age.total,SD.AGE.total,Mean.MMSE.total,SD.MMSE.total,Mean.ADAS.tota
l,SD.ADAS.total))

                 [,1]
Mean.Age.total   0.00
SD.AGE.total     7.25
Mean.MMSE.total 28.00
SD.MMSE.total    2.69
Mean.ADAS.total 17.41
SD.ADAS.total    9.59
```

6.3. *Mean age by eduction level*

```
mean.AGEraw<-
round(c(mean(initialdata$AGEraw[initialdata$edu.cat=="1post"],na.rm=TRUE),mean(i
nitialdata$AGEraw[initialdata$edu.cat=="2tertiary"],na.rm=TRUE),mean(initialdata
$AGEraw[initialdata$edu.cat=="3mid"],na.rm=TRUE),mean(initialdata$AGEraw[initial
data$edu.cat=="4early"],na.rm=TRUE)),2)

sd.AGEraw<-
round(c(sd(initialdata$AGEraw[initialdata$edu.cat=="1post"],na.rm=TRUE),sd(initi
aldata$AGEraw[initialdata$edu.cat=="2tertiary"],na.rm=TRUE),sd(initialdata$AGEra
w[initialdata$edu.cat=="3mid"],na.rm=TRUE),sd(initialdata$AGEraw[initialdata$edu
.cat=="4early"],na.rm=TRUE)),2)

Education<-c("Post-graduate","Tertiary","Mid", "Early")

data.frame(Education, mean.AGEraw, sd.AGEraw)

      Education mean.AGEraw sd.AGEraw
1 Post-graduate       73.31      7.31
2      Tertiary       73.94      7.19
3           Mid       73.56      7.53
4         Early       75.35      6.74
```

6.4. *Explantory variables information by pain-releiver use*

6.4.1. *MMSE*

```
Aspirin<-mean(initialdata$MMSE.bl[initialdata$aspirin==1])
Aspirin.sd<-sd(initialdata$MMSE.bl[initialdata$aspirin==1])
Celecoxib<-mean(initialdata$MMSE.bl[initialdata$celex==1])
Celecoxib.sd<-sd(initialdata$MMSE.bl[initialdata$celex==1])
Diclofenac<-mean(initialdata$MMSE.bl[initialdata$diclo==1])
Diclofenac.sd<-sd(initialdata$MMSE.bl[initialdata$diclo==1])
Ibuprofen<-mean(initialdata$MMSE.bl[initialdata$Ibu==1])
Ibuprofen.sd<-sd(initialdata$MMSE.bl[initialdata$Ibu==1])
Naproxen<-mean(initialdata$MMSE.bl[initialdata$naprox==1])
Naproxen.sd<-sd(initialdata$MMSE.bl[initialdata$naprox==1])
Paracetamol<-mean(initialdata$MMSE.bl[initialdata$parac==1])
Paracetamol.sd<-sd(initialdata$MMSE.bl[initialdata$parac==1])

no.painrelief<-mean(initialdata$MMSE.bl[initialdata$No.Painrelief==1])
no.painrelief.sd<-sd(initialdata$MMSE.bl[initialdata$No.Painrelief==1])

MMSE.table<-
data.frame(Aspirin,Aspirin.sd,Celecoxib,Celecoxib.sd,Diclofenac,Diclofenac.sd,Ib
uprofen,Ibuprofen.sd,Naproxen,Naproxen.sd,Paracetamol,Paracetamol.sd,no.painreli
ef,no.painrelief.sd)

MMSE.table

   Aspirin Aspirin.sd Celecoxib Celecoxib.sd Diclofenac Diclofenac.sd
1 27.34959    2.60763     27.25     2.594255   27.96667      2.385059
  Ibuprofen Ibuprofen.sd Naproxen Naproxen.sd Paracetamol Paracetamol.sd
1  27.73333     2.189692 27.63978    2.412653     27.3675       2.525547
  no.painrelief no.painrelief.sd
1      26.42797          2.83051
```

```
lm0<-glm.nb(round(30-MMSE.bl)~1,data=initialdata)

lm1<-glm.nb(round(30-MMSE.bl)~aspirin+
celex+diclo+Ibu+naprox+parac,data=initialdata)
summary(lm1)


Call:
glm.nb(formula = round(30 - MMSE.bl) ~ aspirin + celex + diclo +
    Ibu + naprox + parac, data = initialdata, init.theta = 1.733486213,
    link = log)

Deviance Residuals:
    Min       1Q   Median       3Q      Max
-1.9600  -0.9649  -0.2836   0.4916   2.3421

Coefficients:
            Estimate Std. Error z value Pr(>|z|)
(Intercept)  1.25734    0.03744  33.580  < 2e-16 ***
aspirin     -0.19295    0.04802  -4.018 5.86e-05 ***
celex       -0.03888    0.12422  -0.313 0.754276
diclo       -0.32975    0.19247  -1.713 0.086667 .
Ibu         -0.26755    0.06897  -3.879 0.000105 ***
naprox      -0.18471    0.07889  -2.341 0.019208 *
parac       -0.07534    0.05750  -1.310 0.190077
---
Signif. codes:  0 '***' 0.001 '**' 0.01 '*' 0.05 '.' 0.1 ' ' 1

(Dispersion parameter for Negative Binomial(1.7335) family taken to be 1)

    Null deviance: 1902.6  on 1618  degrees of freedom
Residual deviance: 1852.9  on 1612  degrees of freedom
AIC: 7126.6

Number of Fisher Scoring iterations: 1

              Theta:  1.733
          Std. Err.:  0.109

 2 x log-likelihood:  -7110.606

par(mfrow=c(2,2)); plot(lm1)
```

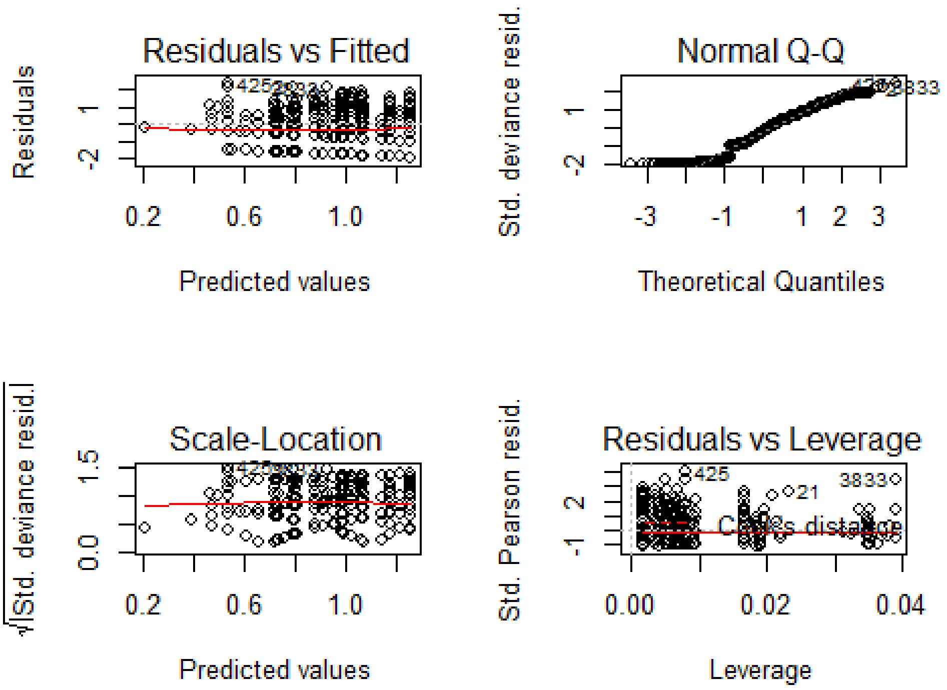


```
anova(lm0,lm1)

Likelihood ratio tests of Negative Binomial Models

Response: round(30 - MMSE.bl)
                                           Model    theta Resid. df
1                                              1 1.634106      1618
2 aspirin + celex + diclo + Ibu + naprox + parac 1.733486      1612
     2 x log-lik.   Test    df LR stat.      Pr(Chi)
1       -7159.351
2       -7110.606 1 vs 2     6 48.74534 8.384167e-09

aspirin.lm<-glm.nb(round(30-MMSE.bl)~
celex+diclo+Ibu+naprox+parac,data=initialdata)
celex.lm<-glm.nb(round(30-MMSE.bl)~aspirin+
diclo+Ibu+naprox+parac,data=initialdata)
diclo.lm<-glm.nb(round(30-MMSE.bl)~aspirin+
celex+Ibu+naprox+parac,data=initialdata)
Ibu.lm<-glm.nb(round(30-MMSE.bl)~aspirin+
celex+diclo+naprox+parac,data=initialdata)
naprox.lm<-glm.nb(round(30-MMSE.bl)~aspirin+
celex+diclo+Ibu+parac,data=initialdata)
parac.lm<-glm.nb(round(30-MMSE.bl)~aspirin+
celex+diclo+Ibu+naprox,data=initialdata)

Drug<-c("Aspirin","Celecoxib","Diclofenac","Ibuprofen","Naproxen","Ibuprofen")
table<-cbind(Drug,rbind(anova(aspirin.lm,lm1)[2,6:8],
anova(celex.lm,lm1)[2,6:8],
anova(diclo.lm,lm1)[2,6:8],
anova(Ibu.lm,lm1)[2,6:8],
anova(naprox.lm,lm1)[2,6:8],
anova(parac.lm,lm1)[2,6:8]))
Adjusted.P.Value<-p.adjust(table[,4],method="bonferroni")
```

```
table<-cbind(table,Adjusted.P.Value)
table

         Drug    df    LR stat.      Pr(Chi) Adjusted.P.Value
2     Aspirin     1 16.01054233 6.299075e-05     0.0003779445
21  Celecoxib     1  0.09710129 7.553363e-01     1.0000000000
22 Diclofenac     1  2.93354306 8.675690e-02     0.5205414207
23  Ibuprofen     1 14.72444695 1.244225e-04     0.0007465348
24   Naproxen     1  5.41874133 1.992172e-02     0.1195302980
25  Ibuprofen     1  1.71015062 1.909658e-01     1.0000000000
```

6.4.2. ADAS

```
Aspirin<-mean(initialdata$ADAS13.bl[initialdata$aspirin==1],na.rm=TRUE)
Aspirin.sd<-sd(initialdata$ADAS13.bl[initialdata$aspirin==1],na.rm=TRUE)
Celecoxib<-mean(initialdata$ADAS13.bl[initialdata$celex==1],na.rm=TRUE)
Celecoxib.sd<-sd(initialdata$ADAS13.bl[initialdata$celex==1],na.rm=TRUE)
Diclofenac<-mean(initialdata$ADAS13.bl[initialdata$diclo==1],na.rm=TRUE)
Diclofenac.sd<-sd(initialdata$ADAS13.bl[initialdata$diclo==1],na.rm=TRUE)
Ibuprofen<-mean(initialdata$ADAS13.bl[initialdata$Ibu==1],na.rm=TRUE)
Ibuprofen.sd<-sd(initialdata$ADAS13.bl[initialdata$Ibu==1],na.rm=TRUE)
Naproxen<-mean(initialdata$ADAS13.bl[initialdata$naprox==1],na.rm=TRUE)
Naproxen.sd<-sd(initialdata$ADAS13.bl[initialdata$naprox==1],na.rm=TRUE)
Paracetamol<-mean(initialdata$ADAS13.bl[initialdata$parac==1],na.rm=TRUE)
Paracetamol.sd<-sd(initialdata$ADAS13.bl[initialdata$parac==1],na.rm=TRUE)
no.painrelief<-
mean(initialdata$ADAS13.bl[initialdata$No.Painrelief==1],na.rm=TRUE)
no.painrelief.sd<-
sd(initialdata$ADAS13.bl[initialdata$No.Painrelief==1],na.rm=TRUE)


ADAS.table<-
data.frame(Aspirin,Aspirin.sd,Celecoxib,Celecoxib.sd,Diclofenac,Diclofenac.sd,Ib
uprofen,Ibuprofen.sd,Naproxen,Naproxen.sd,Paracetamol,Paracetamol.sd,no.painreli
ef,no.painrelief.sd)

ADAS.table

   Aspirin Aspirin.sd Celecoxib Celecoxib.sd Diclofenac Diclofenac.sd
1 16.37426   9.014807  15.81492     8.324503   12.29897      8.450123
  Ibuprofen Ibuprofen.sd Naproxen Naproxen.sd Paracetamol Paracetamol.sd
1  14.99209     8.451613 15.44269    8.941314    16.20937       8.792672
  no.painrelief no.painrelief.sd
1      19.69797         10.34947

lm0<-glm.nb(round(ADAS13.bl*3)~1,data=initialdata)
lm1<-glm.nb(round(ADAS13.bl*3)~aspirin+
celex+diclo+Ibu+naprox+parac,data=initialdata)
summary(lm1)


Call:
glm.nb(formula = round(ADAS13.bl * 3) ~ aspirin + celex + diclo +
    Ibu + naprox + parac, data = initialdata, init.theta = 3.423409156,
    link = log)

Deviance Residuals:
    Min       1Q   Median       3Q      Max
-4.3000  -0.9026  -0.1479   0.5227   2.4920
```

```
Coefficients:
            Estimate Std. Error z value Pr(>|z|)    
(Intercept)  4.06734    0.02229 182.441  < 2e-16 ***
aspirin     -0.11240    0.02806  -4.006 6.18e-05 ***
celex       -0.08914    0.07238  -1.232  0.21810    
diclo       -0.32162    0.10633  -3.025  0.00249 ** 
Ibu         -0.15733    0.03872  -4.063 4.85e-05 ***
naprox      -0.09776    0.04438  -2.203  0.02759 *  
parac       -0.04834    0.03315  -1.458  0.14475    
---
Signif. codes:  0 '***' 0.001 '**' 0.01 '*' 0.05 '.' 0.1 ' ' 1

(Dispersion parameter for Negative Binomial(3.4234) family taken to be 1)

    Null deviance: 1751.0  on 1606  degrees of freedom
Residual deviance: 1692.5  on 1600  degrees of freedom
  (12 observations deleted due to missingness)
AIC: 15064

Number of Fisher Scoring iterations: 1

              Theta:  3.423 
          Std. Err.:  0.126 

 2 x log-likelihood:  -15047.771 

anova(lm0,lm1)

Likelihood ratio tests of Negative Binomial Models

Response: round(ADAS13.bl * 3)
                                           Model    theta Resid. df
1                                              1 3.302247      1606
2 aspirin + celex + diclo + Ibu + naprox + parac 3.423409      1600
     2 x log-lik.   Test    df LR stat.      Pr(Chi)
1       -15105.28                                   
2       -15047.77 1 vs 2     6 57.51281 1.438381e-10

par(mfrow=c(2,2)); plot(lm1)
```

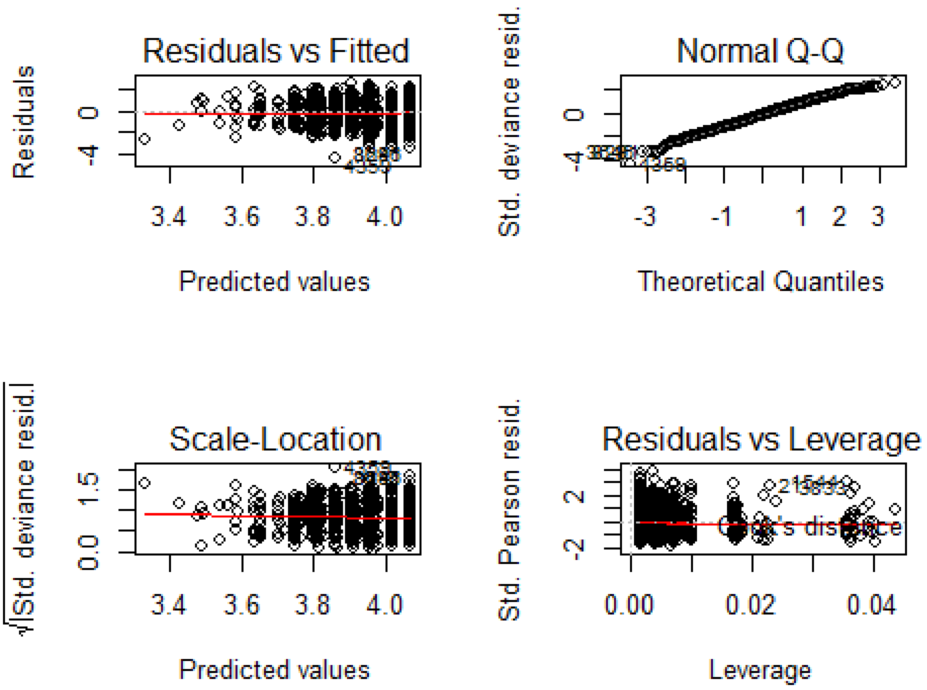


```
aspirin.lm<-glm.nb(round(ADAS13.bl*3)~
celex+diclo+Ibu+naprox+parac,data=initialdata)
celex.lm<-glm.nb(round(ADAS13.bl*3)~aspirin+
diclo+Ibu+naprox+parac,data=initialdata)
diclo.lm<-glm.nb(round(ADAS13.bl*3)~aspirin+
celex+Ibu+naprox+parac,data=initialdata)
Ibu.lm<-glm.nb(round(ADAS13.bl*3)~aspirin+
celex+diclo+naprox+parac,data=initialdata)
naprox.lm<-glm.nb(round(ADAS13.bl*3)~aspirin+
celex+diclo+Ibu+parac,data=initialdata)
parac.lm<-glm.nb(round(ADAS13.bl*3)~aspirin+
celex+diclo+Ibu+naprox,data=initialdata)

Drug<-c("Aspirin","Celecoxib","Diclofenac","Ibuprofen","Naproxen","Ibuprofen")
table<-cbind(Drug,rbind(anova(aspirin.lm,lm1)[2,6:8],
anova(celex.lm,lm1)[2,6:8],
anova(diclo.lm,lm1)[2,6:8],
anova(Ibu.lm,lm1)[2,6:8],
anova(naprox.lm,lm1)[2,6:8],
anova(parac.lm,lm1)[2,6:8]))
Adjusted.P.Value<-p.adjust(table[,4],method="bonferroni")
table<-cbind(table,Adjusted.P.Value)
table

         Drug    df  LR stat.      Pr(Chi) Adjusted.P.Value
2     Aspirin     1 15.930344 6.571667e-05      0.000394300
21  Celecoxib     1  1.479511 2.238510e-01      1.000000000
22 Diclofenac     1  8.419487 3.712204e-03      0.022273225
23  Ibuprofen     1 15.925698 6.587817e-05      0.000395269
24   Naproxen     1  4.733385 2.958261e-02      0.177495664
25  Ibuprofen     1  2.103388 1.469732e-01      0.881839124
```

6.4.3. Education

```
Aspirin<-table(initialdata$edu.cat,by=initialdata$aspirin)[,2]
Celecoxib<-table(initialdata$edu.cat,by=initialdata$celex)[,2]
Diclofenac<-table(initialdata$edu.cat,by=initialdata$diclo)[,2]
Ibuprofen<-table(initialdata$edu.cat,by=initialdata$Ibu)[,2]
Naproxen<-table(initialdata$edu.cat,by=initialdata$naprox)[,2]
Paracetamol<-table(initialdata$edu.cat,by=initialdata$parac)[,2]
No.Painrelief<-table(initialdata$edu.cat,by=initialdata$No.Painrelief)[,2]

Education.table<-
data.frame(t(cbind(Aspirin,Celecoxib,Diclofenac,Ibuprofen,Naproxen,Paracetamol,N
o.Painrelief)))
names(Education.table)<-c("Primary","Secondary","Tertiary","Post-grad")
Education.table$Primary.percent<-
Education.table[,1]/(Education.table[,2]+Education.table[,3]+Education.table[,4]
+Education.table[,1])*100
Education.table$Secondary.percent<-
Education.table[,2]/(Education.table[,2]+Education.table[,3]+Education.table[,4]
+Education.table[,1])*100
Education.table$Tertiary.percent<-
Education.table[,3]/(Education.table[,2]+Education.table[,1]+Education.table[,3]
+Education.table[,4])*100
Education.table$Postgrad.percent<-
Education.table[,4]/(Education.table[,2]+Education.table[,1]+Education.table[,3]
+Education.table[,4])*100
Education.table

              Primary Secondary Tertiary Post-grad Primary.percent
Aspirin           318       251      166       126        36.93380
Celecoxib          21        18       12        13        32.81250
Diclofenac         10        11        3         6        33.33333
Ibuprofen          83        77       50        45        32.54902
Naproxen           56        59       40        31        30.10753
Paracetamol       128       119       92        61        32.00000
No.Painrelief     161       126       92        93        34.11017
              Secondary.percent Tertiary.percent Postgrad.percent
Aspirin                29.15215         19.27991         14.63415
Celecoxib              28.12500         18.75000         20.31250
Diclofenac             36.66667         10.00000         20.00000
Ibuprofen              30.19608         19.60784         17.64706
Naproxen               31.72043         21.50538         16.66667
Paracetamol            29.75000         23.00000         15.25000
No.Painrelief          26.69492         19.49153         19.70339

Education.table.chisq<-chisq.test(Education.table[,1:4])
Education.table.chisq


    Pearson's Chi-squared test

data:  Education.table[, 1:4]
X-squared = 15.702, df = 18, p-value = 0.6133
```

6.4.4. Diagnosis

```
Aspirin<-table(initialdata$diagn,by=initialdata$aspirin)[,2]
Celecoxib<-table(initialdata$diagn,by=initialdata$celex)[,2]
Diclofenac<-table(initialdata$diagn,by=initialdata$diclo)[,2]
Ibuprofen<-table(initialdata$diagn,by=initialdata$Ibu)[,2]
```

```
Naproxen<-table(initialdata$diagn,by=initialdata$naprox)[,2]
Paracetamol<-table(initialdata$diagn,by=initialdata$parac)[,2]
No.Painrelief<-table(initialdata$diagn,by=initialdata$No.Painrelief)[,1]

Diagnosis.table<-
data.frame(t(cbind(Aspirin,Celecoxib,Diclofenac,Ibuprofen,Naproxen,Paracetamol,N
o.Painrelief)))
Diagnosis.table<-Diagnosis.table[,2:5]
names(Diagnosis.table)<-c("CN","EMCI","LMCI","AD")
Diagnosis.table$CN.percent<-
Diagnosis.table[,1]/(Diagnosis.table[,2]+Diagnosis.table[,3]+Diagnosis.table[,4]
+Diagnosis.table[,1])*100

Diagnosis.table$EMCI.percent<-
Diagnosis.table[,2]/(Diagnosis.table[,2]+Diagnosis.table[,3]+Diagnosis.table[,4]
+Diagnosis.table[,1])*100

Diagnosis.table$LMCI.percent<-
Diagnosis.table[,3]/(Diagnosis.table[,2]+Diagnosis.table[,3]+Diagnosis.table[,4]
+Diagnosis.table[,1])*100

Diagnosis.table$AD.percent<-
Diagnosis.table[,4]/(Diagnosis.table[,2]+Diagnosis.table[,3]+Diagnosis.table[,4]
+Diagnosis.table[,1])*100

Diagnosis.table$total<-
(Diagnosis.table[,5]+Diagnosis.table[,3]+Diagnosis.table[,4]+Diagnosis.table[,1]
)

Diagnosis.table

               CN EMCI LMCI  AD CN.percent EMCI.percent LMCI.percent
Aspirin       247  174  302 138   28.68757     20.20906     35.07549
Celecoxib      19   13   25   7   29.68750     20.31250     39.06250
Diclofenac     14    5    8   3   46.66667     16.66667     26.66667
Ibuprofen      79   55   92  29   30.98039     21.56863     36.07843
Naproxen       52   45   68  21   27.95699     24.19355     36.55914
Paracetamol   119   75  140  66   29.75000     18.75000     35.00000
No.Painrelief 316  239  399 193   27.55013     20.83697     34.78640
              AD.percent     total
Aspirin         16.02787 715.68757
Celecoxib       10.93750  80.68750
Diclofenac      10.00000  71.66667
Ibuprofen       11.37255 230.98039
Naproxen        11.29032 168.95699
Paracetamol     16.50000 354.75000
No.Painrelief   16.82650 935.55013

chisq<-chisq.test(Diagnosis.table[,1:4])


chisq


    Pearson's Chi-squared test

data:  Diagnosis.table[, 1:4]
X-squared = 16.018, df = 18, p-value = 0.5913
```

```
contrib<-100*chisq$residuals^2/chisq$statistic
contrib

                        CN       EMCI        LMCI         AD
Aspirin        0.006422259 0.38127839 0.005275827  0.8636755
Celecoxib      0.123161661 0.01507559 1.754906342  5.4230442
Diclofenac    20.923751048 1.40094473 3.822127704  3.6862821
Ibuprofen      2.764449028 0.73857709 0.403970804 17.7062696
Naproxen       0.251589544 7.31800340 0.671064282 13.4312919
Paracetamol    0.875371732 4.11144039 0.012803893  1.5182119
No.Painrelief  3.563511609 0.20999316 0.246566813  7.7709395

res1 <- cor.mtest(contrib, conf.level = 0.95)

col2 <- colorRampPalette(c("#67001F", "#B2182B", "#D6604D", "#F4A582",
                           "#FDDBC7", "#FFFFFF", "#D1E5F0", "#92C5DE",
                           "#4393C3", "#2166AC", "#053061"))


pearson<-chisq$residuals

#pdf("Risidual.#pdf", width=16/2.54, heigh=12/2.54 , useDingbats=F)
corrplot(contrib, p.mat = res1$p, insig = "blank",pch.col=1,is.cor=F,tl.col=1,
cl.pos="b", number.digits=2,number.cex=0.5,cl.cex=1,cl.length=5, cl.lim=c(0,50),
col=rev(col2(200)))
```

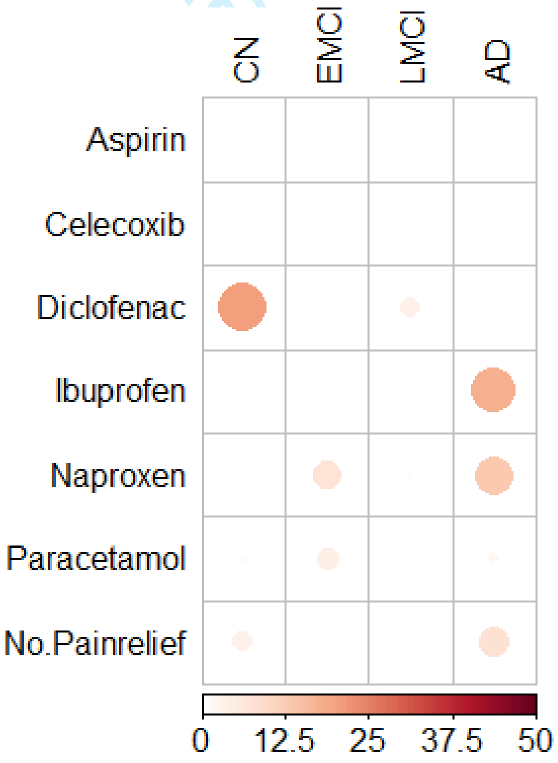


```
#dev.off()

#pdf("Percentage.#pdf", width=16/2.54, heigh=12/2.54, useDingbats=F)

corrplot(pearson, p.mat = res1$p, insig = "blank",pch.col=1,is.cor=F,tl.col=1,
cl.pos="b", number.digits=2,number.cex=0.5,cl.cex=1,cl.length=5, cl.lim=c(-
4,4),col=rev(col2(200)))
```

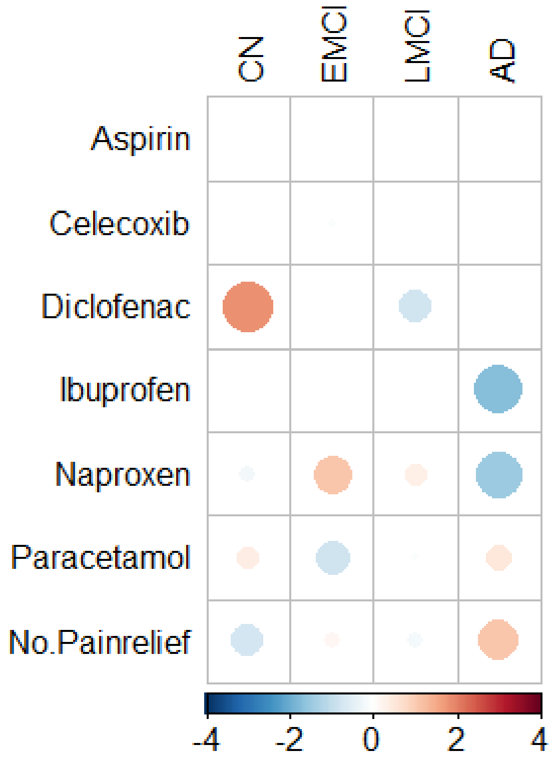


```
#dev.off()

Diagnosis.table$Painrelief<-rownames(Diagnosis.table)

melt.diag<-melt(Diagnosis.table,id="Painrelief")
percent<-c("CN.percent","EMCI.percent","LMCI.percent","AD.percent")
melt.diag<-melt.diag[(melt.diag$variable%in%percent),]

positions<-
c("No.Painrelief","Aspirin","Celecoxib","Diclofenac","Ibuprofen","Naproxen","Par
acetamol")

#pdf("Proportions.#pdf", width=16/2.54, heigh=12/2.54, useDingbats=F)
qplot(x=Painrelief,y=value,fill=variable,data=melt.diag,geom="col")+
theme(axis.text.x = element_text(size=10, angle=60, hjust=1),
plot.background=element_rect(0),panel.background=element_rect(0),axis.line=eleme
nt_line(1, size=1))+
  scale_y_continuous(expand = c(0, 0), breaks=seq(0,
100,by=20),limits=c(0,100))+
  scale_fill_manual(values=c("#7cafe2","#4b912299","#e4630066","#f78a8a"))+
 scale_x_discrete(limits = positions)
```

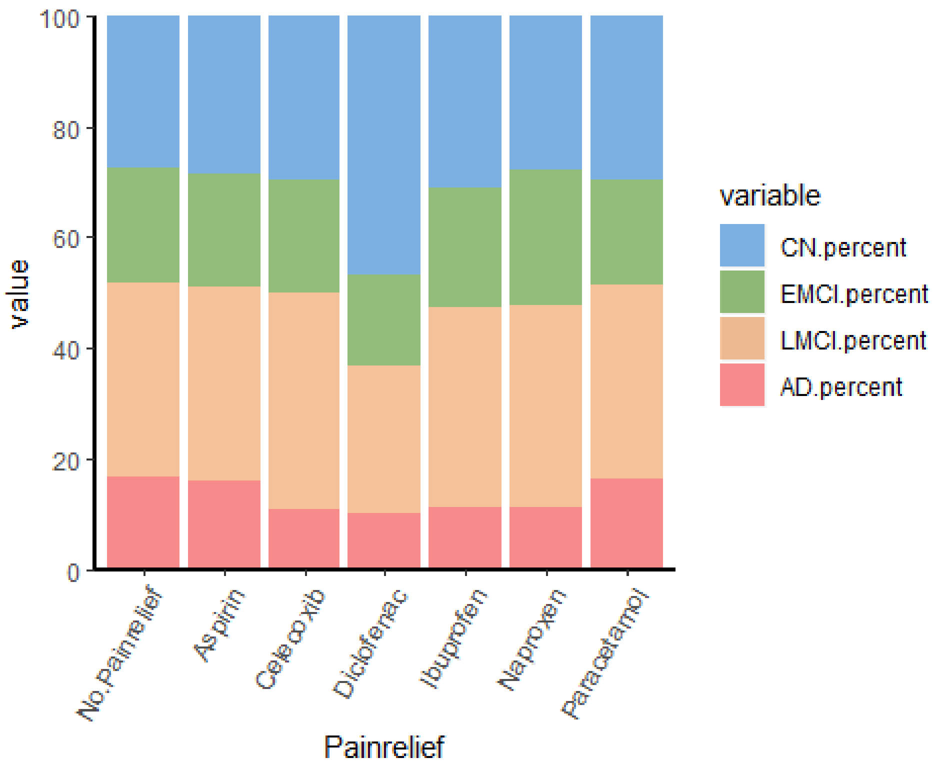


```
#dev.off()
```

6.4.5. APOE

```
Aspirin<-table(initialdata$APOE4,by=initialdata$aspirin)[,2]
Celecoxib<-table(initialdata$APOE4,by=initialdata$celex)[,2]
Diclofenac<-table(initialdata$APOE4,by=initialdata$diclo)[,2]
Ibuprofen<-table(initialdata$APOE4,by=initialdata$Ibu)[,2]
Naproxen<-table(initialdata$APOE4,by=initialdata$naprox)[,2]
Paracetamol<-table(initialdata$APOE4,by=initialdata$parac)[,2]
No.Painrelief<-table(initialdata$APOE4,by=initialdata$No.Painrelief)[,2]


APOE4.table<-
data.frame(t(cbind(Aspirin,Celecoxib,Diclofenac,Ibuprofen,Naproxen,Paracetamol,N
o.Painrelief)))
names(APOE4.table)<-c("WT","HET","HOMO")
APOE4.table$WT.Percent<-
APOE4.table[,1]/(APOE4.table[,2]+APOE4.table[,3]+APOE4.table[,1])*100
APOE4.table$HET.Percent<-
APOE4.table[,2]/(APOE4.table[,2]+APOE4.table[,3]+APOE4.table[,1])*100
APOE4.table$HOMO.Percent<-
APOE4.table[,3]/(APOE4.table[,2]+APOE4.table[,3]+APOE4.table[,1])*100

APOE4.table

              WT  HET  HOMO  WT.Percent  HET.Percent  HOMO.Percent
Aspirin      467  305    89    54.23926     35.42393     10.336818
Celecoxib     35   24     5    54.68750     37.50000      7.812500
Diclofenac    18   10     2    60.00000     33.33333      6.666667
Ibuprofen    145   88    22    56.86275     34.50980      8.627451
Naproxen     102   67    17    54.83871     36.02151      9.139785
```

```
Paracetamol   231 133   36   57.75000    33.25000     9.000000
No.Painrelief 236 184   52   50.00000    38.98305    11.016949

APOE4.table.chisq<-chisq.test(APOE4.table[,1:3])
APOE4.table.chisq


    Pearson's Chi-squared test

data:  APOE4.table[, 1:3]
X-squared = 7.5119, df = 12, p-value = 0.822
```

6.4.6. Gender

```
Aspirin<-table(initialdata$Gender,by=initialdata$aspirin)[,2]
Celecoxib<-table(initialdata$Gender,by=initialdata$celex)[,2]
Diclofenac<-table(initialdata$Gender,by=initialdata$diclo)[,2]
Ibuprofen<-table(initialdata$Gender,by=initialdata$Ibu)[,2]
Naproxen<-table(initialdata$Gender,by=initialdata$naprox)[,2]
Paracetamol<-table(initialdata$Gender,by=initialdata$parac)[,2]
No.Painrelief<-table(initialdata$Gender,by=initialdata$No.Painrelief)[,2]


Gender.table<-
data.frame(t(cbind(Aspirin,Celecoxib,Diclofenac,Ibuprofen,Naproxen,Paracetamol,N
o.Painrelief)))
names(Gender.table)<-c("Male","Female")
Gender.table$Male.Percent<-
Gender.table[,1]/(Gender.table[,2]+Gender.table[,1])*100
Gender.table$Female.Percent<-
Gender.table[,2]/(Gender.table[,2]+Gender.table[,1])*100


Gender.table

              Male Female Male.Percent Female.Percent
Aspirin        323    538     37.51452       62.48548
Celecoxib       31     33     48.43750       51.56250
Diclofenac      15     15     50.00000       50.00000
Ibuprofen      117    138     45.88235       54.11765
Naproxen        95     91     51.07527       48.92473
Paracetamol    204    196     51.00000       49.00000
No.Painrelief  223    249     47.24576       52.75424

Gender.table.chisq<-chisq.test(Gender.table[,1:2])
Gender.table.chisq


    Pearson's Chi-squared test

data:  Gender.table[, 1:2]
X-squared = 29.522, df = 6, p-value = 4.844e-05

contrib<-100*Gender.table.chisq$residuals^2/Gender.table.chisq$statistic
contrib

                    Male     Female
Aspirin       31.5133437 25.2106750
Celecoxib      0.7777227  0.6221781
Diclofenac     0.7056841  0.5645473
```

```
Ibuprofen      0.4018248  0.3214599
Naproxen       6.2327840  4.9862272
Paracetamol   13.1012605 10.4810084
No.Painrelief  2.8229357  2.2583486

res1 <- cor.mtest(contrib, conf.level = 0.95)

col2 <- colorRampPalette(c("#67001F", "#B2182B", "#D6604D", "#F4A582",
                           "#FDDBC7", "#FFFFFF", "#D1E5F0", "#92C5DE",
                           "#4393C3", "#2166AC", "#053061"))


pearson<-Gender.table.chisq$residuals


corrplot(contrib, p.mat = res1$p, insig = "blank",pch.col=1,is.cor=F,tl.col=1,
cl.pos="b", number.digits=2,number.cex=0.5,cl.cex=1,cl.length=3, cl.lim=c(0,50),
col=rev(col2(200)))
```

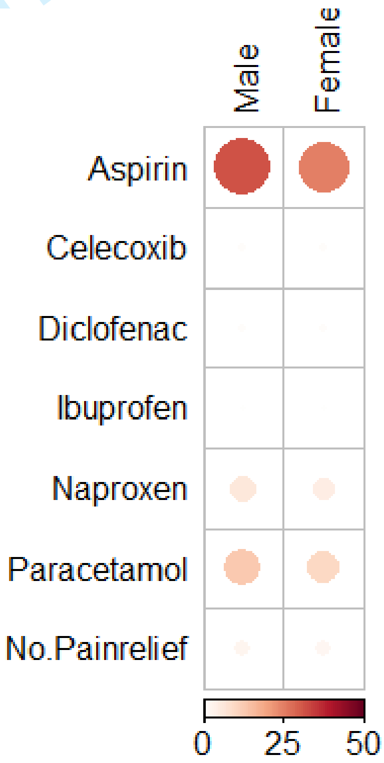


```
corrplot(pearson, p.mat = res1$p, insig = "blank",pch.col=1,is.cor=F,tl.col=1,
cl.pos="b", number.digits=2,number.cex=0.5,cl.cex=1,cl.length=3, cl.lim=c(-
8,8),col=rev(col2(200)))
```

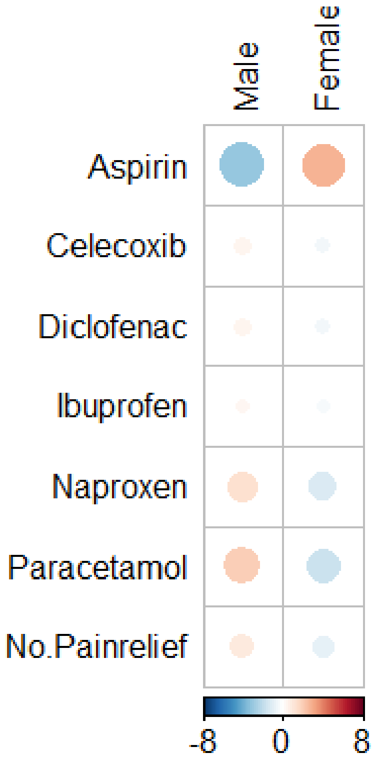

6.4.7. AGE

```
Aspirin<-mean(initialdata$AGEraw[initialdata$aspirin==1])
Aspirin.sd<-sd(initialdata$AGEraw[initialdata$aspirin==1])
Celecoxib<-mean(initialdata$AGEraw[initialdata$celex==1])
Celecoxib.sd<-sd(initialdata$AGEraw[initialdata$celex==1])
Diclofenac<-mean(initialdata$AGEraw[initialdata$diclo==1])
Diclofenac.sd<-sd(initialdata$AGEraw[initialdata$diclo==1])
Ibuprofen<-mean(initialdata$AGEraw[initialdata$Ibu==1])
Ibuprofen.sd<-sd(initialdata$AGEraw[initialdata$Ibu==1])
Naproxen<-mean(initialdata$AGEraw[initialdata$naprox==1])
Naproxen.sd<-sd(initialdata$AGEraw[initialdata$naprox==1])
Paracetamol<-mean(initialdata$AGEraw[initialdata$parac==1])
Paracetamol.sd<-sd(initialdata$AGEraw[initialdata$parac==1])

No.Painrelief<-mean(initialdata$AGEraw[initialdata$No.Painrelief==1])
No.Painrelief.sd<-sd(initialdata$AGEraw[initialdata$No.Painrelief==1])

AGE.table<-
data.frame(Aspirin,Aspirin.sd,Celecoxib,Celecoxib.sd,Diclofenac,Diclofenac.sd,Ib
uprofen,Ibuprofen.sd,Naproxen,Naproxen.sd,Paracetamol,Paracetamol.sd,No.Painreli
ef,No.Painrelief.sd)

AGE.table

   Aspirin Aspirin.sd Celecoxib Celecoxib.sd Diclofenac Diclofenac.sd
1 74.26469   6.685986  73.42344     6.940146      75.22       6.37362
  Ibuprofen Ibuprofen.sd Naproxen Naproxen.sd Paracetamol Paracetamol.sd
1   72.7149     6.809825  72.8957    7.085919      74.345       7.224428
  No.Painrelief No.Painrelief.sd
1      73.89915         8.079612
```

```
lm0<-lm(AGEraw~1,data=initialdata)
lm1<-lm(AGEraw~aspirin+ celex+diclo+Ibu+naprox+parac,data=initialdata)
summary(lm1)


Call:
lm(formula = AGEraw ~ aspirin + celex + diclo + Ibu + naprox +
    parac, data = initialdata)

Residuals:
     Min       1Q   Median       3Q      Max
-19.4619  -4.4620   0.0369   5.0772  18.4227

Coefficients:
            Estimate Std. Error t value Pr(>|t|)
(Intercept)  73.6222     0.2877 255.912  < 2e-16 ***
aspirin       0.8141     0.3616   2.252  0.02447 *
celex        -0.8279     0.9264  -0.894  0.37164
diclo         1.1589     1.3345   0.868  0.38530
Ibu          -1.4590     0.4977  -2.931  0.00342 **
naprox       -1.2129     0.5718  -2.121  0.03406 *
parac         0.8397     0.4267   1.968  0.04927 *
---
Signif. codes:  0 '***' 0.001 '**' 0.01 '*' 0.05 '.' 0.1 ' ' 1

Residual standard error: 7.218 on 1612 degrees of freedom
Multiple R-squared:  0.01352,   Adjusted R-squared:  0.009851
F-statistic: 3.683 on 6 and 1612 DF,  p-value: 0.001224

anova(lm0,lm1)

Analysis of Variance Table

Model 1: AGEraw ~ 1
Model 2: AGEraw ~ aspirin + celex + diclo + Ibu + naprox + parac
  Res.Df   RSS Df Sum of Sq      F   Pr(>F)
1   1618 85132
2   1612 83981  6    1151.2 3.6829 0.001224 **
---
Signif. codes:  0 '***' 0.001 '**' 0.01 '*' 0.05 '.' 0.1 ' ' 1

par(mfrow=c(2,2)); plot(lm1)
```

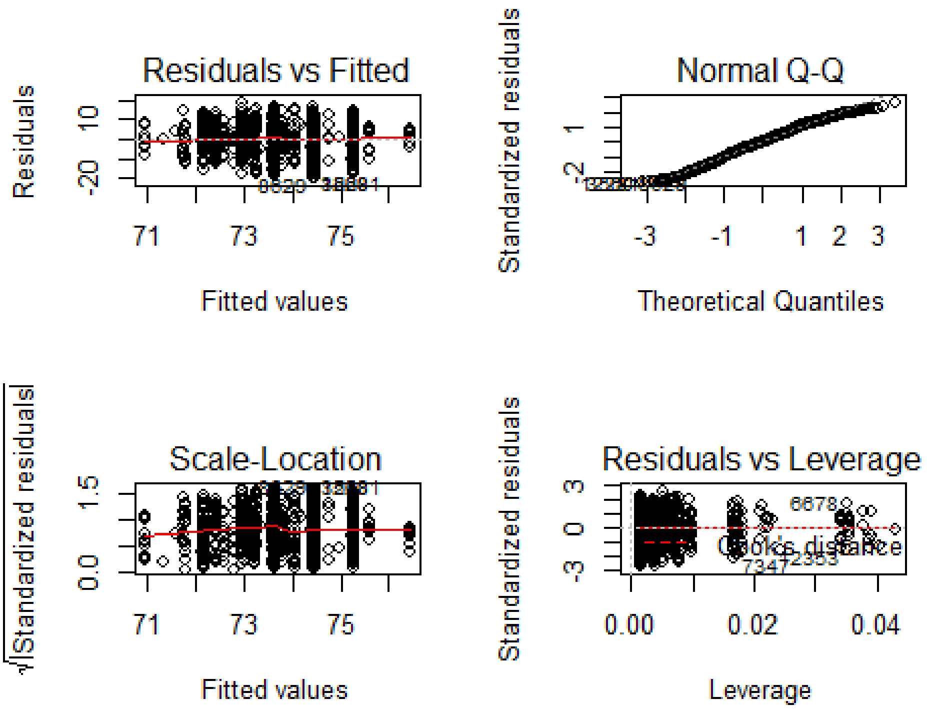


```
aspirin.lm1<-lm(AGEraw~ celex+diclo+Ibu+naprox+parac,data=initialdata)
celex.lm1<-lm(AGEraw~aspirin+ diclo+Ibu+naprox+parac,data=initialdata)
diclo.lm1<-lm(AGEraw~aspirin+ celex+Ibu+naprox+parac,data=initialdata)
Ibu.lm1<-lm(AGEraw~aspirin+ celex+diclo+naprox+parac,data=initialdata)
naprox.lm1<-lm(AGEraw~aspirin+ celex+diclo+Ibu+parac,data=initialdata)
parac.lm1<-lm(AGEraw~aspirin+ celex+diclo+Ibu+naprox,data=initialdata)

Drug<-c("Aspirin","Celecoxib","Diclofenac","Ibuprofen","Naproxen","Ibuprofen")
table<-cbind(Drug,rbind(anova(aspirin.lm1,lm1)[2,5:6],
anova(celex.lm1,lm1)[2,5:6],
anova(diclo.lm1,lm1)[2,5:6],
anova(Ibu.lm1,lm1)[2,5:6],
anova(naprox.lm1,lm1)[2,5:6],
anova(parac.lm1,lm1)[2,5:6]))
Adjusted.P.Value<-p.adjust(table[,3],method="bonferroni")
table<-cbind(table,Adjusted.P.Value)
table

         Drug         F      Pr(>F) Adjusted.P.Value
2     Aspirin 5.0703195 0.024473042       0.14683825
21  Celecoxib 0.7986302 0.371636630       1.00000000
22 Diclofenac 0.7541348 0.385299554       1.00000000
23  Ibuprofen 8.5926115 0.003422854       0.02053712
24   Naproxen 4.4994038 0.034058935       0.20435361
25  Ibuprofen 3.8720763 0.049266946       0.29560168
```

6.4.8. Smoking status

```
Aspirin<-table(initialdata$smoke,by=initialdata$aspirin)[,2]
Celecoxib<-table(initialdata$smoke,by=initialdata$celex)[,2]
Diclofenac<-table(initialdata$smoke,by=initialdata$diclo)[,2]
Ibuprofen<-table(initialdata$smoke,by=initialdata$Ibu)[,2]
Naproxen<-table(initialdata$smoke,by=initialdata$naprox)[,2]
```

```
Paracetamol<-table(initialdata$smoke,by=initialdata$parac)[,2]
No.Painrelief<-table(initialdata$smoke,by=initialdata$No.Painrelief)[,2]

Smoking.table<-
data.frame(t(cbind(Aspirin,Celecoxib,Diclofenac,Ibuprofen,Naproxen,Paracetamol,N
o.Painrelief)))
names(Smoking.table)<-c("Non-smoker","Smoker")
Smoking.table$percent<-
Smoking.table[,2]/(Smoking.table[,2]+Smoking.table[,1])*100
Smoking.table

              Non-smoker Smoker  percent
Aspirin              656    205 23.80952
Celecoxib             47     17 26.56250
Diclofenac            22      8 26.66667
Ibuprofen            184     71 27.84314
Naproxen             146     40 21.50538
Paracetamol          293    107 26.75000
No.Painrelief        345    127 26.90678

chisq.test(Smoking.table[,1:2])


    Pearson's Chi-squared test

data:  Smoking.table[, 1:2]
X-squared = 4.4658, df = 6, p-value = 0.6139
```

6.4.9. Arthritis

```
Aspirin<-table(initialdata$arthrit,by=initialdata$aspirin)[,2]
Celecoxib<-table(initialdata$arthrit,by=initialdata$celex)[,2]
Diclofenac<-table(initialdata$arthrit,by=initialdata$diclo)[,2]
Ibuprofen<-table(initialdata$arthrit,by=initialdata$Ibu)[,2]
Naproxen<-table(initialdata$arthrit,by=initialdata$naprox)[,2]
Paracetamol<-table(initialdata$arthrit,by=initialdata$parac)[,2]
No.Painrelief<-table(initialdata$arthrit,by=initialdata$No.Painrelief)[,2]

Arthritis.table<-
data.frame(t(cbind(Aspirin,Celecoxib,Diclofenac,Ibuprofen,Naproxen,Paracetamol,N
o.Painrelief)))
names(Arthritis.table)<-c("Non-Arthritis","Arthritis")
Arthritis.table$percent<-
Arthritis.table[,2]/(Arthritis.table[,2]+Arthritis.table[,1])*100
Arthritis.table

              Non-Arthritis Arthritis  percent
Aspirin                 519       342 39.72125
Celecoxib                17        47 73.43750
Diclofenac                7        23 76.66667
Ibuprofen               128       127 49.80392
Naproxen                 88        98 52.68817
Paracetamol             185       215 53.75000
No.Painrelief           340       132 27.96610

chisq.test(Arthritis.table[,1:2])


    Pearson's Chi-squared test
```

```
data:  Arthritis.table[, 1:2]
X-squared = 115.78, df = 6, p-value < 2.2e-16

chisq<-chisq.test(Arthritis.table[,1:2])


chisq


    Pearson's Chi-squared test

data:  Arthritis.table[, 1:2]
X-squared = 115.78, df = 6, p-value < 2.2e-16

contrib<-100*chisq$residuals^2/chisq$statistic
contrib

              Non-Arthritis Arthritis
Aspirin            1.764408  2.302338
Celecoxib          8.817711 11.506037
Diclofenac         5.069318  6.614842
Ibuprofen          1.602310  2.090820
Naproxen           2.455328  3.203904
Paracetamol        6.554582  8.552930
No.Painrelief     17.122585 22.342886

res1 <- cor.mtest(contrib, conf.level = 0.95)

col2 <- colorRampPalette(c("#67001F", "#B2182B", "#D6604D", "#F4A582",
                           "#FDDBC7", "#FFFFFF", "#D1E5F0", "#92C5DE",
                           "#4393C3", "#2166AC", "#053061"))


pearson<-chisq$residuals


corrplot(contrib, p.mat = res1$p, insig = "blank",pch.col=1,is.cor=F,tl.col=1,
cl.pos="b", number.digits=2,number.cex=0.5,cl.cex=1,cl.length=3, cl.lim=c(0,50),
col=rev(col2(200)))
```

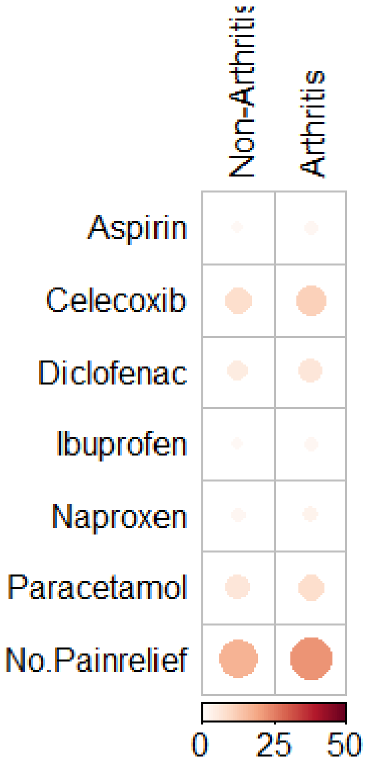


```
corrplot(pearson, p.mat = res1$p, insig = "blank",pch.col=1,is.cor=F,tl.col=1,
cl.pos="b", number.digits=2,number.cex=0.5,cl.cex=1,cl.length=3, cl.lim=c(-
8,8),col=rev(col2(200)))
```

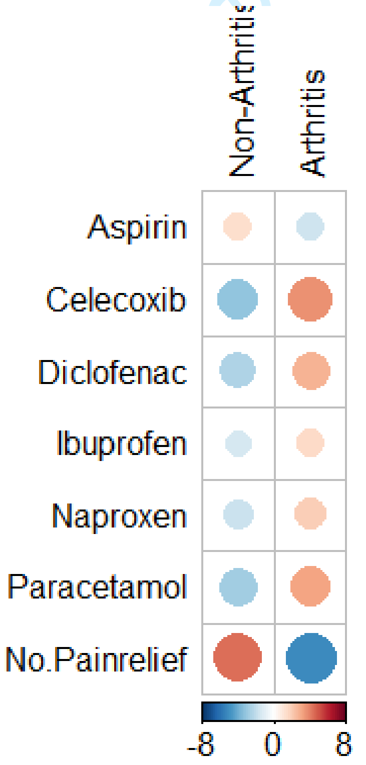

6.4.10. Headache

```
Aspirin<-table(initialdata$headache,by=initialdata$aspirin)[,2]
Celecoxib<-table(initialdata$headache,by=initialdata$celex)[,2]
Diclofenac<-table(initialdata$headache,by=initialdata$diclo)[,2]
Ibuprofen<-table(initialdata$headache,by=initialdata$Ibu)[,2]
Naproxen<-table(initialdata$headache,by=initialdata$naprox)[,2]
Paracetamol<-table(initialdata$headache,by=initialdata$parac)[,2]
No.Painrelief<-table(initialdata$headache,by=initialdata$No.Painrelief)[,2]


Headache.table<-
data.frame(t(cbind(Aspirin,Celecoxib,Diclofenac,Ibuprofen,Naproxen,Paracetamol,N
o.Painrelief)))
names(Headache.table)<-c("Non-Headache","Headache")
Headache.table$percent<-
Headache.table[,2]/(Headache.table[,2]+Headache.table[,1])*100
Headache.table

              Non-Headache Headache   percent
Aspirin                794       67  7.781649
Celecoxib               53       11 17.187500
Diclofenac              26        4 13.333333
Ibuprofen              224       31 12.156863
Naproxen               166       20 10.752688
Paracetamol            348       52 13.000000
No.Painrelief          439       33  6.991525

chisq.test(Headache.table[,1:2])


    Pearson's Chi-squared test

data:  Headache.table[, 1:2]
X-squared = 19.22, df = 6, p-value = 0.003807

chisq<-chisq.test(Headache.table[,1:2])


chisq


    Pearson's Chi-squared test

data:  Headache.table[, 1:2]
X-squared = 19.22, df = 6, p-value = 0.003807

contrib<-100*chisq$residuals^2/chisq$statistic
contrib

              Non-Headache  Headache
Aspirin          1.6603313 15.613207
Celecoxib        2.1141203 19.880489
Diclofenac       0.2391368  2.248763
Ibuprofen        0.9505996  8.939125
Naproxen         0.1393088  1.310014
Paracetamol      2.6428646 24.852626
No.Painrelief    1.8656315 17.543783

res1 <- cor.mtest(contrib, conf.level = 0.95)
```

```
col2 <- colorRampPalette(c("#67001F", "#B2182B", "#D6604D", "#F4A582",
                           "#FDDBC7", "#FFFFFF", "#D1E5F0", "#92C5DE",
                           "#4393C3", "#2166AC", "#053061"))


pearson<-chisq$residuals


corrplot(contrib, p.mat = res1$p, insig = "blank",pch.col=1,is.cor=F,tl.col=1,
cl.pos="b", number.digits=2,number.cex=0.5,cl.cex=1,cl.length=3, cl.lim=c(0,50),
col=rev(col2(200)))
```

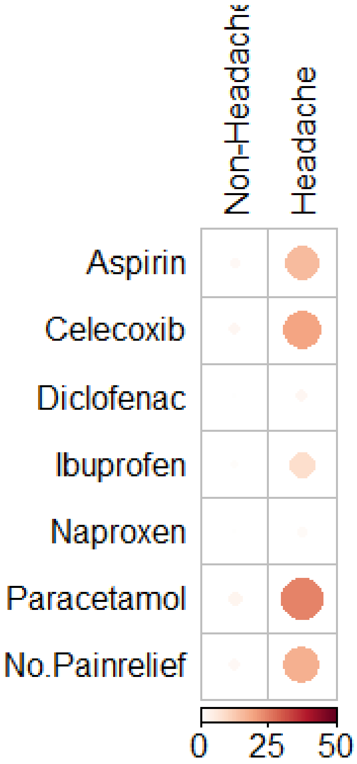


```
corrplot(pearson, p.mat = res1$p, insig = "blank",pch.col=1,is.cor=F,tl.col=1,
cl.pos="b", number.digits=2,number.cex=0.5,cl.cex=1,cl.length=3, cl.lim=c(-
8,8),col=rev(col2(200)))
```

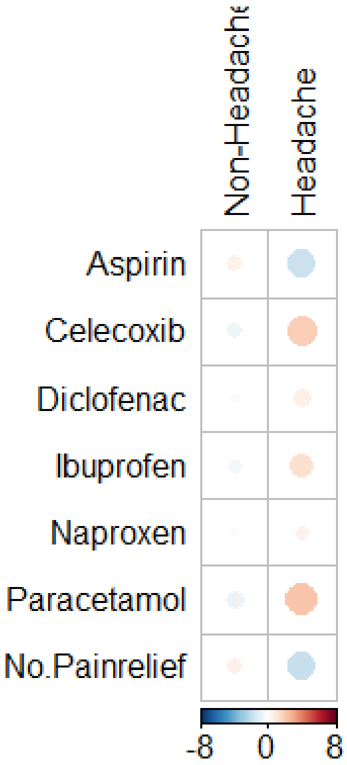

6.4.11. Cardiovascular disease

```
Aspirin<-table(initialdata$vasc,by=initialdata$aspirin)[,2]
Celecoxib<-table(initialdata$vasc,by=initialdata$celex)[,2]
Diclofenac<-table(initialdata$vasc,by=initialdata$diclo)[,2]
Ibuprofen<-table(initialdata$vasc,by=initialdata$Ibu)[,2]
Naproxen<-table(initialdata$vasc,by=initialdata$naprox)[,2]
Paracetamol<-table(initialdata$vasc,by=initialdata$parac)[,2]
No.Painrelief<-table(initialdata$vasc,by=initialdata$No.Painrelief)[,2]

Cardiovascular.Pathology.Table<-
data.frame(t(cbind(Aspirin,Celecoxib,Diclofenac,Ibuprofen,Naproxen,Paracetamol,N
o.Painrelief)))
names(Cardiovascular.Pathology.Table)<-c("Non-cardiovascular","Cardiovascular")
Cardiovascular.Pathology.Table$percent<-
Cardiovascular.Pathology.Table[,2]/(Cardiovascular.Pathology.Table[,2]+Cardiovas
cular.Pathology.Table[,1])*100
Cardiovascular.Pathology.Table

              Non-cardiovascular Cardiovascular  percent
Aspirin                      306            555 64.45993
Celecoxib                     20             44 68.75000
Diclofenac                    14             16 53.33333
Ibuprofen                     97            158 61.96078
Naproxen                      72            114 61.29032
Paracetamol                  127            273 68.25000
No.Painrelief                211            261 55.29661

chisq<-chisq.test(Cardiovascular.Pathology.Table[,1:2])

chisq
```

```
    Pearson's Chi-squared test

data:  Cardiovascular.Pathology.Table[, 1:2]
X-squared = 19.803, df = 6, p-value = 0.003002

contrib<-100*chisq$residuals^2/chisq$statistic
contrib

              Non-cardiovascular Cardiovascular
Aspirin                3.7955384     2.26236526
Celecoxib              3.2154874     1.91662058
Diclofenac             3.5242587     2.10066652
Ibuprofen              0.1658447     0.09885327
Naproxen               0.4679111     0.27890267
Paracetamol           16.9351185    10.09433173
No.Painrelief         34.5501621    20.59393898

res1 <- cor.mtest(contrib, conf.level = 0.95)

col2 <- colorRampPalette(c("#67001F", "#B2182B", "#D6604D", "#F4A582",
                           "#FDDBC7", "#FFFFFF", "#D1E5F0", "#92C5DE",
                           "#4393C3", "#2166AC", "#053061"))


pearson<-chisq$residuals


corrplot(contrib, p.mat = res1$p, insig = "blank",pch.col=1,is.cor=F,tl.col=1,
cl.pos="b", number.digits=2,number.cex=0.5,cl.cex=1,cl.length=3, cl.lim=c(0,50),
col=rev(col2(200)))
```

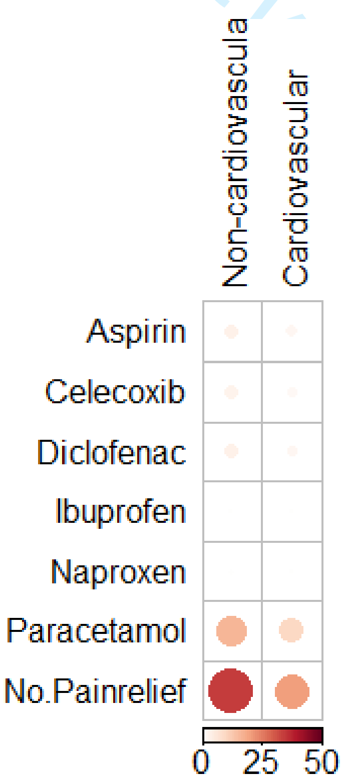

```
corrplot(pearson, p.mat = res1$p, insig = "blank",pch.col=1,is.cor=F,tl.col=1,
cl.pos="b", number.digits=2,number.cex=0.5,cl.cex=1,cl.length=3, cl.lim=c(-
8,8),col=rev(col2(200)))
```

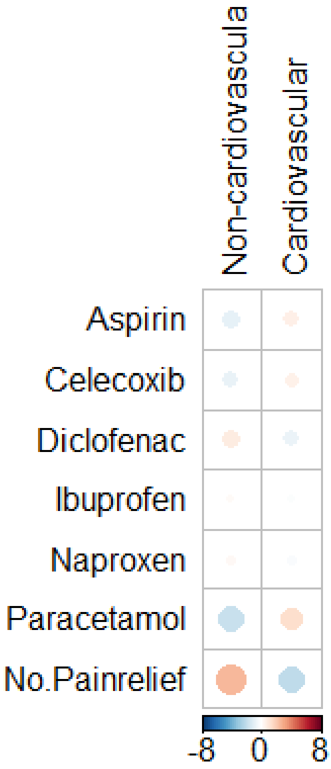

6.4.12. Diabetes

```
Aspirin<-table(initialdata$diab,by=initialdata$aspirin)[,2]
Celecoxib<-table(initialdata$diab,by=initialdata$celex)[,2]
Diclofenac<-table(initialdata$diab,by=initialdata$diclo)[,2]
Ibuprofen<-table(initialdata$diab,by=initialdata$Ibu)[,2]
Naproxen<-table(initialdata$diab,by=initialdata$naprox)[,2]
Paracetamol<-table(initialdata$diab,by=initialdata$parac)[,2]
No.Painrelief<-table(initialdata$headache,by=initialdata$No.Painrelief)[,2]


Diabetes.Pathology.Table<-
data.frame(t(cbind(Aspirin,Celecoxib,Diclofenac,Ibuprofen,Naproxen,Paracetamol,N
o.Painrelief)))
names(Diabetes.Pathology.Table)<-c("Non-Diabetes","Diabetes")
Diabetes.Pathology.Table$percent<-
Diabetes.Pathology.Table[,2]/(Cardiovascular.Pathology.Table[,2]+Diabetes.Pathol
ogy.Table[,1])*100
Diabetes.Pathology.Table

              Non-Diabetes Diabetes  percent
Aspirin                769       92 6.948640
Celecoxib               57        7 6.930693
Diclofenac              26        4 9.523810
Ibuprofen              229       26 6.718346
Naproxen               165       21 7.526882
Paracetamol            354       46 7.336523
No.Painrelief          439       33 4.714286
```

```
chisq.test(Diabetes.Pathology.Table[,1:2])


    Pearson's Chi-squared test

data:  Diabetes.Pathology.Table[, 1:2]
X-squared = 6.9006, df = 6, p-value = 0.3301
```

For Review Only

7. Analysis of cognitive decline using the MMSE score

Mini-mental state examination (MMSE) is a cognitive assessment with a focus on memory that is often used as the primary measure of Alzheimer's disease progression in clinical trials. It is a score out of 30 with higher scores corrisponding to better cognitive performance.

7.2. *Dependent variable check*

Rows with no MMSE score were removed and variables were checked for correct categorization.

```
data<-read.csv("CleanedFinalData.csv", header=T)
MMSEdata<-data[!is.na(data$MMSE),]
MMSEdata$ID<-as.factor(MMSEdata$ID)
MMSEdata$APOE4<-as.factor(MMSEdata$APOE4)
```

7.3. *Generation of dependent variables appropriate for different distributions*

```
MMSEdata$neg.b.MMSE<-round(30-MMSEdata$MMSE)
MMSEdata$fail<-abs(30-MMSEdata$MMSE)
MMSEdata$success<-30-MMSEdata$fail
MMSEdata$MMSEscore<-rep(30,length(MMSEdata$neg.b.MMSE))
MMSEdata$proportion<-MMSEdata$fail/MMSEdata$MMSEscore
```

7.4. *Transformation to obtain normal approximation*

The total errors were then generated by contructing a linear model of with MMSE as the dependent variable and patient ID as the explainitory variable. It has been shown that if the total errors are homoscedastic and normal then the multi-level linear model errors will likely also be homoscedastic and normal distributed (Gurka *et al.* 2006). Therefore, total errors are an excellent starting point for model dianostics. From this it was found that even with boxcox optimized transformation the residuals while normally distributed fail to have even homoscidadicity. This is due to the categorical nature of the MMSE score and the high number of zero values. Therefore, a generalized linear model utilizing a non-gaussian distribution were performed

```
qqp(MMSEdata$MMSE[MMSEdata$M==0], dist="norm")
```

For Review Only

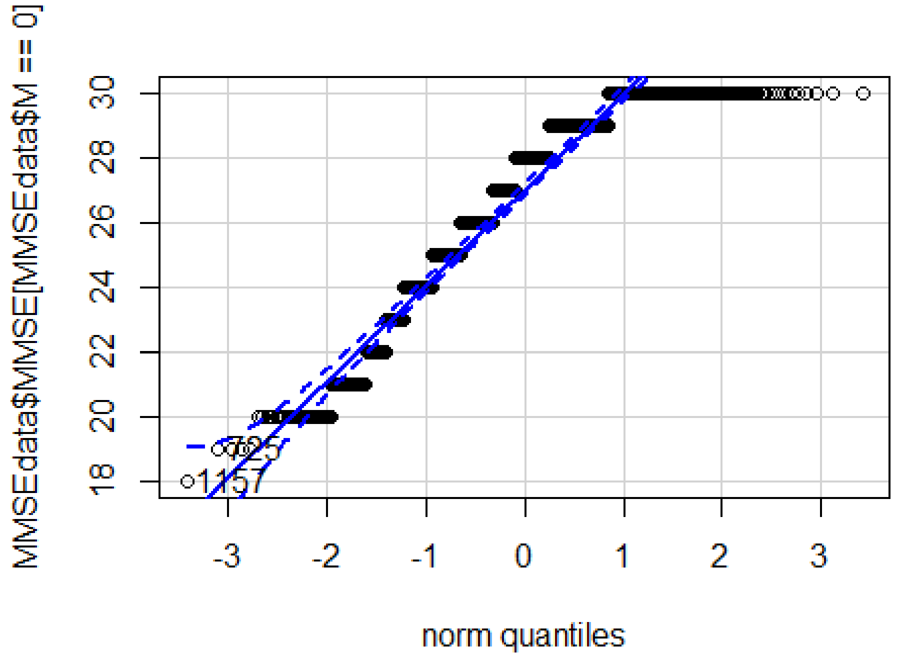


```
[1] 1157  725

m1<-lm((MMSE+1)~ID, data=MMSEdata)
par(mfrow=c(2,2))
plot(m1)
```

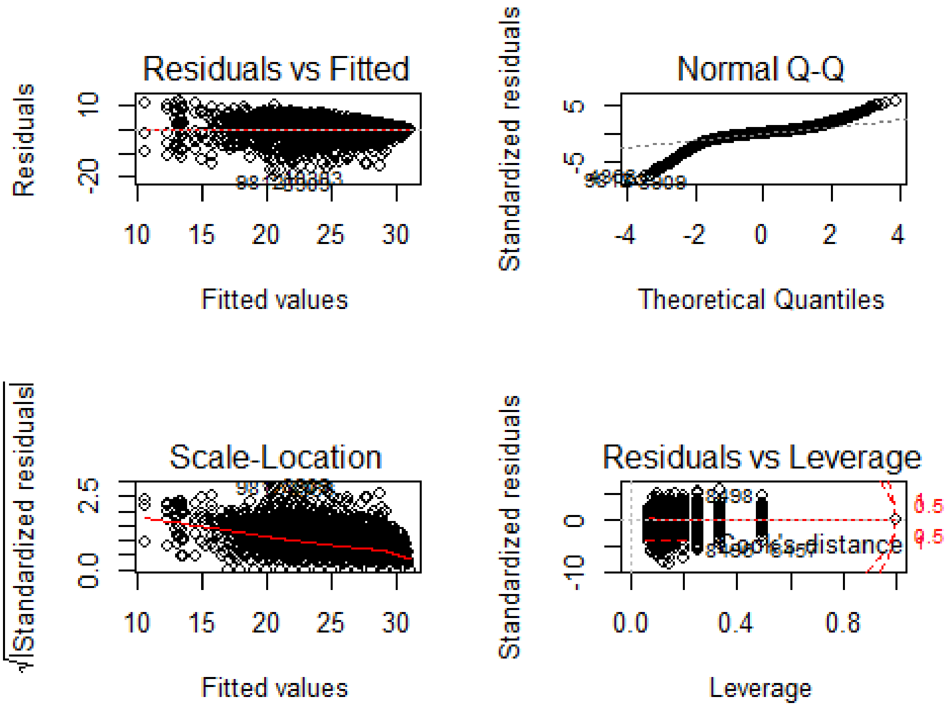

```
boxcox<-boxcox(m1,lambda = seq(-5, 5, 1/100),plotit = TRUE )
selectedlambda<-boxcox$x[boxcox$y==max(boxcox$y)]
selectedlambda

[1] 4.83

MMSEdata$tMMSE<-(MMSEdata$MMSE)^selectedlambda
m1.t<-lmer(tMMSE~diagn+edu.cat+Gender+APOE4+M+(1|ID),data=MMSEdata)
summary(m1.t)

Linear mixed model fit by REML. t-tests use Satterthwaite's method [
lmerModLmerTest]
Formula: tMMSE ~ diagn + edu.cat + Gender + APOE4 + M + (1 | ID)
   Data: MMSEdata

REML criterion at convergence: 286545.4

Scaled residuals:
    Min      1Q  Median      3Q     Max
-4.5078 -0.6006  0.0264  0.6219  3.6068

Random effects:
 Groups   Name        Variance  Std.Dev.
 ID       (Intercept) 4.469e+12 2113945
 Residual             4.556e+12 2134557
Number of obs: 8878, groups:  ID, 1619

Fixed effects:
                 Estimate Std. Error       df t value Pr(>|t|)
(Intercept)      13018776     159085     1681  81.835  < 2e-16 ***
diagn2EMCI       -1345340     176612     1585  -7.618 4.42e-14 ***
diagn3LMCI       -3879497     154816     1568 -25.059  < 2e-16 ***
diagn4AD         -7930905     185780     1801 -42.690  < 2e-16 ***
edu.cat2tertiary  -331073     147570     1624  -2.244    0.025 *
edu.cat3mid       -966745     167226     1624  -5.781 8.88e-09 ***
edu.cat4early    -1099664     177772     1666  -6.186 7.76e-10 ***
GenderMale        -192114     120445     1638  -1.595    0.111
APOE41            -756413     128778     1638  -5.874 5.15e-09 ***
APOE42           -1066035     205088     1672  -5.198 2.26e-07 ***
M                  -28174        952   922964 -29.595  < 2e-16 ***
---
Signif. codes:  0 '***' 0.001 '**' 0.01 '*' 0.05 '.' 0.1 ' ' 1

Correlation of Fixed Effects:
            (Intr) d2EMCI d3LMCI dgn4AD ed.ct2 ed.ct3 ed.ct4 GndrMl APOE41
diagn2EMCI  -0.419
diagn3LMCI  -0.450  0.500
diagn4AD    -0.349  0.430  0.524
ed.ct2trtry -0.416  0.013 -0.009 -0.043
edu.cat3mid -0.436 -0.025  0.025 -0.038  0.404
edu.cat4rly -0.345 -0.036 -0.069 -0.128  0.386  0.357
GenderMale  -0.452 -0.043 -0.081 -0.047  0.049  0.175  0.142
APOE41      -0.192 -0.094 -0.178 -0.207 -0.032 -0.032 -0.048  0.001
APOE42      -0.070 -0.077 -0.178 -0.235 -0.046 -0.029 -0.021 -0.012  0.306
M           -0.165  0.034  0.037  0.097  0.000 -0.003  0.003 -0.004  0.009
            APOE42
diagn2EMCI
diagn3LMCI
diagn4AD
ed.ct2trtry
```

```
edu.cat3mid
edu.cat4rly
GenderMale
APOE41
APOE42
M            0.007

mcp.fnc(m1.t)
```

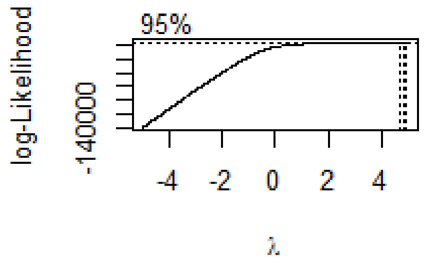

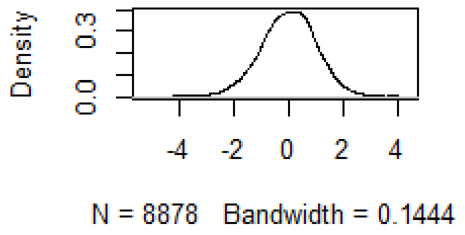

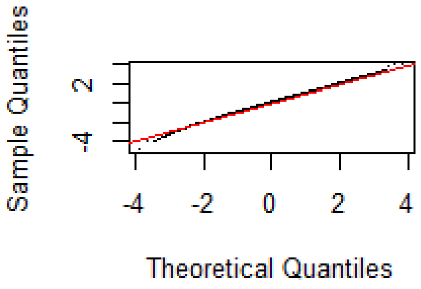

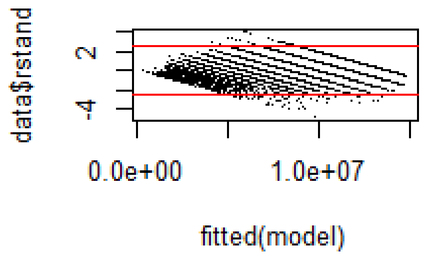

```
qqp(MMSEdata$tMMSE[MMSEdata$M==0], dist="norm")
```

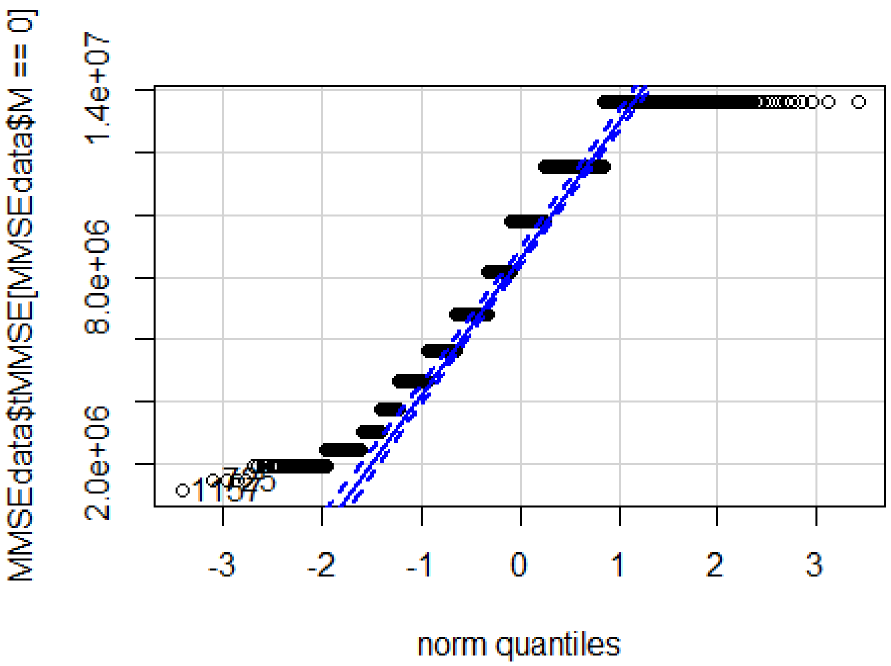


```
[1] 1157  725
```

7.5. *Selecting non-gaussian model*

The data was plotted against expected distributions including normal, poisson, negative binomial, binomial, exponential or gamma distributions within each time point. Some transformations were required including converting the MMSE score to the count of mistakes (30-MMSE) for the Poisson, negative binomial and exponential distributions. For the Poisson distribution there was oberdispersion as seen by the vast difference in mean and variance, therefore, poisson is not likely. This is also evident in the dependent variable not following a poisson distribution. The binomial model seems appropriate theoretically as the MMSE could be considered 30 trials with a proportion of failures occuring. However, the large number of zeros makes logistic regression of the binomial distribution inappropriate. The negative binomial and gamma models appear to be the most appropriate models. However, given the categorical nature of the data the negative binomial model was chosen for analysis. This is an approximate method for distribution selection as it does not take into account the explanitory variables effects on the distribution. This was approached used as a starting point for model analysis. Distributions were compared once the final model was established and the negative binomial model proved to be the most appropriate. Furthermore, residual analysis of the final selected model confirmed the appropriateness of the negative binomial model.

```
hist(MMSEdata$MMSE[MMSEdata$M==0])
```

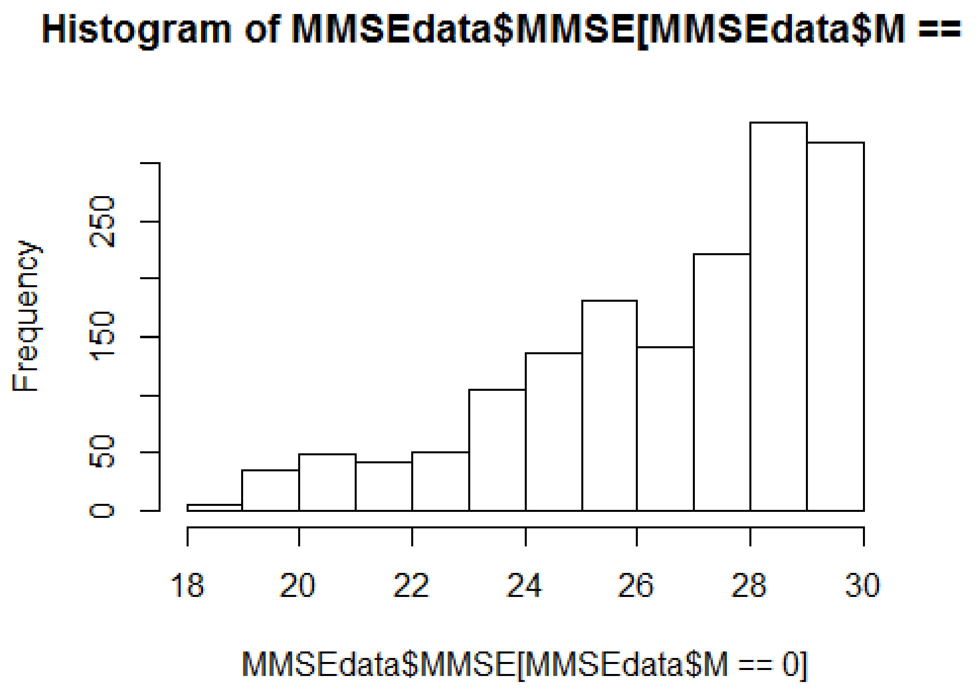


```
hist(MMSEdata$MMSE[MMSEdata$M==12])
```

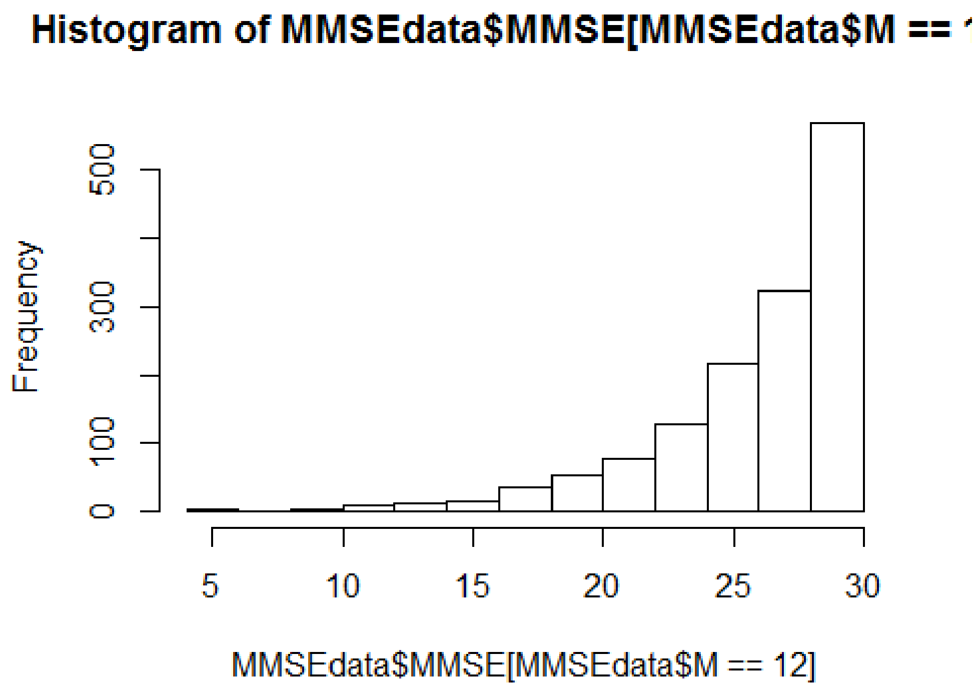


```
hist(MMSEdata$MMSE[MMSEdata$M==24])
```

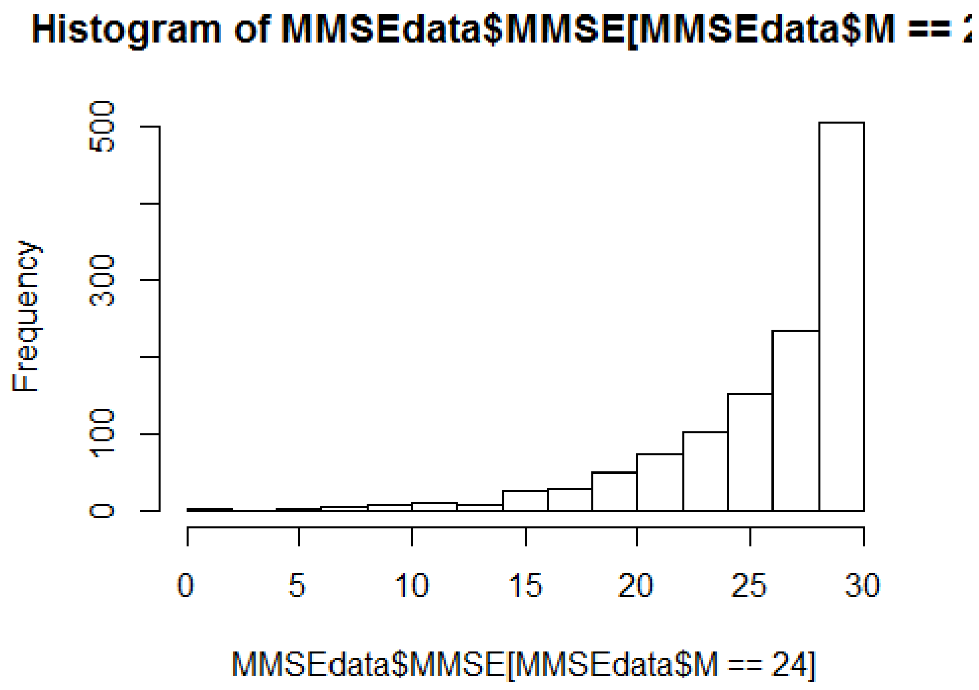


```
hist(MMSEdata$MMSE[MMSEdata$M==48])
```

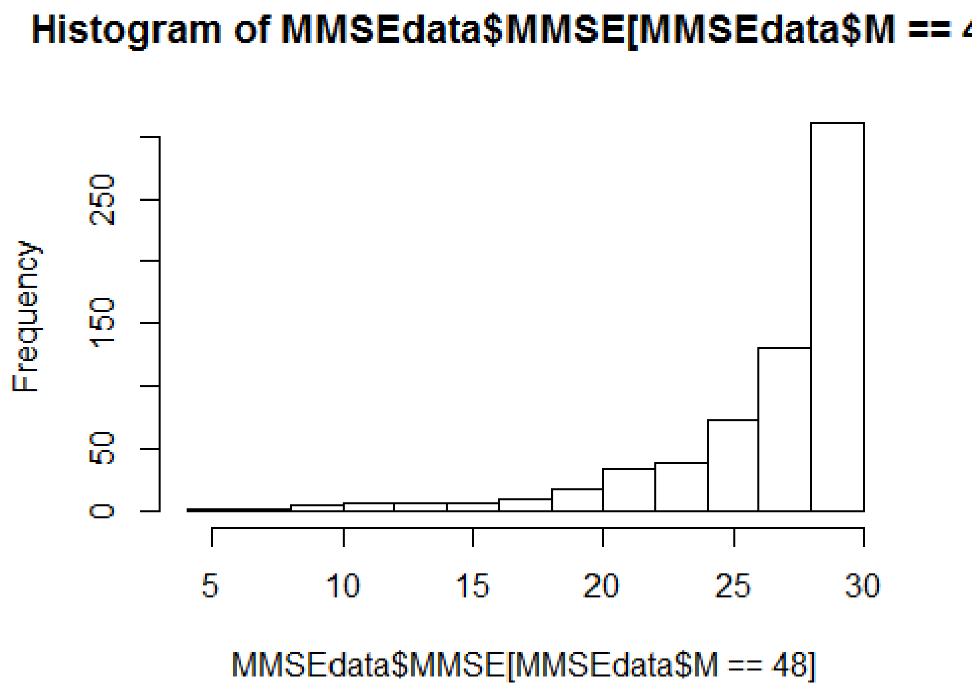


```
hist(MMSEdata$MMSE[MMSEdata$M==72])
```

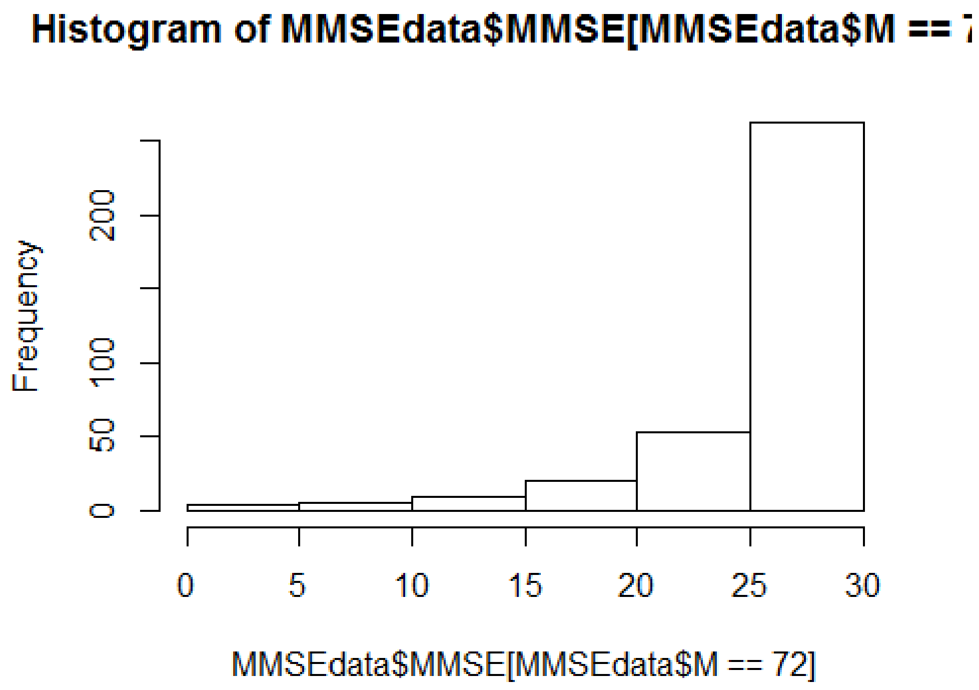


```
hist(MMSEdata$MMSE[MMSEdata$M==120])
```

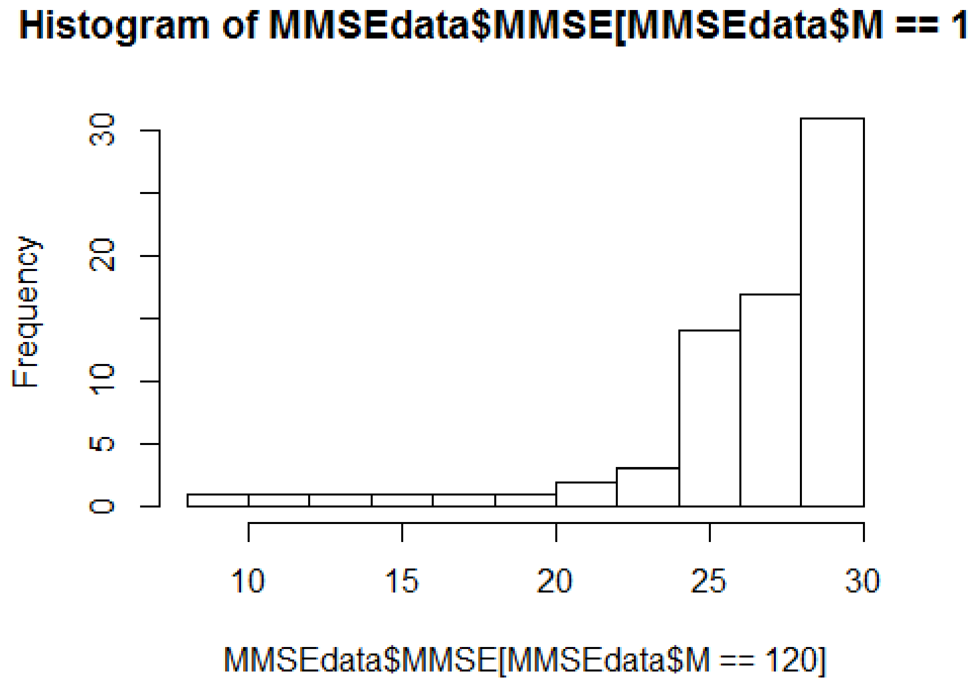


```
qqp(MMSEdata$MMSE[MMSEdata$M==0], "norm", main="Normal distribution model
Month=0")
```

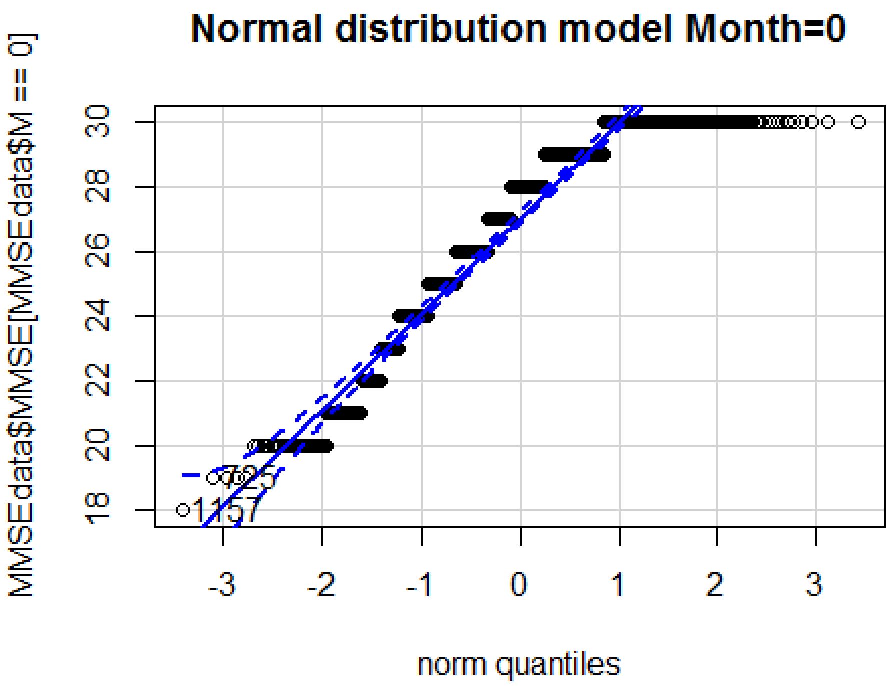


```
[1] 1157  725

qqp(MMSEdata$MMSE[MMSEdata$M==12], "norm", main="Normal distribution model
Month=12")
```

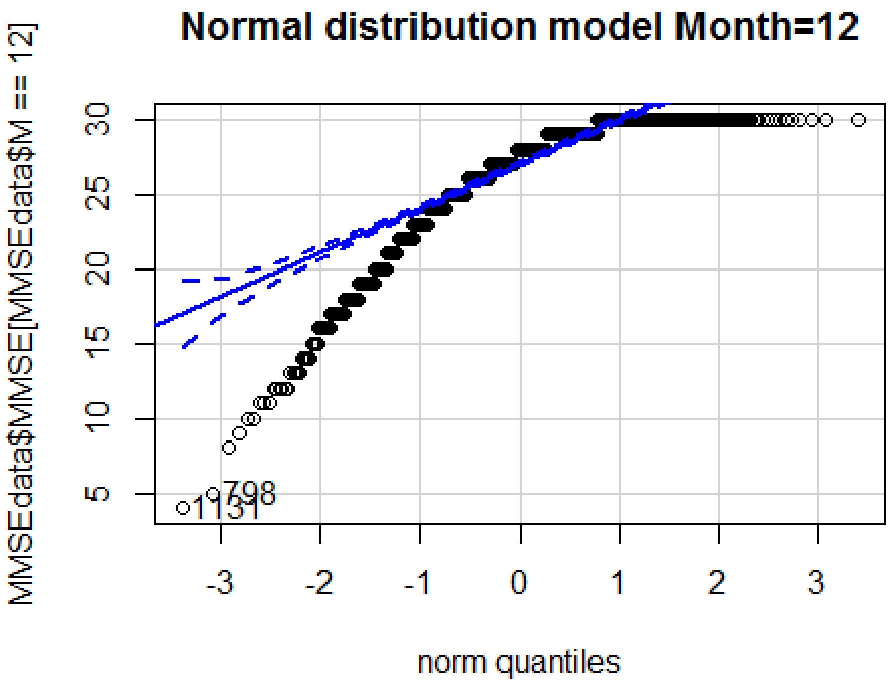


```
[1] 1131  798
```

```
qqp(MMSEdata$MMSE[MMSEdata$M==24], "norm", main="Normal distribution model Month=24")
```

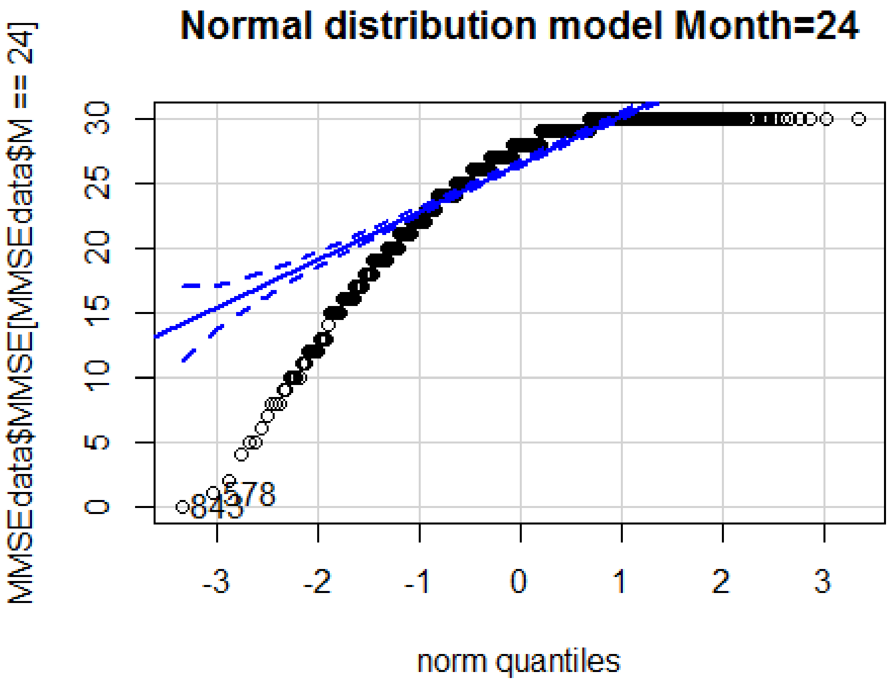


```
[1] 843 578

qqp(MMSEdata$MMSE[MMSEdata$M==48], "norm", main="Normal distribution model Month=48")
```

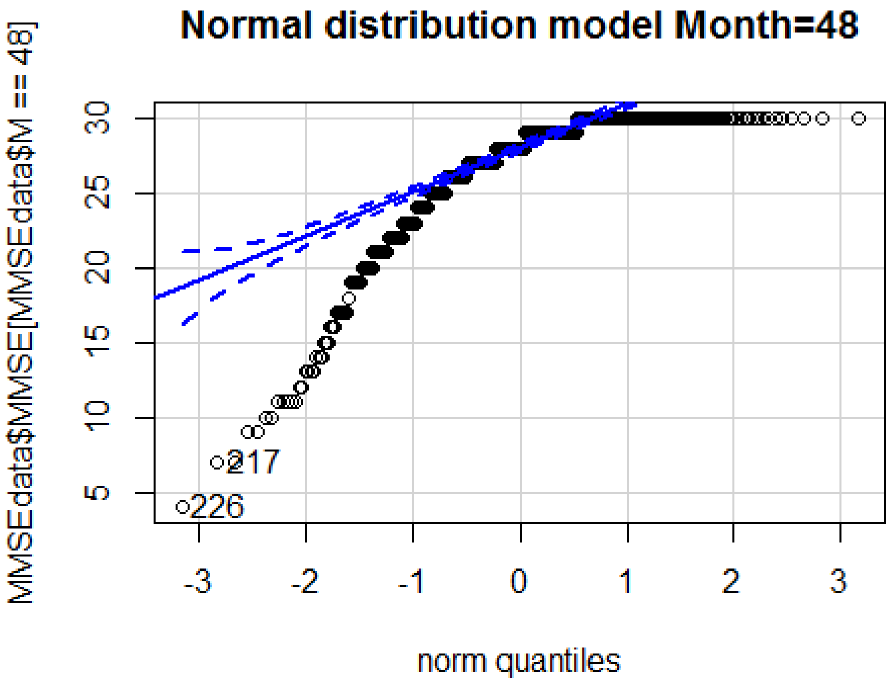

```
[1] 226 217

qqp(MMSEdata$MMSE[MMSEdata$M==72], "norm", main="Normal distribution model
Month=72")
```

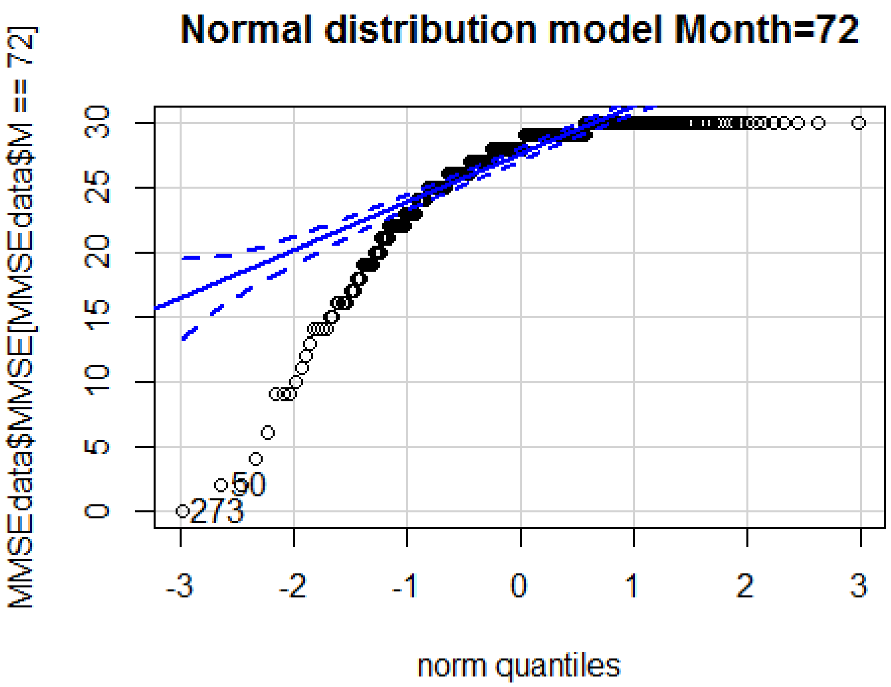


```
[1] 273  50

qqp(MMSEdata$MMSE[MMSEdata$M==120], "norm", main="Normal distribution model
Month=120")
```

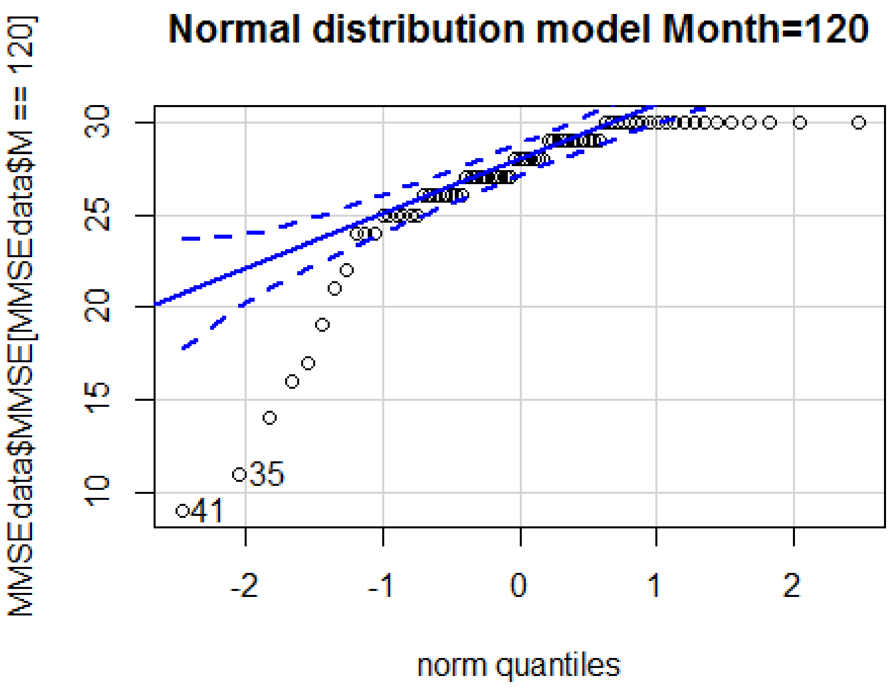


```
[1] 41 35
hist(MMSEdata$neg.b.MMSE[MMSEdata$M==0])
```

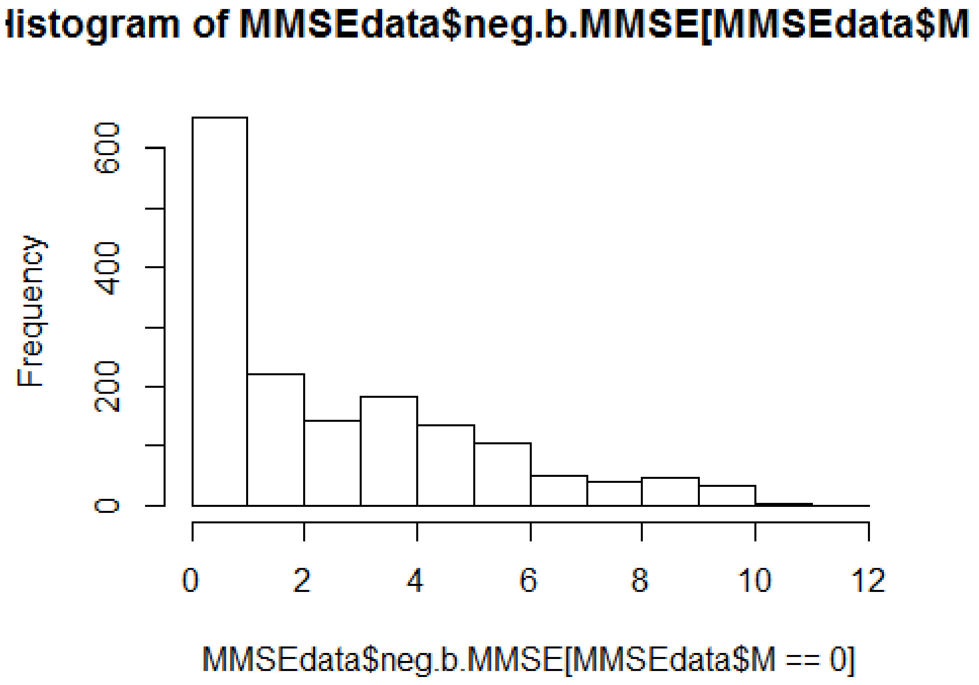


```
hist(MMSEdata$neg.b.MMSE[MMSEdata$M==12])
```

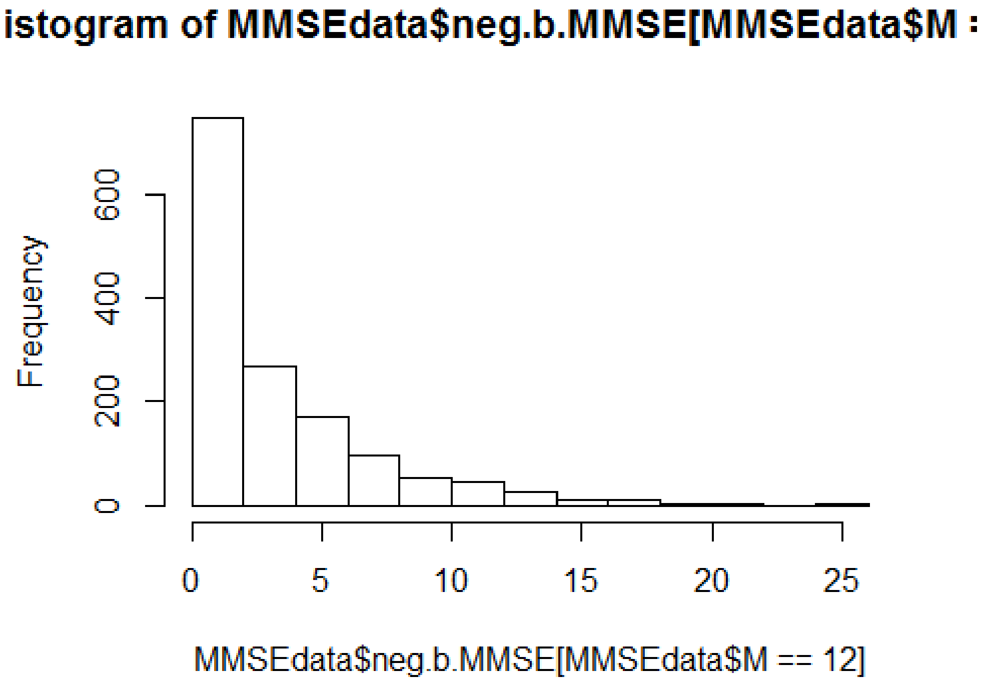


```
hist(MMSEdata$neg.b.MMSE[MMSEdata$M==24])
```

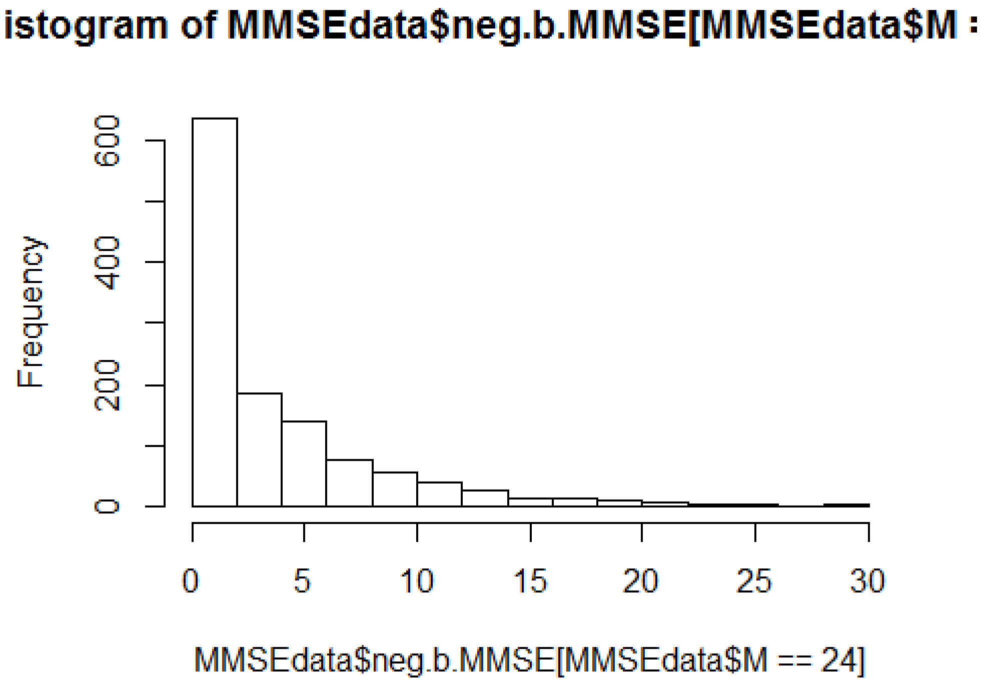


```
hist(MMSEdata$neg.b.MMSE[MMSEdata$M==48])
```

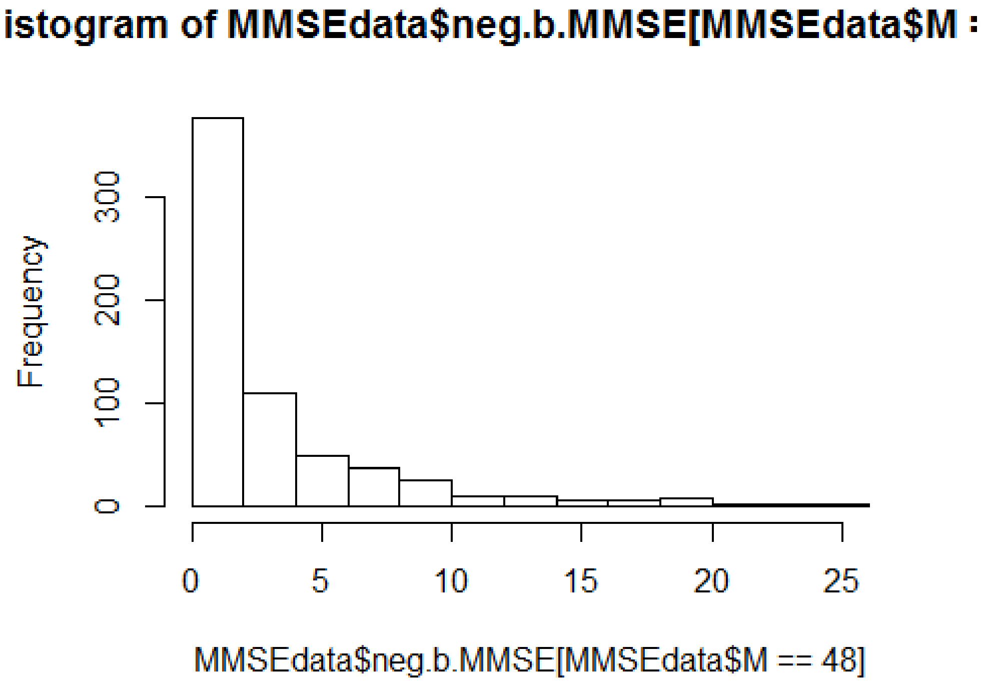


```
hist(MMSEdata$neg.b.MMSE[MMSEdata$M==72])
```

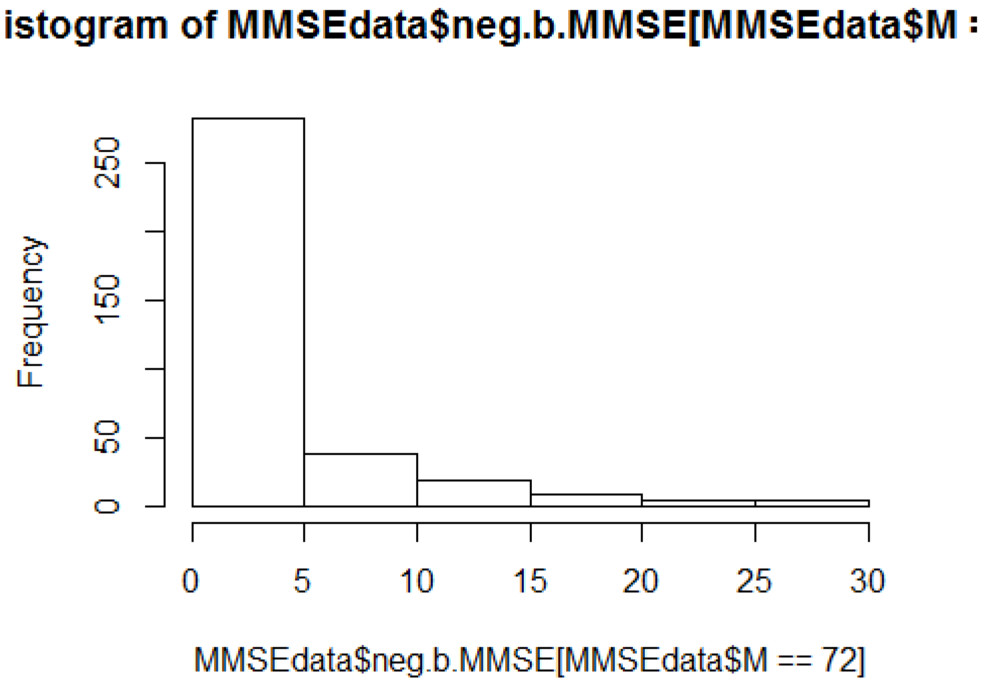


```
hist(MMSEdata$neg.b.MMSE[MMSEdata$M==120])
```

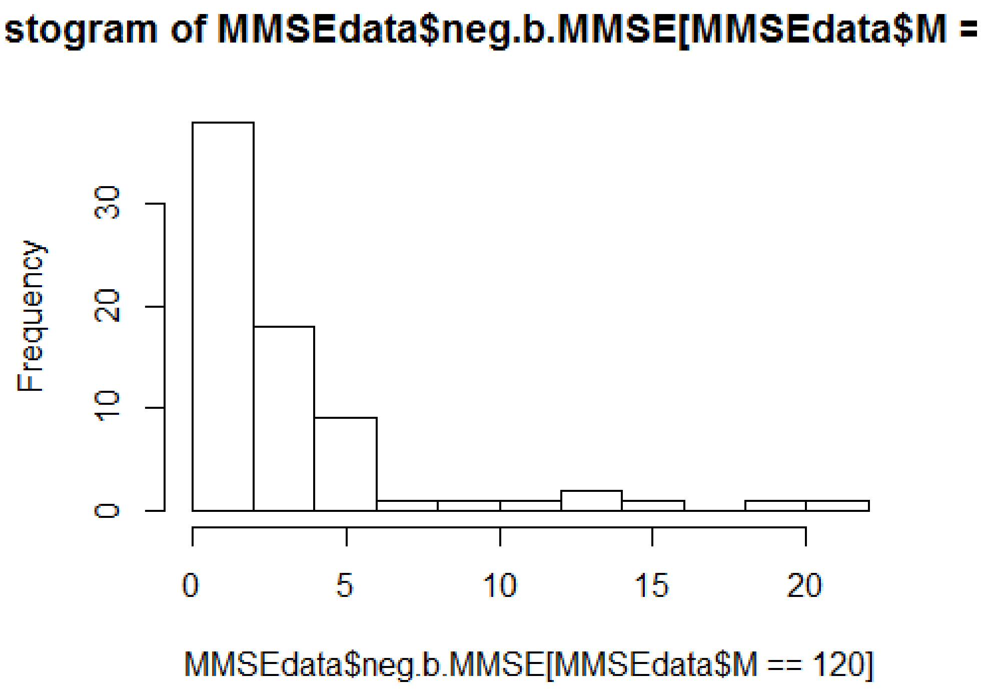


```
nbinom<-fitdistr(MMSEdata$neg.b.MMSE[MMSEdata$M==0], "negative binomial")
qqp(MMSEdata$neg.b.MMSE[MMSEdata$M==0], "nbinom", size=nbinom$estimate[[1]],
mu=nbinom$estimate[[2]], main="Negative binomial model Month=0")
```

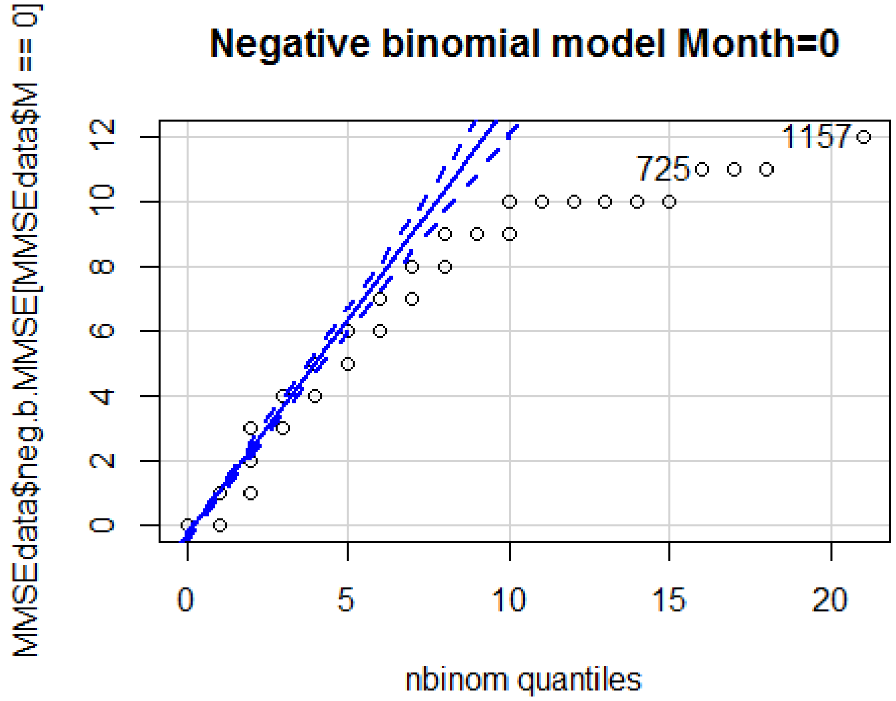


```
[1] 1157  725
```

```
nbinom<-fitdistr(MMSEdata$neg.b.MMSE[MMSEdata$M==12], "negative binomial")
qqp(MMSEdata$neg.b.MMSE[MMSEdata$M==12], "nbinom", size=nbinom$estimate[[1]],
mu=nbinom$estimate[[2]], main="Negative binomial model Month=12")
```

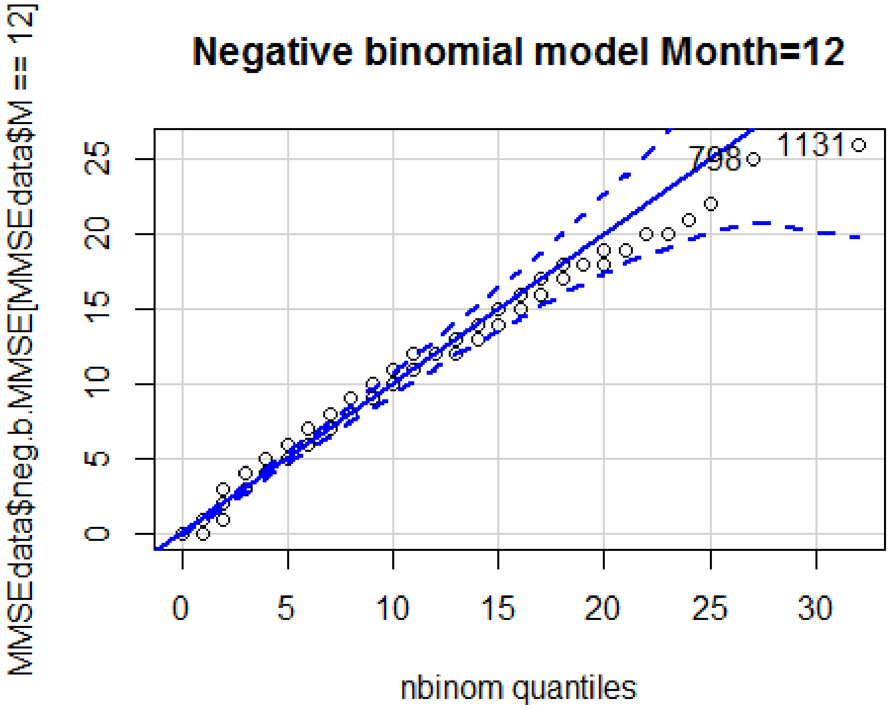


```
[1] 1131  798

nbinom<-fitdistr(MMSEdata$neg.b.MMSE[MMSEdata$M==24], "negative binomial")
qqp(MMSEdata$neg.b.MMSE[MMSEdata$M==24], "nbinom", size=nbinom$estimate[[1]],
mu=nbinom$estimate[[2]], main="Negative binomial model Month=24")
```

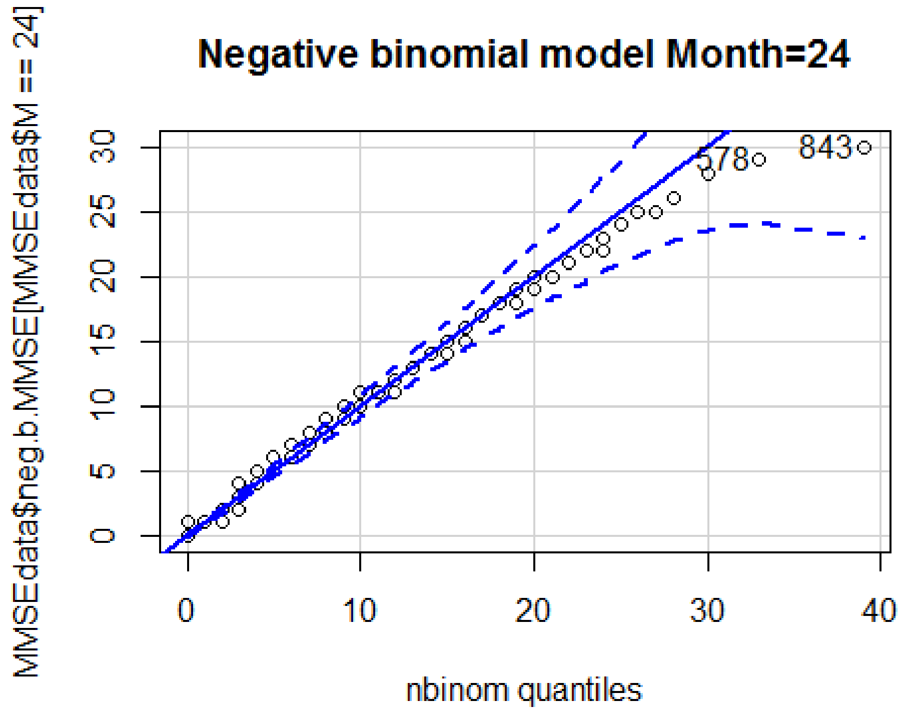


```
[1] 843 578

nbinom<-fitdistr(MMSEdata$neg.b.MMSE[MMSEdata$M==48], "negative binomial")
qqp(MMSEdata$neg.b.MMSE[MMSEdata$M==48], "nbinom", size=nbinom$estimate[[1]],
mu=nbinom$estimate[[2]], main="Negative binomial model Month=48")
```

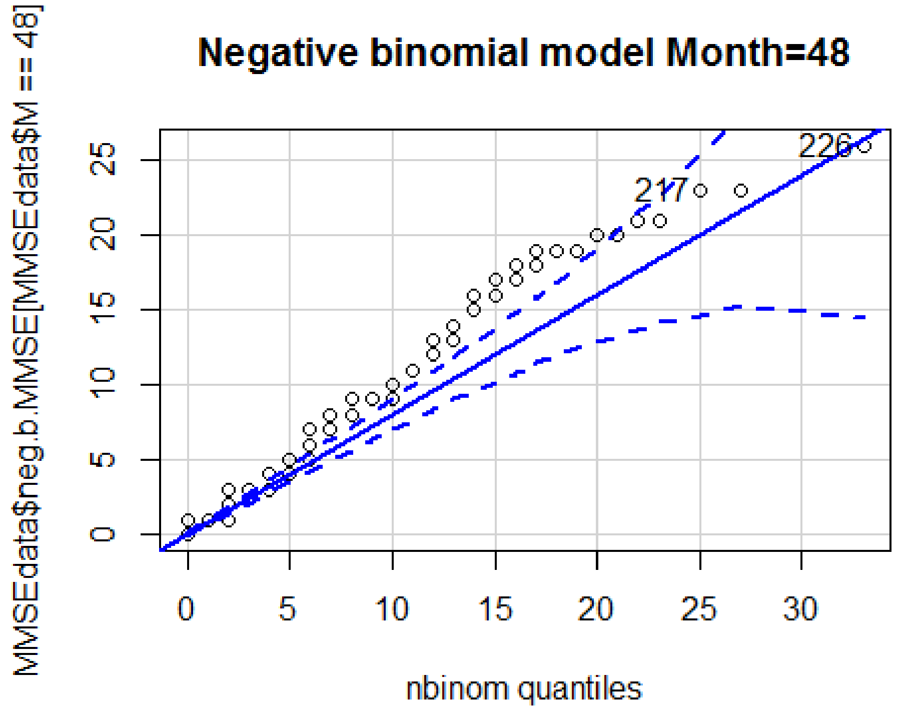


```
[1] 226 217
```

```
nbinom<-fitdistr(MMSEdata$neg.b.MMSE[MMSEdata$M==72], "negative binomial")
qqp(MMSEdata$neg.b.MMSE[MMSEdata$M==72], "nbinom", size=nbinom$estimate[[1]],
mu=nbinom$estimate[[2]], main="Negative binomial model Month=72")
```

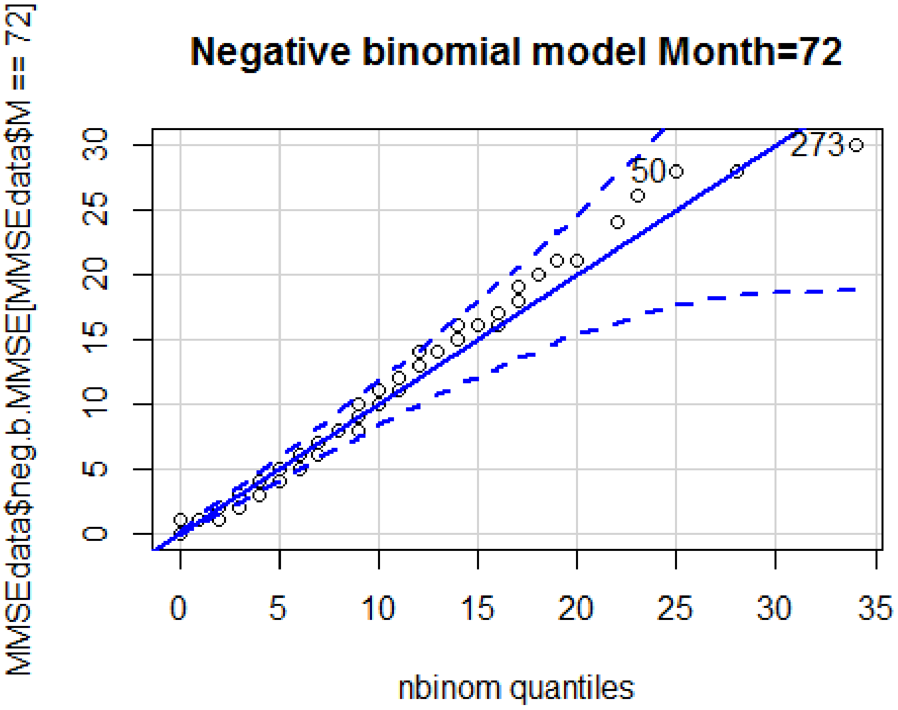


```
[1] 273  50

nbinom<-fitdistr(MMSEdata$neg.b.MMSE[MMSEdata$M==120], "negative binomial")
qqp(MMSEdata$neg.b.MMSE[MMSEdata$M==120], "nbinom", size=nbinom$estimate[[1]],
mu=nbinom$estimate[[2]], main="Negative binomial model Month=120")
```

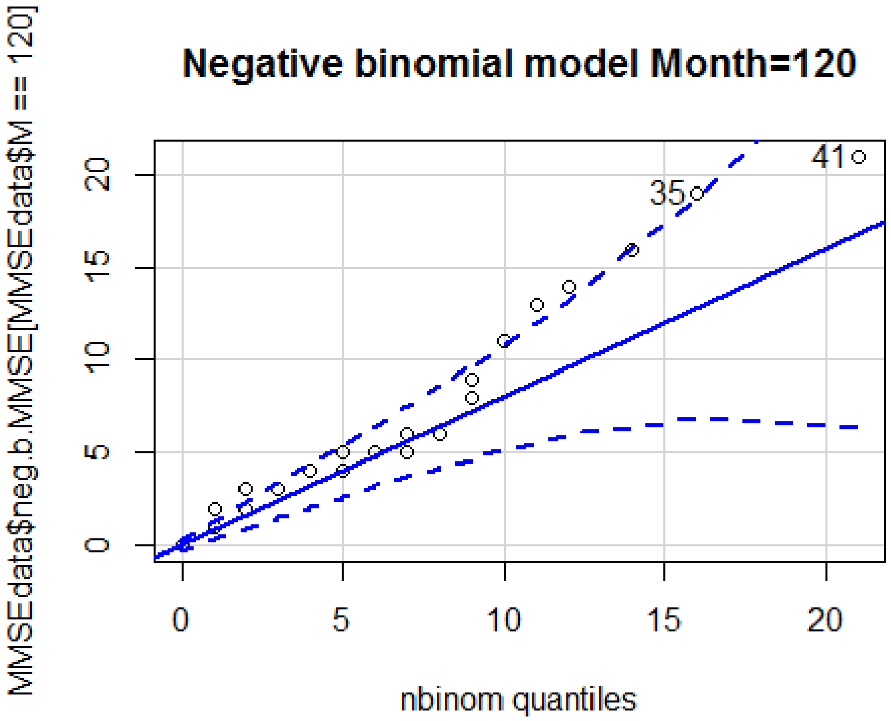


```
[1] 41 35

poisson <- fitdistr(MMSEdata$neg.b.MMSE[MMSEdata$M==0]+1, "Poisson")
qqp(MMSEdata$neg.b.MMSE, "pois", lambda=poisson$estimate, main="Poisson model
Month=0")
```

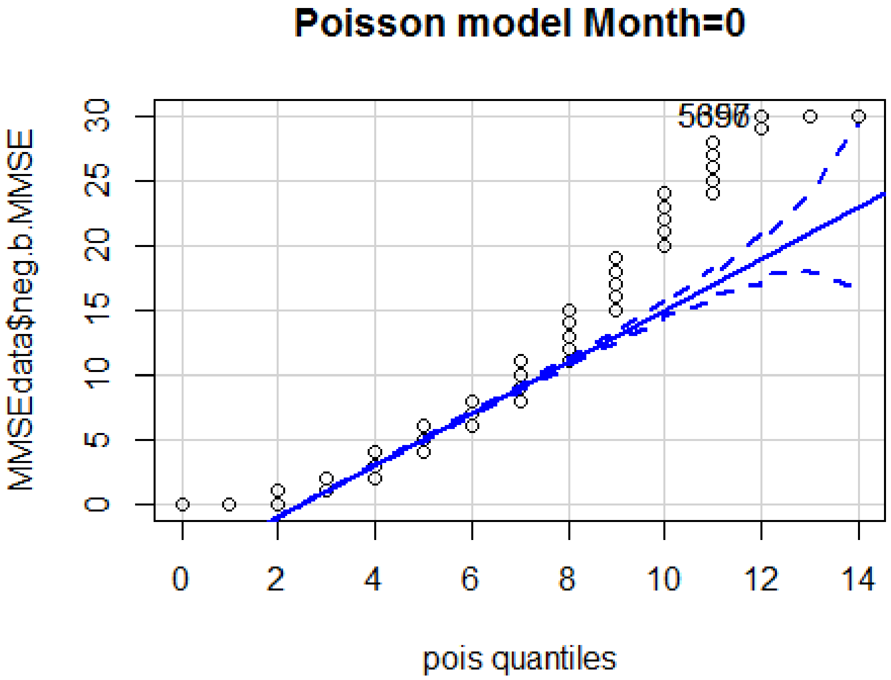


```
[1] 5357 5696
```

```
poisson <- fitdistr(MMSEdata$neg.b.MMSE[MMSEdata$M==12], "Poisson")
qqp(MMSEdata$neg.b.MMSE, "pois", lambda=poisson$estimate, main="Poisson model
Month=12")
```

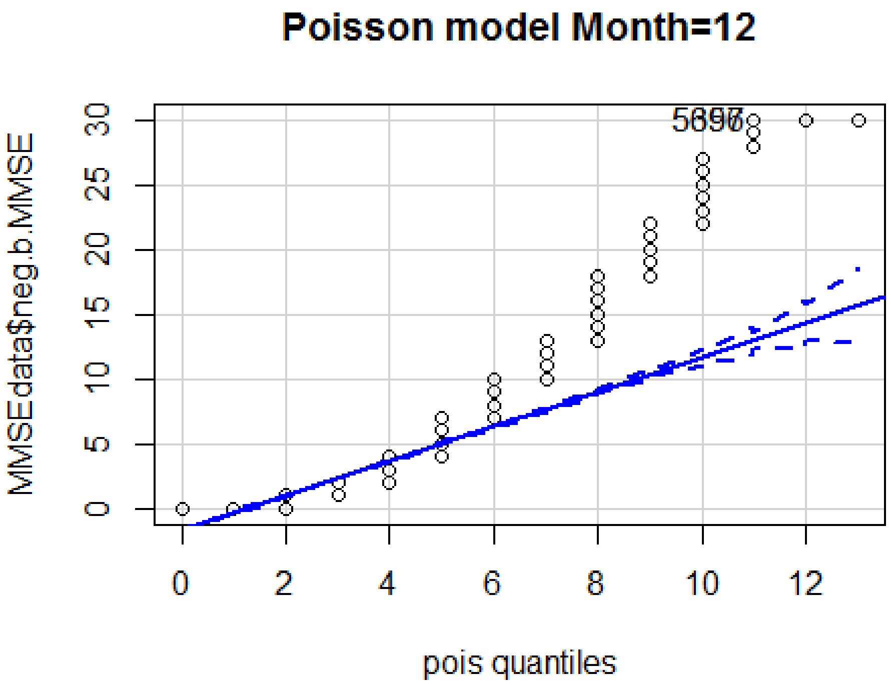


```
[1] 5357 5696

poisson <- fitdistr(MMSEdata$neg.b.MMSE[MMSEdata$M==24], "Poisson")
qqp(MMSEdata$neg.b.MMSE, "pois", lambda=poisson$estimate, main="Poisson model
Month=24")
```

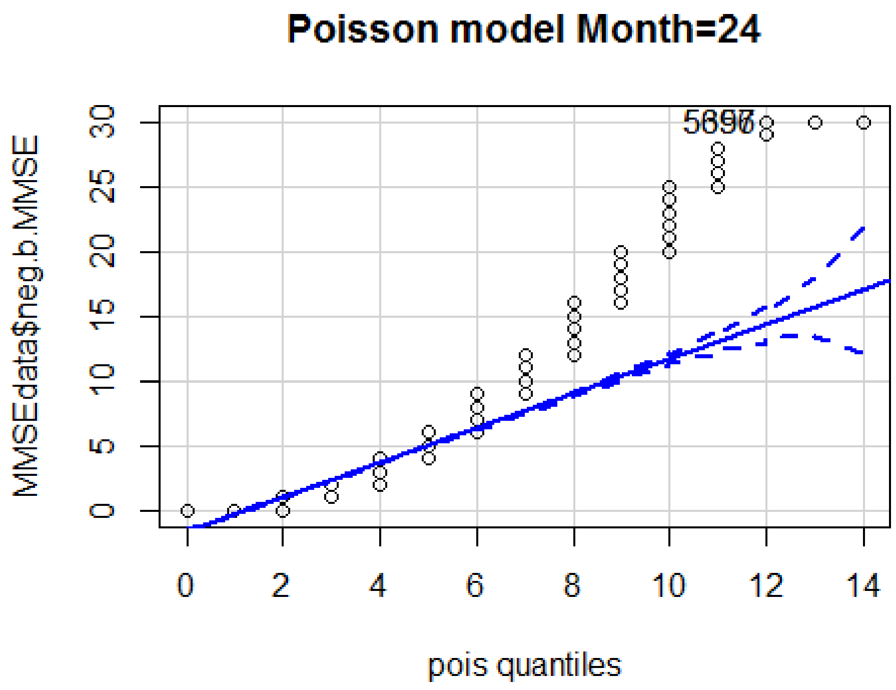


```
[1] 5357 5696

poisson <- fitdistr(MMSEdata$neg.b.MMSE[MMSEdata$M==48], "Poisson")
qqp(MMSEdata$neg.b.MMSE, "pois", lambda=poisson$estimate, main="Poisson model
Month=48")
```

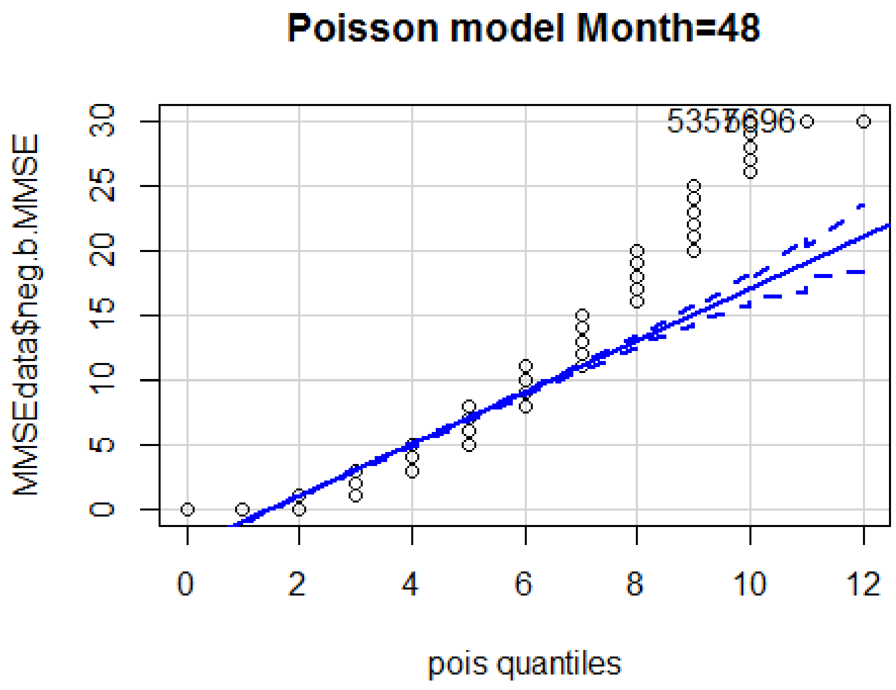


```
[1] 5357 5696
```

```
poisson <- fitdistr(MMSEdata$neg.b.MMSE[MMSEdata$M==72], "Poisson")
qqp(MMSEdata$neg.b.MMSE, "pois", lambda=poisson$estimate, main="Poisson model
Month=72")
```

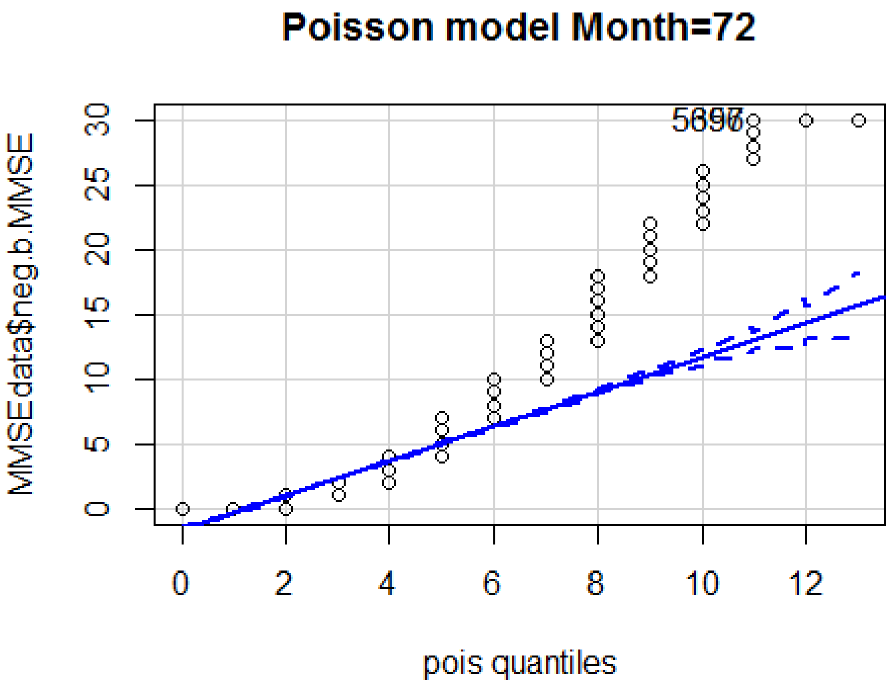


```
[1] 5357 5696

poisson <- fitdistr(MMSEdata$neg.b.MMSE[MMSEdata$M==120], "Poisson")
qqp(MMSEdata$neg.b.MMSE, "pois", lambda=poisson$estimate, main="Poisson model
Month=120")
```

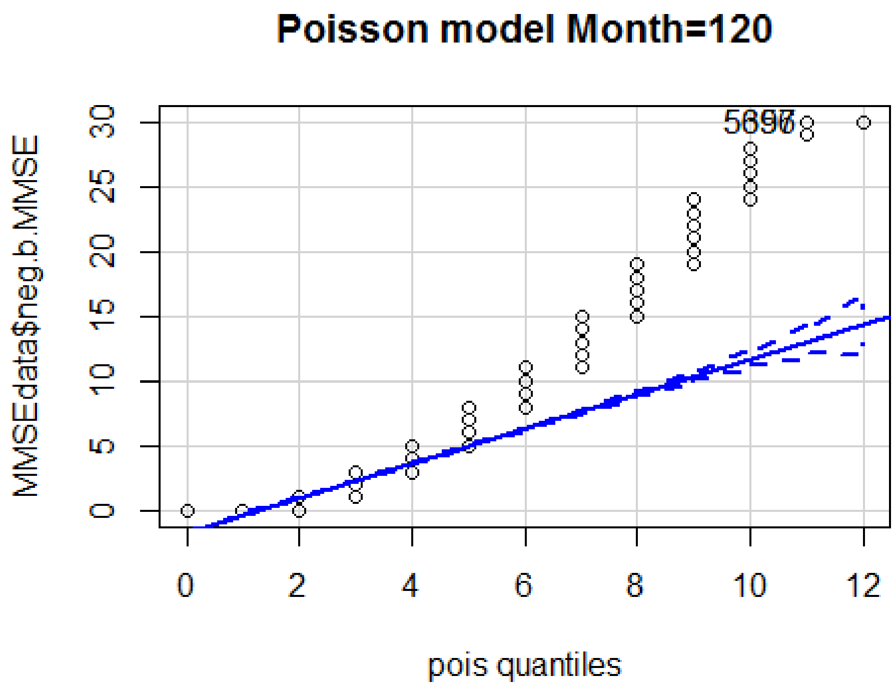


```
[1] 5357 5696

mean(MMSEdata$neg.b.MMSE[MMSEdata$M==0])

[1] 2.943175

var(MMSEdata$neg.b.MMSE[MMSEdata$M==0])

[1] 7.247696

mean(MMSEdata$neg.b.MMSE[MMSEdata$M==12])

[1] 3.579349

var(MMSEdata$neg.b.MMSE[MMSEdata$M==12])

[1] 15.47411

mean(MMSEdata$neg.b.MMSE[MMSEdata$M==24])

[1] 3.897266

var(MMSEdata$neg.b.MMSE[MMSEdata$M==24])

[1] 21.89491

mean(MMSEdata$neg.b.MMSE[MMSEdata$M==48])

[1] 3.273011

var(MMSEdata$neg.b.MMSE[MMSEdata$M==48])

[1] 18.91441

mean(MMSEdata$neg.b.MMSE[MMSEdata$M==72])
```

```
[1] 3.63662

var(MMSEdata$neg.b.MMSE[MMSEdata$M==72])

[1] 26.68962

mean(MMSEdata$neg.b.MMSE[MMSEdata$M==120])

[1] 3.369863

var(MMSEdata$neg.b.MMSE[MMSEdata$M==120])

[1] 19.09741

gamma <- fitdistr((MMSEdata$neg.b.MMSE[MMSEdata$M==0]+1), "gamma")
qqp(MMSEdata$neg.b.MMSE[MMSEdata$M==0]+1, "gamma", shape = gamma$estimate[[1]],
rate = gamma$estimate[[2]], main="Gamma model Month=0")
```

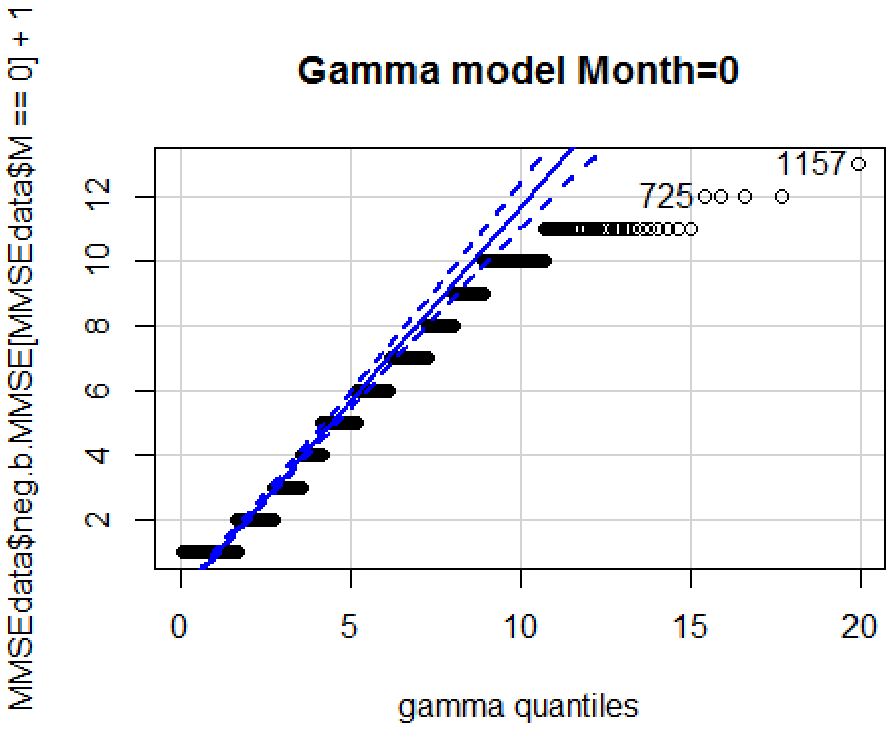


```
[1] 1157  725

gamma <- fitdistr((MMSEdata$neg.b.MMSE[MMSEdata$M==12]+1), "gamma")
qqp(MMSEdata$neg.b.MMSE[MMSEdata$M==12]+1, "gamma", shape = gamma$estimate[[1]],
rate = gamma$estimate[[2]], main="Gamma model Month=12")
```

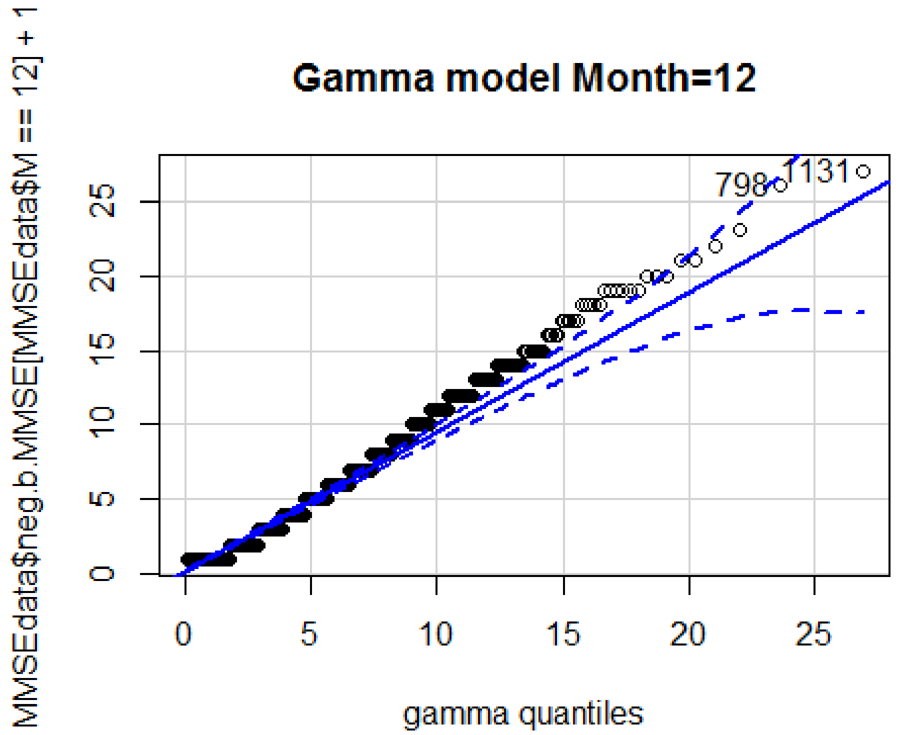


```
[1] 1131  798

gamma <- fitdistr((MMSEdata$neg.b.MMSE[MMSEdata$M==24]+1), "gamma")
qqp(MMSEdata$neg.b.MMSE[MMSEdata$M==24]+1, "gamma", shape = gamma$estimate[[1]], rate = gamma$estimate[[2]], main="Gamma model Month=24")
```

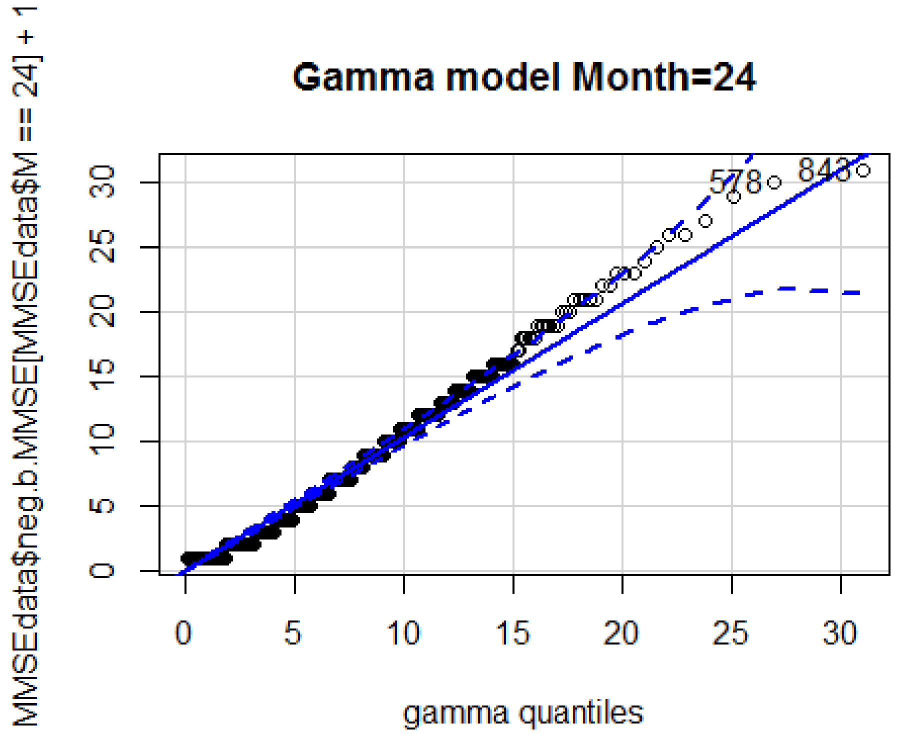


```
[1] 843 578
```

```
gamma <- fitdistr((MMSEdata$neg.b.MMSE[MMSEdata$M==48]+1), "gamma")
qqp(MMSEdata$neg.b.MMSE[MMSEdata$M==48]+1, "gamma", shape = gamma$estimate[[1]],
rate = gamma$estimate[[2]], main="Gamma model Month=48")
```

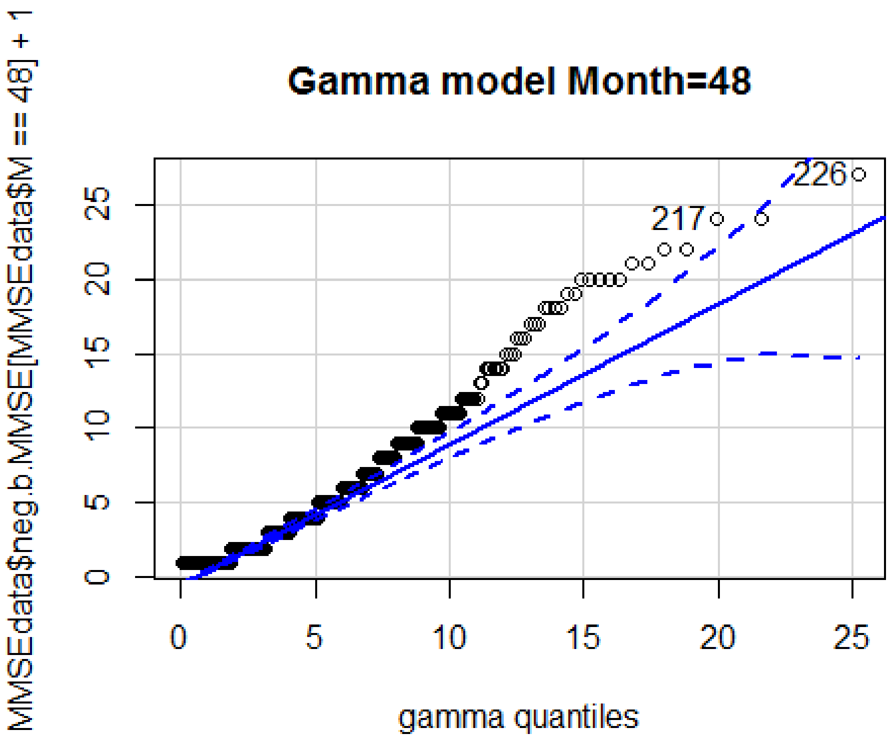


```
[1] 226 217

gamma <- fitdistr((MMSEdata$neg.b.MMSE[MMSEdata$M==72]+1), "gamma")
qqp(MMSEdata$neg.b.MMSE[MMSEdata$M==72]+1, "gamma", shape = gamma$estimate[[1]],
rate = gamma$estimate[[2]], main="Gamma model Month=72")
```

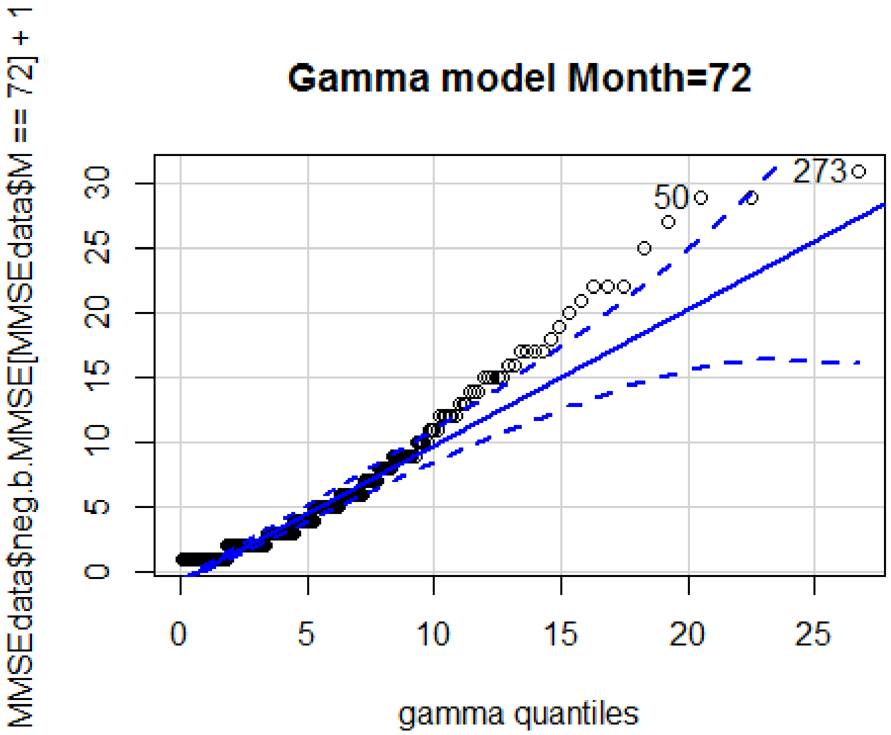


```
[1] 273  50

gamma <- fitdistr((MMSEdata$neg.b.MMSE[MMSEdata$M==120]+1), "gamma")
qqp(MMSEdata$neg.b.MMSE[MMSEdata$M==120]+1, "gamma", shape =
gamma$estimate[[1]], rate = gamma$estimate[[2]], main="Gamma model Month=120")
```

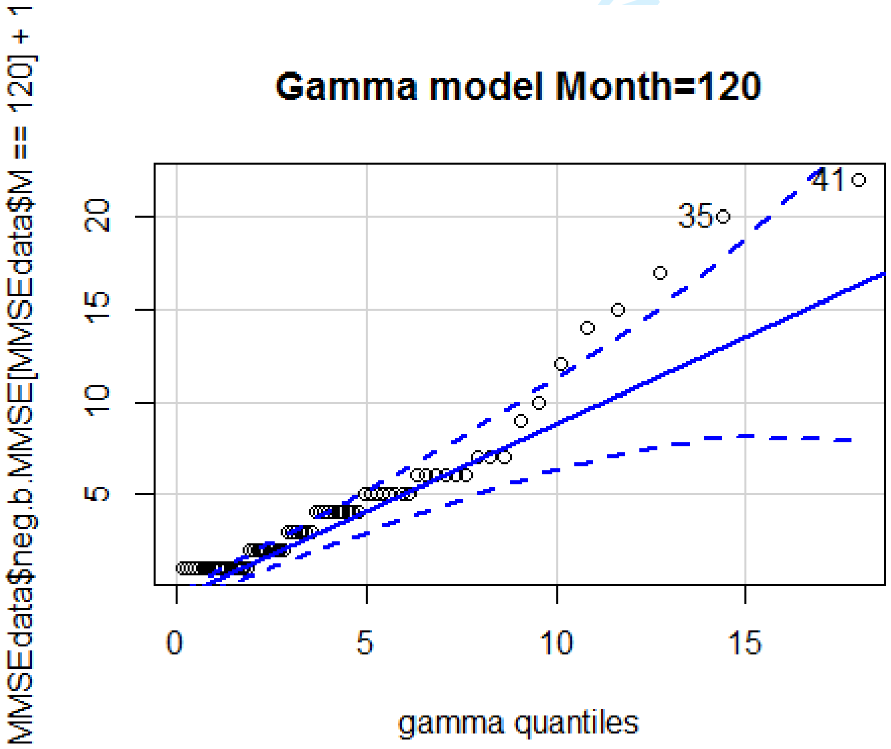


```
[1] 41 35
```

```
par(mar = c(3,3,3,3))
hist(MMSEdata$proportion)
```

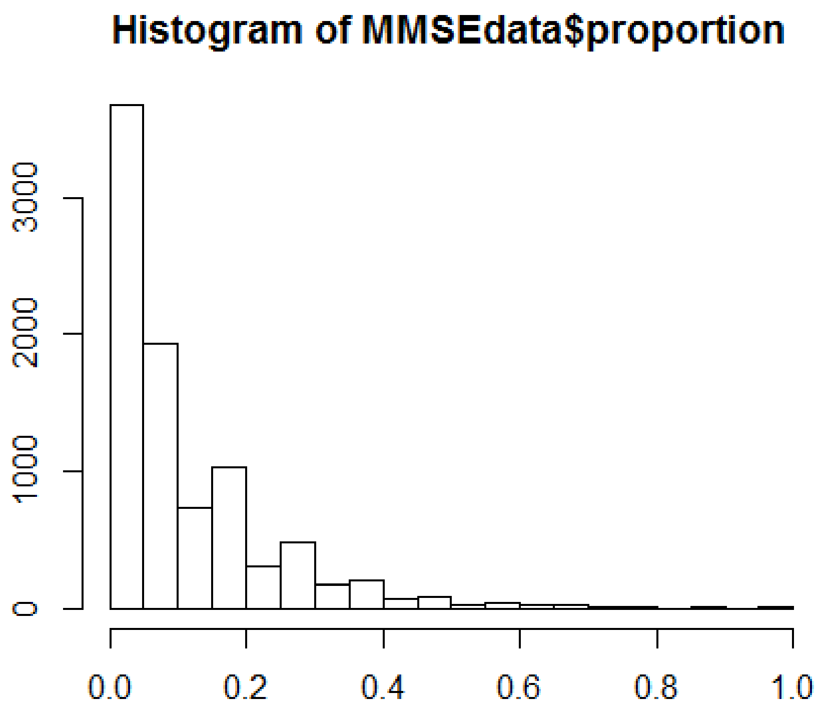


```
data<-MMSEdata$proportion[MMSEdata$M==0]
params<-fitdistr(data, "logistic")
qqp(data, dist="logis",  params$estimate[[1]]);title(main="Logistic model
Month=0", line=2)
```

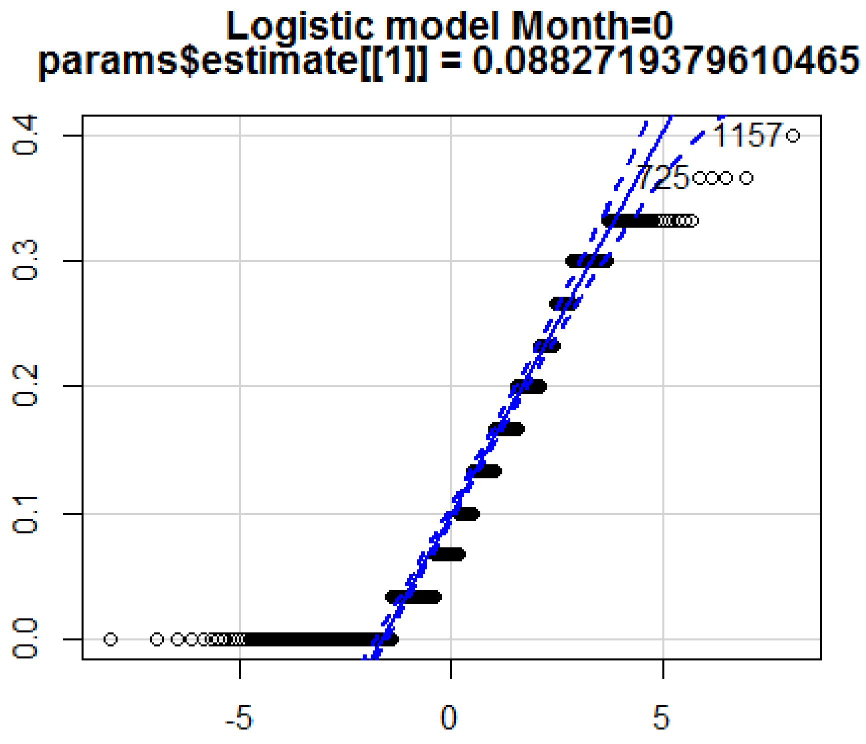

```
data<-MMSEdata$proportion[MMSEdata$M==12]
params<-fitdistr(data, "logistic")
qqp(data, dist="logis",  params$estimate[[1]]);title(main="Logistic model
Month=12", line=2)
```

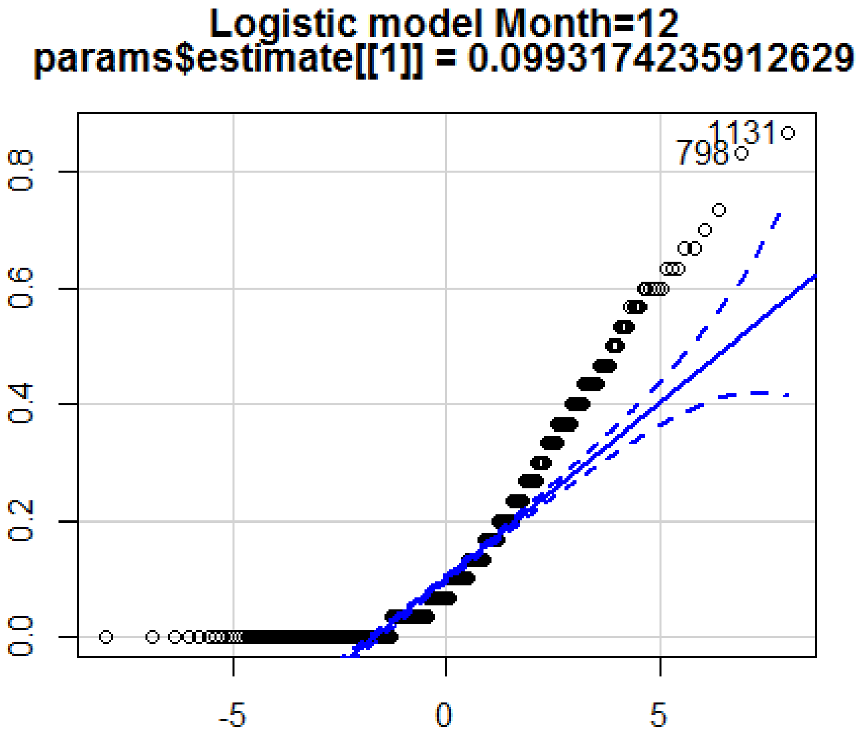


```
data<-MMSEdata$proportion[MMSEdata$M==24]
params<-fitdistr(data, "logistic")
qqp(data, dist="logis",  params$estimate[[1]]);title(main="Logistic model
Month=24", line=2)
```

Only

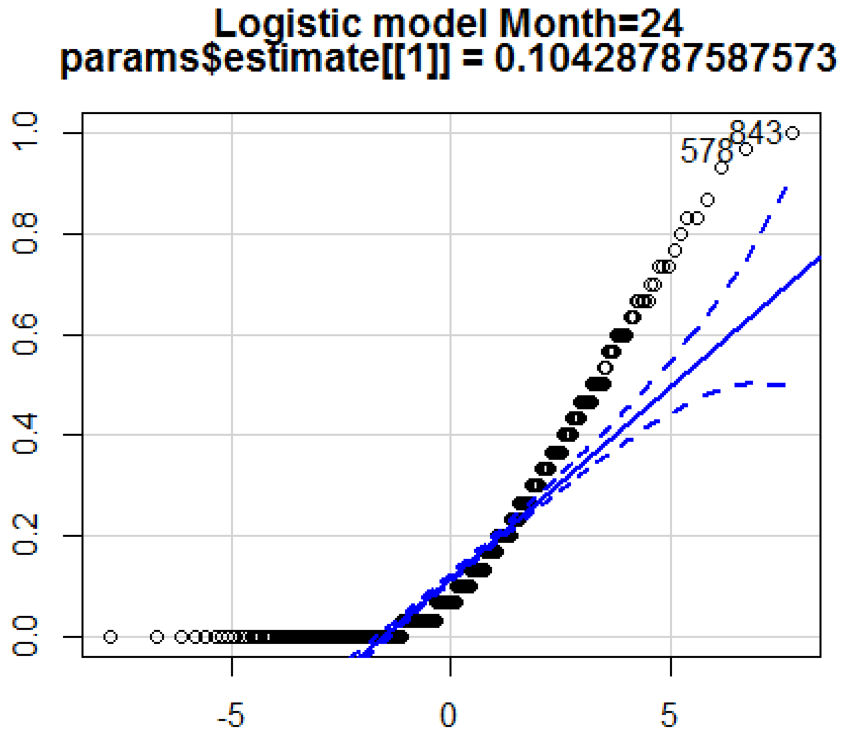


```
data<-MMSEdata$proportion[MMSEdata$M==48]
params<-fitdistr(data, "logistic")
qqp(data, dist="logis",  params$estimate[[1]]);title(main="Logistic model
Month=48", line=2)
```

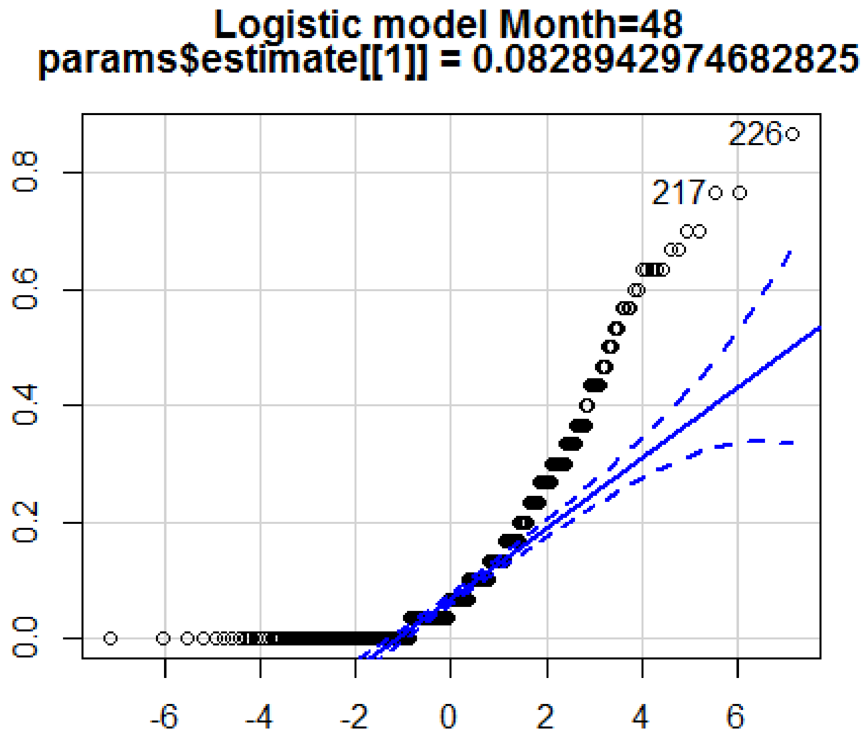


```
data<-MMSEdata$proportion[MMSEdata$M==72]
params<-fitdistr(data, "logistic")
```

```
qqp(data, dist="logis",  params$estimate[[1]]);title(main="Logistic model
Month=72", line=2)
```

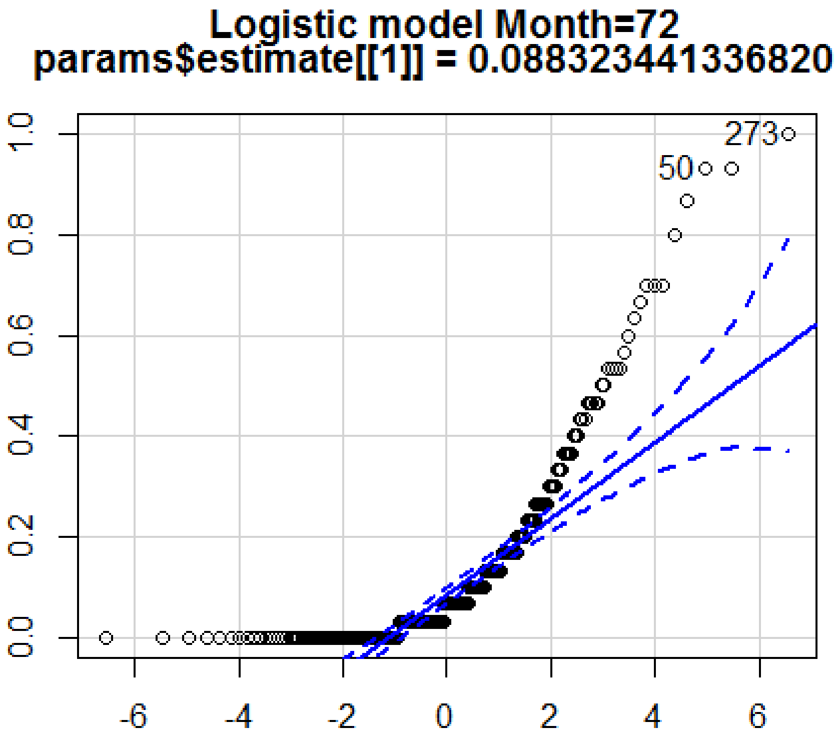


```
data<-MMSEdata$proportion[MMSEdata$M==120]
params<-fitdistr(data, "logistic")
qqp(data, dist="logis",params$estimate[[1]]);title(main="Logistic model
Month=120", line=2)
```

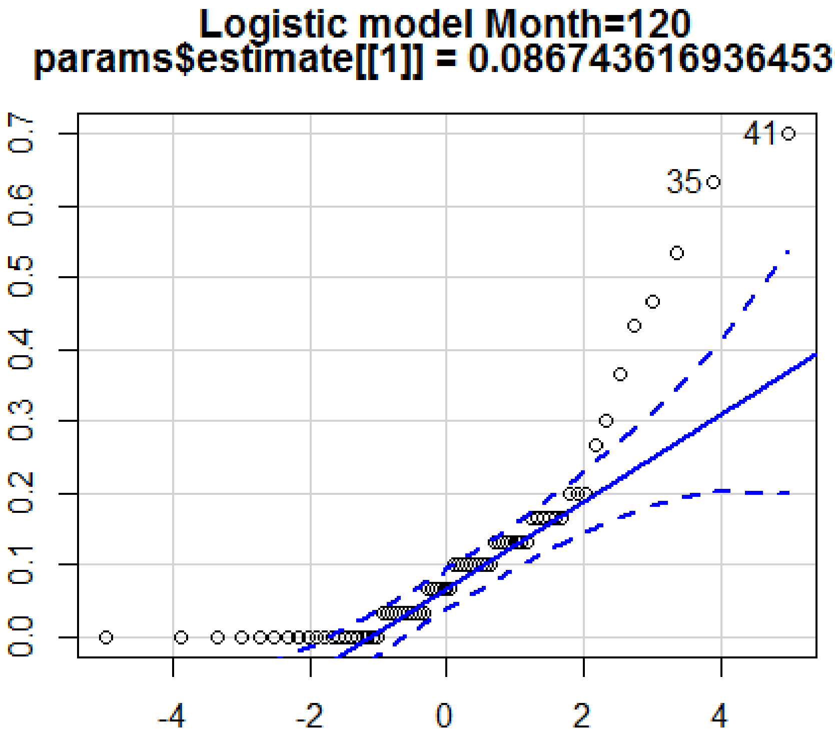

```
data<-MMSEdata$neg.b.MMSE[MMSEdata$M==0]
params<-fitdistr(data, "exponential")
qqp(data, "exp",rate = params$estimate, main="Exponential model Month=0")
```

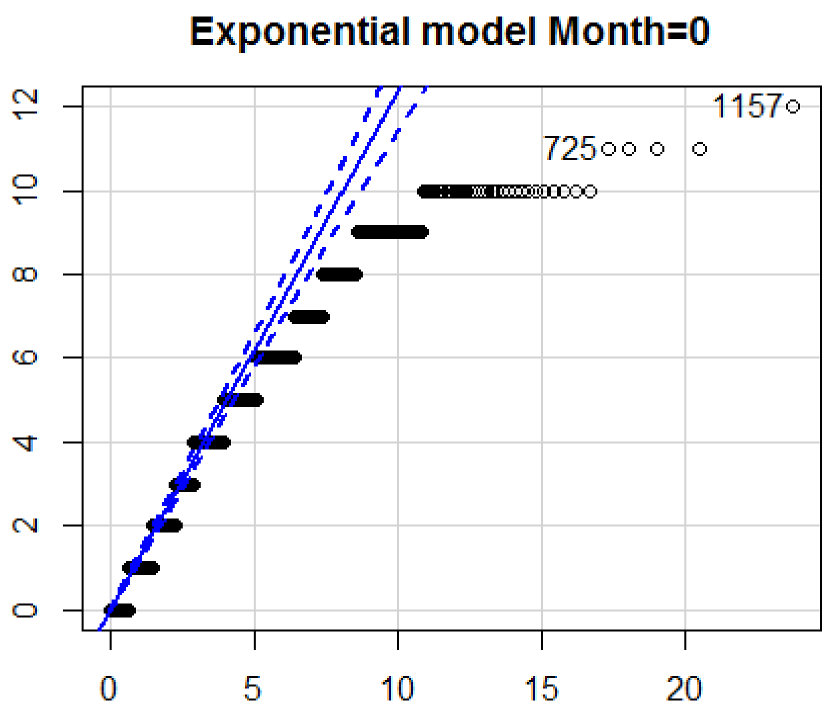


```
[1] 1157  725

data<-MMSEdata$neg.b.MMSE[MMSEdata$M==12]
params<-fitdistr(data, "exponential")
qqp(data, "exp",rate = params$estimate, main="Exponential model Month=12")
```

For Peer Review Only

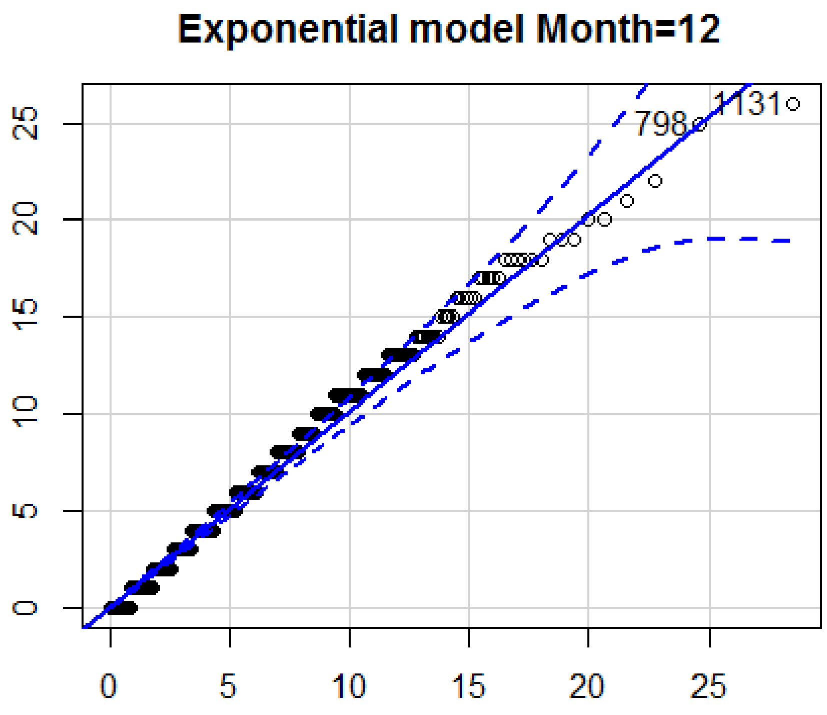


```
[1] 1131  798

data<-MMSEdata$neg.b.MMSE[MMSEdata$M==24]
params<-fitdistr(data, "exponential")
qqp(data, "exp",rate = params$estimate, main="Exponential model Month=24")
```

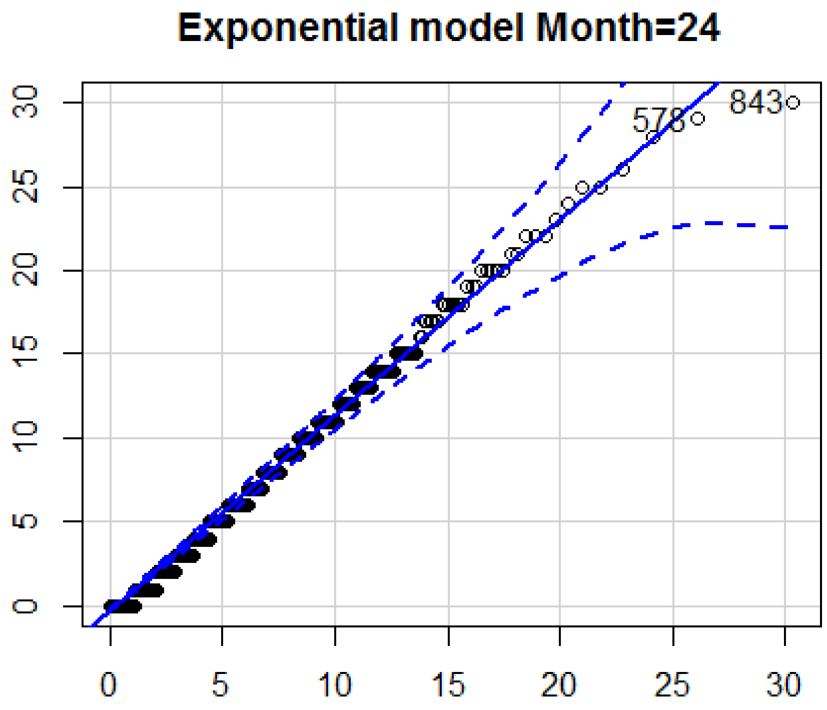


```
[1] 843 578
```

```
data<-MMSEdata$neg.b.MMSE[MMSEdata$M==48]
params<-fitdistr(data, "exponential")
qqp(data, "exp",rate = params$estimate, main="Exponential model Month=48")
```

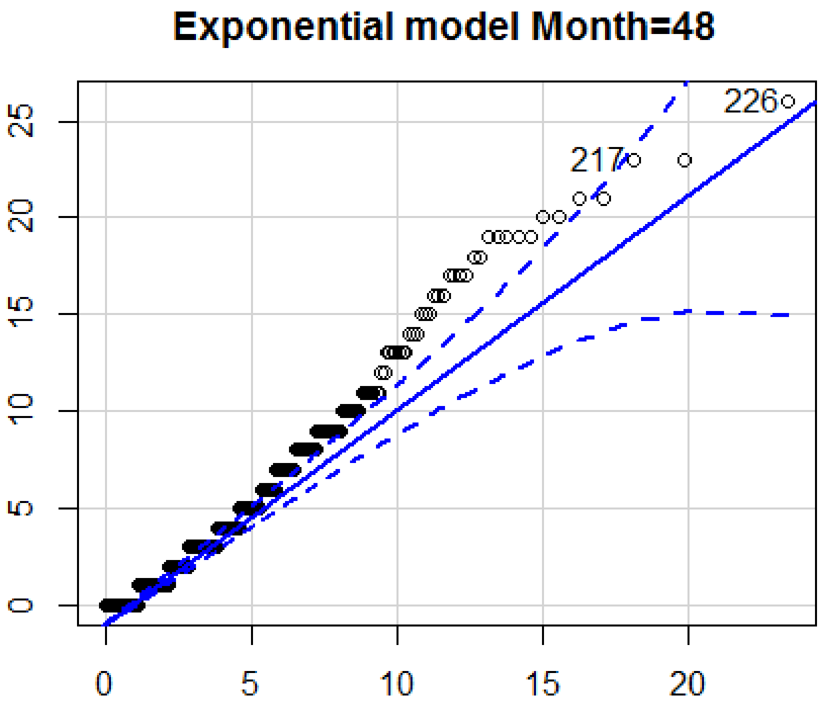


```
[1] 226 217

data<-MMSEdata$neg.b.MMSE[MMSEdata$M==72]
params<-fitdistr(data, "exponential")
qqp(data, "exp",rate = params$estimate, main="Exponential model Month=72")
```

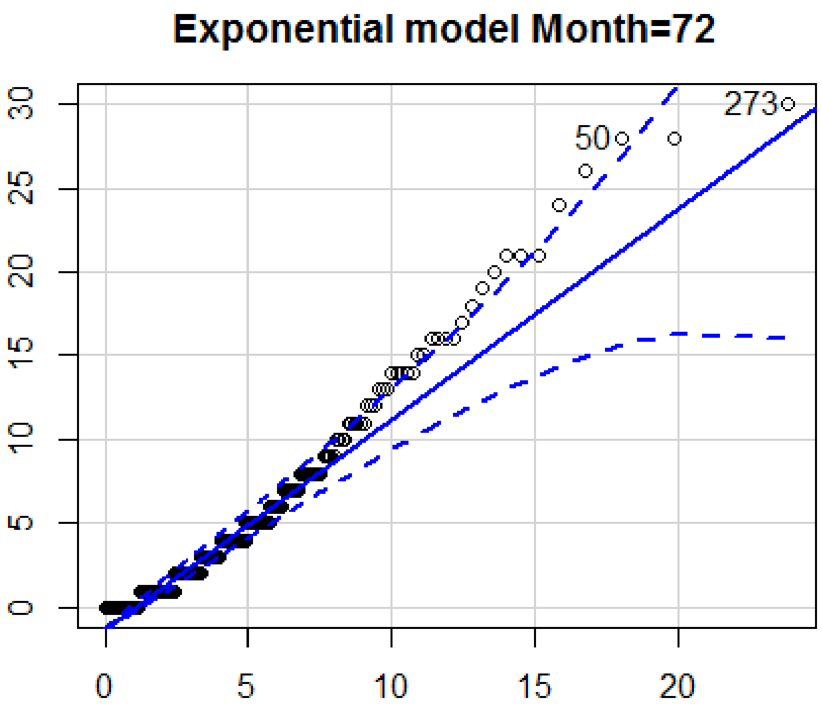


```
[1] 273  50

data<-MMSEdata$neg.b.MMSE[MMSEdata$M==120]
params<-fitdistr(data, "exponential")
qqp(data, "exp",rate = params$estimate, main="Exponential model Month=120")
```

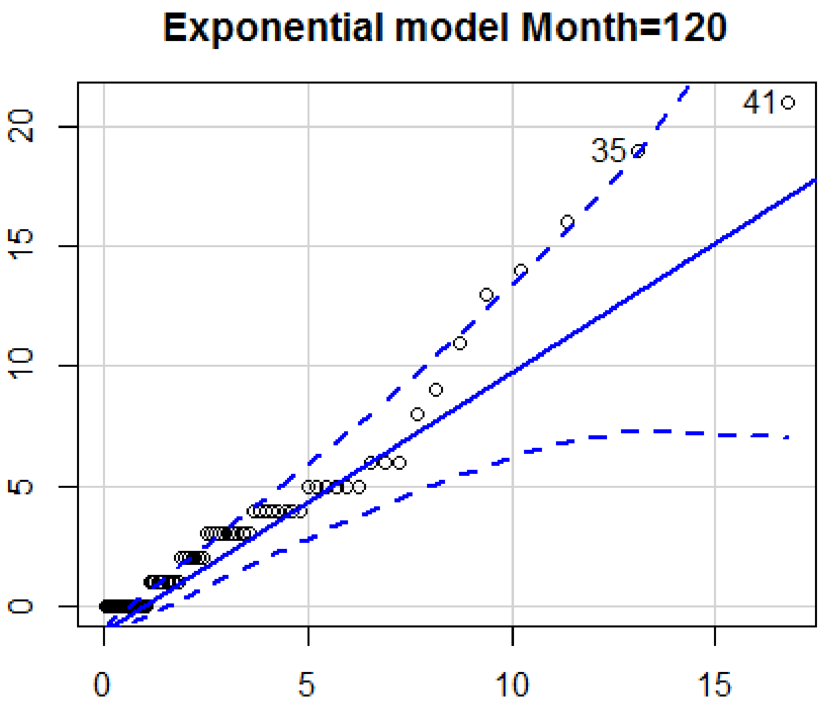


```
[1] 41 35
```

7.6. *Build base negative binomial model and comparing the different parameterization methods.*

There are two commonly used parameterization methods used in the negative binomial model. To evaluate which to continue with all biological relavent main effect explainitory variables were include in the models and initial model diagnostic were performed. Using the AIC and log-likelihood statistics we can see that the Nbinom1 parameterization method is prefered. This uses a varaince proportional to the mean method of parameterization.

```
neg.m.binom1 <- glmmadmb(neg.b.MMSE~AGE + APOE4 + M + Gender + edu.cat + diagn +
vasc+diab+ aspirin+ naprox+ diclo+ parac+ celex+ Ibu+diagn*M+(1|ID),
family="nbinom", data=MMSEdata)

neg.m.binom2 <- glmmadmb(neg.b.MMSE~AGE + APOE4 + M + Gender + edu.cat + diagn +
vasc+diab+ aspirin+ naprox+ diclo+ parac+ celex+ Ibu+diagn*M+(1|ID),
family="nbinom1", data=MMSEdata)

AIC(logLik(neg.m.binom1))

[1] 34108.8

AIC(logLik(neg.m.binom2))

[1] 34090.6

logLik(neg.m.binom1)

'log Lik.' -17029.4 (df=25)

logLik(neg.m.binom2)

'log Lik.' -17020.3 (df=25)
```

7.7. *Observing the distribution of the residuals in for the initial model.*

Residuals within each explanitory variables collectively and within each individual show no trends and are homoskedastic centred around zero. Therefore this model is accurate and will be used to investiage the effects of the input variables (including pain medications) going forward.

```
augDat <- data.frame(MMSEdata,resid=residuals(neg.m.binom2,type="pearson"),
fitted=fitted(neg.m.binom2))
ggplot(augDat,aes(x=Gender,y=resid,group=ID))+geom_boxplot()+coord_flip()
```

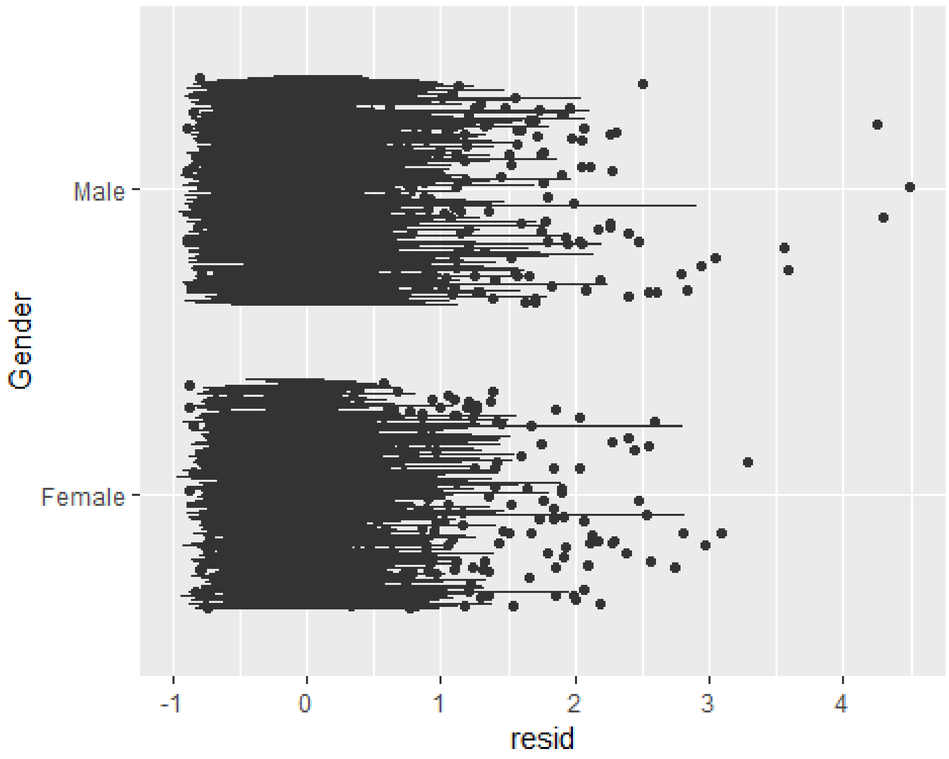


```
ggplot(augDat,aes(x=AGE,y=resid,group=ID))+geom_boxplot()+coord_flip()
```

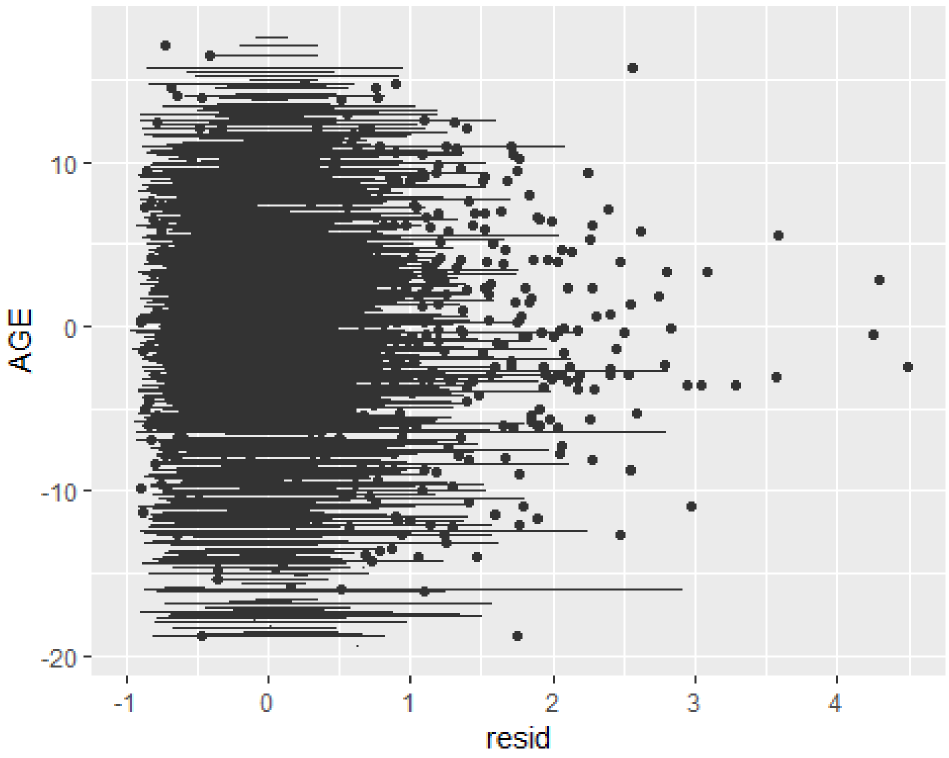


```
ggplot(augDat,aes(x=edu.cat,y=resid,group=ID))+geom_boxplot()+coord_flip()
```

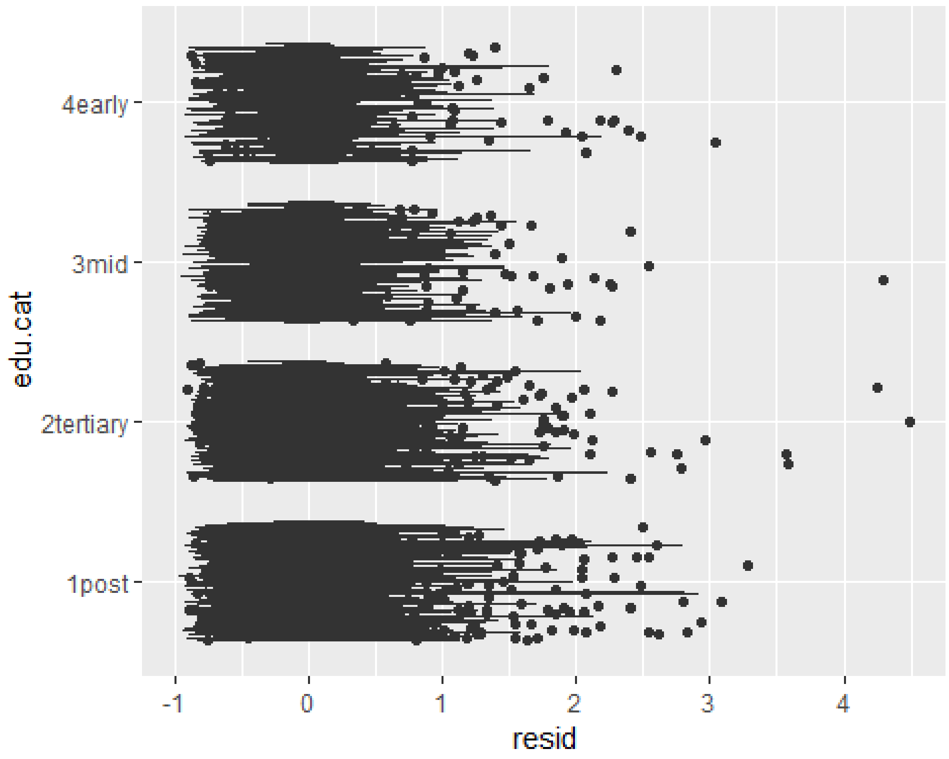


```
ggplot(augDat,aes(x=diagn,y=resid,group=ID))+geom_boxplot()+coord_flip()
```

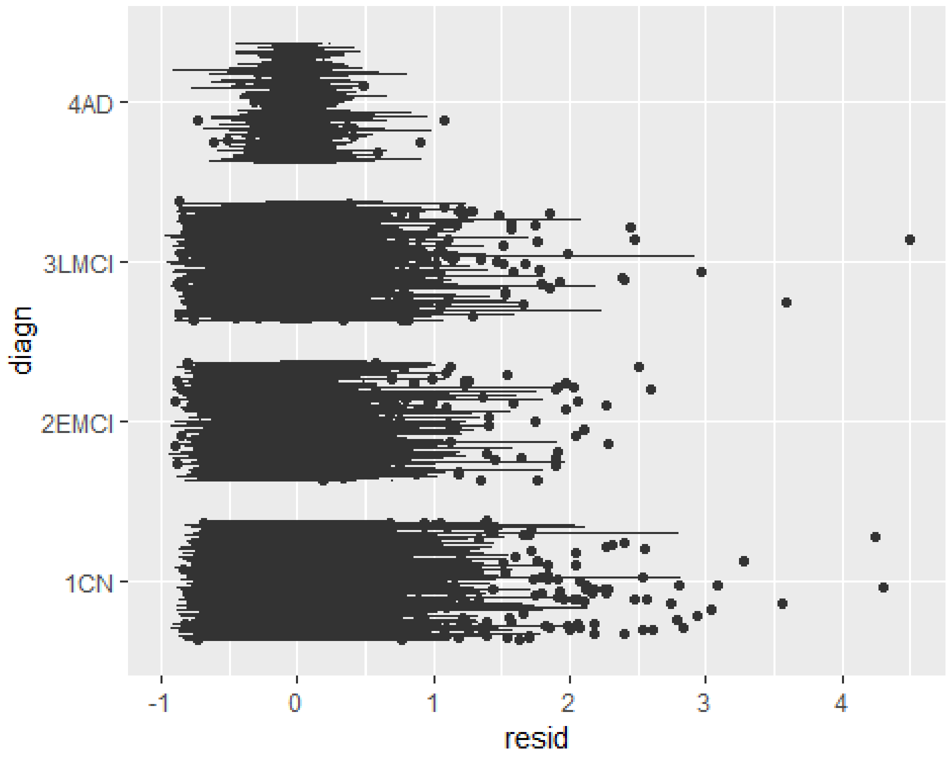


```
ggplot(augDat,aes(x=APOE4,y=resid,group=ID))+geom_boxplot()+coord_flip()
```

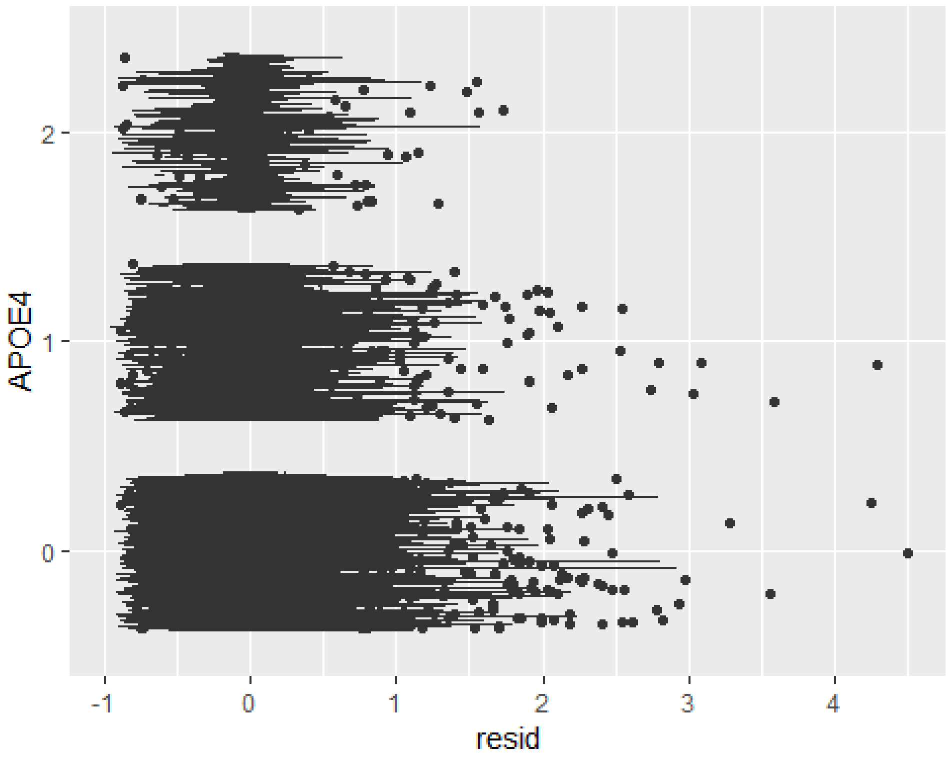


```
ggplot(augDat,aes(x=M,y=resid,group=ID))+geom_boxplot()+coord_flip()
```

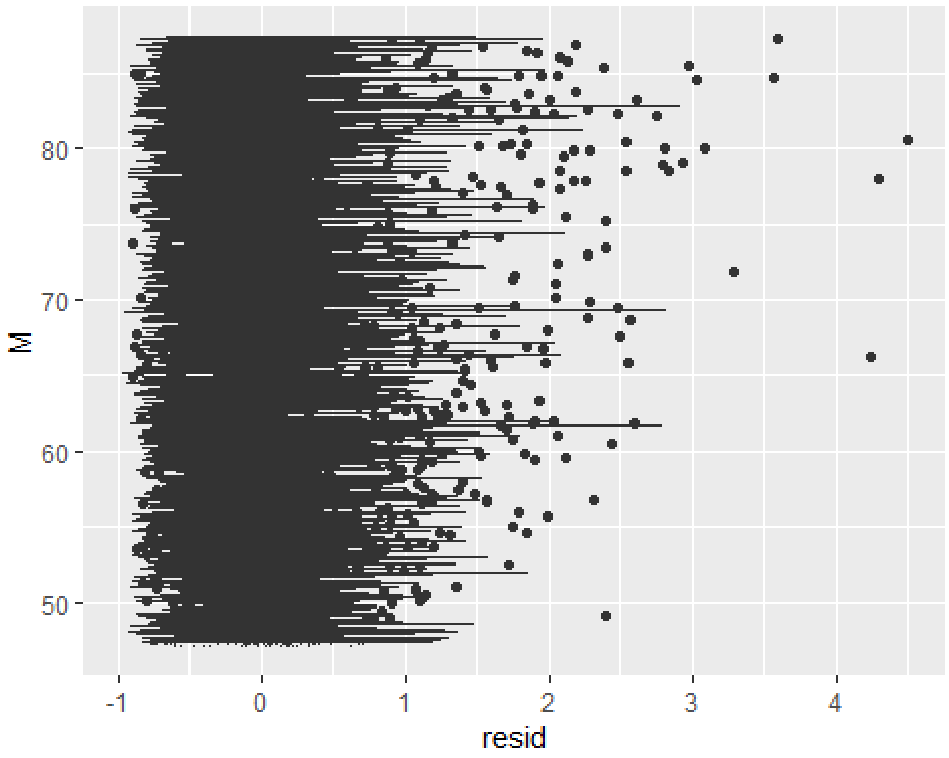


```
ggplot(augDat,aes(x=diclo,y=resid,group=ID))+geom_boxplot()+coord_flip()
```

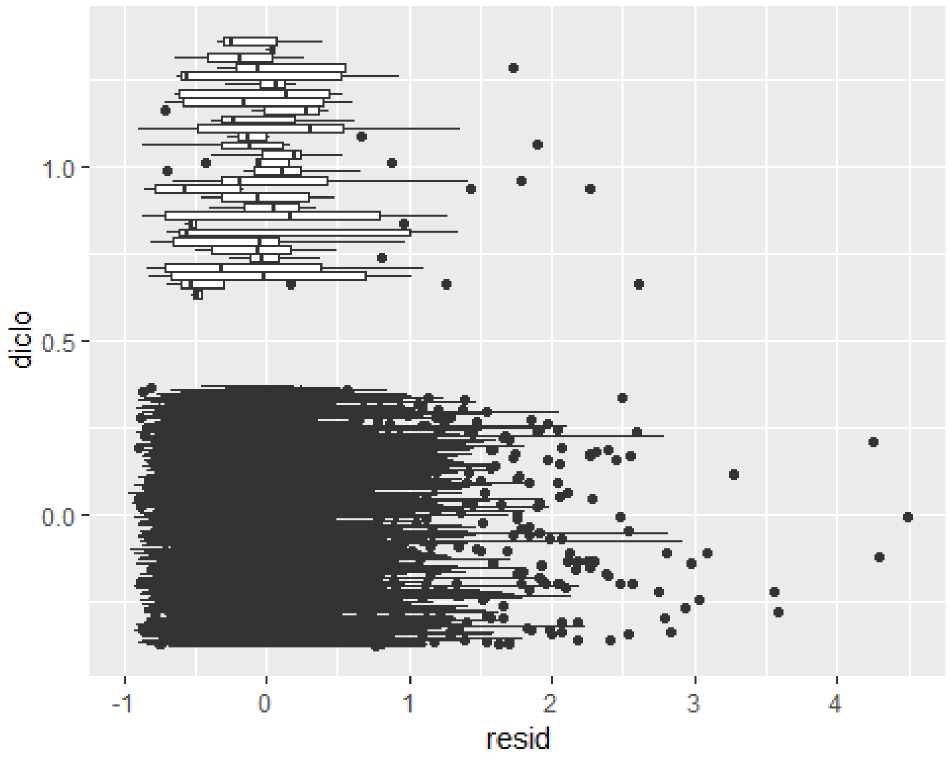


```
ggplot(augDat,aes(x=vasc,y=resid,group=ID))+geom_boxplot()+coord_flip()
```

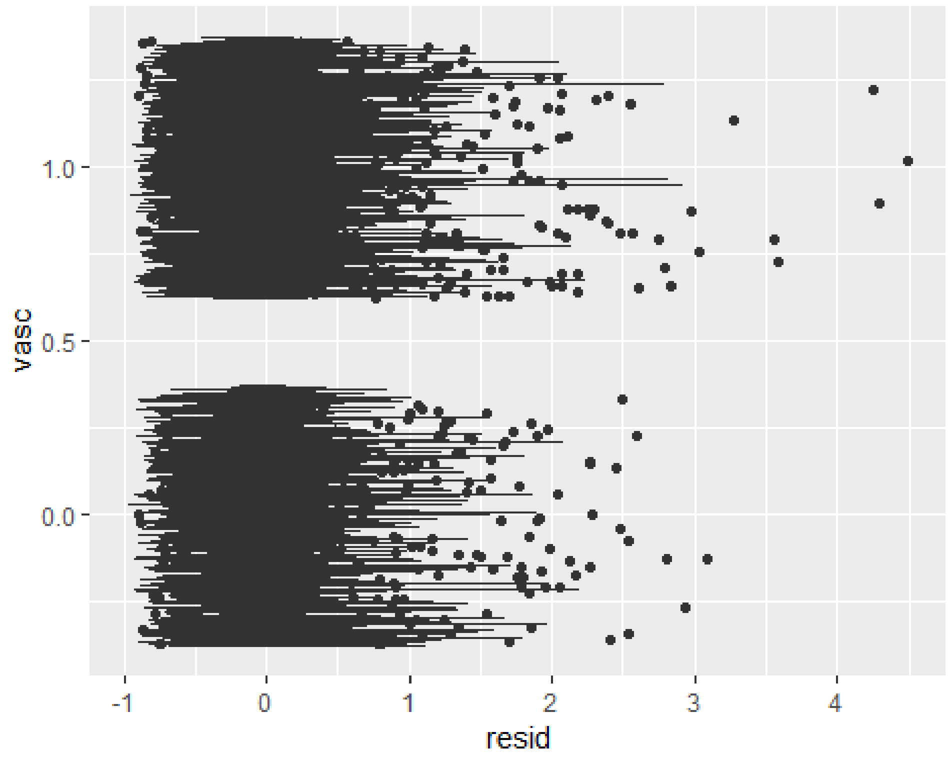


```
ggplot(augDat,aes(x=Ibu,y=resid,group=ID))+geom_boxplot()+coord_flip()
```

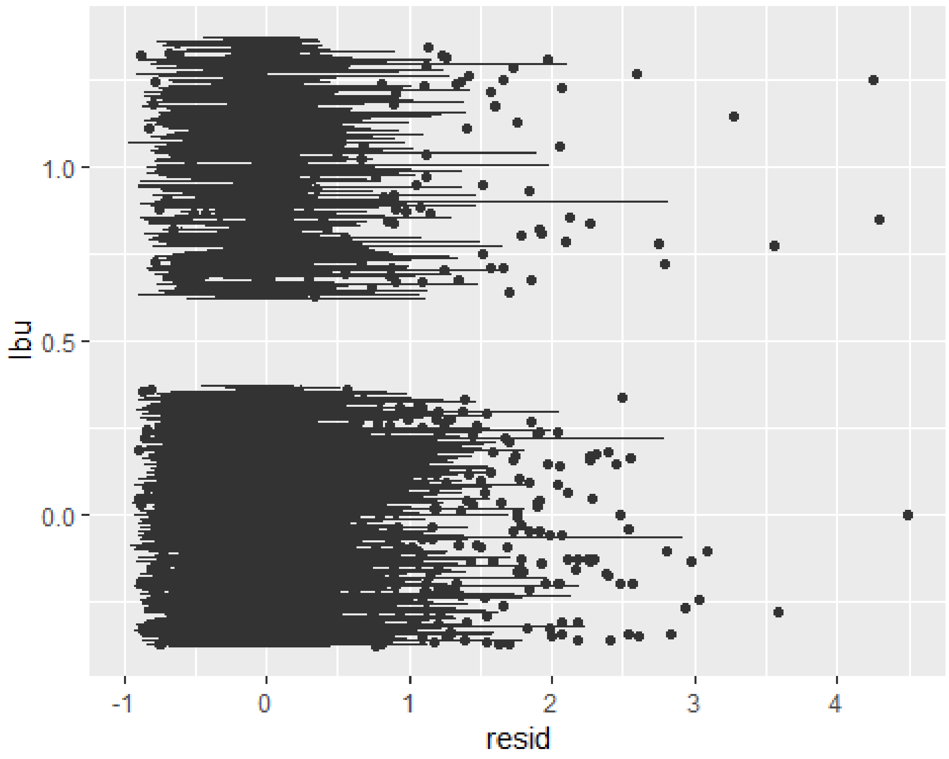


```
ggplot(augDat,aes(x=aspirin,y=resid,group=ID))+geom_boxplot()+coord_flip()
```

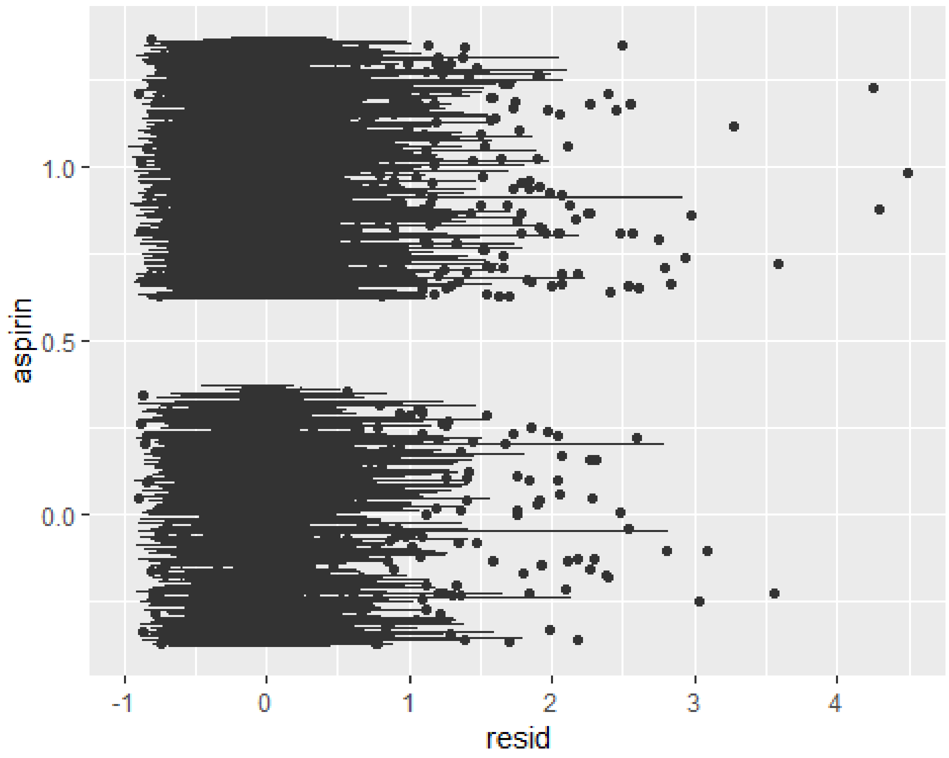


```
ggplot(augDat,aes(x=diab,y=resid,group=ID))+geom_boxplot()+coord_flip()
```

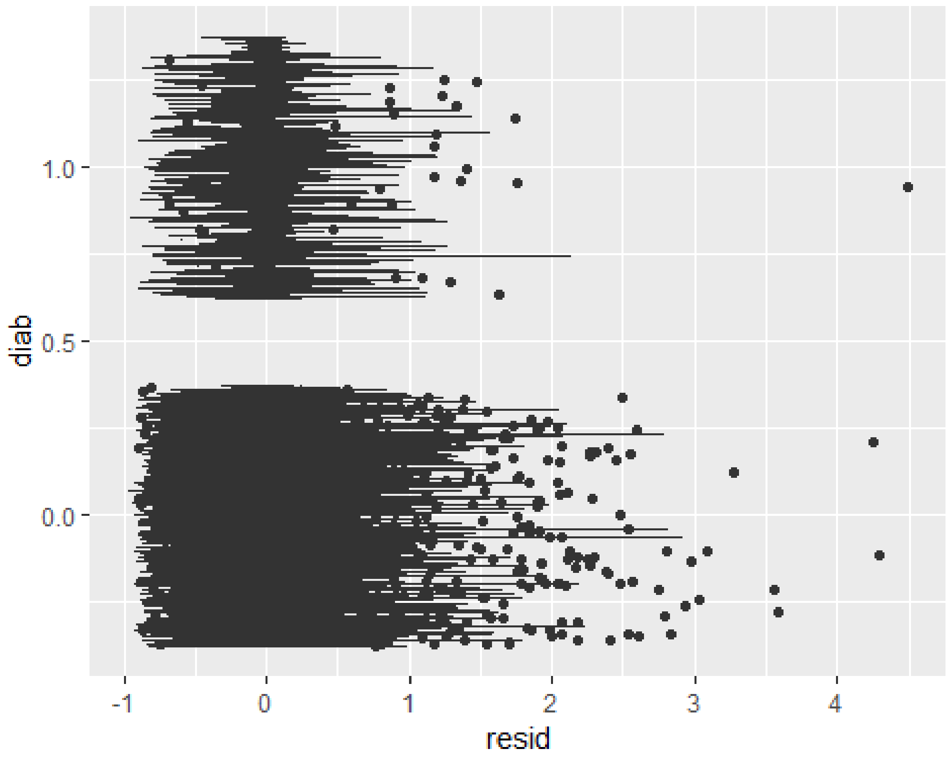


```
ggplot(augDat,aes(x=naprox,y=resid,group=ID))+geom_boxplot()+coord_flip()
```

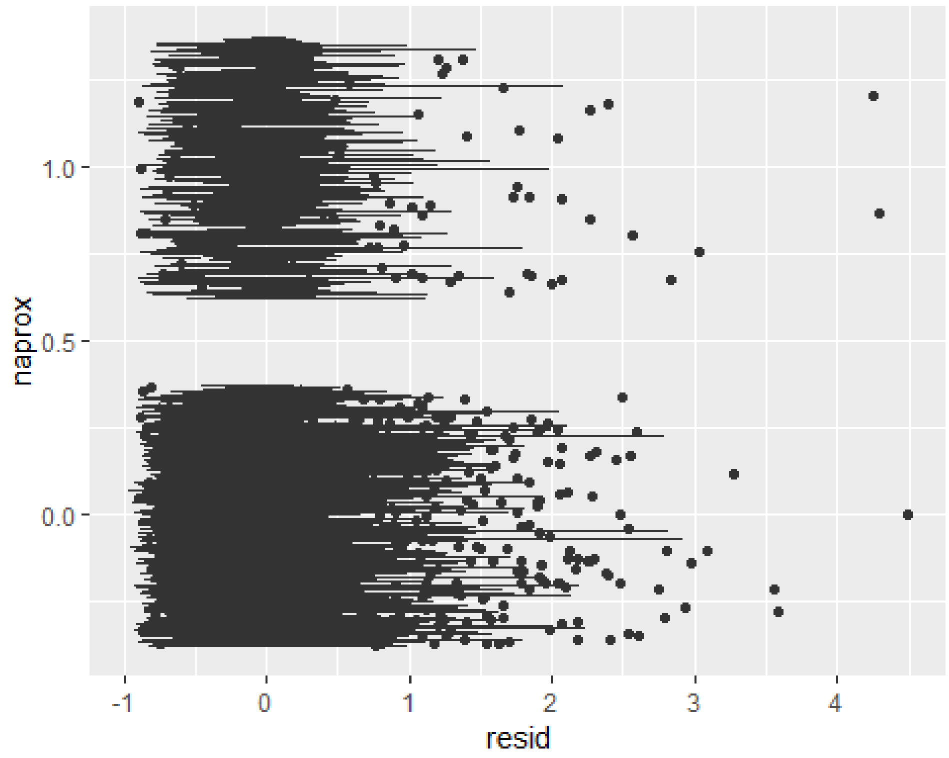


```
ggplot(augDat,aes(x=celex,y=resid,group=ID))+geom_boxplot()+coord_flip()
```

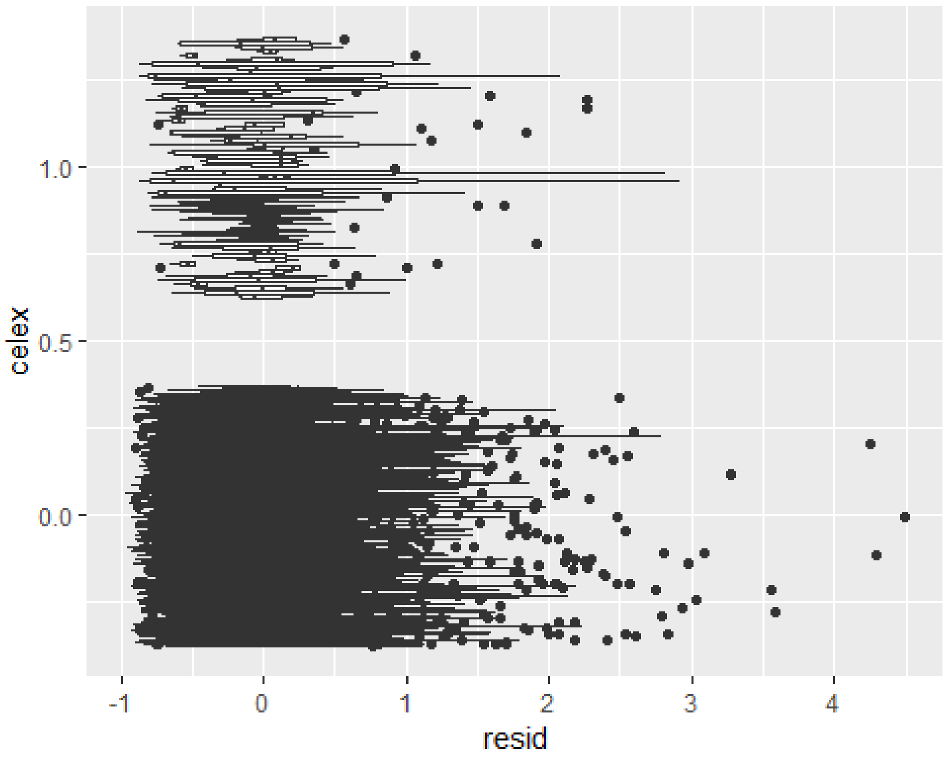


```
ggplot(augDat,aes(x=Gender,y=resid))+geom_boxplot()+coord_flip()
```

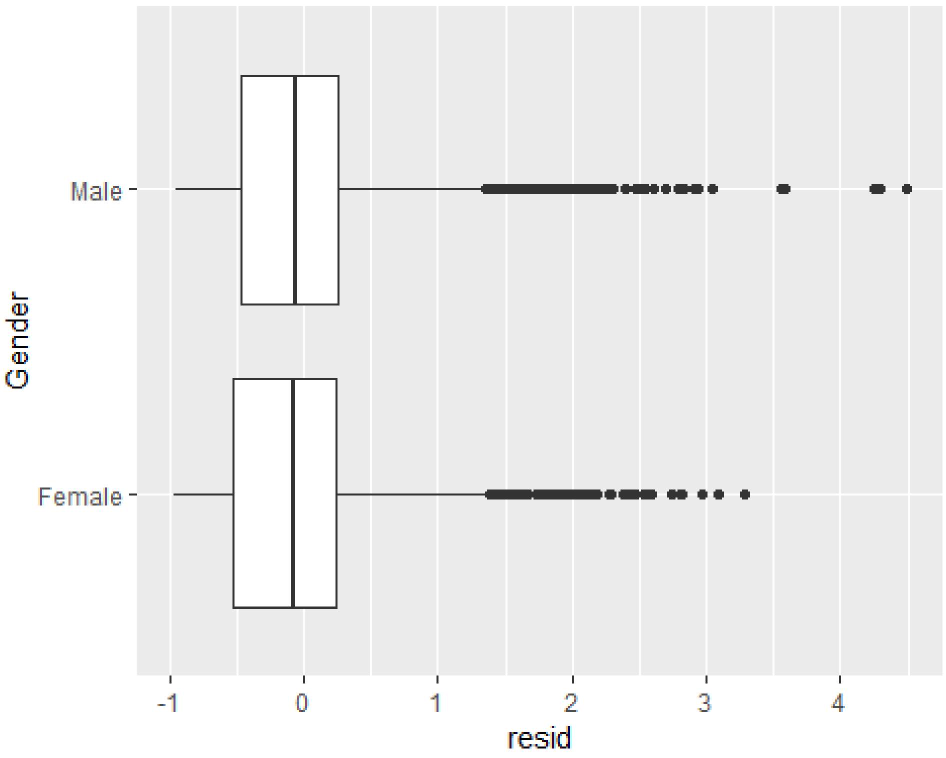


```
ggplot(augDat,aes(x=AGE,y=resid))+geom_point()+coord_flip()+geom_smooth(method=l
m)
```

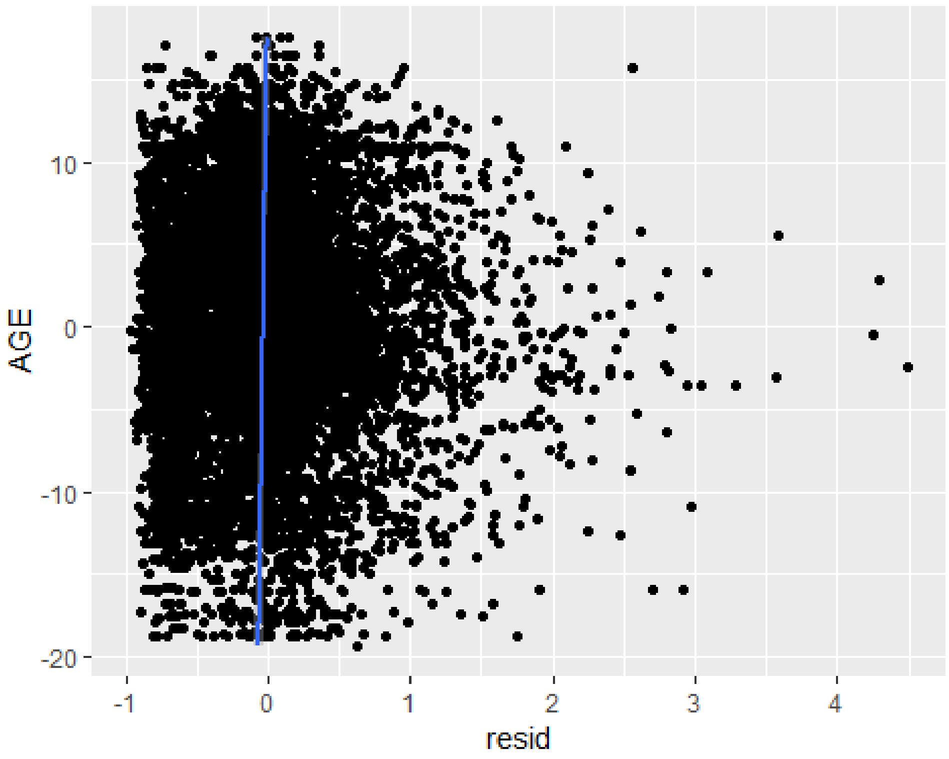


```
ggplot(augDat,aes(x=edu.cat,y=resid))+geom_boxplot()+coord_flip()
```

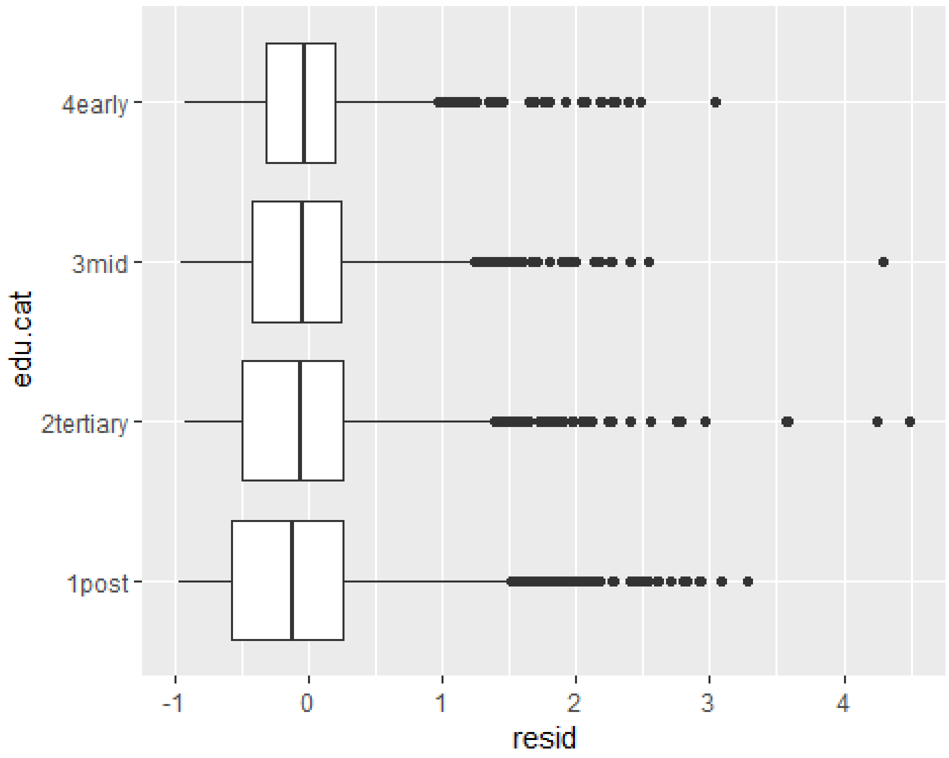


```
ggplot(augDat,aes(x=diagn,y=resid))+geom_boxplot()+coord_flip()
```

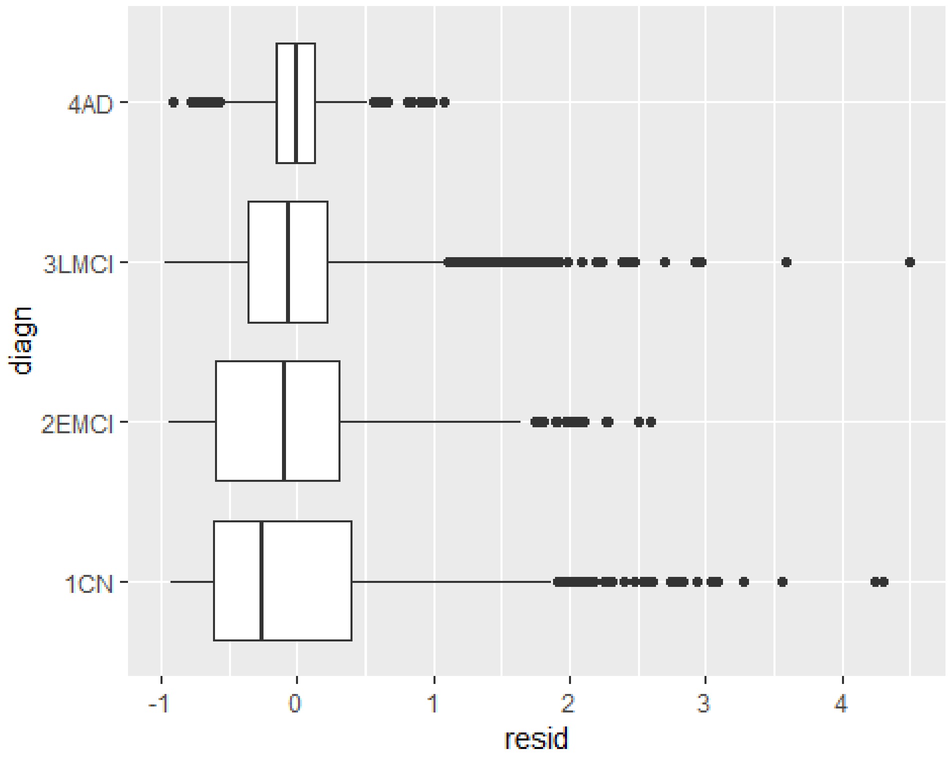


```
ggplot(augDat,aes(x=APOE4,y=resid))+geom_boxplot()+coord_flip()
```

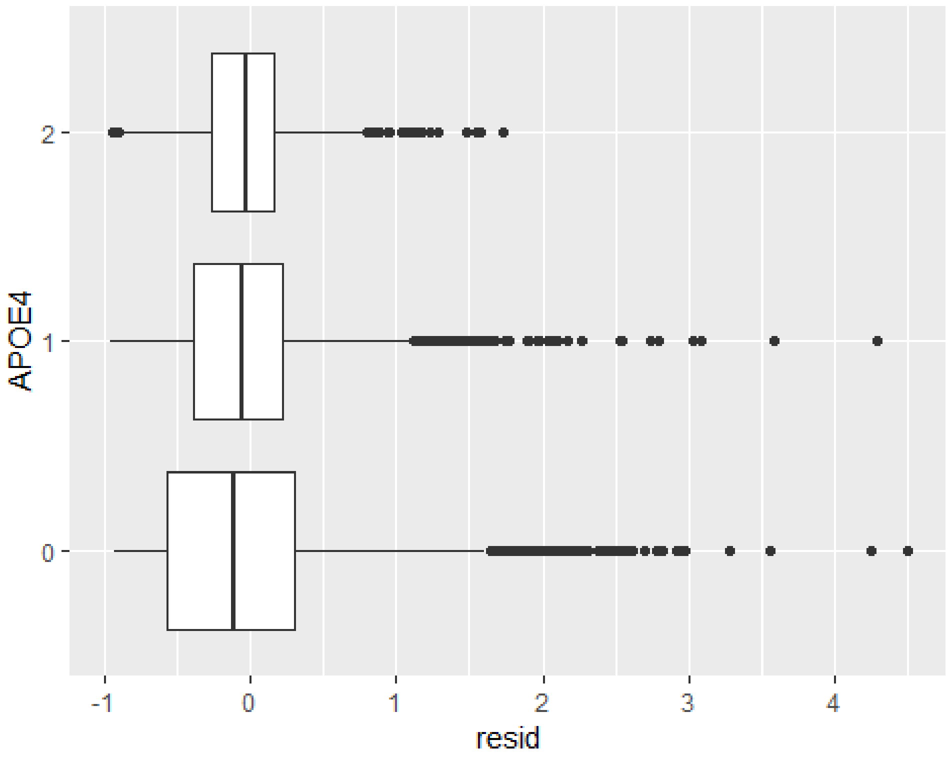


```
ggplot(augDat,aes(x=as.factor(M),y=resid))+geom_boxplot()+coord_flip()
```

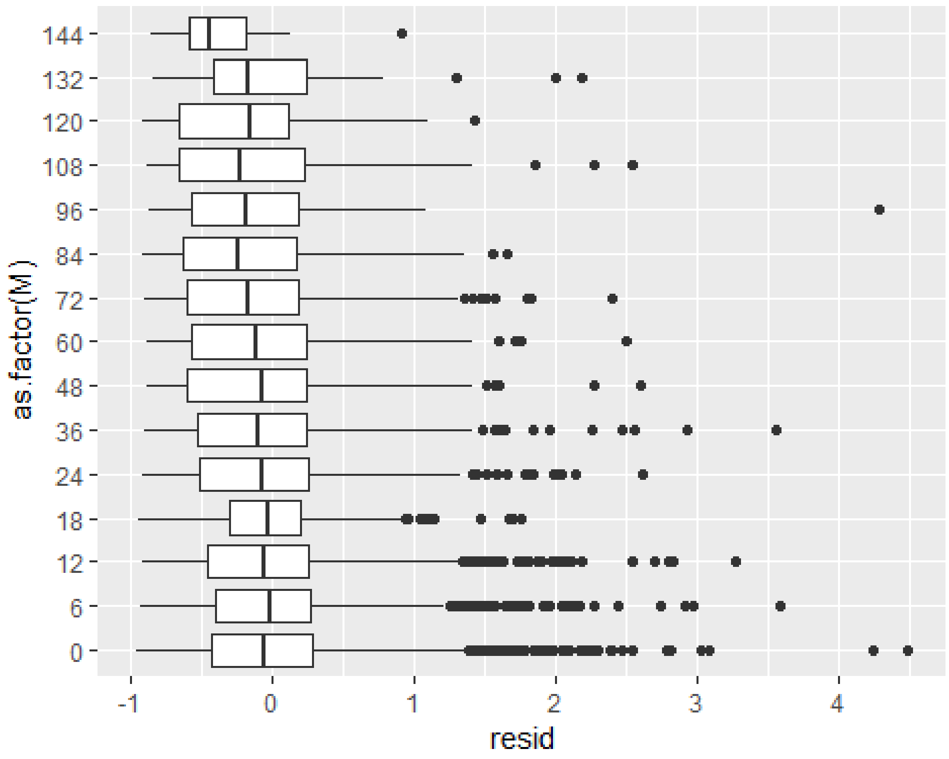


```
ggplot(augDat,aes(x=as.factor(diclo),y=resid))+geom_boxplot()+coord_flip()
```

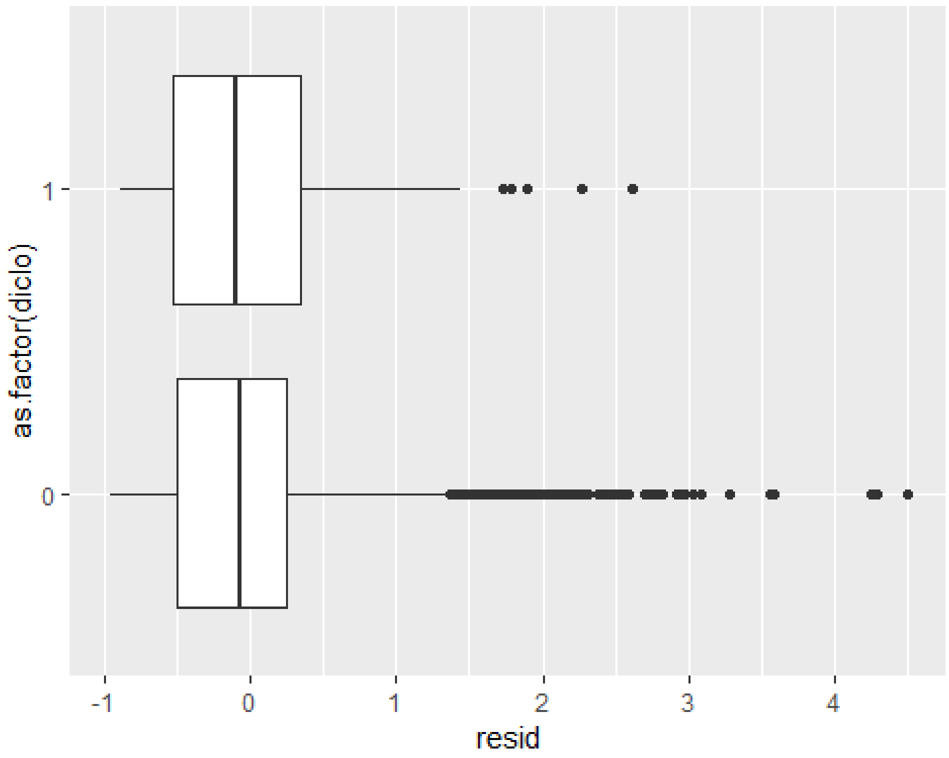


```
ggplot(augDat,aes(x=as.factor(vasc),y=resid))+geom_boxplot()+coord_flip()
```

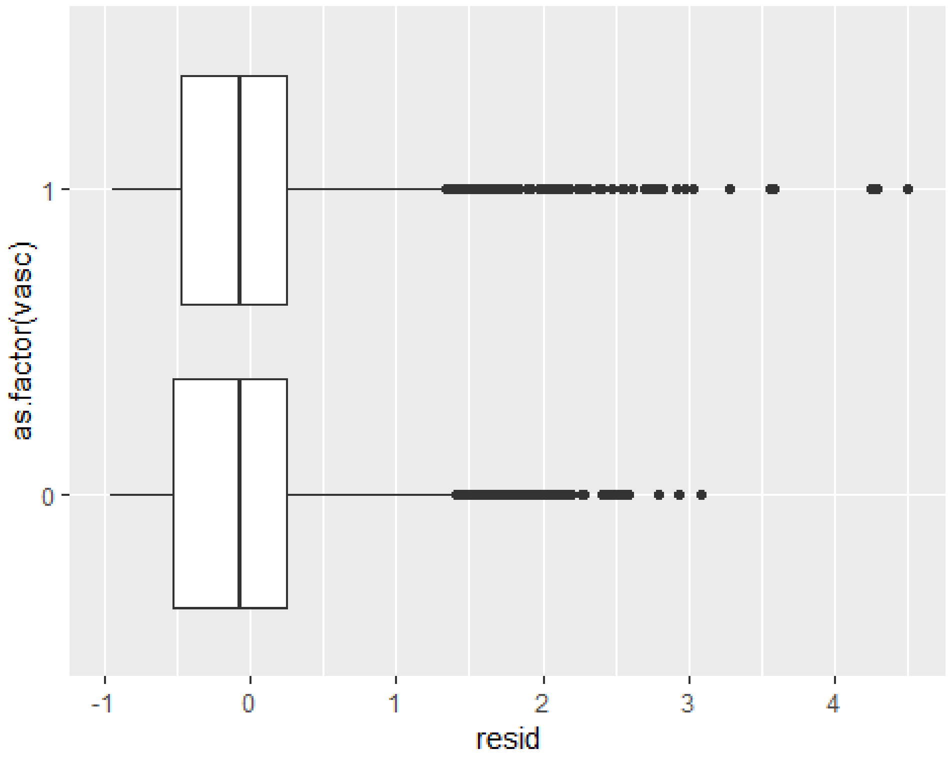


```
ggplot(augDat,aes(x=as.factor(Ibu),y=resid))+geom_boxplot()+coord_flip()
```

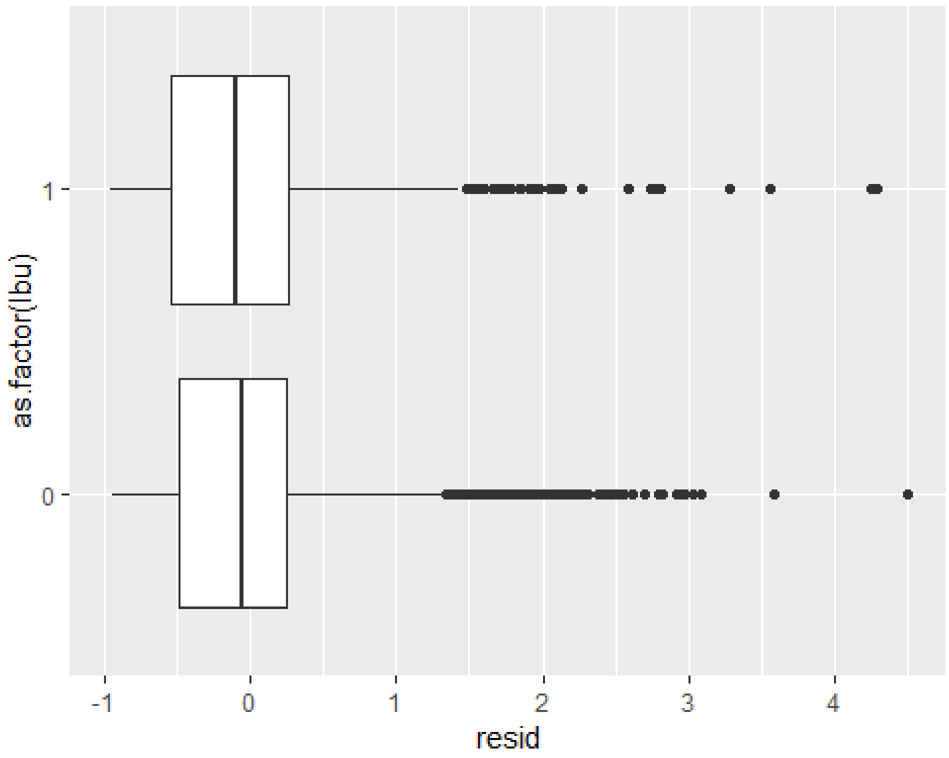


```
ggplot(augDat,aes(x=as.factor(aspirin),y=resid))+geom_boxplot()+coord_flip()
```

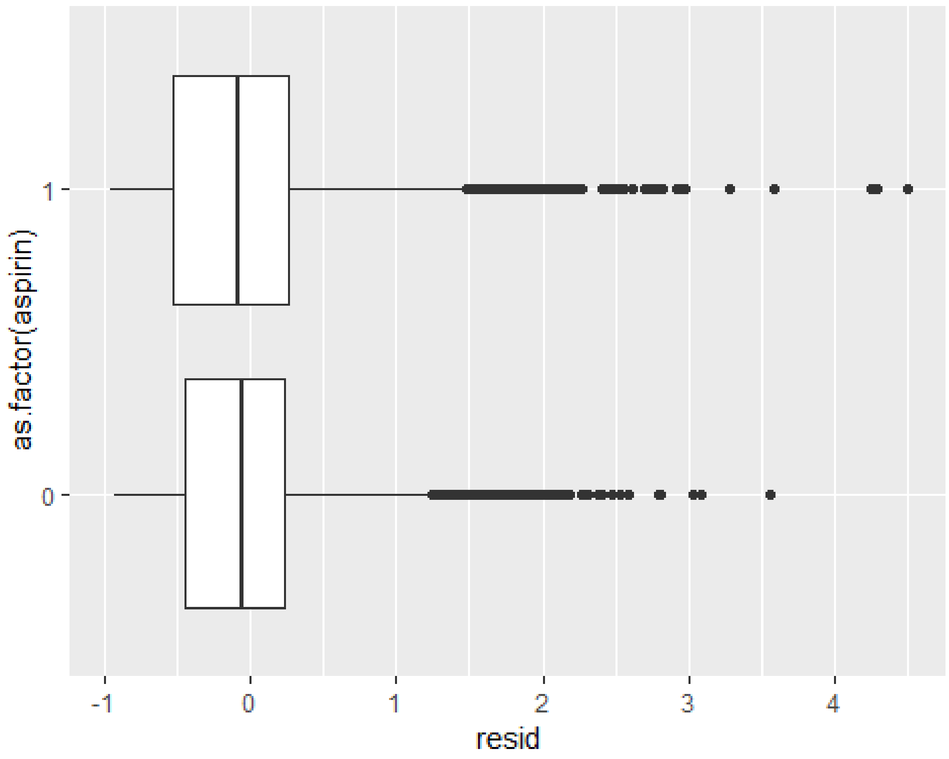


```
ggplot(augDat,aes(x=as.factor(diab),y=resid))+geom_boxplot()+coord_flip()
```

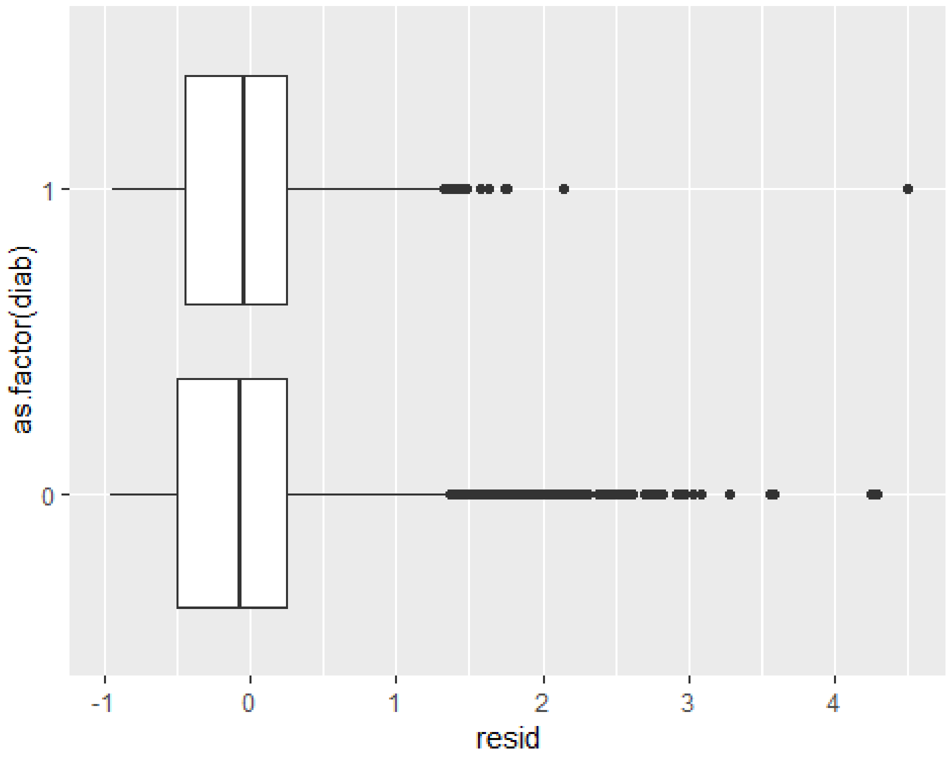


```
ggplot(augDat,aes(x=as.factor(naprox),y=resid))+geom_boxplot()+coord_flip()
```

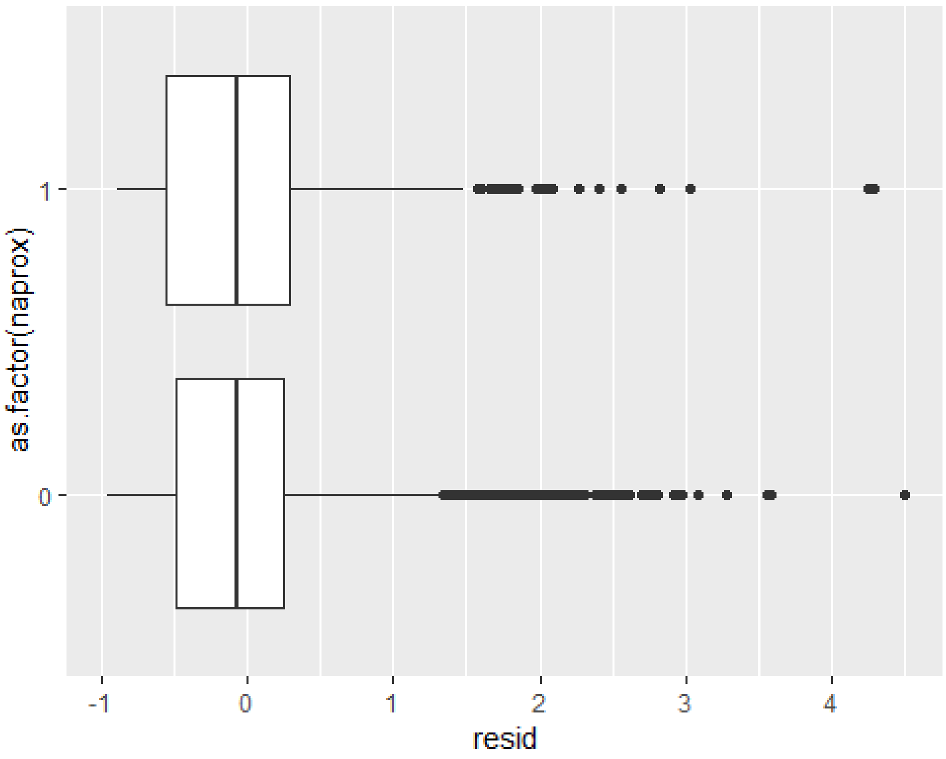


```
ggplot(augDat,aes(x=as.factor(celex),y=resid))+geom_boxplot()+coord_flip()
```

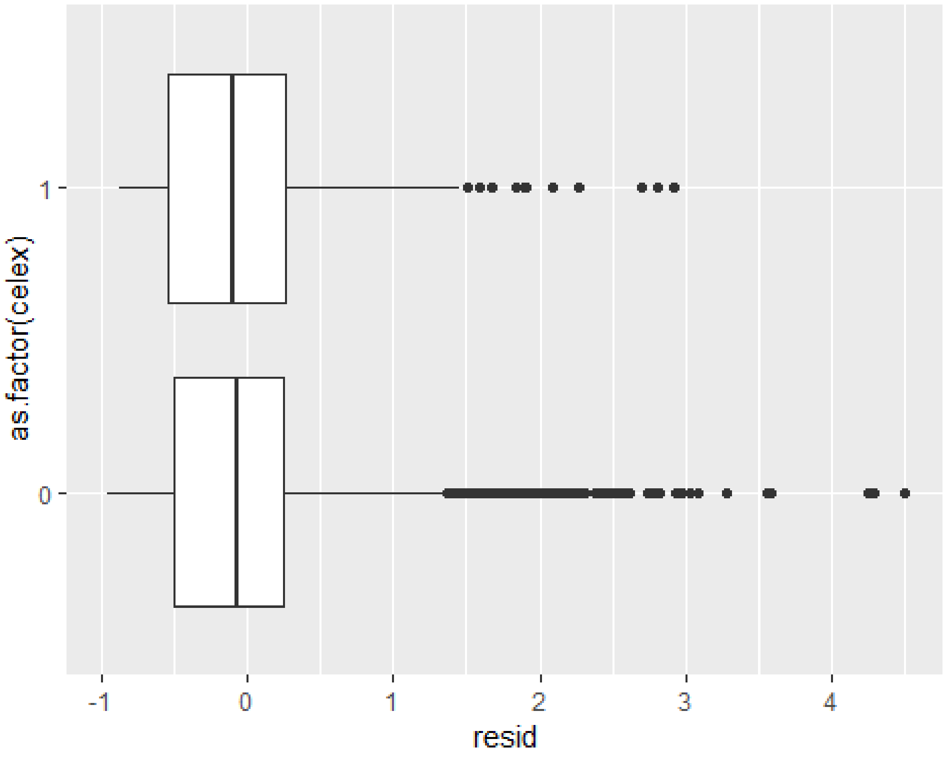

7.8. *Analysing all variables in isolation*

7.8.1. *Main effect of gender*

Adding the main effect of gender to the model did significantly improve the model based on the log-likelihood statistic.

```
main.M.M<-glmmadmb(neg.b.MMSE~M+ (1|ID), family="nbinom1", data=MMSEdata)
main.M.Gender <- glmmadmb(neg.b.MMSE~ M + Gender+ (1|ID), family="nbinom1",
data=MMSEdata)
summary(main.M.Gender)


Call:
glmmadmb(formula = neg.b.MMSE ~ M + Gender + (1 | ID), data = MMSEdata,
    family = "nbinom1")

AIC: 35674.4

Coefficients:
            Estimate Std. Error z value Pr(>|z|)
(Intercept) 0.561427   0.045491   12.34   <2e-16 ***
M           0.012465   0.000293   42.48   <2e-16 ***
GenderMale  0.116607   0.059335    1.97    0.049 *
---
Signif. codes:  0 '***' 0.001 '**' 0.01 '*' 0.05 '.' 0.1 ' ' 1

Number of observations: total=8878, ID=1619
Random effect variance(s):
Group=ID
            Variance StdDev
(Intercept)    1.265  1.125

Negative binomial dispersion parameter: 1.0853 (std. err.: 0.018723)

Log-likelihood: -17832.2

anova(main.M.M,main.M.Gender)

Analysis of Deviance Table

Model 1: neg.b.MMSE ~ M
Model 2: neg.b.MMSE ~ M + Gender
  NoPar LogLik Df Deviance Pr(>Chi)
1     4 -17834
2     5 -17832  1        4   0.0455 *
---
Signif. codes:  0 '***' 0.001 '**' 0.01 '*' 0.05 '.' 0.1 ' ' 1
```

7.8.2. *Main effect of age*

Adding the main effect of age at the begining of the study (AGE) to the model did improve the model as measured by the log-likelihood statistic.

```
main.M.AGE <- glmmadmb(neg.b.MMSE~ M + AGE+ (1|ID), family="nbinom1",
data=MMSEdata)
summary(main.M.AGE)
```

```
Call:
glmmadmb(formula = neg.b.MMSE ~ M + AGE + (1 | ID), data = MMSEdata,
    family = "nbinom1")

AIC: 35646.2

Coefficients:
            Estimate Std. Error z value Pr(>|z|)
(Intercept) 0.624381   0.030347   20.57  < 2e-16 ***
M           0.012464   0.000293   42.49  < 2e-16 ***
AGE         0.022869   0.004026    5.68  1.3e-08 ***
---
Signif. codes:  0 '***' 0.001 '**' 0.01 '*' 0.05 '.' 0.1 ' ' 1

Number of observations: total=8878, ID=1619
Random effect variance(s):
Group=ID
            Variance StdDev
(Intercept)    1.243  1.115

Negative binomial dispersion parameter: 1.0855 (std. err.: 0.018719)

Log-likelihood: -17818.1

anova(main.M.M,main.M.AGE)

Analysis of Deviance Table

Model 1: neg.b.MMSE ~ M
Model 2: neg.b.MMSE ~ M + AGE
  NoPar LogLik Df Deviance  Pr(>Chi)
1     4 -17834
2     5 -17818  1     32.2 1.391e-08 ***
---
Signif. codes:  0 '***' 0.001 '**' 0.01 '*' 0.05 '.' 0.1 ' ' 1
```

7.8.3. Main effect of education level

Adding the main effect of education (edu.cat) to the model did improve the model as measured by the log-likelihood statistic.

```
main.M.edu.cat <- glmmadmb(neg.b.MMSE~ M + edu.cat+ (1|ID), family="nbinom1",
data=MMSEdata)
summary(main.M.edu.cat)


Call:
glmmadmb(formula = neg.b.MMSE ~ M + edu.cat + (1 | ID), data = MMSEdata,
    family = "nbinom1")

AIC: 35607.2

Coefficients:
                 Estimate Std. Error z value Pr(>|z|)
(Intercept)      0.349889   0.050142    6.98  3.0e-12 ***
M                0.012452   0.000293   42.45  < 2e-16 ***
edu.cat2tertiary 0.279298   0.072945    3.83  0.00013 ***
edu.cat3mid      0.380995   0.081148    4.70  2.7e-06 ***
```

```
edu.cat4early    0.726814   0.084890    8.56  < 2e-16 ***
---
Signif. codes:  0 '***' 0.001 '**' 0.01 '*' 0.05 '.' 0.1 ' ' 1

Number of observations: total=8878, ID=1619
Random effect variance(s):
Group=ID
            Variance StdDev
(Intercept)    1.206  1.098

Negative binomial dispersion parameter: 1.0858 (std. err.: 0.018735)

Log-likelihood: -17796.6

anova(main.M.M,main.M.edu.cat)

Analysis of Deviance Table

Model 1: neg.b.MMSE ~ M
Model 2: neg.b.MMSE ~ M + edu.cat
  NoPar LogLik Df Deviance  Pr(>Chi)
1     4 -17834
2     7 -17797  3     75.2 3.331e-16 ***
---
Signif. codes:  0 '***' 0.001 '**' 0.01 '*' 0.05 '.' 0.1 ' ' 1
```

7.8.4. Main effect of diagnosis

Adding the main effect of initial diagnosis (diag) to the model did improve the model as measured by the log-likelihood statistic.

```
main.M.diagn <- glmmadmb(neg.b.MMSE~ M + diagn+ (1|ID), family="nbinom1",
data=MMSEdata)
summary(main.M.diagn)


Call:
glmmadmb(formula = neg.b.MMSE ~ M + diagn + (1 | ID), data = MMSEdata,
    family = "nbinom1")

AIC: 34355.2

Coefficients:
            Estimate Std. Error z value Pr(>|z|)
(Intercept) -0.49857    0.04281   -11.6   <2e-16 ***
M            0.01268    0.00029    43.7   <2e-16 ***
diagn2EMCI   0.67645    0.06109    11.1   <2e-16 ***
diagn3LMCI   1.44331    0.05161    28.0   <2e-16 ***
diagn4AD     2.43281    0.05776    42.1   <2e-16 ***
---
Signif. codes:  0 '***' 0.001 '**' 0.01 '*' 0.05 '.' 0.1 ' ' 1

Number of observations: total=8878, ID=1619
Random effect variance(s):
Group=ID
            Variance StdDev
(Intercept)   0.4685 0.6844

Negative binomial dispersion parameter: 1.0963 (std. err.: 0.018888)
```

```
Log-likelihood: -17170.6

anova(main.M.M,main.M.diagn)

Analysis of Deviance Table

Model 1: neg.b.MMSE ~ M
Model 2: neg.b.MMSE ~ M + diagn
  NoPar LogLik Df Deviance  Pr(>Chi)
1     4 -17834
2     7 -17171  3   1327.2 < 2.2e-16 ***
---
Signif. codes:  0 '***' 0.001 '**' 0.01 '*' 0.05 '.' 0.1 ' ' 1
```

7.8.5. Main effect of APOE status

```
main.M.APOE4<-glmmadmb(neg.b.MMSE~M+APOE4+ (1|ID), family="nbinom1",
data=MMSEdata)
main.M.Gender <- glmmadmb(neg.b.MMSE~ M + Gender+ (1|ID), family="nbinom1",
data=MMSEdata)
summary(main.M.APOE4)


Call:
glmmadmb(formula = neg.b.MMSE ~ M + APOE4 + (1 | ID), data = MMSEdata,
    family = "nbinom1")

AIC: 35494

Coefficients:
            Estimate Std. Error z value Pr(>|z|)
(Intercept) 0.271544   0.040061    6.78  1.2e-11 ***
M           0.012464   0.000293   42.52  < 2e-16 ***
APOE41      0.661684   0.059409   11.14  < 2e-16 ***
APOE42      1.038219   0.092176   11.26  < 2e-16 ***
---
Signif. codes:  0 '***' 0.001 '**' 0.01 '*' 0.05 '.' 0.1 ' ' 1

Number of observations: total=8878, ID=1619
Random effect variance(s):
Group=ID
            Variance StdDev
(Intercept)    1.111  1.054

Negative binomial dispersion parameter: 1.0865 (std. err.: 0.018743)

Log-likelihood: -17741

anova(main.M.M,main.M.APOE4)

Analysis of Deviance Table

Model 1: neg.b.MMSE ~ M
Model 2: neg.b.MMSE ~ M + APOE4
  NoPar LogLik Df Deviance  Pr(>Chi)
1     4 -17834
2     6 -17741  2    186.4 < 2.2e-16 ***
---
Signif. codes:  0 '***' 0.001 '**' 0.01 '*' 0.05 '.' 0.1 ' ' 1
```

7.8.6. Main effect of cardiovascular pathology

Adding the main effect of cardiovascular co-morbidity (vasc) to the model did significantly improve the model as measured by the log-likelihood statistic.

```
main.M.vasc <- glmmadmb(neg.b.MMSE~ M + vasc+ (1|ID), family="nbinom1",
data=MMSEdata)
summary(main.M.vasc)


Call:
glmmadmb(formula = neg.b.MMSE ~ M + vasc + (1 | ID), data = MMSEdata,
    family = "nbinom1")

AIC: 35678.2

Coefficients:
            Estimate Std. Error z value Pr(>|z|)
(Intercept) 0.609684   0.048296   12.62   <2e-16 ***
M           0.012465   0.000293   42.47   <2e-16 ***
vasc        0.028585   0.060548    0.47     0.64
---
Signif. codes:  0 '***' 0.001 '**' 0.01 '*' 0.05 '.' 0.1 ' ' 1

Number of observations: total=8878, ID=1619
Random effect variance(s):
Group=ID
            Variance StdDev
(Intercept)    1.266  1.125

Negative binomial dispersion parameter: 1.0855 (std. err.: 0.018729)

Log-likelihood: -17834.1

anova(main.M.M,main.M.vasc)

Analysis of Deviance Table

Model 1: neg.b.MMSE ~ M
Model 2: neg.b.MMSE ~ M + vasc
  NoPar LogLik Df Deviance Pr(>Chi)
1     4 -17834
2     5 -17834  1      0.2   0.6547
```

7.8.7. Main effect of diabetes

Adding the main effect of diabetes co-morbidity (diab) to the model did not improve the model as measured by the log-likelihood statistic.

```
main.M.diab <- glmmadmb(neg.b.MMSE~ M + diab+ (1|ID), family="nbinom1",
data=MMSEdata)
summary(main.M.diab)


Call:
glmmadmb(formula = neg.b.MMSE ~ M + diab + (1 | ID), data = MMSEdata,
    family = "nbinom1")

AIC: 35678.2
```

```
Coefficients:
            Estimate Std. Error z value Pr(>|z|)
(Intercept) 0.624510   0.031996    19.5   <2e-16 ***
M           0.012466   0.000293    42.5   <2e-16 ***
diab        0.030173   0.101240     0.3     0.77
---
Signif. codes:  0 '***' 0.001 '**' 0.01 '*' 0.05 '.' 0.1 ' ' 1

Number of observations: total=8878, ID=1619
Random effect variance(s):
Group=ID
            Variance StdDev
(Intercept)    1.267  1.125

Negative binomial dispersion parameter: 1.0855 (std. err.: 0.018728)

Log-likelihood: -17834.1

anova(main.M.M,main.M.diab)

Analysis of Deviance Table

Model 1: neg.b.MMSE ~ M
Model 2: neg.b.MMSE ~ M + diab
  NoPar LogLik Df Deviance Pr(>Chi)
1     4 -17834
2     5 -17834  1      0.2   0.6547
```

7.8.8. Main effect of smoking

Adding the main effect of smoking at the begining of the study (AGE) to the model did improve the model as measured by the log-likelihood statistic.

```
main.M.smoke <- glmmadmb(neg.b.MMSE~ M + smoke+ (1|ID), family="nbinom1",
data=MMSEdata)
summary(main.M.smoke)


Call:
glmmadmb(formula = neg.b.MMSE ~ M + smoke + (1 | ID), data = MMSEdata,
    family = "nbinom1")

AIC: 35678.2

Coefficients:
            Estimate Std. Error z value Pr(>|z|)
(Intercept) 0.619744   0.035120   17.65   <2e-16 ***
M           0.012465   0.000293   42.48   <2e-16 ***
smoke       0.029579   0.067375    0.44     0.66
---
Signif. codes:  0 '***' 0.001 '**' 0.01 '*' 0.05 '.' 0.1 ' ' 1

Number of observations: total=8878, ID=1619
Random effect variance(s):
Group=ID
            Variance StdDev
(Intercept)    1.267  1.125
```

```
Negative binomial dispersion parameter: 1.0855 (std. err.: 0.018728)

Log-likelihood: -17834.1

anova(main.M.M,main.M.smoke)

Analysis of Deviance Table

Model 1: neg.b.MMSE ~ M
Model 2: neg.b.MMSE ~ M + smoke
  NoPar LogLik Df Deviance Pr(>Chi)
1     4 -17834
2     5 -17834  1      0.2   0.6547
```

7.8.9. *Main effect of headache*

Adding the main effect of headache at the begining of the study (AGE) to the model did improve the model as measured by the log-likelihood statistic.

```
main.M.headache <- glmmadmb(neg.b.MMSE~ M + headache+ (1|ID), family="nbinom1",
data=MMSEdata)
summary(main.M.headache)


Call:
glmmadmb(formula = neg.b.MMSE ~ M + headache + (1 | ID), data = MMSEdata,
    family = "nbinom1")

AIC: 35674.2

Coefficients:
             Estimate Std. Error z value Pr(>|z|)
(Intercept)  0.645609   0.031778   20.32   <2e-16 ***
M            0.012476   0.000293   42.51   <2e-16 ***
headache    -0.213805   0.105090   -2.03    0.042 *
---
Signif. codes:  0 '***' 0.001 '**' 0.01 '*' 0.05 '.' 0.1 ' ' 1

Number of observations: total=8878, ID=1619
Random effect variance(s):
Group=ID
            Variance StdDev
(Intercept)    1.263  1.124

Negative binomial dispersion parameter: 1.0855 (std. err.: 0.018722)

Log-likelihood: -17832.1

anova(main.M.M,main.M.headache)

Analysis of Deviance Table

Model 1: neg.b.MMSE ~ M
Model 2: neg.b.MMSE ~ M + headache
  NoPar LogLik Df Deviance Pr(>Chi)
1     4 -17834
2     5 -17832  1      4.2  0.04042 *
---
Signif. codes:  0 '***' 0.001 '**' 0.01 '*' 0.05 '.' 0.1 ' ' 1
```

7.8.10. Main effect of arthritis

Adding the main effect of headache at the begining of the study (AGE) to the model did improve the model as measured by the log-likelihood statistic.

```
main.M.arthrit <- glmmadmb(neg.b.MMSE~ M + arthrit+ (1|ID), family="nbinom1",
data=MMSEdata)
summary(main.M.arthrit)


Call:
glmmadmb(formula = neg.b.MMSE ~ M + arthrit + (1 | ID), data = MMSEdata,
    family = "nbinom1")

AIC: 35668

Coefficients:
             Estimate Std. Error z value Pr(>|z|)
(Intercept)  0.702304   0.038229   18.37   <2e-16 ***
M            0.012476   0.000293   42.51   <2e-16 ***
arthrit     -0.193263   0.060229   -3.21   0.0013 **
---
Signif. codes:  0 '***' 0.001 '**' 0.01 '*' 0.05 '.' 0.1 ' ' 1

Number of observations: total=8878, ID=1619
Random effect variance(s):
Group=ID
            Variance StdDev
(Intercept)    1.257  1.121

Negative binomial dispersion parameter: 1.0857 (std. err.: 0.01873)

Log-likelihood: -17829

anova(main.M.M,main.M.arthrit)

Analysis of Deviance Table

Model 1: neg.b.MMSE ~ M
Model 2: neg.b.MMSE ~ M + arthrit
  NoPar LogLik Df Deviance Pr(>Chi)
1     4 -17834
2     5 -17829  1     10.4  0.00126 **
---
Signif. codes:  0 '***' 0.001 '**' 0.01 '*' 0.05 '.' 0.1 ' ' 1
```

7.8.11. Main effect of diclofenac

Adding the main effect of diclofenac to the model did not improve the model as measured by the log-likelihood statistic.

```
main.M.diclo <- glmmadmb(neg.b.MMSE~M+diclo+ (1|ID), family="nbinom1",
data=MMSEdata)
summary(main.M.diclo)


Call:
glmmadmb(formula = neg.b.MMSE ~ M + diclo + (1 | ID), data = MMSEdata,
    family = "nbinom1")
```

```
AIC: 35671.6

Coefficients:
             Estimate Std. Error z value Pr(>|z|)
(Intercept)  0.637814   0.030752   20.74   <2e-16 ***
M            0.012475   0.000293   42.52   <2e-16 ***
diclo       -0.566154   0.219460   -2.58   0.0099 **
---
Signif. codes:  0 '***' 0.001 '**' 0.01 '*' 0.05 '.' 0.1 ' ' 1

Number of observations: total=8878, ID=1619
Random effect variance(s):
Group=ID
            Variance StdDev
(Intercept)    1.261  1.123

Negative binomial dispersion parameter: 1.0853 (std. err.: 0.018722)

Log-likelihood: -17830.8

anova(main.M.M,main.M.diclo)

Analysis of Deviance Table

Model 1: neg.b.MMSE ~ M
Model 2: neg.b.MMSE ~ M + diclo
  NoPar LogLik Df Deviance Pr(>Chi)
1     4 -17834
2     5 -17831  1      6.8 0.009116 **
---
Signif. codes:  0 '***' 0.001 '**' 0.01 '*' 0.05 '.' 0.1 ' ' 1
```

7.8.12. Main effect of paracetamol

Adding the main effect of paracetamol to the model did improve the model as measured by the log-likelihood statistic.

```
main.M.parac <- glmmadmb(neg.b.MMSE~ M + parac+ (1|ID), family="nbinom1",
data=MMSEdata)
summary(main.M.parac)


Call:
glmmadmb(formula = neg.b.MMSE ~ M + parac + (1 | ID), data = MMSEdata,
    family = "nbinom1")

AIC: 35670

Coefficients:
             Estimate Std. Error z value Pr(>|z|)
(Intercept)  0.676397   0.034739    19.5   <2e-16 ***
M            0.012491   0.000294    42.5   <2e-16 ***
parac       -0.196590   0.067781    -2.9   0.0037 **
---
Signif. codes:  0 '***' 0.001 '**' 0.01 '*' 0.05 '.' 0.1 ' ' 1

Number of observations: total=8878, ID=1619
Random effect variance(s):
```

```
Group=ID
            Variance StdDev
(Intercept)    1.258  1.122

Negative binomial dispersion parameter: 1.0855 (std. err.: 0.018723)

Log-likelihood: -17830

anova(main.M.M,main.M.parac)

Analysis of Deviance Table

Model 1: neg.b.MMSE ~ M
Model 2: neg.b.MMSE ~ M + parac
  NoPar LogLik Df Deviance Pr(>Chi)
1     4 -17834
2     5 -17830  1      8.4 0.003752 **
---
Signif. codes:  0 '***' 0.001 '**' 0.01 '*' 0.05 '.' 0.1 ' ' 1
```

7.8.13. Main effect celecoxib

Adding the main effect of celecoxib to the model did not improve the model as measured by the log-likelihood statistic.

```
main.M.celex <- glmmadmb(neg.b.MMSE~ M + celex+ (1|ID), family="nbinom1",
data=MMSEdata)
summary(main.M.celex)


Call:
glmmadmb(formula = neg.b.MMSE ~ M + celex + (1 | ID), data = MMSEdata,
    family = "nbinom1")

AIC: 35674.4

Coefficients:
             Estimate Std. Error z value Pr(>|z|)
(Intercept)  0.639311   0.031086    20.6   <2e-16 ***
M            0.012470   0.000293    42.5   <2e-16 ***
celex       -0.302623   0.151440    -2.0    0.046 *
---
Signif. codes:  0 '***' 0.001 '**' 0.01 '*' 0.05 '.' 0.1 ' ' 1

Number of observations: total=8878, ID=1619
Random effect variance(s):
Group=ID
            Variance StdDev
(Intercept)    1.263  1.124

Negative binomial dispersion parameter: 1.0856 (std. err.: 0.018726)

Log-likelihood: -17832.2

anova(main.M.M,main.M.celex)

Analysis of Deviance Table

Model 1: neg.b.MMSE ~ M
```

```
Model 2: neg.b.MMSE ~ M + celex
  NoPar LogLik Df Deviance Pr(>Chi)
1     4 -17834
2     5 -17832  1        4   0.0455 *
---
Signif. codes:  0 '***' 0.001 '**' 0.01 '*' 0.05 '.' 0.1 ' ' 1
```

7.8.14. Main effect of naproxen

Adding the main effect of naproxen to the model did significantly improve the model as measured by the log-likelihood statistic.

```
main.M.naprox <- glmmadmb(neg.b.MMSE~ M + naprox+ (1|ID), family="nbinom1",
data=MMSEdata)
summary(main.M.naprox)


Call:
glmmadmb(formula = neg.b.MMSE ~ M + naprox + (1 | ID), data = MMSEdata,
    family = "nbinom1")

AIC: 35671

Coefficients:
             Estimate Std. Error z value Pr(>|z|)
(Intercept)  0.656194   0.032227   20.36   <2e-16 ***
M            0.012480   0.000294   42.52   <2e-16 ***
naprox      -0.248943   0.091866   -2.71   0.0067 **
---
Signif. codes:  0 '***' 0.001 '**' 0.01 '*' 0.05 '.' 0.1 ' ' 1

Number of observations: total=8878, ID=1619
Random effect variance(s):
Group=ID
            Variance StdDev
(Intercept)    1.259  1.122

Negative binomial dispersion parameter: 1.0858 (std. err.: 0.01873)

Log-likelihood: -17830.5

anova(main.M.M,main.M.naprox)

Analysis of Deviance Table

Model 1: neg.b.MMSE ~ M
Model 2: neg.b.MMSE ~ M + naprox
  NoPar LogLik Df Deviance Pr(>Chi)
1     4 -17834
2     5 -17831  1      7.4 0.006522 **
---
Signif. codes:  0 '***' 0.001 '**' 0.01 '*' 0.05 '.' 0.1 ' ' 1
```

7.8.15. Main effect of aspirin

Adding the main effect of aspirin use (aspirin) to the model did improve the model as measured by the log-likelihood statistic.

```
main.M.aspirin <- glmmadmb(neg.b.MMSE~ M + aspirin+ (1|ID), family="nbinom1",
data=MMSEdata)
summary(main.M.aspirin)


Call:
glmmadmb(formula = neg.b.MMSE ~ M + aspirin + (1 | ID), data = MMSEdata,
    family = "nbinom1")

AIC: 35651.4

Coefficients:
             Estimate Std. Error z value Pr(>|z|)
(Intercept)  0.789436   0.043143    18.3  < 2e-16 ***
M            0.012512   0.000293    42.6  < 2e-16 ***
aspirin     -0.304442   0.058497    -5.2  1.9e-07 ***
---
Signif. codes:  0 '***' 0.001 '**' 0.01 '*' 0.05 '.' 0.1 ' ' 1

Number of observations: total=8878, ID=1619
Random effect variance(s):
Group=ID
            Variance StdDev
(Intercept)    1.243  1.115

Negative binomial dispersion parameter: 1.0855 (std. err.: 0.01871)

Log-likelihood: -17820.7

anova(main.M.M,main.M.aspirin)

Analysis of Deviance Table

Model 1: neg.b.MMSE ~ M
Model 2: neg.b.MMSE ~ M + aspirin
  NoPar LogLik Df Deviance  Pr(>Chi)
1     4 -17834
2     5 -17821  1       27 2.035e-07 ***
---
Signif. codes:  0 '***' 0.001 '**' 0.01 '*' 0.05 '.' 0.1 ' ' 1
```

7.8.16. Main effect of ibuprofen

Adding the main effect of ibuprofen use (Ibu) to the model did improve the model as measured by the log-likelihood statistic.

```
main.M.Ibu <- glmmadmb(neg.b.MMSE~ M + Ibu+ (1|ID), family="nbinom1",
data=MMSEdata)
summary(main.M.Ibu)


Call:
glmmadmb(formula = neg.b.MMSE ~ M + Ibu + (1 | ID), data = MMSEdata,
    family = "nbinom1")

AIC: 35657.2

Coefficients:
             Estimate Std. Error z value Pr(>|z|)
```

```
(Intercept)  0.686112   0.032787   20.93  < 2e-16 ***
M            0.012490   0.000293   42.57  < 2e-16 ***
Ibu         -0.371462   0.080479   -4.62  3.9e-06 ***
---
Signif. codes:  0 '***' 0.001 '**' 0.01 '*' 0.05 '.' 0.1 ' ' 1

Number of observations: total=8878, ID=1619
Random effect variance(s):
Group=ID
            Variance StdDev
(Intercept)    1.248  1.117

Negative binomial dispersion parameter: 1.0856 (std. err.: 0.01872)

Log-likelihood: -17823.6

anova(main.M.M,main.M.Ibu)

Analysis of Deviance Table

Model 1: neg.b.MMSE ~ M
Model 2: neg.b.MMSE ~ M + Ibu
  NoPar LogLik Df Deviance  Pr(>Chi)
1     4 -17834
2     5 -17824  1     21.2 4.138e-06 ***
---
Signif. codes:  0 '***' 0.001 '**' 0.01 '*' 0.05 '.' 0.1 ' ' 1
```

7.9. *Building combined main effect model*

```
main.M.combined <- glmmadmb(neg.b.MMSE~M+AGE+ APOE4+Gender+  edu.cat + diagn+
headache+arthrit+ diclo+parac+naprox+aspirin+Ibu+ (1|ID), family="nbinom1",
data=MMSEdata)
summary(main.M.combined)


Call:
glmmadmb(formula = neg.b.MMSE ~ M + AGE + APOE4 + Gender + edu.cat +
    diagn + headache + arthrit + diclo + parac + naprox + aspirin +
    Ibu + (1 | ID), data = MMSEdata, family = "nbinom1")

AIC: 34211.2

Coefficients:
                 Estimate Std. Error z value Pr(>|z|)
(Intercept)      -0.63958    0.06138  -10.42  < 2e-16 ***
M                 0.01274    0.00029   43.93  < 2e-16 ***
AGE               0.01833    0.00265    6.91  4.9e-12 ***
APOE41            0.26721    0.04007    6.67  2.6e-11 ***
APOE42            0.39733    0.06229    6.38  1.8e-10 ***
GenderMale        0.04831    0.03851    1.25    0.210
edu.cat2tertiary  0.11353    0.04655    2.44    0.015 *
edu.cat3mid       0.31431    0.05227    6.01  1.8e-09 ***
edu.cat4early     0.30784    0.05484    5.61  2.0e-08 ***
diagn2EMCI        0.66305    0.05960   11.13  < 2e-16 ***
diagn3LMCI        1.34688    0.05097   26.43  < 2e-16 ***
diagn4AD          2.22312    0.05849   38.01  < 2e-16 ***
headache         -0.05894    0.06593   -0.89    0.371
arthrit          -0.06332    0.03882   -1.63    0.103
```

```
diclo            -0.12938    0.13789   -0.94    0.348
parac            -0.03719    0.04346   -0.86    0.392
naprox           -0.00065    0.05791   -0.01    0.991
aspirin          -0.07873    0.03733   -2.11    0.035 *
Ibu              -0.09416    0.05090   -1.85    0.064 .
---
Signif. codes:  0 '***' 0.001 '**' 0.01 '*' 0.05 '.' 0.1 ' ' 1

Number of observations: total=8878, ID=1619
Random effect variance(s):
Group=ID
            Variance StdDev
(Intercept)   0.4157 0.6447

Negative binomial dispersion parameter: 1.096 (std. err.: 0.018805)

Log-likelihood: -17084.6

anova(main.M.M,main.M.combined)

Analysis of Deviance Table

Model 1: neg.b.MMSE ~ M
Model 2: neg.b.MMSE ~ M + AGE + APOE4 + Gender + edu.cat + diagn + headache +
arthrit + diclo + parac + naprox + aspirin + Ibu
  NoPar LogLik Df Deviance  Pr(>Chi)
1     4 -17834
2    21 -17085 17   1499.2 < 2.2e-16 ***
---
Signif. codes:  0 '***' 0.001 '**' 0.01 '*' 0.05 '.' 0.1 ' ' 1
```

7.10. *Dropping non-significant terms*

7.10.1. Narpoxen

Dropping naproxen from the model did not signficantly worsen the model

```
main.M.combined.drop.naprox <- glmmadmb(neg.b.MMSE~M+AGE+ APOE4+Gender+  edu.cat
+ diagn+ headache+arthrit+ diclo+parac+aspirin+Ibu+ (1|ID), family="nbinom1",
data=MMSEdata)
summary(main.M.combined.drop.naprox)


Call:
glmmadmb(formula = neg.b.MMSE ~ M + AGE + APOE4 + Gender + edu.cat +
    diagn + headache + arthrit + diclo + parac + aspirin + Ibu +
    (1 | ID), data = MMSEdata, family = "nbinom1")

AIC: 34209.2

Coefficients:
                 Estimate Std. Error z value Pr(>|z|)
(Intercept)      -0.63962    0.06128  -10.44  < 2e-16 ***
M                 0.01274    0.00029   43.94  < 2e-16 ***
AGE               0.01833    0.00265    6.92  4.5e-12 ***
APOE41            0.26721    0.04007    6.67  2.6e-11 ***
APOE42            0.39734    0.06228    6.38  1.8e-10 ***
GenderMale        0.04832    0.03850    1.26    0.209
```

```
edu.cat2tertiary  0.11352    0.04653    2.44    0.015 *
edu.cat3mid       0.31430    0.05226    6.01  1.8e-09 ***
edu.cat4early     0.30782    0.05483    5.61  2.0e-08 ***
diagn2EMCI        0.66304    0.05959   11.13  < 2e-16 ***
diagn3LMCI        1.34688    0.05097   26.43  < 2e-16 ***
diagn4AD          2.22315    0.05845   38.04  < 2e-16 ***
headache         -0.05894    0.06593   -0.89    0.371
arthrit          -0.06335    0.03870   -1.64    0.102
diclo            -0.12938    0.13789   -0.94    0.348
parac            -0.03725    0.04310   -0.86    0.387
aspirin          -0.07875    0.03731   -2.11    0.035 *
Ibu              -0.09419    0.05082   -1.85    0.064 .
---
Signif. codes:  0 '***' 0.001 '**' 0.01 '*' 0.05 '.' 0.1 ' ' 1

Number of observations: total=8878, ID=1619
Random effect variance(s):
Group=ID
            Variance StdDev
(Intercept)   0.4157 0.6447

Negative binomial dispersion parameter: 1.096 (std. err.: 0.018805)

Log-likelihood: -17084.6

anova(main.M.combined, main.M.combined.drop.naprox)

Analysis of Deviance Table

Model 1: neg.b.MMSE ~ M + AGE + APOE4 + Gender + edu.cat + diagn + headache +
arthrit + diclo + parac + aspirin + Ibu
Model 2: neg.b.MMSE ~ M + AGE + APOE4 + Gender + edu.cat + diagn + headache +
arthrit + diclo + parac + naprox + aspirin + Ibu
  NoPar LogLik Df Deviance Pr(>Chi)
1    20 -17085
2    21 -17085  1        0        1
```

7.10.2. Diclofenac

Dropping diclofenac from the model did not signficantly worsen the model

```
main.M.combined.drop.diclo <- glmmadmb(neg.b.MMSE~M+AGE+ APOE4+Gender+  edu.cat
+ diagn+ headache+arthrit+ parac+aspirin+Ibu+ (1|ID), family="nbinom1",
data=MMSEdata)
summary(main.M.combined.drop.diclo)


Call:
glmmadmb(formula = neg.b.MMSE ~ M + AGE + APOE4 + Gender + edu.cat +
    diagn + headache + arthrit + parac + aspirin + Ibu + (1 |
    ID), data = MMSEdata, family = "nbinom1")

AIC: 34208.2

Coefficients:
                 Estimate Std. Error z value Pr(>|z|)
(Intercept)      -0.64138    0.06127  -10.47  < 2e-16 ***
M                 0.01273    0.00029   43.93  < 2e-16 ***
AGE               0.01831    0.00265    6.91  4.8e-12 ***
```

```
APOE41            0.26710    0.04008    6.66  2.7e-11 ***
APOE42            0.39712    0.06230    6.37  1.8e-10 ***
GenderMale        0.04863    0.03850    1.26    0.207
edu.cat2tertiary  0.11277    0.04654    2.42    0.015 *
edu.cat3mid       0.31591    0.05225    6.05  1.5e-09 ***
edu.cat4early     0.30691    0.05484    5.60  2.2e-08 ***
diagn2EMCI        0.66485    0.05957   11.16  < 2e-16 ***
diagn3LMCI        1.34913    0.05092   26.49  < 2e-16 ***
diagn4AD          2.22573    0.05840   38.11  < 2e-16 ***
headache         -0.06012    0.06594   -0.91    0.362
arthrit          -0.06673    0.03855   -1.73    0.083 .
parac            -0.03790    0.04310   -0.88    0.379
aspirin          -0.08021    0.03729   -2.15    0.031 *
Ibu              -0.09404    0.05083   -1.85    0.064 .
---
Signif. codes:  0 '***' 0.001 '**' 0.01 '*' 0.05 '.' 0.1 ' ' 1

Number of observations: total=8878, ID=1619
Random effect variance(s):
Group=ID
            Variance StdDev
(Intercept)   0.4159 0.6449

Negative binomial dispersion parameter: 1.0961 (std. err.: 0.018807)

Log-likelihood: -17085.1

anova(main.M.combined.drop.naprox, main.M.combined.drop.diclo)

Analysis of Deviance Table

Model 1: neg.b.MMSE ~ M + AGE + APOE4 + Gender + edu.cat + diagn + headache +
arthrit + parac + aspirin + Ibu
Model 2: neg.b.MMSE ~ M + AGE + APOE4 + Gender + edu.cat + diagn + headache +
arthrit + diclo + parac + aspirin + Ibu
  NoPar LogLik Df Deviance Pr(>Chi)
1    19 -17085
2    20 -17085  1        1   0.3173
```

7.10.3. Headache

Dropping paracetamol from the model did not signficantly worsen the model

```
main.M.combined.drop.headache<- glmmadmb(neg.b.MMSE~M+AGE+ APOE4+Gender+
edu.cat + diagn+ arthrit+ parac+aspirin+Ibu+ (1|ID), family="nbinom1",
data=MMSEdata)
summary(main.M.combined.drop.headache)


Call:
glmmadmb(formula = neg.b.MMSE ~ M + AGE + APOE4 + Gender + edu.cat +
    diagn + arthrit + parac + aspirin + Ibu + (1 | ID), data = MMSEdata,
    family = "nbinom1")

AIC: 34207

Coefficients:
                 Estimate Std. Error z value Pr(>|z|)
(Intercept)      -0.64760    0.06093  -10.63  < 2e-16 ***
```

```
M                 0.01273    0.00029   43.92  < 2e-16 ***
AGE               0.01859    0.00263    7.06  1.7e-12 ***
APOE41            0.26828    0.04007    6.70  2.2e-11 ***
APOE42            0.39808    0.06232    6.39  1.7e-10 ***
GenderMale        0.05142    0.03840    1.34    0.180
edu.cat2tertiary  0.11416    0.04653    2.45    0.014 *
edu.cat3mid       0.31592    0.05227    6.04  1.5e-09 ***
edu.cat4early     0.30660    0.05486    5.59  2.3e-08 ***
diagn2EMCI        0.66505    0.05959   11.16  < 2e-16 ***
diagn3LMCI        1.34870    0.05094   26.48  < 2e-16 ***
diagn4AD          2.22593    0.05842   38.10  < 2e-16 ***
arthrit          -0.06899    0.03848   -1.79    0.073 .
parac            -0.04047    0.04303   -0.94    0.347
aspirin          -0.07972    0.03730   -2.14    0.033 *
Ibu              -0.09467    0.05084   -1.86    0.063 .
---
Signif. codes:  0 '***' 0.001 '**' 0.01 '*' 0.05 '.' 0.1 ' ' 1

Number of observations: total=8878, ID=1619
Random effect variance(s):
Group=ID
            Variance StdDev
(Intercept)   0.4163 0.6452

Negative binomial dispersion parameter: 1.0961 (std. err.: 0.018809)

Log-likelihood: -17085.5

anova(main.M.combined.drop.diclo, main.M.combined.drop.headache)

Analysis of Deviance Table

Model 1: neg.b.MMSE ~ M + AGE + APOE4 + Gender + edu.cat + diagn + arthrit +
parac + aspirin + Ibu
Model 2: neg.b.MMSE ~ M + AGE + APOE4 + Gender + edu.cat + diagn + headache +
arthrit + parac + aspirin + Ibu
  NoPar LogLik Df Deviance Pr(>Chi)
1    18 -17086
2    19 -17085  1      0.8   0.3711
```

7.10.4. Paracetamol

Dropping paracetamol from the model did not signficantly worsen the model

```
main.M.combined.drop.parac<- glmmadmb(neg.b.MMSE~M+AGE+ APOE4+Gender+  edu.cat +
diagn+ arthrit+ aspirin+Ibu+ (1|ID), family="nbinom1", data=MMSEdata)
summary(main.M.combined.drop.parac)


Call:
glmmadmb(formula = neg.b.MMSE ~ M + AGE + APOE4 + Gender + edu.cat +
    diagn + arthrit + aspirin + Ibu + (1 | ID), data = MMSEdata,
    family = "nbinom1")

AIC: 34205.8

Coefficients:
                 Estimate Std. Error z value Pr(>|z|)
(Intercept)      -0.65579    0.06035  -10.87  < 2e-16 ***
```

```
M                  0.01272    0.00029   43.91  < 2e-16 ***
AGE                0.01852    0.00263    7.03  2.0e-12 ***
APOE41             0.27010    0.04004    6.75  1.5e-11 ***
APOE42             0.39910    0.06234    6.40  1.5e-10 ***
GenderMale         0.05408    0.03831    1.41    0.158
edu.cat2tertiary   0.11321    0.04654    2.43    0.015 *
edu.cat3mid        0.31432    0.05226    6.01  1.8e-09 ***
edu.cat4early      0.30751    0.05487    5.60  2.1e-08 ***
diagn2EMCI         0.66580    0.05961   11.17  < 2e-16 ***
diagn3LMCI         1.34868    0.05096   26.46  < 2e-16 ***
diagn4AD           2.22737    0.05843   38.12  < 2e-16 ***
arthrit           -0.07433    0.03808   -1.95    0.051 .
aspirin           -0.08246    0.03721   -2.22    0.027 *
Ibu               -0.09906    0.05065   -1.96    0.051 .
---
Signif. codes:  0 '***' 0.001 '**' 0.01 '*' 0.05 '.' 0.1 ' ' 1

Number of observations: total=8878, ID=1619
Random effect variance(s):
Group=ID
            Variance StdDev
(Intercept)   0.4167 0.6455

Negative binomial dispersion parameter: 1.0961 (std. err.: 0.018812)

Log-likelihood: -17085.9

anova(main.M.combined.drop.headache, main.M.combined.drop.parac)

Analysis of Deviance Table

Model 1: neg.b.MMSE ~ M + AGE + APOE4 + Gender + edu.cat + diagn + arthrit +
aspirin + Ibu
Model 2: neg.b.MMSE ~ M + AGE + APOE4 + Gender + edu.cat + diagn + arthrit +
parac + aspirin + Ibu
  NoPar LogLik Df Deviance Pr(>Chi)
1    17 -17086
2    18 -17086  1      0.8   0.3711
```

7.10.5. Gender

Dropping gender from the model did not signficantly worsen the model

```
main.M.combined.drop.Gender<- glmmadmb(neg.b.MMSE~M+AGE+ APOE4+  edu.cat +
diagn+ arthrit+ aspirin+Ibu+ (1|ID), family="nbinom1", data=MMSEdata)
summary(main.M.combined.drop.Gender)


Call:
glmmadmb(formula = neg.b.MMSE ~ M + AGE + APOE4 + edu.cat + diagn +
    arthrit + aspirin + Ibu + (1 | ID), data = MMSEdata, family = "nbinom1")

AIC: 34205.8

Coefficients:
                 Estimate Std. Error z value Pr(>|z|)
(Intercept)      -0.62546    0.05632  -11.11  < 2e-16 ***
M                 0.01271    0.00029   43.90  < 2e-16 ***
AGE               0.01899    0.00261    7.27  3.5e-13 ***
```

```
APOE41            0.27082    0.04003    6.77  1.3e-11 ***
APOE42            0.40151    0.06231    6.44  1.2e-10 ***
edu.cat2tertiary  0.10977    0.04647    2.36    0.018 *
edu.cat3mid       0.30146    0.05145    5.86  4.6e-09 ***
edu.cat4early     0.29590    0.05424    5.46  4.9e-08 ***
diagn2EMCI        0.67059    0.05951   11.27  < 2e-16 ***
diagn3LMCI        1.35404    0.05082   26.65  < 2e-16 ***
diagn4AD          2.23096    0.05837   38.22  < 2e-16 ***
arthrit          -0.07919    0.03792   -2.09    0.037 *
aspirin          -0.07573    0.03689   -2.05    0.040 *
Ibu              -0.09861    0.05064   -1.95    0.052 .
---
Signif. codes:  0 '***' 0.001 '**' 0.01 '*' 0.05 '.' 0.1 ' ' 1

Number of observations: total=8878, ID=1619
Random effect variance(s):
Group=ID
            Variance StdDev
(Intercept)   0.4165 0.6454

Negative binomial dispersion parameter: 1.0964 (std. err.: 0.018819)

Log-likelihood: -17086.9

anova(main.M.combined.drop.parac, main.M.combined.drop.Gender)

Analysis of Deviance Table

Model 1: neg.b.MMSE ~ M + AGE + APOE4 + edu.cat + diagn + arthrit + aspirin +
Ibu
Model 2: neg.b.MMSE ~ M + AGE + APOE4 + Gender + edu.cat + diagn + arthrit +
aspirin + Ibu
  NoPar LogLik Df Deviance Pr(>Chi)
1    16 -17087
2    17 -17086  1        2   0.1573
```

7.10.6. Arthritis

Dropping arthritis from the model did signficantly worsen the model

```
main.M.combined.drop.arthritis<- glmmadmb(neg.b.MMSE~M+AGE+ APOE4+edu.cat +
diagn+  aspirin+Ibu+ (1|ID), family="nbinom1", data=MMSEdata)
summary(main.M.combined.drop.arthritis)


Call:
glmmadmb(formula = neg.b.MMSE ~ M + AGE + APOE4 + edu.cat + diagn +
    aspirin + Ibu + (1 | ID), data = MMSEdata, family = "nbinom1")

AIC: 34208.2

Coefficients:
                 Estimate Std. Error z value Pr(>|z|)
(Intercept)      -0.65402    0.05477  -11.94  < 2e-16 ***
M                 0.01271    0.00029   43.88  < 2e-16 ***
AGE               0.01847    0.00260    7.10  1.3e-12 ***
APOE41            0.26812    0.04007    6.69  2.2e-11 ***
APOE42            0.40472    0.06238    6.49  8.7e-11 ***
edu.cat2tertiary  0.10544    0.04649    2.27    0.023 *
```

```
edu.cat3mid        0.29621    0.05146    5.76  8.6e-09 ***
edu.cat4early      0.28913    0.05422    5.33  9.7e-08 ***
diagn2EMCI         0.67134    0.05959   11.27  < 2e-16 ***
diagn3LMCI         1.35926    0.05083   26.74  < 2e-16 ***
diagn4AD           2.24004    0.05830   38.42  < 2e-16 ***
aspirin           -0.07645    0.03694   -2.07    0.039 *
Ibu               -0.10809    0.05051   -2.14    0.032 *
---
Signif. codes:  0 '***' 0.001 '**' 0.01 '*' 0.05 '.' 0.1 ' ' 1

Number of observations: total=8878, ID=1619
Random effect variance(s):
Group=ID
            Variance StdDev
(Intercept)    0.418 0.6466

Negative binomial dispersion parameter: 1.0964 (std. err.: 0.01882)

Log-likelihood: -17089.1

anova(main.M.combined.drop.Gender, main.M.combined.drop.arthritis)

Analysis of Deviance Table

Model 1: neg.b.MMSE ~ M + AGE + APOE4 + edu.cat + diagn + aspirin + Ibu
Model 2: neg.b.MMSE ~ M + AGE + APOE4 + edu.cat + diagn + arthrit + aspirin +
Ibu
  NoPar LogLik Df Deviance Pr(>Chi)
1    15 -17089
2    16 -17087  1      4.4  0.03594 *
---
Signif. codes:  0 '***' 0.001 '**' 0.01 '*' 0.05 '.' 0.1 ' ' 1
```

7.10.7. Ibuprofen

Dropping ibuprofen from the model did signficantly worsen the model

```
main.M.combined.drop.Ibu<- glmmadmb(neg.b.MMSE~M+AGE+ APOE4+edu.cat + diagn+
arthrit+  aspirin+ (1|ID), family="nbinom1", data=MMSEdata)
summary(main.M.combined.drop.Ibu)


Call:
glmmadmb(formula = neg.b.MMSE ~ M + AGE + APOE4 + edu.cat + diagn +
    arthrit + aspirin + (1 | ID), data = MMSEdata, family = "nbinom1")

AIC: 34207.6

Coefficients:
                 Estimate Std. Error z value Pr(>|z|)
(Intercept)      -0.64036    0.05589  -11.46  < 2e-16 ***
M                 0.01270    0.00029   43.86  < 2e-16 ***
AGE               0.01937    0.00261    7.43  1.1e-13 ***
APOE41            0.27249    0.04007    6.80  1.0e-11 ***
APOE42            0.40400    0.06237    6.48  9.3e-11 ***
edu.cat2tertiary  0.10734    0.04650    2.31    0.021 *
edu.cat3mid       0.30021    0.05150    5.83  5.6e-09 ***
edu.cat4early     0.29196    0.05426    5.38  7.4e-08 ***
diagn2EMCI        0.67264    0.05956   11.29  < 2e-16 ***
```

```
diagn3LMCI        1.35639    0.05086   26.67  < 2e-16 ***
diagn4AD          2.24025    0.05826   38.46  < 2e-16 ***
arthrit          -0.08581    0.03781   -2.27    0.023 *
aspirin          -0.07688    0.03693   -2.08    0.037 *
---
Signif. codes:  0 '***' 0.001 '**' 0.01 '*' 0.05 '.' 0.1 ' ' 1

Number of observations: total=8878, ID=1619
Random effect variance(s):
Group=ID
            Variance StdDev
(Intercept)   0.4177 0.6463

Negative binomial dispersion parameter: 1.0964 (std. err.: 0.018828)

Log-likelihood: -17088.8

anova(main.M.combined.drop.arthritis, main.M.combined.drop.Ibu)

Analysis of Deviance Table

Model 1: neg.b.MMSE ~ M + AGE + APOE4 + edu.cat + diagn + aspirin + Ibu
Model 2: neg.b.MMSE ~ M + AGE + APOE4 + edu.cat + diagn + arthrit + aspirin
  NoPar LogLik Df Deviance  Pr(>Chi)
1    15 -17089
2    15 -17089  0      0.6 < 2.2e-16 ***
---
Signif. codes:  0 '***' 0.001 '**' 0.01 '*' 0.05 '.' 0.1 ' ' 1
```

7.10.8. Aspirin

Dropping aspirin from the model did not signficantly worsen the model

```
main.M.combined.drop.aspirin<- glmmadmb(neg.b.MMSE~M+AGE+ APOE4+edu.cat + diagn+
arthrit+ Ibu+ aspirin+ (1|ID), family="nbinom1", data=MMSEdata)
summary(main.M.combined.drop.aspirin)


Call:
glmmadmb(formula = neg.b.MMSE ~ M + AGE + APOE4 + edu.cat + diagn +
    arthrit + Ibu + aspirin + (1 | ID), data = MMSEdata, family = "nbinom1")

AIC: 34205.8

Coefficients:
                 Estimate Std. Error z value Pr(>|z|)
(Intercept)      -0.62546    0.05632  -11.11  < 2e-16 ***
M                 0.01271    0.00029   43.90  < 2e-16 ***
AGE               0.01899    0.00261    7.27  3.5e-13 ***
APOE41            0.27082    0.04003    6.77  1.3e-11 ***
APOE42            0.40151    0.06231    6.44  1.2e-10 ***
edu.cat2tertiary  0.10977    0.04647    2.36    0.018 *
edu.cat3mid       0.30146    0.05145    5.86  4.6e-09 ***
edu.cat4early     0.29590    0.05424    5.46  4.9e-08 ***
diagn2EMCI        0.67059    0.05951   11.27  < 2e-16 ***
diagn3LMCI        1.35404    0.05082   26.65  < 2e-16 ***
diagn4AD          2.23096    0.05837   38.22  < 2e-16 ***
arthrit          -0.07919    0.03792   -2.09    0.037 *
Ibu              -0.09861    0.05064   -1.95    0.052 .
```

```
aspirin          -0.07573    0.03689   -2.05    0.040 *
---
Signif. codes:  0 '***' 0.001 '**' 0.01 '*' 0.05 '.' 0.1 ' ' 1

Number of observations: total=8878, ID=1619
Random effect variance(s):
Group=ID
            Variance StdDev
(Intercept)   0.4165 0.6454

Negative binomial dispersion parameter: 1.0964 (std. err.: 0.018819)

Log-likelihood: -17086.9

anova(main.M.combined.drop.Ibu, main.M.combined.drop.aspirin)

Analysis of Deviance Table

Model 1: neg.b.MMSE ~ M + AGE + APOE4 + edu.cat + diagn + arthrit + aspirin
Model 2: neg.b.MMSE ~ M + AGE + APOE4 + edu.cat + diagn + arthrit + Ibu +
aspirin
  NoPar LogLik Df Deviance Pr(>Chi)
1    15 -17089
2    16 -17087  1      3.8  0.05125 .
---
Signif. codes:  0 '***' 0.001 '**' 0.01 '*' 0.05 '.' 0.1 ' ' 1
```

7.11. *Building combined main effect model*

```
main.M.final<- glmmadmb(neg.b.MMSE~ M+AGE+ APOE4+edu.cat + diagn+ arthrit+ Ibu+
aspirin+ (1|ID), family="nbinom1", data=MMSEdata)
summary(main.M.final)


Call:
glmmadmb(formula = neg.b.MMSE ~ M + AGE + APOE4 + edu.cat + diagn +
    arthrit + Ibu + aspirin + (1 | ID), data = MMSEdata, family = "nbinom1")

AIC: 34205.8

Coefficients:
                 Estimate Std. Error z value Pr(>|z|)
(Intercept)      -0.62546    0.05632  -11.11  < 2e-16 ***
M                 0.01271    0.00029   43.90  < 2e-16 ***
AGE               0.01899    0.00261    7.27  3.5e-13 ***
APOE41            0.27082    0.04003    6.77  1.3e-11 ***
APOE42            0.40151    0.06231    6.44  1.2e-10 ***
edu.cat2tertiary  0.10977    0.04647    2.36    0.018 *
edu.cat3mid       0.30146    0.05145    5.86  4.6e-09 ***
edu.cat4early     0.29590    0.05424    5.46  4.9e-08 ***
diagn2EMCI        0.67059    0.05951   11.27  < 2e-16 ***
diagn3LMCI        1.35404    0.05082   26.65  < 2e-16 ***
diagn4AD          2.23096    0.05837   38.22  < 2e-16 ***
arthrit          -0.07919    0.03792   -2.09    0.037 *
Ibu              -0.09861    0.05064   -1.95    0.052 .
aspirin          -0.07573    0.03689   -2.05    0.040 *
---
Signif. codes:  0 '***' 0.001 '**' 0.01 '*' 0.05 '.' 0.1 ' ' 1
```

```
Number of observations: total=8878, ID=1619
Random effect variance(s):
Group=ID
            Variance StdDev
(Intercept)   0.4165 0.6454

Negative binomial dispersion parameter: 1.0964 (std. err.: 0.018819)

Log-likelihood: -17086.9

anova(main.M.combined,main.M.final)

Analysis of Deviance Table

Model 1: neg.b.MMSE ~ M + AGE + APOE4 + edu.cat + diagn + arthrit + Ibu +
aspirin
Model 2: neg.b.MMSE ~ M + AGE + APOE4 + Gender + edu.cat + diagn + headache +
arthrit + diclo + parac + naprox + aspirin + Ibu
  NoPar LogLik Df Deviance Pr(>Chi)
1    16 -17087
2    21 -17085  5      4.6   0.4666
```

7.12. *Removing each explanitory variable in isolation*

7.12.1. *Main effect of age at the start of the study*

```
main.M.final.drop.AGE<- glmmadmb(neg.b.MMSE~ M+ APOE4+edu.cat + diagn+ arthrit+
Ibu+ aspirin+ (1|ID), family="nbinom1", data=MMSEdata)
anova(main.M.final,main.M.final.drop.AGE)

Analysis of Deviance Table

Model 1: neg.b.MMSE ~ M + APOE4 + edu.cat + diagn + arthrit + Ibu + aspirin
Model 2: neg.b.MMSE ~ M + AGE + APOE4 + edu.cat + diagn + arthrit + Ibu +
aspirin
  NoPar LogLik Df Deviance  Pr(>Chi)
1    15 -17113
2    16 -17087  1     52.6 4.089e-13 ***
---
Signif. codes:  0 '***' 0.001 '**' 0.01 '*' 0.05 '.' 0.1 ' ' 1
```

7.12.2. *Main effect of APOE4 genotype*

```
main.M.final.drop.APOE4<- glmmadmb(neg.b.MMSE~M+AGE+ edu.cat + diagn+ arthrit+
Ibu+ aspirin+ (1|ID), family="nbinom1", data=MMSEdata)
anova(main.M.final.drop.APOE4,main.M.final)

Analysis of Deviance Table

Model 1: neg.b.MMSE ~ M + AGE + edu.cat + diagn + arthrit + Ibu + aspirin
Model 2: neg.b.MMSE ~ M + AGE + APOE4 + edu.cat + diagn + arthrit + Ibu +
aspirin
  NoPar LogLik Df Deviance  Pr(>Chi)
1    14 -17119
2    16 -17087  2     64.2 1.144e-14 ***
---
Signif. codes:  0 '***' 0.001 '**' 0.01 '*' 0.05 '.' 0.1 ' ' 1
```

7.12.3. Main effect of education level

```
main.M.final.drop.edu.cat<- glmmadmb(neg.b.MMSE~ M+AGE+ APOE4+ diagn+ arthrit+
Ibu+ aspirin+ (1|ID), family="nbinom1", data=MMSEdata)
anova(main.M.final.drop.edu.cat,main.M.final)

Analysis of Deviance Table

Model 1: neg.b.MMSE ~ M + AGE + APOE4 + diagn + arthrit + Ibu + aspirin
Model 2: neg.b.MMSE ~ M + AGE + APOE4 + edu.cat + diagn + arthrit + Ibu +
aspirin
  NoPar LogLik Df Deviance  Pr(>Chi)
1    13 -17111
2    16 -17087  3     48.2 1.931e-10 ***
---
Signif. codes:  0 '***' 0.001 '**' 0.01 '*' 0.05 '.' 0.1 ' ' 1
```

7.12.4. Main effect of initial Alzhiemer's diagnosis

```
main.M.final.drop.diagn<- glmmadmb(neg.b.MMSE~ M+AGE+ APOE4+edu.cat + arthrit+
Ibu+ aspirin+ (1|ID), family="nbinom1", data=MMSEdata)
anova(main.M.final.drop.diagn,main.M.final)

Analysis of Deviance Table

Model 1: neg.b.MMSE ~ M + AGE + APOE4 + edu.cat + arthrit + Ibu + aspirin
Model 2: neg.b.MMSE ~ M + AGE + APOE4 + edu.cat + diagn + arthrit + Ibu +
aspirin
  NoPar LogLik Df Deviance  Pr(>Chi)
1    13 -17653
2    16 -17087  3   1132.8 < 2.2e-16 ***
---
Signif. codes:  0 '***' 0.001 '**' 0.01 '*' 0.05 '.' 0.1 ' ' 1
```

7.12.5. Main effect of initial Arthritis

```
main.M.final.drop.arthrit<- glmmadmb(neg.b.MMSE~ M+AGE+ diagn+ APOE4+edu.cat +
Ibu+ aspirin+ (1|ID), family="nbinom1", data=MMSEdata)
anova(main.M.final.drop.arthrit,main.M.final)

Analysis of Deviance Table

Model 1: neg.b.MMSE ~ M + AGE + diagn + APOE4 + edu.cat + Ibu + aspirin
Model 2: neg.b.MMSE ~ M + AGE + APOE4 + edu.cat + diagn + arthrit + Ibu +
aspirin
  NoPar LogLik Df Deviance Pr(>Chi)
1    15 -17089
2    16 -17087  1      4.4  0.03594 *
---
Signif. codes:  0 '***' 0.001 '**' 0.01 '*' 0.05 '.' 0.1 ' ' 1
```

7.12.6. Main effect of initial Ibuprofen

```
main.M.final.drop.Ibu<- glmmadmb(neg.b.MMSE~ M+AGE+ diagn+ APOE4+edu.cat +
arthrit+  aspirin+ (1|ID), family="nbinom1", data=MMSEdata)
anova(main.M.final.drop.Ibu,main.M.final)

Analysis of Deviance Table
```

```
Model 1: neg.b.MMSE ~ M + AGE + diagn + APOE4 + edu.cat + arthrit + aspirin
Model 2: neg.b.MMSE ~ M + AGE + APOE4 + edu.cat + diagn + arthrit + Ibu +
aspirin
  NoPar LogLik Df Deviance Pr(>Chi)
1    15 -17089
2    16 -17087  1      3.8  0.05125 .
---
Signif. codes:  0 '***' 0.001 '**' 0.01 '*' 0.05 '.' 0.1 ' ' 1
```

7.12.7. Main effect of initial Aspirin

```
main.M.final.drop.aspirin<- glmmadmb(neg.b.MMSE~ M+AGE+ diagn+ APOE4+edu.cat +
arthrit+ Ibu+  (1|ID), family="nbinom1", data=MMSEdata)
anova(main.M.final.drop.aspirin,main.M.final)

Analysis of Deviance Table

Model 1: neg.b.MMSE ~ M + AGE + diagn + APOE4 + edu.cat + arthrit + Ibu
Model 2: neg.b.MMSE ~ M + AGE + APOE4 + edu.cat + diagn + arthrit + Ibu +
aspirin
  NoPar LogLik Df Deviance Pr(>Chi)
1    15 -17089
2    16 -17087  1      4.2  0.04042 *
---
Signif. codes:  0 '***' 0.001 '**' 0.01 '*' 0.05 '.' 0.1 ' ' 1
```

7.12.8. AIC summary of main effect models

```
AIC(main.M.final,main.M.final.drop.AGE,main.M.final.drop.APOE4,main.M.final.drop
.edu.cat,main.M.final.drop.diagn,main.M.final.drop.arthrit,main.M.final.drop.Ibu
,main.M.final.drop.aspirin)

                          df     AIC
main.M.final              16 34205.8
main.M.final.drop.AGE     15 34256.4
main.M.final.drop.APOE4   14 34266.0
main.M.final.drop.edu.cat 13 34248.0
main.M.final.drop.diagn   13 35332.6
main.M.final.drop.arthrit 15 34208.2
main.M.final.drop.Ibu     15 34207.6
main.M.final.drop.aspirin 15 34208.0
```

7.13. *Investigating interaction terms*

7.13.1. The effects of diagnosis on cognitive decline progression

```
neg.m.interaction.diagn<- glmmadmb(neg.b.MMSE~ M+AGE+ diagn+ APOE4+edu.cat +
arthrit+  aspirin+ Ibu+ diagn*M+(1|ID), family="nbinom1", data=MMSEdata)
summary(neg.m.interaction.diagn)
anova(neg.m.interaction.diagn,main.M.final)
```

7.13.2. The effects of smoking on cognitive decline progression

```
neg.m.interaction.smoke<- glmmadmb(neg.b.MMSE~ M+AGE+ diagn+ APOE4+edu.cat +
arthrit+  aspirin+ Ibu+ smoke*M+(1|ID), family="nbinom1", data=MMSEdata)
summary(neg.m.interaction.smoke)
anova(neg.m.interaction.smoke,main.M.final)
```

7.13.3. The effects of arthritis on cognitive decline progression

```
neg.m.interaction.arthrit<- glmmadmb(neg.b.MMSE~ M+AGE+ diagn+ APOE4+edu.cat +
arthrit+  aspirin+ Ibu+ arthrit*M+(1|ID), family="nbinom1", data=MMSEdata)
summary(neg.m.interaction.arthrit)
anova(neg.m.interaction.arthrit, main.M.final)
```

7.13.4. The effects of cardiovascular disease on cognitive decline progression

```
neg.m.interaction.vasc<- glmmadmb(neg.b.MMSE~ M+AGE+ diagn+ APOE4+edu.cat +
arthrit+  aspirin+ Ibu+ vasc*M+(1|ID), family="nbinom1", data=MMSEdata)
summary(neg.m.interaction.vasc)
anova(neg.m.interaction.vasc, main.M.final)
```

7.13.5. The effect of headaches on cognitive decline progression

```
neg.m.interaction.headache<- glmmadmb(neg.b.MMSE~ M+AGE+ diagn+ APOE4+edu.cat +
arthrit+  aspirin+ Ibu+ headache*M+(1|ID), family="nbinom1", data=MMSEdata)
summary(neg.m.interaction.headache)


Call:
glmmadmb(formula = neg.b.MMSE ~ M + AGE + diagn + APOE4 + edu.cat +
    arthrit + aspirin + Ibu + headache * M + (1 | ID), data = MMSEdata,
    family = "nbinom1")

AIC: 34207.2

Coefficients:
                  Estimate Std. Error z value Pr(>|z|)
(Intercept)      -0.622894   0.056624  -11.00  < 2e-16 ***
M                 0.012863   0.000311   41.34  < 2e-16 ***
AGE               0.018671   0.002631    7.10  1.3e-12 ***
diagn2EMCI        0.669791   0.059487   11.26  < 2e-16 ***
diagn3LMCI        1.354534   0.050799   26.66  < 2e-16 ***
diagn4AD          2.231267   0.058354   38.24  < 2e-16 ***
APOE41            0.269515   0.040043    6.73  1.7e-11 ***
APOE42            0.399837   0.062294    6.42  1.4e-10 ***
edu.cat2tertiary  0.108453   0.046464    2.33    0.020 *
edu.cat3mid       0.302485   0.051435    5.88  4.1e-09 ***
edu.cat4early     0.296776   0.054227    5.47  4.4e-08 ***
arthrit          -0.076333   0.038034   -2.01    0.045 *
aspirin          -0.076649   0.036884   -2.08    0.038 *
Ibu              -0.097742   0.050634   -1.93    0.054 .
headache         -0.040218   0.070122   -0.57    0.566
M:headache       -0.001011   0.000817   -1.24    0.216
---
Signif. codes:  0 '***' 0.001 '**' 0.01 '*' 0.05 '.' 0.1 ' ' 1

Number of observations: total=8878, ID=1619
Random effect variance(s):
Group=ID
            Variance StdDev
(Intercept)   0.4162 0.6451

Negative binomial dispersion parameter: 1.0963 (std. err.: 0.018817)

Log-likelihood: -17085.6
```

```
anova(neg.m.interaction.headache, main.M.final)

Analysis of Deviance Table

Model 1: neg.b.MMSE ~ M + AGE + APOE4 + edu.cat + diagn + arthrit + Ibu +
aspirin
Model 2: neg.b.MMSE ~ M + AGE + diagn + APOE4 + edu.cat + arthrit + aspirin +
Ibu + headache * M
  NoPar LogLik Df Deviance Pr(>Chi)
1    16 -17087
2    18 -17086  2      2.6   0.2725
```

7.13.6. The effect of diabetes on cognitive decline progression

```
neg.m.interaction.diabetes<- glmmadmb(neg.b.MMSE~ AGE + APOE4  + M + edu.cat +
diab*M+(1|ID), family="nbinom1", data=MMSEdata)
summary(neg.m.interaction.headache)


Call:
glmmadmb(formula = neg.b.MMSE ~ M + AGE + diagn + APOE4 + edu.cat +
    arthrit + aspirin + Ibu + headache * M + (1 | ID), data = MMSEdata,
    family = "nbinom1")

AIC: 34207.2

Coefficients:
                  Estimate Std. Error z value Pr(>|z|)
(Intercept)      -0.622894   0.056624  -11.00  < 2e-16 ***
M                 0.012863   0.000311   41.34  < 2e-16 ***
AGE               0.018671   0.002631    7.10  1.3e-12 ***
diagn2EMCI        0.669791   0.059487   11.26  < 2e-16 ***
diagn3LMCI        1.354534   0.050799   26.66  < 2e-16 ***
diagn4AD          2.231267   0.058354   38.24  < 2e-16 ***
APOE41            0.269515   0.040043    6.73  1.7e-11 ***
APOE42            0.399837   0.062294    6.42  1.4e-10 ***
edu.cat2tertiary  0.108453   0.046464    2.33    0.020 *
edu.cat3mid       0.302485   0.051435    5.88  4.1e-09 ***
edu.cat4early     0.296776   0.054227    5.47  4.4e-08 ***
arthrit          -0.076333   0.038034   -2.01    0.045 *
aspirin          -0.076649   0.036884   -2.08    0.038 *
Ibu              -0.097742   0.050634   -1.93    0.054 .
headache         -0.040218   0.070122   -0.57    0.566
M:headache       -0.001011   0.000817   -1.24    0.216
---
Signif. codes:  0 '***' 0.001 '**' 0.01 '*' 0.05 '.' 0.1 ' ' 1

Number of observations: total=8878, ID=1619
Random effect variance(s):
Group=ID
            Variance StdDev
(Intercept)   0.4162 0.6451

Negative binomial dispersion parameter: 1.0963 (std. err.: 0.018817)

Log-likelihood: -17085.6

anova(neg.m.interaction.headache, main.M.final)
```

```
Analysis of Deviance Table

Model 1: neg.b.MMSE ~ M + AGE + APOE4 + edu.cat + diagn + arthrit + Ibu +
aspirin
Model 2: neg.b.MMSE ~ M + AGE + diagn + APOE4 + edu.cat + arthrit + aspirin +
Ibu + headache * M
  NoPar LogLik Df Deviance Pr(>Chi)
1    16 -17087
2    18 -17086  2      2.6   0.2725
```

7.13.7. The effect of AGE on cognitive decline progression

```
neg.m.interaction.AGE<- glmmadmb(neg.b.MMSE~ M+AGE+ diagn+ APOE4+edu.cat +
arthrit+  aspirin+ Ibu+ AGE*M+(1|ID), family="nbinom1", data=MMSEdata)
summary(neg.m.interaction.AGE)


Call:
glmmadmb(formula = neg.b.MMSE ~ M + AGE + diagn + APOE4 + edu.cat +
    arthrit + aspirin + Ibu + AGE * M + (1 | ID), data = MMSEdata,
    family = "nbinom1")

AIC: 34206.4

Coefficients:
                  Estimate Std. Error z value Pr(>|z|)
(Intercept)      -6.25e-01   5.63e-02  -11.10  < 2e-16 ***
M                 1.27e-02   2.89e-04   43.91  < 2e-16 ***
AGE               2.01e-02   2.76e-03    7.26  3.8e-13 ***
diagn2EMCI        6.69e-01   5.95e-02   11.23  < 2e-16 ***
diagn3LMCI        1.35e+00   5.08e-02   26.61  < 2e-16 ***
diagn4AD          2.23e+00   5.84e-02   38.18  < 2e-16 ***
APOE41            2.71e-01   4.00e-02    6.76  1.4e-11 ***
APOE42            4.02e-01   6.23e-02    6.45  1.1e-10 ***
edu.cat2tertiary  1.10e-01   4.65e-02    2.37    0.018 *
edu.cat3mid       3.02e-01   5.15e-02    5.87  4.3e-09 ***
edu.cat4early     2.96e-01   5.42e-02    5.46  4.8e-08 ***
arthrit          -7.91e-02   3.79e-02   -2.09    0.037 *
aspirin          -7.51e-02   3.69e-02   -2.04    0.042 *
Ibu              -9.83e-02   5.07e-02   -1.94    0.052 .
M:AGE            -4.95e-05   4.15e-05   -1.19    0.233
---
Signif. codes:  0 '***' 0.001 '**' 0.01 '*' 0.05 '.' 0.1 ' ' 1

Number of observations: total=8878, ID=1619
Random effect variance(s):
Group=ID
            Variance StdDev
(Intercept)   0.4168 0.6456

Negative binomial dispersion parameter: 1.0958 (std. err.: 0.018799)

Log-likelihood: -17086.2

anova(neg.m.interaction.AGE, main.M.final)

Analysis of Deviance Table

Model 1: neg.b.MMSE ~ M + AGE + APOE4 + edu.cat + diagn + arthrit + Ibu +
```

```
aspirin
Model 2: neg.b.MMSE ~ M + AGE + diagn + APOE4 + edu.cat + arthrit + aspirin +
Ibu + AGE * M
  NoPar LogLik Df Deviance Pr(>Chi)
1    16 -17087
2    17 -17086  1      1.4   0.2367
```

7.13.8. The effect of APOE status on cognitive decline progression

```
neg.m.interaction.APOE4<- glmmadmb(neg.b.MMSE~ M+AGE+ diagn+ APOE4+edu.cat +
arthrit+  aspirin+ Ibu+ APOE4*M+(1|ID), family="nbinom1", data=MMSEdata)
summary(neg.m.interaction.APOE4)


Call:
glmmadmb(formula = neg.b.MMSE ~ M + AGE + diagn + APOE4 + edu.cat +
    arthrit + aspirin + Ibu + APOE4 * M + (1 | ID), data = MMSEdata,
    family = "nbinom1")

AIC: 34035.2

Coefficients:
                  Estimate Std. Error z value Pr(>|z|)
(Intercept)      -0.512972   0.056183   -9.13  < 2e-16 ***
M                 0.008965   0.000409   21.91  < 2e-16 ***
AGE               0.018754   0.002583    7.26  3.8e-13 ***
diagn2EMCI        0.645102   0.058866   10.96  < 2e-16 ***
diagn3LMCI        1.328945   0.050267   26.44  < 2e-16 ***
diagn4AD          2.220171   0.057702   38.48  < 2e-16 ***
APOE41            0.106553   0.042195    2.53   0.0116 *
APOE42            0.200645   0.064379    3.12   0.0018 **
edu.cat2tertiary  0.107548   0.045957    2.34   0.0193 *
edu.cat3mid       0.300319   0.050880    5.90  3.6e-09 ***
edu.cat4early     0.293369   0.053636    5.47  4.5e-08 ***
arthrit          -0.075767   0.037501   -2.02   0.0433 *
aspirin          -0.070526   0.036481   -1.93   0.0532 .
Ibu              -0.087739   0.050090   -1.75   0.0798 .
M:APOE41          0.006697   0.000605   11.07  < 2e-16 ***
M:APOE42          0.009104   0.000888   10.25  < 2e-16 ***
---
Signif. codes:  0 '***' 0.001 '**' 0.01 '*' 0.05 '.' 0.1 ' ' 1

Number of observations: total=8878, ID=1619
Random effect variance(s):
Group=ID
            Variance StdDev
(Intercept)   0.4071 0.6381

Negative binomial dispersion parameter: 1.0786 (std. err.: 0.018495)

Log-likelihood: -16999.6

anova(neg.m.interaction.APOE4, main.M.final)

Analysis of Deviance Table

Model 1: neg.b.MMSE ~ M + AGE + APOE4 + edu.cat + diagn + arthrit + Ibu +
aspirin
Model 2: neg.b.MMSE ~ M + AGE + diagn + APOE4 + edu.cat + arthrit + aspirin +
```

```
Ibu + APOE4 * M
  NoPar LogLik Df Deviance  Pr(>Chi)
1    16 -17087
2    18 -17000  2    174.6 < 2.2e-16 ***
---
Signif. codes:  0 '***' 0.001 '**' 0.01 '*' 0.05 '.' 0.1 ' ' 1
```

7.13.9. The effect of education status on cognitive decline progression

```
neg.m.interaction.edu.cat<- glmmadmb(neg.b.MMSE~ M+AGE+ diagn+ APOE4+edu.cat +
arthrit+  aspirin+ Ibu+ edu.cat*M+(1|ID), family="nbinom1", data=MMSEdata)
summary(neg.m.interaction.edu.cat)


Call:
glmmadmb(formula = neg.b.MMSE ~ M + AGE + diagn + APOE4 + edu.cat +
    arthrit + aspirin + Ibu + edu.cat * M + (1 | ID), data = MMSEdata,
    family = "nbinom1")

AIC: 34190.4

Coefficients:
                    Estimate Std. Error z value Pr(>|z|)
(Intercept)        -0.666744   0.057715  -11.55  < 2e-16 ***
M                   0.014305   0.000512   27.92  < 2e-16 ***
AGE                 0.019084   0.002619    7.29  3.2e-13 ***
diagn2EMCI          0.671285   0.059658   11.25  < 2e-16 ***
diagn3LMCI          1.353628   0.050977   26.55  < 2e-16 ***
diagn4AD            2.228878   0.058533   38.08  < 2e-16 ***
APOE41              0.272754   0.040149    6.79  1.1e-11 ***
APOE42              0.400370   0.062504    6.41  1.5e-10 ***
edu.cat2tertiary    0.195640   0.050160    3.90  9.6e-05 ***
edu.cat3mid         0.347488   0.055431    6.27  3.6e-10 ***
edu.cat4early       0.332884   0.057500    5.79  7.1e-09 ***
arthrit            -0.080200   0.038028   -2.11    0.035 *
aspirin            -0.077685   0.037002   -2.10    0.036 *
Ibu                -0.096494   0.050786   -1.90    0.057 .
M:edu.cat2tertiary -0.003435   0.000743   -4.62  3.8e-06 ***
M:edu.cat3mid      -0.001761   0.000788   -2.24    0.025 *
M:edu.cat4early    -0.001364   0.000800   -1.71    0.088 .
---
Signif. codes:  0 '***' 0.001 '**' 0.01 '*' 0.05 '.' 0.1 ' ' 1

Number of observations: total=8878, ID=1619
Random effect variance(s):
Group=ID
            Variance StdDev
(Intercept)   0.4198 0.6479

Negative binomial dispersion parameter: 1.0905 (std. err.: 0.018704)

Log-likelihood: -17076.2

anova(neg.m.interaction.edu.cat, main.M.final)

Analysis of Deviance Table

Model 1: neg.b.MMSE ~ M + AGE + APOE4 + edu.cat + diagn + arthrit + Ibu +
aspirin
```

```
Model 2: neg.b.MMSE ~ M + AGE + diagn + APOE4 + edu.cat + arthrit + aspirin +
Ibu + edu.cat * M
  NoPar LogLik Df Deviance  Pr(>Chi)
1    16 -17087
2    19 -17076  3     21.4 8.694e-05 ***
---
Signif. codes:  0 '***' 0.001 '**' 0.01 '*' 0.05 '.' 0.1 ' ' 1
```

7.13.10. The effect of diabetes on cognitive decline progression

```
neg.m.interaction.diab<- glmmadmb(neg.b.MMSE~ M+AGE+ diagn+ APOE4+edu.cat +
arthrit+  aspirin+ Ibu+ diab*M+(1|ID), family="nbinom1", data=MMSEdata)
summary(neg.m.interaction.diab)


Call:
glmmadmb(formula = neg.b.MMSE ~ M + AGE + diagn + APOE4 + edu.cat +
    arthrit + aspirin + Ibu + diab * M + (1 | ID), data = MMSEdata,
    family = "nbinom1")

AIC: 34206.8

Coefficients:
                  Estimate Std. Error z value Pr(>|z|)
(Intercept)      -0.630872   0.056409  -11.18  < 2e-16 ***
M                 0.012798   0.000304   42.08  < 2e-16 ***
AGE               0.019139   0.002613    7.32  2.4e-13 ***
diagn2EMCI        0.667306   0.059556   11.20  < 2e-16 ***
diagn3LMCI        1.353897   0.050812   26.65  < 2e-16 ***
diagn4AD          2.230897   0.058365   38.22  < 2e-16 ***
APOE41            0.270920   0.040030    6.77  1.3e-11 ***
APOE42            0.405501   0.062352    6.50  7.9e-11 ***
edu.cat2tertiary  0.104437   0.046588    2.24    0.025 *
edu.cat3mid       0.298771   0.051462    5.81  6.4e-09 ***
edu.cat4early     0.290395   0.054327    5.35  9.0e-08 ***
arthrit          -0.080695   0.037944   -2.13    0.033 *
aspirin          -0.078968   0.036950   -2.14    0.033 *
Ibu              -0.099308   0.050641   -1.96    0.050 *
diab              0.117440   0.067135    1.75    0.080 .
M:diab           -0.000860   0.000961   -0.90    0.371
---
Signif. codes:  0 '***' 0.001 '**' 0.01 '*' 0.05 '.' 0.1 ' ' 1

Number of observations: total=8878, ID=1619
Random effect variance(s):
Group=ID
            Variance StdDev
(Intercept)   0.4164 0.6453

Negative binomial dispersion parameter: 1.0962 (std. err.: 0.01881)

Log-likelihood: -17085.4

anova(neg.m.interaction.diab, main.M.final)

Analysis of Deviance Table

Model 1: neg.b.MMSE ~ M + AGE + APOE4 + edu.cat + diagn + arthrit + Ibu +
aspirin
```

```
Model 2: neg.b.MMSE ~ M + AGE + diagn + APOE4 + edu.cat + arthrit + aspirin +
Ibu + diab * M
  NoPar LogLik Df Deviance Pr(>Chi)
1    16 -17087
2    18 -17085  2        3   0.2231
```

7.13.11. The effect of Gender on cognitive decline progression

```
neg.m.interaction.Gender<- glmmadmb(neg.b.MMSE~ M+AGE+ diagn+ APOE4+edu.cat +
arthrit+  aspirin+ Ibu+ Gender*M+(1|ID), family="nbinom1", data=MMSEdata)
summary(neg.m.interaction.Gender)


Call:
glmmadmb(formula = neg.b.MMSE ~ M + AGE + diagn + APOE4 + edu.cat +
    arthrit + aspirin + Ibu + Gender * M + (1 | ID), data = MMSEdata,
    family = "nbinom1")

AIC: 34200

Coefficients:
                  Estimate Std. Error z value Pr(>|z|)
(Intercept)      -0.679186   0.060904  -11.15  < 2e-16 ***
M                 0.013618   0.000432   31.54  < 2e-16 ***
AGE               0.018415   0.002631    7.00  2.6e-12 ***
diagn2EMCI        0.666937   0.059570   11.20  < 2e-16 ***
diagn3LMCI        1.350372   0.050927   26.52  < 2e-16 ***
diagn4AD          2.229036   0.058386   38.18  < 2e-16 ***
APOE41            0.269898   0.040007    6.75  1.5e-11 ***
APOE42            0.398775   0.062284    6.40  1.5e-10 ***
edu.cat2tertiary  0.112503   0.046499    2.42   0.0155 *
edu.cat3mid       0.312253   0.052225    5.98  2.2e-09 ***
edu.cat4early     0.308158   0.054825    5.62  1.9e-08 ***
arthrit          -0.074219   0.038047   -1.95   0.0511 .
aspirin          -0.080611   0.037178   -2.17   0.0301 *
Ibu              -0.099996   0.050611   -1.98   0.0482 *
GenderMale        0.092803   0.040687    2.28   0.0226 *
M:GenderMale     -0.001598   0.000568   -2.81   0.0049 **
---
Signif. codes:  0 '***' 0.001 '**' 0.01 '*' 0.05 '.' 0.1 ' ' 1

Number of observations: total=8878, ID=1619
Random effect variance(s):
Group=ID
            Variance StdDev
(Intercept)   0.4159 0.6449

Negative binomial dispersion parameter: 1.0949 (std. err.: 0.018787)

Log-likelihood: -17082

anova(neg.m.interaction.Gender, main.M.final)

Analysis of Deviance Table

Model 1: neg.b.MMSE ~ M + AGE + APOE4 + edu.cat + diagn + arthrit + Ibu +
aspirin
Model 2: neg.b.MMSE ~ M + AGE + diagn + APOE4 + edu.cat + arthrit + aspirin +
Ibu + Gender * M
```

```
  NoPar LogLik Df Deviance Pr(>Chi)
1    16 -17087
2    18 -17082  2      9.8 0.007447 **
---
Signif. codes:  0 '***' 0.001 '**' 0.01 '*' 0.05 '.' 0.1 ' ' 1
```

7.13.12. The effect of aspirin on cognitive decline progression

```
neg.m.interaction.aspirin<- glmmadmb(neg.b.MMSE~ M+AGE+ diagn+ APOE4+edu.cat +
arthrit+  aspirin+ Ibu+ aspirin*M+(1|ID), family="nbinom1", data=MMSEdata)
summary(neg.m.interaction.aspirin)


Call:
glmmadmb(formula = neg.b.MMSE ~ M + AGE + diagn + APOE4 + edu.cat +
    arthrit + aspirin + Ibu + aspirin * M + (1 | ID), data = MMSEdata,
    family = "nbinom1")

AIC: 34207.8

Coefficients:
                 Estimate Std. Error z value Pr(>|z|)
(Intercept)      -6.25e-01   5.70e-02  -10.97  < 2e-16 ***
M                 1.27e-02   5.14e-04   24.70  < 2e-16 ***
AGE               1.90e-02   2.61e-03    7.27  3.5e-13 ***
diagn2EMCI        6.71e-01   5.95e-02   11.27  < 2e-16 ***
diagn3LMCI        1.35e+00   5.08e-02   26.64  < 2e-16 ***
diagn4AD          2.23e+00   5.84e-02   38.20  < 2e-16 ***
APOE41            2.71e-01   4.00e-02    6.77  1.3e-11 ***
APOE42            4.01e-01   6.23e-02    6.44  1.2e-10 ***
edu.cat2tertiary  1.10e-01   4.65e-02    2.36    0.018 *
edu.cat3mid       3.01e-01   5.15e-02    5.86  4.6e-09 ***
edu.cat4early     2.96e-01   5.42e-02    5.46  4.9e-08 ***
arthrit          -7.92e-02   3.79e-02   -2.09    0.037 *
aspirin          -7.64e-02   3.94e-02   -1.94    0.053 .
Ibu              -9.86e-02   5.06e-02   -1.95    0.051 .
M:aspirin         2.98e-05   6.12e-04    0.05    0.961
---
Signif. codes:  0 '***' 0.001 '**' 0.01 '*' 0.05 '.' 0.1 ' ' 1

Number of observations: total=8878, ID=1619
Random effect variance(s):
Group=ID
            Variance StdDev
(Intercept)   0.4165 0.6454

Negative binomial dispersion parameter: 1.0964 (std. err.: 0.01882)

Log-likelihood: -17086.9

anova(neg.m.interaction.aspirin, main.M.final)

Analysis of Deviance Table

Model 1: neg.b.MMSE ~ M + AGE + APOE4 + edu.cat + diagn + arthrit + Ibu +
aspirin
Model 2: neg.b.MMSE ~ M + AGE + diagn + APOE4 + edu.cat + arthrit + aspirin +
Ibu + aspirin * M
  NoPar LogLik Df Deviance Pr(>Chi)
```

```
1    16 -17087
2    17 -17087  1        0        1
```

7.13.13. The effect of paracetamol on cognitive decline progression

```
neg.m.interaction.parac<- glmmadmb(neg.b.MMSE~ M+AGE+ diagn+ APOE4+edu.cat +
arthrit+  aspirin+ Ibu+ parac*M+(1|ID), family="nbinom1", data=MMSEdata)
summary(neg.m.interaction.parac)


Call:
glmmadmb(formula = neg.b.MMSE ~ M + AGE + diagn + APOE4 + edu.cat +
    arthrit + aspirin + Ibu + parac * M + (1 | ID), data = MMSEdata,
    family = "nbinom1")

AIC: 34205.6

Coefficients:
                  Estimate Std. Error z value Pr(>|z|)
(Intercept)      -0.608599   0.056903  -10.70  < 2e-16 ***
M                 0.012330   0.000367   33.64  < 2e-16 ***
AGE               0.018980   0.002608    7.28  3.4e-13 ***
diagn2EMCI        0.670032   0.059424   11.28  < 2e-16 ***
diagn3LMCI        1.353505   0.050738   26.68  < 2e-16 ***
diagn4AD          2.227485   0.058308   38.20  < 2e-16 ***
APOE41            0.267892   0.040016    6.69  2.2e-11 ***
APOE42            0.398512   0.062219    6.40  1.5e-10 ***
edu.cat2tertiary  0.111113   0.046405    2.39    0.017 *
edu.cat3mid       0.303188   0.051418    5.90  3.7e-09 ***
edu.cat4early     0.294959   0.054149    5.45  5.1e-08 ***
arthrit          -0.071750   0.038318   -1.87    0.061 .
aspirin          -0.072595   0.036917   -1.97    0.049 *
Ibu              -0.094215   0.050770   -1.86    0.063 .
parac            -0.073662   0.045911   -1.60    0.109
M:parac           0.001028   0.000582    1.77    0.077 .
---
Signif. codes:  0 '***' 0.001 '**' 0.01 '*' 0.05 '.' 0.1 ' ' 1

Number of observations: total=8878, ID=1619
Random effect variance(s):
Group=ID
            Variance StdDev
(Intercept)    0.415 0.6442

Negative binomial dispersion parameter: 1.0963 (std. err.: 0.018823)

Log-likelihood: -17084.8

anova(neg.m.interaction.parac, main.M.final)

Analysis of Deviance Table

Model 1: neg.b.MMSE ~ M + AGE + APOE4 + edu.cat + diagn + arthrit + Ibu +
aspirin
Model 2: neg.b.MMSE ~ M + AGE + diagn + APOE4 + edu.cat + arthrit + aspirin +
Ibu + parac * M
  NoPar LogLik Df Deviance Pr(>Chi)
1    16 -17087
2    18 -17085  2      4.2   0.1225
```

7.13.14. The effect of diclofenac on cognitive decline progression

```
neg.m.interaction.diclo<- glmmadmb(neg.b.MMSE~ M+AGE+ diagn+ APOE4+edu.cat +
arthrit+  aspirin+ Ibu+ diclo*M+(1|ID), family="nbinom1", data=MMSEdata)
summary(neg.m.interaction.diclo)


Call:
glmmadmb(formula = neg.b.MMSE ~ M + AGE + diagn + APOE4 + edu.cat +
    arthrit + aspirin + Ibu + diclo * M + (1 | ID), data = MMSEdata,
    family = "nbinom1")

AIC: 34203.6

Coefficients:
                  Estimate Std. Error z value Pr(>|z|)
(Intercept)      -0.626274   0.056361  -11.11  < 2e-16 ***
M                 0.012841   0.000294   43.63  < 2e-16 ***
AGE               0.018991   0.002612    7.27  3.6e-13 ***
diagn2EMCI        0.668420   0.059541   11.23  < 2e-16 ***
diagn3LMCI        1.351852   0.050875   26.57  < 2e-16 ***
diagn4AD          2.229460   0.058438   38.15  < 2e-16 ***
APOE41            0.270280   0.040036    6.75  1.5e-11 ***
APOE42            0.402109   0.062312    6.45  1.1e-10 ***
edu.cat2tertiary  0.110101   0.046477    2.37    0.018 *
edu.cat3mid       0.299486   0.051475    5.82  6.0e-09 ***
edu.cat4early     0.297169   0.054250    5.48  4.3e-08 ***
arthrit          -0.075956   0.038107   -1.99    0.046 *
aspirin          -0.074011   0.036924   -2.00    0.045 *
Ibu              -0.099897   0.050649   -1.97    0.049 *
diclo            -0.002017   0.148740   -0.01    0.989
M:diclo          -0.003669   0.001586   -2.31    0.021 *
---
Signif. codes:  0 '***' 0.001 '**' 0.01 '*' 0.05 '.' 0.1 ' ' 1

Number of observations: total=8878, ID=1619
Random effect variance(s):
Group=ID
            Variance StdDev
(Intercept)   0.4167 0.6455

Negative binomial dispersion parameter: 1.0961 (std. err.: 0.018805)

Log-likelihood: -17083.8

anova(neg.m.interaction.diclo, main.M.final)

Analysis of Deviance Table

Model 1: neg.b.MMSE ~ M + AGE + APOE4 + edu.cat + diagn + arthrit + Ibu +
aspirin
Model 2: neg.b.MMSE ~ M + AGE + diagn + APOE4 + edu.cat + arthrit + aspirin +
Ibu + diclo * M
  NoPar LogLik Df Deviance Pr(>Chi)
1    16 -17087
2    18 -17084  2      6.2  0.04505 *
---
Signif. codes:  0 '***' 0.001 '**' 0.01 '*' 0.05 '.' 0.1 ' ' 1
```

7.13.15. *The effect of ibuprofen on cognitive decline progression*

```
neg.m.interaction.Ibu<- glmmadmb(neg.b.MMSE~ M+AGE+ diagn+ APOE4+edu.cat +
arthrit+  aspirin+ Ibu+ Ibu*M+(1|ID), family="nbinom1", data=MMSEdata)
summary(neg.m.interaction.Ibu)


Call:
glmmadmb(formula = neg.b.MMSE ~ M + AGE + diagn + APOE4 + edu.cat +
    arthrit + aspirin + Ibu + Ibu * M + (1 | ID), data = MMSEdata,
    family = "nbinom1")

AIC: 34206

Coefficients:
                  Estimate Std. Error z value Pr(>|z|)
(Intercept)      -0.630697   0.056465  -11.17  < 2e-16 ***
M                 0.012919   0.000326   39.63  < 2e-16 ***
AGE               0.019030   0.002612    7.28  3.2e-13 ***
diagn2EMCI        0.670231   0.059521   11.26  < 2e-16 ***
diagn3LMCI        1.354026   0.050830   26.64  < 2e-16 ***
diagn4AD          2.232670   0.058398   38.23  < 2e-16 ***
APOE41            0.270362   0.040045    6.75  1.5e-11 ***
APOE42            0.401170   0.062327    6.44  1.2e-10 ***
edu.cat2tertiary  0.110195   0.046480    2.37    0.018 *
edu.cat3mid       0.302288   0.051464    5.87  4.3e-09 ***
edu.cat4early     0.296246   0.054252    5.46  4.7e-08 ***
arthrit          -0.079526   0.037928   -2.10    0.036 *
aspirin          -0.075577   0.036900   -2.05    0.041 *
Ibu              -0.070843   0.054549   -1.30    0.194
M:Ibu            -0.000933   0.000684   -1.36    0.173
---
Signif. codes:  0 '***' 0.001 '**' 0.01 '*' 0.05 '.' 0.1 ' ' 1

Number of observations: total=8878, ID=1619
Random effect variance(s):
Group=ID
            Variance StdDev
(Intercept)   0.4168 0.6456

Negative binomial dispersion parameter: 1.0962 (std. err.: 0.01881)

Log-likelihood: -17086

anova(neg.m.interaction.Ibu, main.M.final)

Analysis of Deviance Table

Model 1: neg.b.MMSE ~ M + AGE + APOE4 + edu.cat + diagn + arthrit + Ibu +
aspirin
Model 2: neg.b.MMSE ~ M + AGE + diagn + APOE4 + edu.cat + arthrit + aspirin +
Ibu + Ibu * M
  NoPar LogLik Df Deviance Pr(>Chi)
1    16 -17087
2    17 -17086  1      1.8   0.1797
```

7.13.16. The effect of naproxin on cognitive decline progression

```
neg.m.interaction.naprox<- glmmadmb(neg.b.MMSE~ M+AGE+ diagn+ APOE4+edu.cat +
arthrit+  aspirin+ Ibu+ naprox*M+(1|ID), family="nbinom1", data=MMSEdata)
summary(neg.m.interaction.naprox)


Call:
glmmadmb(formula = neg.b.MMSE ~ M + AGE + diagn + APOE4 + edu.cat +
    arthrit + aspirin + Ibu + naprox * M + (1 | ID), data = MMSEdata,
    family = "nbinom1")

AIC: 34209.8

Coefficients:
                  Estimate Std. Error z value Pr(>|z|)
(Intercept)      -6.24e-01   5.66e-02  -11.04  < 2e-16 ***
M                 1.27e-02   3.21e-04   39.60  < 2e-16 ***
AGE               1.90e-02   2.62e-03    7.25  4.1e-13 ***
diagn2EMCI        6.71e-01   5.95e-02   11.27  < 2e-16 ***
diagn3LMCI        1.35e+00   5.08e-02   26.65  < 2e-16 ***
diagn4AD          2.23e+00   5.84e-02   38.16  < 2e-16 ***
APOE41            2.71e-01   4.00e-02    6.76  1.4e-11 ***
APOE42            4.01e-01   6.23e-02    6.44  1.2e-10 ***
edu.cat2tertiary  1.10e-01   4.65e-02    2.37    0.018 *
edu.cat3mid       3.02e-01   5.15e-02    5.86  4.6e-09 ***
edu.cat4early     2.96e-01   5.42e-02    5.46  4.8e-08 ***
arthrit          -7.85e-02   3.81e-02   -2.06    0.039 *
aspirin          -7.55e-02   3.69e-02   -2.05    0.041 *
Ibu              -9.80e-02   5.08e-02   -1.93    0.054 .
naprox           -1.21e-02   6.16e-02   -0.20    0.844
M:naprox          5.31e-05   7.24e-04    0.07    0.942
---
Signif. codes:  0 '***' 0.001 '**' 0.01 '*' 0.05 '.' 0.1 ' ' 1

Number of observations: total=8878, ID=1619
Random effect variance(s):
Group=ID
            Variance StdDev
(Intercept)   0.4165 0.6454

Negative binomial dispersion parameter: 1.0965 (std. err.: 0.01882)

Log-likelihood: -17086.9

anova(neg.m.interaction.naprox, main.M.final)

Analysis of Deviance Table

Model 1: neg.b.MMSE ~ M + AGE + APOE4 + edu.cat + diagn + arthrit + Ibu +
aspirin
Model 2: neg.b.MMSE ~ M + AGE + diagn + APOE4 + edu.cat + arthrit + aspirin +
Ibu + naprox * M
  NoPar LogLik Df Deviance Pr(>Chi)
1    16 -17087
2    18 -17087  2        0        1
```

7.13.17. *The effect of celecoxib on cognitive decline progression*

```
neg.m.interaction.celex<- glmmadmb(neg.b.MMSE~ M+AGE+ diagn+ APOE4+edu.cat +
arthrit+  aspirin+ Ibu+ celex*M+(1|ID), family="nbinom1", data=MMSEdata)
summary(neg.m.interaction.celex)


Call:
glmmadmb(formula = neg.b.MMSE ~ M + AGE + diagn + APOE4 + edu.cat +
    arthrit + aspirin + Ibu + celex * M + (1 | ID), data = MMSEdata,
    family = "nbinom1")

AIC: 34207

Coefficients:
                  Estimate Std. Error z value Pr(>|z|)
(Intercept)      -0.625735   0.056319  -11.11  < 2e-16 ***
M                 0.012803   0.000297   43.07  < 2e-16 ***
AGE               0.018867   0.002612    7.22  5.1e-13 ***
diagn2EMCI        0.669602   0.059478   11.26  < 2e-16 ***
diagn3LMCI        1.354011   0.050790   26.66  < 2e-16 ***
diagn4AD          2.229325   0.058368   38.19  < 2e-16 ***
APOE41            0.270877   0.040009    6.77  1.3e-11 ***
APOE42            0.401966   0.062275    6.45  1.1e-10 ***
edu.cat2tertiary  0.109332   0.046445    2.35    0.019 *
edu.cat3mid       0.301778   0.051418    5.87  4.4e-09 ***
edu.cat4early     0.298141   0.054238    5.50  3.9e-08 ***
arthrit          -0.072957   0.038294   -1.91    0.057 .
aspirin          -0.074538   0.036909   -2.02    0.043 *
Ibu              -0.102492   0.050722   -2.02    0.043 *
celex            -0.055192   0.102170   -0.54    0.589
M:celex          -0.001603   0.001258   -1.27    0.203
---
Signif. codes:  0 '***' 0.001 '**' 0.01 '*' 0.05 '.' 0.1 ' ' 1

Number of observations: total=8878, ID=1619
Random effect variance(s):
Group=ID
            Variance StdDev
(Intercept)    0.416  0.645

Negative binomial dispersion parameter: 1.0961 (std. err.: 0.018814)

Log-likelihood: -17085.5

anova(neg.m.interaction.celex, main.M.final)

Analysis of Deviance Table

Model 1: neg.b.MMSE ~ M + AGE + APOE4 + edu.cat + diagn + arthrit + Ibu +
aspirin
Model 2: neg.b.MMSE ~ M + AGE + diagn + APOE4 + edu.cat + arthrit + aspirin +
Ibu + celex * M
  NoPar LogLik Df Deviance Pr(>Chi)
1    16 -17087
2    18 -17086  2      2.8   0.2466
```

7.14. *Combined interaction model*

```
neg.m.combined<- glmmadmb(neg.b.MMSE~ M+AGE+ diagn+ APOE4+edu.cat + arthrit+
aspirin+ Ibu+ diagn*M +smoke*M +APOE4*M + Gender*M+ edu.cat*M +parac*M
+diclo*M+(1|ID), family="nbinom1", data=MMSEdata)
summary(neg.m.combined)


Call:
glmmadmb(formula = neg.b.MMSE ~ M + AGE + diagn + APOE4 + edu.cat +
    arthrit + aspirin + Ibu + diagn * M + smoke * M + APOE4 *
    M + Gender * M + edu.cat * M + parac * M + diclo * M + (1 |
    ID), data = MMSEdata, family = "nbinom1")

AIC: 33938

Coefficients:
                    Estimate Std. Error z value Pr(>|z|)
(Intercept)        -0.558108   0.065338   -8.54  < 2e-16 ***
M                   0.010455   0.000882   11.85  < 2e-16 ***
AGE                 0.018189   0.002613    6.96  3.4e-12 ***
diagn2EMCI          0.708477   0.065683   10.79  < 2e-16 ***
diagn3LMCI          1.237416   0.055474   22.31  < 2e-16 ***
diagn4AD            2.092022   0.062269   33.60  < 2e-16 ***
APOE41              0.122878   0.042414    2.90  0.00377 **
APOE42              0.224098   0.064831    3.46  0.00055 ***
edu.cat2tertiary    0.203593   0.049595    4.11  4.0e-05 ***
edu.cat3mid         0.363400   0.055678    6.53  6.7e-11 ***
edu.cat4early       0.359314   0.057532    6.25  4.2e-10 ***
arthrit            -0.061424   0.038388   -1.60  0.10958
aspirin            -0.073283   0.037104   -1.98  0.04826 *
Ibu                -0.082645   0.050465   -1.64  0.10149
smoke               0.023716   0.044411    0.53  0.59334
GenderMale          0.089032   0.040831    2.18  0.02922 *
parac              -0.075116   0.045679   -1.64  0.10009
diclo               0.045664   0.146630    0.31  0.75548
M:diagn2EMCI       -0.003342   0.001044   -3.20  0.00137 **
M:diagn3LMCI        0.002840   0.000726    3.91  9.2e-05 ***
M:diagn4AD          0.008767   0.001438    6.10  1.1e-09 ***
M:smoke            -0.000279   0.000643   -0.43  0.66454
M:APOE41            0.005847   0.000618    9.46  < 2e-16 ***
M:APOE42            0.007730   0.000932    8.29  < 2e-16 ***
M:GenderMale       -0.001707   0.000593   -2.88  0.00397 **
M:edu.cat2tertiary -0.003734   0.000743   -5.03  5.0e-07 ***
M:edu.cat3mid      -0.002220   0.000814   -2.73  0.00638 **
M:edu.cat4early    -0.002458   0.000819   -3.00  0.00269 **
M:parac             0.001256   0.000591    2.13  0.03349 *
M:diclo            -0.004743   0.001618   -2.93  0.00337 **
---
Signif. codes:  0 '***' 0.001 '**' 0.01 '*' 0.05 '.' 0.1 ' ' 1

Number of observations: total=8878, ID=1619
Random effect variance(s):
Group=ID
            Variance StdDev
(Intercept)   0.4117 0.6416

Negative binomial dispersion parameter: 1.0582 (std. err.: 0.018199)

Log-likelihood: -16937
```

```
anova(neg.m.combined, main.M.final)

Analysis of Deviance Table

Model 1: neg.b.MMSE ~ M + AGE + APOE4 + edu.cat + diagn + arthrit + Ibu +
aspirin
Model 2: neg.b.MMSE ~ M + AGE + diagn + APOE4 + edu.cat + arthrit + aspirin +
Ibu + diagn * M + smoke * M + APOE4 * M + Gender * M + edu.cat * M + parac * M +
diclo * M
  NoPar LogLik Df Deviance  Pr(>Chi)
1    16 -17087
2    32 -16937 16    299.8 < 2.2e-16 ***
---
Signif. codes:  0 '***' 0.001 '**' 0.01 '*' 0.05 '.' 0.1 ' ' 1
```

7.14.1. Dropping non-significant terms

```
neg.m.combined.drop.smoke<- glmmadmb(neg.b.MMSE~ M+AGE+ diagn+ APOE4+edu.cat +
arthrit+  aspirin+ Ibu+ diagn*M +APOE4*M + Gender*M+ edu.cat*M +parac*M
+diclo*M+(1|ID), family="nbinom1", data=MMSEdata)
summary(neg.m.combined.drop.smoke)


Call:
glmmadmb(formula = neg.b.MMSE ~ M + AGE + diagn + APOE4 + edu.cat +
    arthrit + aspirin + Ibu + diagn * M + APOE4 * M + Gender *
    M + edu.cat * M + parac * M + diclo * M + (1 | ID), data = MMSEdata,
    family = "nbinom1")

AIC: 33934.4

Coefficients:
                    Estimate Std. Error z value Pr(>|z|)
(Intercept)        -0.553420   0.064793   -8.54  < 2e-16 ***
M                   0.010381   0.000863   12.03  < 2e-16 ***
AGE                 0.018181   0.002612    6.96  3.4e-12 ***
diagn2EMCI          0.707268   0.065640   10.77  < 2e-16 ***
diagn3LMCI          1.237085   0.055466   22.30  < 2e-16 ***
diagn4AD            2.091430   0.062254   33.60  < 2e-16 ***
APOE41              0.122679   0.042405    2.89  0.00382 **
APOE42              0.222583   0.064771    3.44  0.00059 ***
edu.cat2tertiary    0.204928   0.049523    4.14  3.5e-05 ***
edu.cat3mid         0.365119   0.055585    6.57  5.1e-11 ***
edu.cat4early       0.360400   0.057491    6.27  3.6e-10 ***
arthrit            -0.060793   0.038355   -1.59  0.11296
aspirin            -0.074093   0.037041   -2.00  0.04547 *
Ibu                -0.082096   0.050448   -1.63  0.10366
GenderMale          0.091510   0.040530    2.26  0.02396 *
parac              -0.075242   0.045658   -1.65  0.09936 .
diclo               0.045378   0.146620    0.31  0.75694
M:diagn2EMCI       -0.003326   0.001043   -3.19  0.00143 **
M:diagn3LMCI        0.002854   0.000725    3.94  8.3e-05 ***
M:diagn4AD          0.008777   0.001437    6.11  1.0e-09 ***
M:APOE41            0.005858   0.000617    9.49  < 2e-16 ***
M:APOE42            0.007754   0.000931    8.33  < 2e-16 ***
M:GenderMale       -0.001726   0.000591   -2.92  0.00351 **
M:edu.cat2tertiary -0.003746   0.000743   -5.04  4.5e-07 ***
M:edu.cat3mid      -0.002245   0.000812   -2.77  0.00569 **
M:edu.cat4early    -0.002476   0.000818   -3.03  0.00247 **
```

```
M:parac             0.001272   0.000589    2.16  0.03094 *
M:diclo            -0.004723   0.001617   -2.92  0.00349 **
---
Signif. codes:  0 '***' 0.001 '**' 0.01 '*' 0.05 '.' 0.1 ' ' 1

Number of observations: total=8878, ID=1619
Random effect variance(s):
Group=ID
            Variance StdDev
(Intercept)   0.4116 0.6415

Negative binomial dispersion parameter: 1.0584 (std. err.: 0.018198)

Log-likelihood: -16937.2

anova(neg.m.combined, neg.m.combined.drop.smoke)

Analysis of Deviance Table

Model 1: neg.b.MMSE ~ M + AGE + diagn + APOE4 + edu.cat + arthrit + aspirin +
Ibu + diagn * M + APOE4 * M + Gender * M + edu.cat * M + parac * M + diclo * M
Model 2: neg.b.MMSE ~ M + AGE + diagn + APOE4 + edu.cat + arthrit + aspirin +
Ibu + diagn * M + smoke * M + APOE4 * M + Gender * M + edu.cat * M + parac * M +
diclo * M
  NoPar LogLik Df Deviance Pr(>Chi)
1    30 -16937
2    32 -16937  2      0.4   0.8187

neg.m.combined.drop.Ibu<- glmmadmb(neg.b.MMSE~ M+AGE+ diagn+ APOE4+edu.cat +
arthrit+  aspirin+ diagn*M +APOE4*M + Gender*M+ edu.cat*M +parac*M
+diclo*M+(1|ID), family="nbinom1", data=MMSEdata)
summary(neg.m.combined.drop.Ibu)


Call:
glmmadmb(formula = neg.b.MMSE ~ M + AGE + diagn + APOE4 + edu.cat +
    arthrit + aspirin + diagn * M + APOE4 * M + Gender * M +
    edu.cat * M + parac * M + diclo * M + (1 | ID), data = MMSEdata,
    family = "nbinom1")

AIC: 33935

Coefficients:
                    Estimate Std. Error z value Pr(>|z|)
(Intercept)        -0.564023   0.064533   -8.74  < 2e-16 ***
M                   0.010365   0.000863   12.01  < 2e-16 ***
AGE                 0.018504   0.002607    7.10  1.3e-12 ***
diagn2EMCI          0.708667   0.065673   10.79  < 2e-16 ***
diagn3LMCI          1.238780   0.055492   22.32  < 2e-16 ***
diagn4AD            2.098663   0.062148   33.77  < 2e-16 ***
APOE41              0.123504   0.042433    2.91  0.00361 **
APOE42              0.224260   0.064815    3.46  0.00054 ***
edu.cat2tertiary    0.203265   0.049544    4.10  4.1e-05 ***
edu.cat3mid         0.364597   0.055621    6.56  5.6e-11 ***
edu.cat4early       0.357117   0.057495    6.21  5.3e-10 ***
arthrit            -0.065485   0.038277   -1.71  0.08712 .
aspirin            -0.074571   0.037070   -2.01  0.04426 *
GenderMale          0.090649   0.040554    2.24  0.02540 *
parac              -0.081469   0.045525   -1.79  0.07353 .
diclo               0.045215   0.146720    0.31  0.75795
```

```
M:diagn2EMCI       -0.003320   0.001043   -3.18  0.00146 **
M:diagn3LMCI        0.002863   0.000725    3.95  7.9e-05 ***
M:diagn4AD          0.008787   0.001437    6.11  9.8e-10 ***
M:APOE41            0.005871   0.000617    9.51  < 2e-16 ***
M:APOE42            0.007765   0.000931    8.34  < 2e-16 ***
M:GenderMale       -0.001722   0.000591   -2.91  0.00360 **
M:edu.cat2tertiary -0.003755   0.000743   -5.06  4.3e-07 ***
M:edu.cat3mid      -0.002258   0.000812   -2.78  0.00541 **
M:edu.cat4early    -0.002485   0.000818   -3.04  0.00238 **
M:parac             0.001267   0.000589    2.15  0.03153 *
M:diclo            -0.004698   0.001617   -2.91  0.00367 **
---
Signif. codes:  0 '***' 0.001 '**' 0.01 '*' 0.05 '.' 0.1 ' ' 1

Number of observations: total=8878, ID=1619
Random effect variance(s):
Group=ID
            Variance StdDev
(Intercept)   0.4123 0.6421

Negative binomial dispersion parameter: 1.0584 (std. err.: 0.018203)

Log-likelihood: -16938.5

anova(neg.m.combined.drop.smoke, neg.m.combined.drop.Ibu)

Analysis of Deviance Table

Model 1: neg.b.MMSE ~ M + AGE + diagn + APOE4 + edu.cat + arthrit + aspirin +
diagn * M + APOE4 * M + Gender * M + edu.cat * M + parac * M + diclo * M
Model 2: neg.b.MMSE ~ M + AGE + diagn + APOE4 + edu.cat + arthrit + aspirin +
Ibu + diagn * M + APOE4 * M + Gender * M + edu.cat * M + parac * M + diclo * M
  NoPar LogLik Df Deviance Pr(>Chi)
1    29 -16939
2    30 -16937  1      2.6   0.1069

neg.m.combined.drop.arthrit<- glmmadmb(neg.b.MMSE~ M+AGE+ diagn+ APOE4+edu.cat +
aspirin+ diagn*M +APOE4*M + Gender*M+ edu.cat*M +parac*M +diclo*M+(1|ID),
family="nbinom1", data=MMSEdata)
summary(neg.m.combined.drop.Ibu)


Call:
glmmadmb(formula = neg.b.MMSE ~ M + AGE + diagn + APOE4 + edu.cat +
    arthrit + aspirin + diagn * M + APOE4 * M + Gender * M +
    edu.cat * M + parac * M + diclo * M + (1 | ID), data = MMSEdata,
    family = "nbinom1")

AIC: 33935

Coefficients:
                    Estimate Std. Error z value Pr(>|z|)
(Intercept)        -0.564023   0.064533   -8.74  < 2e-16 ***
M                   0.010365   0.000863   12.01  < 2e-16 ***
AGE                 0.018504   0.002607    7.10  1.3e-12 ***
diagn2EMCI          0.708667   0.065673   10.79  < 2e-16 ***
diagn3LMCI          1.238780   0.055492   22.32  < 2e-16 ***
diagn4AD            2.098663   0.062148   33.77  < 2e-16 ***
APOE41              0.123504   0.042433    2.91  0.00361 **
APOE42              0.224260   0.064815    3.46  0.00054 ***
```

```
edu.cat2tertiary    0.203265   0.049544    4.10  4.1e-05 ***
edu.cat3mid         0.364597   0.055621    6.56  5.6e-11 ***
edu.cat4early       0.357117   0.057495    6.21  5.3e-10 ***
arthrit            -0.065485   0.038277   -1.71  0.08712 .
aspirin            -0.074571   0.037070   -2.01  0.04426 *
GenderMale          0.090649   0.040554    2.24  0.02540 *
parac              -0.081469   0.045525   -1.79  0.07353 .
diclo               0.045215   0.146720    0.31  0.75795
M:diagn2EMCI       -0.003320   0.001043   -3.18  0.00146 **
M:diagn3LMCI        0.002863   0.000725    3.95  7.9e-05 ***
M:diagn4AD          0.008787   0.001437    6.11  9.8e-10 ***
M:APOE41            0.005871   0.000617    9.51  < 2e-16 ***
M:APOE42            0.007765   0.000931    8.34  < 2e-16 ***
M:GenderMale       -0.001722   0.000591   -2.91  0.00360 **
M:edu.cat2tertiary -0.003755   0.000743   -5.06  4.3e-07 ***
M:edu.cat3mid      -0.002258   0.000812   -2.78  0.00541 **
M:edu.cat4early    -0.002485   0.000818   -3.04  0.00238 **
M:parac             0.001267   0.000589    2.15  0.03153 *
M:diclo            -0.004698   0.001617   -2.91  0.00367 **
---
Signif. codes:  0 '***' 0.001 '**' 0.01 '*' 0.05 '.' 0.1 ' ' 1

Number of observations: total=8878, ID=1619
Random effect variance(s):
Group=ID
            Variance StdDev
(Intercept)   0.4123 0.6421

Negative binomial dispersion parameter: 1.0584 (std. err.: 0.018203)

Log-likelihood: -16938.5

anova(neg.m.combined.drop.arthrit, neg.m.combined.drop.Ibu)

Analysis of Deviance Table

Model 1: neg.b.MMSE ~ M + AGE + diagn + APOE4 + edu.cat + aspirin + diagn * M +
APOE4 * M + Gender * M + edu.cat * M + parac * M + diclo * M
Model 2: neg.b.MMSE ~ M + AGE + diagn + APOE4 + edu.cat + arthrit + aspirin +
diagn * M + APOE4 * M + Gender * M + edu.cat * M + parac * M + diclo * M
  NoPar LogLik Df Deviance Pr(>Chi)
1    28 -16940
2    29 -16939  1        3  0.08326 .
---
Signif. codes:  0 '***' 0.001 '**' 0.01 '*' 0.05 '.' 0.1 ' ' 1
```

7.15. *Final full model and plots of the coeffecients*

The full model of all significant interaction terms is now created and non-significant terms will be removed.

```
neg.mmse<- glmmadmb(neg.b.MMSE~ M+AGE+ diagn+ APOE4+edu.cat + aspirin+ diagn*M
+APOE4*M + Gender*M+ edu.cat*M +parac*M +diclo*M+(1|ID), family="nbinom1",
data=MMSEdata)

summary(neg.mmse)


Call:
```

```
glmmadmb(formula = neg.b.MMSE ~ M + AGE + diagn + APOE4 + edu.cat +
    aspirin + diagn * M + APOE4 * M + Gender * M + edu.cat *
    M + parac * M + diclo * M + (1 | ID), data = MMSEdata, family = "nbinom1")

AIC: 33936

Coefficients:
                    Estimate Std. Error z value Pr(>|z|)
(Intercept)        -0.588327   0.063059   -9.33  < 2e-16 ***
M                   0.010356   0.000863   12.00  < 2e-16 ***
AGE                 0.018083   0.002598    6.96  3.4e-12 ***
diagn2EMCI          0.708348   0.065722   10.78  < 2e-16 ***
diagn3LMCI          1.242150   0.055504   22.38  < 2e-16 ***
diagn4AD            2.105171   0.062093   33.90  < 2e-16 ***
APOE41              0.120675   0.042440    2.84  0.00446 **
APOE42              0.226424   0.064864    3.49  0.00048 ***
edu.cat2tertiary    0.200328   0.049559    4.04  5.3e-05 ***
edu.cat3mid         0.361968   0.055648    6.50  7.8e-11 ***
edu.cat4early       0.352873   0.057489    6.14  8.4e-10 ***
aspirin            -0.074933   0.037108   -2.02  0.04345 *
GenderMale          0.095723   0.040487    2.36  0.01806 *
parac              -0.093084   0.045053   -2.07  0.03882 *
diclo               0.022617   0.146270    0.15  0.87712
M:diagn2EMCI       -0.003321   0.001043   -3.18  0.00145 **
M:diagn3LMCI        0.002862   0.000725    3.95  7.9e-05 ***
M:diagn4AD          0.008798   0.001437    6.12  9.3e-10 ***
M:APOE41            0.005879   0.000617    9.52  < 2e-16 ***
M:APOE42            0.007765   0.000931    8.34  < 2e-16 ***
M:GenderMale       -0.001724   0.000591   -2.92  0.00355 **
M:edu.cat2tertiary -0.003752   0.000743   -5.05  4.3e-07 ***
M:edu.cat3mid      -0.002262   0.000812   -2.79  0.00532 **
M:edu.cat4early    -0.002500   0.000818   -3.06  0.00224 **
M:parac             0.001285   0.000589    2.18  0.02928 *
M:diclo            -0.004681   0.001617   -2.89  0.00380 **
---
Signif. codes:  0 '***' 0.001 '**' 0.01 '*' 0.05 '.' 0.1 ' ' 1

Number of observations: total=8878, ID=1619
Random effect variance(s):
Group=ID
            Variance StdDev
(Intercept)   0.4134  0.643

Negative binomial dispersion parameter: 1.0582 (std. err.: 0.018199)

Log-likelihood: -16940

anova(neg.m.combined, neg.mmse)

Analysis of Deviance Table

Model 1: neg.b.MMSE ~ M + AGE + diagn + APOE4 + edu.cat + aspirin + diagn * M +
APOE4 * M + Gender * M + edu.cat * M + parac * M + diclo * M
Model 2: neg.b.MMSE ~ M + AGE + diagn + APOE4 + edu.cat + arthrit + aspirin +
Ibu + diagn * M + smoke * M + APOE4 * M + Gender * M + edu.cat * M + parac * M +
diclo * M
  NoPar LogLik Df Deviance Pr(>Chi)
1    28 -16940
2    32 -16937  4        6   0.1991
```

7.15.1. Coeffecient plot

```
coefplot(neg.mmse)
```

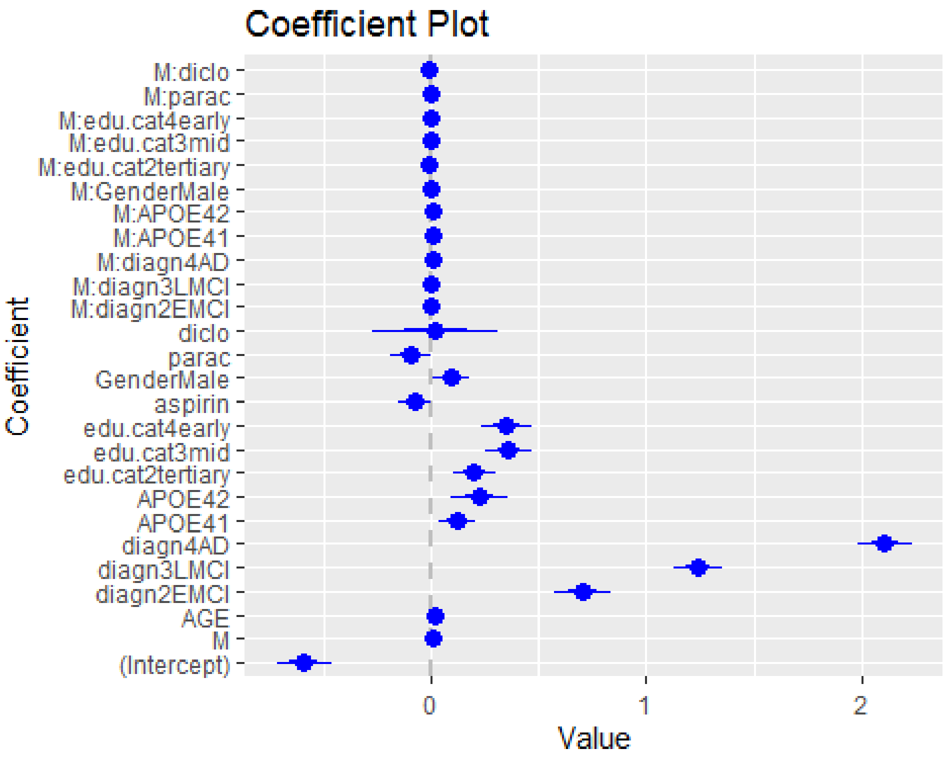


```
str(MMSEdata$APOE4)

 Factor w/ 3 levels "0","1","2": 1 2 2 2 2 1 1 1 1 1 ...

neg.mmse.APOEnumeric<- glmmadmb(neg.b.MMSE~ M+AGE+ diagn+
as.numeric(APOE4)+edu.cat + aspirin+ diagn*M +as.numeric(APOE4)*M + Gender*M+
edu.cat*M +parac*M +diclo*M+(1|ID), family="nbinom1", data=MMSEdata)

summary(neg.mmse.APOEnumeric)


Call:
glmmadmb(formula = neg.b.MMSE ~ M + AGE + diagn + as.numeric(APOE4) +
    edu.cat + aspirin + diagn * M + as.numeric(APOE4) * M + Gender *
    M + edu.cat * M + parac * M + diclo * M + (1 | ID), data = MMSEdata,
    family = "nbinom1")

AIC: 33943.2

Coefficients:
                     Estimate Std. Error z value Pr(>|z|)
(Intercept)         -0.709461   0.072662   -9.76  < 2e-16 ***
M                    0.006382   0.000999    6.39  1.7e-10 ***
AGE                  0.018248   0.002593    7.04  2.0e-12 ***
diagn2EMCI           0.710113   0.065688   10.81  < 2e-16 ***
diagn3LMCI           1.243616   0.055465   22.42  < 2e-16 ***
diagn4AD             2.105614   0.062079   33.92  < 2e-16 ***
as.numeric(APOE4)    0.118605   0.029612    4.01  6.2e-05 ***
edu.cat2tertiary     0.199296   0.049567    4.02  5.8e-05 ***
edu.cat3mid          0.363931   0.055656    6.54  6.2e-11 ***
```

```
edu.cat4early        0.352677   0.057495    6.13  8.6e-10 ***
aspirin             -0.077025   0.037098   -2.08   0.0379 *
GenderMale           0.097695   0.040486    2.41   0.0158 *
parac               -0.089862   0.045026   -2.00   0.0460 *
diclo                0.037217   0.146120    0.25   0.7990
M:diagn2EMCI        -0.003361   0.001044   -3.22   0.0013 **
M:diagn3LMCI         0.002849   0.000725    3.93  8.5e-05 ***
M:diagn4AD           0.008748   0.001439    6.08  1.2e-09 ***
M:as.numeric(APOE4)  0.004496   0.000426   10.56  < 2e-16 ***
M:GenderMale        -0.001836   0.000591   -3.11   0.0019 **
M:edu.cat2tertiary  -0.003677   0.000743   -4.95  7.4e-07 ***
M:edu.cat3mid       -0.002349   0.000812   -2.89   0.0038 **
M:edu.cat4early     -0.002420   0.000818   -2.96   0.0031 **
M:parac              0.001115   0.000587    1.90   0.0576 .
M:diclo             -0.005241   0.001614   -3.25   0.0012 **
---
Signif. codes:  0 '***' 0.001 '**' 0.01 '*' 0.05 '.' 0.1 ' ' 1

Number of observations: total=8878, ID=1619
Random effect variance(s):
Group=ID
            Variance StdDev
(Intercept)   0.4134  0.643

Negative binomial dispersion parameter: 1.0599 (std. err.: 0.018261)

Log-likelihood: -16945.6

anova(neg.mmse.APOEnumeric, neg.mmse)

Analysis of Deviance Table

Model 1: neg.b.MMSE ~ M + AGE + diagn + as.numeric(APOE4) + edu.cat + aspirin +
diagn * M + as.numeric(APOE4) * M + Gender * M + edu.cat * M + parac * M + diclo
* M
Model 2: neg.b.MMSE ~ M + AGE + diagn + APOE4 + edu.cat + aspirin + diagn * M +
APOE4 * M + Gender * M + edu.cat * M + parac * M + diclo * M
  NoPar LogLik Df Deviance Pr(>Chi)
1    26 -16946
2    28 -16940  2     11.2 0.003698 **
---
Signif. codes:  0 '***' 0.001 '**' 0.01 '*' 0.05 '.' 0.1 ' ' 1
```

7.15.2. Coeffecient plot of interaction terms

```
coefficients<-
c("M:diclo","M:parac","M:APOE41","M:APOE42","M:edu.cat2tertiary","M:edu.cat3mid"
,"M:edu.cat4early","M:diagn2EMCI","M:diagn3LMCI","M:diagn4AD","M:GenderMale","M"
)
coefplot(neg.mmse,coefficients=coefficients,main="Interaction terms
coeffecients")
```

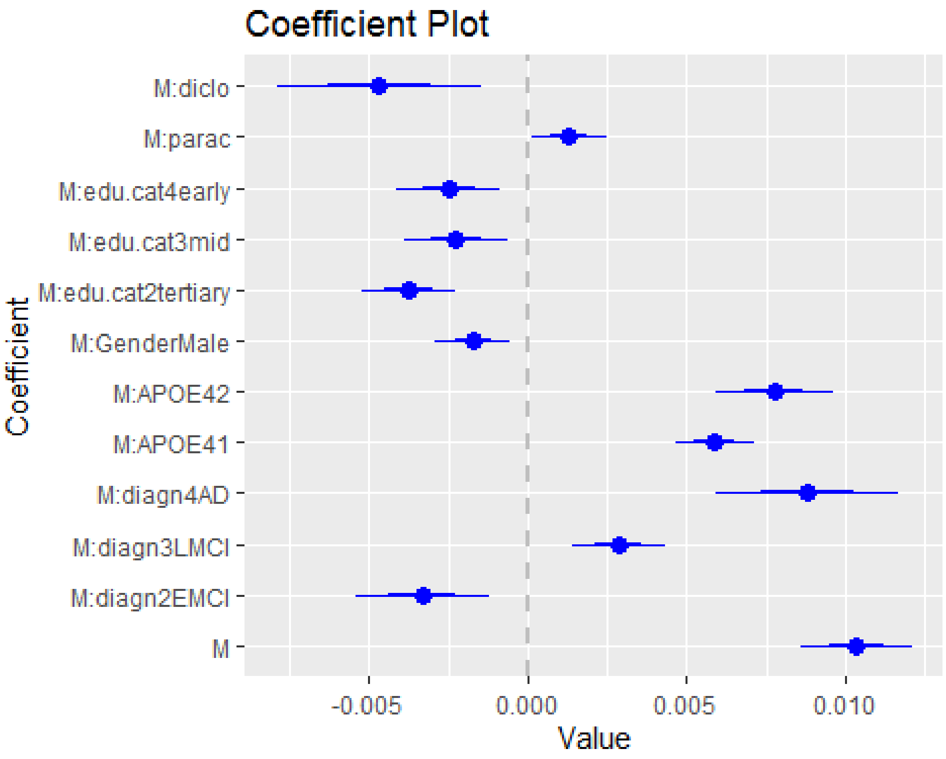

7.16. *Dropping terms of the model to evaluate the significance of each variable in the ful model.*

```
Age.MMSE<- glmmadmb(neg.b.MMSE~ M+ diagn+ APOE4+edu.cat + aspirin+ diagn*M
+APOE4*M + Gender*M+ edu.cat*M +parac*M +diclo*M+(1|ID), family="nbinom1",
data=MMSEdata)

No.APOE4.MMSE<- glmmadmb(neg.b.MMSE~ M+AGE+ diagn+ edu.cat + aspirin+ diagn*M +
Gender*M+ edu.cat*M +parac*M +diclo*M+(1|ID), family="nbinom1", data=MMSEdata)

APOE4.MMSE.Main<- glmmadmb(neg.b.MMSE~ M+AGE+ diagn+ APOE4+edu.cat + aspirin+
diagn*M + Gender*M+ edu.cat*M +parac*M +diclo*M+(1|ID), family="nbinom1",
data=MMSEdata)

No.Education.MMSE<- glmmadmb(neg.b.MMSE~ M+AGE+ diagn+ APOE4+aspirin+ diagn*M
+APOE4*M + Gender*M+ parac*M +diclo*M+(1|ID), family="nbinom1", data=MMSEdata)

Education.MMSE.Main<- glmmadmb(neg.b.MMSE~ M+AGE+ diagn+ APOE4+edu.cat +
aspirin+ APOE4*M+diagn*M + Gender*M +parac*M +diclo*M+(1|ID), family="nbinom1",
data=MMSEdata)

No.Diagn.MMSE<- glmmadmb(neg.b.MMSE~ M+AGE+ APOE4+edu.cat + aspirin+ APOE4*M +
Gender*M+ edu.cat*M +parac*M +diclo*M+(1|ID), family="nbinom1", data=MMSEdata)

Diagn.MMSE.Main<- glmmadmb(neg.b.MMSE~ M+AGE+ diagn+ APOE4+edu.cat + aspirin+
APOE4*M + Gender*M+ edu.cat*M +parac*M +diclo*M+(1|ID), family="nbinom1",
data=MMSEdata)

No.Gender.MMSE<- glmmadmb(neg.b.MMSE~ M+AGE+ diagn+ APOE4+edu.cat + aspirin+
diagn*M +APOE4*M +  edu.cat*M +parac*M +diclo*M+(1|ID), family="nbinom1",
data=MMSEdata)

Gender.MMSE.Main<- glmmadmb(neg.b.MMSE~ M+AGE+ diagn+ APOE4+edu.cat + aspirin+
diagn*M +APOE4*M + Gender+ edu.cat*M +parac*M +diclo*M+(1|ID), family="nbinom1",
```

```
data=MMSEdata)

No.Aspirin.MMSE<- glmmadmb(neg.b.MMSE~ M+AGE+ diagn+ APOE4+edu.cat + diagn*M
+APOE4*M + Gender*M+ edu.cat*M +parac*M +diclo*M+(1|ID), family="nbinom1",
data=MMSEdata)


No.Paracet.MMSE<- glmmadmb(neg.b.MMSE~ M+AGE+ diagn+ APOE4+edu.cat + aspirin+
diagn*M +APOE4*M + Gender*M+ edu.cat*M +diclo*M+(1|ID), family="nbinom1",
data=MMSEdata)


Paracet.MMSE.Main<- glmmadmb(neg.b.MMSE~ M+AGE+ diagn+ APOE4+edu.cat + aspirin+
diagn*M +APOE4*M + Gender*M+ edu.cat*M +diclo*M+parac+(1|ID), family="nbinom1",
data=MMSEdata)

No.Diclofen.MMSE<- glmmadmb(neg.b.MMSE~ M+AGE+ diagn+ APOE4+edu.cat + aspirin+
diagn*M +APOE4*M + Gender*M+ edu.cat*M +parac*M + (1|ID), family="nbinom1",
data=MMSEdata)

Diclofen.MMSE.Main<- glmmadmb(neg.b.MMSE~ M+AGE+ diagn+ APOE4+edu.cat + aspirin+
diagn*M +APOE4*M + Gender*M+ edu.cat*M +parac*M +diclo+(1|ID), family="nbinom1",
data=MMSEdata)

Model<-c("Main effect of Age", "Main effect of APOE4", "Main effect of
Education", "Main effect of Diagnosis","Main effect of Gender", "Main effect of
Aspirin","Main effect of Paracetamol","Main effect of Diclofenac","Interaction
effect of Education","Interaction effect of Diagnosis","Interaction effect of
APOE4","Interaction effect of Gender","Interaction effect of
Paracetamol","Interaction effect of Diclofenac")

M.age<-anova(neg.mmse, Age.MMSE)
M.APOE4<-anova(No.APOE4.MMSE, APOE4.MMSE.Main)
M.Education<-anova(Education.MMSE.Main,No.Education.MMSE)
M.Diagn<-anova(No.Diagn.MMSE, Diagn.MMSE.Main)
M.Gender<-anova(No.Gender.MMSE, Gender.MMSE.Main)
M.Aspirin<-anova(No.Aspirin.MMSE, neg.mmse)
M.Paracetamol<-anova(No.Paracet.MMSE, Paracet.MMSE.Main)
M.Diclofenac<-anova(No.Diclofen.MMSE, Diclofen.MMSE.Main)

I.Education<-anova(neg.mmse, Education.MMSE.Main)
I.Diagnosis<-anova(neg.mmse, Diagn.MMSE.Main)
I.APOE4<-anova(neg.mmse, APOE4.MMSE.Main)
I.Gender<-anova(neg.mmse, Gender.MMSE.Main)
I.Paracetamol<-anova(neg.mmse, Paracet.MMSE.Main)
I.Diclofenac<-anova(neg.mmse, Diclofen.MMSE.Main)

MMSE.PValue<-
rbind(M.age[2,3:5],M.APOE4[2,3:5],M.Education[2,3:5],M.Diagn[2,3:5],M.Gender[2,3
:5],M.Aspirin[2,3:5],M.Paracetamol[2,3:5],M.Diclofenac[2,3:5],I.Education[2,3:5]
,I.Diagnosis[2,3:5],I.APOE4[2,3:5],I.Gender[2,3:5],I.Paracetamol[2,3:5],I.Diclof
enac[2,3:5])

data.frame(Model,MMSE.PValue)

                                Model Df Deviance     Pr..Chi.
2                  Main effect of Age  1     48.2 3.848921e-12
21               Main effect of APOE4  2     60.6 6.927792e-14
22           Main effect of Education  3     47.4 2.857187e-10
```

```
23           Main effect of Diagnosis  3   1147.2 0.000000e+00
24              Main effect of Gender  1      2.2 1.380107e-01
25             Main effect of Aspirin  1      4.0 4.550026e-02
26         Main effect of Paracetamol  1      1.8 1.797125e-01
27          Main effect of Diclofenac  1      1.0 3.173105e-01
28    Interaction effect of Education  3     26.6 7.140958e-06
29    Interaction effect of Diagnosis  3     79.8 0.000000e+00
210       Interaction effect of APOE4  2    120.6 0.000000e+00
211      Interaction effect of Gender  1      8.4 3.752210e-03
212 Interaction effect of Paracetamol  1      4.8 2.845974e-02
213  Interaction effect of Diclofenac  1      8.4 3.752210e-03
```

7.17. *Evaluating the progession and main-effects of each pain medication*

```
neg.M.base<- glmmadmb(neg.b.MMSE~ AGE + APOE4  + M + edu.cat + diagn+ diagn*M
+APOE4*M + Gender*M+ edu.cat*M + (1|ID), family="nbinom1", data=MMSEdata)

neg.M.M.aspirin<- glmmadmb(neg.b.MMSE~ AGE + APOE4  + M + edu.cat + diagn+
diagn*M +APOE4*M + Gender*M+ edu.cat*M + aspirin+(1|ID), family="nbinom1",
data=MMSEdata)

neg.M.M.celecoxib<- glmmadmb(neg.b.MMSE~ AGE + APOE4  + M + edu.cat + diagn+
diagn*M +APOE4*M + Gender*M+ edu.cat*M + celex+(1|ID), family="nbinom1",
data=MMSEdata)

neg.M.M.diclofenac<- glmmadmb(neg.b.MMSE~ AGE + APOE4  + M + edu.cat + diagn+
diagn*M +APOE4*M + Gender*M+ edu.cat*M +diclo+ (1|ID), family="nbinom1",
data=MMSEdata)

neg.M.M.ibuprofen<- glmmadmb(neg.b.MMSE~ AGE + APOE4  + M + edu.cat + diagn+
diagn*M +APOE4*M + Gender*M+ edu.cat*M +Ibu+ (1|ID), family="nbinom1",
data=MMSEdata)

neg.M.M.naproxen<- glmmadmb(neg.b.MMSE~ AGE + APOE4  + M + edu.cat + diagn+
diagn*M +APOE4*M + Gender*M+ edu.cat*M +naprox+ (1|ID), family="nbinom1",
data=MMSEdata)

neg.M.M.paracetamol<- glmmadmb(neg.b.MMSE~ AGE + APOE4  + M + edu.cat + diagn+
diagn*M +APOE4*M + Gender*M+ edu.cat*M + parac+ (1|ID), family="nbinom1",
data=MMSEdata)

neg.M.I.aspirin<- glmmadmb(neg.b.MMSE~ AGE + APOE4  + M + edu.cat + diagn+
diagn*M +APOE4*M + Gender*M+ edu.cat*M +aspirin*M+ (1|ID), family="nbinom1",
data=MMSEdata)

neg.M.I.celecoxib<- glmmadmb(neg.b.MMSE~ AGE + APOE4  + M + edu.cat + diagn+
diagn*M +APOE4*M + Gender*M+ edu.cat*M +celex*M+ (1|ID), family="nbinom1",
data=MMSEdata)

neg.M.I.diclofenac<- glmmadmb(neg.b.MMSE~ AGE + APOE4  + M + edu.cat + diagn+
diagn*M +APOE4*M + Gender*M+ edu.cat*M +diclo*M+ (1|ID), family="nbinom1",
data=MMSEdata)

neg.M.I.ibuprofen<- glmmadmb(neg.b.MMSE~ AGE + APOE4  + M + edu.cat + diagn+
diagn*M +APOE4*M + Gender*M+ edu.cat*M +Ibu*M+ (1|ID), family="nbinom1",
data=MMSEdata)

neg.M.I.naproxen<- glmmadmb(neg.b.MMSE~ AGE + APOE4  + M + edu.cat + diagn+
diagn*M +APOE4*M + Gender*M+ edu.cat*M + naprox*M+ (1|ID), family="nbinom1",
```

```
data=MMSEdata)

neg.M.I.paracetamol<- glmmadmb(neg.b.MMSE~ AGE + APOE4  + M + edu.cat + diagn+
diagn*M +APOE4*M + Gender*M+ edu.cat*M +parac*M+ (1|ID), family="nbinom1",
data=MMSEdata)

anova.M.aspirin<-anova(neg.M.M.aspirin, neg.M.base)
anova.M.celecoxib<-anova(neg.M.M.celecoxib, neg.M.base)
anova.M.diclofenac<-anova(neg.M.M.diclofenac, neg.M.base)
anova.M.ibuprofen<-anova(neg.M.M.ibuprofen, neg.M.base)
anova.M.naproxen<-anova(neg.M.M.naproxen, neg.M.base)
anova.M.paracetamol<-anova(neg.M.M.paracetamol, neg.M.base)

anova.I.aspirin<-anova(neg.M.M.aspirin, neg.M.I.aspirin)
anova.I.celecoxib<-anova(neg.M.M.celecoxib, neg.M.I.celecoxib)
anova.I.diclofenac<-anova(neg.M.M.diclofenac, neg.M.I.diclofenac)
anova.I.ibuprofen<-anova(neg.M.M.ibuprofen, neg.M.I.ibuprofen)
anova.I.naproxen<-anova(neg.M.M.naproxen, neg.M.I.naproxen)
anova.I.paracetamol<-anova(neg.M.M.paracetamol, neg.M.I.paracetamol)
Model.Painrelief.MMSE<- c("Main effect of Aspirin", "Main effect of Celecoxib",
"Main effect of Diclofenac", "Main effect of Ibuprofen", "Main effect of
Naproxen","Main effect of Paracetamol","Interaction effect of
Aspirin","Interaction effect of Celecoxib","Interaction effect of
Diclofenac","Interaction effect of Ibuprofen","Interaction effect of
Naproxen","Interaction effect of Paracetamol")

MMSE.Painrelief.PValue<-rbind(anova.M.aspirin[2,3:5], anova.M.celecoxib[2,3:5],
anova.M.diclofenac[2,3:5], anova.M.ibuprofen[2,3:5], anova.M.naproxen[2,3:5],
anova.M.paracetamol[2,3:5], anova.I.aspirin[2,3:5], anova.I.celecoxib[2,3:5],
anova.I.diclofenac[2,3:5], anova.I.ibuprofen[2,3:5], anova.I.naproxen[2,3:5],
anova.I.paracetamol[2,3:5])


data.frame(Model.Painrelief.MMSE, MMSE.Painrelief.PValue)

                Model.Painrelief.MMSE Df Deviance    Pr..Chi.
2              Main effect of Aspirin  1      4.8 0.028459737
21           Main effect of Celecoxib  1      2.2 0.138010738
22          Main effect of Diclofenac  1      1.2 0.273321678
23           Main effect of Ibuprofen  1      3.6 0.057779571
24            Main effect of Naproxen  1      0.2 0.654720846
25         Main effect of Paracetamol  1      2.6 0.106863715
26      Interaction effect of Aspirin  1      4.0 0.045500264
27    Interaction effect of Celecoxib  1      2.4 0.121335250
28   Interaction effect of Diclofenac  1      7.4 0.006522388
29    Interaction effect of Ibuprofen  1      0.0 1.000000000
210    Interaction effect of Naproxen  1      0.0 1.000000000
211 Interaction effect of Paracetamol  1      3.6 0.057779571
```

7.18. *Month as a factor*

Including month as a factor was not possible due to the extremely large degrees of freedom that would be required in the model. This, and the fact that the error distributions showed no sign of inappropriate fitting, suggest that month modelled as a numerical variable is appropriate.

```
try(neg.m.M.factor <- glmmadmb(neg.b.MMSE~ AGE + APOE4  + as.factor(M) + edu.cat
+ diagn+aspirin + diagn*as.factor(M) +APOE4*as.factor(M) + Gender*as.factor(M)+
edu.cat*as.factor(M) +parac*as.factor(M) +diclo*as.factor(M)+(1|ID),
```

```
family="nbinom1", data=MMSEdata))

try(neg.m.M.factor.simple <- glmmadmb(neg.b.MMSE~ AGE + APOE4  + as.factor(M) +
edu.cat + diagn +aspirin + diagn*as.factor(M) +diclo*as.factor(M)+(1|ID),
family="nbinom1", data=MMSEdata))
```

7.19. *Years education as a numerical variable*

Treating years education as a numerical variable destabilized the model, causing failure to converge.

```
MMSEdata$Yrs.edu<-as.numeric(MMSEdata$Yrs.edu)
try(neg.m.Yrs.edu<- glmmadmb(neg.b.MMSE~ AGE + APOE4  + M + Yrs.edu +
diagn+aspirin+ diagn*M +APOE4*M + Gender*M+ Yrs.edu*M +parac*M +diclo*M+(1|ID),
family="nbinom1", data=MMSEdata))
try(anova(neg.m.Yrs.edu,neg.mmse))

Analysis of Deviance Table

Model 1: neg.b.MMSE ~ AGE + APOE4 + M + Yrs.edu + diagn + aspirin + diagn * M +
APOE4 * M + Gender * M + Yrs.edu * M + parac * M + diclo * M
Model 2: neg.b.MMSE ~ M + AGE + diagn + APOE4 + edu.cat + aspirin + diagn * M +
APOE4 * M + Gender * M + edu.cat * M + parac * M + diclo * M
  NoPar LogLik Df Deviance Pr(>Chi)
1    24 -16949
2    28 -16940  4     17.6 0.001477 **
---
Signif. codes:  0 '***' 0.001 '**' 0.01 '*' 0.05 '.' 0.1 ' ' 1
```

7.20. *APOE4 and NSAIDs*

There is evidence that NSAIDs interact are only protective in the presence of the APOE4 gene. Therefore, a three way interaction term was investigated.

```
neg.m.APOE.diclo <- glmmadmb(neg.b.MMSE~ AGE + APOE4  + M + edu.cat + aspirin+
diagn+ diagn*M +APOE4*M + Gender*M+ edu.cat*M +parac*M
+diclo*M+diclo*APOE4+(1|ID), family="nbinom1", data=MMSEdata)
summary(neg.m.APOE.diclo)


Call:
glmmadmb(formula = neg.b.MMSE ~ AGE + APOE4 + M + edu.cat + aspirin +
    diagn + diagn * M + APOE4 * M + Gender * M + edu.cat * M +
    parac * M + diclo * M + diclo * APOE4 + (1 | ID), data = MMSEdata,
    family = "nbinom1")

AIC: 33939

Coefficients:
                    Estimate Std. Error z value Pr(>|z|)
(Intercept)        -0.586477   0.063201   -9.28  < 2e-16 ***
AGE                 0.018022   0.002598    6.94  4.0e-12 ***
APOE41              0.120373   0.042674    2.82  0.00479 **
APOE42              0.220098   0.065157    3.38  0.00073 ***
M                   0.010354   0.000863   12.00  < 2e-16 ***
edu.cat2tertiary    0.200344   0.049549    4.04  5.3e-05 ***
edu.cat3mid         0.362179   0.055633    6.51  7.5e-11 ***
edu.cat4early       0.353961   0.057501    6.16  7.5e-10 ***
```

```
aspirin             -0.073973   0.037121   -1.99  0.04629 *
diagn2EMCI           0.707705   0.065736   10.77  < 2e-16 ***
diagn3LMCI           1.240176   0.055552   22.32  < 2e-16 ***
diagn4AD             2.105114   0.062143   33.88  < 2e-16 ***
GenderMale           0.094606   0.040499    2.34  0.01949 *
parac               -0.093733   0.045043   -2.08  0.03744 *
diclo               -0.021649   0.193610   -0.11  0.91097
M:diagn2EMCI        -0.003318   0.001043   -3.18  0.00147 **
M:diagn3LMCI         0.002860   0.000725    3.94  8.0e-05 ***
M:diagn4AD           0.008800   0.001437    6.12  9.2e-10 ***
APOE41:M             0.005877   0.000617    9.52  < 2e-16 ***
APOE42:M             0.007738   0.000931    8.31  < 2e-16 ***
M:GenderMale        -0.001725   0.000591   -2.92  0.00353 **
M:edu.cat2tertiary -0.003741   0.000743   -5.04  4.7e-07 ***
M:edu.cat3mid       -0.002254   0.000812   -2.78  0.00550 **
M:edu.cat4early     -0.002486   0.000818   -3.04  0.00238 **
M:parac              0.001282   0.000589    2.18  0.02962 *
M:diclo             -0.004729   0.001621   -2.92  0.00353 **
APOE41:diclo         0.011430   0.290830    0.04  0.96865
APOE42:diclo         0.490448   0.503930    0.97  0.33043
---
Signif. codes:  0 '***' 0.001 '**' 0.01 '*' 0.05 '.' 0.1 ' ' 1

Number of observations: total=8878, ID=1619
Random effect variance(s):
Group=ID
            Variance StdDev
(Intercept)    0.413 0.6427

Negative binomial dispersion parameter: 1.0582 (std. err.: 0.018202)

Log-likelihood: -16939.5

anova(neg.mmse,neg.m.APOE.diclo)

Analysis of Deviance Table

Model 1: neg.b.MMSE ~ M + AGE + diagn + APOE4 + edu.cat + aspirin + diagn * M +
APOE4 * M + Gender * M + edu.cat * M + parac * M + diclo * M
Model 2: neg.b.MMSE ~ AGE + APOE4 + M + edu.cat + aspirin + diagn + diagn * M +
APOE4 * M + Gender * M + edu.cat * M + parac * M + diclo * M + diclo * APOE4
  NoPar LogLik Df Deviance Pr(>Chi)
1    28 -16940
2    30 -16940  2        1   0.6065

neg.m.APOE.diclo.M <- glmmadmb(neg.b.MMSE~ AGE + APOE4  + M + edu.cat + aspirin+
diagn+ diagn*M +APOE4*M + Gender*M+ edu.cat*M +parac*M
+diclo*M+diclo*APOE4*M+(1|ID), family="nbinom1", data=MMSEdata)
summary(neg.m.APOE.diclo)


Call:
glmmadmb(formula = neg.b.MMSE ~ AGE + APOE4 + M + edu.cat + aspirin +
    diagn + diagn * M + APOE4 * M + Gender * M + edu.cat * M +
    parac * M + diclo * M + diclo * APOE4 + (1 | ID), data = MMSEdata,
    family = "nbinom1")

AIC: 33939

Coefficients:
```

```
                    Estimate Std. Error z value Pr(>|z|)
(Intercept)        -0.586477   0.063201   -9.28  < 2e-16 ***
AGE                 0.018022   0.002598    6.94  4.0e-12 ***
APOE41              0.120373   0.042674    2.82  0.00479 **
APOE42              0.220098   0.065157    3.38  0.00073 ***
M                   0.010354   0.000863   12.00  < 2e-16 ***
edu.cat2tertiary    0.200344   0.049549    4.04  5.3e-05 ***
edu.cat3mid         0.362179   0.055633    6.51  7.5e-11 ***
edu.cat4early       0.353961   0.057501    6.16  7.5e-10 ***
aspirin            -0.073973   0.037121   -1.99  0.04629 *
diagn2EMCI          0.707705   0.065736   10.77  < 2e-16 ***
diagn3LMCI          1.240176   0.055552   22.32  < 2e-16 ***
diagn4AD            2.105114   0.062143   33.88  < 2e-16 ***
GenderMale          0.094606   0.040499    2.34  0.01949 *
parac              -0.093733   0.045043   -2.08  0.03744 *
diclo              -0.021649   0.193610   -0.11  0.91097
M:diagn2EMCI       -0.003318   0.001043   -3.18  0.00147 **
M:diagn3LMCI        0.002860   0.000725    3.94  8.0e-05 ***
M:diagn4AD          0.008800   0.001437    6.12  9.2e-10 ***
APOE41:M            0.005877   0.000617    9.52  < 2e-16 ***
APOE42:M            0.007738   0.000931    8.31  < 2e-16 ***
M:GenderMale       -0.001725   0.000591   -2.92  0.00353 **
M:edu.cat2tertiary -0.003741   0.000743   -5.04  4.7e-07 ***
M:edu.cat3mid      -0.002254   0.000812   -2.78  0.00550 **
M:edu.cat4early    -0.002486   0.000818   -3.04  0.00238 **
M:parac             0.001282   0.000589    2.18  0.02962 *
M:diclo            -0.004729   0.001621   -2.92  0.00353 **
APOE41:diclo        0.011430   0.290830    0.04  0.96865
APOE42:diclo        0.490448   0.503930    0.97  0.33043
---
Signif. codes:  0 '***' 0.001 '**' 0.01 '*' 0.05 '.' 0.1 ' ' 1

Number of observations: total=8878, ID=1619
Random effect variance(s):
Group=ID
            Variance StdDev
(Intercept)    0.413 0.6427

Negative binomial dispersion parameter: 1.0582 (std. err.: 0.018202)

Log-likelihood: -16939.5

anova(neg.m.APOE.diclo.M,neg.m.APOE.diclo)

Analysis of Deviance Table

Model 1: neg.b.MMSE ~ AGE + APOE4 + M + edu.cat + aspirin + diagn + diagn * M +
APOE4 * M + Gender * M + edu.cat * M + parac * M + diclo * M + diclo * APOE4
Model 2: neg.b.MMSE ~ AGE + APOE4 + M + edu.cat + aspirin + diagn + diagn * M +
APOE4 * M + Gender * M + edu.cat * M + parac * M + diclo * M + diclo * APOE4 * M
  NoPar LogLik Df Deviance Pr(>Chi)
1    30 -16940
2    32 -16935  2      8.4    0.015 *
---
Signif. codes:  0 '***' 0.001 '**' 0.01 '*' 0.05 '.' 0.1 ' ' 1

MMSEdata$x<-
MMSEdata$diclo+MMSEdata$naprox+MMSEdata$celex+MMSEdata$parac+MMSEdata$aspirin+MM
SEdata$Ibu
MMSEdata$NSAID<-1*(MMSEdata$x>0)
```

```
neg.m.APOE.NSAID <- glmmadmb(neg.b.MMSE~ AGE + APOE4  + M + edu.cat + diagn+
diagn*M +APOE4*M + Gender*M+ edu.cat*M +NSAID*M+NSAID*APOE4+(1|ID),
family="nbinom1", data=MMSEdata)
summary(neg.m.APOE.NSAID)


Call:
glmmadmb(formula = neg.b.MMSE ~ AGE + APOE4 + M + edu.cat + diagn +
    diagn * M + APOE4 * M + Gender * M + edu.cat * M + NSAID *
    M + NSAID * APOE4 + (1 | ID), data = MMSEdata, family = "nbinom1")

AIC: 33939

Coefficients:
                    Estimate Std. Error z value Pr(>|z|)
(Intercept)        -0.509109   0.072880   -6.99  2.8e-12 ***
AGE                 0.017754   0.002592    6.85  7.5e-12 ***
APOE41              0.072895   0.072338    1.01  0.31360
APOE42              0.122366   0.110740    1.10  0.26916
M                   0.010264   0.001056    9.72  < 2e-16 ***
edu.cat2tertiary    0.197074   0.049499    3.98  6.9e-05 ***
edu.cat3mid         0.359029   0.055529    6.47  1.0e-10 ***
edu.cat4early       0.352386   0.057458    6.13  8.6e-10 ***
diagn2EMCI          0.714212   0.065636   10.88  < 2e-16 ***
diagn3LMCI          1.238644   0.055380   22.37  < 2e-16 ***
diagn4AD            2.095731   0.062073   33.76  < 2e-16 ***
GenderMale          0.097978   0.040091    2.44  0.01453 *
NSAID              -0.201718   0.059833   -3.37  0.00075 ***
M:diagn2EMCI       -0.003285   0.001040   -3.16  0.00158 **
M:diagn3LMCI        0.002989   0.000723    4.13  3.6e-05 ***
M:diagn4AD          0.008921   0.001448    6.16  7.3e-10 ***
APOE41:M            0.005836   0.000619    9.43  < 2e-16 ***
APOE42:M            0.007504   0.000920    8.16  3.5e-16 ***
M:GenderMale       -0.001878   0.000592   -3.17  0.00150 **
M:edu.cat2tertiary -0.003562   0.000740   -4.81  1.5e-06 ***
M:edu.cat3mid      -0.002069   0.000810   -2.55  0.01063 *
M:edu.cat4early    -0.002644   0.000819   -3.23  0.00125 **
M:NSAID             0.000490   0.000806    0.61  0.54307
APOE41:NSAID        0.077151   0.085034    0.91  0.36425
APOE42:NSAID        0.162113   0.129500    1.25  0.21063
---
Signif. codes:  0 '***' 0.001 '**' 0.01 '*' 0.05 '.' 0.1 ' ' 1

Number of observations: total=8878, ID=1619
Random effect variance(s):
Group=ID
            Variance StdDev
(Intercept)   0.4123 0.6421

Negative binomial dispersion parameter: 1.0588 (std. err.: 0.018211)

Log-likelihood: -16942.5

neg.m.NSAID<- glmmadmb(neg.b.MMSE~ AGE + APOE4  + M + edu.cat + diagn+ diagn*M
+APOE4*M + Gender*M+ edu.cat*M +NSAID*M+(1|ID), family="nbinom1", data=MMSEdata)
summary(neg.m.NSAID)


Call:
```

```
glmmadmb(formula = neg.b.MMSE ~ AGE + APOE4 + M + edu.cat + diagn +
    diagn * M + APOE4 * M + Gender * M + edu.cat * M + NSAID *
    M + (1 | ID), data = MMSEdata, family = "nbinom1")

AIC: 33937

Coefficients:
                    Estimate Std. Error z value Pr(>|z|)
(Intercept)        -0.547319   0.066231   -8.26  < 2e-16 ***
AGE                 0.017766   0.002591    6.86  7.1e-12 ***
APOE41              0.126465   0.042371    2.98  0.00284 **
APOE42              0.234726   0.064770    3.62  0.00029 ***
M                   0.010271   0.001057    9.72  < 2e-16 ***
edu.cat2tertiary    0.197337   0.049518    3.99  6.7e-05 ***
edu.cat3mid         0.359542   0.055557    6.47  9.7e-11 ***
edu.cat4early       0.356310   0.057419    6.21  5.5e-10 ***
diagn2EMCI          0.715000   0.065654   10.89  < 2e-16 ***
diagn3LMCI          1.239568   0.055403   22.37  < 2e-16 ***
diagn4AD            2.096789   0.062094   33.77  < 2e-16 ***
GenderMale          0.099481   0.040095    2.48  0.01310 *
NSAID              -0.151098   0.042914   -3.52  0.00043 ***
M:diagn2EMCI       -0.003287   0.001040   -3.16  0.00157 **
M:diagn3LMCI        0.002981   0.000723    4.12  3.7e-05 ***
M:diagn4AD          0.008910   0.001449    6.15  7.7e-10 ***
APOE41:M            0.005877   0.000617    9.52  < 2e-16 ***
APOE42:M            0.007582   0.000919    8.25  < 2e-16 ***
M:GenderMale       -0.001878   0.000592   -3.17  0.00150 **
M:edu.cat2tertiary -0.003562   0.000740   -4.81  1.5e-06 ***
M:edu.cat3mid      -0.002069   0.000810   -2.55  0.01064 *
M:edu.cat4early    -0.002638   0.000819   -3.22  0.00128 **
M:NSAID             0.000456   0.000806    0.57  0.57160
---
Signif. codes:  0 '***' 0.001 '**' 0.01 '*' 0.05 '.' 0.1 ' ' 1

Number of observations: total=8878, ID=1619
Random effect variance(s):
Group=ID
            Variance StdDev
(Intercept)   0.4128 0.6425

Negative binomial dispersion parameter: 1.0589 (std. err.: 0.018216)

Log-likelihood: -16943.5

anova(neg.m.NSAID,neg.m.APOE.NSAID)

Analysis of Deviance Table

Model 1: neg.b.MMSE ~ AGE + APOE4 + M + edu.cat + diagn + diagn * M + APOE4 * M
+ Gender * M + edu.cat * M + NSAID * M
Model 2: neg.b.MMSE ~ AGE + APOE4 + M + edu.cat + diagn + diagn * M + APOE4 * M
+ Gender * M + edu.cat * M + NSAID * M + NSAID * APOE4
  NoPar LogLik Df Deviance Pr(>Chi)
1    25 -16944
2    27 -16943  2        2   0.3679

neg.m.NSAID.APOE.M<- glmmadmb(neg.b.MMSE~ AGE + APOE4  + M + edu.cat + diagn+
diagn*M +APOE4*M + Gender*M+ edu.cat*M +NSAID*M*APOE4+(1|ID), family="nbinom1",
data=MMSEdata)
summary(neg.m.NSAID.APOE.M)
```

```
Call:
glmmadmb(formula = neg.b.MMSE ~ AGE + APOE4 + M + edu.cat + diagn +
    diagn * M + APOE4 * M + Gender * M + edu.cat * M + NSAID *
    M * APOE4 + (1 | ID), data = MMSEdata, family = "nbinom1")

AIC: 33942.4

Coefficients:
                    Estimate Std. Error z value Pr(>|z|)
(Intercept)        -0.503386   0.074096   -6.79  1.1e-11 ***
AGE                 0.017764   0.002592    6.85  7.2e-12 ***
APOE41              0.057797   0.076194    0.76  0.44812
APOE42              0.128431   0.115200    1.11  0.26492
M                   0.009939   0.001291    7.70  1.4e-14 ***
edu.cat2tertiary    0.196929   0.049488    3.98  6.9e-05 ***
edu.cat3mid         0.359072   0.055517    6.47  9.9e-11 ***
edu.cat4early       0.351934   0.057454    6.13  9.0e-10 ***
diagn2EMCI          0.714388   0.065623   10.89  < 2e-16 ***
diagn3LMCI          1.238965   0.055367   22.38  < 2e-16 ***
diagn4AD            2.095683   0.062056   33.77  < 2e-16 ***
GenderMale          0.097906   0.040080    2.44  0.01457 *
NSAID              -0.208913   0.061912   -3.37  0.00074 ***
M:diagn2EMCI       -0.003288   0.001040   -3.16  0.00157 **
M:diagn3LMCI        0.002968   0.000724    4.10  4.1e-05 ***
M:diagn4AD          0.008900   0.001449    6.14  8.1e-10 ***
APOE41:M            0.006747   0.001564    4.31  1.6e-05 ***
APOE42:M            0.006939   0.002479    2.80  0.00512 **
M:GenderMale       -0.001869   0.000592   -3.16  0.00160 **
M:edu.cat2tertiary -0.003555   0.000740   -4.80  1.6e-06 ***
M:edu.cat3mid      -0.002074   0.000810   -2.56  0.01050 *
M:edu.cat4early    -0.002612   0.000822   -3.18  0.00148 **
M:NSAID             0.000865   0.001165    0.74  0.45745
APOE41:NSAID        0.096597   0.090420    1.07  0.28538
APOE42:NSAID        0.154618   0.135500    1.14  0.25383
APOE41:M:NSAID     -0.001074   0.001688   -0.64  0.52460
APOE42:M:NSAID      0.000650   0.002638    0.25  0.80541
---
Signif. codes:  0 '***' 0.001 '**' 0.01 '*' 0.05 '.' 0.1 ' ' 1

Number of observations: total=8878, ID=1619
Random effect variance(s):
Group=ID
            Variance StdDev
(Intercept)   0.4119 0.6418

Negative binomial dispersion parameter: 1.0589 (std. err.: 0.01821)

Log-likelihood: -16942.2

anova(neg.m.NSAID.APOE.M,neg.m.APOE.NSAID)

Analysis of Deviance Table

Model 1: neg.b.MMSE ~ AGE + APOE4 + M + edu.cat + diagn + diagn * M + APOE4 * M
+ Gender * M + edu.cat * M + NSAID * M + NSAID * APOE4
Model 2: neg.b.MMSE ~ AGE + APOE4 + M + edu.cat + diagn + diagn * M + APOE4 * M
+ Gender * M + edu.cat * M + NSAID * M * APOE4
  NoPar LogLik Df Deviance Pr(>Chi)
```

```
1    27 -16943
2    29 -16942  2      0.6   0.7408

AIC(neg.m.APOE.diclo)

[1] 33939

AIC(neg.m.APOE.diclo.M)

[1] 33934.6

AIC(neg.m.APOE.NSAID)

[1] 33939

AIC(neg.m.NSAID)

[1] 33937

AIC(neg.m.NSAID.APOE.M)

[1] 33942.4

AIC(neg.mmse)

[1] 33936
```

7.21. *looking at the distribution of the residuals in the final model, for each variable separately*

Residuals within each explanitory variables collectively and within each individual show no trends and are homoskedastic centred around zero. Therefore this model is accurate and will be used to investiage the effects of the input variables (including pain medications) going forward.

```
augDat <- data.frame(MMSEdata,resid=residuals(neg.mmse,type="pearson"),
fitted=fitted(neg.mmse))
ggplot(augDat,aes(x=Gender,y=resid,group=ID))+geom_boxplot()+coord_flip()
```

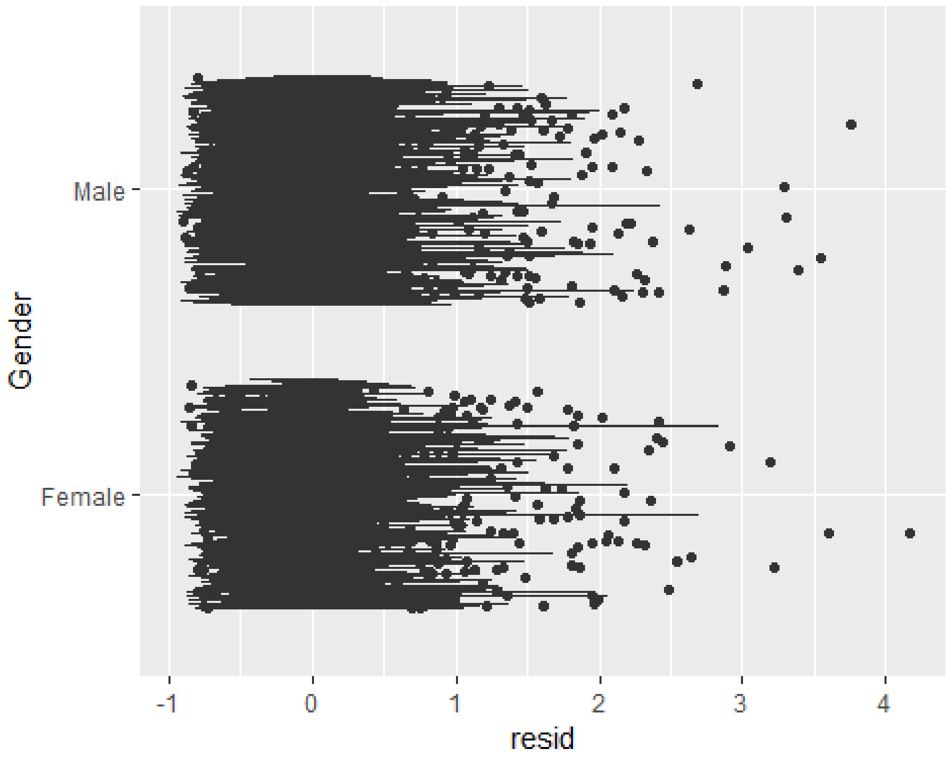


```
ggplot(augDat,aes(x=AGE,y=resid,group=ID))+geom_boxplot()+coord_flip()
```

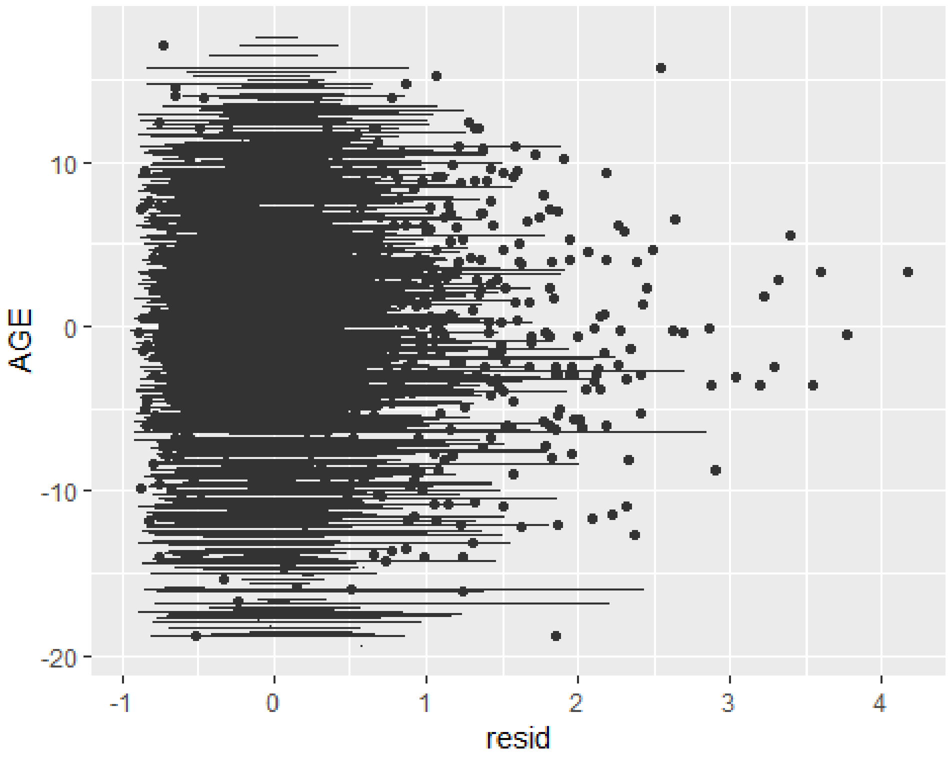


```
ggplot(augDat,aes(x=edu.cat,y=resid,group=ID))+geom_boxplot()+coord_flip()
```

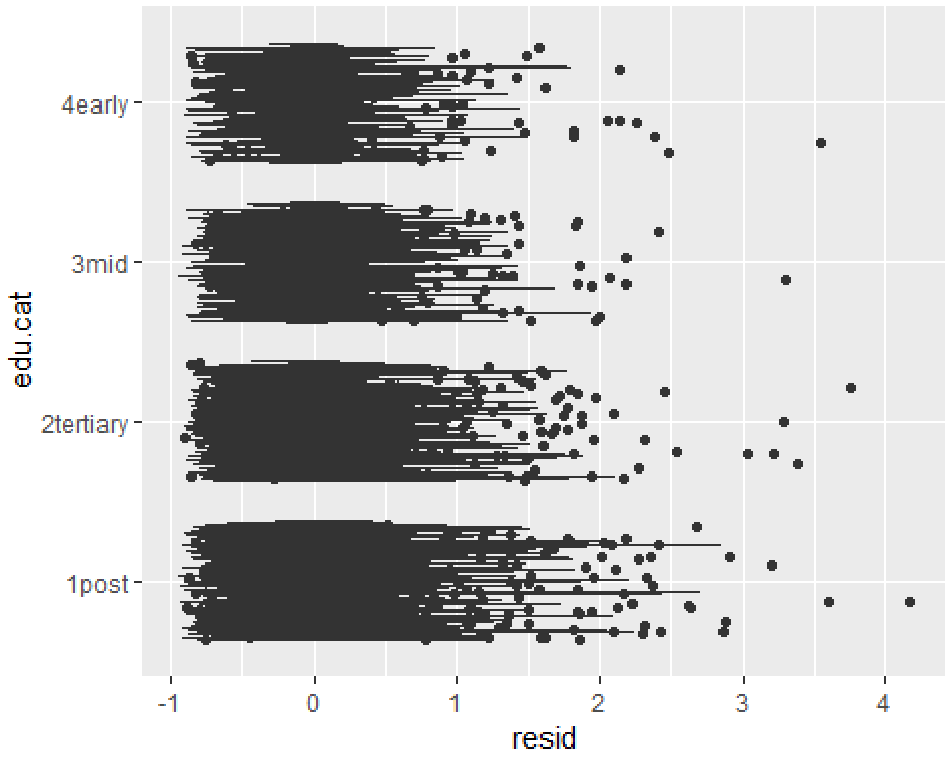


```
ggplot(augDat,aes(x=diagn,y=resid,group=ID))+geom_boxplot()+coord_flip()
```

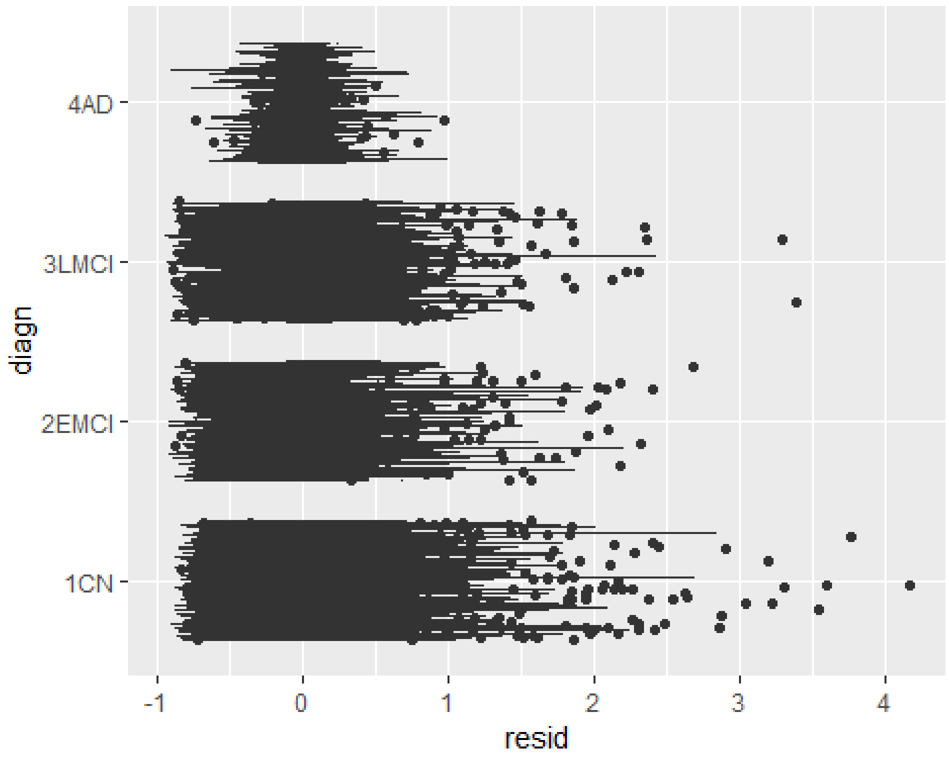


```
ggplot(augDat,aes(x=APOE4,y=resid,group=ID))+geom_boxplot()+coord_flip()
```

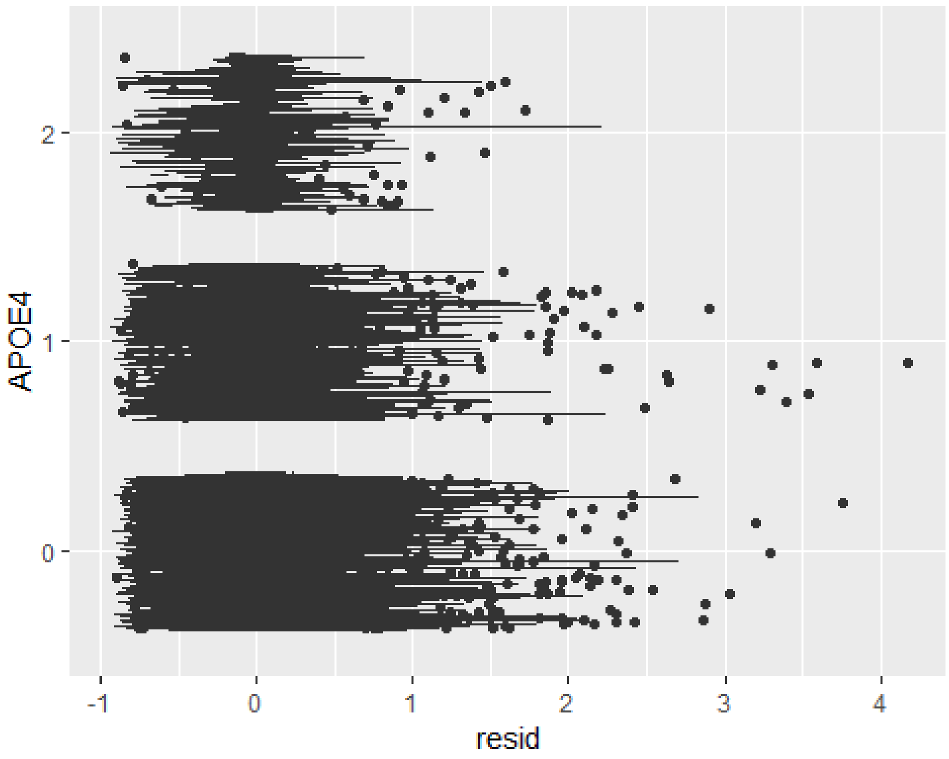


```
ggplot(augDat,aes(x=M,y=resid,group=ID))+geom_boxplot()+coord_flip()
```

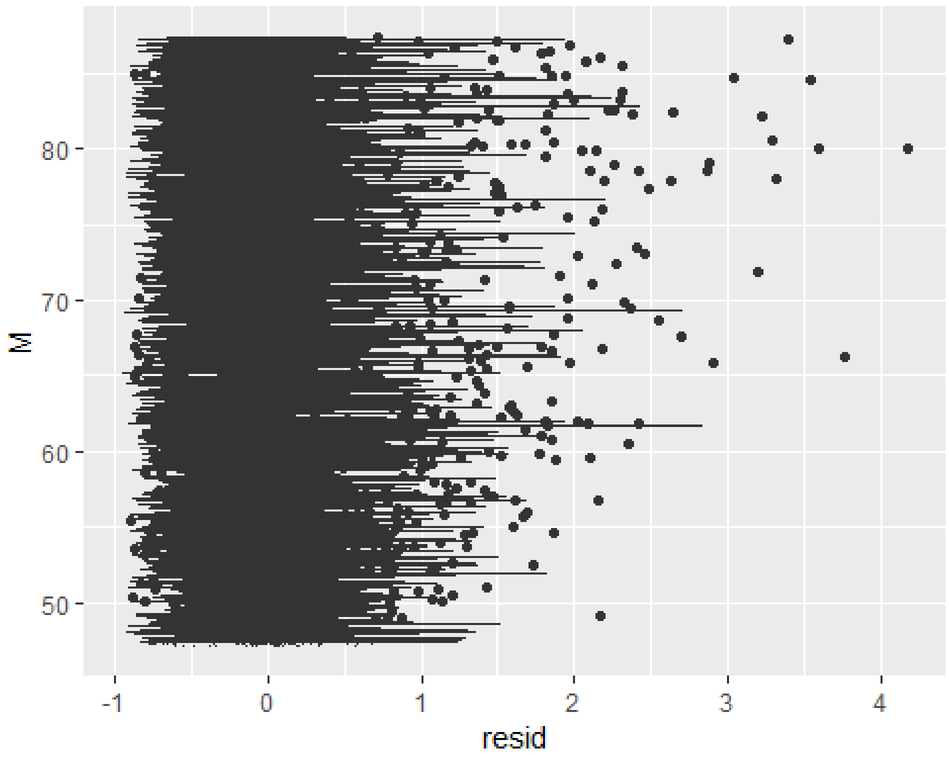


```
ggplot(augDat,aes(x=diclo,y=resid,group=ID))+geom_boxplot()+coord_flip()
```

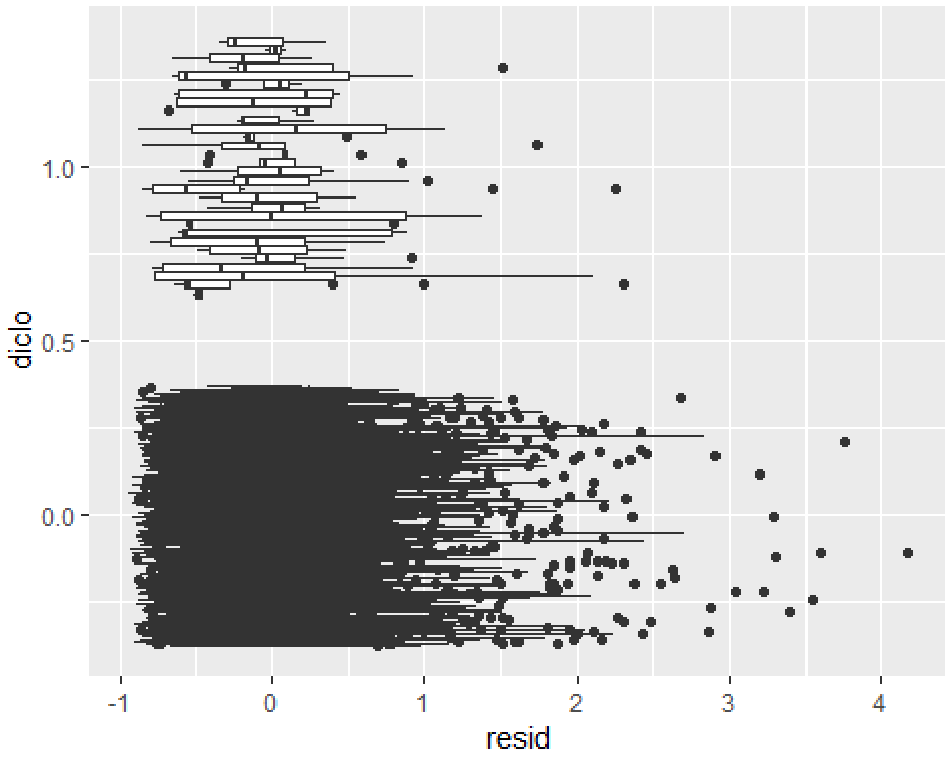


```
ggplot(augDat,aes(x=parac,y=resid,group=ID))+geom_boxplot()+coord_flip()
```

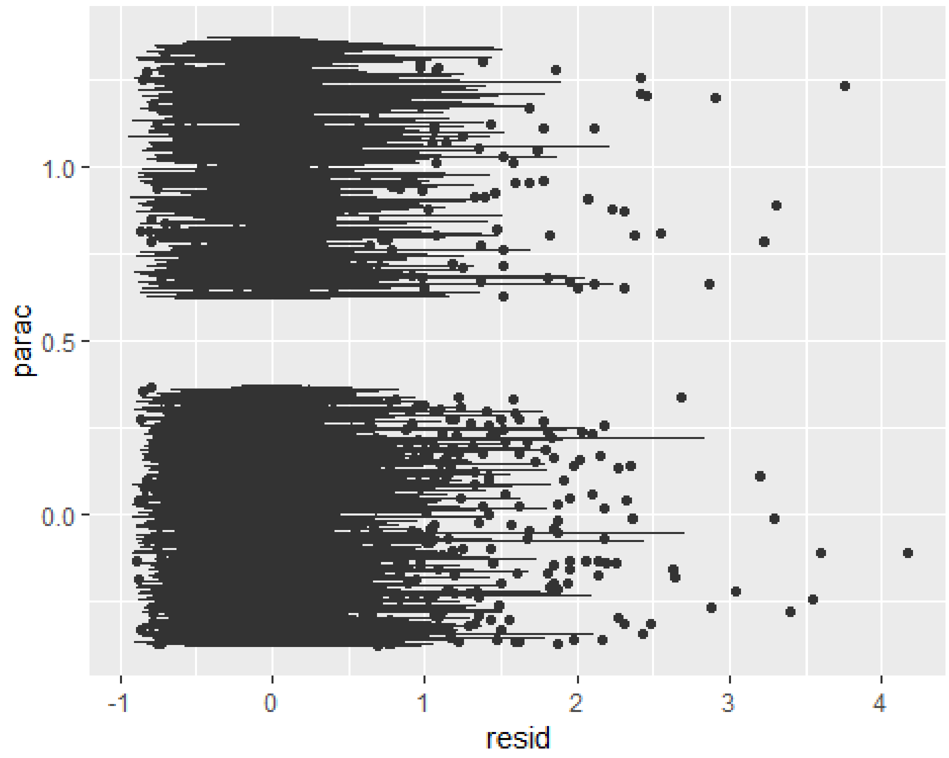


```
ggplot(augDat,aes(x=Gender,y=resid))+geom_boxplot()+coord_flip()
```

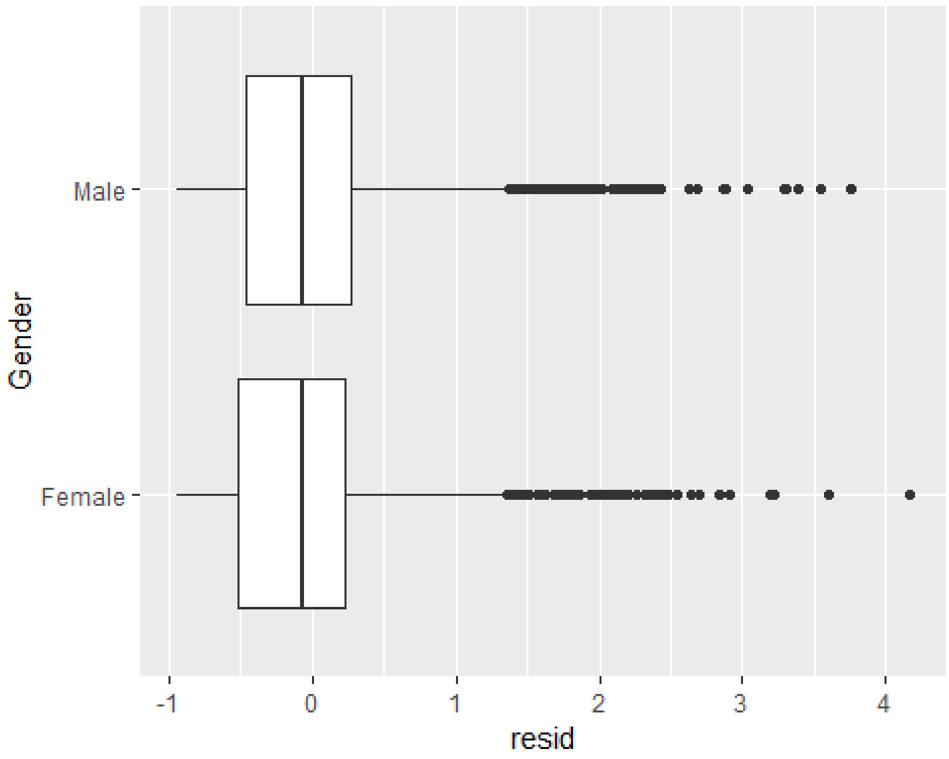


```
ggplot(augDat,aes(x=AGE,y=resid))+geom_point()+coord_flip()
+geom_smooth(method=lm)
```

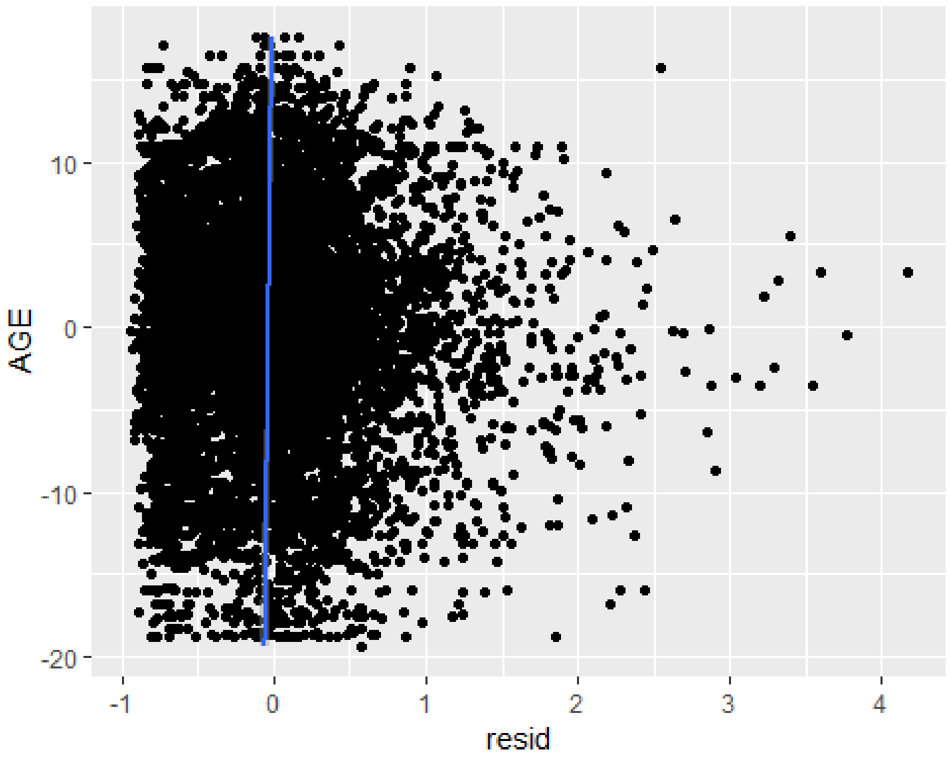


```
ggplot(augDat,aes(x=edu.cat,y=resid))+geom_boxplot()+coord_flip()
```

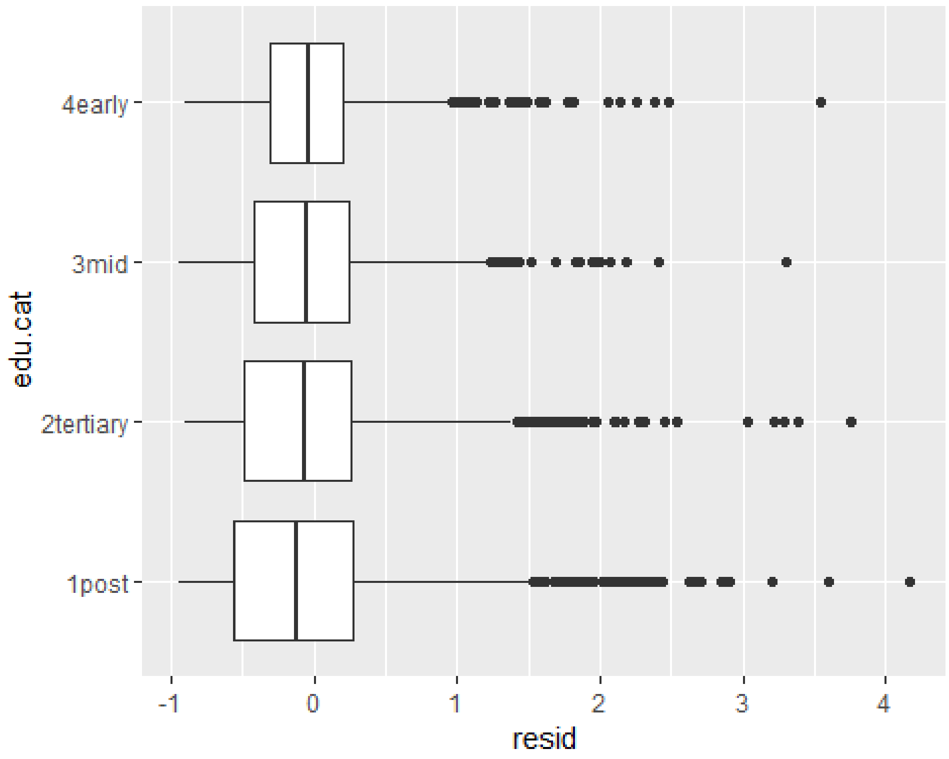


```
ggplot(augDat,aes(x=diagn,y=resid))+geom_boxplot()+coord_flip()
```

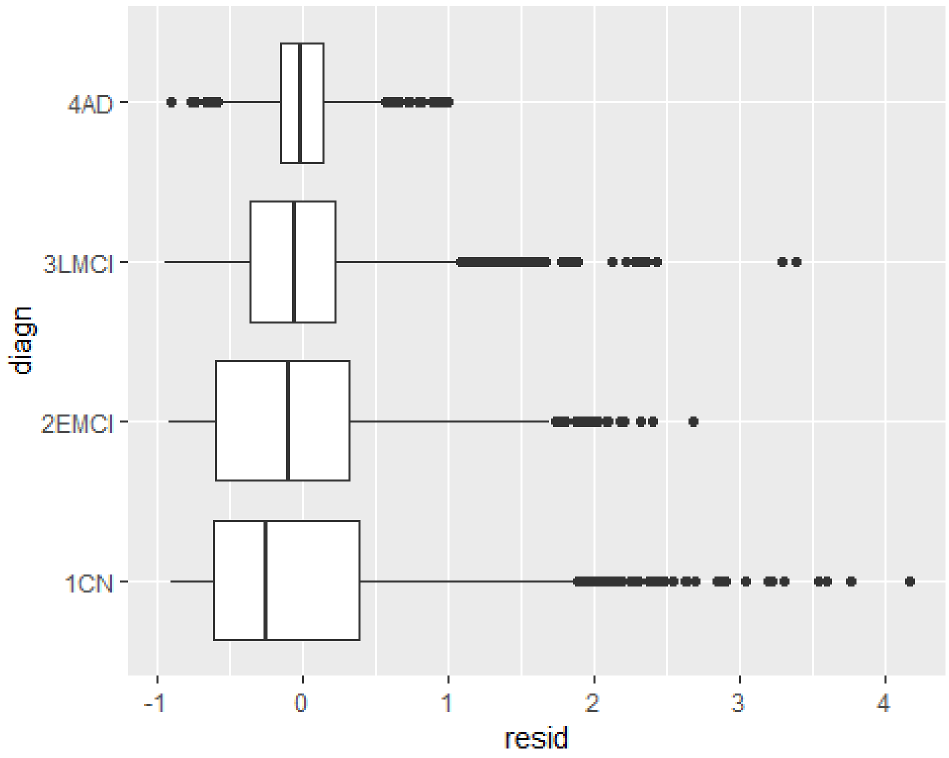


```
ggplot(augDat,aes(x=as.factor(APOE4),y=resid))+geom_boxplot()+coord_flip()
```

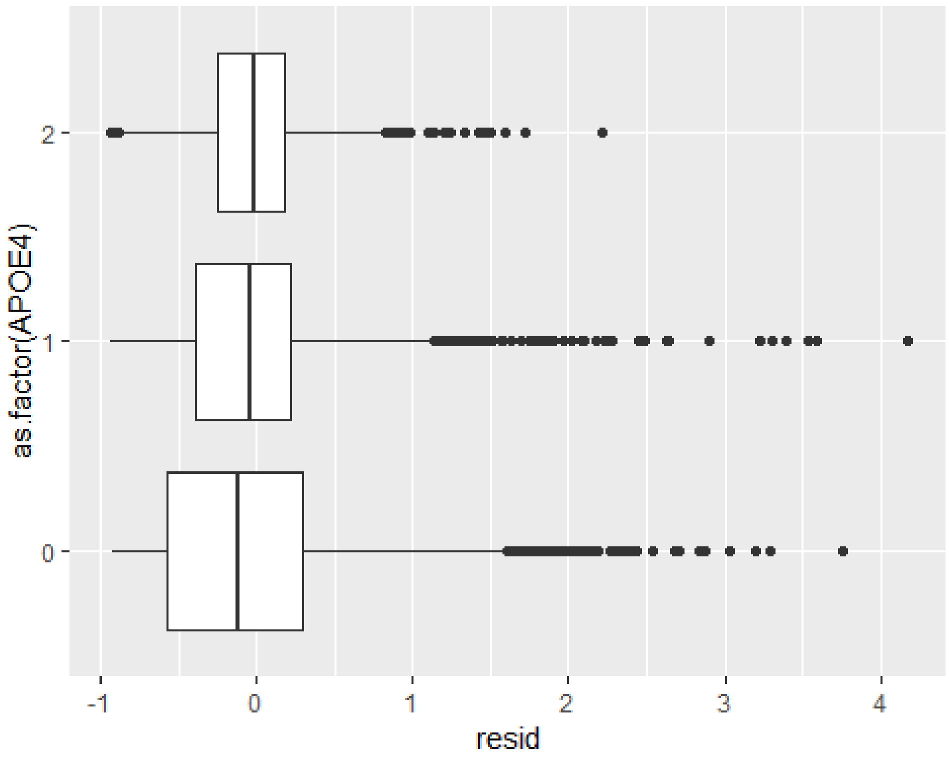


```
ggplot(augDat,aes(x=as.factor(M),y=resid))+geom_boxplot()+coord_flip()
```

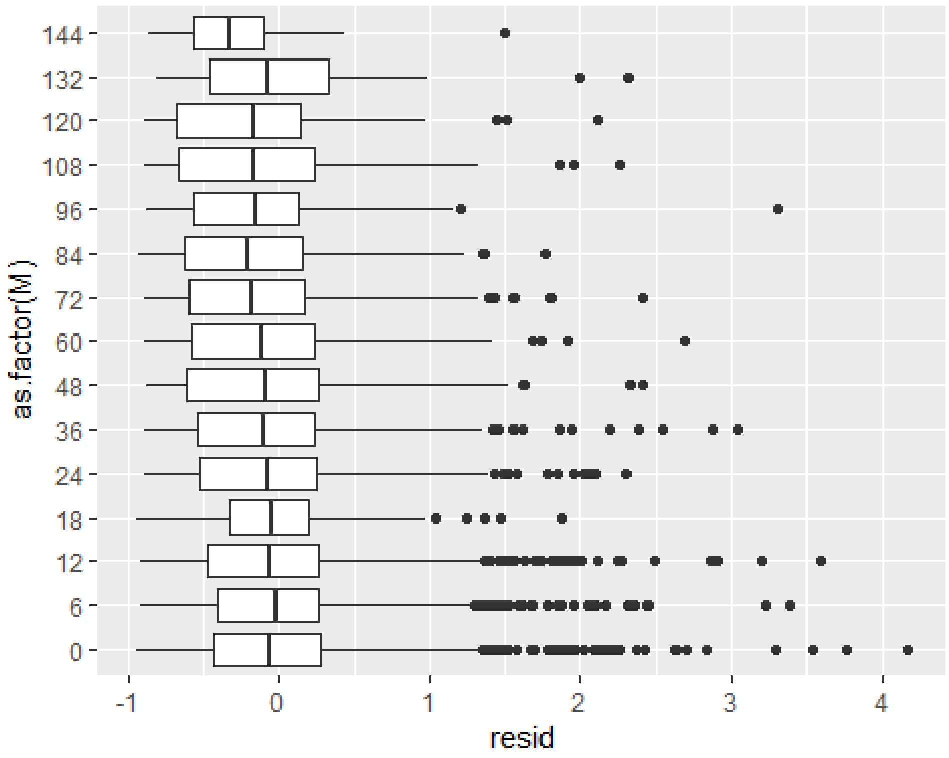


```
ggplot(augDat,aes(x=as.factor(diclo),y=resid))+geom_boxplot()+coord_flip()
```

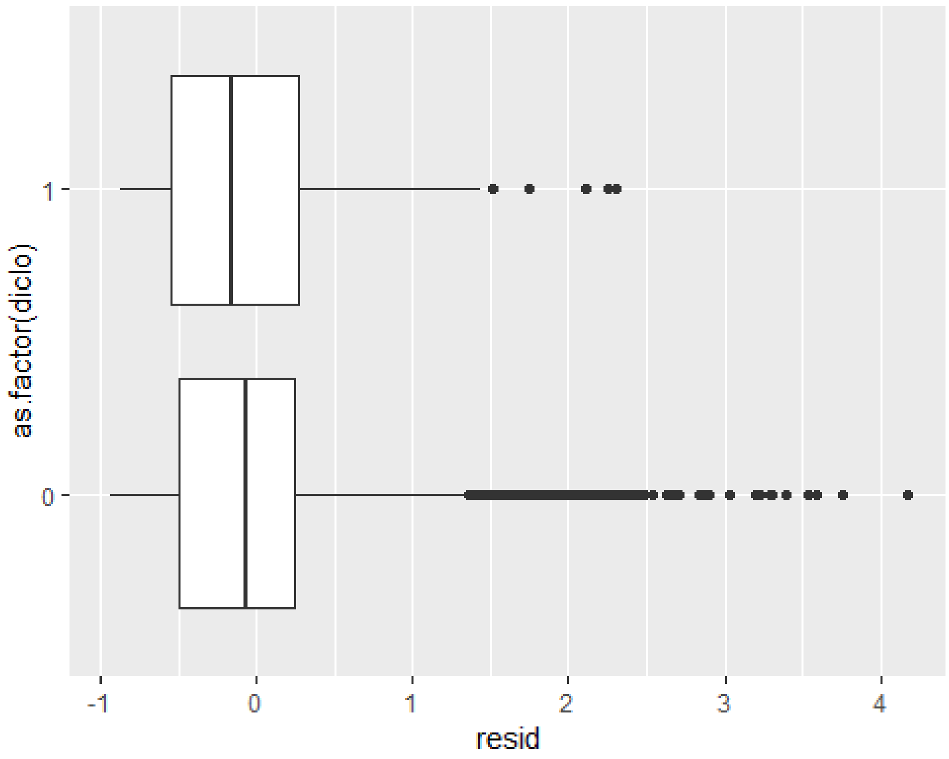


```
ggplot(augDat,aes(x=as.factor(parac),y=resid))+geom_boxplot()+coord_flip()
```

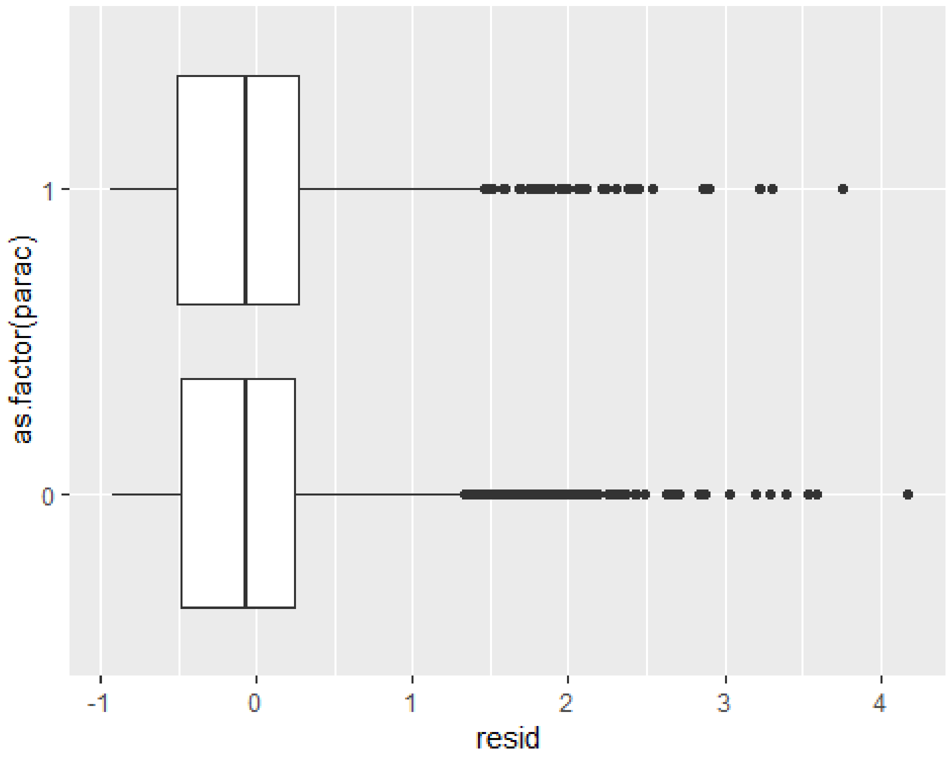

7.22. *Checking for multicolinearity*

```
cov2cor(vcov(neg.mmse))
```

```
                    (Intercept)       M     AGE diagn2EMCI diagn3LMCI
(Intercept)              1.0000  0.1126 -0.0395    -0.0077    -0.0440
M                        0.1126  1.0000  0.0134    -0.1140    -0.1141
AGE                     -0.0395  0.0134  1.0000    -0.0530    -0.0176
diagn2EMCI              -0.0077 -0.1140 -0.0530     1.0000     0.0125
diagn3LMCI              -0.0440 -0.1141 -0.0176     0.0125     1.0000
diagn4AD                -0.2847 -0.2739 -0.0324     0.1227     0.2973
APOE41                  -0.0292 -0.0585 -0.0090    -0.0159    -0.0088
APOE42                  -0.0300 -0.0538  0.0282     0.0015    -0.0108
edu.cat2tertiary        -0.0027  0.0081  0.0014     0.0127    -0.0049
edu.cat3mid             -0.0176  0.0097  0.0244     0.0020     0.0009
edu.cat4early           -0.0274  0.0119  0.0045    -0.0160     0.0123
aspirin                  0.0639  0.1479  0.0231    -0.0235     0.0222
GenderMale              -0.0131  0.0194 -0.0073    -0.0048     0.0128
parac                    0.0569  0.1074  0.0125    -0.0030     0.0156
diclo                    0.0204  0.0681  0.0041    -0.0174    -0.0015
M:diagn2EMCI             0.0055 -0.0467  0.0030    -0.0099    -0.0066
M:diagn3LMCI             0.0153 -0.3742 -0.0007    -0.0161    -0.0175
M:diagn4AD               0.0084 -0.2487 -0.0042    -0.0085    -0.0066
M:APOE41                -0.0141 -0.1019  0.0019     0.0011     0.0033
M:APOE42                -0.0061 -0.1031 -0.0050    -0.0013    -0.0022
M:GenderMale             0.0030  0.0403  0.0027    -0.0010     0.0030
M:edu.cat2tertiary       0.0126  0.0259 -0.0028    -0.0020    -0.0054
M:edu.cat3mid            0.0089 -0.0574 -0.0012    -0.0019    -0.0001
M:edu.cat4early          0.0170 -0.0852  0.0013    -0.0015     0.0022
M:parac                 -0.0109 -0.0618  0.0014     0.0012    -0.0044
M:diclo                  0.0062  0.0183 -0.0002    -0.0009    -0.0053
                    diagn4AD  APOE41  APOE42 edu.cat2tertiary edu.cat3mid
(Intercept)          -0.2847 -0.0292 -0.0300          -0.0027     -0.0176
M                    -0.2739 -0.0585 -0.0538           0.0081      0.0097
AGE                  -0.0324 -0.0090  0.0282           0.0014      0.0244
diagn2EMCI            0.1227 -0.0159  0.0015           0.0127      0.0020
diagn3LMCI            0.2973 -0.0088 -0.0108          -0.0049      0.0009
diagn4AD              1.0000  0.0059  0.0170          -0.0071      0.0167
APOE41                0.0059  1.0000  0.0633           0.0233      0.0055
APOE42                0.0170  0.0633  1.0000          -0.0027      0.0147
edu.cat2tertiary     -0.0071  0.0233 -0.0027           1.0000      0.0068
edu.cat3mid           0.0167  0.0055  0.0147           0.0068      1.0000
edu.cat4early         0.0197  0.0043  0.0040           0.0448      0.0518
aspirin               0.0111 -0.0148  0.0047          -0.0163     -0.0247
GenderMale            0.0104  0.0068  0.0075           0.0036     -0.0104
parac                 0.0053  0.0107  0.0180          -0.0010      0.0121
diclo                -0.0057 -0.0179  0.0082           0.0021     -0.0239
M:diagn2EMCI         -0.0162 -0.0024 -0.0034          -0.0018     -0.0020
M:diagn3LMCI         -0.0817  0.0002 -0.0056          -0.0093      0.0007
M:diagn4AD           -0.0589 -0.0050 -0.0096           0.0005     -0.0025
M:APOE41             -0.0118  0.0186  0.0079          -0.0086     -0.0013
M:APOE42             -0.0194  0.0062  0.0110          -0.0009     -0.0032
M:GenderMale          0.0064  0.0011 -0.0012           0.0020     -0.0066
M:edu.cat2tertiary   -0.0017 -0.0077 -0.0022           0.0367     -0.0029
M:edu.cat3mid        -0.0158 -0.0001 -0.0030          -0.0036      0.0225
M:edu.cat4early      -0.0217  0.0007 -0.0019          -0.0039     -0.0049
M:parac              -0.0107 -0.0003  0.0010          -0.0051      0.0003
M:diclo              -0.0028  0.0024 -0.0022           0.0043      0.0007
                    edu.cat4early aspirin GenderMale   parac   diclo
(Intercept)               -0.0274  0.0639    -0.0131  0.0569  0.0204
M                          0.0119  0.1479     0.0194  0.1074  0.0681
AGE                        0.0045  0.0231    -0.0073  0.0125  0.0041
diagn2EMCI                -0.0160 -0.0235    -0.0048 -0.0030 -0.0174
```

```
diagn3LMCI              0.0123  0.0222     0.0128  0.0156 -0.0015
diagn4AD                0.0197  0.0111     0.0104  0.0053 -0.0057
APOE41                  0.0043 -0.0148     0.0068  0.0107 -0.0179
APOE42                  0.0040  0.0047     0.0075  0.0180  0.0082
edu.cat2tertiary        0.0448 -0.0163     0.0036 -0.0010  0.0021
edu.cat3mid             0.0518 -0.0247    -0.0104  0.0121 -0.0239
edu.cat4early           1.0000  0.0034     0.0050  0.0163  0.0104
aspirin                 0.0034  1.0000     0.0219 -0.0028  0.0188
GenderMale              0.0050  0.0219     1.0000  0.0020  0.0052
parac                   0.0163 -0.0028     0.0020  1.0000  0.0221
diclo                   0.0104  0.0188     0.0052  0.0221  1.0000
M:diagn2EMCI           -0.0006  0.0010    -0.0018 -0.0007 -0.0033
M:diagn3LMCI            0.0034  0.0211     0.0017  0.0148 -0.0025
M:diagn4AD             -0.0006  0.0091     0.0001  0.0098  0.0012
M:APOE41               -0.0004  0.0068    -0.0016  0.0017  0.0035
M:APOE42               -0.0022  0.0031    -0.0043  0.0038 -0.0025
M:GenderMale           -0.0013 -0.0015     0.0275 -0.0079 -0.0018
M:edu.cat2tertiary     -0.0022  0.0034    -0.0025 -0.0021  0.0058
M:edu.cat3mid          -0.0045  0.0049     0.0009  0.0020  0.0011
M:edu.cat4early         0.0260  0.0061     0.0005  0.0035  0.0059
M:parac                -0.0016  0.0047    -0.0099  0.0082  0.0009
M:diclo                 0.0035  0.0023    -0.0006  0.0011  0.0254
                   M:diagn2EMCI M:diagn3LMCI M:diagn4AD M:APOE41 M:APOE42
(Intercept)              0.0055       0.0153     0.0084  -0.0141  -0.0061
M                       -0.0467      -0.3742    -0.2487  -0.1019  -0.1031
AGE                      0.0030      -0.0007    -0.0042   0.0019  -0.0050
diagn2EMCI              -0.0099      -0.0161    -0.0085   0.0011  -0.0013
diagn3LMCI              -0.0066      -0.0175    -0.0066   0.0033  -0.0022
diagn4AD                -0.0162      -0.0817    -0.0589  -0.0118  -0.0194
APOE41                  -0.0024       0.0002    -0.0050   0.0186   0.0062
APOE42                  -0.0034      -0.0056    -0.0096   0.0079   0.0110
edu.cat2tertiary        -0.0018      -0.0093     0.0005  -0.0086  -0.0009
edu.cat3mid             -0.0020       0.0007    -0.0025  -0.0013  -0.0032
edu.cat4early           -0.0006       0.0034    -0.0006  -0.0004  -0.0022
aspirin                  0.0010       0.0211     0.0091   0.0068   0.0031
GenderMale              -0.0018       0.0017     0.0001  -0.0016  -0.0043
parac                   -0.0007       0.0148     0.0098   0.0017   0.0038
diclo                   -0.0033      -0.0025     0.0012   0.0035  -0.0025
M:diagn2EMCI             1.0000       0.1806     0.1188  -0.0120  -0.0106
M:diagn3LMCI             0.1806       1.0000     0.2985  -0.0224  -0.0804
M:diagn4AD               0.1188       0.2985     1.0000   0.0144  -0.0051
M:APOE41                -0.0120      -0.0224     0.0144   1.0000   0.1866
M:APOE42                -0.0106      -0.0804    -0.0051   0.1866   1.0000
M:GenderMale            -0.0187       0.0480     0.0108   0.0449   0.0179
M:edu.cat2tertiary       0.0069      -0.0364     0.0014   0.0056   0.0344
M:edu.cat3mid           -0.0361       0.0582     0.0152   0.0076  -0.0395
M:edu.cat4early         -0.0312       0.0116     0.0065   0.0452   0.0586
M:parac                  0.0146      -0.0004     0.0104   0.0205   0.0075
M:diclo                 -0.0055      -0.0275    -0.0067   0.0077  -0.0909
                   M:GenderMale M:edu.cat2tertiary M:edu.cat3mid
(Intercept)              0.0030             0.0126        0.0089
M                        0.0403             0.0259       -0.0574
AGE                      0.0027            -0.0028       -0.0012
diagn2EMCI              -0.0010            -0.0020       -0.0019
diagn3LMCI               0.0030            -0.0054       -0.0001
diagn4AD                 0.0064            -0.0017       -0.0158
APOE41                   0.0011            -0.0077       -0.0001
APOE42                  -0.0012            -0.0022       -0.0030
edu.cat2tertiary         0.0020             0.0367       -0.0036
```

```
edu.cat3mid             -0.0066             -0.0029        0.0225
edu.cat4early           -0.0013             -0.0022       -0.0045
aspirin                 -0.0015              0.0034        0.0049
GenderMale               0.0275             -0.0025        0.0009
parac                   -0.0079             -0.0021        0.0020
diclo                   -0.0018              0.0058        0.0011
M:diagn2EMCI            -0.0187              0.0069       -0.0361
M:diagn3LMCI             0.0480             -0.0364        0.0582
M:diagn4AD               0.0108              0.0014        0.0152
M:APOE41                 0.0449              0.0056        0.0076
M:APOE42                 0.0179              0.0344       -0.0395
M:GenderMale             1.0000             -0.0474       -0.0650
M:edu.cat2tertiary      -0.0474              1.0000       -0.0008
M:edu.cat3mid           -0.0650             -0.0008        1.0000
M:edu.cat4early          0.0291              0.0985        0.1324
M:parac                 -0.1157             -0.0741        0.0529
M:diclo                 -0.0336              0.0676        0.0022
                   M:edu.cat4early M:parac M:diclo
(Intercept)                 0.0170 -0.0109  0.0062
M                          -0.0852 -0.0618  0.0183
AGE                         0.0013  0.0014 -0.0002
diagn2EMCI                 -0.0015  0.0012 -0.0009
diagn3LMCI                  0.0022 -0.0044 -0.0053
diagn4AD                   -0.0217 -0.0107 -0.0028
APOE41                      0.0007 -0.0003  0.0024
APOE42                     -0.0019  0.0010 -0.0022
edu.cat2tertiary           -0.0039 -0.0051  0.0043
edu.cat3mid                -0.0049  0.0003  0.0007
edu.cat4early               0.0260 -0.0016  0.0035
aspirin                     0.0061  0.0047  0.0023
GenderMale                  0.0005 -0.0099 -0.0006
parac                       0.0035  0.0082  0.0011
diclo                       0.0059  0.0009  0.0254
M:diagn2EMCI               -0.0312  0.0146 -0.0055
M:diagn3LMCI                0.0116 -0.0004 -0.0275
M:diagn4AD                  0.0065  0.0104 -0.0067
M:APOE41                    0.0452  0.0205  0.0077
M:APOE42                    0.0586  0.0075 -0.0909
M:GenderMale                0.0291 -0.1157 -0.0336
M:edu.cat2tertiary          0.0985 -0.0741  0.0676
M:edu.cat3mid               0.1324  0.0529  0.0022
M:edu.cat4early             1.0000  0.0616  0.0572
M:parac                     0.0616  1.0000 -0.0054
M:diclo                     0.0572 -0.0054  1.0000

abs(cov2cor(vcov(neg.mmse)))>0.4

                   (Intercept)     M   AGE diagn2EMCI diagn3LMCI diagn4AD
(Intercept)               TRUE FALSE FALSE      FALSE      FALSE    FALSE
M                        FALSE  TRUE FALSE      FALSE      FALSE    FALSE
AGE                      FALSE FALSE  TRUE      FALSE      FALSE    FALSE
diagn2EMCI               FALSE FALSE FALSE       TRUE      FALSE    FALSE
diagn3LMCI               FALSE FALSE FALSE      FALSE       TRUE    FALSE
diagn4AD                 FALSE FALSE FALSE      FALSE      FALSE     TRUE
APOE41                   FALSE FALSE FALSE      FALSE      FALSE    FALSE
APOE42                   FALSE FALSE FALSE      FALSE      FALSE    FALSE
edu.cat2tertiary         FALSE FALSE FALSE      FALSE      FALSE    FALSE
edu.cat3mid              FALSE FALSE FALSE      FALSE      FALSE    FALSE
edu.cat4early            FALSE FALSE FALSE      FALSE      FALSE    FALSE
```

```
aspirin                  FALSE FALSE FALSE      FALSE      FALSE    FALSE
GenderMale               FALSE FALSE FALSE      FALSE      FALSE    FALSE
parac                    FALSE FALSE FALSE      FALSE      FALSE    FALSE
diclo                    FALSE FALSE FALSE      FALSE      FALSE    FALSE
M:diagn2EMCI             FALSE FALSE FALSE      FALSE      FALSE    FALSE
M:diagn3LMCI             FALSE FALSE FALSE      FALSE      FALSE    FALSE
M:diagn4AD               FALSE FALSE FALSE      FALSE      FALSE    FALSE
M:APOE41                 FALSE FALSE FALSE      FALSE      FALSE    FALSE
M:APOE42                 FALSE FALSE FALSE      FALSE      FALSE    FALSE
M:GenderMale             FALSE FALSE FALSE      FALSE      FALSE    FALSE
M:edu.cat2tertiary       FALSE FALSE FALSE      FALSE      FALSE    FALSE
M:edu.cat3mid            FALSE FALSE FALSE      FALSE      FALSE    FALSE
M:edu.cat4early          FALSE FALSE FALSE      FALSE      FALSE    FALSE
M:parac                  FALSE FALSE FALSE      FALSE      FALSE    FALSE
M:diclo                  FALSE FALSE FALSE      FALSE      FALSE    FALSE
                   APOE41 APOE42 edu.cat2tertiary edu.cat3mid
(Intercept)         FALSE  FALSE            FALSE       FALSE
M                   FALSE  FALSE            FALSE       FALSE
AGE                 FALSE  FALSE            FALSE       FALSE
diagn2EMCI          FALSE  FALSE            FALSE       FALSE
diagn3LMCI          FALSE  FALSE            FALSE       FALSE
diagn4AD            FALSE  FALSE            FALSE       FALSE
APOE41               TRUE  FALSE            FALSE       FALSE
APOE42              FALSE   TRUE            FALSE       FALSE
edu.cat2tertiary    FALSE  FALSE             TRUE       FALSE
edu.cat3mid         FALSE  FALSE            FALSE        TRUE
edu.cat4early       FALSE  FALSE            FALSE       FALSE
aspirin             FALSE  FALSE            FALSE       FALSE
GenderMale          FALSE  FALSE            FALSE       FALSE
parac               FALSE  FALSE            FALSE       FALSE
diclo               FALSE  FALSE            FALSE       FALSE
M:diagn2EMCI        FALSE  FALSE            FALSE       FALSE
M:diagn3LMCI        FALSE  FALSE            FALSE       FALSE
M:diagn4AD          FALSE  FALSE            FALSE       FALSE
M:APOE41            FALSE  FALSE            FALSE       FALSE
M:APOE42            FALSE  FALSE            FALSE       FALSE
M:GenderMale        FALSE  FALSE            FALSE       FALSE
M:edu.cat2tertiary  FALSE  FALSE            FALSE       FALSE
M:edu.cat3mid       FALSE  FALSE            FALSE       FALSE
M:edu.cat4early     FALSE  FALSE            FALSE       FALSE
M:parac             FALSE  FALSE            FALSE       FALSE
M:diclo             FALSE  FALSE            FALSE       FALSE
                   edu.cat4early aspirin GenderMale parac diclo
(Intercept)                FALSE   FALSE      FALSE FALSE FALSE
M                          FALSE   FALSE      FALSE FALSE FALSE
AGE                        FALSE   FALSE      FALSE FALSE FALSE
diagn2EMCI                 FALSE   FALSE      FALSE FALSE FALSE
diagn3LMCI                 FALSE   FALSE      FALSE FALSE FALSE
diagn4AD                   FALSE   FALSE      FALSE FALSE FALSE
APOE41                     FALSE   FALSE      FALSE FALSE FALSE
APOE42                     FALSE   FALSE      FALSE FALSE FALSE
edu.cat2tertiary           FALSE   FALSE      FALSE FALSE FALSE
edu.cat3mid                FALSE   FALSE      FALSE FALSE FALSE
edu.cat4early               TRUE   FALSE      FALSE FALSE FALSE
aspirin                    FALSE    TRUE      FALSE FALSE FALSE
GenderMale                 FALSE   FALSE       TRUE FALSE FALSE
parac                      FALSE   FALSE      FALSE  TRUE FALSE
diclo                      FALSE   FALSE      FALSE FALSE  TRUE
M:diagn2EMCI               FALSE   FALSE      FALSE FALSE FALSE
```

```
M:diagn3LMCI            FALSE   FALSE      FALSE FALSE FALSE
M:diagn4AD              FALSE   FALSE      FALSE FALSE FALSE
M:APOE41                FALSE   FALSE      FALSE FALSE FALSE
M:APOE42                FALSE   FALSE      FALSE FALSE FALSE
M:GenderMale            FALSE   FALSE      FALSE FALSE FALSE
M:edu.cat2tertiary      FALSE   FALSE      FALSE FALSE FALSE
M:edu.cat3mid           FALSE   FALSE      FALSE FALSE FALSE
M:edu.cat4early         FALSE   FALSE      FALSE FALSE FALSE
M:parac                 FALSE   FALSE      FALSE FALSE FALSE
M:diclo                 FALSE   FALSE      FALSE FALSE FALSE
                   M:diagn2EMCI M:diagn3LMCI M:diagn4AD M:APOE41 M:APOE42
(Intercept)               FALSE        FALSE      FALSE    FALSE    FALSE
M                         FALSE        FALSE      FALSE    FALSE    FALSE
AGE                       FALSE        FALSE      FALSE    FALSE    FALSE
diagn2EMCI                FALSE        FALSE      FALSE    FALSE    FALSE
diagn3LMCI                FALSE        FALSE      FALSE    FALSE    FALSE
diagn4AD                  FALSE        FALSE      FALSE    FALSE    FALSE
APOE41                    FALSE        FALSE      FALSE    FALSE    FALSE
APOE42                    FALSE        FALSE      FALSE    FALSE    FALSE
edu.cat2tertiary          FALSE        FALSE      FALSE    FALSE    FALSE
edu.cat3mid               FALSE        FALSE      FALSE    FALSE    FALSE
edu.cat4early             FALSE        FALSE      FALSE    FALSE    FALSE
aspirin                   FALSE        FALSE      FALSE    FALSE    FALSE
GenderMale                FALSE        FALSE      FALSE    FALSE    FALSE
parac                     FALSE        FALSE      FALSE    FALSE    FALSE
diclo                     FALSE        FALSE      FALSE    FALSE    FALSE
M:diagn2EMCI               TRUE        FALSE      FALSE    FALSE    FALSE
M:diagn3LMCI              FALSE         TRUE      FALSE    FALSE    FALSE
M:diagn4AD                FALSE        FALSE       TRUE    FALSE    FALSE
M:APOE41                  FALSE        FALSE      FALSE     TRUE    FALSE
M:APOE42                  FALSE        FALSE      FALSE    FALSE     TRUE
M:GenderMale              FALSE        FALSE      FALSE    FALSE    FALSE
M:edu.cat2tertiary        FALSE        FALSE      FALSE    FALSE    FALSE
M:edu.cat3mid             FALSE        FALSE      FALSE    FALSE    FALSE
M:edu.cat4early           FALSE        FALSE      FALSE    FALSE    FALSE
M:parac                   FALSE        FALSE      FALSE    FALSE    FALSE
M:diclo                   FALSE        FALSE      FALSE    FALSE    FALSE
                   M:GenderMale M:edu.cat2tertiary M:edu.cat3mid
(Intercept)               FALSE              FALSE         FALSE
M                         FALSE              FALSE         FALSE
AGE                       FALSE              FALSE         FALSE
diagn2EMCI                FALSE              FALSE         FALSE
diagn3LMCI                FALSE              FALSE         FALSE
diagn4AD                  FALSE              FALSE         FALSE
APOE41                    FALSE              FALSE         FALSE
APOE42                    FALSE              FALSE         FALSE
edu.cat2tertiary          FALSE              FALSE         FALSE
edu.cat3mid               FALSE              FALSE         FALSE
edu.cat4early             FALSE              FALSE         FALSE
aspirin                   FALSE              FALSE         FALSE
GenderMale                FALSE              FALSE         FALSE
parac                     FALSE              FALSE         FALSE
diclo                     FALSE              FALSE         FALSE
M:diagn2EMCI              FALSE              FALSE         FALSE
M:diagn3LMCI              FALSE              FALSE         FALSE
M:diagn4AD                FALSE              FALSE         FALSE
M:APOE41                  FALSE              FALSE         FALSE
M:APOE42                  FALSE              FALSE         FALSE
M:GenderMale               TRUE              FALSE         FALSE
```

```
M:edu.cat2tertiary        FALSE              TRUE          FALSE
M:edu.cat3mid             FALSE             FALSE           TRUE
M:edu.cat4early           FALSE             FALSE          FALSE
M:parac                   FALSE             FALSE          FALSE
M:diclo                   FALSE             FALSE          FALSE
                   M:edu.cat4early M:parac M:diclo
(Intercept)                  FALSE   FALSE   FALSE
M                            FALSE   FALSE   FALSE
AGE                          FALSE   FALSE   FALSE
diagn2EMCI                   FALSE   FALSE   FALSE
diagn3LMCI                   FALSE   FALSE   FALSE
diagn4AD                     FALSE   FALSE   FALSE
APOE41                       FALSE   FALSE   FALSE
APOE42                       FALSE   FALSE   FALSE
edu.cat2tertiary             FALSE   FALSE   FALSE
edu.cat3mid                  FALSE   FALSE   FALSE
edu.cat4early                FALSE   FALSE   FALSE
aspirin                      FALSE   FALSE   FALSE
GenderMale                   FALSE   FALSE   FALSE
parac                        FALSE   FALSE   FALSE
diclo                        FALSE   FALSE   FALSE
M:diagn2EMCI                 FALSE   FALSE   FALSE
M:diagn3LMCI                 FALSE   FALSE   FALSE
M:diagn4AD                   FALSE   FALSE   FALSE
M:APOE41                     FALSE   FALSE   FALSE
M:APOE42                     FALSE   FALSE   FALSE
M:GenderMale                 FALSE   FALSE   FALSE
M:edu.cat2tertiary           FALSE   FALSE   FALSE
M:edu.cat3mid                FALSE   FALSE   FALSE
M:edu.cat4early               TRUE   FALSE   FALSE
M:parac                      FALSE    TRUE   FALSE
M:diclo                      FALSE   FALSE    TRUE
```

7.23. *Checking other distributions*

Now that the final model has been generated, model families were investigated to see if the negative binomial model with the parameterization method of varaince proportional to the mean method is still the most optimal model as measured by AIC.

```
try(glmerB1.mmse<-glmer(cbind(success,fail)~M+AGE+ diagn+ APOE4+edu.cat +
aspirin+ diagn*M +APOE4*M + Gender*M+ edu.cat*M +parac*M +diclo*M+(1|ID),
family="binomial", data=MMSEdata))
try(summary(glmerB1.mmse))

Generalized linear mixed model fit by maximum likelihood (Laplace
  Approximation) [glmerMod]
 Family: binomial  ( logit )
Formula:
cbind(success, fail) ~ M + AGE + diagn + APOE4 + edu.cat + aspirin +
    diagn * M + APOE4 * M + Gender * M + edu.cat * M + parac *
    M + diclo * M + (1 | ID)
   Data: MMSEdata

     AIC      BIC   logLik deviance df.resid
 34398.9  34590.4 -17172.5  34344.9     8851

Scaled residuals:
    Min      1Q  Median      3Q     Max
```

```
-7.2833 -0.5548  0.1652  0.7518  3.2329

Random effects:
 Groups Name        Variance Std.Dev.
 ID     (Intercept) 0.5915   0.7691
Number of obs: 8878, groups:  ID, 1619

Fixed effects:
                     Estimate Std. Error z value Pr(>|z|)
(Intercept)         4.0457935  0.0709888  56.992  < 2e-16 ***
M                  -0.0123987  0.0009109 -13.612  < 2e-16 ***
AGE                -0.0198226  0.0030303  -6.541 6.10e-11 ***
diagn2EMCI         -0.7589389  0.0735403 -10.320  < 2e-16 ***
diagn3LMCI         -1.3119183  0.0625963 -20.958  < 2e-16 ***
diagn4AD           -2.3705168  0.0710191 -33.379  < 2e-16 ***
APOE41             -0.1138966  0.0490337  -2.323 0.020189 *
APOE42             -0.2007254  0.0759452  -2.643 0.008217 **
edu.cat2tertiary   -0.2454184  0.0570209  -4.304 1.68e-05 ***
edu.cat3mid        -0.4230724  0.0641749  -6.592 4.32e-11 ***
edu.cat4early      -0.4060740  0.0666984  -6.088 1.14e-09 ***
aspirin             0.0916950  0.0432400   2.121 0.033955 *
GenderMale         -0.1330080  0.0467888  -2.843 0.004473 **
parac               0.1116710  0.0519934   2.148 0.031730 *
diclo              -0.0158003  0.1683552  -0.094 0.925227
M:diagn2EMCI        0.0038679  0.0010681   3.621 0.000293 ***
M:diagn3LMCI       -0.0057309  0.0007595  -7.545 4.52e-14 ***
M:diagn4AD         -0.0179992  0.0017122 -10.512  < 2e-16 ***
M:APOE41           -0.0078183  0.0006723 -11.630  < 2e-16 ***
M:APOE42           -0.0121701  0.0010775 -11.295  < 2e-16 ***
M:GenderMale        0.0032389  0.0006492   4.989 6.06e-07 ***
M:edu.cat2tertiary  0.0052033  0.0008044   6.469 9.87e-11 ***
M:edu.cat3mid       0.0032502  0.0008854   3.671 0.000241 ***
M:edu.cat4early     0.0030098  0.0009090   3.311 0.000929 ***
M:parac            -0.0013581  0.0006415  -2.117 0.034252 *
M:diclo             0.0051679  0.0017648   2.928 0.003408 **
---
Signif. codes:  0 '***' 0.001 '**' 0.01 '*' 0.05 '.' 0.1 ' ' 1


Correlation matrix not shown by default, as p = 26 > 12.
Use print(x, correlation=TRUE)  or
    vcov(x)        if you need it

convergence code: 0
Model failed to converge with max|grad| = 0.885614 (tol = 0.001, component 1)
Model is nearly unidentifiable: very large eigenvalue
 - Rescale variables?
failure to converge in 10000 evaluations

try(glmerB2.mmse<-glmer(cbind(success,fail)~M+AGE+ diagn+ APOE4+edu.cat +
aspirin+ diagn*M +APOE4*M + Gender*M+ edu.cat*M +parac*M +diclo*M+(1|ID),
family=binomial(link=probit), data=MMSEdata))
summary(glmerB2.mmse)

Generalized linear mixed model fit by maximum likelihood (Laplace
  Approximation) [glmerMod]
 Family: binomial  ( probit )
Formula:
cbind(success, fail) ~ M + AGE + diagn + APOE4 + edu.cat + aspirin +
    diagn * M + APOE4 * M + Gender * M + edu.cat * M + parac *
```

```
    M + diclo * M + (1 | ID)
   Data: MMSEdata

     AIC      BIC   logLik deviance df.resid
 34621.3  34812.8 -17283.6  34567.3     8851

Scaled residuals:
    Min      1Q  Median      3Q     Max
-7.7096 -0.5642  0.1721  0.7426  3.1295

Random effects:
 Groups Name        Variance Std.Dev.
 ID     (Intercept) 0.151    0.3886
Number of obs: 8878, groups:  ID, 1619

Fixed effects:
                     Estimate Std. Error z value Pr(>|z|)
(Intercept)         2.1256106  0.0336725  63.126  < 2e-16 ***
M                  -0.0051989  0.0003427 -15.170  < 2e-16 ***
AGE                -0.0094159  0.0015263  -6.169 6.86e-10 ***
diagn2EMCI         -0.3426741  0.0358568  -9.557  < 2e-16 ***
diagn3LMCI         -0.6127038  0.0309480 -19.798  < 2e-16 ***
diagn4AD           -1.1817406  0.0355171 -33.272  < 2e-16 ***
APOE41             -0.0522684  0.0248358  -2.105 0.035329 *
APOE42             -0.0817331  0.0386214  -2.116 0.034322 *
edu.cat2tertiary   -0.1092294  0.0284663  -3.837 0.000124 ***
edu.cat3mid        -0.1925867  0.0324290  -5.939 2.87e-09 ***
edu.cat4early      -0.1849245  0.0333194  -5.550 2.86e-08 ***
aspirin             0.0422692  0.0217214   1.946 0.051659 .
GenderMale         -0.0615304  0.0234877  -2.620 0.008801 **
parac               0.0542463  0.0263254   2.061 0.039340 *
diclo              -0.0052408  0.0835016  -0.063 0.949955
M:diagn2EMCI        0.0017444  0.0005109   3.414 0.000640 ***
M:diagn3LMCI       -0.0037724  0.0004094  -9.214  < 2e-16 ***
M:diagn4AD         -0.0118397  0.0010058 -11.771  < 2e-16 ***
M:APOE41           -0.0041963  0.0003858 -10.877  < 2e-16 ***
M:APOE42           -0.0072745  0.0005986 -12.152  < 2e-16 ***
M:GenderMale        0.0016534  0.0003486   4.743 2.10e-06 ***
M:edu.cat2tertiary  0.0023051  0.0004238   5.439 5.36e-08 ***
M:edu.cat3mid       0.0011547  0.0005149   2.243 0.024923 *
M:edu.cat4early     0.0009740  0.0004466   2.181 0.029181 *
M:parac            -0.0006795  0.0003724  -1.825 0.068016 .
M:diclo             0.0023249  0.0008882   2.618 0.008857 **
---
Signif. codes:  0 '***' 0.001 '**' 0.01 '*' 0.05 '.' 0.1 ' ' 1


Correlation matrix not shown by default, as p = 26 > 12.
Use print(x, correlation=TRUE)  or
    vcov(x)        if you need it

convergence code: 0
Model failed to converge with max|grad| = 0.985004 (tol = 0.001, component 1)
Model is nearly unidentifiable: very large eigenvalue
 - Rescale variables?
failure to converge in 10000 evaluations

glmerB3.mmse<-glmer(cbind(success,fail)~M+AGE+ diagn+ APOE4+edu.cat + aspirin+
diagn*M +APOE4*M + Gender*M+ edu.cat*M +parac*M +diclo*M+(1|ID),
```

```
family=binomial(link=cloglog), data=MMSEdata)
summary(glmerB3.mmse)

Generalized linear mixed model fit by maximum likelihood (Laplace
  Approximation) [glmerMod]
 Family: binomial  ( cloglog )
Formula:
cbind(success, fail) ~ M + AGE + diagn + APOE4 + edu.cat + aspirin +
    diagn * M + APOE4 * M + Gender * M + edu.cat * M + parac *
    M + diclo * M + (1 | ID)
   Data: MMSEdata

     AIC      BIC   logLik deviance df.resid
 35043.6  35235.1 -17494.8  34989.6     8851

Scaled residuals:
    Min      1Q  Median      3Q     Max
-8.0721 -0.5798  0.1716  0.7350  3.0193

Random effects:
 Groups Name        Variance Std.Dev.
 ID     (Intercept) 0.08917  0.2986
Number of obs: 8878, groups:  ID, 1619

Fixed effects:
                     Estimate Std. Error z value Pr(>|z|)
(Intercept)         1.4079800  0.0258504  54.467  < 2e-16 ***
M                  -0.0030783  0.0003283  -9.376  < 2e-16 ***
AGE                -0.0065993  0.0011718  -5.632 1.78e-08 ***
diagn2EMCI         -0.2309073  0.0268067  -8.614  < 2e-16 ***
diagn3LMCI         -0.4342364  0.0229659 -18.908  < 2e-16 ***
diagn4AD           -0.9011078  0.0273257 -32.977  < 2e-16 ***
APOE41             -0.0376401  0.0187520  -2.007  0.04472 *
APOE42             -0.0443933  0.0297857  -1.490  0.13611
edu.cat2tertiary   -0.0706756  0.0215905  -3.273  0.00106 **
edu.cat3mid        -0.1309874  0.0244110  -5.366 8.05e-08 ***
edu.cat4early      -0.1272049  0.0257165  -4.946 7.56e-07 ***
aspirin             0.0283460  0.0166421   1.703  0.08852 .
GenderMale         -0.0408614  0.0178665  -2.287  0.02219 *
parac               0.0394382  0.0197491   1.997  0.04583 *
diclo               0.0005850  0.0629124   0.009  0.99258
M:diagn2EMCI        0.0012201  0.0003822   3.193  0.00141 **
M:diagn3LMCI       -0.0033271  0.0002813 -11.828  < 2e-16 ***
M:diagn4AD         -0.0115027  0.0009300 -12.369  < 2e-16 ***
M:APOE41           -0.0032495  0.0002669 -12.175  < 2e-16 ***
M:APOE42           -0.0066054  0.0004940 -13.372  < 2e-16 ***
M:GenderMale        0.0011680  0.0002565   4.553 5.28e-06 ***
M:edu.cat2tertiary  0.0014060  0.0003066   4.585 4.54e-06 ***
M:edu.cat3mid       0.0004144  0.0003439   1.205  0.22826
M:edu.cat4early     0.0002567  0.0003767   0.682  0.49549
M:parac            -0.0005003  0.0002521  -1.985  0.04718 *
M:diclo             0.0014406  0.0006561   2.196  0.02811 *
---
Signif. codes:  0 '***' 0.001 '**' 0.01 '*' 0.05 '.' 0.1 ' ' 1


Correlation matrix not shown by default, as p = 26 > 12.
Use print(x, correlation=TRUE)  or
    vcov(x)        if you need it
```

```
convergence code: 0
Model failed to converge with max|grad| = 1.68004 (tol = 0.001, component 1)
Model is nearly unidentifiable: very large eigenvalue
 - Rescale variables?
failure to converge in 10000 evaluations

glmerP1.mmse<-glmer(neg.b.MMSE~M+AGE+ diagn+ APOE4+edu.cat + aspirin+ diagn*M
+APOE4*M + Gender*M+ edu.cat*M +parac*M +diclo*M+(1|ID), family="poisson",
data=MMSEdata)
summary(glmerP1.mmse)

Generalized linear mixed model fit by maximum likelihood (Laplace
  Approximation) [glmerMod]
 Family: poisson  ( log )
Formula:
neg.b.MMSE ~ M + AGE + diagn + APOE4 + edu.cat + aspirin + diagn *
    M + APOE4 * M + Gender * M + edu.cat * M + parac * M + diclo *
    M + (1 | ID)
   Data: MMSEdata

     AIC      BIC   logLik deviance df.resid
 33945.3  34136.8 -16945.6  33891.3     8851

Scaled residuals:
    Min      1Q  Median      3Q     Max
-2.4160 -0.7380 -0.1472  0.4982  6.8113

Random effects:
 Groups Name        Variance Std.Dev.
 ID     (Intercept) 0.4199   0.648
Number of obs: 8878, groups:  ID, 1619

Fixed effects:
                     Estimate Std. Error z value Pr(>|z|)
(Intercept)        -0.5843395  0.0622136  -9.392  < 2e-16 ***
M                   0.0104785  0.0008291  12.638  < 2e-16 ***
AGE                 0.0182338  0.0025949   7.027 2.11e-12 ***
diagn2EMCI          0.7032050  0.0648633  10.841  < 2e-16 ***
diagn3LMCI          1.2347291  0.0548653  22.505  < 2e-16 ***
diagn4AD            2.1017871  0.0615261  34.161  < 2e-16 ***
APOE41              0.1202217  0.0422239   2.847 0.004410 **
APOE42              0.2309916  0.0647525   3.567 0.000361 ***
edu.cat2tertiary    0.2009658  0.0492473   4.081 4.49e-05 ***
edu.cat3mid         0.3597684  0.0553364   6.501 7.95e-11 ***
edu.cat4early       0.3497061  0.0572422   6.109 1.00e-09 ***
aspirin            -0.0794993  0.0370667  -2.145 0.031972 *
GenderMale          0.0969278  0.0402826   2.406 0.016120 *
parac              -0.0956706  0.0448314  -2.134 0.032842 *
diclo               0.0201426  0.1455151   0.138 0.889906
M:diagn2EMCI       -0.0032630  0.0010011  -3.259 0.001116 **
M:diagn3LMCI        0.0029143  0.0006970   4.181 2.90e-05 ***
M:diagn4AD          0.0087605  0.0013891   6.306 2.86e-10 ***
M:APOE41            0.0058592  0.0005962   9.828  < 2e-16 ***
M:APOE42            0.0077079  0.0009010   8.555  < 2e-16 ***
M:GenderMale       -0.0017639  0.0005714  -3.087 0.002021 **
M:edu.cat2tertiary -0.0038352  0.0007163  -5.354 8.58e-08 ***
M:edu.cat3mid      -0.0023327  0.0007840  -2.975 0.002927 **
M:edu.cat4early    -0.0025846  0.0007902  -3.271 0.001072 **
M:parac             0.0013325  0.0005692   2.341 0.019227 *
```

```
M:diclo             -0.0046960  0.0015571  -3.016 0.002563 **
---
Signif. codes:  0 '***' 0.001 '**' 0.01 '*' 0.05 '.' 0.1 ' ' 1


Correlation matrix not shown by default, as p = 26 > 12.
Use print(x, correlation=TRUE)  or
    vcov(x)        if you need it

convergence code: 0
Model failed to converge with max|grad| = 1.81792 (tol = 0.001, component 1)
Model is nearly unidentifiable: very large eigenvalue
 - Rescale variables?
failure to converge in 10000 evaluations

glmerP2.mmse<-glmer(neg.b.MMSE~M+AGE+ diagn+ APOE4+edu.cat + aspirin+ diagn*M
+APOE4*M + Gender*M+ edu.cat*M +parac*M +diclo*M+(1|ID),
family=poisson(link=sqrt), data=MMSEdata)
summary(glmerP2.mmse)

Generalized linear mixed model fit by maximum likelihood (Laplace
  Approximation) [glmerMod]
 Family: poisson  ( sqrt )
Formula:
neg.b.MMSE ~ M + AGE + diagn + APOE4 + edu.cat + aspirin + diagn *
    M + APOE4 * M + Gender * M + edu.cat * M + parac * M + diclo *
    M + (1 | ID)
   Data: MMSEdata

     AIC      BIC   logLik deviance df.resid
 34526.4  34717.9 -17236.2  34472.4     8851

Scaled residuals:
    Min      1Q  Median      3Q     Max
-2.5222 -0.7037 -0.1507  0.5398  8.1956

Random effects:
 Groups Name        Variance Std.Dev.
 ID     (Intercept) 0.2783   0.5275
Number of obs: 8878, groups:  ID, 1619

Fixed effects:
                     Estimate Std. Error z value Pr(>|z|)
(Intercept)         0.7561636  0.0449700  16.815  < 2e-16 ***
M                   0.0036989  0.0005856   6.317 2.67e-10 ***
AGE                 0.0120439  0.0020975   5.742 9.36e-09 ***
diagn2EMCI          0.3869089  0.0470569   8.222  < 2e-16 ***
diagn3LMCI          0.7774911  0.0402885  19.298  < 2e-16 ***
diagn4AD            1.6545143  0.0494000  33.492  < 2e-16 ***
APOE41              0.0808192  0.0334751   2.414  0.01577 *
APOE42              0.0975871  0.0538635   1.812  0.07003 .
edu.cat2tertiary    0.1024872  0.0383281   2.674  0.00750 **
edu.cat3mid         0.2076190  0.0434798   4.775 1.80e-06 ***
edu.cat4early       0.2122161  0.0462316   4.590 4.43e-06 ***
aspirin            -0.0468205  0.0297108  -1.576  0.11505
GenderMale          0.0579500  0.0318460   1.820  0.06881 .
parac              -0.0684758  0.0351082  -1.950  0.05113 .
diclo              -0.0014112  0.1105359  -0.013  0.98981
M:diagn2EMCI       -0.0017666  0.0007131  -2.477  0.01324 *
M:diagn3LMCI        0.0062790  0.0005259  11.939  < 2e-16 ***
```

```
M:diagn4AD          0.0202745  0.0019231  10.542  < 2e-16 ***
M:APOE41            0.0055304  0.0005048  10.956  < 2e-16 ***
M:APOE42            0.0116775  0.0009721  12.012  < 2e-16 ***
M:GenderMale       -0.0013402  0.0004786  -2.801  0.00510 **
M:edu.cat2tertiary -0.0016890  0.0005594  -3.019  0.00254 **
M:edu.cat3mid       0.0004038  0.0006456   0.625  0.53170
M:edu.cat4early     0.0004154  0.0007329   0.567  0.57081
M:parac             0.0009383  0.0004712   1.991  0.04644 *
M:diclo            -0.0024562  0.0011967  -2.052  0.04012 *
---
Signif. codes:  0 '***' 0.001 '**' 0.01 '*' 0.05 '.' 0.1 ' ' 1


Correlation matrix not shown by default, as p = 26 > 12.
Use print(x, correlation=TRUE)  or
    vcov(x)        if you need it

convergence code: 0
Model failed to converge with max|grad| = 0.00891603 (tol = 0.001, component 1)
Model is nearly unidentifiable: very large eigenvalue
 - Rescale variables?

glmerB4.mmse<-glmer(cbind(success,fail)~M.Z+AGE+ diagn+ APOE4+edu.cat + aspirin+
APOE4*M.Z+edu.cat*M.Z+diagn*M.Z+ parac*M.Z+diclo*M.Z+(1|ID), family="binomial",
data=MMSEdata)
summary(glmerB4.mmse)

Generalized linear mixed model fit by maximum likelihood (Laplace
  Approximation) [glmerMod]
 Family: binomial  ( logit )
Formula: cbind(success, fail) ~ M.Z + AGE + diagn + APOE4 + edu.cat +
    aspirin + APOE4 * M.Z + edu.cat * M.Z + diagn * M.Z + parac *
    M.Z + diclo * M.Z + (1 | ID)
   Data: MMSEdata

     AIC      BIC   logLik deviance df.resid
 34421.1  34598.4 -17185.6  34371.1     8853

Scaled residuals:
    Min      1Q  Median      3Q     Max
-6.8589 -0.5523  0.1643  0.7536  3.0817

Random effects:
 Groups Name        Variance Std.Dev.
 ID     (Intercept) 0.592    0.7694
Number of obs: 8878, groups:  ID, 1619

Fixed effects:
                      Estimate Std. Error z value Pr(>|z|)
(Intercept)           3.678902   0.061378  59.938  < 2e-16 ***
M.Z                  -0.285888   0.022422 -12.750  < 2e-16 ***
AGE                  -0.020546   0.003009  -6.828 8.61e-12 ***
diagn2EMCI           -0.655686   0.067766  -9.676  < 2e-16 ***
diagn3LMCI           -1.472870   0.058049 -25.373  < 2e-16 ***
diagn4AD             -2.872303   0.073108 -39.288  < 2e-16 ***
APOE41               -0.331552   0.046513  -7.128 1.02e-12 ***
APOE42               -0.536065   0.073410  -7.302 2.83e-13 ***
edu.cat2tertiary     -0.098018   0.053741  -1.824 0.068168 .
edu.cat3mid          -0.324750   0.059668  -5.443 5.25e-08 ***
edu.cat4early        -0.308649   0.063115  -4.890 1.01e-06 ***
```

```
aspirin               0.083212   0.042858   1.942 0.052190 .
parac                 0.081212   0.048933   1.660 0.096981 .
diclo                 0.132478   0.158614   0.835 0.403592
M.Z:APOE41           -0.223431   0.018720 -11.935  < 2e-16 ***
M.Z:APOE42           -0.338140   0.030070 -11.245  < 2e-16 ***
M.Z:edu.cat2tertiary  0.141475   0.022434   6.306 2.86e-10 ***
M.Z:edu.cat3mid       0.061577   0.023968   2.569 0.010196 *
M.Z:edu.cat4early     0.061278   0.024947   2.456 0.014036 *
M.Z:diagn2EMCI        0.113832   0.029764   3.825 0.000131 ***
M.Z:diagn3LMCI       -0.150333   0.021082  -7.131 9.98e-13 ***
M.Z:diagn4AD         -0.498080   0.047735 -10.434  < 2e-16 ***
M.Z:parac            -0.039911   0.017884  -2.232 0.025640 *
M.Z:diclo             0.156443   0.049214   3.179 0.001479 **
---
Signif. codes:  0 '***' 0.001 '**' 0.01 '*' 0.05 '.' 0.1 ' ' 1


Correlation matrix not shown by default, as p = 24 > 12.
Use print(x, correlation=TRUE)  or
    vcov(x)        if you need it

convergence code: 0
Model failed to converge with max|grad| = 0.0758989 (tol = 0.001, component 1)
failure to converge in 10000 evaluations

glmerB5.mmse<-glmer(cbind(success,fail)~M.Z+AGE+ diagn+ APOE4+edu.cat + aspirin+
APOE4*M.Z+edu.cat*M.Z+diagn*M.Z+ parac*M.Z+diclo*M.Z+(1|ID),
family=binomial(link=probit), data=MMSEdata)
summary(glmerB5.mmse)

Generalized linear mixed model fit by maximum likelihood (Laplace
  Approximation) [glmerMod]
 Family: binomial  ( probit )
Formula: cbind(success, fail) ~ M.Z + AGE + diagn + APOE4 + edu.cat +
    aspirin + APOE4 * M.Z + edu.cat * M.Z + diagn * M.Z + parac *
    M.Z + diclo * M.Z + (1 | ID)
   Data: MMSEdata

     AIC      BIC   logLik deviance df.resid
 34643.0  34820.3 -17296.5  34593.0     8853

Scaled residuals:
    Min      1Q  Median      3Q     Max
-7.2759 -0.5664  0.1701  0.7453  3.0662

Random effects:
 Groups Name        Variance Std.Dev.
 ID     (Intercept) 0.1515   0.3892
Number of obs: 8878, groups:  ID, 1619

Fixed effects:
                       Estimate Std. Error  z value Pr(>|z|)
(Intercept)           1.9688096  0.0006489  3034.08   <2e-16 ***
M.Z                  -0.1155742  0.0006346  -182.11   <2e-16 ***
AGE                  -0.0096567  0.0005968   -16.18   <2e-16 ***
diagn2EMCI           -0.2912915  0.0006478  -449.63   <2e-16 ***
diagn3LMCI           -0.7147598  0.0006476 -1103.79   <2e-16 ***
diagn4AD             -1.5077939  0.0006471 -2330.04   <2e-16 ***
APOE41               -0.1687045  0.0006477  -260.48   <2e-16 ***
APOE42               -0.2831016  0.0006473  -437.35   <2e-16 ***
```

```
edu.cat2tertiary     -0.0430555  0.0006477   -66.47   <2e-16 ***
edu.cat3mid          -0.1569556  0.0006476  -242.38   <2e-16 ***
edu.cat4early        -0.1537171  0.0006473  -237.47   <2e-16 ***
aspirin               0.0419122  0.0006694    62.61   <2e-16 ***
parac                 0.0395140  0.0006476    61.01   <2e-16 ***
diclo                 0.0694188  0.0006474   107.23   <2e-16 ***
M.Z:APOE41           -0.1196262  0.0006422  -186.29   <2e-16 ***
M.Z:APOE42           -0.2033737  0.0006463  -314.65   <2e-16 ***
M.Z:edu.cat2tertiary  0.0634387  0.0006653    95.35   <2e-16 ***
M.Z:edu.cat3mid       0.0171000  0.0006665    25.66   <2e-16 ***
M.Z:edu.cat4early     0.0174339  0.0006452    27.02   <2e-16 ***
M.Z:diagn2EMCI        0.0529094  0.0006454    81.97   <2e-16 ***
M.Z:diagn3LMCI       -0.0991587  0.0006628  -149.59   <2e-16 ***
M.Z:diagn4AD         -0.3283573  0.0006468  -507.68   <2e-16 ***
M.Z:parac            -0.0206157  0.0006423   -32.10   <2e-16 ***
M.Z:diclo             0.0705545  0.0006468   109.08   <2e-16 ***
---
Signif. codes:  0 '***' 0.001 '**' 0.01 '*' 0.05 '.' 0.1 ' ' 1


Correlation matrix not shown by default, as p = 24 > 12.
Use print(x, correlation=TRUE)  or
    vcov(x)        if you need it

convergence code: 0
Model failed to converge with max|grad| = 0.119649 (tol = 0.001, component 1)
Model is nearly unidentifiable: very large eigenvalue
 - Rescale variables?

glmerB6.mmse<-glmer(cbind(success,fail)~M.Z+AGE+ diagn+ APOE4+edu.cat + aspirin+
APOE4*M.Z+edu.cat*M.Z+diagn*M.Z+ parac*M.Z+diclo*M.Z+(1|ID),
family=binomial(link=cloglog), data=MMSEdata)
summary(glmerB6.mmse)

Generalized linear mixed model fit by maximum likelihood (Laplace
  Approximation) [glmerMod]
 Family: binomial  ( cloglog )
Formula: cbind(success, fail) ~ M.Z + AGE + diagn + APOE4 + edu.cat +
    aspirin + APOE4 * M.Z + edu.cat * M.Z + diagn * M.Z + parac *
    M.Z + diclo * M.Z + (1 | ID)
   Data: MMSEdata

     AIC      BIC   logLik deviance df.resid
 35061.0  35238.2 -17505.5  35011.0     8853

Scaled residuals:
    Min      1Q  Median      3Q     Max
-7.6918 -0.5716  0.1754  0.7351  3.0497

Random effects:
 Groups Name        Variance Std.Dev.
 ID     (Intercept) 0.08935  0.2989
Number of obs: 8878, groups:  ID, 1619

Fixed effects:
                       Estimate Std. Error z value Pr(>|z|)
(Intercept)           1.319e+00  2.283e-02  57.771  < 2e-16 ***
M.Z                  -6.394e-02  7.793e-03  -8.204 2.32e-16 ***
AGE                  -6.932e-03  1.164e-03  -5.954 2.62e-09 ***
diagn2EMCI           -1.983e-01  2.528e-02  -7.845 4.31e-15 ***
```

```
diagn3LMCI           -5.258e-01  2.178e-02 -24.146  < 2e-16 ***
diagn4AD             -1.220e+00  3.138e-02 -38.870  < 2e-16 ***
APOE41               -1.286e-01  1.800e-02  -7.141 9.25e-13 ***
APOE42               -2.280e-01  2.906e-02  -7.846 4.31e-15 ***
edu.cat2tertiary     -3.183e-02  2.063e-02  -1.543 0.122899
edu.cat3mid          -1.193e-01  2.301e-02  -5.185 2.16e-07 ***
edu.cat4early        -1.187e-01  2.461e-02  -4.824 1.41e-06 ***
aspirin               2.663e-02  1.650e-02   1.614 0.106528
parac                 2.720e-02  1.880e-02   1.447 0.148006
diclo                 4.783e-02  5.993e-02   0.798 0.424866
M.Z:APOE41           -9.088e-02  7.443e-03 -12.211  < 2e-16 ***
M.Z:APOE42           -1.825e-01  1.378e-02 -13.246  < 2e-16 ***
M.Z:edu.cat2tertiary  3.677e-02  8.543e-03   4.304 1.67e-05 ***
M.Z:edu.cat3mid       2.292e-04  9.279e-03   0.025 0.980291
M.Z:edu.cat4early     2.401e-05  1.039e-02   0.002 0.998156
M.Z:diagn2EMCI        3.573e-02  1.065e-02   3.354 0.000798 ***
M.Z:diagn3LMCI       -8.866e-02  7.788e-03 -11.384  < 2e-16 ***
M.Z:diagn4AD         -3.213e-01  2.594e-02 -12.387  < 2e-16 ***
M.Z:parac            -1.640e-02  7.011e-03  -2.339 0.019322 *
M.Z:diclo             4.251e-02  1.830e-02   2.322 0.020222 *
---
Signif. codes:  0 '***' 0.001 '**' 0.01 '*' 0.05 '.' 0.1 ' ' 1


Correlation matrix not shown by default, as p = 24 > 12.
Use print(x, correlation=TRUE)  or
    vcov(x)        if you need it

convergence code: 0
Model failed to converge with max|grad| = 0.105304 (tol = 0.001, component 1)
Model is nearly unidentifiable: very large eigenvalue
 - Rescale variables?

glmerP3.mmse<-glmer(neg.b.MMSE~M.Z+AGE+ diagn+ APOE4+edu.cat + aspirin+
APOE4*M.Z+edu.cat*M.Z+diagn*M.Z+ parac*M.Z+diclo*M.Z+(1|ID), family="poisson",
data=MMSEdata)
summary(glmerP3.mmse)

Generalized linear mixed model fit by maximum likelihood (Laplace
  Approximation) [glmerMod]
 Family: poisson  ( log )
Formula: neg.b.MMSE ~ M.Z + AGE + diagn + APOE4 + edu.cat + aspirin +
    APOE4 * M.Z + edu.cat * M.Z + diagn * M.Z + parac * M.Z +
    diclo * M.Z + (1 | ID)
   Data: MMSEdata

     AIC      BIC   logLik deviance df.resid
 33952.6  34129.9 -16951.3  33902.6     8853

Scaled residuals:
    Min      1Q  Median      3Q     Max
-2.3556 -0.7372 -0.1470  0.4992  6.5483

Random effects:
 Groups Name        Variance Std.Dev.
 ID     (Intercept) 0.4215   0.6492
Number of obs: 8878, groups:  ID, 1619

Fixed effects:
                      Estimate Std. Error z value Pr(>|z|)
```

```
(Intercept)          -0.266011   0.053394  -4.982 6.29e-07 ***
M.Z                   0.261652   0.020692  12.645  < 2e-16 ***
AGE                   0.018565   0.002579   7.198 6.13e-13 ***
diagn2EMCI            0.602959   0.058926  10.233  < 2e-16 ***
diagn3LMCI            1.311641   0.050321  26.066  < 2e-16 ***
diagn4AD              2.341503   0.061768  37.908  < 2e-16 ***
APOE41                0.285208   0.039880   7.152 8.58e-13 ***
APOE42                0.442039   0.062491   7.074 1.51e-12 ***
edu.cat2tertiary      0.094624   0.046191   2.049 0.040507 *
edu.cat3mid           0.287348   0.051203   5.612 2.00e-08 ***
edu.cat4early         0.273695   0.053998   5.069 4.01e-07 ***
aspirin              -0.069264   0.036780  -1.883 0.059673 .
parac                -0.063644   0.041987  -1.516 0.129568
diclo                -0.127397   0.136759  -0.932 0.351575
M.Z:APOE41            0.164303   0.016594   9.901  < 2e-16 ***
M.Z:APOE42            0.211186   0.025137   8.401  < 2e-16 ***
M.Z:edu.cat2tertiary -0.106002   0.019982  -5.305 1.13e-07 ***
M.Z:edu.cat3mid      -0.049266   0.021291  -2.314 0.020670 *
M.Z:edu.cat4early    -0.059933   0.021612  -2.773 0.005551 **
M.Z:diagn2EMCI       -0.096435   0.027902  -3.456 0.000548 ***
M.Z:diagn3LMCI        0.077277   0.019365   3.991 6.59e-05 ***
M.Z:diagn4AD          0.243934   0.038728   6.299 3.00e-10 ***
M.Z:parac             0.036374   0.015872   2.292 0.021919 *
M.Z:diclo            -0.134572   0.043383  -3.102 0.001923 **
---
Signif. codes:  0 '***' 0.001 '**' 0.01 '*' 0.05 '.' 0.1 ' ' 1


Correlation matrix not shown by default, as p = 24 > 12.
Use print(x, correlation=TRUE)  or
    vcov(x)        if you need it

convergence code: 0
Model failed to converge with max|grad| = 0.32314 (tol = 0.001, component 1)
failure to converge in 10000 evaluations

glmerP4.mmse<-glmer(neg.b.MMSE~M.Z+AGE+ diagn+ APOE4+edu.cat + aspirin+
APOE4*M.Z+edu.cat*M.Z+diagn*M.Z+ parac*M.Z+diclo*M.Z+(1|ID),
family=poisson(link=sqrt), data=MMSEdata)
summary(glmerP4.mmse)

Generalized linear mixed model fit by maximum likelihood (Laplace
  Approximation) [glmerMod]
 Family: poisson  ( sqrt )
Formula: neg.b.MMSE ~ M.Z + AGE + diagn + APOE4 + edu.cat + aspirin +
    APOE4 * M.Z + edu.cat * M.Z + diagn * M.Z + parac * M.Z +
    diclo * M.Z + (1 | ID)
   Data: MMSEdata

     AIC      BIC   logLik deviance df.resid
 34523.4  34700.6 -17236.7  34473.4     8853

Scaled residuals:
    Min      1Q  Median      3Q     Max
-2.5553 -0.7035 -0.1523  0.5356  7.9587

Random effects:
 Groups Name        Variance Std.Dev.
 ID     (Intercept) 0.2759   0.5252
Number of obs: 8878, groups:  ID, 1619
```

```
Fixed effects:
                      Estimate Std. Error z value Pr(>|z|)    
(Intercept)           0.867786   0.039902  21.748  < 2e-16 ***
M.Z                   0.077963   0.013609   5.729 1.01e-08 ***
AGE                   0.012370   0.002075   5.963 2.48e-09 ***
diagn2EMCI            0.336801   0.044333   7.597 3.03e-14 ***
diagn3LMCI            0.948660   0.038292  24.775  < 2e-16 ***
diagn4AD              2.212926   0.058943  37.544  < 2e-16 ***
APOE41                0.238021   0.032153   7.403 1.33e-13 ***
APOE42                0.422553   0.052432   8.059 7.69e-16 ***
edu.cat2tertiary      0.058226   0.036649   1.589  0.11211    
edu.cat3mid           0.215115   0.040941   5.254 1.49e-07 ***
edu.cat4early         0.219827   0.044133   4.981 6.33e-07 ***
aspirin              -0.041452   0.029324  -1.414  0.15749    
parac                -0.041226   0.033369  -1.235  0.21666    
diclo                -0.076505   0.105054  -0.728  0.46647    
M.Z:APOE41            0.155192   0.014108  11.001  < 2e-16 ***
M.Z:APOE42            0.324422   0.027105  11.969  < 2e-16 ***
M.Z:edu.cat2tertiary -0.043559   0.015558  -2.800  0.00511 ** 
M.Z:edu.cat3mid       0.024040   0.017363   1.385  0.16619    
M.Z:edu.cat4early     0.020095   0.020158   0.997  0.31881    
M.Z:diagn2EMCI       -0.051406   0.019847  -2.590  0.00960 ** 
M.Z:diagn3LMCI        0.169363   0.014533  11.654  < 2e-16 ***
M.Z:diagn4AD          0.563872   0.053635  10.513  < 2e-16 ***
M.Z:parac             0.029491   0.013104   2.250  0.02442 *  
M.Z:diclo            -0.071808   0.033401  -2.150  0.03157 *  
---
Signif. codes:  0 '***' 0.001 '**' 0.01 '*' 0.05 '.' 0.1 ' ' 1


Correlation matrix not shown by default, as p = 24 > 12.
Use print(x, correlation=TRUE)  or
    vcov(x)        if you need it

lme1.mmse<-lmer(neg.b.MMSE~M.Z+AGE+ diagn+ APOE4+edu.cat + aspirin+
APOE4*M.Z+edu.cat*M.Z+diagn*M.Z+ parac*M.Z+diclo*M.Z+(1|ID), data=MMSEdata)
summary(lme1.mmse)

Linear mixed model fit by REML. t-tests use Satterthwaite's method [
lmerModLmerTest]
Formula: neg.b.MMSE ~ M.Z + AGE + diagn + APOE4 + edu.cat + aspirin +
    APOE4 * M.Z + edu.cat * M.Z + diagn * M.Z + parac * M.Z +
    diclo * M.Z + (1 | ID)
   Data: MMSEdata

REML criterion at convergence: 41677.5

Scaled residuals:
    Min      1Q  Median      3Q     Max 
-4.0635 -0.4687 -0.0956  0.3871  8.3206 

Random effects:
 Groups   Name        Variance Std.Dev.
 ID       (Intercept) 4.831    2.198   
 Residual             4.564    2.136   
Number of obs: 8878, groups:  ID, 1619

Fixed effects:
                       Estimate Std. Error         df t value Pr(>|t|)
```

```
(Intercept)            8.132e-01  1.671e-01  1.579e+03   4.867 1.24e-06 ***
M.Z                    1.721e-01  5.700e-02  7.863e+03   3.020 0.002537 **
AGE                    2.809e-02  8.704e-03  1.634e+03   3.227 0.001275 **
diagn2EMCI             8.443e-01  1.855e-01  1.573e+03   4.552 5.73e-06 ***
diagn3LMCI             3.245e+00  1.602e-01  1.543e+03  20.255  < 2e-16 ***
diagn4AD               1.013e+01  2.481e-01  3.961e+03  40.835  < 2e-16 ***
APOE41                 8.820e-01  1.346e-01  1.635e+03   6.551 7.64e-11 ***
APOE42                 1.684e+00  2.199e-01  1.748e+03   7.659 3.08e-14 ***
edu.cat2tertiary       8.471e-02  1.534e-01  1.613e+03   0.552 0.580928
edu.cat3mid            6.493e-01  1.715e-01  1.610e+03   3.786 0.000159 ***
edu.cat4early          6.617e-01  1.850e-01  1.679e+03   3.577 0.000357 ***
aspirin               -1.197e-01  1.229e-01  1.607e+03  -0.974 0.330301
parac                 -2.118e-01  1.398e-01  1.552e+03  -1.515 0.129893
diclo                 -2.320e-01  4.399e-01  1.491e+03  -0.527 0.598052
M.Z:APOE41             7.561e-01  5.979e-02  7.947e+03  12.646  < 2e-16 ***
M.Z:APOE42             1.784e+00  1.159e-01  7.977e+03  15.395  < 2e-16 ***
M.Z:edu.cat2tertiary -1.995e-01  6.615e-02  7.946e+03  -3.016 0.002570 **
M.Z:edu.cat3mid        3.442e-02  7.337e-02  7.945e+03   0.469 0.639049
M.Z:edu.cat4early      9.701e-02  8.497e-02  7.982e+03   1.142 0.253584
M.Z:diagn2EMCI        -1.219e-01  8.306e-02  7.712e+03  -1.468 0.142125
M.Z:diagn3LMCI         1.002e+00  6.148e-02  7.851e+03  16.296  < 2e-16 ***
M.Z:diagn4AD           4.249e+00  2.273e-01  7.808e+03  18.691  < 2e-16 ***
M.Z:parac              1.539e-01  5.582e-02  7.882e+03   2.756 0.005859 **
M.Z:diclo             -2.442e-01  1.400e-01  7.800e+03  -1.744 0.081137 .
---
Signif. codes:  0 '***' 0.001 '**' 0.01 '*' 0.05 '.' 0.1 ' ' 1


Correlation matrix not shown by default, as p = 24 > 12.
Use print(x, correlation=TRUE)  or
    vcov(x)        if you need it

mcp.fnc(lme1.mmse)
```

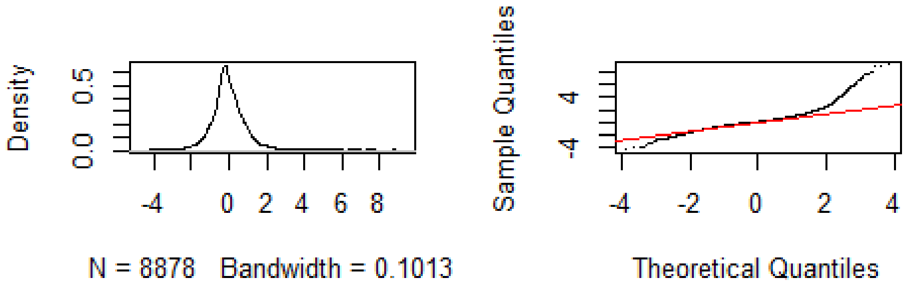

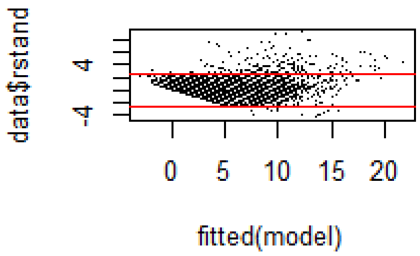

```
lme2.mmse<-lmer(neg.b.MMSE~M+AGE+ diagn+ APOE4+edu.cat + aspirin+ diagn*M
+APOE4*M + Gender*M+ edu.cat*M +parac*M +diclo*M+(1|ID), data=MMSEdata)
summary(lme2.mmse)

Linear mixed model fit by REML. t-tests use Satterthwaite's method [
lmerModLmerTest]
Formula:
neg.b.MMSE ~ M + AGE + diagn + APOE4 + edu.cat + aspirin + diagn *
    M + APOE4 * M + Gender * M + edu.cat * M + parac * M + diclo *
    M + (1 | ID)
   Data: MMSEdata

REML criterion at convergence: 41735.9

Scaled residuals:
    Min      1Q  Median      3Q     Max
-4.0107 -0.4738 -0.0929  0.3890  8.2097

Random effects:
 Groups   Name        Variance Std.Dev.
 ID       (Intercept) 4.830    2.198
 Residual             4.549    2.133
Number of obs: 8878, groups:  ID, 1619

Fixed effects:
                     Estimate Std. Error         df t value Pr(>|t|)
(Intercept)         5.328e-01  1.880e-01  1.880e+03   2.833 0.004654 **
M                   1.309e-02  2.430e-03  7.869e+03   5.387 7.39e-08 ***
AGE                 2.829e-02  8.766e-03  1.629e+03   3.227 0.001274 **
diagn2EMCI          9.588e-01  1.966e-01  1.991e+03   4.876 1.17e-06 ***
diagn3LMCI          2.229e+00  1.686e-01  1.883e+03  13.219  < 2e-16 ***
diagn4AD            5.916e+00  2.072e-01  2.300e+03  28.553  < 2e-16 ***
APOE41              1.355e-01  1.402e-01  1.959e+03   0.967 0.333887
APOE42             -8.658e-02  2.260e-01  1.998e+03  -0.383 0.701712
edu.cat2tertiary    2.952e-01  1.603e-01  1.944e+03   1.841 0.065774 .
edu.cat3mid         6.681e-01  1.819e-01  1.950e+03   3.672 0.000247 ***
edu.cat4early       5.988e-01  1.936e-01  1.969e+03   3.093 0.002006 **
aspirin            -1.066e-01  1.240e-01  1.608e+03  -0.860 0.389901
GenderMale          1.548e-01  1.332e-01  1.948e+03   1.162 0.245274
parac              -3.509e-01  1.471e-01  1.890e+03  -2.386 0.017112 *
diclo              -2.837e-03  4.629e-01  1.833e+03  -0.006 0.995112
M:diagn2EMCI       -3.832e-03  2.975e-03  7.710e+03  -1.288 0.197734
M:diagn3LMCI        3.747e-02  2.220e-03  7.848e+03  16.876  < 2e-16 ***
M:diagn4AD          1.527e-01  8.138e-03  7.805e+03  18.767  < 2e-16 ***
M:APOE41            2.702e-02  2.140e-03  7.944e+03  12.625  < 2e-16 ***
M:APOE42            6.444e-02  4.149e-03  7.974e+03  15.530  < 2e-16 ***
M:GenderMale       -1.055e-02  2.013e-03  7.939e+03  -5.241 1.64e-07 ***
M:edu.cat2tertiary -8.055e-03  2.374e-03  7.944e+03  -3.393 0.000695 ***
M:edu.cat3mid      -2.297e-03  2.711e-03  7.935e+03  -0.847 0.396877
M:edu.cat4early     1.247e-03  3.071e-03  7.976e+03   0.406 0.684792
M:parac             4.706e-03  2.004e-03  7.875e+03   2.348 0.018885 *
M:diclo            -7.867e-03  5.013e-03  7.797e+03  -1.569 0.116642
---
Signif. codes:  0 '***' 0.001 '**' 0.01 '*' 0.05 '.' 0.1 ' ' 1


Correlation matrix not shown by default, as p = 26 > 12.
Use print(x, correlation=TRUE)  or
    vcov(x)        if you need it
```

```
mcp.fnc(lme2.mmse)
```

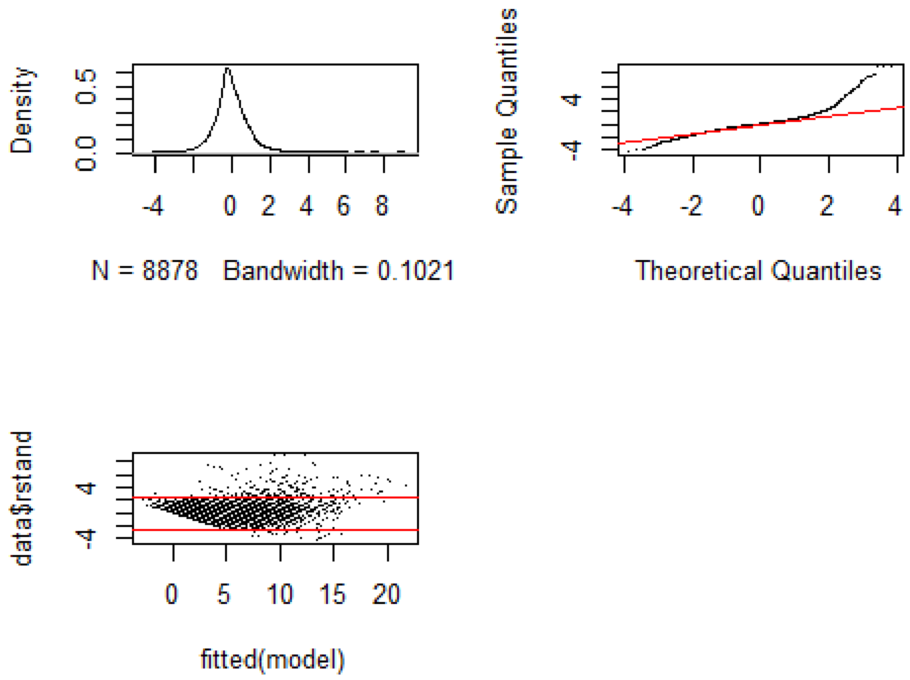


```
neg.binom.zero.mmse<-glmmadmb(neg.b.MMSE~M+AGE+ diagn+ APOE4+edu.cat + aspirin+
diagn*M +APOE4*M + Gender*M+ edu.cat*M +parac*M +diclo*M+(1|ID),
family="nbinom1", data=MMSEdata, zeroInflation=TRUE)
anova(neg.binom.zero.mmse, neg.mmse)

Analysis of Deviance Table

Model 1: neg.b.MMSE ~ M + AGE + diagn + APOE4 + edu.cat + aspirin + diagn * M +
APOE4 * M + Gender * M + edu.cat * M + parac * M + diclo * M
Model 2: neg.b.MMSE ~ M + AGE + diagn + APOE4 + edu.cat + aspirin + diagn * M +
APOE4 * M + Gender * M + edu.cat * M + parac * M + diclo * M
  NoPar LogLik Df Deviance Pr(>Chi)
1    28 -16940
2    29 -16938  1        4   0.0455 *
---
Signif. codes:  0 '***' 0.001 '**' 0.01 '*' 0.05 '.' 0.1 ' ' 1

neg.binom.link.parameterisation.mmse<-glmmadmb(neg.b.MMSE~M+AGE+ diagn+
APOE4+edu.cat + aspirin+ diagn*M +APOE4*M + Gender*M+ edu.cat*M +parac*M
+diclo*M+(1|ID), family="nbinom", data=MMSEdata)


AIC.MMSE<-c(
AIC(logLik(neg.mmse)),
AIC(logLik(glmerB1.mmse)),
AIC(logLik(glmerB2.mmse)),
AIC(logLik(glmerB3.mmse)),
AIC(logLik(glmerB4.mmse)),
AIC(logLik(glmerB5.mmse)),
AIC(logLik(glmerB6.mmse)),
AIC(logLik(glmerP1.mmse)),
AIC(logLik(glmerP2.mmse)),
```

```
AIC(logLik(glmerP3.mmse)),
AIC(logLik(glmerP4.mmse)),
AIC(logLik(lme1.mmse)),
AIC(logLik(lme2.mmse)),
AIC(logLik(neg.binom.zero.mmse)),
AIC(logLik(neg.binom.link.parameterisation.mmse)))

Model<-c("Negative Binomial", "Binomial (logit)", "Binomial (probit)","Binomial
(Cloglog)","Binomial (logit, centered)", "Binomial (probit, centered)","Binomial
(Cloglog, centered)","Poisson (log)","Poisson (sqrt)","Poisson (log,
centered)","Poisson (sqrt, centered)","MLM (centered)","MLM","Negative binomial
(zero inflated)","Negative binomial (classical parameterisation)")

data.frame(Model,AIC.MMSE)

                                             Model AIC.MMSE
1                                Negative Binomial 33936.00
2                                 Binomial (logit) 34398.91
3                                Binomial (probit) 34621.30
4                               Binomial (Cloglog) 35043.63
5                       Binomial (logit, centered) 34421.14
6                      Binomial (probit, centered) 34642.97
7                     Binomial (Cloglog, centered) 35060.95
8                                    Poisson (log) 33945.30
9                                   Poisson (sqrt) 34526.45
10                         Poisson (log, centered) 33952.64
11                        Poisson (sqrt, centered) 34523.36
12                                  MLM (centered) 41729.48
13                                             MLM 41791.95
14                 Negative binomial (zero inflated) 33934.00
15 Negative binomial (classical parameterisation) 33947.80
```

w Only

8. Analysis of cognitive decline using the ADAS score

Alzheimer's Disease Assessment Scale-Cog (ADAS) is a cognitive assessment with a focus on memory that is often used as the primary measure of Alzheimer's disease progression in clinical trials. It is a score out of 90 with higher scores corresponding to worse cognitive performance.

8.2. *Dependent variable check*

Those with no ADAS score were removed and variables were checked for correct categorization.

```
data<-read.csv("CleanedFinalData.csv", header=T)
data$ADAS<-data$ADAS13
ADASdata<-data[!is.na(data$ADAS),]
ADASdata$ID<-as.factor(ADASdata$ID)
ADASdata$APOE4<-as.factor(ADASdata$APOE4)
```

8.3. *Generation of dependent variables appropriate for different distributions*

```
ADASdata$neg.b.ADAS<-round(3*ADASdata$ADAS13)
ADASdata$fail<-ADASdata$neg.b.ADAS
ADASdata$success<-270-ADASdata$fail
ADASdata$ADASscore<-rep(270,length(ADASdata$neg.b.ADAS))
ADASdata$proportion<-ADASdata$fail/ADASdata$ADASscore
```

8.4. *Transformation to obtain normal approximation*

The total errors were then generated by contructing a linear model of with ADAS as the dependent variable and patient ID as the explainitory variable. It has been shown that if the total errors are homoscedastic and normal then the multi-level linear model errors will likely also be homoscedastic and normal distributed (Gurka *et al.* 2006). Therefore, total errors are an excellent starting point for model dianostics. From this it was found that even with boxcox optimized transformation the residuals while normally distributed fail to have even homoscidadicity. This is due to the categorical nature of the ADAS score and the high number of zero values.Therefore, a generalized linear model utilizing a non-gaussian distribution were performed.

```
qqp(ADASdata$ADAS[ADASdata$M==0], dist="norm")
```

For Review Only

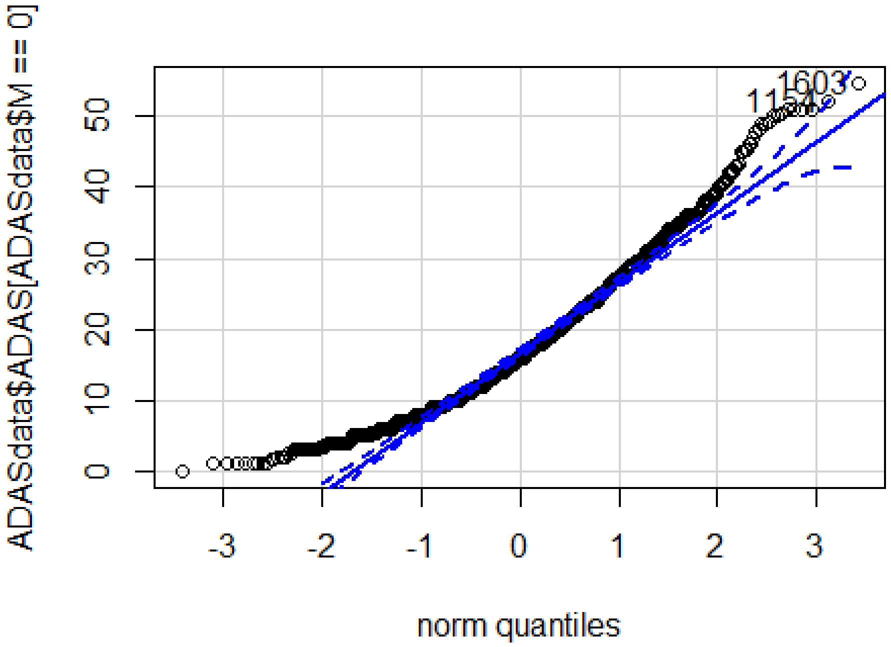


```
[1] 1603 1154

m1<-lm((ADAS+1)~ID, data=ADASdata)
par(mfrow=c(2,2))
plot(m1)
```

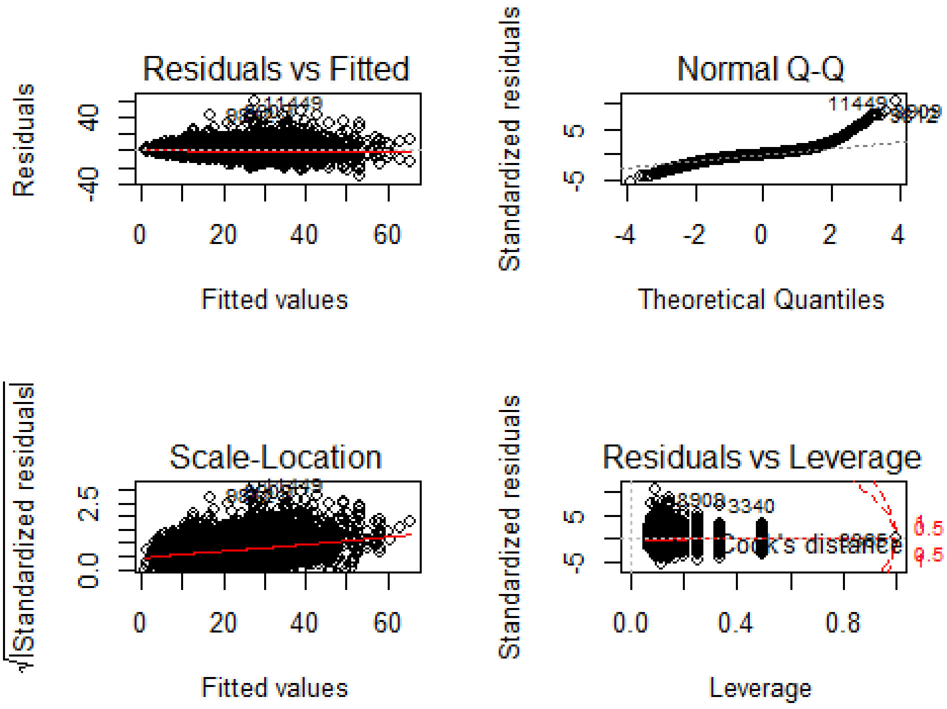

```
boxcox<-boxcox(m1,lambda = seq(-5, 5, 1/100),plotit = TRUE )
selectedlambda<-boxcox$x[boxcox$y==max(boxcox$y)]
selectedlambda

[1] 0.26

ADASdata$tADAS<-(ADASdata$ADAS)^selectedlambda
m1.t<-lmer(tADAS~diagn+edu.cat+Gender+APOE4+M+(1|ID),data=ADASdata)
summary(m1.t)

Linear mixed model fit by REML. t-tests use Satterthwaite's method [
lmerModLmerTest]
Formula: tADAS ~ diagn + edu.cat + Gender + APOE4 + M + (1 | ID)
   Data: ADASdata

REML criterion at convergence: -3572.7

Scaled residuals:
    Min      1Q  Median      3Q     Max
-7.9140 -0.4878  0.0033  0.5021  7.3893

Random effects:
 Groups   Name        Variance Std.Dev.
 ID       (Intercept) 0.05238  0.2289
 Residual             0.02475  0.1573
Number of obs: 8770, groups:  ID, 1618

Fixed effects:
                  Estimate Std. Error        df t value Pr(>|t|)
(Intercept)      1.601e+00  1.632e-02 1.643e+03  98.082  < 2e-16 ***
diagn2EMCI       1.368e-01  1.824e-02 1.593e+03   7.496 1.08e-13 ***
diagn3LMCI       4.034e-01  1.602e-02 1.585e+03  25.182  < 2e-16 ***
diagn4AD         7.078e-01  1.894e-02 1.711e+03  37.364  < 2e-16 ***
edu.cat2tertiary 3.264e-02  1.521e-02 1.618e+03   2.146    0.032 *
edu.cat3mid      6.943e-02  1.723e-02 1.620e+03   4.029 5.85e-05 ***
edu.cat4early    9.608e-02  1.825e-02 1.642e+03   5.264 1.59e-07 ***
GenderMale       4.996e-02  1.240e-02 1.628e+03   4.030 5.84e-05 ***
APOE41           7.855e-02  1.326e-02 1.627e+03   5.925 3.80e-09 ***
APOE42           9.955e-02  2.107e-02 1.642e+03   4.726 2.49e-06 ***
M                2.837e-03  7.139e-05 7.484e+03  39.740  < 2e-16 ***
---
Signif. codes:  0 '***' 0.001 '**' 0.01 '*' 0.05 '.' 0.1 ' ' 1

Correlation of Fixed Effects:
            (Intr) d2EMCI d3LMCI dgn4AD ed.ct2 ed.ct3 ed.ct4 GndrMl APOE41
diagn2EMCI  -0.421
diagn3LMCI  -0.453  0.501
diagn4AD    -0.351  0.436  0.531
ed.ct2trtry -0.418  0.011 -0.009 -0.043
edu.cat3mid -0.438 -0.027  0.024 -0.037  0.405
edu.cat4rly -0.348 -0.037 -0.069 -0.129  0.388  0.358
GenderMale  -0.453 -0.044 -0.083 -0.048  0.050  0.174  0.142
APOE41      -0.192 -0.094 -0.178 -0.209 -0.030 -0.032 -0.047  0.002
APOE42      -0.072 -0.075 -0.177 -0.236 -0.045 -0.028 -0.019 -0.010  0.307
M           -0.118  0.024  0.027  0.070  0.000 -0.002  0.003 -0.002  0.005
            APOE42
diagn2EMCI
diagn3LMCI
diagn4AD
ed.ct2trtry
```

```
edu.cat3mid
edu.cat4rly
GenderMale
APOE41
APOE42
M              0.005

mcp.fnc(m1.t)
```

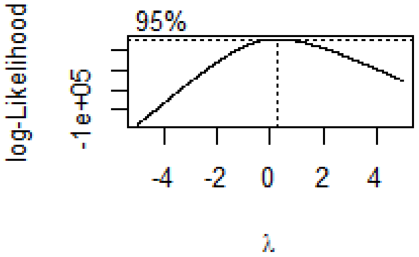

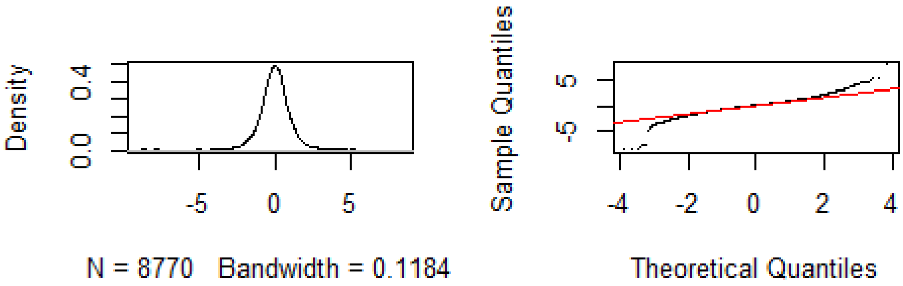

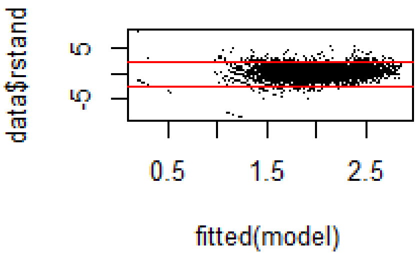

```
qqp(ADASdata$tADAS[ADASdata$M==0], dist="norm")
```

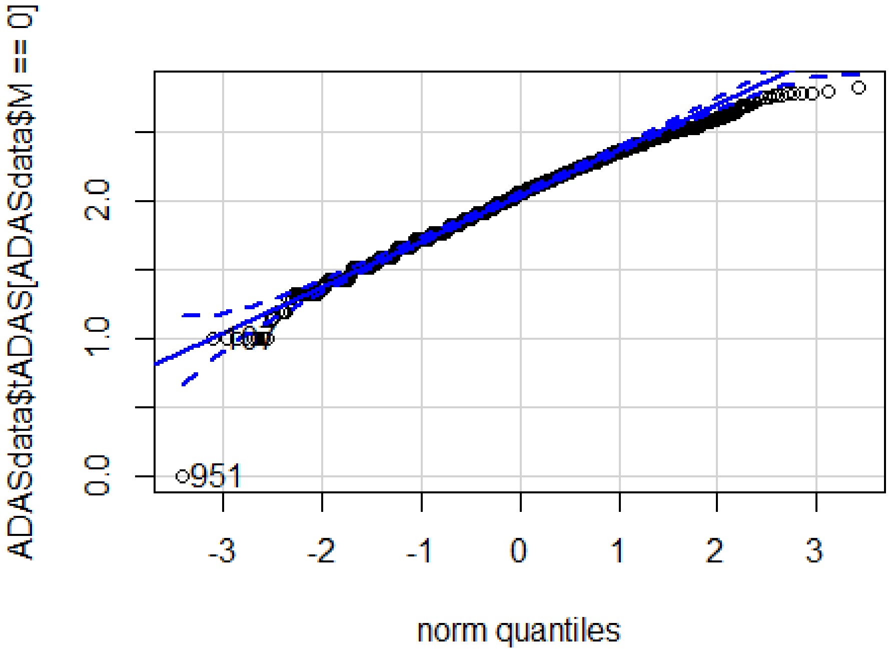


```
[1] 951 107
```

8.5. *Selecting non-gaussian model*

The data was plotted against expected distributions including normal, Poisson, negative binomial, binomial, exponential or gamma distributions within each time point. Some transformations were required including converting the ADAS score to whole numbers (with a round (3xADAS) function) for the Poisson, negative binomial and exponential distributions. This is because 1/3 marks are received in the ADAS task. For the Poisson distribution there was over dispersion as seen by the vast difference in mean and variance, supporting the use of negative binomial models. This is also evident in the dependent variable not following a Poisson distribution. The binomial model seems appropriate theoretically as the MMSE score could be considered 270 trials with a proportion of failures occurring. However, the large number of zeros made logistic regression of the binomial distribution inappropriate. Furthermore, the exponential model was a poor fit. The negative binomial and gamma models appear to be the most appropriate models. However, given the categorical nature of the data the negative binomial model was chosen for analysis. This is an approximate method for distribution selection as it does not take into account the explanatory variables effects on the distribution. This approach was used as a starting point for model analysis. Distributions were compared once the final model was established and the negative binomial model proved to be the most appropriate. Furthermore, residual analysis of the final selected model confirmed the appropriateness of the negative binomial model.

```
hist(ADASdata$ADAS[ADASdata$M==0])
```

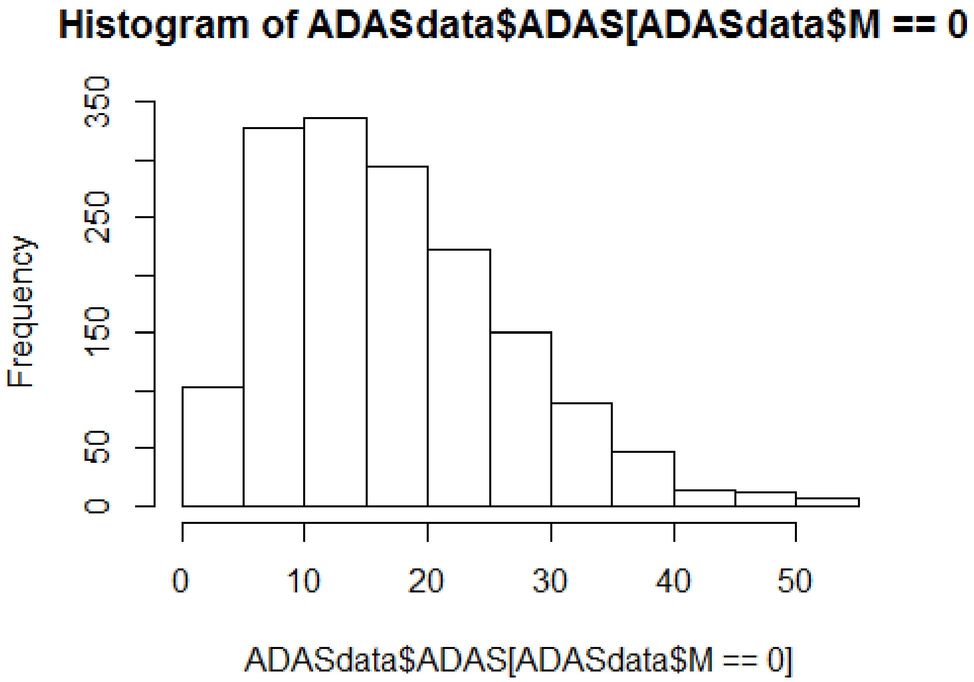


```
hist(ADASdata$ADAS[ADASdata$M==12])
```

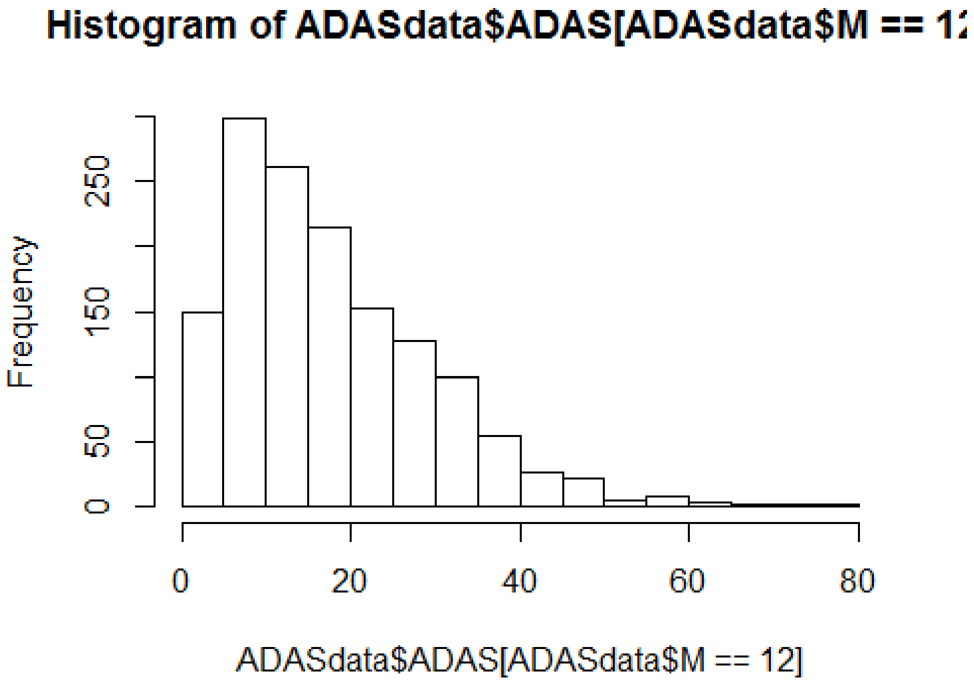


```
hist(ADASdata$ADAS[ADASdata$M==24])
```

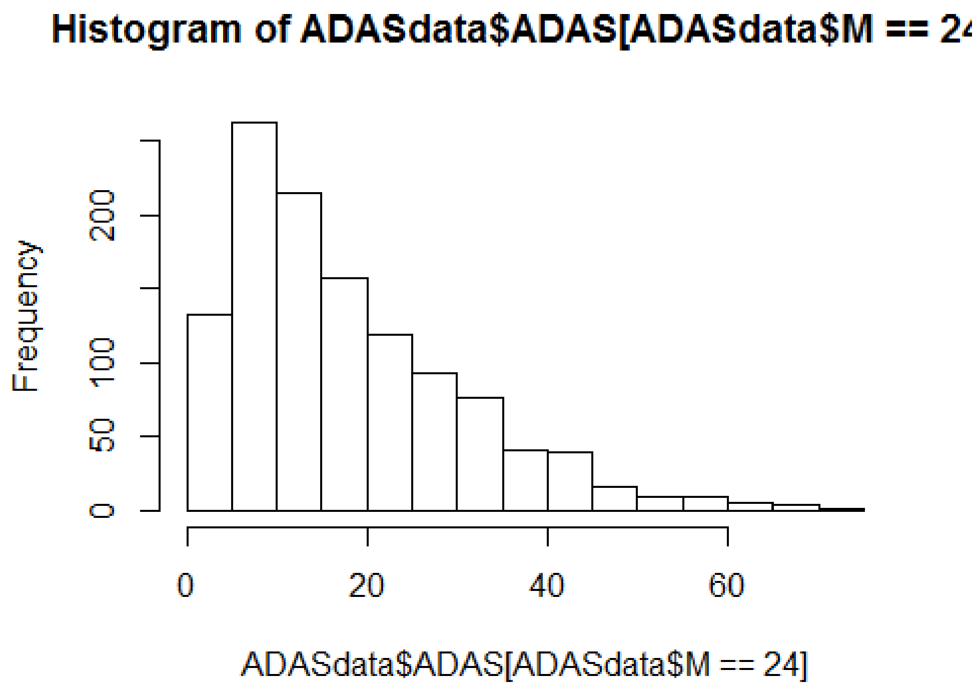


```
hist(ADASdata$ADAS[ADASdata$M==48])
```

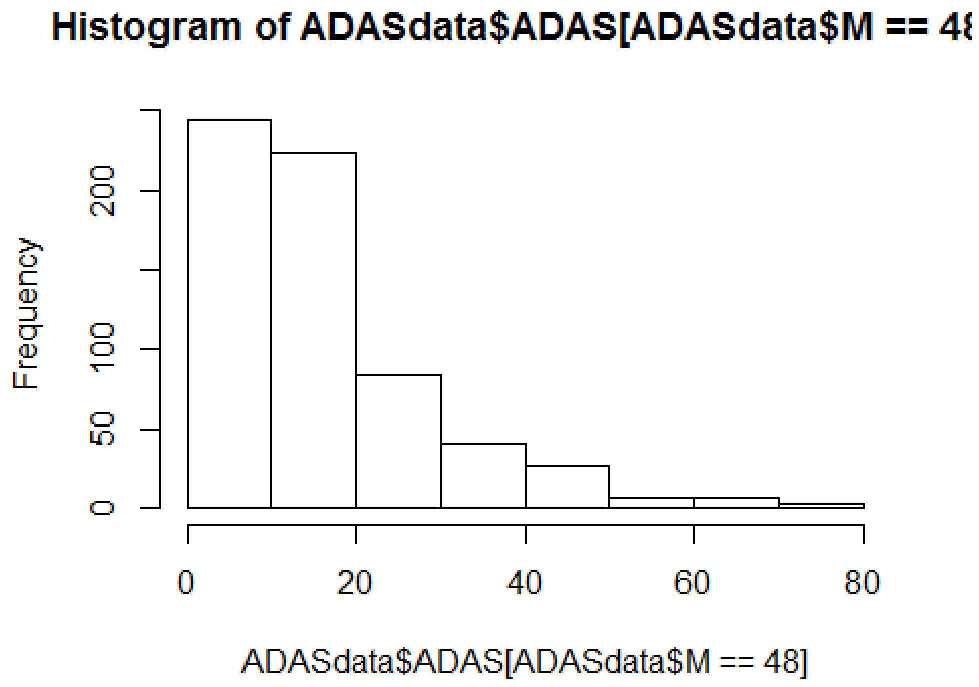


```
hist(ADASdata$ADAS[ADASdata$M==72])
```

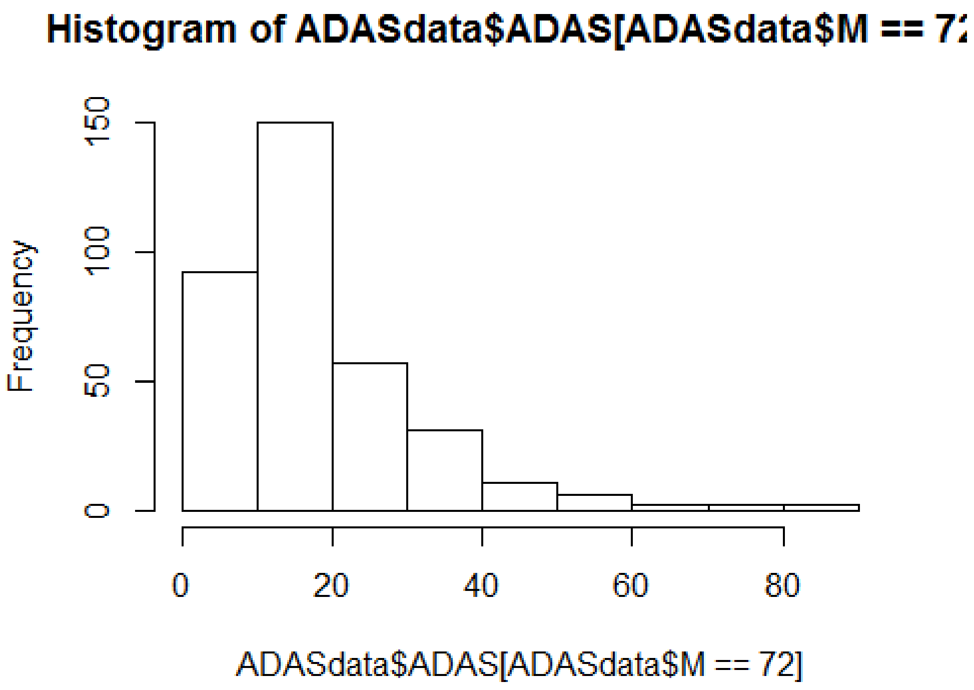


```
hist(ADASdata$ADAS[ADASdata$M==120])
```

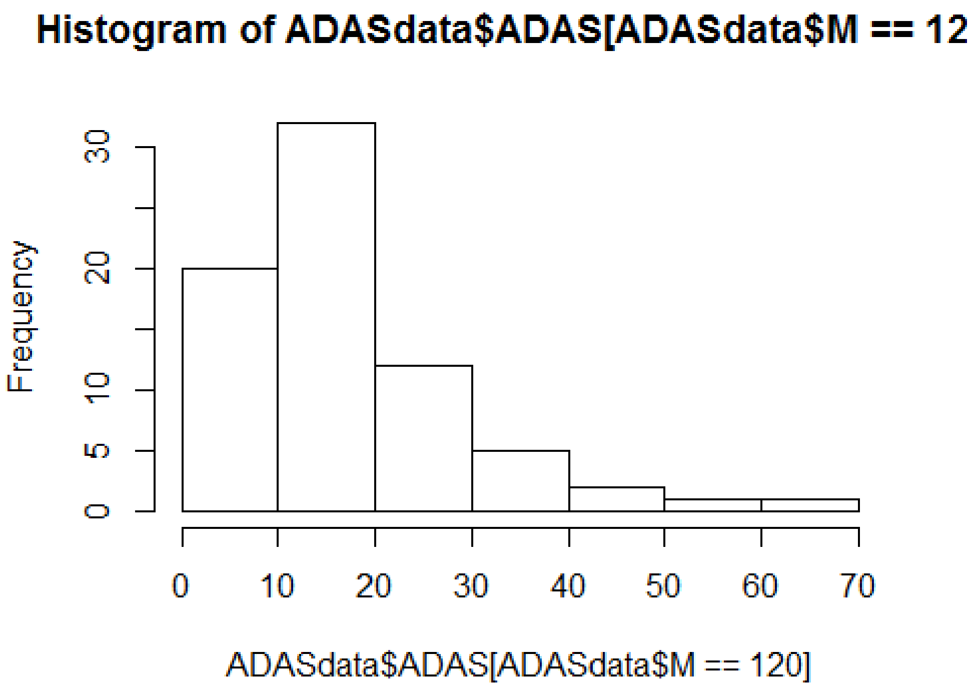


```
qqp(ADASdata$ADAS[ADASdata$M==0], "norm", main="Normal distribution model
Month=0")
```

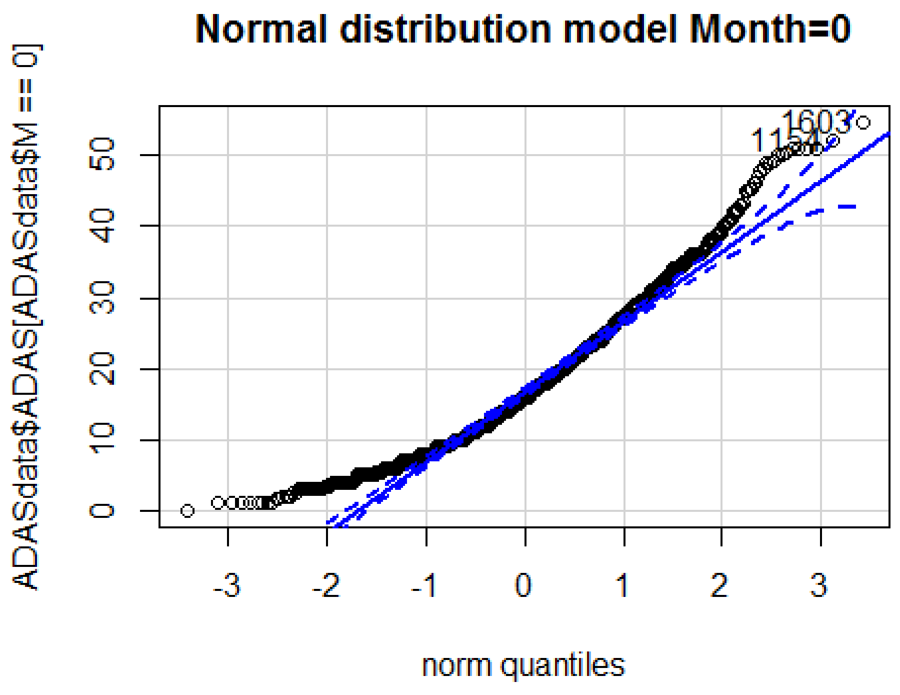


```
[1] 1603 1154

qqp(ADASdata$ADAS[ADASdata$M==12], "norm", main="Normal distribution model
Month=12")
```

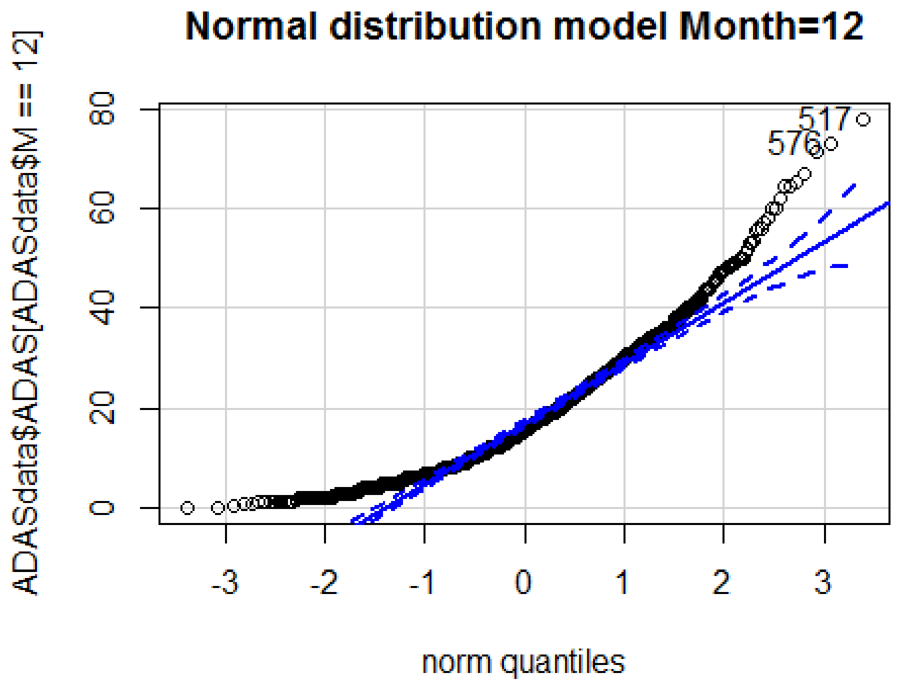


```
[1] 517 576
```

```
qqp(ADASdata$ADAS[ADASdata$M==24], "norm", main="Normal distribution model
Month=24")
```

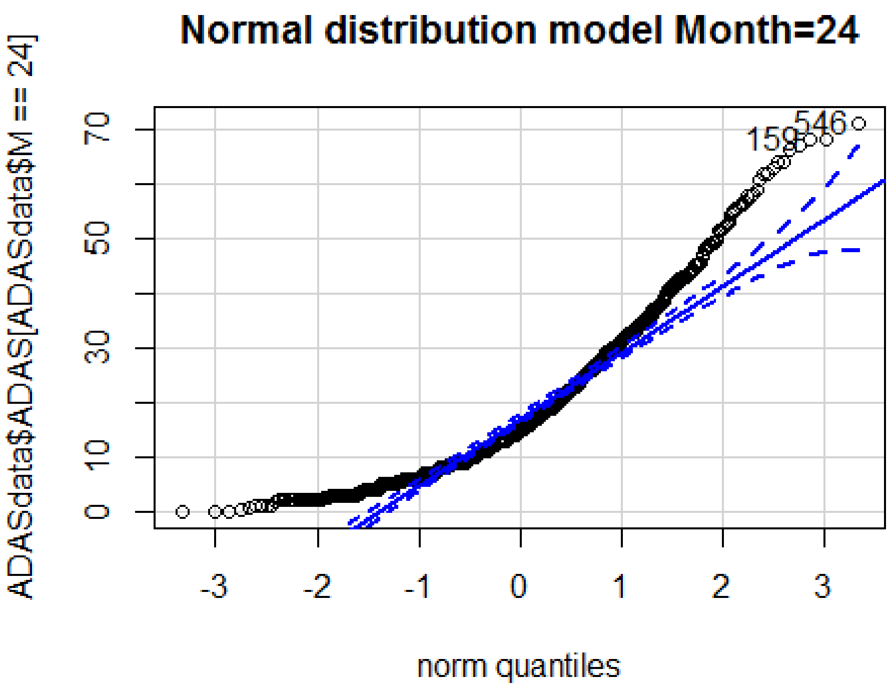


```
[1] 546 159

qqp(ADASdata$ADAS[ADASdata$M==48], "norm", main="Normal distribution model
Month=48")
```

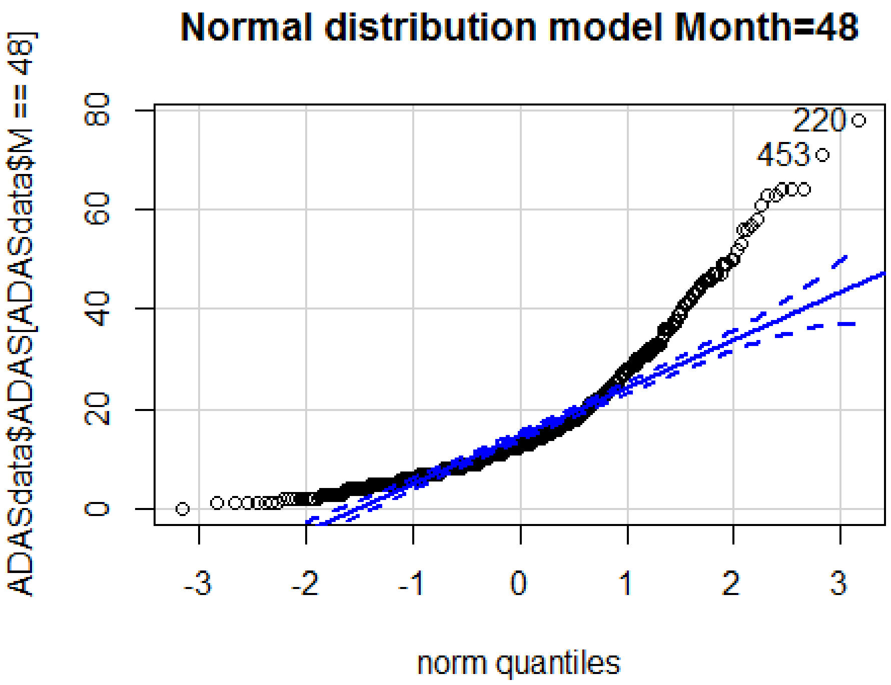

```
[1] 220 453

qqp(ADASdata$ADAS[ADASdata$M==72], "norm", main="Normal distribution model
Month=72")
```

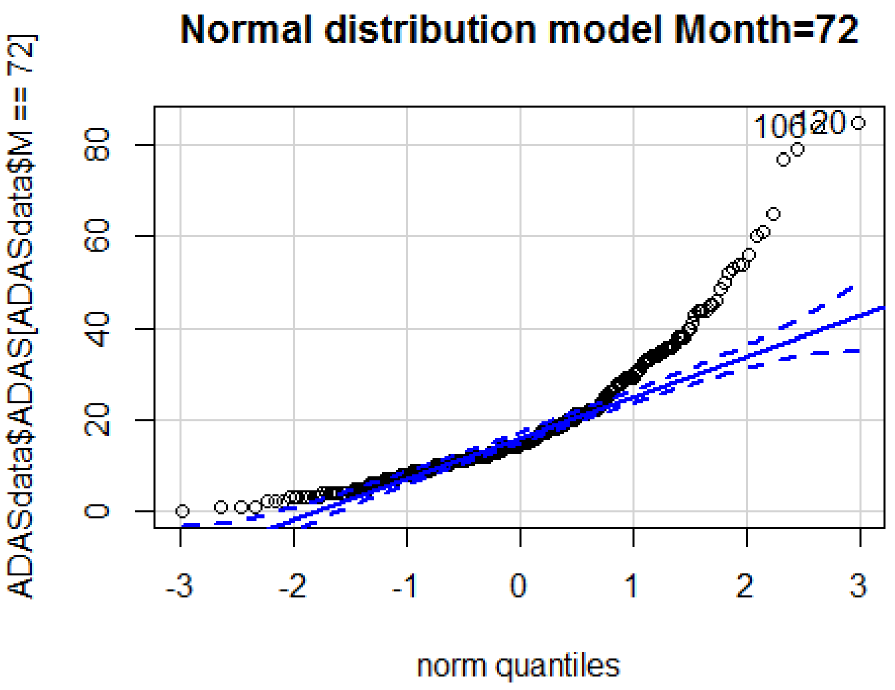


```
[1] 120 106

qqp(ADASdata$ADAS[ADASdata$M==120], "norm", main="Normal distribution model
Month=120")
```

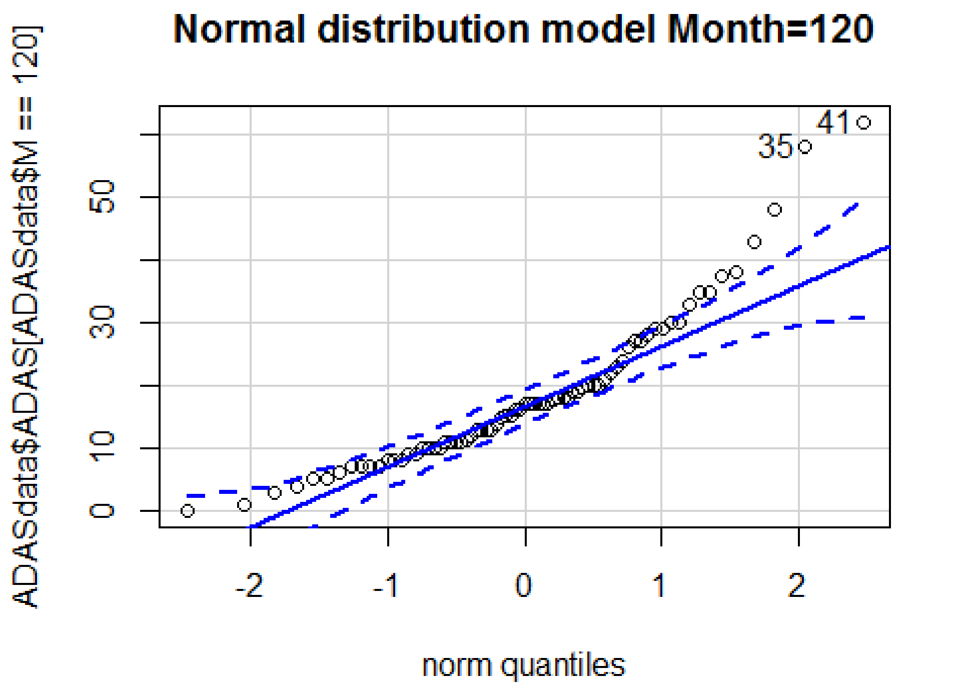


```
[1] 41 35

hist(ADASdata$neg.b.ADAS[ADASdata$M==0])
```

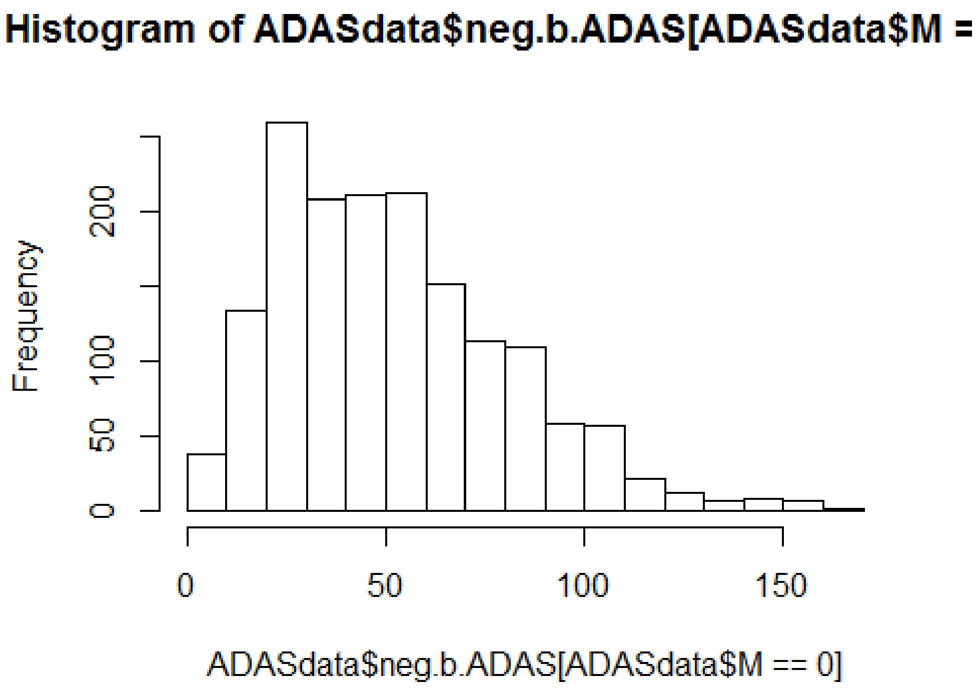


```
hist(ADASdata$neg.b.ADAS[ADASdata$M==12])
```

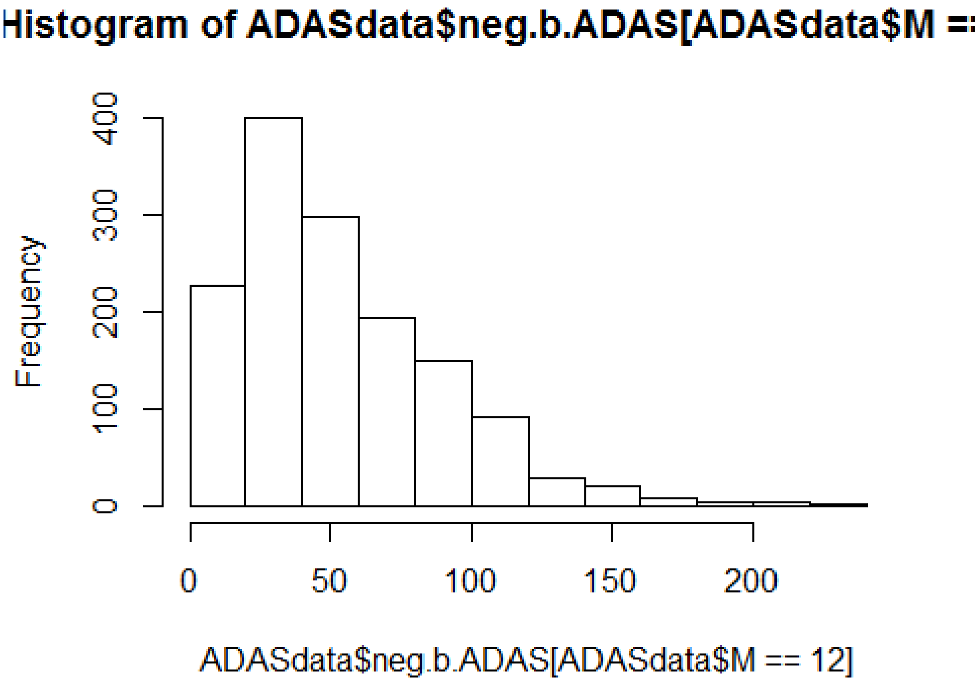


```
hist(ADASdata$neg.b.ADAS[ADASdata$M==24])
```

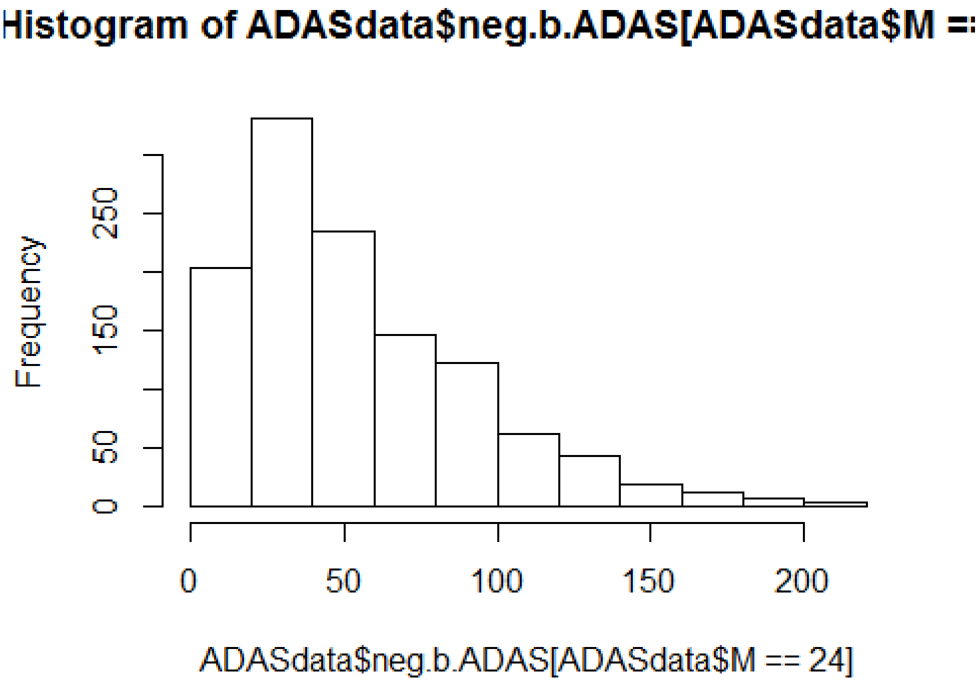


```
hist(ADASdata$neg.b.ADAS[ADASdata$M==48])
```

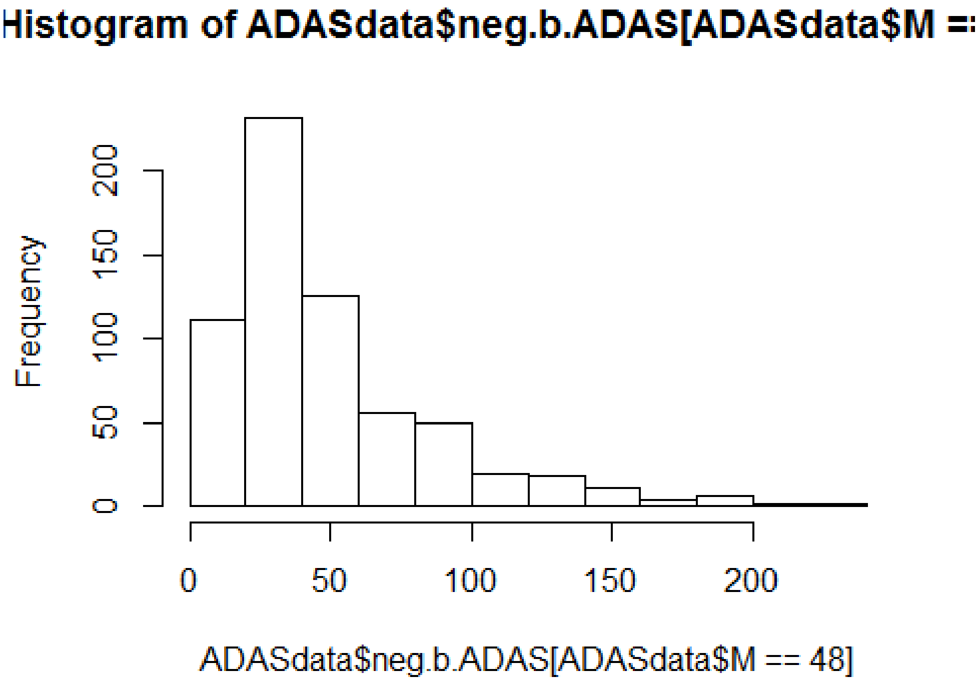


```
hist(ADASdata$neg.b.ADAS[ADASdata$M==72])
```

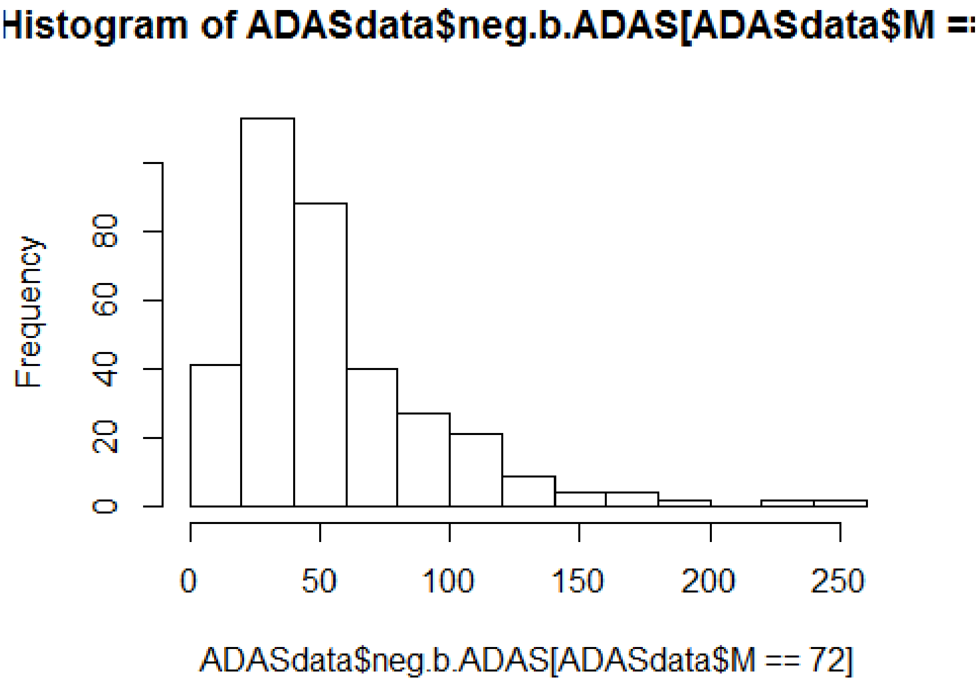


```
hist(ADASdata$neg.b.ADAS[ADASdata$M==120])
```

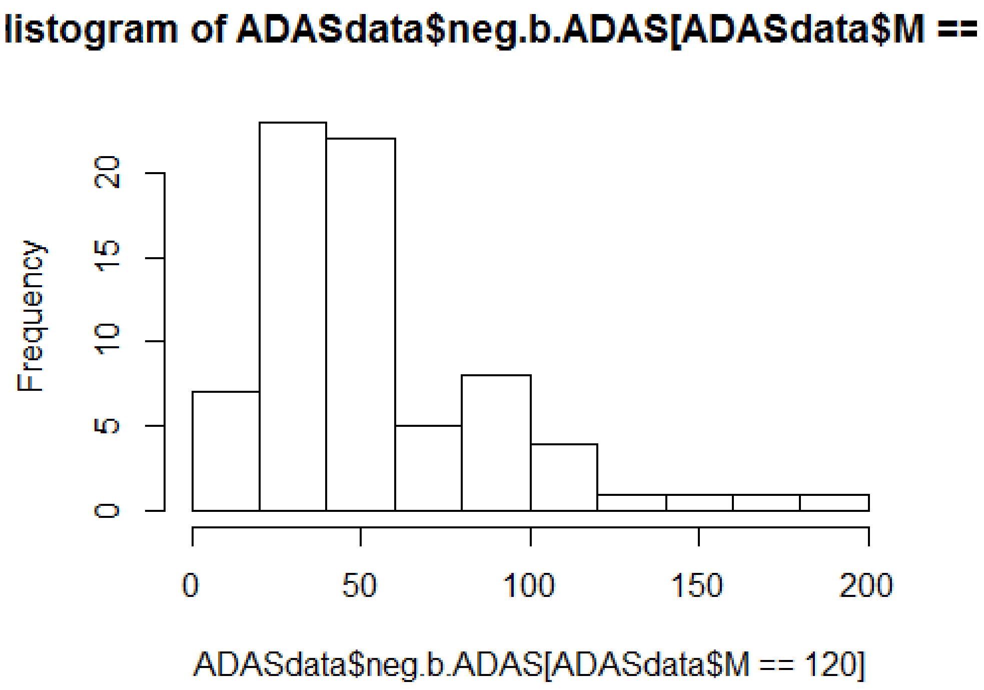


```
nbinom<-fitdistr(ADASdata$neg.b.ADAS[ADASdata$M==0], "negative binomial")
qqp(ADASdata$neg.b.ADAS[ADASdata$M==0], "nbinom", size=nbinom$estimate[[1]],
mu=nbinom$estimate[[2]], main="Negative binomial model Month=0")
```

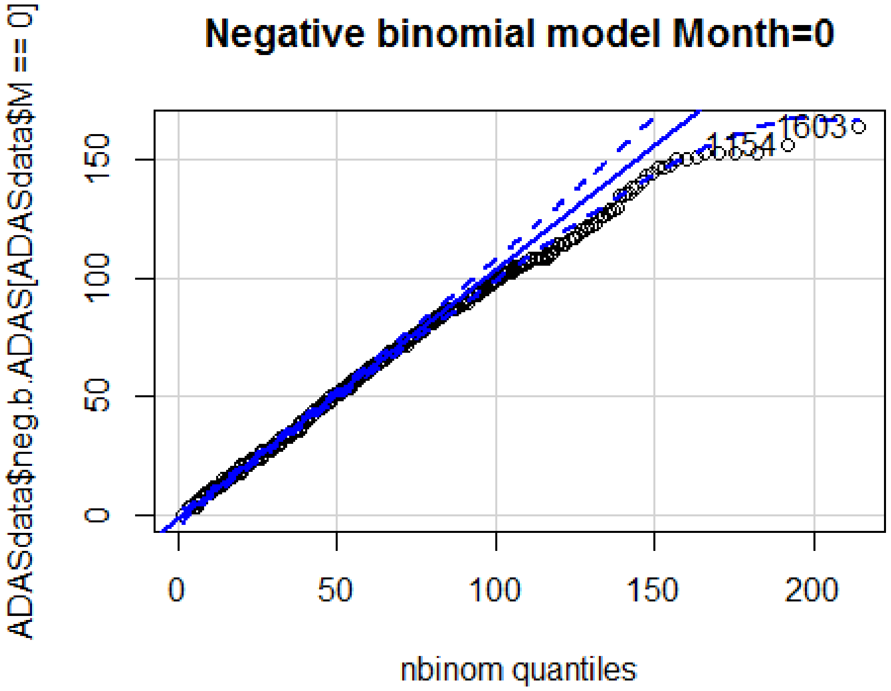


```
[1] 1603 1154
```

```
nbinom<-fitdistr(ADASdata$neg.b.ADAS[ADASdata$M==12], "negative binomial")
qqp(ADASdata$neg.b.ADAS[ADASdata$M==12], "nbinom", size=nbinom$estimate[[1]],
mu=nbinom$estimate[[2]], main="Negative binomial model Month=12")
```

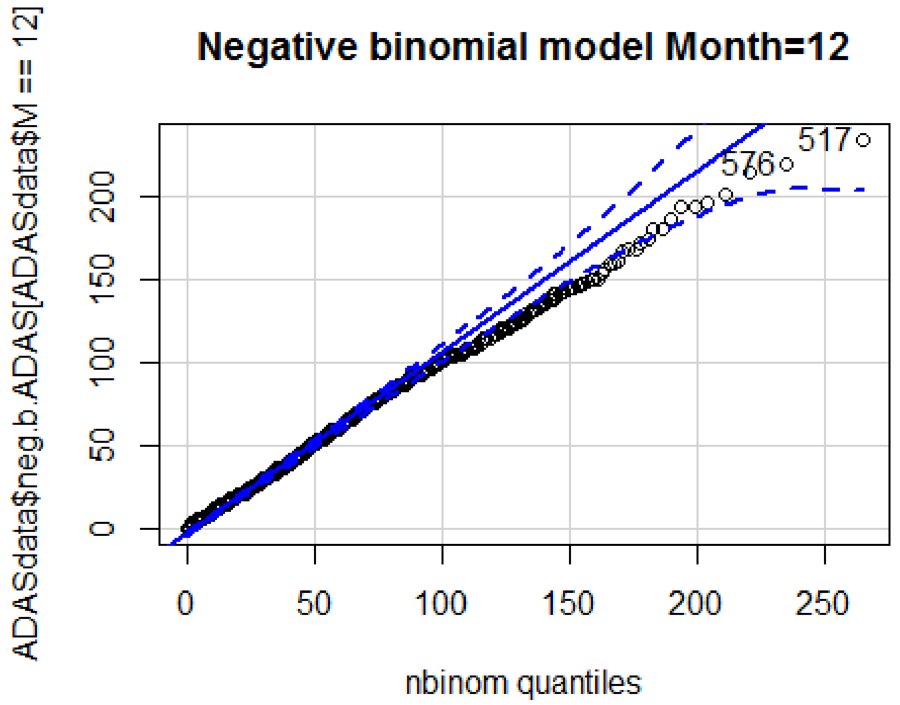


```
[1] 517 576

nbinom<-fitdistr(ADASdata$neg.b.ADAS[ADASdata$M==24], "negative binomial")
qqp(ADASdata$neg.b.ADAS[ADASdata$M==24], "nbinom", size=nbinom$estimate[[1]],
mu=nbinom$estimate[[2]], main="Negative binomial model Month=24")
```

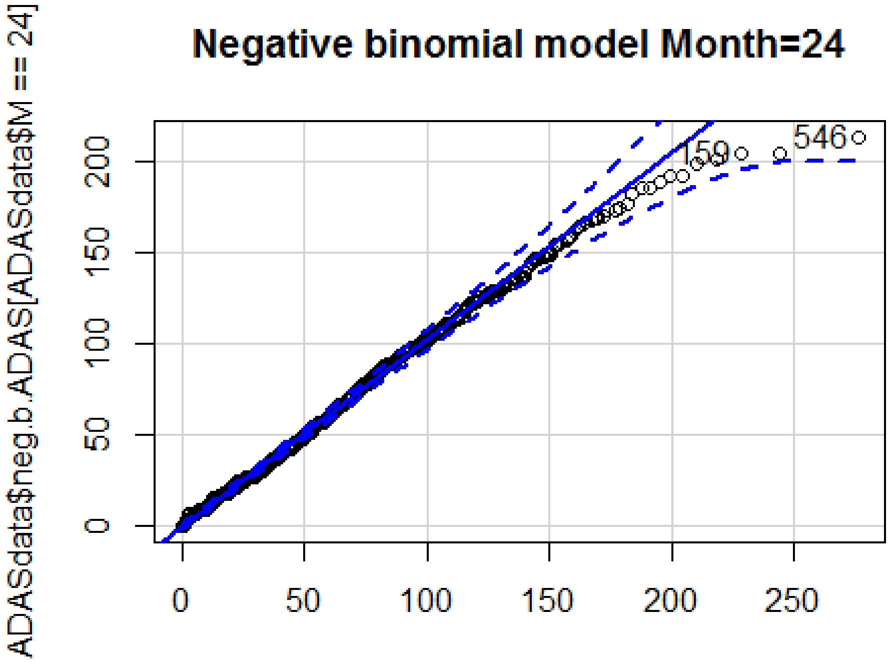


```
[1] 546 159

nbinom<-fitdistr(ADASdata$neg.b.ADAS[ADASdata$M==48], "negative binomial")
qqp(ADASdata$neg.b.ADAS[ADASdata$M==48], "nbinom", size=nbinom$estimate[[1]],
mu=nbinom$estimate[[2]], main="Negative binomial model Month=48")
```

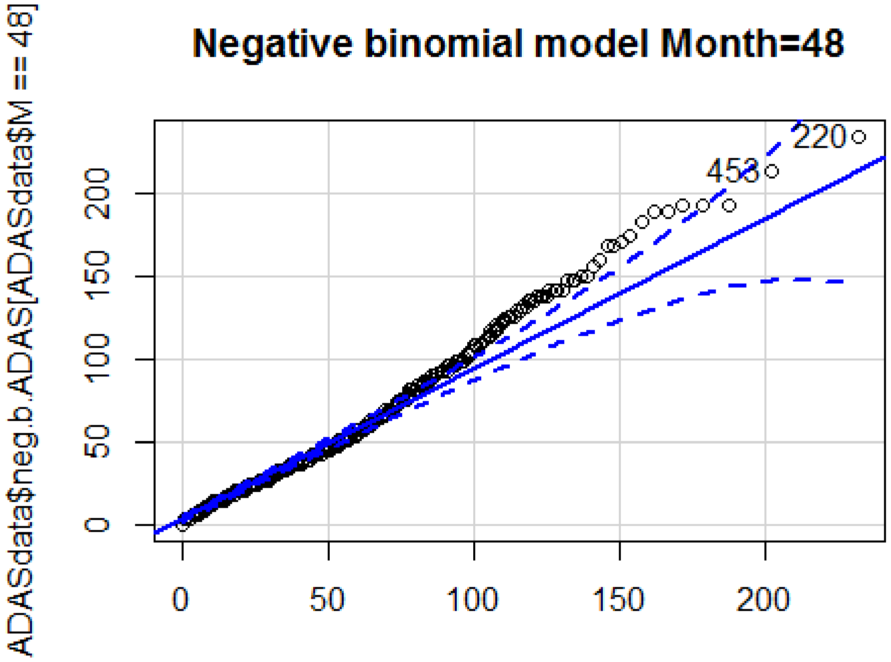


```
[1] 220 453
```

```
nbinom<-fitdistr(ADASdata$neg.b.ADAS[ADASdata$M==72], "negative binomial")
qqp(ADASdata$neg.b.ADAS[ADASdata$M==72], "nbinom", size=nbinom$estimate[[1]],
mu=nbinom$estimate[[2]], main="Negative binomial model Month=72")
```

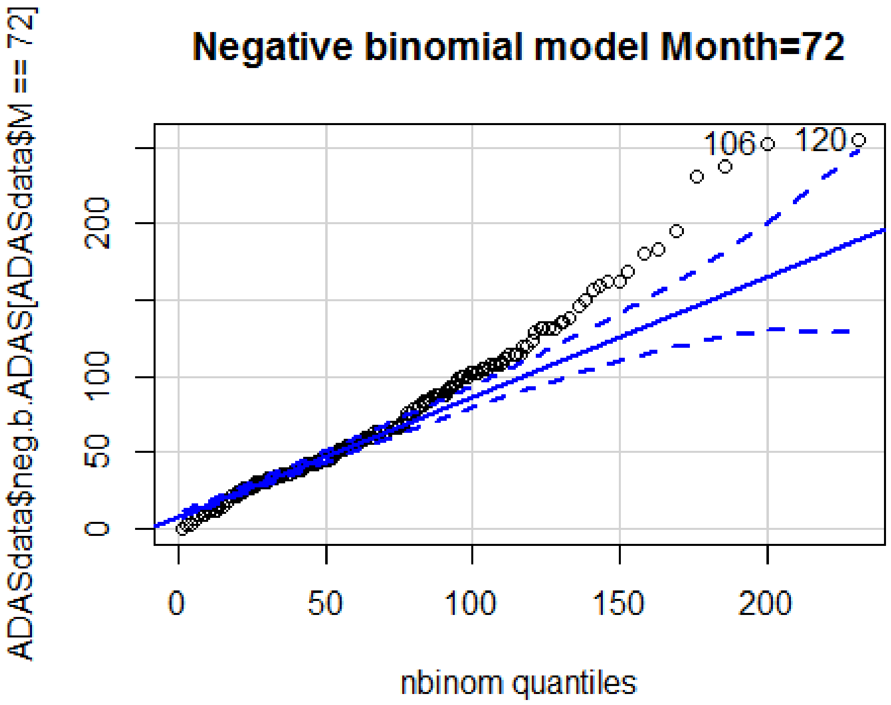


```
[1] 120 106

nbinom<-fitdistr(ADASdata$neg.b.ADAS[ADASdata$M==120], "negative binomial")
qqp(ADASdata$neg.b.ADAS[ADASdata$M==120], "nbinom", size=nbinom$estimate[[1]],
mu=nbinom$estimate[[2]], main="Negative binomial model Month=120")
```

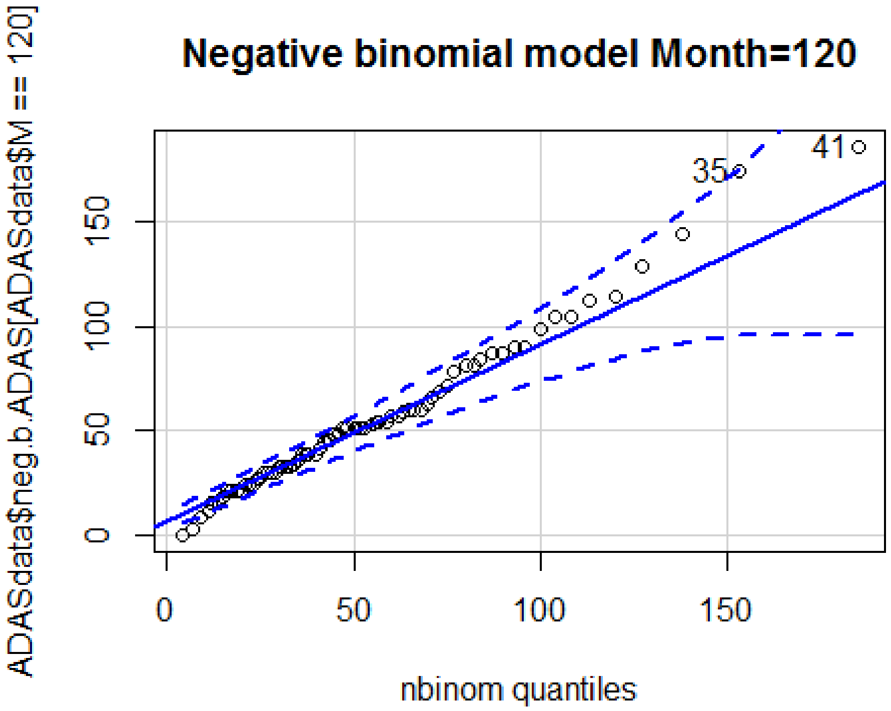


```
[1] 41 35

poisson <- fitdistr(ADASdata$neg.b.ADAS[ADASdata$M==0]+1, "Poisson")
qqp(ADASdata$neg.b.ADAS, "pois", lambda=poisson$estimate, main="Poisson model
Month=0")
```

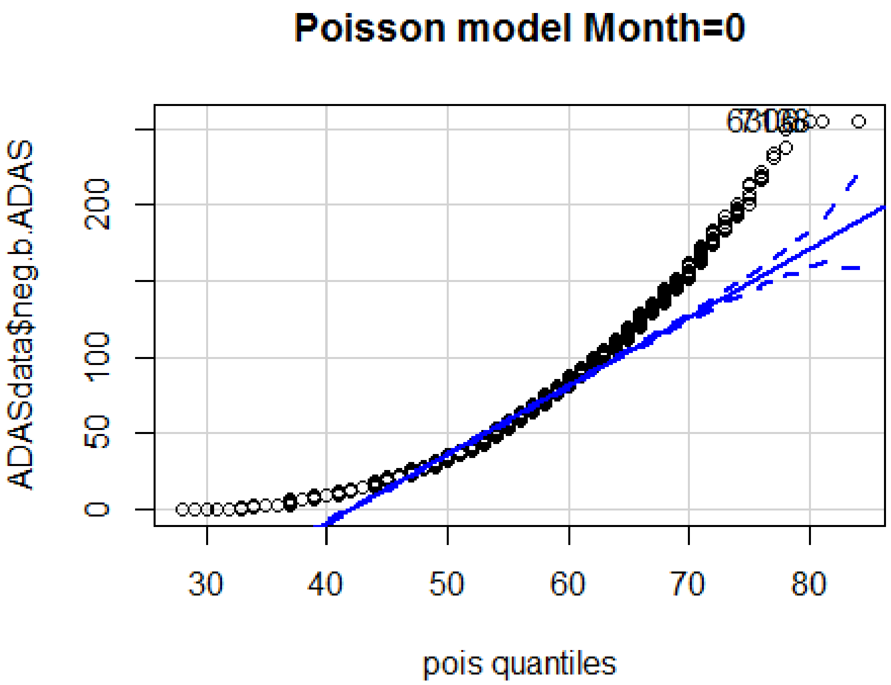


```
[1] 6306 7138
```

```
poisson <- fitdistr(ADASdata$neg.b.ADAS[ADASdata$M==12], "Poisson")
qqp(ADASdata$neg.b.ADAS, "pois", lambda=poisson$estimate, main="Poisson model
Month=12")
```

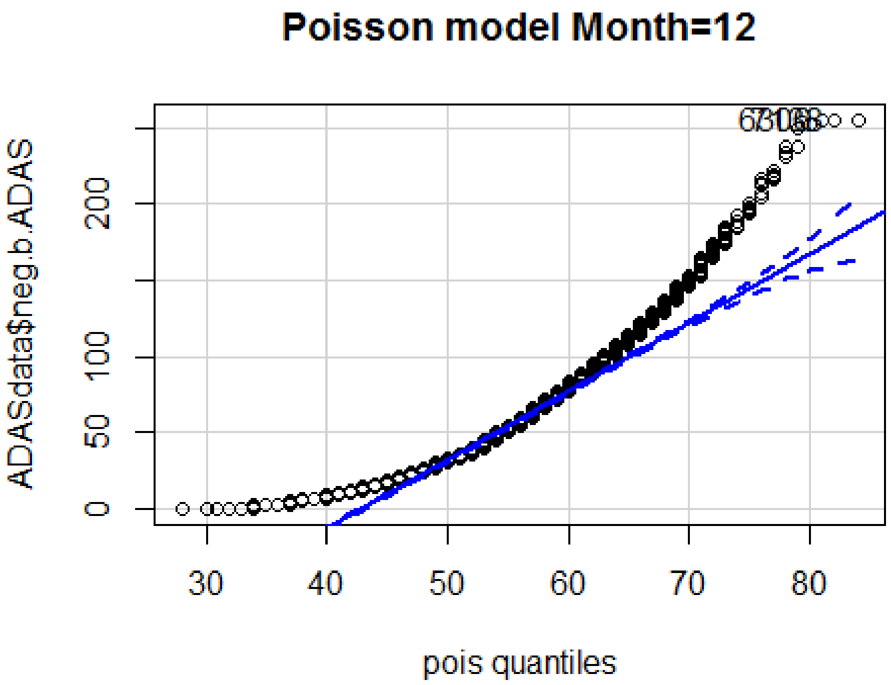


```
[1] 6306 7138

poisson <- fitdistr(ADASdata$neg.b.ADAS[ADASdata$M==24], "Poisson")
qqp(ADASdata$neg.b.ADAS, "pois", lambda=poisson$estimate, main="Poisson model
Month=24")
```

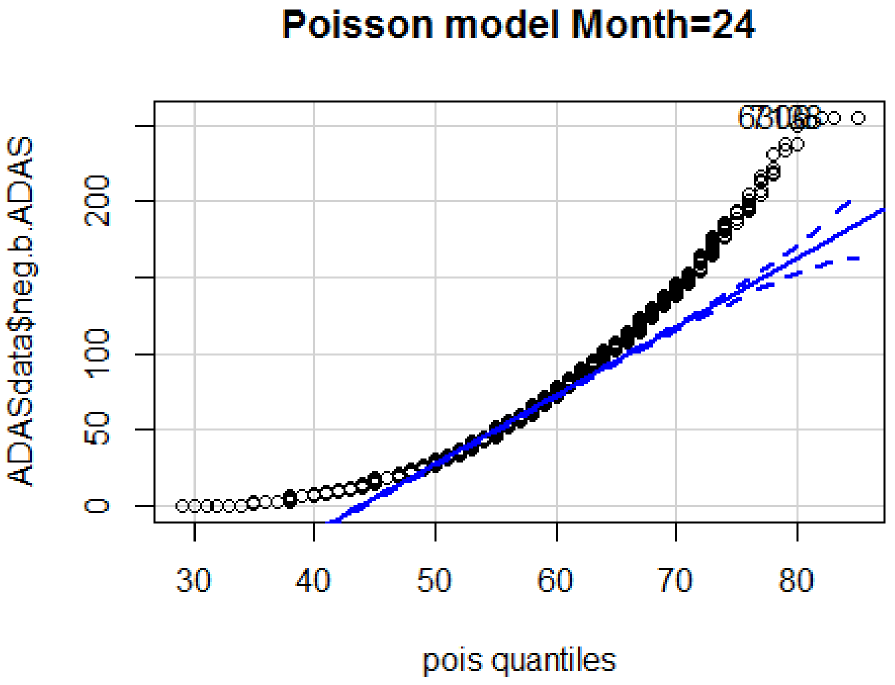


```
[1] 6306 7138

poisson <- fitdistr(ADASdata$neg.b.ADAS[ADASdata$M==48], "Poisson")
qqp(ADASdata$neg.b.ADAS, "pois", lambda=poisson$estimate, main="Poisson model
Month=48")
```

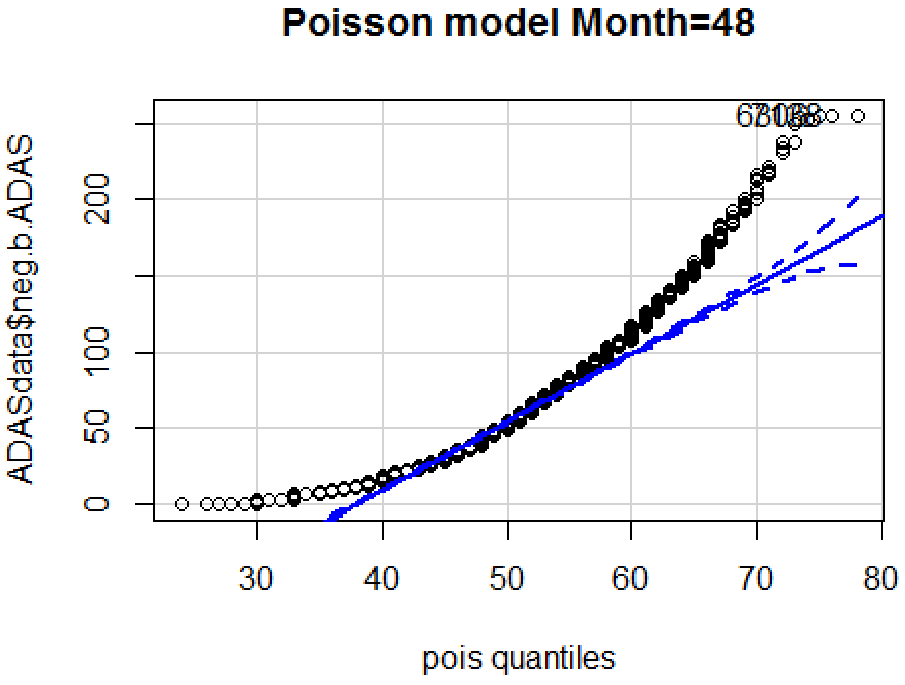


```
[1] 6306 7138
```

```
poisson <- fitdistr(ADASdata$neg.b.ADAS[ADASdata$M==72], "Poisson")
qqp(ADASdata$neg.b.ADAS, "pois", lambda=poisson$estimate, main="Poisson model
Month=72")
```

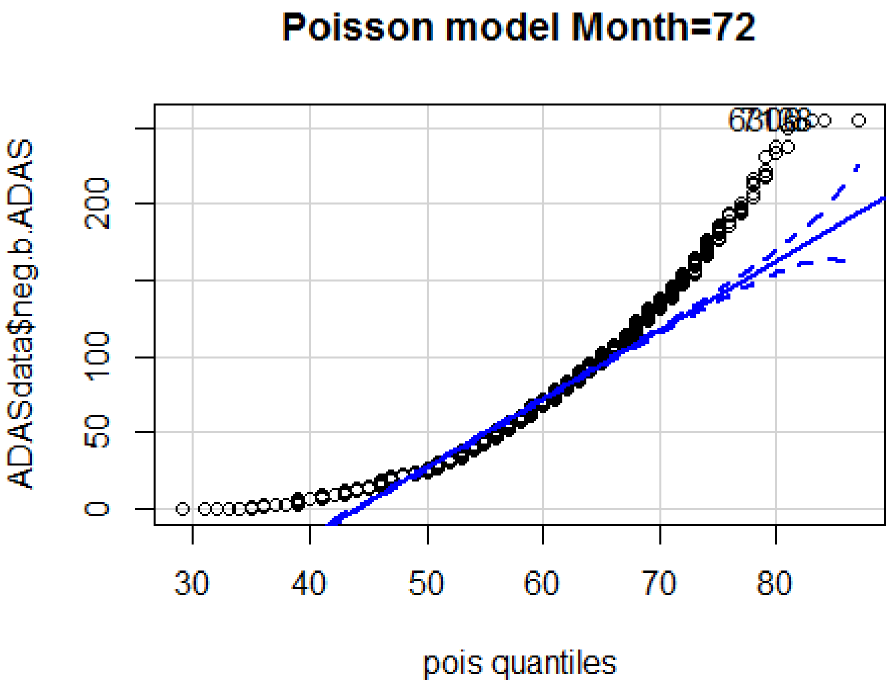


```
[1] 6306 7138

poisson <- fitdistr(ADASdata$neg.b.ADAS[ADASdata$M==120], "Poisson")
qqp(ADASdata$neg.b.ADAS, "pois", lambda=poisson$estimate, main="Poisson model
Month=120")
```

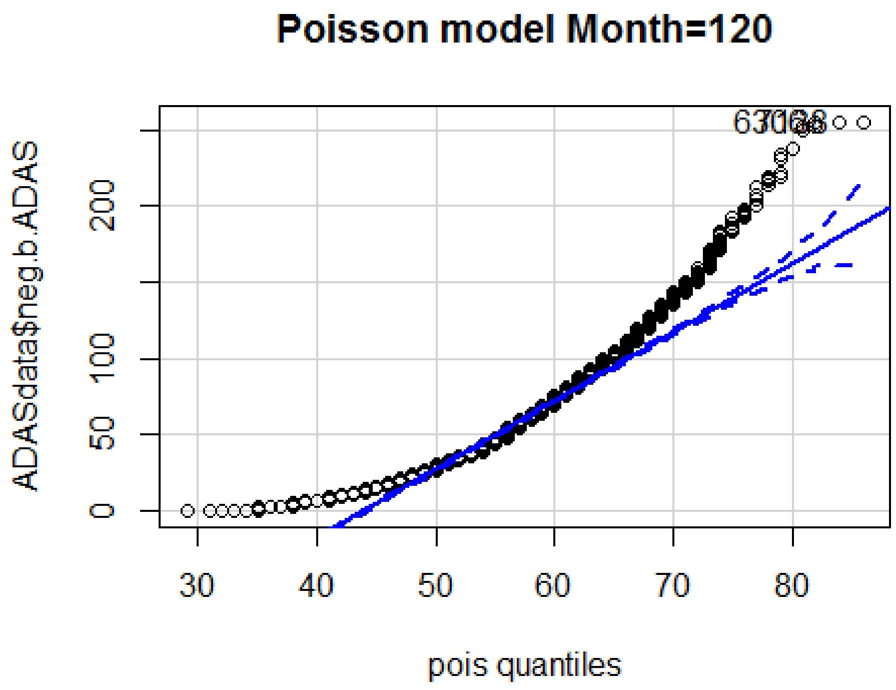


```
[1] 6306 7138
mean(ADASdata$neg.b.ADAS[ADASdata$M==0])
[1] 52.23211
var(ADASdata$neg.b.ADAS[ADASdata$M==0])
[1] 828.3178
mean(ADASdata$neg.b.ADAS[ADASdata$M==12])
[1] 53.92857
var(ADASdata$neg.b.ADAS[ADASdata$M==12])
[1] 1282.4
mean(ADASdata$neg.b.ADAS[ADASdata$M==24])
[1] 54.69595
var(ADASdata$neg.b.ADAS[ADASdata$M==24])
[1] 1483.316
mean(ADASdata$neg.b.ADAS[ADASdata$M==48])
[1] 49.02997
var(ADASdata$neg.b.ADAS[ADASdata$M==48])
[1] 1410.121
mean(ADASdata$neg.b.ADAS[ADASdata$M==72])
```

```
[1] 55.54958

var(ADASdata$neg.b.ADAS[ADASdata$M==72])

[1] 1629.714

mean(ADASdata$neg.b.ADAS[ADASdata$M==120])

[1] 55.09589

var(ADASdata$neg.b.ADAS[ADASdata$M==120])

[1] 1342.56

gamma <- fitdistr((ADASdata$neg.b.ADAS[ADASdata$M==0]+1), "gamma")
qqp(ADASdata$neg.b.ADAS[ADASdata$M==0]+1, "gamma", shape = gamma$estimate[[1]],
rate = gamma$estimate[[2]], main="Gamma model Month=0")
```

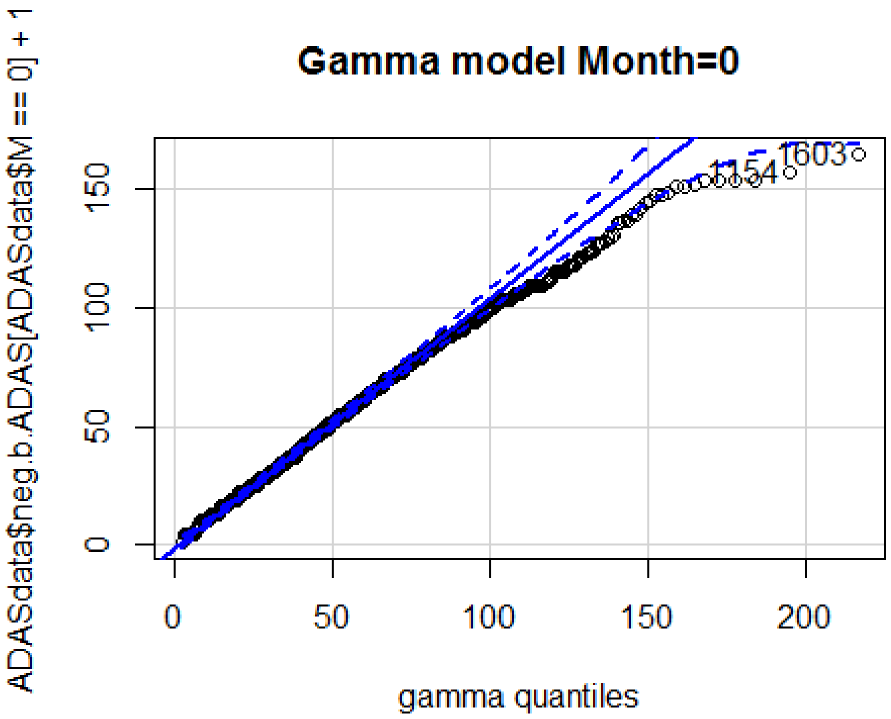


```
[1] 1603 1154

gamma <- fitdistr((ADASdata$neg.b.ADAS[ADASdata$M==12]+1), "gamma")
qqp(ADASdata$neg.b.ADAS[ADASdata$M==12]+1, "gamma", shape = gamma$estimate[[1]],
rate = gamma$estimate[[2]], main="Gamma model Month=12")
```

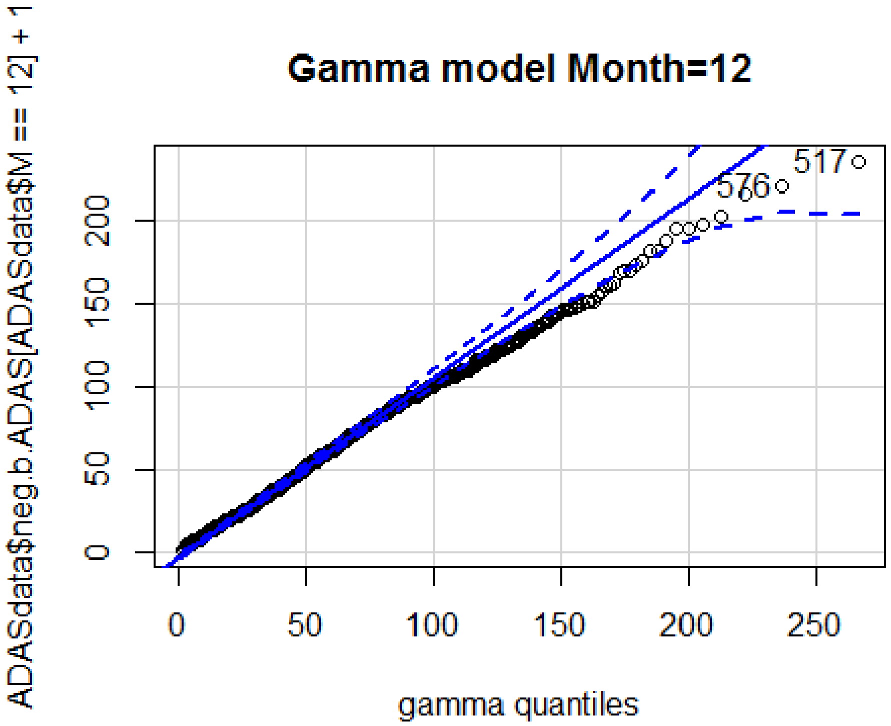


```
[1] 517 576

gamma <- fitdistr((ADASdata$neg.b.ADAS[ADASdata$M==24]+1), "gamma")
qqp(ADASdata$neg.b.ADAS[ADASdata$M==24]+1, "gamma", shape = gamma$estimate[[1]],
rate = gamma$estimate[[2]], main="Gamma model Month=24")
```

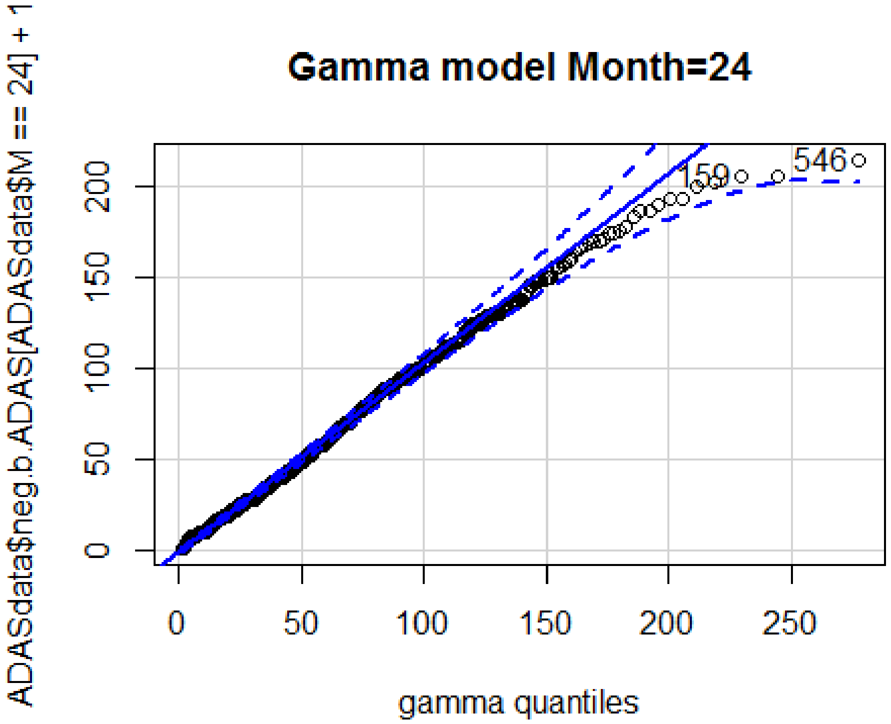


```
[1] 546 159
```

```
gamma <- fitdistr((ADASdata$neg.b.ADAS[ADASdata$M==48]+1), "gamma")
qqp(ADASdata$neg.b.ADAS[ADASdata$M==48]+1, "gamma", shape = gamma$estimate[[1]],
rate = gamma$estimate[[2]], main="Gamma model Month=48")
```

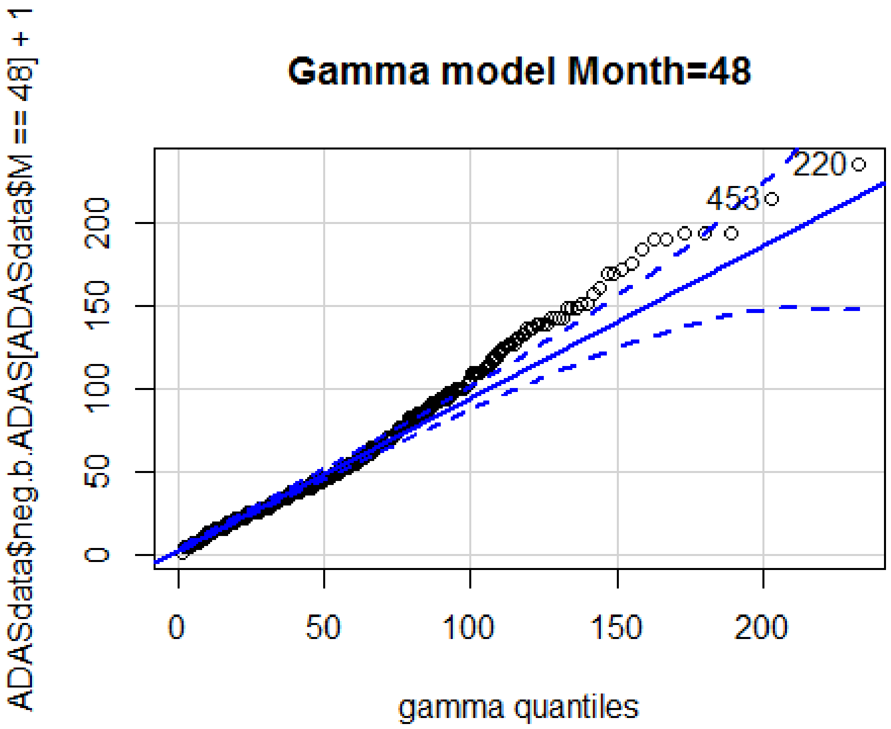


```
[1] 220 453

gamma <- fitdistr((ADASdata$neg.b.ADAS[ADASdata$M==72]+1), "gamma")
qqp(ADASdata$neg.b.ADAS[ADASdata$M==72]+1, "gamma", shape = gamma$estimate[[1]],
rate = gamma$estimate[[2]], main="Gamma model Month=72")
```

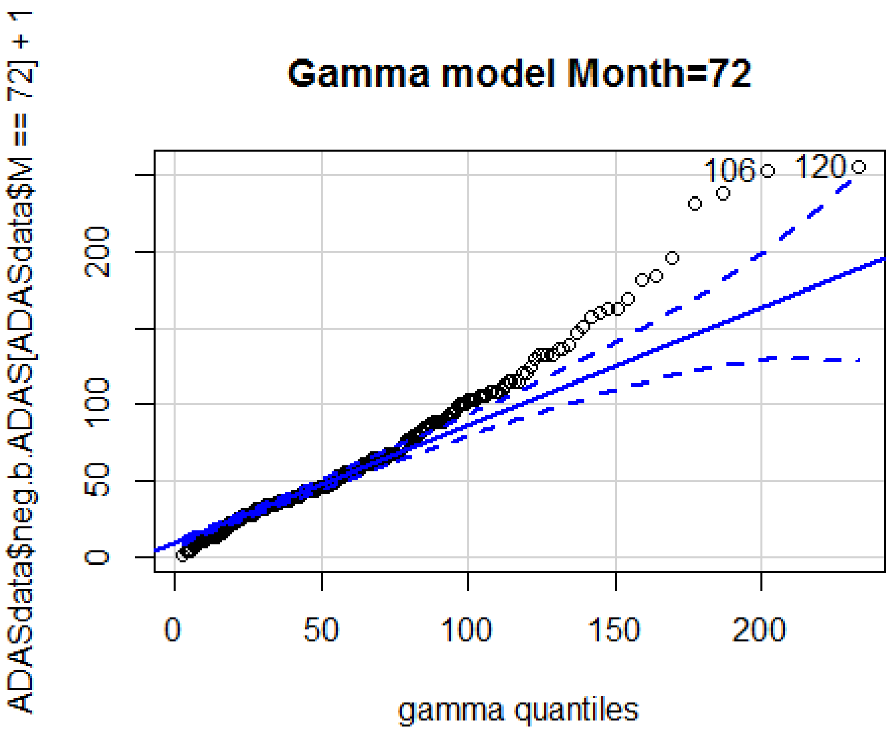


```
[1] 120 106

gamma <- fitdistr((ADASdata$neg.b.ADAS[ADASdata$M==120]+1), "gamma")
qqp(ADASdata$neg.b.ADAS[ADASdata$M==120]+1, "gamma", shape =
gamma$estimate[[1]], rate = gamma$estimate[[2]], main="Gamma model Month=120")
```

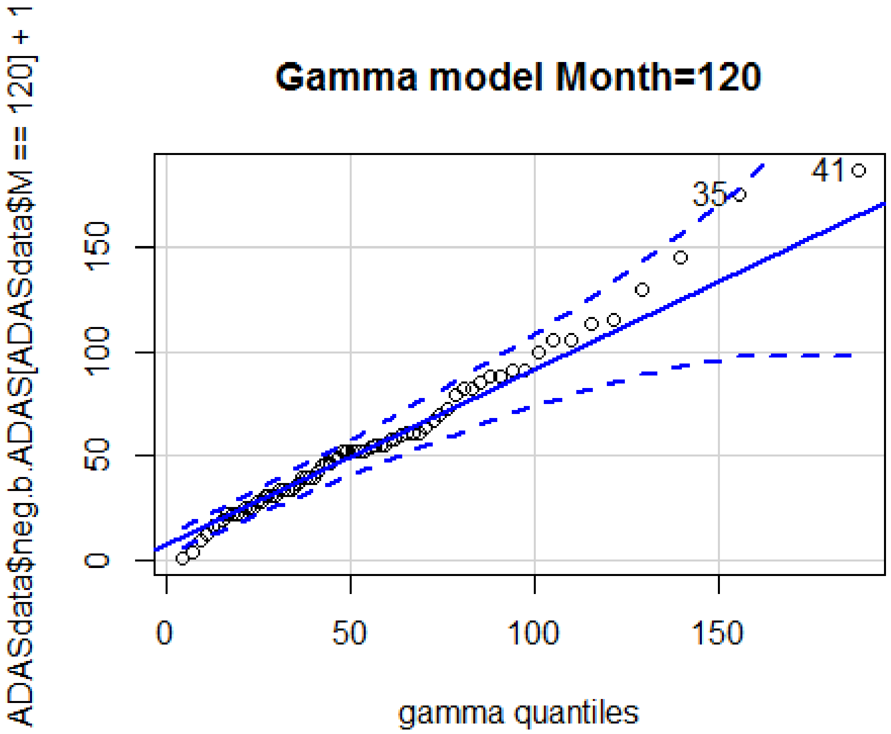


```
[1] 41 35
```

```
par(mar = c(3,3,3,3))
hist(ADASdata$proportion)
```

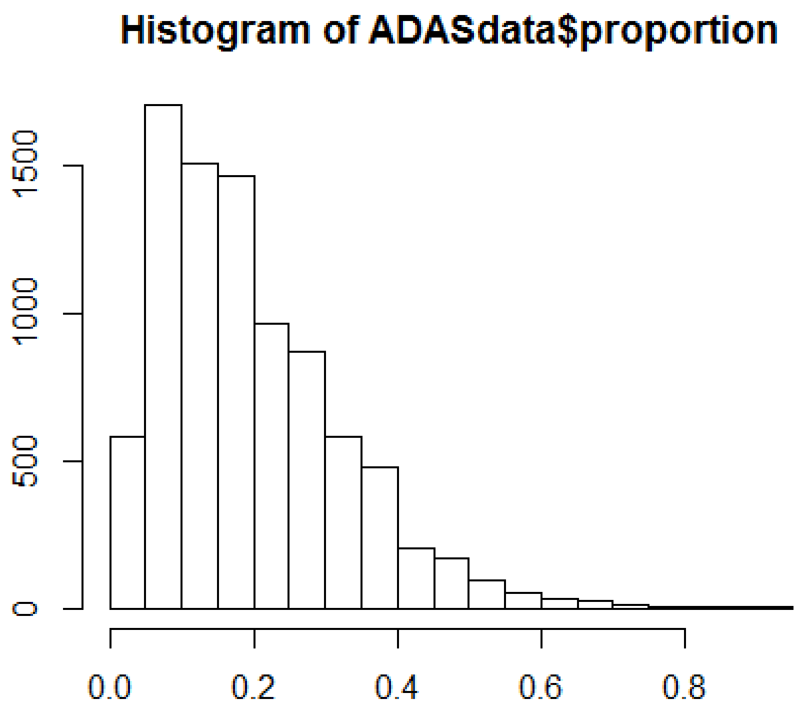


```
data<-ADASdata$proportion[ADASdata$M==0]
params<-fitdistr(data, "logistic")
qqp(data, dist="logis",  params$estimate[[1]]);title(main="Logistic model
Month=0", line=2)
```

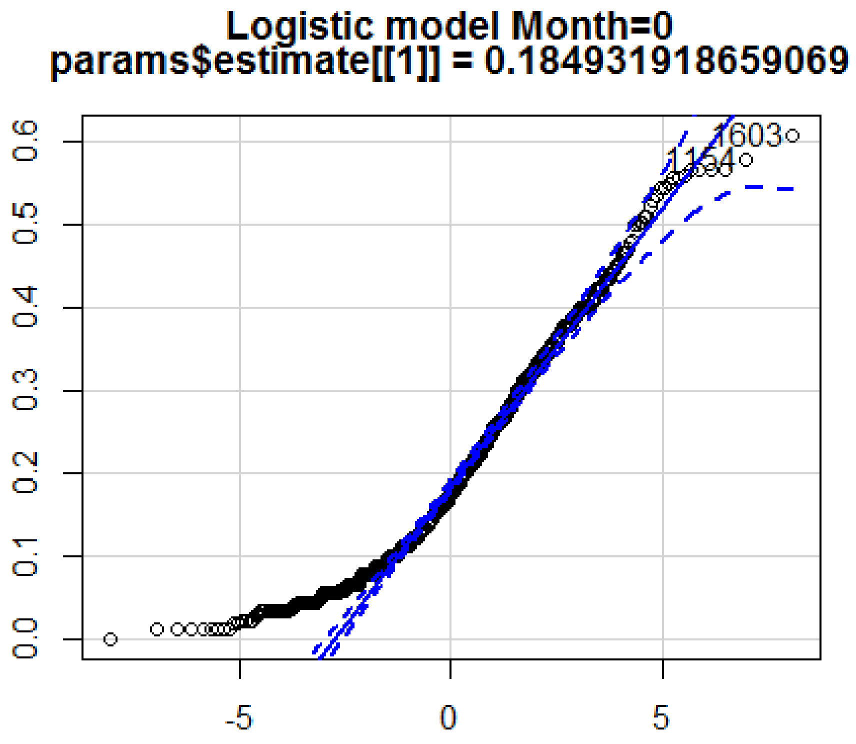

```
data<-ADASdata$proportion[ADASdata$M==12]
params<-fitdistr(data, "logistic")
qqp(data, dist="logis",  params$estimate[[1]]);title(main="Logistic model
Month=12", line=2)
```

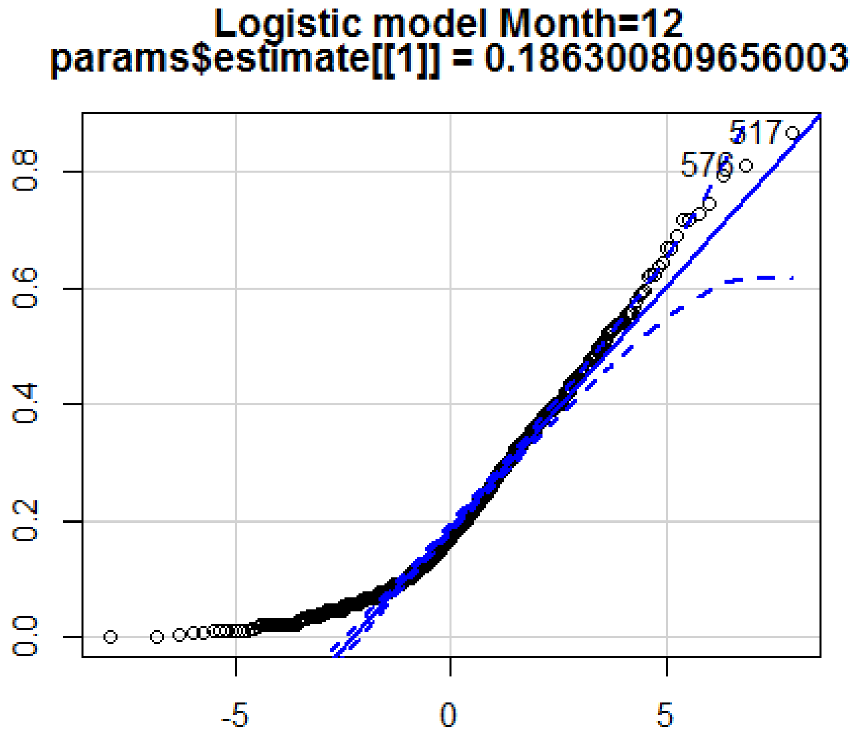


```
data<-ADASdata$proportion[ADASdata$M==24]
params<-fitdistr(data, "logistic")
qqp(data, dist="logis",  params$estimate[[1]]);title(main="Logistic model
Month=24", line=2)
```

Only

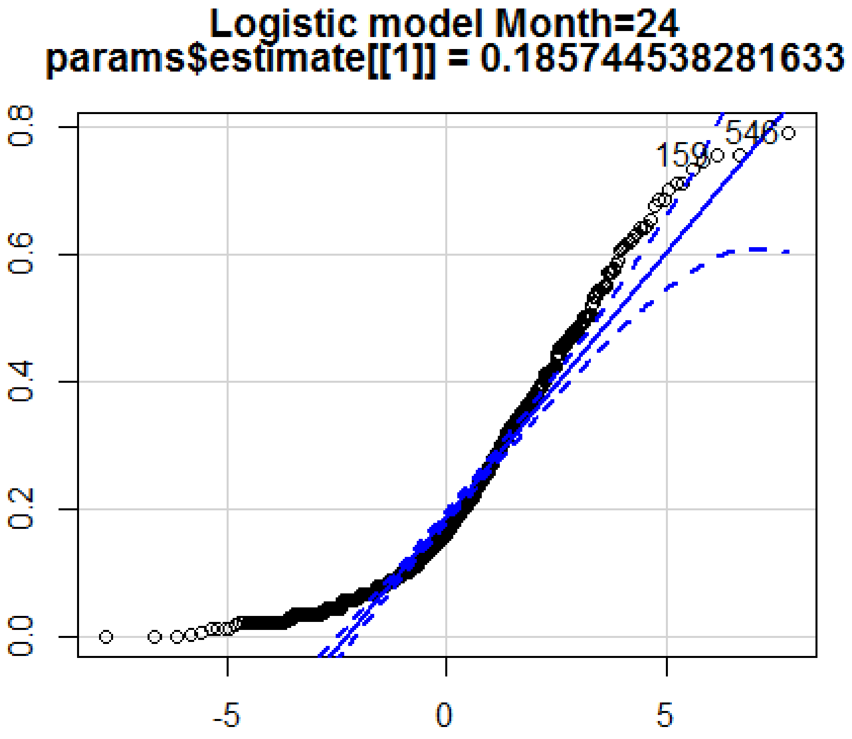


```
data<-ADASdata$proportion[ADASdata$M==48]
params<-fitdistr(data, "logistic")
qqp(data, dist="logis",  params$estimate[[1]]);title(main="Logistic model
Month=48", line=2)
```

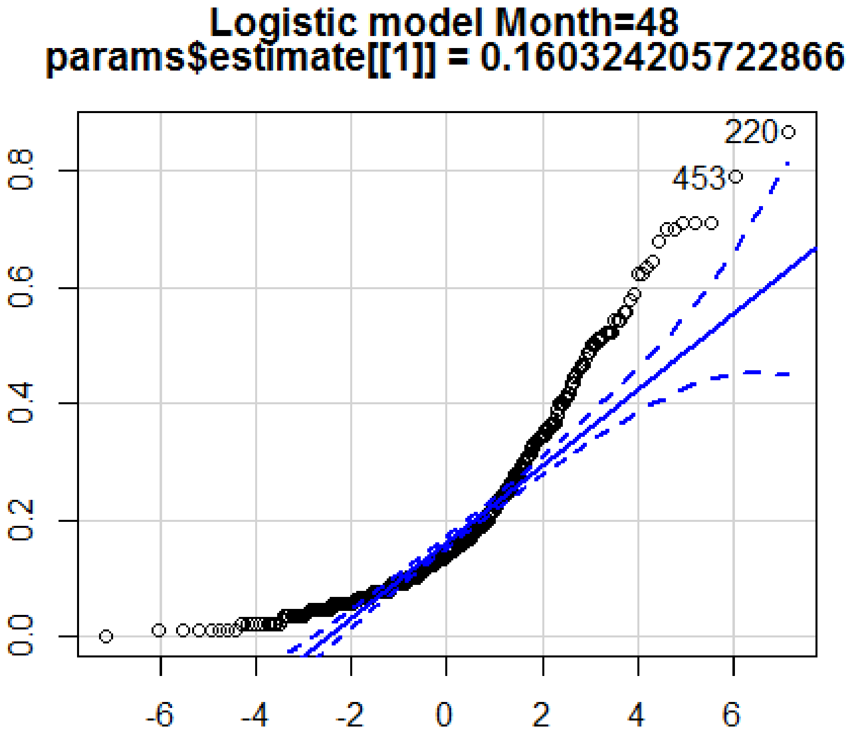


```
data<-ADASdata$proportion[ADASdata$M==72]
params<-fitdistr(data, "logistic")
```

```
qqp(data, dist="logis",  params$estimate[[1]]);title(main="Logistic model
Month=72", line=2)
```

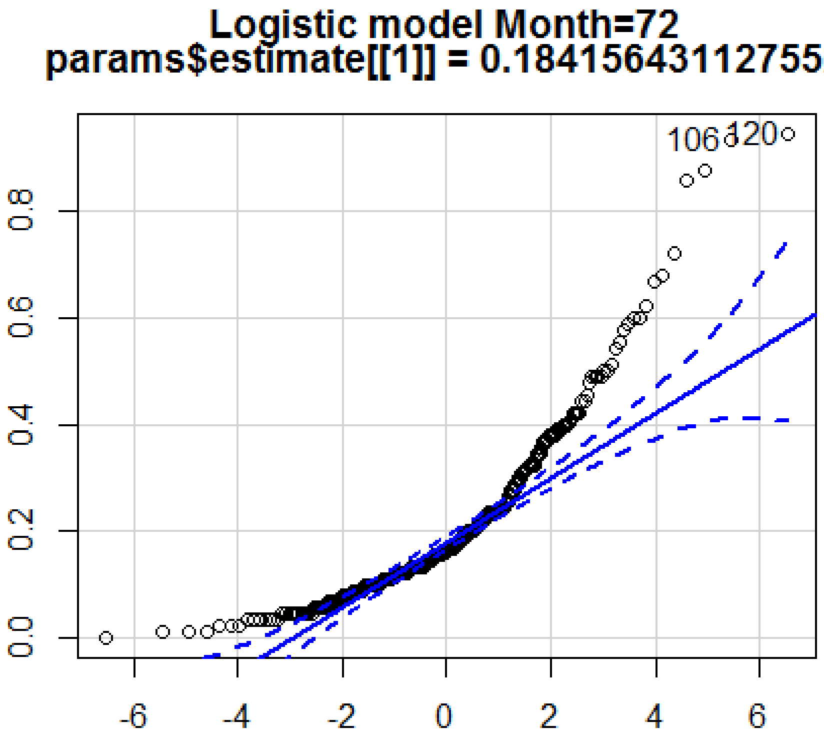


```
data<-ADASdata$proportion[ADASdata$M==120]
params<-fitdistr(data, "logistic")
qqp(data, dist="logis",params$estimate[[1]]);title(main="Logistic model
Month=120", line=2)
```

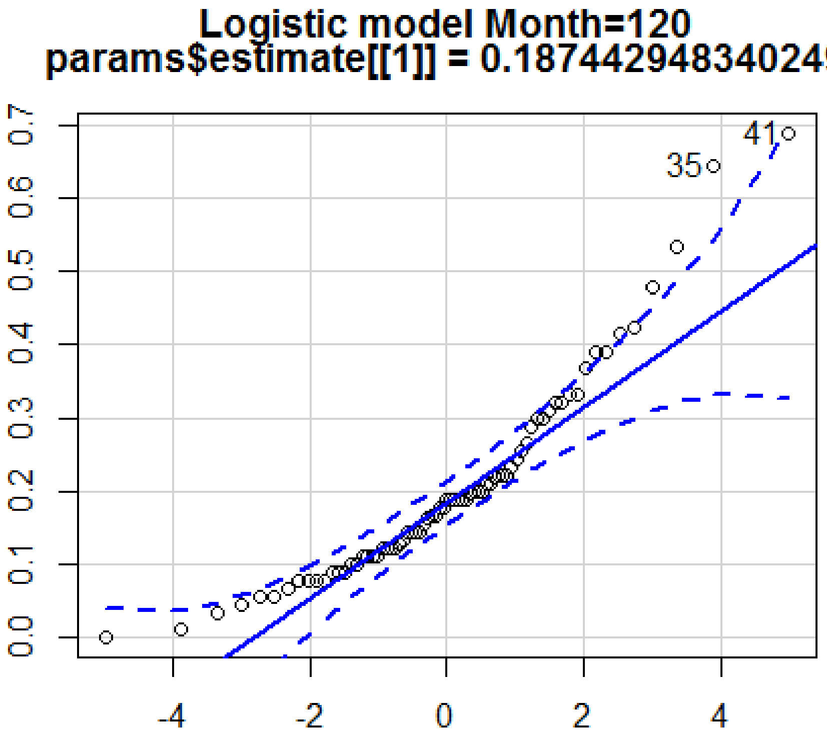

```
data<-ADASdata$neg.b.ADAS[ADASdata$M==0]
params<-fitdistr(data, "exponential")
qqp(data, "exp",rate = params$estimate, main="Exponential model Month=0")
```

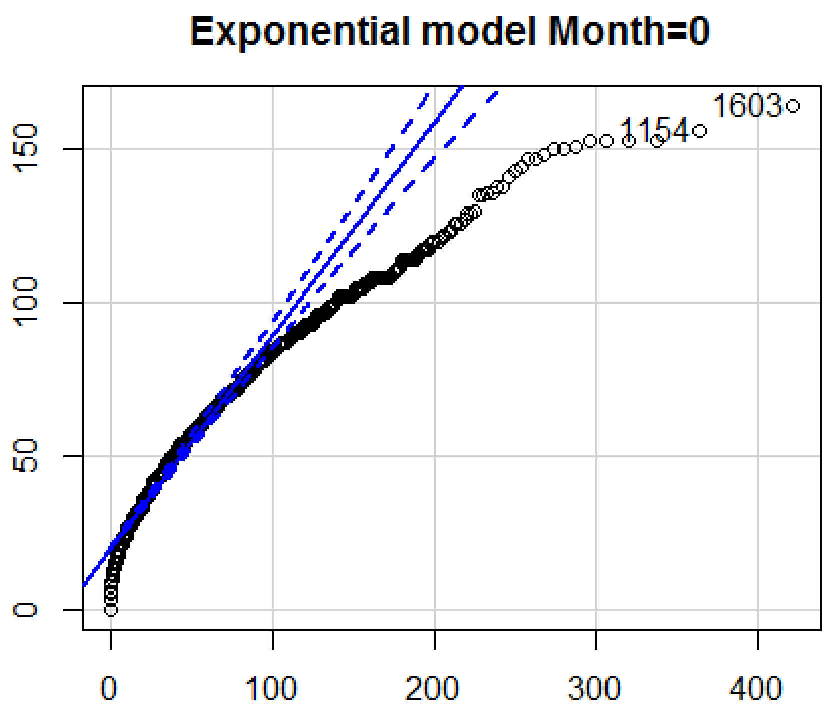


```
[1] 1603 1154

data<-ADASdata$neg.b.ADAS[ADASdata$M==12]
params<-fitdistr(data, "exponential")
qqp(data, "exp",rate = params$estimate, main="Exponential model Month=12")
```

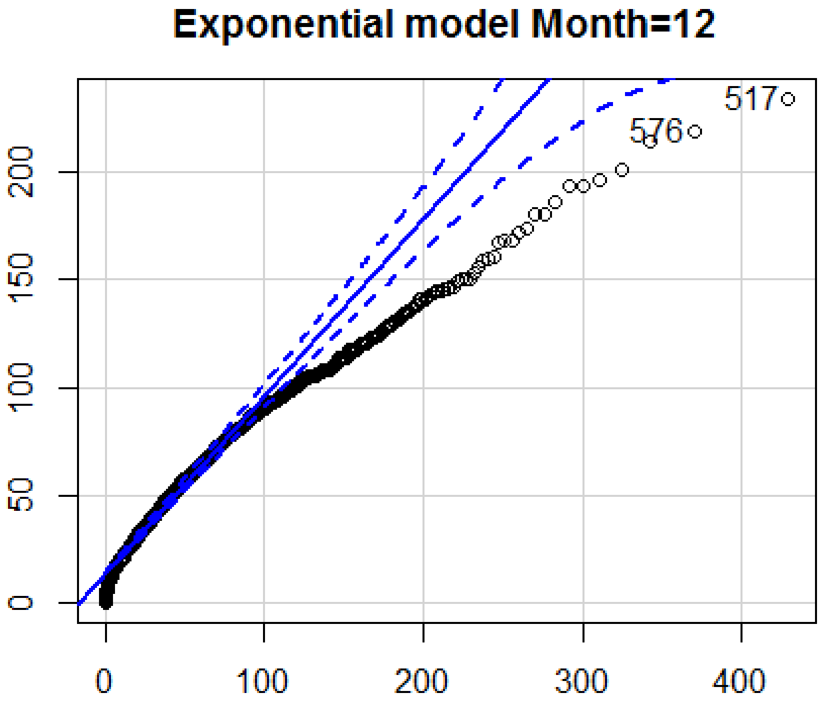


```
[1] 517 576

data<-ADASdata$neg.b.ADAS[ADASdata$M==24]
params<-fitdistr(data, "exponential")
qqp(data, "exp",rate = params$estimate, main="Exponential model Month=24")
```

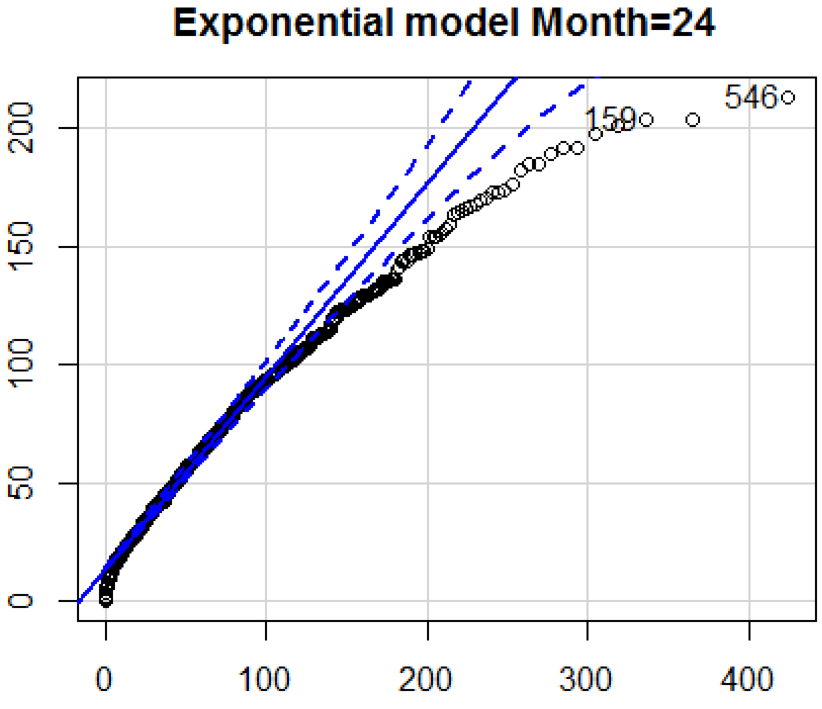


```
[1] 546 159
```

```
data<-ADASdata$neg.b.ADAS[ADASdata$M==48]
params<-fitdistr(data, "exponential")
qqp(data, "exp",rate = params$estimate, main="Exponential model Month=48")
```

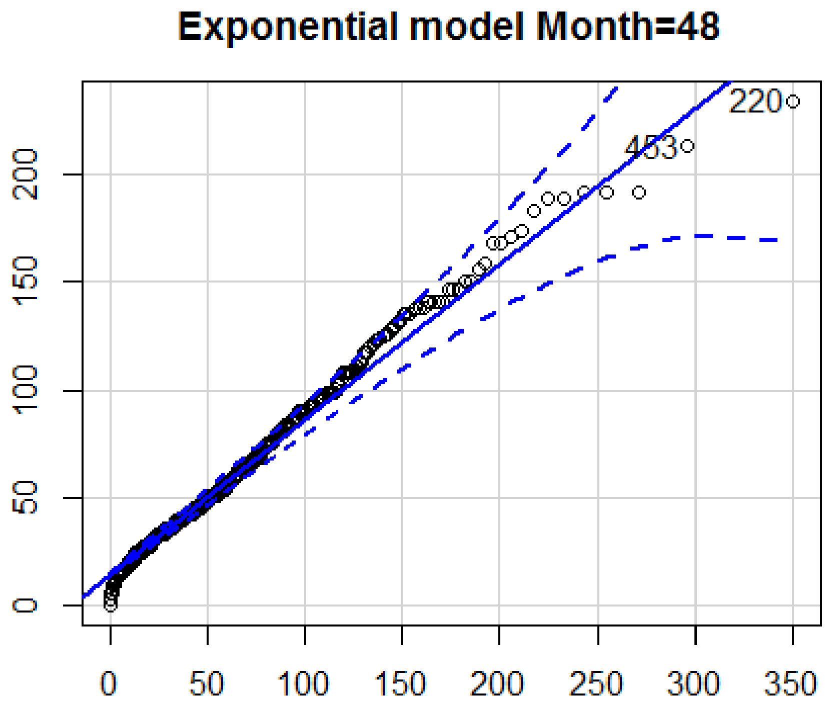


```
[1] 220 453

data<-ADASdata$neg.b.ADAS[ADASdata$M==72]
params<-fitdistr(data, "exponential")
qqp(data, "exp",rate = params$estimate, main="Exponential model Month=72")
```

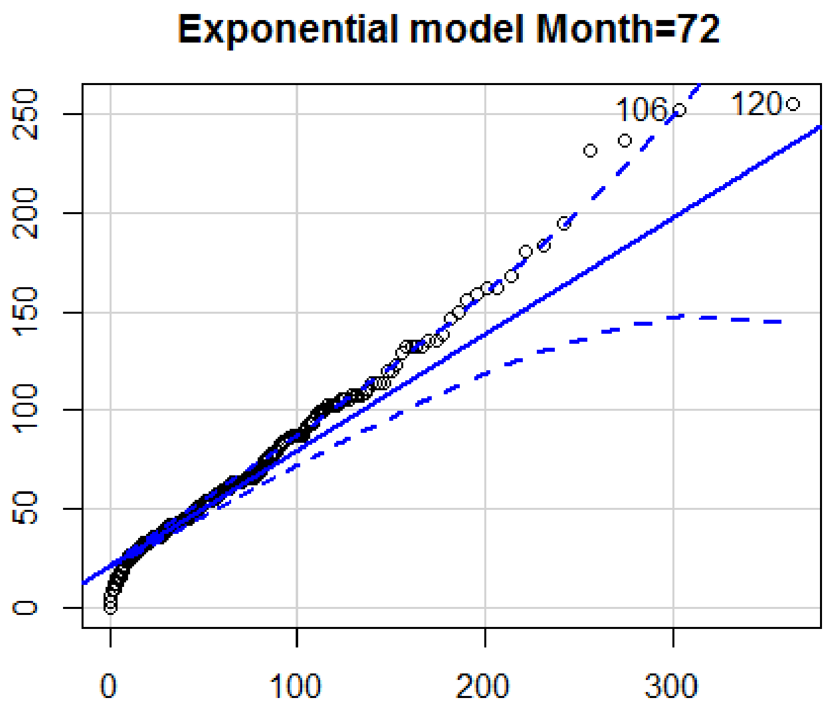


```
[1] 120 106

data<-ADASdata$neg.b.ADAS[ADASdata$M==120]
params<-fitdistr(data, "exponential")
qqp(data, "exp",rate = params$estimate, main="Exponential model Month=120")
```

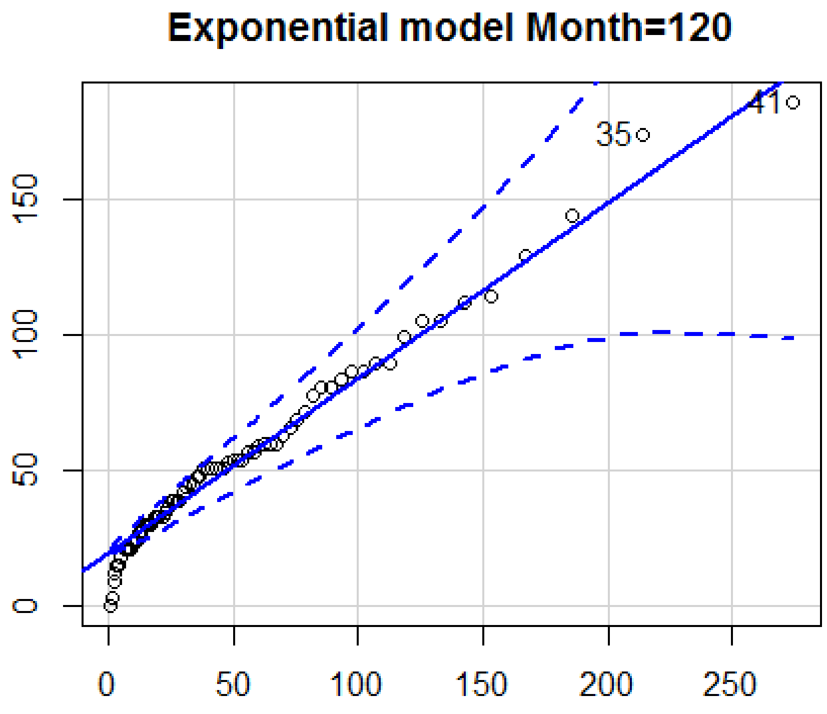


```
[1] 41 35
```

8.6. *Build base negative binomial model and comparing the different paramaterization methods.*

There are two commonly used paramaterization methods used in the negative binomial model. To evaluate which to continue with all biological relavent main effect explainitory variables were include in the models and initial model diagnostic were performed. Using the AIC and log-likelihood statistics we can see that the Nbinom1 paramaterization method is prefered. This uses a varaince proportional to the mean method of paramaterization.

```
neg.a.binom1 <- glmmadmb(neg.b.ADAS~AGE + APOE4 + M + Gender + edu.cat + diagn +
vasc+diab+aspirin+parac+diclo+naprox+celex+Ibu+(1|ID), family="nbinom",
data=ADASdata)

neg.a.binom2 <- glmmadmb(neg.b.ADAS~AGE + APOE4 + M + Gender + edu.cat + diagn +
vasc+diab+aspirin+parac+diclo+naprox+celex+Ibu+ (1|ID), family="nbinom1",
data=ADASdata)

AIC(logLik(neg.a.binom1))

[1] 73176.2

AIC(logLik(neg.a.binom2))

[1] 72520.6

logLik(neg.a.binom1)

'log Lik.' -36566.1 (df=22)

logLik(neg.a.binom2)

'log Lik.' -36238.3 (df=22)
```

8.7. *Observing the distribution of the residuals in for the initial model.*

Residuals within each explanitory variables collectively and within each individual show no trends and are homoskedastic centred around zero. Therefore this model is accurate and will be used to investiage the effects of the input variables (including pain medications) going forward.

```
augDat <- data.frame(ADASdata,resid=residuals(neg.a.binom2,type="pearson"),
fitted=fitted(neg.a.binom2))
ggplot(augDat,aes(x=Gender,y=resid,group=ID))+geom_boxplot()+coord_flip()
```

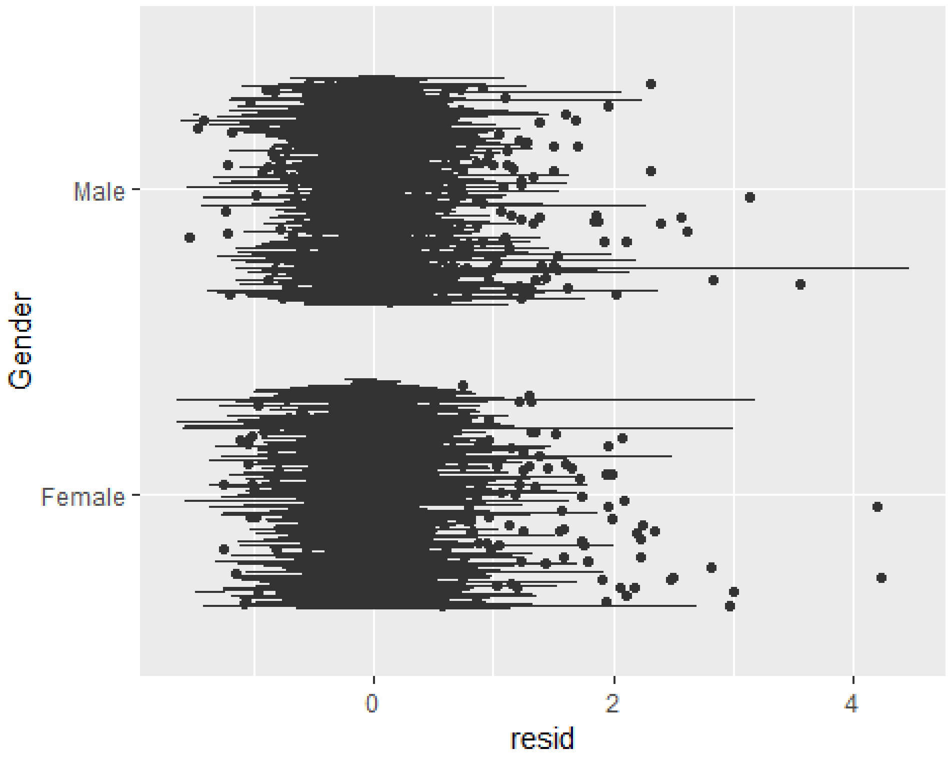


```
ggplot(augDat,aes(x=AGE,y=resid,group=ID))+geom_boxplot()+coord_flip()
```

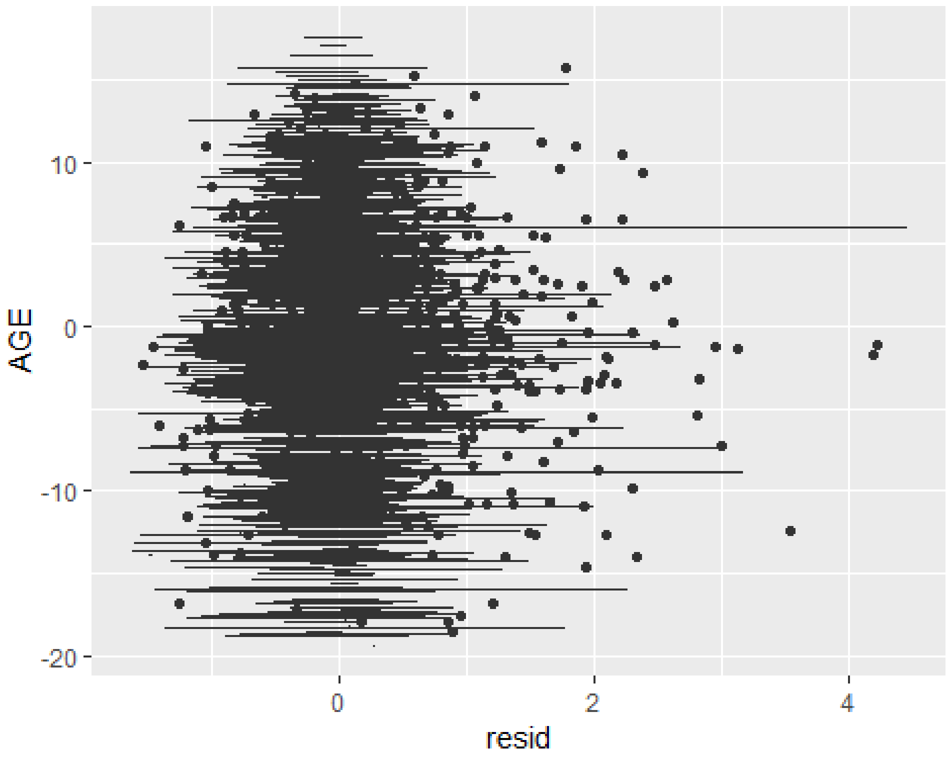


```
ggplot(augDat,aes(x=edu.cat,y=resid,group=ID))+geom_boxplot()+coord_flip()
```

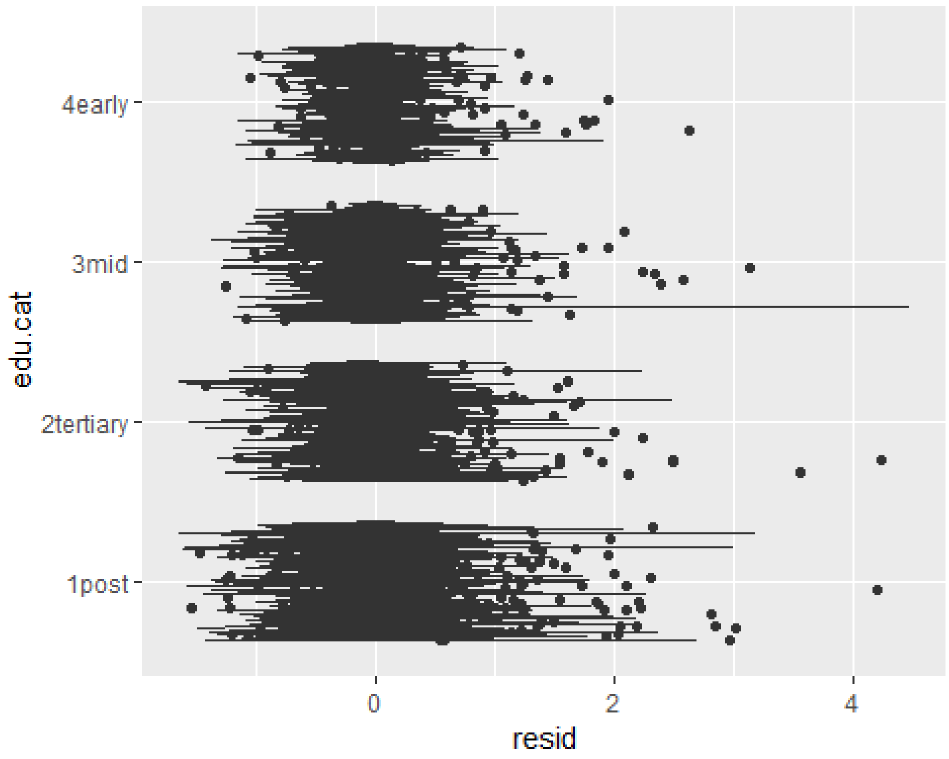


```
ggplot(augDat,aes(x=diagn,y=resid,group=ID))+geom_boxplot()+coord_flip()
```

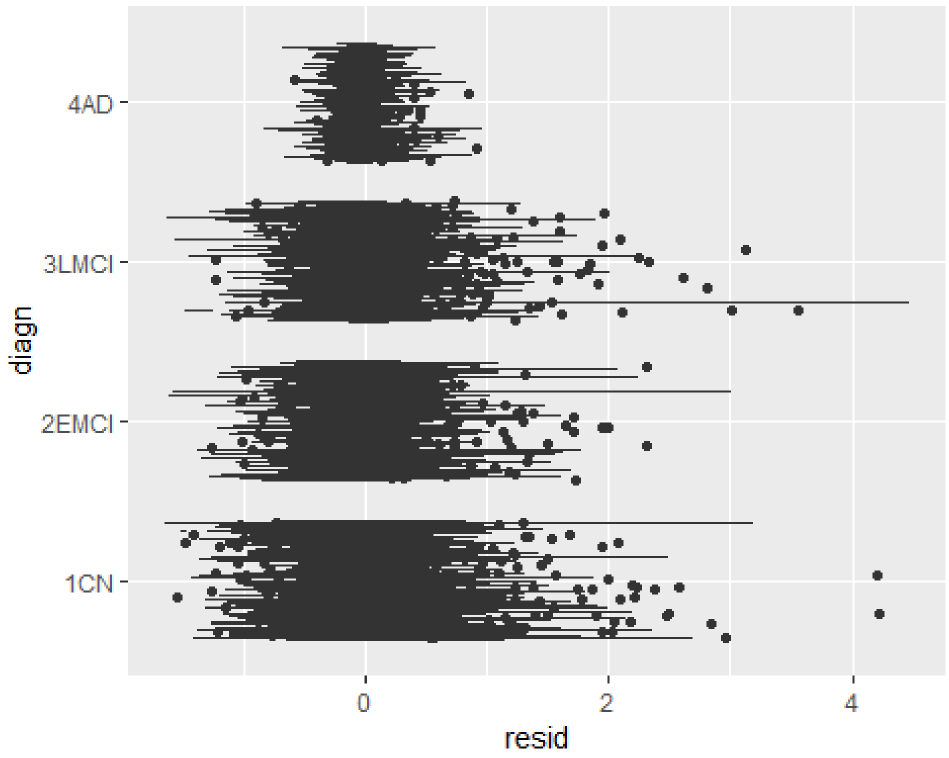


```
ggplot(augDat,aes(x=APOE4,y=resid,group=ID))+geom_boxplot()+coord_flip()
```

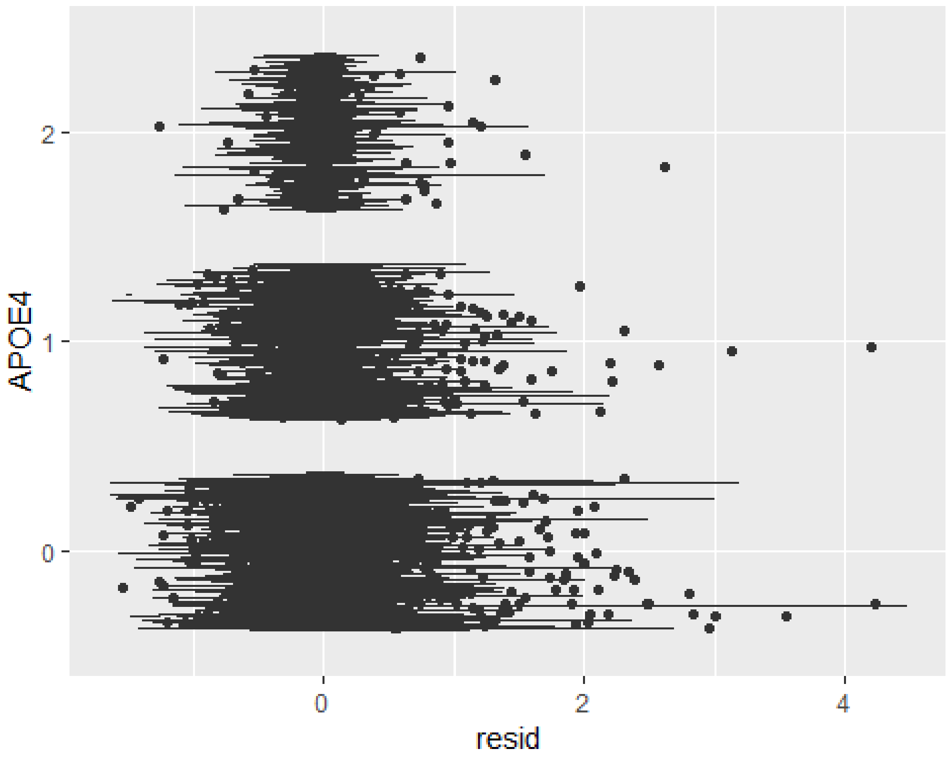


```
ggplot(augDat,aes(x=M,y=resid,group=ID))+geom_boxplot()+coord_flip()
```

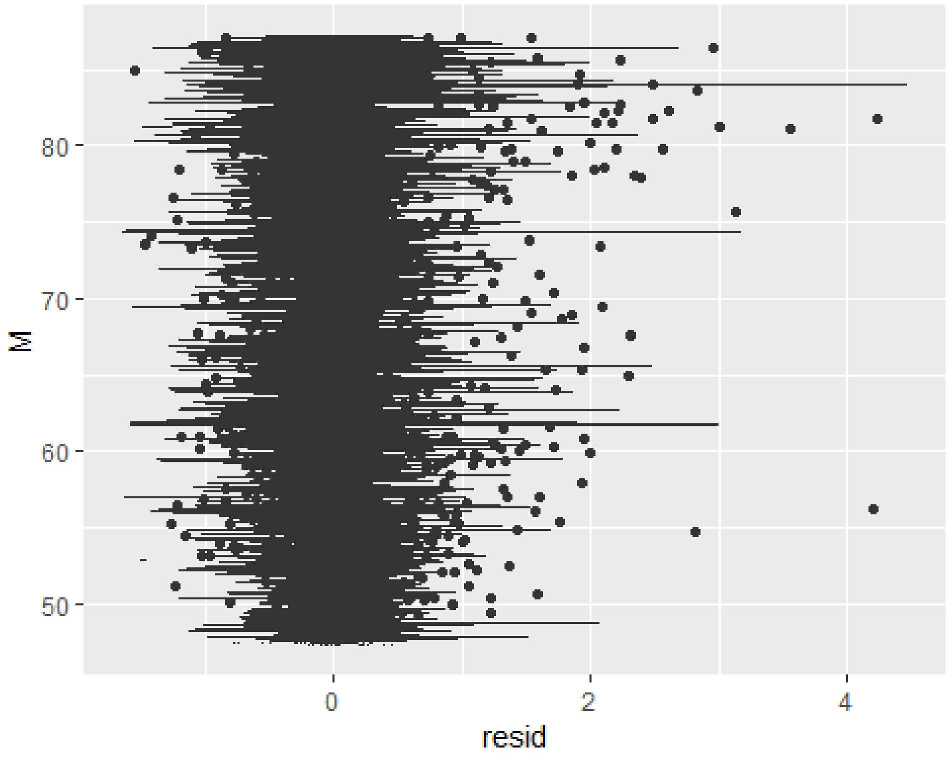


```
ggplot(augDat,aes(x=diclo,y=resid,group=ID))+geom_boxplot()+coord_flip()
```

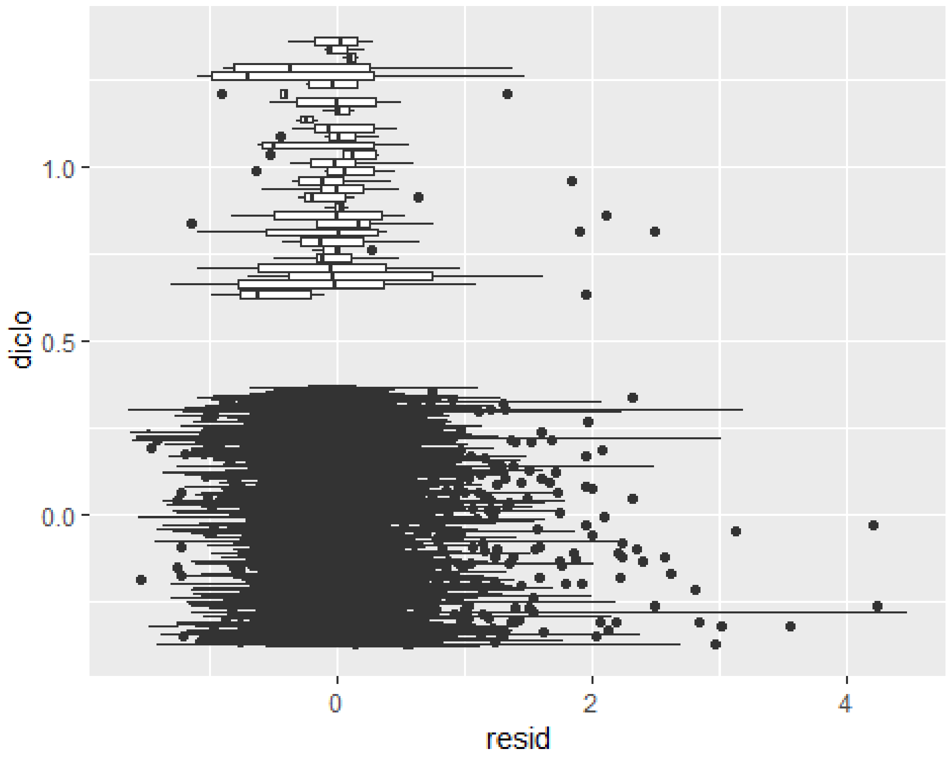


```
ggplot(augDat,aes(x=vasc,y=resid,group=ID))+geom_boxplot()+coord_flip()
```

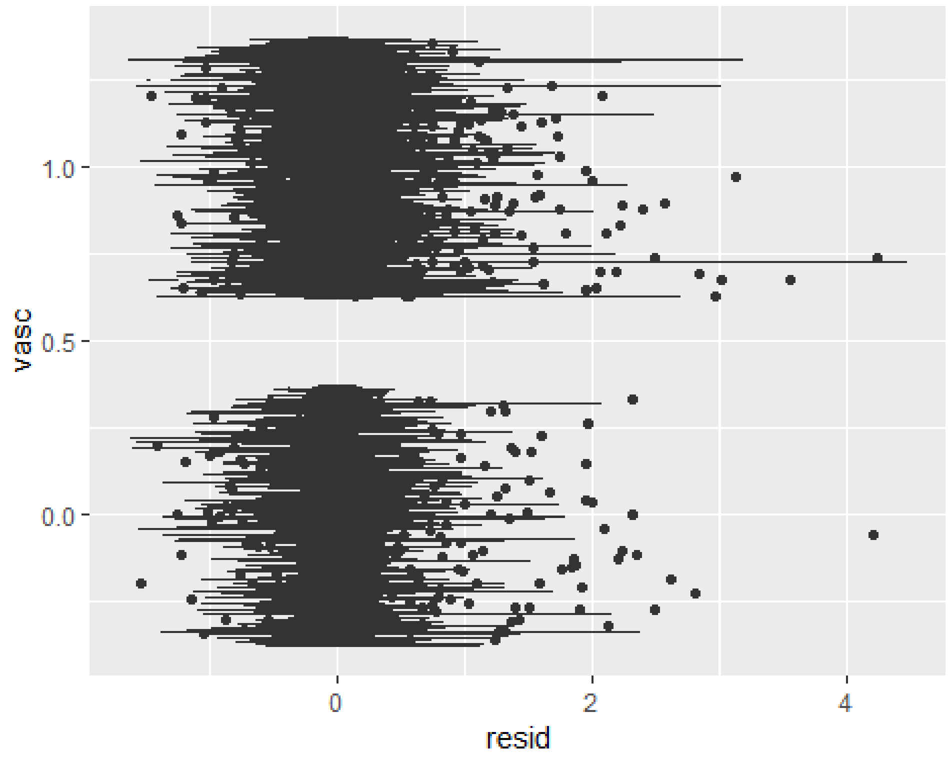


```
ggplot(augDat,aes(x=Ibu,y=resid,group=ID))+geom_boxplot()+coord_flip()
```

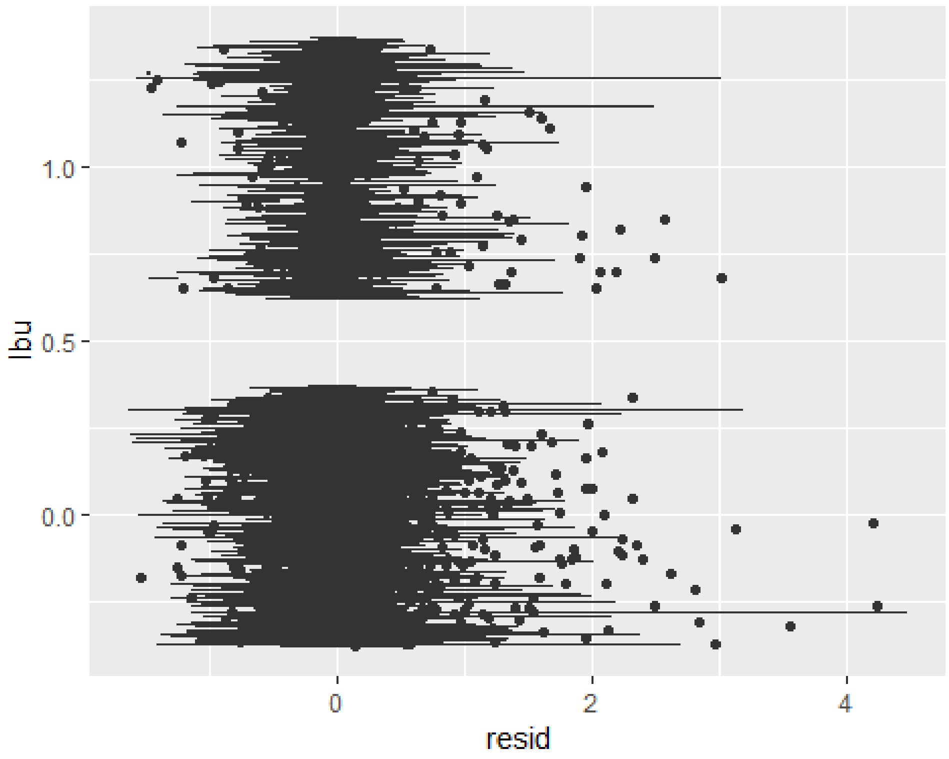


```
ggplot(augDat,aes(x=aspirin,y=resid,group=ID))+geom_boxplot()+coord_flip()
```

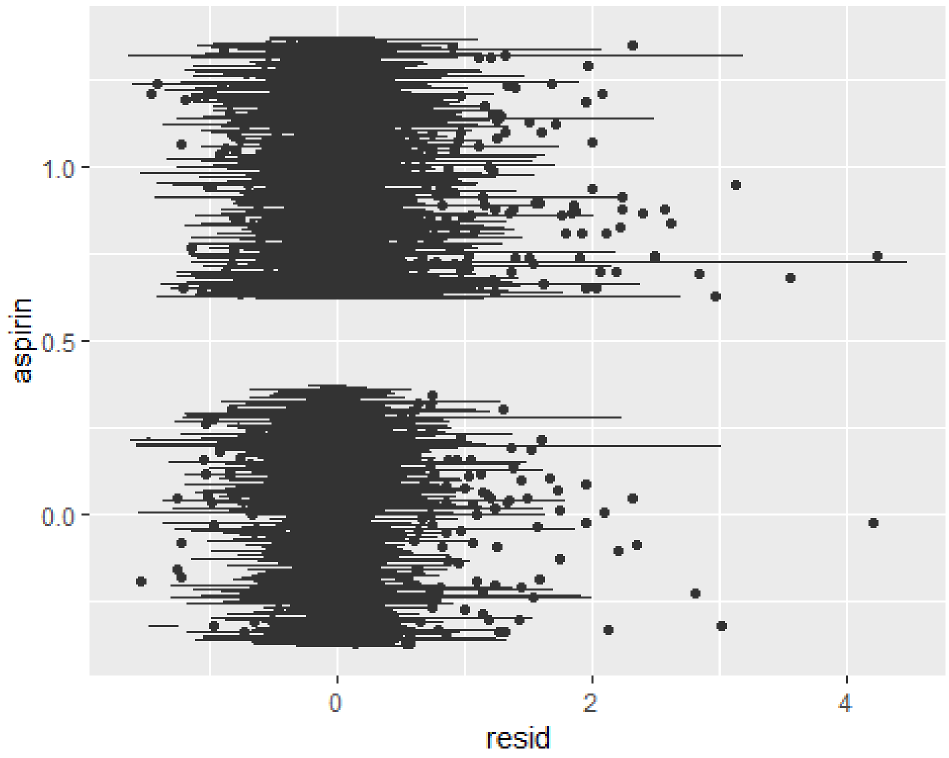


```
ggplot(augDat,aes(x=diab,y=resid,group=ID))+geom_boxplot()+coord_flip()
```

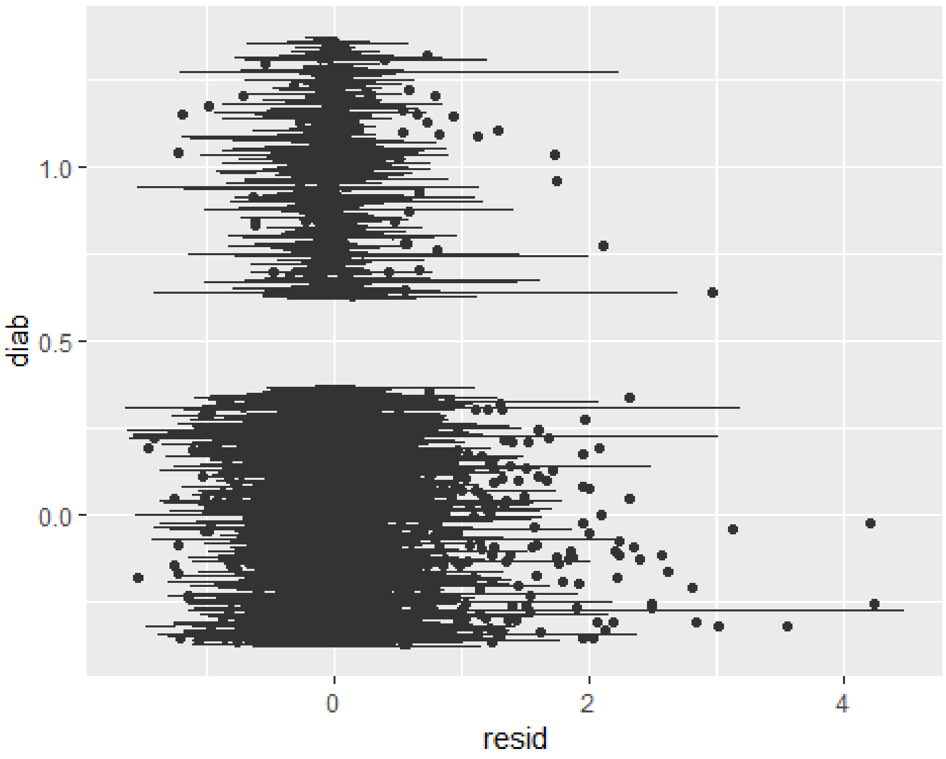


```
ggplot(augDat,aes(x=naprox,y=resid,group=ID))+geom_boxplot()+coord_flip()
```

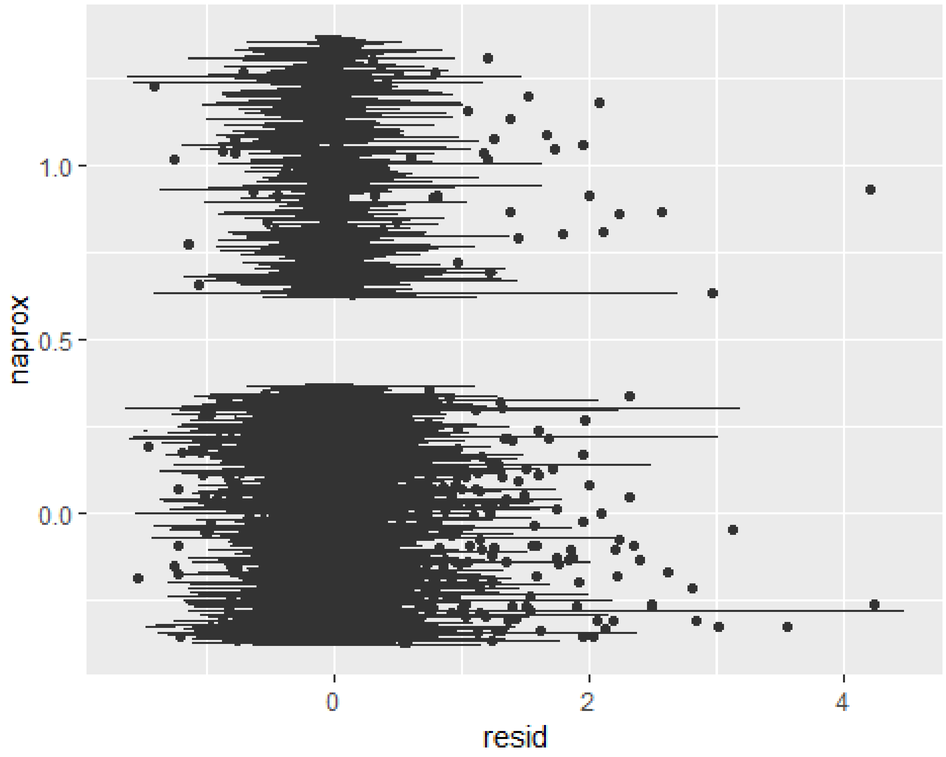


```
ggplot(augDat,aes(x=celex,y=resid,group=ID))+geom_boxplot()+coord_flip()
```

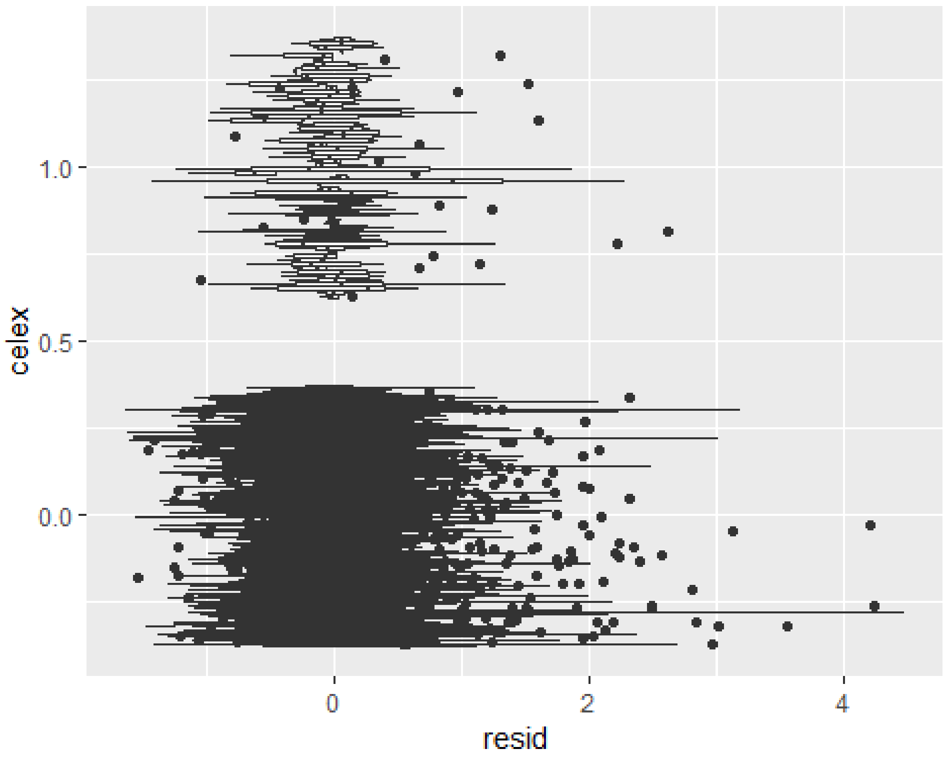


```
ggplot(augDat,aes(x=Gender,y=resid))+geom_boxplot()+coord_flip()
```

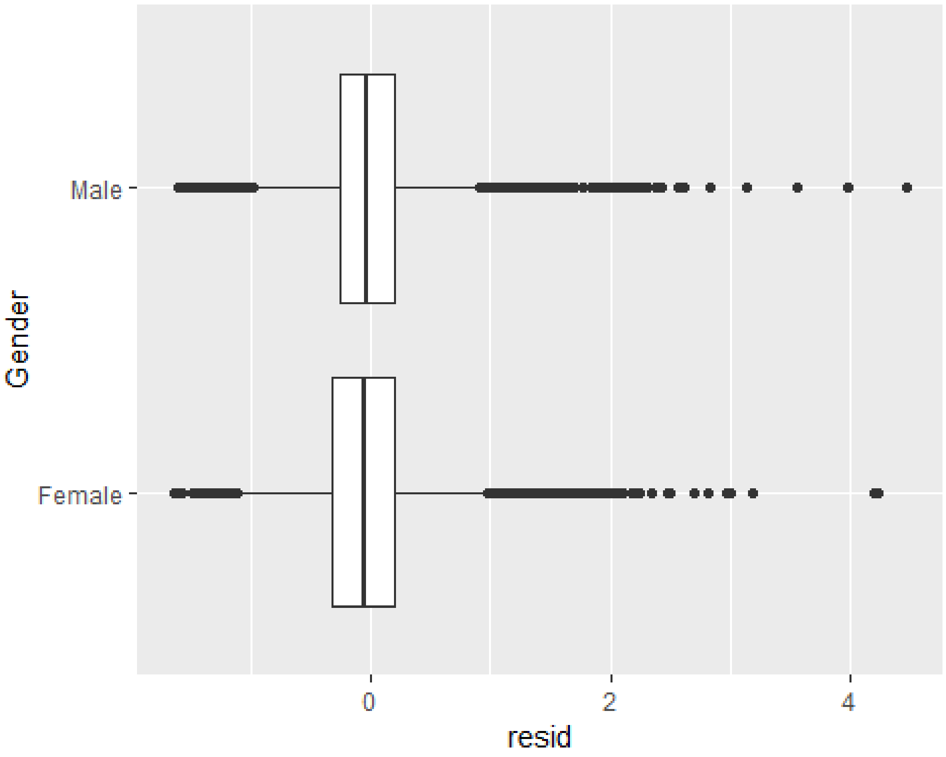


```
ggplot(augDat,aes(x=AGE,y=resid))+geom_point()+coord_flip()+geom_smooth(method=l
m)
```

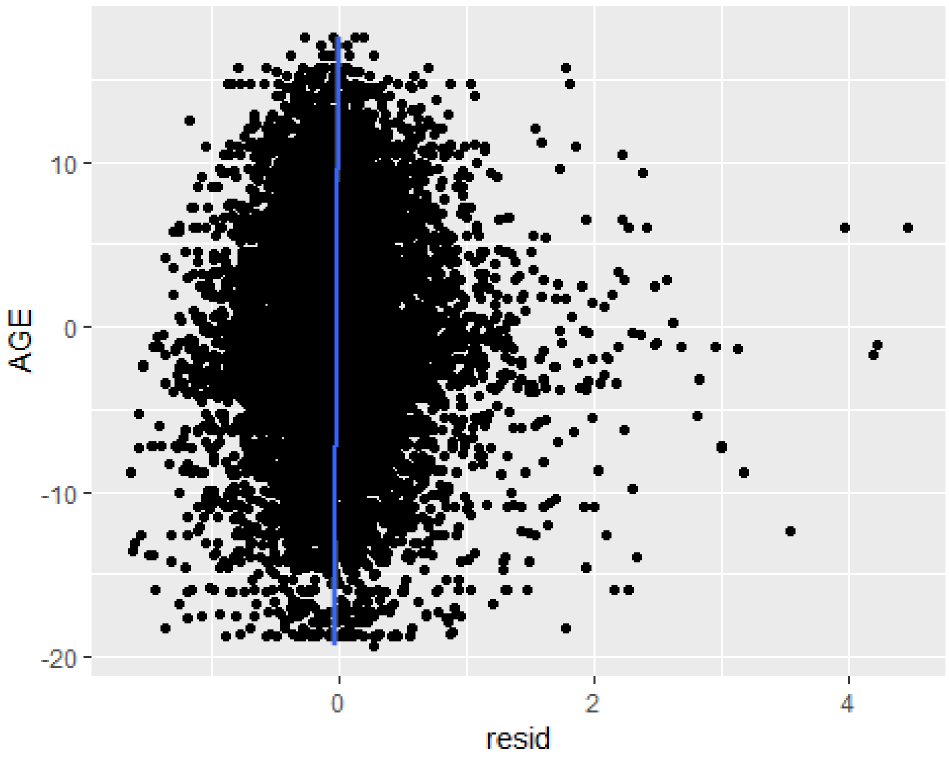


```
ggplot(augDat,aes(x=edu.cat,y=resid))+geom_boxplot()+coord_flip()
```

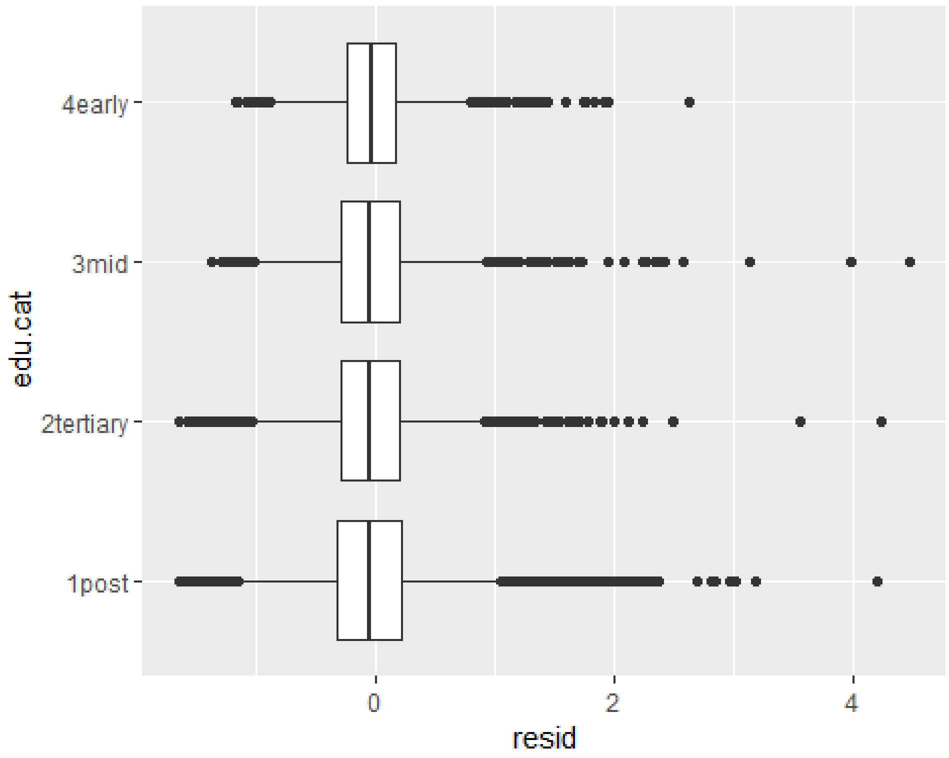


```
ggplot(augDat,aes(x=diagn,y=resid))+geom_boxplot()+coord_flip()
```

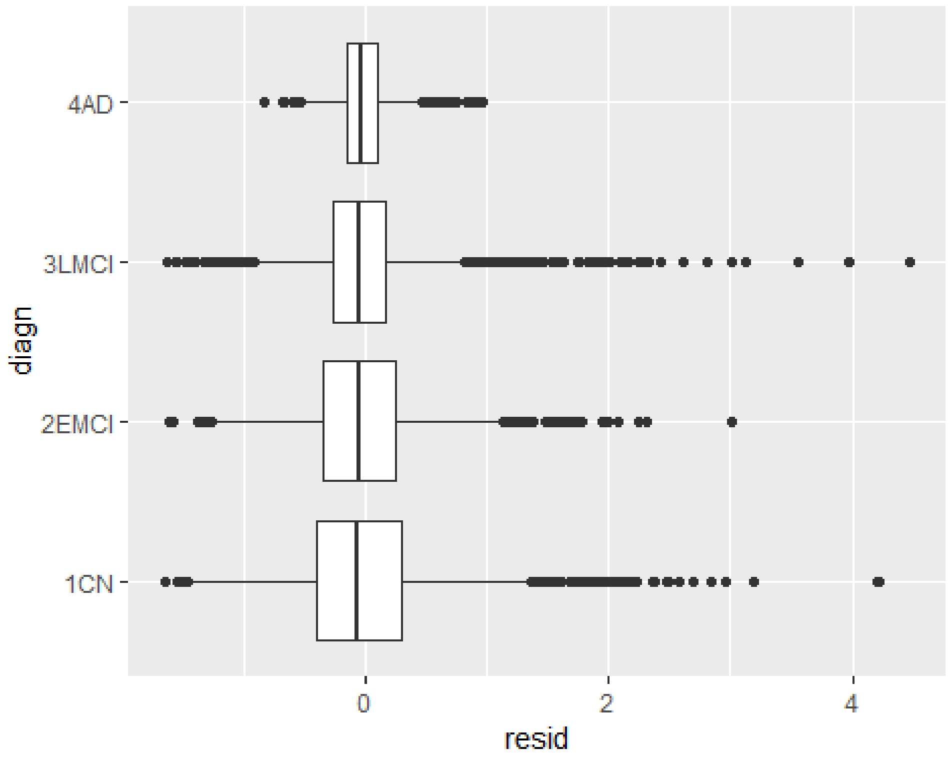


```
ggplot(augDat,aes(x=as.factor(APOE4),y=resid))+geom_boxplot()+coord_flip()
```

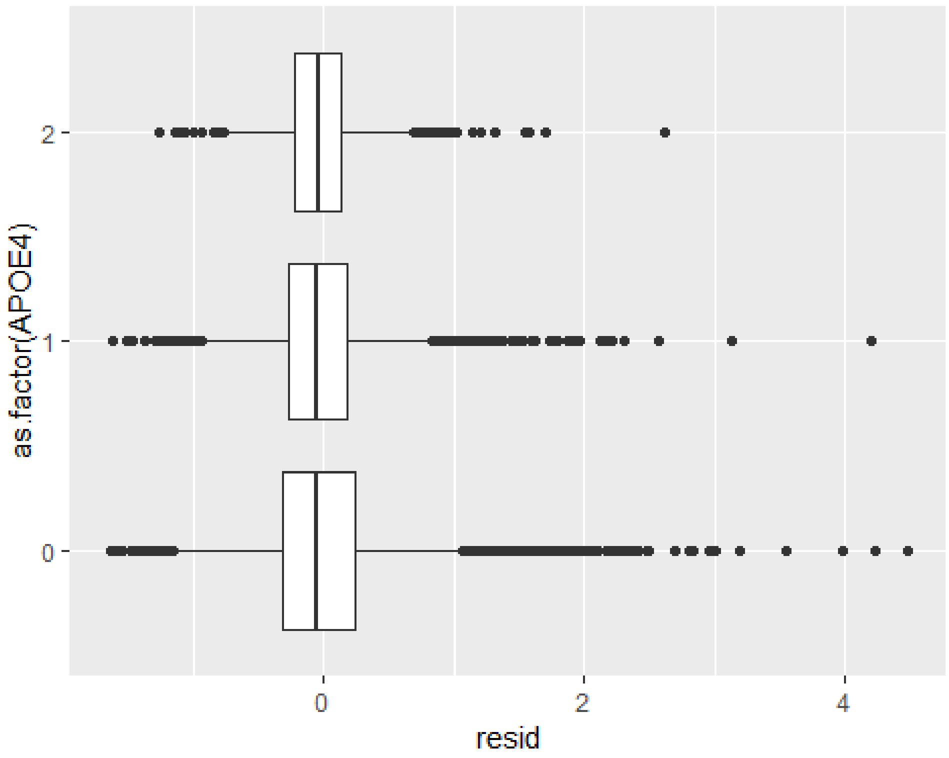


```
ggplot(augDat,aes(x=as.factor(M),y=resid))+geom_boxplot()+coord_flip()
```

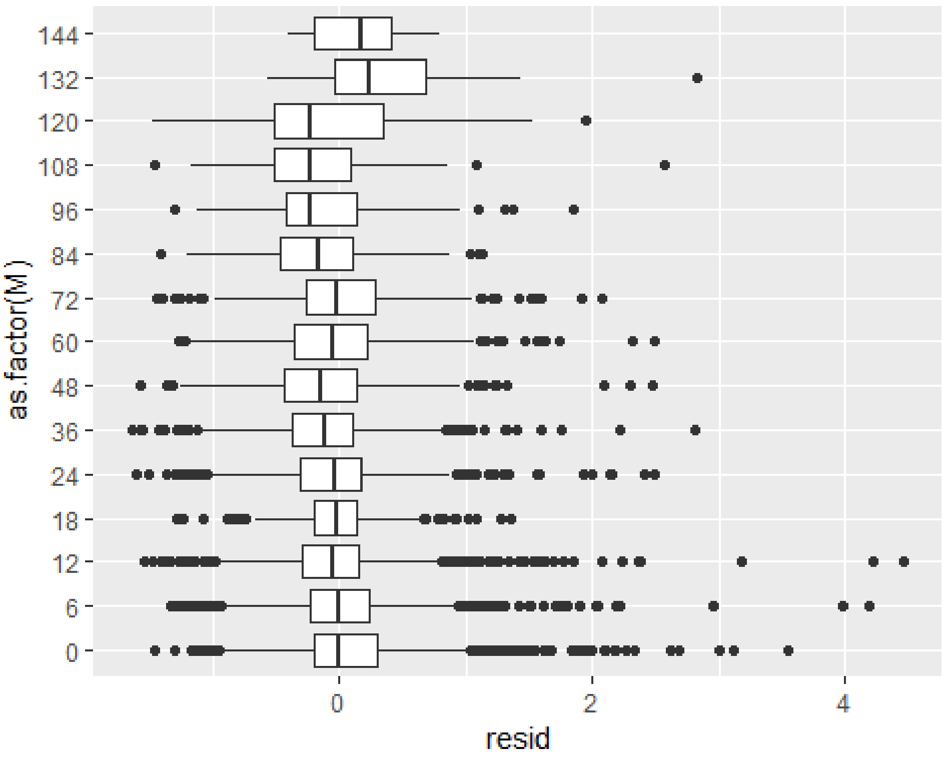


```
ggplot(augDat,aes(x=as.factor(diclo),y=resid))+geom_boxplot()+coord_flip()
```

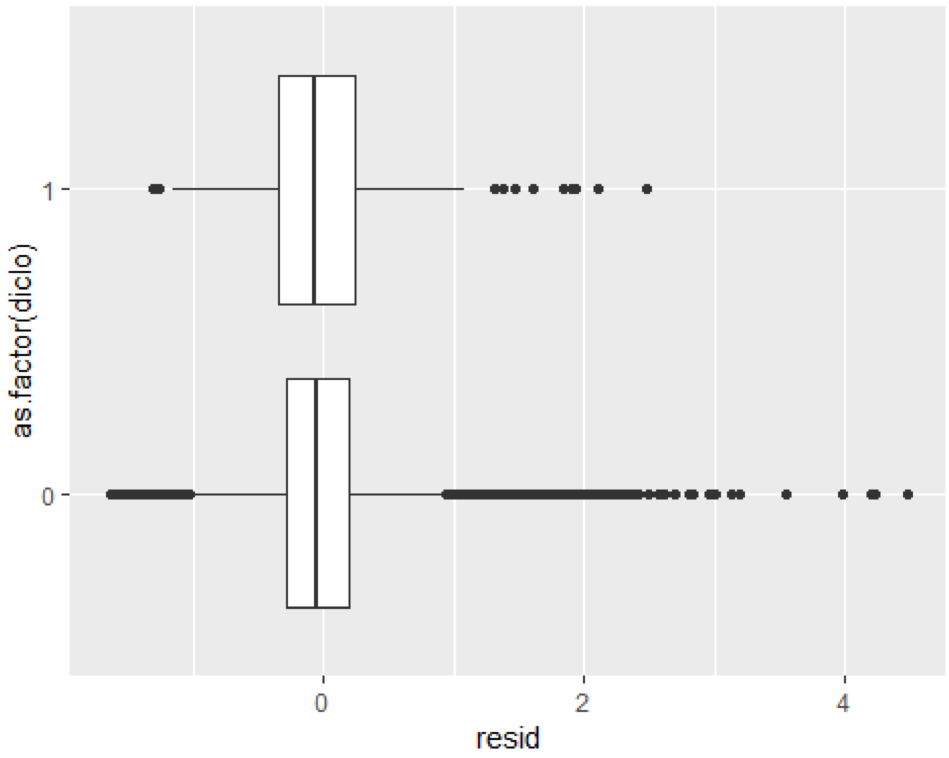


```
ggplot(augDat,aes(x=as.factor(vasc),y=resid))+geom_boxplot()+coord_flip()
```

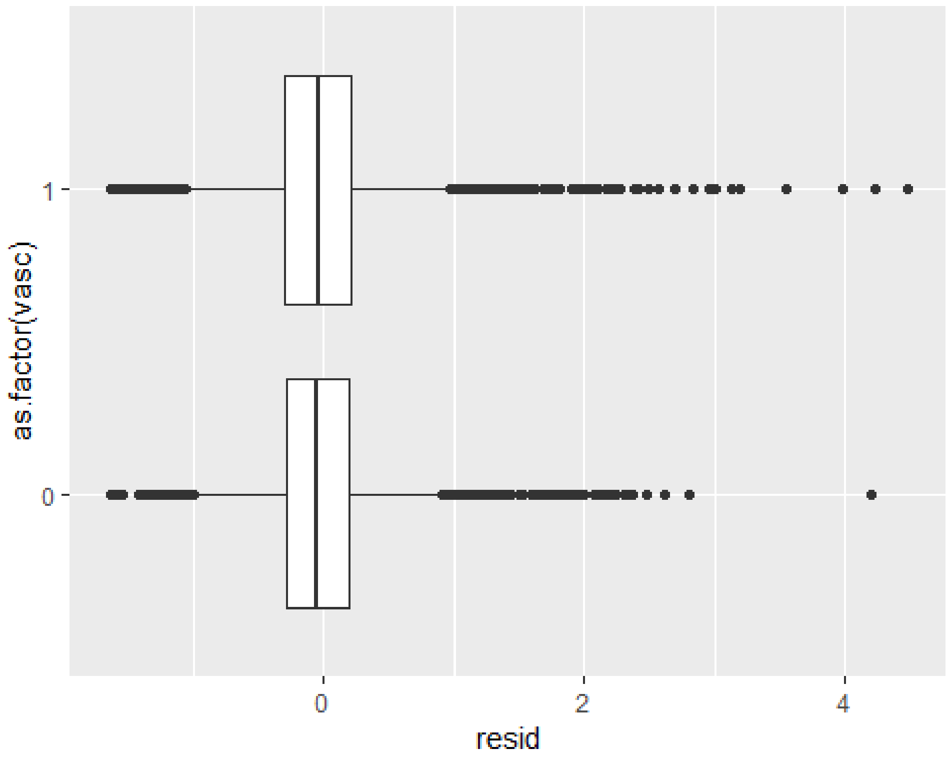


```
ggplot(augDat,aes(x=as.factor(Ibu),y=resid))+geom_boxplot()+coord_flip()
```

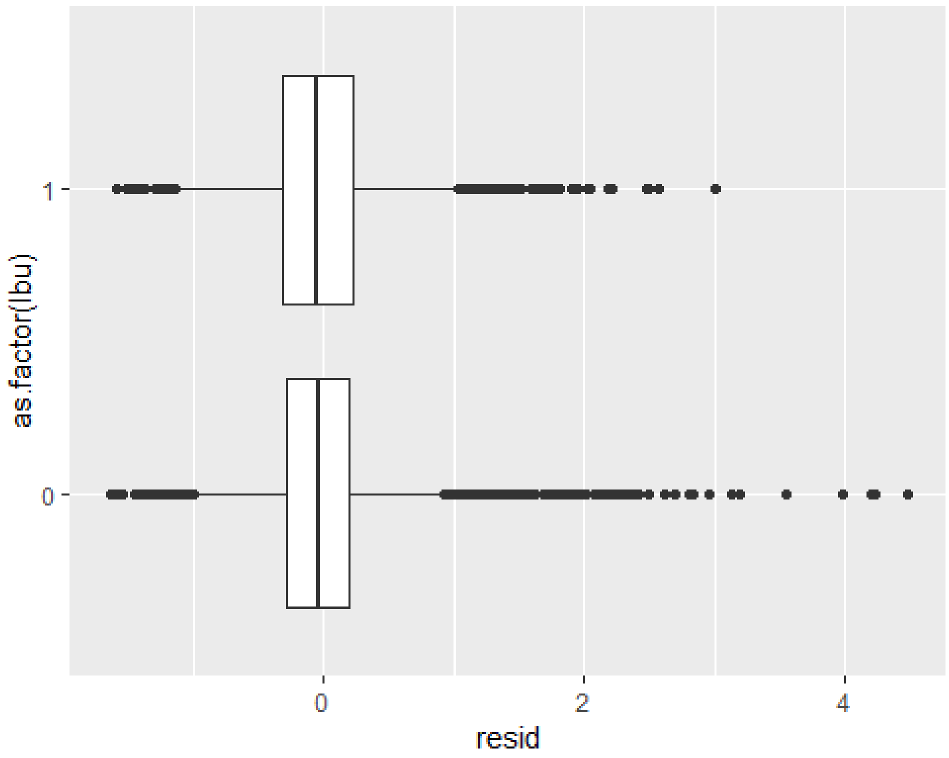


```
ggplot(augDat,aes(x=as.factor(aspirin),y=resid))+geom_boxplot()+coord_flip()
```

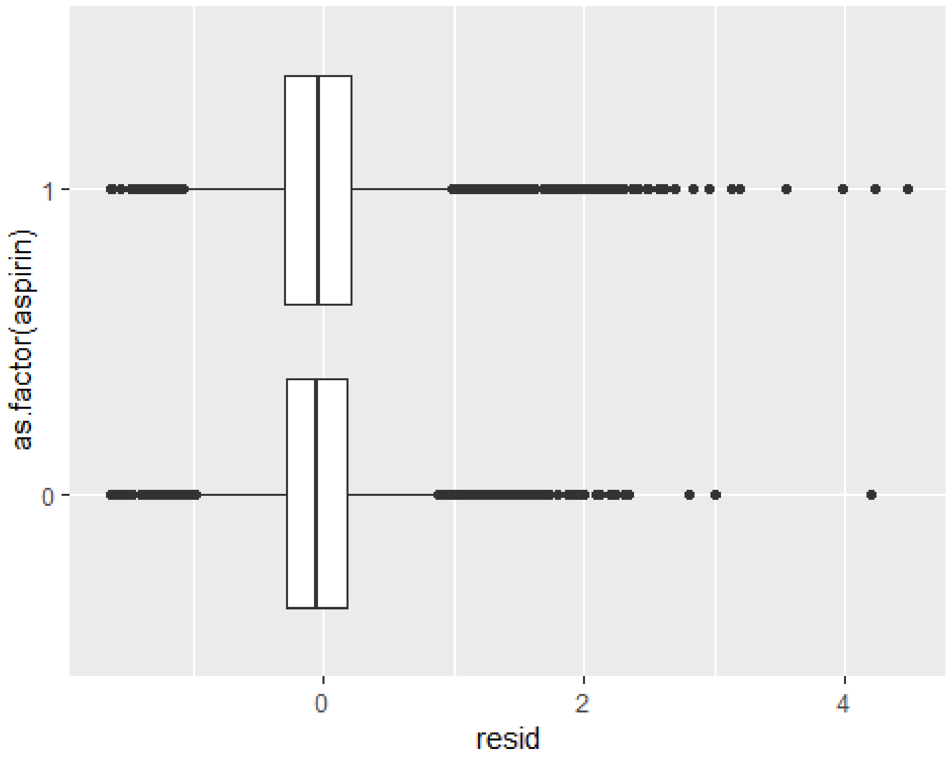


```
ggplot(augDat,aes(x=as.factor(diab),y=resid))+geom_boxplot()+coord_flip()
```

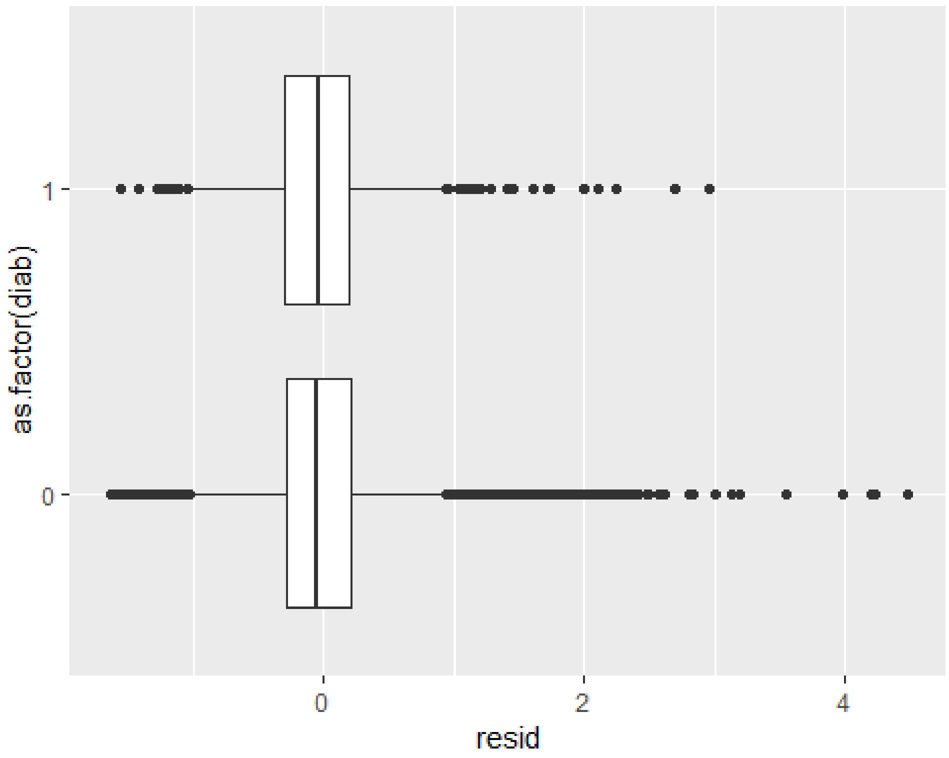


```
ggplot(augDat,aes(x=as.factor(naprox),y=resid))+geom_boxplot()+coord_flip()
```

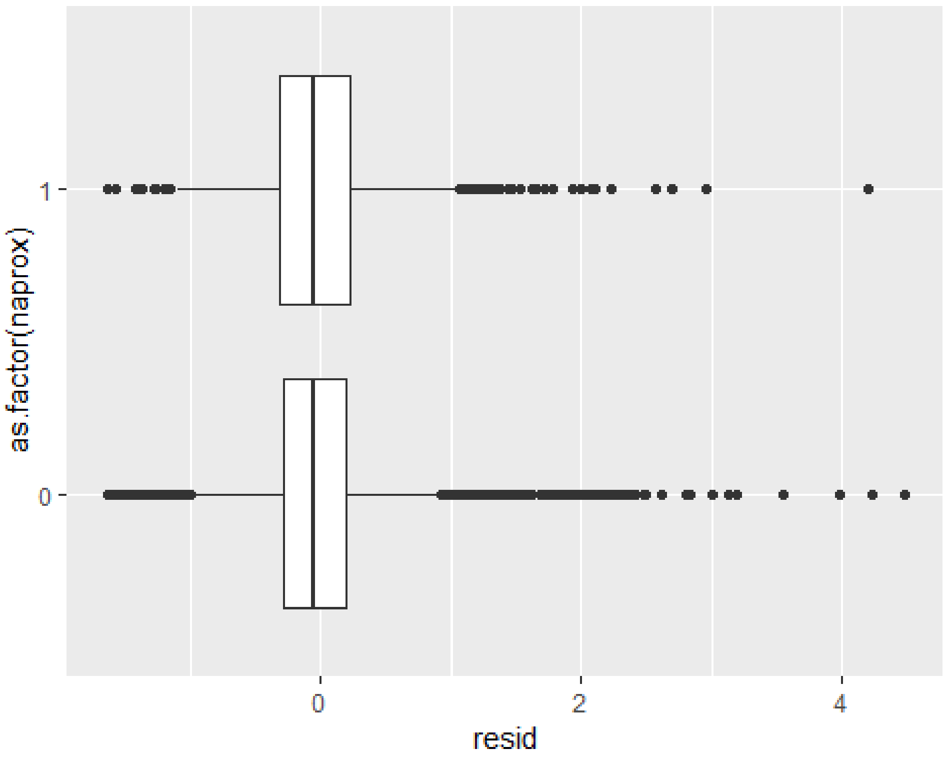


```
ggplot(augDat,aes(x=as.factor(celex),y=resid))+geom_boxplot()+coord_flip()
```

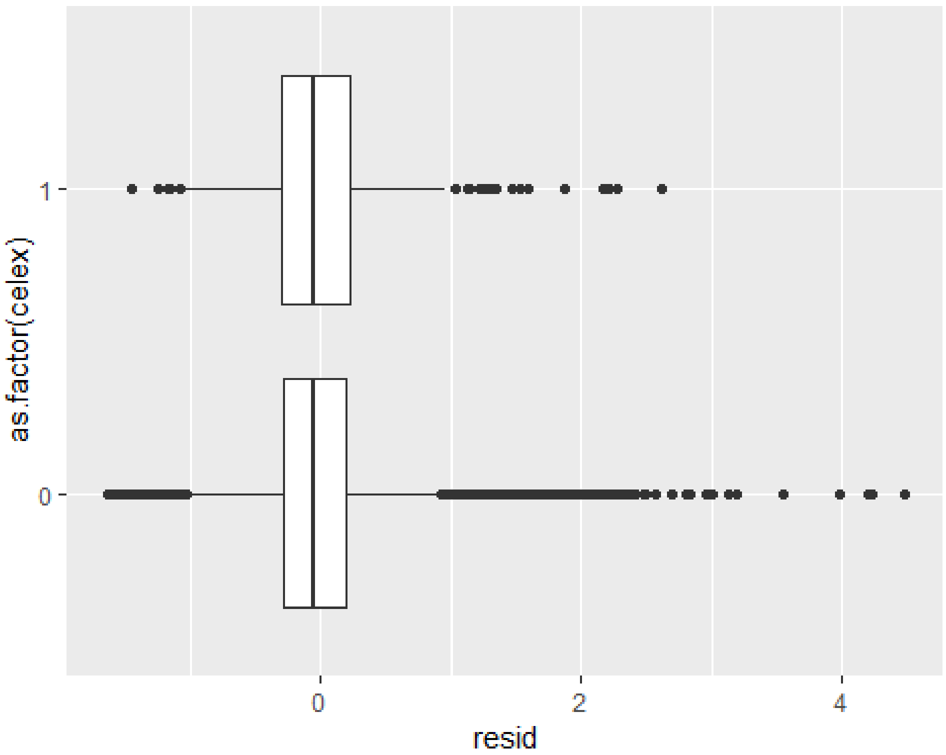

8.8. *Analysing all variables in isolation*

8.8.1. *Main effect of gender*

Adding the main effect of gender to the model did significantly improve the model based on the log-likelihood statistic.

```
main.A.M<-glmmadmb(neg.b.ADAS~M+ (1|ID), family="nbinom1", data=ADASdata)
main.A.Gender <- glmmadmb(neg.b.ADAS~ M + Gender+ (1|ID), family="nbinom1",
data=ADASdata)
summary(main.A.Gender)


Call:
glmmadmb(formula = neg.b.ADAS ~ M + Gender + (1 | ID), data = ADASdata,
    family = "nbinom1")

AIC: 73964.2

Coefficients:
            Estimate Std. Error z value Pr(>|z|)
(Intercept) 3.685405   0.025933   142.1  < 2e-16 ***
M           0.006653   0.000125    53.2  < 2e-16 ***
GenderMale  0.120365   0.034347     3.5  0.00046 ***
---
Signif. codes:  0 '***' 0.001 '**' 0.01 '*' 0.05 '.' 0.1 ' ' 1

Number of observations: total=8770, ID=1618
Random effect variance(s):
Group=ID
            Variance StdDev
(Intercept)   0.4516  0.672

Negative binomial dispersion parameter: 3.2926 (std. err.: 0.055721)

Log-likelihood: -36977.1

anova(main.A.M,main.A.Gender)

Analysis of Deviance Table

Model 1: neg.b.ADAS ~ M
Model 2: neg.b.ADAS ~ M + Gender
  NoPar LogLik Df Deviance  Pr(>Chi)
1     4 -36983
2     5 -36977  1     12.2 0.0004779 ***
---
Signif. codes:  0 '***' 0.001 '**' 0.01 '*' 0.05 '.' 0.1 ' ' 1
```

8.8.2. *Main effect of age*

Adding the main effect of age at the begining of the study (AGE) to the model did improve the model as measured by the log-likelihood statistic.

```
main.A.AGE <- glmmadmb(neg.b.ADAS~ M + AGE+ (1|ID), family="nbinom1",
data=ADASdata)
summary(main.A.AGE)
```

```
Call:
glmmadmb(formula = neg.b.ADAS ~ M + AGE + (1 | ID), data = ADASdata,
    family = "nbinom1")

AIC: 73924.6

Coefficients:
            Estimate Std. Error z value Pr(>|z|)
(Intercept) 3.751368   0.017080  219.64  < 2e-16 ***
M           0.006653   0.000125   53.22  < 2e-16 ***
AGE         0.016839   0.002326    7.24  4.5e-13 ***
---
Signif. codes:  0 '***' 0.001 '**' 0.01 '*' 0.05 '.' 0.1 ' ' 1

Number of observations: total=8770, ID=1618
Random effect variance(s):
Group=ID
            Variance StdDev
(Intercept)   0.4408 0.6639

Negative binomial dispersion parameter: 3.2922 (std. err.: 0.055705)

Log-likelihood: -36957.3

anova(main.A.M,main.A.AGE)

Analysis of Deviance Table

Model 1: neg.b.ADAS ~ M
Model 2: neg.b.ADAS ~ M + AGE
  NoPar LogLik Df Deviance  Pr(>Chi)
1     4 -36983
2     5 -36957  1     51.8 6.145e-13 ***
---
Signif. codes:  0 '***' 0.001 '**' 0.01 '*' 0.05 '.' 0.1 ' ' 1
```

8.8.3. Main effect of education level

Adding the main effect of education (edu.cat) to the model did improve the model as measured by the log-likelihood statistic.

```
main.A.edu.cat <- glmmadmb(neg.b.ADAS~ M + edu.cat+ (1|ID), family="nbinom1",
data=ADASdata)
summary(main.A.edu.cat)


Call:
glmmadmb(formula = neg.b.ADAS ~ M + edu.cat + (1 | ID), data = ADASdata,
    family = "nbinom1")

AIC: 73922.6

Coefficients:
                 Estimate Std. Error z value Pr(>|z|)
(Intercept)      3.619878   0.028653  126.34  < 2e-16 ***
M                0.006655   0.000125   53.24  < 2e-16 ***
edu.cat2tertiary 0.145944   0.042412    3.44  0.00058 ***
edu.cat3mid      0.138921   0.047354    2.93  0.00335 **
```

```
edu.cat4early    0.380343   0.049750    7.65  2.1e-14 ***
---
Signif. codes:  0 '***' 0.001 '**' 0.01 '*' 0.05 '.' 0.1 ' ' 1

Number of observations: total=8770, ID=1618
Random effect variance(s):
Group=ID
            Variance StdDev
(Intercept)   0.4384 0.6621

Negative binomial dispersion parameter: 3.2928 (std. err.: 0.055725)

Log-likelihood: -36954.3

anova(main.A.M,main.A.edu.cat)

Analysis of Deviance Table

Model 1: neg.b.ADAS ~ M
Model 2: neg.b.ADAS ~ M + edu.cat
  NoPar LogLik Df Deviance  Pr(>Chi)
1     4 -36983
2     7 -36954  3     57.8 1.734e-12 ***
---
Signif. codes:  0 '***' 0.001 '**' 0.01 '*' 0.05 '.' 0.1 ' ' 1
```

8.8.4. Main effect of diagnosis

Adding the main effect of initial diagnosis (diag) to the model did improve the model as measured by the log-likelihood statistic.

```
main.A.diagn <- glmmadmb(neg.b.ADAS~ M + diagn+ (1|ID), family="nbinom1",
data=ADASdata)
summary(main.A.diagn)


Call:
glmmadmb(formula = neg.b.ADAS ~ M + diagn + (1 | ID), data = ADASdata,
    family = "nbinom1")

AIC: 72687.4

Coefficients:
            Estimate Std. Error z value Pr(>|z|)
(Intercept) 3.112011   0.023147  134.45   <2e-16 ***
M           0.006711   0.000124   53.93   <2e-16 ***
diagn2EMCI  0.321955   0.034833    9.24   <2e-16 ***
diagn3LMCI  0.839691   0.029676   28.30   <2e-16 ***
diagn4AD    1.401136   0.033763   41.50   <2e-16 ***
---
Signif. codes:  0 '***' 0.001 '**' 0.01 '*' 0.05 '.' 0.1 ' ' 1

Number of observations: total=8770, ID=1618
Random effect variance(s):
Group=ID
            Variance StdDev
(Intercept)   0.1907 0.4367

Negative binomial dispersion parameter: 3.3057 (std. err.: 0.05611)
```

```
Log-likelihood: -36336.7

anova(main.A.M,main.A.diagn)

Analysis of Deviance Table

Model 1: neg.b.ADAS ~ M
Model 2: neg.b.ADAS ~ M + diagn
  NoPar LogLik Df Deviance  Pr(>Chi)
1     4 -36983
2     7 -36337  3     1293 < 2.2e-16 ***
---
Signif. codes:  0 '***' 0.001 '**' 0.01 '*' 0.05 '.' 0.1 ' ' 1
```

8.8.5. Main effect of APOE status

```
main.A.APOE4<-glmmadmb(neg.b.ADAS~M+APOE4+ (1|ID), family="nbinom1",
data=ADASdata)
main.A.Gender <- glmmadmb(neg.b.ADAS~ M + Gender+ (1|ID), family="nbinom1",
data=ADASdata)
summary(main.A.APOE4)


Call:
glmmadmb(formula = neg.b.ADAS ~ M + APOE4 + (1 | ID), data = ADASdata,
    family = "nbinom1")

AIC: 73794.2

Coefficients:
            Estimate Std. Error z value Pr(>|z|)
(Intercept) 3.545538   0.022640   156.6   <2e-16 ***
M           0.006660   0.000125    53.3   <2e-16 ***
APOE41      0.385965   0.034578    11.2   <2e-16 ***
APOE42      0.601403   0.054310    11.1   <2e-16 ***
---
Signif. codes:  0 '***' 0.001 '**' 0.01 '*' 0.05 '.' 0.1 ' ' 1

Number of observations: total=8770, ID=1618
Random effect variance(s):
Group=ID
            Variance StdDev
(Intercept)   0.4031 0.6349

Negative binomial dispersion parameter: 3.2948 (std. err.: 0.055794)

Log-likelihood: -36891.1

anova(main.A.M,main.A.APOE4)

Analysis of Deviance Table

Model 1: neg.b.ADAS ~ M
Model 2: neg.b.ADAS ~ M + APOE4
  NoPar LogLik Df Deviance  Pr(>Chi)
1     4 -36983
2     6 -36891  2    184.2 < 2.2e-16 ***
---
Signif. codes:  0 '***' 0.001 '**' 0.01 '*' 0.05 '.' 0.1 ' ' 1
```

8.8.6. Main effect of cardiovascular pathology

Adding the main effect of cardiovascular co-morbidity (vasc) to the model did significantly improve the model as measured by the log-likelihood statistic.

```
main.A.vasc <- glmmadmb(neg.b.ADAS~ M + vasc+ (1|ID), family="nbinom1",
data=ADASdata)
summary(main.A.vasc)


Call:
glmmadmb(formula = neg.b.ADAS ~ M + vasc + (1 | ID), data = ADASdata,
    family = "nbinom1")

AIC: 73976.4

Coefficients:
            Estimate Std. Error z value Pr(>|z|)
(Intercept) 3.751769   0.027713  135.38   <2e-16 ***
M           0.006656   0.000125   53.23   <2e-16 ***
vasc        0.002068   0.035144    0.06     0.95
---
Signif. codes:  0 '***' 0.001 '**' 0.01 '*' 0.05 '.' 0.1 ' ' 1

Number of observations: total=8770, ID=1618
Random effect variance(s):
Group=ID
            Variance StdDev
(Intercept)   0.4548 0.6744

Negative binomial dispersion parameter: 3.2929 (std. err.: 0.055733)

Log-likelihood: -36983.2

anova(main.A.M,main.A.vasc)

Analysis of Deviance Table

Model 1: neg.b.ADAS ~ M
Model 2: neg.b.ADAS ~ M + vasc
  NoPar LogLik Df Deviance Pr(>Chi)
1     4 -36983
2     5 -36983  1        0        1
```

8.8.7. Main effect of diabetes

Adding the main effect of diabetes co-morbidity (diab) to the model did not improve the model as measured by the log-likelihood statistic.

```
main.A.diab <- glmmadmb(neg.b.ADAS~ M + diab+ (1|ID), family="nbinom1",
data=ADASdata)
summary(main.A.diab)


Call:
glmmadmb(formula = neg.b.ADAS ~ M + diab + (1 | ID), data = ADASdata,
    family = "nbinom1")

AIC: 73976.4
```

```
Coefficients:
             Estimate Std. Error z value Pr(>|z|)
(Intercept)  3.754193   0.018169  206.63   <2e-16 ***
M            0.006656   0.000125   53.23   <2e-16 ***
diab        -0.012424   0.058942   -0.21     0.83
---
Signif. codes:  0 '***' 0.001 '**' 0.01 '*' 0.05 '.' 0.1 ' ' 1

Number of observations: total=8770, ID=1618
Random effect variance(s):
Group=ID
            Variance StdDev
(Intercept)   0.4548 0.6744

Negative binomial dispersion parameter: 3.2929 (std. err.: 0.055733)

Log-likelihood: -36983.2

anova(main.A.M,main.A.diab)

Analysis of Deviance Table

Model 1: neg.b.ADAS ~ M
Model 2: neg.b.ADAS ~ M + diab
  NoPar LogLik Df Deviance Pr(>Chi)
1     4 -36983
2     5 -36983  1        0        1
```

8.8.8. *Main effect of smoking*

Adding the main effect of smoking at the begining of the study (AGE) to the model did improve the model as measured by the log-likelihood statistic.

```
main.A.smoke <- glmmadmb(neg.b.ADAS~ M + smoke+ (1|ID), family="nbinom1",
data=ADASdata)
summary(main.A.smoke)


Call:
glmmadmb(formula = neg.b.ADAS ~ M + smoke + (1 | ID), data = ADASdata,
    family = "nbinom1")

AIC: 73976

Coefficients:
            Estimate Std. Error z value Pr(>|z|)
(Intercept) 3.746344   0.019997  187.35   <2e-16 ***
M           0.006656   0.000125   53.23   <2e-16 ***
smoke       0.026343   0.039232    0.67      0.5
---
Signif. codes:  0 '***' 0.001 '**' 0.01 '*' 0.05 '.' 0.1 ' ' 1

Number of observations: total=8770, ID=1618
Random effect variance(s):
Group=ID
            Variance StdDev
(Intercept)   0.4547 0.6743
```

```
Negative binomial dispersion parameter: 3.2929 (std. err.: 0.055733)

Log-likelihood: -36983

anova(main.A.M,main.A.smoke)

Analysis of Deviance Table

Model 1: neg.b.ADAS ~ M
Model 2: neg.b.ADAS ~ M + smoke
  NoPar LogLik Df Deviance Pr(>Chi)
1     4 -36983
2     5 -36983  1      0.4   0.5271
```

8.8.9. *Main effect of headache*

Adding the main effect of headache at the begining of the study (AGE) to the model did improve the model as measured by the log-likelihood statistic.

```
main.A.headache <- glmmadmb(neg.b.ADAS~ M + headache+ (1|ID), family="nbinom1",
data=ADASdata)
summary(main.A.headache)


Call:
glmmadmb(formula = neg.b.ADAS ~ M + headache + (1 | ID), data = ADASdata,
    family = "nbinom1")

AIC: 73966

Coefficients:
             Estimate Std. Error z value Pr(>|z|)
(Intercept)  3.769872   0.018030  209.09   <2e-16 ***
M            0.006660   0.000125   53.27   <2e-16 ***
headache    -0.197430   0.061015   -3.24   0.0012 **
---
Signif. codes:  0 '***' 0.001 '**' 0.01 '*' 0.05 '.' 0.1 ' ' 1

Number of observations: total=8770, ID=1618
Random effect variance(s):
Group=ID
            Variance StdDev
(Intercept)   0.4518 0.6722

Negative binomial dispersion parameter: 3.2927 (std. err.: 0.055726)

Log-likelihood: -36978

anova(main.A.M,main.A.headache)

Analysis of Deviance Table

Model 1: neg.b.ADAS ~ M
Model 2: neg.b.ADAS ~ M + headache
  NoPar LogLik Df Deviance Pr(>Chi)
1     4 -36983
2     5 -36978  1     10.4  0.00126 **
---
Signif. codes:  0 '***' 0.001 '**' 0.01 '*' 0.05 '.' 0.1 ' ' 1
```

8.8.10. Main effect of arthritis

Adding the main effect of headache at the begining of the study (AGE) to the model did improve the model as measured by the log-likelihood statistic.

```
main.A.arthrit <- glmmadmb(neg.b.ADAS~ M + arthrit+ (1|ID), family="nbinom1",
data=ADASdata)
summary(main.A.arthrit)


Call:
glmmadmb(formula = neg.b.ADAS ~ M + arthrit + (1 | ID), data = ADASdata,
    family = "nbinom1")

AIC: 73967.8

Coefficients:
             Estimate Std. Error z value Pr(>|z|)
(Intercept)  3.792670   0.021942  172.85   <2e-16 ***
M            0.006659   0.000125   53.26   <2e-16 ***
arthrit     -0.102313   0.034997   -2.92   0.0035 **
---
Signif. codes:  0 '***' 0.001 '**' 0.01 '*' 0.05 '.' 0.1 ' ' 1

Number of observations: total=8770, ID=1618
Random effect variance(s):
Group=ID
            Variance StdDev
(Intercept)   0.4522 0.6725

Negative binomial dispersion parameter: 3.2929 (std. err.: 0.055733)

Log-likelihood: -36978.9

anova(main.A.M,main.A.arthrit)

Analysis of Deviance Table

Model 1: neg.b.ADAS ~ M
Model 2: neg.b.ADAS ~ M + arthrit
  NoPar LogLik Df Deviance Pr(>Chi)
1     4 -36983
2     5 -36979  1      8.6 0.003362 **
---
Signif. codes:  0 '***' 0.001 '**' 0.01 '*' 0.05 '.' 0.1 ' ' 1
```

8.8.11. Main effect of diclofenac

Adding the main effect of diclofenac to the model did not improve the model as measured by the log-likelihood statistic.

```
main.A.diclo <- glmmadmb(neg.b.ADAS~M+diclo+ (1|ID), family="nbinom1",
data=ADASdata)
summary(main.A.diclo)


Call:
glmmadmb(formula = neg.b.ADAS ~ M + diclo + (1 | ID), data = ADASdata,
    family = "nbinom1")
```

```
AIC: 73963.4

Coefficients:
             Estimate Std. Error z value Pr(>|z|)
(Intercept)  3.761482   0.017415   216.0  < 2e-16 ***
M            0.006659   0.000125    53.3  < 2e-16 ***
diclo       -0.455421   0.126530    -3.6  0.00032 ***
---
Signif. codes:  0 '***' 0.001 '**' 0.01 '*' 0.05 '.' 0.1 ' ' 1

Number of observations: total=8770, ID=1618
Random effect variance(s):
Group=ID
            Variance StdDev
(Intercept)   0.4511 0.6716

Negative binomial dispersion parameter: 3.2927 (std. err.: 0.055725)

Log-likelihood: -36976.7

anova(main.A.M,main.A.diclo)

Analysis of Deviance Table

Model 1: neg.b.ADAS ~ M
Model 2: neg.b.ADAS ~ M + diclo
  NoPar LogLik Df Deviance  Pr(>Chi)
1     4 -36983
2     5 -36977  1       13 0.0003115 ***
---
Signif. codes:  0 '***' 0.001 '**' 0.01 '*' 0.05 '.' 0.1 ' ' 1
```

8.8.12. Main effect of paracetamol

Adding the main effect of paracetamol to the model did improve the model as measured by the log-likelihood statistic.

```
main.A.parac <- glmmadmb(neg.b.ADAS~ M + parac+ (1|ID), family="nbinom1",
data=ADASdata)
summary(main.A.parac)


Call:
glmmadmb(formula = neg.b.ADAS ~ M + parac + (1 | ID), data = ADASdata,
    family = "nbinom1")

AIC: 73967.8

Coefficients:
             Estimate Std. Error z value Pr(>|z|)
(Intercept)  3.781592   0.019841  190.59   <2e-16 ***
M            0.006663   0.000125   53.28   <2e-16 ***
parac       -0.115191   0.039454   -2.92   0.0035 **
---
Signif. codes:  0 '***' 0.001 '**' 0.01 '*' 0.05 '.' 0.1 ' ' 1

Number of observations: total=8770, ID=1618
Random effect variance(s):
```

```
Group=ID
            Variance StdDev
(Intercept)   0.4523 0.6725

Negative binomial dispersion parameter: 3.2927 (std. err.: 0.055726)

Log-likelihood: -36978.9

anova(main.A.M,main.A.parac)

Analysis of Deviance Table

Model 1: neg.b.ADAS ~ M
Model 2: neg.b.ADAS ~ M + parac
  NoPar LogLik Df Deviance Pr(>Chi)
1     4 -36983
2     5 -36979  1      8.6 0.003362 **
---
Signif. codes:  0 '***' 0.001 '**' 0.01 '*' 0.05 '.' 0.1 ' ' 1
```

8.8.13. Main effect celecoxib

Adding the main effect of celecoxib to the model did not improve the model as measured by the log-likelihood statistic.

```
main.A.celex <- glmmadmb(neg.b.ADAS~ M + celex+ (1|ID), family="nbinom1",
data=ADASdata)
summary(main.A.celex)


Call:
glmmadmb(formula = neg.b.ADAS ~ M + celex + (1 | ID), data = ADASdata,
    family = "nbinom1")

AIC: 73975.2

Coefficients:
             Estimate Std. Error z value Pr(>|z|)
(Intercept)  3.756720   0.017664  212.68   <2e-16 ***
M            0.006657   0.000125   53.24   <2e-16 ***
celex       -0.092952   0.087559   -1.06     0.29
---
Signif. codes:  0 '***' 0.001 '**' 0.01 '*' 0.05 '.' 0.1 ' ' 1

Number of observations: total=8770, ID=1618
Random effect variance(s):
Group=ID
            Variance StdDev
(Intercept)   0.4545 0.6741

Negative binomial dispersion parameter: 3.2929 (std. err.: 0.055733)

Log-likelihood: -36982.6

anova(main.A.M,main.A.celex)

Analysis of Deviance Table

Model 1: neg.b.ADAS ~ M
```

```
Model 2: neg.b.ADAS ~ M + celex
  NoPar LogLik Df Deviance Pr(>Chi)
1     4 -36983
2     5 -36983  1      1.2   0.2733
```

8.8.14. Main effect of naproxen

Adding the main effect of naproxen to the model did significantly improve the model as measured by the log-likelihood statistic.

```
main.A.naprox <- glmmadmb(neg.b.ADAS~ M + naprox+ (1|ID), family="nbinom1",
data=ADASdata)
summary(main.A.naprox)


Call:
glmmadmb(formula = neg.b.ADAS ~ M + naprox + (1 | ID), data = ADASdata,
    family = "nbinom1")

AIC: 73967.8

Coefficients:
             Estimate Std. Error z value Pr(>|z|)
(Intercept)  3.771189   0.018333  205.70   <2e-16 ***
M            0.006660   0.000125   53.26   <2e-16 ***
naprox      -0.157431   0.053357   -2.95   0.0032 **
---
Signif. codes:  0 '***' 0.001 '**' 0.01 '*' 0.05 '.' 0.1 ' ' 1

Number of observations: total=8770, ID=1618
Random effect variance(s):
Group=ID
            Variance StdDev
(Intercept)   0.4522 0.6725

Negative binomial dispersion parameter: 3.2928 (std. err.: 0.055729)

Log-likelihood: -36978.9

anova(main.A.M,main.A.naprox)

Analysis of Deviance Table

Model 1: neg.b.ADAS ~ M
Model 2: neg.b.ADAS ~ M + naprox
  NoPar LogLik Df Deviance Pr(>Chi)
1     4 -36983
2     5 -36979  1      8.6 0.003362 **
---
Signif. codes:  0 '***' 0.001 '**' 0.01 '*' 0.05 '.' 0.1 ' ' 1
```

8.8.15. Main effect of aspirin

Adding the main effect of aspirin use (aspirin) to the model did improve the model as measured by the log-likelihood statistic.

```
main.A.aspirin <- glmmadmb(neg.b.ADAS~ M + aspirin+ (1|ID), family="nbinom1", 
data=ADASdata)
summary(main.A.aspirin)


Call:
glmmadmb(formula = neg.b.ADAS ~ M + aspirin + (1 | ID), data = ADASdata, 
    family = "nbinom1")

AIC: 73962.2 

Coefficients:
             Estimate Std. Error z value Pr(>|z|)    
(Intercept)  3.821872   0.025024  152.73  < 2e-16 ***
M            0.006666   0.000125   53.30  < 2e-16 ***
aspirin     -0.129156   0.034112   -3.79  0.00015 ***
---
Signif. codes:  0 '***' 0.001 '**' 0.01 '*' 0.05 '.' 0.1 ' ' 1

Number of observations: total=8770, ID=1618 
Random effect variance(s):
Group=ID
            Variance StdDev
(Intercept)   0.4502  0.671

Negative binomial dispersion parameter: 3.2933 (std. err.: 0.055742)

Log-likelihood: -36976.1 

anova(main.A.M,main.A.aspirin)

Analysis of Deviance Table

Model 1: neg.b.ADAS ~ M
Model 2: neg.b.ADAS ~ M + aspirin
  NoPar LogLik Df Deviance  Pr(>Chi)    
1     4 -36983                          
2     5 -36976  1     14.2 0.0001644 ***
---
Signif. codes:  0 '***' 0.001 '**' 0.01 '*' 0.05 '.' 0.1 ' ' 1
```

8.8.16. Main effect of ibuprofen

Adding the main effect of ibuprofen use (Ibu) to the model did improve the model as measured by the log-likelihood statistic.

```
main.A.Ibu <- glmmadmb(neg.b.ADAS~ M + Ibu+ (1|ID), family="nbinom1", 
data=ADASdata)
summary(main.A.Ibu)


Call:
glmmadmb(formula = neg.b.ADAS ~ M + Ibu + (1 | ID), data = ADASdata, 
    family = "nbinom1")

AIC: 73946.2 

Coefficients:
             Estimate Std. Error z value Pr(>|z|)    
```

```
(Intercept)  3.793494   0.018651  203.39  < 2e-16 ***
M            0.006663   0.000125   53.30  < 2e-16 ***
Ibu         -0.256593   0.046522   -5.52  3.5e-08 ***
---
Signif. codes:  0 '***' 0.001 '**' 0.01 '*' 0.05 '.' 0.1 ' ' 1

Number of observations: total=8770, ID=1618
Random effect variance(s):
Group=ID
            Variance StdDev
(Intercept)   0.4464 0.6681

Negative binomial dispersion parameter: 3.2923 (std. err.: 0.055716)

Log-likelihood: -36968.1

anova(main.A.M,main.A.Ibu)

Analysis of Deviance Table

Model 1: neg.b.ADAS ~ M
Model 2: neg.b.ADAS ~ M + Ibu
  NoPar LogLik Df Deviance  Pr(>Chi)
1     4 -36983
2     5 -36968  1     30.2 3.897e-08 ***
---
Signif. codes:  0 '***' 0.001 '**' 0.01 '*' 0.05 '.' 0.1 ' ' 1
```

8.9. *Building combined main effect model*

```
main.A.combined <- glmmadmb(neg.b.ADAS~M+AGE+ APOE4+Gender+  edu.cat + diagn+
headache+arthrit+ diclo+parac+naprox+aspirin+Ibu+ (1|ID), family="nbinom1",
data=ADASdata)
summary(main.A.combined)


Call:
glmmadmb(formula = neg.b.ADAS ~ M + AGE + APOE4 + Gender + edu.cat +
    diagn + headache + arthrit + diclo + parac + naprox + aspirin +
    Ibu + (1 | ID), data = ADASdata, family = "nbinom1")

AIC: 72513.2

Coefficients:
                  Estimate Std. Error z value Pr(>|z|)
(Intercept)       3.017811   0.034249   88.11  < 2e-16 ***
M                 0.006727   0.000124   54.10  < 2e-16 ***
AGE               0.013673   0.001569    8.71  < 2e-16 ***
APOE41            0.163086   0.023681    6.89  5.7e-12 ***
APOE42            0.241815   0.037636    6.43  1.3e-10 ***
GenderMale        0.063162   0.022660    2.79  0.00531 **
edu.cat2tertiary  0.058493   0.027209    2.15  0.03157 *
edu.cat3mid       0.118337   0.030791    3.84  0.00012 ***
edu.cat4early     0.152681   0.032597    4.68  2.8e-06 ***
diagn2EMCI        0.325307   0.033546    9.70  < 2e-16 ***
diagn3LMCI        0.777334   0.028977   26.83  < 2e-16 ***
diagn4AD          1.274572   0.033966   37.52  < 2e-16 ***
headache         -0.088027   0.038992   -2.26  0.02397 *
arthrit          -0.017290   0.022850   -0.76  0.44924
```

```
diclo           -0.220562   0.080433   -2.74  0.00610 **
parac           -0.013643   0.025693   -0.53  0.59542
naprox          -0.010708   0.034122   -0.31  0.75367
aspirin         -0.018929   0.021981   -0.86  0.38915
Ibu             -0.094950   0.029884   -3.18  0.00149 **
---
Signif. codes:  0 '***' 0.001 '**' 0.01 '*' 0.05 '.' 0.1 ' ' 1

Number of observations: total=8770, ID=1618
Random effect variance(s):
Group=ID
            Variance StdDev
(Intercept)   0.1672 0.4089

Negative binomial dispersion parameter: 3.3029 (std. err.: 0.055996)

Log-likelihood: -36235.6

anova(main.A.M,main.A.combined)

Analysis of Deviance Table

Model 1: neg.b.ADAS ~ M
Model 2: neg.b.ADAS ~ M + AGE + APOE4 + Gender + edu.cat + diagn + headache +
arthrit + diclo + parac + naprox + aspirin + Ibu
  NoPar LogLik Df Deviance  Pr(>Chi)
1     4 -36983
2    21 -36236 17   1495.2 < 2.2e-16 ***
---
Signif. codes:  0 '***' 0.001 '**' 0.01 '*' 0.05 '.' 0.1 ' ' 1
```

8.10. *Dropping non-significant terms*

8.10.1. Narpoxen

Dropping naproxen from the model did not signficantly worsen the model

```
main.A.combined.drop.naprox <- glmmadmb(neg.b.ADAS~M+AGE+ APOE4+Gender+  edu.cat
+ diagn+ headache+arthrit+ diclo+parac+aspirin+Ibu+ (1|ID), family="nbinom1",
data=ADASdata)
summary(main.A.combined.drop.naprox)


Call:
glmmadmb(formula = neg.b.ADAS ~ M + AGE + APOE4 + Gender + edu.cat +
    diagn + headache + arthrit + diclo + parac + aspirin + Ibu +
    (1 | ID), data = ADASdata, family = "nbinom1")

AIC: 72511.2

Coefficients:
                  Estimate Std. Error z value Pr(>|z|)
(Intercept)       3.017192   0.034193   88.24  < 2e-16 ***
M                 0.006727   0.000124   54.10  < 2e-16 ***
AGE               0.013697   0.001567    8.74  < 2e-16 ***
APOE41            0.163108   0.023681    6.89  5.7e-12 ***
APOE42            0.241901   0.037635    6.43  1.3e-10 ***
GenderMale        0.063369   0.022651    2.80  0.00515 **
```

```
edu.cat2tertiary  0.058214   0.027195    2.14  0.03230 *
edu.cat3mid       0.118172   0.030787    3.84  0.00012 ***
edu.cat4early     0.152481   0.032591    4.68  2.9e-06 ***
diagn2EMCI        0.325072   0.033538    9.69  < 2e-16 ***
diagn3LMCI        0.777288   0.028977   26.82  < 2e-16 ***
diagn4AD          1.275002   0.033939   37.57  < 2e-16 ***
headache         -0.088009   0.038993   -2.26  0.02401 *
arthrit          -0.017787   0.022796   -0.78  0.43524
diclo            -0.220646   0.080433   -2.74  0.00608 **
parac            -0.014713   0.025466   -0.58  0.56342
aspirin          -0.019106   0.021974   -0.87  0.38459
Ibu              -0.095481   0.029837   -3.20  0.00137 **
---
Signif. codes:  0 '***' 0.001 '**' 0.01 '*' 0.05 '.' 0.1 ' ' 1

Number of observations: total=8770, ID=1618
Random effect variance(s):
Group=ID
            Variance StdDev
(Intercept)   0.1672 0.4089

Negative binomial dispersion parameter: 3.3029 (std. err.: 0.055997)

Log-likelihood: -36235.6

anova(main.A.combined, main.A.combined.drop.naprox)

Analysis of Deviance Table

Model 1: neg.b.ADAS ~ M + AGE + APOE4 + Gender + edu.cat + diagn + headache +
arthrit + diclo + parac + aspirin + Ibu
Model 2: neg.b.ADAS ~ M + AGE + APOE4 + Gender + edu.cat + diagn + headache +
arthrit + diclo + parac + naprox + aspirin + Ibu
  NoPar LogLik Df Deviance Pr(>Chi)
1    20 -36236
2    21 -36236  1        0        1
```

8.10.2. Paracetamol

Dropping paracetamol from the model did not signficantly worsen the model

```
main.A.combined.drop.parac<- glmmadmb(neg.b.ADAS~M+AGE+ APOE4+Gender+  edu.cat +
diagn+ headache+arthrit+ diclo+aspirin+Ibu+ (1|ID), family="nbinom1",
data=ADASdata)
summary(main.A.combined.drop.parac)


Call:
glmmadmb(formula = neg.b.ADAS ~ M + AGE + APOE4 + Gender + edu.cat +
    diagn + headache + arthrit + diclo + aspirin + Ibu + (1 |
    ID), data = ADASdata, family = "nbinom1")

AIC: 72509.6

Coefficients:
                  Estimate Std. Error z value Pr(>|z|)
(Intercept)       3.014473   0.033873   88.99  < 2e-16 ***
M                 0.006725   0.000124   54.10  < 2e-16 ***
AGE               0.013664   0.001566    8.72  < 2e-16 ***
```

```
APOE41            0.163741   0.023659    6.92  4.5e-12 ***
APOE42            0.242298   0.037633    6.44  1.2e-10 ***
GenderMale        0.064281   0.022599    2.84  0.00445 **
edu.cat2tertiary  0.057853   0.027190    2.13  0.03336 *
edu.cat3mid       0.117554   0.030771    3.82  0.00013 ***
edu.cat4early     0.152769   0.032591    4.69  2.8e-06 ***
diagn2EMCI        0.325278   0.033540    9.70  < 2e-16 ***
diagn3LMCI        0.777241   0.028980   26.82  < 2e-16 ***
diagn4AD          1.275416   0.033935   37.58  < 2e-16 ***
headache         -0.089581   0.038902   -2.30  0.02129 *
arthrit          -0.019697   0.022557   -0.87  0.38256
diclo            -0.221612   0.080423   -2.76  0.00586 **
aspirin          -0.020072   0.021913   -0.92  0.35967
Ibu              -0.097059   0.029715   -3.27  0.00109 **
---
Signif. codes:  0 '***' 0.001 '**' 0.01 '*' 0.05 '.' 0.1 ' ' 1

Number of observations: total=8770, ID=1618
Random effect variance(s):
Group=ID
            Variance StdDev
(Intercept)   0.1672 0.4089

Negative binomial dispersion parameter: 3.303 (std. err.: 0.056)

Log-likelihood: -36235.8

anova(main.A.combined.drop.naprox, main.A.combined.drop.parac)

Analysis of Deviance Table

Model 1: neg.b.ADAS ~ M + AGE + APOE4 + Gender + edu.cat + diagn + headache +
arthrit + diclo + aspirin + Ibu
Model 2: neg.b.ADAS ~ M + AGE + APOE4 + Gender + edu.cat + diagn + headache +
arthrit + diclo + parac + aspirin + Ibu
  NoPar LogLik Df Deviance Pr(>Chi)
1    19 -36236
2    20 -36236  1      0.4   0.5271
```

8.10.3. Arthritis

Dropping arthritis from the model did not signficantly worsen the model

```
main.A.combined.drop.arthritis<- glmmadmb(neg.b.ADAS~M+AGE+ APOE4+Gender+
edu.cat + diagn+ headache+ diclo+aspirin+Ibu+ (1|ID), family="nbinom1",
data=ADASdata)
summary(main.A.combined.drop.arthritis)


Call:
glmmadmb(formula = neg.b.ADAS ~ M + AGE + APOE4 + Gender + edu.cat +
    diagn + headache + diclo + aspirin + Ibu + (1 | ID), data = ADASdata,
    family = "nbinom1")

AIC: 72508.2

Coefficients:
                  Estimate Std. Error z value Pr(>|z|)
(Intercept)       3.006958   0.032775   91.75  < 2e-16 ***
```

```
M                  0.006724   0.000124   54.10  < 2e-16 ***
AGE                0.013512   0.001557    8.68  < 2e-16 ***
APOE41             0.163040   0.023651    6.89  5.4e-12 ***
APOE42             0.242894   0.037637    6.45  1.1e-10 ***
GenderMale         0.065879   0.022531    2.92  0.00346 **
edu.cat2tertiary   0.056927   0.027177    2.09  0.03620 *
edu.cat3mid        0.116546   0.030758    3.79  0.00015 ***
edu.cat4early      0.151499   0.032567    4.65  3.3e-06 ***
diagn2EMCI         0.325251   0.033549    9.69  < 2e-16 ***
diagn3LMCI         0.778288   0.028963   26.87  < 2e-16 ***
diagn4AD           1.277357   0.033871   37.71  < 2e-16 ***
headache          -0.092242   0.038792   -2.38  0.01741 *
diclo             -0.228398   0.080070   -2.85  0.00434 **
aspirin           -0.020381   0.021916   -0.93  0.35240
Ibu               -0.099410   0.029601   -3.36  0.00078 ***
---
Signif. codes:  0 '***' 0.001 '**' 0.01 '*' 0.05 '.' 0.1 ' ' 1

Number of observations: total=8770, ID=1618
Random effect variance(s):
Group=ID
            Variance StdDev
(Intercept)   0.1673 0.4091

Negative binomial dispersion parameter: 3.303 (std. err.: 0.056)

Log-likelihood: -36236.1

anova(main.A.combined.drop.parac, main.A.combined.drop.arthritis)

Analysis of Deviance Table

Model 1: neg.b.ADAS ~ M + AGE + APOE4 + Gender + edu.cat + diagn + headache +
diclo + aspirin + Ibu
Model 2: neg.b.ADAS ~ M + AGE + APOE4 + Gender + edu.cat + diagn + headache +
arthrit + diclo + aspirin + Ibu
  NoPar LogLik Df Deviance Pr(>Chi)
1    18 -36236
2    19 -36236  1      0.6   0.4386
```

8.10.4. Aspirin

Dropping aspirin from the model did not signficantly worsen the model

```
main.A.combined.drop.aspirin<- glmmadmb(neg.b.ADAS~M+AGE+ APOE4+Gender+  edu.cat
+ diagn+ headache+ diclo+Ibu+ (1|ID), family="nbinom1", data=ADASdata)
summary(main.A.combined.drop.aspirin)


Call:
glmmadmb(formula = neg.b.ADAS ~ M + AGE + APOE4 + Gender + edu.cat +
    diagn + headache + diclo + Ibu + (1 | ID), data = ADASdata,
    family = "nbinom1")

AIC: 72507.2

Coefficients:
                  Estimate Std. Error z value Pr(>|z|)
(Intercept)       2.996170   0.030673   97.68  < 2e-16 ***
```

```
M                  0.006721   0.000124   54.10  < 2e-16 ***
AGE                0.013441   0.001556    8.64  < 2e-16 ***
APOE41             0.163353   0.023658    6.90  5.0e-12 ***
APOE42             0.242161   0.037644    6.43  1.3e-10 ***
GenderMale         0.063235   0.022359    2.83  0.00468 **
edu.cat2tertiary   0.057039   0.027187    2.10  0.03590 *
edu.cat3mid        0.116509   0.030770    3.79  0.00015 ***
edu.cat4early      0.152613   0.032558    4.69  2.8e-06 ***
diagn2EMCI         0.325540   0.033561    9.70  < 2e-16 ***
diagn3LMCI         0.779652   0.028938   26.94  < 2e-16 ***
diagn4AD           1.280977   0.033661   38.06  < 2e-16 ***
headache          -0.091763   0.038804   -2.36  0.01804 *
diclo             -0.232052   0.080003   -2.90  0.00373 **
Ibu               -0.099927   0.029608   -3.38  0.00074 ***
---
Signif. codes:  0 '***' 0.001 '**' 0.01 '*' 0.05 '.' 0.1 ' ' 1

Number of observations: total=8770, ID=1618
Random effect variance(s):
Group=ID
            Variance StdDev
(Intercept)   0.1675 0.4092

Negative binomial dispersion parameter: 3.3029 (std. err.: 0.056)

Log-likelihood: -36236.6

anova(main.A.combined.drop.arthritis, main.A.combined.drop.aspirin)

Analysis of Deviance Table

Model 1: neg.b.ADAS ~ M + AGE + APOE4 + Gender + edu.cat + diagn + headache +
diclo + Ibu
Model 2: neg.b.ADAS ~ M + AGE + APOE4 + Gender + edu.cat + diagn + headache +
diclo + aspirin + Ibu
  NoPar LogLik Df Deviance Pr(>Chi)
1    17 -36237
2    18 -36236  1        1   0.3173
```

8.11. *Building combined main effect model*

```
main.A.final<- glmmadmb(neg.b.ADAS~M+AGE+ APOE4+Gender+  edu.cat + diagn+
headache+ diclo+Ibu+ (1|ID), family="nbinom1", data=ADASdata)
summary(main.A.final)


Call:
glmmadmb(formula = neg.b.ADAS ~ M + AGE + APOE4 + Gender + edu.cat +
    diagn + headache + diclo + Ibu + (1 | ID), data = ADASdata,
    family = "nbinom1")

AIC: 72507.2

Coefficients:
                   Estimate Std. Error z value Pr(>|z|)
(Intercept)        2.996170   0.030673   97.68  < 2e-16 ***
M                  0.006721   0.000124   54.10  < 2e-16 ***
AGE                0.013441   0.001556    8.64  < 2e-16 ***
APOE41             0.163353   0.023658    6.90  5.0e-12 ***
```

```
APOE42             0.242161   0.037644    6.43  1.3e-10 ***
GenderMale         0.063235   0.022359    2.83  0.00468 **
edu.cat2tertiary   0.057039   0.027187    2.10  0.03590 *
edu.cat3mid        0.116509   0.030770    3.79  0.00015 ***
edu.cat4early      0.152613   0.032558    4.69  2.8e-06 ***
diagn2EMCI         0.325540   0.033561    9.70  < 2e-16 ***
diagn3LMCI         0.779652   0.028938   26.94  < 2e-16 ***
diagn4AD           1.280977   0.033661   38.06  < 2e-16 ***
headache          -0.091763   0.038804   -2.36  0.01804 *
diclo             -0.232052   0.080003   -2.90  0.00373 **
Ibu               -0.099927   0.029608   -3.38  0.00074 ***
---
Signif. codes:  0 '***' 0.001 '**' 0.01 '*' 0.05 '.' 0.1 ' ' 1

Number of observations: total=8770, ID=1618
Random effect variance(s):
Group=ID
            Variance StdDev
(Intercept)   0.1675 0.4092

Negative binomial dispersion parameter: 3.3029 (std. err.: 0.056)

Log-likelihood: -36236.6

anova(main.A.combined,main.A.final)

Analysis of Deviance Table

Model 1: neg.b.ADAS ~ M + AGE + APOE4 + Gender + edu.cat + diagn + headache + 
diclo + Ibu
Model 2: neg.b.ADAS ~ M + AGE + APOE4 + Gender + edu.cat + diagn + headache + 
arthrit + diclo + parac + naprox + aspirin + Ibu
  NoPar LogLik Df Deviance Pr(>Chi)
1    17 -36237
2    21 -36236  4        2   0.7358
```

8.12. *Removing each explanitory variable in isolation*

8.12.1. *Main effect of age at the start of the study*

```
main.A.final.drop.AGE<- glmmadmb(neg.b.ADAS~M+ APOE4+Gender+  edu.cat + diagn+
headache+ diclo+Ibu+ (1|ID), family="nbinom1", data=ADASdata)
anova(main.A.final,main.A.final.drop.AGE)

Analysis of Deviance Table

Model 1: neg.b.ADAS ~ M + APOE4 + Gender + edu.cat + diagn + headache + diclo + 
Ibu
Model 2: neg.b.ADAS ~ M + AGE + APOE4 + Gender + edu.cat + diagn + headache + 
diclo + Ibu
  NoPar LogLik Df Deviance  Pr(>Chi)
1    16 -36273
2    17 -36237  1     73.2 < 2.2e-16 ***
---
Signif. codes:  0 '***' 0.001 '**' 0.01 '*' 0.05 '.' 0.1 ' ' 1
```

8.12.2. Main effect of APOE4 genotype

```
main.A.final.drop.APOE4<- glmmadmb(neg.b.ADAS~M+AGE+ Gender+  edu.cat + diagn+
headache+ diclo+Ibu+ (1|ID), family="nbinom1", data=ADASdata)
anova(main.A.final.drop.APOE4,main.A.final)

Analysis of Deviance Table

Model 1: neg.b.ADAS ~ M + AGE + Gender + edu.cat + diagn + headache + diclo +
Ibu
Model 2: neg.b.ADAS ~ M + AGE + APOE4 + Gender + edu.cat + diagn + headache +
diclo + Ibu
  NoPar LogLik Df Deviance  Pr(>Chi)
1    15 -36270
2    17 -36237  2       66 4.663e-15 ***
---
Signif. codes:  0 '***' 0.001 '**' 0.01 '*' 0.05 '.' 0.1 ' ' 1
```

8.12.3. Main effect of education level

```
main.A.final.drop.edu.cat<- glmmadmb(neg.b.ADAS~M+AGE+ APOE4+Gender+  diagn+
headache+ diclo+Ibu+ (1|ID), family="nbinom1", data=ADASdata)
anova(main.A.final.drop.edu.cat,main.A.final)

Analysis of Deviance Table

Model 1: neg.b.ADAS ~ M + AGE + APOE4 + Gender + diagn + headache + diclo + Ibu
Model 2: neg.b.ADAS ~ M + AGE + APOE4 + Gender + edu.cat + diagn + headache +
diclo + Ibu
  NoPar LogLik Df Deviance  Pr(>Chi)
1    14 -36250
2    17 -36237  3       27 5.887e-06 ***
---
Signif. codes:  0 '***' 0.001 '**' 0.01 '*' 0.05 '.' 0.1 ' ' 1
```

8.12.4. Main effect of initial Alzhiemer's diagnosis

```
main.A.final.drop.diagn<- glmmadmb(neg.b.ADAS~M+AGE+ APOE4+Gender+  edu.cat +
headache+ diclo+Ibu+ (1|ID), family="nbinom1", data=ADASdata)
anova(main.A.final.drop.diagn,main.A.final)

Analysis of Deviance Table

Model 1: neg.b.ADAS ~ M + AGE + APOE4 + Gender + edu.cat + headache + diclo +
Ibu
Model 2: neg.b.ADAS ~ M + AGE + APOE4 + Gender + edu.cat + diagn + headache +
diclo + Ibu
  NoPar LogLik Df Deviance  Pr(>Chi)
1    14 -36799
2    17 -36237  3   1124.8 < 2.2e-16 ***
---
Signif. codes:  0 '***' 0.001 '**' 0.01 '*' 0.05 '.' 0.1 ' ' 1
```

8.12.5. Main effect of headaches

```
main.A.final.drop.headaches<- glmmadmb(neg.b.ADAS~M+AGE+ APOE4+Gender+  edu.cat
+ diagn+  diclo+Ibu+ (1|ID), family="nbinom1", data=ADASdata)
anova(main.A.final.drop.headaches,main.A.final)
```

```
Analysis of Deviance Table

Model 1: neg.b.ADAS ~ M + AGE + APOE4 + Gender + edu.cat + diagn + diclo + Ibu
Model 2: neg.b.ADAS ~ M + AGE + APOE4 + Gender + edu.cat + diagn + headache +
diclo + Ibu
  NoPar LogLik Df Deviance Pr(>Chi)
1    16 -36239
2    17 -36237  1      5.6  0.01796 *
---
Signif. codes:  0 '***' 0.001 '**' 0.01 '*' 0.05 '.' 0.1 ' ' 1
```

8.12.6. Main effect of Gender

```
main.A.final.drop.Gender<- glmmadmb(neg.b.ADAS~M+AGE+ APOE4+  edu.cat + diagn+
headache+ diclo+Ibu+ (1|ID), family="nbinom1", data=ADASdata)
anova(main.A.final.drop.Gender,main.A.final)

Analysis of Deviance Table

Model 1: neg.b.ADAS ~ M + AGE + APOE4 + edu.cat + diagn + headache + diclo + Ibu
Model 2: neg.b.ADAS ~ M + AGE + APOE4 + Gender + edu.cat + diagn + headache +
diclo + Ibu
  NoPar LogLik Df Deviance Pr(>Chi)
1    16 -36241
2    17 -36237  1        8 0.004678 **
---
Signif. codes:  0 '***' 0.001 '**' 0.01 '*' 0.05 '.' 0.1 ' ' 1
```

8.12.7. Main effect of Ibuprofen

```
main.A.final.drop.Ibuprofen<- glmmadmb(neg.b.ADAS~M+AGE+ APOE4+Gender+  edu.cat
+ diagn+ headache+ diclo+ (1|ID), family="nbinom1", data=ADASdata)
anova(main.A.final.drop.Ibuprofen,main.A.final)

Analysis of Deviance Table

Model 1: neg.b.ADAS ~ M + AGE + APOE4 + Gender + edu.cat + diagn + headache +
diclo
Model 2: neg.b.ADAS ~ M + AGE + APOE4 + Gender + edu.cat + diagn + headache +
diclo + Ibu
  NoPar LogLik Df Deviance  Pr(>Chi)
1    16 -36242
2    17 -36237  1     11.4 0.0007344 ***
---
Signif. codes:  0 '***' 0.001 '**' 0.01 '*' 0.05 '.' 0.1 ' ' 1
```

8.12.8. Main effect of Diclofenac

```
main.A.final.drop.Diclofenac<- glmmadmb(neg.b.ADAS~M+AGE+ APOE4+Gender+  edu.cat
+ diagn+ headache+ Ibu+ (1|ID), family="nbinom1", data=ADASdata)
anova(main.A.final.drop.Diclofenac,main.A.final)

Analysis of Deviance Table

Model 1: neg.b.ADAS ~ M + AGE + APOE4 + Gender + edu.cat + diagn + headache +
Ibu
Model 2: neg.b.ADAS ~ M + AGE + APOE4 + Gender + edu.cat + diagn + headache +
diclo + Ibu
  NoPar LogLik Df Deviance Pr(>Chi)
```

```
1    16 -36241
2    17 -36237  1      8.4 0.003752 **
---
Signif. codes:  0 '***' 0.001 '**' 0.01 '*' 0.05 '.' 0.1 ' ' 1
```

8.12.9. AIC summary of main effect models

```
AIC(main.A.final,main.A.final.drop.AGE,main.A.final.drop.APOE4,main.A.final.drop
.edu.cat,main.A.final.drop.diagn,main.A.final.drop.Diclofenac,main.A.final.drop.
Ibuprofen,main.A.final.drop.Gender,main.A.final.drop.headaches)

                             df     AIC
main.A.final                 17 72507.2
main.A.final.drop.AGE        16 72578.4
main.A.final.drop.APOE4      15 72569.2
main.A.final.drop.edu.cat    14 72528.2
main.A.final.drop.diagn      14 73626.0
main.A.final.drop.Diclofenac 16 72513.6
main.A.final.drop.Ibuprofen  16 72516.6
main.A.final.drop.Gender     16 72513.2
main.A.final.drop.headaches  16 72510.8
```

8.13. *Investigating interaction terms*

8.13.1. The effects of diagnosis on cognitive decline progression

```
neg.a.interaction.diagn<- glmmadmb(neg.b.ADAS~M+AGE+ APOE4+Gender+  edu.cat +
diagn+ headache+ diclo+Ibu+ diagn*M+(1|ID), family="nbinom1", data=ADASdata)
summary(neg.a.interaction.diagn)
anova(neg.a.interaction.diagn,main.A.final)
```

8.13.2. The effects of smoking on cognitive decline progression

```
neg.a.interaction.smoke<- glmmadmb(neg.b.ADAS~M+AGE+ APOE4+Gender+  edu.cat +
diagn+ headache+ diclo+Ibu+smoke*M+ (1|ID), family="nbinom1", data=ADASdata)
summary(neg.a.interaction.smoke)
anova(neg.a.interaction.smoke,main.A.final)
```

8.13.3. The effects of arthritis on cognitive decline progression

```
neg.a.interaction.arthrit<- glmmadmb(neg.b.ADAS~M+AGE+ APOE4+Gender+  edu.cat +
diagn+ headache+ diclo+Ibu+arthrit*M+ (1|ID), family="nbinom1", data=ADASdata)
summary(neg.a.interaction.arthrit)
anova(neg.a.interaction.arthrit, main.A.final)
```

8.13.4. The effects of cardiovascular disease on cognitive decline progression

```
neg.a.interaction.vasc<- glmmadmb(neg.b.ADAS~M+AGE+ APOE4+Gender+  edu.cat +
diagn+ headache+ diclo+Ibu+vasc*M+ (1|ID), family="nbinom1", data=ADASdata)
summary(neg.a.interaction.vasc)
anova(neg.a.interaction.vasc, main.A.final)
```

8.13.5. The effect of headaches on cognitive decline progression

```
neg.a.interaction.headache<- glmmadmb(neg.b.ADAS~M+AGE+ APOE4+Gender+  edu.cat +
diagn+ headache+ diclo+Ibu+headache*M+ (1|ID), family="nbinom1", data=ADASdata)
summary(neg.a.interaction.headache)
```

```
Call:
glmmadmb(formula = neg.b.ADAS ~ M + AGE + APOE4 + Gender + edu.cat +
    diagn + headache + diclo + Ibu + headache * M + (1 | ID),
    data = ADASdata, family = "nbinom1")

AIC: 72503.4

Coefficients:
                  Estimate Std. Error z value Pr(>|z|)
(Intercept)       2.993245   0.030702   97.49  < 2e-16 ***
M                 0.006837   0.000133   51.33  < 2e-16 ***
AGE               0.013457   0.001556    8.65  < 2e-16 ***
APOE41            0.163477   0.023662    6.91  4.9e-12 ***
APOE42            0.241777   0.037650    6.42  1.3e-10 ***
GenderMale        0.063623   0.022363    2.85  0.00444 **
edu.cat2tertiary  0.057155   0.027191    2.10  0.03556 *
edu.cat3mid       0.116559   0.030775    3.79  0.00015 ***
edu.cat4early     0.152558   0.032562    4.69  2.8e-06 ***
diagn2EMCI        0.325371   0.033565    9.69  < 2e-16 ***
diagn3LMCI        0.779868   0.028942   26.95  < 2e-16 ***
diagn4AD          1.281613   0.033667   38.07  < 2e-16 ***
headache         -0.067821   0.040039   -1.69  0.09029 .
diclo            -0.232237   0.080016   -2.90  0.00370 **
Ibu              -0.100235   0.029613   -3.38  0.00071 ***
M:headache       -0.000871   0.000363   -2.40  0.01628 *
---
Signif. codes:  0 '***' 0.001 '**' 0.01 '*' 0.05 '.' 0.1 ' ' 1

Number of observations: total=8770, ID=1618
Random effect variance(s):
Group=ID
            Variance StdDev
(Intercept)   0.1675 0.4093

Negative binomial dispersion parameter: 3.2997 (std. err.: 0.055949)

Log-likelihood: -36233.7

anova(neg.a.interaction.headache, main.A.final)

Analysis of Deviance Table

Model 1: neg.b.ADAS ~ M + AGE + APOE4 + Gender + edu.cat + diagn + headache +
diclo + Ibu
Model 2: neg.b.ADAS ~ M + AGE + APOE4 + Gender + edu.cat + diagn + headache +
diclo + Ibu + headache * M
  NoPar LogLik Df Deviance Pr(>Chi)
1    17 -36237
2    18 -36234  1      5.8  0.01603 *
---
Signif. codes:  0 '***' 0.001 '**' 0.01 '*' 0.05 '.' 0.1 ' ' 1
```

8.13.6. The effect of diabetes on cognitive decline progression

```
neg.a.interaction.diabetes<- glmmadmb(neg.b.ADAS~M+AGE+ APOE4+Gender+  edu.cat +
diagn+ headache+ diclo+Ibu+ diab*M+(1|ID), family="nbinom1", data=ADASdata)
summary(neg.a.interaction.headache)
```

```
Call:
glmmadmb(formula = neg.b.ADAS ~ M + AGE + APOE4 + Gender + edu.cat +
    diagn + headache + diclo + Ibu + headache * M + (1 | ID),
    data = ADASdata, family = "nbinom1")

AIC: 72503.4

Coefficients:
                  Estimate Std. Error z value Pr(>|z|)
(Intercept)       2.993245   0.030702   97.49  < 2e-16 ***
M                 0.006837   0.000133   51.33  < 2e-16 ***
AGE               0.013457   0.001556    8.65  < 2e-16 ***
APOE41            0.163477   0.023662    6.91  4.9e-12 ***
APOE42            0.241777   0.037650    6.42  1.3e-10 ***
GenderMale        0.063623   0.022363    2.85  0.00444 **
edu.cat2tertiary  0.057155   0.027191    2.10  0.03556 *
edu.cat3mid       0.116559   0.030775    3.79  0.00015 ***
edu.cat4early     0.152558   0.032562    4.69  2.8e-06 ***
diagn2EMCI        0.325371   0.033565    9.69  < 2e-16 ***
diagn3LMCI        0.779868   0.028942   26.95  < 2e-16 ***
diagn4AD          1.281613   0.033667   38.07  < 2e-16 ***
headache         -0.067821   0.040039   -1.69  0.09029 .
diclo            -0.232237   0.080016   -2.90  0.00370 **
Ibu              -0.100235   0.029613   -3.38  0.00071 ***
M:headache       -0.000871   0.000363   -2.40  0.01628 *
---
Signif. codes:  0 '***' 0.001 '**' 0.01 '*' 0.05 '.' 0.1 ' ' 1

Number of observations: total=8770, ID=1618
Random effect variance(s):
Group=ID
            Variance StdDev
(Intercept)   0.1675 0.4093

Negative binomial dispersion parameter: 3.2997 (std. err.: 0.055949)

Log-likelihood: -36233.7

anova(neg.a.interaction.diabetes, main.A.final)

Analysis of Deviance Table

Model 1: neg.b.ADAS ~ M + AGE + APOE4 + Gender + edu.cat + diagn + headache +
diclo + Ibu
Model 2: neg.b.ADAS ~ M + AGE + APOE4 + Gender + edu.cat + diagn + headache +
diclo + Ibu + diab * M
  NoPar LogLik Df Deviance Pr(>Chi)
1    17 -36237
2    19 -36236  2      1.8   0.4066
```

8.13.7. The effect of AGE on cognitive decline progression

```
neg.a.interaction.AGE<- glmmadmb(neg.b.ADAS~M+AGE+ APOE4+Gender+  edu.cat +
diagn+ headache+ diclo+Ibu+AGE*M+ (1|ID), family="nbinom1", data=ADASdata)
summary(neg.a.interaction.AGE)


Call:
```

```
glmmadmb(formula = neg.b.ADAS ~ M + AGE + APOE4 + Gender + edu.cat +
    diagn + headache + diclo + Ibu + AGE * M + (1 | ID), data = ADASdata,
    family = "nbinom1")

AIC: 72508.2

Coefficients:
                  Estimate Std. Error z value Pr(>|z|)
(Intercept)       3.00e+00   3.07e-02   97.65  < 2e-16 ***
M                 6.73e-03   1.24e-04   54.10  < 2e-16 ***
AGE               1.38e-02   1.60e-03    8.62  < 2e-16 ***
APOE41            1.63e-01   2.37e-02    6.90  5.1e-12 ***
APOE42            2.43e-01   3.77e-02    6.44  1.2e-10 ***
GenderMale        6.31e-02   2.24e-02    2.82  0.00479 **
edu.cat2tertiary  5.70e-02   2.72e-02    2.10  0.03595 *
edu.cat3mid       1.17e-01   3.08e-02    3.79  0.00015 ***
edu.cat4early     1.53e-01   3.26e-02    4.69  2.8e-06 ***
diagn2EMCI        3.25e-01   3.36e-02    9.68  < 2e-16 ***
diagn3LMCI        7.79e-01   2.90e-02   26.92  < 2e-16 ***
diagn4AD          1.28e+00   3.37e-02   38.03  < 2e-16 ***
headache         -9.20e-02   3.88e-02   -2.37  0.01782 *
diclo            -2.32e-01   8.00e-02   -2.90  0.00374 **
Ibu              -9.98e-02   2.96e-02   -3.37  0.00075 ***
M:AGE            -1.80e-05   1.88e-05   -0.96  0.33760
---
Signif. codes:  0 '***' 0.001 '**' 0.01 '*' 0.05 '.' 0.1 ' ' 1

Number of observations: total=8770, ID=1618
Random effect variance(s):
Group=ID
            Variance StdDev
(Intercept)   0.1676 0.4094

Negative binomial dispersion parameter: 3.3016 (std. err.: 0.055987)

Log-likelihood: -36236.1

anova(neg.a.interaction.AGE, main.A.final)

Analysis of Deviance Table

Model 1: neg.b.ADAS ~ M + AGE + APOE4 + Gender + edu.cat + diagn + headache +
diclo + Ibu
Model 2: neg.b.ADAS ~ M + AGE + APOE4 + Gender + edu.cat + diagn + headache +
diclo + Ibu + AGE * M
  NoPar LogLik Df Deviance Pr(>Chi)
1    17 -36237
2    18 -36236  1        1   0.3173
```

8.13.8. The effect of APOE status on cognitive decline progression

```
neg.a.interaction.APOE4<- glmmadmb(neg.b.ADAS~M+AGE+ APOE4+Gender+  edu.cat +
diagn+ headache+ diclo+Ibu+ APOE4*M+(1|ID), family="nbinom1", data=ADASdata)
summary(neg.a.interaction.APOE4)


Call:
glmmadmb(formula = neg.b.ADAS ~ M + AGE + APOE4 + Gender + edu.cat +
    diagn + headache + diclo + Ibu + APOE4 * M + (1 | ID), data = ADASdata,
```

```
    family = "nbinom1")

AIC: 72285.8

Coefficients:
                  Estimate Std. Error z value Pr(>|z|)
(Intercept)       3.036500   0.030693   98.93  < 2e-16 ***
M                 0.005142   0.000165   31.22  < 2e-16 ***
AGE               0.013391   0.001552    8.63  < 2e-16 ***
APOE41            0.093169   0.024278    3.84  0.00012 ***
APOE42            0.145675   0.038410    3.79  0.00015 ***
GenderMale        0.062943   0.022310    2.82  0.00478 **
edu.cat2tertiary  0.056311   0.027127    2.08  0.03791 *
edu.cat3mid       0.116544   0.030702    3.80  0.00015 ***
edu.cat4early     0.151799   0.032486    4.67    3e-06 ***
diagn2EMCI        0.318188   0.033479    9.50  < 2e-16 ***
diagn3LMCI        0.773176   0.028872   26.78  < 2e-16 ***
diagn4AD          1.284425   0.033580   38.25  < 2e-16 ***
headache         -0.089277   0.038721   -2.31  0.02113 *
diclo            -0.227894   0.079816   -2.86  0.00430 **
Ibu              -0.095656   0.029545   -3.24  0.00121 **
M:APOE41          0.003174   0.000260   12.20  < 2e-16 ***
M:APOE42          0.005050   0.000437   11.56  < 2e-16 ***
---
Signif. codes:  0 '***' 0.001 '**' 0.01 '*' 0.05 '.' 0.1 ' ' 1

Number of observations: total=8770, ID=1618
Random effect variance(s):
Group=ID
            Variance StdDev
(Intercept)   0.1671 0.4088

Negative binomial dispersion parameter: 3.2043 (std. err.: 0.054319)

Log-likelihood: -36123.9

anova(neg.a.interaction.APOE4, main.A.final)

Analysis of Deviance Table

Model 1: neg.b.ADAS ~ M + AGE + APOE4 + Gender + edu.cat + diagn + headache +
diclo + Ibu
Model 2: neg.b.ADAS ~ M + AGE + APOE4 + Gender + edu.cat + diagn + headache +
diclo + Ibu + APOE4 * M
  NoPar LogLik Df Deviance  Pr(>Chi)
1    17 -36237
2    19 -36124  2    225.4 < 2.2e-16 ***
---
Signif. codes:  0 '***' 0.001 '**' 0.01 '*' 0.05 '.' 0.1 ' ' 1
```

8.13.9. The effect of education status on cognitive decline progression

```
neg.a.interaction.edu.cat<- glmmadmb(neg.b.ADAS~M+AGE+ APOE4+Gender+  edu.cat +
diagn+ headache+ diclo+Ibu+edu.cat*M+ (1|ID), family="nbinom1", data=ADASdata)
summary(neg.a.interaction.edu.cat)


Call:
glmmadmb(formula = neg.b.ADAS ~ M + AGE + APOE4 + Gender + edu.cat +
```

```
    diagn + headache + diclo + Ibu + edu.cat * M + (1 | ID),
    data = ADASdata, family = "nbinom1")

AIC: 72478

Coefficients:
                    Estimate Std. Error z value Pr(>|z|)
(Intercept)         2.978833   0.031011   96.06  < 2e-16 ***
M                   0.007460   0.000211   35.31  < 2e-16 ***
AGE                 0.013451   0.001559    8.63  < 2e-16 ***
APOE41              0.164254   0.023701    6.93  4.2e-12 ***
APOE42              0.241581   0.037713    6.41  1.5e-10 ***
GenderMale          0.062936   0.022400    2.81  0.00496 **
edu.cat2tertiary    0.098538   0.028138    3.50  0.00046 ***
edu.cat3mid         0.131083   0.031805    4.12  3.8e-05 ***
edu.cat4early       0.166818   0.033436    4.99  6.1e-07 ***
diagn2EMCI          0.325769   0.033618    9.69  < 2e-16 ***
diagn3LMCI          0.779189   0.028994   26.87  < 2e-16 ***
diagn4AD            1.279701   0.033719   37.95  < 2e-16 ***
headache           -0.091994   0.038874   -2.37  0.01796 *
diclo              -0.232956   0.080146   -2.91  0.00365 **
Ibu                -0.099232   0.029661   -3.35  0.00082 ***
M:edu.cat2tertiary -0.001848   0.000315   -5.86  4.5e-09 ***
M:edu.cat3mid      -0.000620   0.000342   -1.81  0.06958 .
M:edu.cat4early    -0.000565   0.000358   -1.58  0.11476
---
Signif. codes:  0 '***' 0.001 '**' 0.01 '*' 0.05 '.' 0.1 ' ' 1

Number of observations: total=8770, ID=1618
Random effect variance(s):
Group=ID
            Variance StdDev
(Intercept)   0.1682 0.4101

Negative binomial dispersion parameter: 3.2834 (std. err.: 0.055665)

Log-likelihood: -36219

anova(neg.a.interaction.edu.cat, main.A.final)

Analysis of Deviance Table

Model 1: neg.b.ADAS ~ M + AGE + APOE4 + Gender + edu.cat + diagn + headache + 
diclo + Ibu
Model 2: neg.b.ADAS ~ M + AGE + APOE4 + Gender + edu.cat + diagn + headache + 
diclo + Ibu + edu.cat * M
  NoPar LogLik Df Deviance  Pr(>Chi)
1    17 -36237
2    20 -36219  3     35.2 1.105e-07 ***
---
Signif. codes:  0 '***' 0.001 '**' 0.01 '*' 0.05 '.' 0.1 ' ' 1
```

8.13.10. The effect of diabetes on cognitive decline progression

```
neg.a.interaction.diab<- glmmadmb(neg.b.ADAS~M+AGE+ APOE4+Gender+  edu.cat +
diagn+ headache+ diclo+Ibu+diab*M+ (1|ID), family="nbinom1", data=ADASdata)
summary(neg.a.interaction.diab)
```

```
Call:
glmmadmb(formula = neg.b.ADAS ~ M + AGE + APOE4 + Gender + edu.cat +
    diagn + headache + diclo + Ibu + diab * M + (1 | ID), data = ADASdata,
    family = "nbinom1")

AIC: 72509.4

Coefficients:
                  Estimate Std. Error z value Pr(>|z|)
(Intercept)       2.994485   0.030713   97.50  < 2e-16 ***
M                 0.006769   0.000130   52.04  < 2e-16 ***
AGE               0.013466   0.001557    8.65  < 2e-16 ***
APOE41            0.163370   0.023661    6.90  5.0e-12 ***
APOE42            0.242948   0.037675    6.45  1.1e-10 ***
GenderMale        0.062491   0.022447    2.78  0.00537 **
edu.cat2tertiary  0.056087   0.027268    2.06  0.03970 *
edu.cat3mid       0.115475   0.030797    3.75  0.00018 ***
edu.cat4early     0.151322   0.032637    4.64  3.5e-06 ***
diagn2EMCI        0.325245   0.033583    9.68  < 2e-16 ***
diagn3LMCI        0.779731   0.028940   26.94  < 2e-16 ***
diagn4AD          1.281152   0.033664   38.06  < 2e-16 ***
headache         -0.091288   0.038813   -2.35  0.01867 *
diclo            -0.231672   0.080023   -2.90  0.00379 **
Ibu              -0.100332   0.029612   -3.39  0.00070 ***
diab              0.029840   0.038418    0.78  0.43732
M:diab           -0.000534   0.000431   -1.24  0.21544
---
Signif. codes:  0 '***' 0.001 '**' 0.01 '*' 0.05 '.' 0.1 ' ' 1

Number of observations: total=8770, ID=1618
Random effect variance(s):
Group=ID
            Variance StdDev
(Intercept)   0.1675 0.4093

Negative binomial dispersion parameter: 3.3019 (std. err.: 0.055983)

Log-likelihood: -36235.7

anova(neg.a.interaction.diab, main.A.final)

Analysis of Deviance Table

Model 1: neg.b.ADAS ~ M + AGE + APOE4 + Gender + edu.cat + diagn + headache +
diclo + Ibu
Model 2: neg.b.ADAS ~ M + AGE + APOE4 + Gender + edu.cat + diagn + headache +
diclo + Ibu + diab * M
  NoPar LogLik Df Deviance Pr(>Chi)
1    17 -36237
2    19 -36236  2      1.8   0.4066
```

8.13.11. The effect of Gender on cognitive decline progression

```
neg.a.interaction.Gender<- glmmadmb(neg.b.ADAS~M+AGE+ APOE4+Gender+  edu.cat +
diagn+ headache+ diclo+Ibu+Gender*M+ (1|ID), family="nbinom1", data=ADASdata)
summary(neg.a.interaction.Gender)
```

```
Call:
glmmadmb(formula = neg.b.ADAS ~ M + AGE + APOE4 + Gender + edu.cat +
    diagn + headache + diclo + Ibu + Gender * M + (1 | ID), data = ADASdata,
    family = "nbinom1")

AIC: 72452

Coefficients:
                  Estimate Std. Error z value Pr(>|z|)
(Intercept)       2.970505   0.030931   96.04  < 2e-16 ***
M                 0.007830   0.000191   41.03  < 2e-16 ***
AGE               0.013341   0.001559    8.56  < 2e-16 ***
APOE41            0.163579   0.023712    6.90  5.3e-12 ***
APOE42            0.242345   0.037731    6.42  1.3e-10 ***
GenderMale        0.104766   0.023072    4.54  5.6e-06 ***
edu.cat2tertiary  0.056269   0.027248    2.07  0.03892 *
edu.cat3mid       0.114572   0.030841    3.71  0.00020 ***
edu.cat4early     0.153623   0.032631    4.71  2.5e-06 ***
diagn2EMCI        0.326624   0.033633    9.71  < 2e-16 ***
diagn3LMCI        0.781496   0.029002   26.95  < 2e-16 ***
diagn4AD          1.283412   0.033736   38.04  < 2e-16 ***
headache         -0.091613   0.038893   -2.36  0.01850 *
diclo            -0.228988   0.080184   -2.86  0.00429 **
Ibu              -0.101246   0.029676   -3.41  0.00065 ***
M:GenderMale     -0.001884   0.000248   -7.58  3.3e-14 ***
---
Signif. codes:  0 '***' 0.001 '**' 0.01 '*' 0.05 '.' 0.1 ' ' 1

Number of observations: total=8770, ID=1618
Random effect variance(s):
Group=ID
            Variance StdDev
(Intercept)   0.1684 0.4104

Negative binomial dispersion parameter: 3.2728 (std. err.: 0.055469)

Log-likelihood: -36208

anova(neg.a.interaction.Gender, main.A.final)

Analysis of Deviance Table

Model 1: neg.b.ADAS ~ M + AGE + APOE4 + Gender + edu.cat + diagn + headache +
diclo + Ibu
Model 2: neg.b.ADAS ~ M + AGE + APOE4 + Gender + edu.cat + diagn + headache +
diclo + Ibu + Gender * M
  NoPar LogLik Df Deviance  Pr(>Chi)
1    17 -36237
2    18 -36208  1     57.2 3.941e-14 ***
---
Signif. codes:  0 '***' 0.001 '**' 0.01 '*' 0.05 '.' 0.1 ' ' 1
```

8.13.12. The effect of aspirin on cognitive decline progression

```
neg.a.interaction.aspirin<- glmmadmb(neg.b.ADAS~M+AGE+ APOE4+Gender+  edu.cat +
diagn+ headache+ diclo+Ibu+aspirin*M+ (1|ID), family="nbinom1", data=ADASdata)
summary(neg.a.interaction.aspirin)
```

```
Call:
glmmadmb(formula = neg.b.ADAS ~ M + AGE + APOE4 + Gender + edu.cat +
    diagn + headache + diclo + Ibu + aspirin * M + (1 | ID),
    data = ADASdata, family = "nbinom1")

AIC: 72498.8

Coefficients:
                  Estimate Std. Error z value Pr(>|z|)
(Intercept)       2.994179   0.033049   90.60  < 2e-16 ***
M                 0.007378   0.000229   32.28  < 2e-16 ***
AGE               0.013553   0.001560    8.69  < 2e-16 ***
APOE41            0.162889   0.023693    6.87  6.2e-12 ***
APOE42            0.243206   0.037704    6.45  1.1e-10 ***
GenderMale        0.066751   0.022572    2.96  0.00310 **
edu.cat2tertiary  0.056277   0.027225    2.07  0.03872 *
edu.cat3mid       0.115801   0.030812    3.76  0.00017 ***
edu.cat4early     0.151793   0.032624    4.65  3.3e-06 ***
diagn2EMCI        0.324355   0.033607    9.65  < 2e-16 ***
diagn3LMCI        0.778754   0.029013   26.84  < 2e-16 ***
diagn4AD          1.280139   0.033940   37.72  < 2e-16 ***
headache         -0.092640   0.038860   -2.38  0.01713 *
diclo            -0.226726   0.080206   -2.83  0.00470 **
Ibu              -0.099038   0.029653   -3.34  0.00084 ***
aspirin          -0.001704   0.022635   -0.08  0.94001
M:aspirin        -0.000919   0.000270   -3.40  0.00068 ***
---
Signif. codes:  0 '***' 0.001 '**' 0.01 '*' 0.05 '.' 0.1 ' ' 1

Number of observations: total=8770, ID=1618
Random effect variance(s):
Group=ID
            Variance StdDev
(Intercept)    0.168 0.4099

Negative binomial dispersion parameter: 3.2943 (std. err.: 0.055868)

Log-likelihood: -36230.4

anova(neg.a.interaction.aspirin, main.A.final)

Analysis of Deviance Table

Model 1: neg.b.ADAS ~ M + AGE + APOE4 + Gender + edu.cat + diagn + headache +
diclo + Ibu
Model 2: neg.b.ADAS ~ M + AGE + APOE4 + Gender + edu.cat + diagn + headache +
diclo + Ibu + aspirin * M
  NoPar LogLik Df Deviance Pr(>Chi)
1    17 -36237
2    19 -36230  2     12.4 0.002029 **
---
Signif. codes:  0 '***' 0.001 '**' 0.01 '*' 0.05 '.' 0.1 ' ' 1
```

8.13.13. The effect of paracetamol on cognitive decline progression

```
neg.a.interaction.parac<- glmmadmb(neg.b.ADAS~M+AGE+ APOE4+Gender+  edu.cat +
diagn+ headache+ diclo+Ibu+parac*M+ (1|ID), family="nbinom1", data=ADASdata)
summary(neg.a.interaction.parac)
```

```
Call:
glmmadmb(formula = neg.b.ADAS ~ M + AGE + APOE4 + Gender + edu.cat +
    diagn + headache + diclo + Ibu + parac * M + (1 | ID), data = ADASdata,
    family = "nbinom1")

AIC: 72502.6

Coefficients:
                  Estimate Std. Error z value Pr(>|z|)
(Intercept)       3.007526   0.031457   95.61  < 2e-16 ***
M                 0.006457   0.000156   41.32  < 2e-16 ***
AGE               0.013482   0.001557    8.66  < 2e-16 ***
APOE41            0.162151   0.023657    6.85  7.2e-12 ***
APOE42            0.240711   0.037615    6.40  1.6e-10 ***
GenderMale        0.061826   0.022395    2.76  0.00577 **
edu.cat2tertiary  0.057689   0.027172    2.12  0.03374 *
edu.cat3mid       0.116911   0.030766    3.80  0.00014 ***
edu.cat4early     0.151943   0.032529    4.67  3.0e-06 ***
diagn2EMCI        0.325530   0.033531    9.71  < 2e-16 ***
diagn3LMCI        0.779367   0.028911   26.96  < 2e-16 ***
diagn4AD          1.278976   0.033657   38.00  < 2e-16 ***
headache         -0.089595   0.038890   -2.30  0.02123 *
diclo            -0.231049   0.079991   -2.89  0.00387 **
Ibu              -0.097793   0.029744   -3.29  0.00101 **
parac            -0.037224   0.025918   -1.44  0.15094
M:parac           0.000717   0.000255    2.81  0.00489 **
---
Signif. codes:  0 '***' 0.001 '**' 0.01 '*' 0.05 '.' 0.1 ' ' 1

Number of observations: total=8770, ID=1618
Random effect variance(s):
Group=ID
            Variance StdDev
(Intercept)   0.1671 0.4088

Negative binomial dispersion parameter: 3.3002 (std. err.: 0.055953)

Log-likelihood: -36232.3

anova(neg.a.interaction.parac, main.A.final)

Analysis of Deviance Table

Model 1: neg.b.ADAS ~ M + AGE + APOE4 + Gender + edu.cat + diagn + headache +
diclo + Ibu
Model 2: neg.b.ADAS ~ M + AGE + APOE4 + Gender + edu.cat + diagn + headache +
diclo + Ibu + parac * M
  NoPar LogLik Df Deviance Pr(>Chi)
1    17 -36237
2    19 -36232  2      8.6  0.01357 *
---
Signif. codes:  0 '***' 0.001 '**' 0.01 '*' 0.05 '.' 0.1 ' ' 1
```

8.13.14. The effect of diclofenac on cognitive decline progression

```
neg.a.interaction.diclo<- glmmadmb(neg.b.ADAS~M+AGE+ APOE4+Gender+  edu.cat +
diagn+ headache+ diclo+Ibu+diclo*M+ (1|ID), family="nbinom1", data=ADASdata)
summary(neg.a.interaction.diclo)
```

```
Call:
glmmadmb(formula = neg.b.ADAS ~ M + AGE + APOE4 + Gender + edu.cat +
    diagn + headache + diclo + Ibu + diclo * M + (1 | ID), data = ADASdata,
    family = "nbinom1")

AIC: 72507.6

Coefficients:
                  Estimate Std. Error z value Pr(>|z|)
(Intercept)       2.995474   0.030681   97.63  < 2e-16 ***
M                 0.006750   0.000126   53.45  < 2e-16 ***
AGE               0.013436   0.001556    8.63  < 2e-16 ***
APOE41            0.163209   0.023661    6.90  5.3e-12 ***
APOE42            0.242229   0.037648    6.43  1.2e-10 ***
GenderMale        0.063397   0.022362    2.84  0.00458 **
edu.cat2tertiary  0.056963   0.027191    2.09  0.03618 *
edu.cat3mid       0.116438   0.030774    3.78  0.00015 ***
edu.cat4early     0.152731   0.032562    4.69  2.7e-06 ***
diagn2EMCI        0.325482   0.033565    9.70  < 2e-16 ***
diagn3LMCI        0.779719   0.028941   26.94  < 2e-16 ***
diagn4AD          1.281247   0.033666   38.06  < 2e-16 ***
headache         -0.091821   0.038808   -2.37  0.01798 *
diclo            -0.203363   0.083081   -2.45  0.01437 *
Ibu              -0.100150   0.029612   -3.38  0.00072 ***
M:diclo          -0.000876   0.000693   -1.26  0.20636
---
Signif. codes:  0 '***' 0.001 '**' 0.01 '*' 0.05 '.' 0.1 ' ' 1

Number of observations: total=8770, ID=1618
Random effect variance(s):
Group=ID
            Variance StdDev
(Intercept)   0.1675 0.4093

Negative binomial dispersion parameter: 3.302 (std. err.: 0.055985)

Log-likelihood: -36235.8

anova(neg.a.interaction.diclo, main.A.final)

Analysis of Deviance Table

Model 1: neg.b.ADAS ~ M + AGE + APOE4 + Gender + edu.cat + diagn + headache +
diclo + Ibu
Model 2: neg.b.ADAS ~ M + AGE + APOE4 + Gender + edu.cat + diagn + headache +
diclo + Ibu + diclo * M
  NoPar LogLik Df Deviance Pr(>Chi)
1    17 -36237
2    18 -36236  1      1.6   0.2059
```

8.13.15. The effect of ibuprofen on cognitive decline progression

```
neg.a.interaction.Ibu<- glmmadmb(neg.b.ADAS~M+AGE+ APOE4+Gender+  edu.cat +
diagn+ headache+ diclo+Ibu+Ibu*M+ (1|ID), family="nbinom1", data=ADASdata)
summary(neg.a.interaction.Ibu)


Call:
```

```
glmmadmb(formula = neg.b.ADAS ~ M + AGE + APOE4 + Gender + edu.cat +
    diagn + headache + diclo + Ibu + Ibu * M + (1 | ID), data = ADASdata,
    family = "nbinom1")

AIC: 72508.6

Coefficients:
                  Estimate Std. Error z value Pr(>|z|)
(Intercept)       2.995124   0.030707   97.54  < 2e-16 ***
M                 0.006771   0.000140   48.32  < 2e-16 ***
AGE               0.013451   0.001556    8.64  < 2e-16 ***
APOE41            0.163245   0.023662    6.90  5.2e-12 ***
APOE42            0.242099   0.037648    6.43  1.3e-10 ***
GenderMale        0.063190   0.022362    2.83  0.00472 **
edu.cat2tertiary  0.057114   0.027191    2.10  0.03569 *
edu.cat3mid       0.116645   0.030774    3.79  0.00015 ***
edu.cat4early     0.152618   0.032562    4.69  2.8e-06 ***
diagn2EMCI        0.325426   0.033565    9.70  < 2e-16 ***
diagn3LMCI        0.779600   0.028941   26.94  < 2e-16 ***
diagn4AD          1.281303   0.033668   38.06  < 2e-16 ***
headache         -0.091849   0.038808   -2.37  0.01795 *
diclo            -0.232437   0.080014   -2.90  0.00367 **
Ibu              -0.093796   0.030663   -3.06  0.00222 **
M:Ibu            -0.000230   0.000299   -0.77  0.44217
---
Signif. codes:  0 '***' 0.001 '**' 0.01 '*' 0.05 '.' 0.1 ' ' 1

Number of observations: total=8770, ID=1618
Random effect variance(s):
Group=ID
            Variance StdDev
(Intercept)   0.1675 0.4093

Negative binomial dispersion parameter: 3.3025 (std. err.: 0.055993)

Log-likelihood: -36236.3

anova(neg.a.interaction.Ibu, main.A.final)

Analysis of Deviance Table

Model 1: neg.b.ADAS ~ M + AGE + APOE4 + Gender + edu.cat + diagn + headache +
diclo + Ibu
Model 2: neg.b.ADAS ~ M + AGE + APOE4 + Gender + edu.cat + diagn + headache +
diclo + Ibu + Ibu * M
  NoPar LogLik Df Deviance Pr(>Chi)
1    17 -36237
2    18 -36236  1      0.6   0.4386
```

8.13.16. The effect of naproxin on cognitive decline progression

```
neg.a.interaction.naprox<- glmmadmb(neg.b.ADAS~M+AGE+ APOE4+Gender+  edu.cat +
diagn+ headache+ diclo+Ibu+naprox*M (1|ID), family="nbinom1", data=ADASdata)
summary(neg.a.interaction.naprox)


Call:
glmmadmb(formula = neg.b.ADAS ~ M + AGE + APOE4 + Gender + edu.cat +
    diagn + headache + diclo + Ibu + naprox * M(1 | ID), data = ADASdata,
```

```
    family = "nbinom1")

AIC: 72504.4

Coefficients:
                  Estimate Std. Error z value Pr(>|z|)
(Intercept)       3.001528   0.030899   97.14  < 2e-16 ***
M                 0.006575   0.000137   48.00  < 2e-16 ***
AGE               0.013424   0.001555    8.63  < 2e-16 ***
APOE41            0.163477   0.023635    6.92  4.6e-12 ***
APOE42            0.241305   0.037608    6.42  1.4e-10 ***
GenderMale        0.062568   0.022355    2.80  0.00513 **
edu.cat2tertiary  0.057595   0.027180    2.12  0.03409 *
edu.cat3mid       0.116517   0.030749    3.79  0.00015 ***
edu.cat4early     0.152924   0.032531    4.70  2.6e-06 ***
diagn2EMCI        0.326133   0.033534    9.73  < 2e-16 ***
diagn3LMCI        0.779605   0.028908   26.97  < 2e-16 ***
diagn4AD          1.278919   0.033677   37.98  < 2e-16 ***
headache         -0.091607   0.038768   -2.36  0.01813 *
diclo            -0.231299   0.079934   -2.89  0.00381 **
Ibu              -0.098405   0.029666   -3.32  0.00091 ***
naprox           -0.039186   0.034797   -1.13  0.26010
M:naprox          0.000824   0.000322    2.56  0.01038 *
---
Signif. codes:  0 '***' 0.001 '**' 0.01 '*' 0.05 '.' 0.1 ' ' 1

Number of observations: total=8770, ID=1618
Random effect variance(s):
Group=ID
            Variance StdDev
(Intercept)   0.1671 0.4088

Negative binomial dispersion parameter: 3.3011 (std. err.: 0.055973)

Log-likelihood: -36233.2

anova(neg.a.interaction.naprox, main.A.final)

Analysis of Deviance Table

Model 1: neg.b.ADAS ~ M + AGE + APOE4 + Gender + edu.cat + diagn + headache +
diclo + Ibu
Model 2: neg.b.ADAS ~ M + AGE + APOE4 + Gender + edu.cat + diagn + headache +
diclo + Ibu + naprox * M
  NoPar LogLik Df Deviance Pr(>Chi)
1    17 -36237
2    19 -36233  2      6.8  0.03337 *
---
Signif. codes:  0 '***' 0.001 '**' 0.01 '*' 0.05 '.' 0.1 ' ' 1
```

8.13.17. The effect of celecoxib on cognitive decline progression

```
neg.a.interaction.celex<- glmmadmb(neg.b.ADAS~M+AGE+ APOE4+Gender+  edu.cat +
diagn+ headache+ diclo+Ibu+celex*M (1|ID), family="nbinom1", data=ADASdata)
summary(neg.a.interaction.celex)


Call:
glmmadmb(formula = neg.b.ADAS ~ M + AGE + APOE4 + Gender + edu.cat +
```

```
    diagn + headache + diclo + Ibu + celex * M(1 | ID), data = ADASdata,
    family = "nbinom1")

AIC: 72510.8

Coefficients:
                  Estimate Std. Error z value Pr(>|z|)
(Intercept)       2.995508   0.030797   97.27  < 2e-16 ***
M                 0.006740   0.000128   52.66  < 2e-16 ***
AGE               0.013440   0.001556    8.64  < 2e-16 ***
APOE41            0.163354   0.023659    6.90  5.0e-12 ***
APOE42            0.242367   0.037648    6.44  1.2e-10 ***
GenderMale        0.063225   0.022361    2.83  0.00469 **
edu.cat2tertiary  0.056902   0.027190    2.09  0.03637 *
edu.cat3mid       0.116466   0.030771    3.78  0.00015 ***
edu.cat4early     0.152566   0.032572    4.68  2.8e-06 ***
diagn2EMCI        0.325535   0.033564    9.70  < 2e-16 ***
diagn3LMCI        0.779680   0.028940   26.94  < 2e-16 ***
diagn4AD          1.281279   0.033704   38.02  < 2e-16 ***
headache         -0.091745   0.038882   -2.36  0.01830 *
diclo            -0.232037   0.080047   -2.90  0.00375 **
Ibu              -0.099851   0.029654   -3.37  0.00076 ***
celex             0.013661   0.056682    0.24  0.80955
M:celex          -0.000327   0.000525   -0.62  0.53293
---
Signif. codes:  0 '***' 0.001 '**' 0.01 '*' 0.05 '.' 0.1 ' ' 1

Number of observations: total=8770, ID=1618
Random effect variance(s):
Group=ID
            Variance StdDev
(Intercept)   0.1675 0.4093

Negative binomial dispersion parameter: 3.3026 (std. err.: 0.055996)

Log-likelihood: -36236.4

anova(neg.a.interaction.celex, main.A.final)

Analysis of Deviance Table

Model 1: neg.b.ADAS ~ M + AGE + APOE4 + Gender + edu.cat + diagn + headache +
diclo + Ibu
Model 2: neg.b.ADAS ~ M + AGE + APOE4 + Gender + edu.cat + diagn + headache +
diclo + Ibu + celex * M
  NoPar LogLik Df Deviance Pr(>Chi)
1    17 -36237
2    19 -36236  2      0.4   0.8187
```

8.14. *Combined interaction model*

```
neg.a.combined<- glmmadmb(neg.b.ADAS~M+AGE+ APOE4+Gender+  edu.cat + diagn+
headache+ diclo+Ibu+ edu.cat*M+
smoke*M+diagn*M+APOE4*M+Gender*M+parac*M+diclo*M+ (1|ID), family="nbinom1",
data=ADASdata)
summary(neg.a.combined)


Call:
```

```
glmmadmb(formula = neg.b.ADAS ~ M + AGE + APOE4 + Gender + edu.cat +
    diagn + headache + diclo + Ibu + edu.cat * M + smoke * M +
    diagn * M + APOE4 * M + Gender * M + parac * M + diclo *
    M + (1 | ID), data = ADASdata, family = "nbinom1")

AIC: 72128.2

Coefficients:
                    Estimate Std. Error z value Pr(>|z|)
(Intercept)         2.989645   0.032762   91.25  < 2e-16 ***
M                   0.007308   0.000347   21.04  < 2e-16 ***
AGE                 0.013325   0.001562    8.53  < 2e-16 ***
APOE41              0.095188   0.024426    3.90  9.7e-05 ***
APOE42              0.148257   0.038673    3.83  0.00013 ***
GenderMale          0.105252   0.023297    4.52  6.2e-06 ***
edu.cat2tertiary    0.104051   0.028158    3.70  0.00022 ***
edu.cat3mid         0.148367   0.031898    4.65  3.3e-06 ***
edu.cat4early       0.179550   0.033490    5.36  8.3e-08 ***
diagn2EMCI          0.358696   0.035137   10.21  < 2e-16 ***
diagn3LMCI          0.771943   0.030022   25.71  < 2e-16 ***
diagn4AD            1.248909   0.034818   35.87  < 2e-16 ***
headache           -0.087011   0.039034   -2.23  0.02581 *
diclo              -0.187060   0.083036   -2.25  0.02427 *
Ibu                -0.094288   0.029861   -3.16  0.00159 **
smoke               0.007713   0.025548    0.30  0.76274
parac              -0.036288   0.025980   -1.40  0.16249
M:edu.cat2tertiary -0.002151   0.000309   -6.96  3.4e-12 ***
M:edu.cat3mid      -0.001548   0.000349   -4.44  9.0e-06 ***
M:edu.cat4early    -0.001315   0.000362   -3.63  0.00028 ***
M:smoke             0.000325   0.000274    1.19  0.23532
M:diagn2EMCI       -0.001647   0.000410   -4.02  5.9e-05 ***
M:diagn3LMCI        0.000199   0.000287    0.69  0.48877
M:diagn4AD          0.004435   0.000727    6.10  1.1e-09 ***
M:APOE41            0.002988   0.000263   11.34  < 2e-16 ***
M:APOE42            0.004855   0.000451   10.77  < 2e-16 ***
M:GenderMale       -0.002095   0.000256   -8.19  2.6e-16 ***
M:parac             0.000598   0.000252    2.38  0.01748 *
M:diclo            -0.001178   0.000681   -1.73  0.08382 .
---
Signif. codes:  0 '***' 0.001 '**' 0.01 '*' 0.05 '.' 0.1 ' ' 1

Number of observations: total=8770, ID=1618
Random effect variance(s):
Group=ID
            Variance StdDev
(Intercept)   0.1693 0.4114

Negative binomial dispersion parameter: 3.1173 (std. err.: 0.052809)

Log-likelihood: -36033.1

anova(neg.a.combined, main.A.final)

Analysis of Deviance Table

Model 1: neg.b.ADAS ~ M + AGE + APOE4 + Gender + edu.cat + diagn + headache +
diclo + Ibu
Model 2: neg.b.ADAS ~ M + AGE + APOE4 + Gender + edu.cat + diagn + headache +
diclo + Ibu + edu.cat * M + smoke * M + diagn * M + APOE4 * M + Gender * M +
```

```
parac * M + diclo * M
  NoPar LogLik Df Deviance  Pr(>Chi)
1    17 -36237
2    31 -36033 14      407 < 2.2e-16 ***
---
Signif. codes:  0 '***' 0.001 '**' 0.01 '*' 0.05 '.' 0.1 ' ' 1
```

8.14.1. Dropping non-significant interactions

```
neg.a.combined.drop.smoke<- glmmadmb(neg.b.ADAS~M+AGE+ APOE4+Gender+  edu.cat +
diagn+ headache+ diclo+Ibu+ edu.cat*M+ diagn*M+APOE4*M+Gender*M+parac*M+diclo*M+
(1|ID), family="nbinom1", data=ADASdata)
summary(neg.a.combined.drop.smoke)


Call:
glmmadmb(formula = neg.b.ADAS ~ M + AGE + APOE4 + Gender + edu.cat +
    diagn + headache + diclo + Ibu + edu.cat * M + diagn * M +
    APOE4 * M + Gender * M + parac * M + diclo * M + (1 | ID),
    data = ADASdata, family = "nbinom1")

AIC: 72126

Coefficients:
                    Estimate Std. Error z value Pr(>|z|)
(Intercept)         2.990323   0.032490   92.04  < 2e-16 ***
M                   0.007399   0.000339   21.82  < 2e-16 ***
AGE                 0.013308   0.001562    8.52  < 2e-16 ***
APOE41              0.095546   0.024425    3.91  9.2e-05 ***
APOE42              0.147989   0.038648    3.83  0.00013 ***
GenderMale          0.106309   0.023148    4.59  4.4e-06 ***
edu.cat2tertiary    0.104703   0.028119    3.72  0.00020 ***
edu.cat3mid         0.148963   0.031845    4.68  2.9e-06 ***
edu.cat4early       0.180123   0.033464    5.38  7.3e-08 ***
diagn2EMCI          0.358418   0.035118   10.21  < 2e-16 ***
diagn3LMCI          0.772093   0.030023   25.72  < 2e-16 ***
diagn4AD            1.248836   0.034817   35.87  < 2e-16 ***
headache           -0.087237   0.039035   -2.23  0.02543 *
diclo              -0.186690   0.083038   -2.25  0.02456 *
Ibu                -0.094001   0.029855   -3.15  0.00164 **
parac              -0.035660   0.025975   -1.37  0.16980
M:edu.cat2tertiary -0.002141   0.000309   -6.93  4.3e-12 ***
M:edu.cat3mid      -0.001521   0.000348   -4.37  1.2e-05 ***
M:edu.cat4early    -0.001295   0.000362   -3.58  0.00034 ***
M:diagn2EMCI       -0.001670   0.000410   -4.08  4.6e-05 ***
M:diagn3LMCI        0.000175   0.000286    0.61  0.54034
M:diagn4AD          0.004423   0.000727    6.08  1.2e-09 ***
M:APOE41            0.002978   0.000263   11.31  < 2e-16 ***
M:APOE42            0.004830   0.000450   10.72  < 2e-16 ***
M:GenderMale       -0.002079   0.000255   -8.14  3.9e-16 ***
M:parac             0.000586   0.000252    2.33  0.01979 *
M:diclo            -0.001185   0.000681   -1.74  0.08212 .
---
Signif. codes:  0 '***' 0.001 '**' 0.01 '*' 0.05 '.' 0.1 ' ' 1

Number of observations: total=8770, ID=1618
Random effect variance(s):
Group=ID
            Variance StdDev
```

```
(Intercept)   0.1693 0.4115

Negative binomial dispersion parameter: 3.1181 (std. err.: 0.05282)

Log-likelihood: -36034

anova(neg.a.combined, neg.a.combined.drop.smoke)

Analysis of Deviance Table

Model 1: neg.b.ADAS ~ M + AGE + APOE4 + Gender + edu.cat + diagn + headache +
diclo + Ibu + edu.cat * M + diagn * M + APOE4 * M + Gender * M + parac * M +
diclo * M
Model 2: neg.b.ADAS ~ M + AGE + APOE4 + Gender + edu.cat + diagn + headache +
diclo + Ibu + edu.cat * M + diagn * M + APOE4 * M + Gender * M + parac * M +
diclo * M
  NoPar LogLik Df Deviance Pr(>Chi)
1    28 -36036
2    29 -36033  1      3   0.08326

neg.a.combined.drop.diclo<- glmmadmb(neg.b.ADAS~M+AGE+ APOE4+Gender+  edu.cat +
diagn+ headache+ diclo+Ibu+ edu.cat*M+ diagn*M+APOE4*M+Gender*M+parac*M+ (1|ID),
family="nbinom1", data=ADASdata)
summary(neg.a.combined.drop.diclo)


Call:
glmmadmb(formula = neg.b.ADAS ~ M + AGE + APOE4 + Gender + edu.cat +
    diagn + headache + diclo + Ibu + edu.cat * M + diagn * M +
    APOE4 * M + Gender * M + parac * M + (1 | ID), data = ADASdata,
    family = "nbinom1")

AIC: 72127

Coefficients:
                    Estimate Std. Error z value Pr(>|z|)
(Intercept)         2.991271   0.032483   92.09  < 2e-16 ***
M                   0.007364   0.000339   21.75  < 2e-16 ***
AGE                 0.013316   0.001562    8.52  < 2e-16 ***
APOE41              0.095729   0.024424    3.92  8.9e-05 ***
APOE42              0.149163   0.038638    3.86  0.00011 ***
GenderMale          0.106536   0.023147    4.60  4.2e-06 ***
edu.cat2tertiary    0.104722   0.028117    3.72  0.00020 ***
edu.cat3mid         0.148404   0.031842    4.66  3.2e-06 ***
edu.cat4early       0.180975   0.033458    5.41  6.3e-08 ***
diagn2EMCI          0.357465   0.035112   10.18  < 2e-16 ***
diagn3LMCI          0.771174   0.030017   25.69  < 2e-16 ***
diagn4AD            1.247515   0.034807   35.84  < 2e-16 ***
headache           -0.087110   0.039032   -2.23  0.02563 *
diclo              -0.224372   0.080266   -2.80  0.00518 **
Ibu                -0.093702   0.029852   -3.14  0.00170 **
parac              -0.035014   0.025971   -1.35  0.17759
M:edu.cat2tertiary -0.002137   0.000309   -6.91  4.7e-12 ***
M:edu.cat3mid      -0.001490   0.000348   -4.29  1.8e-05 ***
M:edu.cat4early    -0.001348   0.000361   -3.74  0.00019 ***
M:diagn2EMCI       -0.001629   0.000409   -3.98  6.8e-05 ***
M:diagn3LMCI        0.000210   0.000286    0.74  0.46177
M:diagn4AD          0.004483   0.000726    6.17  6.8e-10 ***
M:APOE41            0.002980   0.000263   11.31  < 2e-16 ***
M:APOE42            0.004757   0.000448   10.61  < 2e-16 ***
```

```
M:GenderMale       -0.002098   0.000255   -8.22  < 2e-16 ***
M:parac             0.000555   0.000251    2.21  0.02703 *
---
Signif. codes:  0 '***' 0.001 '**' 0.01 '*' 0.05 '.' 0.1 ' ' 1

Number of observations: total=8770, ID=1618
Random effect variance(s):
Group=ID
            Variance StdDev
(Intercept)   0.1693 0.4114

Negative binomial dispersion parameter: 3.1196 (std. err.: 0.052844)

Log-likelihood: -36035.5

anova(neg.a.combined, neg.a.combined.drop.diclo)

Analysis of Deviance Table

Model 1: neg.b.ADAS ~ M + AGE + APOE4 + Gender + edu.cat + diagn + headache +
diclo + Ibu + edu.cat * M + diagn * M + APOE4 * M + Gender * M + parac * M
Model 2: neg.b.ADAS ~ M + AGE + APOE4 + Gender + edu.cat + diagn + headache +
diclo + Ibu + edu.cat * M + smoke * M + diagn * M + APOE4 * M + Gender * M +
parac * M + diclo * M
  NoPar LogLik Df Deviance Pr(>Chi)
1    28 -36036
2    31 -36033  3      4.8    0.187
```

8.15. *Final full model and plots of the coeffecients*

The full model of all significant interaction terms is now created and non-significant terms will be removed.

```
neg.ADAS<- glmmadmb(neg.b.ADAS~M+AGE+ APOE4+Gender+  edu.cat + diagn+ headache+
diclo+Ibu+ edu.cat*M+diagn*M+APOE4*M+Gender*M+parac*M+ (1|ID), family="nbinom1",
data=ADASdata)

summary(neg.ADAS)


Call:
glmmadmb(formula = neg.b.ADAS ~ M + AGE + APOE4 + Gender + edu.cat +
    diagn + headache + diclo + Ibu + edu.cat * M + diagn * M +
    APOE4 * M + Gender * M + parac * M + (1 | ID), data = ADASdata,
    family = "nbinom1")

AIC: 72127

Coefficients:
                    Estimate Std. Error z value Pr(>|z|)
(Intercept)         2.991271   0.032483   92.09  < 2e-16 ***
M                   0.007364   0.000339   21.75  < 2e-16 ***
AGE                 0.013316   0.001562    8.52  < 2e-16 ***
APOE41              0.095729   0.024424    3.92  8.9e-05 ***
APOE42              0.149163   0.038638    3.86  0.00011 ***
GenderMale          0.106536   0.023147    4.60  4.2e-06 ***
edu.cat2tertiary    0.104722   0.028117    3.72  0.00020 ***
edu.cat3mid         0.148404   0.031842    4.66  3.2e-06 ***
edu.cat4early       0.180975   0.033458    5.41  6.3e-08 ***
```

```
diagn2EMCI          0.357465   0.035112   10.18  < 2e-16 ***
diagn3LMCI          0.771174   0.030017   25.69  < 2e-16 ***
diagn4AD            1.247515   0.034807   35.84  < 2e-16 ***
headache           -0.087110   0.039032   -2.23  0.02563 *
diclo              -0.224372   0.080266   -2.80  0.00518 **
Ibu                -0.093702   0.029852   -3.14  0.00170 **
parac              -0.035014   0.025971   -1.35  0.17759
M:edu.cat2tertiary -0.002137   0.000309   -6.91  4.7e-12 ***
M:edu.cat3mid      -0.001490   0.000348   -4.29  1.8e-05 ***
M:edu.cat4early    -0.001348   0.000361   -3.74  0.00019 ***
M:diagn2EMCI       -0.001629   0.000409   -3.98  6.8e-05 ***
M:diagn3LMCI        0.000210   0.000286    0.74  0.46177
M:diagn4AD          0.004483   0.000726    6.17  6.8e-10 ***
M:APOE41            0.002980   0.000263   11.31  < 2e-16 ***
M:APOE42            0.004757   0.000448   10.61  < 2e-16 ***
M:GenderMale       -0.002098   0.000255   -8.22  < 2e-16 ***
M:parac             0.000555   0.000251    2.21  0.02703 *
---
Signif. codes:  0 '***' 0.001 '**' 0.01 '*' 0.05 '.' 0.1 ' ' 1

Number of observations: total=8770, ID=1618
Random effect variance(s):
Group=ID
            Variance StdDev
(Intercept)   0.1693 0.4114

Negative binomial dispersion parameter: 3.1196 (std. err.: 0.052844)

Log-likelihood: -36035.5

anova(neg.a.combined, neg.ADAS)

Analysis of Deviance Table

Model 1: neg.b.ADAS ~ M + AGE + APOE4 + Gender + edu.cat + diagn + headache +
diclo + Ibu + edu.cat * M + diagn * M + APOE4 * M + Gender * M + parac * M
Model 2: neg.b.ADAS ~ M + AGE + APOE4 + Gender + edu.cat + diagn + headache +
diclo + Ibu + edu.cat * M + smoke * M + diagn * M + APOE4 * M + Gender * M +
parac * M + diclo * M
  NoPar LogLik Df Deviance Pr(>Chi)
1    28 -36036
2    31 -36033  3      4.8    0.187
```

8.15.1. Coeffecient plot

```
coefplot(neg.ADAS)
```

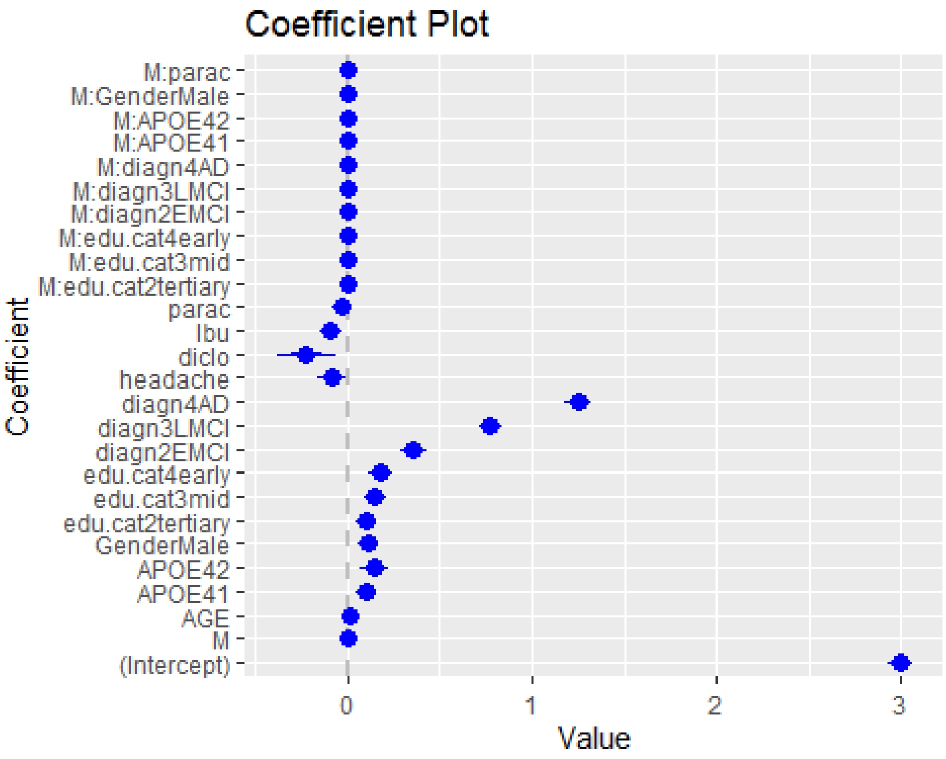

8.15.2. Coeffecient plot of interaction terms

```
coefficients<-
c("M:parac","M:APOE41","M:APOE42","M:edu.cat2tertiary","M:edu.cat3mid","M:edu.ca
t4early","M:diagn2EMCI","M:diagn3LMCI","M:diagn4AD","M")
coefplot(neg.ADAS,coefficients=coefficients,main="Interaction terms
coeffecients")
```

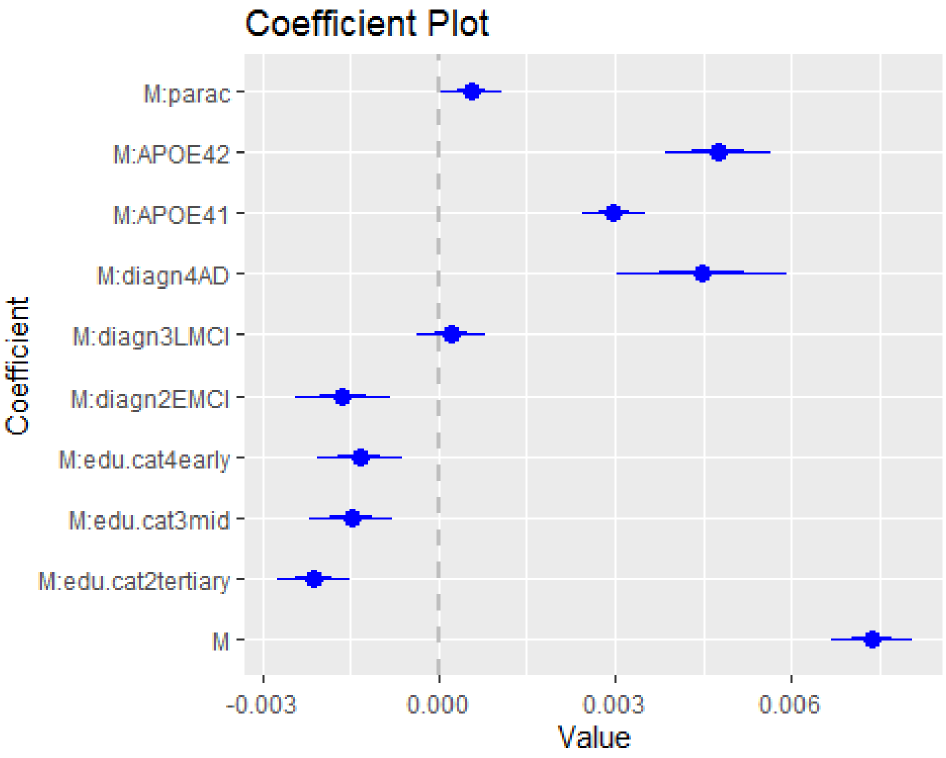

8.16. *Dropping terms of the model to evaluate the significance of each variable in the ful model.*

```
No.Age.ADAS<- glmmadmb(neg.b.ADAS~M+APOE4+Gender+  edu.cat + diagn+ headache+ diclo+Ibu+ edu.cat*M+ diagn*M+APOE4*M+Gender*M+parac*M+ (1|ID), family="nbinom1", data=ADASdata)


No.APOE4.ADAS<- glmmadmb(neg.b.ADAS~M+AGE+ Gender+  edu.cat + diagn+ headache+ diclo+Ibu+ edu.cat*M+ diagn*M+Gender*M+parac*M+ (1|ID), family="nbinom1", data=ADASdata)

APOE4.ADAS.Main<- glmmadmb(neg.b.ADAS~M+AGE+ APOE4+Gender+  edu.cat + diagn+ headache+ diclo+Ibu+ edu.cat*M+ diagn*M+Gender*M+parac*M+ (1|ID), family="nbinom1", data=ADASdata)

Education.ADAS.Main<- glmmadmb(neg.b.ADAS~M+AGE+ APOE4+Gender+  edu.cat + diagn+ headache+ diclo+Ibu+ diagn*M+APOE4*M+Gender*M+parac*M+ (1|ID), family="nbinom1", data=ADASdata)

No.Education.ADAS<- glmmadmb(neg.b.ADAS~M+AGE+ APOE4+Gender+  diagn+ headache+ diclo+Ibu+ diagn*M+APOE4*M+Gender*M+parac*M+ (1|ID), family="nbinom1", data=ADASdata)

Diagn.ADAS.Main<- glmmadmb(neg.b.ADAS~M+AGE+ APOE4+Gender+  edu.cat + diagn+ headache+ diclo+Ibu+ edu.cat*M+APOE4*M+Gender*M+parac*M+ (1|ID), family="nbinom1", data=ADASdata)

No.Diagn.ADAS<- glmmadmb(neg.b.ADAS~M+AGE+ APOE4+Gender+  edu.cat + headache+ diclo+Ibu+ edu.cat*M+APOE4*M+Gender*M+parac*M+ (1|ID), family="nbinom1", data=ADASdata)

Gender.ADAS.Main<- glmmadmb(neg.b.ADAS~M+AGE+ APOE4+Gender+  edu.cat + diagn+ headache+ diclo+Ibu+ edu.cat*M+ diagn*M+APOE4*M+parac*M+ (1|ID), family="nbinom1", data=ADASdata)

No.Gender.ADAS<- glmmadmb(neg.b.ADAS~M+AGE+ APOE4+  edu.cat + diagn+ headache+ diclo+Ibu+ edu.cat*M+ diagn*M+APOE4*M+parac*M+ (1|ID), family="nbinom1", data=ADASdata)

No.Paracet.ADAS<- glmmadmb(neg.b.ADAS~M+AGE+ APOE4+Gender+  edu.cat + diagn+ headache+ diclo+Ibu+ edu.cat*M+ diagn*M+APOE4*M+Gender*M+ (1|ID), family="nbinom1", data=ADASdata)

Paracet.ADAS.Main<- glmmadmb(neg.b.ADAS~parac+M+AGE+ APOE4+Gender+  edu.cat + diagn+ headache+ diclo+Ibu+ edu.cat*M+ diagn*M+APOE4*M+Gender*M+ (1|ID), family="nbinom1", data=ADASdata)

No.Diclofen.ADAS<- glmmadmb(neg.b.ADAS~M+AGE+ APOE4+Gender+  edu.cat + diagn+ headache+ Ibu+ edu.cat*M+ diagn*M+APOE4*M+Gender*M+parac*M+(1|ID), family="nbinom1", data=ADASdata)

No.Headache.ADAS<- glmmadmb(neg.b.ADAS~M+AGE+ APOE4+Gender+  edu.cat + diagn+ diclo+Ibu+ edu.cat*M+ diagn*M+APOE4*M+Gender*M+parac*M+ (1|ID), family="nbinom1", data=ADASdata)

No.Ibuprofen.ADAS<- glmmadmb(neg.b.ADAS~M+AGE+ APOE4+Gender+  edu.cat + diagn+ headache+ diclo+edu.cat*M+ diagn*M+APOE4*M+Gender*M+parac*M+ (1|ID), family="nbinom1", data=ADASdata)
```

```
summary(neg.ADAS)


Call:
glmmadmb(formula = neg.b.ADAS ~ M + AGE + APOE4 + Gender + edu.cat +
    diagn + headache + diclo + Ibu + edu.cat * M + diagn * M +
    APOE4 * M + Gender * M + parac * M + (1 | ID), data = ADASdata,
    family = "nbinom1")

AIC: 72127

Coefficients:
                    Estimate Std. Error z value Pr(>|z|)
(Intercept)         2.991271   0.032483   92.09  < 2e-16 ***
M                   0.007364   0.000339   21.75  < 2e-16 ***
AGE                 0.013316   0.001562    8.52  < 2e-16 ***
APOE41              0.095729   0.024424    3.92  8.9e-05 ***
APOE42              0.149163   0.038638    3.86  0.00011 ***
GenderMale          0.106536   0.023147    4.60  4.2e-06 ***
edu.cat2tertiary    0.104722   0.028117    3.72  0.00020 ***
edu.cat3mid         0.148404   0.031842    4.66  3.2e-06 ***
edu.cat4early       0.180975   0.033458    5.41  6.3e-08 ***
diagn2EMCI          0.357465   0.035112   10.18  < 2e-16 ***
diagn3LMCI          0.771174   0.030017   25.69  < 2e-16 ***
diagn4AD            1.247515   0.034807   35.84  < 2e-16 ***
headache           -0.087110   0.039032   -2.23  0.02563 *
diclo              -0.224372   0.080266   -2.80  0.00518 **
Ibu                -0.093702   0.029852   -3.14  0.00170 **
parac              -0.035014   0.025971   -1.35  0.17759
M:edu.cat2tertiary -0.002137   0.000309   -6.91  4.7e-12 ***
M:edu.cat3mid      -0.001490   0.000348   -4.29  1.8e-05 ***
M:edu.cat4early    -0.001348   0.000361   -3.74  0.00019 ***
M:diagn2EMCI       -0.001629   0.000409   -3.98  6.8e-05 ***
M:diagn3LMCI        0.000210   0.000286    0.74  0.46177
M:diagn4AD          0.004483   0.000726    6.17  6.8e-10 ***
M:APOE41            0.002980   0.000263   11.31  < 2e-16 ***
M:APOE42            0.004757   0.000448   10.61  < 2e-16 ***
M:GenderMale       -0.002098   0.000255   -8.22  < 2e-16 ***
M:parac             0.000555   0.000251    2.21  0.02703 *
---
Signif. codes:  0 '***' 0.001 '**' 0.01 '*' 0.05 '.' 0.1 ' ' 1

Number of observations: total=8770, ID=1618
Random effect variance(s):
Group=ID
            Variance StdDev
(Intercept)   0.1693 0.4114

Negative binomial dispersion parameter: 3.1196 (std. err.: 0.052844)

Log-likelihood: -36035.5

Model<-c("Main effect of Age", "Main effect of APOE4", "Main effect of
Education", "Main effect of Diagnosis","Main effect of Gender", "Main effect of
Diclofenac","Main effect of Headache","Main effect of Ibuprofen","Main effect of
Paracetamol","Interaction effect of Education","Interaction effect of
Diagnosis","Interaction effect of APOE4","Interaction effect of
Gender","Interaction effect of Paracetamol")
```

```
M.age<-anova(neg.ADAS, No.Age.ADAS)
M.APOE4<-anova(APOE4.ADAS.Main, No.APOE4.ADAS)
M.Education<-anova(No.Education.ADAS, Education.ADAS.Main)
M.Diagn<-anova(Diagn.ADAS.Main, No.Diagn.ADAS)
M.Gender<-anova(No.Gender.ADAS, Gender.ADAS.Main)
M.Diclofenac<-anova(neg.ADAS, No.Diclofen.ADAS)
M.Headache<-anova(neg.ADAS, No.Headache.ADAS)
M.Ibuprofen<-anova(neg.ADAS, No.Ibuprofen.ADAS)
M.Paracetamol<-anova(Paracet.ADAS.Main, No.Paracet.ADAS)


I.Education<-anova(neg.ADAS, Education.ADAS.Main)
I.Diagnosis<-anova(neg.ADAS, Diagn.ADAS.Main)
I.APOE4<-anova(neg.ADAS, APOE4.ADAS.Main)
I.Gender<-anova(neg.ADAS, Gender.ADAS.Main)
I.Paracetamol<-anova(neg.ADAS, Paracet.ADAS.Main)



ADAS.PValue<-
rbind(M.age[2,3:5],M.APOE4[2,3:5],M.Education[2,3:5],M.Diagn[2,3:5],M.Gender[2,3
:5],M.Diclofenac[2,3:5],M.Headache[2,3:5],M.Ibuprofen[2,3:5],M.Paracetamol[2,3:5
],I.Education[2,3:5],I.Diagnosis[2,3:5],I.APOE4[2,3:5],I.Gender[2,3:5],I.Paracet
amol[2,3:5])

ADAS.Final.model.dataframe<-data.frame(Model,ADAS.PValue)
ADAS.model.final<-neg.ADAS
print(ADAS.Final.model.dataframe)

                                Model Df Deviance     Pr..Chi.
2                   Main effect of Age  1     71.4 0.000000e+00
21                Main effect of APOE4  2     63.6 1.543210e-14
22            Main effect of Education  3     26.2 8.660640e-06
23            Main effect of Diagnosis  3   1124.4 0.000000e+00
24               Main effect of Gender  1      7.4 6.522388e-03
25           Main effect of Diclofenac  1      7.8 5.224623e-03
26             Main effect of Headache  1      5.0 2.534732e-02
27            Main effect of Ibuprofen  1      9.8 1.745119e-03
28          Main effect of Paracetamol  1      0.8 3.710934e-01
29     Interaction effect of Education  3     50.6 5.952605e-11
210    Interaction effect of Diagnosis  3     65.4 4.118927e-14
211        Interaction effect of APOE4  2    186.2 0.000000e+00
212       Interaction effect of Gender  1     67.0 2.220446e-16
213 Interaction effect of Paracetamol  1      4.8 2.845974e-02

print(summary(ADAS.model.final))


Call:
glmmadmb(formula = neg.b.ADAS ~ M + AGE + APOE4 + Gender + edu.cat +
    diagn + headache + diclo + Ibu + edu.cat * M + diagn * M +
    APOE4 * M + Gender * M + parac * M + (1 | ID), data = ADASdata,
    family = "nbinom1")

AIC: 72127

Coefficients:
                    Estimate Std. Error z value Pr(>|z|)
(Intercept)         2.991271   0.032483   92.09  < 2e-16 ***
M                   0.007364   0.000339   21.75  < 2e-16 ***
```

```
AGE                  0.013316   0.001562    8.52  < 2e-16 ***
APOE41               0.095729   0.024424    3.92  8.9e-05 ***
APOE42               0.149163   0.038638    3.86  0.00011 ***
GenderMale           0.106536   0.023147    4.60  4.2e-06 ***
edu.cat2tertiary     0.104722   0.028117    3.72  0.00020 ***
edu.cat3mid          0.148404   0.031842    4.66  3.2e-06 ***
edu.cat4early        0.180975   0.033458    5.41  6.3e-08 ***
diagn2EMCI           0.357465   0.035112   10.18  < 2e-16 ***
diagn3LMCI           0.771174   0.030017   25.69  < 2e-16 ***
diagn4AD             1.247515   0.034807   35.84  < 2e-16 ***
headache            -0.087110   0.039032   -2.23  0.02563 *
diclo               -0.224372   0.080266   -2.80  0.00518 **
Ibu                 -0.093702   0.029852   -3.14  0.00170 **
parac               -0.035014   0.025971   -1.35  0.17759
M:edu.cat2tertiary -0.002137   0.000309   -6.91  4.7e-12 ***
M:edu.cat3mid       -0.001490   0.000348   -4.29  1.8e-05 ***
M:edu.cat4early     -0.001348   0.000361   -3.74  0.00019 ***
M:diagn2EMCI        -0.001629   0.000409   -3.98  6.8e-05 ***
M:diagn3LMCI         0.000210   0.000286    0.74  0.46177
M:diagn4AD           0.004483   0.000726    6.17  6.8e-10 ***
M:APOE41             0.002980   0.000263   11.31  < 2e-16 ***
M:APOE42             0.004757   0.000448   10.61  < 2e-16 ***
M:GenderMale        -0.002098   0.000255   -8.22  < 2e-16 ***
M:parac              0.000555   0.000251    2.21  0.02703 *
---
Signif. codes:  0 '***' 0.001 '**' 0.01 '*' 0.05 '.' 0.1 ' ' 1

Number of observations: total=8770, ID=1618
Random effect variance(s):
Group=ID
            Variance StdDev
(Intercept)   0.1693 0.4114

Negative binomial dispersion parameter: 3.1196 (std. err.: 0.052844)

Log-likelihood: -36035.5
```

8.17. *Evaluating the progession and main-effects of each pain medication*

```
neg.a.base<- glmmadmb(neg.b.ADAS~ AGE + APOE4  + M + edu.cat + diagn+ headache+
diagn*M +APOE4*M + Gender*M+ edu.cat*M + (1|ID), family="nbinom1",
data=ADASdata)

neg.a.M.aspirin<- glmmadmb(neg.b.ADAS~ AGE + APOE4  + M + edu.cat + diagn+
headache+ diagn*M +APOE4*M + Gender*M+ edu.cat*M + aspirin+ (1|ID),
family="nbinom1", data=ADASdata)
neg.a.M.celecoxib<- glmmadmb(neg.b.ADAS~ AGE + APOE4  + M + edu.cat + diagn+
headache+ diagn*M +APOE4*M + Gender*M+ edu.cat*M + celex+ (1|ID),
family="nbinom1", data=ADASdata)
neg.a.M.diclofenac<- glmmadmb(neg.b.ADAS~ AGE + APOE4  + M + edu.cat + diagn+
headache+ diagn*M +APOE4*M + Gender*M+ edu.cat*M +diclo+ (1|ID),
family="nbinom1", data=ADASdata)
neg.a.M.ibuprofen<- glmmadmb(neg.b.ADAS~ AGE + APOE4  + M + edu.cat + diagn+
headache+ diagn*M +APOE4*M + Gender*M+ edu.cat*M +Ibu+ (1|ID), family="nbinom1",
data=ADASdata)
neg.a.M.naproxen<- glmmadmb(neg.b.ADAS~ AGE + APOE4  + M + edu.cat + diagn+
headache+ diagn*M +APOE4*M + Gender*M+ edu.cat*M +naprox+ (1|ID),
family="nbinom1", data=ADASdata)
neg.a.M.paracetamol<- glmmadmb(neg.b.ADAS~ AGE + APOE4  + M + edu.cat + diagn+
```

```
headache+ diagn*M +APOE4*M + Gender*M+ edu.cat*M + parac+ (1|ID),
family="nbinom1", data=ADASdata)
neg.a.I.aspirin<- glmmadmb(neg.b.ADAS~ AGE + APOE4  + M + edu.cat + diagn+
headache+ diagn*M +APOE4*M + Gender*M+ edu.cat*M +aspirin*M+ (1|ID),
family="nbinom1", data=ADASdata)
neg.a.I.celecoxib<- glmmadmb(neg.b.ADAS~ AGE + APOE4  + M + edu.cat + diagn+
headache+ diagn*M +APOE4*M + Gender*M+ edu.cat*M +celex*M+ (1|ID),
family="nbinom1", data=ADASdata)
neg.a.I.diclofenac<- glmmadmb(neg.b.ADAS~ AGE + APOE4  + M + edu.cat + diagn+
headache+ diagn*M +APOE4*M + Gender*M+ edu.cat*M +diclo*M+ (1|ID),
family="nbinom1", data=ADASdata)
neg.a.I.ibuprofen<- glmmadmb(neg.b.ADAS~ AGE + APOE4  + M + edu.cat + diagn+
headache+ diagn*M +APOE4*M + Gender*M+ edu.cat*M +Ibu*M+ (1|ID),
family="nbinom1", data=ADASdata)
neg.a.I.naproxen<- glmmadmb(neg.b.ADAS~ AGE + APOE4  + M + edu.cat + diagn+
headache+ diagn*M +APOE4*M + Gender*M+ edu.cat*M + naprox*M+ (1|ID),
family="nbinom1", data=ADASdata)
neg.a.I.paracetamol<- glmmadmb(neg.b.ADAS~ AGE + APOE4  + M + edu.cat + diagn+
headache+ diagn*M +APOE4*M + Gender*M+ edu.cat*M +parac*M+ (1|ID),
family="nbinom1", data=ADASdata)

anova.M.aspirin<-anova(neg.a.M.aspirin, neg.a.base)
anova.M.celecoxib<-anova(neg.a.M.celecoxib, neg.a.base)
anova.M.diclofenac<-anova(neg.a.M.diclofenac, neg.a.base)
anova.M.ibuprofen<-anova(neg.a.M.ibuprofen, neg.a.base)
anova.M.naproxen<-anova(neg.a.M.naproxen, neg.a.base)
anova.M.paracetamol<-anova(neg.a.M.paracetamol, neg.a.base)

anova.I.aspirin<-anova(neg.a.M.aspirin, neg.a.I.aspirin)
anova.I.celecoxib<-anova(neg.a.M.celecoxib, neg.a.I.celecoxib)
anova.I.diclofenac<-anova(neg.a.M.diclofenac, neg.a.I.diclofenac)
anova.I.ibuprofen<-anova(neg.a.M.ibuprofen, neg.a.I.ibuprofen)
anova.I.naproxen<-anova(neg.a.M.naproxen, neg.a.I.naproxen)
anova.I.paracetamol<-anova(neg.a.M.paracetamol, neg.a.I.paracetamol)
Model.Painrelief.ADAS<- c("Main effect of Aspirin", "Main effect of Celecoxib",
"Main effect of Diclofenac", "Main effect of Ibuprofen", "Main effect of
Naproxen","Main effect of Paracetamol","Interaction effect of
Aspirin","Interaction effect of Celecoxib","Interaction effect of
Diclofenac","Interaction effect of Ibuprofen","Interaction effect of
Naproxen","Interaction effect of Paracetamol")

ADAS.Painrelief.PValue<-rbind(anova.M.aspirin[2,3:5], anova.M.celecoxib[2,3:5],
anova.M.diclofenac[2,3:5], anova.M.ibuprofen[2,3:5], anova.M.naproxen[2,3:5],
anova.M.paracetamol[2,3:5], anova.I.aspirin[2,3:5], anova.I.celecoxib[2,3:5],
anova.I.diclofenac[2,3:5], anova.I.ibuprofen[2,3:5], anova.I.naproxen[2,3:5],
anova.I.paracetamol[2,3:5])

ADAS.Painrelief.Model.Dataframe<-data.frame(Model.Painrelief.ADAS,
ADAS.Painrelief.PValue)
print(ADAS.Painrelief.Model.Dataframe)

                Model.Painrelief.ADAS Df Deviance    Pr..Chi.
2               Main effect of Aspirin  1      1.2 0.273321678
21            Main effect of Celecoxib  1      0.0 1.000000000
22           Main effect of Diclofenac  1      8.0 0.004677735
23            Main effect of Ibuprofen  1     10.6 0.001130877
24             Main effect of Naproxen  1      0.6 0.438578026
25          Main effect of Paracetamol  1      1.6 0.205903211
26        Interaction effect of Aspirin  1      1.0 0.317310508
```

```
27    Interaction effect of Celecoxib  1      1.0 0.317310508
28   Interaction effect of Diclofenac  1      2.4 0.121335250
29    Interaction effect of Ibuprofen  1      1.0 0.317310508
210    Interaction effect of Naproxen  1      3.6 0.057779571
211 Interaction effect of Paracetamol  1      4.8 0.028459737
```

8.18. *APOE4 and NSAIDs*

There is evidence that NSAIDs interact are only protective in the presence of the APOE4 gene. Therefore, a three way interaction term was investigated.

```
neg.a.APOE.diclo <- glmmadmb(neg.b.ADAS~ AGE + APOE4  + M + edu.cat + diagn+
headache+ diagn*M +APOE4*M + Gender*M+ edu.cat*M +parac*M
+diclo*M+diclo*APOE4+(1|ID), family="nbinom1", data=ADASdata)
summary(neg.a.APOE.diclo)


Call:
glmmadmb(formula = neg.b.ADAS ~ AGE + APOE4 + M + edu.cat + diagn +
    headache + diagn * M + APOE4 * M + Gender * M + edu.cat *
    M + parac * M + diclo * M + diclo * APOE4 + (1 | ID), data = ADASdata,
    family = "nbinom1")

AIC: 72137.2

Coefficients:
                     Estimate Std. Error z value Pr(>|z|)
(Intercept)          2.977934   0.032345   92.07  < 2e-16 ***
AGE                  0.013649   0.001563    8.73  < 2e-16 ***
APOE41               0.095030   0.024640    3.86  0.00011 ***
APOE42               0.148349   0.038937    3.81  0.00014 ***
M                    0.007389   0.000339   21.80  < 2e-16 ***
edu.cat2tertiary     0.102095   0.028178    3.62  0.00029 ***
edu.cat3mid          0.148260   0.031926    4.64  3.4e-06 ***
edu.cat4early        0.175896   0.033545    5.24  1.6e-07 ***
diagn2EMCI           0.359074   0.035214   10.20  < 2e-16 ***
diagn3LMCI           0.773097   0.030125   25.66  < 2e-16 ***
diagn4AD             1.256524   0.034829   36.08  < 2e-16 ***
headache            -0.089474   0.039175   -2.28  0.02237 *
GenderMale           0.105668   0.023215    4.55  5.3e-06 ***
parac               -0.044327   0.025910   -1.71  0.08711 .
diclo               -0.235744   0.108100   -2.18  0.02920 *
M:diagn2EMCI        -0.001665   0.000410   -4.06  4.8e-05 ***
M:diagn3LMCI         0.000181   0.000287    0.63  0.52706
M:diagn4AD           0.004429   0.000727    6.09  1.1e-09 ***
APOE41:M             0.002983   0.000263   11.33  < 2e-16 ***
APOE42:M             0.004828   0.000451   10.72  < 2e-16 ***
M:GenderMale        -0.002077   0.000255   -8.13  4.3e-16 ***
M:edu.cat2tertiary -0.002144   0.000309   -6.94  4.0e-12 ***
M:edu.cat3mid       -0.001528   0.000348   -4.39  1.1e-05 ***
M:edu.cat4early     -0.001299   0.000362   -3.59  0.00033 ***
M:parac              0.000584   0.000252    2.32  0.02020 *
M:diclo             -0.001159   0.000683   -1.70  0.08946 .
APOE41:diclo         0.100350   0.173010    0.58  0.56190
APOE42:diclo         0.171960   0.317000    0.54  0.58750
---
Signif. codes:  0 '***' 0.001 '**' 0.01 '*' 0.05 '.' 0.1 ' ' 1

Number of observations: total=8770, ID=1618
```

```
Random effect variance(s):
Group=ID
            Variance StdDev
(Intercept)   0.1703 0.4126

Negative binomial dispersion parameter: 3.1186 (std. err.: 0.052833)

Log-likelihood: -36038.6

anova(neg.a.I.diclofenac,neg.a.APOE.diclo)

Analysis of Deviance Table

Model 1: neg.b.ADAS ~ AGE + APOE4 + M + edu.cat + diagn + headache + diagn * M +
APOE4 * M + Gender * M + edu.cat * M + diclo * M
Model 2: neg.b.ADAS ~ AGE + APOE4 + M + edu.cat + diagn + headache + diagn * M +
APOE4 * M + Gender * M + edu.cat * M + parac * M + diclo * M + diclo * APOE4
  NoPar LogLik Df Deviance Pr(>Chi)
1    26 -36042
2    30 -36039  4      7.4   0.1162

neg.a.APOE.diclo.M <- glmmadmb(neg.b.ADAS~ AGE + APOE4  + M + edu.cat + diagn+
headache+ diagn*M +APOE4*M + Gender*M+ edu.cat*M +parac*M
+diclo*M+diclo*APOE4*M+(1|ID), family="nbinom1", data=ADASdata)
summary(neg.a.APOE.diclo.M)


Call:
glmmadmb(formula = neg.b.ADAS ~ AGE + APOE4 + M + edu.cat + diagn +
    headache + diagn * M + APOE4 * M + Gender * M + edu.cat *
    M + parac * M + diclo * M + diclo * APOE4 * M + (1 | ID),
    data = ADASdata, family = "nbinom1")

AIC: 72139.4

Coefficients:
                    Estimate Std. Error z value Pr(>|z|)
(Intercept)         2.977624   0.032344   92.06  < 2e-16 ***
AGE                 0.013644   0.001563    8.73  < 2e-16 ***
APOE41              0.096237   0.024653    3.90  9.5e-05 ***
APOE42              0.149489   0.038984    3.83  0.00013 ***
M                   0.007391   0.000339   21.80  < 2e-16 ***
edu.cat2tertiary    0.101862   0.028182    3.61  0.00030 ***
edu.cat3mid         0.147906   0.031927    4.63  3.6e-06 ***
edu.cat4early       0.176107   0.033554    5.25  1.5e-07 ***
diagn2EMCI          0.359109   0.035211   10.20  < 2e-16 ***
diagn3LMCI          0.773070   0.030127   25.66  < 2e-16 ***
diagn4AD            1.256556   0.034827   36.08  < 2e-16 ***
headache           -0.089535   0.039171   -2.29  0.02227 *
GenderMale          0.105457   0.023214    4.54  5.6e-06 ***
parac              -0.044508   0.025911   -1.72  0.08585 .
diclo              -0.207116   0.110200   -1.88  0.06018 .
M:diagn2EMCI       -0.001661   0.000410   -4.06  5.0e-05 ***
M:diagn3LMCI        0.000194   0.000287    0.67  0.50045
M:diagn4AD          0.004449   0.000727    6.12  9.6e-10 ***
APOE41:M            0.002925   0.000267   10.95  < 2e-16 ***
APOE42:M            0.004764   0.000464   10.26  < 2e-16 ***
M:GenderMale       -0.002059   0.000256   -8.05  8.5e-16 ***
M:edu.cat2tertiary -0.002130   0.000311   -6.86  7.0e-12 ***
M:edu.cat3mid      -0.001517   0.000349   -4.35  1.4e-05 ***
```

```
M:edu.cat4early    -0.001323   0.000366   -3.61  0.00030 ***
M:parac             0.000601   0.000253    2.38  0.01739 *
M:diclo            -0.001985   0.000943   -2.11  0.03524 *
APOE41:diclo        0.039314   0.178770    0.22  0.82594
APOE42:diclo        0.129003   0.323360    0.40  0.68993
APOE41:M:diclo      0.002167   0.001630    1.33  0.18356
APOE42:M:diclo      0.001195   0.001745    0.68  0.49336
---
Signif. codes:  0 '***' 0.001 '**' 0.01 '*' 0.05 '.' 0.1 ' ' 1

Number of observations: total=8770, ID=1618
Random effect variance(s):
Group=ID
            Variance StdDev
(Intercept)   0.1702 0.4126

Negative binomial dispersion parameter: 3.118 (std. err.: 0.052824)

Log-likelihood: -36037.7

anova(neg.a.APOE.diclo.M,neg.a.APOE.diclo)

Analysis of Deviance Table

Model 1: neg.b.ADAS ~ AGE + APOE4 + M + edu.cat + diagn + headache + diagn * M +
APOE4 * M + Gender * M + edu.cat * M + parac * M + diclo * M + diclo * APOE4
Model 2: neg.b.ADAS ~ AGE + APOE4 + M + edu.cat + diagn + headache + diagn * M +
APOE4 * M + Gender * M + edu.cat * M + parac * M + diclo * M + diclo * APOE4 * M
  NoPar LogLik Df Deviance Pr(>Chi)
1    30 -36039
2    32 -36038  2      1.8   0.4066

ADASdata$x<-
ADASdata$diclo+ADASdata$naprox+ADASdata$celex+ADASdata$aspirin+ADASdata$Ibu
ADASdata$NSAID<-1*(ADASdata$x>0)

neg.a.APOE.NSAID <- glmmadmb(neg.b.ADAS~ AGE + APOE4  + M + edu.cat + diagn+
headache+ diagn*M +APOE4*M + Gender*M+ edu.cat*M +NSAID*M+NSAID*APOE4+(1|ID),
family="nbinom1", data=ADASdata)
summary(neg.a.APOE.NSAID)


Call:
glmmadmb(formula = neg.b.ADAS ~ AGE + APOE4 + M + edu.cat + diagn +
    headache + diagn * M + APOE4 * M + Gender * M + edu.cat *
    M + NSAID * M + NSAID * APOE4 + (1 | ID), data = ADASdata,
    family = "nbinom1")

AIC: 72145

Coefficients:
                    Estimate Std. Error z value Pr(>|z|)
(Intercept)         2.986999   0.038079   78.44  < 2e-16 ***
AGE                 0.013518   0.001566    8.63  < 2e-16 ***
APOE41              0.099274   0.039469    2.52  0.01190 *
APOE42              0.167317   0.061627    2.71  0.00663 **
M                   0.007687   0.000399   19.28  < 2e-16 ***
edu.cat2tertiary    0.101319   0.028226    3.59  0.00033 ***
edu.cat3mid         0.148455   0.031949    4.65  3.4e-06 ***
edu.cat4early       0.176128   0.033600    5.24  1.6e-07 ***
```

```
diagn2EMCI           0.366031   0.035237   10.39  < 2e-16 ***
diagn3LMCI           0.774956   0.030142   25.71  < 2e-16 ***
diagn4AD             1.255280   0.035095   35.77  < 2e-16 ***
headache            -0.096176   0.039062   -2.46  0.01381 *
GenderMale           0.114436   0.023295    4.91  9.0e-07 ***
NSAID               -0.049279   0.032735   -1.51  0.13222
M:diagn2EMCI        -0.001704   0.000409   -4.17  3.0e-05 ***
M:diagn3LMCI         0.000197   0.000287    0.69  0.49091
M:diagn4AD           0.004341   0.000731    5.94  2.9e-09 ***
APOE41:M             0.002981   0.000264   11.30  < 2e-16 ***
APOE42:M             0.004857   0.000447   10.86  < 2e-16 ***
M:GenderMale        -0.002099   0.000258   -8.15  3.6e-16 ***
M:edu.cat2tertiary -0.002128   0.000309   -6.88  5.9e-12 ***
M:edu.cat3mid       -0.001448   0.000348   -4.17  3.1e-05 ***
M:edu.cat4early     -0.001345   0.000363   -3.71  0.00021 ***
M:NSAID             -0.000149   0.000308   -0.49  0.62718
APOE41:NSAID        -0.001197   0.048427   -0.02  0.98029
APOE42:NSAID        -0.022182   0.075307   -0.29  0.76833
---
Signif. codes:  0 '***' 0.001 '**' 0.01 '*' 0.05 '.' 0.1 ' ' 1

Number of observations: total=8770, ID=1618
Random effect variance(s):
Group=ID
            Variance StdDev
(Intercept)   0.1709 0.4134

Negative binomial dispersion parameter: 3.1215 (std. err.: 0.05289)

Log-likelihood: -36044.5

neg.a.NSAID<- glmmadmb(neg.b.ADAS~ AGE + APOE4  + M + edu.cat + diagn+ headache+
diagn*M +APOE4*M + Gender*M+ edu.cat*M +NSAID*M+(1|ID), family="nbinom1",
data=ADASdata)
summary(neg.a.NSAID)


Call:
glmmadmb(formula = neg.b.ADAS ~ AGE + APOE4 + M + edu.cat + diagn +
    headache + diagn * M + APOE4 * M + Gender * M + edu.cat *
    M + NSAID * M + (1 | ID), data = ADASdata, family = "nbinom1")

AIC: 72141.2

Coefficients:
                    Estimate Std. Error z value Pr(>|z|)
(Intercept)          2.989221   0.034804   85.89  < 2e-16 ***
AGE                  0.013502   0.001563    8.64  < 2e-16 ***
APOE41               0.098411   0.024501    4.02  5.9e-05 ***
APOE42               0.153218   0.038792    3.95  7.8e-05 ***
M                    0.007685   0.000399   19.28  < 2e-16 ***
edu.cat2tertiary     0.101110   0.028217    3.58  0.00034 ***
edu.cat3mid          0.148399   0.031943    4.65  3.4e-06 ***
edu.cat4early        0.175838   0.033573    5.24  1.6e-07 ***
diagn2EMCI           0.365844   0.035231   10.38  < 2e-16 ***
diagn3LMCI           0.774804   0.030139   25.71  < 2e-16 ***
diagn4AD             1.255459   0.035086   35.78  < 2e-16 ***
headache            -0.096429   0.039039   -2.47  0.01351 *
GenderMale           0.114264   0.023285    4.91  9.2e-07 ***
```

```
NSAID              -0.052173   0.023694   -2.20  0.02767 *
M:diagn2EMCI       -0.001704   0.000409   -4.17  3.0e-05 ***
M:diagn3LMCI        0.000198   0.000287    0.69  0.48999
M:diagn4AD          0.004341   0.000731    5.94  2.9e-09 ***
APOE41:M            0.002981   0.000264   11.30  < 2e-16 ***
APOE42:M            0.004851   0.000447   10.86  < 2e-16 ***
M:GenderMale       -0.002099   0.000258   -8.15  3.6e-16 ***
M:edu.cat2tertiary -0.002128   0.000309   -6.88  5.9e-12 ***
M:edu.cat3mid      -0.001448   0.000348   -4.17  3.1e-05 ***
M:edu.cat4early    -0.001345   0.000363   -3.71  0.00021 ***
M:NSAID            -0.000147   0.000307   -0.48  0.63172
---
Signif. codes:  0 '***' 0.001 '**' 0.01 '*' 0.05 '.' 0.1 ' ' 1

Number of observations: total=8770, ID=1618
Random effect variance(s):
Group=ID
            Variance StdDev
(Intercept)   0.1709 0.4134

Negative binomial dispersion parameter: 3.1215 (std. err.: 0.05289)

Log-likelihood: -36044.6

anova(neg.a.NSAID,neg.a.APOE.NSAID)

Analysis of Deviance Table

Model 1: neg.b.ADAS ~ AGE + APOE4 + M + edu.cat + diagn + headache + diagn * M +
APOE4 * M + Gender * M + edu.cat * M + NSAID * M
Model 2: neg.b.ADAS ~ AGE + APOE4 + M + edu.cat + diagn + headache + diagn * M +
APOE4 * M + Gender * M + edu.cat * M + NSAID * M + NSAID * APOE4
  NoPar LogLik Df Deviance Pr(>Chi)
1    26 -36045
2    28 -36044  2      0.2   0.9048

neg.a.NSAID.APOE.M<- glmmadmb(neg.b.ADAS~ AGE + APOE4  + M + edu.cat + diagn+
headache+ diagn*M +APOE4*M + Gender*M+ edu.cat*M +NSAID*M*APOE4+(1|ID),
family="nbinom1", data=ADASdata)
summary(neg.a.NSAID.APOE.M)


Call:
glmmadmb(formula = neg.b.ADAS ~ AGE + APOE4 + M + edu.cat + diagn +
    headache + diagn * M + APOE4 * M + Gender * M + edu.cat *
    M + NSAID * M * APOE4 + (1 | ID), data = ADASdata, family = "nbinom1")

AIC: 72148.2

Coefficients:
                    Estimate Std. Error z value Pr(>|z|)
(Intercept)         2.983185   0.038368   77.75  < 2e-16 ***
AGE                 0.013512   0.001566    8.63  < 2e-16 ***
APOE41              0.106853   0.040400    2.64  0.00817 **
APOE42              0.171086   0.062904    2.72  0.00653 **
M                   0.007887   0.000464   16.99  < 2e-16 ***
edu.cat2tertiary    0.101578   0.028236    3.60  0.00032 ***
edu.cat3mid         0.148580   0.031961    4.65  3.3e-06 ***
edu.cat4early       0.176559   0.033614    5.25  1.5e-07 ***
diagn2EMCI          0.366164   0.035249   10.39  < 2e-16 ***
```

```
diagn3LMCI          0.774838   0.030153   25.70  < 2e-16 ***
diagn4AD            1.255284   0.035108   35.75  < 2e-16 ***
headache           -0.096178   0.039075   -2.46  0.01384 *
GenderMale          0.114450   0.023303    4.91  9.0e-07 ***
NSAID              -0.044415   0.033249   -1.34  0.18160
M:diagn2EMCI       -0.001710   0.000409   -4.18  2.9e-05 ***
M:diagn3LMCI        0.000218   0.000288    0.76  0.44808
M:diagn4AD          0.004360   0.000733    5.95  2.7e-09 ***
APOE41:M            0.002531   0.000573    4.41  1.0e-05 ***
APOE42:M            0.004639   0.001049    4.42  9.8e-06 ***
M:GenderMale       -0.002100   0.000258   -8.15  3.5e-16 ***
M:edu.cat2tertiary -0.002139   0.000309   -6.91  4.7e-12 ***
M:edu.cat3mid      -0.001454   0.000348   -4.18  2.9e-05 ***
M:edu.cat4early    -0.001372   0.000364   -3.77  0.00016 ***
M:NSAID            -0.000394   0.000422   -0.93  0.35027
APOE41:NSAID       -0.011610   0.049852   -0.23  0.81585
APOE42:NSAID       -0.027171   0.077158   -0.35  0.72473
APOE41:M:NSAID      0.000565   0.000638    0.88  0.37628
APOE42:M:NSAID      0.000258   0.001144    0.23  0.82180
---
Signif. codes:  0 '***' 0.001 '**' 0.01 '*' 0.05 '.' 0.1 ' ' 1

Number of observations: total=8770, ID=1618
Random effect variance(s):
Group=ID
            Variance StdDev
(Intercept)    0.171 0.4135

Negative binomial dispersion parameter: 3.1206 (std. err.: 0.05288)

Log-likelihood: -36044.1

anova(neg.a.NSAID.APOE.M,neg.a.APOE.NSAID)

Analysis of Deviance Table

Model 1: neg.b.ADAS ~ AGE + APOE4 + M + edu.cat + diagn + headache + diagn * M +
APOE4 * M + Gender * M + edu.cat * M + NSAID * M + NSAID * APOE4
Model 2: neg.b.ADAS ~ AGE + APOE4 + M + edu.cat + diagn + headache + diagn * M +
APOE4 * M + Gender * M + edu.cat * M + NSAID * M * APOE4
  NoPar LogLik Df Deviance Pr(>Chi)
1    28 -36044
2    30 -36044  2      0.8   0.6703

AIC(neg.a.APOE.diclo)

[1] 72137.2

AIC(neg.a.APOE.diclo.M)

[1] 72139.4

AIC(neg.a.APOE.NSAID)

[1] 72145

AIC(neg.a.NSAID)

[1] 72141.2

AIC(neg.a.NSAID.APOE.M)
```

```
[1] 72148.2

AIC(neg.ADAS)

[1] 72127
```

8.18.1. *General NSAID use is not associated with slower cognitive decline*

```
neg.a.NSAID.main.effect<- glmmadmb(neg.b.ADAS~ AGE + APOE4  + M + edu.cat +
diagn+ headache+ diagn*M +APOE4*M + Gender*M+ edu.cat*M +NSAID+(1|ID),
family="nbinom1", data=ADASdata)
anova(neg.a.NSAID.main.effect, neg.a.NSAID)

Analysis of Deviance Table

Model 1: neg.b.ADAS ~ AGE + APOE4 + M + edu.cat + diagn + headache + diagn * M +
APOE4 * M + Gender * M + edu.cat * M + NSAID
Model 2: neg.b.ADAS ~ AGE + APOE4 + M + edu.cat + diagn + headache + diagn * M +
APOE4 * M + Gender * M + edu.cat * M + NSAID * M
  NoPar LogLik Df Deviance Pr(>Chi)
1    25 -36045
2    26 -36045  1      0.2   0.6547
```

8.18.2. *General NSAID use is associated with a fixed effect on cognitive performance*

```
anova(neg.a.NSAID.main.effect, neg.a.base)

Analysis of Deviance Table

Model 1: neg.b.ADAS ~ AGE + APOE4 + M + edu.cat + diagn + headache + diagn * M +
APOE4 * M + Gender * M + edu.cat * M
Model 2: neg.b.ADAS ~ AGE + APOE4 + M + edu.cat + diagn + headache + diagn * M +
APOE4 * M + Gender * M + edu.cat * M + NSAID
  NoPar LogLik Df Deviance Pr(>Chi)
1    24 -36048
2    25 -36045  1      5.6  0.01796 *
---
Signif. codes:  0 '***' 0.001 '**' 0.01 '*' 0.05 '.' 0.1 ' ' 1
```

8.19. *Month as a factor*

Including month as a factor was not possible due to the extremely large degrees of freedom that would be required in the model. This, and the fact that the error distributions showed no sign of inappropriate fitting, suggest that month modelled as a numerical variable is appropriate.

```
try(neg.a.M.factor <- glmmadmb(neg.b.ADAS~ AGE + APOE4  + as.factor(M) + edu.cat
+ diagn+ headache+ diagn*as.factor(M) +APOE4*as.factor(M) + Gender*as.factor(M)+
edu.cat*as.factor(M) +parac*as.factor(M) +diclo*as.factor(M)+(1|ID),
family="nbinom1", data=ADASdata))

try(neg.a.M.factor.simple <- glmmadmb(neg.b.ADAS~ AGE + APOE4  + as.factor(M) +
edu.cat + diagn+ headache+ + diagn*as.factor(M) +diclo*as.factor(M)+(1|ID),
family="nbinom1", data=ADASdata))
```

8.20. *Years education as a numerical variable*

```
ADASdata$Yrs.edu<-as.numeric(ADASdata$Yrs.edu)
try(neg.a.Yrs.edu<- glmmadmb(neg.b.ADAS~ AGE + APOE4  + M + Yrs.edu + diagn+
```

```
headache+ diagn*M +APOE4*M + Gender*M+ Yrs.edu*M +parac*M +(1|ID),
family="nbinom1", data=ADASdata))
try(anova(neg.a.Yrs.edu,neg.ADAS))

Analysis of Deviance Table

Model 1: neg.b.ADAS ~ AGE + APOE4 + M + Yrs.edu + diagn + headache + diagn * M +
APOE4 * M + Gender * M + Yrs.edu * M + parac * M
Model 2: neg.b.ADAS ~ M + AGE + APOE4 + Gender + edu.cat + diagn + headache +
diclo + Ibu + edu.cat * M + diagn * M + APOE4 * M + Gender * M + parac * M
  NoPar LogLik Df Deviance  Pr(>Chi)
1    22 -36057
2    28 -36036  6       43 1.166e-07 ***
---
Signif. codes:  0 '***' 0.001 '**' 0.01 '*' 0.05 '.' 0.1 ' ' 1
```

8.21. *looking at the distribution of the residuals in the final model, for each variable separately*

Residuals within each explanitory variables collectively and within each individual show no trends and are homoskedastic centred around zero. Therefore this model is accurate and will be used to investiage the effects of the input variables (including pain medicationss) going forward.

```
augDat <- data.frame(ADASdata,resid=residuals(neg.ADAS,type="pearson"),
fitted=fitted(neg.ADAS))
ggplot(augDat,aes(x=Gender,y=resid,group=ID))+geom_boxplot()+coord_flip()
```

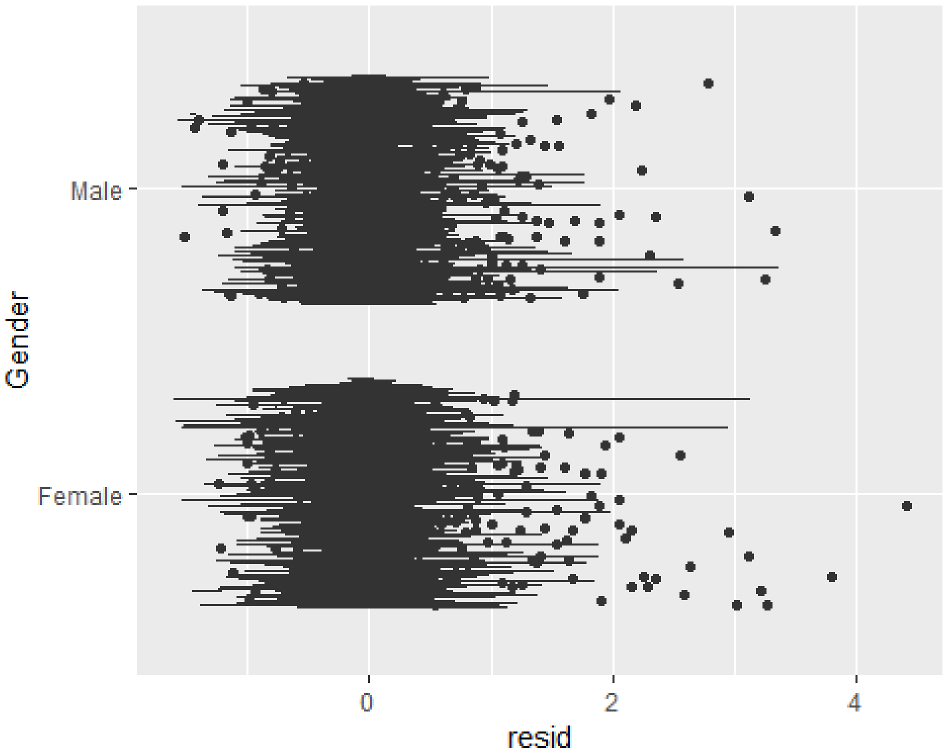


```
ggplot(augDat,aes(x=AGE,y=resid,group=ID))+geom_boxplot()+coord_flip()
```

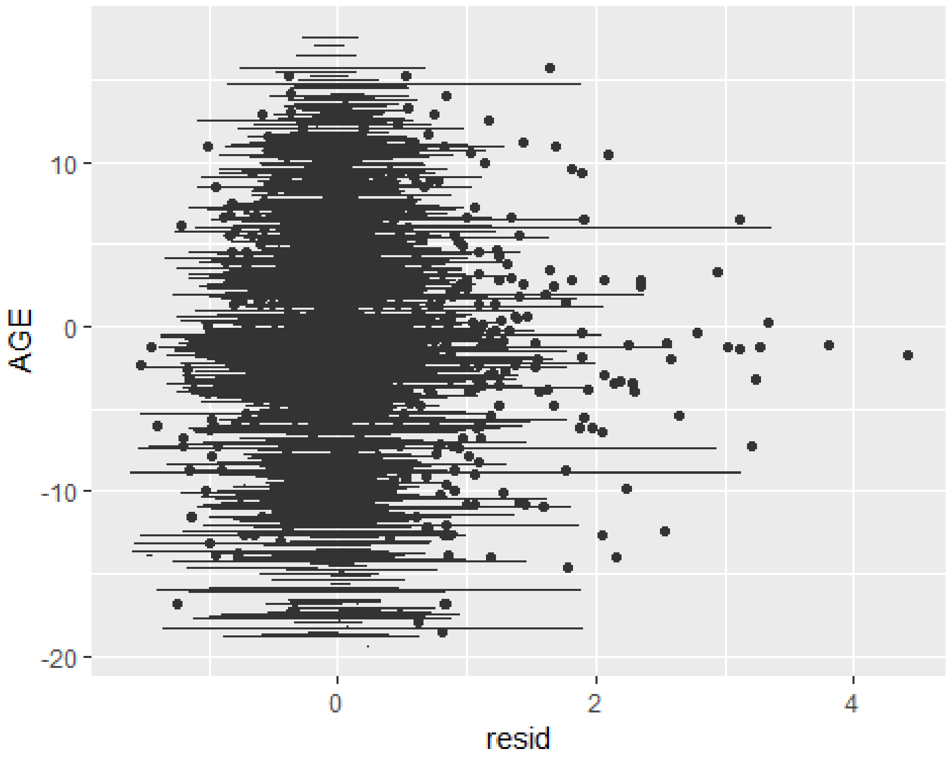


```
ggplot(augDat,aes(x=edu.cat,y=resid,group=ID))+geom_boxplot()+coord_flip()
```

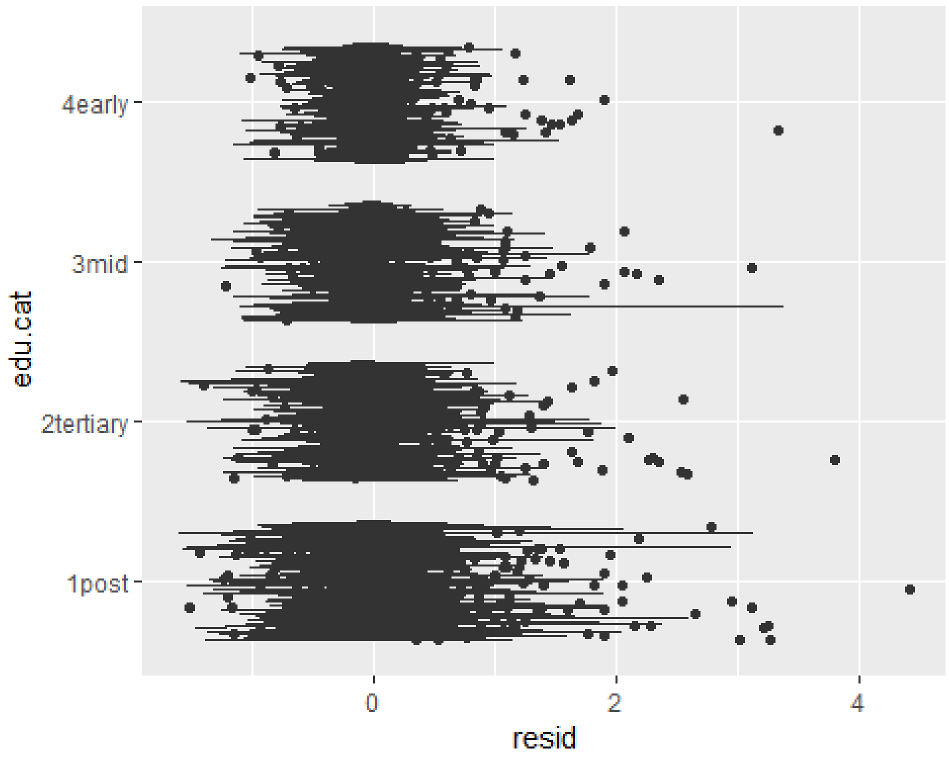


```
ggplot(augDat,aes(x=diagn,y=resid,group=ID))+geom_boxplot()+coord_flip()
```

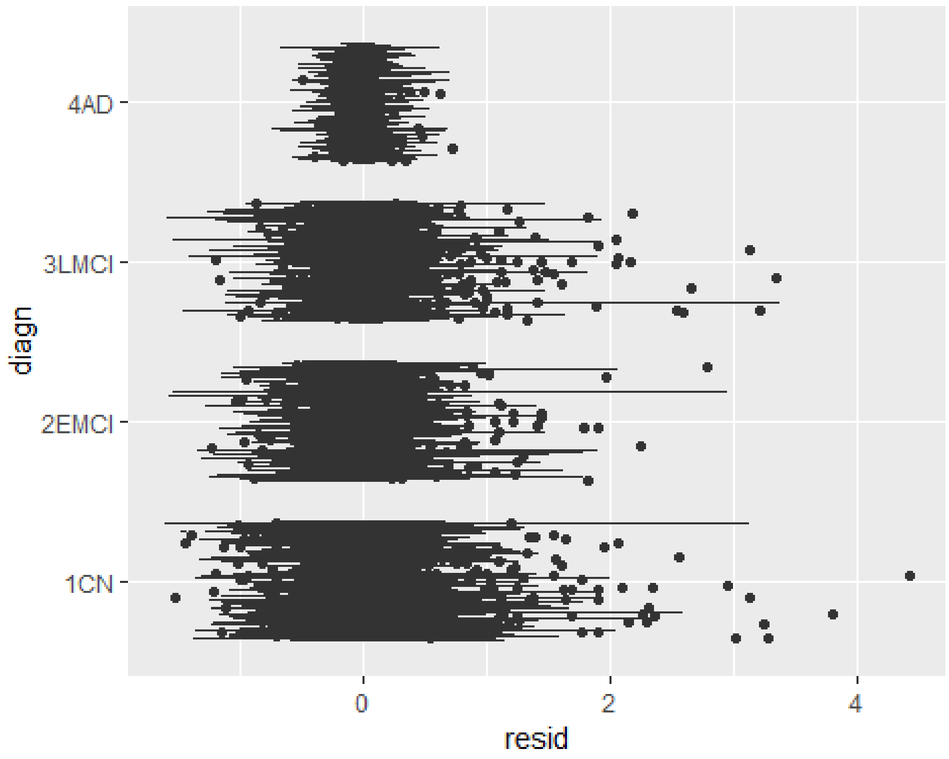


```
ggplot(augDat,aes(x=APOE4,y=resid,group=ID))+geom_boxplot()+coord_flip()
```

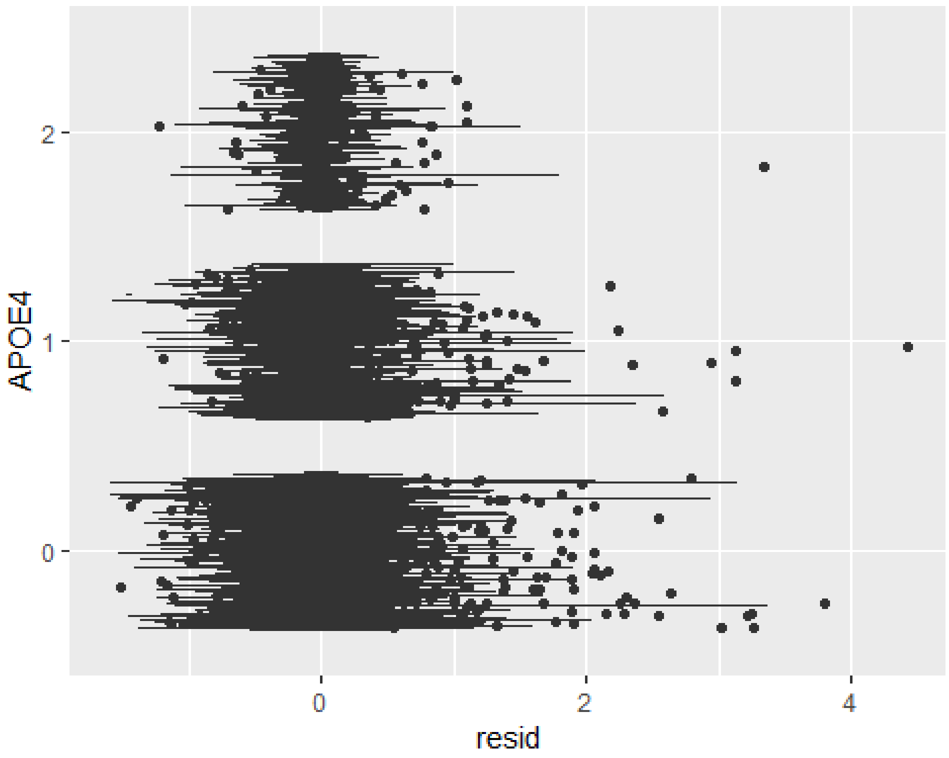


```
ggplot(augDat,aes(x=M,y=resid,group=ID))+geom_boxplot()+coord_flip()
```

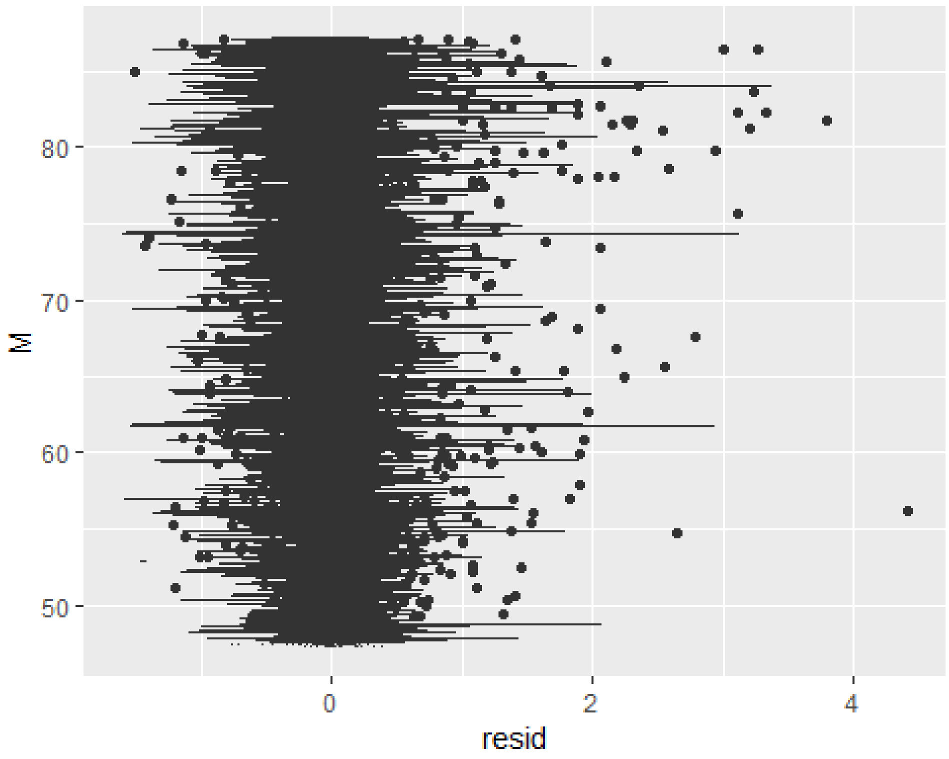


```
ggplot(augDat,aes(x=diclo,y=resid,group=ID))+geom_boxplot()+coord_flip()
```

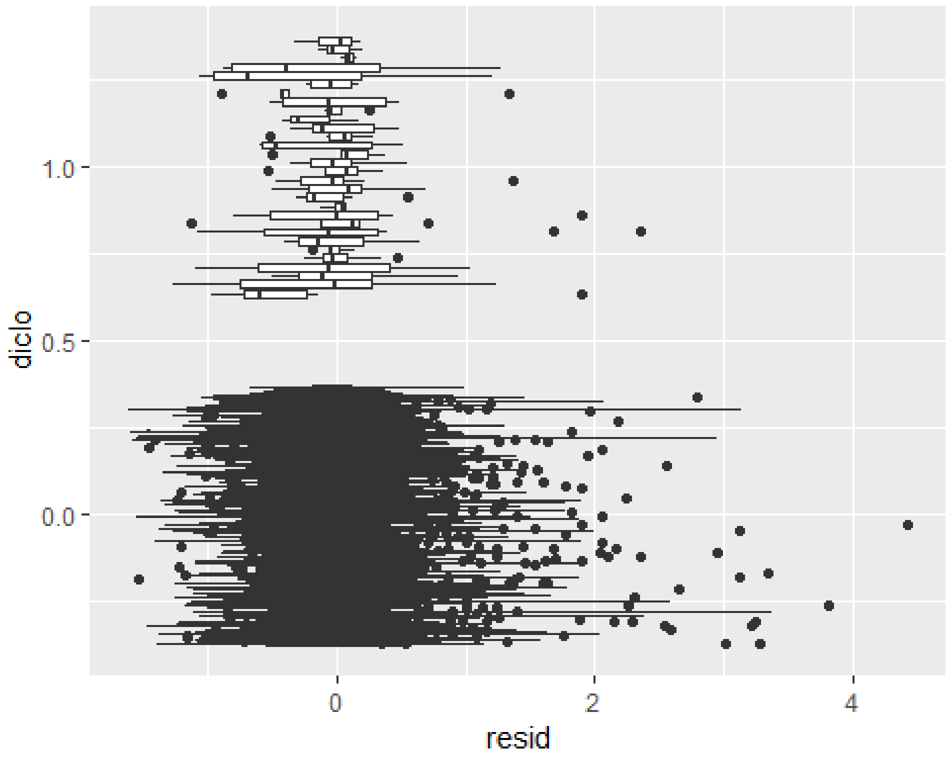


```
ggplot(augDat,aes(x=parac,y=resid,group=ID))+geom_boxplot()+coord_flip()
```

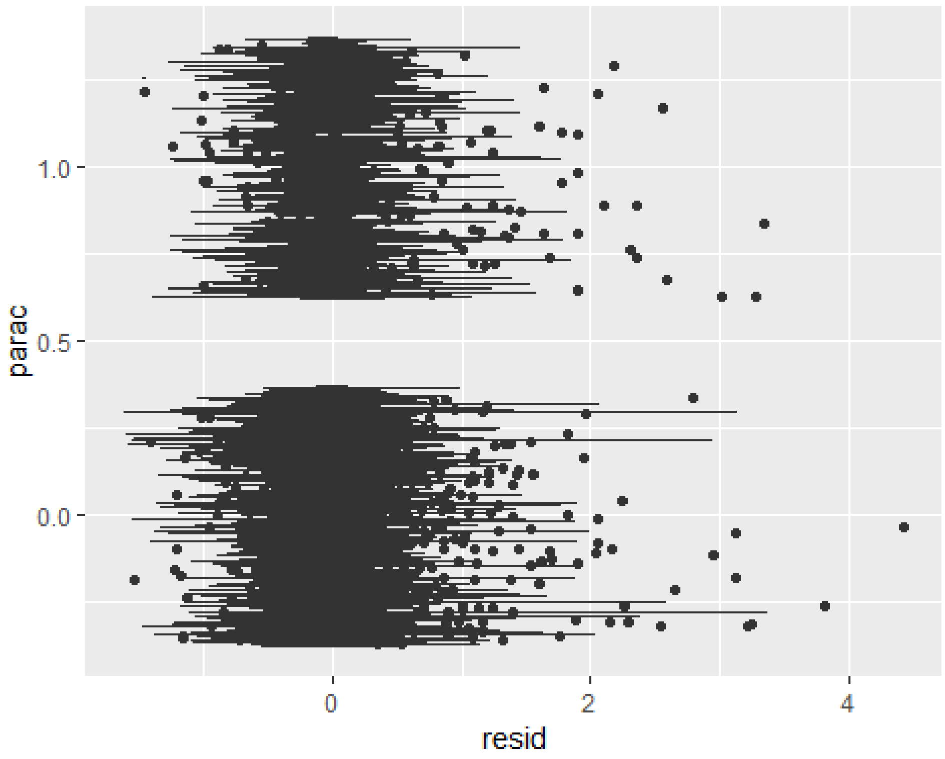


```
ggplot(augDat,aes(x=Gender,y=resid))+geom_boxplot()+coord_flip()
```

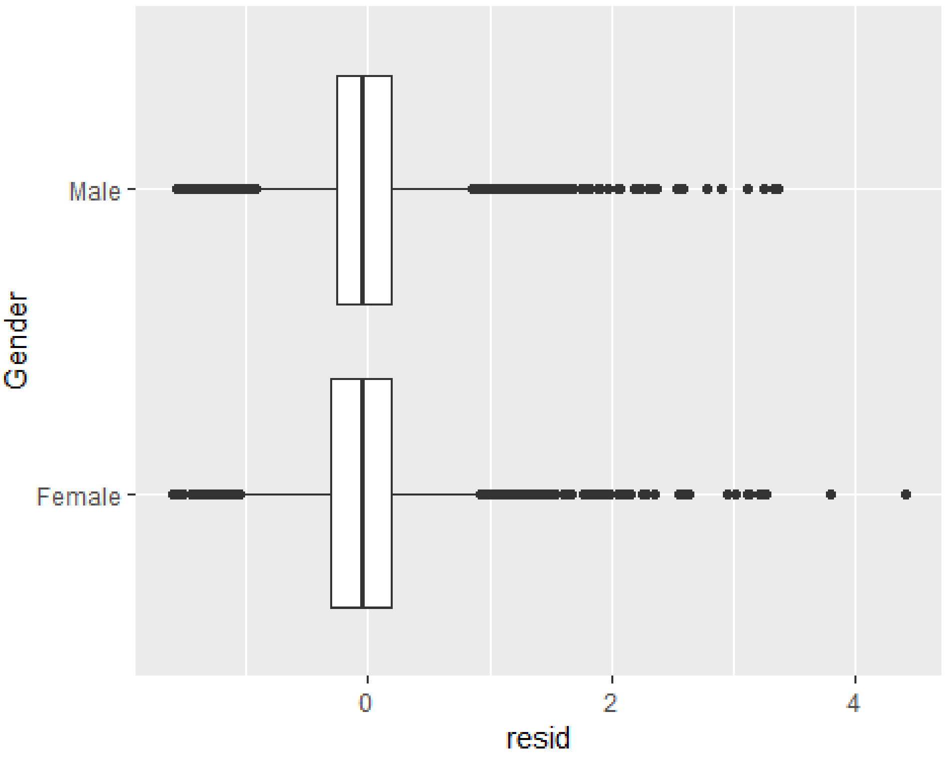


```
ggplot(augDat,aes(x=AGE,y=resid))+geom_point()+coord_flip()+geom_smooth(method=l
m)
```

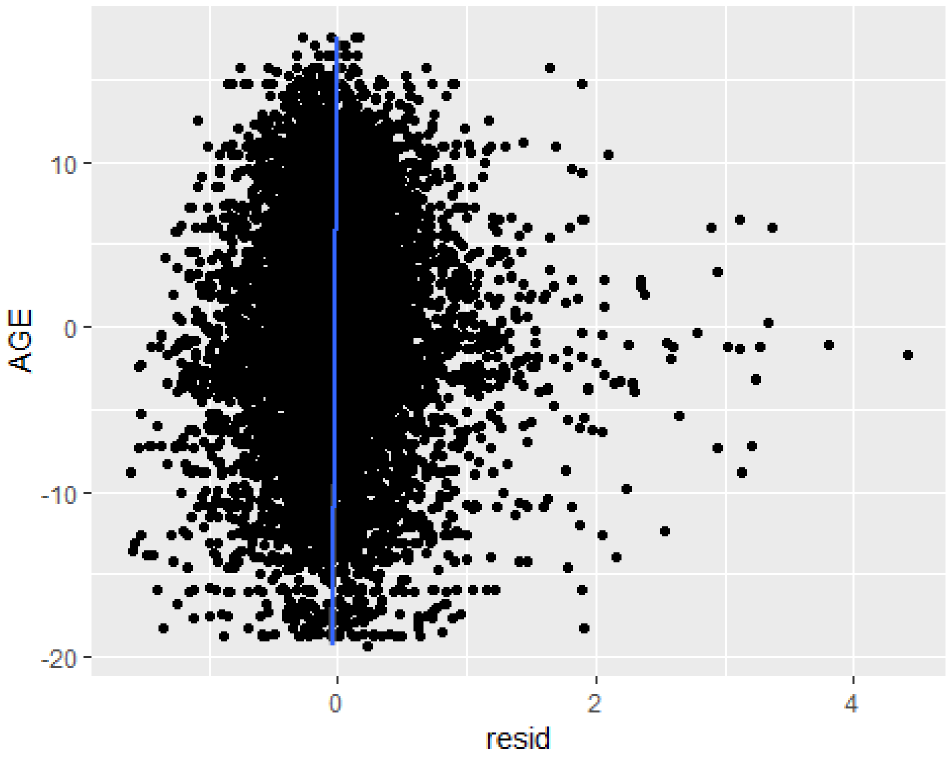


```
ggplot(augDat,aes(x=edu.cat,y=resid))+geom_boxplot()+coord_flip()
```

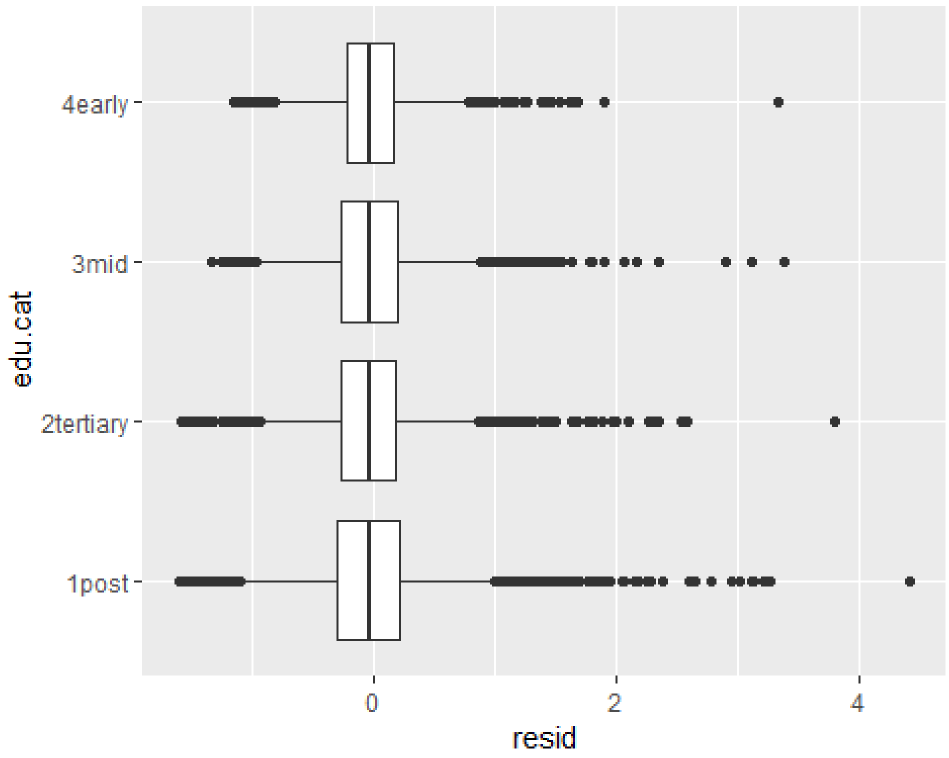


```
ggplot(augDat,aes(x=diagn,y=resid))+geom_boxplot()+coord_flip()
```

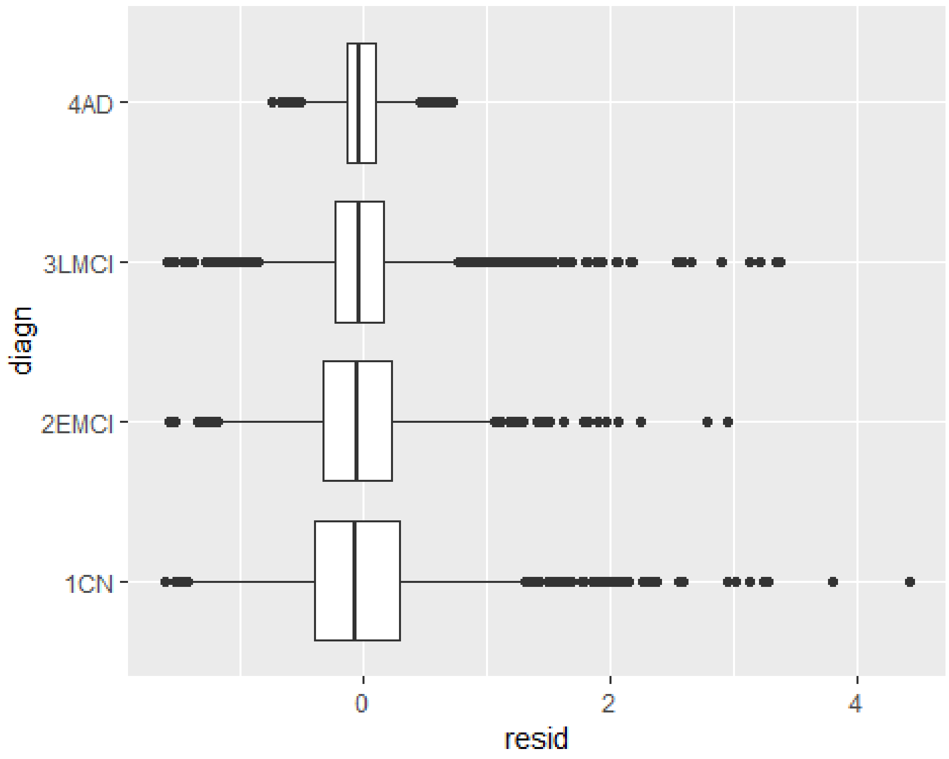


```
ggplot(augDat,aes(x=APOE4,y=resid))+geom_boxplot()+coord_flip()
```

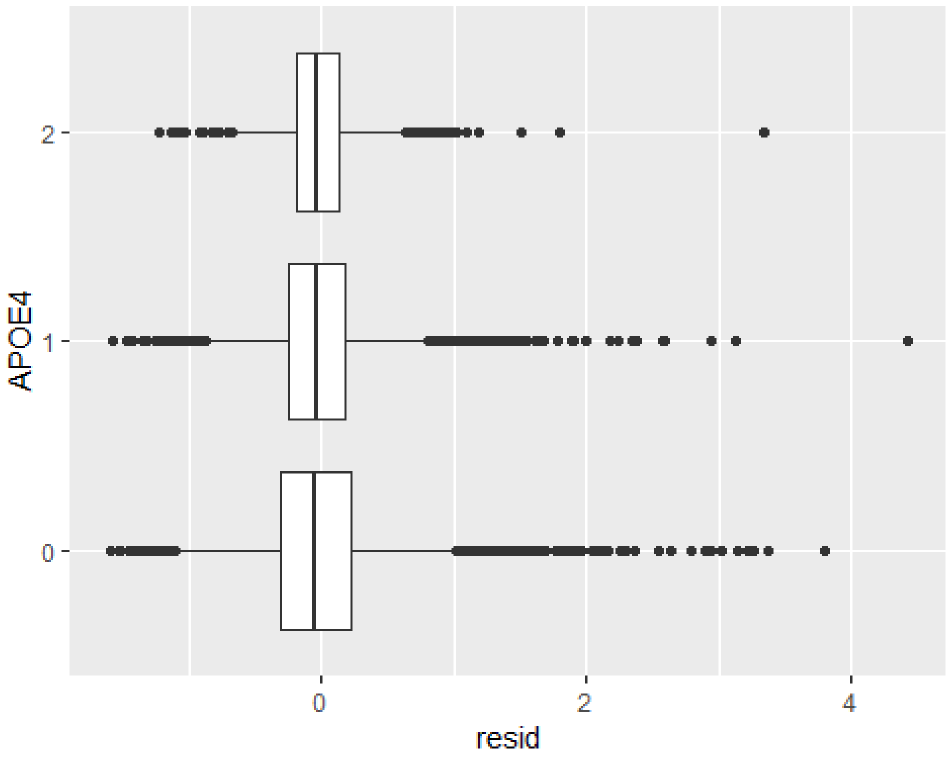


```
ggplot(augDat,aes(x=as.factor(M),y=resid))+geom_boxplot()+coord_flip()
```

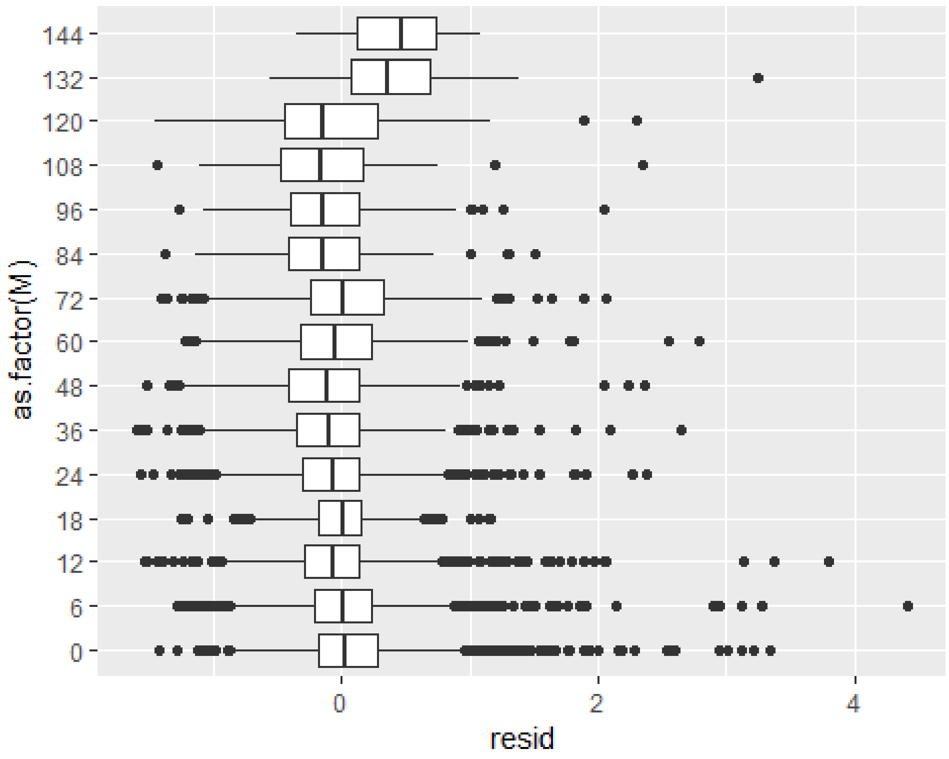


```
ggplot(augDat,aes(x=as.factor(diclo),y=resid))+geom_boxplot()+coord_flip()
```

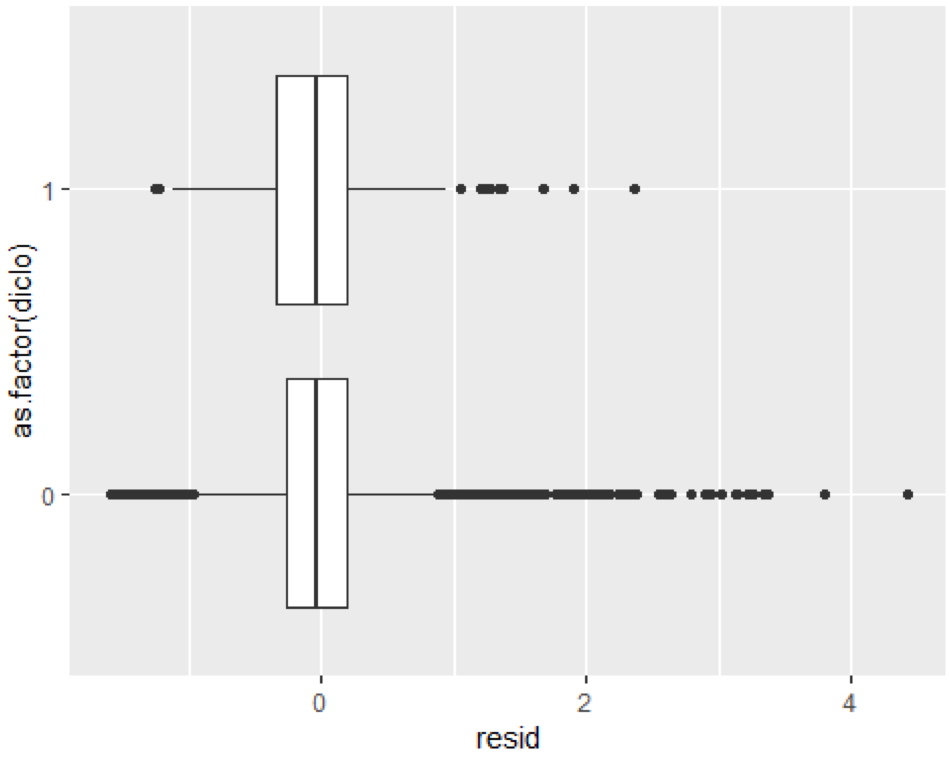


```
ggplot(augDat,aes(x=as.factor(parac),y=resid))+geom_boxplot()+coord_flip()
```

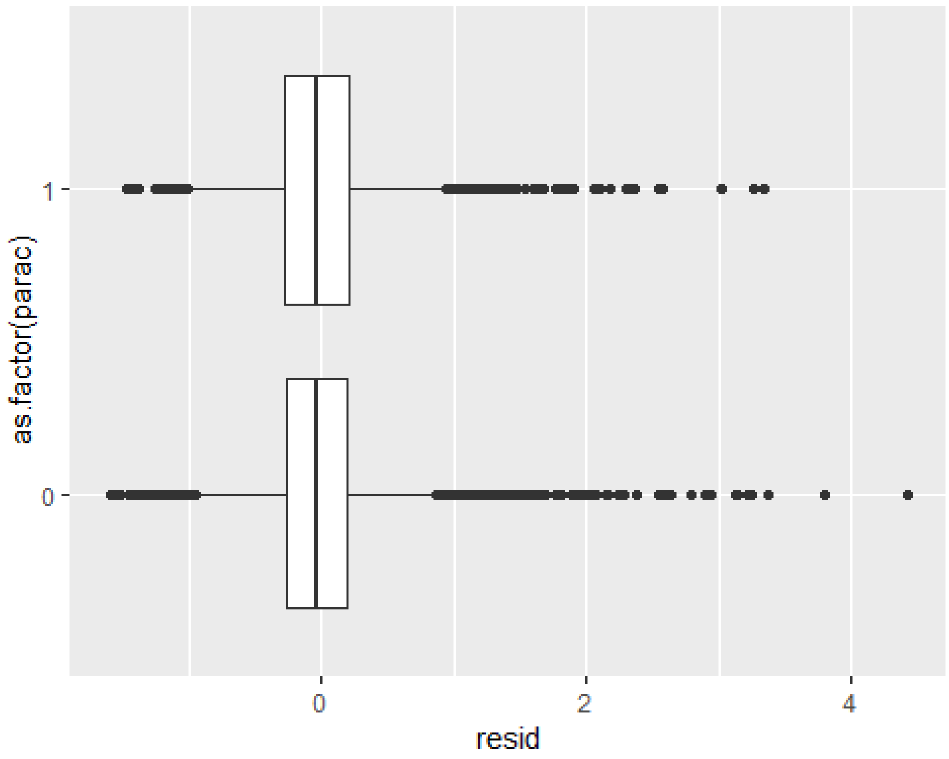

8.22. *Checking for multicollinearity*

```
cov2cor(vcov(neg.ADAS))

                     (Intercept)       M     AGE  APOE41  APOE42 GenderMale
(Intercept)               1.0000  0.1566 -0.0195 -0.0331 -0.0484     0.0075
M                         0.1566  1.0000 -0.0003 -0.1254 -0.1755     0.0192
AGE                      -0.0195 -0.0003  1.0000 -0.0106  0.0106    -0.0144
APOE41                   -0.0331 -0.1254 -0.0106  1.0000  0.0406     0.0069
APOE42                   -0.0484 -0.1755  0.0106  0.0406  1.0000     0.0101
GenderMale                0.0075  0.0192 -0.0144  0.0069  0.0101     1.0000
edu.cat2tertiary         -0.0014  0.0102  0.0074  0.0165 -0.0057    -0.0031
edu.cat3mid               0.0039  0.0381  0.0196  0.0008  0.0098    -0.0227
edu.cat4early            -0.0266 -0.0369 -0.0043 -0.0010  0.0077     0.0082
diagn2EMCI               -0.0161 -0.1669 -0.0337 -0.0061  0.0170    -0.0033
diagn3LMCI                0.0386 -0.1162 -0.0161  0.0216  0.0446     0.0147
diagn4AD                 -0.1914 -0.4425 -0.0332 -0.0337 -0.0330     0.0154
headache                  0.0418  0.1439 -0.0075  0.0096 -0.0128     0.0048
diclo                     0.0271  0.1052  0.0012 -0.0138  0.0141     0.0131
Ibu                       0.0455  0.1238  0.0070 -0.0181 -0.0057    -0.0137
parac                     0.0632  0.1673  0.0128  0.0136  0.0230     0.0024
M:edu.cat2tertiary        0.0046  0.0174 -0.0013 -0.0042 -0.0023     0.0021
M:edu.cat3mid             0.0015  0.0022  0.0018  0.0003 -0.0013    -0.0098
M:edu.cat4early           0.0033 -0.0895  0.0040  0.0009 -0.0020    -0.0057
M:diagn2EMCI             -0.0026 -0.0302  0.0001 -0.0005 -0.0011    -0.0004
M:diagn3LMCI              0.0027 -0.1939 -0.0014 -0.0009 -0.0046     0.0061
M:diagn4AD               -0.0010 -0.1233 -0.0031 -0.0052 -0.0058     0.0020
M:APOE41                 -0.0073 -0.0659 -0.0003  0.0355  0.0042    -0.0010
M:APOE42                 -0.0066 -0.0573 -0.0065  0.0021  0.0277    -0.0020
M:GenderMale              0.0081 -0.0073  0.0033  0.0003 -0.0021     0.0378
M:parac                  -0.0104 -0.0256  0.0038 -0.0001  0.0006    -0.0062
                     edu.cat2tertiary edu.cat3mid edu.cat4early diagn2EMCI
(Intercept)                   -0.0014      0.0039       -0.0266    -0.0161
```

M	0.0102	0.0381	-0.0369	-0.1669
AGE	0.0074	0.0196	-0.0043	-0.0337
APOE41	0.0165	0.0008	-0.0010	-0.0061
APOE42	-0.0057	0.0098	0.0077	0.0170
GenderMale	-0.0031	-0.0227	0.0082	-0.0033
edu.cat2tertiary	1.0000	0.0002	0.0265	0.0087
edu.cat3mid	0.0002	1.0000	0.0286	0.0031
edu.cat4early	0.0265	0.0286	1.0000	-0.0043
diagn2EMCI	0.0087	0.0031	-0.0043	1.0000
diagn3LMCI	-0.0080	-0.0143	0.0411	-0.0201
diagn4AD	-0.0135	-0.0127	-0.0099	0.0853
headache	-0.0007	-0.0047	-0.0047	-0.0120
diclo	0.0009	-0.0271	0.0133	-0.0147
Ibu	-0.0002	0.0153	0.0072	-0.0025
parac	0.0005	0.0094	0.0148	-0.0037
M:edu.cat2tertiary	0.0451	-0.0021	-0.0001	-0.0003
M:edu.cat3mid	-0.0008	0.0384	-0.0020	-0.0018
M:edu.cat4early	-0.0021	-0.0023	0.0393	-0.0005
M:diagn2EMCI	0.0001	-0.0020	0.0003	0.0133
M:diagn3LMCI	-0.0014	0.0031	-0.0040	-0.0053
M:diagn4AD	0.0021	-0.0004	-0.0041	-0.0024
M:APOE41	-0.0057	0.0003	-0.0025	0.0012
M:APOE42	-0.0026	-0.0014	-0.0021	0.0007
M:GenderMale	-0.0030	0.0063	0.0055	-0.0012
M:parac	-0.0010	-0.0020	-0.0023	0.0008

	diagn3LMCI	diagn4AD	headache	diclo	Ibu	parac
(Intercept)	0.0386	-0.1914	0.0418	0.0271	0.0455	0.0632
M	-0.1162	-0.4425	0.1439	0.1052	0.1238	0.1673
AGE	-0.0161	-0.0332	-0.0075	0.0012	0.0070	0.0128
APOE41	0.0216	-0.0337	0.0096	-0.0138	-0.0181	0.0136
APOE42	0.0446	-0.0330	-0.0128	0.0141	-0.0057	0.0230
GenderMale	0.0147	0.0154	0.0048	0.0131	-0.0137	0.0024
edu.cat2tertiary	-0.0080	-0.0135	-0.0007	0.0009	-0.0002	0.0005
edu.cat3mid	-0.0143	-0.0127	-0.0047	-0.0271	0.0153	0.0094
edu.cat4early	0.0411	-0.0099	-0.0047	0.0133	0.0072	0.0148
diagn2EMCI	-0.0201	0.0853	-0.0120	-0.0147	-0.0025	-0.0037
diagn3LMCI	1.0000	0.2222	0.0001	-0.0036	0.0012	0.0127
diagn4AD	0.2222	1.0000	0.0023	-0.0072	0.0018	-0.0045
headache	0.0001	0.0023	1.0000	-0.0137	-0.0095	0.0019
diclo	-0.0036	-0.0072	-0.0137	1.0000	-0.0099	0.0113
Ibu	0.0012	0.0018	-0.0095	-0.0099	1.0000	0.0036
parac	0.0127	-0.0045	0.0019	0.0113	0.0036	1.0000
M:edu.cat2tertiary	-0.0022	0.0016	-0.0015	0.0002	0.0026	-0.0023
M:edu.cat3mid	0.0004	-0.0025	0.0028	0.0009	0.0014	-0.0008
M:edu.cat4early	0.0037	-0.0169	-0.0001	0.0026	0.0026	0.0035
M:diagn2EMCI	0.0000	0.0009	0.0000	-0.0010	-0.0007	-0.0026
M:diagn3LMCI	0.0218	-0.0126	0.0048	0.0011	0.0049	0.0033
M:diagn4AD	0.0018	-0.0025	0.0015	0.0014	0.0008	0.0004
M:APOE41	-0.0022	-0.0137	0.0014	0.0024	0.0032	0.0021
M:APOE42	-0.0042	-0.0154	0.0039	0.0016	0.0031	0.0052
M:GenderMale	-0.0010	-0.0055	0.0005	0.0013	0.0026	-0.0052
M:parac	-0.0038	-0.0032	0.0010	0.0031	0.0055	0.0276

	M:edu.cat2tertiary	M:edu.cat3mid	M:edu.cat4early
(Intercept)	0.0046	0.0015	0.0033
M	0.0174	0.0022	-0.0895
AGE	-0.0013	0.0018	0.0040
APOE41	-0.0042	0.0003	0.0009
APOE42	-0.0023	-0.0013	-0.0020
GenderMale	0.0021	-0.0098	-0.0057

```
edu.cat2tertiary                0.0451       -0.0008         -0.0021
edu.cat3mid                    -0.0021        0.0384         -0.0023
edu.cat4early                  -0.0001       -0.0020          0.0393
diagn2EMCI                     -0.0003       -0.0018         -0.0005
diagn3LMCI                     -0.0022        0.0004          0.0037
diagn4AD                        0.0016       -0.0025         -0.0169
headache                       -0.0015        0.0028         -0.0001
diclo                           0.0002        0.0009          0.0026
Ibu                             0.0026        0.0014          0.0026
parac                          -0.0023       -0.0008          0.0035
M:edu.cat2tertiary              1.0000       -0.0054          0.0628
M:edu.cat3mid                  -0.0054        1.0000          0.0781
M:edu.cat4early                 0.0628        0.0781          1.0000
M:diagn2EMCI                    0.0090       -0.0252          0.0129
M:diagn3LMCI                   -0.0021        0.0188          0.0343
M:diagn4AD                      0.0063       -0.0056         -0.0119
M:APOE41                       -0.0145       -0.0113          0.0226
M:APOE42                        0.0068       -0.0357          0.0382
M:GenderMale                   -0.0125       -0.0111          0.0278
M:parac                        -0.0220        0.0264          0.0435
                   M:diagn2EMCI M:diagn3LMCI M:diagn4AD M:APOE41 M:APOE42
(Intercept)             -0.0026       0.0027    -0.0010  -0.0073  -0.0066
M                       -0.0302      -0.1939    -0.1233  -0.0659  -0.0573
AGE                      0.0001      -0.0014    -0.0031  -0.0003  -0.0065
APOE41                  -0.0005      -0.0009    -0.0052   0.0355   0.0021
APOE42                  -0.0011      -0.0046    -0.0058   0.0042   0.0277
GenderMale              -0.0004       0.0061     0.0020  -0.0010  -0.0020
edu.cat2tertiary         0.0001      -0.0014     0.0021  -0.0057  -0.0026
edu.cat3mid             -0.0020       0.0031    -0.0004   0.0003  -0.0014
edu.cat4early            0.0003      -0.0040    -0.0041  -0.0025  -0.0021
diagn2EMCI               0.0133      -0.0053    -0.0024   0.0012   0.0007
diagn3LMCI               0.0000       0.0218     0.0018  -0.0022  -0.0042
diagn4AD                 0.0009      -0.0126    -0.0025  -0.0137  -0.0154
headache                 0.0000       0.0048     0.0015   0.0014   0.0039
diclo                   -0.0010       0.0011     0.0014   0.0024   0.0016
Ibu                     -0.0007       0.0049     0.0008   0.0032   0.0031
parac                   -0.0026       0.0033     0.0004   0.0021   0.0052
M:edu.cat2tertiary       0.0090      -0.0021     0.0063  -0.0145   0.0068
M:edu.cat3mid           -0.0252       0.0188    -0.0056  -0.0113  -0.0357
M:edu.cat4early          0.0129       0.0343    -0.0119   0.0226   0.0382
M:diagn2EMCI             1.0000       0.1025     0.0643   0.0082  -0.0060
M:diagn3LMCI             0.1025       1.0000     0.1694  -0.0215  -0.0604
M:diagn4AD               0.0643       0.1694     1.0000  -0.0096  -0.0037
M:APOE41                 0.0082      -0.0215    -0.0096   1.0000   0.1037
M:APOE42                -0.0060      -0.0604    -0.0037   0.1037   1.0000
M:GenderMale            -0.0262       0.0525     0.0122   0.0298   0.0155
M:parac                  0.0171      -0.0304    -0.0132   0.0006   0.0112
                   M:GenderMale M:parac
(Intercept)              0.0081 -0.0104
M                       -0.0073 -0.0256
AGE                      0.0033  0.0038
APOE41                   0.0003 -0.0001
APOE42                  -0.0021  0.0006
GenderMale               0.0378 -0.0062
edu.cat2tertiary        -0.0030 -0.0010
edu.cat3mid              0.0063 -0.0020
edu.cat4early            0.0055 -0.0023
diagn2EMCI              -0.0012  0.0008
diagn3LMCI              -0.0010 -0.0038
```

```
diagn4AD                 -0.0055 -0.0032
headache                  0.0005  0.0010
diclo                     0.0013  0.0031
Ibu                       0.0026  0.0055
parac                    -0.0052  0.0276
M:edu.cat2tertiary       -0.0125 -0.0220
M:edu.cat3mid            -0.0111  0.0264
M:edu.cat4early           0.0278  0.0435
M:diagn2EMCI             -0.0262  0.0171
M:diagn3LMCI              0.0525 -0.0304
M:diagn4AD                0.0122 -0.0132
M:APOE41                  0.0298  0.0006
M:APOE42                  0.0155  0.0112
M:GenderMale              1.0000 -0.0654
M:parac                  -0.0654  1.0000

abs(cov2cor(vcov(neg.ADAS)))>0.4

                   (Intercept)     M   AGE APOE41 APOE42 GenderMale
(Intercept)               TRUE FALSE FALSE  FALSE  FALSE      FALSE
M                        FALSE  TRUE FALSE  FALSE  FALSE      FALSE
AGE                      FALSE FALSE  TRUE  FALSE  FALSE      FALSE
APOE41                   FALSE FALSE FALSE   TRUE  FALSE      FALSE
APOE42                   FALSE FALSE FALSE  FALSE   TRUE      FALSE
GenderMale               FALSE FALSE FALSE  FALSE  FALSE       TRUE
edu.cat2tertiary         FALSE FALSE FALSE  FALSE  FALSE      FALSE
edu.cat3mid              FALSE FALSE FALSE  FALSE  FALSE      FALSE
edu.cat4early            FALSE FALSE FALSE  FALSE  FALSE      FALSE
diagn2EMCI               FALSE FALSE FALSE  FALSE  FALSE      FALSE
diagn3LMCI               FALSE FALSE FALSE  FALSE  FALSE      FALSE
diagn4AD                 FALSE  TRUE FALSE  FALSE  FALSE      FALSE
headache                 FALSE FALSE FALSE  FALSE  FALSE      FALSE
diclo                    FALSE FALSE FALSE  FALSE  FALSE      FALSE
Ibu                      FALSE FALSE FALSE  FALSE  FALSE      FALSE
parac                    FALSE FALSE FALSE  FALSE  FALSE      FALSE
M:edu.cat2tertiary       FALSE FALSE FALSE  FALSE  FALSE      FALSE
M:edu.cat3mid            FALSE FALSE FALSE  FALSE  FALSE      FALSE
M:edu.cat4early          FALSE FALSE FALSE  FALSE  FALSE      FALSE
M:diagn2EMCI             FALSE FALSE FALSE  FALSE  FALSE      FALSE
M:diagn3LMCI             FALSE FALSE FALSE  FALSE  FALSE      FALSE
M:diagn4AD               FALSE FALSE FALSE  FALSE  FALSE      FALSE
M:APOE41                 FALSE FALSE FALSE  FALSE  FALSE      FALSE
M:APOE42                 FALSE FALSE FALSE  FALSE  FALSE      FALSE
M:GenderMale             FALSE FALSE FALSE  FALSE  FALSE      FALSE
M:parac                  FALSE FALSE FALSE  FALSE  FALSE      FALSE
                   edu.cat2tertiary edu.cat3mid edu.cat4early diagn2EMCI
(Intercept)                   FALSE       FALSE         FALSE      FALSE
M                             FALSE       FALSE         FALSE      FALSE
AGE                           FALSE       FALSE         FALSE      FALSE
APOE41                        FALSE       FALSE         FALSE      FALSE
APOE42                        FALSE       FALSE         FALSE      FALSE
GenderMale                    FALSE       FALSE         FALSE      FALSE
edu.cat2tertiary               TRUE       FALSE         FALSE      FALSE
edu.cat3mid                   FALSE        TRUE         FALSE      FALSE
edu.cat4early                 FALSE       FALSE          TRUE      FALSE
diagn2EMCI                    FALSE       FALSE         FALSE       TRUE
diagn3LMCI                    FALSE       FALSE         FALSE      FALSE
diagn4AD                      FALSE       FALSE         FALSE      FALSE
headache                      FALSE       FALSE         FALSE      FALSE
```

```
diclo                         FALSE       FALSE         FALSE      FALSE
Ibu                           FALSE       FALSE         FALSE      FALSE
parac                         FALSE       FALSE         FALSE      FALSE
M:edu.cat2tertiary            FALSE       FALSE         FALSE      FALSE
M:edu.cat3mid                 FALSE       FALSE         FALSE      FALSE
M:edu.cat4early               FALSE       FALSE         FALSE      FALSE
M:diagn2EMCI                  FALSE       FALSE         FALSE      FALSE
M:diagn3LMCI                  FALSE       FALSE         FALSE      FALSE
M:diagn4AD                    FALSE       FALSE         FALSE      FALSE
M:APOE41                      FALSE       FALSE         FALSE      FALSE
M:APOE42                      FALSE       FALSE         FALSE      FALSE
M:GenderMale                  FALSE       FALSE         FALSE      FALSE
M:parac                       FALSE       FALSE         FALSE      FALSE
                   diagn3LMCI diagn4AD headache diclo   Ibu parac
(Intercept)             FALSE    FALSE    FALSE FALSE FALSE FALSE
M                       FALSE     TRUE    FALSE FALSE FALSE FALSE
AGE                     FALSE    FALSE    FALSE FALSE FALSE FALSE
APOE41                  FALSE    FALSE    FALSE FALSE FALSE FALSE
APOE42                  FALSE    FALSE    FALSE FALSE FALSE FALSE
GenderMale              FALSE    FALSE    FALSE FALSE FALSE FALSE
edu.cat2tertiary        FALSE    FALSE    FALSE FALSE FALSE FALSE
edu.cat3mid             FALSE    FALSE    FALSE FALSE FALSE FALSE
edu.cat4early           FALSE    FALSE    FALSE FALSE FALSE FALSE
diagn2EMCI              FALSE    FALSE    FALSE FALSE FALSE FALSE
diagn3LMCI               TRUE    FALSE    FALSE FALSE FALSE FALSE
diagn4AD                FALSE     TRUE    FALSE FALSE FALSE FALSE
headache                FALSE    FALSE     TRUE FALSE FALSE FALSE
diclo                   FALSE    FALSE    FALSE  TRUE FALSE FALSE
Ibu                     FALSE    FALSE    FALSE FALSE  TRUE FALSE
parac                   FALSE    FALSE    FALSE FALSE FALSE  TRUE
M:edu.cat2tertiary      FALSE    FALSE    FALSE FALSE FALSE FALSE
M:edu.cat3mid           FALSE    FALSE    FALSE FALSE FALSE FALSE
M:edu.cat4early         FALSE    FALSE    FALSE FALSE FALSE FALSE
M:diagn2EMCI            FALSE    FALSE    FALSE FALSE FALSE FALSE
M:diagn3LMCI            FALSE    FALSE    FALSE FALSE FALSE FALSE
M:diagn4AD              FALSE    FALSE    FALSE FALSE FALSE FALSE
M:APOE41                FALSE    FALSE    FALSE FALSE FALSE FALSE
M:APOE42                FALSE    FALSE    FALSE FALSE FALSE FALSE
M:GenderMale            FALSE    FALSE    FALSE FALSE FALSE FALSE
M:parac                 FALSE    FALSE    FALSE FALSE FALSE FALSE
                   M:edu.cat2tertiary M:edu.cat3mid M:edu.cat4early
(Intercept)                     FALSE         FALSE           FALSE
M                               FALSE         FALSE           FALSE
AGE                             FALSE         FALSE           FALSE
APOE41                          FALSE         FALSE           FALSE
APOE42                          FALSE         FALSE           FALSE
GenderMale                      FALSE         FALSE           FALSE
edu.cat2tertiary                FALSE         FALSE           FALSE
edu.cat3mid                     FALSE         FALSE           FALSE
edu.cat4early                   FALSE         FALSE           FALSE
diagn2EMCI                      FALSE         FALSE           FALSE
diagn3LMCI                      FALSE         FALSE           FALSE
diagn4AD                        FALSE         FALSE           FALSE
headache                        FALSE         FALSE           FALSE
diclo                           FALSE         FALSE           FALSE
Ibu                             FALSE         FALSE           FALSE
parac                           FALSE         FALSE           FALSE
M:edu.cat2tertiary               TRUE         FALSE           FALSE
M:edu.cat3mid                   FALSE          TRUE           FALSE
```

```
M:edu.cat4early                FALSE         FALSE            TRUE
M:diagn2EMCI                   FALSE         FALSE           FALSE
M:diagn3LMCI                   FALSE         FALSE           FALSE
M:diagn4AD                     FALSE         FALSE           FALSE
M:APOE41                       FALSE         FALSE           FALSE
M:APOE42                       FALSE         FALSE           FALSE
M:GenderMale                   FALSE         FALSE           FALSE
M:parac                        FALSE         FALSE           FALSE
                   M:diagn2EMCI M:diagn3LMCI M:diagn4AD M:APOE41 M:APOE42
(Intercept)               FALSE        FALSE      FALSE    FALSE    FALSE
M                         FALSE        FALSE      FALSE    FALSE    FALSE
AGE                       FALSE        FALSE      FALSE    FALSE    FALSE
APOE41                    FALSE        FALSE      FALSE    FALSE    FALSE
APOE42                    FALSE        FALSE      FALSE    FALSE    FALSE
GenderMale                FALSE        FALSE      FALSE    FALSE    FALSE
edu.cat2tertiary          FALSE        FALSE      FALSE    FALSE    FALSE
edu.cat3mid               FALSE        FALSE      FALSE    FALSE    FALSE
edu.cat4early             FALSE        FALSE      FALSE    FALSE    FALSE
diagn2EMCI                FALSE        FALSE      FALSE    FALSE    FALSE
diagn3LMCI                FALSE        FALSE      FALSE    FALSE    FALSE
diagn4AD                  FALSE        FALSE      FALSE    FALSE    FALSE
headache                  FALSE        FALSE      FALSE    FALSE    FALSE
diclo                     FALSE        FALSE      FALSE    FALSE    FALSE
Ibu                       FALSE        FALSE      FALSE    FALSE    FALSE
parac                     FALSE        FALSE      FALSE    FALSE    FALSE
M:edu.cat2tertiary        FALSE        FALSE      FALSE    FALSE    FALSE
M:edu.cat3mid             FALSE        FALSE      FALSE    FALSE    FALSE
M:edu.cat4early           FALSE        FALSE      FALSE    FALSE    FALSE
M:diagn2EMCI               TRUE        FALSE      FALSE    FALSE    FALSE
M:diagn3LMCI              FALSE         TRUE      FALSE    FALSE    FALSE
M:diagn4AD                FALSE        FALSE       TRUE    FALSE    FALSE
M:APOE41                  FALSE        FALSE      FALSE     TRUE    FALSE
M:APOE42                  FALSE        FALSE      FALSE    FALSE     TRUE
M:GenderMale              FALSE        FALSE      FALSE    FALSE    FALSE
M:parac                   FALSE        FALSE      FALSE    FALSE    FALSE
                   M:GenderMale M:parac
(Intercept)               FALSE   FALSE
M                         FALSE   FALSE
AGE                       FALSE   FALSE
APOE41                    FALSE   FALSE
APOE42                    FALSE   FALSE
GenderMale                FALSE   FALSE
edu.cat2tertiary          FALSE   FALSE
edu.cat3mid               FALSE   FALSE
edu.cat4early             FALSE   FALSE
diagn2EMCI                FALSE   FALSE
diagn3LMCI                FALSE   FALSE
diagn4AD                  FALSE   FALSE
headache                  FALSE   FALSE
diclo                     FALSE   FALSE
Ibu                       FALSE   FALSE
parac                     FALSE   FALSE
M:edu.cat2tertiary        FALSE   FALSE
M:edu.cat3mid             FALSE   FALSE
M:edu.cat4early           FALSE   FALSE
M:diagn2EMCI              FALSE   FALSE
M:diagn3LMCI              FALSE   FALSE
M:diagn4AD                FALSE   FALSE
M:APOE41                  FALSE   FALSE
```

```
M:APOE42                   FALSE   FALSE
M:GenderMale                TRUE   FALSE
M:parac                    FALSE    TRUE
```

8.23. *Checking other distributions*

The negative binomial GLMM was still the optimal model with the final selected explanatory models.

```
glmerB1.ADAS<-glmer(cbind(success,fail)~AGE + APOE4  + M + edu.cat + diagn+
diagn*M +APOE4*M + Gender*M+ edu.cat*M +parac*M +(1|ID), family="binomial",
data=ADASdata)
try(summary(glmerB1.ADAS))

Generalized linear mixed model fit by maximum likelihood (Laplace
  Approximation) [glmerMod]
 Family: binomial  ( logit )
Formula:
cbind(success, fail) ~ AGE + APOE4 + M + edu.cat + diagn + diagn *
    M + APOE4 * M + Gender * M + edu.cat * M + parac * M + (1 |      ID)
   Data: ADASdata

     AIC      BIC   logLik deviance df.resid
 86593.1  86763.0 -43272.6  86545.1     8746

Scaled residuals:
     Min       1Q   Median       3Q      Max
-17.8102  -0.9797   0.1092   1.1490   8.0992

Random effects:
 Groups Name        Variance Std.Dev.
 ID     (Intercept) 0.296    0.5441
Number of obs: 8770, groups:  ID, 1618

Fixed effects:
                     Estimate Std. Error z value Pr(>|z|)
(Intercept)         2.5863055  0.0389396  66.418  < 2e-16 ***
AGE                -0.0163042  0.0019781  -8.242  < 2e-16 ***
APOE41             -0.1120434  0.0305435  -3.668 0.000244 ***
APOE42             -0.1574813  0.0488504  -3.224 0.001265 **
M                  -0.0092655  0.0002136 -43.388  < 2e-16 ***
edu.cat2tertiary   -0.1224699  0.0350274  -3.496 0.000472 ***
edu.cat3mid        -0.1801287  0.0396889  -4.539 5.67e-06 ***
edu.cat4early      -0.2018236  0.0419884  -4.807 1.53e-06 ***
diagn2EMCI         -0.4079295  0.0429006  -9.509  < 2e-16 ***
diagn3LMCI         -0.9113991  0.0371037 -24.564  < 2e-16 ***
diagn4AD           -1.5830416  0.0431188 -36.713  < 2e-16 ***
GenderMale         -0.1323070  0.0287986  -4.594 4.34e-06 ***
parac               0.0703709  0.0322214   2.184 0.028964 *
M:diagn2EMCI        0.0016024  0.0002542   6.303 2.92e-10 ***
M:diagn3LMCI       -0.0017421  0.0001815  -9.599  < 2e-16 ***
M:diagn4AD         -0.0109736  0.0005311 -20.662  < 2e-16 ***
APOE41:M           -0.0044099  0.0001714 -25.732  < 2e-16 ***
APOE42:M           -0.0074793  0.0003071 -24.357  < 2e-16 ***
M:GenderMale        0.0031335  0.0001658  18.899  < 2e-16 ***
M:edu.cat2tertiary  0.0030335  0.0001983  15.298  < 2e-16 ***
M:edu.cat3mid       0.0021859  0.0002235   9.779  < 2e-16 ***
M:edu.cat4early     0.0018102  0.0002395   7.557 4.11e-14 ***
M:parac            -0.0006302  0.0001626  -3.876 0.000106 ***
```

```
---
Signif. codes:  0 '***' 0.001 '**' 0.01 '*' 0.05 '.' 0.1 ' ' 1


Correlation matrix not shown by default, as p = 23 > 12.
Use print(x, correlation=TRUE)  or
    vcov(x)        if you need it

convergence code: 0
Model failed to converge with max|grad| = 0.0596937 (tol = 0.001, component 1)
Model is nearly unidentifiable: very large eigenvalue
 - Rescale variables?
Model is nearly unidentifiable: large eigenvalue ratio
 - Rescale variables?

try(glmerB2.ADAS<-glmer(cbind(success,fail)~AGE + APOE4  + M + edu.cat + diagn+
diagn*M +APOE4*M + Gender*M+ edu.cat*M +parac*M +(1|ID),
family=binomial(link=probit), data=ADASdata))
try(summary(glmerB2.ADAS))

Generalized linear mixed model fit by maximum likelihood (Laplace
  Approximation) [glmerMod]
 Family: binomial  ( probit )
Formula:
cbind(success, fail) ~ AGE + APOE4 + M + edu.cat + diagn + diagn *
    M + APOE4 * M + Gender * M + edu.cat * M + parac * M + (1 |      ID)
   Data: ADASdata

     AIC      BIC   logLik deviance df.resid
 87510.2  87680.1 -43731.1  87462.2     8746

Scaled residuals:
     Min       1Q   Median       3Q      Max
-18.4974  -0.9864   0.1165   1.1538   8.4049

Random effects:
 Groups Name        Variance Std.Dev.
 ID     (Intercept) 0.09008  0.3001
Number of obs: 8770, groups:  ID, 1618

Fixed effects:
                     Estimate Std. Error z value Pr(>|z|)
(Intercept)         1.467e+00  2.141e-02  68.525  < 2e-16 ***
AGE                -8.530e-03  1.091e-03  -7.817 5.39e-15 ***
APOE41             -6.261e-02  1.684e-02  -3.718 0.000201 ***
APOE42             -8.048e-02  2.697e-02  -2.984 0.002844 **
M                  -4.606e-03  1.147e-04 -40.148  < 2e-16 ***
edu.cat2tertiary   -5.904e-02  1.930e-02  -3.059 0.002223 **
edu.cat3mid        -9.137e-02  2.187e-02  -4.178 2.94e-05 ***
edu.cat4early      -9.974e-02  2.316e-02  -4.306 1.66e-05 ***
diagn2EMCI         -2.136e-01  2.360e-02  -9.051  < 2e-16 ***
diagn3LMCI         -4.923e-01  2.043e-02 -24.102  < 2e-16 ***
diagn4AD           -8.811e-01  2.380e-02 -37.027  < 2e-16 ***
GenderMale         -6.477e-02  1.587e-02  -4.081 4.49e-05 ***
parac               3.889e-02  1.776e-02   2.190 0.028523 *
M:diagn2EMCI        8.219e-04  1.374e-04   5.982 2.20e-09 ***
M:diagn3LMCI       -1.297e-03  9.976e-05 -13.001  < 2e-16 ***
M:diagn4AD         -7.397e-03  3.216e-04 -22.996  < 2e-16 ***
APOE41:M           -2.501e-03  9.537e-05 -26.221  < 2e-16 ***
APOE42:M           -4.476e-03  1.773e-04 -25.242  < 2e-16 ***
```

```
M:GenderMale        1.636e-03  9.160e-05  17.859  < 2e-16 ***
M:edu.cat2tertiary  1.490e-03  1.088e-04  13.698  < 2e-16 ***
M:edu.cat3mid       1.002e-03  1.230e-04   8.151 3.60e-16 ***
M:edu.cat4early     7.395e-04  1.351e-04   5.475 4.36e-08 ***
M:parac            -3.545e-04  9.000e-05  -3.939 8.20e-05 ***
---
Signif. codes:  0 '***' 0.001 '**' 0.01 '*' 0.05 '.' 0.1 ' ' 1


Correlation matrix not shown by default, as p = 23 > 12.
Use print(x, correlation=TRUE)  or
    vcov(x)        if you need it

convergence code: 0
Model failed to converge with max|grad| = 0.0855426 (tol = 0.001, component 1)
Model is nearly unidentifiable: very large eigenvalue
 - Rescale variables?
Model is nearly unidentifiable: large eigenvalue ratio
 - Rescale variables?

try(glmerB3.ADAS<-glmer(cbind(success,fail)~AGE + APOE4  + M + edu.cat + diagn+
diagn*M +APOE4*M + Gender*M+ edu.cat*M +parac*M +(1|ID),
family=binomial(link=cloglog), data=ADASdata))
try(summary(glmerB3.ADAS))

Generalized linear mixed model fit by maximum likelihood (Laplace
  Approximation) [glmerMod]
 Family: binomial  ( cloglog )
Formula:
cbind(success, fail) ~ AGE + APOE4 + M + edu.cat + diagn + diagn *
    M + APOE4 * M + Gender * M + edu.cat * M + parac * M + (1 |      ID)
   Data: ADASdata

     AIC      BIC   logLik deviance df.resid
 89342.5  89512.4 -44647.2  89294.5     8746

Scaled residuals:
     Min       1Q   Median       3Q      Max
-19.5815  -0.9910   0.1233   1.1764   8.7688

Random effects:
 Groups Name        Variance Std.Dev.
 ID     (Intercept) 0.06834  0.2614
Number of obs: 8770, groups:  ID, 1618

Fixed effects:
                     Estimate Std. Error z value Pr(>|z|)
(Intercept)         9.621e-01  1.857e-02  51.809  < 2e-16 ***
AGE                -6.643e-03  9.506e-04  -6.988 2.79e-12 ***
APOE41             -5.678e-02  1.465e-02  -3.875 0.000106 ***
APOE42             -6.129e-02  2.351e-02  -2.607 0.009133 **
M                  -3.288e-03  9.269e-05 -35.470  < 2e-16 ***
edu.cat2tertiary   -3.974e-02  1.679e-02  -2.367 0.017921 *
edu.cat3mid        -6.893e-02  1.902e-02  -3.624 0.000290 ***
edu.cat4early      -7.027e-02  2.017e-02  -3.484 0.000494 ***
diagn2EMCI         -1.684e-01  2.046e-02  -8.230  < 2e-16 ***
diagn3LMCI         -4.077e-01  1.774e-02 -22.985  < 2e-16 ***
diagn4AD           -7.640e-01  2.076e-02 -36.798  < 2e-16 ***
GenderMale         -4.497e-02  1.381e-02  -3.257 0.001128 **
parac               3.304e-02  1.544e-02   2.139 0.032396 *
```

```
M:diagn2EMCI        6.626e-04  1.110e-04   5.972 2.35e-09 ***
M:diagn3LMCI       -1.346e-03  8.364e-05 -16.090  < 2e-16 ***
M:diagn4AD         -8.207e-03  3.273e-04 -25.071  < 2e-16 ***
APOE41:M           -2.071e-03  8.084e-05 -25.614  < 2e-16 ***
APOE42:M           -4.118e-03  1.613e-04 -25.531  < 2e-16 ***
M:GenderMale        1.223e-03  7.668e-05  15.945  < 2e-16 ***
M:edu.cat2tertiary  9.918e-04  8.999e-05  11.021  < 2e-16 ***
M:edu.cat3mid       5.818e-04  1.022e-04   5.691 1.26e-08 ***
M:edu.cat4early     2.871e-04  1.170e-04   2.453 0.014158 *
M:parac            -2.995e-04  7.552e-05  -3.967 7.29e-05 ***
---
Signif. codes:  0 '***' 0.001 '**' 0.01 '*' 0.05 '.' 0.1 ' ' 1


Correlation matrix not shown by default, as p = 23 > 12.
Use print(x, correlation=TRUE)  or
    vcov(x)        if you need it

convergence code: 0
Model failed to converge with max|grad| = 0.0928155 (tol = 0.001, component 1)
Model is nearly unidentifiable: very large eigenvalue
 - Rescale variables?
Model is nearly unidentifiable: large eigenvalue ratio
 - Rescale variables?

glmerP1.ADAS<-glmer(neg.b.ADAS~AGE + APOE4  + M + edu.cat + diagn+ diagn*M
+APOE4*M + Gender*M+ edu.cat*M +parac*M +(1|ID), family="poisson",
data=ADASdata)
summary(glmerP1.ADAS)

Generalized linear mixed model fit by maximum likelihood (Laplace
  Approximation) [glmerMod]
 Family: poisson  ( log )
Formula: neg.b.ADAS ~ AGE + APOE4 + M + edu.cat + diagn + diagn * M +
    APOE4 * M + Gender * M + edu.cat * M + parac * M + (1 | ID)
   Data: ADASdata

     AIC      BIC   logLik deviance df.resid
 79198.6  79368.5 -39575.3  79150.6     8746

Scaled residuals:
    Min      1Q  Median      3Q     Max
-5.3755 -1.0228 -0.0928  0.8547 13.0713

Random effects:
 Groups Name        Variance Std.Dev.
 ID     (Intercept) 0.1867   0.4321
Number of obs: 8770, groups:  ID, 1618

Fixed effects:
                     Estimate Std. Error z value Pr(>|z|)
(Intercept)         2.9513646  0.0311849  94.641  < 2e-16 ***
AGE                 0.0142615  0.0015758   9.050  < 2e-16 ***
APOE41              0.0993993  0.0243732   4.078 4.54e-05 ***
APOE42              0.1558897  0.0389078   4.007 6.16e-05 ***
M                   0.0076611  0.0001906  40.187  < 2e-16 ***
edu.cat2tertiary    0.1034941  0.0279689   3.700 0.000215 ***
edu.cat3mid         0.1496090  0.0316907   4.721 2.35e-06 ***
edu.cat4early       0.1800627  0.0334826   5.378 7.54e-08 ***
diagn2EMCI          0.3535316  0.0343667  10.287  < 2e-16 ***
```

```
diagn3LMCI          0.7743031  0.0296669  26.100  < 2e-16 ***
diagn4AD            1.2628971  0.0343908  36.722  < 2e-16 ***
GenderMale          0.1123906  0.0229869   4.889 1.01e-06 ***
parac              -0.0519200  0.0257157  -2.019 0.043487 *
M:diagn2EMCI       -0.0013999  0.0002302  -6.081 1.19e-09 ***
M:diagn3LMCI        0.0002909  0.0001608   1.809 0.070414 .
M:diagn4AD          0.0047517  0.0004144  11.466  < 2e-16 ***
APOE41:M            0.0029447  0.0001488  19.792  < 2e-16 ***
APOE42:M            0.0046996  0.0002537  18.525  < 2e-16 ***
M:GenderMale       -0.0021303  0.0001439 -14.801  < 2e-16 ***
M:edu.cat2tertiary -0.0022231  0.0001743 -12.757  < 2e-16 ***
M:edu.cat3mid      -0.0016253  0.0001954  -8.317  < 2e-16 ***
M:edu.cat4early    -0.0015501  0.0002047  -7.571 3.70e-14 ***
M:parac             0.0004980  0.0001416   3.516 0.000438 ***
---
Signif. codes:  0 '***' 0.001 '**' 0.01 '*' 0.05 '.' 0.1 ' ' 1


Correlation matrix not shown by default, as p = 23 > 12.
Use print(x, correlation=TRUE)  or
    vcov(x)        if you need it

convergence code: 0
Model failed to converge with max|grad| = 0.0267976 (tol = 0.001, component 1)
Model is nearly unidentifiable: very large eigenvalue
 - Rescale variables?
Model is nearly unidentifiable: large eigenvalue ratio
 - Rescale variables?

glmerP2.ADAS<-glmer(neg.b.ADAS~AGE + APOE4  + M + edu.cat + diagn+ diagn*M
+APOE4*M + Gender*M+ edu.cat*M +parac*M +(1|ID), family=poisson(link=sqrt),
data=ADASdata)
summary(glmerP2.ADAS)

Generalized linear mixed model fit by maximum likelihood (Laplace
  Approximation) [glmerMod]
 Family: poisson  ( sqrt )
Formula: neg.b.ADAS ~ AGE + APOE4 + M + edu.cat + diagn + diagn * M +
    APOE4 * M + Gender * M + edu.cat * M + parac * M + (1 | ID)
   Data: ADASdata

     AIC      BIC   logLik deviance df.resid
 81283.7  81453.6 -40617.8  81235.7     8746

Scaled residuals:
    Min      1Q  Median      3Q     Max
-5.5594 -1.0465 -0.1037  0.8665 15.7141

Random effects:
 Groups Name        Variance Std.Dev.
 ID     (Intercept) 2.024    1.423
Number of obs: 8770, groups:  ID, 1618

Fixed effects:
                     Estimate Std. Error z value Pr(>|z|)
(Intercept)         4.4917346  0.1014626  44.270  < 2e-16 ***
AGE                 0.0398710  0.0051927   7.678 1.61e-14 ***
APOE41              0.3401883  0.0801608   4.244 2.20e-05 ***
APOE42              0.4197018  0.1286754   3.262 0.001107 **
M                   0.0176477  0.0005732  30.787  < 2e-16 ***
```

```
edu.cat2tertiary    0.2240191  0.0918110   2.440 0.014687 *  
edu.cat3mid         0.3808625  0.1040279   3.661 0.000251 ***
edu.cat4early       0.4237827  0.1103966   3.839 0.000124 ***
diagn2EMCI          0.9862148  0.1119317   8.811  < 2e-16 ***
diagn3LMCI          2.3704572  0.0969958  24.439  < 2e-16 ***
diagn4AD            4.2876832  0.1137643  37.689  < 2e-16 ***
GenderMale          0.2562140  0.0755249   3.392 0.000693 ***
parac              -0.1702044  0.0844444  -2.016 0.043844 *  
M:diagn2EMCI       -0.0038149  0.0007004  -5.447 5.13e-08 ***
M:diagn3LMCI        0.0065412  0.0005278  12.393  < 2e-16 ***
M:diagn4AD          0.0372989  0.0020118  18.540  < 2e-16 ***
APOE41:M            0.0107746  0.0005094  21.151  < 2e-16 ***
APOE42:M            0.0206987  0.0010071  20.553  < 2e-16 ***
M:GenderMale       -0.0059206  0.0004794 -12.350  < 2e-16 ***
M:edu.cat2tertiary -0.0047889  0.0005639  -8.492  < 2e-16 ***
M:edu.cat3mid      -0.0024048  0.0006418  -3.747 0.000179 ***
M:edu.cat4early    -0.0011322  0.0007393  -1.531 0.125654    
M:parac             0.0016974  0.0004744   3.578 0.000346 ***
---
Signif. codes:  0 '***' 0.001 '**' 0.01 '*' 0.05 '.' 0.1 ' ' 1


Correlation matrix not shown by default, as p = 23 > 12.
Use print(x, correlation=TRUE)  or
    vcov(x)        if you need it

convergence code: 0
Model failed to converge with max|grad| = 0.00327936 (tol = 0.001, component 1)
Model is nearly unidentifiable: very large eigenvalue
 - Rescale variables?
Model is nearly unidentifiable: large eigenvalue ratio
 - Rescale variables?

glmerB4.ADAS<-glmer(cbind(success,fail)~Gender+AGE.Z + APOE4  + M.Z + edu.cat + 
diagn +Ibu+vasc*M.Z+APOE4*M.Z+edu.cat*M.Z+diagn*M.Z+ parac*M.Z+diclo*M.Z+(1|ID), 
family="binomial", data=ADASdata)
summary(glmerB4.ADAS)

Generalized linear mixed model fit by maximum likelihood (Laplace
  Approximation) [glmerMod]
 Family: binomial  ( logit )
Formula: cbind(success, fail) ~ Gender + AGE.Z + APOE4 + M.Z + edu.cat +  
    diagn + Ibu + vasc * M.Z + APOE4 * M.Z + edu.cat * M.Z +  
    diagn * M.Z + parac * M.Z + diclo * M.Z + (1 | ID)
   Data: ADASdata

     AIC      BIC   logLik deviance df.resid 
 86910.5  87108.7 -43427.2  86854.5     8742 

Scaled residuals: 
     Min       1Q   Median       3Q      Max 
-16.6848  -0.9937   0.1190   1.1622   8.4376 

Random effects:
 Groups Name        Variance Std.Dev.
 ID     (Intercept) 0.2908   0.5393  
Number of obs: 8770, groups:  ID, 1618

Fixed effects:
                      Estimate Std. Error z value Pr(>|z|)
```

```
(Intercept)           2.317997   0.041776  55.486  < 2e-16 ***
GenderMale           -0.066337   0.028424  -2.334 0.019603 *
AGE.Z                -0.111977   0.013838  -8.092 5.88e-16 ***
APOE41               -0.232049   0.030131  -7.701 1.35e-14 ***
APOE42               -0.359863   0.048284  -7.453 9.12e-14 ***
M.Z                  -0.208753   0.005768 -36.194  < 2e-16 ***
edu.cat2tertiary     -0.045966   0.034533  -1.331 0.183167
edu.cat3mid          -0.126402   0.039105  -3.232 0.001228 **
edu.cat4early        -0.164147   0.041543  -3.951 7.78e-05 ***
diagn2EMCI           -0.354494   0.042178  -8.405  < 2e-16 ***
diagn3LMCI           -0.946907   0.036596 -25.874  < 2e-16 ***
diagn4AD             -1.866494   0.043861 -42.554  < 2e-16 ***
Ibu                   0.121652   0.037795   3.219 0.001288 **
vasc                 -0.003462   0.028361  -0.122 0.902840
parac                 0.036858   0.032043   1.150 0.250030
diclo                 0.272056   0.101190   2.689 0.007176 **
M.Z:vasc              0.015028   0.004691   3.203 0.001358 **
APOE41:M.Z           -0.126348   0.004783 -26.414  < 2e-16 ***
APOE42:M.Z           -0.210659   0.008593 -24.515  < 2e-16 ***
M.Z:edu.cat2tertiary  0.075455   0.005526  13.655  < 2e-16 ***
M.Z:edu.cat3mid       0.032276   0.006027   5.355 8.56e-08 ***
M.Z:edu.cat4early     0.025597   0.006626   3.863 0.000112 ***
M.Z:diagn2EMCI        0.052859   0.007115   7.429 1.09e-13 ***
M.Z:diagn3LMCI       -0.036067   0.005049  -7.143 9.10e-13 ***
M.Z:diagn4AD         -0.299317   0.014833 -20.179  < 2e-16 ***
M.Z:parac            -0.025517   0.004600  -5.547 2.91e-08 ***
M.Z:diclo             0.057155   0.012227   4.675 2.95e-06 ***
---
Signif. codes:  0 '***' 0.001 '**' 0.01 '*' 0.05 '.' 0.1 ' ' 1


Correlation matrix not shown by default, as p = 27 > 12.
Use print(x, correlation=TRUE)  or
    vcov(x)        if you need it

convergence code: 0
Model failed to converge with max|grad| = 0.00565363 (tol = 0.001, component 1)

glmerB5.ADAS<-glmer(cbind(success,fail)~Gender+AGE.Z + APOE4  + M.Z + edu.cat +
diagn +Ibu+vasc*M.Z+APOE4*M.Z+edu.cat*M.Z+diagn*M.Z+ parac*M.Z+diclo*M.Z+(1|ID),
family=binomial(link=probit), data=ADASdata)
summary(glmerB5.ADAS)

Generalized linear mixed model fit by maximum likelihood (Laplace
  Approximation) [glmerMod]
 Family: binomial  ( probit )
Formula: cbind(success, fail) ~ Gender + AGE.Z + APOE4 + M.Z + edu.cat +
    diagn + Ibu + vasc * M.Z + APOE4 * M.Z + edu.cat * M.Z +
    diagn * M.Z + parac * M.Z + diclo * M.Z + (1 | ID)
   Data: ADASdata

     AIC      BIC   logLik deviance df.resid
 87796.6  87994.8 -43870.3  87740.6     8742

Scaled residuals:
     Min       1Q   Median       3Q      Max
-17.4783  -0.9872   0.1231   1.1758   8.6981

Random effects:
 Groups Name        Variance Std.Dev.
```

```
 ID     (Intercept) 0.08872  0.2979
Number of obs: 8770, groups:  ID, 1618

Fixed effects:
                      Estimate Std. Error z value Pr(>|z|)
(Intercept)           1.334735   0.023035  57.943  < 2e-16 ***
GenderMale           -0.031488   0.015693  -2.006 0.044808 *
AGE.Z                -0.058526   0.007642  -7.658 1.89e-14 ***
APOE41               -0.130594   0.016642  -7.847 4.25e-15 ***
APOE42               -0.201550   0.026700  -7.549 4.40e-14 ***
M.Z                  -0.102399   0.003109 -32.940  < 2e-16 ***
edu.cat2tertiary     -0.021791   0.019067  -1.143 0.253093
edu.cat3mid          -0.067495   0.021591  -3.126 0.001771 **
edu.cat4early        -0.085885   0.022956  -3.741 0.000183 ***
diagn2EMCI           -0.186314   0.023256  -8.011 1.13e-15 ***
diagn3LMCI           -0.521676   0.020190 -25.838  < 2e-16 ***
diagn4AD             -1.075500   0.024385 -44.105  < 2e-16 ***
Ibu                   0.063996   0.020854   3.069 0.002149 **
vasc                 -0.002721   0.015663  -0.174 0.862101
parac                 0.020542   0.017693   1.161 0.245628
diclo                 0.142688   0.055782   2.558 0.010528 *
M.Z:vasc              0.006965   0.002586   2.694 0.007067 **
APOE41:M.Z           -0.070922   0.002663 -26.637  < 2e-16 ***
APOE42:M.Z           -0.124893   0.004954 -25.210  < 2e-16 ***
M.Z:edu.cat2tertiary  0.036534   0.003030  12.057  < 2e-16 ***
M.Z:edu.cat3mid       0.013055   0.003318   3.934 8.35e-05 ***
M.Z:edu.cat4early     0.008148   0.003740   2.179 0.029338 *
M.Z:diagn2EMCI        0.026894   0.003847   6.991 2.72e-12 ***
M.Z:diagn3LMCI       -0.029095   0.002772 -10.497  < 2e-16 ***
M.Z:diagn4AD         -0.202869   0.008981 -22.589  < 2e-16 ***
M.Z:parac            -0.014531   0.002544  -5.713 1.11e-08 ***
M.Z:diclo             0.028403   0.006619   4.291 1.78e-05 ***
---
Signif. codes:  0 '***' 0.001 '**' 0.01 '*' 0.05 '.' 0.1 ' ' 1


Correlation matrix not shown by default, as p = 27 > 12.
Use print(x, correlation=TRUE)  or
    vcov(x)        if you need it

convergence code: 0
Model failed to converge with max|grad| = 0.00358838 (tol = 0.001, component 1)
Model is nearly unidentifiable: very large eigenvalue
 - Rescale variables?

glmerB6.ADAS<-glmer(cbind(success,fail)~Gender+AGE.Z + APOE4  + M.Z + edu.cat +
diagn +Ibu+vasc*M.Z+APOE4*M.Z+edu.cat*M.Z+diagn*M.Z+ parac*M.Z+diclo*M.Z+(1|ID),
family=binomial(link=cloglog), data=ADASdata)
summary(glmerB6.ADAS)

Generalized linear mixed model fit by maximum likelihood (Laplace
  Approximation) [glmerMod]
 Family: binomial  ( cloglog )
Formula: cbind(success, fail) ~ Gender + AGE.Z + APOE4 + M.Z + edu.cat +
    diagn + Ibu + vasc * M.Z + APOE4 * M.Z + edu.cat * M.Z +
    diagn * M.Z + parac * M.Z + diclo * M.Z + (1 | ID)
   Data: ADASdata

     AIC      BIC   logLik deviance df.resid
 89575.2  89773.4 -44759.6  89519.2     8742
```

```
Scaled residuals: 
     Min       1Q   Median       3Q      Max 
-18.7825  -0.9973   0.1241   1.1907   8.9898 

Random effects:
 Groups Name        Variance Std.Dev.
 ID     (Intercept) 0.06751  0.2598  
Number of obs: 8770, groups:  ID, 1618

Fixed effects:
                       Estimate Std. Error z value Pr(>|z|)    
(Intercept)           0.8680430  0.0200566  43.280  < 2e-16 ***
GenderMale           -0.0211095  0.0136873  -1.542  0.12301    
AGE.Z                -0.0455022  0.0066686  -6.823 8.89e-12 ***
APOE41               -0.1129678  0.0145202  -7.780 7.25e-15 ***
APOE42               -0.1726452  0.0233390  -7.397 1.39e-13 ***
M.Z                  -0.0716609  0.0025194 -28.444  < 2e-16 ***
edu.cat2tertiary     -0.0154598  0.0166278  -0.930  0.35250    
edu.cat3mid          -0.0559166  0.0188305  -2.969  0.00298 ** 
edu.cat4early        -0.0674700  0.0200391  -3.367  0.00076 ***
diagn2EMCI           -0.1467235  0.0202381  -7.250 4.17e-13 ***
diagn3LMCI           -0.4398379  0.0175864 -25.010  < 2e-16 ***
diagn4AD             -0.9831020  0.0216044 -45.505  < 2e-16 ***
Ibu                   0.0522215  0.0181728   2.874  0.00406 ** 
vasc                 -0.0030391  0.0136641  -0.222  0.82399    
parac                 0.0176447  0.0154307   1.143  0.25284    
diclo                 0.1138669  0.0485652   2.345  0.01905 *  
M.Z:vasc              0.0038032  0.0021543   1.765  0.07750 .  
APOE41:M.Z           -0.0580470  0.0022576 -25.712  < 2e-16 ***
APOE42:M.Z           -0.1139255  0.0045008 -25.312  < 2e-16 ***
M.Z:edu.cat2tertiary  0.0237946  0.0025051   9.499  < 2e-16 ***
M.Z:edu.cat3mid       0.0052721  0.0027629   1.908  0.05637 .  
M.Z:edu.cat4early    -0.0005197  0.0032470  -0.160  0.87285    
M.Z:diagn2EMCI        0.0210330  0.0031085   6.766 1.32e-11 ***
M.Z:diagn3LMCI       -0.0317835  0.0023196 -13.702  < 2e-16 ***
M.Z:diagn4AD         -0.2265862  0.0091396 -24.792  < 2e-16 ***
M.Z:parac            -0.0123593  0.0021309  -5.800 6.63e-09 ***
M.Z:diclo             0.0195741  0.0053735   3.643  0.00027 ***
---
Signif. codes:  0 '***' 0.001 '**' 0.01 '*' 0.05 '.' 0.1 ' ' 1


Correlation matrix not shown by default, as p = 27 > 12.
Use print(x, correlation=TRUE)  or
    vcov(x)        if you need it

convergence code: 0
Model failed to converge with max|grad| = 0.00204316 (tol = 0.001, component 1)
Model is nearly unidentifiable: very large eigenvalue
 - Rescale variables?

glmerP3.ADAS<-glmer(neg.b.ADAS~Gender+AGE.Z + APOE4  + M.Z + edu.cat + diagn
+Ibu+vasc*M.Z+APOE4*M.Z+edu.cat*M.Z+diagn*M.Z+ parac*M.Z+diclo*M.Z+(1|ID),
family="poisson", data=ADASdata)
summary(glmerP3.ADAS)

Generalized linear mixed model fit by maximum likelihood (Laplace
  Approximation) [glmerMod]
 Family: poisson  ( log )
```

```
Formula: neg.b.ADAS ~ Gender + AGE.Z + APOE4 + M.Z + edu.cat + diagn +
    Ibu + vasc * M.Z + APOE4 * M.Z + edu.cat * M.Z + diagn *
    M.Z + parac * M.Z + diclo * M.Z + (1 | ID)
   Data: ADASdata

     AIC      BIC   logLik deviance df.resid
 79378.9  79577.2 -39661.5  79322.9     8742

Scaled residuals:
    Min      1Q  Median      3Q     Max
-5.5233 -1.0233 -0.1020  0.8611 12.6186

Random effects:
 Groups Name        Variance Std.Dev.
 ID     (Intercept) 0.1834   0.4283
Number of obs: 8770, groups:  ID, 1618

Fixed effects:
                       Estimate Std. Error z value Pr(>|z|)
(Intercept)           3.1763076  0.0333559  95.225  < 2e-16 ***
GenderMale            0.0662341  0.0226507   2.924 0.003454 **
AGE.Z                 0.0979820  0.0110248   8.887  < 2e-16 ***
APOE41                0.1793677  0.0240067   7.472 7.92e-14 ***
APOE42                0.2828036  0.0384166   7.361 1.82e-13 ***
M.Z                   0.1824930  0.0051668  35.320  < 2e-16 ***
edu.cat2tertiary      0.0476044  0.0275238   1.730 0.083706 .
edu.cat3mid           0.1084255  0.0311681   3.479 0.000504 ***
edu.cat4early         0.1469074  0.0330870   4.440 8.99e-06 ***
diagn2EMCI            0.3075100  0.0336832   9.129  < 2e-16 ***
diagn3LMCI            0.7728421  0.0291934  26.473  < 2e-16 ***
diagn4AD              1.3781395  0.0348366  39.560  < 2e-16 ***
Ibu                  -0.1003625  0.0301300  -3.331 0.000865 ***
vasc                  0.0025883  0.0226012   0.115 0.908824
parac                -0.0250900  0.0255272  -0.983 0.325669
diclo                -0.2318095  0.0807620  -2.870 0.004101 **
M.Z:vasc             -0.0141558  0.0041065  -3.447 0.000566 ***
APOE41:M.Z            0.0851593  0.0041525  20.508  < 2e-16 ***
APOE42:M.Z            0.1336381  0.0071144  18.784  < 2e-16 ***
M.Z:edu.cat2tertiary -0.0561587  0.0048606 -11.554  < 2e-16 ***
M.Z:edu.cat3mid      -0.0264518  0.0052797  -5.010 5.44e-07 ***
M.Z:edu.cat4early    -0.0248495  0.0056470  -4.400 1.08e-05 ***
M.Z:diagn2EMCI       -0.0456143  0.0064436  -7.079 1.45e-12 ***
M.Z:diagn3LMCI        0.0002083  0.0044827   0.046 0.962931
M.Z:diagn4AD          0.1274495  0.0115809  11.005  < 2e-16 ***
M.Z:parac             0.0194996  0.0040162   4.855 1.20e-06 ***
M.Z:diclo            -0.0442464  0.0107816  -4.104 4.06e-05 ***
---
Signif. codes:  0 '***' 0.001 '**' 0.01 '*' 0.05 '.' 0.1 ' ' 1


Correlation matrix not shown by default, as p = 27 > 12.
Use print(x, correlation=TRUE)  or
    vcov(x)        if you need it

convergence code: 0
Model failed to converge with max|grad| = 0.00943841 (tol = 0.001, component 1)
Model is nearly unidentifiable: very large eigenvalue
 - Rescale variables?
```

```
glmerP4.ADAS<-glmer(neg.b.ADAS~Gender+AGE.Z + APOE4  + M.Z + edu.cat + diagn
+Ibu+vasc*M.Z+APOE4*M.Z+edu.cat*M.Z+diagn*M.Z+ parac*M.Z+diclo*M.Z+(1|ID),
family=poisson(link=sqrt), data=ADASdata)
summary(glmerP4.ADAS)

Generalized linear mixed model fit by maximum likelihood (Laplace
  Approximation) [glmerMod]
 Family: poisson  ( sqrt )
Formula: neg.b.ADAS ~ Gender + AGE.Z + APOE4 + M.Z + edu.cat + diagn +
    Ibu + vasc * M.Z + APOE4 * M.Z + edu.cat * M.Z + diagn *
    M.Z + parac * M.Z + diclo * M.Z + (1 | ID)
   Data: ADASdata

     AIC      BIC   logLik deviance df.resid
 81416.9  81615.1 -40680.5  81360.9     8742

Scaled residuals:
    Min      1Q  Median      3Q     Max
-5.7176 -1.0466 -0.1080  0.8703 15.2534

Random effects:
 Groups Name        Variance Std.Dev.
 ID     (Intercept) 2.002    1.415
Number of obs: 8770, groups:  ID, 1618

Fixed effects:
                      Estimate Std. Error z value Pr(>|z|)
(Intercept)           4.998461   0.109610  45.602  < 2e-16 ***
GenderMale            0.141698   0.074807   1.894 0.058200 .
AGE.Z                 0.273511   0.036451   7.503 6.21e-14 ***
APOE41                0.632433   0.079403   7.965 1.65e-15 ***
APOE42                0.978901   0.127737   7.663 1.81e-14 ***
M.Z                   0.400042   0.015760  25.383  < 2e-16 ***
edu.cat2tertiary      0.108196   0.090911   1.190 0.233995
edu.cat3mid           0.328681   0.102942   3.193 0.001409 **
edu.cat4early         0.420100   0.109653   3.831 0.000128 ***
diagn2EMCI            0.862966   0.110604   7.802 6.08e-15 ***
diagn3LMCI            2.525032   0.096097  26.276  < 2e-16 ***
diagn4AD              5.276834   0.119346  44.215  < 2e-16 ***
Ibu                  -0.288944   0.099249  -2.911 0.003599 **
vasc                  0.020287   0.074729   0.271 0.786027
parac                -0.084413   0.084311  -1.001 0.316723
diclo                -0.645964   0.265136  -2.436 0.014836 *
M.Z:vasc             -0.020469   0.013541  -1.512 0.130637
APOE41:M.Z            0.301097   0.014223  21.170  < 2e-16 ***
APOE42:M.Z            0.570816   0.028083  20.326  < 2e-16 ***
M.Z:edu.cat2tertiary -0.116061   0.015694  -7.395 1.41e-13 ***
M.Z:edu.cat3mid      -0.016593   0.017390  -0.954 0.340000
M.Z:edu.cat4early     0.009033   0.020486   0.441 0.659259
M.Z:diagn2EMCI       -0.119550   0.019624  -6.092 1.12e-09 ***
M.Z:diagn3LMCI        0.153835   0.014642  10.507  < 2e-16 ***
M.Z:diagn4AD          1.027864   0.056150  18.306  < 2e-16 ***
M.Z:parac             0.068119   0.013404   5.082 3.73e-07 ***
M.Z:diclo            -0.109573   0.033022  -3.318 0.000906 ***
---
Signif. codes:  0 '***' 0.001 '**' 0.01 '*' 0.05 '.' 0.1 ' ' 1


Correlation matrix not shown by default, as p = 27 > 12.
```

```
Use print(x, correlation=TRUE)  or
    vcov(x)        if you need it

lme1.ADAS<-lmer(neg.b.ADAS~Gender+AGE.Z + APOE4  + M.Z + edu.cat + diagn
+Ibu+vasc*M.Z+APOE4*M.Z+edu.cat*M.Z+diagn*M.Z+ parac*M.Z+diclo*M.Z+(1|ID),
data=ADASdata)
summary(lme1.ADAS)

Linear mixed model fit by REML. t-tests use Satterthwaite's method [
lmerModLmerTest]
Formula: neg.b.ADAS ~ Gender + AGE.Z + APOE4 + M.Z + edu.cat + diagn +
    Ibu + vasc * M.Z + APOE4 * M.Z + edu.cat * M.Z + diagn *
    M.Z + parac * M.Z + diclo * M.Z + (1 | ID)
   Data: ADASdata

REML criterion at convergence: 75937.9

Scaled residuals:
    Min      1Q  Median      3Q     Max
-4.8445 -0.5021 -0.0536  0.4103 10.3239

Random effects:
 Groups   Name        Variance Std.Dev.
 ID       (Intercept) 447.5    21.15
 Residual             219.0    14.80
Number of obs: 8770, groups:  ID, 1618

Fixed effects:
                      Estimate Std. Error        df t value Pr(>|t|)
(Intercept)            26.3933     1.7044 1580.4602  15.486  < 2e-16 ***
GenderMale              0.5638     1.1646 1589.9135   0.484  0.62837
AGE.Z                   3.1113     0.5690 1613.1164   5.468 5.28e-08 ***
APOE41                  9.8063     1.2406 1618.1943   7.904 4.94e-15 ***
APOE42                 14.8817     2.0136 1696.9022   7.391 2.28e-13 ***
M.Z                     4.7134     0.4696 7506.2591  10.038  < 2e-16 ***
edu.cat2tertiary        0.4909     1.4176 1602.9043   0.346  0.72918
edu.cat3mid             3.9557     1.6056 1603.7933   2.464  0.01386 *
edu.cat4early           4.4706     1.7189 1646.4989   2.601  0.00938 **
diagn2EMCI             10.4598     1.7182 1572.9664   6.087 1.44e-09 ***
diagn3LMCI             35.4604     1.4898 1555.9607  23.803  < 2e-16 ***
diagn4AD               86.7603     2.1060 3102.7196  41.197  < 2e-16 ***
Ibu                    -3.5383     1.5363 1544.1928  -2.303  0.02141 *
vasc                    0.3143     1.1684 1624.8110   0.269  0.78800
parac                  -1.5085     1.3083 1564.2552  -1.153  0.24910
diclo                  -7.9435     4.0885 1516.5223  -1.943  0.05221 .
M.Z:vasc               -0.3041     0.4023 7544.6768  -0.756  0.44978
APOE41:M.Z              5.8069     0.4216 7533.5705  13.775  < 2e-16 ***
APOE42:M.Z             11.4991     0.8356 7558.1021  13.761  < 2e-16 ***
M.Z:edu.cat2tertiary   -2.0792     0.4667 7531.5264  -4.455 8.50e-06 ***
M.Z:edu.cat3mid        -0.2566     0.5163 7533.2699  -0.497  0.61917
M.Z:edu.cat4early       0.2448     0.6046 7554.9570   0.405  0.68560
M.Z:diagn2EMCI         -1.1623     0.5839 7405.8638  -1.991  0.04656 *
M.Z:diagn3LMCI          4.5970     0.4341 7471.7748  10.591  < 2e-16 ***
M.Z:diagn4AD           24.1367     1.6434 7509.4826  14.687  < 2e-16 ***
M.Z:parac               1.1776     0.3985 7493.0388   2.955  0.00313 **
M.Z:diclo              -1.8291     0.9892 7447.9361  -1.849  0.06450 .
---
Signif. codes:  0 '***' 0.001 '**' 0.01 '*' 0.05 '.' 0.1 ' ' 1
```

```
Correlation matrix not shown by default, as p = 27 > 12.
Use print(x, correlation=TRUE)  or
    vcov(x)        if you need it

mcp.fnc(lme1.ADAS)
```

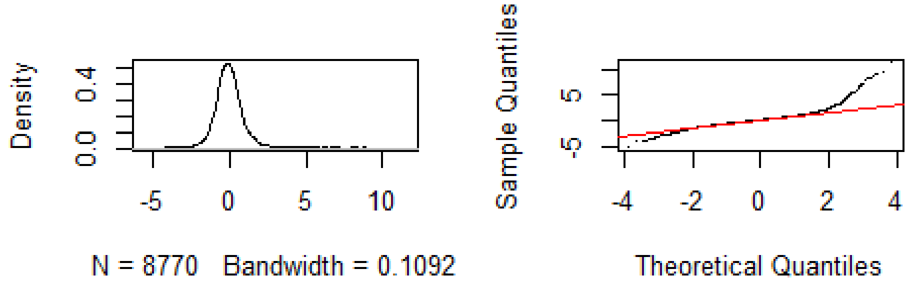

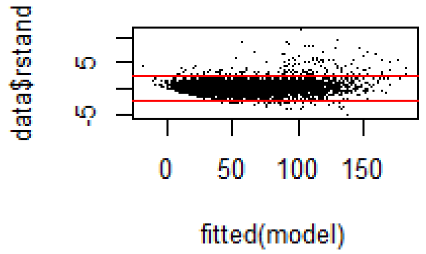


```
lme2.ADAS<-lmer(neg.b.ADAS~AGE + APOE4  + M + edu.cat + diagn+ diagn*M +APOE4*M
+ Gender*M+ edu.cat*M +parac*M +(1|ID), data=ADASdata)
summary(lme2.ADAS)

Linear mixed model fit by REML. t-tests use Satterthwaite's method [
lmerModLmerTest]
Formula: neg.b.ADAS ~ AGE + APOE4 + M + edu.cat + diagn + diagn * M +
    APOE4 * M + Gender * M + edu.cat * M + parac * M + (1 | ID)
   Data: ADASdata

REML criterion at convergence: 75993.8

Scaled residuals:
    Min      1Q  Median      3Q     Max
-4.7650 -0.5008 -0.0536  0.4129 10.6414

Random effects:
 Groups   Name        Variance Std.Dev.
 ID       (Intercept) 450.2    21.22
 Residual             217.7    14.75
Number of obs: 8770, groups:  ID, 1618

Fixed effects:
                     Estimate Std. Error         df t value Pr(>|t|)
(Intercept)          20.11950    1.60355 1758.61106  12.547  < 2e-16 ***
AGE                   0.45444    0.08083 1615.32086   5.622 2.22e-08 ***
APOE41                4.14735    1.26995 1784.02940   3.266  0.00111 **
```

```
APOE42                 3.57406    2.04310 1807.15622   1.749  0.08040 .
M                      0.22253    0.01699 7484.46096  13.101  < 2e-16 ***
edu.cat2tertiary       2.61060    1.45324 1775.39612   1.796  0.07260 .
edu.cat3mid            4.80390    1.64710 1777.81494   2.917  0.00358 **
edu.cat4early          4.52399    1.75003 1790.69488   2.585  0.00981 **
diagn2EMCI            11.61679    1.77508 1794.71041   6.544 7.77e-11 ***
diagn3LMCI            30.79396    1.52912 1738.60307  20.138  < 2e-16 ***
diagn4AD              63.14219    1.83641 1982.96086  34.383  < 2e-16 ***
GenderMale             2.47587    1.19637 1782.83688   2.069  0.03864 *
parac                 -2.82513    1.33194 1743.99334  -2.121  0.03406 *
M:diagn2EMCI          -0.03399    0.02078 7403.06281  -1.636  0.10198
M:diagn3LMCI           0.18147    0.01561 7468.33024  11.629  < 2e-16 ***
M:diagn4AD             0.87327    0.05870 7506.71607  14.876  < 2e-16 ***
APOE41:M               0.20818    0.01506 7530.39924  13.827  < 2e-16 ***
APOE42:M               0.41594    0.02987 7554.27140  13.926  < 2e-16 ***
M:GenderMale          -0.09588    0.01418 7529.62994  -6.764 1.45e-11 ***
M:edu.cat2tertiary    -0.08466    0.01672 7528.70833  -5.065 4.19e-07 ***
M:edu.cat3mid         -0.03869    0.01901 7526.61307  -2.036  0.04181 *
M:edu.cat4early       -0.01513    0.02175 7549.86972  -0.696  0.48663
M:parac                0.03057    0.01406 7485.96486   2.174  0.02971 *
---
Signif. codes:  0 '***' 0.001 '**' 0.01 '*' 0.05 '.' 0.1 ' ' 1


Correlation matrix not shown by default, as p = 23 > 12.
Use print(x, correlation=TRUE)  or
    vcov(x)        if you need it

mcp.fnc(lme2.ADAS)
```

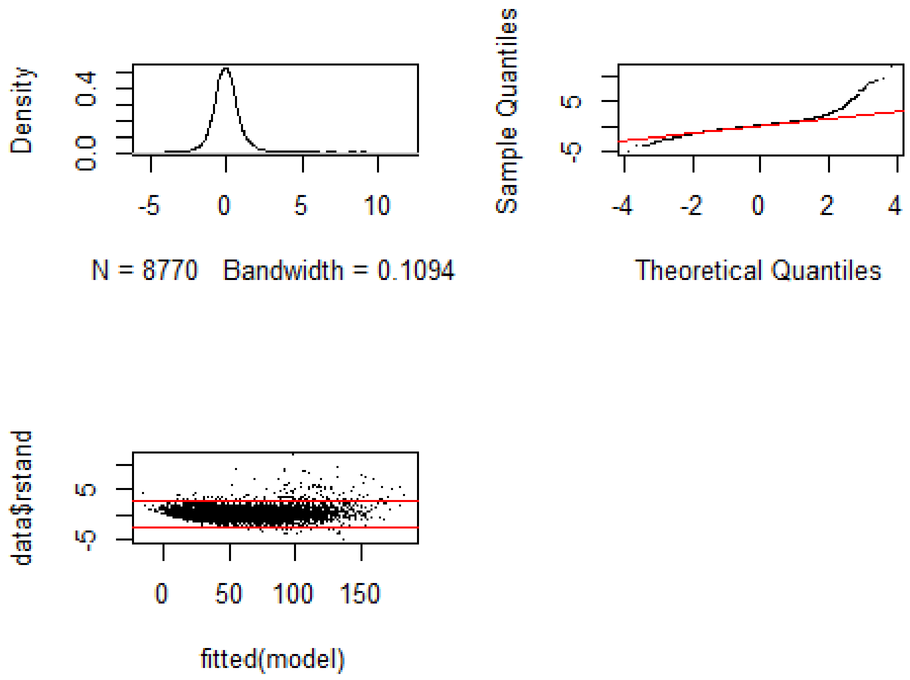


```
neg.binom.zero.ADAS<-glmmadmb(neg.b.ADAS~AGE + APOE4  + M + edu.cat + diagn+
diagn*M +APOE4*M + Gender*M+ edu.cat*M +parac*M +(1|ID), family="nbinom1",
data=ADASdata, zeroInflation=TRUE)
anova(neg.binom.zero.ADAS, neg.ADAS)
```

```
Analysis of Deviance Table

Model 1: neg.b.ADAS ~ AGE + APOE4 + M + edu.cat + diagn + diagn * M + APOE4 * M
+ Gender * M + edu.cat * M + parac * M
Model 2: neg.b.ADAS ~ M + AGE + APOE4 + Gender + edu.cat + diagn + headache +
diclo + Ibu + edu.cat * M + diagn * M + APOE4 * M + Gender * M + parac * M
  NoPar LogLik Df Deviance Pr(>Chi)
1    26 -36013
2    28 -36036  2    -44.4        1

summary(neg.binom.zero.ADAS)


Call:
glmmadmb(formula = neg.b.ADAS ~ AGE + APOE4 + M + edu.cat + diagn +
    diagn * M + APOE4 * M + Gender * M + edu.cat * M + parac *
    M + (1 | ID), data = ADASdata, family = "nbinom1", zeroInflation = TRUE)

AIC: 72078.6

Coefficients:
                    Estimate Std. Error z value Pr(>|z|)
(Intercept)         2.961948   0.031862   92.96  < 2e-16 ***
AGE                 0.013958   0.001554    8.98  < 2e-16 ***
APOE41              0.099600   0.024471    4.07  4.7e-05 ***
APOE42              0.152828   0.038731    3.95  8.0e-05 ***
M                   0.007271   0.000336   21.64  < 2e-16 ***
edu.cat2tertiary    0.100668   0.028156    3.58  0.00035 ***
edu.cat3mid         0.151274   0.031902    4.74  2.1e-06 ***
edu.cat4early       0.173064   0.033504    5.17  2.4e-07 ***
diagn2EMCI          0.359679   0.035203   10.22  < 2e-16 ***
diagn3LMCI          0.773107   0.030031   25.74  < 2e-16 ***
diagn4AD            1.258076   0.034715   36.24  < 2e-16 ***
GenderMale          0.114745   0.023115    4.96  6.9e-07 ***
parac              -0.051078   0.025790   -1.98  0.04764 *
M:diagn2EMCI       -0.001707   0.000440   -3.88  0.00010 ***
M:diagn3LMCI        0.000306   0.000283    1.08  0.27942
M:diagn4AD          0.004653   0.000713    6.53  6.7e-11 ***
APOE41:M            0.002942   0.000264   11.16  < 2e-16 ***
APOE42:M            0.004875   0.000443   11.01  < 2e-16 ***
M:GenderMale       -0.002328   0.000254   -9.16  < 2e-16 ***
M:edu.cat2tertiary -0.001998   0.000309   -6.46  1.0e-10 ***
M:edu.cat3mid      -0.001585   0.000349   -4.54  5.6e-06 ***
M:edu.cat4early    -0.001226   0.000358   -3.43  0.00061 ***
M:parac             0.000610   0.000248    2.46  0.01408 *
---
Signif. codes:  0 '***' 0.001 '**' 0.01 '*' 0.05 '.' 0.1 ' ' 1

Number of observations: total=8770, ID=1618
Random effect variance(s):
Group=ID
            Variance StdDev
(Intercept)   0.1711 0.4136

Negative binomial dispersion parameter: 2.9913 (std. err.: 0.056385)
Zero-inflation: 9.1282e-05  (std. err.:  1.3381e-05 )

Log-likelihood: -36013.3

summary(neg.ADAS)
```

```
Call:
glmmadmb(formula = neg.b.ADAS ~ M + AGE + APOE4 + Gender + edu.cat +
    diagn + headache + diclo + Ibu + edu.cat * M + diagn * M +
    APOE4 * M + Gender * M + parac * M + (1 | ID), data = ADASdata,
    family = "nbinom1")

AIC: 72127

Coefficients:
                    Estimate Std. Error z value Pr(>|z|)
(Intercept)         2.991271   0.032483   92.09  < 2e-16 ***
M                   0.007364   0.000339   21.75  < 2e-16 ***
AGE                 0.013316   0.001562    8.52  < 2e-16 ***
APOE41              0.095729   0.024424    3.92  8.9e-05 ***
APOE42              0.149163   0.038638    3.86  0.00011 ***
GenderMale          0.106536   0.023147    4.60  4.2e-06 ***
edu.cat2tertiary    0.104722   0.028117    3.72  0.00020 ***
edu.cat3mid         0.148404   0.031842    4.66  3.2e-06 ***
edu.cat4early       0.180975   0.033458    5.41  6.3e-08 ***
diagn2EMCI          0.357465   0.035112   10.18  < 2e-16 ***
diagn3LMCI          0.771174   0.030017   25.69  < 2e-16 ***
diagn4AD            1.247515   0.034807   35.84  < 2e-16 ***
headache           -0.087110   0.039032   -2.23  0.02563 *
diclo              -0.224372   0.080266   -2.80  0.00518 **
Ibu                -0.093702   0.029852   -3.14  0.00170 **
parac              -0.035014   0.025971   -1.35  0.17759
M:edu.cat2tertiary -0.002137   0.000309   -6.91  4.7e-12 ***
M:edu.cat3mid      -0.001490   0.000348   -4.29  1.8e-05 ***
M:edu.cat4early    -0.001348   0.000361   -3.74  0.00019 ***
M:diagn2EMCI       -0.001629   0.000409   -3.98  6.8e-05 ***
M:diagn3LMCI        0.000210   0.000286    0.74  0.46177
M:diagn4AD          0.004483   0.000726    6.17  6.8e-10 ***
M:APOE41            0.002980   0.000263   11.31  < 2e-16 ***
M:APOE42            0.004757   0.000448   10.61  < 2e-16 ***
M:GenderMale       -0.002098   0.000255   -8.22  < 2e-16 ***
M:parac             0.000555   0.000251    2.21  0.02703 *
---
Signif. codes:  0 '***' 0.001 '**' 0.01 '*' 0.05 '.' 0.1 ' ' 1

Number of observations: total=8770, ID=1618
Random effect variance(s):
Group=ID
            Variance StdDev
(Intercept)   0.1693 0.4114

Negative binomial dispersion parameter: 3.1196 (std. err.: 0.052844)

Log-likelihood: -36035.5

neg.binom.link.parameterisation.zero.ADAS<-glmmadmb(neg.b.ADAS~AGE + APOE4  + M
+ edu.cat + diagn+ diagn*M +APOE4*M + Gender*M+ edu.cat*M +parac*M +(1|ID),
family="nbinom", data=ADASdata)
summary(neg.binom.link.parameterisation.zero.ADAS)


Call:
glmmadmb(formula = neg.b.ADAS ~ AGE + APOE4 + M + edu.cat + diagn +
    diagn * M + APOE4 * M + Gender * M + edu.cat * M + parac *
```

```
    M + (1 | ID), data = ADASdata, family = "nbinom")

AIC: 72923.4

Coefficients:
                     Estimate Std. Error z value Pr(>|z|)
(Intercept)          2.98e+00   3.13e-02   95.31  < 2e-16 ***
AGE                  1.42e-02   1.56e-03    9.11  < 2e-16 ***
APOE41               1.08e-01   2.45e-02    4.40  1.1e-05 ***
APOE42               1.59e-01   3.92e-02    4.06  4.8e-05 ***
M                    6.40e-03   3.16e-04   20.24  < 2e-16 ***
edu.cat2tertiary     8.67e-02   2.81e-02    3.08   0.0021 **
edu.cat3mid          1.36e-01   3.19e-02    4.27  2.0e-05 ***
edu.cat4early        1.60e-01   3.37e-02    4.75  2.0e-06 ***
diagn2EMCI           3.55e-01   3.46e-02   10.27  < 2e-16 ***
diagn3LMCI           7.76e-01   2.97e-02   26.09  < 2e-16 ***
diagn4AD             1.25e+00   3.51e-02   35.64  < 2e-16 ***
GenderMale           1.08e-01   2.31e-02    4.66  3.2e-06 ***
parac               -4.69e-02   2.58e-02   -1.82   0.0692 .
M:diagn2EMCI        -1.69e-03   3.80e-04   -4.45  8.8e-06 ***
M:diagn3LMCI        -8.93e-05   2.78e-04   -0.32   0.7484
M:diagn4AD           4.58e-03   9.48e-04    4.83  1.3e-06 ***
APOE41:M             2.54e-03   2.67e-04    9.50  < 2e-16 ***
APOE42:M             4.62e-03   5.03e-04    9.20  < 2e-16 ***
M:GenderMale        -1.78e-03   2.55e-04   -6.98  3.0e-12 ***
M:edu.cat2tertiary -1.47e-03   3.02e-04   -4.89  1.0e-06 ***
M:edu.cat3mid       -9.61e-04   3.41e-04   -2.82   0.0048 **
M:edu.cat4early     -5.49e-04   3.81e-04   -1.44   0.1498
M:parac              5.43e-04   2.51e-04    2.16   0.0305 *
---
Signif. codes:  0 '***' 0.001 '**' 0.01 '*' 0.05 '.' 0.1 ' ' 1

Number of observations: total=8770, ID=1618
Random effect variance(s):
Group=ID
            Variance StdDev
(Intercept)   0.1721 0.4149

Negative binomial dispersion parameter: 22.451 (std. err.: 0.62103)

Log-likelihood: -36436.7

AIC.ADAS<-c(
  AIC(logLik(neg.ADAS)),
  AIC(logLik(glmerB1.ADAS)),
  AIC(logLik(glmerB2.ADAS)),
  AIC(logLik(glmerB3.ADAS)),
  AIC(logLik(glmerB4.ADAS)),
  AIC(logLik(glmerB5.ADAS)),
  AIC(logLik(glmerB6.ADAS)),
  AIC(logLik(glmerP1.ADAS)),
  AIC(logLik(glmerP2.ADAS)),
  AIC(logLik(glmerP3.ADAS)),
  AIC(logLik(glmerP4.ADAS)),
  AIC(logLik(lme1.ADAS)),
  AIC(logLik(lme2.ADAS)),
  AIC(logLik(neg.binom.zero.ADAS)),
  AIC(logLik(neg.binom.link.parameterisation.zero.ADAS)))
```

```
Model<-c("Negative Binomial", "Binomial (logit)", "Binomial (probit)","Binomial
(Cloglog)","Binomial (logit, centered)", "Binomial (probit, centered)","Binomial
(Cloglog, centered)","Poisson (log)","Poisson (sqrt)","Poisson (log,
centered)","Poisson (sqrt, centered)","MLM (centered)","MLM","Negative binomial
(zero inflated)","Negative binomial (classical parameterisation)")

data.frame(Model,AIC.ADAS)

                                             Model AIC.ADAS
1                                Negative Binomial 72127.00
2                                 Binomial (logit) 86593.11
3                                Binomial (probit) 87510.20
4                               Binomial (Cloglog) 89342.49
5                       Binomial (logit, centered) 86910.45
6                      Binomial (probit, centered) 87796.63
7                     Binomial (Cloglog, centered) 89575.21
8                                    Poisson (log) 79198.62
9                                   Poisson (sqrt) 81283.65
10                         Poisson (log, centered) 79378.94
11                        Poisson (sqrt, centered) 81416.93
12                                  MLM (centered) 75995.89
13                                             MLM 76043.83
14                Negative binomial (zero inflated) 72078.60
15 Negative binomial (classical parameterisation) 72923.40
```

For Review Only

9. Plotting predicted decline for ADAS and MMSE

9.2. *Generating model matrix for each pain medications MMSE*

9.2.1. *Building the models for pain medications use*

```
naproxen<-glmmadmb(neg.b.MMSE~M+AGE+ APOE4+Gender+  edu.cat + diagn+
edu.cat*M+diagn*M+APOE4*M+Gender*M+naprox*M+ (1|ID), family="nbinom1",
data=MMSEdata)

aspirin<-glmmadmb(neg.b.MMSE~M+AGE+ APOE4+Gender+  edu.cat + diagn+
edu.cat*M+diagn*M+APOE4*M+Gender*M+aspirin*M+ (1|ID), family="nbinom1",
data=MMSEdata)

paracetamol<-glmmadmb(neg.b.MMSE~M+AGE+ APOE4+Gender+  edu.cat + diagn+
edu.cat*M+diagn*M+APOE4*M+Gender*M+parac*M+ (1|ID), family="nbinom1",
data=MMSEdata)

celecoxib<-glmmadmb(neg.b.MMSE~M+AGE+ APOE4+Gender+  edu.cat + diagn+
edu.cat*M+diagn*M+APOE4*M+Gender*M+celex*M+ (1|ID), family="nbinom1",
data=MMSEdata)

ibuprofen<-glmmadmb(neg.b.MMSE~M+AGE+ APOE4+Gender+  edu.cat + diagn+
edu.cat*M+diagn*M+APOE4*M+Gender*M+Ibu*M+ (1|ID), family="nbinom1",
data=MMSEdata)

diclofenac<-glmmadmb(neg.b.MMSE~M+AGE+ APOE4+Gender+  edu.cat + diagn+
edu.cat*M+diagn*M+APOE4*M+Gender*M+diclo*M+ (1|ID), family="nbinom1",
data=MMSEdata)

NSAIDS<-glmmadmb(neg.b.MMSE~M+AGE+ APOE4+Gender+  edu.cat + diagn+
edu.cat*M+diagn*M+APOE4*M+Gender*M+NSAID*M+ (1|ID), family="nbinom1",
data=MMSEdata)

Base<-glmmadmb(neg.b.MMSE~M+AGE+ APOE4+Gender+  edu.cat + diagn+
edu.cat*M+diagn*M+APOE4*M+Gender*M+ (1|ID), family="nbinom1", data=MMSEdata)

paracetamol.NSAID<-glmmadmb(neg.b.MMSE~M+AGE+ APOE4+Gender+  edu.cat + diagn+
edu.cat*M+diagn*M+APOE4*M+Gender*M+parac*M+ NSAID*M+(1|ID), family="nbinom1",
data=MMSEdata)

Painrelief<-glmmadmb(neg.b.MMSE~M+AGE+ APOE4+Gender+  edu.cat + diagn+
edu.cat*M+diagn*M+APOE4*M+Gender*M+Painrelief*M+ (1|ID), family="nbinom1",
data=MMSEdata)

anova(naproxen,Base)

Analysis of Deviance Table

Model 1: neg.b.MMSE ~ M + AGE + APOE4 + Gender + edu.cat + diagn + edu.cat * M +
diagn * M + APOE4 * M + Gender * M
Model 2: neg.b.MMSE ~ M + AGE + APOE4 + Gender + edu.cat + diagn + edu.cat * M +
diagn * M + APOE4 * M + Gender * M + naprox * M
  NoPar LogLik Df Deviance Pr(>Chi)
1    23 -16950
2    25 -16950  2      0.2   0.9048

anova(aspirin,Base)
```

```
Analysis of Deviance Table

Model 1: neg.b.MMSE ~ M + AGE + APOE4 + Gender + edu.cat + diagn + edu.cat * M +
diagn * M + APOE4 * M + Gender * M
Model 2: neg.b.MMSE ~ M + AGE + APOE4 + Gender + edu.cat + diagn + edu.cat * M +
diagn * M + APOE4 * M + Gender * M + aspirin * M
  NoPar LogLik Df Deviance Pr(>Chi)
1    23 -16950
2    25 -16946  2      8.8  0.01228 *
---
Signif. codes:  0 '***' 0.001 '**' 0.01 '*' 0.05 '.' 0.1 ' ' 1

anova(paracetamol,Base)

Analysis of Deviance Table

Model 1: neg.b.MMSE ~ M + AGE + APOE4 + Gender + edu.cat + diagn + edu.cat * M +
diagn * M + APOE4 * M + Gender * M
Model 2: neg.b.MMSE ~ M + AGE + APOE4 + Gender + edu.cat + diagn + edu.cat * M +
diagn * M + APOE4 * M + Gender * M + parac * M
  NoPar LogLik Df Deviance Pr(>Chi)
1    23 -16950
2    25 -16947  2      6.2  0.04505 *
---
Signif. codes:  0 '***' 0.001 '**' 0.01 '*' 0.05 '.' 0.1 ' ' 1

anova(celecoxib,Base)

Analysis of Deviance Table

Model 1: neg.b.MMSE ~ M + AGE + APOE4 + Gender + edu.cat + diagn + edu.cat * M +
diagn * M + APOE4 * M + Gender * M
Model 2: neg.b.MMSE ~ M + AGE + APOE4 + Gender + edu.cat + diagn + edu.cat * M +
diagn * M + APOE4 * M + Gender * M + celex * M
  NoPar LogLik Df Deviance Pr(>Chi)
1    23 -16950
2    25 -16948  2      4.6   0.1003

anova(ibuprofen,Base)

Analysis of Deviance Table

Model 1: neg.b.MMSE ~ M + AGE + APOE4 + Gender + edu.cat + diagn + edu.cat * M +
diagn * M + APOE4 * M + Gender * M
Model 2: neg.b.MMSE ~ M + AGE + APOE4 + Gender + edu.cat + diagn + edu.cat * M +
diagn * M + APOE4 * M + Gender * M + Ibu * M
  NoPar LogLik Df Deviance Pr(>Chi)
1    23 -16950
2    25 -16948  2      3.6   0.1653

anova(diclofenac,Base)

Analysis of Deviance Table

Model 1: neg.b.MMSE ~ M + AGE + APOE4 + Gender + edu.cat + diagn + edu.cat * M +
diagn * M + APOE4 * M + Gender * M
Model 2: neg.b.MMSE ~ M + AGE + APOE4 + Gender + edu.cat + diagn + edu.cat * M +
diagn * M + APOE4 * M + Gender * M + diclo * M
  NoPar LogLik Df Deviance Pr(>Chi)
1    23 -16950
```

```
2    25 -16946  2      8.6  0.01357 *
---
Signif. codes:  0 '***' 0.001 '**' 0.01 '*' 0.05 '.' 0.1 ' ' 1

anova(NSAIDS,Base)

Analysis of Deviance Table

Model 1: neg.b.MMSE ~ M + AGE + APOE4 + Gender + edu.cat + diagn + edu.cat * M +
diagn * M + APOE4 * M + Gender * M
Model 2: neg.b.MMSE ~ M + AGE + APOE4 + Gender + edu.cat + diagn + edu.cat * M +
diagn * M + APOE4 * M + Gender * M + NSAID * M
  NoPar LogLik Df Deviance Pr(>Chi)
1    23 -16950
2    25 -16944  2     12.8 0.001662 **
---
Signif. codes:  0 '***' 0.001 '**' 0.01 '*' 0.05 '.' 0.1 ' ' 1

anova(paracetamol.NSAID,paracetamol)

Analysis of Deviance Table

Model 1: neg.b.MMSE ~ M + AGE + APOE4 + Gender + edu.cat + diagn + edu.cat * M +
diagn * M + APOE4 * M + Gender * M + parac * M
Model 2: neg.b.MMSE ~ M + AGE + APOE4 + Gender + edu.cat + diagn + edu.cat * M +
diagn * M + APOE4 * M + Gender * M + parac * M + NSAID * M
  NoPar LogLik Df Deviance Pr(>Chi)
1    25 -16947
2    27 -16942  2      9.6  0.00823 **
---
Signif. codes:  0 '***' 0.001 '**' 0.01 '*' 0.05 '.' 0.1 ' ' 1

anova(Painrelief,Base)

Analysis of Deviance Table

Model 1: neg.b.MMSE ~ M + AGE + APOE4 + Gender + edu.cat + diagn + edu.cat * M +
diagn * M + APOE4 * M + Gender * M
Model 2: neg.b.MMSE ~ M + AGE + APOE4 + Gender + edu.cat + diagn + edu.cat * M +
diagn * M + APOE4 * M + Gender * M + Painrelief * M
  NoPar LogLik Df Deviance Pr(>Chi)
1    23 -16950
2    25 -16944  2     12.8 0.001662 **
---
Signif. codes:  0 '***' 0.001 '**' 0.01 '*' 0.05 '.' 0.1 ' ' 1
```

9.2.2. Generating the model matrices for each pain medication

9.2.2.1. Naproxen

```
newdat.naprox<-expand.grid(ID = 120,  M =
c(0,6,12,18,24,36,48,60,72,84,96,108,120), AGE = 0,APOE4 = c("0","1","2"),
Gender = c("Male","Female"),  edu.cat = c(levels(MMSEdata$edu.cat)),
diagn=c(levels(MMSEdata$diagn)),naprox = c(0,1))

mm <- model.matrix(delete.response(terms(naproxen)),newdat.naprox)


newdat.naprox$manualEstimate = drop(mm %*% fixef(naproxen))
```

```
varMatrix <- mm %*% vcov(naproxen) %*% t(mm)
VarPointEstimates<-diag(varMatrix)

newdat.naprox$manualSE = sqrt(VarPointEstimates)
newdat.naprox$predictSE = predict(naproxen, newdata = newdat.naprox,type =
'link',se.fit = TRUE)$se.fit
newdat.naprox$MMSEestimate<-30-exp(newdat.naprox$manualEstimate)
newdat.naprox$UCI = 30-exp((newdat.naprox$manualEstimate
+1.96*newdat.naprox$predictSE))
newdat.naprox$LCI = 30-exp((newdat.naprox$manualEstimate -
1.96*newdat.naprox$predictSE))

newdat.naprox.only<-newdat.naprox[newdat.naprox$naprox==1,]
newdat.naprox.only$pain.medication<-
rep("naprox",length(newdat.naprox.only$naprox))

newdat.naprox.only<-subset(newdat.naprox.only,select=-c(naprox))
```

9.2.2.2. *Aspirin*

```
newdat.aspirin<-expand.grid(ID = 120,  M =
c(0,6,12,18,24,36,48,60,72,84,96,108,120), AGE = 0,APOE4 = c("0","1","2"),
Gender = c("Male","Female"),  edu.cat = c(levels(MMSEdata$edu.cat)),
diagn=c(levels(MMSEdata$diagn)),aspirin = c(0,1))

mm <- model.matrix(delete.response(terms(aspirin)),newdat.aspirin)
newdat.aspirin$manualEstimate = drop(mm %*% fixef(aspirin))
varMatrix <- mm %*% vcov(aspirin) %*% t(mm)
VarPointEstimates<-diag(varMatrix)

newdat.aspirin$manualSE = sqrt(VarPointEstimates)
newdat.aspirin$predictSE = predict(aspirin, newdata = newdat.aspirin,type =
'link',se.fit = TRUE)$se.fit
newdat.aspirin$MMSEestimate<-30-exp(newdat.aspirin$manualEstimate)
newdat.aspirin$UCI = 30-exp((newdat.aspirin$manualEstimate
+1.96*newdat.aspirin$predictSE))
newdat.aspirin$LCI = 30-exp((newdat.aspirin$manualEstimate -
1.96*newdat.aspirin$predictSE))

newdat.aspirin.only<-newdat.aspirin[newdat.aspirin$aspirin==1,]
newdat.aspirin.only$pain.medication<-
rep("aspirin",length(newdat.aspirin.only$aspirin))

newdat.aspirin.only<-subset(newdat.aspirin.only,select=-c(aspirin))
```

9.2.2.3. *Paracetamol*

```
newdat.paracetamol<-expand.grid(ID = 120,  M =
c(0,6,12,18,24,36,48,60,72,84,96,108,120), AGE = 0,APOE4 = c("0","1","2"),
Gender = c("Male","Female"),  edu.cat = c(levels(MMSEdata$edu.cat)),
diagn=c(levels(MMSEdata$diagn)),parac = c(0,1))

mm <- model.matrix(delete.response(terms(paracetamol)),newdat.paracetamol)
newdat.paracetamol$manualEstimate = drop(mm %*% fixef(paracetamol))
varMatrix <- mm %*% vcov(paracetamol) %*% t(mm)
VarPointEstimates<-diag(varMatrix)

newdat.paracetamol$manualSE = sqrt(VarPointEstimates)
newdat.paracetamol$predictSE = predict(paracetamol, newdata =
```

```
newdat.paracetamol,type = 'link',se.fit = TRUE)$se.fit
newdat.paracetamol$MMSEestimate<-30-exp(newdat.paracetamol$manualEstimate)
newdat.paracetamol$UCI = 30-exp((newdat.paracetamol$manualEstimate
+1.96*newdat.paracetamol$predictSE))
newdat.paracetamol$LCI = 30-exp((newdat.paracetamol$manualEstimate -
1.96*newdat.paracetamol$predictSE))

newdat.paracetamol.only<-newdat.paracetamol[newdat.paracetamol$parac==1,]
newdat.paracetamol.only$pain.medication<-
rep("paracetamol",length(newdat.paracetamol.only$parac))
newdat.paracetamol.only<-subset(newdat.paracetamol.only,select=-c(parac))
```

9.2.2.4. Celecoxib

```
newdat.celecoxib<-expand.grid(ID = 120,  M =
c(0,6,12,18,24,36,48,60,72,84,96,108,120), AGE = 0,APOE4 = c("0","1","2"),
Gender = c("Male","Female"),  edu.cat = c(levels(MMSEdata$edu.cat)),
diagn=c(levels(MMSEdata$diagn)),celex = c(0,1))

mm <- model.matrix(delete.response(terms(celecoxib)),newdat.celecoxib)
newdat.celecoxib$manualEstimate = drop(mm %*% fixef(celecoxib))
varMatrix <- mm %*% vcov(celecoxib) %*% t(mm)
VarPointEstimates<-diag(varMatrix)

newdat.celecoxib$manualSE = sqrt(VarPointEstimates)
newdat.celecoxib$predictSE = predict(celecoxib, newdata = newdat.celecoxib,type
= 'link',se.fit = TRUE)$se.fit
newdat.celecoxib$MMSEestimate<-30-exp(newdat.celecoxib$manualEstimate)
newdat.celecoxib$UCI = 30-exp((newdat.celecoxib$manualEstimate
+1.96*newdat.celecoxib$predictSE))
newdat.celecoxib$LCI = 30-exp((newdat.celecoxib$manualEstimate -
1.96*newdat.celecoxib$predictSE))

newdat.celecoxib.only<-newdat.celecoxib[newdat.celecoxib$celex==1,]
newdat.celecoxib.only$pain.medication<-
rep("celecoxib",length(newdat.celecoxib.only$celex))

newdat.celecoxib.only<-subset(newdat.celecoxib.only,select=-c(celex))
```

9.2.2.5. Ibuprofen

```
newdat.ibuprofen<-expand.grid(ID = 120,  M =
c(0,6,12,18,24,36,48,60,72,84,96,108,120), AGE = 0,APOE4 = c("0","1","2"),
Gender = c("Male","Female"),  edu.cat = c(levels(MMSEdata$edu.cat)),
diagn=c(levels(MMSEdata$diagn)),Ibu = c(0,1))

mm <- model.matrix(delete.response(terms(ibuprofen)),newdat.ibuprofen)
newdat.ibuprofen$manualEstimate = drop(mm %*% fixef(ibuprofen))
varMatrix <- mm %*% vcov(ibuprofen) %*% t(mm)
VarPointEstimates<-diag(varMatrix)

newdat.ibuprofen$manualSE = sqrt(VarPointEstimates)
newdat.ibuprofen$predictSE = predict(ibuprofen, newdata = newdat.ibuprofen,type
= 'link',se.fit = TRUE)$se.fit
newdat.ibuprofen$MMSEestimate<-30-exp(newdat.ibuprofen$manualEstimate)
newdat.ibuprofen$UCI = 30-exp((newdat.ibuprofen$manualEstimate
+1.96*newdat.ibuprofen$predictSE))
newdat.ibuprofen$LCI = 30-exp((newdat.ibuprofen$manualEstimate -
1.96*newdat.ibuprofen$predictSE))
```

```
newdat.ibuprofen.only<-newdat.ibuprofen[newdat.ibuprofen$Ibu==1,]
newdat.ibuprofen.only$pain.medication<-
rep("ibuprofen",length(newdat.ibuprofen.only$Ibu))

newdat.ibuprofen.only<-subset(newdat.ibuprofen.only,select=-c(Ibu))
```

9.2.2.6. Diclofenac

```
newdat.diclofenac<-expand.grid(ID = 120,  M =
c(0,6,12,18,24,36,48,60,72,84,96,108,120), AGE = 0,APOE4 = c("0","1","2"),
Gender = c("Male","Female"),  edu.cat = c(levels(MMSEdata$edu.cat)),
diagn=c(levels(MMSEdata$diagn)),diclo = c(0,1))

mm <- model.matrix(delete.response(terms(diclofenac)),newdat.diclofenac)
newdat.diclofenac$manualEstimate = drop(mm %*% fixef(diclofenac))
varMatrix <- mm %*% vcov(diclofenac) %*% t(mm)
VarPointEstimates<-diag(varMatrix)

newdat.diclofenac$manualSE = sqrt(VarPointEstimates)
newdat.diclofenac$predictSE = predict(diclofenac, newdata =
newdat.diclofenac,type = 'link',se.fit = TRUE)$se.fit
newdat.diclofenac$MMSEestimate<-30-exp(newdat.diclofenac$manualEstimate)
newdat.diclofenac$UCI = 30-exp((newdat.diclofenac$manualEstimate
+1.96*newdat.diclofenac$predictSE))
newdat.diclofenac$LCI = 30-exp((newdat.diclofenac$manualEstimate -
1.96*newdat.diclofenac$predictSE))

newdat.diclofenac.only<-newdat.diclofenac[newdat.diclofenac$diclo==1,]
newdat.diclofenac.only$pain.medication<-
rep("diclofenac",length(newdat.diclofenac.only$diclo))

newdat.diclofenac.only<-subset(newdat.diclofenac.only,select=-c(diclo))
```

9.2.2.7. NSAID

```
newdat.NSAIDS<-expand.grid(ID = 120,  M =
c(0,6,12,18,24,36,48,60,72,84,96,108,120), AGE = 0,APOE4 = c("0","1","2"),
Gender = c("Male","Female"),  edu.cat = c(levels(MMSEdata$edu.cat)),
diagn=c(levels(MMSEdata$diagn)),NSAID = c(0,1))

mm <- model.matrix(delete.response(terms(NSAIDS)),newdat.NSAIDS)
newdat.NSAIDS$manualEstimate = drop(mm %*% fixef(NSAIDS))
varMatrix <- mm %*% vcov(NSAIDS) %*% t(mm)
VarPointEstimates<-diag(varMatrix)

newdat.NSAIDS$manualSE = sqrt(VarPointEstimates)
newdat.NSAIDS$predictSE = predict(NSAIDS, newdata = newdat.NSAIDS,type =
'link',se.fit = TRUE)$se.fit
newdat.NSAIDS$MMSEestimate<-30-exp(newdat.NSAIDS$manualEstimate)
newdat.NSAIDS$UCI = 30-exp((newdat.NSAIDS$manualEstimate
+1.96*newdat.NSAIDS$predictSE))
newdat.NSAIDS$LCI = 30-exp((newdat.NSAIDS$manualEstimate -
1.96*newdat.NSAIDS$predictSE))

newdat.NSAIDS.only<-newdat.NSAIDS[newdat.NSAIDS$NSAID==1,]
newdat.NSAIDS.only$pain.medication<-rep("NSAIDS",length(newdat.NSAIDS.only$ID))
```

9.2.2.8. No.Painrelief

```
newdat.No.Painrelief<-expand.grid(ID = 120,  M =
c(0,6,12,18,24,36,48,60,72,84,96,108,120), AGE = 0,APOE4 = c("0","1","2"),
Gender = c("Male","Female"),  edu.cat = c(levels(MMSEdata$edu.cat)),
diagn=c(levels(MMSEdata$diagn)),Painrelief = c(0,1))

mm <- model.matrix(delete.response(terms(Painrelief)), newdat.No.Painrelief)
newdat.No.Painrelief$manualEstimate = drop(mm %*% fixef(Painrelief))
varMatrix <- mm %*% vcov(Painrelief) %*% t(mm)
VarPointEstimates<-diag(varMatrix)

newdat.No.Painrelief$manualSE = sqrt(VarPointEstimates)
newdat.No.Painrelief$predictSE = predict(Painrelief, newdata =
newdat.No.Painrelief,type = 'link',se.fit = TRUE)$se.fit
newdat.No.Painrelief$MMSEestimate<-30-exp(newdat.No.Painrelief$manualEstimate)
newdat.No.Painrelief$UCI = 30-exp((newdat.No.Painrelief$manualEstimate
+1.96*newdat.No.Painrelief$predictSE))
newdat.No.Painrelief$LCI = 30-exp((newdat.No.Painrelief$manualEstimate -
1.96*newdat.No.Painrelief$predictSE))

newdat.No.Painrelief.only<-
newdat.No.Painrelief[newdat.No.Painrelief$Painrelief==0,]
newdat.No.Painrelief.only$pain.medication<-
rep("No.Painrelief",length(newdat.No.Painrelief.only$ID))
```

9.2.3. Combining the model matrix

```
combined<-
rbind(newdat.ibuprofen.only,newdat.diclofenac.only,newdat.NSAIDS.only[,-
8],newdat.naprox.only,newdat.aspirin.only,newdat.celecoxib.only,newdat.paracetam
ol.only,newdat.No.Painrelief.only[,-8])
combined$pain.medication<-as.factor(combined$pain.medication)
levels(combined$pain.medication)

[1] "aspirin"       "celecoxib"     "diclofenac"    "ibuprofen"
[5] "naprox"        "No.Painrelief" "NSAIDS"        "paracetamol"

write.csv(combined,"CombinedMMSEestimates.csv")
```

9.3. Generating model matrix for each pain medications ADAS

9.3.1. Building models

```
naproxen.adas<-glmmadmb(neg.b.ADAS~M+AGE+ APOE4+Gender+ headache+ edu.cat +
diagn+   edu.cat*M+diagn*M+APOE4*M+Gender*M+naprox*M+ (1|ID), family="nbinom1",
data=ADASdata)

aspirin.adas<-glmmadmb(neg.b.ADAS~M+AGE+ APOE4+Gender+ headache+ edu.cat +
diagn+   edu.cat*M+diagn*M+APOE4*M+Gender*M+aspirin*M+ (1|ID), family="nbinom1",
data=ADASdata)

paracetamol.adas<-glmmadmb(neg.b.ADAS~M+AGE+ APOE4+Gender+ headache+ edu.cat +
diagn+   edu.cat*M+diagn*M+APOE4*M+Gender*M+parac*M+ (1|ID), family="nbinom1",
data=ADASdata)

celecoxib.adas<-glmmadmb(neg.b.ADAS~M+AGE+ APOE4+Gender+ headache+ edu.cat +
diagn+   edu.cat*M+diagn*M+APOE4*M+Gender*M+celex*M+ (1|ID), family="nbinom1",
```

```
data=ADASdata)

ibuprofen.adas<-glmmadmb(neg.b.ADAS~M+AGE+ APOE4+Gender+ headache+ edu.cat +
diagn+   edu.cat*M+diagn*M+APOE4*M+Gender*M+Ibu*M+ (1|ID), family="nbinom1",
data=ADASdata)

diclofenac.adas<-glmmadmb(neg.b.ADAS~M+AGE+ APOE4+Gender+ headache+ edu.cat +
diagn+   edu.cat*M+diagn*M+APOE4*M+Gender*M+diclo*M+ (1|ID), family="nbinom1",
data=ADASdata)

NSAIDS.adas<-glmmadmb(neg.b.ADAS~M+AGE+ APOE4+Gender+ headache+ edu.cat + diagn+
edu.cat*M+diagn*M+APOE4*M+Gender*M+NSAID*M+ (1|ID), family="nbinom1",
data=ADASdata)

Base.adas<-glmmadmb(neg.b.ADAS~M+AGE+ APOE4+Gender+ headache+ edu.cat + diagn+
edu.cat*M+diagn*M+APOE4*M+Gender*M+ (1|ID), family="nbinom1", data=ADASdata)

paracetamol.NSAID.adas<-glmmadmb(neg.b.ADAS~M+AGE+ APOE4+Gender+ headache+
edu.cat + diagn+   edu.cat*M+diagn*M+APOE4*M+Gender*M+parac*M+ NSAID*M+(1|ID),
family="nbinom1", data=ADASdata)

Painrelief.adas<-glmmadmb(neg.b.ADAS~M+AGE+ APOE4+Gender+ headache+ edu.cat +
diagn+   edu.cat*M+diagn*M+APOE4*M+Gender*M+ Painrelief+ (1|ID),
family="nbinom1", data=ADASdata)

anova(naproxen.adas,Base.adas)

Analysis of Deviance Table

Model 1: neg.b.ADAS ~ M + AGE + APOE4 + Gender + headache + edu.cat + diagn +
edu.cat * M + diagn * M + APOE4 * M + Gender * M
Model 2: neg.b.ADAS ~ M + AGE + APOE4 + Gender + headache + edu.cat + diagn +
edu.cat * M + diagn * M + APOE4 * M + Gender * M + naprox * M
  NoPar LogLik Df Deviance Pr(>Chi)
1    24 -36048
2    26 -36045  2      4.2   0.1225

anova(aspirin.adas,Base.adas)

Analysis of Deviance Table

Model 1: neg.b.ADAS ~ M + AGE + APOE4 + Gender + headache + edu.cat + diagn +
edu.cat * M + diagn * M + APOE4 * M + Gender * M
Model 2: neg.b.ADAS ~ M + AGE + APOE4 + Gender + headache + edu.cat + diagn +
edu.cat * M + diagn * M + APOE4 * M + Gender * M + aspirin * M
  NoPar LogLik Df Deviance Pr(>Chi)
1    24 -36048
2    26 -36046  2      2.2   0.3329

anova(paracetamol.adas,Base.adas)

Analysis of Deviance Table

Model 1: neg.b.ADAS ~ M + AGE + APOE4 + Gender + headache + edu.cat + diagn +
edu.cat * M + diagn * M + APOE4 * M + Gender * M
Model 2: neg.b.ADAS ~ M + AGE + APOE4 + Gender + headache + edu.cat + diagn +
edu.cat * M + diagn * M + APOE4 * M + Gender * M + parac * M
  NoPar LogLik Df Deviance Pr(>Chi)
1    24 -36048
2    26 -36044  2      6.4  0.04076 *
```

```
---
Signif. codes:  0 '***' 0.001 '**' 0.01 '*' 0.05 '.' 0.1 ' ' 1

anova(celecoxib.adas,Base.adas)

Analysis of Deviance Table

Model 1: neg.b.ADAS ~ M + AGE + APOE4 + Gender + headache + edu.cat + diagn +
edu.cat * M + diagn * M + APOE4 * M + Gender * M
Model 2: neg.b.ADAS ~ M + AGE + APOE4 + Gender + headache + edu.cat + diagn +
edu.cat * M + diagn * M + APOE4 * M + Gender * M + celex * M
  NoPar LogLik Df Deviance Pr(>Chi)
1    24 -36048
2    26 -36047  2        1   0.6065

anova(ibuprofen.adas,Base.adas)

Analysis of Deviance Table

Model 1: neg.b.ADAS ~ M + AGE + APOE4 + Gender + headache + edu.cat + diagn +
edu.cat * M + diagn * M + APOE4 * M + Gender * M
Model 2: neg.b.ADAS ~ M + AGE + APOE4 + Gender + headache + edu.cat + diagn +
edu.cat * M + diagn * M + APOE4 * M + Gender * M + Ibu * M
  NoPar LogLik Df Deviance Pr(>Chi)
1    24 -36048
2    26 -36042  2     11.6 0.003028 **
---
Signif. codes:  0 '***' 0.001 '**' 0.01 '*' 0.05 '.' 0.1 ' ' 1

anova(diclofenac.adas,Base.adas)

Analysis of Deviance Table

Model 1: neg.b.ADAS ~ M + AGE + APOE4 + Gender + headache + edu.cat + diagn +
edu.cat * M + diagn * M + APOE4 * M + Gender * M
Model 2: neg.b.ADAS ~ M + AGE + APOE4 + Gender + headache + edu.cat + diagn +
edu.cat * M + diagn * M + APOE4 * M + Gender * M + diclo * M
  NoPar LogLik Df Deviance Pr(>Chi)
1    24 -36048
2    26 -36042  2     10.4 0.005517 **
---
Signif. codes:  0 '***' 0.001 '**' 0.01 '*' 0.05 '.' 0.1 ' ' 1

anova(NSAIDS.adas,Base.adas)

Analysis of Deviance Table

Model 1: neg.b.ADAS ~ M + AGE + APOE4 + Gender + headache + edu.cat + diagn +
edu.cat * M + diagn * M + APOE4 * M + Gender * M
Model 2: neg.b.ADAS ~ M + AGE + APOE4 + Gender + headache + edu.cat + diagn +
edu.cat * M + diagn * M + APOE4 * M + Gender * M + NSAID * M
  NoPar LogLik Df Deviance Pr(>Chi)
1    24 -36048
2    26 -36045  2      5.8  0.05502 .
---
Signif. codes:  0 '***' 0.001 '**' 0.01 '*' 0.05 '.' 0.1 ' ' 1

anova(paracetamol.NSAID.adas,paracetamol.adas)

Analysis of Deviance Table
```

```
Model 1: neg.b.ADAS ~ M + AGE + APOE4 + Gender + headache + edu.cat + diagn +
edu.cat * M + diagn * M + APOE4 * M + Gender * M + parac * M
Model 2: neg.b.ADAS ~ M + AGE + APOE4 + Gender + headache + edu.cat + diagn +
edu.cat * M + diagn * M + APOE4 * M + Gender * M + parac * M + NSAID * M
  NoPar LogLik Df Deviance Pr(>Chi)
1    26 -36044
2    28 -36042  2      5.4  0.06721 .
---
Signif. codes:  0 '***' 0.001 '**' 0.01 '*' 0.05 '.' 0.1 ' ' 1

anova(Painrelief.adas,Base.adas)

Analysis of Deviance Table

Model 1: neg.b.ADAS ~ M + AGE + APOE4 + Gender + headache + edu.cat + diagn +
edu.cat * M + diagn * M + APOE4 * M + Gender * M
Model 2: neg.b.ADAS ~ M + AGE + APOE4 + Gender + headache + edu.cat + diagn +
edu.cat * M + diagn * M + APOE4 * M + Gender * M + Painrelief
  NoPar LogLik Df Deviance Pr(>Chi)
1    24 -36048
2    25 -36043  1      9.4  0.00217 **
---
Signif. codes:  0 '***' 0.001 '**' 0.01 '*' 0.05 '.' 0.1 ' ' 1
```

9.3.2. Building the model matrix with 95% Laplance confidence interavals

9.3.2.1. Naproxen

```
newdat.naprox<-expand.grid(ID = 120,  M =
c(0,6,12,18,24,36,48,60,72,84,96,108,120), AGE = 0,APOE4 = c("0","1","2"),
Gender = c("Male","Female"),  edu.cat = c(levels(ADASdata$edu.cat)),
diagn=c(levels(ADASdata$diagn)),naprox = c(0,1),headache = c(0,1))

mm <- model.matrix(delete.response(terms(naproxen.adas)),newdat.naprox)


newdat.naprox$manualEstimate = drop(mm %*% fixef(naproxen.adas))
varMatrix <- mm %*% vcov(naproxen.adas) %*% t(mm)
VarPointEstimates<-diag(varMatrix)

newdat.naprox$manualSE = sqrt(VarPointEstimates)
newdat.naprox$predictSE = predict(naproxen.adas, newdata = newdat.naprox,type =
'link',se.fit = TRUE)$se.fit
newdat.naprox$ADASestimate<-exp(newdat.naprox$manualEstimate)
newdat.naprox$UCI = exp((newdat.naprox$manualEstimate
+1.96*newdat.naprox$predictSE))
newdat.naprox$LCI = exp((newdat.naprox$manualEstimate -
1.96*newdat.naprox$predictSE))

newdat.naprox.only<-newdat.naprox[newdat.naprox$naprox==1,]
newdat.naprox.only$pain.medication<-
rep("naprox",length(newdat.naprox.only$naprox))

newdat.naprox.only<-subset(newdat.naprox.only,select=-c(naprox))
```

9.3.2.2. *Aspirin*

```
newdat.aspirin<-expand.grid(ID = 120,  M =
c(0,6,12,18,24,36,48,60,72,84,96,108,120), AGE = 0,APOE4 = c("0","1","2"),
Gender = c("Male","Female"),  edu.cat = c(levels(ADASdata$edu.cat)),
diagn=c(levels(ADASdata$diagn)),aspirin = c(0,1),headache = c(0,1))

mm <- model.matrix(delete.response(terms(aspirin.adas)),newdat.aspirin)
newdat.aspirin$manualEstimate = drop(mm %*% fixef(aspirin.adas))
varMatrix <- mm %*% vcov(aspirin.adas) %*% t(mm)
VarPointEstimates<-diag(varMatrix)

newdat.aspirin$manualSE = sqrt(VarPointEstimates)
newdat.aspirin$predictSE = predict(aspirin.adas, newdata = newdat.aspirin,type =
'link',se.fit = TRUE)$se.fit
newdat.aspirin$ADASestimate<-exp(newdat.aspirin$manualEstimate)
newdat.aspirin$UCI = exp((newdat.aspirin$manualEstimate
+1.96*newdat.aspirin$predictSE))
newdat.aspirin$LCI = exp((newdat.aspirin$manualEstimate -
1.96*newdat.aspirin$predictSE))

newdat.aspirin.only<-newdat.aspirin[newdat.aspirin$aspirin==1,]
newdat.aspirin.only$pain.medication<-
rep("aspirin",length(newdat.aspirin.only$aspirin))

newdat.aspirin.only<-subset(newdat.aspirin.only,select=-c(aspirin))
```

9.3.2.3. *Paracetamol*

```
newdat.paracetamol<-expand.grid(ID = 120,  M =
c(0,6,12,18,24,36,48,60,72,84,96,108,120), AGE = 0,APOE4 = c("0","1","2"),
Gender = c("Male","Female"),  edu.cat = c(levels(ADASdata$edu.cat)),
diagn=c(levels(ADASdata$diagn)),parac = c(0,1),headache = c(0,1))

mm <- model.matrix(delete.response(terms(paracetamol.adas)),newdat.paracetamol)
newdat.paracetamol$manualEstimate = drop(mm %*% fixef(paracetamol.adas))
varMatrix <- mm %*% vcov(paracetamol.adas) %*% t(mm)
VarPointEstimates<-diag(varMatrix)

newdat.paracetamol$manualSE = sqrt(VarPointEstimates)
newdat.paracetamol$predictSE = predict(paracetamol.adas, newdata =
newdat.paracetamol,type = 'link',se.fit = TRUE)$se.fit
newdat.paracetamol$ADASestimate<-exp(newdat.paracetamol$manualEstimate)
newdat.paracetamol$UCI = exp((newdat.paracetamol$manualEstimate
+1.96*newdat.paracetamol$predictSE))
newdat.paracetamol$LCI = exp((newdat.paracetamol$manualEstimate -
1.96*newdat.paracetamol$predictSE))

newdat.paracetamol.only<-newdat.paracetamol[newdat.paracetamol$parac==1,]
newdat.paracetamol.only$pain.medication<-
rep("paracetamol",length(newdat.paracetamol.only$parac))
newdat.paracetamol.only<-subset(newdat.paracetamol.only,select=-c(parac))
```

9.3.2.4. *Celecoxib*

```
newdat.celecoxib<-expand.grid(ID = 120,  M =
c(0,6,12,18,24,36,48,60,72,84,96,108,120), AGE = 0,APOE4 = c("0","1","2"),
Gender = c("Male","Female"),  edu.cat = c(levels(ADASdata$edu.cat)),
diagn=c(levels(ADASdata$diagn)),celex = c(0,1),headache = c(0,1))
```

```
mm <- model.matrix(delete.response(terms(celecoxib.adas)),newdat.celecoxib)
newdat.celecoxib$manualEstimate = drop(mm %*% fixef(celecoxib.adas))
varMatrix <- mm %*% vcov(celecoxib.adas) %*% t(mm)
VarPointEstimates<-diag(varMatrix)

newdat.celecoxib$manualSE = sqrt(VarPointEstimates)
newdat.celecoxib$predictSE = predict(celecoxib.adas, newdata =
newdat.celecoxib,type = 'link',se.fit = TRUE)$se.fit
newdat.celecoxib$ADASestimate<-exp(newdat.celecoxib$manualEstimate)
newdat.celecoxib$UCI = exp((newdat.celecoxib$manualEstimate
+1.96*newdat.celecoxib$predictSE))
newdat.celecoxib$LCI = exp((newdat.celecoxib$manualEstimate -
1.96*newdat.celecoxib$predictSE))

newdat.celecoxib.only<-newdat.celecoxib[newdat.celecoxib$celex==1,]
newdat.celecoxib.only$pain.medication<-
rep("celecoxib",length(newdat.celecoxib.only$celex))

newdat.celecoxib.only<-subset(newdat.celecoxib.only,select=-c(celex))
```

9.3.2.5. *Ibuprofen*

```
newdat.ibuprofen<-expand.grid(ID = 120,  M =
c(0,6,12,18,24,36,48,60,72,84,96,108,120), AGE = 0,APOE4 = c("0","1","2"),
Gender = c("Male","Female"),  edu.cat = c(levels(ADASdata$edu.cat)),
diagn=c(levels(ADASdata$diagn)),Ibu = c(0,1),headache = c(0,1))

mm <- model.matrix(delete.response(terms(ibuprofen.adas)),newdat.ibuprofen)
newdat.ibuprofen$manualEstimate = drop(mm %*% fixef(ibuprofen.adas))
varMatrix <- mm %*% vcov(ibuprofen.adas) %*% t(mm)
VarPointEstimates<-diag(varMatrix)

newdat.ibuprofen$manualSE = sqrt(VarPointEstimates)
newdat.ibuprofen$predictSE = predict(ibuprofen.adas, newdata =
newdat.ibuprofen,type = 'link',se.fit = TRUE)$se.fit
newdat.ibuprofen$ADASestimate<-exp(newdat.ibuprofen$manualEstimate)
newdat.ibuprofen$UCI = exp((newdat.ibuprofen$manualEstimate
+1.96*newdat.ibuprofen$predictSE))
newdat.ibuprofen$LCI = exp((newdat.ibuprofen$manualEstimate -
1.96*newdat.ibuprofen$predictSE))

newdat.ibuprofen.only<-newdat.ibuprofen[newdat.ibuprofen$Ibu==1,]
newdat.ibuprofen.only$pain.medication<-
rep("ibuprofen",length(newdat.ibuprofen.only$Ibu))

newdat.ibuprofen.only<-subset(newdat.ibuprofen.only,select=-c(Ibu))
```

9.3.2.6. *Diclofenac*

```
newdat.diclofenac<-expand.grid(ID = 120,  M =
c(0,6,12,18,24,36,48,60,72,84,96,108,120), AGE = 0,APOE4 = c("0","1","2"),
Gender = c("Male","Female"),  edu.cat = c(levels(ADASdata$edu.cat)),
diagn=c(levels(ADASdata$diagn)),diclo = c(0,1),headache = c(0,1))

mm <- model.matrix(delete.response(terms(diclofenac.adas)),newdat.diclofenac)
newdat.diclofenac$manualEstimate = drop(mm %*% fixef(diclofenac.adas))
varMatrix <- mm %*% vcov(diclofenac.adas) %*% t(mm)
VarPointEstimates<-diag(varMatrix)
```

```
newdat.diclofenac$manualSE = sqrt(VarPointEstimates)
newdat.diclofenac$predictSE = predict(diclofenac.adas, newdata =
newdat.diclofenac,type = 'link',se.fit = TRUE)$se.fit
newdat.diclofenac$ADASestimate<-exp(newdat.diclofenac$manualEstimate)
newdat.diclofenac$UCI = exp((newdat.diclofenac$manualEstimate
+1.96*newdat.diclofenac$predictSE))
newdat.diclofenac$LCI = exp((newdat.diclofenac$manualEstimate -
1.96*newdat.diclofenac$predictSE))

newdat.diclofenac.only<-newdat.diclofenac[newdat.diclofenac$diclo==1,]
newdat.diclofenac.only$pain.medication<-
rep("diclofenac",length(newdat.diclofenac.only$diclo))

newdat.diclofenac.only<-subset(newdat.diclofenac.only,select=-c(diclo))
```

9.3.2.7. NSAID

```
newdat.NSAIDS<-expand.grid(ID = 120,  M =
c(0,6,12,18,24,36,48,60,72,84,96,108,120), AGE = 0,APOE4 = c("0","1","2"),
Gender = c("Male","Female"),  edu.cat = c(levels(ADASdata$edu.cat)),
diagn=c(levels(ADASdata$diagn)),NSAID = c(0,1),headache = c(0,1))

mm <- model.matrix(delete.response(terms(NSAIDS.adas)),newdat.NSAIDS)
newdat.NSAIDS$manualEstimate = drop(mm %*% fixef(NSAIDS.adas))
varMatrix <- mm %*% vcov(NSAIDS.adas) %*% t(mm)
VarPointEstimates<-diag(varMatrix)

newdat.NSAIDS$manualSE = sqrt(VarPointEstimates)
newdat.NSAIDS$predictSE = predict(NSAIDS.adas, newdata = newdat.NSAIDS,type =
'link',se.fit = TRUE)$se.fit
newdat.NSAIDS$ADASestimate<-exp(newdat.NSAIDS$manualEstimate)
newdat.NSAIDS$UCI = exp((newdat.NSAIDS$manualEstimate
+1.96*newdat.NSAIDS$predictSE))
newdat.NSAIDS$LCI = exp((newdat.NSAIDS$manualEstimate -
1.96*newdat.NSAIDS$predictSE))

newdat.NSAIDS.only<-newdat.NSAIDS[newdat.NSAIDS$NSAID==1,]
newdat.NSAIDS.only$pain.medication<-rep("NSAIDS",length(newdat.NSAIDS.only$ID))
```

9.3.2.8. No Pain Medication

```
newdat.No.Painrelief<-expand.grid(ID = 120,  M =
c(0,6,12,18,24,36,48,60,72,84,96,108,120), AGE = 0,APOE4 = c("0","1","2"),
Gender = c("Male","Female"),  edu.cat = c(levels(ADASdata$edu.cat)),
diagn=c(levels(ADASdata$diagn)), Painrelief = c(0,1),headache = c(0,1))

mm <- model.matrix(delete.response(terms(Painrelief.adas)),newdat.No.Painrelief)
newdat.No.Painrelief$manualEstimate = drop(mm %*% fixef(Painrelief.adas))
varMatrix <- mm %*% vcov(Painrelief.adas) %*% t(mm)
VarPointEstimates<-diag(varMatrix)

newdat.No.Painrelief$manualSE = sqrt(VarPointEstimates)
newdat.No.Painrelief$predictSE = predict(Painrelief.adas, newdata =
newdat.No.Painrelief,type = 'link',se.fit = TRUE)$se.fit
newdat.No.Painrelief$ADASestimate<-exp(newdat.No.Painrelief$manualEstimate)
newdat.No.Painrelief$UCI = exp((newdat.No.Painrelief$manualEstimate
+1.96*newdat.No.Painrelief$predictSE))
newdat.No.Painrelief$LCI = exp((newdat.No.Painrelief$manualEstimate -
```

```
1.96*newdat.No.Painrelief$predictSE))

newdat.No.Painrelief.only<-
newdat.No.Painrelief[newdat.No.Painrelief$Painrelief==0,]
newdat.No.Painrelief.only$pain.medication<-
rep("No.Painrelief",length(newdat.No.Painrelief.only$ID))
```

9.3.2.9. *Combining the model matrix*

```
combined<-
rbind(newdat.ibuprofen.only,newdat.diclofenac.only,newdat.NSAIDS.only[,-
8],newdat.naprox.only,newdat.aspirin.only,newdat.celecoxib.only,newdat.paracetam
ol.only,newdat.No.Painrelief.only[,-8])
combined$pain.medication<-as.factor(combined$pain.medication)


write.csv(combined,"CombinedADASestimates.csv")
```

9.4. *Cleaning model matrices*

```
newdat<-read.csv("CombinedMMSEestimates.csv",header=T)
newdat$pain.medication<-as.factor(newdat$pain.medication)
newdatADAS<-read.csv("CombinedADASestimates.csv",header=T)
newdatADAS$ADASestimate<-newdatADAS$ADASestimate/3
newdatADAS$UCI<-newdatADAS$UCI/3
newdatADAS$LCI<-newdatADAS$LCI/3
newdatADAS$pain.medication<-as.factor(newdatADAS$pain.medication)
```

9.5. *Graphing LMCI with and without diclofenac use*

9.5.1. *MMSE*

```
red<-newdat[newdat$APOE4==0 & newdat$Gender=="Female" & newdat$edu.cat=="1post"&
newdat$diagn=="3LMCI"& newdat$pain.medication=="diclofenac",]
blue<-newdat[newdat$APOE4==0 & newdat$Gender=="Female" &
newdat$edu.cat=="1post"& newdat$diagn=="3LMCI"&
newdat$pain.medication=="No.Painrelief",]

plotdat<-rbind(red,blue)


Month<-c(0,6,12,18,24,36,48,60,72,84,96,108,120)
yaxismin<-max((floor(min(plotdat$MMSEestimate))-1),5)

xmax<-min(min(max(blue$M[which(blue$UCI>5,
arr.ind=TRUE)]),120),min(max(red$M[which(red$UCI>5, arr.ind=TRUE)]),120))

#PDF("MMSEDiclofenac.#PDF", width=16/2.54, heigh=12/2.54 )
plot(Month,red$MMSEestimate,pch=NA,ylab="MMSE", xlab="Month",ylim=c(yaxismin,
30),xlim=c(-2,xmax),axes = FALSE)
axis(1,seq(0,xmax,by=12))
axis(2,at=seq(30,yaxismin,by=-3), cex.axis=0.8)
polygon(c(rev(Month), Month), c(rev(red$LCI), red$UCI), col = rgb(0.8,0,0,0.2),
border = NA)
polygon(c(rev(Month), Month), c(rev(blue$LCI), blue$UCI), col =
rgb(0,0,0.8,0.2), border = NA)
lines(Month,red$MMSEestimate,col="#db4c4c",lty=1,lwd=3)
lines(Month,blue$MMSEestimate,col="#66a3e0",lty=1,lwd=3)
```

```
legend("bottomleft", bty = "n", legend = as.character(c("Non-User", "Diclofenac
User")),
       pch = 15, col = c("#7cafe2","#f78a8a"), cex = 1, horiz=F)
```

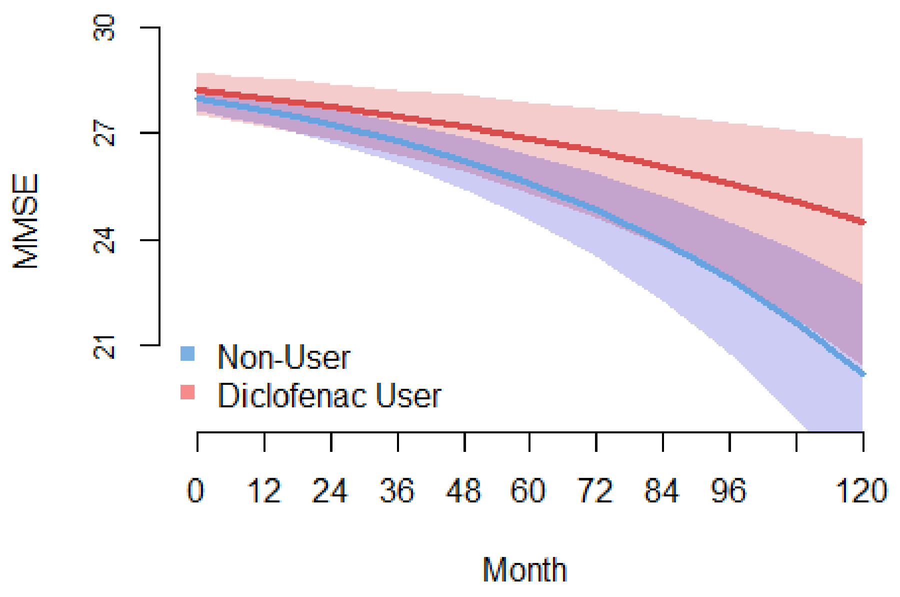


```
#dev.off()
```

9.5.2. ADAS

```
redADAS<-newdatADAS[newdatADAS$APOE4==0 & newdatADAS$headache==0 &
newdatADAS$Gender=="Female" & newdatADAS$edu.cat=="1post"&
newdatADAS$diagn=="3LMCI"& newdatADAS$pain.medication=="diclofenac",]
blueADAS<-newdatADAS[newdatADAS$APOE4==0 & newdatADAS$headache==0 &
newdatADAS$Gender=="Female" & newdatADAS$edu.cat=="1post"&
newdatADAS$diagn=="3LMCI"& newdatADAS$pain.medication=="No.Painrelief",]

plotdat<-rbind(redADAS,blueADAS)


Month<-c(0,6,12,18,24,36,48,60,72,84,96,108,120)
yaxismin<-max(min(c(plotdat$LCI)-5),0)
yaxismax<-min(max(redADAS$UCI,blueADAS$UCI)+5,90)
xmax<-min(min(max(blueADAS$M[which(blueADAS$UCI<70,
arr.ind=TRUE)]),120),min(max(redADAS$M[which(redADAS$UCI<70,
arr.ind=TRUE)]),120))

#PDF("ADASDiclofenac.#PDF", width=16/2.54, heigh=12/2.54 )
plot(Month,redADAS$ADASestimate,pch=NA,ylab="ADAS",
xlab="Month",ylim=c(yaxismin, yaxismax),xlim=c(-2,xmax),axes = FALSE)
axis(1,seq(0,xmax,by=12))
axis(2,at=seq(0,yaxismax,by=10),cex.axis=0.8)
polygon(c(rev(Month), Month), c(rev(redADAS$LCI), redADAS$UCI), col =
rgb(0.8,0,0,0.2), border = NA)
polygon(c(rev(Month), Month), c(rev(blueADAS$LCI), blueADAS$UCI), col =
```

```
rgb(0,0,0.8,0.2), border = NA)
lines(Month,redADAS$ADASestimate,col="#db4c4c",lty=1,lwd=3)
lines(Month,blueADAS$ADASestimate,col="#66a3e0",lty=1,lwd=3)
legend("topleft", bty = "n", legend = as.character(c("Non-User", "Diclofenac
User")),
       pch = 15, col = c("#7cafe2","#f78a8a"), cex = 1, horiz=F)
```

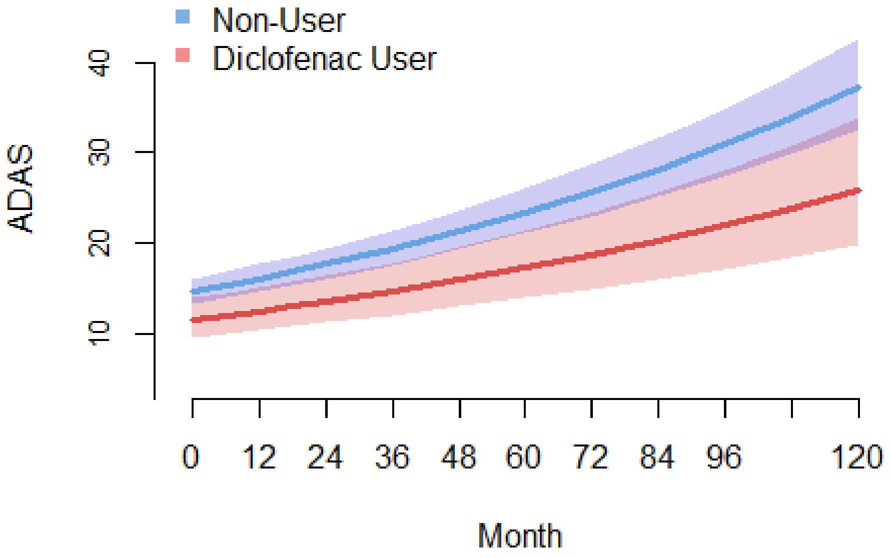


```
#dev.off()
```

9.6. *Graphing LMCI with and without Ibuprofen use*

9.6.1. MMSE

```
red<-newdat[newdat$APOE4==0 & newdat$Gender=="Female" & newdat$edu.cat=="1post"&
newdat$diagn=="3LMCI"& newdat$pain.medication=="ibuprofen",]
blue<-newdat[newdat$APOE4==0 & newdat$Gender=="Female" &
newdat$edu.cat=="1post"& newdat$diagn=="3LMCI"&
newdat$pain.medication=="No.Painrelief",]

plotdat<-rbind(red,blue)


Month<-c(0,6,12,18,24,36,48,60,72,84,96,108,120)
yaxismin<-max((floor(min(plotdat$MMSEestimate))-1),5)

xmax<-min(min(max(blue$M[which(blue$UCI>5,
arr.ind=TRUE)]),120),min(max(red$M[which(red$UCI>5, arr.ind=TRUE)]),120))

#PDF("MMSEIbuprofen.#PDF", width=16/2.54, heigh=12/2.54 )
plot(Month,red$MMSEestimate,pch=NA,ylab="MMSE", xlab="Month",ylim=c(yaxismin,
30),xlim=c(-2,xmax),axes = FALSE)
axis(1,seq(0,xmax,by=12))
```

```
axis(2,at=seq(30,yaxismin,by=-3), cex.axis=0.8)
polygon(c(rev(Month), Month), c(rev(red$LCI), red$UCI), col = rgb(0.8,0,0,0.2),
border = NA)
polygon(c(rev(Month), Month), c(rev(blue$LCI), blue$UCI), col =
rgb(0,0,0.8,0.2), border = NA)
lines(Month,red$MMSEestimate,col="#db4c4c",lty=1,lwd=3)
lines(Month,blue$MMSEestimate,col="#66a3e0",lty=1,lwd=3)
legend("bottomleft", bty = "n", legend = as.character(c("Non-User", "Ibuprofen
User")),
       pch = 15, col = c("#7cafe2","#f78a8a"), cex = 1, horiz=F)
```

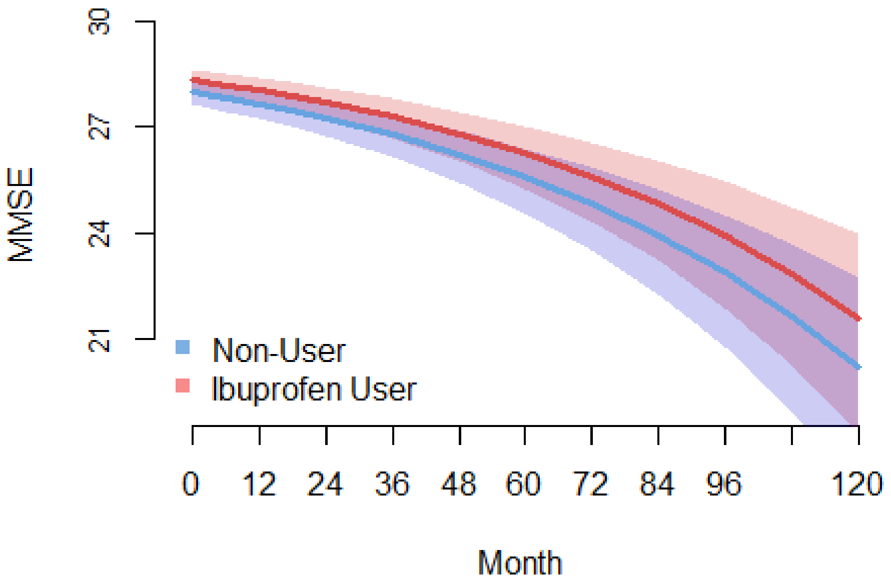


```
#dev.off()
```

9.6.2. ADAS

```
redADAS<-newdatADAS[newdatADAS$APOE4==0 & newdatADAS$headache==0 &
newdatADAS$Gender=="Female" & newdatADAS$edu.cat=="1post"&
newdatADAS$diagn=="3LMCI"& newdatADAS$pain.medication=="ibuprofen",]
blueADAS<-newdatADAS[newdatADAS$APOE4==0 & newdatADAS$headache==0 &
newdatADAS$Gender=="Female" & newdatADAS$edu.cat=="1post"&
newdatADAS$diagn=="3LMCI"& newdatADAS$pain.medication=="No.Painrelief",]

plotdat<-rbind(redADAS,blueADAS)


Month<-c(0,6,12,18,24,36,48,60,72,84,96,108,120)
yaxismin<-max(min(c(plotdat$LCI)-5),0)
yaxismax<-min(max(redADAS$UCI,blueADAS$UCI)+5,90)
xmax<-min(min(max(blueADAS$M[which(blueADAS$UCI<70,
arr.ind=TRUE)]),120),min(max(redADAS$M[which(redADAS$UCI<70,
arr.ind=TRUE)]),120))

#PDF("ADASIbuprofen.#PDF", width=16/2.54, heigh=12/2.54 )
```

```
plot(Month,redADAS$ADASestimate,pch=NA,ylab="ADAS",
xlab="Month",ylim=c(yaxismin, yaxismax),xlim=c(-2,xmax),axes = FALSE)
axis(1,seq(0,xmax,by=12))
axis(2,at=seq(0,yaxismax,by=10),cex.axis=0.8)
polygon(c(rev(Month), Month), c(rev(redADAS$LCI), redADAS$UCI), col =
rgb(0.8,0,0,0.2), border = NA)
polygon(c(rev(Month), Month), c(rev(blueADAS$LCI), blueADAS$UCI), col =
rgb(0,0,0.8,0.2), border = NA)
lines(Month,redADAS$ADASestimate,col="#db4c4c",lty=1,lwd=3)
lines(Month,blueADAS$ADASestimate,col="#66a3e0",lty=1,lwd=3)
legend("topleft", bty = "n", legend = as.character(c("Non-User", "Ibuprofen
User")),
       pch = 15, col = c("#7cafe2","#f78a8a"), cex = 1, horiz=F)
```

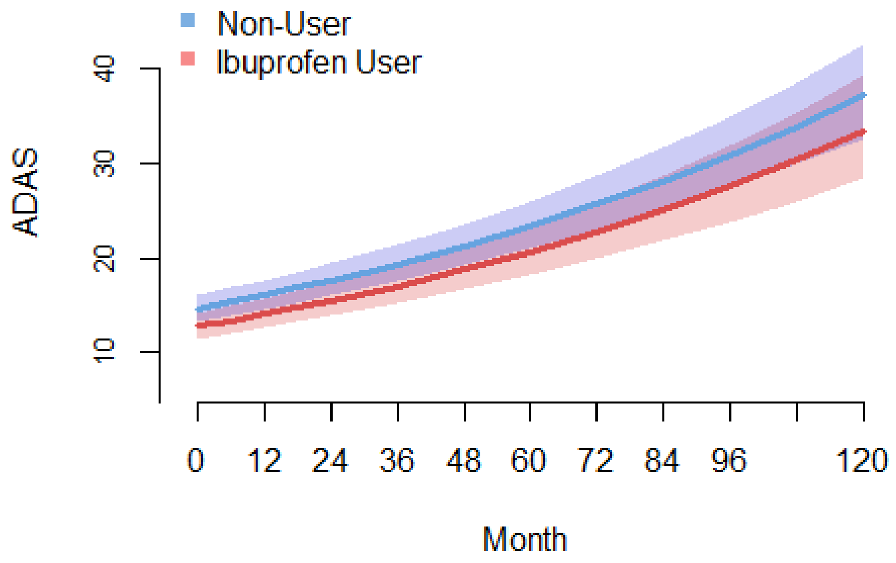


```
#dev.off()
```

9.7. *Graphing LMCI with and without Naproxen use*

9.7.1. MMSE

```
red<-newdat[newdat$APOE4==0 & newdat$Gender=="Female" & newdat$edu.cat=="1post"&
newdat$diagn=="3LMCI"& newdat$pain.medication=="naprox",]
blue<-newdat[newdat$APOE4==0 & newdat$Gender=="Female" &
newdat$edu.cat=="1post"& newdat$diagn=="3LMCI"&
newdat$pain.medication=="No.Painrelief",]

plotdat<-rbind(red,blue)


Month<-c(0,6,12,18,24,36,48,60,72,84,96,108,120)
yaxismin<-max((floor(min(plotdat$MMSEestimate))-1),5)
```

```
xmax<-min(min(max(blue$M[which(blue$UCI>5,
arr.ind=TRUE)]),120),min(max(red$M[which(red$UCI>5, arr.ind=TRUE)]),120))

#PDF("MMSENaproxen.#PDF", width=16/2.54, heigh=12/2.54 )
plot(Month,red$MMSEestimate,pch=NA,ylab="MMSE", xlab="Month",ylim=c(yaxismin,
30),xlim=c(-2,xmax),axes = FALSE)
axis(1,seq(0,xmax,by=12))
axis(2,at=seq(30,yaxismin,by=-3), cex.axis=0.8)
polygon(c(rev(Month), Month), c(rev(red$LCI), red$UCI), col = rgb(0.8,0,0,0.2),
border = NA)
polygon(c(rev(Month), Month), c(rev(blue$LCI), blue$UCI), col =
rgb(0,0,0.8,0.2), border = NA)
lines(Month,red$MMSEestimate,col="#db4c4c",lty=1,lwd=3)
lines(Month,blue$MMSEestimate,col="#66a3e0",lty=1,lwd=3)
legend("bottomleft", bty = "n", legend = as.character(c("Non-User", "Naproxen
User")),
       pch = 15, col = c("#7cafe2","#f78a8a"), cex = 1, horiz=F)
```

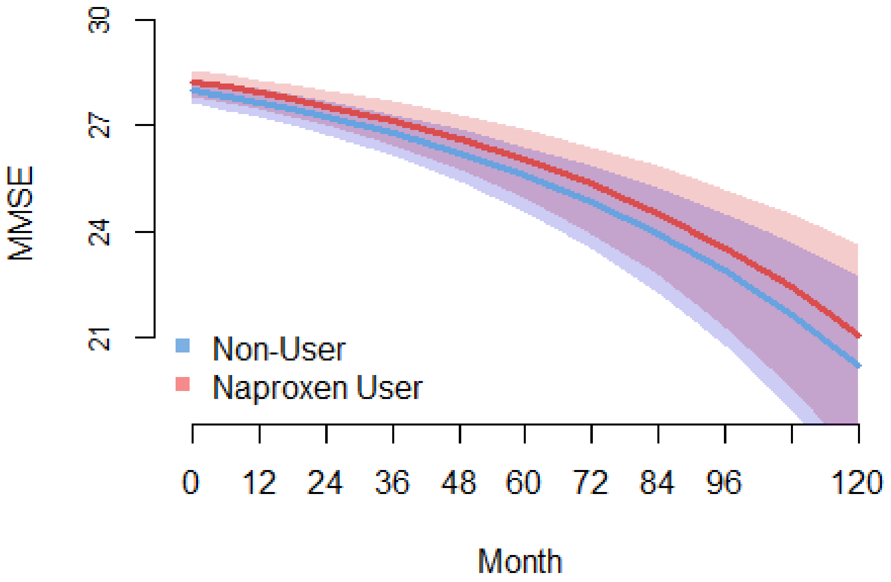


```
#dev.off()
```

9.7.2. ADAS

```
redADAS<-newdatADAS[newdatADAS$APOE4==0 & newdatADAS$headache==0 &
newdatADAS$Gender=="Female" & newdatADAS$edu.cat=="1post"&
newdatADAS$diagn=="3LMCI"& newdatADAS$pain.medication=="naprox",]
blueADAS<-newdatADAS[newdatADAS$APOE4==0 & newdatADAS$headache==0 &
newdatADAS$Gender=="Female" & newdatADAS$edu.cat=="1post"&
newdatADAS$diagn=="3LMCI"& newdatADAS$pain.medication=="No.Painrelief",]

plotdat<-rbind(redADAS,blueADAS)


Month<-c(0,6,12,18,24,36,48,60,72,84,96,108,120)
```

```
yaxismin<-max(min(c(plotdat$LCI)-5),0)
yaxismax<-min(max(redADAS$UCI,blueADAS$UCI)+5,90)
xmax<-min(min(max(blueADAS$M[which(blueADAS$UCI<70,
arr.ind=TRUE)]),120),min(max(redADAS$M[which(redADAS$UCI<70,
arr.ind=TRUE)]),120))

#PDF("ADASNaproxen.#PDF", width=16/2.54, heigh=12/2.54 )
plot(Month,redADAS$ADASestimate,pch=NA,ylab="ADAS",
xlab="Month",ylim=c(yaxismin, yaxismax),xlim=c(-2,xmax),axes = FALSE)
axis(1,seq(0,xmax,by=12))
axis(2,at=seq(0,yaxismax,by=10),cex.axis=0.8)
polygon(c(rev(Month), Month), c(rev(redADAS$LCI), redADAS$UCI), col =
rgb(0.8,0,0,0.2), border = NA)
polygon(c(rev(Month), Month), c(rev(blueADAS$LCI), blueADAS$UCI), col =
rgb(0,0,0.8,0.2), border = NA)
lines(Month,redADAS$ADASestimate,col="#db4c4c",lty=1,lwd=3)
lines(Month,blueADAS$ADASestimate,col="#66a3e0",lty=1,lwd=3)
legend("topleft", bty = "n", legend = as.character(c("Non-User", "Naproxen
User")),
       pch = 15, col = c("#7cafe2","#f78a8a"), cex = 1, horiz=F)
```

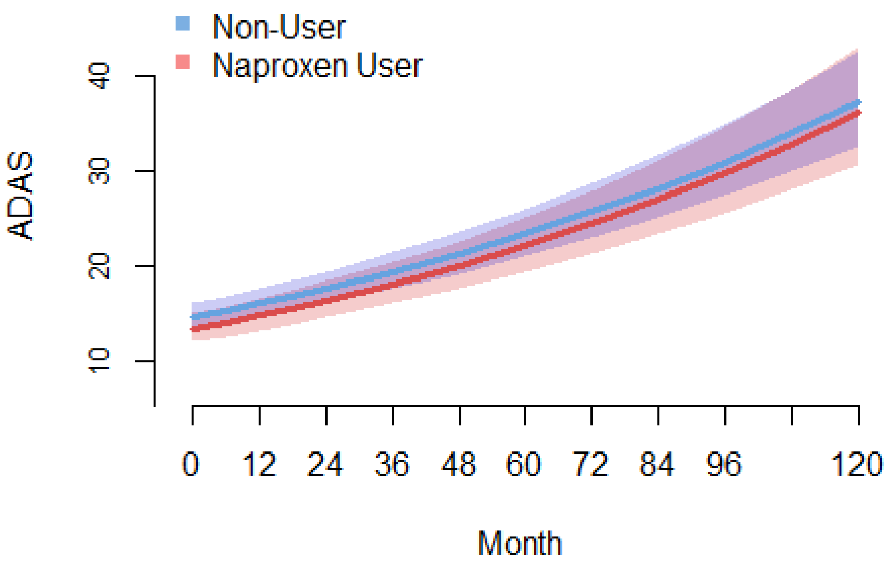


```
#dev.off()
```

9.8. *Graphing LMCI with and without Aspirin use*

9.8.1. *MMSE*

```
red<-newdat[newdat$APOE4==0 & newdat$Gender=="Female" & newdat$edu.cat=="1post"&
newdat$diagn=="3LMCI"& newdat$pain.medication=="aspirin",]
blue<-newdat[newdat$APOE4==0 & newdat$Gender=="Female" &
newdat$edu.cat=="1post"& newdat$diagn=="3LMCI"&
newdat$pain.medication=="No.Painrelief",]
```

```
plotdat<-rbind(red,blue)


Month<-c(0,6,12,18,24,36,48,60,72,84,96,108,120)
yaxismin<-max((floor(min(plotdat$MMSEestimate))-1),5)

xmax<-min(min(max(blue$M[which(blue$UCI>5,
arr.ind=TRUE)]),120),min(max(red$M[which(red$UCI>5, arr.ind=TRUE)]),120))

#PDF("MMSEAspirin.#PDF", width=16/2.54, heigh=12/2.54 )
plot(Month,red$MMSEestimate,pch=NA,ylab="MMSE", xlab="Month",ylim=c(yaxismin,
30),xlim=c(-2,xmax),axes = FALSE)
axis(1,seq(0,xmax,by=12))
axis(2,at=seq(30,yaxismin,by=-3), cex.axis=0.8)
polygon(c(rev(Month), Month), c(rev(red$LCI), red$UCI), col = rgb(0.8,0,0,0.2),
border = NA)
polygon(c(rev(Month), Month), c(rev(blue$LCI), blue$UCI), col =
rgb(0,0,0.8,0.2), border = NA)
lines(Month,red$MMSEestimate,col="#db4c4c",lty=1,lwd=3)
lines(Month,blue$MMSEestimate,col="#66a3e0",lty=1,lwd=3)
legend("bottomleft", bty = "n", legend = as.character(c("Non-User", "Aspirin
User")),
       pch = 15, col = c("#7cafe2","#f78a8a"), cex = 1, horiz=F)
```

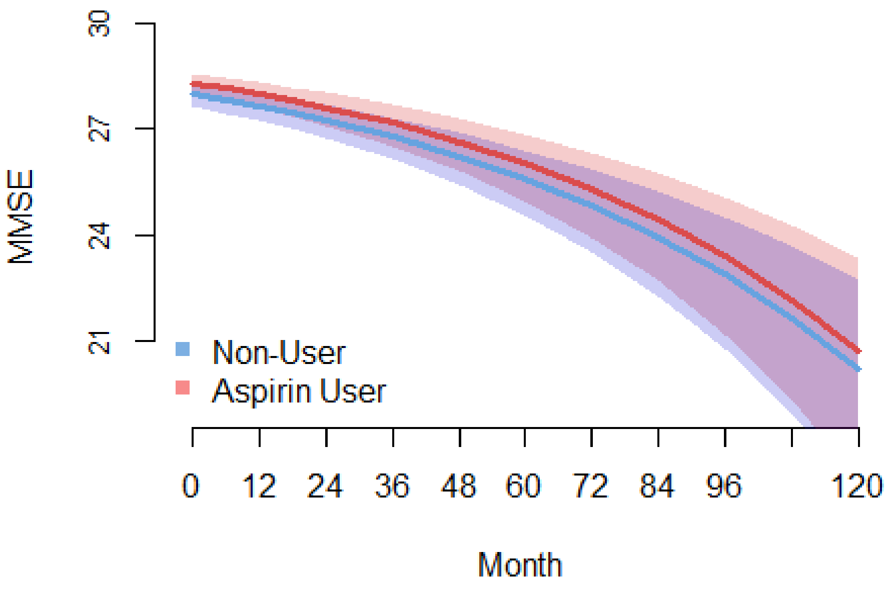


```
#dev.off()
```

9.8.2. ADAS

```
redADAS<-newdatADAS[newdatADAS$APOE4==0 & newdatADAS$headache==0 &
newdatADAS$Gender=="Female" & newdatADAS$edu.cat=="1post"&
newdatADAS$diagn=="3LMCI"& newdatADAS$pain.medication=="aspirin",]
blueADAS<-newdatADAS[newdatADAS$APOE4==0 & newdatADAS$headache==0 &
```

```
newdatADAS$Gender==“Female" & newdatADAS$edu.cat=="1post"&
newdatADAS$diagn=="3LMCI"& newdatADAS$pain.medication=="No.Painrelief",]

plotdat<-rbind(redADAS,blueADAS)


Month<-c(0,6,12,18,24,36,48,60,72,84,96,108,120)
yaxismin<-max(min(c(plotdat$LCI)-5),0)
yaxismax<-min(max(redADAS$UCI,blueADAS$UCI)+5,90)
xmax<-min(min(max(blueADAS$M[which(blueADAS$UCI<70,
arr.ind=TRUE)]),120),min(max(redADAS$M[which(redADAS$UCI<70,
arr.ind=TRUE)]),120))

#PDF("ADASAspirin.#PDF", width=16/2.54, heigh=12/2.54 )
plot(Month,redADAS$ADASestimate,pch=NA,ylab="ADAS",
xlab="Month",ylim=c(yaxismin, yaxismax),xlim=c(-2,xmax),axes = FALSE)
axis(1,seq(0,xmax,by=12))
axis(2,at=seq(0,yaxismax,by=10),cex.axis=0.8)
polygon(c(rev(Month), Month), c(rev(redADAS$LCI), redADAS$UCI), col =
rgb(0.8,0,0,0.2), border = NA)
polygon(c(rev(Month), Month), c(rev(blueADAS$LCI), blueADAS$UCI), col =
rgb(0,0,0.8,0.2), border = NA)
lines(Month,redADAS$ADASestimate,col="#db4c4c",lty=1,lwd=3)
lines(Month,blueADAS$ADASestimate,col="#66a3e0",lty=1,lwd=3)
legend("topleft", bty = "n", legend = as.character(c("Non-User", "Aspirin
User")),
       pch = 15, col = c("#7cafe2","#f78a8a"), cex = 1, horiz=F)
```

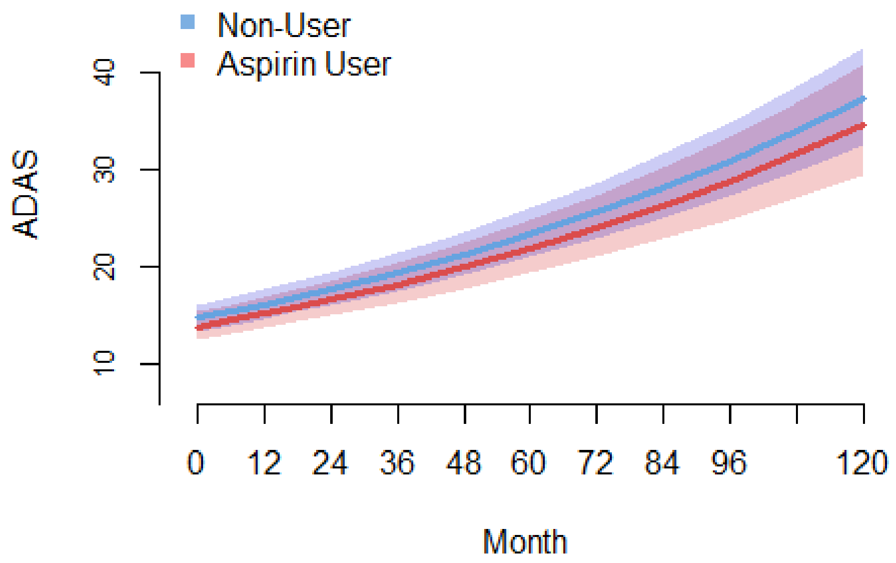


```
#dev.off()
```

9.9. *Graphing LMCI with and without Celecoxib use*

9.9.1. *MMSE*

```
red<-newdat[newdat$APOE4==0 & newdat$Gender=="Female" & newdat$edu.cat=="1post"&
newdat$diagn=="3LMCI"& newdat$pain.medication=="celecoxib",]
blue<-newdat[newdat$APOE4==0 & newdat$Gender=="Female" &
newdat$edu.cat=="1post"& newdat$diagn=="3LMCI"&
newdat$pain.medication=="No.Painrelief",]

plotdat<-rbind(red,blue)


Month<-c(0,6,12,18,24,36,48,60,72,84,96,108,120)
yaxismin<-max((floor(min(plotdat$MMSEestimate))-1),5)

xmax<-min(min(max(blue$M[which(blue$UCI>5,
arr.ind=TRUE)]),120),min(max(red$M[which(red$UCI>5, arr.ind=TRUE)]),120))

#PDF("MMSECelecoxib.#PDF", width=16/2.54, heigh=12/2.54 )
plot(Month,red$MMSEestimate,pch=NA,ylab="MMSE", xlab="Month",ylim=c(yaxismin,
30),xlim=c(-2,xmax),axes = FALSE)
axis(1,seq(0,xmax,by=12))
axis(2,at=seq(30,yaxismin,by=-3), cex.axis=0.8)
polygon(c(rev(Month), Month), c(rev(red$LCI), red$UCI), col = rgb(0.8,0,0,0.2),
border = NA)
polygon(c(rev(Month), Month), c(rev(blue$LCI), blue$UCI), col =
rgb(0,0,0.8,0.2), border = NA)
lines(Month,red$MMSEestimate,col="#db4c4c",lty=1,lwd=3)
lines(Month,blue$MMSEestimate,col="#66a3e0",lty=1,lwd=3)
legend("bottomleft", bty = "n", legend = as.character(c("Non-User", "Celecoxib
User")),
       pch = 15, col = c("#7cafe2","#f78a8a"), cex = 1, horiz=F)
```

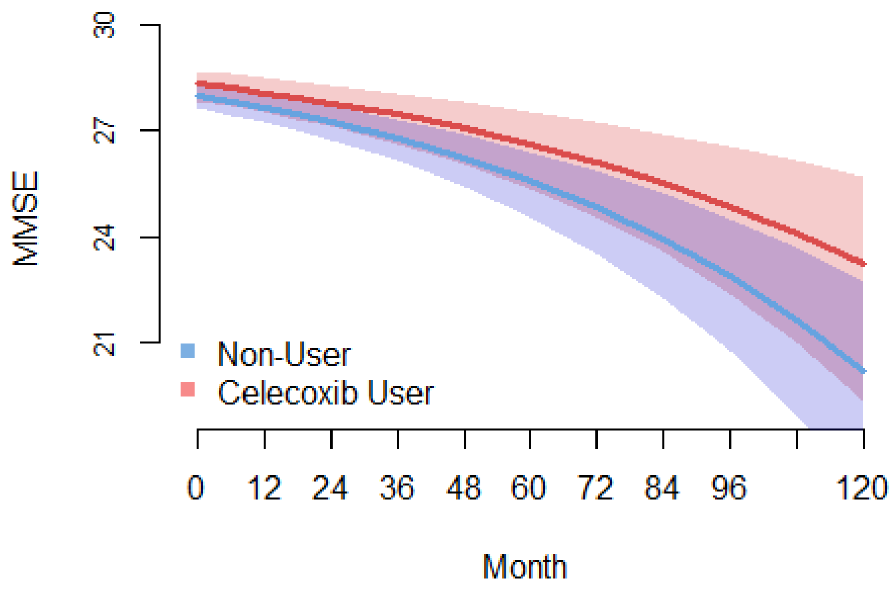

```
#dev.off()
```

9.9.2. ADAS

```
redADAS<-newdatADAS[newdatADAS$APOE4==0 & newdatADAS$headache==0 &
newdatADAS$Gender=="Female" & newdatADAS$edu.cat=="1post"&
newdatADAS$diagn=="3LMCI"& newdatADAS$pain.medication=="celecoxib",]
blueADAS<-newdatADAS[newdatADAS$APOE4==0 & newdatADAS$headache==0 &
newdatADAS$Gender=="Female" & newdatADAS$edu.cat=="1post"&
newdatADAS$diagn=="3LMCI"& newdatADAS$pain.medication=="No.Painrelief",]

plotdat<-rbind(redADAS,blueADAS)


Month<-c(0,6,12,18,24,36,48,60,72,84,96,108,120)
yaxismin<-max(min(c(plotdat$LCI)-5),0)
yaxismax<-min(max(redADAS$UCI,blueADAS$UCI)+5,90)
xmax<-min(min(max(blueADAS$M[which(blueADAS$UCI<70,
arr.ind=TRUE)]),120),min(max(redADAS$M[which(redADAS$UCI<70,
arr.ind=TRUE)]),120))

#PDF("ADASCelecoxib.#PDF", width=16/2.54, heigh=12/2.54 )
plot(Month,redADAS$ADASestimate,pch=NA,ylab="ADAS",
xlab="Month",ylim=c(yaxismin, yaxismax),xlim=c(-2,xmax),axes = FALSE)
axis(1,seq(0,xmax,by=12))
axis(2,at=seq(0,yaxismax,by=10),cex.axis=0.8)
polygon(c(rev(Month), Month), c(rev(redADAS$LCI), redADAS$UCI), col =
rgb(0.8,0,0,0.2), border = NA)
polygon(c(rev(Month), Month), c(rev(blueADAS$LCI), blueADAS$UCI), col =
rgb(0,0,0.8,0.2), border = NA)
lines(Month,redADAS$ADASestimate,col="#db4c4c",lty=1,lwd=3)
lines(Month,blueADAS$ADASestimate,col="#66a3e0",lty=1,lwd=3)
legend("topleft", bty = "n", legend = as.character(c("Non-User", "Celecoxib
User")),
       pch = 15, col = c("#7cafe2","#f78a8a"), cex = 1, horiz=F)
```

Only

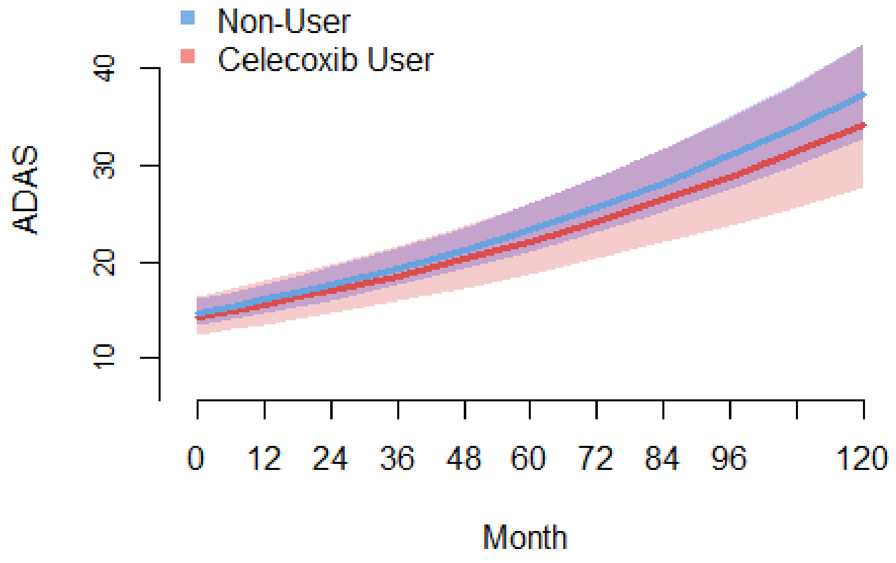


```
#dev.off()
```

9.10. *Graphing LMCI with and without Paracetamol use*

9.10.1. MMSE

```
red<-newdat[newdat$APOE4==0 & newdat$Gender=="Female" & newdat$edu.cat=="1post"&
newdat$diagn=="3LMCI"& newdat$pain.medication=="paracetamol",]
blue<-newdat[newdat$APOE4==0 & newdat$Gender=="Female" &
newdat$edu.cat=="1post"& newdat$diagn=="3LMCI"&
newdat$pain.medication=="No.Painrelief",]

plotdat<-rbind(red,blue)


Month<-c(0,6,12,18,24,36,48,60,72,84,96,108,120)
yaxismin<-max((floor(min(plotdat$MMSEestimate))-1),5)

xmax<-min(min(max(blue$M[which(blue$UCI>5,
arr.ind=TRUE)]),120),min(max(red$M[which(red$UCI>5, arr.ind=TRUE)]),120))

#PDF("MMSEParacetamol.#PDF", width=16/2.54, heigh=12/2.54 )
plot(Month,red$MMSEestimate,pch=NA,ylab="MMSE", xlab="Month",ylim=c(yaxismin,
30),xlim=c(-2,xmax),axes = FALSE)
axis(1,seq(0,xmax,by=12))
axis(2,at=seq(30,yaxismin,by=-3), cex.axis=0.8)
polygon(c(rev(Month), Month), c(rev(red$LCI), red$UCI), col = rgb(0.8,0,0,0.2),
border = NA)
polygon(c(rev(Month), Month), c(rev(blue$LCI), blue$UCI), col =
rgb(0,0,0.8,0.2), border = NA)
lines(Month,red$MMSEestimate,col="#db4c4c",lty=1,lwd=3)
lines(Month,blue$MMSEestimate,col="#66a3e0",lty=1,lwd=3)
```

```
legend("bottomleft", bty = "n", legend = as.character(c("Non-User", "Paracetamol
User")),
        pch = 15, col = c("#7cafe2","#f78a8a"), cex = 1, horiz=F)
```

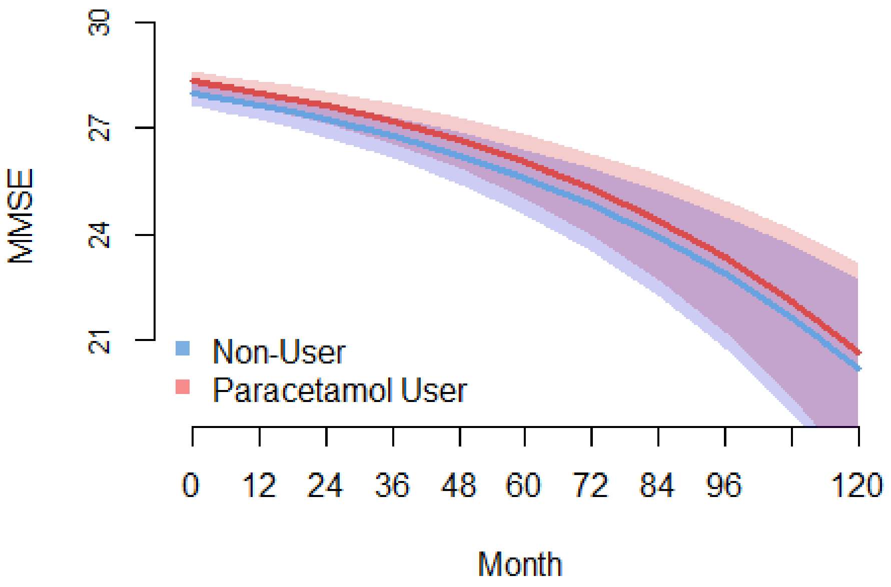


```
#dev.off()
```

9.10.2. ADAS

```
redADAS<-newdatADAS[newdatADAS$APOE4==0 & newdatADAS$headache==0 &
newdatADAS$Gender=="Female" & newdatADAS$edu.cat=="1post"&
newdatADAS$diagn=="3LMCI"& newdatADAS$pain.medication=="paracetamol",]
blueADAS<-newdatADAS[newdatADAS$APOE4==0 & newdatADAS$headache==0 &
newdatADAS$Gender=="Female" & newdatADAS$edu.cat=="1post"&
newdatADAS$diagn=="3LMCI"& newdatADAS$pain.medication=="No.Painrelief",]

plotdat<-rbind(redADAS,blueADAS)


Month<-c(0,6,12,18,24,36,48,60,72,84,96,108,120)
yaxismin<-max(min(c(plotdat$LCI)-5),0)
yaxismax<-min(max(redADAS$UCI,blueADAS$UCI)+5,90)
xmax<-min(min(max(blueADAS$M[which(blueADAS$UCI<70,
arr.ind=TRUE)]),120),min(max(redADAS$M[which(redADAS$UCI<70,
arr.ind=TRUE)]),120))

#PDF("ADASParacetamol.#PDF", width=16/2.54, heigh=12/2.54 )
plot(Month,redADAS$ADASestimate,pch=NA,ylab="ADAS",
xlab="Month",ylim=c(yaxismin, yaxismax),xlim=c(-2,xmax),axes = FALSE)
axis(1,seq(0,xmax,by=12))
axis(2,at=seq(0,yaxismax,by=10),cex.axis=0.8)
polygon(c(rev(Month), Month), c(rev(redADAS$LCI), redADAS$UCI), col =
rgb(0.8,0,0,0.2), border = NA)
polygon(c(rev(Month), Month), c(rev(blueADAS$LCI), blueADAS$UCI), col =
```

```
rgb(0,0,0.8,0.2), border = NA)
lines(Month,redADAS$ADASestimate,col="#db4c4c",lty=1,lwd=3)
lines(Month,blueADAS$ADASestimate,col="#66a3e0",lty=1,lwd=3)
legend("topleft", bty = "n", legend = as.character(c("Non-User", "Paracetamol User")),
       pch = 15, col = c("#7cafe2","#f78a8a"), cex = 1, horiz=F)
```

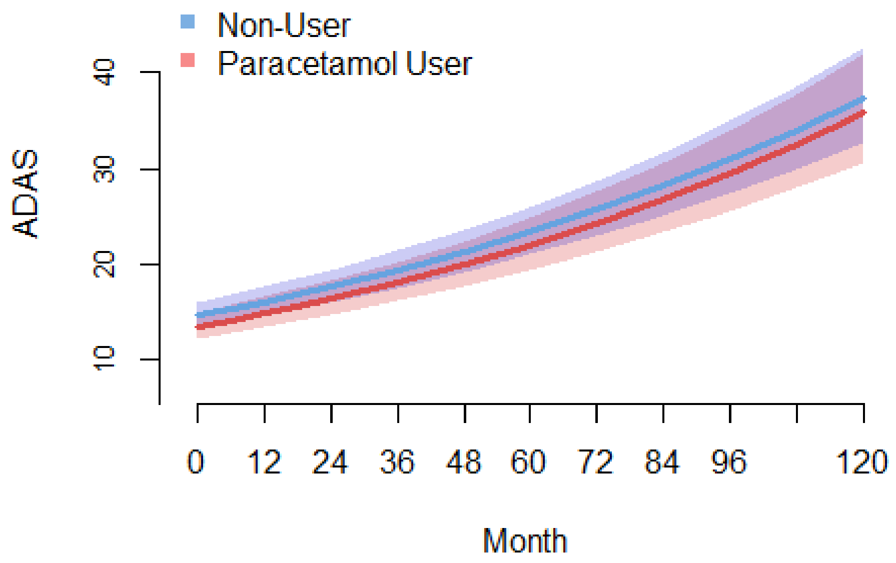


```
#dev.off()
```

9.11. *Graphing LMCI with and without NSAID use*

9.11.1. MMSE

```
red<-newdat[newdat$APOE4==0 & newdat$Gender=="Female" & newdat$edu.cat=="1post"&
newdat$diagn=="3LMCI"& newdat$pain.medication=="NSAIDS",]
blue<-newdat[newdat$APOE4==0 & newdat$Gender=="Female" &
newdat$edu.cat=="1post"& newdat$diagn=="3LMCI"&
newdat$pain.medication=="No.Painrelief",]

plotdat<-rbind(red,blue)


Month<-c(0,6,12,18,24,36,48,60,72,84,96,108,120)
yaxismin<-max((floor(min(plotdat$MMSEestimate))-1),5)

xmax<-min(min(max(blue$M[which(blue$UCI>5,
arr.ind=TRUE)]),120),min(max(red$M[which(red$UCI>5, arr.ind=TRUE)]),120))

#PDF("MMSENSAID.#PDF", width=16/2.54, heigh=12/2.54 )
plot(Month,red$MMSEestimate,pch=NA,ylab="MMSE", xlab="Month",ylim=c(yaxismin,
30),xlim=c(-2,xmax),axes = FALSE)
axis(1,seq(0,xmax,by=12))
```

```
axis(2,at=seq(30,yaxismin,by=-3), cex.axis=0.8)
polygon(c(rev(Month), Month), c(rev(red$LCI), red$UCI), col = rgb(0.8,0,0,0.2),
border = NA)
polygon(c(rev(Month), Month), c(rev(blue$LCI), blue$UCI), col =
rgb(0,0,0.8,0.2), border = NA)
lines(Month,red$MMSEestimate,col="#db4c4c",lty=1,lwd=3)
lines(Month,blue$MMSEestimate,col="#66a3e0",lty=1,lwd=3)
legend("bottomleft", bty = "n", legend = as.character(c("Non-User", "NSAID
User")),
       pch = 15, col = c("#7cafe2","#f78a8a"), cex = 1, horiz=F)
```

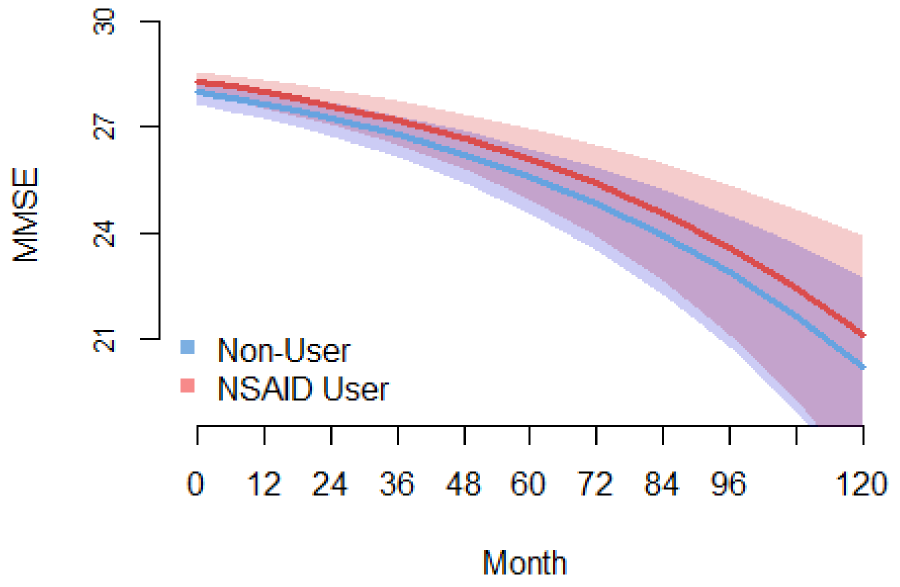


```
#dev.off()
```

9.11.2. ADAS

```
redADAS<-newdatADAS[newdatADAS$APOE4==0 & newdatADAS$headache==0 &
newdatADAS$Gender=="Female" & newdatADAS$edu.cat=="1post"&
newdatADAS$diagn=="3LMCI"& newdatADAS$pain.medication=="NSAIDS",]
blueADAS<-newdatADAS[newdatADAS$APOE4==0 & newdatADAS$headache==0 &
newdatADAS$Gender=="Female" & newdatADAS$edu.cat=="1post"&
newdatADAS$diagn=="3LMCI"& newdatADAS$pain.medication=="No.Painrelief",]

plotdat<-rbind(redADAS,blueADAS)


Month<-c(0,6,12,18,24,36,48,60,72,84,96,108,120)
yaxismin<-max(min(c(plotdat$LCI)-5),0)
yaxismax<-min(max(redADAS$UCI,blueADAS$UCI)+5,90)
xmax<-min(min(max(blueADAS$M[which(blueADAS$UCI<70,
arr.ind=TRUE)]),120),min(max(redADAS$M[which(redADAS$UCI<70,
arr.ind=TRUE)]),120))

#PDF("ADASNSAID.#PDF", width=16/2.54, heigh=12/2.54 )
```

```
plot(Month,redADAS$ADASestimate,pch=NA,ylab="ADAS",
xlab="Month",ylim=c(yaxismin, yaxismax),xlim=c(-2,xmax),axes = FALSE)
axis(1,seq(0,xmax,by=12))
axis(2,at=seq(0,yaxismax,by=10),cex.axis=0.8)
polygon(c(rev(Month), Month), c(rev(redADAS$LCI), redADAS$UCI), col =
rgb(0.8,0,0,0.2), border = NA)
polygon(c(rev(Month), Month), c(rev(blueADAS$LCI), blueADAS$UCI), col =
rgb(0,0,0.8,0.2), border = NA)
lines(Month,redADAS$ADASestimate,col="#db4c4c",lty=1,lwd=3)
lines(Month,blueADAS$ADASestimate,col="#66a3e0",lty=1,lwd=3)
legend("topleft", bty = "n", legend = as.character(c("Non-User", "NSAID User")),
       pch = 15, col = c("#7cafe2","#f78a8a"), cex = 1, horiz=F)
```

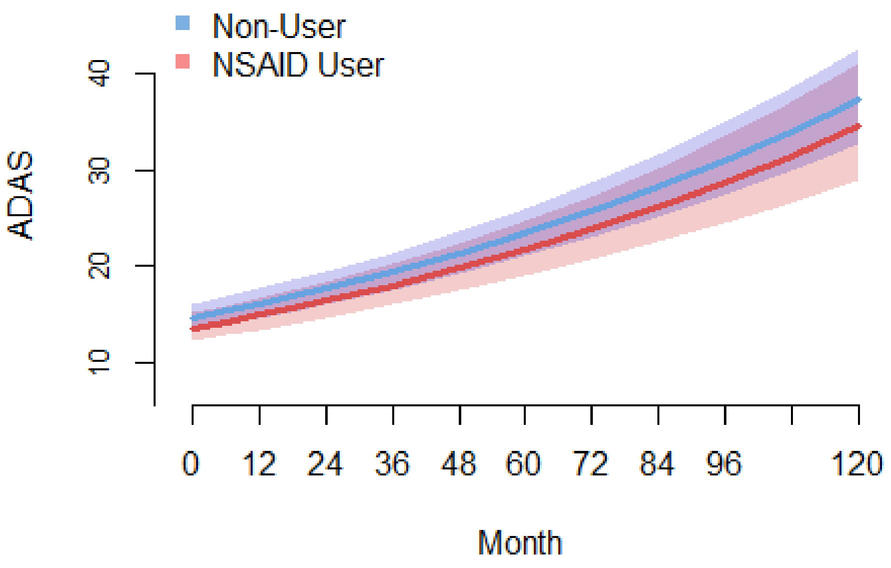


```
#dev.off()
```

9.12. *Graphing LMCI with and without APOE genotypes*

9.12.1. MMSE

```
red<-newdat[newdat$APOE4==2 & newdat$Gender=="Female" & newdat$edu.cat=="1post"&
newdat$diagn=="3LMCI"& newdat$pain.medication=="No.Painrelief",]
orange<-newdat[newdat$APOE4==1 & newdat$Gender=="Female" &
newdat$edu.cat=="1post"& newdat$diagn=="3LMCI"&
newdat$pain.medication=="No.Painrelief",]
blue<-newdat[newdat$APOE4==0 & newdat$Gender=="Female" &
newdat$edu.cat=="1post"& newdat$diagn=="3LMCI"&
newdat$pain.medication=="No.Painrelief",]

plotdat<-rbind(red,blue,orange)


Month<-c(0,6,12,18,24,36,48,60,72,84,96,108,120)
```

```
yaxismin<-max((floor(min(plotdat$MMSEestimate))-1),5)

xmax<-min(min(max(blue$M[which(blue$UCI>5,
arr.ind=TRUE)]),120),min(max(red$M[which(red$UCI>5, arr.ind=TRUE)]),120))

#PDF("MMSEAPOE.#PDF", width=16/2.54, heigh=12/2.54 )
plot(Month,red$MMSEestimate,pch=NA,ylab="MMSE", xlab="Month",ylim=c(yaxismin,
30),xlim=c(-2,xmax),axes = FALSE)
axis(1,seq(0,xmax,by=12))
axis(2,at=seq(30,yaxismin,by=-3), cex.axis=0.8)
polygon(c(rev(Month), Month), c(rev(red$LCI), red$UCI), col = rgb(0.8,0,0,0.2),
border = NA)
polygon(c(rev(Month), Month), c(rev(blue$LCI), blue$UCI), col =
rgb(0,0,0.8,0.2), border = NA)
polygon(c(rev(Month), Month), c(rev(orange$LCI), orange$UCI), col =
rgb(0.8,0.3,0,0.3), border = NA)
lines(Month,red$MMSEestimate,col="#db4c4c",lty=1,lwd=3)
lines(Month,blue$MMSEestimate,col="#66a3e0",lty=1,lwd=3)
lines(Month,orange$MMSEestimate,col="#e4630099",lty=1,lwd=3)
legend("bottomleft", bty = "n", legend = as.character(c("APOE4-/-", "APOE4+/-
","APOE4+/+")),
       pch = 15, col = c("#7cafe2","#e4630066","#f78a8a"), cex = 1, horiz=F)
```

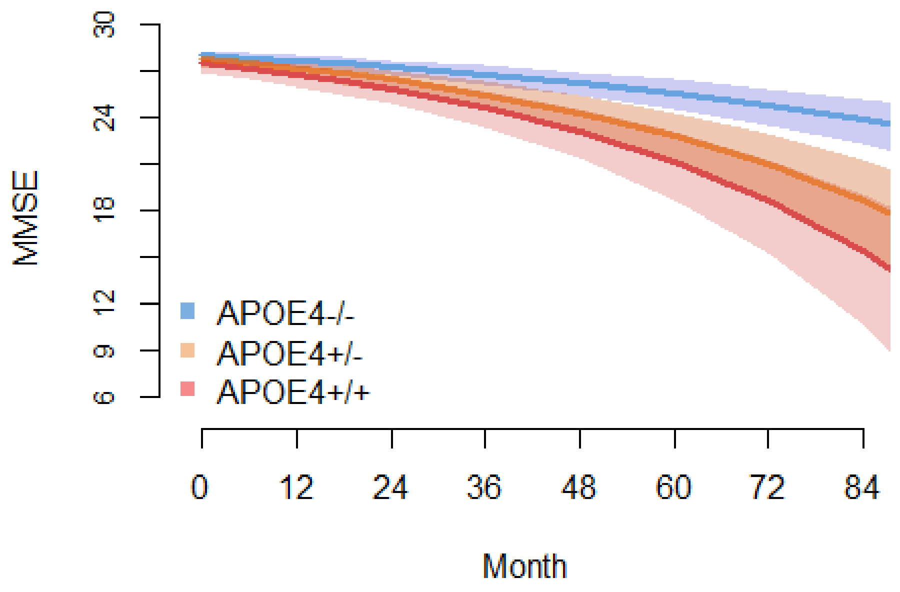


```
#dev.off()
```

9.12.2. ADAS

```
redADAS<-newdatADAS[newdatADAS$APOE4==2 & newdatADAS$headache==0 &
newdatADAS$Gender=="Female" & newdatADAS$edu.cat=="1post"&
newdatADAS$diagn=="3LMCI"& newdatADAS$pain.medication=="No.Painrelief",]

orangeADAS<-newdatADAS[newdatADAS$APOE4==1 & newdatADAS$headache==0 &
newdatADAS$Gender=="Female" & newdatADAS$edu.cat=="1post"&
```

```
newdatADAS$diagn=="3LMCI"& newdatADAS$pain.medication=="No.Painrelief",]

blueADAS<-newdatADAS[newdatADAS$APOE4==0 & newdatADAS$headache==0 &
newdatADAS$Gender=="Female" & newdatADAS$edu.cat=="1post"&
newdatADAS$diagn=="3LMCI"& newdatADAS$pain.medication=="No.Painrelief",]



plotdat<-rbind(redADAS,orangeADAS, blueADAS)


Month<-c(0,6,12,18,24,36,48,60,72,84,96,108,120)
yaxismin<-max(min(c(plotdat$LCI)-5),0)
yaxismax<-min(max(redADAS$UCI,blueADAS$UCI)+5,90)
xmax<-min(min(max(blueADAS$M[which(blueADAS$UCI<70,
arr.ind=TRUE)]),120),min(max(redADAS$M[which(redADAS$UCI<70,
arr.ind=TRUE)]),120))

#PDF("ADASAPOE.#PDF", width=16/2.54, heigh=12/2.54 )
plot(Month,redADAS$ADASestimate,pch=NA,ylab="ADAS",
xlab="Month",ylim=c(yaxismin, yaxismax),xlim=c(-2,xmax),axes = FALSE)
axis(1,seq(0,xmax,by=12))
axis(2,at=seq(0,yaxismax,by=10),cex.axis=0.8)
polygon(c(rev(Month), Month), c(rev(redADAS$LCI), redADAS$UCI), col =
rgb(0.8,0,0,0.2), border = NA)
polygon(c(rev(Month), Month), c(rev(blueADAS$LCI), blueADAS$UCI), col =
rgb(0,0,0.8,0.2), border = NA)
polygon(c(rev(Month), Month), c(rev(orangeADAS$LCI), orangeADAS$UCI), col =
rgb(0.8,0.3,0,0.3), border = NA)

lines(Month,redADAS$ADASestimate,col="#db4c4c",lty=1,lwd=3)
lines(Month,blueADAS$ADASestimate,col="#66a3e0",lty=1,lwd=3)
lines(Month,orangeADAS$ADASestimate,col="#e4630099",lty=1,lwd=3)

legend("topleft", bty = "n", legend = as.character(c("APOE4-/-", "APOE4+/-
","APOE4+/+")),
       pch = 15, col = c("#7cafe2","#e4630066","#f78a8a"), cex = 1, horiz=F)
```

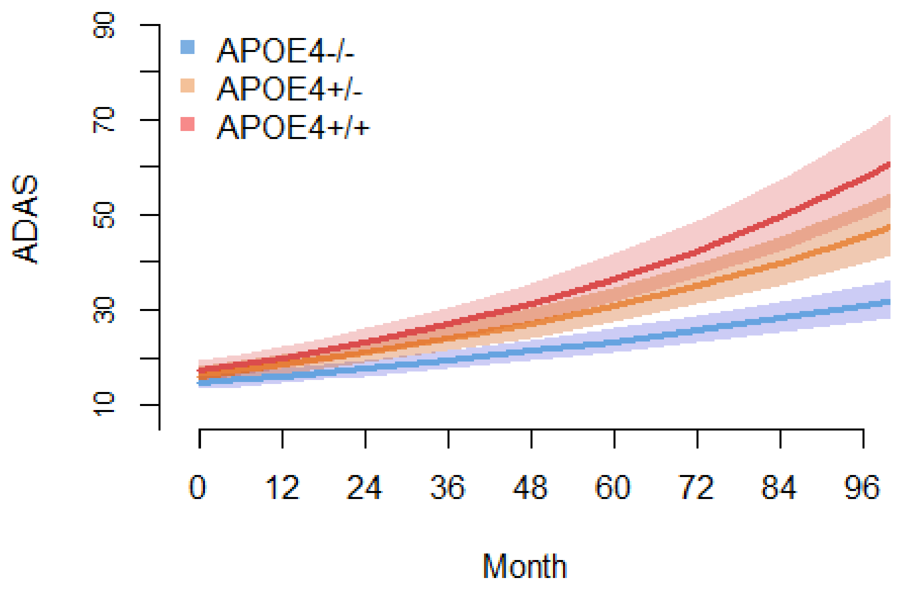


```
#dev.off()
```

9.13. *Graphing LMCI between Genders*

9.13.1. MMSE

```
red<-newdat[newdat$APOE4==0 & newdat$Gender=="Female" & newdat$edu.cat=="1post"&
newdat$diagn=="3LMCI"& newdat$pain.medication=="No.Painrelief",]
blue<-newdat[newdat$APOE4==0 & newdat$Gender=="Male" & newdat$edu.cat=="1post"&
newdat$diagn=="3LMCI"& newdat$pain.medication=="No.Painrelief",]

plotdat<-rbind(red,blue)


Month<-c(0,6,12,18,24,36,48,60,72,84,96,108,120)
yaxismin<-max((floor(min(plotdat$MMSEestimate))-1),5)

xmax<-min(min(max(blue$M[which(blue$UCI>5,
arr.ind=TRUE)]),120),min(max(red$M[which(red$UCI>5, arr.ind=TRUE)]),120))

#PDF("MMSEGender.#PDF", width=16/2.54, heigh=12/2.54 )
plot(Month,red$MMSEestimate,pch=NA,ylab="MMSE", xlab="Month",ylim=c(yaxismin,
30),xlim=c(-2,xmax),axes = FALSE)
axis(1,seq(0,xmax,by=12))
axis(2,at=seq(30,yaxismin,by=-3), cex.axis=0.8)
polygon(c(rev(Month), Month), c(rev(red$LCI), red$UCI), col = rgb(0.8,0,0,0.2),
border = NA)
polygon(c(rev(Month), Month), c(rev(blue$LCI), blue$UCI), col =
rgb(0,0,0.8,0.2), border = NA)
lines(Month,red$MMSEestimate,col="#db4c4c",lty=1,lwd=3)
lines(Month,blue$MMSEestimate,col="#66a3e0",lty=1,lwd=3)
```

```
legend("bottomleft", bty = "n", legend = as.character(c("Male", "Female")),
       pch = 15, col = c("#7cafe2","#f78a8a"), cex = 1, horiz=F)
```

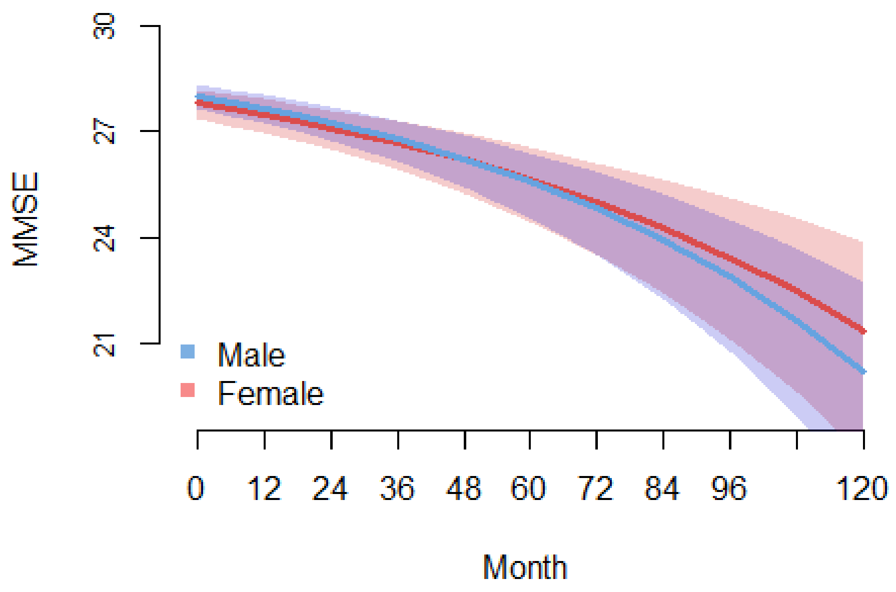


```
#dev.off()
```

9.13.2. ADAS

```
redADAS<-newdatADAS[newdatADAS$APOE4==0 & newdatADAS$headache==0 &
newdatADAS$Gender=="Female" & newdatADAS$edu.cat=="1post"&
newdatADAS$diagn=="3LMCI"& newdatADAS$pain.medication=="No.Painrelief",]
blueADAS<-newdatADAS[newdatADAS$APOE4==0 & newdatADAS$headache==0 &
newdatADAS$Gender=="Male" & newdatADAS$edu.cat=="1post"&
newdatADAS$diagn=="3LMCI"& newdatADAS$pain.medication=="No.Painrelief",]

plotdat<-rbind(redADAS,blueADAS)


Month<-c(0,6,12,18,24,36,48,60,72,84,96,108,120)
yaxismin<-max(min(c(plotdat$LCI)-5),0)
yaxismax<-min(max(redADAS$UCI,blueADAS$UCI)+5,90)
xmax<-min(min(max(blueADAS$M[which(blueADAS$UCI<70,
arr.ind=TRUE)]),120),min(max(redADAS$M[which(redADAS$UCI<70,
arr.ind=TRUE)]),120))

#PDF("ADASGender.#PDF", width=16/2.54, heigh=12/2.54 )
plot(Month,redADAS$ADASestimate,pch=NA,ylab="ADAS",
xlab="Month",ylim=c(yaxismin, yaxismax),xlim=c(-2,xmax),axes = FALSE)
axis(1,seq(0,xmax,by=12))
axis(2,at=seq(0,yaxismax,by=10),cex.axis=0.8)
polygon(c(rev(Month), Month), c(rev(redADAS$LCI), redADAS$UCI), col =
rgb(0.8,0,0,0.2), border = NA)
polygon(c(rev(Month), Month), c(rev(blueADAS$LCI), blueADAS$UCI), col =
rgb(0,0,0.8,0.2), border = NA)
```

```
lines(Month,redADAS$ADASestimate,col="#db4c4c",lty=1,lwd=3)
lines(Month,blueADAS$ADASestimate,col="#66a3e0",lty=1,lwd=3)
legend("topleft", bty = "n", legend = as.character(c("Male", "Female")),
       pch = 15, col = c("#7cafe2","#f78a8a"), cex = 1, horiz=F)
```

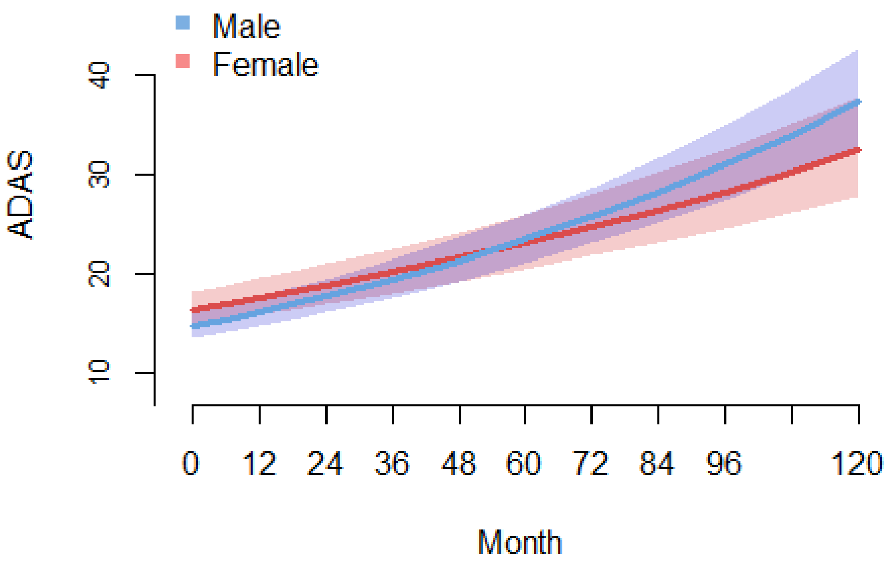


```
#dev.off()
```

9.14. *Graphing Cogntive decline of diagnosis*

9.14.1. MMSE

```
red<-newdat[newdat$APOE4==0 & newdat$Gender=="Female" & newdat$edu.cat=="1post"&
newdat$diagn=="4AD"& newdat$pain.medication=="No.Painrelief",]
orange<-newdat[newdat$APOE4==0 & newdat$Gender=="Female" &
newdat$edu.cat=="1post"& newdat$diagn=="3LMCI"&
newdat$pain.medication=="No.Painrelief",]
blue<-newdat[newdat$APOE4==0 & newdat$Gender=="Female" &
newdat$edu.cat=="1post"& newdat$diagn=="1CN"&
newdat$pain.medication=="No.Painrelief",]
green<-newdat[newdat$APOE4==0 & newdat$Gender=="Female" &
newdat$edu.cat=="1post"& newdat$diagn=="2EMCI"&
newdat$pain.medication=="No.Painrelief",]

plotdat<-rbind(red,blue,orange,green)


Month<-c(0,6,12,18,24,36,48,60,72,84,96,108,120)
yaxismin<-max((floor(min(plotdat$MMSEestimate))-1),5)

xmax<-min(min(max(blue$M[which(blue$UCI>5,
arr.ind=TRUE)]),120),min(max(red$M[which(red$UCI>5, arr.ind=TRUE)]),120))
```

```
#PDF("MMSEDiagnosis.#PDF", width=16/2.54, heigh=12/2.54 )
plot(Month,red$MMSEestimate,pch=NA,ylab="MMSE", xlab="Month",ylim=c(yaxismin,
30),xlim=c(-2,xmax),axes = FALSE)
axis(1,seq(0,xmax,by=12))
axis(2,at=seq(30,yaxismin,by=-3), cex.axis=0.8)
polygon(c(rev(Month), Month), c(rev(red$LCI), red$UCI), col = rgb(0.8,0,0,0.2),
border = NA)
polygon(c(rev(Month), Month), c(rev(blue$LCI), blue$UCI), col =
rgb(0,0,0.8,0.2), border = NA)
polygon(c(rev(Month), Month), c(rev(orange$LCI), orange$UCI), col =
rgb(0.8,0.3,0,0.3), border = NA)
polygon(c(rev(Month), Month), c(rev(green$LCI), green$UCI), col =
rgb(191/255,208/255,52/255,0.4), border = NA)
lines(Month,red$MMSEestimate,col="#db4c4c",lty=1,lwd=3)
lines(Month,blue$MMSEestimate,col="#66a3e0",lty=1,lwd=3)
lines(Month,orange$MMSEestimate,col="#e4630099",lty=1,lwd=3)
lines(Month,green$MMSEestimate,col="#bfd03499",lty=1,lwd=3)
legend("bottomleft", bty = "n", legend = as.character(c("CN",
"EMCI","LMCI","AD")),
       pch = 15, col = c("#7cafe2","#bfd03499","#e4630066","#f78a8a"), cex = 1,
horiz=F)
```

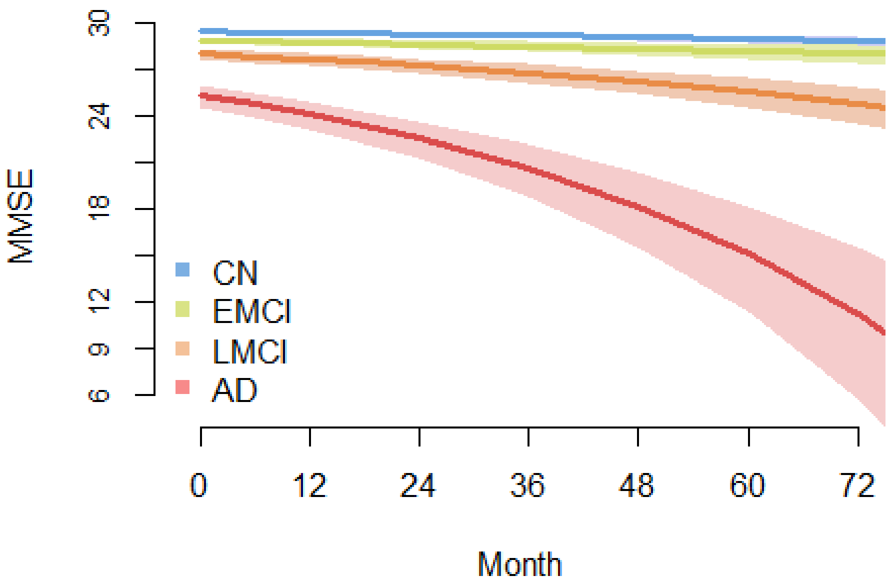


```
#dev.off()
```

9.14.2. ADAS

```
redADAS<-newdatADAS[newdatADAS$APOE4==0& newdatADAS$headache==0  &
newdatADAS$Gender=="Female" & newdatADAS$edu.cat=="1post"&
newdatADAS$diagn=="4AD"& newdatADAS$pain.medication=="No.Painrelief",]

orangeADAS<-newdatADAS[newdatADAS$APOE4==0& newdatADAS$headache==0  &
newdatADAS$Gender=="Female" & newdatADAS$edu.cat=="1post"&
newdatADAS$diagn=="3LMCI"& newdatADAS$pain.medication=="No.Painrelief",]
```

```
blueADAS<-newdatADAS[newdatADAS$APOE4==0 & newdatADAS$headache==0 &
newdatADAS$Gender=="Female" & newdatADAS$edu.cat=="1post"&
newdatADAS$diagn=="1CN"& newdatADAS$pain.medication=="No.Painrelief",]

greenADAS<-newdatADAS[newdatADAS$APOE4==0 & newdatADAS$headache==0 &
newdatADAS$Gender=="Female" & newdatADAS$edu.cat=="1post"&
newdatADAS$diagn=="2EMCI"& newdatADAS$pain.medication=="No.Painrelief",]



plotdat<-rbind(redADAS,orangeADAS, blueADAS, greenADAS)


Month<-c(0,6,12,18,24,36,48,60,72,84,96,108,120)
yaxismin<-max(min(c(plotdat$LCI)-5),0)
yaxismax<-min(max(redADAS$UCI,blueADAS$UCI)+5,90)
xmax<-min(min(max(blueADAS$M[which(blueADAS$UCI<70,
arr.ind=TRUE)]),120),min(max(redADAS$M[which(redADAS$UCI<90,
arr.ind=TRUE)]),120))

#PDF("ADASDiagnosis.#PDF", width=16/2.54, heigh=12/2.54 )
plot(Month,redADAS$ADASestimate,pch=NA,ylab="ADAS",
xlab="Month",ylim=c(yaxismin, yaxismax),xlim=c(-2,xmax),axes = FALSE)
axis(1,seq(0,xmax,by=12))
axis(2,at=seq(0,yaxismax,by=10),cex.axis=0.8)
polygon(c(rev(Month), Month), c(rev(redADAS$LCI), redADAS$UCI), col =
rgb(0.8,0,0,0.2), border = NA)
polygon(c(rev(Month), Month), c(rev(blueADAS$LCI), blueADAS$UCI), col =
rgb(0,0,0.8,0.2), border = NA)
polygon(c(rev(Month), Month), c(rev(orangeADAS$LCI), orangeADAS$UCI), col =
rgb(0.8,0.3,0,0.3), border = NA)
polygon(c(rev(Month), Month), c(rev(greenADAS$LCI), greenADAS$UCI), col =
rgb(191/255,208/255,52/255,0.4), border = NA)
lines(Month,redADAS$ADASestimate,col="#db4c4c",lty=1,lwd=3)
lines(Month,blueADAS$ADASestimate,col="#66a3e0",lty=1,lwd=3)
lines(Month,orangeADAS$ADASestimate,col="#e4630099",lty=1,lwd=3)
lines(Month,greenADAS$ADASestimate,col="#bfd03499",lty=1,lwd=3)
legend("topleft", bty = "n", legend = as.character(c("CN", "EMCI","LMCI","AD")),
       pch = 15, col = c("#7cafe2","#bfd03499","#e4630066","#f78a8a"), cex = 1,
horiz=F)
```

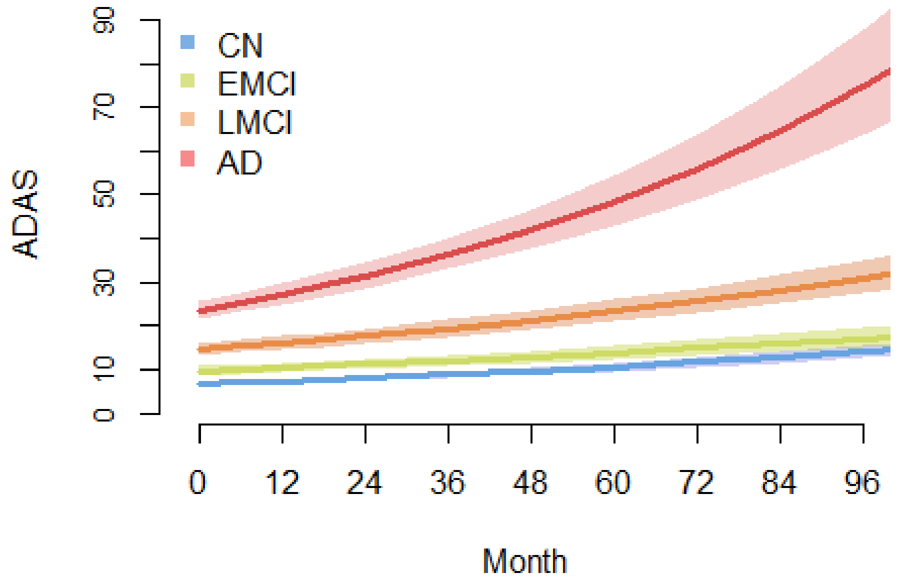


```
#dev.off()
```

9.15. *Graphing Education level*

9.15.1. *MMSE*

```
red<-newdat[newdat$APOE4==0 & newdat$Gender=="Female" & newdat$edu.cat=="1post"&
newdat$diagn=="3LMCI"& newdat$pain.medication=="No.Painrelief",]
orange<-newdat[newdat$APOE4==0 & newdat$Gender==“Female" &
newdat$edu.cat=="2tertiary"& newdat$diagn=="3LMCI"&
newdat$pain.medication=="No.Painrelief",]
blue<-newdat[newdat$APOE4==0 & newdat$Gender=="Female" &
newdat$edu.cat=="4early"& newdat$diagn=="3LMCI"&
newdat$pain.medication=="No.Painrelief",]
green<-newdat[newdat$APOE4==0 & newdat$Gender=="Female" &
newdat$edu.cat=="3mid"& newdat$diagn=="3LMCI"&
newdat$pain.medication=="No.Painrelief",]

plotdat<-rbind(red,blue,orange,green)


Month<-c(0,6,12,18,24,36,48,60,72,84,96,108,120)
yaxismin<-max((floor(min(plotdat$MMSEestimate))-1),5)

xmax<-min(min(max(blue$M[which(blue$UCI>5,
arr.ind=TRUE)]),120),min(max(red$M[which(red$UCI>5, arr.ind=TRUE)]),120))

#PDF("MMSEEducation.#PDF", width=16/2.54, heigh=12/2.54 )
plot(Month,red$MMSEestimate,pch=NA,ylab="MMSE", xlab="Month",ylim=c(yaxismin,
30),xlim=c(-2,xmax),axes = FALSE)
axis(1,seq(0,xmax,by=12))
axis(2,at=seq(30,yaxismin,by=-3), cex.axis=0.8)
```

```
polygon(c(rev(Month), Month), c(rev(red$LCI), red$UCI), col = rgb(0.8,0,0,0.2),
border = NA)
polygon(c(rev(Month), Month), c(rev(blue$LCI), blue$UCI), col =
rgb(0,0,0.8,0.2), border = NA)
polygon(c(rev(Month), Month), c(rev(orange$LCI), orange$UCI), col =
rgb(0.8,0.3,0,0.3), border = NA)
polygon(c(rev(Month), Month), c(rev(green$LCI), green$UCI), col =
rgb(191/255,208/255,52/255,0.4), border = NA)
lines(Month,red$MMSEestimate,col="#db4c4c",lty=1,lwd=3)
lines(Month,blue$MMSEestimate,col="#66a3e0",lty=1,lwd=3)
lines(Month,orange$MMSEestimate,col="#e4630099",lty=1,lwd=3)
lines(Month,green$MMSEestimate,col="#4b912299",lty=1,lwd=3)
legend("bottomleft", bty = "n", legend = as.character(c("Primary",
"Secondary","Tertiary","Post-graduate")),
       pch = 15, col = c("#7cafe2","#4b912299","#e4630066","#f78a8a"), cex = 1,
horiz=F)
```

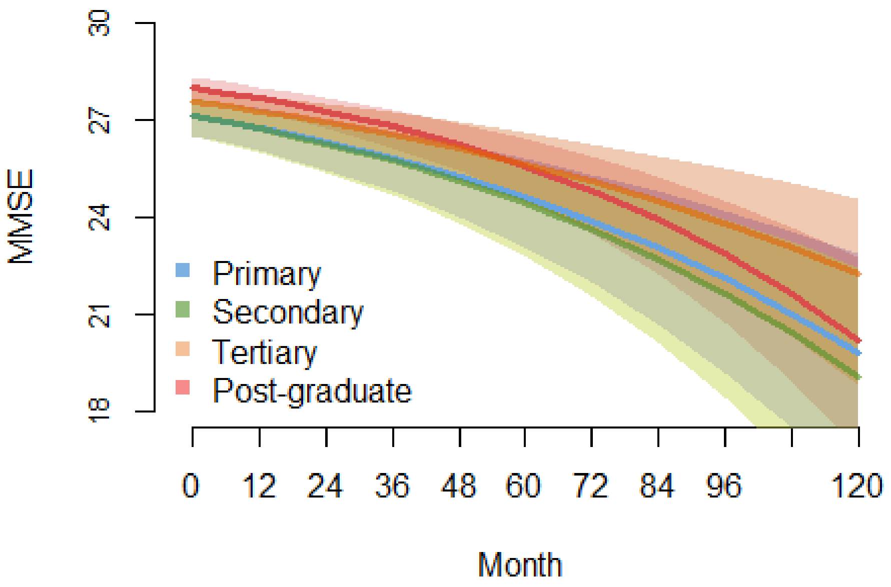


```
#dev.off()
```

9.15.2. ADAS

```
redADAS<-newdatADAS[newdatADAS$APOE4==0 & newdatADAS$headache==0 &
newdatADAS$Gender=="Female" & newdatADAS$edu.cat=="1post"&
newdatADAS$diagn=="3LMCI"& newdatADAS$pain.medication=="No.Painrelief",]

orangeADAS<-newdatADAS[newdatADAS$APOE4==0 & newdatADAS$headache==0 &
newdatADAS$Gender=="Female" & newdatADAS$edu.cat=="2tertiary"&
newdatADAS$diagn=="3LMCI"& newdatADAS$pain.medication=="No.Painrelief",]

blueADAS<-newdatADAS[newdatADAS$APOE4==0 & newdatADAS$headache==0 &
newdatADAS$Gender=="Female" & newdatADAS$edu.cat=="4early"&
newdatADAS$diagn=="3LMCI"& newdatADAS$pain.medication=="No.Painrelief",]
```

```
greenADAS<-newdatADAS[newdatADAS$APOE4==0 & newdatADAS$headache==0 &
newdatADAS$Gender=="Female" & newdatADAS$edu.cat=="3mid"&
newdatADAS$diagn=="3LMCI"& newdatADAS$pain.medication=="No.Painrelief",]



plotdat<-rbind(redADAS,orangeADAS, blueADAS, greenADAS)


Month<-c(0,6,12,18,24,36,48,60,72,84,96,108,120)
yaxismin<-max(min(c(plotdat$LCI)-5),0)
yaxismax<-min(max(redADAS$UCI,blueADAS$UCI)+5,90)
xmax<-min(min(max(blueADAS$M[which(blueADAS$UCI<70,
arr.ind=TRUE)]),120),min(max(redADAS$M[which(redADAS$UCI<70,
arr.ind=TRUE)]),120))

#PDF("ADASEducation.#PDF", width=16/2.54, heigh=12/2.54 )
plot(Month,redADAS$ADASestimate,pch=NA,ylab="ADAS",
xlab="Month",ylim=c(yaxismin, yaxismax),xlim=c(-2,xmax),axes = FALSE)
axis(1,seq(0,xmax,by=12))
axis(2,at=seq(0,yaxismax,by=10),cex.axis=0.8)
polygon(c(rev(Month), Month), c(rev(redADAS$LCI), redADAS$UCI), col =
rgb(0.8,0,0,0.2), border = NA)
polygon(c(rev(Month), Month), c(rev(blueADAS$LCI), blueADAS$UCI), col =
rgb(0,0,0.8,0.2), border = NA)
polygon(c(rev(Month), Month), c(rev(orangeADAS$LCI), orangeADAS$UCI), col =
rgb(0.8,0.3,0,0.3), border = NA)
polygon(c(rev(Month), Month), c(rev(greenADAS$LCI), greenADAS$UCI), col =
rgb(191/255,208/255,52/255,0.4), border = NA)
lines(Month,redADAS$ADASestimate,col="#db4c4c",lty=1,lwd=3)
lines(Month,blueADAS$ADASestimate,col="#66a3e0",lty=1,lwd=3)
lines(Month,orangeADAS$ADASestimate,col="#e4630099",lty=1,lwd=3)
lines(Month,greenADAS$ADASestimate,col="#4b912299",lty=1,lwd=3)
legend("topleft", bty = "n", legend = as.character(c("Primary",
"Secondary","Tertiary","Post-graduate")),
       pch = 15, col = c("#7cafe2","#4b912299","#e4630066","#f78a8a"), cex = 1,
horiz=F)
```

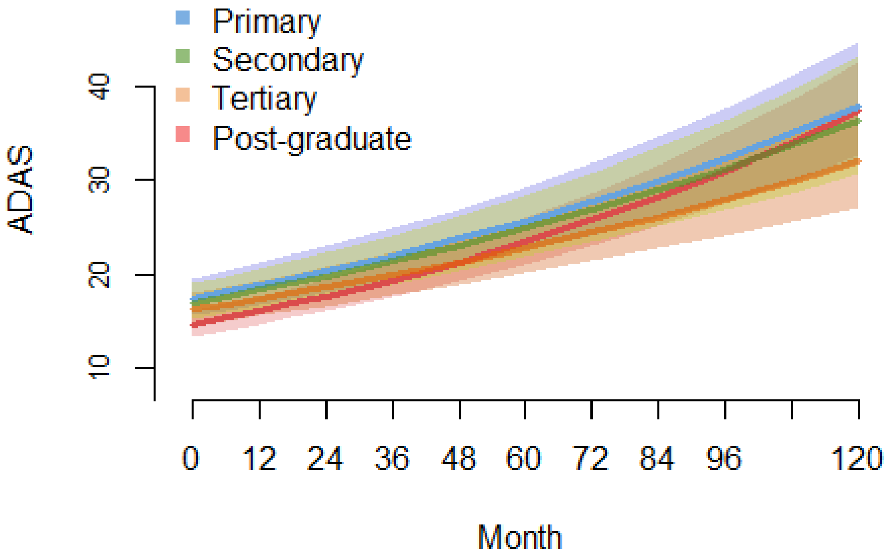


```
#dev.off()
```

10. Supplement references

1. Szekely CA, Breitner JC, Fitzpatrick AL, et al. NSAID use and dementia risk in the Cardiovascular Health Study: role of APOE and NSAID type. *Neurology* 2008; **70**(1): 17-24.

2. Gurka MJ, Edwards LJ, Muller KE, Kupper LL. Extending the Box–Cox transformation to the linear mixed model. *Journal of the Royal Statistical Society: Series A (Statistics in Society)* 2006; **169**(2): 273-88.

3. Bates D, Maechler M, Bolker B, Walker S. lme4: Linear mixed-effects models using Eigen and S4. *R package version* 2014; **1**(7): 1-23.

4. Bolker BM, Brooks ME, Clark CJ, et al. GLMMs in action: gene-by-environment interaction in total fruit production of wild populations of Arabidopsis thaliana Revised version, part 2. *See http://glmm wdfiles com/local-files/examples/Banta_2011_part1 pdf* 2011.

5. Hardin JW, Hilbe JM, Hilbe J. Generalized linear models and extensions: Stata press; 2007.

6. Fournier DA, Skaug HJ, Ancheta J, et al. AD Model Builder: usingautomatic differentiation for statistical inference of highly parameterized complex nonlinear models. *Optim Methods Softw* 2012; **27**: 233-49.

7. Skaug H, Fournier D, Nielsen A, Magnusson A, Bolker B. Generalized Linear Mixed Models using AD Model Builder. *R package version 075* 2013.

8. Hosmer JDW, Lemeshow S, Sturdivant RX. Model-Building Strategies and Methods for Logistic Regression. Applied Logistic Regression: John Wiley & Sons, Inc.; 2013: 89-151.

9. Byers AL, Allore H, Gill TM, Peduzzi PN. Application of negative binomial modeling for discrete outcomes: a case study in aging research. *J Clin Epidemiol* 2003; **56**(6): 559-64.

10. Goulet JL, Buta E, Bathulapalli H, Gueorguieva R, Brandt CA. Statistical Models for the Analysis of Zero-Inflated Pain Intensity Numeric Rating Scale Data. *The Journal of Pain* 2017; **18**(3): 340-8.

11. Nussmeier NA, Miao Y, Roach GW, et al. Predictive value of the National Institutes of Health Stroke Scale and the Mini-Mental State Examination for neurologic outcome after coronary artery bypass graft surgery. *The Journal of Thoracic and Cardiovascular Surgery* 2010; **139**(4): 901-12.
12. Musicco M, Palmer K, Salamone G, et al. Predictors of progression of cognitive decline in Alzheimer's disease: the role of vascular and sociodemographic factors. *Journal of Neurology* 2009; **256**(8): 1288-95.
13. Scarmeas N, Albert SM, Manly JJ, Stern Y. Education and rates of cognitive decline in incident Alzheimer's disease. *Journal of Neurology Neurosurgery and Psychiatry* 2006; **77**(3): 308-16.
14. Stern Y. What is cognitive reserve? Theory and research application of the reserve concept. *Journal of the International Neuropsychological Society* 2002; **8**(3): 448-60.

For Review Only
