# Supplementary material for: Anti-inflammatories in Alzheimer’s disease—potential therapy or spurious correlate?
Source: Brain Commun. 2020 Jul 24;2(2):fcaa109. doi: 10.1093/braincomms/fcaa109 (PMC7585697; doi:10.1093/braincomms/fcaa109)
Supplement: fcaa109_Supplementary_Data [file fcaa109_supplementary_data.zip › Supplementary_material.docx]

Supplement

Contents

[1. Cell culture experiment demonstrating differential effects of pain-relievers on IL-1β release 10](#_Toc41047074)

[2. Additional methods outline 11](#_Toc41047075)

[2.1. Brief outline of the methods 11](#_Toc41047076)

[2.2. Preliminary distribution analysis 12](#_Toc41047077)

[2.3. Model construction 13](#_Toc41047078)

[2.3.1. Selecting parameterisation method 13](#_Toc41047079)

[2.3.2. Building of initial main effect model 13](#_Toc41047080)

[2.3.3. Inclusion of biologically relevant two-way interaction terms 14](#_Toc41047081)

[2.4. Assumption check of residuals 14](#_Toc41047082)

[2.5. Building final model with different distribution families 14](#_Toc41047083)

[3. Additional results summary 15](#_Toc41047084)

[4. Packages used 15](#_Toc41047085)

[5. Data Cleaning 16](#_Toc41047086)

[5.2. Recurrent medicine data set 16](#_Toc41047087)

[5.2.1. Data Cleaning ensuring only the oral route of administration was considered in the analysis 16](#_Toc41047088)

[5.2.2. Identifying users of diclofenac 16](#_Toc41047089)

[5.2.3. Identifying users of aspirin 17](#_Toc41047090)

[5.2.4. Identifying users of paracetamol 17](#_Toc41047091)

[5.2.5. Identifying users of ibuprofen 18](#_Toc41047092)

[5.2.6. Identifying users of naproxen 18](#_Toc41047093)

[5.2.7. Identifying users of indomethacin 19](#_Toc41047094)

[5.2.8. Identifying users of celecoxib 19](#_Toc41047095)

[5.3. Combing drug users data frame with outcome variable data frame 20](#_Toc41047096)

[5.4. Searching for confounding preexisting condition 20](#_Toc41047097)

[5.4.1. Indentifying those with Diabetes 20](#_Toc41047098)

[5.4.2. Indentifying those with Cardiovascular disease 21](#_Toc41047099)

[5.4.3. Indentifying those with Arthritis 21](#_Toc41047100)

[5.4.4. Indentifying those with Migraine or headache 21](#_Toc41047101)

[5.4.5. Indentifying smokers 22](#_Toc41047102)

[5.5. Finaldrug table with disease summary 22](#_Toc41047103)

[5.6. Renaming columns and data clean 22](#_Toc41047104)

[5.7. Centering data 23](#_Toc41047105)

[5.8. Removing missing data from explanitory variables 23](#_Toc41047106)

[6. Constructing tables of participants at the begining of the study 23](#_Toc41047107)

[6.2. Tabling Explanatory variables by cogntive diagnosis 23](#_Toc41047108)

[6.3. Mean age by eduction level 25](#_Toc41047109)

[6.4. Explanatory variables information by pain-reliever use 26](#_Toc41047110)

[6.4.1. Mean MMSE scores for each pain-reliever 26](#_Toc41047111)

[6.4.2. Mean ADAS scores for each pain-reliever 28](#_Toc41047112)

[6.4.3. Education level for each pain-reliever 31](#_Toc41047113)

[6.4.4. Diagnosis prevalence for each pain-reliever 32](#_Toc41047114)

[6.4.4.1. Anaylsis of prevalence 35](#_Toc41047115)

[6.4.4.2. Significance of drug effects on prevalence 38](#_Toc41047116)

[6.4.5. APOE genotype proportions by pain reliever use 39](#_Toc41047117)

[6.4.6. Gender proportions by pain reliever use 39](#_Toc41047118)

[6.4.7. Mean age by pain reliever use 42](#_Toc41047119)

[6.4.8. Smoking status proportions by pain reliever use 44](#_Toc41047120)

[6.4.9. Arthritis proportions by pain reliever use 44](#_Toc41047121)

[6.4.10. Headache sufferer proportions by pain reliever use 47](#_Toc41047122)

[6.4.11. Cardiovascular disease proportions by pain reliever use 49](#_Toc41047123)

[6.4.12. Diabetes proportions by pain reliever use 51](#_Toc41047124)

[6.4.1. Tabling data completeness by NSAID 52](#_Toc41047125)

[7. Analysis of cognitive decline using the MMSE score 55](#_Toc41047126)

[7.2. Dependent variable check 55](#_Toc41047127)

[7.3. Generation of dependent variables appropriate for different distributions 55](#_Toc41047128)

[7.4. Transformation to obtain normal approximation 55](#_Toc41047129)

[7.5. Selecting non-gaussian model 60](#_Toc41047130)

[7.6. Build base negative binomial model and comparing the different parameterization methods. 99](#_Toc41047131)

[7.7. Observing the distribution of the residuals in for the initial model. 100](#_Toc41047132)

[7.8. Analysing all variables in isolation 116](#_Toc41047133)

[7.8.1. Main effect of gender 116](#_Toc41047134)

[7.8.2. Main effect of age 116](#_Toc41047135)

[7.8.3. Main effect of education level 117](#_Toc41047136)

[7.8.4. Main effect of diagnosis 118](#_Toc41047137)

[7.8.5. Main effect of APOE status 119](#_Toc41047138)

[7.8.6. Main effect of cardiovascular pathology 120](#_Toc41047139)

[7.8.7. Main effect of diabetes 120](#_Toc41047140)

[7.8.8. Main effect of smoking 121](#_Toc41047141)

[7.8.9. Main effect of headache 122](#_Toc41047142)

[7.8.10. Main effect of arthritis 123](#_Toc41047143)

[7.8.11. Main effect of diclofenac 123](#_Toc41047144)

[7.8.12. Main effect of paracetamol 124](#_Toc41047145)

[7.8.13. Main effect celecoxib 125](#_Toc41047146)

[7.8.14. Main effect of naproxen 126](#_Toc41047147)

[7.8.15. Main effect of aspirin 126](#_Toc41047148)

[7.8.16. Main effect of ibuprofen 127](#_Toc41047149)

[7.9. Building combined main effect model 128](#_Toc41047150)

[7.10. Dropping non-significant terms 129](#_Toc41047151)

[7.10.1. Narpoxen 129](#_Toc41047152)

[7.10.2. Diclofenac 130](#_Toc41047153)

[7.10.3. Headache 131](#_Toc41047154)

[7.10.4. Paracetamol 132](#_Toc41047155)

[7.10.5. Gender 133](#_Toc41047156)

[7.10.6. Arthritis 134](#_Toc41047157)

[7.10.7. Ibuprofen 135](#_Toc41047158)

[7.10.8. Aspirin 136](#_Toc41047159)

[7.11. Building combined main effect model 137](#_Toc41047160)

[7.12. Removing each explanitory variable in isolation 138](#_Toc41047161)

[7.12.1. Main effect of age at the start of the study 138](#_Toc41047162)

[7.12.2. Main effect of APOE4 genotype 138](#_Toc41047163)

[7.12.3. Main effect of education level 139](#_Toc41047164)

[7.12.4. Main effect of initial Alzhiemer’s diagnosis 139](#_Toc41047165)

[7.12.5. Main effect of initial Arthritis 139](#_Toc41047166)

[7.12.6. Main effect of initial Ibuprofen 139](#_Toc41047167)

[7.12.7. Main effect of initial Aspirin 140](#_Toc41047168)

[7.12.8. AIC summary of main effect models 140](#_Toc41047169)

[7.13. Investigating interaction terms 140](#_Toc41047170)

[7.13.1. The effects of diagnosis on cognitive decline progression 140](#_Toc41047171)

[7.13.2. The effects of smoking on cognitive decline progression 140](#_Toc41047172)

[7.13.3. The effects of arthritis on cognitive decline progression 141](#_Toc41047173)

[7.13.4. The effects of cardiovascular disease on cognitive decline progression 141](#_Toc41047174)

[7.13.5. The effect of headaches on cognitive decline progression 141](#_Toc41047175)

[7.13.6. The effect of diabetes on cognitive decline progression 142](#_Toc41047176)

[7.13.7. The effect of AGE on cognitive decline progression 143](#_Toc41047177)

[7.13.8. The effect of APOE status on cognitive decline progression 144](#_Toc41047178)

[7.13.9. The effect of education status on cognitive decline progression 145](#_Toc41047179)

[7.13.10. The effect of diabetes on cognitive decline progression 146](#_Toc41047180)

[7.13.11. The effect of Gender on cognitive decline progression 147](#_Toc41047181)

[7.13.12. The effect of aspirin on cognitive decline progression 148](#_Toc41047182)

[7.13.13. The effect of paracetamol on cognitive decline progression 149](#_Toc41047183)

[7.13.14. The effect of diclofenac on cognitive decline progression 150](#_Toc41047184)

[7.13.15. The effect of ibuprofen on cognitive decline progression 151](#_Toc41047185)

[7.13.16. The effect of naproxin on cognitive decline progression 152](#_Toc41047186)

[7.13.17. The effect of celecoxib on cognitive decline progression 153](#_Toc41047187)

[7.14. Combined interaction model 154](#_Toc41047188)

[7.14.1. Dropping non-significant terms 155](#_Toc41047189)

[7.15. Final full model and plots of the coeffecients 158](#_Toc41047190)

[7.15.1. Coeffecient plot 160](#_Toc41047191)

[7.15.2. Coeffecient plot of interaction terms 161](#_Toc41047192)

[7.16. Dropping terms of the model to evaluate the significance of each variable in the ful model. 162](#_Toc41047193)

[7.17. Evaluating the progession and main-effects of each pain medication 164](#_Toc41047194)

[7.18. Evaluating the progession and main-effects of each pain medication over short peroid with few deaths or missing data points 166](#_Toc41047195)

[7.19. Month as a factor 172](#_Toc41047196)

[7.20. Years education as a numerical variable 173](#_Toc41047197)

[7.21. APOE4 and NSAIDs 173](#_Toc41047198)

[7.22. looking at the distribution of the residuals in the final model, for each variable separately 179](#_Toc41047199)

[7.23. Checking for multicolinearity 190](#_Toc41047200)

[7.24. Checking other distributions 195](#_Toc41047201)

[8. Analysis of cognitive decline using the ADAS score 212](#_Toc41047202)

[8.2. Dependent variable check 212](#_Toc41047203)

[8.3. Generation of dependent variables appropriate for different distributions 212](#_Toc41047204)

[8.4. Transformation to obtain normal approximation 212](#_Toc41047205)

[8.5. Selecting non-gaussian model 217](#_Toc41047206)

[8.6. Build base negative binomial model and comparing the different parameterization methods. 256](#_Toc41047207)

[8.7. Observing the distribution of the residuals in for the initial model. 257](#_Toc41047208)

[8.8. Analysing all variables in isolation 273](#_Toc41047209)

[8.8.1. Main effect of gender 273](#_Toc41047210)

[8.8.2. Main effect of age 273](#_Toc41047211)

[8.8.3. Main effect of education level 274](#_Toc41047212)

[8.8.4. Main effect of diagnosis 275](#_Toc41047213)

[8.8.5. Main effect of APOE status 276](#_Toc41047214)

[8.8.6. Main effect of cardiovascular pathology 277](#_Toc41047215)

[8.8.7. Main effect of diabetes 277](#_Toc41047216)

[8.8.8. Main effect of smoking 278](#_Toc41047217)

[8.8.9. Main effect of headache 279](#_Toc41047218)

[8.8.10. Main effect of arthritis 280](#_Toc41047219)

[8.8.11. Main effect of diclofenac 280](#_Toc41047220)

[8.8.12. Main effect of paracetamol 281](#_Toc41047221)

[8.8.13. Main effect celecoxib 282](#_Toc41047222)

[8.8.14. Main effect of naproxen 283](#_Toc41047223)

[8.8.15. Main effect of aspirin 283](#_Toc41047224)

[8.8.16. Main effect of ibuprofen 284](#_Toc41047225)

[8.9. Building combined main effect model 285](#_Toc41047226)

[8.10. Dropping non-significant terms 286](#_Toc41047227)

[8.10.1. Narpoxen 286](#_Toc41047228)

[8.10.2. Paracetamol 287](#_Toc41047229)

[8.10.3. Arthritis 288](#_Toc41047230)

[8.10.4. Aspirin 289](#_Toc41047231)

[8.11. Building combined main effect model 290](#_Toc41047232)

[8.12. Removing each explanitory variable in isolation 291](#_Toc41047233)

[8.12.1. Main effect of age at the start of the study 291](#_Toc41047234)

[8.12.2. Main effect of APOE4 genotype 292](#_Toc41047235)

[8.12.3. Main effect of education level 292](#_Toc41047236)

[8.12.4. Main effect of initial Alzhiemer’s diagnosis 292](#_Toc41047237)

[8.12.5. Main effect of headaches 292](#_Toc41047238)

[8.12.6. Main effect of Gender 293](#_Toc41047239)

[8.12.7. Main effect of Ibuprofen 293](#_Toc41047240)

[8.12.8. Main effect of Diclofenac 293](#_Toc41047241)

[8.12.9. AIC summary of main effect models 294](#_Toc41047242)

[8.13. Investigating interaction terms 294](#_Toc41047243)

[8.13.1. The effects of diagnosis on cognitive decline progression 294](#_Toc41047244)

[8.13.2. The effects of smoking on cognitive decline progression 294](#_Toc41047245)

[8.13.3. The effects of arthritis on cognitive decline progression 294](#_Toc41047246)

[8.13.4. The effects of cardiovascular disease on cognitive decline progression 294](#_Toc41047247)

[8.13.5. The effect of headaches on cognitive decline progression 295](#_Toc41047248)

[8.13.6. The effect of diabetes on cognitive decline progression 296](#_Toc41047249)

[8.13.7. The effect of AGE on cognitive decline progression 297](#_Toc41047250)

[8.13.8. The effect of APOE status on cognitive decline progression 298](#_Toc41047251)

[8.13.9. The effect of education status on cognitive decline progression 299](#_Toc41047252)

[8.13.10. The effect of diabetes on cognitive decline progression 300](#_Toc41047253)

[8.13.11. The effect of Gender on cognitive decline progression 301](#_Toc41047254)

[8.13.12. The effect of aspirin on cognitive decline progression 302](#_Toc41047255)

[8.13.13. The effect of paracetamol on cognitive decline progression 303](#_Toc41047256)

[8.13.14. The effect of diclofenac on cognitive decline progression 304](#_Toc41047257)

[8.13.15. The effect of ibuprofen on cognitive decline progression 305](#_Toc41047258)

[8.13.16. The effect of naproxin on cognitive decline progression 306](#_Toc41047259)

[8.13.17. The effect of celecoxib on cognitive decline progression 307](#_Toc41047260)

[8.14. Combined interaction model 308](#_Toc41047261)

[8.14.1. Dropping non-significant interactions 309](#_Toc41047262)

[8.15. Final full model and plots of the coeffecients 311](#_Toc41047263)

[8.15.1. Coeffecient plot 312](#_Toc41047264)

[8.15.2. Coeffecient plot of interaction terms 313](#_Toc41047265)

[8.16. Dropping terms of the model to evaluate the significance of each variable in the ful model. 314](#_Toc41047266)

[8.17. Evaluating the progession and main-effects of each pain medication 318](#_Toc41047267)

[8.18. Evaluating the progession and main-effects of each pain medication with shortened timeframe 319](#_Toc41047268)

[8.19. APOE4 and NSAIDs 326](#_Toc41047269)

[8.19.1. General NSAID use is not associated with slower cognitive decline 332](#_Toc41047270)

[8.19.2. General NSAID use is associated with a fixed effect on cognitive performance 332](#_Toc41047271)

[8.20. Month as a factor 333](#_Toc41047272)

[8.21. Years education as a numerical variable 333](#_Toc41047273)

[8.22. looking at the distribution of the residuals in the final model, for each variable separately 333](#_Toc41047274)

[8.23. Checking for multicollinearity 343](#_Toc41047275)

[8.24. Checking other distributions 349](#_Toc41047276)

[9. Plotting predicted decline for ADAS and MMSE 367](#_Toc41047277)

[9.2. Generating model matrix for each pain medications MMSE 367](#_Toc41047278)

[9.2.1. Building the models for pain medications use 367](#_Toc41047279)

[9.2.2. Generating the model matrices for each pain medication 369](#_Toc41047280)

[9.2.2.1. Naproxen 369](#_Toc41047281)

[9.2.2.2. Aspirin 370](#_Toc41047282)

[9.2.2.3. Paracetamol 370](#_Toc41047283)

[9.2.2.4. Celecoxib 371](#_Toc41047284)

[9.2.2.5. Ibuprofen 371](#_Toc41047285)

[9.2.2.6. Diclofenac 372](#_Toc41047286)

[9.2.2.7. NSAID 372](#_Toc41047287)

[9.2.2.8. No.Painrelief 372](#_Toc41047288)

[9.2.3. Combining the model matrix 373](#_Toc41047289)

[9.3. Generating model matrix for each pain medications ADAS 373](#_Toc41047290)

[9.3.1. Building models 373](#_Toc41047291)

[9.3.2. Building the model matrix with 95% Laplance confidence interavals 376](#_Toc41047292)

[9.3.2.1. Naproxen 376](#_Toc41047293)

[9.3.2.2. Aspirin 376](#_Toc41047294)

[9.3.2.3. Paracetamol 377](#_Toc41047295)

[9.3.2.4. Celecoxib 377](#_Toc41047296)

[9.3.2.5. Ibuprofen 378](#_Toc41047297)

[9.3.2.6. Diclofenac 378](#_Toc41047298)

[9.3.2.7. NSAID 379](#_Toc41047299)

[9.3.2.8. No Pain Medication 379](#_Toc41047300)

[9.3.2.9. Combining the model matrix 379](#_Toc41047301)

[9.4. Cleaning model matrices 380](#_Toc41047302)

[9.5. Graphing LMCI with and without diclofenac use 380](#_Toc41047303)

[9.5.1. MMSE 380](#_Toc41047304)

[9.5.2. ADAS 381](#_Toc41047305)

[9.6. Graphing LMCI with and without Ibuprofen use 382](#_Toc41047306)

[9.6.1. MMSE 382](#_Toc41047307)

[9.6.2. ADAS 383](#_Toc41047308)

[9.7. Graphing LMCI with and without Naproxen use 384](#_Toc41047309)

[9.7.1. MMSE 384](#_Toc41047310)

[9.7.2. ADAS 385](#_Toc41047311)

[9.8. Graphing LMCI with and without Aspirin use 386](#_Toc41047312)

[9.8.1. MMSE 386](#_Toc41047313)

[9.8.2. ADAS 387](#_Toc41047314)

[9.9. Graphing LMCI with and without Celecoxib use 388](#_Toc41047315)

[9.9.1. MMSE 388](#_Toc41047316)

[9.9.2. ADAS 389](#_Toc41047317)

[9.10. Graphing LMCI with and without Paracetamol use 390](#_Toc41047318)

[9.10.1. MMSE 390](#_Toc41047319)

[9.10.2. ADAS 391](#_Toc41047320)

[9.11. Graphing LMCI with and without NSAID use 393](#_Toc41047321)

[9.11.1. MMSE 393](#_Toc41047322)

[9.11.2. ADAS 394](#_Toc41047323)

[9.12. Graphing LMCI with and without APOE genotypes 395](#_Toc41047324)

[9.12.1. MMSE 395](#_Toc41047325)

[9.12.2. ADAS 396](#_Toc41047326)

[9.13. Graphing LMCI between Genders 397](#_Toc41047327)

[9.13.1. MMSE 397](#_Toc41047328)

[9.13.2. ADAS 398](#_Toc41047329)

[9.14. Graphing Cogntive decline of diagnosis 399](#_Toc41047330)

[9.14.1. MMSE 399](#_Toc41047331)

[9.14.2. ADAS 401](#_Toc41047332)

[9.15. Graphing Education level 402](#_Toc41047333)

[9.15.1. MMSE 402](#_Toc41047334)

[9.15.2. ADAS 403](#_Toc41047335)

[10. Supplement references 405](#_Toc41047336)

# Cell culture experiment demonstrating differential effects of pain-relievers on IL-1β release

As a putative mechanism of action, a series of pain-relievers were screened for the additional anti-inflammatory mechanism of action of NLRP3 inhibition (Sup. Fig. 1.1). The NLRP3 receptor has been shown in animal models to be integral in the pathophysiology of Alzheimer’s disease. Here we show that diclofenac, the only pain-reliever associated with slowed cognitive decline, is also the only pain-reliever present in the ADNI dataset that inhibits NLRP3. Mefenamic acid is more potent NLRP3 inhibitor and has been found to be therapeutic in animal models of AD, however, was not used in the ADNI dataset due to its most common indication being dysmenorrhoea (period pain). This suggests that if diclofenac is validated in other epidemiological dataset and NLRP3 is found to be a key mechanism of action of diclofenac in preclinical animal models, then mefenamic acid should also be investigated as putative therapy due to greater potency at the NLRP3 receptor and reduced side effects.


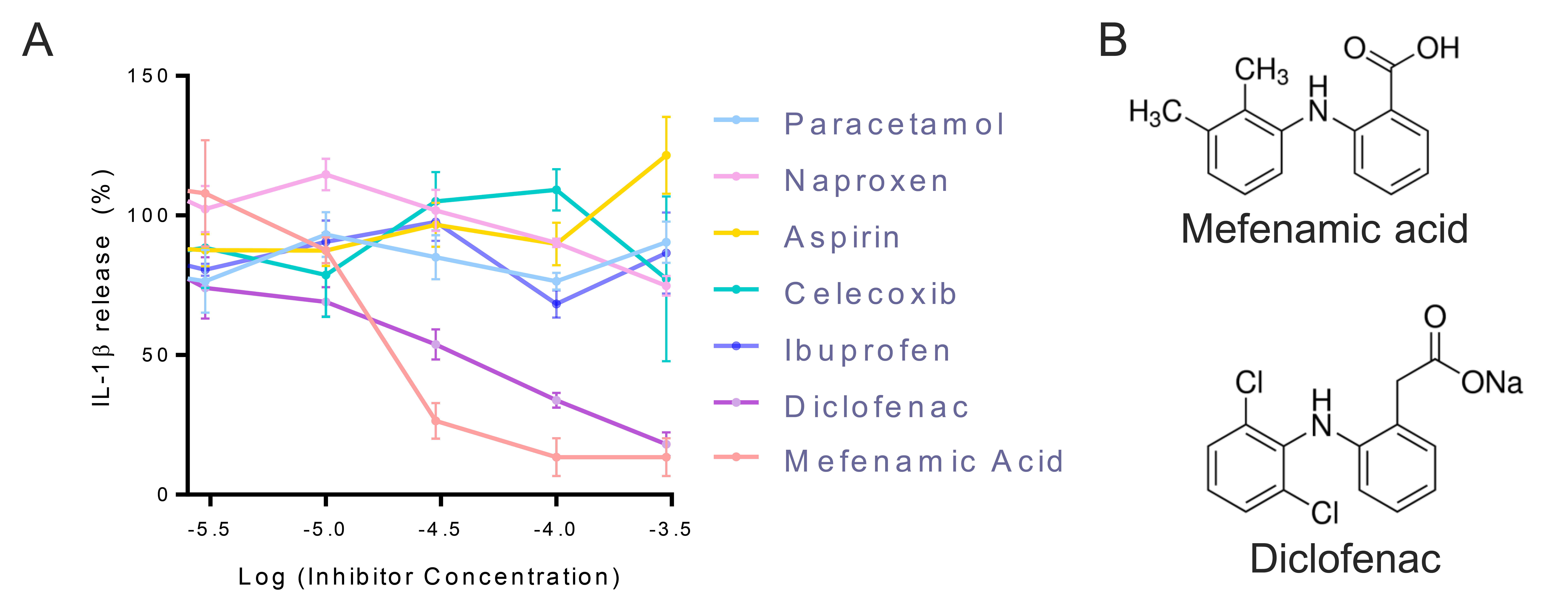


*Supplemental figure 1.1: Effects of common pain-relievers on IL-1β production from mouse bone marrow derived macrophages (BMDMs). A) Bone marrow was extracted from four 12 week old C57/B6j male mice, these were differentiated into primary BMDMs through growth in 70% DMEM (containing 10% FBS, PenStrep) supplemented with 30% L929 mouse fibroblast-conditioned media for 7-10 days. Before experiments, cells were seeded overnight at 1 x 10^6^ ml^-1^ in 96-well plates. The BMDMs were then treated with lipopolysaccharide (1µg ml^-1^) for 4 hours in complete DMEM, the media was then replaced with serum free media and the drugs added at a range of concentrations (vehicle DSMO). Following a 15 minute incubation, the cells were stimulated with the NLRP3 activator nigericin (10µg ml^-1^). After 1 hour the supernatant was removed an analysed for IL-1β concentration using ELISA assays (DY 401 DuoSet, R&D systems). B) The chemical structures of the two effective NLRP3 inhibiting pain-relievers.*

# Additional methods outline

## Brief outline of the methods

1. Application for access to the Alzheimer's Disease Neuroimaging Initiative (ADNI) data was applied for on 21^st^ October 2016, this was approved, and the datasets were download on the 3^rd^ of May 2018.
2. The datasets containing patient summaries, medical history, drug use and cognitive summaries were extensively cleaned dealing with inputting and spelling errors, and then merged by patient ID to create a final dataset for analysis. For the dependent variables of MMSE and ADAS score, a similar method of analysis was followed.
3. For each time point an initial assessment of data distribution was applied with negative binomial model selected as the optimal model distribution.
4. To select the parameterization method to initially construct the putative models, a simple main effect model with biologically relevant explanatory variables was applied using both available parameterization methods, these were then compared using the Akaike information criterion (AIC).
5. Model construction consisted of investigating the main effect of each explanatory variable in isolation utilizing the negative binomial GLMM (all models included the additional variable of time (month -“M”)). These were compared against the null model using a Chi-squared distribution of the log likelihood statistic to calculate the corresponding p value, with p<0.05 set at the threshold of significance.
6. All the significant main effects were then included in the model and then the coefficients were evaluated for continued input into the model in the presence of the other explanatory variables based of the Wald statistic approximation and log likelihood/chi squared method. Non-significant variables were dropped to create the final main effect model
7. Interaction terms were then investigated using the same method as the main effects with initial investigation to biologically relevant interaction terms added to the final main effect model in isolation, then all significant interaction terms were included and re-evaluated using the log likelihood/chi squared method and non-significant variables dropped.
8. Appropriate explanatory variables were then altered to numerical or factor variables and improvement in the model was evaluated as above.
9. The final model was selected base of Chi-square/log likelihood comparisons and AIC values.
10. The Pearson residuals were investigated ensuring no trends when plotted against any explanatory variables.
11. Covariance matrix was constructed, and no substantial multicollinearity was observed.
12. Each interaction term was then dropped and compared against the final model using Chi-square/log likelihood to establish the significance of each interaction term.
13. Using the same explanatory variable model design as the final model, other GLMM families, as well as negative binomial variants, were attempted and compared against the final negative binomial model using AIC values.
14. Finally, the biologically relevant three-way interaction of APOE4, NSAID and Time was investigated and found to not significantly improve the model as determined by the Chi-square/log likelihood method ^1^.

## Preliminary distribution analysis

Several possible distributions could be applied to the data, for simplicity each were tested within each time point. Normal distributions, with and without transformation, were first investigated. Previously it has been reported that a Box-Cox transformation can be utilized in multi-level linear models (MLM)(supplement 7.3-7.4 & 8.3-8.4) ^2^. To do this, total errors are generated by modelling the dependent variable using a simple linear model with the random effect (subject ID) as the explanatory variable. The Box-Cox method is then applied, and the data transformed using the predicted lambda. If normality and heteroscedasticity of the total errors is achieved, it has been demonstrated that the Pearson residuals generated by the MLM will also adhere to the assumptions of the model ^2^. Because of the upper and lower constrains of the cognitive scores, reduced variance at higher values of the MMSE score was observed and a stretching transformation was selected with a lambda of 4.79, the converse was true for the ADAS score and a lambda of 0.24 was applied (supplement 7.3-7.4 & 8.3-8.4). MLMs were then applied on the transformed scores using the LMER package with main biologically relevant explanatory variables included as well as the random effect of subject ID ^3^(supplement 4.2 & 5.2). Normality and heteroscedasticity was greatly improved in the models with the transformed dependent variables, however, the high numbers of cognitively normal subjects who received identical scores and the categorical nature of the scoring systems were still evident in the residual plots and so other modelling approaches were pursued (supplement 7.3-7.5 & 8.3-8.5). Similarly, GLMMs utilizing the Gamma distribution were relatively accurate models but were considered suboptimal because of the categorical nature of the scoring systems. While the exponential distribution is also a continuous distribution, and thus not appropriate for the score datasets, it was also a poor fit for both cognitive scores.

Models for discrete datasets were then investigated (supplement 7.5 & 8.5). Both MMSE and ADAS could be described as an aggregate of a series of Bernoulli trials and thus could be analysed as proportional data using logistic regression. Although this is an approximation as each trial (a point on the scale) is not identical because they correspond to different tasks in the assessments. Regardless, the numerous proportions of zero of data prevented these models from converging and so logistic regression was not appropriate (supplement 7.5 & 8.5). Poisson models were investigated as failures in the examinations could be counted and the rate of failures modelled (supplement 7.5 & 8.5). For this the MMSE score had to be converted into number of failures (30-MMSE), this has the advantage of now having the same directional relationship with disease severity as the ADAS score, with higher values correlating with worse cognitive performance and greater disease severity. The Poisson model did not perform optimally as there was substantial overdispersal and so negative binomial models were explored on the same converted variables and found to be a good model to continue the analyses with (supplement 7.5 & 8.5).

## Model construction

### Selecting parameterisation method

From the initial distribution analyses it was found that the variance was greater than the mean indicating that the data is over dispersed supporting the use of negative binomial models (supplement 7.6 & 8.6)^4,5^. There are several parameterisation methods which describe the relationship between the mean and the variance in the negative binomial model. The two most common (and the only methods available in the glmmadmb package ^6,7^) are the ‘nbinom1’ method, which assumes the variance = k × mean, and ‘nbinom2’ method, which assumes the variance = mean(1 + mean/k). The latter is most commonly used, particularly in count datasets, and is derived from a Gamma/Poisson model of a heterogeneous relationship between variance and mean ^5^. The former describes a simple proportional relationship between variance and mean and is less commonly used due to its inflexibility ^5^. Prior to the model construction, the parameterisation method must be selected. For this two-negative binomial GLMMs were constructed with biologically relevant explanatory variables utilizing each of the parameterisation methods. The method that provided the lowest AIC score and maximized the log likelihood was selected. For both ADAS and MMSE scores the ‘nbinom1’ parameterisation was selected (supplement 7.6 & 8.6). The parameterisation method was then re-evaluated at the end of the analyses using the final models and ‘nbinom1’ remain the optimal parameterisation method (supplement 7.6 & 8.6). This is interesting as it is the less flexible method and not commonly used on count datasets ^5^. Indicating these scores may have differences from traditional count distributions ^5^. Initial residual diagnostics were run on the biologically relevant hypothesized models to ensure future analyses using the negative binomial GLMM were prudent (supplement 7.6 & 8.6).

### Building of initial main effect model

Building of the GLMMs followed the protocol outlined by Hosmer et al. ^8^. To construct the negative binomial GLMM models the package glmmadmb was used ^6,7^. This package estimates parameters using the maximum likelihood method with the Laplace approximation to assess the marginal likelihood and provides coefficient summaries based on Wald approximations. As the aim of this study was to investigate disease progression, the baseline model was one with the explanatory variable of time (month) included as well as subject ID as a random effect (supplement 7.8 & 8.8). Then each biologically relevant explanatory variable was, in isolation, added to the baseline model and then compared against the baseline model using the Chi-squared/log likelihood method and AIC to investigate if the model was significantly improved by the inclusion of the variable (supplement 7.8 & 8.8). All significant variables were then included in the model and their continued input into the model in the presence of the other explanatory variables was evaluated using the Wald approximation statistics, Chi-squared/log likelihood method and AIC (supplement 7.10 & 8.10). Variables that ceased to significantly contribute to the model were dropped and the final main effect model of all significant variables was then constructed (supplement 7.11 & 8.11). This main effect model was then added to with biologically relevant interaction terms.

### Inclusion of biologically relevant two-way interaction terms

The inference of multivariable interaction terms becomes difficult, therefore, a common approach is to exclusively investigate biologically relevant two-way interaction terms ^8^. Similar to the main effect analyses, each two-way interaction term was added to the main effect model in isolation (supplement 7.13 & 8.13). Significant improvement in the model was assessed with the Chi-squared/log likelihood method and AIC values (supplement 7.13 & 8.13). A final model was constructed including all significant interactions and Wald approximation statistics were scrutinized for non-significant coefficients (supplement 7.14-7.16 & 8.14-8.16). Each interaction was then dropped in isolation from the model and compared against the full model including the variables that were dropped as main effect terms. The worsening of the model was assessed with the Chi-squared/log likelihood method and AIC values (supplement 7.14-7.16 & 8.14-8.16). Covariance matrices were constructed of the final model and no substantial multicollinearity was found between explanatory variables (supplement 7.22 & 8.22). The full model was then tested with time (month) treated as a factor and years in education treated as a numeric variable (rather than being grouped into education levels of early, middle, tertiary and post-graduate) (supplement 7.18-7.19 & 8.18-8.19). From this it was found that treating month as a factor introduced substantially more degrees of freedom into the model (supplement 7.18 & 8.18). This caused levels of the models to not have sufficient data to stabilize the model causing issues of model convergence, even when more simple models with fewer explanatory variables were attempted. Treating years in education as a numeric variable worsened the model; this is probably due to the lack of correlation between years and the (log) dependent variable (supplement 7.19 &8.19). Indicating the model benefited from the more flexible relationship allowed by treating education as factor variable. Finally, coefficient plots were generated with Laplace approximated confidence intervals to allow a quick visualisation of significant effects with direction (supplement 7.15 & 8.15).

## Assumption check of residuals

The Pearson residuals were extracted plotted against the explanatory variables grouped by ID. The ungrouped Pearson residuals were also plotted. No trends were observed for any explanatory variable indicating the appropriateness of the negative binomial models (supplement 7.21 & 8.21).

## Building final model with different distribution families

To confirm the appropriateness of the negative binomial model further models were run using the same explanatory variable model design. Normal MLMs were run as well as GLMMs utilising Poisson, binomial (logistic), zero inflated negative binomials and negative binomial with alternative parameterization methods (supplement 7.23 & 8.23). All available link functions were analysed for the Poisson and binomial (logistic) models (supplement 7.23 & 8.23). Centred explanatory variables were also applied for these models. All of these models were compared using AIC and the original negative binomial models were confirmed as the preferred models (supplement 7.23 & 8.23). Interestingly, the Wald approximate statistics were very similar using many of the models confirming the significances of the model terms (supplement 7.23 & 8.23). This provides greater support to the inferences of this study.

# Additional results summary

In the present study a range of models were attempted. The negative binomial model was not expected to be the selected model; however, every evaluation of the models found that negative binomial GLMM outperformed models based on other distributions. While the canonical form of a negative binomial model is the number of successes in a series of Bernoulli trials before a specified number of failures, this does not appear appropriate for the cognitive scores investigated here, however, Hardin *et al.* ^5^ noted “the negative binomial is rarely used in canonical form. Its primary use is to serve as an over dispersed Poisson regression model”. Interestingly, the Poisson models appeared to be the next best GLMMs as measured by AIC (supplement 7.23 & 8.23), however, over-dispersion was clearly evident (supplement 7.5 & 8.5). Hence, negative binomial models were pursued in the present study. This suggests that the MMSE and ADAS scores can be model as a  *count*  of the number of mistakes which occur during testing. This concept is support by previous research which has successfully used negative binomial GLMMs for similar score based systems and further analyses in the present study confirmed the use of the negative binomial GLM (supplement 7.23 & 8.23)^9-11^.

The results of the present study found that education had a capricious but significant relationship with cognitive decline. Here it was seen that those with the longest time spent in education had the fastest cognitive decline. This effect has previously been reported by Musicco *et al.* 2009 and Scarmeas *et al.* 2006. The proposed mechanism is cognitive reserve, those with greater intelligence have a delayed diagnosis as a greater loss of brain function is required before substantial symptoms set in. However, in the present study the slowest progressing group was those educated to the tertiary level, while those with secondary and early education levels declined at a rate in the middle. Therefore, this study neither supports nor refutes the cognitive reserve hypothesis ^12-14^. The effect seen possibly suggests hidden variables are influence cognitive decline, for example perhaps those with post-graduate level education represent an atypical group with a number of factors exterior to their education that could alter disease progression. Therefore, while it is useful to include the education variable in the model to reduce the level of unexplained variation, no clear inferences can be made without further research.

# Packages used

require(knitr)

require(lme4)

require(LMERConvenienceFunctions)

require(lmerTest)

require(MASS)

require(bbmle)

require(R2admb)

require(glmmADMB)

require(ggplot2)

require(coefplot)

require(car)

require(corrplot)

require(reshape2)

# Data Cleaning

## Recurrent medicine data set

The RECCMEDs data set contains all regularly taken medicines.

setwd("C:\\users\\mqbssjrn\\Dropbox\\Science\\Projects\\Epidemiology ADNI\\Analysis")
#setwd("C:\\Users\\jackr\\Dropbox\\Science\\Projects\\Epidemiology ADNI\\Analysis")
Meds<-read.csv("RECCMEDSnew.csv", header=T)

### Data Cleaning ensuring only the oral route of administration was considered in the analysis

There are multiple routes of administration of pain medications, however, topical skin application will fail to reach biological relevant concentrations in the plasma in the patient and will only have action at the site of application. Furthermore, intravenous administration will unlikely be taken at a frequency that will be relevant for the slow and progressive nature of AD. Therefore, oral administration was selected as the administration route of interest and a data frame containing only drugs administered orally was created. Then patient IDs were extracted from this data frame for each of the pain medications of interest.

Route<-c("po","PO","P.O.","P.O","p.o","p.o.","6","oral","Oral","ORAL","orally")
Meds$route<-ifelse(Meds$CMROUTE %in% Route,1,0)
Meds<-Meds[Meds$route==1,]

### Identifying users of diclofenac

diclofenac<-c("Diclofemac Sodium","diclofen sodium","diclofenac","Diclofenac","diclofenac NA","DICLOFENAC NA","Diclofenac SOD","diclofenac sodium","Diclofenac sodium","Diclofenac Sodium Solution 1.5%","Diclofenac 0.01%","Diclofenac Sodium", "diclofen", "Diclofen","voltaren", "Voltaren", "VOLTAREN","Volteren")
Meds$diclofenac[Meds$CMMED %in% diclofenac | Meds$CMMEDO %in% diclofenac] <- 1
dicloIDS<-Meds[Meds$diclofenac==1,3]
dicloIDS<-sort(unique(dicloIDS))


drugrow<-c()
for(i in 1:27){
 drugrow<-append(drugrow,grep(diclofenac[i],Meds$CMMED ))
 drugrow<-append(drugrow,grep(diclofenac[i],Meds$CMMEDO ))
}

dicloIDS<-Meds[drugrow,3]
dicloIDS<-sort(unique(dicloIDS))


Meds$diclofenac<-c()
Meds$diclofenac<-(Meds$RID %in% dicloIDS)*Meds$CMCONT
dicloIDS<-unique(Meds[Meds$diclofenac==1,3])
length(dicloIDS)

[1] 46

### Identifying users of aspirin

aspirin<-c("Aspirin","aspirin","ASPIRIN","Baby aspirin","baby aspirin","Apirin","asprin","Asprin","ASA (baby Asprin)","Aspirn","Aspirin (e.c.)","Aspirin bayer","Aspirin enteric coated k.p.","apirin","apirin","Baby Asprin","aspririn","ASA-baby asprin","Baby asprin","Enteric coated aspirin","Baby Aspirin","Aspirin (e.c.)","Aspirin buffered325","Aspirin, ibuprofen", "naproxen","Aspirn","aspirine")
Meds$aspirin<-numeric(length(Meds$ID))
Meds$aspirin[Meds$CMMED %in% aspirin | Meds$CMMEDO %in% aspirin] <- 1
AspIDS<-Meds[Meds$aspirin==1,3]
AspIDS<-unique(AspIDS)


drugrow<-c()
for(i in 1:27){
 drugrow<-append(drugrow,grep(aspirin[i],Meds$CMMED ))
 drugrow<-append(drugrow,grep(aspirin[i],Meds$CMMEDO ))
}

AspIDS<-Meds[drugrow,3]
AspIDS<-sort(unique(AspIDS))


Meds$aspir<-c()
Meds$aspir<-(Meds$RID %in% AspIDS)*Meds$CMCONT
AspIDS<-unique(Meds[Meds$aspir==1,3])
length(AspIDS)

[1] 1253

### Identifying users of paracetamol

parac<-c("paracetamol","panadol","Paracetamol","Panadol","acetaminophen","Acetaminophen","Acetamin","acetamin", "acetomenophen","Acetomenophen","ACETOMINOPHEN","acetominophen","Acetominophen","ACETAMINOPHEN","tylenol","Tylenol","TYLENOL","Tylox","Tyenol", "Vicodin","vicodin","Vicodan","Vicodan","VICODIN")
Meds$parac<-numeric(length(Meds$ID))
Meds$parac[Meds$CMMED %in% parac | Meds$CMMEDO %in% parac] <- 1
paracIDS<-Meds[Meds$parac==1,3]
paracIDS<-unique(paracIDS)

drugrow<-c()
for(i in 1:24){
 drugrow<-append(drugrow,grep(parac[i],Meds$CMMED ))
 drugrow<-append(drugrow,grep(parac[i],Meds$CMMEDO ))
}

paracIDS<-Meds[drugrow,3]
paracIDS<-sort(unique(paracIDS))


Meds$parac<-c()
Meds$parac<-(Meds$RID %in% paracIDS)*Meds$CMCONT
paracIDS<-unique(Meds[Meds$parac==1,3])
length(paracIDS)

[1] 503

### Identifying users of ibuprofen

Ibu<-c("Ibuprofen", "ibuprofen", "IBUPROFEN","Advil", "advil","ADVIL", "Motrin","motrin","MOTRIN")
Meds$Ibu<-numeric(length(Meds$ID))
Meds$Ibu[Meds$CMMED %in% Ibu | Meds$CMMEDO %in% Ibu] <- 1
IbuIDS<-Meds[Meds$Ibu==1,3]
IbuIDS<-unique(IbuIDS)

drugrow<-c()
for(i in 1:9){
 drugrow<-append(drugrow,grep(Ibu[i],Meds$CMMED ))
 drugrow<-append(drugrow,grep(Ibu[i],Meds$CMMEDO ))
}

IbuIDS<-Meds[drugrow,3]
IbuIDS<-sort(unique(IbuIDS))


Meds$Ibu<-c()
Meds$Ibu<-(Meds$RID %in% IbuIDS)*Meds$CMCONT
IbuIDS<-unique(Meds[Meds$Ibu==1,3])
length(IbuIDS)

[1] 324

### Identifying users of naproxen

naprox<-c("Naproxen", "naproxen", "NAPROXEN","Aleve", "aleve","ALEVE", "Accord", "Anaprox", "Antalgin", "Apranax", "Feminax Ultra", "Flanax", "Inza", "Maxidol", "Midol Extended Relief", "Nalgesin", "Naposin", "Naprelan", "Naprogesic", "Naprosyn", "Narocin", "Pronaxen", "Proxen", "Soproxen", "Synflex", "MotriMax", "Xenobid","naprox","Naprox")
Meds$naprox<-numeric(length(Meds$ID))
Meds$naprox[Meds$CMMED %in% naprox | Meds$CMMEDO %in% naprox] <- 1
naproxIDS<-Meds[Meds$naprox==1,3]
naproxIDS<-unique(naproxIDS)

drugrow<-c()
for(i in 1:29){
 drugrow<-append(drugrow,grep(naprox[i],Meds$CMMED ))
 drugrow<-append(drugrow,grep(naprox[i],Meds$CMMEDO ))
}

naproxIDS<-Meds[drugrow,3]
naproxIDS<-sort(unique(naproxIDS))


Meds$naprox<-c()
Meds$naprox<-(Meds$RID %in% naproxIDS)*Meds$CMCONT
naproxIDS<-unique(Meds[Meds$naprox==1,3])
length(naproxIDS)

[1] 241

### Identifying users of indomethacin

Not enough subjects to include in future analysis.

indo<-c("Indomethacin", "indomethacin", "INDOMETHACIN","Indometacin", "indometacin","INDOMETACIN", "Indocin", "indocin", "INDOCIN", "Tiverbex", "tiverbex")
Meds$indo<-numeric(length(Meds$ID))
Meds$indo[Meds$CMMED %in% indo | Meds$CMMEDO %in% indo] <- 1
indoIDS<-Meds[Meds$indo==1,3]
indoIDS<-unique(indoIDS)

drugrow<-c()
for(i in 1:11){
 drugrow<-append(drugrow,grep(indo[i],Meds$CMMED ))
 drugrow<-append(drugrow,grep(indo[i],Meds$CMMEDO ))
}

indoIDS<-Meds[drugrow,3]
indoIDS<-sort(unique(indoIDS))


Meds$indo<-c()
Meds$indo<-(Meds$RID %in% indoIDS)*Meds$CMCONT
indoIDS<-unique(Meds[Meds$indo==1,3])
length(indoIDS)

[1] 16

### Identifying users of celecoxib

celex<-c("Celecoxib", "celecoxib", "CELECOX","Celebrex", "celebrex","CELEBREX","CELEBREX")

Meds$celex<-numeric(length(Meds$ID))
Meds$celex[Meds$CMMED %in% celex | Meds$CMMEDO %in% celex] <- 1
celexIDS<-Meds[Meds$celex==1,3]
celexIDS<-unique(celexIDS)

drugrow<-c()
for(i in 1:7){
 drugrow<-append(drugrow,grep(celex[i],Meds$CMMED ))
 drugrow<-append(drugrow,grep(celex[i],Meds$CMMEDO ))
}

celexIDS<-Meds[drugrow,3]
celexIDS<-sort(unique(celexIDS))


Meds$celex<-c()
Meds$celex<-(Meds$RID %in% celexIDS)*Meds$CMCONT
celexIDS<-unique(Meds[Meds$celex==1,3])
length(celexIDS)

[1] 87

## Combing drug users data frame with outcome variable data frame

The ADNIMERGE dataset is a complied data set of key variables from the ADNI study. Information about pain medication use was merged with the ADNIMERGE dataset.

setwd("C:\\users\\mqbssjrn\\Dropbox\\Science\\Projects\\Epidemiology ADNI\\Analysis")
#setwd("C:\\Users\\jackr\\Dropbox\\Science\\Projects\\Epidemiology ADNI\\Analysis")
summary<-read.csv("ADNIMERGEnew.csv", header=T)
Finaldrug<-summary[,which(names(summary) %in% c("RID","DX_bl","AGE","PTGENDER","PTEDUCAT","APOE4","CDRSB","ADAS11","ADAS13","MMSE","Hippocampus","WholeBrain","CDRSB_bl","ADAS11_bl","ADAS13_bl","MMSE_bl","M"))]
Finaldrug$aspirin<-(Finaldrug$RID %in% AspIDS)*1
Finaldrug$parac<-(Finaldrug$RID %in% paracIDS)*1
Finaldrug$diclo<-(Finaldrug$RID %in% dicloIDS)*1
Finaldrug$Ibu<-(Finaldrug$RID %in% IbuIDS)*1
Finaldrug$naprox<-(Finaldrug$RID %in% naproxIDS)*1
Finaldrug$celex<-(Finaldrug$RID %in% celexIDS)*1

length(unique(Finaldrug$RID[Finaldrug$diclo==1]))

[1] 35

## Searching for confounding preexisting condition

The RECMHIST is a dataset of pre-existing condition. Diabetes and vascular diseases have been linked to changes in AD incidence and progression. Additionally, arthritis and headaches were the leading indications of pain medication use. Therefore, patient IDs where extracted and merged with the cognitive scoring dataset to include these variables in the analyses.

### Indentifying those with Diabetes

medhist<-read.csv("RECMHISTnew.csv", header=T)
diseases<-data.frame(table(medhist$MHDESC))
diabetes<-c("Disbetes", "diabetes","Diabetes", "DIABETES", "diabetic","Diabetic","DIABETIC","diabetes", "diabetic","DIABETIC","DIABETES")

diseaserow<-c()
for(i in 1:11){
 diseaserow<-append(diseaserow,grep(diabetes[i],medhist$MHDESC ))
 diseaserow<-append(diseaserow,grep(diabetes[i],medhist$MHDESC ))
}

diabetesIDS<-medhist[diseaserow,3]
diabetesIDS<-sort(unique(diabetesIDS))


medhist$diabetes<-c()
medhist$diabetes<-(medhist$RID %in% diabetesIDS)*1
diabetesIDS<-unique(medhist[medhist$diabetes==1,3])
length(diabetesIDS)

[1] 234

### Indentifying those with Cardiovascular disease

cardiovasc<-c("cholest", "pressure","hypertension", "Cholest", "Pressure","Hypertension","stroke","Stroke","Heart attack","heart attack","Hyoertension", "blood pressure", "cholesterol", "Cholesterol", "PRESSURE", "hypercholesterolemia","Hypercholesterolemia","pressure","CHOLESTEROL")

diseaserow<-c()
for(i in 1:19){
 diseaserow<-append(diseaserow,grep(cardiovasc[i],medhist$MHDESC ))
 diseaserow<-append(diseaserow,grep(cardiovasc[i],medhist$MHDESC ))
}

cardiovascIDS<-medhist[diseaserow,3]
cardiovascIDS<-sort(unique(cardiovascIDS))


medhist$cardiovasc<-c()
medhist$cardiovasc<-(medhist$RID %in% cardiovascIDS)*1
cardiovascIDS<-unique(medhist[medhist$cardiovasc==1,3])
length(cardiovascIDS)

[1] 1441

### Indentifying those with Arthritis

arthrit<-c("Arthritis", "arthritis","ARTHRITIS", "arthritic", "Arthritic","Arthitis","osteoarthritis","Osteoarthritis", "OSTEOARTHRITIS","ARTHRITIC","Arthritric")

diseaserow<-c()
for(i in 1:11){
 diseaserow<-append(diseaserow,grep(arthrit[i],medhist$MHDESC ))
}

arthritIDS<-medhist[diseaserow,3]
arthritIDS<-sort(unique(arthritIDS))


medhist$arthrit<-c()
medhist$arthrit<-(medhist$RID %in% arthritIDS)*1
arthritIDS<-unique(medhist[medhist$arthrit==1,3])
length(arthritIDS)

[1] 896

### Indentifying those with Migraine or headache

headache<-c("headache", "Headache","HEADACHE", "Migraine", "migraine","MIGRAINE")


diseaserow<-c()
for(i in 1:11){
 diseaserow<-append(diseaserow,grep(headache[i],medhist$MHDESC ))
}

headacheIDS<-medhist[diseaserow,3]
headacheIDS<-sort(unique(headacheIDS))


medhist$headache<-c()
medhist$headache<-(medhist$RID %in% headacheIDS)*1
headacheIDS<-unique(medhist[medhist$headache==1,3])
length(headacheIDS)

[1] 205

### Indentifying smokers

smoke<-c("Smoker", "smoker","SMOKER", "SMOKE", "Smoke","smoke","Smoking","smoking","SMOKING")

diseaserow<-c()
for(i in 1:9){
 diseaserow<-append(diseaserow,grep(smoke[i],medhist$MHDESC ))
}

smokeIDS<-medhist[diseaserow,3]
smokeIDS<-sort(unique(smokeIDS))


medhist$smoke<-c()
medhist$smoke<-(medhist$RID %in% smokeIDS)*1
smokeIDS<-unique(medhist[medhist$smoke==1,3])
length(smokeIDS)

[1] 579

## Finaldrug table with disease summary

Finaldrug$diab<-(Finaldrug$RID %in% diabetesIDS)*1
Finaldrug$arthrit<-(Finaldrug$RID %in% arthritIDS)*1
Finaldrug$vasc<-(Finaldrug$RID %in% cardiovascIDS)*1
Finaldrug$smoke<-(Finaldrug$RID %in% smokeIDS)*1
Finaldrug$headache<-(Finaldrug$RID %in% headacheIDS)*1

## Renaming columns and data clean

names(Finaldrug)[1]<-"ID"
names(Finaldrug)[2]<-"diagn"
names(Finaldrug)[4]<-"Gender"
names(Finaldrug)[5]<-"Yrs.edu"
names(Finaldrug)[11]<-"Hippo"
names(Finaldrug)[12]<-"Brain"
names(Finaldrug)[13]<-"CDRSB.bl"
names(Finaldrug)[14]<-"ADAS11.bl"
names(Finaldrug)[15]<-"ADAS13.bl"
names(Finaldrug)[16]<-"MMSE.bl"
names(Finaldrug)[17]<-"M"
Finaldrug$diagn<-as.character(Finaldrug$diagn)
Finaldrug$diagn[Finaldrug$diagn=="CN"]<-"1CN"
Finaldrug$diagn[Finaldrug$diagn=="EMCI"]<-"2EMCI"
Finaldrug$diagn[Finaldrug$diagn=="LMCI"]<-"3LMCI"
Finaldrug$diagn[Finaldrug$diagn=="AD"]<-"4AD"
Finaldrug<-Finaldrug[Finaldrug$diagn!="SMC",]
Finaldrug$diagn<-as.factor(Finaldrug$diagn)

## Centering data

Future analyses required the cantering of data to aid convergence of the model by adjusting the explanatory variables to comparable values.

Finaldrug$Yrs.ed.Z<-(Finaldrug$Yrs.edu-mean(Finaldrug$Yrs.edu))/sd(Finaldrug$Yrs.edu)
Finaldrug$AGE.Z<-(Finaldrug$AGE-mean(Finaldrug$AGE))/sd(Finaldrug$AGE)
Finaldrug$AGEraw<-Finaldrug$AGE
Finaldrug$AGE<-Finaldrug$AGEraw-mean(Finaldrug$AGEraw)

Finaldrug$M.Z<-(Finaldrug$M-mean(Finaldrug$M))/sd(Finaldrug$M)
Finaldrug$x<-Finaldrug$diclo+Finaldrug$naprox+Finaldrug$celex+Finaldrug$parac+Finaldrug$aspirin+Finaldrug$Ibu
Finaldrug$Painrelief<-1*(Finaldrug$x>0)
Finaldrug$No.Painrelief<-(Finaldrug$Painrelief-1)^2
Finaldrug$x<-Finaldrug$diclo+Finaldrug$naprox+Finaldrug$celex+Finaldrug$aspirin+Finaldrug$Ibu
Finaldrug$NSAID<-1*(Finaldrug$x>0)

write.csv(Finaldrug, file="Finaldrug.csv")

## Removing missing data from explanitory variables

setwd("C:\\users\\mqbssjrn\\Dropbox\\Science\\Projects\\Epidemiology ADNI\\Analysis")
#setwd("C:\\Users\\jackr\\Dropbox\\Science\\Projects\\Epidemiology ADNI\\Analysis")
Fulldata<-read.csv("Finaldrug.csv", header=T)

data<-Fulldata[!is.na(Fulldata$APOE4),]
data<-data[!is.na(data$Gender),]
data<-data[!is.na(data$AGE),]
data<-data[!is.na(data$Yrs.edu),]
data<-data[!is.na(data$diagn),]
data$APOE4<-as.factor(data$APOE4)
data$ID<-as.factor(data$ID)
data$edu.cat[data$Yrs.edu<13]<-"4early"
data$edu.cat[data$Yrs.edu>=13 & data$Yrs.edu<16]<-"3mid"
data$edu.cat[data$Yrs.edu>=16& data$Yrs.edu<18]<-"2tertiary"
data$edu.cat[data$Yrs.edu>=18]<-"1post"
fulldata<-data
write.csv(data, file="CleanedFinalData.csv")

# Constructing tables of participants at the begining of the study

## Tabling Explanatory variables by cogntive diagnosis

initialdata<-fulldata[fulldata$M==0,]

Particpants<-length(initialdata$ID)
Particpants

[1] 1619

Gender<-table(initialdata$diagn, by=initialdata$Gender)

Mean.Age<-round(c(0,mean(initialdata$AGE[initialdata$diagn=="1CN"]),mean(initialdata$AGE[initialdata$diagn=="2EMCI"]),mean(initialdata$AGE[initialdata$diagn=="3LMCI"]),mean(initialdata$AGE[initialdata$diagn=="4AD"])),2)
SD.AGE<-round(c(0,sd(initialdata$AGE[initialdata$diagn=="1CN"]),sd(initialdata$AGE[initialdata$diagn=="2EMCI"]),sd(initialdata$AGE[initialdata$diagn=="3LMCI"]),sd(initialdata$AGE[initialdata$diagn=="4AD"])),2)
Mean.Age.total<-round(mean(initialdata$AGE,2))
SD.AGE.total<-round(sd(initialdata$AGE),2)

Aspirin<-table(initialdata$diagn,by=initialdata$aspirin)[,2]
Celecoxib<-table(initialdata$diagn,by=initialdata$celex)[,2]
Diclofenac<-table(initialdata$diagn,by=initialdata$diclo)[,2]
Ibuprofen<-table(initialdata$diagn,by=initialdata$Ibu)[,2]
Naproxen<-table(initialdata$diagn,by=initialdata$naprox)[,2]
Paracetamol<-table(initialdata$diagn,by=initialdata$parac)[,2]
No.Painrelief<-table(initialdata$diagn,by=initialdata$Painrelief)[,1]

Cardiovascular.Pathology<-table(initialdata$diagn,by=initialdata$vasc)[,2]
Diabetes<-table(initialdata$diagn,by=initialdata$diab)[,2]
Smoker<-table(initialdata$diagn,by=initialdata$smoke)[,2]
Headache<-table(initialdata$diagn,by=initialdata$headache)[,2]
Arthritis<-table(initialdata$diagn,by=initialdata$arthrit)[,2]

APOE4.1<-table(initialdata$diagn,by=initialdata$APOE4)[,2]
APOE4.2<-table(initialdata$diagn,by=initialdata$APOE4)[,3]
Early.Education<-table(initialdata$diagn,by=initialdata$edu.cat)[,4]
Mid.Education<-table(initialdata$diagn,by=initialdata$edu.cat)[,3]
Tertiary.Education<-table(initialdata$diagn,by=initialdata$edu.cat)[,2]
Postgraduate.Education<-table(initialdata$diagn,by=initialdata$edu.cat)[,1]


Mean.MMSE<-round(c(0,mean(initialdata$MMSE[initialdata$diagn=="1CN"],na.rm=TRUE),mean(initialdata$MMSE[initialdata$diagn=="2EMCI"],na.rm=TRUE),mean(initialdata$MMSE[initialdata$diagn=="3LMCI"],na.rm=TRUE),mean(initialdata$MMSE[initialdata$diagn=="4AD"],na.rm=TRUE)),2)
SD.MMSE<-round(c(0,sd(initialdata$MMSE[initialdata$diagn=="1CN"],na.rm=TRUE),sd(initialdata$MMSE[initialdata$diagn=="2EMCI"],na.rm=TRUE),sd(initialdata$MMSE[initialdata$diagn=="3LMCI"],na.rm=TRUE),sd(initialdata$MMSE[initialdata$diagn=="4AD"],na.rm=TRUE)),2)

Mean.MMSE.total<-round(mean(initialdata$MMSE,2))
SD.MMSE.total<-round(sd(initialdata$MMSE),2)

Mean.ADAS13<-round(c(0,mean(initialdata$ADAS13[initialdata$diagn=="1CN"],na.rm=TRUE),mean(initialdata$ADAS13[initialdata$diagn=="2EMCI"],na.rm=TRUE),mean(initialdata$ADAS13[initialdata$diagn=="3LMCI"],na.rm=TRUE),mean(initialdata$ADAS13[initialdata$diagn=="4AD"],na.rm=TRUE)),2)

SD.ADAS13<-round(c(0,sd(initialdata$ADAS13[initialdata$diagn=="1CN"],na.rm=TRUE),sd(initialdata$ADAS13[initialdata$diagn=="2EMCI"],na.rm=TRUE),sd(initialdata$ADAS13[initialdata$diagn=="3LMCI"],na.rm=TRUE),sd(initialdata$ADAS13[initialdata$diagn=="4AD"],na.rm=TRUE)),2)

Mean.ADAS.total<-round(mean(initialdata$ADAS13,na.rm=TRUE),2)
SD.ADAS.total<-round(sd(initialdata$ADAS13,na.rm=TRUE),2)

table<-t(cbind(Gender, Mean.Age, SD.AGE,Aspirin,Celecoxib,Diclofenac,Ibuprofen,Naproxen,Paracetamol,No.Painrelief,Cardiovascular.Pathology,Diabetes,Smoker, Arthritis, Headache, APOE4.1,APOE4.2,Early.Education,Mid.Education,Tertiary.Education,Postgraduate.Education, Mean.MMSE,SD.MMSE,Mean.ADAS13,SD.ADAS13))[,2:5]
table

1CN 2EMCI 3LMCI 4AD
Female 206.00 136.00 219.00 149.00
Male 209.00 170.00 345.00 185.00
Mean.Age 1.00 -2.54 0.23 1.15
SD.AGE 5.73 7.40 7.50 7.82
Aspirin 247.00 174.00 302.00 138.00
Celecoxib 19.00 13.00 25.00 7.00
Diclofenac 14.00 5.00 8.00 3.00
Ibuprofen 79.00 55.00 92.00 29.00
Naproxen 52.00 45.00 68.00 21.00
Paracetamol 119.00 75.00 140.00 66.00
No.Painrelief 99.00 67.00 165.00 141.00
Cardiovascular.Pathology 271.00 182.00 348.00 195.00
Diabetes 37.00 37.00 48.00 28.00
Smoker 109.00 69.00 150.00 84.00
Arthritis 183.00 126.00 208.00 109.00
Headache 36.00 30.00 49.00 23.00
APOE4.1 103.00 110.00 234.00 157.00
APOE4.2 11.00 21.00 73.00 65.00
Early.Education 44.00 45.00 102.00 82.00
Mid.Education 89.00 74.00 88.00 68.00
Tertiary.Education 120.00 76.00 167.00 100.00
Postgraduate.Education 162.00 111.00 207.00 84.00
Mean.MMSE 29.07 28.33 27.18 23.18
SD.MMSE 1.12 1.57 1.81 2.06
Mean.ADAS13 9.33 12.63 18.69 29.96
SD.ADAS13 4.33 5.40 6.52 8.05

t(cbind(Mean.Age.total,SD.AGE.total,Mean.MMSE.total,SD.MMSE.total,Mean.ADAS.total,SD.ADAS.total))

[,1]
Mean.Age.total 0.00
SD.AGE.total 7.25
Mean.MMSE.total 28.00
SD.MMSE.total 2.69
Mean.ADAS.total 17.41
SD.ADAS.total 9.59

## Mean age by eduction level

mean.AGEraw<-round(c(mean(initialdata$AGEraw[initialdata$edu.cat=="1post"],na.rm=TRUE),mean(initialdata$AGEraw[initialdata$edu.cat=="2tertiary"],na.rm=TRUE),mean(initialdata$AGEraw[initialdata$edu.cat=="3mid"],na.rm=TRUE),mean(initialdata$AGEraw[initialdata$edu.cat=="4early"],na.rm=TRUE)),2)

sd.AGEraw<-round(c(sd(initialdata$AGEraw[initialdata$edu.cat=="1post"],na.rm=TRUE),sd(initialdata$AGEraw[initialdata$edu.cat=="2tertiary"],na.rm=TRUE),sd(initialdata$AGEraw[initialdata$edu.cat=="3mid"],na.rm=TRUE),sd(initialdata$AGEraw[initialdata$edu.cat=="4early"],na.rm=TRUE)),2)

Education<-c("Post-graduate","Tertiary","Mid", "Early")

data.frame(Education, mean.AGEraw, sd.AGEraw)

Education mean.AGEraw sd.AGEraw
1 Post-graduate 73.31 7.31
2 Tertiary 73.94 7.19
3 Mid 73.56 7.53
4 Early 75.35 6.74

## Explanatory variables information by pain-reliever use

### Mean MMSE scores for each pain-reliever

Aspirin<-mean(initialdata$MMSE.bl[initialdata$aspirin==1])
Aspirin.sd<-sd(initialdata$MMSE.bl[initialdata$aspirin==1])
Celecoxib<-mean(initialdata$MMSE.bl[initialdata$celex==1])
Celecoxib.sd<-sd(initialdata$MMSE.bl[initialdata$celex==1])
Diclofenac<-mean(initialdata$MMSE.bl[initialdata$diclo==1])
Diclofenac.sd<-sd(initialdata$MMSE.bl[initialdata$diclo==1])
Ibuprofen<-mean(initialdata$MMSE.bl[initialdata$Ibu==1])
Ibuprofen.sd<-sd(initialdata$MMSE.bl[initialdata$Ibu==1])
Naproxen<-mean(initialdata$MMSE.bl[initialdata$naprox==1])
Naproxen.sd<-sd(initialdata$MMSE.bl[initialdata$naprox==1])
Paracetamol<-mean(initialdata$MMSE.bl[initialdata$parac==1])
Paracetamol.sd<-sd(initialdata$MMSE.bl[initialdata$parac==1])

no.painrelief<-mean(initialdata$MMSE.bl[initialdata$No.Painrelief==1])
no.painrelief.sd<-sd(initialdata$MMSE.bl[initialdata$No.Painrelief==1])

MMSE.table<-data.frame(Aspirin,Aspirin.sd,Celecoxib,Celecoxib.sd,Diclofenac,Diclofenac.sd,Ibuprofen,Ibuprofen.sd,Naproxen,Naproxen.sd,Paracetamol,Paracetamol.sd,no.painrelief,no.painrelief.sd)

MMSE.table

Aspirin Aspirin.sd Celecoxib Celecoxib.sd Diclofenac Diclofenac.sd
1 27.34959 2.60763 27.25 2.594255 27.96667 2.385059
 Ibuprofen Ibuprofen.sd Naproxen Naproxen.sd Paracetamol Paracetamol.sd
1 27.73333 2.189692 27.63978 2.412653 27.3675 2.525547
 no.painrelief no.painrelief.sd
1 26.42797 2.83051

lm0<-glm.nb(round(30-MMSE.bl)~1,data=initialdata)

lm1<-glm.nb(round(30-MMSE.bl)~aspirin+ celex+diclo+Ibu+naprox+parac,data=initialdata)
summary(lm1)

Call:
glm.nb(formula = round(30 - MMSE.bl) ~ aspirin + celex + diclo +
 Ibu + naprox + parac, data = initialdata, init.theta = 1.733486213,
 link = log)

Deviance Residuals:
 Min 1Q Median 3Q Max
-1.9600 -0.9649 -0.2836 0.4916 2.3421

Coefficients:
 Estimate Std. Error z value Pr(>|z|)
(Intercept) 1.25734 0.03744 33.580 < 2e-16 ***
aspirin -0.19295 0.04802 -4.018 5.86e-05 ***
celex -0.03888 0.12422 -0.313 0.754276
diclo -0.32975 0.19247 -1.713 0.086667 .
Ibu -0.26755 0.06897 -3.879 0.000105 ***
naprox -0.18471 0.07889 -2.341 0.019208 *
parac -0.07534 0.05750 -1.310 0.190077
---
Signif. codes: 0 '***' 0.001 '**' 0.01 '*' 0.05 '.' 0.1 ' ' 1

(Dispersion parameter for Negative Binomial(1.7335) family taken to be 1)

 Null deviance: 1902.6 on 1618 degrees of freedom
Residual deviance: 1852.9 on 1612 degrees of freedom
AIC: 7126.6

Number of Fisher Scoring iterations: 1

 Theta: 1.733
 Std. Err.: 0.109

 2 x log-likelihood: -7110.606

par(mfrow=c(2,2)); plot(lm1)

Figure description: Residual distribution plots for evaluating the assumptions of the least-square methods.


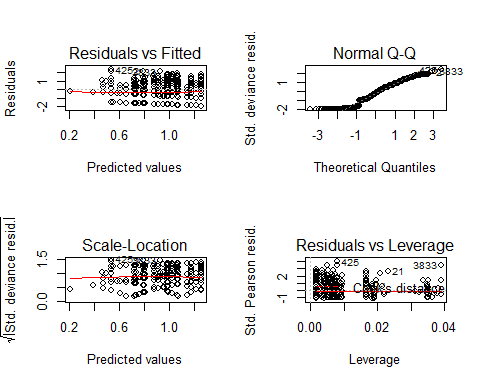


anova(lm0,lm1)

Likelihood ratio tests of Negative Binomial Models

Response: round(30 - MMSE.bl)
 Model theta Resid. df
1 1 1.634106 1618
2 aspirin + celex + diclo + Ibu + naprox + parac 1.733486 1612
 2 x log-lik. Test df LR stat. Pr(Chi)
1 -7159.351
2 -7110.606 1 vs 2 6 48.74534 8.384167e-09

aspirin.lm<-glm.nb(round(30-MMSE.bl)~ celex+diclo+Ibu+naprox+parac,data=initialdata)
celex.lm<-glm.nb(round(30-MMSE.bl)~aspirin+ diclo+Ibu+naprox+parac,data=initialdata)
diclo.lm<-glm.nb(round(30-MMSE.bl)~aspirin+ celex+Ibu+naprox+parac,data=initialdata)
Ibu.lm<-glm.nb(round(30-MMSE.bl)~aspirin+ celex+diclo+naprox+parac,data=initialdata)
naprox.lm<-glm.nb(round(30-MMSE.bl)~aspirin+ celex+diclo+Ibu+parac,data=initialdata)
parac.lm<-glm.nb(round(30-MMSE.bl)~aspirin+ celex+diclo+Ibu+naprox,data=initialdata)

Drug<-c("Aspirin","Celecoxib","Diclofenac","Ibuprofen","Naproxen","Ibuprofen")
table<-cbind(Drug,rbind(anova(aspirin.lm,lm1)[2,6:8],
anova(celex.lm,lm1)[2,6:8],
anova(diclo.lm,lm1)[2,6:8],
anova(Ibu.lm,lm1)[2,6:8],
anova(naprox.lm,lm1)[2,6:8],
anova(parac.lm,lm1)[2,6:8]))
Adjusted.P.Value<-p.adjust(table[,4],method="bonferroni")
table<-cbind(table,Adjusted.P.Value)
table

Drug df LR stat. Pr(Chi) Adjusted.P.Value
2 Aspirin 1 16.01054233 6.299075e-05 0.0003779445
21 Celecoxib 1 0.09710129 7.553363e-01 1.0000000000
22 Diclofenac 1 2.93354306 8.675690e-02 0.5205414207
23 Ibuprofen 1 14.72444695 1.244225e-04 0.0007465348
24 Naproxen 1 5.41874133 1.992172e-02 0.1195302980
25 Ibuprofen 1 1.71015062 1.909658e-01 1.0000000000

### Mean ADAS scores for each pain-reliever

Aspirin<-mean(initialdata$ADAS13.bl[initialdata$aspirin==1],na.rm=TRUE)
Aspirin.sd<-sd(initialdata$ADAS13.bl[initialdata$aspirin==1],na.rm=TRUE)
Celecoxib<-mean(initialdata$ADAS13.bl[initialdata$celex==1],na.rm=TRUE)
Celecoxib.sd<-sd(initialdata$ADAS13.bl[initialdata$celex==1],na.rm=TRUE)
Diclofenac<-mean(initialdata$ADAS13.bl[initialdata$diclo==1],na.rm=TRUE)
Diclofenac.sd<-sd(initialdata$ADAS13.bl[initialdata$diclo==1],na.rm=TRUE)
Ibuprofen<-mean(initialdata$ADAS13.bl[initialdata$Ibu==1],na.rm=TRUE)
Ibuprofen.sd<-sd(initialdata$ADAS13.bl[initialdata$Ibu==1],na.rm=TRUE)
Naproxen<-mean(initialdata$ADAS13.bl[initialdata$naprox==1],na.rm=TRUE)
Naproxen.sd<-sd(initialdata$ADAS13.bl[initialdata$naprox==1],na.rm=TRUE)
Paracetamol<-mean(initialdata$ADAS13.bl[initialdata$parac==1],na.rm=TRUE)
Paracetamol.sd<-sd(initialdata$ADAS13.bl[initialdata$parac==1],na.rm=TRUE)
no.painrelief<-mean(initialdata$ADAS13.bl[initialdata$No.Painrelief==1],na.rm=TRUE)
no.painrelief.sd<-sd(initialdata$ADAS13.bl[initialdata$No.Painrelief==1],na.rm=TRUE)


ADAS.table<-data.frame(Aspirin,Aspirin.sd,Celecoxib,Celecoxib.sd,Diclofenac,Diclofenac.sd,Ibuprofen,Ibuprofen.sd,Naproxen,Naproxen.sd,Paracetamol,Paracetamol.sd,no.painrelief,no.painrelief.sd)

ADAS.table

Aspirin Aspirin.sd Celecoxib Celecoxib.sd Diclofenac Diclofenac.sd
1 16.37426 9.014807 15.81492 8.324503 12.29897 8.450123
 Ibuprofen Ibuprofen.sd Naproxen Naproxen.sd Paracetamol Paracetamol.sd
1 14.99209 8.451613 15.44269 8.941314 16.20937 8.792672
 no.painrelief no.painrelief.sd
1 19.69797 10.34947

lm0<-glm.nb(round(ADAS13.bl*3)~1,data=initialdata)
lm1<-glm.nb(round(ADAS13.bl*3)~aspirin+ celex+diclo+Ibu+naprox+parac,data=initialdata)
summary(lm1)

Call:
glm.nb(formula = round(ADAS13.bl * 3) ~ aspirin + celex + diclo +
 Ibu + naprox + parac, data = initialdata, init.theta = 3.423409156,
 link = log)

Deviance Residuals:
 Min 1Q Median 3Q Max
-4.3000 -0.9026 -0.1479 0.5227 2.4920

Coefficients:
 Estimate Std. Error z value Pr(>|z|)
(Intercept) 4.06734 0.02229 182.441 < 2e-16 ***
aspirin -0.11240 0.02806 -4.006 6.18e-05 ***
celex -0.08914 0.07238 -1.232 0.21810
diclo -0.32162 0.10633 -3.025 0.00249 **
Ibu -0.15733 0.03872 -4.063 4.85e-05 ***
naprox -0.09776 0.04438 -2.203 0.02759 *
parac -0.04834 0.03315 -1.458 0.14475
---
Signif. codes: 0 '***' 0.001 '**' 0.01 '*' 0.05 '.' 0.1 ' ' 1

(Dispersion parameter for Negative Binomial(3.4234) family taken to be 1)

 Null deviance: 1751.0 on 1606 degrees of freedom
Residual deviance: 1692.5 on 1600 degrees of freedom
 (12 observations deleted due to missingness)
AIC: 15064

Number of Fisher Scoring iterations: 1

 Theta: 3.423
 Std. Err.: 0.126

 2 x log-likelihood: -15047.771

anova(lm0,lm1)

Likelihood ratio tests of Negative Binomial Models

Response: round(ADAS13.bl * 3)
 Model theta Resid. df
1 1 3.302247 1606
2 aspirin + celex + diclo + Ibu + naprox + parac 3.423409 1600
 2 x log-lik. Test df LR stat. Pr(Chi)
1 -15105.28
2 -15047.77 1 vs 2 6 57.51281 1.438381e-10

par(mfrow=c(2,2)); plot(lm1)

Figure description: Residual distribution plots for evaluating the assumptions of the least-square methods.


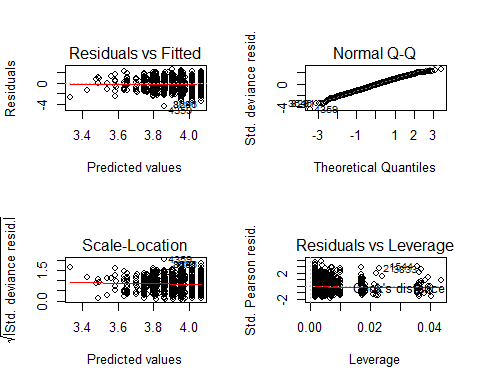


aspirin.lm<-glm.nb(round(ADAS13.bl*3)~ celex+diclo+Ibu+naprox+parac,data=initialdata)
celex.lm<-glm.nb(round(ADAS13.bl*3)~aspirin+ diclo+Ibu+naprox+parac,data=initialdata)
diclo.lm<-glm.nb(round(ADAS13.bl*3)~aspirin+ celex+Ibu+naprox+parac,data=initialdata)
Ibu.lm<-glm.nb(round(ADAS13.bl*3)~aspirin+ celex+diclo+naprox+parac,data=initialdata)
naprox.lm<-glm.nb(round(ADAS13.bl*3)~aspirin+ celex+diclo+Ibu+parac,data=initialdata)
parac.lm<-glm.nb(round(ADAS13.bl*3)~aspirin+ celex+diclo+Ibu+naprox,data=initialdata)

Drug<-c("Aspirin","Celecoxib","Diclofenac","Ibuprofen","Naproxen","Ibuprofen")
table<-cbind(Drug,rbind(anova(aspirin.lm,lm1)[2,6:8],
anova(celex.lm,lm1)[2,6:8],
anova(diclo.lm,lm1)[2,6:8],
anova(Ibu.lm,lm1)[2,6:8],
anova(naprox.lm,lm1)[2,6:8],
anova(parac.lm,lm1)[2,6:8]))
Adjusted.P.Value<-p.adjust(table[,4],method="bonferroni")
table<-cbind(table,Adjusted.P.Value)
table

Drug df LR stat. Pr(Chi) Adjusted.P.Value
2 Aspirin 1 15.930344 6.571667e-05 0.000394300
21 Celecoxib 1 1.479511 2.238510e-01 1.000000000
22 Diclofenac 1 8.419487 3.712204e-03 0.022273225
23 Ibuprofen 1 15.925698 6.587817e-05 0.000395269
24 Naproxen 1 4.733385 2.958261e-02 0.177495664
25 Ibuprofen 1 2.103388 1.469732e-01 0.881839124

### Education level for each pain-reliever

Aspirin<-table(initialdata$edu.cat,by=initialdata$aspirin)[,2]
Celecoxib<-table(initialdata$edu.cat,by=initialdata$celex)[,2]
Diclofenac<-table(initialdata$edu.cat,by=initialdata$diclo)[,2]
Ibuprofen<-table(initialdata$edu.cat,by=initialdata$Ibu)[,2]
Naproxen<-table(initialdata$edu.cat,by=initialdata$naprox)[,2]
Paracetamol<-table(initialdata$edu.cat,by=initialdata$parac)[,2]
No.Painrelief<-table(initialdata$edu.cat,by=initialdata$No.Painrelief)[,2]

Education.table<-data.frame(t(cbind(Aspirin,Celecoxib,Diclofenac,Ibuprofen,Naproxen,Paracetamol,No.Painrelief)))
names(Education.table)<-c("Primary","Secondary","Tertiary","Post-grad")
Education.table$Primary.percent<-Education.table[,1]/(Education.table[,2]+Education.table[,3]+Education.table[,4]+Education.table[,1])*100
Education.table$Secondary.percent<-Education.table[,2]/(Education.table[,2]+Education.table[,3]+Education.table[,4]+Education.table[,1])*100
Education.table$Tertiary.percent<-Education.table[,3]/(Education.table[,2]+Education.table[,1]+Education.table[,3]+Education.table[,4])*100
Education.table$Postgrad.percent<-Education.table[,4]/(Education.table[,2]+Education.table[,1]+Education.table[,3]+Education.table[,4])*100
Education.table

Primary Secondary Tertiary Post-grad Primary.percent
Aspirin 318 251 166 126 36.93380
Celecoxib 21 18 12 13 32.81250
Diclofenac 10 11 3 6 33.33333
Ibuprofen 83 77 50 45 32.54902
Naproxen 56 59 40 31 30.10753
Paracetamol 128 119 92 61 32.00000
No.Painrelief 161 126 92 93 34.11017
 Secondary.percent Tertiary.percent Postgrad.percent
Aspirin 29.15215 19.27991 14.63415
Celecoxib 28.12500 18.75000 20.31250
Diclofenac 36.66667 10.00000 20.00000
Ibuprofen 30.19608 19.60784 17.64706
Naproxen 31.72043 21.50538 16.66667
Paracetamol 29.75000 23.00000 15.25000
No.Painrelief 26.69492 19.49153 19.70339

Education.table.chisq<-chisq.test(Education.table[,1:4])
Education.table.chisq

Pearson's Chi-squared test

data: Education.table[, 1:4]
X-squared = 15.702, df = 18, p-value = 0.6133

### Diagnosis prevalence for each pain-reliever

Aspirin<-table(initialdata$diagn,by=initialdata$aspirin)[,2]
Celecoxib<-table(initialdata$diagn,by=initialdata$celex)[,2]
Diclofenac<-table(initialdata$diagn,by=initialdata$diclo)[,2]
Ibuprofen<-table(initialdata$diagn,by=initialdata$Ibu)[,2]
Naproxen<-table(initialdata$diagn,by=initialdata$naprox)[,2]
Paracetamol<-table(initialdata$diagn,by=initialdata$parac)[,2]
No.Painrelief<-table(initialdata$diagn,by=initialdata$No.Painrelief)[,1]

Diagnosis.table<-data.frame(t(cbind(Aspirin,Celecoxib,Diclofenac,Ibuprofen,Naproxen,Paracetamol,No.Painrelief)))
Diagnosis.table<-Diagnosis.table[,2:5]
names(Diagnosis.table)<-c("CN","EMCI","LMCI","AD")
Diagnosis.table$CN.percent<-Diagnosis.table[,1]/(Diagnosis.table[,2]+Diagnosis.table[,3]+Diagnosis.table[,4]+Diagnosis.table[,1])*100

Diagnosis.table$EMCI.percent<-Diagnosis.table[,2]/(Diagnosis.table[,2]+Diagnosis.table[,3]+Diagnosis.table[,4]+Diagnosis.table[,1])*100

Diagnosis.table$LMCI.percent<-Diagnosis.table[,3]/(Diagnosis.table[,2]+Diagnosis.table[,3]+Diagnosis.table[,4]+Diagnosis.table[,1])*100

Diagnosis.table$AD.percent<-Diagnosis.table[,4]/(Diagnosis.table[,2]+Diagnosis.table[,3]+Diagnosis.table[,4]+Diagnosis.table[,1])*100

Diagnosis.table$total<-(Diagnosis.table[,5]+Diagnosis.table[,3]+Diagnosis.table[,4]+Diagnosis.table[,1])

Diagnosis.table

CN EMCI LMCI AD CN.percent EMCI.percent LMCI.percent
Aspirin 247 174 302 138 28.68757 20.20906 35.07549
Celecoxib 19 13 25 7 29.68750 20.31250 39.06250
Diclofenac 14 5 8 3 46.66667 16.66667 26.66667
Ibuprofen 79 55 92 29 30.98039 21.56863 36.07843
Naproxen 52 45 68 21 27.95699 24.19355 36.55914
Paracetamol 119 75 140 66 29.75000 18.75000 35.00000
No.Painrelief 316 239 399 193 27.55013 20.83697 34.78640
 AD.percent total
Aspirin 16.02787 715.68757
Celecoxib 10.93750 80.68750
Diclofenac 10.00000 71.66667
Ibuprofen 11.37255 230.98039
Naproxen 11.29032 168.95699
Paracetamol 16.50000 354.75000
No.Painrelief 16.82650 935.55013

chisq<-chisq.test(Diagnosis.table[,1:4])


chisq

Pearson's Chi-squared test

data: Diagnosis.table[, 1:4]
X-squared = 16.018, df = 18, p-value = 0.5913

contrib<-100*chisq$residuals^2/chisq$statistic
contrib

CN EMCI LMCI AD
Aspirin 0.006422259 0.38127839 0.005275827 0.8636755
Celecoxib 0.123161661 0.01507559 1.754906342 5.4230442
Diclofenac 20.923751048 1.40094473 3.822127704 3.6862821
Ibuprofen 2.764449028 0.73857709 0.403970804 17.7062696
Naproxen 0.251589544 7.31800340 0.671064282 13.4312919
Paracetamol 0.875371732 4.11144039 0.012803893 1.5182119
No.Painrelief 3.563511609 0.20999316 0.246566813 7.7709395

res1 <- cor.mtest(contrib, conf.level = 0.95)

col2 <- colorRampPalette(c("#67001F", "#B2182B", "#D6604D", "#F4A582",
 "#FDDBC7", "#FFFFFF", "#D1E5F0", "#92C5DE",
 "#4393C3", "#2166AC", "#053061"))


pearson<-chisq$residuals

#pdf("Risidual.#pdf", width=16/2.54, heigh=12/2.54 , useDingbats=F)
corrplot(contrib, p.mat = res1$p, insig = "blank",pch.col=1,is.cor=F,tl.col=1, cl.pos="b", number.digits=2,number.cex=0.5,cl.cex=1,cl.length=5, cl.lim=c(0,50), col=rev(col2(200)))

Figure description: Percentage contribution to the significant difference in proportions of each cognitive diagnosis by pain-reliever.


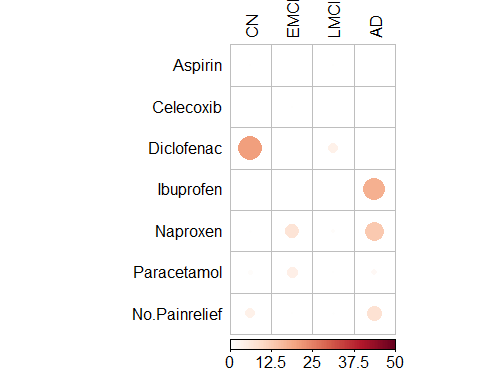


#dev.off()

#pdf("Percentage.#pdf", width=16/2.54, heigh=12/2.54, useDingbats=F)

corrplot(pearson, p.mat = res1$p, insig = "blank",pch.col=1,is.cor=F,tl.col=1, cl.pos="b", number.digits=2,number.cex=0.5,cl.cex=1,cl.length=5, cl.lim=c(-4,4),col=rev(col2(200)))

Figure description: Z-scores of the proportions of each cognitive diagnosis per pain-reliever.


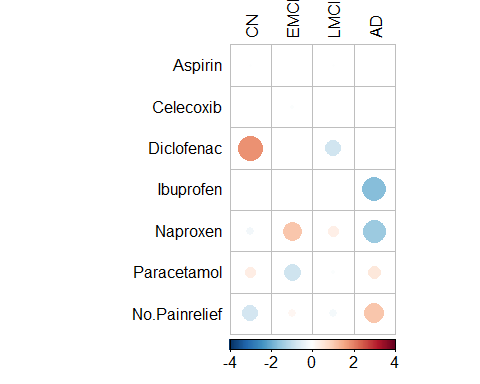


#dev.off()

Diagnosis.table$Painrelief<-rownames(Diagnosis.table)

melt.diag<-melt(Diagnosis.table,id="Painrelief")
percent<-c("CN.percent","EMCI.percent","LMCI.percent","AD.percent")
melt.diag<-melt.diag[(melt.diag$variable%in%percent),]

positions<-c("No.Painrelief","Aspirin","Celecoxib","Diclofenac","Ibuprofen","Naproxen","Paracetamol")

#pdf("Proportions.#pdf", width=16/2.54, heigh=12/2.54, useDingbats=F)
qplot(x=Painrelief,y=value,fill=variable,data=melt.diag,geom="col")+ theme(axis.text.x = element_text(size=10, angle=60, hjust=1), plot.background=element_rect(0),panel.background=element_rect(0),axis.line=element_line(1, size=1))+
 scale_y_continuous(expand = c(0, 0), breaks=seq(0, 100,by=20),limits=c(0,100))+
 scale_fill_manual(values=c("#7cafe2","#4b912299","#e4630066","#f78a8a"))+
 scale_x_discrete(limits = positions)

Figure description: Graphically summary of cognitive diagnosis proportions per NSAID


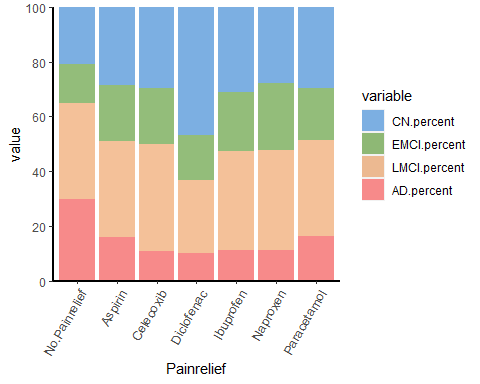


#dev.off()

#### Anaylsis of prevalence

initialdata$AD<-initialdata$diagn == "4AD"
str(initialdata$APOE4)

Factor w/ 3 levels "0","1","2": 1 2 1 1 1 2 2 1 2 2 ...

logr.Full<-glm(AD ~ AGE+Gender+edu.cat+APOE4, data=initialdata, binomial(link = "logit"))
step.logr<-stepAIC(logr.Full, direction= "both", trace = F)

Error in stepAIC(logr.Full, direction = "both", trace = F): functions step and MASS::stepAIC are **not** currently compatible with glmmADMB. Sorry.

summary(step.logr)

Error in summary(step.logr): object 'step.logr' not found

aspirin.logr<-glm(AD ~ AGE+edu.cat+APOE4+aspirin, data=initialdata, binomial(link = "logit"))
parac.logr<-glm(AD ~ AGE+edu.cat+APOE4+parac, data=initialdata, binomial(link = "logit"))
diclo.logr<-glm(AD ~ AGE+edu.cat+APOE4+diclo, data=initialdata, binomial(link = "logit"))
Ibu.logr<-glm(AD ~ AGE+edu.cat+APOE4+Ibu, data=initialdata, binomial(link = "logit"))
celex.logr<-glm(AD ~ AGE+edu.cat+APOE4+celex, data=initialdata, binomial(link = "logit"))
naprox.logr<-glm(AD ~ AGE+edu.cat+APOE4+naprox, data=initialdata, binomial(link = "logit"))
No.Drug.logr<-glm(AD ~ AGE+edu.cat+APOE4+No.Painrelief, data=initialdata, binomial(link = "logit"))

Deviance<-c(
anova(aspirin.logr, step.logr)[[4]][2],
anova(celex.logr, step.logr)[[4]][2],
anova(diclo.logr, step.logr)[[4]][2],
anova(Ibu.logr, step.logr)[[4]][2],
anova(naprox.logr, step.logr)[[4]][2],
anova(parac.logr, step.logr)[[4]][2],
anova(No.Drug.logr, step.logr)[[4]][2]
)

Error in anova.glm(aspirin.logr, step.logr): object 'step.logr' not found

Drug<-c("Aspirin", "Celecoxib", "Diclofenac", "Ibuprofen", "Naproxen", "Paracetamol", "No Painrelief")

Prevalance<-data.frame(Drug, Deviance)

Error in data.frame(Drug, Deviance): object 'Deviance' not found

Prevalance$P.value<- dchisq(abs(Prevalance$Deviance),c(rep(1,7)))

Error in dchisq(abs(Prevalance$Deviance), c(rep(1, 7))): object 'Prevalance' not found

print(Prevalance)

Error in print(Prevalance): object 'Prevalance' not found

newdata.aspirin<-data.frame(AGE=mean(initialdata$AGE), edu.cat = "1post", APOE4="0", aspirin=1)
newdata.celex<-data.frame(AGE=mean(initialdata$AGE), edu.cat = "1post", APOE4="0", celex=1)
newdata.diclo<-data.frame(AGE=mean(initialdata$AGE), edu.cat = "1post", APOE4="0", diclo=1)
newdata.Ibu<-data.frame(AGE=mean(initialdata$AGE), edu.cat = "1post", APOE4="0", Ibu=1)
newdata.Naprox<-data.frame(AGE=mean(initialdata$AGE), edu.cat = "1post", APOE4="0", naprox=1)
newdata.parac<-data.frame(AGE=mean(initialdata$AGE), edu.cat = "1post", APOE4="0", parac=1)
newdata.No.Drug<-data.frame(AGE=mean(initialdata$AGE), edu.cat = "1post", APOE4="0", No.Painrelief=1)


Proportion<-c(
 predict(aspirin.logr,newdata.aspirin, type="link", se.fit = T)[[1]],
 predict(celex.logr,newdata.celex, type="link", se.fit = T)[[1]],
 predict(diclo.logr,newdata.diclo, type="link", se.fit = T)[[1]],
 predict(Ibu.logr,newdata.Ibu, type="link", se.fit = T)[[1]],
 predict(naprox.logr,newdata.Naprox, type="link", se.fit = T)[[1]],
 predict(parac.logr,newdata.parac, type="link", se.fit = T)[[1]],
 predict(No.Drug.logr,newdata.No.Drug, type="link", se.fit = T)[[1]])

SE<-c(
 predict(aspirin.logr,newdata.aspirin, type="link", se.fit = T)[[2]],
 predict(celex.logr,newdata.celex, type="link", se.fit = T)[[2]],
 predict(diclo.logr,newdata.diclo, type="link", se.fit = T)[[2]],
 predict(Ibu.logr,newdata.Ibu, type="link", se.fit = T)[[2]],
 predict(naprox.logr,newdata.Naprox, type="link", se.fit = T)[[2]],
 predict(parac.logr,newdata.parac, type="link", se.fit = T)[[2]],
 predict(No.Drug.logr,newdata.No.Drug, type="link", se.fit = T)[[2]])

Upper<-Proportion+1.96*SE
Lower<-Proportion-1.96*SE

inverse_logit = function(x){
 exp(x)/(1+exp(x))
}

Predicted.value<-inverse_logit(Proportion)
Predicted.Upper<-inverse_logit(Upper)
Predicted.Lower<-inverse_logit(Lower)

Predicted.prevalence<-data.frame(Drug,Predicted.value, Predicted.Upper,Predicted.Lower)
print(Predicted.prevalence)

Drug Predicted.value Predicted.Upper Predicted.Lower
1 Aspirin 0.07259638 0.09713992 0.05388396
2 Celecoxib 0.04655658 0.10232394 0.02048915
3 Diclofenac 0.04213525 0.13305180 0.01245127
4 Ibuprofen 0.05097601 0.07959483 0.03228631
5 Naproxen 0.05007503 0.08244996 0.02999690
6 Paracetamol 0.07548478 0.10611354 0.05317088
7 No Painrelief 0.14593354 0.19125172 0.10989460

library(ggthemes)


ggplot(Predicted.prevalence, aes(x = Drug, y = Predicted.value)) +
 geom_point(size = 4) +
 geom_errorbar(aes(ymax = Predicted.Upper, ymin = Predicted.Lower), width=0.2) + theme_stata()+
 theme(axis.text.x = element_text(face = "bold", color = "#993333", size = 10, angle = 45, hjust = 1))

Figure description: Adjusted predicted proportions of AD individuals per pain-reliever subgroup with 95% confidence intervals.


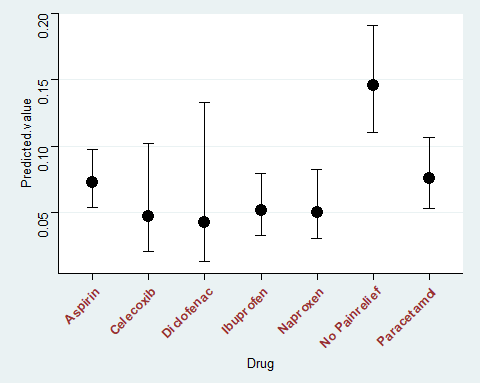


#### Significance of drug effects on prevalence

Drugs<-c("aspirin","celex","diclo","Ibu","naprox","parac")
Deviance<-numeric(6)
P.value<-numeric(6)
for(i in 1:6){

 column<-Drugs[i]

 dat<-initialdata[initialdata$No.Painrelief==1|initialdata[[column]]==1,]
 formula
 log1<-glm(AD ~ AGE+edu.cat+APOE4, data=dat, binomial(link = "logit"))
 formula<-paste("AD ~ AGE+edu.cat+APOE4+",paste(column))

 log2<-glm(paste(formula), data=dat, binomial(link = "logit"))
 Deviance[i]<-anova(log1,log2)[[4]][2]
 P.value[i]<-dchisq(abs(Deviance[i]),1)
 }
prevalence<-data.frame(Drugs,Deviance,P.value)
prevalence$adjusted.pvalue<-p.adjust(prevalence$P.value,method="holm")

print(prevalence)

Drugs Deviance P.value adjusted.pvalue
1 aspirin 30.939393 1.371672e-08 8.230032e-08
2 celex 11.188842 4.434977e-04 8.869955e-04
3 diclo 6.301739 6.804161e-03 6.804161e-03
4 Ibu 29.277174 3.237338e-08 1.618669e-07
5 naprox 23.495671 6.507228e-07 2.602891e-06
6 parac 18.863041 7.362868e-06 2.208860e-05

### APOE genotype proportions by pain reliever use

Aspirin<-table(initialdata$APOE4,by=initialdata$aspirin)[,2]
Celecoxib<-table(initialdata$APOE4,by=initialdata$celex)[,2]
Diclofenac<-table(initialdata$APOE4,by=initialdata$diclo)[,2]
Ibuprofen<-table(initialdata$APOE4,by=initialdata$Ibu)[,2]
Naproxen<-table(initialdata$APOE4,by=initialdata$naprox)[,2]
Paracetamol<-table(initialdata$APOE4,by=initialdata$parac)[,2]
No.Painrelief<-table(initialdata$APOE4,by=initialdata$No.Painrelief)[,2]


APOE4.table<-data.frame(t(cbind(Aspirin,Celecoxib,Diclofenac,Ibuprofen,Naproxen,Paracetamol,No.Painrelief)))
names(APOE4.table)<-c("WT","HET","HOMO")
APOE4.table$WT.Percent<-APOE4.table[,1]/(APOE4.table[,2]+APOE4.table[,3]+APOE4.table[,1])*100
APOE4.table$HET.Percent<-APOE4.table[,2]/(APOE4.table[,2]+APOE4.table[,3]+APOE4.table[,1])*100
APOE4.table$HOMO.Percent<-APOE4.table[,3]/(APOE4.table[,2]+APOE4.table[,3]+APOE4.table[,1])*100

APOE4.table

WT HET HOMO WT.Percent HET.Percent HOMO.Percent
Aspirin 467 305 89 54.23926 35.42393 10.336818
Celecoxib 35 24 5 54.68750 37.50000 7.812500
Diclofenac 18 10 2 60.00000 33.33333 6.666667
Ibuprofen 145 88 22 56.86275 34.50980 8.627451
Naproxen 102 67 17 54.83871 36.02151 9.139785
Paracetamol 231 133 36 57.75000 33.25000 9.000000
No.Painrelief 236 184 52 50.00000 38.98305 11.016949

APOE4.table.chisq<-chisq.test(APOE4.table[,1:3])
APOE4.table.chisq

Pearson's Chi-squared test

data: APOE4.table[, 1:3]
X-squared = 7.5119, df = 12, p-value = 0.822

### Gender proportions by pain reliever use

Aspirin<-table(initialdata$Gender,by=initialdata$aspirin)[,2]
Celecoxib<-table(initialdata$Gender,by=initialdata$celex)[,2]
Diclofenac<-table(initialdata$Gender,by=initialdata$diclo)[,2]
Ibuprofen<-table(initialdata$Gender,by=initialdata$Ibu)[,2]
Naproxen<-table(initialdata$Gender,by=initialdata$naprox)[,2]
Paracetamol<-table(initialdata$Gender,by=initialdata$parac)[,2]
No.Painrelief<-table(initialdata$Gender,by=initialdata$No.Painrelief)[,2]


Gender.table<-data.frame(t(cbind(Aspirin,Celecoxib,Diclofenac,Ibuprofen,Naproxen,Paracetamol,No.Painrelief)))
names(Gender.table)<-c("Male","Female")
Gender.table$Male.Percent<-Gender.table[,1]/(Gender.table[,2]+Gender.table[,1])*100
Gender.table$Female.Percent<-Gender.table[,2]/(Gender.table[,2]+Gender.table[,1])*100


Gender.table

Male Female Male.Percent Female.Percent
Aspirin 323 538 37.51452 62.48548
Celecoxib 31 33 48.43750 51.56250
Diclofenac 15 15 50.00000 50.00000
Ibuprofen 117 138 45.88235 54.11765
Naproxen 95 91 51.07527 48.92473
Paracetamol 204 196 51.00000 49.00000
No.Painrelief 223 249 47.24576 52.75424

Gender.table.chisq<-chisq.test(Gender.table[,1:2])
Gender.table.chisq

Pearson's Chi-squared test

data: Gender.table[, 1:2]
X-squared = 29.522, df = 6, p-value = 4.844e-05

contrib<-100*Gender.table.chisq$residuals^2/Gender.table.chisq$statistic
contrib

Male Female
Aspirin 31.5133437 25.2106750
Celecoxib 0.7777227 0.6221781
Diclofenac 0.7056841 0.5645473
Ibuprofen 0.4018248 0.3214599
Naproxen 6.2327840 4.9862272
Paracetamol 13.1012605 10.4810084
No.Painrelief 2.8229357 2.2583486

res1 <- cor.mtest(contrib, conf.level = 0.95)

col2 <- colorRampPalette(c("#67001F", "#B2182B", "#D6604D", "#F4A582",
 "#FDDBC7", "#FFFFFF", "#D1E5F0", "#92C5DE",
 "#4393C3", "#2166AC", "#053061"))


pearson<-Gender.table.chisq$residuals


corrplot(contrib, p.mat = res1$p, insig = "blank",pch.col=1,is.cor=F,tl.col=1, cl.pos="b", number.digits=2,number.cex=0.5,cl.cex=1,cl.length=3, cl.lim=c(0,50), col=rev(col2(200)))

Figure description: Percentage contribution to the significant difference in proportions of genders by pain-reliever.


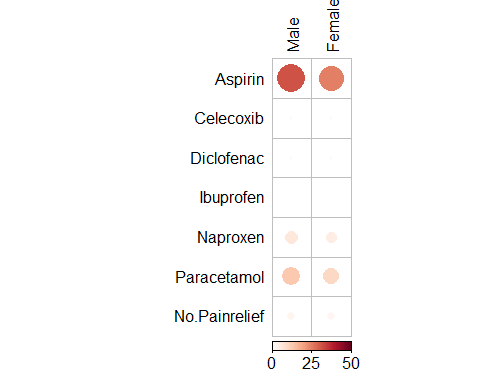


corrplot(pearson, p.mat = res1$p, insig = "blank",pch.col=1,is.cor=F,tl.col=1, cl.pos="b", number.digits=2,number.cex=0.5,cl.cex=1,cl.length=3, cl.lim=c(-8,8),col=rev(col2(200)))

Figure description: Z-scores of the proportions of gender per pain-reliever.


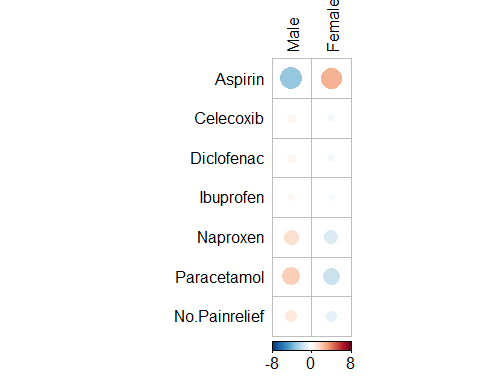


### Mean age by pain reliever use

Aspirin<-mean(initialdata$AGEraw[initialdata$aspirin==1])
Aspirin.sd<-sd(initialdata$AGEraw[initialdata$aspirin==1])
Celecoxib<-mean(initialdata$AGEraw[initialdata$celex==1])
Celecoxib.sd<-sd(initialdata$AGEraw[initialdata$celex==1])
Diclofenac<-mean(initialdata$AGEraw[initialdata$diclo==1])
Diclofenac.sd<-sd(initialdata$AGEraw[initialdata$diclo==1])
Ibuprofen<-mean(initialdata$AGEraw[initialdata$Ibu==1])
Ibuprofen.sd<-sd(initialdata$AGEraw[initialdata$Ibu==1])
Naproxen<-mean(initialdata$AGEraw[initialdata$naprox==1])
Naproxen.sd<-sd(initialdata$AGEraw[initialdata$naprox==1])
Paracetamol<-mean(initialdata$AGEraw[initialdata$parac==1])
Paracetamol.sd<-sd(initialdata$AGEraw[initialdata$parac==1])

No.Painrelief<-mean(initialdata$AGEraw[initialdata$No.Painrelief==1])
No.Painrelief.sd<-sd(initialdata$AGEraw[initialdata$No.Painrelief==1])

AGE.table<-data.frame(Aspirin,Aspirin.sd,Celecoxib,Celecoxib.sd,Diclofenac,Diclofenac.sd,Ibuprofen,Ibuprofen.sd,Naproxen,Naproxen.sd,Paracetamol,Paracetamol.sd,No.Painrelief,No.Painrelief.sd)

AGE.table

Aspirin Aspirin.sd Celecoxib Celecoxib.sd Diclofenac Diclofenac.sd
1 74.26469 6.685986 73.42344 6.940146 75.22 6.37362
 Ibuprofen Ibuprofen.sd Naproxen Naproxen.sd Paracetamol Paracetamol.sd
1 72.7149 6.809825 72.8957 7.085919 74.345 7.224428
 No.Painrelief No.Painrelief.sd
1 73.89915 8.079612

lm0<-lm(AGEraw~1,data=initialdata)
lm1<-lm(AGEraw~aspirin+ celex+diclo+Ibu+naprox+parac,data=initialdata)
summary(lm1)

Call:
lm(formula = AGEraw ~ aspirin + celex + diclo + Ibu + naprox +
 parac, data = initialdata)

Residuals:
 Min 1Q Median 3Q Max
-19.4619 -4.4620 0.0369 5.0772 18.4227

Coefficients:
 Estimate Std. Error t value Pr(>|t|)
(Intercept) 73.6222 0.2877 255.912 < 2e-16 ***
aspirin 0.8141 0.3616 2.252 0.02447 *
celex -0.8279 0.9264 -0.894 0.37164
diclo 1.1589 1.3345 0.868 0.38530
Ibu -1.4590 0.4977 -2.931 0.00342 **
naprox -1.2129 0.5718 -2.121 0.03406 *
parac 0.8397 0.4267 1.968 0.04927 *
---
Signif. codes: 0 '***' 0.001 '**' 0.01 '*' 0.05 '.' 0.1 ' ' 1

Residual standard error: 7.218 on 1612 degrees of freedom
Multiple R-squared: 0.01352, Adjusted R-squared: 0.009851
F-statistic: 3.683 on 6 and 1612 DF, p-value: 0.001224

anova(lm0,lm1)

Analysis of Variance Table

Model 1: AGEraw ~ 1
Model 2: AGEraw ~ aspirin + celex + diclo + Ibu + naprox + parac
 Res.Df RSS Df Sum of Sq F Pr(>F)
1 1618 85132
2 1612 83981 6 1151.2 3.6829 0.001224 **
---
Signif. codes: 0 '***' 0.001 '**' 0.01 '*' 0.05 '.' 0.1 ' ' 1

par(mfrow=c(2,2)); plot(lm1)

Figure description: Residual distribution plots for evaluating the assumptions of the least-square methods.


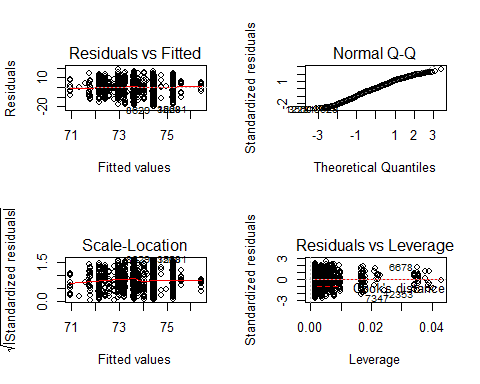


aspirin.lm1<-lm(AGEraw~ celex+diclo+Ibu+naprox+parac,data=initialdata)
celex.lm1<-lm(AGEraw~aspirin+ diclo+Ibu+naprox+parac,data=initialdata)
diclo.lm1<-lm(AGEraw~aspirin+ celex+Ibu+naprox+parac,data=initialdata)
Ibu.lm1<-lm(AGEraw~aspirin+ celex+diclo+naprox+parac,data=initialdata)
naprox.lm1<-lm(AGEraw~aspirin+ celex+diclo+Ibu+parac,data=initialdata)
parac.lm1<-lm(AGEraw~aspirin+ celex+diclo+Ibu+naprox,data=initialdata)

Drug<-c("Aspirin","Celecoxib","Diclofenac","Ibuprofen","Naproxen","Ibuprofen")
table<-cbind(Drug,rbind(anova(aspirin.lm1,lm1)[2,5:6],
anova(celex.lm1,lm1)[2,5:6],
anova(diclo.lm1,lm1)[2,5:6],
anova(Ibu.lm1,lm1)[2,5:6],
anova(naprox.lm1,lm1)[2,5:6],
anova(parac.lm1,lm1)[2,5:6]))
Adjusted.P.Value<-p.adjust(table[,3],method="bonferroni")
table<-cbind(table,Adjusted.P.Value)
table

Drug F Pr(>F) Adjusted.P.Value
2 Aspirin 5.0703195 0.024473042 0.14683825
21 Celecoxib 0.7986302 0.371636630 1.00000000
22 Diclofenac 0.7541348 0.385299554 1.00000000
23 Ibuprofen 8.5926115 0.003422854 0.02053712
24 Naproxen 4.4994038 0.034058935 0.20435361
25 Ibuprofen 3.8720763 0.049266946 0.29560168

### Smoking status proportions by pain reliever use

Aspirin<-table(initialdata$smoke,by=initialdata$aspirin)[,2]
Celecoxib<-table(initialdata$smoke,by=initialdata$celex)[,2]
Diclofenac<-table(initialdata$smoke,by=initialdata$diclo)[,2]
Ibuprofen<-table(initialdata$smoke,by=initialdata$Ibu)[,2]
Naproxen<-table(initialdata$smoke,by=initialdata$naprox)[,2]
Paracetamol<-table(initialdata$smoke,by=initialdata$parac)[,2]
No.Painrelief<-table(initialdata$smoke,by=initialdata$No.Painrelief)[,2]

Smoking.table<-data.frame(t(cbind(Aspirin,Celecoxib,Diclofenac,Ibuprofen,Naproxen,Paracetamol,No.Painrelief)))
names(Smoking.table)<-c("Non-smoker","Smoker")
Smoking.table$percent<-Smoking.table[,2]/(Smoking.table[,2]+Smoking.table[,1])*100
Smoking.table

Non-smoker Smoker percent
Aspirin 656 205 23.80952
Celecoxib 47 17 26.56250
Diclofenac 22 8 26.66667
Ibuprofen 184 71 27.84314
Naproxen 146 40 21.50538
Paracetamol 293 107 26.75000
No.Painrelief 345 127 26.90678

chisq.test(Smoking.table[,1:2])

Pearson's Chi-squared test

data: Smoking.table[, 1:2]
X-squared = 4.4658, df = 6, p-value = 0.6139

### Arthritis proportions by pain reliever use

Aspirin<-table(initialdata$arthrit,by=initialdata$aspirin)[,2]
Celecoxib<-table(initialdata$arthrit,by=initialdata$celex)[,2]
Diclofenac<-table(initialdata$arthrit,by=initialdata$diclo)[,2]
Ibuprofen<-table(initialdata$arthrit,by=initialdata$Ibu)[,2]
Naproxen<-table(initialdata$arthrit,by=initialdata$naprox)[,2]
Paracetamol<-table(initialdata$arthrit,by=initialdata$parac)[,2]
No.Painrelief<-table(initialdata$arthrit,by=initialdata$No.Painrelief)[,2]

Arthritis.table<-data.frame(t(cbind(Aspirin,Celecoxib,Diclofenac,Ibuprofen,Naproxen,Paracetamol,No.Painrelief)))
names(Arthritis.table)<-c("Non-Arthritis","Arthritis")
Arthritis.table$percent<-Arthritis.table[,2]/(Arthritis.table[,2]+Arthritis.table[,1])*100
Arthritis.table

Non-Arthritis Arthritis percent
Aspirin 519 342 39.72125
Celecoxib 17 47 73.43750
Diclofenac 7 23 76.66667
Ibuprofen 128 127 49.80392
Naproxen 88 98 52.68817
Paracetamol 185 215 53.75000
No.Painrelief 340 132 27.96610

chisq.test(Arthritis.table[,1:2])

Pearson's Chi-squared test

data: Arthritis.table[, 1:2]
X-squared = 115.78, df = 6, p-value < 2.2e-16

chisq<-chisq.test(Arthritis.table[,1:2])


chisq

Pearson's Chi-squared test

data: Arthritis.table[, 1:2]
X-squared = 115.78, df = 6, p-value < 2.2e-16

contrib<-100*chisq$residuals^2/chisq$statistic
contrib

Non-Arthritis Arthritis
Aspirin 1.764408 2.302338
Celecoxib 8.817711 11.506037
Diclofenac 5.069318 6.614842
Ibuprofen 1.602310 2.090820
Naproxen 2.455328 3.203904
Paracetamol 6.554582 8.552930
No.Painrelief 17.122585 22.342886

res1 <- cor.mtest(contrib, conf.level = 0.95)

col2 <- colorRampPalette(c("#67001F", "#B2182B", "#D6604D", "#F4A582",
 "#FDDBC7", "#FFFFFF", "#D1E5F0", "#92C5DE",
 "#4393C3", "#2166AC", "#053061"))


pearson<-chisq$residuals


corrplot(contrib, p.mat = res1$p, insig = "blank",pch.col=1,is.cor=F,tl.col=1, cl.pos="b", number.digits=2,number.cex=0.5,cl.cex=1,cl.length=3, cl.lim=c(0,50), col=rev(col2(200)))

Figure description: Percentage contribution to the significant difference in proportions of Arthritis sufferers by pain-reliever.


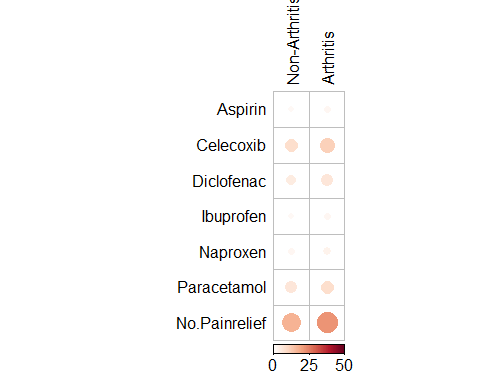


corrplot(pearson, p.mat = res1$p, insig = "blank",pch.col=1,is.cor=F,tl.col=1, cl.pos="b", number.digits=2,number.cex=0.5,cl.cex=1,cl.length=3, cl.lim=c(-8,8),col=rev(col2(200)))

Figure description: Z-scores of the proportions of arthritis sufferers per pain-reliever.


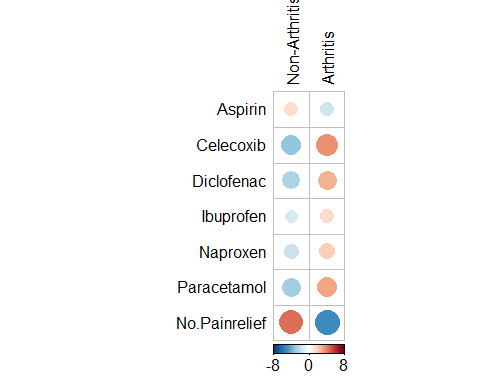


### Headache sufferer proportions by pain reliever use

Aspirin<-table(initialdata$headache,by=initialdata$aspirin)[,2]
Celecoxib<-table(initialdata$headache,by=initialdata$celex)[,2]
Diclofenac<-table(initialdata$headache,by=initialdata$diclo)[,2]
Ibuprofen<-table(initialdata$headache,by=initialdata$Ibu)[,2]
Naproxen<-table(initialdata$headache,by=initialdata$naprox)[,2]
Paracetamol<-table(initialdata$headache,by=initialdata$parac)[,2]
No.Painrelief<-table(initialdata$headache,by=initialdata$No.Painrelief)[,2]


Headache.table<-data.frame(t(cbind(Aspirin,Celecoxib,Diclofenac,Ibuprofen,Naproxen,Paracetamol,No.Painrelief)))
names(Headache.table)<-c("Non-Headache","Headache")
Headache.table$percent<-Headache.table[,2]/(Headache.table[,2]+Headache.table[,1])*100
Headache.table

Non-Headache Headache percent
Aspirin 794 67 7.781649
Celecoxib 53 11 17.187500
Diclofenac 26 4 13.333333
Ibuprofen 224 31 12.156863
Naproxen 166 20 10.752688
Paracetamol 348 52 13.000000
No.Painrelief 439 33 6.991525

chisq.test(Headache.table[,1:2])

Pearson's Chi-squared test

data: Headache.table[, 1:2]
X-squared = 19.22, df = 6, p-value = 0.003807

chisq<-chisq.test(Headache.table[,1:2])


chisq

Pearson's Chi-squared test

data: Headache.table[, 1:2]
X-squared = 19.22, df = 6, p-value = 0.003807

contrib<-100*chisq$residuals^2/chisq$statistic
contrib

Non-Headache Headache
Aspirin 1.6603313 15.613207
Celecoxib 2.1141203 19.880489
Diclofenac 0.2391368 2.248763
Ibuprofen 0.9505996 8.939125
Naproxen 0.1393088 1.310014
Paracetamol 2.6428646 24.852626
No.Painrelief 1.8656315 17.543783

res1 <- cor.mtest(contrib, conf.level = 0.95)

col2 <- colorRampPalette(c("#67001F", "#B2182B", "#D6604D", "#F4A582",
 "#FDDBC7", "#FFFFFF", "#D1E5F0", "#92C5DE",
 "#4393C3", "#2166AC", "#053061"))


pearson<-chisq$residuals


corrplot(contrib, p.mat = res1$p, insig = "blank",pch.col=1,is.cor=F,tl.col=1, cl.pos="b", number.digits=2,number.cex=0.5,cl.cex=1,cl.length=3, cl.lim=c(0,50), col=rev(col2(200)))

Figure description: Percentage contribution to the significant difference in proportions of those with headaches by pain-reliever.


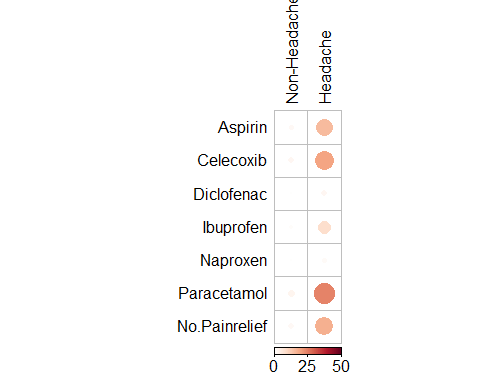


corrplot(pearson, p.mat = res1$p, insig = "blank",pch.col=1,is.cor=F,tl.col=1, cl.pos="b", number.digits=2,number.cex=0.5,cl.cex=1,cl.length=3, cl.lim=c(-8,8),col=rev(col2(200)))

Figure description: Z-scores of the proportions of headache sufferers per pain-reliever.


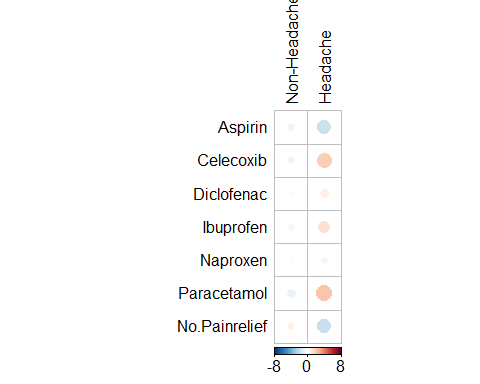


### Cardiovascular disease proportions by pain reliever use

Aspirin<-table(initialdata$vasc,by=initialdata$aspirin)[,2]
Celecoxib<-table(initialdata$vasc,by=initialdata$celex)[,2]
Diclofenac<-table(initialdata$vasc,by=initialdata$diclo)[,2]
Ibuprofen<-table(initialdata$vasc,by=initialdata$Ibu)[,2]
Naproxen<-table(initialdata$vasc,by=initialdata$naprox)[,2]
Paracetamol<-table(initialdata$vasc,by=initialdata$parac)[,2]
No.Painrelief<-table(initialdata$vasc,by=initialdata$No.Painrelief)[,2]

Cardiovascular.Pathology.Table<-data.frame(t(cbind(Aspirin,Celecoxib,Diclofenac,Ibuprofen,Naproxen,Paracetamol,No.Painrelief)))
names(Cardiovascular.Pathology.Table)<-c("Non-cardiovascular","Cardiovascular")
Cardiovascular.Pathology.Table$percent<-Cardiovascular.Pathology.Table[,2]/(Cardiovascular.Pathology.Table[,2]+Cardiovascular.Pathology.Table[,1])*100
Cardiovascular.Pathology.Table

Non-cardiovascular Cardiovascular percent
Aspirin 306 555 64.45993
Celecoxib 20 44 68.75000
Diclofenac 14 16 53.33333
Ibuprofen 97 158 61.96078
Naproxen 72 114 61.29032
Paracetamol 127 273 68.25000
No.Painrelief 211 261 55.29661

chisq<-chisq.test(Cardiovascular.Pathology.Table[,1:2])

chisq

Pearson's Chi-squared test

data: Cardiovascular.Pathology.Table[, 1:2]
X-squared = 19.803, df = 6, p-value = 0.003002

contrib<-100*chisq$residuals^2/chisq$statistic
contrib

Non-cardiovascular Cardiovascular
Aspirin 3.7955384 2.26236526
Celecoxib 3.2154874 1.91662058
Diclofenac 3.5242587 2.10066652
Ibuprofen 0.1658447 0.09885327
Naproxen 0.4679111 0.27890267
Paracetamol 16.9351185 10.09433173
No.Painrelief 34.5501621 20.59393898

res1 <- cor.mtest(contrib, conf.level = 0.95)

col2 <- colorRampPalette(c("#67001F", "#B2182B", "#D6604D", "#F4A582",
 "#FDDBC7", "#FFFFFF", "#D1E5F0", "#92C5DE",
 "#4393C3", "#2166AC", "#053061"))


pearson<-chisq$residuals


corrplot(contrib, p.mat = res1$p, insig = "blank",pch.col=1,is.cor=F,tl.col=1, cl.pos="b", number.digits=2,number.cex=0.5,cl.cex=1,cl.length=3, cl.lim=c(0,50), col=rev(col2(200)))

Figure description: Percentage contribution to the significant difference in proportions of those with evidence of cardiovascular pathology by pain-reliever.


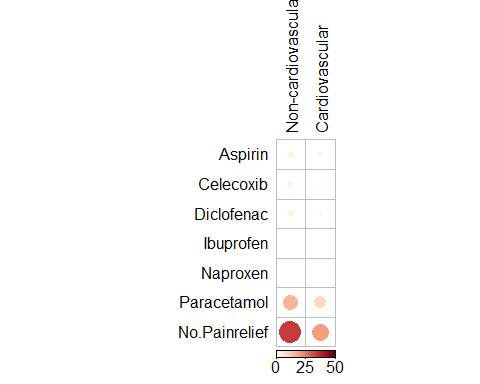


corrplot(pearson, p.mat = res1$p, insig = "blank",pch.col=1,is.cor=F,tl.col=1, cl.pos="b", number.digits=2,number.cex=0.5,cl.cex=1,cl.length=3, cl.lim=c(-8,8),col=rev(col2(200)))

Figure description: Z-scores of the proportions of those with evidence of cardiovascular pathology per pain-reliever.


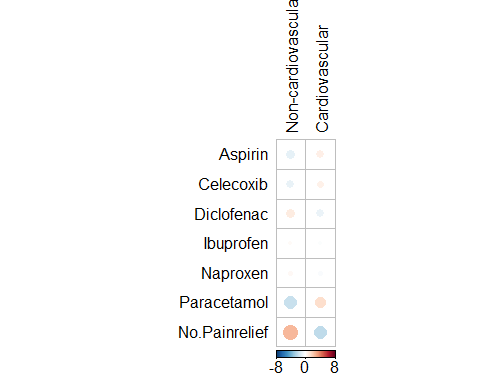


### Diabetes proportions by pain reliever use

Aspirin<-table(initialdata$diab,by=initialdata$aspirin)[,2]
Celecoxib<-table(initialdata$diab,by=initialdata$celex)[,2]
Diclofenac<-table(initialdata$diab,by=initialdata$diclo)[,2]
Ibuprofen<-table(initialdata$diab,by=initialdata$Ibu)[,2]
Naproxen<-table(initialdata$diab,by=initialdata$naprox)[,2]
Paracetamol<-table(initialdata$diab,by=initialdata$parac)[,2]
No.Painrelief<-table(initialdata$headache,by=initialdata$No.Painrelief)[,2]


Diabetes.Pathology.Table<-data.frame(t(cbind(Aspirin,Celecoxib,Diclofenac,Ibuprofen,Naproxen,Paracetamol,No.Painrelief)))
names(Diabetes.Pathology.Table)<-c("Non-Diabetes","Diabetes")
Diabetes.Pathology.Table$percent<-Diabetes.Pathology.Table[,2]/(Cardiovascular.Pathology.Table[,2]+Diabetes.Pathology.Table[,1])*100
Diabetes.Pathology.Table

Non-Diabetes Diabetes percent
Aspirin 769 92 6.948640
Celecoxib 57 7 6.930693
Diclofenac 26 4 9.523810
Ibuprofen 229 26 6.718346
Naproxen 165 21 7.526882
Paracetamol 354 46 7.336523
No.Painrelief 439 33 4.714286

chisq.test(Diabetes.Pathology.Table[,1:2])

Pearson's Chi-squared test

data: Diabetes.Pathology.Table[, 1:2]
X-squared = 6.9006, df = 6, p-value = 0.3301

### Tabling data completeness by NSAID

NSAID.names<-colnames(fulldata[,c(19:24,36)])

date.table<-dcast(data.frame(t(table(fulldata[,"diagn"], by=fulldata$M))), by~Var2)

Using Freq as value column: use value.var to override.

date.table[,NSAID.names]<-NA

date.table$CN.percent<-date.table[,3]/(date.table[,3]+date.table[,4]+date.table[,5]+date.table[,6])*100
date.table$EMCI.percent<-date.table[,4]/(date.table[,3]+date.table[,4]+date.table[,5]+date.table[,6])*100
date.table$LMCI.percent<-date.table[,5]/(date.table[,3]+date.table[,4]+date.table[,5]+date.table[,6])*100
date.table$AD.percent<-date.table[,6]/(date.table[,3]+date.table[,4]+date.table[,5]+date.table[,6])*100

for(i in 1:length(NSAID.names)){

NSAID.i<-NSAID.names[i]

M.table<-data.frame(t(table(fulldata[,NSAID.i], by=fulldata$M)))
M.table<-dcast(M.table, by~Var2)
M.table$percent<-M.table[,3]/(M.table[,2]+M.table[,3])*100
date.table[,NSAID.i]<-M.table$percent
}

Using Freq as value column: use value.var to override.
Using Freq as value column: use value.var to override.
Using Freq as value column: use value.var to override.
Using Freq as value column: use value.var to override.
Using Freq as value column: use value.var to override.
Using Freq as value column: use value.var to override.
Using Freq as value column: use value.var to override.

date.table<-date.table[,c(-2)]

col.names<-c("Months", "CN Number", 'EMCI Number', "LMCI Number", "AD Number", "Aspirin Percent", "Paracetamol Percent", "Diclofenac Percent", "Ibuprofen Percent", "Naproxen Percent", "Celecoxib Percent", "No Painrelief Percent", "CN Percent", "EMCI Percent", "LMCI Percent", "AD Percent")

colnames(date.table)<-col.names

date.table

Months CN Number EMCI Number LMCI Number AD Number Aspirin Percent
1 0 415 306 564 334 53.18098
2 3 173 290 152 119 55.44959
3 6 400 275 542 307 53.67454
4 12 387 283 513 272 54.70790
5 18 375 229 465 224 56.68987
6 24 363 254 442 180 56.98144
7 30 253 142 312 43 58.80000
8 36 221 222 373 26 58.55107
9 42 107 32 147 22 62.98701
10 48 236 185 233 12 63.36336
11 54 84 0 114 3 67.16418
12 60 139 112 164 6 66.03325
13 66 103 1 113 0 73.73272
14 72 189 61 146 1 71.03275
15 78 105 0 108 0 73.70892
16 84 104 23 98 0 75.55556
17 90 73 0 56 0 77.51938
18 96 74 0 81 0 78.06452
19 102 5 0 2 0 100.00000
20 108 61 0 58 0 79.83193
21 114 0 0 2 0 50.00000
22 120 49 0 33 0 76.82927
23 126 3 0 1 0 75.00000
24 132 42 0 15 0 84.21053
25 144 13 0 4 0 76.47059
 Paracetamol Percent Diclofenac Percent Ibuprofen Percent Naproxen Percent
1 24.70661 1.852996 15.75046 11.48857
2 24.79564 1.907357 17.98365 13.76022
3 25.85302 1.837270 16.20735 11.87664
4 26.11684 1.924399 16.70103 12.23368
5 27.30085 2.088167 17.16937 12.68368
6 27.84504 2.017756 17.35270 12.75222
7 31.20000 2.266667 18.66667 13.33333
8 29.21615 2.256532 18.88361 14.25178
9 34.74026 3.246753 19.15584 13.96104
10 33.93393 2.852853 22.22222 15.16517
11 41.29353 2.985075 22.38806 14.42786
12 36.57957 2.612827 22.56532 17.57720
13 38.70968 2.764977 22.58065 16.12903
14 37.53149 3.022670 24.43325 17.38035
15 39.90610 4.225352 24.41315 16.90141
16 41.33333 4.444444 26.22222 18.66667
17 37.98450 3.875969 25.58140 18.60465
18 43.87097 5.161290 27.74194 18.06452
19 14.28571 0.000000 42.85714 28.57143
20 41.17647 4.201681 26.89076 18.48739
21 50.00000 0.000000 0.00000 0.00000
22 41.46341 8.536585 20.73171 20.73171
23 0.00000 0.000000 50.00000 25.00000
24 47.36842 8.771930 29.82456 22.80702
25 35.29412 5.882353 29.41176 23.52941
 Celecoxib Percent No Painrelief Percent CN Percent EMCI Percent LMCI Percent
1 3.953057 29.153799 25.63311 18.9005559 34.83632
2 3.814714 24.386921 23.56948 39.5095368 20.70845
3 4.002625 28.083990 26.24672 18.0446194 35.56430
4 4.123711 26.872852 26.59794 19.4501718 35.25773
5 3.944316 25.135344 29.00232 17.7107502 35.96288
6 3.954802 25.020178 29.29782 20.5004036 35.67393
7 3.733333 23.600000 33.73333 18.9333333 41.60000
8 4.631829 22.565321 26.24703 26.3657957 44.29929
9 3.896104 21.103896 34.74026 10.3896104 47.72727
10 4.804805 18.468468 35.43544 27.7777778 34.98498
11 5.970149 16.417910 41.79104 0.0000000 56.71642
12 5.700713 17.102138 33.01663 26.6033254 38.95487
13 6.451613 14.285714 47.46544 0.4608295 52.07373
14 6.801008 11.838791 47.60705 15.3652393 36.77582
15 5.633803 10.798122 49.29577 0.0000000 50.70423
16 5.333333 9.333333 46.22222 10.2222222 43.55556
17 6.976744 10.077519 56.58915 0.0000000 43.41085
18 7.096774 6.451613 47.74194 0.0000000 52.25806
19 0.000000 0.000000 71.42857 0.0000000 28.57143
20 8.403361 7.563025 51.26050 0.0000000 48.73950
21 0.000000 50.000000 0.00000 0.0000000 100.00000
22 6.097561 7.317073 59.75610 0.0000000 40.24390
23 0.000000 0.000000 75.00000 0.0000000 25.00000
24 7.017544 5.263158 73.68421 0.0000000 26.31579
25 5.882353 17.647059 76.47059 0.0000000 23.52941
 AD Percent
1 20.6300185
2 16.2125341
3 20.1443570
4 18.6941581
5 17.3240526
6 14.5278450
7 5.7333333
8 3.0878860
9 7.1428571
10 1.8018018
11 1.4925373
12 1.4251781
13 0.0000000
14 0.2518892
15 0.0000000
16 0.0000000
17 0.0000000
18 0.0000000
19 0.0000000
20 0.0000000
21 0.0000000
22 0.0000000
23 0.0000000
24 0.0000000
25 0.0000000

######

# Analysis of cognitive decline using the MMSE score

Mini-mental state examination (MMSE) is a cognitive assessment with a focus on memory that is often used as the primary measure of Alzheimer’s disease progression in clinical trials. It is a score out of 30 with higher scores corresponding to better cognitive performance.

## Dependent variable check

Rows with no MMSE score were removed and variables were checked for correct categorization.

data<-read.csv("CleanedFinalData.csv", header=T)
MMSEdata<-data[!is.na(data$MMSE),]
MMSEdata$ID<-as.factor(MMSEdata$ID)
MMSEdata$APOE4<-as.factor(MMSEdata$APOE4)

## Generation of dependent variables appropriate for different distributions

MMSEdata$neg.b.MMSE<-round(30-MMSEdata$MMSE)
MMSEdata$fail<-abs(30-MMSEdata$MMSE)
MMSEdata$success<-30-MMSEdata$fail
MMSEdata$MMSEscore<-rep(30,length(MMSEdata$neg.b.MMSE))
MMSEdata$proportion<-MMSEdata$fail/MMSEdata$MMSEscore

## Transformation to obtain normal approximation

The total errors were then generated by constructing a linear model of with MMSE as the dependent variable and patient ID as the explanatory variable. It has been shown that if the total errors are homoscedastic and normal then the multi-level linear model errors will likely also be homoscedastic and normal distributed (Gurka *et al.* 2006). Therefore, total errors are an excellent starting point for model diagnostics. From this it was found that even with BoxCox optimized transformation the residuals while normally distributed fail to have even homoscedasticity. This is due to the categorical nature of the MMSE score and the high number of zero values. Therefore, a generalized linear model utilizing a non-gaussian distribution were performed

qqp(MMSEdata$MMSE[MMSEdata$M==0], dist="norm")

Figure description: Plot of MMSE scores against quantile with expected normal distribution overlay (blue) .


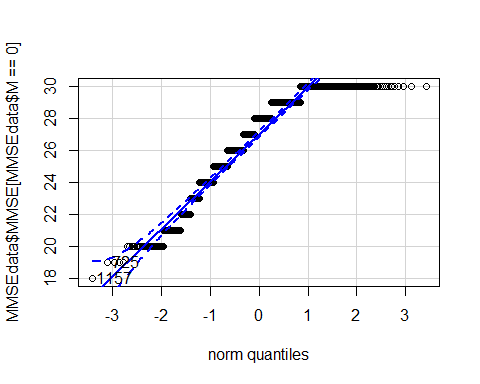


[1] 1157 725

m1<-lm((MMSE+1)~ID, data=MMSEdata)
par(mfrow=c(2,2))
plot(m1)

Figure description: Residual distribution plots for evaluating the assumptions of the least-square methods.


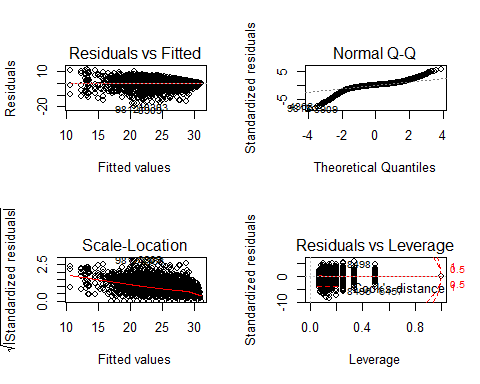


boxcox<-boxcox(m1,lambda = seq(-5, 5, 1/100),plotit = TRUE )
selectedlambda<-boxcox$x[boxcox$y==max(boxcox$y)]
selectedlambda

[1] 4.83

MMSEdata$tMMSE<-(MMSEdata$MMSE)^selectedlambda
m1.t<-lmer(tMMSE~diagn+edu.cat+Gender+APOE4+M+(1|ID),data=MMSEdata)
summary(m1.t)

Linear mixed model fit by REML. t-tests use Satterthwaite's method [
lmerModLmerTest]
Formula: tMMSE ~ diagn + edu.cat + Gender + APOE4 + M + (1 | ID)
 Data: MMSEdata

REML criterion at convergence: 286545.4

Scaled residuals:
 Min 1Q Median 3Q Max
-4.5078 -0.6006 0.0264 0.6219 3.6068

Random effects:
 Groups Name Variance Std.Dev.
 ID (Intercept) 4.469e+12 2113945
 Residual 4.556e+12 2134557
Number of obs: 8878, groups: ID, 1619

Fixed effects:
 Estimate Std. Error df t value Pr(>|t|)
(Intercept) 13018776 159085 1681 81.835 < 2e-16 ***
diagn2EMCI -1345340 176612 1585 -7.618 4.42e-14 ***
diagn3LMCI -3879497 154816 1568 -25.059 < 2e-16 ***
diagn4AD -7930905 185780 1801 -42.690 < 2e-16 ***
edu.cat2tertiary -331073 147570 1624 -2.244 0.025 *
edu.cat3mid -966745 167226 1624 -5.781 8.88e-09 ***
edu.cat4early -1099664 177772 1666 -6.186 7.76e-10 ***
GenderMale -192114 120445 1638 -1.595 0.111
APOE41 -756413 128778 1638 -5.874 5.15e-09 ***
APOE42 -1066035 205088 1672 -5.198 2.26e-07 ***
M -28174 952 922964 -29.595 < 2e-16 ***
---
Signif. codes: 0 '***' 0.001 '**' 0.01 '*' 0.05 '.' 0.1 ' ' 1

Correlation of Fixed Effects:
 (Intr) d2EMCI d3LMCI dgn4AD ed.ct2 ed.ct3 ed.ct4 GndrMl APOE41
diagn2EMCI -0.419
diagn3LMCI -0.450 0.500
diagn4AD -0.349 0.430 0.524
ed.ct2trtry -0.416 0.013 -0.009 -0.043
edu.cat3mid -0.436 -0.025 0.025 -0.038 0.404
edu.cat4rly -0.345 -0.036 -0.069 -0.128 0.386 0.357
GenderMale -0.452 -0.043 -0.081 -0.047 0.049 0.175 0.142
APOE41 -0.192 -0.094 -0.178 -0.207 -0.032 -0.032 -0.048 0.001
APOE42 -0.070 -0.077 -0.178 -0.235 -0.046 -0.029 -0.021 -0.012 0.306
M -0.165 0.034 0.037 0.097 0.000 -0.003 0.003 -0.004 0.009
 APOE42
diagn2EMCI
diagn3LMCI
diagn4AD
ed.ct2trtry
edu.cat3mid
edu.cat4rly
GenderMale
APOE41
APOE42
M 0.007

mcp.fnc(m1.t)

Figure description: Box Cox plot of log-likelihood verse lambda showing the maximum is achieved at 4.8


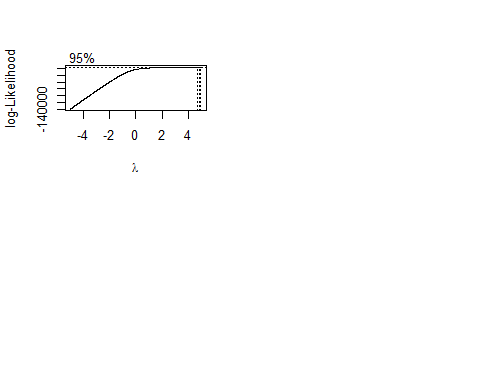


Figure description: Residual distribution plots for evaluating the assumptions of the least-square methods. Normality close to being achieved, however, heteroskedasticity is still an issue due the discrete nature of the data.


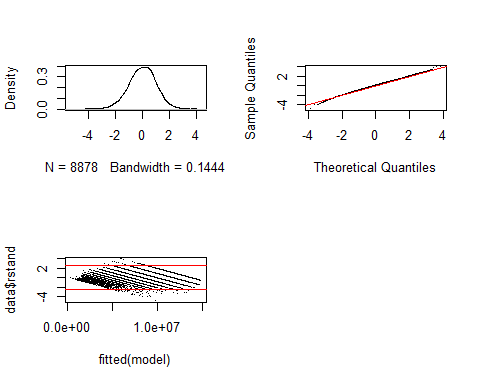


qqp(MMSEdata$tMMSE[MMSEdata$M==0], dist="norm")

Figure description: Plot of MMSE scores against quantile with expected normal distribution overlay (blue) .


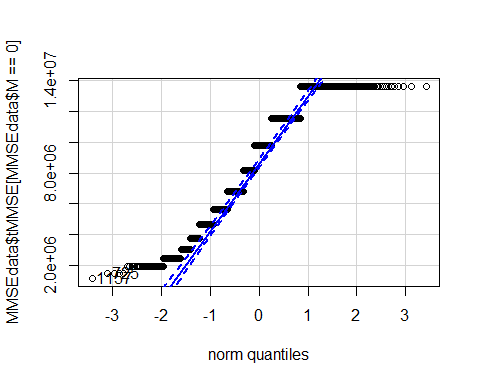


[1] 1157 725

## Selecting non-gaussian model

The data was plotted against expected distributions including normal, Poisson, negative binomial, binomial, exponential or gamma distributions within each time point. Some transformations were required including converting the MMSE score to the count of mistakes (30-MMSE) for the Poisson, negative binomial and exponential distributions. For the Poisson distribution there was overdispersion as seen by the vast difference in mean and variance, therefore, Poisson is not likely. This is also evident in the dependent variable not following a Poisson distribution. The binomial model seems appropriate theoretically as the MMSE could be considered 30 trials with a proportion of failures occurring. However, the large number of zeros makes logistic regression of the binomial distribution inappropriate. The negative binomial and gamma models appear to be the most appropriate models. However, given the categorical nature of the data the negative binomial model was chosen for analysis. This is an approximate method for distribution selection as it does not consider the explanatory variables effects on the distribution. This was approached used as a starting point for model analysis. Distributions were compared once the final model was established and the negative binomial model proved to be the most appropriate. Furthermore, residual analysis of the final selected model confirmed the appropriateness of the negative binomial model.

hist(MMSEdata$MMSE[MMSEdata$M==0])

Figure description: Plot of the frequency of MMSE scores at month 0.


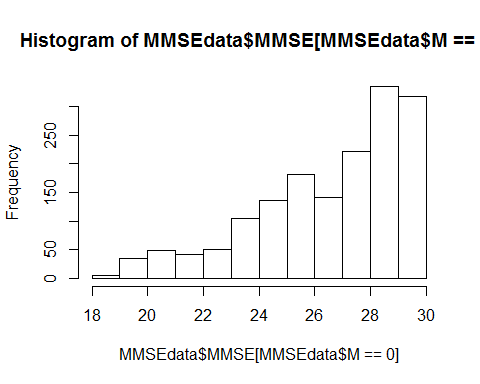


hist(MMSEdata$MMSE[MMSEdata$M==12])

Figure description: Plot of the frequency of MMSE scores at month 12.


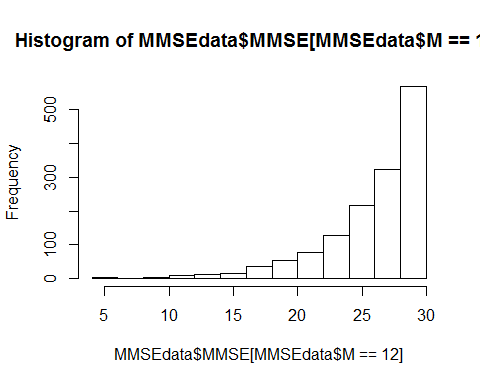


hist(MMSEdata$MMSE[MMSEdata$M==24])

Figure description: Plot of the frequency of MMSE scores at month 24


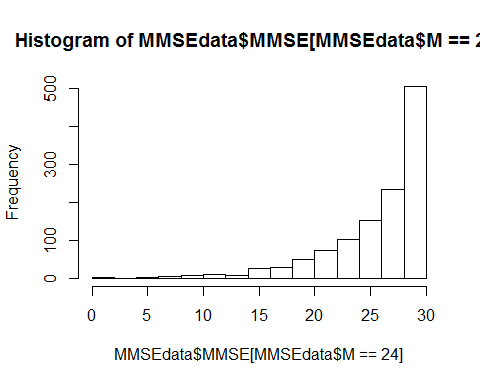


hist(MMSEdata$MMSE[MMSEdata$M==48])

Figure description: Plot of the frequency of MMSE scores at month 48.


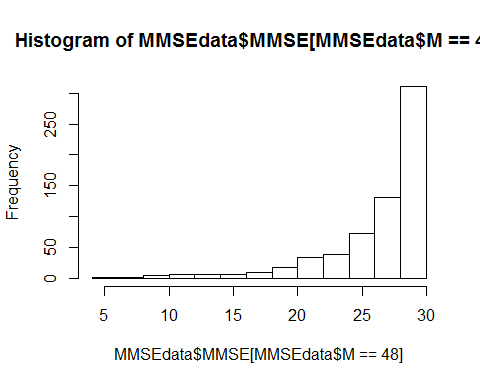


hist(MMSEdata$MMSE[MMSEdata$M==72])

Figure description: Plot of the frequency of MMSE scores at month 72


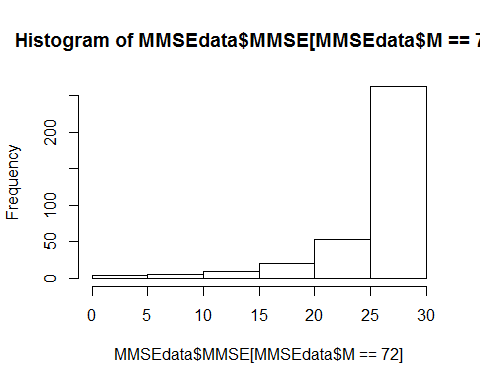


hist(MMSEdata$MMSE[MMSEdata$M==120])

Figure description: Plot of the frequency of MMSE scores at month 120


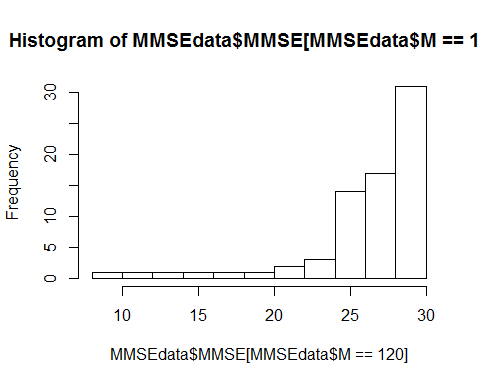


qqp(MMSEdata$MMSE[MMSEdata$M==0], "norm", main="Normal distribution model Month=0")

Figure description: Plot of MMSE scores against quantile with expected normal distribution overlay (blue) , month 0.


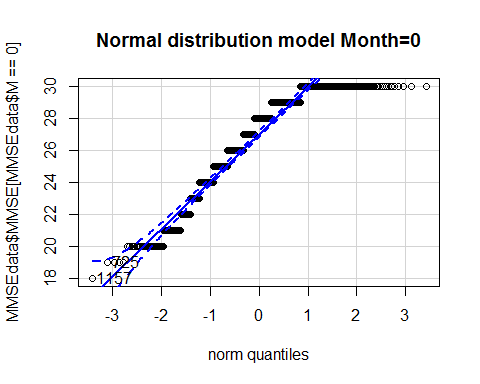


[1] 1157 725

qqp(MMSEdata$MMSE[MMSEdata$M==12], "norm", main="Normal distribution model Month=12")

Figure description: Plot of MMSE scores against quantile with expected normal distribution overlay (blue) , month 12.


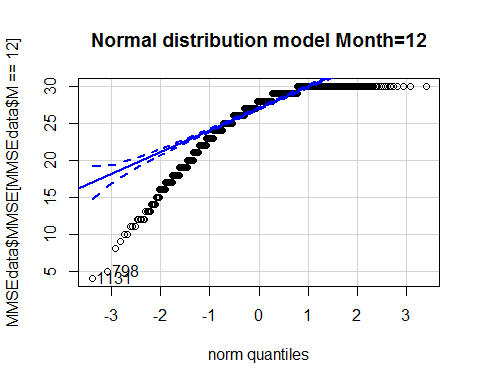


[1] 1131 798

qqp(MMSEdata$MMSE[MMSEdata$M==24], "norm", main="Normal distribution model Month=24")

Figure description: Plot of MMSE scores against quantile with expected normal distribution overlay (blue) , month 24


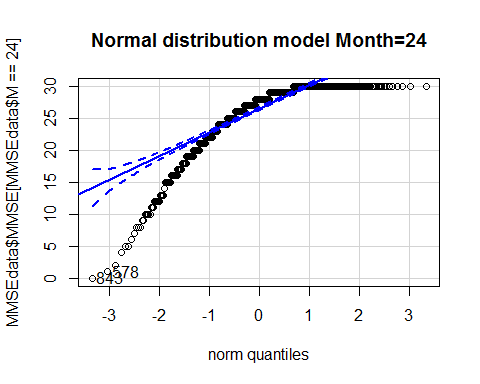


[1] 843 578

qqp(MMSEdata$MMSE[MMSEdata$M==48], "norm", main="Normal distribution model Month=48")

Figure description: Plot of MMSE scores against quantile with expected normal distribution overlay (blue) , month 48.


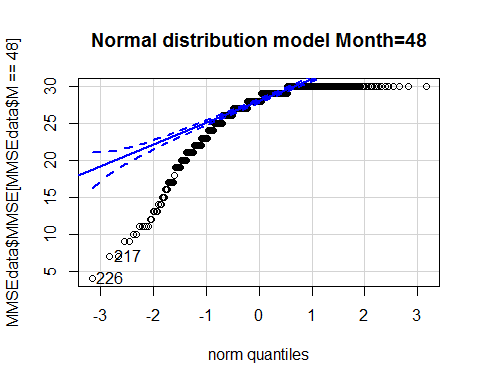


[1] 226 217

qqp(MMSEdata$MMSE[MMSEdata$M==72], "norm", main="Normal distribution model Month=72")


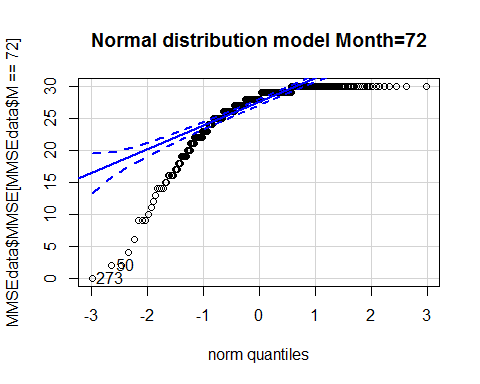


[1] 273 50

qqp(MMSEdata$MMSE[MMSEdata$M==120], "norm", main="Normal distribution model Month=120")

Figure description: Plot of MMSE scores against quantile with expected normal distribution overlay (blue) , month 120.


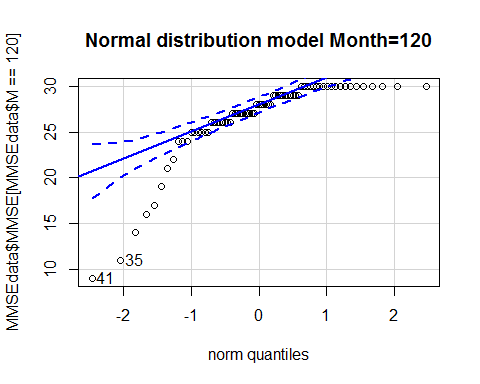


[1] 41 35

hist(MMSEdata$neg.b.MMSE[MMSEdata$M==0])

Figure description: Frequency plot of MMSE failures with month 0.


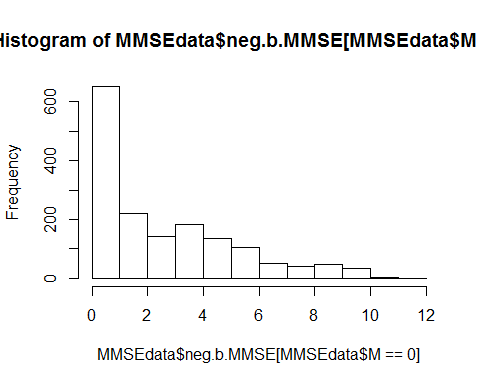


hist(MMSEdata$neg.b.MMSE[MMSEdata$M==12])

Figure description: Frequency plot of MMSE failures with month 12.


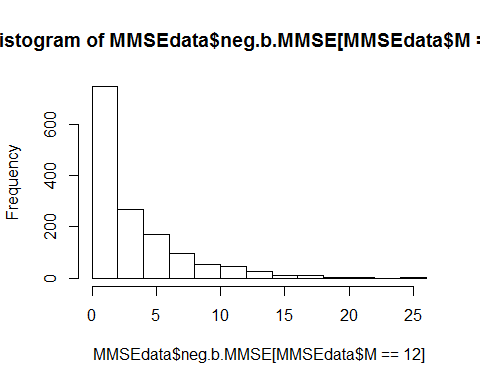


hist(MMSEdata$neg.b.MMSE[MMSEdata$M==24])

Figure description: Frequency plot of MMSE failures with month 24.


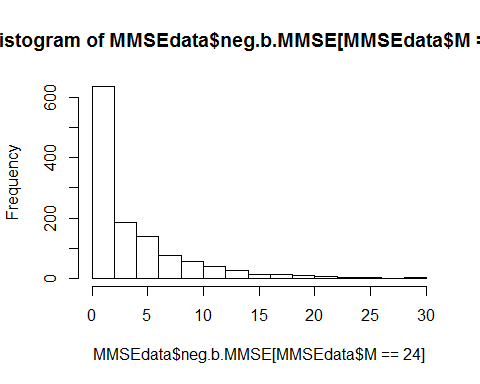


hist(MMSEdata$neg.b.MMSE[MMSEdata$M==48])

Figure description: Frequency plot of MMSE failures with month 48.


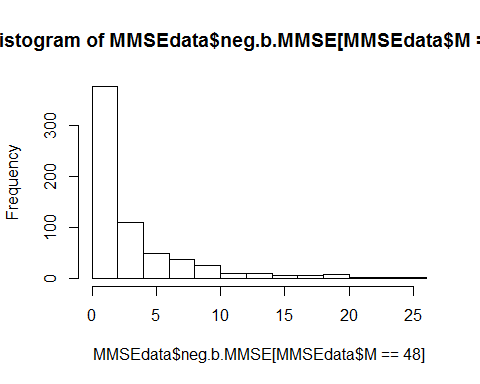


hist(MMSEdata$neg.b.MMSE[MMSEdata$M==72])

Figure description: Frequency plot of MMSE failures with month 72.


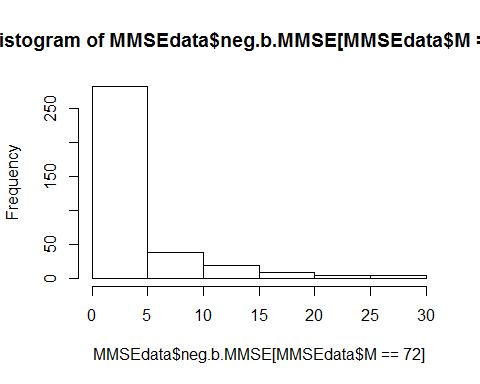


hist(MMSEdata$neg.b.MMSE[MMSEdata$M==120])

Figure description: Frequency plot of MMSE failures with month 120.


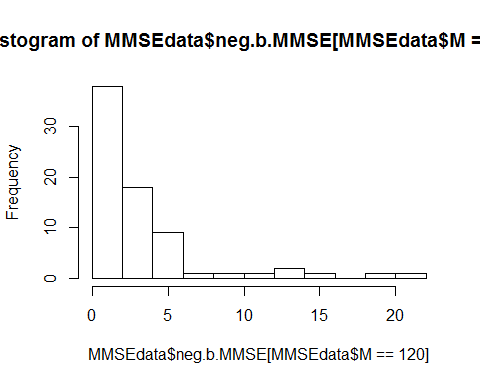


nbinom<-fitdistr(MMSEdata$neg.b.MMSE[MMSEdata$M==0], "negative binomial")
qqp(MMSEdata$neg.b.MMSE[MMSEdata$M==0], "nbinom", size=nbinom$estimate[[1]], mu=nbinom$estimate[[2]], main="Negative binomial model Month=0")

Figure description: Plot of MMSE scores against negative binomial quantile with expected negative binomial distribution overlay (blue) , month 0.


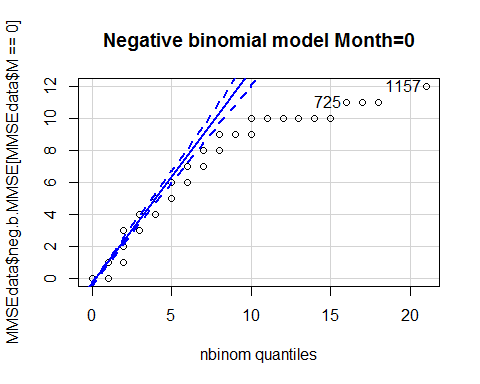


[1] 1157 725

nbinom<-fitdistr(MMSEdata$neg.b.MMSE[MMSEdata$M==12], "negative binomial")
qqp(MMSEdata$neg.b.MMSE[MMSEdata$M==12], "nbinom", size=nbinom$estimate[[1]], mu=nbinom$estimate[[2]], main="Negative binomial model Month=12")

Figure description: Plot of MMSE scores against negative binomial quantile with expected negative binomial distribution overlay (blue) , month 12.


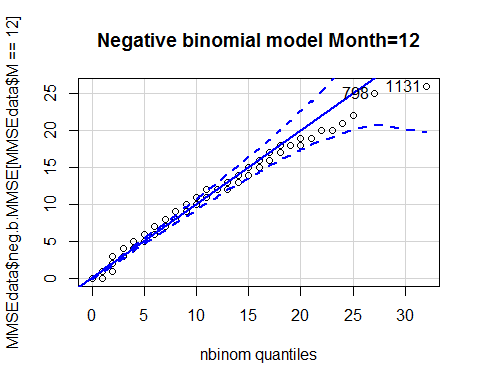


[1] 1131 798

nbinom<-fitdistr(MMSEdata$neg.b.MMSE[MMSEdata$M==24], "negative binomial")
qqp(MMSEdata$neg.b.MMSE[MMSEdata$M==24], "nbinom", size=nbinom$estimate[[1]], mu=nbinom$estimate[[2]], main="Negative binomial model Month=24")

Figure description: Plot of MMSE scores against negative binomial quantile with expected negative binomial distribution overlay (blue) , month 24.


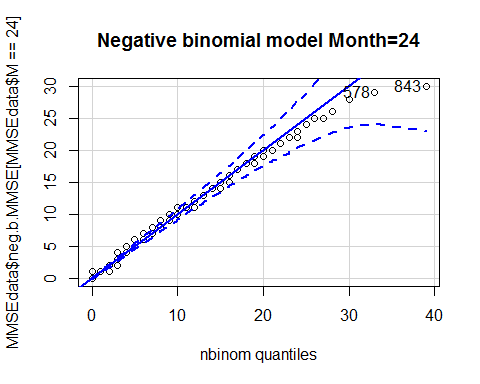


[1] 843 578

nbinom<-fitdistr(MMSEdata$neg.b.MMSE[MMSEdata$M==48], "negative binomial")
qqp(MMSEdata$neg.b.MMSE[MMSEdata$M==48], "nbinom", size=nbinom$estimate[[1]], mu=nbinom$estimate[[2]], main="Negative binomial model Month=48")

Figure description: Plot of MMSE scores against negative binomial quantile with expected negative binomial distribution overlay (blue) , month 48.


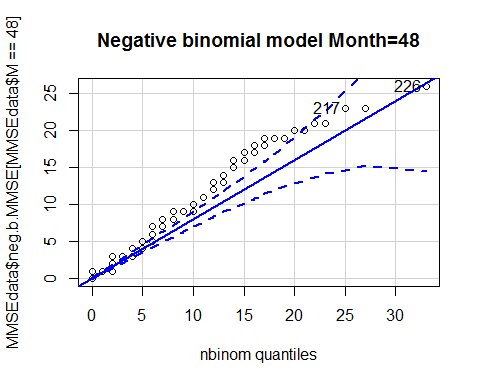


[1] 226 217

nbinom<-fitdistr(MMSEdata$neg.b.MMSE[MMSEdata$M==72], "negative binomial")
qqp(MMSEdata$neg.b.MMSE[MMSEdata$M==72], "nbinom", size=nbinom$estimate[[1]], mu=nbinom$estimate[[2]], main="Negative binomial model Month=72")

Figure description: Plot of MMSE scores against negative binomial quantile with expected negative binomial distribution overlay (blue) , month 72.


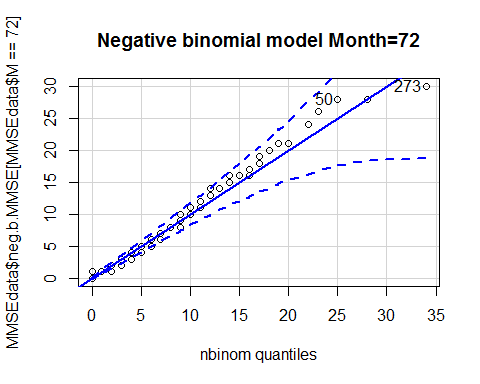


[1] 273 50

nbinom<-fitdistr(MMSEdata$neg.b.MMSE[MMSEdata$M==120], "negative binomial")
qqp(MMSEdata$neg.b.MMSE[MMSEdata$M==120], "nbinom", size=nbinom$estimate[[1]], mu=nbinom$estimate[[2]], main="Negative binomial model Month=120")

Figure description: Plot of MMSE scores against negative binomial quantile with expected negative binomial distribution overlay (blue) , month 120.


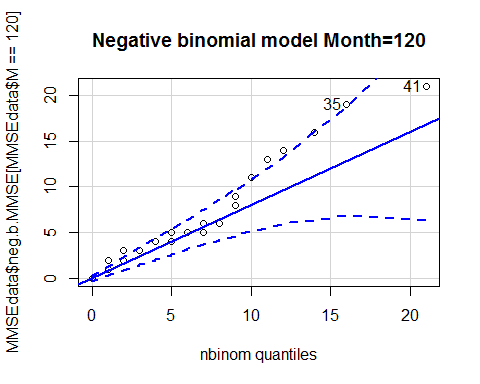


[1] 41 35

poisson <- fitdistr(MMSEdata$neg.b.MMSE[MMSEdata$M==0]+1, "Poisson")
qqp(MMSEdata$neg.b.MMSE, "pois", lambda=poisson$estimate, main="Poisson model Month=0")

Figure description: Plot of MMSE scores against Poisson quantile with expected Poisson distribution overlay (blue) , month 0.


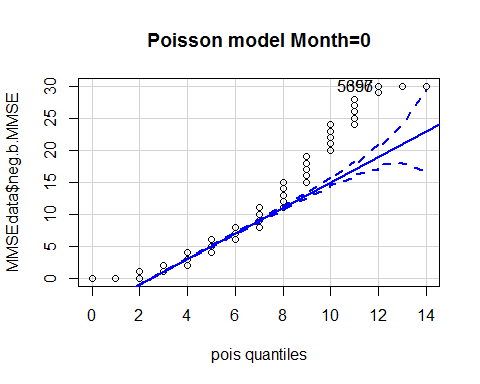


[1] 5357 5696

poisson <- fitdistr(MMSEdata$neg.b.MMSE[MMSEdata$M==12], "Poisson")
qqp(MMSEdata$neg.b.MMSE, "pois", lambda=poisson$estimate, main="Poisson model Month=12")

Figure description: Plot of MMSE scores against Poisson quantile with expected Poisson distribution overlay (blue) , month 12.


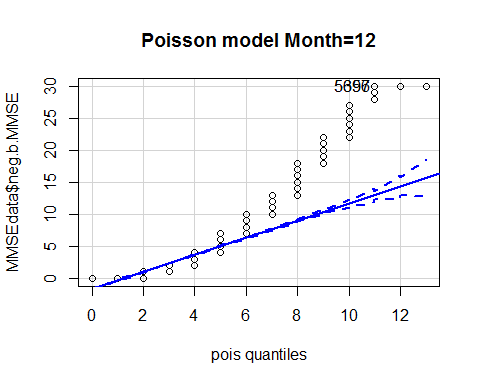


[1] 5357 5696

poisson <- fitdistr(MMSEdata$neg.b.MMSE[MMSEdata$M==24], "Poisson")
qqp(MMSEdata$neg.b.MMSE, "pois", lambda=poisson$estimate, main="Poisson model Month=24")

Figure description: Plot of MMSE scores against Poisson quantile with expected Poisson distribution overlay (blue) , month 24.


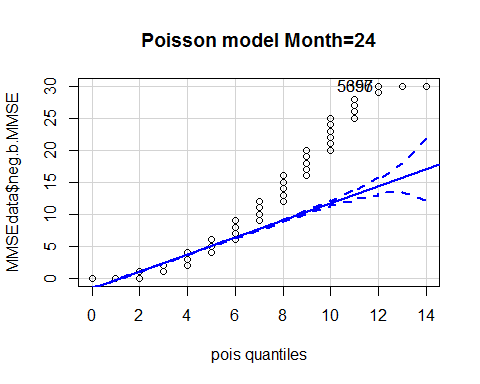


[1] 5357 5696

poisson <- fitdistr(MMSEdata$neg.b.MMSE[MMSEdata$M==48], "Poisson")
qqp(MMSEdata$neg.b.MMSE, "pois", lambda=poisson$estimate, main="Poisson model Month=48")

Figure description: Plot of MMSE scores against Poisson quantile with expected Poisson distribution overlay (blue) , month 48.


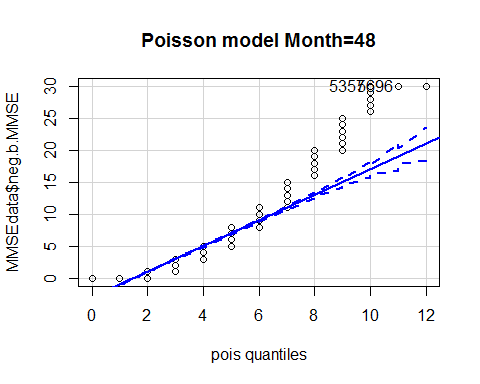


[1] 5357 5696

poisson <- fitdistr(MMSEdata$neg.b.MMSE[MMSEdata$M==72], "Poisson")
qqp(MMSEdata$neg.b.MMSE, "pois", lambda=poisson$estimate, main="Poisson model Month=72")

Figure description: Plot of MMSE scores against Poisson quantile with expected Poisson distribution overlay (blue) , month 72.


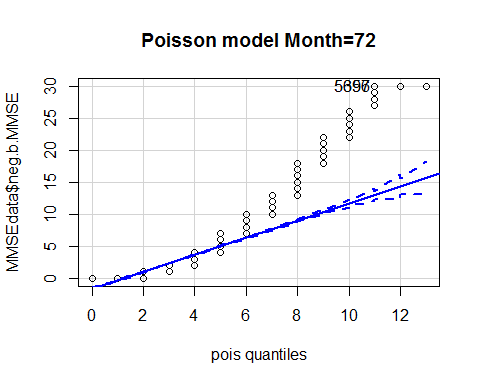


[1] 5357 5696

poisson <- fitdistr(MMSEdata$neg.b.MMSE[MMSEdata$M==120], "Poisson")
qqp(MMSEdata$neg.b.MMSE, "pois", lambda=poisson$estimate, main="Poisson model Month=120")

Figure description: Plot of MMSE scores against Poisson quantile with expected Poisson distribution overlay (blue) , month 120.


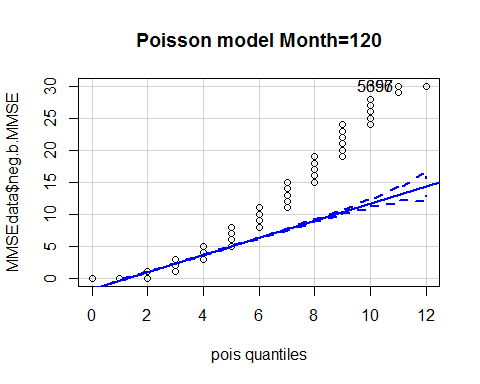


[1] 5357 5696

mean(MMSEdata$neg.b.MMSE[MMSEdata$M==0])

[1] 2.943175

var(MMSEdata$neg.b.MMSE[MMSEdata$M==0])

[1] 7.247696

mean(MMSEdata$neg.b.MMSE[MMSEdata$M==12])

[1] 3.579349

var(MMSEdata$neg.b.MMSE[MMSEdata$M==12])

[1] 15.47411

mean(MMSEdata$neg.b.MMSE[MMSEdata$M==24])

[1] 3.897266

var(MMSEdata$neg.b.MMSE[MMSEdata$M==24])

[1] 21.89491

mean(MMSEdata$neg.b.MMSE[MMSEdata$M==48])

[1] 3.273011

var(MMSEdata$neg.b.MMSE[MMSEdata$M==48])

[1] 18.91441

mean(MMSEdata$neg.b.MMSE[MMSEdata$M==72])

[1] 3.63662

var(MMSEdata$neg.b.MMSE[MMSEdata$M==72])

[1] 26.68962

mean(MMSEdata$neg.b.MMSE[MMSEdata$M==120])

[1] 3.369863

var(MMSEdata$neg.b.MMSE[MMSEdata$M==120])

[1] 19.09741

gamma <- fitdistr((MMSEdata$neg.b.MMSE[MMSEdata$M==0]+1), "gamma")
qqp(MMSEdata$neg.b.MMSE[MMSEdata$M==0]+1, "gamma", shape = gamma$estimate[[1]], rate = gamma$estimate[[2]], main="Gamma model Month=0")

Figure description: Plot of MMSE scores against gamma quantile with expected gamma distribution overlay (blue) , month 0.


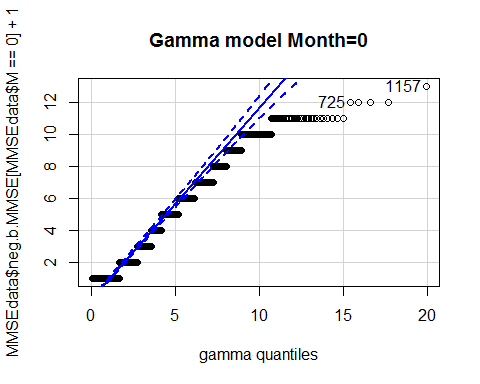


[1] 1157 725

gamma <- fitdistr((MMSEdata$neg.b.MMSE[MMSEdata$M==12]+1), "gamma")
qqp(MMSEdata$neg.b.MMSE[MMSEdata$M==12]+1, "gamma", shape = gamma$estimate[[1]], rate = gamma$estimate[[2]], main="Gamma model Month=12")

Figure description: Plot of MMSE scores against gamma quantile with expected gamma distribution overlay (blue) , month 12.


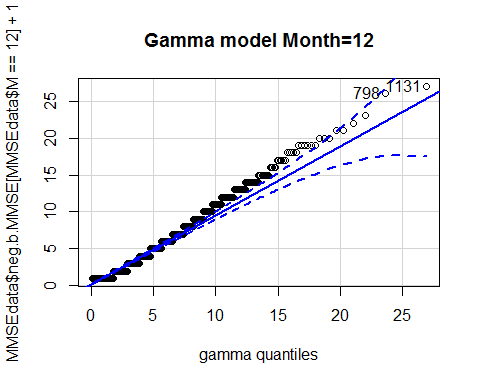


[1] 1131 798

gamma <- fitdistr((MMSEdata$neg.b.MMSE[MMSEdata$M==24]+1), "gamma")
qqp(MMSEdata$neg.b.MMSE[MMSEdata$M==24]+1, "gamma", shape = gamma$estimate[[1]], rate = gamma$estimate[[2]], main="Gamma model Month=24")

Figure description: Plot of MMSE scores against gamma quantile with expected gamma distribution overlay (blue) , month 24.


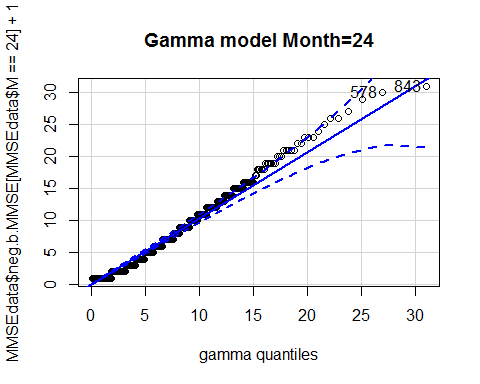


[1] 843 578

gamma <- fitdistr((MMSEdata$neg.b.MMSE[MMSEdata$M==48]+1), "gamma")
qqp(MMSEdata$neg.b.MMSE[MMSEdata$M==48]+1, "gamma", shape = gamma$estimate[[1]], rate = gamma$estimate[[2]], main="Gamma model Month=48")

Figure description: Plot of MMSE scores against gamma quantile with expected gamma distribution overlay (blue) , month 48.


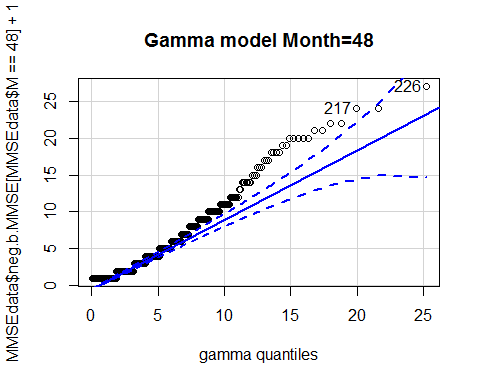


[1] 226 217

gamma <- fitdistr((MMSEdata$neg.b.MMSE[MMSEdata$M==72]+1), "gamma")
qqp(MMSEdata$neg.b.MMSE[MMSEdata$M==72]+1, "gamma", shape = gamma$estimate[[1]], rate = gamma$estimate[[2]], main="Gamma model Month=72")

Figure description: Plot of MMSE scores against gamma quantile with expected gamma distribution overlay (blue) , month 72.


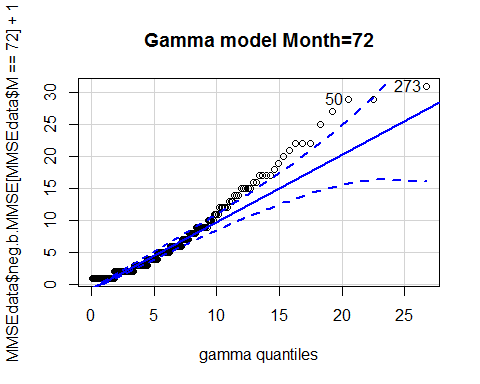


[1] 273 50

gamma <- fitdistr((MMSEdata$neg.b.MMSE[MMSEdata$M==120]+1), "gamma")
qqp(MMSEdata$neg.b.MMSE[MMSEdata$M==120]+1, "gamma", shape = gamma$estimate[[1]], rate = gamma$estimate[[2]], main="Gamma model Month=120")

Figure description: Plot of MMSE scores against gamma quantile with expected gamma distribution overlay (blue) , month 120.


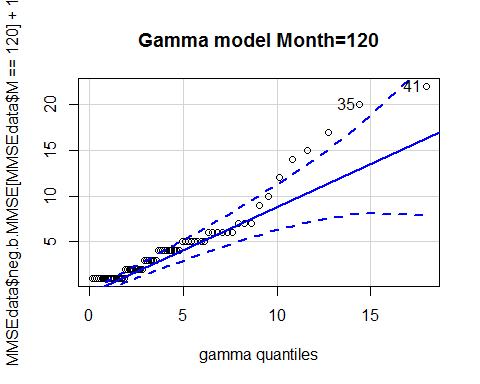


[1] 41 35

par(mar = c(3,3,3,3))
hist(MMSEdata$proportion)


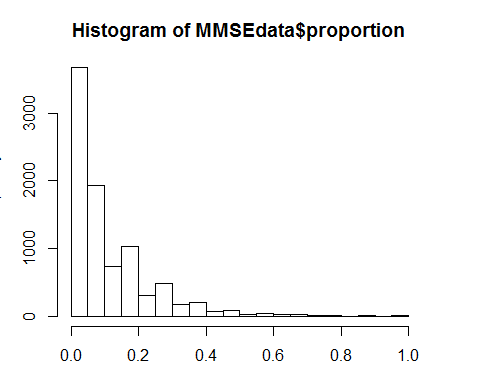


data<-MMSEdata$proportion[MMSEdata$M==0]
params<-fitdistr(data, "logistic")
qqp(data, dist="logis", params$estimate[[1]]);title(main="Logistic model Month=0", line=2)

Figure description: Plot of MMSE scores against logistic quantile with expected logistic distribution overlay (blue) , month 0.


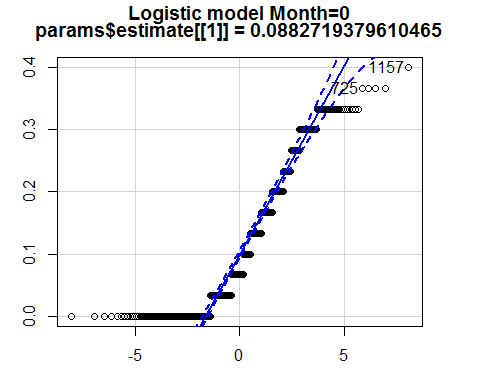


data<-MMSEdata$proportion[MMSEdata$M==12]
params<-fitdistr(data, "logistic")
qqp(data, dist="logis", params$estimate[[1]]);title(main="Logistic model Month=12", line=2)

Figure description: Plot of MMSE scores against logistic quantile with expected logistic distribution overlay (blue) , month 12.


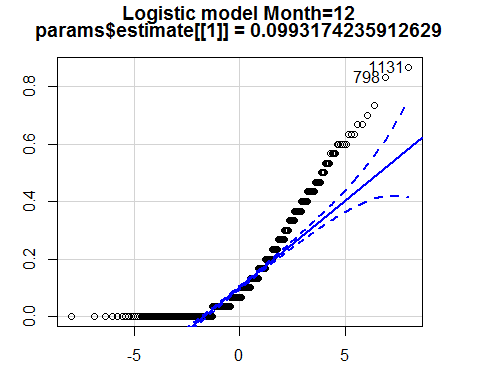


data<-MMSEdata$proportion[MMSEdata$M==24]
params<-fitdistr(data, "logistic")
qqp(data, dist="logis", params$estimate[[1]]);title(main="Logistic model Month=24", line=2)

Figure description: Plot of MMSE scores against logistic quantile with expected logistic distribution overlay (blue) , month 24.


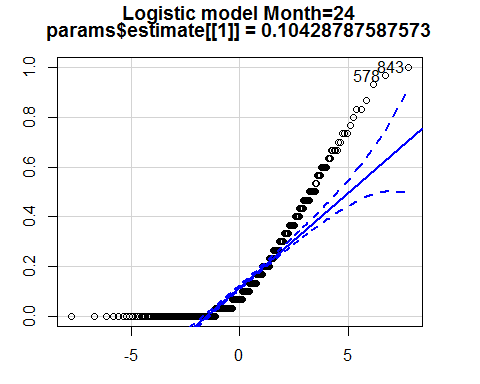


data<-MMSEdata$proportion[MMSEdata$M==48]
params<-fitdistr(data, "logistic")
qqp(data, dist="logis", params$estimate[[1]]);title(main="Logistic model Month=48", line=2)

Figure description: Plot of MMSE scores against logistic quantile with expected logistic distribution overlay (blue) , month 48.


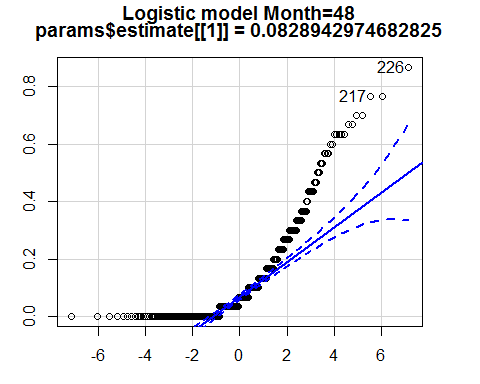


data<-MMSEdata$proportion[MMSEdata$M==72]
params<-fitdistr(data, "logistic")
qqp(data, dist="logis", params$estimate[[1]]);title(main="Logistic model Month=72", line=2)

Figure description: Plot of MMSE scores against logistic quantile with expected logistic distribution overlay (blue) , month 72.


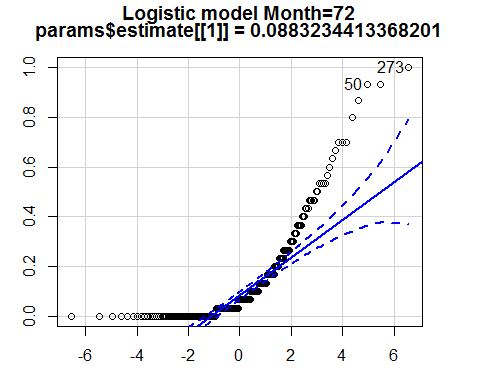


data<-MMSEdata$proportion[MMSEdata$M==120]
params<-fitdistr(data, "logistic")
qqp(data, dist="logis",params$estimate[[1]]);title(main="Logistic model Month=120", line=2)

Figure description: Plot of MMSE scores against logistic quantile with expected logistic distribution overlay (blue) , month 120.


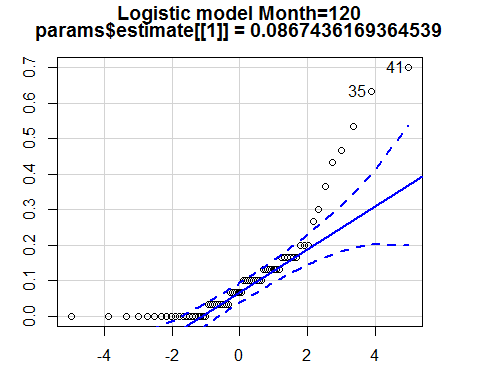


data<-MMSEdata$neg.b.MMSE[MMSEdata$M==0]
params<-fitdistr(data, "exponential")
qqp(data, "exp",rate = params$estimate, main="Exponential model Month=0")

Figure description: Plot of MMSE scores against exponential quantile with expected exponential distribution overlay (blue) , month 0.


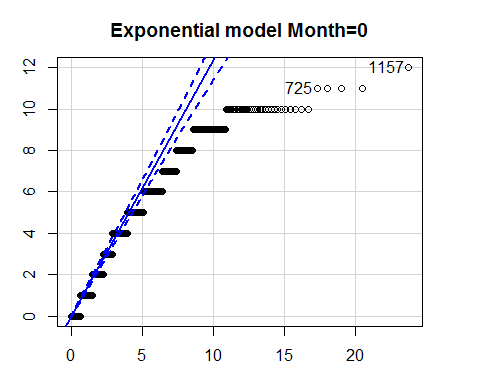


[1] 1157 725

data<-MMSEdata$neg.b.MMSE[MMSEdata$M==12]
params<-fitdistr(data, "exponential")
qqp(data, "exp",rate = params$estimate, main="Exponential model Month=12")

Figure description: Plot of MMSE scores against exponential quantile with expected exponential distribution overlay (blue) , month 12.


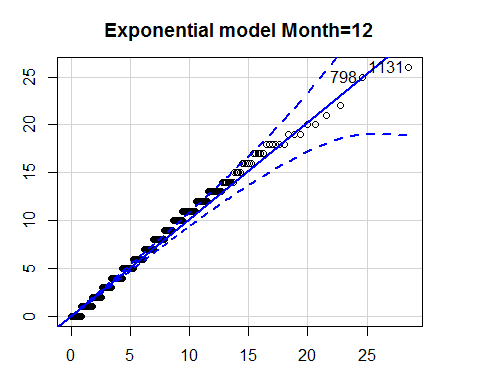


[1] 1131 798

data<-MMSEdata$neg.b.MMSE[MMSEdata$M==24]
params<-fitdistr(data, "exponential")
qqp(data, "exp",rate = params$estimate, main="Exponential model Month=24")

Figure description: Plot of MMSE scores against exponential quantile with expected exponential distribution overlay (blue) , month 24.


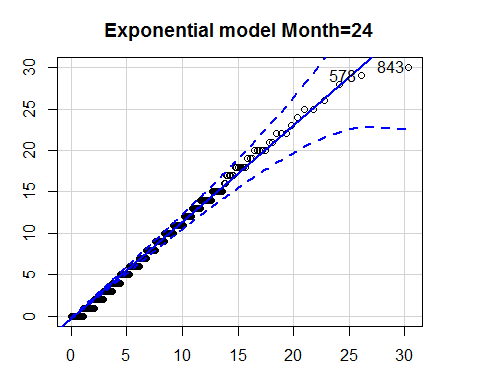


[1] 843 578

data<-MMSEdata$neg.b.MMSE[MMSEdata$M==48]
params<-fitdistr(data, "exponential")
qqp(data, "exp",rate = params$estimate, main="Exponential model Month=48")

Figure description: Plot of MMSE scores against exponential quantile with expected exponential distribution overlay (blue) , month 48.


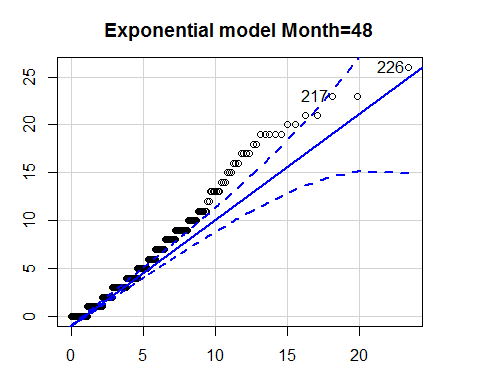


[1] 226 217

data<-MMSEdata$neg.b.MMSE[MMSEdata$M==72]
params<-fitdistr(data, "exponential")
qqp(data, "exp",rate = params$estimate, main="Exponential model Month=72")

Figure description: Plot of MMSE scores against exponential quantile with expected exponential distribution overlay (blue) , month 72.


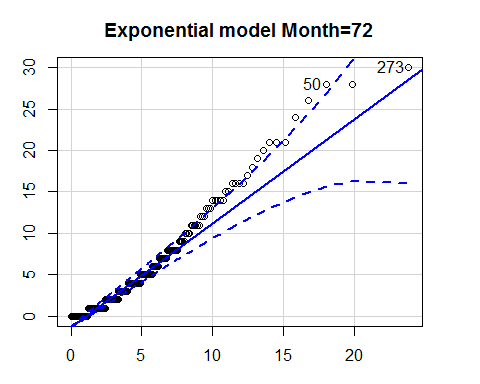


[1] 273 50

data<-MMSEdata$neg.b.MMSE[MMSEdata$M==120]
params<-fitdistr(data, "exponential")
qqp(data, "exp",rate = params$estimate, main="Exponential model Month=120")

Figure description: Plot of MMSE scores against exponential quantile with expected exponential distribution overlay (blue) , month 120.


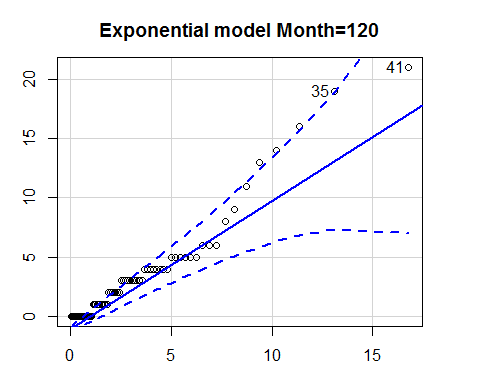


[1] 41 35

## Build base negative binomial model and comparing the different parameterization methods.

There are two commonly used parameterization methods used in the negative binomial model. To evaluate which to continue with all biological relevant main effect explanatory variables were include in the models and initial model diagnostic were performed. Using the AIC and log-likelihood statistics we can see that the Nbinom1 parameterization method is preferred. This uses a variance proportional to the mean method of parameterization.

neg.m.binom1 <- glmmadmb(neg.b.MMSE~AGE + APOE4 + M + Gender + edu.cat + diagn + vasc+diab+ aspirin+ naprox+ diclo+ parac+ celex+ Ibu+diagn*M+(1|ID), family="nbinom", data=MMSEdata)

neg.m.binom2 <- glmmadmb(neg.b.MMSE~AGE + APOE4 + M + Gender + edu.cat + diagn + vasc+diab+ aspirin+ naprox+ diclo+ parac+ celex+ Ibu+diagn*M+(1|ID), family="nbinom1", data=MMSEdata)

AIC(logLik(neg.m.binom1))

[1] 34108.8

AIC(logLik(neg.m.binom2))

[1] 34090.6

logLik(neg.m.binom1)

'log Lik.' -17029.4 (df=25)

logLik(neg.m.binom2)

'log Lik.' -17020.3 (df=25)

## Observing the distribution of the residuals in for the initial model.

Residuals within each explanatory variable collectively and within each individual show no trends and are homoscedastic centred around zero. Therefore, this model is accurate and will be used to investigate the effects of the input variables (including pain medications) going forward.

augDat <- data.frame(MMSEdata,resid=residuals(neg.m.binom2,type="pearson"),
fitted=fitted(neg.m.binom2))
ggplot(augDat,aes(x=Gender,y=resid,group=ID))+geom_boxplot()+coord_flip()

Figure description: Box Plots of the Pearson residuals per participant ID showing centred clustering around zero. Grouped by gender.


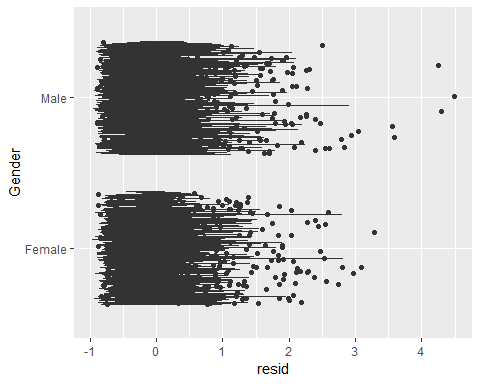


ggplot(augDat,aes(x=AGE,y=resid,group=ID))+geom_boxplot()+coord_flip()

Figure description: Box Plots of the Pearson residuals per participant ID showing centred clustering around zero. Plotted against Age.


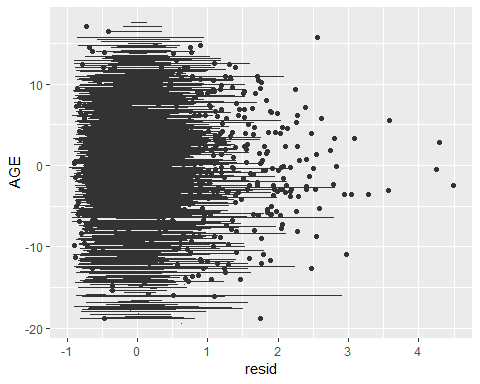


ggplot(augDat,aes(x=edu.cat,y=resid,group=ID))+geom_boxplot()+coord_flip()

Figure description: Box Plots of the Pearson residuals per participant ID showing centred clustering around zero. Plotted against Education level.


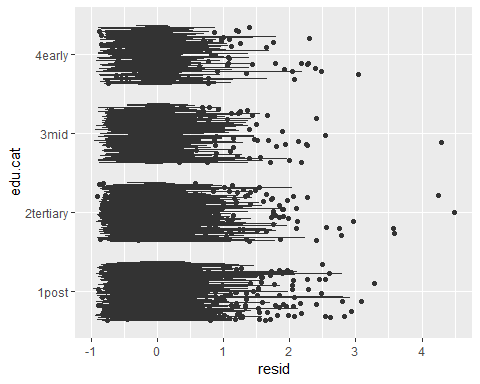


ggplot(augDat,aes(x=diagn,y=resid,group=ID))+geom_boxplot()+coord_flip()

Figure description: Box Plots of the Pearson residuals per participant ID showing centred clustering around zero. Plotted against cognitive diagnosis.


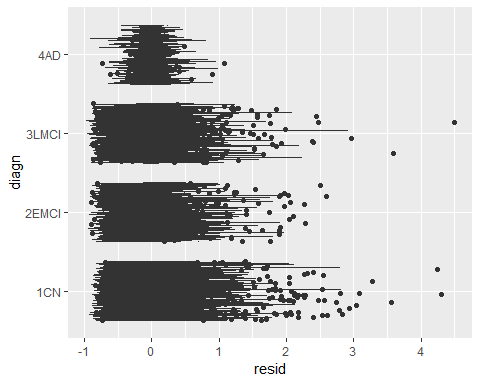


ggplot(augDat,aes(x=APOE4,y=resid,group=ID))+geom_boxplot()+coord_flip()

Figure description: Box Plots of the Pearson residuals per participant ID showing centred clustering around zero. Plotted against ApoE4 status.


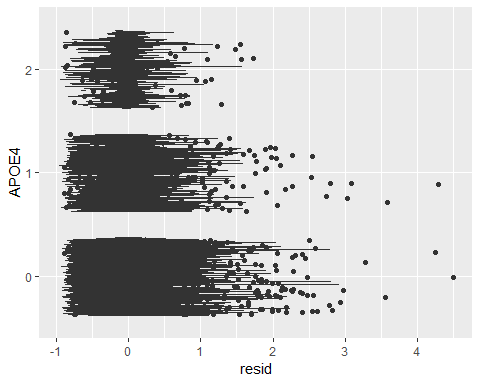


ggplot(augDat,aes(x=M,y=resid,group=ID))+geom_boxplot()+coord_flip()

Figure description: Box Plots of the Pearson residuals per participant ID showing centred clustering around zero. Plotted against time (months).


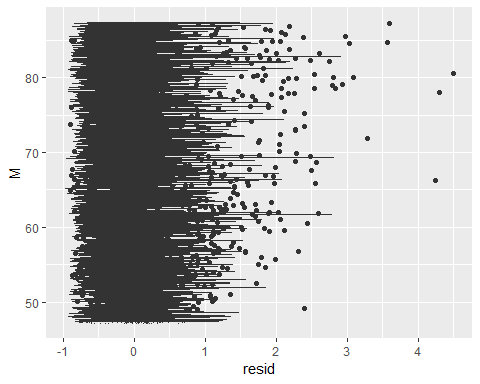


ggplot(augDat,aes(x=diclo,y=resid,group=ID))+geom_boxplot()+coord_flip()

Figure description: Box Plots of the Pearson residuals per participant ID showing centred clustering around zero. Plotted by diclofenac use.


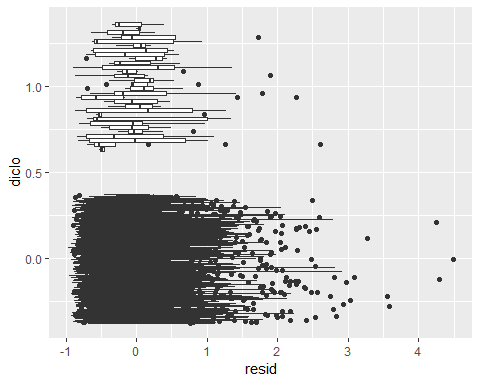


ggplot(augDat,aes(x=vasc,y=resid,group=ID))+geom_boxplot()+coord_flip()

Figure description: Box Plots of the Pearson residuals per participant ID showing centred clustering around zero. Plotted by cardiovascular status.


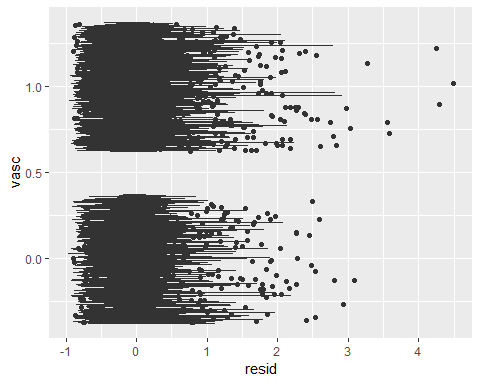


ggplot(augDat,aes(x=Ibu,y=resid,group=ID))+geom_boxplot()+coord_flip()

Figure description: Box Plots of the Pearson residuals per participant ID showing centred clustering around zero. Plotted by ibuprofen use.


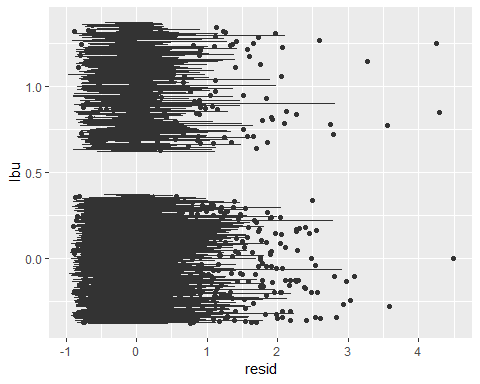


ggplot(augDat,aes(x=aspirin,y=resid,group=ID))+geom_boxplot()+coord_flip()

Figure description: Box Plots of the Pearson residuals per participant ID showing centred clustering around zero. Plotted by aspirin use.


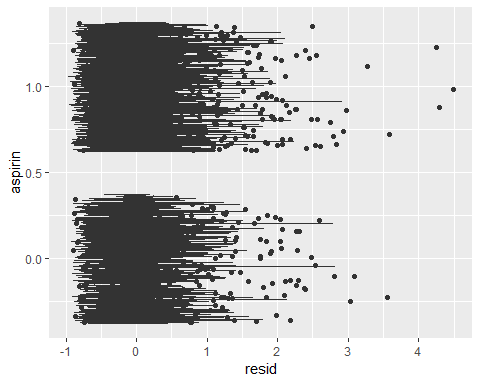


ggplot(augDat,aes(x=diab,y=resid,group=ID))+geom_boxplot()+coord_flip()

Figure description: Box Plots of the Pearson residuals per participant ID showing centred clustering around zero. Plotted by diabetes status.


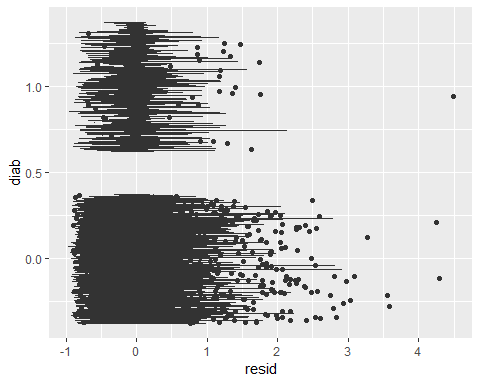


ggplot(augDat,aes(x=naprox,y=resid,group=ID))+geom_boxplot()+coord_flip()

Figure description: Box Plots of the Pearson residuals per participant ID showing centred clustering around zero. Plotted by naproxen use.


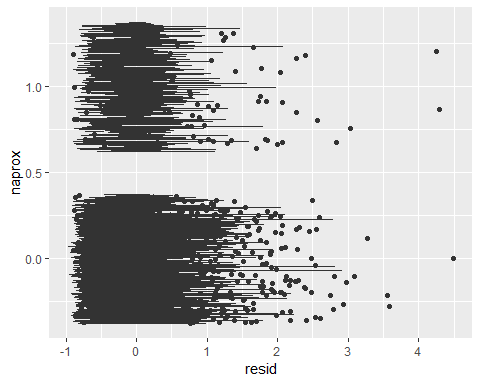


ggplot(augDat,aes(x=celex,y=resid,group=ID))+geom_boxplot()+coord_flip()

Figure description: Box Plots of the Pearson residuals per participant ID showing centred clustering around zero. Plotted by celecoxib use.


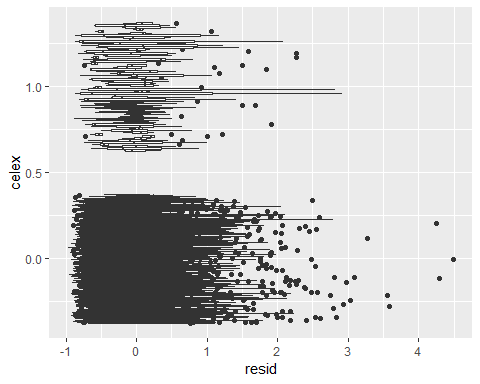


ggplot(augDat,aes(x=Gender,y=resid))+geom_boxplot()+coord_flip()

Figure description: Box Plots of the Pearson residuals at the participant level, showing centred clustering around zero. Plotted by gender.


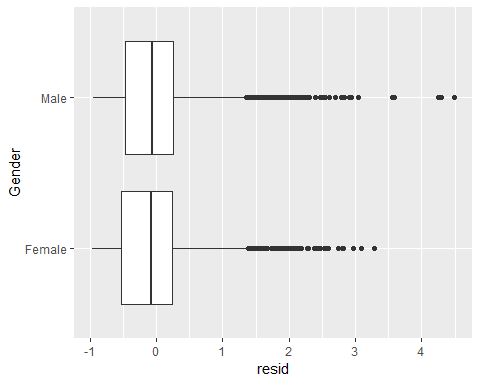


ggplot(augDat,aes(x=AGE,y=resid))+geom_point()+coord_flip()+geom_smooth(method=lm)

Figure description: Box Plots of the Pearson residuals at the participant level, showing centred clustering around zero. Plotted by against age.


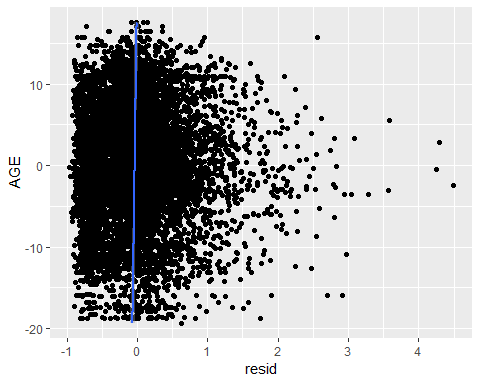


ggplot(augDat,aes(x=edu.cat,y=resid))+geom_boxplot()+coord_flip()

Figure description: Box Plots of the Pearson residuals at the participant level, showing centred clustering around zero. Plotted by education level.


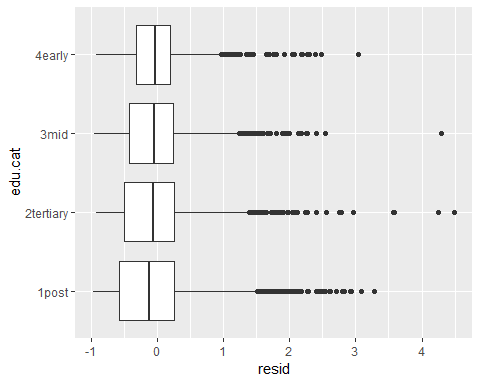


ggplot(augDat,aes(x=diagn,y=resid))+geom_boxplot()+coord_flip()

Figure description: Box Plots of the Pearson residuals at the participant level, showing centred clustering around zero. Plotted by cognitive diagnosis.


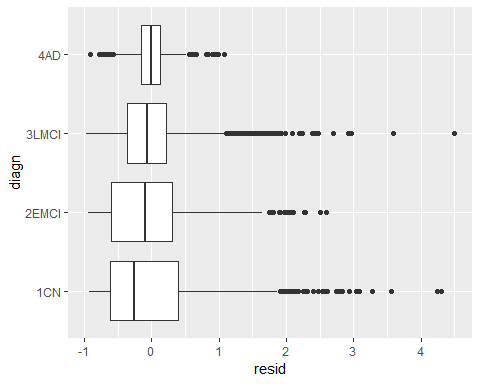


ggplot(augDat,aes(x=APOE4,y=resid))+geom_boxplot()+coord_flip()

Figure description: Box Plots of the Pearson residuals at the participant level, showing centred clustering around zero. Plotted by ApoE4 status.


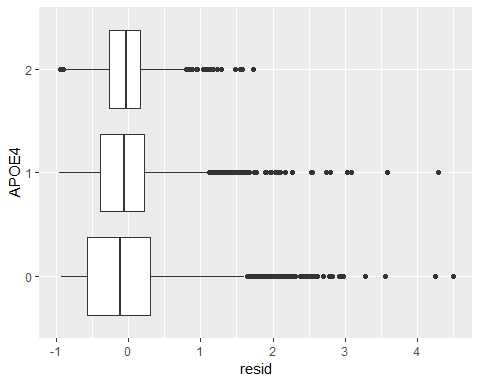


ggplot(augDat,aes(x=as.factor(M),y=resid))+geom_boxplot()+coord_flip()

Figure description: Box Plots of the Pearson residuals at the participant level, showing centred clustering around zero. Plotted by time (month).


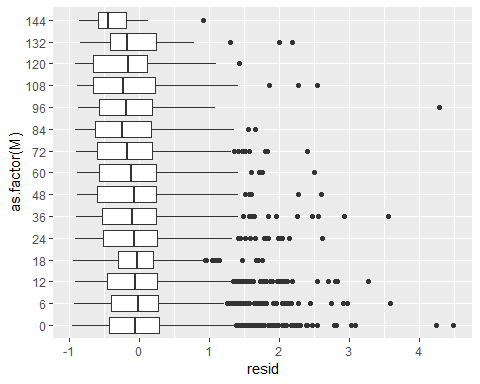


ggplot(augDat,aes(x=as.factor(diclo),y=resid))+geom_boxplot()+coord_flip()

Figure description: Box Plots of the Pearson residuals at the participant level, showing centred clustering around zero. Plotted by diclofenac use.


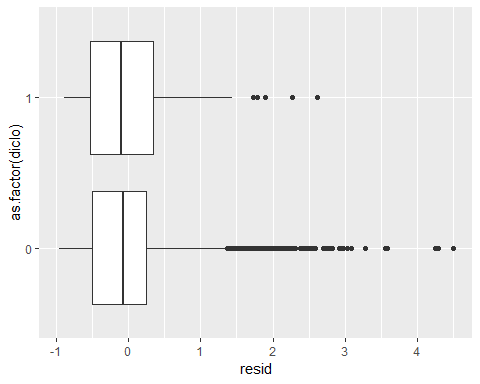


ggplot(augDat,aes(x=as.factor(vasc),y=resid))+geom_boxplot()+coord_flip()

Figure description: Box Plots of the Pearson residuals at the participant level, showing centred clustering around zero. Plotted by cardiovascular status.


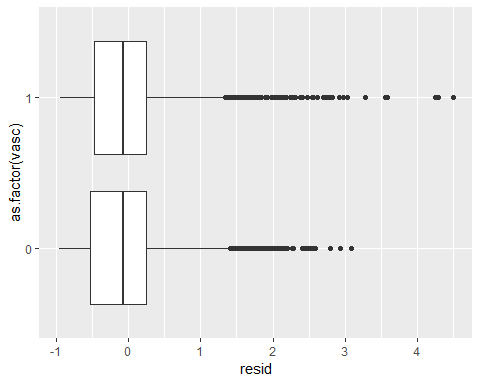


ggplot(augDat,aes(x=as.factor(Ibu),y=resid))+geom_boxplot()+coord_flip()

Figure description: Box Plots of the Pearson residuals at the participant level, showing centred clustering around zero. Plotted by ibuprofen use.


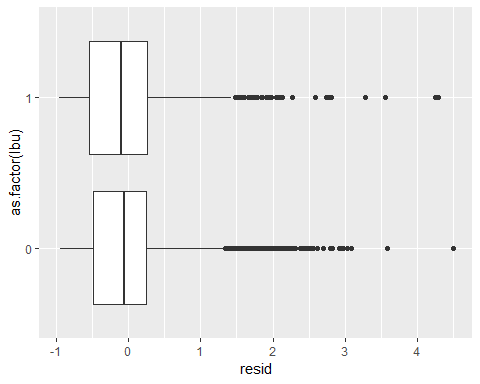


ggplot(augDat,aes(x=as.factor(aspirin),y=resid))+geom_boxplot()+coord_flip()

Figure description: Box Plots of the Pearson residuals at the participant level, showing centred clustering around zero. Plotted by aspirin use.


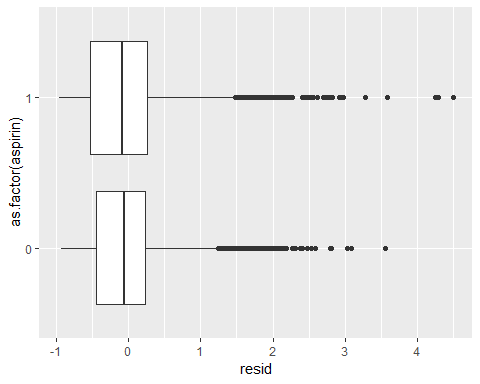


ggplot(augDat,aes(x=as.factor(diab),y=resid))+geom_boxplot()+coord_flip()

Figure description: Box Plots of the Pearson residuals at the participant level, showing centred clustering around zero. Plotted by diabetes status.


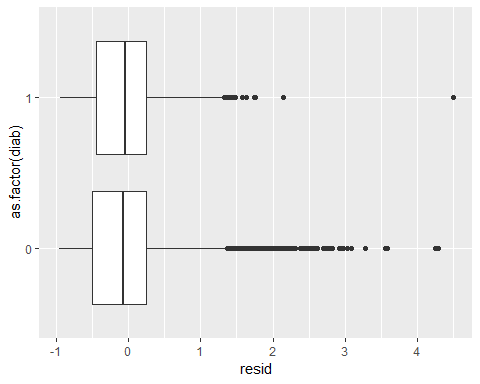


ggplot(augDat,aes(x=as.factor(naprox),y=resid))+geom_boxplot()+coord_flip()

Figure description: Box Plots of the Pearson residuals at the participant level, showing centred clustering around zero. Plotted by naproxen use.


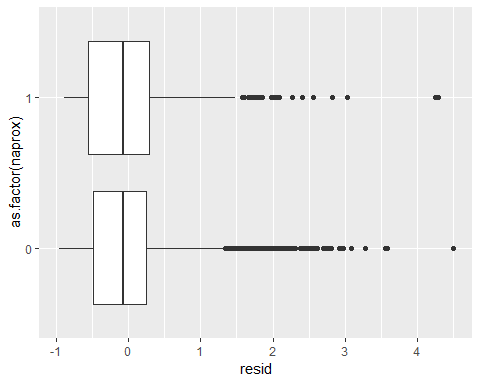


ggplot(augDat,aes(x=as.factor(celex),y=resid))+geom_boxplot()+coord_flip()

Figure description: Box Plots of the Pearson residuals at the participant level, showing centred clustering around zero. Plotted by celecoxib use.


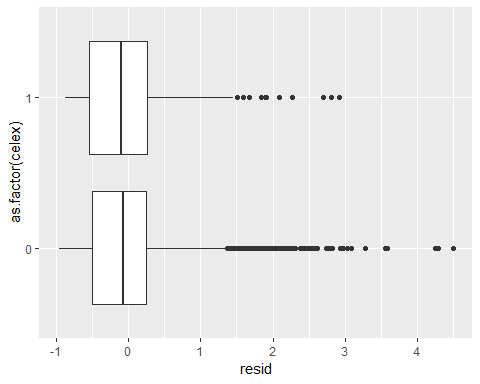


## Analysing all variables in isolation

### Main effect of gender

Adding the main effect of gender to the model did significantly improve the model based on the log-likelihood statistic.

main.M.M<-glmmadmb(neg.b.MMSE~M+ (1|ID), family="nbinom1", data=MMSEdata)
main.M.Gender <- glmmadmb(neg.b.MMSE~ M + Gender+ (1|ID), family="nbinom1", data=MMSEdata)
summary(main.M.Gender)

Call:
glmmadmb(formula = neg.b.MMSE ~ M + Gender + (1 | ID), data = MMSEdata,
 family = "nbinom1")

AIC: 35674.4

Coefficients:
 Estimate Std. Error z value Pr(>|z|)
(Intercept) 0.561427 0.045491 12.34 <2e-16 ***
M 0.012465 0.000293 42.48 <2e-16 ***
GenderMale 0.116607 0.059335 1.97 0.049 *
---
Signif. codes: 0 '***' 0.001 '**' 0.01 '*' 0.05 '.' 0.1 ' ' 1

Number of observations: total=8878, ID=1619
Random effect variance(s):
Group=ID
 Variance StdDev
(Intercept) 1.265 1.125

Negative binomial dispersion parameter: 1.0853 (std. err.: 0.018723)

Log-likelihood: -17832.2

anova(main.M.M,main.M.Gender)

Analysis of Deviance Table

Model 1: neg.b.MMSE ~ M
Model 2: neg.b.MMSE ~ M + Gender
 NoPar LogLik Df Deviance Pr(>Chi)
1 4 -17834
2 5 -17832 1 4 0.0455 *
---
Signif. codes: 0 '***' 0.001 '**' 0.01 '*' 0.05 '.' 0.1 ' ' 1

### Main effect of age

Adding the main effect of age at the beginning of the study (AGE) to the model did improve the model as measured by the log-likelihood statistic.

main.M.AGE <- glmmadmb(neg.b.MMSE~ M + AGE+ (1|ID), family="nbinom1", data=MMSEdata)
summary(main.M.AGE)

Call:
glmmadmb(formula = neg.b.MMSE ~ M + AGE + (1 | ID), data = MMSEdata,
 family = "nbinom1")

AIC: 35646.2

Coefficients:
 Estimate Std. Error z value Pr(>|z|)
(Intercept) 0.624381 0.030347 20.57 < 2e-16 ***
M 0.012464 0.000293 42.49 < 2e-16 ***
AGE 0.022869 0.004026 5.68 1.3e-08 ***
---
Signif. codes: 0 '***' 0.001 '**' 0.01 '*' 0.05 '.' 0.1 ' ' 1

Number of observations: total=8878, ID=1619
Random effect variance(s):
Group=ID
 Variance StdDev
(Intercept) 1.243 1.115

Negative binomial dispersion parameter: 1.0855 (std. err.: 0.018719)

Log-likelihood: -17818.1

anova(main.M.M,main.M.AGE)

Analysis of Deviance Table

Model 1: neg.b.MMSE ~ M
Model 2: neg.b.MMSE ~ M + AGE
 NoPar LogLik Df Deviance Pr(>Chi)
1 4 -17834
2 5 -17818 1 32.2 1.391e-08 ***
---
Signif. codes: 0 '***' 0.001 '**' 0.01 '*' 0.05 '.' 0.1 ' ' 1

### Main effect of education level

Adding the main effect of education (edu.cat) to the model did improve the model as measured by the log-likelihood statistic.

main.M.edu.cat <- glmmadmb(neg.b.MMSE~ M + edu.cat+ (1|ID), family="nbinom1", data=MMSEdata)
summary(main.M.edu.cat)

Call:
glmmadmb(formula = neg.b.MMSE ~ M + edu.cat + (1 | ID), data = MMSEdata,
 family = "nbinom1")

AIC: 35607.2

Coefficients:
 Estimate Std. Error z value Pr(>|z|)
(Intercept) 0.349889 0.050142 6.98 3.0e-12 ***
M 0.012452 0.000293 42.45 < 2e-16 ***
edu.cat2tertiary 0.279298 0.072945 3.83 0.00013 ***
edu.cat3mid 0.380995 0.081148 4.70 2.7e-06 ***
edu.cat4early 0.726814 0.084890 8.56 < 2e-16 ***
---
Signif. codes: 0 '***' 0.001 '**' 0.01 '*' 0.05 '.' 0.1 ' ' 1

Number of observations: total=8878, ID=1619
Random effect variance(s):
Group=ID
 Variance StdDev
(Intercept) 1.206 1.098

Negative binomial dispersion parameter: 1.0858 (std. err.: 0.018735)

Log-likelihood: -17796.6

anova(main.M.M,main.M.edu.cat)

Analysis of Deviance Table

Model 1: neg.b.MMSE ~ M
Model 2: neg.b.MMSE ~ M + edu.cat
 NoPar LogLik Df Deviance Pr(>Chi)
1 4 -17834
2 7 -17797 3 75.2 3.331e-16 ***
---
Signif. codes: 0 '***' 0.001 '**' 0.01 '*' 0.05 '.' 0.1 ' ' 1

### Main effect of diagnosis

Adding the main effect of initial diagnosis (diag) to the model did improve the model as measured by the log-likelihood statistic.

main.M.diagn <- glmmadmb(neg.b.MMSE~ M + diagn+ (1|ID), family="nbinom1", data=MMSEdata)
summary(main.M.diagn)

Call:
glmmadmb(formula = neg.b.MMSE ~ M + diagn + (1 | ID), data = MMSEdata,
 family = "nbinom1")

AIC: 34355.2

Coefficients:
 Estimate Std. Error z value Pr(>|z|)
(Intercept) -0.49857 0.04281 -11.6 <2e-16 ***
M 0.01268 0.00029 43.7 <2e-16 ***
diagn2EMCI 0.67645 0.06109 11.1 <2e-16 ***
diagn3LMCI 1.44331 0.05161 28.0 <2e-16 ***
diagn4AD 2.43281 0.05776 42.1 <2e-16 ***
---
Signif. codes: 0 '***' 0.001 '**' 0.01 '*' 0.05 '.' 0.1 ' ' 1

Number of observations: total=8878, ID=1619
Random effect variance(s):
Group=ID
 Variance StdDev
(Intercept) 0.4685 0.6844

Negative binomial dispersion parameter: 1.0963 (std. err.: 0.018888)

Log-likelihood: -17170.6

anova(main.M.M,main.M.diagn)

Analysis of Deviance Table

Model 1: neg.b.MMSE ~ M
Model 2: neg.b.MMSE ~ M + diagn
 NoPar LogLik Df Deviance Pr(>Chi)
1 4 -17834
2 7 -17171 3 1327.2 < 2.2e-16 ***
---
Signif. codes: 0 '***' 0.001 '**' 0.01 '*' 0.05 '.' 0.1 ' ' 1

### Main effect of APOE status

main.M.APOE4<-glmmadmb(neg.b.MMSE~M+APOE4+ (1|ID), family="nbinom1", data=MMSEdata)
main.M.Gender <- glmmadmb(neg.b.MMSE~ M + Gender+ (1|ID), family="nbinom1", data=MMSEdata)
summary(main.M.APOE4)

Call:
glmmadmb(formula = neg.b.MMSE ~ M + APOE4 + (1 | ID), data = MMSEdata,
 family = "nbinom1")

AIC: 35494

Coefficients:
 Estimate Std. Error z value Pr(>|z|)
(Intercept) 0.271544 0.040061 6.78 1.2e-11 ***
M 0.012464 0.000293 42.52 < 2e-16 ***
APOE41 0.661684 0.059409 11.14 < 2e-16 ***
APOE42 1.038219 0.092176 11.26 < 2e-16 ***
---
Signif. codes: 0 '***' 0.001 '**' 0.01 '*' 0.05 '.' 0.1 ' ' 1

Number of observations: total=8878, ID=1619
Random effect variance(s):
Group=ID
 Variance StdDev
(Intercept) 1.111 1.054

Negative binomial dispersion parameter: 1.0865 (std. err.: 0.018743)

Log-likelihood: -17741

anova(main.M.M,main.M.APOE4)

Analysis of Deviance Table

Model 1: neg.b.MMSE ~ M
Model 2: neg.b.MMSE ~ M + APOE4
 NoPar LogLik Df Deviance Pr(>Chi)
1 4 -17834
2 6 -17741 2 186.4 < 2.2e-16 ***
---
Signif. codes: 0 '***' 0.001 '**' 0.01 '*' 0.05 '.' 0.1 ' ' 1

### Main effect of cardiovascular pathology

Adding the main effect of cardiovascular co-morbidity (vasc) to the model did significantly improve the model as measured by the log-likelihood statistic.

main.M.vasc <- glmmadmb(neg.b.MMSE~ M + vasc+ (1|ID), family="nbinom1", data=MMSEdata)
summary(main.M.vasc)

Call:
glmmadmb(formula = neg.b.MMSE ~ M + vasc + (1 | ID), data = MMSEdata,
 family = "nbinom1")

AIC: 35678.2

Coefficients:
 Estimate Std. Error z value Pr(>|z|)
(Intercept) 0.609684 0.048296 12.62 <2e-16 ***
M 0.012465 0.000293 42.47 <2e-16 ***
vasc 0.028585 0.060548 0.47 0.64
---
Signif. codes: 0 '***' 0.001 '**' 0.01 '*' 0.05 '.' 0.1 ' ' 1

Number of observations: total=8878, ID=1619
Random effect variance(s):
Group=ID
 Variance StdDev
(Intercept) 1.266 1.125

Negative binomial dispersion parameter: 1.0855 (std. err.: 0.018729)

Log-likelihood: -17834.1

anova(main.M.M,main.M.vasc)

Analysis of Deviance Table

Model 1: neg.b.MMSE ~ M
Model 2: neg.b.MMSE ~ M + vasc
 NoPar LogLik Df Deviance Pr(>Chi)
1 4 -17834
2 5 -17834 1 0.2 0.6547

### Main effect of diabetes

Adding the main effect of diabetes co-morbidity (diab) to the model did not improve the model as measured by the log-likelihood statistic.

main.M.diab <- glmmadmb(neg.b.MMSE~ M + diab+ (1|ID), family="nbinom1", data=MMSEdata)
summary(main.M.diab)

Call:
glmmadmb(formula = neg.b.MMSE ~ M + diab + (1 | ID), data = MMSEdata,
 family = "nbinom1")

AIC: 35678.2

Coefficients:
 Estimate Std. Error z value Pr(>|z|)
(Intercept) 0.624510 0.031996 19.5 <2e-16 ***
M 0.012466 0.000293 42.5 <2e-16 ***
diab 0.030173 0.101240 0.3 0.77
---
Signif. codes: 0 '***' 0.001 '**' 0.01 '*' 0.05 '.' 0.1 ' ' 1

Number of observations: total=8878, ID=1619
Random effect variance(s):
Group=ID
 Variance StdDev
(Intercept) 1.267 1.125

Negative binomial dispersion parameter: 1.0855 (std. err.: 0.018728)

Log-likelihood: -17834.1

anova(main.M.M,main.M.diab)

Analysis of Deviance Table

Model 1: neg.b.MMSE ~ M
Model 2: neg.b.MMSE ~ M + diab
 NoPar LogLik Df Deviance Pr(>Chi)
1 4 -17834
2 5 -17834 1 0.2 0.6547

### Main effect of smoking

Adding the main effect of smoking at the beginning of the study (AGE) to the model did improve the model as measured by the log-likelihood statistic.

main.M.smoke <- glmmadmb(neg.b.MMSE~ M + smoke+ (1|ID), family="nbinom1", data=MMSEdata)
summary(main.M.smoke)

Call:
glmmadmb(formula = neg.b.MMSE ~ M + smoke + (1 | ID), data = MMSEdata,
 family = "nbinom1")

AIC: 35678.2

Coefficients:
 Estimate Std. Error z value Pr(>|z|)
(Intercept) 0.619744 0.035120 17.65 <2e-16 ***
M 0.012465 0.000293 42.48 <2e-16 ***
smoke 0.029579 0.067375 0.44 0.66
---
Signif. codes: 0 '***' 0.001 '**' 0.01 '*' 0.05 '.' 0.1 ' ' 1

Number of observations: total=8878, ID=1619
Random effect variance(s):
Group=ID
 Variance StdDev
(Intercept) 1.267 1.125

Negative binomial dispersion parameter: 1.0855 (std. err.: 0.018728)

Log-likelihood: -17834.1

anova(main.M.M,main.M.smoke)

Analysis of Deviance Table

Model 1: neg.b.MMSE ~ M
Model 2: neg.b.MMSE ~ M + smoke
 NoPar LogLik Df Deviance Pr(>Chi)
1 4 -17834
2 5 -17834 1 0.2 0.6547

### Main effect of headache

Adding the main effect of headache at the beginning of the study (AGE) to the model did improve the model as measured by the log-likelihood statistic.

main.M.headache <- glmmadmb(neg.b.MMSE~ M + headache+ (1|ID), family="nbinom1", data=MMSEdata)
summary(main.M.headache)

Call:
glmmadmb(formula = neg.b.MMSE ~ M + headache + (1 | ID), data = MMSEdata,
 family = "nbinom1")

AIC: 35674.2

Coefficients:
 Estimate Std. Error z value Pr(>|z|)
(Intercept) 0.645609 0.031778 20.32 <2e-16 ***
M 0.012476 0.000293 42.51 <2e-16 ***
headache -0.213805 0.105090 -2.03 0.042 *
---
Signif. codes: 0 '***' 0.001 '**' 0.01 '*' 0.05 '.' 0.1 ' ' 1

Number of observations: total=8878, ID=1619
Random effect variance(s):
Group=ID
 Variance StdDev
(Intercept) 1.263 1.124

Negative binomial dispersion parameter: 1.0855 (std. err.: 0.018722)

Log-likelihood: -17832.1

anova(main.M.M,main.M.headache)

Analysis of Deviance Table

Model 1: neg.b.MMSE ~ M
Model 2: neg.b.MMSE ~ M + headache
 NoPar LogLik Df Deviance Pr(>Chi)
1 4 -17834
2 5 -17832 1 4.2 0.04042 *
---
Signif. codes: 0 '***' 0.001 '**' 0.01 '*' 0.05 '.' 0.1 ' ' 1

### Main effect of arthritis

Adding the main effect of headache at the beginning of the study (AGE) to the model did improve the model as measured by the log-likelihood statistic.

main.M.arthrit <- glmmadmb(neg.b.MMSE~ M + arthrit+ (1|ID), family="nbinom1", data=MMSEdata)
summary(main.M.arthrit)

Call:
glmmadmb(formula = neg.b.MMSE ~ M + arthrit + (1 | ID), data = MMSEdata,
 family = "nbinom1")

AIC: 35668

Coefficients:
 Estimate Std. Error z value Pr(>|z|)
(Intercept) 0.702304 0.038229 18.37 <2e-16 ***
M 0.012476 0.000293 42.51 <2e-16 ***
arthrit -0.193263 0.060229 -3.21 0.0013 **
---
Signif. codes: 0 '***' 0.001 '**' 0.01 '*' 0.05 '.' 0.1 ' ' 1

Number of observations: total=8878, ID=1619
Random effect variance(s):
Group=ID
 Variance StdDev
(Intercept) 1.257 1.121

Negative binomial dispersion parameter: 1.0857 (std. err.: 0.01873)

Log-likelihood: -17829

anova(main.M.M,main.M.arthrit)

Analysis of Deviance Table

Model 1: neg.b.MMSE ~ M
Model 2: neg.b.MMSE ~ M + arthrit
 NoPar LogLik Df Deviance Pr(>Chi)
1 4 -17834
2 5 -17829 1 10.4 0.00126 **
---
Signif. codes: 0 '***' 0.001 '**' 0.01 '*' 0.05 '.' 0.1 ' ' 1

### Main effect of diclofenac

Adding the main effect of diclofenac to the model did not improve the model as measured by the log-likelihood statistic.

main.M.diclo <- glmmadmb(neg.b.MMSE~M+diclo+ (1|ID), family="nbinom1", data=MMSEdata)
summary(main.M.diclo)

Call:
glmmadmb(formula = neg.b.MMSE ~ M + diclo + (1 | ID), data = MMSEdata,
 family = "nbinom1")

AIC: 35671.6

Coefficients:
 Estimate Std. Error z value Pr(>|z|)
(Intercept) 0.637814 0.030752 20.74 <2e-16 ***
M 0.012475 0.000293 42.52 <2e-16 ***
diclo -0.566154 0.219460 -2.58 0.0099 **
---
Signif. codes: 0 '***' 0.001 '**' 0.01 '*' 0.05 '.' 0.1 ' ' 1

Number of observations: total=8878, ID=1619
Random effect variance(s):
Group=ID
 Variance StdDev
(Intercept) 1.261 1.123

Negative binomial dispersion parameter: 1.0853 (std. err.: 0.018722)

Log-likelihood: -17830.8

anova(main.M.M,main.M.diclo)

Analysis of Deviance Table

Model 1: neg.b.MMSE ~ M
Model 2: neg.b.MMSE ~ M + diclo
 NoPar LogLik Df Deviance Pr(>Chi)
1 4 -17834
2 5 -17831 1 6.8 0.009116 **
---
Signif. codes: 0 '***' 0.001 '**' 0.01 '*' 0.05 '.' 0.1 ' ' 1

### Main effect of paracetamol

Adding the main effect of paracetamol to the model did improve the model as measured by the log-likelihood statistic.

main.M.parac <- glmmadmb(neg.b.MMSE~ M + parac+ (1|ID), family="nbinom1", data=MMSEdata)
summary(main.M.parac)

Call:
glmmadmb(formula = neg.b.MMSE ~ M + parac + (1 | ID), data = MMSEdata,
 family = "nbinom1")

AIC: 35670

Coefficients:
 Estimate Std. Error z value Pr(>|z|)
(Intercept) 0.676397 0.034739 19.5 <2e-16 ***
M 0.012491 0.000294 42.5 <2e-16 ***
parac -0.196590 0.067781 -2.9 0.0037 **
---
Signif. codes: 0 '***' 0.001 '**' 0.01 '*' 0.05 '.' 0.1 ' ' 1

Number of observations: total=8878, ID=1619
Random effect variance(s):
Group=ID
 Variance StdDev
(Intercept) 1.258 1.122

Negative binomial dispersion parameter: 1.0855 (std. err.: 0.018723)

Log-likelihood: -17830

anova(main.M.M,main.M.parac)

Analysis of Deviance Table

Model 1: neg.b.MMSE ~ M
Model 2: neg.b.MMSE ~ M + parac
 NoPar LogLik Df Deviance Pr(>Chi)
1 4 -17834
2 5 -17830 1 8.4 0.003752 **
---
Signif. codes: 0 '***' 0.001 '**' 0.01 '*' 0.05 '.' 0.1 ' ' 1

### Main effect celecoxib

Adding the main effect of celecoxib to the model did not improve the model as measured by the log-likelihood statistic.

main.M.celex <- glmmadmb(neg.b.MMSE~ M + celex+ (1|ID), family="nbinom1", data=MMSEdata)
summary(main.M.celex)

Call:
glmmadmb(formula = neg.b.MMSE ~ M + celex + (1 | ID), data = MMSEdata,
 family = "nbinom1")

AIC: 35674.4

Coefficients:
 Estimate Std. Error z value Pr(>|z|)
(Intercept) 0.639311 0.031086 20.6 <2e-16 ***
M 0.012470 0.000293 42.5 <2e-16 ***
celex -0.302623 0.151440 -2.0 0.046 *
---
Signif. codes: 0 '***' 0.001 '**' 0.01 '*' 0.05 '.' 0.1 ' ' 1

Number of observations: total=8878, ID=1619
Random effect variance(s):
Group=ID
 Variance StdDev
(Intercept) 1.263 1.124

Negative binomial dispersion parameter: 1.0856 (std. err.: 0.018726)

Log-likelihood: -17832.2

anova(main.M.M,main.M.celex)

Analysis of Deviance Table

Model 1: neg.b.MMSE ~ M
Model 2: neg.b.MMSE ~ M + celex
 NoPar LogLik Df Deviance Pr(>Chi)
1 4 -17834
2 5 -17832 1 4 0.0455 *
---
Signif. codes: 0 '***' 0.001 '**' 0.01 '*' 0.05 '.' 0.1 ' ' 1

### Main effect of naproxen

Adding the main effect of naproxen to the model did significantly improve the model as measured by the log-likelihood statistic.

main.M.naprox <- glmmadmb(neg.b.MMSE~ M + naprox+ (1|ID), family="nbinom1", data=MMSEdata)
summary(main.M.naprox)

Call:
glmmadmb(formula = neg.b.MMSE ~ M + naprox + (1 | ID), data = MMSEdata,
 family = "nbinom1")

AIC: 35671

Coefficients:
 Estimate Std. Error z value Pr(>|z|)
(Intercept) 0.656194 0.032227 20.36 <2e-16 ***
M 0.012480 0.000294 42.52 <2e-16 ***
naprox -0.248943 0.091866 -2.71 0.0067 **
---
Signif. codes: 0 '***' 0.001 '**' 0.01 '*' 0.05 '.' 0.1 ' ' 1

Number of observations: total=8878, ID=1619
Random effect variance(s):
Group=ID
 Variance StdDev
(Intercept) 1.259 1.122

Negative binomial dispersion parameter: 1.0858 (std. err.: 0.01873)

Log-likelihood: -17830.5

anova(main.M.M,main.M.naprox)

Analysis of Deviance Table

Model 1: neg.b.MMSE ~ M
Model 2: neg.b.MMSE ~ M + naprox
 NoPar LogLik Df Deviance Pr(>Chi)
1 4 -17834
2 5 -17831 1 7.4 0.006522 **
---
Signif. codes: 0 '***' 0.001 '**' 0.01 '*' 0.05 '.' 0.1 ' ' 1

### Main effect of aspirin

Adding the main effect of aspirin use (aspirin) to the model did improve the model as measured by the log-likelihood statistic.

main.M.aspirin <- glmmadmb(neg.b.MMSE~ M + aspirin+ (1|ID), family="nbinom1", data=MMSEdata)
summary(main.M.aspirin)

Call:
glmmadmb(formula = neg.b.MMSE ~ M + aspirin + (1 | ID), data = MMSEdata,
 family = "nbinom1")

AIC: 35651.4

Coefficients:
 Estimate Std. Error z value Pr(>|z|)
(Intercept) 0.789436 0.043143 18.3 < 2e-16 ***
M 0.012512 0.000293 42.6 < 2e-16 ***
aspirin -0.304442 0.058497 -5.2 1.9e-07 ***
---
Signif. codes: 0 '***' 0.001 '**' 0.01 '*' 0.05 '.' 0.1 ' ' 1

Number of observations: total=8878, ID=1619
Random effect variance(s):
Group=ID
 Variance StdDev
(Intercept) 1.243 1.115

Negative binomial dispersion parameter: 1.0855 (std. err.: 0.01871)

Log-likelihood: -17820.7

anova(main.M.M,main.M.aspirin)

Analysis of Deviance Table

Model 1: neg.b.MMSE ~ M
Model 2: neg.b.MMSE ~ M + aspirin
 NoPar LogLik Df Deviance Pr(>Chi)
1 4 -17834
2 5 -17821 1 27 2.035e-07 ***
---
Signif. codes: 0 '***' 0.001 '**' 0.01 '*' 0.05 '.' 0.1 ' ' 1

### Main effect of ibuprofen

Adding the main effect of ibuprofen use (Ibu) to the model did improve the model as measured by the log-likelihood statistic.

main.M.Ibu <- glmmadmb(neg.b.MMSE~ M + Ibu+ (1|ID), family="nbinom1", data=MMSEdata)
summary(main.M.Ibu)

Call:
glmmadmb(formula = neg.b.MMSE ~ M + Ibu + (1 | ID), data = MMSEdata,
 family = "nbinom1")

AIC: 35657.2

Coefficients:
 Estimate Std. Error z value Pr(>|z|)
(Intercept) 0.686112 0.032787 20.93 < 2e-16 ***
M 0.012490 0.000293 42.57 < 2e-16 ***
Ibu -0.371462 0.080479 -4.62 3.9e-06 ***
---
Signif. codes: 0 '***' 0.001 '**' 0.01 '*' 0.05 '.' 0.1 ' ' 1

Number of observations: total=8878, ID=1619
Random effect variance(s):
Group=ID
 Variance StdDev
(Intercept) 1.248 1.117

Negative binomial dispersion parameter: 1.0856 (std. err.: 0.01872)

Log-likelihood: -17823.6

anova(main.M.M,main.M.Ibu)

Analysis of Deviance Table

Model 1: neg.b.MMSE ~ M
Model 2: neg.b.MMSE ~ M + Ibu
 NoPar LogLik Df Deviance Pr(>Chi)
1 4 -17834
2 5 -17824 1 21.2 4.138e-06 ***
---
Signif. codes: 0 '***' 0.001 '**' 0.01 '*' 0.05 '.' 0.1 ' ' 1

## Building combined main effect model

main.M.combined <- glmmadmb(neg.b.MMSE~M+AGE+ APOE4+Gender+ edu.cat + diagn+ headache+arthrit+ diclo+parac+naprox+aspirin+Ibu+ (1|ID), family="nbinom1", data=MMSEdata)
summary(main.M.combined)

Call:
glmmadmb(formula = neg.b.MMSE ~ M + AGE + APOE4 + Gender + edu.cat +
 diagn + headache + arthrit + diclo + parac + naprox + aspirin +
 Ibu + (1 | ID), data = MMSEdata, family = "nbinom1")

AIC: 34211.2

Coefficients:
 Estimate Std. Error z value Pr(>|z|)
(Intercept) -0.63958 0.06138 -10.42 < 2e-16 ***
M 0.01274 0.00029 43.93 < 2e-16 ***
AGE 0.01833 0.00265 6.91 4.9e-12 ***
APOE41 0.26721 0.04007 6.67 2.6e-11 ***
APOE42 0.39733 0.06229 6.38 1.8e-10 ***
GenderMale 0.04831 0.03851 1.25 0.210
edu.cat2tertiary 0.11353 0.04655 2.44 0.015 *
edu.cat3mid 0.31431 0.05227 6.01 1.8e-09 ***
edu.cat4early 0.30784 0.05484 5.61 2.0e-08 ***
diagn2EMCI 0.66305 0.05960 11.13 < 2e-16 ***
diagn3LMCI 1.34688 0.05097 26.43 < 2e-16 ***
diagn4AD 2.22312 0.05849 38.01 < 2e-16 ***
headache -0.05894 0.06593 -0.89 0.371
arthrit -0.06332 0.03882 -1.63 0.103
diclo -0.12938 0.13789 -0.94 0.348
parac -0.03719 0.04346 -0.86 0.392
naprox -0.00065 0.05791 -0.01 0.991
aspirin -0.07873 0.03733 -2.11 0.035 *
Ibu -0.09416 0.05090 -1.85 0.064 .
---
Signif. codes: 0 '***' 0.001 '**' 0.01 '*' 0.05 '.' 0.1 ' ' 1

Number of observations: total=8878, ID=1619
Random effect variance(s):
Group=ID
 Variance StdDev
(Intercept) 0.4157 0.6447

Negative binomial dispersion parameter: 1.096 (std. err.: 0.018805)

Log-likelihood: -17084.6

anova(main.M.M,main.M.combined)

Analysis of Deviance Table

Model 1: neg.b.MMSE ~ M
Model 2: neg.b.MMSE ~ M + AGE + APOE4 + Gender + edu.cat + diagn + headache + arthrit + diclo + parac + naprox + aspirin + Ibu
 NoPar LogLik Df Deviance Pr(>Chi)
1 4 -17834
2 21 -17085 17 1499.2 < 2.2e-16 ***
---
Signif. codes: 0 '***' 0.001 '**' 0.01 '*' 0.05 '.' 0.1 ' ' 1

## Dropping non-significant terms

### Narpoxen

Dropping naproxen from the model did not significantly worsen the model

main.M.combined.drop.naprox <- glmmadmb(neg.b.MMSE~M+AGE+ APOE4+Gender+ edu.cat + diagn+ headache+arthrit+ diclo+parac+aspirin+Ibu+ (1|ID), family="nbinom1", data=MMSEdata)
summary(main.M.combined.drop.naprox)

Call:
glmmadmb(formula = neg.b.MMSE ~ M + AGE + APOE4 + Gender + edu.cat +
 diagn + headache + arthrit + diclo + parac + aspirin + Ibu +
 (1 | ID), data = MMSEdata, family = "nbinom1")

AIC: 34209.2

Coefficients:
 Estimate Std. Error z value Pr(>|z|)
(Intercept) -0.63962 0.06128 -10.44 < 2e-16 ***
M 0.01274 0.00029 43.94 < 2e-16 ***
AGE 0.01833 0.00265 6.92 4.5e-12 ***
APOE41 0.26721 0.04007 6.67 2.6e-11 ***
APOE42 0.39734 0.06228 6.38 1.8e-10 ***
GenderMale 0.04832 0.03850 1.26 0.209
edu.cat2tertiary 0.11352 0.04653 2.44 0.015 *
edu.cat3mid 0.31430 0.05226 6.01 1.8e-09 ***
edu.cat4early 0.30782 0.05483 5.61 2.0e-08 ***
diagn2EMCI 0.66304 0.05959 11.13 < 2e-16 ***
diagn3LMCI 1.34688 0.05097 26.43 < 2e-16 ***
diagn4AD 2.22315 0.05845 38.04 < 2e-16 ***
headache -0.05894 0.06593 -0.89 0.371
arthrit -0.06335 0.03870 -1.64 0.102
diclo -0.12938 0.13789 -0.94 0.348
parac -0.03725 0.04310 -0.86 0.387
aspirin -0.07875 0.03731 -2.11 0.035 *
Ibu -0.09419 0.05082 -1.85 0.064 .
---
Signif. codes: 0 '***' 0.001 '**' 0.01 '*' 0.05 '.' 0.1 ' ' 1

Number of observations: total=8878, ID=1619
Random effect variance(s):
Group=ID
 Variance StdDev
(Intercept) 0.4157 0.6447

Negative binomial dispersion parameter: 1.096 (std. err.: 0.018805)

Log-likelihood: -17084.6

anova(main.M.combined, main.M.combined.drop.naprox)

Analysis of Deviance Table

Model 1: neg.b.MMSE ~ M + AGE + APOE4 + Gender + edu.cat + diagn + headache + arthrit + diclo + parac + aspirin + Ibu
Model 2: neg.b.MMSE ~ M + AGE + APOE4 + Gender + edu.cat + diagn + headache + arthrit + diclo + parac + naprox + aspirin + Ibu
 NoPar LogLik Df Deviance Pr(>Chi)
1 20 -17085
2 21 -17085 1 0 1

### Diclofenac

Dropping diclofenac from the model did not significantly worsen the model

main.M.combined.drop.diclo <- glmmadmb(neg.b.MMSE~M+AGE+ APOE4+Gender+ edu.cat + diagn+ headache+arthrit+ parac+aspirin+Ibu+ (1|ID), family="nbinom1", data=MMSEdata)
summary(main.M.combined.drop.diclo)

Call:
glmmadmb(formula = neg.b.MMSE ~ M + AGE + APOE4 + Gender + edu.cat +
 diagn + headache + arthrit + parac + aspirin + Ibu + (1 |
 ID), data = MMSEdata, family = "nbinom1")

AIC: 34208.2

Coefficients:
 Estimate Std. Error z value Pr(>|z|)
(Intercept) -0.64138 0.06127 -10.47 < 2e-16 ***
M 0.01273 0.00029 43.93 < 2e-16 ***
AGE 0.01831 0.00265 6.91 4.8e-12 ***
APOE41 0.26710 0.04008 6.66 2.7e-11 ***
APOE42 0.39712 0.06230 6.37 1.8e-10 ***
GenderMale 0.04863 0.03850 1.26 0.207
edu.cat2tertiary 0.11277 0.04654 2.42 0.015 *
edu.cat3mid 0.31591 0.05225 6.05 1.5e-09 ***
edu.cat4early 0.30691 0.05484 5.60 2.2e-08 ***
diagn2EMCI 0.66485 0.05957 11.16 < 2e-16 ***
diagn3LMCI 1.34913 0.05092 26.49 < 2e-16 ***
diagn4AD 2.22573 0.05840 38.11 < 2e-16 ***
headache -0.06012 0.06594 -0.91 0.362
arthrit -0.06673 0.03855 -1.73 0.083 .
parac -0.03790 0.04310 -0.88 0.379
aspirin -0.08021 0.03729 -2.15 0.031 *
Ibu -0.09404 0.05083 -1.85 0.064 .
---
Signif. codes: 0 '***' 0.001 '**' 0.01 '*' 0.05 '.' 0.1 ' ' 1

Number of observations: total=8878, ID=1619
Random effect variance(s):
Group=ID
 Variance StdDev
(Intercept) 0.4159 0.6449

Negative binomial dispersion parameter: 1.0961 (std. err.: 0.018807)

Log-likelihood: -17085.1

anova(main.M.combined.drop.naprox, main.M.combined.drop.diclo)

Analysis of Deviance Table

Model 1: neg.b.MMSE ~ M + AGE + APOE4 + Gender + edu.cat + diagn + headache + arthrit + parac + aspirin + Ibu
Model 2: neg.b.MMSE ~ M + AGE + APOE4 + Gender + edu.cat + diagn + headache + arthrit + diclo + parac + aspirin + Ibu
 NoPar LogLik Df Deviance Pr(>Chi)
1 19 -17085
2 20 -17085 1 1 0.3173

### Headache

Dropping paracetamol from the model did not significantly worsen the model

main.M.combined.drop.headache<- glmmadmb(neg.b.MMSE~M+AGE+ APOE4+Gender+ edu.cat + diagn+ arthrit+ parac+aspirin+Ibu+ (1|ID), family="nbinom1", data=MMSEdata)
summary(main.M.combined.drop.headache)

Call:
glmmadmb(formula = neg.b.MMSE ~ M + AGE + APOE4 + Gender + edu.cat +
 diagn + arthrit + parac + aspirin + Ibu + (1 | ID), data = MMSEdata,
 family = "nbinom1")

AIC: 34207

Coefficients:
 Estimate Std. Error z value Pr(>|z|)
(Intercept) -0.64760 0.06093 -10.63 < 2e-16 ***
M 0.01273 0.00029 43.92 < 2e-16 ***
AGE 0.01859 0.00263 7.06 1.7e-12 ***
APOE41 0.26828 0.04007 6.70 2.2e-11 ***
APOE42 0.39808 0.06232 6.39 1.7e-10 ***
GenderMale 0.05142 0.03840 1.34 0.180
edu.cat2tertiary 0.11416 0.04653 2.45 0.014 *
edu.cat3mid 0.31592 0.05227 6.04 1.5e-09 ***
edu.cat4early 0.30660 0.05486 5.59 2.3e-08 ***
diagn2EMCI 0.66505 0.05959 11.16 < 2e-16 ***
diagn3LMCI 1.34870 0.05094 26.48 < 2e-16 ***
diagn4AD 2.22593 0.05842 38.10 < 2e-16 ***
arthrit -0.06899 0.03848 -1.79 0.073 .
parac -0.04047 0.04303 -0.94 0.347
aspirin -0.07972 0.03730 -2.14 0.033 *
Ibu -0.09467 0.05084 -1.86 0.063 .
---
Signif. codes: 0 '***' 0.001 '**' 0.01 '*' 0.05 '.' 0.1 ' ' 1

Number of observations: total=8878, ID=1619
Random effect variance(s):
Group=ID
 Variance StdDev
(Intercept) 0.4163 0.6452

Negative binomial dispersion parameter: 1.0961 (std. err.: 0.018809)

Log-likelihood: -17085.5

anova(main.M.combined.drop.diclo, main.M.combined.drop.headache)

Analysis of Deviance Table

Model 1: neg.b.MMSE ~ M + AGE + APOE4 + Gender + edu.cat + diagn + arthrit + parac + aspirin + Ibu
Model 2: neg.b.MMSE ~ M + AGE + APOE4 + Gender + edu.cat + diagn + headache + arthrit + parac + aspirin + Ibu
 NoPar LogLik Df Deviance Pr(>Chi)
1 18 -17086
2 19 -17085 1 0.8 0.3711

### Paracetamol

Dropping paracetamol from the model did not significantly worsen the model

main.M.combined.drop.parac<- glmmadmb(neg.b.MMSE~M+AGE+ APOE4+Gender+ edu.cat + diagn+ arthrit+ aspirin+Ibu+ (1|ID), family="nbinom1", data=MMSEdata)
summary(main.M.combined.drop.parac)

Call:
glmmadmb(formula = neg.b.MMSE ~ M + AGE + APOE4 + Gender + edu.cat +
 diagn + arthrit + aspirin + Ibu + (1 | ID), data = MMSEdata,
 family = "nbinom1")

AIC: 34205.8

Coefficients:
 Estimate Std. Error z value Pr(>|z|)
(Intercept) -0.65579 0.06035 -10.87 < 2e-16 ***
M 0.01272 0.00029 43.91 < 2e-16 ***
AGE 0.01852 0.00263 7.03 2.0e-12 ***
APOE41 0.27010 0.04004 6.75 1.5e-11 ***
APOE42 0.39910 0.06234 6.40 1.5e-10 ***
GenderMale 0.05408 0.03831 1.41 0.158
edu.cat2tertiary 0.11321 0.04654 2.43 0.015 *
edu.cat3mid 0.31432 0.05226 6.01 1.8e-09 ***
edu.cat4early 0.30751 0.05487 5.60 2.1e-08 ***
diagn2EMCI 0.66580 0.05961 11.17 < 2e-16 ***
diagn3LMCI 1.34868 0.05096 26.46 < 2e-16 ***
diagn4AD 2.22737 0.05843 38.12 < 2e-16 ***
arthrit -0.07433 0.03808 -1.95 0.051 .
aspirin -0.08246 0.03721 -2.22 0.027 *
Ibu -0.09906 0.05065 -1.96 0.051 .
---
Signif. codes: 0 '***' 0.001 '**' 0.01 '*' 0.05 '.' 0.1 ' ' 1

Number of observations: total=8878, ID=1619
Random effect variance(s):
Group=ID
 Variance StdDev
(Intercept) 0.4167 0.6455

Negative binomial dispersion parameter: 1.0961 (std. err.: 0.018812)

Log-likelihood: -17085.9

anova(main.M.combined.drop.headache, main.M.combined.drop.parac)

Analysis of Deviance Table

Model 1: neg.b.MMSE ~ M + AGE + APOE4 + Gender + edu.cat + diagn + arthrit + aspirin + Ibu
Model 2: neg.b.MMSE ~ M + AGE + APOE4 + Gender + edu.cat + diagn + arthrit + parac + aspirin + Ibu
 NoPar LogLik Df Deviance Pr(>Chi)
1 17 -17086
2 18 -17086 1 0.8 0.3711

### Gender

Dropping gender from the model did not significantly worsen the model

main.M.combined.drop.Gender<- glmmadmb(neg.b.MMSE~M+AGE+ APOE4+ edu.cat + diagn+ arthrit+ aspirin+Ibu+ (1|ID), family="nbinom1", data=MMSEdata)
summary(main.M.combined.drop.Gender)

Call:
glmmadmb(formula = neg.b.MMSE ~ M + AGE + APOE4 + edu.cat + diagn +
 arthrit + aspirin + Ibu + (1 | ID), data = MMSEdata, family = "nbinom1")

AIC: 34205.8

Coefficients:
 Estimate Std. Error z value Pr(>|z|)
(Intercept) -0.62546 0.05632 -11.11 < 2e-16 ***
M 0.01271 0.00029 43.90 < 2e-16 ***
AGE 0.01899 0.00261 7.27 3.5e-13 ***
APOE41 0.27082 0.04003 6.77 1.3e-11 ***
APOE42 0.40151 0.06231 6.44 1.2e-10 ***
edu.cat2tertiary 0.10977 0.04647 2.36 0.018 *
edu.cat3mid 0.30146 0.05145 5.86 4.6e-09 ***
edu.cat4early 0.29590 0.05424 5.46 4.9e-08 ***
diagn2EMCI 0.67059 0.05951 11.27 < 2e-16 ***
diagn3LMCI 1.35404 0.05082 26.65 < 2e-16 ***
diagn4AD 2.23096 0.05837 38.22 < 2e-16 ***
arthrit -0.07919 0.03792 -2.09 0.037 *
aspirin -0.07573 0.03689 -2.05 0.040 *
Ibu -0.09861 0.05064 -1.95 0.052 .
---
Signif. codes: 0 '***' 0.001 '**' 0.01 '*' 0.05 '.' 0.1 ' ' 1

Number of observations: total=8878, ID=1619
Random effect variance(s):
Group=ID
 Variance StdDev
(Intercept) 0.4165 0.6454

Negative binomial dispersion parameter: 1.0964 (std. err.: 0.018819)

Log-likelihood: -17086.9

anova(main.M.combined.drop.parac, main.M.combined.drop.Gender)

Analysis of Deviance Table

Model 1: neg.b.MMSE ~ M + AGE + APOE4 + edu.cat + diagn + arthrit + aspirin + Ibu
Model 2: neg.b.MMSE ~ M + AGE + APOE4 + Gender + edu.cat + diagn + arthrit + aspirin + Ibu
 NoPar LogLik Df Deviance Pr(>Chi)
1 16 -17087
2 17 -17086 1 2 0.1573

### Arthritis

Dropping arthritis from the model did significantly worsen the model

main.M.combined.drop.arthritis<- glmmadmb(neg.b.MMSE~M+AGE+ APOE4+edu.cat + diagn+ aspirin+Ibu+ (1|ID), family="nbinom1", data=MMSEdata)
summary(main.M.combined.drop.arthritis)

Call:
glmmadmb(formula = neg.b.MMSE ~ M + AGE + APOE4 + edu.cat + diagn +
 aspirin + Ibu + (1 | ID), data = MMSEdata, family = "nbinom1")

AIC: 34208.2

Coefficients:
 Estimate Std. Error z value Pr(>|z|)
(Intercept) -0.65402 0.05477 -11.94 < 2e-16 ***
M 0.01271 0.00029 43.88 < 2e-16 ***
AGE 0.01847 0.00260 7.10 1.3e-12 ***
APOE41 0.26812 0.04007 6.69 2.2e-11 ***
APOE42 0.40472 0.06238 6.49 8.7e-11 ***
edu.cat2tertiary 0.10544 0.04649 2.27 0.023 *
edu.cat3mid 0.29621 0.05146 5.76 8.6e-09 ***
edu.cat4early 0.28913 0.05422 5.33 9.7e-08 ***
diagn2EMCI 0.67134 0.05959 11.27 < 2e-16 ***
diagn3LMCI 1.35926 0.05083 26.74 < 2e-16 ***
diagn4AD 2.24004 0.05830 38.42 < 2e-16 ***
aspirin -0.07645 0.03694 -2.07 0.039 *
Ibu -0.10809 0.05051 -2.14 0.032 *
---
Signif. codes: 0 '***' 0.001 '**' 0.01 '*' 0.05 '.' 0.1 ' ' 1

Number of observations: total=8878, ID=1619
Random effect variance(s):
Group=ID
 Variance StdDev
(Intercept) 0.418 0.6466

Negative binomial dispersion parameter: 1.0964 (std. err.: 0.01882)

Log-likelihood: -17089.1

anova(main.M.combined.drop.Gender, main.M.combined.drop.arthritis)

Analysis of Deviance Table

Model 1: neg.b.MMSE ~ M + AGE + APOE4 + edu.cat + diagn + aspirin + Ibu
Model 2: neg.b.MMSE ~ M + AGE + APOE4 + edu.cat + diagn + arthrit + aspirin + Ibu
 NoPar LogLik Df Deviance Pr(>Chi)
1 15 -17089
2 16 -17087 1 4.4 0.03594 *
---
Signif. codes: 0 '***' 0.001 '**' 0.01 '*' 0.05 '.' 0.1 ' ' 1

### Ibuprofen

Dropping ibuprofen from the model did significantly worsen the model

main.M.combined.drop.Ibu<- glmmadmb(neg.b.MMSE~M+AGE+ APOE4+edu.cat + diagn+ arthrit+ aspirin+ (1|ID), family="nbinom1", data=MMSEdata)
summary(main.M.combined.drop.Ibu)

Call:
glmmadmb(formula = neg.b.MMSE ~ M + AGE + APOE4 + edu.cat + diagn +
 arthrit + aspirin + (1 | ID), data = MMSEdata, family = "nbinom1")

AIC: 34207.6

Coefficients:
 Estimate Std. Error z value Pr(>|z|)
(Intercept) -0.64036 0.05589 -11.46 < 2e-16 ***
M 0.01270 0.00029 43.86 < 2e-16 ***
AGE 0.01937 0.00261 7.43 1.1e-13 ***
APOE41 0.27249 0.04007 6.80 1.0e-11 ***
APOE42 0.40400 0.06237 6.48 9.3e-11 ***
edu.cat2tertiary 0.10734 0.04650 2.31 0.021 *
edu.cat3mid 0.30021 0.05150 5.83 5.6e-09 ***
edu.cat4early 0.29196 0.05426 5.38 7.4e-08 ***
diagn2EMCI 0.67264 0.05956 11.29 < 2e-16 ***
diagn3LMCI 1.35639 0.05086 26.67 < 2e-16 ***
diagn4AD 2.24025 0.05826 38.46 < 2e-16 ***
arthrit -0.08581 0.03781 -2.27 0.023 *
aspirin -0.07688 0.03693 -2.08 0.037 *
---
Signif. codes: 0 '***' 0.001 '**' 0.01 '*' 0.05 '.' 0.1 ' ' 1

Number of observations: total=8878, ID=1619
Random effect variance(s):
Group=ID
 Variance StdDev
(Intercept) 0.4177 0.6463

Negative binomial dispersion parameter: 1.0964 (std. err.: 0.018828)

Log-likelihood: -17088.8

anova(main.M.combined.drop.arthritis, main.M.combined.drop.Ibu)

Analysis of Deviance Table

Model 1: neg.b.MMSE ~ M + AGE + APOE4 + edu.cat + diagn + aspirin + Ibu
Model 2: neg.b.MMSE ~ M + AGE + APOE4 + edu.cat + diagn + arthrit + aspirin
 NoPar LogLik Df Deviance Pr(>Chi)
1 15 -17089
2 15 -17089 0 0.6 < 2.2e-16 ***
---
Signif. codes: 0 '***' 0.001 '**' 0.01 '*' 0.05 '.' 0.1 ' ' 1

### Aspirin

Dropping aspirin from the model did not significantly worsen the model

main.M.combined.drop.aspirin<- glmmadmb(neg.b.MMSE~M+AGE+ APOE4+edu.cat + diagn+ arthrit+ Ibu+ aspirin+ (1|ID), family="nbinom1", data=MMSEdata)
summary(main.M.combined.drop.aspirin)

Call:
glmmadmb(formula = neg.b.MMSE ~ M + AGE + APOE4 + edu.cat + diagn +
 arthrit + Ibu + aspirin + (1 | ID), data = MMSEdata, family = "nbinom1")

AIC: 34205.8

Coefficients:
 Estimate Std. Error z value Pr(>|z|)
(Intercept) -0.62546 0.05632 -11.11 < 2e-16 ***
M 0.01271 0.00029 43.90 < 2e-16 ***
AGE 0.01899 0.00261 7.27 3.5e-13 ***
APOE41 0.27082 0.04003 6.77 1.3e-11 ***
APOE42 0.40151 0.06231 6.44 1.2e-10 ***
edu.cat2tertiary 0.10977 0.04647 2.36 0.018 *
edu.cat3mid 0.30146 0.05145 5.86 4.6e-09 ***
edu.cat4early 0.29590 0.05424 5.46 4.9e-08 ***
diagn2EMCI 0.67059 0.05951 11.27 < 2e-16 ***
diagn3LMCI 1.35404 0.05082 26.65 < 2e-16 ***
diagn4AD 2.23096 0.05837 38.22 < 2e-16 ***
arthrit -0.07919 0.03792 -2.09 0.037 *
Ibu -0.09861 0.05064 -1.95 0.052 .
aspirin -0.07573 0.03689 -2.05 0.040 *
---
Signif. codes: 0 '***' 0.001 '**' 0.01 '*' 0.05 '.' 0.1 ' ' 1

Number of observations: total=8878, ID=1619
Random effect variance(s):
Group=ID
 Variance StdDev
(Intercept) 0.4165 0.6454

Negative binomial dispersion parameter: 1.0964 (std. err.: 0.018819)

Log-likelihood: -17086.9

anova(main.M.combined.drop.Ibu, main.M.combined.drop.aspirin)

Analysis of Deviance Table

Model 1: neg.b.MMSE ~ M + AGE + APOE4 + edu.cat + diagn + arthrit + aspirin
Model 2: neg.b.MMSE ~ M + AGE + APOE4 + edu.cat + diagn + arthrit + Ibu + aspirin
 NoPar LogLik Df Deviance Pr(>Chi)
1 15 -17089
2 16 -17087 1 3.8 0.05125 .
---
Signif. codes: 0 '***' 0.001 '**' 0.01 '*' 0.05 '.' 0.1 ' ' 1

## Building combined main effect model

main.M.final<- glmmadmb(neg.b.MMSE~ M+AGE+ APOE4+edu.cat + diagn+ arthrit+ Ibu+ aspirin+ (1|ID), family="nbinom1", data=MMSEdata)
summary(main.M.final)

Call:
glmmadmb(formula = neg.b.MMSE ~ M + AGE + APOE4 + edu.cat + diagn +
 arthrit + Ibu + aspirin + (1 | ID), data = MMSEdata, family = "nbinom1")

AIC: 34205.8

Coefficients:
 Estimate Std. Error z value Pr(>|z|)
(Intercept) -0.62546 0.05632 -11.11 < 2e-16 ***
M 0.01271 0.00029 43.90 < 2e-16 ***
AGE 0.01899 0.00261 7.27 3.5e-13 ***
APOE41 0.27082 0.04003 6.77 1.3e-11 ***
APOE42 0.40151 0.06231 6.44 1.2e-10 ***
edu.cat2tertiary 0.10977 0.04647 2.36 0.018 *
edu.cat3mid 0.30146 0.05145 5.86 4.6e-09 ***
edu.cat4early 0.29590 0.05424 5.46 4.9e-08 ***
diagn2EMCI 0.67059 0.05951 11.27 < 2e-16 ***
diagn3LMCI 1.35404 0.05082 26.65 < 2e-16 ***
diagn4AD 2.23096 0.05837 38.22 < 2e-16 ***
arthrit -0.07919 0.03792 -2.09 0.037 *
Ibu -0.09861 0.05064 -1.95 0.052 .
aspirin -0.07573 0.03689 -2.05 0.040 *
---
Signif. codes: 0 '***' 0.001 '**' 0.01 '*' 0.05 '.' 0.1 ' ' 1

Number of observations: total=8878, ID=1619
Random effect variance(s):
Group=ID
 Variance StdDev
(Intercept) 0.4165 0.6454

Negative binomial dispersion parameter: 1.0964 (std. err.: 0.018819)

Log-likelihood: -17086.9

anova(main.M.combined,main.M.final)

Analysis of Deviance Table

Model 1: neg.b.MMSE ~ M + AGE + APOE4 + edu.cat + diagn + arthrit + Ibu + aspirin
Model 2: neg.b.MMSE ~ M + AGE + APOE4 + Gender + edu.cat + diagn + headache + arthrit + diclo + parac + naprox + aspirin + Ibu
 NoPar LogLik Df Deviance Pr(>Chi)
1 16 -17087
2 21 -17085 5 4.6 0.4666

## Removing each explanitory variable in isolation

### Main effect of age at the start of the study

main.M.final.drop.AGE<- glmmadmb(neg.b.MMSE~ M+ APOE4+edu.cat + diagn+ arthrit+ Ibu+ aspirin+ (1|ID), family="nbinom1", data=MMSEdata)
anova(main.M.final,main.M.final.drop.AGE)

Analysis of Deviance Table

Model 1: neg.b.MMSE ~ M + APOE4 + edu.cat + diagn + arthrit + Ibu + aspirin
Model 2: neg.b.MMSE ~ M + AGE + APOE4 + edu.cat + diagn + arthrit + Ibu + aspirin
 NoPar LogLik Df Deviance Pr(>Chi)
1 15 -17113
2 16 -17087 1 52.6 4.089e-13 ***
---
Signif. codes: 0 '***' 0.001 '**' 0.01 '*' 0.05 '.' 0.1 ' ' 1

### Main effect of APOE4 genotype

main.M.final.drop.APOE4<- glmmadmb(neg.b.MMSE~M+AGE+ edu.cat + diagn+ arthrit+ Ibu+ aspirin+ (1|ID), family="nbinom1", data=MMSEdata)
anova(main.M.final.drop.APOE4,main.M.final)

Analysis of Deviance Table

Model 1: neg.b.MMSE ~ M + AGE + edu.cat + diagn + arthrit + Ibu + aspirin
Model 2: neg.b.MMSE ~ M + AGE + APOE4 + edu.cat + diagn + arthrit + Ibu + aspirin
 NoPar LogLik Df Deviance Pr(>Chi)
1 14 -17119
2 16 -17087 2 64.2 1.144e-14 ***
---
Signif. codes: 0 '***' 0.001 '**' 0.01 '*' 0.05 '.' 0.1 ' ' 1

### Main effect of education level

main.M.final.drop.edu.cat<- glmmadmb(neg.b.MMSE~ M+AGE+ APOE4+ diagn+ arthrit+ Ibu+ aspirin+ (1|ID), family="nbinom1", data=MMSEdata)
anova(main.M.final.drop.edu.cat,main.M.final)

Analysis of Deviance Table

Model 1: neg.b.MMSE ~ M + AGE + APOE4 + diagn + arthrit + Ibu + aspirin
Model 2: neg.b.MMSE ~ M + AGE + APOE4 + edu.cat + diagn + arthrit + Ibu + aspirin
 NoPar LogLik Df Deviance Pr(>Chi)
1 13 -17111
2 16 -17087 3 48.2 1.931e-10 ***
---
Signif. codes: 0 '***' 0.001 '**' 0.01 '*' 0.05 '.' 0.1 ' ' 1

### Main effect of initial Alzhiemer’s diagnosis

main.M.final.drop.diagn<- glmmadmb(neg.b.MMSE~ M+AGE+ APOE4+edu.cat + arthrit+ Ibu+ aspirin+ (1|ID), family="nbinom1", data=MMSEdata)
anova(main.M.final.drop.diagn,main.M.final)

Analysis of Deviance Table

Model 1: neg.b.MMSE ~ M + AGE + APOE4 + edu.cat + arthrit + Ibu + aspirin
Model 2: neg.b.MMSE ~ M + AGE + APOE4 + edu.cat + diagn + arthrit + Ibu + aspirin
 NoPar LogLik Df Deviance Pr(>Chi)
1 13 -17653
2 16 -17087 3 1132.8 < 2.2e-16 ***
---
Signif. codes: 0 '***' 0.001 '**' 0.01 '*' 0.05 '.' 0.1 ' ' 1

### Main effect of initial Arthritis

main.M.final.drop.arthrit<- glmmadmb(neg.b.MMSE~ M+AGE+ diagn+ APOE4+edu.cat + Ibu+ aspirin+ (1|ID), family="nbinom1", data=MMSEdata)
anova(main.M.final.drop.arthrit,main.M.final)

Analysis of Deviance Table

Model 1: neg.b.MMSE ~ M + AGE + diagn + APOE4 + edu.cat + Ibu + aspirin
Model 2: neg.b.MMSE ~ M + AGE + APOE4 + edu.cat + diagn + arthrit + Ibu + aspirin
 NoPar LogLik Df Deviance Pr(>Chi)
1 15 -17089
2 16 -17087 1 4.4 0.03594 *
---
Signif. codes: 0 '***' 0.001 '**' 0.01 '*' 0.05 '.' 0.1 ' ' 1

### Main effect of initial Ibuprofen

main.M.final.drop.Ibu<- glmmadmb(neg.b.MMSE~ M+AGE+ diagn+ APOE4+edu.cat + arthrit+ aspirin+ (1|ID), family="nbinom1", data=MMSEdata)
anova(main.M.final.drop.Ibu,main.M.final)

Analysis of Deviance Table

Model 1: neg.b.MMSE ~ M + AGE + diagn + APOE4 + edu.cat + arthrit + aspirin
Model 2: neg.b.MMSE ~ M + AGE + APOE4 + edu.cat + diagn + arthrit + Ibu + aspirin
 NoPar LogLik Df Deviance Pr(>Chi)
1 15 -17089
2 16 -17087 1 3.8 0.05125 .
---
Signif. codes: 0 '***' 0.001 '**' 0.01 '*' 0.05 '.' 0.1 ' ' 1

### Main effect of initial Aspirin

main.M.final.drop.aspirin<- glmmadmb(neg.b.MMSE~ M+AGE+ diagn+ APOE4+edu.cat + arthrit+ Ibu+ (1|ID), family="nbinom1", data=MMSEdata)
anova(main.M.final.drop.aspirin,main.M.final)

Analysis of Deviance Table

Model 1: neg.b.MMSE ~ M + AGE + diagn + APOE4 + edu.cat + arthrit + Ibu
Model 2: neg.b.MMSE ~ M + AGE + APOE4 + edu.cat + diagn + arthrit + Ibu + aspirin
 NoPar LogLik Df Deviance Pr(>Chi)
1 15 -17089
2 16 -17087 1 4.2 0.04042 *
---
Signif. codes: 0 '***' 0.001 '**' 0.01 '*' 0.05 '.' 0.1 ' ' 1

### AIC summary of main effect models

AIC(main.M.final,main.M.final.drop.AGE,main.M.final.drop.APOE4,main.M.final.drop.edu.cat,main.M.final.drop.diagn,main.M.final.drop.arthrit,main.M.final.drop.Ibu,main.M.final.drop.aspirin)

df AIC
main.M.final 16 34205.8
main.M.final.drop.AGE 15 34256.4
main.M.final.drop.APOE4 14 34266.0
main.M.final.drop.edu.cat 13 34248.0
main.M.final.drop.diagn 13 35332.6
main.M.final.drop.arthrit 15 34208.2
main.M.final.drop.Ibu 15 34207.6
main.M.final.drop.aspirin 15 34208.0

## Investigating interaction terms

### The effects of diagnosis on cognitive decline progression

neg.m.interaction.diagn<- glmmadmb(neg.b.MMSE~ M+AGE+ diagn+ APOE4+edu.cat + arthrit+ aspirin+ Ibu+ diagn*M+(1|ID), family="nbinom1", data=MMSEdata)
summary(neg.m.interaction.diagn)
anova(neg.m.interaction.diagn,main.M.final)

### The effects of smoking on cognitive decline progression

neg.m.interaction.smoke<- glmmadmb(neg.b.MMSE~ M+AGE+ diagn+ APOE4+edu.cat + arthrit+ aspirin+ Ibu+ smoke*M+(1|ID), family="nbinom1", data=MMSEdata)
summary(neg.m.interaction.smoke)
anova(neg.m.interaction.smoke,main.M.final)

### The effects of arthritis on cognitive decline progression

neg.m.interaction.arthrit<- glmmadmb(neg.b.MMSE~ M+AGE+ diagn+ APOE4+edu.cat + arthrit+ aspirin+ Ibu+ arthrit*M+(1|ID), family="nbinom1", data=MMSEdata)
summary(neg.m.interaction.arthrit)
anova(neg.m.interaction.arthrit, main.M.final)

### The effects of cardiovascular disease on cognitive decline progression

neg.m.interaction.vasc<- glmmadmb(neg.b.MMSE~ M+AGE+ diagn+ APOE4+edu.cat + arthrit+ aspirin+ Ibu+ vasc*M+(1|ID), family="nbinom1", data=MMSEdata)
summary(neg.m.interaction.vasc)
anova(neg.m.interaction.vasc, main.M.final)

### The effect of headaches on cognitive decline progression

neg.m.interaction.headache<- glmmadmb(neg.b.MMSE~ M+AGE+ diagn+ APOE4+edu.cat + arthrit+ aspirin+ Ibu+ headache*M+(1|ID), family="nbinom1", data=MMSEdata)
summary(neg.m.interaction.headache)

Call:
glmmadmb(formula = neg.b.MMSE ~ M + AGE + diagn + APOE4 + edu.cat +
 arthrit + aspirin + Ibu + headache * M + (1 | ID), data = MMSEdata,
 family = "nbinom1")

AIC: 34207.2

Coefficients:
 Estimate Std. Error z value Pr(>|z|)
(Intercept) -0.622894 0.056624 -11.00 < 2e-16 ***
M 0.012863 0.000311 41.34 < 2e-16 ***
AGE 0.018671 0.002631 7.10 1.3e-12 ***
diagn2EMCI 0.669791 0.059487 11.26 < 2e-16 ***
diagn3LMCI 1.354534 0.050799 26.66 < 2e-16 ***
diagn4AD 2.231267 0.058354 38.24 < 2e-16 ***
APOE41 0.269515 0.040043 6.73 1.7e-11 ***
APOE42 0.399837 0.062294 6.42 1.4e-10 ***
edu.cat2tertiary 0.108453 0.046464 2.33 0.020 *
edu.cat3mid 0.302485 0.051435 5.88 4.1e-09 ***
edu.cat4early 0.296776 0.054227 5.47 4.4e-08 ***
arthrit -0.076333 0.038034 -2.01 0.045 *
aspirin -0.076649 0.036884 -2.08 0.038 *
Ibu -0.097742 0.050634 -1.93 0.054 .
headache -0.040218 0.070122 -0.57 0.566
M:headache -0.001011 0.000817 -1.24 0.216
---
Signif. codes: 0 '***' 0.001 '**' 0.01 '*' 0.05 '.' 0.1 ' ' 1

Number of observations: total=8878, ID=1619
Random effect variance(s):
Group=ID
 Variance StdDev
(Intercept) 0.4162 0.6451

Negative binomial dispersion parameter: 1.0963 (std. err.: 0.018817)

Log-likelihood: -17085.6

anova(neg.m.interaction.headache, main.M.final)

Analysis of Deviance Table

Model 1: neg.b.MMSE ~ M + AGE + APOE4 + edu.cat + diagn + arthrit + Ibu + aspirin
Model 2: neg.b.MMSE ~ M + AGE + diagn + APOE4 + edu.cat + arthrit + aspirin + Ibu + headache * M
 NoPar LogLik Df Deviance Pr(>Chi)
1 16 -17087
2 18 -17086 2 2.6 0.2725

### The effect of diabetes on cognitive decline progression

neg.m.interaction.diabetes<- glmmadmb(neg.b.MMSE~ AGE + APOE4 + M + edu.cat + diab*M+(1|ID), family="nbinom1", data=MMSEdata)
summary(neg.m.interaction.headache)

Call:
glmmadmb(formula = neg.b.MMSE ~ M + AGE + diagn + APOE4 + edu.cat +
 arthrit + aspirin + Ibu + headache * M + (1 | ID), data = MMSEdata,
 family = "nbinom1")

AIC: 34207.2

Coefficients:
 Estimate Std. Error z value Pr(>|z|)
(Intercept) -0.622894 0.056624 -11.00 < 2e-16 ***
M 0.012863 0.000311 41.34 < 2e-16 ***
AGE 0.018671 0.002631 7.10 1.3e-12 ***
diagn2EMCI 0.669791 0.059487 11.26 < 2e-16 ***
diagn3LMCI 1.354534 0.050799 26.66 < 2e-16 ***
diagn4AD 2.231267 0.058354 38.24 < 2e-16 ***
APOE41 0.269515 0.040043 6.73 1.7e-11 ***
APOE42 0.399837 0.062294 6.42 1.4e-10 ***
edu.cat2tertiary 0.108453 0.046464 2.33 0.020 *
edu.cat3mid 0.302485 0.051435 5.88 4.1e-09 ***
edu.cat4early 0.296776 0.054227 5.47 4.4e-08 ***
arthrit -0.076333 0.038034 -2.01 0.045 *
aspirin -0.076649 0.036884 -2.08 0.038 *
Ibu -0.097742 0.050634 -1.93 0.054 .
headache -0.040218 0.070122 -0.57 0.566
M:headache -0.001011 0.000817 -1.24 0.216
---
Signif. codes: 0 '***' 0.001 '**' 0.01 '*' 0.05 '.' 0.1 ' ' 1

Number of observations: total=8878, ID=1619
Random effect variance(s):
Group=ID
 Variance StdDev
(Intercept) 0.4162 0.6451

Negative binomial dispersion parameter: 1.0963 (std. err.: 0.018817)

Log-likelihood: -17085.6

anova(neg.m.interaction.headache, main.M.final)

Analysis of Deviance Table

Model 1: neg.b.MMSE ~ M + AGE + APOE4 + edu.cat + diagn + arthrit + Ibu + aspirin
Model 2: neg.b.MMSE ~ M + AGE + diagn + APOE4 + edu.cat + arthrit + aspirin + Ibu + headache * M
 NoPar LogLik Df Deviance Pr(>Chi)
1 16 -17087
2 18 -17086 2 2.6 0.2725

### The effect of AGE on cognitive decline progression

neg.m.interaction.AGE<- glmmadmb(neg.b.MMSE~ M+AGE+ diagn+ APOE4+edu.cat + arthrit+ aspirin+ Ibu+ AGE*M+(1|ID), family="nbinom1", data=MMSEdata)
summary(neg.m.interaction.AGE)

Call:
glmmadmb(formula = neg.b.MMSE ~ M + AGE + diagn + APOE4 + edu.cat +
 arthrit + aspirin + Ibu + AGE * M + (1 | ID), data = MMSEdata,
 family = "nbinom1")

AIC: 34206.4

Coefficients:
 Estimate Std. Error z value Pr(>|z|)
(Intercept) -6.25e-01 5.63e-02 -11.10 < 2e-16 ***
M 1.27e-02 2.89e-04 43.91 < 2e-16 ***
AGE 2.01e-02 2.76e-03 7.26 3.8e-13 ***
diagn2EMCI 6.69e-01 5.95e-02 11.23 < 2e-16 ***
diagn3LMCI 1.35e+00 5.08e-02 26.61 < 2e-16 ***
diagn4AD 2.23e+00 5.84e-02 38.18 < 2e-16 ***
APOE41 2.71e-01 4.00e-02 6.76 1.4e-11 ***
APOE42 4.02e-01 6.23e-02 6.45 1.1e-10 ***
edu.cat2tertiary 1.10e-01 4.65e-02 2.37 0.018 *
edu.cat3mid 3.02e-01 5.15e-02 5.87 4.3e-09 ***
edu.cat4early 2.96e-01 5.42e-02 5.46 4.8e-08 ***
arthrit -7.91e-02 3.79e-02 -2.09 0.037 *
aspirin -7.51e-02 3.69e-02 -2.04 0.042 *
Ibu -9.83e-02 5.07e-02 -1.94 0.052 .
M:AGE -4.95e-05 4.15e-05 -1.19 0.233
---
Signif. codes: 0 '***' 0.001 '**' 0.01 '*' 0.05 '.' 0.1 ' ' 1

Number of observations: total=8878, ID=1619
Random effect variance(s):
Group=ID
 Variance StdDev
(Intercept) 0.4168 0.6456

Negative binomial dispersion parameter: 1.0958 (std. err.: 0.018799)

Log-likelihood: -17086.2

anova(neg.m.interaction.AGE, main.M.final)

Analysis of Deviance Table

Model 1: neg.b.MMSE ~ M + AGE + APOE4 + edu.cat + diagn + arthrit + Ibu + aspirin
Model 2: neg.b.MMSE ~ M + AGE + diagn + APOE4 + edu.cat + arthrit + aspirin + Ibu + AGE * M
 NoPar LogLik Df Deviance Pr(>Chi)
1 16 -17087
2 17 -17086 1 1.4 0.2367

### The effect of APOE status on cognitive decline progression

neg.m.interaction.APOE4<- glmmadmb(neg.b.MMSE~ M+AGE+ diagn+ APOE4+edu.cat + arthrit+ aspirin+ Ibu+ APOE4*M+(1|ID), family="nbinom1", data=MMSEdata)
summary(neg.m.interaction.APOE4)

Call:
glmmadmb(formula = neg.b.MMSE ~ M + AGE + diagn + APOE4 + edu.cat +
 arthrit + aspirin + Ibu + APOE4 * M + (1 | ID), data = MMSEdata,
 family = "nbinom1")

AIC: 34035.2

Coefficients:
 Estimate Std. Error z value Pr(>|z|)
(Intercept) -0.512972 0.056183 -9.13 < 2e-16 ***
M 0.008965 0.000409 21.91 < 2e-16 ***
AGE 0.018754 0.002583 7.26 3.8e-13 ***
diagn2EMCI 0.645102 0.058866 10.96 < 2e-16 ***
diagn3LMCI 1.328945 0.050267 26.44 < 2e-16 ***
diagn4AD 2.220171 0.057702 38.48 < 2e-16 ***
APOE41 0.106553 0.042195 2.53 0.0116 *
APOE42 0.200645 0.064379 3.12 0.0018 **
edu.cat2tertiary 0.107548 0.045957 2.34 0.0193 *
edu.cat3mid 0.300319 0.050880 5.90 3.6e-09 ***
edu.cat4early 0.293369 0.053636 5.47 4.5e-08 ***
arthrit -0.075767 0.037501 -2.02 0.0433 *
aspirin -0.070526 0.036481 -1.93 0.0532 .
Ibu -0.087739 0.050090 -1.75 0.0798 .
M:APOE41 0.006697 0.000605 11.07 < 2e-16 ***
M:APOE42 0.009104 0.000888 10.25 < 2e-16 ***
---
Signif. codes: 0 '***' 0.001 '**' 0.01 '*' 0.05 '.' 0.1 ' ' 1

Number of observations: total=8878, ID=1619
Random effect variance(s):
Group=ID
 Variance StdDev
(Intercept) 0.4071 0.6381

Negative binomial dispersion parameter: 1.0786 (std. err.: 0.018495)

Log-likelihood: -16999.6

anova(neg.m.interaction.APOE4, main.M.final)

Analysis of Deviance Table

Model 1: neg.b.MMSE ~ M + AGE + APOE4 + edu.cat + diagn + arthrit + Ibu + aspirin
Model 2: neg.b.MMSE ~ M + AGE + diagn + APOE4 + edu.cat + arthrit + aspirin + Ibu + APOE4 * M
 NoPar LogLik Df Deviance Pr(>Chi)
1 16 -17087
2 18 -17000 2 174.6 < 2.2e-16 ***
---
Signif. codes: 0 '***' 0.001 '**' 0.01 '*' 0.05 '.' 0.1 ' ' 1

### The effect of education status on cognitive decline progression

neg.m.interaction.edu.cat<- glmmadmb(neg.b.MMSE~ M+AGE+ diagn+ APOE4+edu.cat + arthrit+ aspirin+ Ibu+ edu.cat*M+(1|ID), family="nbinom1", data=MMSEdata)
summary(neg.m.interaction.edu.cat)

Call:
glmmadmb(formula = neg.b.MMSE ~ M + AGE + diagn + APOE4 + edu.cat +
 arthrit + aspirin + Ibu + edu.cat * M + (1 | ID), data = MMSEdata,
 family = "nbinom1")

AIC: 34190.4

Coefficients:
 Estimate Std. Error z value Pr(>|z|)
(Intercept) -0.666744 0.057715 -11.55 < 2e-16 ***
M 0.014305 0.000512 27.92 < 2e-16 ***
AGE 0.019084 0.002619 7.29 3.2e-13 ***
diagn2EMCI 0.671285 0.059658 11.25 < 2e-16 ***
diagn3LMCI 1.353628 0.050977 26.55 < 2e-16 ***
diagn4AD 2.228878 0.058533 38.08 < 2e-16 ***
APOE41 0.272754 0.040149 6.79 1.1e-11 ***
APOE42 0.400370 0.062504 6.41 1.5e-10 ***
edu.cat2tertiary 0.195640 0.050160 3.90 9.6e-05 ***
edu.cat3mid 0.347488 0.055431 6.27 3.6e-10 ***
edu.cat4early 0.332884 0.057500 5.79 7.1e-09 ***
arthrit -0.080200 0.038028 -2.11 0.035 *
aspirin -0.077685 0.037002 -2.10 0.036 *
Ibu -0.096494 0.050786 -1.90 0.057 .
M:edu.cat2tertiary -0.003435 0.000743 -4.62 3.8e-06 ***
M:edu.cat3mid -0.001761 0.000788 -2.24 0.025 *
M:edu.cat4early -0.001364 0.000800 -1.71 0.088 .
---
Signif. codes: 0 '***' 0.001 '**' 0.01 '*' 0.05 '.' 0.1 ' ' 1

Number of observations: total=8878, ID=1619
Random effect variance(s):
Group=ID
 Variance StdDev
(Intercept) 0.4198 0.6479

Negative binomial dispersion parameter: 1.0905 (std. err.: 0.018704)

Log-likelihood: -17076.2

anova(neg.m.interaction.edu.cat, main.M.final)

Analysis of Deviance Table

Model 1: neg.b.MMSE ~ M + AGE + APOE4 + edu.cat + diagn + arthrit + Ibu + aspirin
Model 2: neg.b.MMSE ~ M + AGE + diagn + APOE4 + edu.cat + arthrit + aspirin + Ibu + edu.cat * M
 NoPar LogLik Df Deviance Pr(>Chi)
1 16 -17087
2 19 -17076 3 21.4 8.694e-05 ***
---
Signif. codes: 0 '***' 0.001 '**' 0.01 '*' 0.05 '.' 0.1 ' ' 1

### The effect of diabetes on cognitive decline progression

neg.m.interaction.diab<- glmmadmb(neg.b.MMSE~ M+AGE+ diagn+ APOE4+edu.cat + arthrit+ aspirin+ Ibu+ diab*M+(1|ID), family="nbinom1", data=MMSEdata)
summary(neg.m.interaction.diab)

Call:
glmmadmb(formula = neg.b.MMSE ~ M + AGE + diagn + APOE4 + edu.cat +
 arthrit + aspirin + Ibu + diab * M + (1 | ID), data = MMSEdata,
 family = "nbinom1")

AIC: 34206.8

Coefficients:
 Estimate Std. Error z value Pr(>|z|)
(Intercept) -0.630872 0.056409 -11.18 < 2e-16 ***
M 0.012798 0.000304 42.08 < 2e-16 ***
AGE 0.019139 0.002613 7.32 2.4e-13 ***
diagn2EMCI 0.667306 0.059556 11.20 < 2e-16 ***
diagn3LMCI 1.353897 0.050812 26.65 < 2e-16 ***
diagn4AD 2.230897 0.058365 38.22 < 2e-16 ***
APOE41 0.270920 0.040030 6.77 1.3e-11 ***
APOE42 0.405501 0.062352 6.50 7.9e-11 ***
edu.cat2tertiary 0.104437 0.046588 2.24 0.025 *
edu.cat3mid 0.298771 0.051462 5.81 6.4e-09 ***
edu.cat4early 0.290395 0.054327 5.35 9.0e-08 ***
arthrit -0.080695 0.037944 -2.13 0.033 *
aspirin -0.078968 0.036950 -2.14 0.033 *
Ibu -0.099308 0.050641 -1.96 0.050 *
diab 0.117440 0.067135 1.75 0.080 .
M:diab -0.000860 0.000961 -0.90 0.371
---
Signif. codes: 0 '***' 0.001 '**' 0.01 '*' 0.05 '.' 0.1 ' ' 1

Number of observations: total=8878, ID=1619
Random effect variance(s):
Group=ID
 Variance StdDev
(Intercept) 0.4164 0.6453

Negative binomial dispersion parameter: 1.0962 (std. err.: 0.01881)

Log-likelihood: -17085.4

anova(neg.m.interaction.diab, main.M.final)

Analysis of Deviance Table

Model 1: neg.b.MMSE ~ M + AGE + APOE4 + edu.cat + diagn + arthrit + Ibu + aspirin
Model 2: neg.b.MMSE ~ M + AGE + diagn + APOE4 + edu.cat + arthrit + aspirin + Ibu + diab * M
 NoPar LogLik Df Deviance Pr(>Chi)
1 16 -17087
2 18 -17085 2 3 0.2231

### The effect of Gender on cognitive decline progression

neg.m.interaction.Gender<- glmmadmb(neg.b.MMSE~ M+AGE+ diagn+ APOE4+edu.cat + arthrit+ aspirin+ Ibu+ Gender*M+(1|ID), family="nbinom1", data=MMSEdata)
summary(neg.m.interaction.Gender)

Call:
glmmadmb(formula = neg.b.MMSE ~ M + AGE + diagn + APOE4 + edu.cat +
 arthrit + aspirin + Ibu + Gender * M + (1 | ID), data = MMSEdata,
 family = "nbinom1")

AIC: 34200

Coefficients:
 Estimate Std. Error z value Pr(>|z|)
(Intercept) -0.679186 0.060904 -11.15 < 2e-16 ***
M 0.013618 0.000432 31.54 < 2e-16 ***
AGE 0.018415 0.002631 7.00 2.6e-12 ***
diagn2EMCI 0.666937 0.059570 11.20 < 2e-16 ***
diagn3LMCI 1.350372 0.050927 26.52 < 2e-16 ***
diagn4AD 2.229036 0.058386 38.18 < 2e-16 ***
APOE41 0.269898 0.040007 6.75 1.5e-11 ***
APOE42 0.398775 0.062284 6.40 1.5e-10 ***
edu.cat2tertiary 0.112503 0.046499 2.42 0.0155 *
edu.cat3mid 0.312253 0.052225 5.98 2.2e-09 ***
edu.cat4early 0.308158 0.054825 5.62 1.9e-08 ***
arthrit -0.074219 0.038047 -1.95 0.0511 .
aspirin -0.080611 0.037178 -2.17 0.0301 *
Ibu -0.099996 0.050611 -1.98 0.0482 *
GenderMale 0.092803 0.040687 2.28 0.0226 *
M:GenderMale -0.001598 0.000568 -2.81 0.0049 **
---
Signif. codes: 0 '***' 0.001 '**' 0.01 '*' 0.05 '.' 0.1 ' ' 1

Number of observations: total=8878, ID=1619
Random effect variance(s):
Group=ID
 Variance StdDev
(Intercept) 0.4159 0.6449

Negative binomial dispersion parameter: 1.0949 (std. err.: 0.018787)

Log-likelihood: -17082

anova(neg.m.interaction.Gender, main.M.final)

Analysis of Deviance Table

Model 1: neg.b.MMSE ~ M + AGE + APOE4 + edu.cat + diagn + arthrit + Ibu + aspirin
Model 2: neg.b.MMSE ~ M + AGE + diagn + APOE4 + edu.cat + arthrit + aspirin + Ibu + Gender * M
 NoPar LogLik Df Deviance Pr(>Chi)
1 16 -17087
2 18 -17082 2 9.8 0.007447 **
---
Signif. codes: 0 '***' 0.001 '**' 0.01 '*' 0.05 '.' 0.1 ' ' 1

### The effect of aspirin on cognitive decline progression

neg.m.interaction.aspirin<- glmmadmb(neg.b.MMSE~ M+AGE+ diagn+ APOE4+edu.cat + arthrit+ aspirin+ Ibu+ aspirin*M+(1|ID), family="nbinom1", data=MMSEdata)
summary(neg.m.interaction.aspirin)

Call:
glmmadmb(formula = neg.b.MMSE ~ M + AGE + diagn + APOE4 + edu.cat +
 arthrit + aspirin + Ibu + aspirin * M + (1 | ID), data = MMSEdata,
 family = "nbinom1")

AIC: 34207.8

Coefficients:
 Estimate Std. Error z value Pr(>|z|)
(Intercept) -6.25e-01 5.70e-02 -10.97 < 2e-16 ***
M 1.27e-02 5.14e-04 24.70 < 2e-16 ***
AGE 1.90e-02 2.61e-03 7.27 3.5e-13 ***
diagn2EMCI 6.71e-01 5.95e-02 11.27 < 2e-16 ***
diagn3LMCI 1.35e+00 5.08e-02 26.64 < 2e-16 ***
diagn4AD 2.23e+00 5.84e-02 38.20 < 2e-16 ***
APOE41 2.71e-01 4.00e-02 6.77 1.3e-11 ***
APOE42 4.01e-01 6.23e-02 6.44 1.2e-10 ***
edu.cat2tertiary 1.10e-01 4.65e-02 2.36 0.018 *
edu.cat3mid 3.01e-01 5.15e-02 5.86 4.6e-09 ***
edu.cat4early 2.96e-01 5.42e-02 5.46 4.9e-08 ***
arthrit -7.92e-02 3.79e-02 -2.09 0.037 *
aspirin -7.64e-02 3.94e-02 -1.94 0.053 .
Ibu -9.86e-02 5.06e-02 -1.95 0.051 .
M:aspirin 2.98e-05 6.12e-04 0.05 0.961
---
Signif. codes: 0 '***' 0.001 '**' 0.01 '*' 0.05 '.' 0.1 ' ' 1

Number of observations: total=8878, ID=1619
Random effect variance(s):
Group=ID
 Variance StdDev
(Intercept) 0.4165 0.6454

Negative binomial dispersion parameter: 1.0964 (std. err.: 0.01882)

Log-likelihood: -17086.9

anova(neg.m.interaction.aspirin, main.M.final)

Analysis of Deviance Table

Model 1: neg.b.MMSE ~ M + AGE + APOE4 + edu.cat + diagn + arthrit + Ibu + aspirin
Model 2: neg.b.MMSE ~ M + AGE + diagn + APOE4 + edu.cat + arthrit + aspirin + Ibu + aspirin * M
 NoPar LogLik Df Deviance Pr(>Chi)
1 16 -17087
2 17 -17087 1 0 1

### The effect of paracetamol on cognitive decline progression

neg.m.interaction.parac<- glmmadmb(neg.b.MMSE~ M+AGE+ diagn+ APOE4+edu.cat + arthrit+ aspirin+ Ibu+ parac*M+(1|ID), family="nbinom1", data=MMSEdata)
summary(neg.m.interaction.parac)

Call:
glmmadmb(formula = neg.b.MMSE ~ M + AGE + diagn + APOE4 + edu.cat +
 arthrit + aspirin + Ibu + parac * M + (1 | ID), data = MMSEdata,
 family = "nbinom1")

AIC: 34205.6

Coefficients:
 Estimate Std. Error z value Pr(>|z|)
(Intercept) -0.608599 0.056903 -10.70 < 2e-16 ***
M 0.012330 0.000367 33.64 < 2e-16 ***
AGE 0.018980 0.002608 7.28 3.4e-13 ***
diagn2EMCI 0.670032 0.059424 11.28 < 2e-16 ***
diagn3LMCI 1.353505 0.050738 26.68 < 2e-16 ***
diagn4AD 2.227485 0.058308 38.20 < 2e-16 ***
APOE41 0.267892 0.040016 6.69 2.2e-11 ***
APOE42 0.398512 0.062219 6.40 1.5e-10 ***
edu.cat2tertiary 0.111113 0.046405 2.39 0.017 *
edu.cat3mid 0.303188 0.051418 5.90 3.7e-09 ***
edu.cat4early 0.294959 0.054149 5.45 5.1e-08 ***
arthrit -0.071750 0.038318 -1.87 0.061 .
aspirin -0.072595 0.036917 -1.97 0.049 *
Ibu -0.094215 0.050770 -1.86 0.063 .
parac -0.073662 0.045911 -1.60 0.109
M:parac 0.001028 0.000582 1.77 0.077 .
---
Signif. codes: 0 '***' 0.001 '**' 0.01 '*' 0.05 '.' 0.1 ' ' 1

Number of observations: total=8878, ID=1619
Random effect variance(s):
Group=ID
 Variance StdDev
(Intercept) 0.415 0.6442

Negative binomial dispersion parameter: 1.0963 (std. err.: 0.018823)

Log-likelihood: -17084.8

anova(neg.m.interaction.parac, main.M.final)

Analysis of Deviance Table

Model 1: neg.b.MMSE ~ M + AGE + APOE4 + edu.cat + diagn + arthrit + Ibu + aspirin
Model 2: neg.b.MMSE ~ M + AGE + diagn + APOE4 + edu.cat + arthrit + aspirin + Ibu + parac * M
 NoPar LogLik Df Deviance Pr(>Chi)
1 16 -17087
2 18 -17085 2 4.2 0.1225

### The effect of diclofenac on cognitive decline progression

neg.m.interaction.diclo<- glmmadmb(neg.b.MMSE~ M+AGE+ diagn+ APOE4+edu.cat + arthrit+ aspirin+ Ibu+ diclo*M+(1|ID), family="nbinom1", data=MMSEdata)
summary(neg.m.interaction.diclo)

Call:
glmmadmb(formula = neg.b.MMSE ~ M + AGE + diagn + APOE4 + edu.cat +
 arthrit + aspirin + Ibu + diclo * M + (1 | ID), data = MMSEdata,
 family = "nbinom1")

AIC: 34203.6

Coefficients:
 Estimate Std. Error z value Pr(>|z|)
(Intercept) -0.626274 0.056361 -11.11 < 2e-16 ***
M 0.012841 0.000294 43.63 < 2e-16 ***
AGE 0.018991 0.002612 7.27 3.6e-13 ***
diagn2EMCI 0.668420 0.059541 11.23 < 2e-16 ***
diagn3LMCI 1.351852 0.050875 26.57 < 2e-16 ***
diagn4AD 2.229460 0.058438 38.15 < 2e-16 ***
APOE41 0.270280 0.040036 6.75 1.5e-11 ***
APOE42 0.402109 0.062312 6.45 1.1e-10 ***
edu.cat2tertiary 0.110101 0.046477 2.37 0.018 *
edu.cat3mid 0.299486 0.051475 5.82 6.0e-09 ***
edu.cat4early 0.297169 0.054250 5.48 4.3e-08 ***
arthrit -0.075956 0.038107 -1.99 0.046 *
aspirin -0.074011 0.036924 -2.00 0.045 *
Ibu -0.099897 0.050649 -1.97 0.049 *
diclo -0.002017 0.148740 -0.01 0.989
M:diclo -0.003669 0.001586 -2.31 0.021 *
---
Signif. codes: 0 '***' 0.001 '**' 0.01 '*' 0.05 '.' 0.1 ' ' 1

Number of observations: total=8878, ID=1619
Random effect variance(s):
Group=ID
 Variance StdDev
(Intercept) 0.4167 0.6455

Negative binomial dispersion parameter: 1.0961 (std. err.: 0.018805)

Log-likelihood: -17083.8

anova(neg.m.interaction.diclo, main.M.final)

Analysis of Deviance Table

Model 1: neg.b.MMSE ~ M + AGE + APOE4 + edu.cat + diagn + arthrit + Ibu + aspirin
Model 2: neg.b.MMSE ~ M + AGE + diagn + APOE4 + edu.cat + arthrit + aspirin + Ibu + diclo * M
 NoPar LogLik Df Deviance Pr(>Chi)
1 16 -17087
2 18 -17084 2 6.2 0.04505 *
---
Signif. codes: 0 '***' 0.001 '**' 0.01 '*' 0.05 '.' 0.1 ' ' 1

### The effect of ibuprofen on cognitive decline progression

neg.m.interaction.Ibu<- glmmadmb(neg.b.MMSE~ M+AGE+ diagn+ APOE4+edu.cat + arthrit+ aspirin+ Ibu+ Ibu*M+(1|ID), family="nbinom1", data=MMSEdata)
summary(neg.m.interaction.Ibu)

Call:
glmmadmb(formula = neg.b.MMSE ~ M + AGE + diagn + APOE4 + edu.cat +
 arthrit + aspirin + Ibu + Ibu * M + (1 | ID), data = MMSEdata,
 family = "nbinom1")

AIC: 34206

Coefficients:
 Estimate Std. Error z value Pr(>|z|)
(Intercept) -0.630697 0.056465 -11.17 < 2e-16 ***
M 0.012919 0.000326 39.63 < 2e-16 ***
AGE 0.019030 0.002612 7.28 3.2e-13 ***
diagn2EMCI 0.670231 0.059521 11.26 < 2e-16 ***
diagn3LMCI 1.354026 0.050830 26.64 < 2e-16 ***
diagn4AD 2.232670 0.058398 38.23 < 2e-16 ***
APOE41 0.270362 0.040045 6.75 1.5e-11 ***
APOE42 0.401170 0.062327 6.44 1.2e-10 ***
edu.cat2tertiary 0.110195 0.046480 2.37 0.018 *
edu.cat3mid 0.302288 0.051464 5.87 4.3e-09 ***
edu.cat4early 0.296246 0.054252 5.46 4.7e-08 ***
arthrit -0.079526 0.037928 -2.10 0.036 *
aspirin -0.075577 0.036900 -2.05 0.041 *
Ibu -0.070843 0.054549 -1.30 0.194
M:Ibu -0.000933 0.000684 -1.36 0.173
---
Signif. codes: 0 '***' 0.001 '**' 0.01 '*' 0.05 '.' 0.1 ' ' 1

Number of observations: total=8878, ID=1619
Random effect variance(s):
Group=ID
 Variance StdDev
(Intercept) 0.4168 0.6456

Negative binomial dispersion parameter: 1.0962 (std. err.: 0.01881)

Log-likelihood: -17086

anova(neg.m.interaction.Ibu, main.M.final)

Analysis of Deviance Table

Model 1: neg.b.MMSE ~ M + AGE + APOE4 + edu.cat + diagn + arthrit + Ibu + aspirin
Model 2: neg.b.MMSE ~ M + AGE + diagn + APOE4 + edu.cat + arthrit + aspirin + Ibu + Ibu * M
 NoPar LogLik Df Deviance Pr(>Chi)
1 16 -17087
2 17 -17086 1 1.8 0.1797

### The effect of naproxin on cognitive decline progression

neg.m.interaction.naprox<- glmmadmb(neg.b.MMSE~ M+AGE+ diagn+ APOE4+edu.cat + arthrit+ aspirin+ Ibu+ naprox*M+(1|ID), family="nbinom1", data=MMSEdata)
summary(neg.m.interaction.naprox)

Call:
glmmadmb(formula = neg.b.MMSE ~ M + AGE + diagn + APOE4 + edu.cat +
 arthrit + aspirin + Ibu + naprox * M + (1 | ID), data = MMSEdata,
 family = "nbinom1")

AIC: 34209.8

Coefficients:
 Estimate Std. Error z value Pr(>|z|)
(Intercept) -6.24e-01 5.66e-02 -11.04 < 2e-16 ***
M 1.27e-02 3.21e-04 39.60 < 2e-16 ***
AGE 1.90e-02 2.62e-03 7.25 4.1e-13 ***
diagn2EMCI 6.71e-01 5.95e-02 11.27 < 2e-16 ***
diagn3LMCI 1.35e+00 5.08e-02 26.65 < 2e-16 ***
diagn4AD 2.23e+00 5.84e-02 38.16 < 2e-16 ***
APOE41 2.71e-01 4.00e-02 6.76 1.4e-11 ***
APOE42 4.01e-01 6.23e-02 6.44 1.2e-10 ***
edu.cat2tertiary 1.10e-01 4.65e-02 2.37 0.018 *
edu.cat3mid 3.02e-01 5.15e-02 5.86 4.6e-09 ***
edu.cat4early 2.96e-01 5.42e-02 5.46 4.8e-08 ***
arthrit -7.85e-02 3.81e-02 -2.06 0.039 *
aspirin -7.55e-02 3.69e-02 -2.05 0.041 *
Ibu -9.80e-02 5.08e-02 -1.93 0.054 .
naprox -1.21e-02 6.16e-02 -0.20 0.844
M:naprox 5.31e-05 7.24e-04 0.07 0.942
---
Signif. codes: 0 '***' 0.001 '**' 0.01 '*' 0.05 '.' 0.1 ' ' 1

Number of observations: total=8878, ID=1619
Random effect variance(s):
Group=ID
 Variance StdDev
(Intercept) 0.4165 0.6454

Negative binomial dispersion parameter: 1.0965 (std. err.: 0.01882)

Log-likelihood: -17086.9

anova(neg.m.interaction.naprox, main.M.final)

Analysis of Deviance Table

Model 1: neg.b.MMSE ~ M + AGE + APOE4 + edu.cat + diagn + arthrit + Ibu + aspirin
Model 2: neg.b.MMSE ~ M + AGE + diagn + APOE4 + edu.cat + arthrit + aspirin + Ibu + naprox * M
 NoPar LogLik Df Deviance Pr(>Chi)
1 16 -17087
2 18 -17087 2 0 1

### The effect of celecoxib on cognitive decline progression

neg.m.interaction.celex<- glmmadmb(neg.b.MMSE~ M+AGE+ diagn+ APOE4+edu.cat + arthrit+ aspirin+ Ibu+ celex*M+(1|ID), family="nbinom1", data=MMSEdata)
summary(neg.m.interaction.celex)

Call:
glmmadmb(formula = neg.b.MMSE ~ M + AGE + diagn + APOE4 + edu.cat +
 arthrit + aspirin + Ibu + celex * M + (1 | ID), data = MMSEdata,
 family = "nbinom1")

AIC: 34207

Coefficients:
 Estimate Std. Error z value Pr(>|z|)
(Intercept) -0.625735 0.056319 -11.11 < 2e-16 ***
M 0.012803 0.000297 43.07 < 2e-16 ***
AGE 0.018867 0.002612 7.22 5.1e-13 ***
diagn2EMCI 0.669602 0.059478 11.26 < 2e-16 ***
diagn3LMCI 1.354011 0.050790 26.66 < 2e-16 ***
diagn4AD 2.229325 0.058368 38.19 < 2e-16 ***
APOE41 0.270877 0.040009 6.77 1.3e-11 ***
APOE42 0.401966 0.062275 6.45 1.1e-10 ***
edu.cat2tertiary 0.109332 0.046445 2.35 0.019 *
edu.cat3mid 0.301778 0.051418 5.87 4.4e-09 ***
edu.cat4early 0.298141 0.054238 5.50 3.9e-08 ***
arthrit -0.072957 0.038294 -1.91 0.057 .
aspirin -0.074538 0.036909 -2.02 0.043 *
Ibu -0.102492 0.050722 -2.02 0.043 *
celex -0.055192 0.102170 -0.54 0.589
M:celex -0.001603 0.001258 -1.27 0.203
---
Signif. codes: 0 '***' 0.001 '**' 0.01 '*' 0.05 '.' 0.1 ' ' 1

Number of observations: total=8878, ID=1619
Random effect variance(s):
Group=ID
 Variance StdDev
(Intercept) 0.416 0.645

Negative binomial dispersion parameter: 1.0961 (std. err.: 0.018814)

Log-likelihood: -17085.5

anova(neg.m.interaction.celex, main.M.final)

Analysis of Deviance Table

Model 1: neg.b.MMSE ~ M + AGE + APOE4 + edu.cat + diagn + arthrit + Ibu + aspirin
Model 2: neg.b.MMSE ~ M + AGE + diagn + APOE4 + edu.cat + arthrit + aspirin + Ibu + celex * M
 NoPar LogLik Df Deviance Pr(>Chi)
1 16 -17087
2 18 -17086 2 2.8 0.2466

## Combined interaction model

neg.m.combined<- glmmadmb(neg.b.MMSE~ M+AGE+ diagn+ APOE4+edu.cat + arthrit+ aspirin+ Ibu+ diagn*M +smoke*M +APOE4*M + Gender*M+ edu.cat*M +parac*M +diclo*M+(1|ID), family="nbinom1", data=MMSEdata)
summary(neg.m.combined)

Call:
glmmadmb(formula = neg.b.MMSE ~ M + AGE + diagn + APOE4 + edu.cat +
 arthrit + aspirin + Ibu + diagn * M + smoke * M + APOE4 *
 M + Gender * M + edu.cat * M + parac * M + diclo * M + (1 |
 ID), data = MMSEdata, family = "nbinom1")

AIC: 33938

Coefficients:
 Estimate Std. Error z value Pr(>|z|)
(Intercept) -0.558108 0.065338 -8.54 < 2e-16 ***
M 0.010455 0.000882 11.85 < 2e-16 ***
AGE 0.018189 0.002613 6.96 3.4e-12 ***
diagn2EMCI 0.708477 0.065683 10.79 < 2e-16 ***
diagn3LMCI 1.237416 0.055474 22.31 < 2e-16 ***
diagn4AD 2.092022 0.062269 33.60 < 2e-16 ***
APOE41 0.122878 0.042414 2.90 0.00377 **
APOE42 0.224098 0.064831 3.46 0.00055 ***
edu.cat2tertiary 0.203593 0.049595 4.11 4.0e-05 ***
edu.cat3mid 0.363400 0.055678 6.53 6.7e-11 ***
edu.cat4early 0.359314 0.057532 6.25 4.2e-10 ***
arthrit -0.061424 0.038388 -1.60 0.10958
aspirin -0.073283 0.037104 -1.98 0.04826 *
Ibu -0.082645 0.050465 -1.64 0.10149
smoke 0.023716 0.044411 0.53 0.59334
GenderMale 0.089032 0.040831 2.18 0.02922 *
parac -0.075116 0.045679 -1.64 0.10009
diclo 0.045664 0.146630 0.31 0.75548
M:diagn2EMCI -0.003342 0.001044 -3.20 0.00137 **
M:diagn3LMCI 0.002840 0.000726 3.91 9.2e-05 ***
M:diagn4AD 0.008767 0.001438 6.10 1.1e-09 ***
M:smoke -0.000279 0.000643 -0.43 0.66454
M:APOE41 0.005847 0.000618 9.46 < 2e-16 ***
M:APOE42 0.007730 0.000932 8.29 < 2e-16 ***
M:GenderMale -0.001707 0.000593 -2.88 0.00397 **
M:edu.cat2tertiary -0.003734 0.000743 -5.03 5.0e-07 ***
M:edu.cat3mid -0.002220 0.000814 -2.73 0.00638 **
M:edu.cat4early -0.002458 0.000819 -3.00 0.00269 **
M:parac 0.001256 0.000591 2.13 0.03349 *
M:diclo -0.004743 0.001618 -2.93 0.00337 **
---
Signif. codes: 0 '***' 0.001 '**' 0.01 '*' 0.05 '.' 0.1 ' ' 1

Number of observations: total=8878, ID=1619
Random effect variance(s):
Group=ID
 Variance StdDev
(Intercept) 0.4117 0.6416

Negative binomial dispersion parameter: 1.0582 (std. err.: 0.018199)

Log-likelihood: -16937

anova(neg.m.combined, main.M.final)

Analysis of Deviance Table

Model 1: neg.b.MMSE ~ M + AGE + APOE4 + edu.cat + diagn + arthrit + Ibu + aspirin
Model 2: neg.b.MMSE ~ M + AGE + diagn + APOE4 + edu.cat + arthrit + aspirin + Ibu + diagn * M + smoke * M + APOE4 * M + Gender * M + edu.cat * M + parac * M + diclo * M
 NoPar LogLik Df Deviance Pr(>Chi)
1 16 -17087
2 32 -16937 16 299.8 < 2.2e-16 ***
---
Signif. codes: 0 '***' 0.001 '**' 0.01 '*' 0.05 '.' 0.1 ' ' 1

### Dropping non-significant terms

neg.m.combined.drop.smoke<- glmmadmb(neg.b.MMSE~ M+AGE+ diagn+ APOE4+edu.cat + arthrit+ aspirin+ Ibu+ diagn*M +APOE4*M + Gender*M+ edu.cat*M +parac*M +diclo*M+(1|ID), family="nbinom1", data=MMSEdata)
summary(neg.m.combined.drop.smoke)

Call:
glmmadmb(formula = neg.b.MMSE ~ M + AGE + diagn + APOE4 + edu.cat +
 arthrit + aspirin + Ibu + diagn * M + APOE4 * M + Gender *
 M + edu.cat * M + parac * M + diclo * M + (1 | ID), data = MMSEdata,
 family = "nbinom1")

AIC: 33934.4

Coefficients:
 Estimate Std. Error z value Pr(>|z|)
(Intercept) -0.553420 0.064793 -8.54 < 2e-16 ***
M 0.010381 0.000863 12.03 < 2e-16 ***
AGE 0.018181 0.002612 6.96 3.4e-12 ***
diagn2EMCI 0.707268 0.065640 10.77 < 2e-16 ***
diagn3LMCI 1.237085 0.055466 22.30 < 2e-16 ***
diagn4AD 2.091430 0.062254 33.60 < 2e-16 ***
APOE41 0.122679 0.042405 2.89 0.00382 **
APOE42 0.222583 0.064771 3.44 0.00059 ***
edu.cat2tertiary 0.204928 0.049523 4.14 3.5e-05 ***
edu.cat3mid 0.365119 0.055585 6.57 5.1e-11 ***
edu.cat4early 0.360400 0.057491 6.27 3.6e-10 ***
arthrit -0.060793 0.038355 -1.59 0.11296
aspirin -0.074093 0.037041 -2.00 0.04547 *
Ibu -0.082096 0.050448 -1.63 0.10366
GenderMale 0.091510 0.040530 2.26 0.02396 *
parac -0.075242 0.045658 -1.65 0.09936 .
diclo 0.045378 0.146620 0.31 0.75694
M:diagn2EMCI -0.003326 0.001043 -3.19 0.00143 **
M:diagn3LMCI 0.002854 0.000725 3.94 8.3e-05 ***
M:diagn4AD 0.008777 0.001437 6.11 1.0e-09 ***
M:APOE41 0.005858 0.000617 9.49 < 2e-16 ***
M:APOE42 0.007754 0.000931 8.33 < 2e-16 ***
M:GenderMale -0.001726 0.000591 -2.92 0.00351 **
M:edu.cat2tertiary -0.003746 0.000743 -5.04 4.5e-07 ***
M:edu.cat3mid -0.002245 0.000812 -2.77 0.00569 **
M:edu.cat4early -0.002476 0.000818 -3.03 0.00247 **
M:parac 0.001272 0.000589 2.16 0.03094 *
M:diclo -0.004723 0.001617 -2.92 0.00349 **
---
Signif. codes: 0 '***' 0.001 '**' 0.01 '*' 0.05 '.' 0.1 ' ' 1

Number of observations: total=8878, ID=1619
Random effect variance(s):
Group=ID
 Variance StdDev
(Intercept) 0.4116 0.6415

Negative binomial dispersion parameter: 1.0584 (std. err.: 0.018198)

Log-likelihood: -16937.2

anova(neg.m.combined, neg.m.combined.drop.smoke)

Analysis of Deviance Table

Model 1: neg.b.MMSE ~ M + AGE + diagn + APOE4 + edu.cat + arthrit + aspirin + Ibu + diagn * M + APOE4 * M + Gender * M + edu.cat * M + parac * M + diclo * M
Model 2: neg.b.MMSE ~ M + AGE + diagn + APOE4 + edu.cat + arthrit + aspirin + Ibu + diagn * M + smoke * M + APOE4 * M + Gender * M + edu.cat * M + parac * M + diclo * M
 NoPar LogLik Df Deviance Pr(>Chi)
1 30 -16937
2 32 -16937 2 0.4 0.8187

neg.m.combined.drop.Ibu<- glmmadmb(neg.b.MMSE~ M+AGE+ diagn+ APOE4+edu.cat + arthrit+ aspirin+ diagn*M +APOE4*M + Gender*M+ edu.cat*M +parac*M +diclo*M+(1|ID), family="nbinom1", data=MMSEdata)
summary(neg.m.combined.drop.Ibu)

Call:
glmmadmb(formula = neg.b.MMSE ~ M + AGE + diagn + APOE4 + edu.cat +
 arthrit + aspirin + diagn * M + APOE4 * M + Gender * M +
 edu.cat * M + parac * M + diclo * M + (1 | ID), data = MMSEdata,
 family = "nbinom1")

AIC: 33935

Coefficients:
 Estimate Std. Error z value Pr(>|z|)
(Intercept) -0.564023 0.064533 -8.74 < 2e-16 ***
M 0.010365 0.000863 12.01 < 2e-16 ***
AGE 0.018504 0.002607 7.10 1.3e-12 ***
diagn2EMCI 0.708667 0.065673 10.79 < 2e-16 ***
diagn3LMCI 1.238780 0.055492 22.32 < 2e-16 ***
diagn4AD 2.098663 0.062148 33.77 < 2e-16 ***
APOE41 0.123504 0.042433 2.91 0.00361 **
APOE42 0.224260 0.064815 3.46 0.00054 ***
edu.cat2tertiary 0.203265 0.049544 4.10 4.1e-05 ***
edu.cat3mid 0.364597 0.055621 6.56 5.6e-11 ***
edu.cat4early 0.357117 0.057495 6.21 5.3e-10 ***
arthrit -0.065485 0.038277 -1.71 0.08712 .
aspirin -0.074571 0.037070 -2.01 0.04426 *
GenderMale 0.090649 0.040554 2.24 0.02540 *
parac -0.081469 0.045525 -1.79 0.07353 .
diclo 0.045215 0.146720 0.31 0.75795
M:diagn2EMCI -0.003320 0.001043 -3.18 0.00146 **
M:diagn3LMCI 0.002863 0.000725 3.95 7.9e-05 ***
M:diagn4AD 0.008787 0.001437 6.11 9.8e-10 ***
M:APOE41 0.005871 0.000617 9.51 < 2e-16 ***
M:APOE42 0.007765 0.000931 8.34 < 2e-16 ***
M:GenderMale -0.001722 0.000591 -2.91 0.00360 **
M:edu.cat2tertiary -0.003755 0.000743 -5.06 4.3e-07 ***
M:edu.cat3mid -0.002258 0.000812 -2.78 0.00541 **
M:edu.cat4early -0.002485 0.000818 -3.04 0.00238 **
M:parac 0.001267 0.000589 2.15 0.03153 *
M:diclo -0.004698 0.001617 -2.91 0.00367 **
---
Signif. codes: 0 '***' 0.001 '**' 0.01 '*' 0.05 '.' 0.1 ' ' 1

Number of observations: total=8878, ID=1619
Random effect variance(s):
Group=ID
 Variance StdDev
(Intercept) 0.4123 0.6421

Negative binomial dispersion parameter: 1.0584 (std. err.: 0.018203)

Log-likelihood: -16938.5

anova(neg.m.combined.drop.smoke, neg.m.combined.drop.Ibu)

Analysis of Deviance Table

Model 1: neg.b.MMSE ~ M + AGE + diagn + APOE4 + edu.cat + arthrit + aspirin + diagn * M + APOE4 * M + Gender * M + edu.cat * M + parac * M + diclo * M
Model 2: neg.b.MMSE ~ M + AGE + diagn + APOE4 + edu.cat + arthrit + aspirin + Ibu + diagn * M + APOE4 * M + Gender * M + edu.cat * M + parac * M + diclo * M
 NoPar LogLik Df Deviance Pr(>Chi)
1 29 -16939
2 30 -16937 1 2.6 0.1069

neg.m.combined.drop.arthrit<- glmmadmb(neg.b.MMSE~ M+AGE+ diagn+ APOE4+edu.cat + aspirin+ diagn*M +APOE4*M + Gender*M+ edu.cat*M +parac*M +diclo*M+(1|ID), family="nbinom1", data=MMSEdata)
summary(neg.m.combined.drop.Ibu)

Call:
glmmadmb(formula = neg.b.MMSE ~ M + AGE + diagn + APOE4 + edu.cat +
 arthrit + aspirin + diagn * M + APOE4 * M + Gender * M +
 edu.cat * M + parac * M + diclo * M + (1 | ID), data = MMSEdata,
 family = "nbinom1")

AIC: 33935

Coefficients:
 Estimate Std. Error z value Pr(>|z|)
(Intercept) -0.564023 0.064533 -8.74 < 2e-16 ***
M 0.010365 0.000863 12.01 < 2e-16 ***
AGE 0.018504 0.002607 7.10 1.3e-12 ***
diagn2EMCI 0.708667 0.065673 10.79 < 2e-16 ***
diagn3LMCI 1.238780 0.055492 22.32 < 2e-16 ***
diagn4AD 2.098663 0.062148 33.77 < 2e-16 ***
APOE41 0.123504 0.042433 2.91 0.00361 **
APOE42 0.224260 0.064815 3.46 0.00054 ***
edu.cat2tertiary 0.203265 0.049544 4.10 4.1e-05 ***
edu.cat3mid 0.364597 0.055621 6.56 5.6e-11 ***
edu.cat4early 0.357117 0.057495 6.21 5.3e-10 ***
arthrit -0.065485 0.038277 -1.71 0.08712 .
aspirin -0.074571 0.037070 -2.01 0.04426 *
GenderMale 0.090649 0.040554 2.24 0.02540 *
parac -0.081469 0.045525 -1.79 0.07353 .
diclo 0.045215 0.146720 0.31 0.75795
M:diagn2EMCI -0.003320 0.001043 -3.18 0.00146 **
M:diagn3LMCI 0.002863 0.000725 3.95 7.9e-05 ***
M:diagn4AD 0.008787 0.001437 6.11 9.8e-10 ***
M:APOE41 0.005871 0.000617 9.51 < 2e-16 ***
M:APOE42 0.007765 0.000931 8.34 < 2e-16 ***
M:GenderMale -0.001722 0.000591 -2.91 0.00360 **
M:edu.cat2tertiary -0.003755 0.000743 -5.06 4.3e-07 ***
M:edu.cat3mid -0.002258 0.000812 -2.78 0.00541 **
M:edu.cat4early -0.002485 0.000818 -3.04 0.00238 **
M:parac 0.001267 0.000589 2.15 0.03153 *
M:diclo -0.004698 0.001617 -2.91 0.00367 **
---
Signif. codes: 0 '***' 0.001 '**' 0.01 '*' 0.05 '.' 0.1 ' ' 1

Number of observations: total=8878, ID=1619
Random effect variance(s):
Group=ID
 Variance StdDev
(Intercept) 0.4123 0.6421

Negative binomial dispersion parameter: 1.0584 (std. err.: 0.018203)

Log-likelihood: -16938.5

anova(neg.m.combined.drop.arthrit, neg.m.combined.drop.Ibu)

Analysis of Deviance Table

Model 1: neg.b.MMSE ~ M + AGE + diagn + APOE4 + edu.cat + aspirin + diagn * M + APOE4 * M + Gender * M + edu.cat * M + parac * M + diclo * M
Model 2: neg.b.MMSE ~ M + AGE + diagn + APOE4 + edu.cat + arthrit + aspirin + diagn * M + APOE4 * M + Gender * M + edu.cat * M + parac * M + diclo * M
 NoPar LogLik Df Deviance Pr(>Chi)
1 28 -16940
2 29 -16939 1 3 0.08326 .
---
Signif. codes: 0 '***' 0.001 '**' 0.01 '*' 0.05 '.' 0.1 ' ' 1

## Final full model and plots of the coeffecients

The full model of all significant interaction terms is now created, and non-significant terms will be removed.

neg.mmse<- glmmadmb(neg.b.MMSE~ M+AGE+ diagn+ APOE4+edu.cat + aspirin+ diagn*M +APOE4*M + Gender*M+ edu.cat*M +parac*M +diclo*M+(1|ID), family="nbinom1", data=MMSEdata)

summary(neg.mmse)

Call:
glmmadmb(formula = neg.b.MMSE ~ M + AGE + diagn + APOE4 + edu.cat +
 aspirin + diagn * M + APOE4 * M + Gender * M + edu.cat *
 M + parac * M + diclo * M + (1 | ID), data = MMSEdata, family = "nbinom1")

AIC: 33936

Coefficients:
 Estimate Std. Error z value Pr(>|z|)
(Intercept) -0.588327 0.063059 -9.33 < 2e-16 ***
M 0.010356 0.000863 12.00 < 2e-16 ***
AGE 0.018083 0.002598 6.96 3.4e-12 ***
diagn2EMCI 0.708348 0.065722 10.78 < 2e-16 ***
diagn3LMCI 1.242150 0.055504 22.38 < 2e-16 ***
diagn4AD 2.105171 0.062093 33.90 < 2e-16 ***
APOE41 0.120675 0.042440 2.84 0.00446 **
APOE42 0.226424 0.064864 3.49 0.00048 ***
edu.cat2tertiary 0.200328 0.049559 4.04 5.3e-05 ***
edu.cat3mid 0.361968 0.055648 6.50 7.8e-11 ***
edu.cat4early 0.352873 0.057489 6.14 8.4e-10 ***
aspirin -0.074933 0.037108 -2.02 0.04345 *
GenderMale 0.095723 0.040487 2.36 0.01806 *
parac -0.093084 0.045053 -2.07 0.03882 *
diclo 0.022617 0.146270 0.15 0.87712
M:diagn2EMCI -0.003321 0.001043 -3.18 0.00145 **
M:diagn3LMCI 0.002862 0.000725 3.95 7.9e-05 ***
M:diagn4AD 0.008798 0.001437 6.12 9.3e-10 ***
M:APOE41 0.005879 0.000617 9.52 < 2e-16 ***
M:APOE42 0.007765 0.000931 8.34 < 2e-16 ***
M:GenderMale -0.001724 0.000591 -2.92 0.00355 **
M:edu.cat2tertiary -0.003752 0.000743 -5.05 4.3e-07 ***
M:edu.cat3mid -0.002262 0.000812 -2.79 0.00532 **
M:edu.cat4early -0.002500 0.000818 -3.06 0.00224 **
M:parac 0.001285 0.000589 2.18 0.02928 *
M:diclo -0.004681 0.001617 -2.89 0.00380 **
---
Signif. codes: 0 '***' 0.001 '**' 0.01 '*' 0.05 '.' 0.1 ' ' 1

Number of observations: total=8878, ID=1619
Random effect variance(s):
Group=ID
 Variance StdDev
(Intercept) 0.4134 0.643

Negative binomial dispersion parameter: 1.0582 (std. err.: 0.018199)

Log-likelihood: -16940

anova(neg.m.combined, neg.mmse)

Analysis of Deviance Table

Model 1: neg.b.MMSE ~ M + AGE + diagn + APOE4 + edu.cat + aspirin + diagn * M + APOE4 * M + Gender * M + edu.cat * M + parac * M + diclo * M
Model 2: neg.b.MMSE ~ M + AGE + diagn + APOE4 + edu.cat + arthrit + aspirin + Ibu + diagn * M + smoke * M + APOE4 * M + Gender * M + edu.cat * M + parac * M + diclo * M
 NoPar LogLik Df Deviance Pr(>Chi)
1 28 -16940
2 32 -16937 4 6 0.1991

### Coeffecient plot

coefplot(neg.mmse)

Figure description: Plot of the coefficients with standard error (small lines) and 95% confidence intervals (larger lines), for the final selected model.


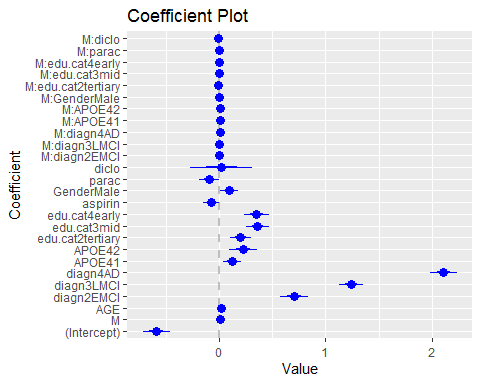


str(MMSEdata$APOE4)

Factor w/ 3 levels "0","1","2": 1 2 2 2 2 1 1 1 1 1 ...

neg.mmse.APOEnumeric<- glmmadmb(neg.b.MMSE~ M+AGE+ diagn+ as.numeric(APOE4)+edu.cat + aspirin+ diagn*M +as.numeric(APOE4)*M + Gender*M+ edu.cat*M +parac*M +diclo*M+(1|ID), family="nbinom1", data=MMSEdata)

summary(neg.mmse.APOEnumeric)

Call:
glmmadmb(formula = neg.b.MMSE ~ M + AGE + diagn + as.numeric(APOE4) +
 edu.cat + aspirin + diagn * M + as.numeric(APOE4) * M + Gender *
 M + edu.cat * M + parac * M + diclo * M + (1 | ID), data = MMSEdata,
 family = "nbinom1")

AIC: 33943.2

Coefficients:
 Estimate Std. Error z value Pr(>|z|)
(Intercept) -0.709461 0.072662 -9.76 < 2e-16 ***
M 0.006382 0.000999 6.39 1.7e-10 ***
AGE 0.018248 0.002593 7.04 2.0e-12 ***
diagn2EMCI 0.710113 0.065688 10.81 < 2e-16 ***
diagn3LMCI 1.243616 0.055465 22.42 < 2e-16 ***
diagn4AD 2.105614 0.062079 33.92 < 2e-16 ***
as.numeric(APOE4) 0.118605 0.029612 4.01 6.2e-05 ***
edu.cat2tertiary 0.199296 0.049567 4.02 5.8e-05 ***
edu.cat3mid 0.363931 0.055656 6.54 6.2e-11 ***
edu.cat4early 0.352677 0.057495 6.13 8.6e-10 ***
aspirin -0.077025 0.037098 -2.08 0.0379 *
GenderMale 0.097695 0.040486 2.41 0.0158 *
parac -0.089862 0.045026 -2.00 0.0460 *
diclo 0.037217 0.146120 0.25 0.7990
M:diagn2EMCI -0.003361 0.001044 -3.22 0.0013 **
M:diagn3LMCI 0.002849 0.000725 3.93 8.5e-05 ***
M:diagn4AD 0.008748 0.001439 6.08 1.2e-09 ***
M:as.numeric(APOE4) 0.004496 0.000426 10.56 < 2e-16 ***
M:GenderMale -0.001836 0.000591 -3.11 0.0019 **
M:edu.cat2tertiary -0.003677 0.000743 -4.95 7.4e-07 ***
M:edu.cat3mid -0.002349 0.000812 -2.89 0.0038 **
M:edu.cat4early -0.002420 0.000818 -2.96 0.0031 **
M:parac 0.001115 0.000587 1.90 0.0576 .
M:diclo -0.005241 0.001614 -3.25 0.0012 **
---
Signif. codes: 0 '***' 0.001 '**' 0.01 '*' 0.05 '.' 0.1 ' ' 1

Number of observations: total=8878, ID=1619
Random effect variance(s):
Group=ID
 Variance StdDev
(Intercept) 0.4134 0.643

Negative binomial dispersion parameter: 1.0599 (std. err.: 0.018261)

Log-likelihood: -16945.6

anova(neg.mmse.APOEnumeric, neg.mmse)

Analysis of Deviance Table

Model 1: neg.b.MMSE ~ M + AGE + diagn + as.numeric(APOE4) + edu.cat + aspirin + diagn * M + as.numeric(APOE4) * M + Gender * M + edu.cat * M + parac * M + diclo * M
Model 2: neg.b.MMSE ~ M + AGE + diagn + APOE4 + edu.cat + aspirin + diagn * M + APOE4 * M + Gender * M + edu.cat * M + parac * M + diclo * M
 NoPar LogLik Df Deviance Pr(>Chi)
1 26 -16946
2 28 -16940 2 11.2 0.003698 **
---
Signif. codes: 0 '***' 0.001 '**' 0.01 '*' 0.05 '.' 0.1 ' ' 1

### Coeffecient plot of interaction terms

coefficients<-c("M:diclo","M:parac","M:APOE41","M:APOE42","M:edu.cat2tertiary","M:edu.cat3mid","M:edu.cat4early","M:diagn2EMCI","M:diagn3LMCI","M:diagn4AD","M:GenderMale","M")
coefplot(neg.mmse,coefficients=coefficients,main="Interaction terms coeffecients")

Figure description: Plot of the coefficients of the interaction terms with standard error (small lines) and 95% confidence intervals (larger lines), for the final selected model.


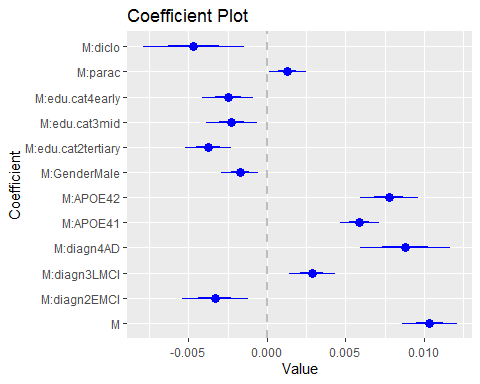


## Dropping terms of the model to evaluate the significance of each variable in the ful model.

Age.MMSE<- glmmadmb(neg.b.MMSE~ M+ diagn+ APOE4+edu.cat + aspirin+ diagn*M +APOE4*M + Gender*M+ edu.cat*M +parac*M +diclo*M+(1|ID), family="nbinom1", data=MMSEdata)

No.APOE4.MMSE<- glmmadmb(neg.b.MMSE~ M+AGE+ diagn+ edu.cat + aspirin+ diagn*M + Gender*M+ edu.cat*M +parac*M +diclo*M+(1|ID), family="nbinom1", data=MMSEdata)

APOE4.MMSE.Main<- glmmadmb(neg.b.MMSE~ M+AGE+ diagn+ APOE4+edu.cat + aspirin+ diagn*M + Gender*M+ edu.cat*M +parac*M +diclo*M+(1|ID), family="nbinom1", data=MMSEdata)

No.Education.MMSE<- glmmadmb(neg.b.MMSE~ M+AGE+ diagn+ APOE4+aspirin+ diagn*M +APOE4*M + Gender*M+ parac*M +diclo*M+(1|ID), family="nbinom1", data=MMSEdata)

Education.MMSE.Main<- glmmadmb(neg.b.MMSE~ M+AGE+ diagn+ APOE4+edu.cat + aspirin+ APOE4*M+diagn*M + Gender*M +parac*M +diclo*M+(1|ID), family="nbinom1", data=MMSEdata)

No.Diagn.MMSE<- glmmadmb(neg.b.MMSE~ M+AGE+ APOE4+edu.cat + aspirin+ APOE4*M + Gender*M+ edu.cat*M +parac*M +diclo*M+(1|ID), family="nbinom1", data=MMSEdata)

Diagn.MMSE.Main<- glmmadmb(neg.b.MMSE~ M+AGE+ diagn+ APOE4+edu.cat + aspirin+ APOE4*M + Gender*M+ edu.cat*M +parac*M +diclo*M+(1|ID), family="nbinom1", data=MMSEdata)

No.Gender.MMSE<- glmmadmb(neg.b.MMSE~ M+AGE+ diagn+ APOE4+edu.cat + aspirin+ diagn*M +APOE4*M + edu.cat*M +parac*M +diclo*M+(1|ID), family="nbinom1", data=MMSEdata)

Gender.MMSE.Main<- glmmadmb(neg.b.MMSE~ M+AGE+ diagn+ APOE4+edu.cat + aspirin+ diagn*M +APOE4*M + Gender+ edu.cat*M +parac*M +diclo*M+(1|ID), family="nbinom1", data=MMSEdata)

No.Aspirin.MMSE<- glmmadmb(neg.b.MMSE~ M+AGE+ diagn+ APOE4+edu.cat + diagn*M +APOE4*M + Gender*M+ edu.cat*M +parac*M +diclo*M+(1|ID), family="nbinom1", data=MMSEdata)


No.Paracet.MMSE<- glmmadmb(neg.b.MMSE~ M+AGE+ diagn+ APOE4+edu.cat + aspirin+ diagn*M +APOE4*M + Gender*M+ edu.cat*M +diclo*M+(1|ID), family="nbinom1", data=MMSEdata)


Paracet.MMSE.Main<- glmmadmb(neg.b.MMSE~ M+AGE+ diagn+ APOE4+edu.cat + aspirin+ diagn*M +APOE4*M + Gender*M+ edu.cat*M +diclo*M+parac+(1|ID), family="nbinom1", data=MMSEdata)

No.Diclofen.MMSE<- glmmadmb(neg.b.MMSE~ M+AGE+ diagn+ APOE4+edu.cat + aspirin+ diagn*M +APOE4*M + Gender*M+ edu.cat*M +parac*M + (1|ID), family="nbinom1", data=MMSEdata)

Diclofen.MMSE.Main<- glmmadmb(neg.b.MMSE~ M+AGE+ diagn+ APOE4+edu.cat + aspirin+ diagn*M +APOE4*M + Gender*M+ edu.cat*M +parac*M +diclo+(1|ID), family="nbinom1", data=MMSEdata)

Model<-c("Main effect of Age", "Main effect of APOE4", "Main effect of Education", "Main effect of Diagnosis","Main effect of Gender", "Main effect of Aspirin","Main effect of Paracetamol","Main effect of Diclofenac","Interaction effect of Education","Interaction effect of Diagnosis","Interaction effect of APOE4","Interaction effect of Gender","Interaction effect of Paracetamol","Interaction effect of Diclofenac")

M.age<-anova(neg.mmse, Age.MMSE)
M.APOE4<-anova(No.APOE4.MMSE, APOE4.MMSE.Main)
M.Education<-anova(Education.MMSE.Main,No.Education.MMSE)
M.Diagn<-anova(No.Diagn.MMSE, Diagn.MMSE.Main)
M.Gender<-anova(No.Gender.MMSE, Gender.MMSE.Main)
M.Aspirin<-anova(No.Aspirin.MMSE, neg.mmse)
M.Paracetamol<-anova(No.Paracet.MMSE, Paracet.MMSE.Main)
M.Diclofenac<-anova(No.Diclofen.MMSE, Diclofen.MMSE.Main)

I.Education<-anova(neg.mmse, Education.MMSE.Main)
I.Diagnosis<-anova(neg.mmse, Diagn.MMSE.Main)
I.APOE4<-anova(neg.mmse, APOE4.MMSE.Main)
I.Gender<-anova(neg.mmse, Gender.MMSE.Main)
I.Paracetamol<-anova(neg.mmse, Paracet.MMSE.Main)
I.Diclofenac<-anova(neg.mmse, Diclofen.MMSE.Main)

MMSE.PValue<-rbind(M.age[2,3:5],M.APOE4[2,3:5],M.Education[2,3:5],M.Diagn[2,3:5],M.Gender[2,3:5],M.Aspirin[2,3:5],M.Paracetamol[2,3:5],M.Diclofenac[2,3:5],I.Education[2,3:5],I.Diagnosis[2,3:5],I.APOE4[2,3:5],I.Gender[2,3:5],I.Paracetamol[2,3:5],I.Diclofenac[2,3:5])

data.frame(Model,MMSE.PValue)

Model Df Deviance Pr..Chi.
2 Main effect of Age 1 48.2 3.848921e-12
21 Main effect of APOE4 2 60.6 6.927792e-14
22 Main effect of Education 3 47.4 2.857187e-10
23 Main effect of Diagnosis 3 1147.2 0.000000e+00
24 Main effect of Gender 1 2.2 1.380107e-01
25 Main effect of Aspirin 1 4.0 4.550026e-02
26 Main effect of Paracetamol 1 1.8 1.797125e-01
27 Main effect of Diclofenac 1 1.0 3.173105e-01
28 Interaction effect of Education 3 26.6 7.140958e-06
29 Interaction effect of Diagnosis 3 79.8 0.000000e+00
210 Interaction effect of APOE4 2 120.6 0.000000e+00
211 Interaction effect of Gender 1 8.4 3.752210e-03
212 Interaction effect of Paracetamol 1 4.8 2.845974e-02
213 Interaction effect of Diclofenac 1 8.4 3.752210e-03

## Evaluating the progession and main-effects of each pain medication

neg.M.base<- glmmadmb(neg.b.MMSE~ AGE + APOE4 + M + edu.cat + diagn+ diagn*M +APOE4*M + Gender*M+ edu.cat*M + (1|ID), family="nbinom1", data=MMSEdata)

neg.M.M.aspirin<- glmmadmb(neg.b.MMSE~ AGE + APOE4 + M + edu.cat + diagn+ diagn*M +APOE4*M + Gender*M+ edu.cat*M + aspirin+(1|ID), family="nbinom1", data=MMSEdata)

neg.M.M.celecoxib<- glmmadmb(neg.b.MMSE~ AGE + APOE4 + M + edu.cat + diagn+ diagn*M +APOE4*M + Gender*M+ edu.cat*M + celex+(1|ID), family="nbinom1", data=MMSEdata)

neg.M.M.diclofenac<- glmmadmb(neg.b.MMSE~ AGE + APOE4 + M + edu.cat + diagn+ diagn*M +APOE4*M + Gender*M+ edu.cat*M +diclo+ (1|ID), family="nbinom1", data=MMSEdata)

neg.M.M.ibuprofen<- glmmadmb(neg.b.MMSE~ AGE + APOE4 + M + edu.cat + diagn+ diagn*M +APOE4*M + Gender*M+ edu.cat*M +Ibu+ (1|ID), family="nbinom1", data=MMSEdata)

neg.M.M.naproxen<- glmmadmb(neg.b.MMSE~ AGE + APOE4 + M + edu.cat + diagn+ diagn*M +APOE4*M + Gender*M+ edu.cat*M +naprox+ (1|ID), family="nbinom1", data=MMSEdata)

neg.M.M.paracetamol<- glmmadmb(neg.b.MMSE~ AGE + APOE4 + M + edu.cat + diagn+ diagn*M +APOE4*M + Gender*M+ edu.cat*M + parac+ (1|ID), family="nbinom1", data=MMSEdata)

neg.M.I.aspirin<- glmmadmb(neg.b.MMSE~ AGE + APOE4 + M + edu.cat + diagn+ diagn*M +APOE4*M + Gender*M+ edu.cat*M +aspirin*M+ (1|ID), family="nbinom1", data=MMSEdata)

neg.M.I.celecoxib<- glmmadmb(neg.b.MMSE~ AGE + APOE4 + M + edu.cat + diagn+ diagn*M +APOE4*M + Gender*M+ edu.cat*M +celex*M+ (1|ID), family="nbinom1", data=MMSEdata)

neg.M.I.diclofenac<- glmmadmb(neg.b.MMSE~ AGE + APOE4 + M + edu.cat + diagn+ diagn*M +APOE4*M + Gender*M+ edu.cat*M +diclo*M+ (1|ID), family="nbinom1", data=MMSEdata)

neg.M.I.ibuprofen<- glmmadmb(neg.b.MMSE~ AGE + APOE4 + M + edu.cat + diagn+ diagn*M +APOE4*M + Gender*M+ edu.cat*M +Ibu*M+ (1|ID), family="nbinom1", data=MMSEdata)

neg.M.I.naproxen<- glmmadmb(neg.b.MMSE~ AGE + APOE4 + M + edu.cat + diagn+ diagn*M +APOE4*M + Gender*M+ edu.cat*M + naprox*M+ (1|ID), family="nbinom1", data=MMSEdata)

neg.M.I.paracetamol<- glmmadmb(neg.b.MMSE~ AGE + APOE4 + M + edu.cat + diagn+ diagn*M +APOE4*M + Gender*M+ edu.cat*M +parac*M+ (1|ID), family="nbinom1", data=MMSEdata)

anova.M.aspirin<-anova(neg.M.M.aspirin, neg.M.base)
anova.M.celecoxib<-anova(neg.M.M.celecoxib, neg.M.base)
anova.M.diclofenac<-anova(neg.M.M.diclofenac, neg.M.base)
anova.M.ibuprofen<-anova(neg.M.M.ibuprofen, neg.M.base)
anova.M.naproxen<-anova(neg.M.M.naproxen, neg.M.base)
anova.M.paracetamol<-anova(neg.M.M.paracetamol, neg.M.base)

anova.I.aspirin<-anova(neg.M.M.aspirin, neg.M.I.aspirin)
anova.I.celecoxib<-anova(neg.M.M.celecoxib, neg.M.I.celecoxib)
anova.I.diclofenac<-anova(neg.M.M.diclofenac, neg.M.I.diclofenac)
anova.I.ibuprofen<-anova(neg.M.M.ibuprofen, neg.M.I.ibuprofen)
anova.I.naproxen<-anova(neg.M.M.naproxen, neg.M.I.naproxen)
anova.I.paracetamol<-anova(neg.M.M.paracetamol, neg.M.I.paracetamol)
Full.Model.Painrelief.MMSE<- c("Main effect of Aspirin", "Main effect of Celecoxib", "Main effect of Diclofenac", "Main effect of Ibuprofen", "Main effect of Naproxen","Main effect of Paracetamol","Interaction effect of Aspirin","Interaction effect of Celecoxib","Interaction effect of Diclofenac","Interaction effect of Ibuprofen","Interaction effect of Naproxen","Interaction effect of Paracetamol")

Full.MMSE.Painrelief.PValue<-rbind(anova.M.aspirin[2,3:5], anova.M.celecoxib[2,3:5], anova.M.diclofenac[2,3:5], anova.M.ibuprofen[2,3:5], anova.M.naproxen[2,3:5], anova.M.paracetamol[2,3:5], anova.I.aspirin[2,3:5], anova.I.celecoxib[2,3:5], anova.I.diclofenac[2,3:5], anova.I.ibuprofen[2,3:5], anova.I.naproxen[2,3:5], anova.I.paracetamol[2,3:5])

Full.MMSE.Painrelief.PValue$Adjuste.PValue<-c(rep("NA",6), p.adjust(Full.MMSE.Painrelief.PValue$`Pr(>Chi)`[7:12], method="holm"))

full.timeframe.data<-data.frame(Full.Model.Painrelief.MMSE, Full.MMSE.Painrelief.PValue)
(full.timeframe.data)

Full.Model.Painrelief.MMSE Df Deviance Pr..Chi.
2 Main effect of Aspirin 1 4.8 0.028459737
21 Main effect of Celecoxib 1 2.2 0.138010738
22 Main effect of Diclofenac 1 1.2 0.273321678
23 Main effect of Ibuprofen 1 3.6 0.057779571
24 Main effect of Naproxen 1 0.2 0.654720846
25 Main effect of Paracetamol 1 2.6 0.106863715
26 Interaction effect of Aspirin 1 4.0 0.045500264
27 Interaction effect of Celecoxib 1 2.4 0.121335250
28 Interaction effect of Diclofenac 1 7.4 0.006522388
29 Interaction effect of Ibuprofen 1 0.0 1.000000000
210 Interaction effect of Naproxen 1 0.0 1.000000000
211 Interaction effect of Paracetamol 1 3.6 0.057779571
 Adjuste.PValue
2 NA
21 NA
22 NA
23 NA
24 NA
25 NA
26 0.227501319481792
27 0.364005751075107
28 0.0391343262387691
29 1
210 1
211 0.23111828449459

## Evaluating the progession and main-effects of each pain medication over short peroid with few deaths or missing data points

MMSEdata.short<-MMSEdata[MMSEdata$M==c(0,6,12,18,24,30,36,48),]

neg.M.base<- glmmadmb(neg.b.MMSE~ AGE + APOE4 + M + edu.cat + diagn+ diagn*M +APOE4*M + Gender*M+ edu.cat*M + (1|ID), family="nbinom1", data=MMSEdata.short)

neg.M.M.aspirin<- glmmadmb(neg.b.MMSE~ AGE + APOE4 + M + edu.cat + diagn+ diagn*M +APOE4*M + Gender*M+ edu.cat*M + aspirin+(1|ID), family="nbinom1", data=MMSEdata.short)

neg.M.M.celecoxib<- glmmadmb(neg.b.MMSE~ AGE + APOE4 + M + edu.cat + diagn+ diagn*M +APOE4*M + Gender*M+ edu.cat*M + celex+(1|ID), family="nbinom1", data=MMSEdata.short)

neg.M.M.diclofenac<- glmmadmb(neg.b.MMSE~ AGE + APOE4 + M + edu.cat + diagn+ diagn*M +APOE4*M + Gender*M+ edu.cat*M +diclo+ (1|ID), family="nbinom1", data=MMSEdata.short)

neg.M.M.ibuprofen<- glmmadmb(neg.b.MMSE~ AGE + APOE4 + M + edu.cat + diagn+ diagn*M +APOE4*M + Gender*M+ edu.cat*M +Ibu+ (1|ID), family="nbinom1", data=MMSEdata.short)

neg.M.M.naproxen<- glmmadmb(neg.b.MMSE~ AGE + APOE4 + M + edu.cat + diagn+ diagn*M +APOE4*M + Gender*M+ edu.cat*M +naprox+ (1|ID), family="nbinom1", data=MMSEdata.short)

neg.M.M.paracetamol<- glmmadmb(neg.b.MMSE~ AGE + APOE4 + M + edu.cat + diagn+ diagn*M +APOE4*M + Gender*M+ edu.cat*M + parac+ (1|ID), family="nbinom1", data=MMSEdata.short)

neg.M.I.aspirin<- glmmadmb(neg.b.MMSE~ AGE + APOE4 + M + edu.cat + diagn+ diagn*M +APOE4*M + Gender*M+ edu.cat*M +aspirin*M+ (1|ID), family="nbinom1", data=MMSEdata.short)

neg.M.I.celecoxib<- glmmadmb(neg.b.MMSE~ AGE + APOE4 + M + edu.cat + diagn+ diagn*M +APOE4*M + Gender*M+ edu.cat*M +celex*M+ (1|ID), family="nbinom1", data=MMSEdata.short)

neg.M.I.diclofenac<- glmmadmb(neg.b.MMSE~ AGE + APOE4 + M + edu.cat + diagn+ diagn*M +APOE4*M + Gender*M+ edu.cat*M +diclo*M+ (1|ID), family="nbinom1", data=MMSEdata.short)

neg.M.I.ibuprofen<- glmmadmb(neg.b.MMSE~ AGE + APOE4 + M + edu.cat + diagn+ diagn*M +APOE4*M + Gender*M+ edu.cat*M +Ibu*M+ (1|ID), family="nbinom1", data=MMSEdata.short)

neg.M.I.naproxen<- glmmadmb(neg.b.MMSE~ AGE + APOE4 + M + edu.cat + diagn+ diagn*M +APOE4*M + Gender*M+ edu.cat*M + naprox*M+ (1|ID), family="nbinom1", data=MMSEdata.short)

neg.M.I.paracetamol<- glmmadmb(neg.b.MMSE~ AGE + APOE4 + M + edu.cat + diagn+ diagn*M +APOE4*M + Gender*M+ edu.cat*M +parac*M+ (1|ID), family="nbinom1", data=MMSEdata.short)

anova.M.aspirin<-anova(neg.M.M.aspirin, neg.M.base)
anova.M.celecoxib<-anova(neg.M.M.celecoxib, neg.M.base)
anova.M.diclofenac<-anova(neg.M.M.diclofenac, neg.M.base)
anova.M.ibuprofen<-anova(neg.M.M.ibuprofen, neg.M.base)
anova.M.naproxen<-anova(neg.M.M.naproxen, neg.M.base)
anova.M.paracetamol<-anova(neg.M.M.paracetamol, neg.M.base)

anova.I.aspirin<-anova(neg.M.M.aspirin, neg.M.I.aspirin)
anova.I.celecoxib<-anova(neg.M.M.celecoxib, neg.M.I.celecoxib)
anova.I.diclofenac<-anova(neg.M.M.diclofenac, neg.M.I.diclofenac)
anova.I.ibuprofen<-anova(neg.M.M.ibuprofen, neg.M.I.ibuprofen)
anova.I.naproxen<-anova(neg.M.M.naproxen, neg.M.I.naproxen)
anova.I.paracetamol<-anova(neg.M.M.paracetamol, neg.M.I.paracetamol)
Model.Painrelief.MMSE<- c("Main effect of Aspirin", "Main effect of Celecoxib", "Main effect of Diclofenac", "Main effect of Ibuprofen", "Main effect of Naproxen","Main effect of Paracetamol","Interaction effect of Aspirin","Interaction effect of Celecoxib","Interaction effect of Diclofenac","Interaction effect of Ibuprofen","Interaction effect of Naproxen","Interaction effect of Paracetamol")

Short.MMSE.Painrelief.PValue<-rbind(anova.M.aspirin[2,3:5], anova.M.celecoxib[2,3:5], anova.M.diclofenac[2,3:5], anova.M.ibuprofen[2,3:5], anova.M.naproxen[2,3:5], anova.M.paracetamol[2,3:5], anova.I.aspirin[2,3:5], anova.I.celecoxib[2,3:5], anova.I.diclofenac[2,3:5], anova.I.ibuprofen[2,3:5], anova.I.naproxen[2,3:5], anova.I.paracetamol[2,3:5])

Short.MMSE.Painrelief.PValue$Adjusted<-c(rep("NA",6), p.adjust(Short.MMSE.Painrelief.PValue$`Pr(>Chi)`[7:12], method="holm"))

short.timeframe.data<-data.frame(Model.Painrelief.MMSE, Short.MMSE.Painrelief.PValue)
(short.timeframe.data)

Model.Painrelief.MMSE Df Deviance Pr..Chi. Adjusted
2 Main effect of Aspirin 1 0.92 0.337474972 NA
21 Main effect of Celecoxib 1 2.56 0.109598583 NA
22 Main effect of Diclofenac 1 0.54 0.462432726 NA
23 Main effect of Ibuprofen 1 10.46 0.001219873 NA
24 Main effect of Naproxen 1 0.02 0.887537084 NA
25 Main effect of Paracetamol 1 0.22 0.639039918 NA
26 Interaction effect of Aspirin 1 0.00 1.000000000 1
27 Interaction effect of Celecoxib 1 1.98 0.159390407 0.861011348333689
28 Interaction effect of Diclofenac 1 0.76 0.383328523 0.882798312914948
29 Interaction effect of Ibuprofen 1 2.14 0.143501891 0.861011348333689
210 Interaction effect of Naproxen 1 1.54 0.214617805 0.861011348333689
211 Interaction effect of Paracetamol 1 1.10 0.294266104 0.882798312914948

summary(neg.M.I.aspirin)

Call:
glmmadmb(formula = neg.b.MMSE ~ AGE + APOE4 + M + edu.cat + diagn +
 diagn * M + APOE4 * M + Gender * M + edu.cat * M + aspirin *
 M + (1 | ID), data = MMSEdata.short, family = "nbinom1")

AIC: 3914.2

Coefficients:
 Estimate Std. Error z value Pr(>|z|)
(Intercept) -4.32e-01 1.55e-01 -2.79 0.00530 **
AGE 1.42e-02 4.82e-03 2.96 0.00312 **
APOE41 6.52e-02 9.84e-02 0.66 0.50713
APOE42 1.52e-01 1.45e-01 1.05 0.29396
M 7.40e-03 6.15e-03 1.20 0.22901
edu.cat2tertiary 3.02e-01 1.09e-01 2.77 0.00563 **
edu.cat3mid 3.04e-01 1.29e-01 2.36 0.01847 *
edu.cat4early 3.04e-01 1.39e-01 2.18 0.02897 *
diagn2EMCI 6.42e-01 1.59e-01 4.04 5.3e-05 ***
diagn3LMCI 1.06e+00 1.44e-01 7.37 1.7e-13 ***
diagn4AD 1.88e+00 1.53e-01 12.32 < 2e-16 ***
GenderMale 9.39e-02 9.59e-02 0.98 0.32763
aspirin -6.28e-02 8.94e-02 -0.70 0.48213
M:diagn2EMCI -7.78e-03 6.16e-03 -1.26 0.20667
M:diagn3LMCI 4.46e-03 5.51e-03 0.81 0.41849
M:diagn4AD 1.70e-02 8.32e-03 2.04 0.04129 *
APOE41:M 1.44e-02 3.94e-03 3.66 0.00025 ***
APOE42:M 2.67e-02 5.38e-03 4.97 6.8e-07 ***
M:GenderMale -3.26e-03 3.73e-03 -0.87 0.38231
M:edu.cat2tertiary -1.47e-02 4.40e-03 -3.35 0.00081 ***
M:edu.cat3mid -6.58e-03 5.12e-03 -1.29 0.19860
M:edu.cat4early -1.82e-03 5.40e-03 -0.34 0.73558
M:aspirin 1.56e-05 3.54e-03 0.00 0.99648
---
Signif. codes: 0 '***' 0.001 '**' 0.01 '*' 0.05 '.' 0.1 ' ' 1

Number of observations: total=977, ID=616
Random effect variance(s):
Group=ID
 Variance StdDev
(Intercept) 0.3133 0.5597

Negative binomial dispersion parameter: 1.001 (std. err.: 3.6706e-06)

Log-likelihood: -1932.12

summary(neg.M.I.celecoxib)

Call:
glmmadmb(formula = neg.b.MMSE ~ AGE + APOE4 + M + edu.cat + diagn +
 diagn * M + APOE4 * M + Gender * M + edu.cat * M + celex *
 M + (1 | ID), data = MMSEdata.short, family = "nbinom1")

AIC: 3910.6

Coefficients:
 Estimate Std. Error z value Pr(>|z|)
(Intercept) -0.46461 0.14918 -3.11 0.00184 **
AGE 0.01348 0.00480 2.81 0.00501 **
APOE41 0.06505 0.09820 0.66 0.50771
APOE42 0.14015 0.14470 0.97 0.33276
M 0.00805 0.00599 1.34 0.17884
edu.cat2tertiary 0.30239 0.10869 2.78 0.00540 **
edu.cat3mid 0.30469 0.12885 2.36 0.01805 *
edu.cat4early 0.31813 0.14040 2.27 0.02346 *
diagn2EMCI 0.63083 0.15859 3.98 7.0e-05 ***
diagn3LMCI 1.06707 0.14373 7.42 1.1e-13 ***
diagn4AD 1.88725 0.15263 12.36 < 2e-16 ***
GenderMale 0.08949 0.09519 0.94 0.34716
celex -0.01020 0.28045 -0.04 0.97099
M:diagn2EMCI -0.00659 0.00618 -1.07 0.28679
M:diagn3LMCI 0.00415 0.00551 0.75 0.45159
M:diagn4AD 0.01701 0.00830 2.05 0.04030 *
APOE41:M 0.01482 0.00395 3.76 0.00017 ***
APOE42:M 0.02716 0.00537 5.06 4.2e-07 ***
M:GenderMale -0.00351 0.00371 -0.95 0.34394
M:edu.cat2tertiary -0.01530 0.00438 -3.49 0.00048 ***
M:edu.cat3mid -0.00675 0.00512 -1.32 0.18755
M:edu.cat4early -0.00176 0.00541 -0.33 0.74467
M:celex -0.01400 0.00998 -1.40 0.16094
---
Signif. codes: 0 '***' 0.001 '**' 0.01 '*' 0.05 '.' 0.1 ' ' 1

Number of observations: total=977, ID=616
Random effect variance(s):
Group=ID
 Variance StdDev
(Intercept) 0.3103 0.5571

Negative binomial dispersion parameter: 1.001 (std. err.: 5.0008e-06)

Log-likelihood: -1930.31

summary(neg.M.I.diclofenac)

Call:
glmmadmb(formula = neg.b.MMSE ~ AGE + APOE4 + M + edu.cat + diagn +
 diagn * M + APOE4 * M + Gender * M + edu.cat * M + diclo *
 M + (1 | ID), data = MMSEdata.short, family = "nbinom1")

AIC: 3913.9

Coefficients:
 Estimate Std. Error z value Pr(>|z|)
(Intercept) -0.46241 0.14943 -3.09 0.00197 **
AGE 0.01380 0.00481 2.87 0.00415 **
APOE41 0.06443 0.09830 0.66 0.51216
APOE42 0.15280 0.14534 1.05 0.29310
M 0.00738 0.00598 1.23 0.21736
edu.cat2tertiary 0.29718 0.10881 2.73 0.00631 **
edu.cat3mid 0.30441 0.12909 2.36 0.01837 *
edu.cat4early 0.31040 0.13857 2.24 0.02509 *
diagn2EMCI 0.63878 0.15865 4.03 5.7e-05 ***
diagn3LMCI 1.06124 0.14383 7.38 1.6e-13 ***
diagn4AD 1.88502 0.15265 12.35 < 2e-16 ***
GenderMale 0.08953 0.09536 0.94 0.34783
diclo -0.07811 0.38729 -0.20 0.84016
M:diagn2EMCI -0.00815 0.00617 -1.32 0.18644
M:diagn3LMCI 0.00452 0.00550 0.82 0.41181
M:diagn4AD 0.01724 0.00830 2.08 0.03780 *
APOE41:M 0.01431 0.00394 3.63 0.00028 ***
APOE42:M 0.02634 0.00539 4.89 1.0e-06 ***
M:GenderMale -0.00342 0.00371 -0.92 0.35651
M:edu.cat2tertiary -0.01442 0.00438 -3.29 0.00101 **
M:edu.cat3mid -0.00635 0.00512 -1.24 0.21466
M:edu.cat4early -0.00178 0.00540 -0.33 0.74123
M:diclo 0.01421 0.01616 0.88 0.37925
---
Signif. codes: 0 '***' 0.001 '**' 0.01 '*' 0.05 '.' 0.1 ' ' 1

Number of observations: total=977, ID=616
Random effect variance(s):
Group=ID
 Variance StdDev
(Intercept) 0.3124 0.5589

Negative binomial dispersion parameter: 1.001 (std. err.: 3.954e-06)

Log-likelihood: -1931.93

summary(neg.M.I.ibuprofen)

Call:
glmmadmb(formula = neg.b.MMSE ~ AGE + APOE4 + M + edu.cat + diagn +
 diagn * M + APOE4 * M + Gender * M + edu.cat * M + Ibu *
 M + (1 | ID), data = MMSEdata.short, family = "nbinom1")

AIC: 3902.6

Coefficients:
 Estimate Std. Error z value Pr(>|z|)
(Intercept) -0.43208 0.14992 -2.88 0.00395 **
AGE 0.01272 0.00478 2.66 0.00774 **
APOE41 0.05390 0.09772 0.55 0.58124
APOE42 0.14631 0.14376 1.02 0.30881
M 0.00886 0.00601 1.47 0.14049
edu.cat2tertiary 0.30155 0.10814 2.79 0.00530 **
edu.cat3mid 0.31050 0.12816 2.42 0.01540 *
edu.cat4early 0.31471 0.13776 2.28 0.02234 *
diagn2EMCI 0.64940 0.15792 4.11 3.9e-05 ***
diagn3LMCI 1.06221 0.14316 7.42 1.2e-13 ***
diagn4AD 1.87613 0.15200 12.34 < 2e-16 ***
GenderMale 0.09320 0.09480 0.98 0.32553
Ibu -0.17326 0.12755 -1.36 0.17435
M:diagn2EMCI -0.00806 0.00613 -1.31 0.18859
M:diagn3LMCI 0.00387 0.00548 0.71 0.47998
M:diagn4AD 0.01669 0.00826 2.02 0.04336 *
APOE41:M 0.01411 0.00393 3.59 0.00032 ***
APOE42:M 0.02624 0.00533 4.92 8.7e-07 ***
M:GenderMale -0.00325 0.00370 -0.88 0.38025
M:edu.cat2tertiary -0.01471 0.00436 -3.38 0.00074 ***
M:edu.cat3mid -0.00644 0.00509 -1.26 0.20613
M:edu.cat4early -0.00158 0.00538 -0.29 0.76873
M:Ibu -0.00768 0.00527 -1.46 0.14467
---
Signif. codes: 0 '***' 0.001 '**' 0.01 '*' 0.05 '.' 0.1 ' ' 1

Number of observations: total=977, ID=616
Random effect variance(s):
Group=ID
 Variance StdDev
(Intercept) 0.3038 0.5512

Negative binomial dispersion parameter: 1.001 (std. err.: 4.9103e-07)

Log-likelihood: -1926.28

summary(neg.M.I.naproxen)

Call:
glmmadmb(formula = neg.b.MMSE ~ AGE + APOE4 + M + edu.cat + diagn +
 diagn * M + APOE4 * M + Gender * M + edu.cat * M + naprox *
 M + (1 | ID), data = MMSEdata.short, family = "nbinom1")

AIC: 3913.6

Coefficients:
 Estimate Std. Error z value Pr(>|z|)
(Intercept) -0.44677 0.15010 -2.98 0.00292 **
AGE 0.01422 0.00482 2.95 0.00321 **
APOE41 0.06925 0.09836 0.70 0.48141
APOE42 0.15862 0.14521 1.09 0.27468
M 0.00662 0.00602 1.10 0.27182
edu.cat2tertiary 0.30161 0.10899 2.77 0.00565 **
edu.cat3mid 0.30304 0.12908 2.35 0.01889 *
edu.cat4early 0.30461 0.13881 2.19 0.02820 *
diagn2EMCI 0.63950 0.15866 4.03 5.6e-05 ***
diagn3LMCI 1.05871 0.14390 7.36 1.9e-13 ***
diagn4AD 1.87513 0.15296 12.26 < 2e-16 ***
GenderMale 0.08657 0.09536 0.91 0.36400
naprox -0.11792 0.14458 -0.82 0.41472
M:diagn2EMCI -0.00765 0.00616 -1.24 0.21407
M:diagn3LMCI 0.00468 0.00551 0.85 0.39577
M:diagn4AD 0.01798 0.00833 2.16 0.03099 *
APOE41:M 0.01426 0.00394 3.62 0.00029 ***
APOE42:M 0.02670 0.00538 4.97 6.8e-07 ***
M:GenderMale -0.00345 0.00372 -0.93 0.35400
M:edu.cat2tertiary -0.01476 0.00437 -3.37 0.00074 ***
M:edu.cat3mid -0.00671 0.00512 -1.31 0.19050
M:edu.cat4early -0.00215 0.00540 -0.40 0.69071
M:naprox 0.00629 0.00505 1.24 0.21325
---
Signif. codes: 0 '***' 0.001 '**' 0.01 '*' 0.05 '.' 0.1 ' ' 1

Number of observations: total=977, ID=616
Random effect variance(s):
Group=ID
 Variance StdDev
(Intercept) 0.3135 0.5599

Negative binomial dispersion parameter: 1.001 (std. err.: 1.0243e-06)

Log-likelihood: -1931.8

summary(neg.M.I.paracetamol)

Call:
glmmadmb(formula = neg.b.MMSE ~ AGE + APOE4 + M + edu.cat + diagn +
 diagn * M + APOE4 * M + Gender * M + edu.cat * M + parac *
 M + (1 | ID), data = MMSEdata.short, family = "nbinom1")

AIC: 3913.8

Coefficients:
 Estimate Std. Error z value Pr(>|z|)
(Intercept) -0.44337 0.15012 -2.95 0.00314 **
AGE 0.01404 0.00481 2.92 0.00350 **
APOE41 0.06260 0.09828 0.64 0.52418
APOE42 0.13569 0.14540 0.93 0.35071
M 0.00638 0.00615 1.04 0.29974
edu.cat2tertiary 0.31334 0.10983 2.85 0.00433 **
edu.cat3mid 0.31563 0.12954 2.44 0.01483 *
edu.cat4early 0.31873 0.13891 2.29 0.02176 *
diagn2EMCI 0.64778 0.15869 4.08 4.5e-05 ***
diagn3LMCI 1.07593 0.14444 7.45 9.4e-14 ***
diagn4AD 1.88937 0.15273 12.37 < 2e-16 ***
GenderMale 0.08296 0.09534 0.87 0.38419
parac -0.10763 0.10281 -1.05 0.29517
M:diagn2EMCI -0.00803 0.00615 -1.31 0.19163
M:diagn3LMCI 0.00391 0.00553 0.71 0.47879
M:diagn4AD 0.01718 0.00831 2.07 0.03869 *
APOE41:M 0.01447 0.00394 3.67 0.00024 ***
APOE42:M 0.02724 0.00539 5.05 4.4e-07 ***
M:GenderMale -0.00289 0.00375 -0.77 0.44056
M:edu.cat2tertiary -0.01499 0.00439 -3.42 0.00063 ***
M:edu.cat3mid -0.00684 0.00512 -1.34 0.18130
M:edu.cat4early -0.00202 0.00541 -0.37 0.70897
M:parac 0.00396 0.00380 1.04 0.29757
---
Signif. codes: 0 '***' 0.001 '**' 0.01 '*' 0.05 '.' 0.1 ' ' 1

Number of observations: total=977, ID=616
Random effect variance(s):
Group=ID
 Variance StdDev
(Intercept) 0.3118 0.5584

Negative binomial dispersion parameter: 1.001 (std. err.: 2.7402e-06)

Log-likelihood: -1931.92

## Month as a factor

Including month as a factor was not possible due to the extremely large degrees of freedom that would be required in the model. This, and the fact that the error distributions showed no sign of inappropriate fitting, suggest that month modelled as a numerical variable is appropriate.

try(neg.m.M.factor <- glmmadmb(neg.b.MMSE~ AGE + APOE4 + as.factor(M) + edu.cat + diagn+aspirin + diagn*as.factor(M) +APOE4*as.factor(M) + Gender*as.factor(M)+ edu.cat*as.factor(M) +parac*as.factor(M) +diclo*as.factor(M)+(1|ID), family="nbinom1", data=MMSEdata))

try(neg.m.M.factor.simple <- glmmadmb(neg.b.MMSE~ AGE + APOE4 + as.factor(M) + edu.cat + diagn +aspirin + diagn*as.factor(M) +diclo*as.factor(M)+(1|ID), family="nbinom1", data=MMSEdata))

## Years education as a numerical variable

Treating years education as a numerical variable destabilized the model, causing failure to converge.

MMSEdata$Yrs.edu<-as.numeric(MMSEdata$Yrs.edu)
try(neg.m.Yrs.edu<- glmmadmb(neg.b.MMSE~ AGE + APOE4 + M + Yrs.edu + diagn+aspirin+ diagn*M +APOE4*M + Gender*M+ Yrs.edu*M +parac*M +diclo*M+(1|ID), family="nbinom1", data=MMSEdata))
try(anova(neg.m.Yrs.edu,neg.mmse))

Analysis of Deviance Table

Model 1: neg.b.MMSE ~ AGE + APOE4 + M + Yrs.edu + diagn + aspirin + diagn * M + APOE4 * M + Gender * M + Yrs.edu * M + parac * M + diclo * M
Model 2: neg.b.MMSE ~ M + AGE + diagn + APOE4 + edu.cat + aspirin + diagn * M + APOE4 * M + Gender * M + edu.cat * M + parac * M + diclo * M
 NoPar LogLik Df Deviance Pr(>Chi)
1 24 -16949
2 28 -16940 4 17.6 0.001477 **
---
Signif. codes: 0 '***' 0.001 '**' 0.01 '*' 0.05 '.' 0.1 ' ' 1

## APOE4 and NSAIDs

There is evidence that NSAIDs interact are only protective in the presence of the APOE4 gene. Therefore, a three-way interaction term was investigated.

neg.m.APOE.diclo <- glmmadmb(neg.b.MMSE~ AGE + APOE4 + M + edu.cat + aspirin+ diagn+ diagn*M +APOE4*M + Gender*M+ edu.cat*M +parac*M +diclo*M+diclo*APOE4+(1|ID), family="nbinom1", data=MMSEdata)
summary(neg.m.APOE.diclo)

Call:
glmmadmb(formula = neg.b.MMSE ~ AGE + APOE4 + M + edu.cat + aspirin +
 diagn + diagn * M + APOE4 * M + Gender * M + edu.cat * M +
 parac * M + diclo * M + diclo * APOE4 + (1 | ID), data = MMSEdata,
 family = "nbinom1")

AIC: 33939

Coefficients:
 Estimate Std. Error z value Pr(>|z|)
(Intercept) -0.586477 0.063201 -9.28 < 2e-16 ***
AGE 0.018022 0.002598 6.94 4.0e-12 ***
APOE41 0.120373 0.042674 2.82 0.00479 **
APOE42 0.220098 0.065157 3.38 0.00073 ***
M 0.010354 0.000863 12.00 < 2e-16 ***
edu.cat2tertiary 0.200344 0.049549 4.04 5.3e-05 ***
edu.cat3mid 0.362179 0.055633 6.51 7.5e-11 ***
edu.cat4early 0.353961 0.057501 6.16 7.5e-10 ***
aspirin -0.073973 0.037121 -1.99 0.04629 *
diagn2EMCI 0.707705 0.065736 10.77 < 2e-16 ***
diagn3LMCI 1.240176 0.055552 22.32 < 2e-16 ***
diagn4AD 2.105114 0.062143 33.88 < 2e-16 ***
GenderMale 0.094606 0.040499 2.34 0.01949 *
parac -0.093733 0.045043 -2.08 0.03744 *
diclo -0.021649 0.193610 -0.11 0.91097
M:diagn2EMCI -0.003318 0.001043 -3.18 0.00147 **
M:diagn3LMCI 0.002860 0.000725 3.94 8.0e-05 ***
M:diagn4AD 0.008800 0.001437 6.12 9.2e-10 ***
APOE41:M 0.005877 0.000617 9.52 < 2e-16 ***
APOE42:M 0.007738 0.000931 8.31 < 2e-16 ***
M:GenderMale -0.001725 0.000591 -2.92 0.00353 **
M:edu.cat2tertiary -0.003741 0.000743 -5.04 4.7e-07 ***
M:edu.cat3mid -0.002254 0.000812 -2.78 0.00550 **
M:edu.cat4early -0.002486 0.000818 -3.04 0.00238 **
M:parac 0.001282 0.000589 2.18 0.02962 *
M:diclo -0.004729 0.001621 -2.92 0.00353 **
APOE41:diclo 0.011430 0.290830 0.04 0.96865
APOE42:diclo 0.490448 0.503930 0.97 0.33043
---
Signif. codes: 0 '***' 0.001 '**' 0.01 '*' 0.05 '.' 0.1 ' ' 1

Number of observations: total=8878, ID=1619
Random effect variance(s):
Group=ID
 Variance StdDev
(Intercept) 0.413 0.6427

Negative binomial dispersion parameter: 1.0582 (std. err.: 0.018202)

Log-likelihood: -16939.5

anova(neg.mmse,neg.m.APOE.diclo)

Analysis of Deviance Table

Model 1: neg.b.MMSE ~ M + AGE + diagn + APOE4 + edu.cat + aspirin + diagn * M + APOE4 * M + Gender * M + edu.cat * M + parac * M + diclo * M
Model 2: neg.b.MMSE ~ AGE + APOE4 + M + edu.cat + aspirin + diagn + diagn * M + APOE4 * M + Gender * M + edu.cat * M + parac * M + diclo * M + diclo * APOE4
 NoPar LogLik Df Deviance Pr(>Chi)
1 28 -16940
2 30 -16940 2 1 0.6065

neg.m.APOE.diclo.M <- glmmadmb(neg.b.MMSE~ AGE + APOE4 + M + edu.cat + aspirin+ diagn+ diagn*M +APOE4*M + Gender*M+ edu.cat*M +parac*M +diclo*M+diclo*APOE4*M+(1|ID), family="nbinom1", data=MMSEdata)
summary(neg.m.APOE.diclo)

Call:
glmmadmb(formula = neg.b.MMSE ~ AGE + APOE4 + M + edu.cat + aspirin +
 diagn + diagn * M + APOE4 * M + Gender * M + edu.cat * M +
 parac * M + diclo * M + diclo * APOE4 + (1 | ID), data = MMSEdata,
 family = "nbinom1")

AIC: 33939

Coefficients:
 Estimate Std. Error z value Pr(>|z|)
(Intercept) -0.586477 0.063201 -9.28 < 2e-16 ***
AGE 0.018022 0.002598 6.94 4.0e-12 ***
APOE41 0.120373 0.042674 2.82 0.00479 **
APOE42 0.220098 0.065157 3.38 0.00073 ***
M 0.010354 0.000863 12.00 < 2e-16 ***
edu.cat2tertiary 0.200344 0.049549 4.04 5.3e-05 ***
edu.cat3mid 0.362179 0.055633 6.51 7.5e-11 ***
edu.cat4early 0.353961 0.057501 6.16 7.5e-10 ***
aspirin -0.073973 0.037121 -1.99 0.04629 *
diagn2EMCI 0.707705 0.065736 10.77 < 2e-16 ***
diagn3LMCI 1.240176 0.055552 22.32 < 2e-16 ***
diagn4AD 2.105114 0.062143 33.88 < 2e-16 ***
GenderMale 0.094606 0.040499 2.34 0.01949 *
parac -0.093733 0.045043 -2.08 0.03744 *
diclo -0.021649 0.193610 -0.11 0.91097
M:diagn2EMCI -0.003318 0.001043 -3.18 0.00147 **
M:diagn3LMCI 0.002860 0.000725 3.94 8.0e-05 ***
M:diagn4AD 0.008800 0.001437 6.12 9.2e-10 ***
APOE41:M 0.005877 0.000617 9.52 < 2e-16 ***
APOE42:M 0.007738 0.000931 8.31 < 2e-16 ***
M:GenderMale -0.001725 0.000591 -2.92 0.00353 **
M:edu.cat2tertiary -0.003741 0.000743 -5.04 4.7e-07 ***
M:edu.cat3mid -0.002254 0.000812 -2.78 0.00550 **
M:edu.cat4early -0.002486 0.000818 -3.04 0.00238 **
M:parac 0.001282 0.000589 2.18 0.02962 *
M:diclo -0.004729 0.001621 -2.92 0.00353 **
APOE41:diclo 0.011430 0.290830 0.04 0.96865
APOE42:diclo 0.490448 0.503930 0.97 0.33043
---
Signif. codes: 0 '***' 0.001 '**' 0.01 '*' 0.05 '.' 0.1 ' ' 1

Number of observations: total=8878, ID=1619
Random effect variance(s):
Group=ID
 Variance StdDev
(Intercept) 0.413 0.6427

Negative binomial dispersion parameter: 1.0582 (std. err.: 0.018202)

Log-likelihood: -16939.5

anova(neg.m.APOE.diclo.M,neg.m.APOE.diclo)

Analysis of Deviance Table

Model 1: neg.b.MMSE ~ AGE + APOE4 + M + edu.cat + aspirin + diagn + diagn * M + APOE4 * M + Gender * M + edu.cat * M + parac * M + diclo * M + diclo * APOE4
Model 2: neg.b.MMSE ~ AGE + APOE4 + M + edu.cat + aspirin + diagn + diagn * M + APOE4 * M + Gender * M + edu.cat * M + parac * M + diclo * M + diclo * APOE4 * M
 NoPar LogLik Df Deviance Pr(>Chi)
1 30 -16940
2 32 -16935 2 8.4 0.015 *
---
Signif. codes: 0 '***' 0.001 '**' 0.01 '*' 0.05 '.' 0.1 ' ' 1

MMSEdata$x<-MMSEdata$diclo+MMSEdata$naprox+MMSEdata$celex+MMSEdata$parac+MMSEdata$aspirin+MMSEdata$Ibu
MMSEdata$NSAID<-1*(MMSEdata$x>0)

neg.m.APOE.NSAID <- glmmadmb(neg.b.MMSE~ AGE + APOE4 + M + edu.cat + diagn+ diagn*M +APOE4*M + Gender*M+ edu.cat*M +NSAID*M+NSAID*APOE4+(1|ID), family="nbinom1", data=MMSEdata)
summary(neg.m.APOE.NSAID)

Call:
glmmadmb(formula = neg.b.MMSE ~ AGE + APOE4 + M + edu.cat + diagn +
 diagn * M + APOE4 * M + Gender * M + edu.cat * M + NSAID *
 M + NSAID * APOE4 + (1 | ID), data = MMSEdata, family = "nbinom1")

AIC: 33939

Coefficients:
 Estimate Std. Error z value Pr(>|z|)
(Intercept) -0.509109 0.072880 -6.99 2.8e-12 ***
AGE 0.017754 0.002592 6.85 7.5e-12 ***
APOE41 0.072895 0.072338 1.01 0.31360
APOE42 0.122366 0.110740 1.10 0.26916
M 0.010264 0.001056 9.72 < 2e-16 ***
edu.cat2tertiary 0.197074 0.049499 3.98 6.9e-05 ***
edu.cat3mid 0.359029 0.055529 6.47 1.0e-10 ***
edu.cat4early 0.352386 0.057458 6.13 8.6e-10 ***
diagn2EMCI 0.714212 0.065636 10.88 < 2e-16 ***
diagn3LMCI 1.238644 0.055380 22.37 < 2e-16 ***
diagn4AD 2.095731 0.062073 33.76 < 2e-16 ***
GenderMale 0.097978 0.040091 2.44 0.01453 *
NSAID -0.201718 0.059833 -3.37 0.00075 ***
M:diagn2EMCI -0.003285 0.001040 -3.16 0.00158 **
M:diagn3LMCI 0.002989 0.000723 4.13 3.6e-05 ***
M:diagn4AD 0.008921 0.001448 6.16 7.3e-10 ***
APOE41:M 0.005836 0.000619 9.43 < 2e-16 ***
APOE42:M 0.007504 0.000920 8.16 3.5e-16 ***
M:GenderMale -0.001878 0.000592 -3.17 0.00150 **
M:edu.cat2tertiary -0.003562 0.000740 -4.81 1.5e-06 ***
M:edu.cat3mid -0.002069 0.000810 -2.55 0.01063 *
M:edu.cat4early -0.002644 0.000819 -3.23 0.00125 **
M:NSAID 0.000490 0.000806 0.61 0.54307
APOE41:NSAID 0.077151 0.085034 0.91 0.36425
APOE42:NSAID 0.162113 0.129500 1.25 0.21063
---
Signif. codes: 0 '***' 0.001 '**' 0.01 '*' 0.05 '.' 0.1 ' ' 1

Number of observations: total=8878, ID=1619
Random effect variance(s):
Group=ID
 Variance StdDev
(Intercept) 0.4123 0.6421

Negative binomial dispersion parameter: 1.0588 (std. err.: 0.018211)

Log-likelihood: -16942.5

neg.m.NSAID<- glmmadmb(neg.b.MMSE~ AGE + APOE4 + M + edu.cat + diagn+ diagn*M +APOE4*M + Gender*M+ edu.cat*M +NSAID*M+(1|ID), family="nbinom1", data=MMSEdata)
summary(neg.m.NSAID)

Call:
glmmadmb(formula = neg.b.MMSE ~ AGE + APOE4 + M + edu.cat + diagn +
 diagn * M + APOE4 * M + Gender * M + edu.cat * M + NSAID *
 M + (1 | ID), data = MMSEdata, family = "nbinom1")

AIC: 33937

Coefficients:
 Estimate Std. Error z value Pr(>|z|)
(Intercept) -0.547319 0.066231 -8.26 < 2e-16 ***
AGE 0.017766 0.002591 6.86 7.1e-12 ***
APOE41 0.126465 0.042371 2.98 0.00284 **
APOE42 0.234726 0.064770 3.62 0.00029 ***
M 0.010271 0.001057 9.72 < 2e-16 ***
edu.cat2tertiary 0.197337 0.049518 3.99 6.7e-05 ***
edu.cat3mid 0.359542 0.055557 6.47 9.7e-11 ***
edu.cat4early 0.356310 0.057419 6.21 5.5e-10 ***
diagn2EMCI 0.715000 0.065654 10.89 < 2e-16 ***
diagn3LMCI 1.239568 0.055403 22.37 < 2e-16 ***
diagn4AD 2.096789 0.062094 33.77 < 2e-16 ***
GenderMale 0.099481 0.040095 2.48 0.01310 *
NSAID -0.151098 0.042914 -3.52 0.00043 ***
M:diagn2EMCI -0.003287 0.001040 -3.16 0.00157 **
M:diagn3LMCI 0.002981 0.000723 4.12 3.7e-05 ***
M:diagn4AD 0.008910 0.001449 6.15 7.7e-10 ***
APOE41:M 0.005877 0.000617 9.52 < 2e-16 ***
APOE42:M 0.007582 0.000919 8.25 < 2e-16 ***
M:GenderMale -0.001878 0.000592 -3.17 0.00150 **
M:edu.cat2tertiary -0.003562 0.000740 -4.81 1.5e-06 ***
M:edu.cat3mid -0.002069 0.000810 -2.55 0.01064 *
M:edu.cat4early -0.002638 0.000819 -3.22 0.00128 **
M:NSAID 0.000456 0.000806 0.57 0.57160
---
Signif. codes: 0 '***' 0.001 '**' 0.01 '*' 0.05 '.' 0.1 ' ' 1

Number of observations: total=8878, ID=1619
Random effect variance(s):
Group=ID
 Variance StdDev
(Intercept) 0.4128 0.6425

Negative binomial dispersion parameter: 1.0589 (std. err.: 0.018216)

Log-likelihood: -16943.5

anova(neg.m.NSAID,neg.m.APOE.NSAID)

Analysis of Deviance Table

Model 1: neg.b.MMSE ~ AGE + APOE4 + M + edu.cat + diagn + diagn * M + APOE4 * M + Gender * M + edu.cat * M + NSAID * M
Model 2: neg.b.MMSE ~ AGE + APOE4 + M + edu.cat + diagn + diagn * M + APOE4 * M + Gender * M + edu.cat * M + NSAID * M + NSAID * APOE4
 NoPar LogLik Df Deviance Pr(>Chi)
1 25 -16944
2 27 -16943 2 2 0.3679

neg.m.NSAID.APOE.M<- glmmadmb(neg.b.MMSE~ AGE + APOE4 + M + edu.cat + diagn+ diagn*M +APOE4*M + Gender*M+ edu.cat*M +NSAID*M*APOE4+(1|ID), family="nbinom1", data=MMSEdata)
summary(neg.m.NSAID.APOE.M)

Call:
glmmadmb(formula = neg.b.MMSE ~ AGE + APOE4 + M + edu.cat + diagn +
 diagn * M + APOE4 * M + Gender * M + edu.cat * M + NSAID *
 M * APOE4 + (1 | ID), data = MMSEdata, family = "nbinom1")

AIC: 33942.4

Coefficients:
 Estimate Std. Error z value Pr(>|z|)
(Intercept) -0.503386 0.074096 -6.79 1.1e-11 ***
AGE 0.017764 0.002592 6.85 7.2e-12 ***
APOE41 0.057797 0.076194 0.76 0.44812
APOE42 0.128431 0.115200 1.11 0.26492
M 0.009939 0.001291 7.70 1.4e-14 ***
edu.cat2tertiary 0.196929 0.049488 3.98 6.9e-05 ***
edu.cat3mid 0.359072 0.055517 6.47 9.9e-11 ***
edu.cat4early 0.351934 0.057454 6.13 9.0e-10 ***
diagn2EMCI 0.714388 0.065623 10.89 < 2e-16 ***
diagn3LMCI 1.238965 0.055367 22.38 < 2e-16 ***
diagn4AD 2.095683 0.062056 33.77 < 2e-16 ***
GenderMale 0.097906 0.040080 2.44 0.01457 *
NSAID -0.208913 0.061912 -3.37 0.00074 ***
M:diagn2EMCI -0.003288 0.001040 -3.16 0.00157 **
M:diagn3LMCI 0.002968 0.000724 4.10 4.1e-05 ***
M:diagn4AD 0.008900 0.001449 6.14 8.1e-10 ***
APOE41:M 0.006747 0.001564 4.31 1.6e-05 ***
APOE42:M 0.006939 0.002479 2.80 0.00512 **
M:GenderMale -0.001869 0.000592 -3.16 0.00160 **
M:edu.cat2tertiary -0.003555 0.000740 -4.80 1.6e-06 ***
M:edu.cat3mid -0.002074 0.000810 -2.56 0.01050 *
M:edu.cat4early -0.002612 0.000822 -3.18 0.00148 **
M:NSAID 0.000865 0.001165 0.74 0.45745
APOE41:NSAID 0.096597 0.090420 1.07 0.28538
APOE42:NSAID 0.154618 0.135500 1.14 0.25383
APOE41:M:NSAID -0.001074 0.001688 -0.64 0.52460
APOE42:M:NSAID 0.000650 0.002638 0.25 0.80541
---
Signif. codes: 0 '***' 0.001 '**' 0.01 '*' 0.05 '.' 0.1 ' ' 1

Number of observations: total=8878, ID=1619
Random effect variance(s):
Group=ID
 Variance StdDev
(Intercept) 0.4119 0.6418

Negative binomial dispersion parameter: 1.0589 (std. err.: 0.01821)

Log-likelihood: -16942.2

anova(neg.m.NSAID.APOE.M,neg.m.APOE.NSAID)

Analysis of Deviance Table

Model 1: neg.b.MMSE ~ AGE + APOE4 + M + edu.cat + diagn + diagn * M + APOE4 * M + Gender * M + edu.cat * M + NSAID * M + NSAID * APOE4
Model 2: neg.b.MMSE ~ AGE + APOE4 + M + edu.cat + diagn + diagn * M + APOE4 * M + Gender * M + edu.cat * M + NSAID * M * APOE4
 NoPar LogLik Df Deviance Pr(>Chi)
1 27 -16943
2 29 -16942 2 0.6 0.7408

AIC(neg.m.APOE.diclo)

[1] 33939

AIC(neg.m.APOE.diclo.M)

[1] 33934.6

AIC(neg.m.APOE.NSAID)

[1] 33939

AIC(neg.m.NSAID)

[1] 33937

AIC(neg.m.NSAID.APOE.M)

[1] 33942.4

AIC(neg.mmse)

[1] 33936

## looking at the distribution of the residuals in the final model, for each variable separately

Residuals within each explanatory variable collectively and within each individual show no trends and are homoscedastic centred around zero. Therefore, this model is accurate and will be used to investigate the effects of the input variables (including pain medications) going forward.

augDat <- data.frame(MMSEdata,resid=residuals(neg.mmse,type="pearson"),
fitted=fitted(neg.mmse))
ggplot(augDat,aes(x=Gender,y=resid,group=ID))+geom_boxplot()+coord_flip()

Figure description: Box Plots of the Pearson residuals per participant ID showing centred clustering around zero. Grouped by gender.


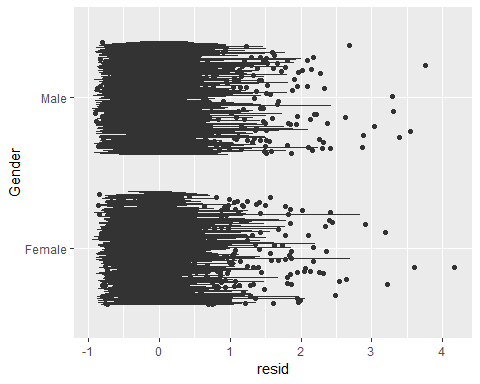


ggplot(augDat,aes(x=AGE,y=resid,group=ID))+geom_boxplot()+coord_flip()

Figure description: Box Plots of the Pearson residuals per participant ID showing centred clustering around zero. Plotted against age.


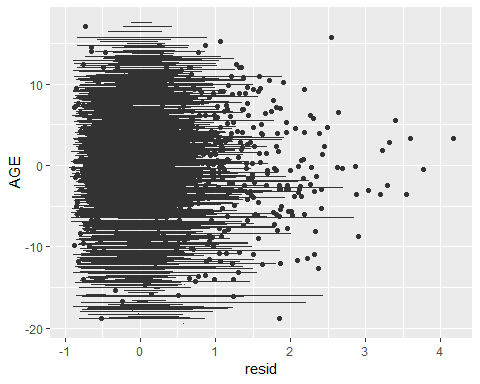


ggplot(augDat,aes(x=edu.cat,y=resid,group=ID))+geom_boxplot()+coord_flip()

Figure description: Box Plots of the Pearson residuals per participant ID showing centred clustering around zero. Grouped by education level.

ggplot(augDat,aes(x=diagn,y=resid,group=ID))+geom_boxplot()+coord_flip()

Figure description: Box Plots of the Pearson residuals per participant ID showing centred clustering around zero. Grouped by cognitive diagnosis.

ggplot(augDat,aes(x=APOE4,y=resid,group=ID))+geom_boxplot()+coord_flip()

Figure description: Box Plots of the Pearson residuals per participant ID showing centred clustering around zero. Grouped by ApoE4 status.

ggplot(augDat,aes(x=M,y=resid,group=ID))+geom_boxplot()+coord_flip()

Figure description: Box Plots of the Pearson residuals per participant ID showing centred clustering around zero. Plotted against time (months).

ggplot(augDat,aes(x=diclo,y=resid,group=ID))+geom_boxplot()+coord_flip()

Figure description: Box Plots of the Pearson residuals per participant ID showing centred clustering around zero. Grouped by diclofenac use.

ggplot(augDat,aes(x=parac,y=resid,group=ID))+geom_boxplot()+coord_flip()

Figure description: Box Plots of the Pearson residuals per participant ID showing centred clustering around zero. Grouped by paracetamol use.

ggplot(augDat,aes(x=Gender,y=resid))+geom_boxplot()+coord_flip()

Figure description: Box Plots of the Pearson residuals at the participant level, showing centred clustering around zero. Plotted by gender.

ggplot(augDat,aes(x=AGE,y=resid))+geom_point()+coord_flip() +geom_smooth(method=lm)

Figure description: Box Plots of the Pearson residuals at the participant level, showing centred clustering around zero. Plotted by age.

ggplot(augDat,aes(x=edu.cat,y=resid))+geom_boxplot()+coord_flip()

Figure description: Box Plots of the Pearson residuals at the participant level, showing centred clustering around zero. Plotted by education level.

ggplot(augDat,aes(x=diagn,y=resid))+geom_boxplot()+coord_flip()

Figure description: Box Plots of the Pearson residuals at the participant level, showing centred clustering around zero. Plotted by cognitive diagnosis.

ggplot(augDat,aes(x=as.factor(APOE4),y=resid))+geom_boxplot()+coord_flip()

Figure description: Box Plots of the Pearson residuals at the participant level, showing centred clustering around zero. Plotted by ApoE4 status.

ggplot(augDat,aes(x=as.factor(M),y=resid))+geom_boxplot()+coord_flip()

Figure description: Box Plots of the Pearson residuals at the participant level, showing centred clustering around zero. Plotted by time (month).

ggplot(augDat,aes(x=as.factor(diclo),y=resid))+geom_boxplot()+coord_flip()

Figure description: Box Plots of the Pearson residuals at the participant level, showing centred clustering around zero. Plotted by diclofenac use.

ggplot(augDat,aes(x=as.factor(parac),y=resid))+geom_boxplot()+coord_flip()

Figure description: Box Plots of the Pearson residuals at the participant level, showing centred clustering around zero. Plotted by paracetamol use.

## Checking for multicolinearity

cov2cor(vcov(neg.mmse))

(Intercept) M AGE diagn2EMCI diagn3LMCI
(Intercept) 1.0000 0.1126 -0.0395 -0.0077 -0.0440
M 0.1126 1.0000 0.0134 -0.1140 -0.1141
AGE -0.0395 0.0134 1.0000 -0.0530 -0.0176
diagn2EMCI -0.0077 -0.1140 -0.0530 1.0000 0.0125
diagn3LMCI -0.0440 -0.1141 -0.0176 0.0125 1.0000
diagn4AD -0.2847 -0.2739 -0.0324 0.1227 0.2973
APOE41 -0.0292 -0.0585 -0.0090 -0.0159 -0.0088
APOE42 -0.0300 -0.0538 0.0282 0.0015 -0.0108
edu.cat2tertiary -0.0027 0.0081 0.0014 0.0127 -0.0049
edu.cat3mid -0.0176 0.0097 0.0244 0.0020 0.0009
edu.cat4early -0.0274 0.0119 0.0045 -0.0160 0.0123
aspirin 0.0639 0.1479 0.0231 -0.0235 0.0222
GenderMale -0.0131 0.0194 -0.0073 -0.0048 0.0128
parac 0.0569 0.1074 0.0125 -0.0030 0.0156
diclo 0.0204 0.0681 0.0041 -0.0174 -0.0015
M:diagn2EMCI 0.0055 -0.0467 0.0030 -0.0099 -0.0066
M:diagn3LMCI 0.0153 -0.3742 -0.0007 -0.0161 -0.0175
M:diagn4AD 0.0084 -0.2487 -0.0042 -0.0085 -0.0066
M:APOE41 -0.0141 -0.1019 0.0019 0.0011 0.0033
M:APOE42 -0.0061 -0.1031 -0.0050 -0.0013 -0.0022
M:GenderMale 0.0030 0.0403 0.0027 -0.0010 0.0030
M:edu.cat2tertiary 0.0126 0.0259 -0.0028 -0.0020 -0.0054
M:edu.cat3mid 0.0089 -0.0574 -0.0012 -0.0019 -0.0001
M:edu.cat4early 0.0170 -0.0852 0.0013 -0.0015 0.0022
M:parac -0.0109 -0.0618 0.0014 0.0012 -0.0044
M:diclo 0.0062 0.0183 -0.0002 -0.0009 -0.0053
 diagn4AD APOE41 APOE42 edu.cat2tertiary edu.cat3mid
(Intercept) -0.2847 -0.0292 -0.0300 -0.0027 -0.0176
M -0.2739 -0.0585 -0.0538 0.0081 0.0097
AGE -0.0324 -0.0090 0.0282 0.0014 0.0244
diagn2EMCI 0.1227 -0.0159 0.0015 0.0127 0.0020
diagn3LMCI 0.2973 -0.0088 -0.0108 -0.0049 0.0009
diagn4AD 1.0000 0.0059 0.0170 -0.0071 0.0167
APOE41 0.0059 1.0000 0.0633 0.0233 0.0055
APOE42 0.0170 0.0633 1.0000 -0.0027 0.0147
edu.cat2tertiary -0.0071 0.0233 -0.0027 1.0000 0.0068
edu.cat3mid 0.0167 0.0055 0.0147 0.0068 1.0000
edu.cat4early 0.0197 0.0043 0.0040 0.0448 0.0518
aspirin 0.0111 -0.0148 0.0047 -0.0163 -0.0247
GenderMale 0.0104 0.0068 0.0075 0.0036 -0.0104
parac 0.0053 0.0107 0.0180 -0.0010 0.0121
diclo -0.0057 -0.0179 0.0082 0.0021 -0.0239
M:diagn2EMCI -0.0162 -0.0024 -0.0034 -0.0018 -0.0020
M:diagn3LMCI -0.0817 0.0002 -0.0056 -0.0093 0.0007
M:diagn4AD -0.0589 -0.0050 -0.0096 0.0005 -0.0025
M:APOE41 -0.0118 0.0186 0.0079 -0.0086 -0.0013
M:APOE42 -0.0194 0.0062 0.0110 -0.0009 -0.0032
M:GenderMale 0.0064 0.0011 -0.0012 0.0020 -0.0066
M:edu.cat2tertiary -0.0017 -0.0077 -0.0022 0.0367 -0.0029
M:edu.cat3mid -0.0158 -0.0001 -0.0030 -0.0036 0.0225
M:edu.cat4early -0.0217 0.0007 -0.0019 -0.0039 -0.0049
M:parac -0.0107 -0.0003 0.0010 -0.0051 0.0003
M:diclo -0.0028 0.0024 -0.0022 0.0043 0.0007
 edu.cat4early aspirin GenderMale parac diclo
(Intercept) -0.0274 0.0639 -0.0131 0.0569 0.0204
M 0.0119 0.1479 0.0194 0.1074 0.0681
AGE 0.0045 0.0231 -0.0073 0.0125 0.0041
diagn2EMCI -0.0160 -0.0235 -0.0048 -0.0030 -0.0174
diagn3LMCI 0.0123 0.0222 0.0128 0.0156 -0.0015
diagn4AD 0.0197 0.0111 0.0104 0.0053 -0.0057
APOE41 0.0043 -0.0148 0.0068 0.0107 -0.0179
APOE42 0.0040 0.0047 0.0075 0.0180 0.0082
edu.cat2tertiary 0.0448 -0.0163 0.0036 -0.0010 0.0021
edu.cat3mid 0.0518 -0.0247 -0.0104 0.0121 -0.0239
edu.cat4early 1.0000 0.0034 0.0050 0.0163 0.0104
aspirin 0.0034 1.0000 0.0219 -0.0028 0.0188
GenderMale 0.0050 0.0219 1.0000 0.0020 0.0052
parac 0.0163 -0.0028 0.0020 1.0000 0.0221
diclo 0.0104 0.0188 0.0052 0.0221 1.0000
M:diagn2EMCI -0.0006 0.0010 -0.0018 -0.0007 -0.0033
M:diagn3LMCI 0.0034 0.0211 0.0017 0.0148 -0.0025
M:diagn4AD -0.0006 0.0091 0.0001 0.0098 0.0012
M:APOE41 -0.0004 0.0068 -0.0016 0.0017 0.0035
M:APOE42 -0.0022 0.0031 -0.0043 0.0038 -0.0025
M:GenderMale -0.0013 -0.0015 0.0275 -0.0079 -0.0018
M:edu.cat2tertiary -0.0022 0.0034 -0.0025 -0.0021 0.0058
M:edu.cat3mid -0.0045 0.0049 0.0009 0.0020 0.0011
M:edu.cat4early 0.0260 0.0061 0.0005 0.0035 0.0059
M:parac -0.0016 0.0047 -0.0099 0.0082 0.0009
M:diclo 0.0035 0.0023 -0.0006 0.0011 0.0254
 M:diagn2EMCI M:diagn3LMCI M:diagn4AD M:APOE41 M:APOE42
(Intercept) 0.0055 0.0153 0.0084 -0.0141 -0.0061
M -0.0467 -0.3742 -0.2487 -0.1019 -0.1031
AGE 0.0030 -0.0007 -0.0042 0.0019 -0.0050
diagn2EMCI -0.0099 -0.0161 -0.0085 0.0011 -0.0013
diagn3LMCI -0.0066 -0.0175 -0.0066 0.0033 -0.0022
diagn4AD -0.0162 -0.0817 -0.0589 -0.0118 -0.0194
APOE41 -0.0024 0.0002 -0.0050 0.0186 0.0062
APOE42 -0.0034 -0.0056 -0.0096 0.0079 0.0110
edu.cat2tertiary -0.0018 -0.0093 0.0005 -0.0086 -0.0009
edu.cat3mid -0.0020 0.0007 -0.0025 -0.0013 -0.0032
edu.cat4early -0.0006 0.0034 -0.0006 -0.0004 -0.0022
aspirin 0.0010 0.0211 0.0091 0.0068 0.0031
GenderMale -0.0018 0.0017 0.0001 -0.0016 -0.0043
parac -0.0007 0.0148 0.0098 0.0017 0.0038
diclo -0.0033 -0.0025 0.0012 0.0035 -0.0025
M:diagn2EMCI 1.0000 0.1806 0.1188 -0.0120 -0.0106
M:diagn3LMCI 0.1806 1.0000 0.2985 -0.0224 -0.0804
M:diagn4AD 0.1188 0.2985 1.0000 0.0144 -0.0051
M:APOE41 -0.0120 -0.0224 0.0144 1.0000 0.1866
M:APOE42 -0.0106 -0.0804 -0.0051 0.1866 1.0000
M:GenderMale -0.0187 0.0480 0.0108 0.0449 0.0179
M:edu.cat2tertiary 0.0069 -0.0364 0.0014 0.0056 0.0344
M:edu.cat3mid -0.0361 0.0582 0.0152 0.0076 -0.0395
M:edu.cat4early -0.0312 0.0116 0.0065 0.0452 0.0586
M:parac 0.0146 -0.0004 0.0104 0.0205 0.0075
M:diclo -0.0055 -0.0275 -0.0067 0.0077 -0.0909
 M:GenderMale M:edu.cat2tertiary M:edu.cat3mid
(Intercept) 0.0030 0.0126 0.0089
M 0.0403 0.0259 -0.0574
AGE 0.0027 -0.0028 -0.0012
diagn2EMCI -0.0010 -0.0020 -0.0019
diagn3LMCI 0.0030 -0.0054 -0.0001
diagn4AD 0.0064 -0.0017 -0.0158
APOE41 0.0011 -0.0077 -0.0001
APOE42 -0.0012 -0.0022 -0.0030
edu.cat2tertiary 0.0020 0.0367 -0.0036
edu.cat3mid -0.0066 -0.0029 0.0225
edu.cat4early -0.0013 -0.0022 -0.0045
aspirin -0.0015 0.0034 0.0049
GenderMale 0.0275 -0.0025 0.0009
parac -0.0079 -0.0021 0.0020
diclo -0.0018 0.0058 0.0011
M:diagn2EMCI -0.0187 0.0069 -0.0361
M:diagn3LMCI 0.0480 -0.0364 0.0582
M:diagn4AD 0.0108 0.0014 0.0152
M:APOE41 0.0449 0.0056 0.0076
M:APOE42 0.0179 0.0344 -0.0395
M:GenderMale 1.0000 -0.0474 -0.0650
M:edu.cat2tertiary -0.0474 1.0000 -0.0008
M:edu.cat3mid -0.0650 -0.0008 1.0000
M:edu.cat4early 0.0291 0.0985 0.1324
M:parac -0.1157 -0.0741 0.0529
M:diclo -0.0336 0.0676 0.0022
 M:edu.cat4early M:parac M:diclo
(Intercept) 0.0170 -0.0109 0.0062
M -0.0852 -0.0618 0.0183
AGE 0.0013 0.0014 -0.0002
diagn2EMCI -0.0015 0.0012 -0.0009
diagn3LMCI 0.0022 -0.0044 -0.0053
diagn4AD -0.0217 -0.0107 -0.0028
APOE41 0.0007 -0.0003 0.0024
APOE42 -0.0019 0.0010 -0.0022
edu.cat2tertiary -0.0039 -0.0051 0.0043
edu.cat3mid -0.0049 0.0003 0.0007
edu.cat4early 0.0260 -0.0016 0.0035
aspirin 0.0061 0.0047 0.0023
GenderMale 0.0005 -0.0099 -0.0006
parac 0.0035 0.0082 0.0011
diclo 0.0059 0.0009 0.0254
M:diagn2EMCI -0.0312 0.0146 -0.0055
M:diagn3LMCI 0.0116 -0.0004 -0.0275
M:diagn4AD 0.0065 0.0104 -0.0067
M:APOE41 0.0452 0.0205 0.0077
M:APOE42 0.0586 0.0075 -0.0909
M:GenderMale 0.0291 -0.1157 -0.0336
M:edu.cat2tertiary 0.0985 -0.0741 0.0676
M:edu.cat3mid 0.1324 0.0529 0.0022
M:edu.cat4early 1.0000 0.0616 0.0572
M:parac 0.0616 1.0000 -0.0054
M:diclo 0.0572 -0.0054 1.0000

abs(cov2cor(vcov(neg.mmse)))>0.4

(Intercept) M AGE diagn2EMCI diagn3LMCI diagn4AD
(Intercept) TRUE FALSE FALSE FALSE FALSE FALSE
M FALSE TRUE FALSE FALSE FALSE FALSE
AGE FALSE FALSE TRUE FALSE FALSE FALSE
diagn2EMCI FALSE FALSE FALSE TRUE FALSE FALSE
diagn3LMCI FALSE FALSE FALSE FALSE TRUE FALSE
diagn4AD FALSE FALSE FALSE FALSE FALSE TRUE
APOE41 FALSE FALSE FALSE FALSE FALSE FALSE
APOE42 FALSE FALSE FALSE FALSE FALSE FALSE
edu.cat2tertiary FALSE FALSE FALSE FALSE FALSE FALSE
edu.cat3mid FALSE FALSE FALSE FALSE FALSE FALSE
edu.cat4early FALSE FALSE FALSE FALSE FALSE FALSE
aspirin FALSE FALSE FALSE FALSE FALSE FALSE
GenderMale FALSE FALSE FALSE FALSE FALSE FALSE
parac FALSE FALSE FALSE FALSE FALSE FALSE
diclo FALSE FALSE FALSE FALSE FALSE FALSE
M:diagn2EMCI FALSE FALSE FALSE FALSE FALSE FALSE
M:diagn3LMCI FALSE FALSE FALSE FALSE FALSE FALSE
M:diagn4AD FALSE FALSE FALSE FALSE FALSE FALSE
M:APOE41 FALSE FALSE FALSE FALSE FALSE FALSE
M:APOE42 FALSE FALSE FALSE FALSE FALSE FALSE
M:GenderMale FALSE FALSE FALSE FALSE FALSE FALSE
M:edu.cat2tertiary FALSE FALSE FALSE FALSE FALSE FALSE
M:edu.cat3mid FALSE FALSE FALSE FALSE FALSE FALSE
M:edu.cat4early FALSE FALSE FALSE FALSE FALSE FALSE
M:parac FALSE FALSE FALSE FALSE FALSE FALSE
M:diclo FALSE FALSE FALSE FALSE FALSE FALSE
 APOE41 APOE42 edu.cat2tertiary edu.cat3mid
(Intercept) FALSE FALSE FALSE FALSE
M FALSE FALSE FALSE FALSE
AGE FALSE FALSE FALSE FALSE
diagn2EMCI FALSE FALSE FALSE FALSE
diagn3LMCI FALSE FALSE FALSE FALSE
diagn4AD FALSE FALSE FALSE FALSE
APOE41 TRUE FALSE FALSE FALSE
APOE42 FALSE TRUE FALSE FALSE
edu.cat2tertiary FALSE FALSE TRUE FALSE
edu.cat3mid FALSE FALSE FALSE TRUE
edu.cat4early FALSE FALSE FALSE FALSE
aspirin FALSE FALSE FALSE FALSE
GenderMale FALSE FALSE FALSE FALSE
parac FALSE FALSE FALSE FALSE
diclo FALSE FALSE FALSE FALSE
M:diagn2EMCI FALSE FALSE FALSE FALSE
M:diagn3LMCI FALSE FALSE FALSE FALSE
M:diagn4AD FALSE FALSE FALSE FALSE
M:APOE41 FALSE FALSE FALSE FALSE
M:APOE42 FALSE FALSE FALSE FALSE
M:GenderMale FALSE FALSE FALSE FALSE
M:edu.cat2tertiary FALSE FALSE FALSE FALSE
M:edu.cat3mid FALSE FALSE FALSE FALSE
M:edu.cat4early FALSE FALSE FALSE FALSE
M:parac FALSE FALSE FALSE FALSE
M:diclo FALSE FALSE FALSE FALSE
 edu.cat4early aspirin GenderMale parac diclo
(Intercept) FALSE FALSE FALSE FALSE FALSE
M FALSE FALSE FALSE FALSE FALSE
AGE FALSE FALSE FALSE FALSE FALSE
diagn2EMCI FALSE FALSE FALSE FALSE FALSE
diagn3LMCI FALSE FALSE FALSE FALSE FALSE
diagn4AD FALSE FALSE FALSE FALSE FALSE
APOE41 FALSE FALSE FALSE FALSE FALSE
APOE42 FALSE FALSE FALSE FALSE FALSE
edu.cat2tertiary FALSE FALSE FALSE FALSE FALSE
edu.cat3mid FALSE FALSE FALSE FALSE FALSE
edu.cat4early TRUE FALSE FALSE FALSE FALSE
aspirin FALSE TRUE FALSE FALSE FALSE
GenderMale FALSE FALSE TRUE FALSE FALSE
parac FALSE FALSE FALSE TRUE FALSE
diclo FALSE FALSE FALSE FALSE TRUE
M:diagn2EMCI FALSE FALSE FALSE FALSE FALSE
M:diagn3LMCI FALSE FALSE FALSE FALSE FALSE
M:diagn4AD FALSE FALSE FALSE FALSE FALSE
M:APOE41 FALSE FALSE FALSE FALSE FALSE
M:APOE42 FALSE FALSE FALSE FALSE FALSE
M:GenderMale FALSE FALSE FALSE FALSE FALSE
M:edu.cat2tertiary FALSE FALSE FALSE FALSE FALSE
M:edu.cat3mid FALSE FALSE FALSE FALSE FALSE
M:edu.cat4early FALSE FALSE FALSE FALSE FALSE
M:parac FALSE FALSE FALSE FALSE FALSE
M:diclo FALSE FALSE FALSE FALSE FALSE
 M:diagn2EMCI M:diagn3LMCI M:diagn4AD M:APOE41 M:APOE42
(Intercept) FALSE FALSE FALSE FALSE FALSE
M FALSE FALSE FALSE FALSE FALSE
AGE FALSE FALSE FALSE FALSE FALSE
diagn2EMCI FALSE FALSE FALSE FALSE FALSE
diagn3LMCI FALSE FALSE FALSE FALSE FALSE
diagn4AD FALSE FALSE FALSE FALSE FALSE
APOE41 FALSE FALSE FALSE FALSE FALSE
APOE42 FALSE FALSE FALSE FALSE FALSE
edu.cat2tertiary FALSE FALSE FALSE FALSE FALSE
edu.cat3mid FALSE FALSE FALSE FALSE FALSE
edu.cat4early FALSE FALSE FALSE FALSE FALSE
aspirin FALSE FALSE FALSE FALSE FALSE
GenderMale FALSE FALSE FALSE FALSE FALSE
parac FALSE FALSE FALSE FALSE FALSE
diclo FALSE FALSE FALSE FALSE FALSE
M:diagn2EMCI TRUE FALSE FALSE FALSE FALSE
M:diagn3LMCI FALSE TRUE FALSE FALSE FALSE
M:diagn4AD FALSE FALSE TRUE FALSE FALSE
M:APOE41 FALSE FALSE FALSE TRUE FALSE
M:APOE42 FALSE FALSE FALSE FALSE TRUE
M:GenderMale FALSE FALSE FALSE FALSE FALSE
M:edu.cat2tertiary FALSE FALSE FALSE FALSE FALSE
M:edu.cat3mid FALSE FALSE FALSE FALSE FALSE
M:edu.cat4early FALSE FALSE FALSE FALSE FALSE
M:parac FALSE FALSE FALSE FALSE FALSE
M:diclo FALSE FALSE FALSE FALSE FALSE
 M:GenderMale M:edu.cat2tertiary M:edu.cat3mid
(Intercept) FALSE FALSE FALSE
M FALSE FALSE FALSE
AGE FALSE FALSE FALSE
diagn2EMCI FALSE FALSE FALSE
diagn3LMCI FALSE FALSE FALSE
diagn4AD FALSE FALSE FALSE
APOE41 FALSE FALSE FALSE
APOE42 FALSE FALSE FALSE
edu.cat2tertiary FALSE FALSE FALSE
edu.cat3mid FALSE FALSE FALSE
edu.cat4early FALSE FALSE FALSE
aspirin FALSE FALSE FALSE
GenderMale FALSE FALSE FALSE
parac FALSE FALSE FALSE
diclo FALSE FALSE FALSE
M:diagn2EMCI FALSE FALSE FALSE
M:diagn3LMCI FALSE FALSE FALSE
M:diagn4AD FALSE FALSE FALSE
M:APOE41 FALSE FALSE FALSE
M:APOE42 FALSE FALSE FALSE
M:GenderMale TRUE FALSE FALSE
M:edu.cat2tertiary FALSE TRUE FALSE
M:edu.cat3mid FALSE FALSE TRUE
M:edu.cat4early FALSE FALSE FALSE
M:parac FALSE FALSE FALSE
M:diclo FALSE FALSE FALSE
 M:edu.cat4early M:parac M:diclo
(Intercept) FALSE FALSE FALSE
M FALSE FALSE FALSE
AGE FALSE FALSE FALSE
diagn2EMCI FALSE FALSE FALSE
diagn3LMCI FALSE FALSE FALSE
diagn4AD FALSE FALSE FALSE
APOE41 FALSE FALSE FALSE
APOE42 FALSE FALSE FALSE
edu.cat2tertiary FALSE FALSE FALSE
edu.cat3mid FALSE FALSE FALSE
edu.cat4early FALSE FALSE FALSE
aspirin FALSE FALSE FALSE
GenderMale FALSE FALSE FALSE
parac FALSE FALSE FALSE
diclo FALSE FALSE FALSE
M:diagn2EMCI FALSE FALSE FALSE
M:diagn3LMCI FALSE FALSE FALSE
M:diagn4AD FALSE FALSE FALSE
M:APOE41 FALSE FALSE FALSE
M:APOE42 FALSE FALSE FALSE
M:GenderMale FALSE FALSE FALSE
M:edu.cat2tertiary FALSE FALSE FALSE
M:edu.cat3mid FALSE FALSE FALSE
M:edu.cat4early TRUE FALSE FALSE
M:parac FALSE TRUE FALSE
M:diclo FALSE FALSE TRUE

## Checking other distributions

Now that the final model has been generated, model families were investigated to see if the negative binomial model with the parameterization method of variance proportional to the mean method is still the most optimal model as measured by AIC.

try(glmerB1.mmse<-glmer(cbind(success,fail)~M+AGE+ diagn+ APOE4+edu.cat + aspirin+ diagn*M +APOE4*M + Gender*M+ edu.cat*M +parac*M +diclo*M+(1|ID), family="binomial", data=MMSEdata))
try(summary(glmerB1.mmse))

Generalized linear mixed model fit by maximum likelihood (Laplace
 Approximation) [glmerMod]
 Family: binomial ( logit )
Formula:
cbind(success, fail) ~ M + AGE + diagn + APOE4 + edu.cat + aspirin +
 diagn * M + APOE4 * M + Gender * M + edu.cat * M + parac *
 M + diclo * M + (1 | ID)
 Data: MMSEdata

 AIC BIC logLik deviance df.resid
 34398.9 34590.4 -17172.5 34344.9 8851

Scaled residuals:
 Min 1Q Median 3Q Max
-7.2833 -0.5548 0.1652 0.7518 3.2329

Random effects:
 Groups Name Variance Std.Dev.
 ID (Intercept) 0.5915 0.7691
Number of obs: 8878, groups: ID, 1619

Fixed effects:
 Estimate Std. Error z value Pr(>|z|)
(Intercept) 4.0457935 0.0709888 56.992 < 2e-16 ***
M -0.0123987 0.0009109 -13.612 < 2e-16 ***
AGE -0.0198226 0.0030303 -6.541 6.10e-11 ***
diagn2EMCI -0.7589389 0.0735403 -10.320 < 2e-16 ***
diagn3LMCI -1.3119183 0.0625963 -20.958 < 2e-16 ***
diagn4AD -2.3705168 0.0710191 -33.379 < 2e-16 ***
APOE41 -0.1138966 0.0490337 -2.323 0.020189 *
APOE42 -0.2007254 0.0759452 -2.643 0.008217 **
edu.cat2tertiary -0.2454184 0.0570209 -4.304 1.68e-05 ***
edu.cat3mid -0.4230724 0.0641749 -6.592 4.32e-11 ***
edu.cat4early -0.4060740 0.0666984 -6.088 1.14e-09 ***
aspirin 0.0916950 0.0432400 2.121 0.033955 *
GenderMale -0.1330080 0.0467888 -2.843 0.004473 **
parac 0.1116710 0.0519934 2.148 0.031730 *
diclo -0.0158003 0.1683552 -0.094 0.925227
M:diagn2EMCI 0.0038679 0.0010681 3.621 0.000293 ***
M:diagn3LMCI -0.0057309 0.0007595 -7.545 4.52e-14 ***
M:diagn4AD -0.0179992 0.0017122 -10.512 < 2e-16 ***
M:APOE41 -0.0078183 0.0006723 -11.630 < 2e-16 ***
M:APOE42 -0.0121701 0.0010775 -11.295 < 2e-16 ***
M:GenderMale 0.0032389 0.0006492 4.989 6.06e-07 ***
M:edu.cat2tertiary 0.0052033 0.0008044 6.469 9.87e-11 ***
M:edu.cat3mid 0.0032502 0.0008854 3.671 0.000241 ***
M:edu.cat4early 0.0030098 0.0009090 3.311 0.000929 ***
M:parac -0.0013581 0.0006415 -2.117 0.034252 *
M:diclo 0.0051679 0.0017648 2.928 0.003408 **
---
Signif. codes: 0 '***' 0.001 '**' 0.01 '*' 0.05 '.' 0.1 ' ' 1

Correlation matrix not shown by default, as p = 26 > 12.
Use print(x, correlation=TRUE) or
 vcov(x) if you need it

convergence code: 0
Model failed to converge with max|grad| = 0.885614 (tol = 0.001, component 1)
Model is nearly unidentifiable: very large eigenvalue
 - Rescale variables?
failure to converge in 10000 evaluations

try(glmerB2.mmse<-glmer(cbind(success,fail)~M+AGE+ diagn+ APOE4+edu.cat + aspirin+ diagn*M +APOE4*M + Gender*M+ edu.cat*M +parac*M +diclo*M+(1|ID), family=binomial(link=probit), data=MMSEdata))
summary(glmerB2.mmse)

Generalized linear mixed model fit by maximum likelihood (Laplace
 Approximation) [glmerMod]
 Family: binomial ( probit )
Formula:
cbind(success, fail) ~ M + AGE + diagn + APOE4 + edu.cat + aspirin +
 diagn * M + APOE4 * M + Gender * M + edu.cat * M + parac *
 M + diclo * M + (1 | ID)
 Data: MMSEdata

 AIC BIC logLik deviance df.resid
 34621.3 34812.8 -17283.6 34567.3 8851

Scaled residuals:
 Min 1Q Median 3Q Max
-7.7096 -0.5642 0.1721 0.7426 3.1295

Random effects:
 Groups Name Variance Std.Dev.
 ID (Intercept) 0.151 0.3886
Number of obs: 8878, groups: ID, 1619

Fixed effects:
 Estimate Std. Error z value Pr(>|z|)
(Intercept) 2.1256106 0.0336725 63.126 < 2e-16 ***
M -0.0051989 0.0003427 -15.170 < 2e-16 ***
AGE -0.0094159 0.0015263 -6.169 6.86e-10 ***
diagn2EMCI -0.3426741 0.0358568 -9.557 < 2e-16 ***
diagn3LMCI -0.6127038 0.0309480 -19.798 < 2e-16 ***
diagn4AD -1.1817406 0.0355171 -33.272 < 2e-16 ***
APOE41 -0.0522684 0.0248358 -2.105 0.035329 *
APOE42 -0.0817331 0.0386214 -2.116 0.034322 *
edu.cat2tertiary -0.1092294 0.0284663 -3.837 0.000124 ***
edu.cat3mid -0.1925867 0.0324290 -5.939 2.87e-09 ***
edu.cat4early -0.1849245 0.0333194 -5.550 2.86e-08 ***
aspirin 0.0422692 0.0217214 1.946 0.051659 .
GenderMale -0.0615304 0.0234877 -2.620 0.008801 **
parac 0.0542463 0.0263254 2.061 0.039340 *
diclo -0.0052408 0.0835016 -0.063 0.949955
M:diagn2EMCI 0.0017444 0.0005109 3.414 0.000640 ***
M:diagn3LMCI -0.0037724 0.0004094 -9.214 < 2e-16 ***
M:diagn4AD -0.0118397 0.0010058 -11.771 < 2e-16 ***
M:APOE41 -0.0041963 0.0003858 -10.877 < 2e-16 ***
M:APOE42 -0.0072745 0.0005986 -12.152 < 2e-16 ***
M:GenderMale 0.0016534 0.0003486 4.743 2.10e-06 ***
M:edu.cat2tertiary 0.0023051 0.0004238 5.439 5.36e-08 ***
M:edu.cat3mid 0.0011547 0.0005149 2.243 0.024923 *
M:edu.cat4early 0.0009740 0.0004466 2.181 0.029181 *
M:parac -0.0006795 0.0003724 -1.825 0.068016 .
M:diclo 0.0023249 0.0008882 2.618 0.008857 **
---
Signif. codes: 0 '***' 0.001 '**' 0.01 '*' 0.05 '.' 0.1 ' ' 1

Correlation matrix not shown by default, as p = 26 > 12.
Use print(x, correlation=TRUE) or
 vcov(x) if you need it

convergence code: 0
Model failed to converge with max|grad| = 0.985004 (tol = 0.001, component 1)
Model is nearly unidentifiable: very large eigenvalue
 - Rescale variables?
failure to converge in 10000 evaluations

glmerB3.mmse<-glmer(cbind(success,fail)~M+AGE+ diagn+ APOE4+edu.cat + aspirin+ diagn*M +APOE4*M + Gender*M+ edu.cat*M +parac*M +diclo*M+(1|ID), family=binomial(link=cloglog), data=MMSEdata)
summary(glmerB3.mmse)

Generalized linear mixed model fit by maximum likelihood (Laplace
 Approximation) [glmerMod]
 Family: binomial ( cloglog )
Formula:
cbind(success, fail) ~ M + AGE + diagn + APOE4 + edu.cat + aspirin +
 diagn * M + APOE4 * M + Gender * M + edu.cat * M + parac *
 M + diclo * M + (1 | ID)
 Data: MMSEdata

 AIC BIC logLik deviance df.resid
 35043.6 35235.1 -17494.8 34989.6 8851

Scaled residuals:
 Min 1Q Median 3Q Max
-8.0721 -0.5798 0.1716 0.7350 3.0193

Random effects:
 Groups Name Variance Std.Dev.
 ID (Intercept) 0.08917 0.2986
Number of obs: 8878, groups: ID, 1619

Fixed effects:
 Estimate Std. Error z value Pr(>|z|)
(Intercept) 1.4079800 0.0258504 54.467 < 2e-16 ***
M -0.0030783 0.0003283 -9.376 < 2e-16 ***
AGE -0.0065993 0.0011718 -5.632 1.78e-08 ***
diagn2EMCI -0.2309073 0.0268067 -8.614 < 2e-16 ***
diagn3LMCI -0.4342364 0.0229659 -18.908 < 2e-16 ***
diagn4AD -0.9011078 0.0273257 -32.977 < 2e-16 ***
APOE41 -0.0376401 0.0187520 -2.007 0.04472 *
APOE42 -0.0443933 0.0297857 -1.490 0.13611
edu.cat2tertiary -0.0706756 0.0215905 -3.273 0.00106 **
edu.cat3mid -0.1309874 0.0244110 -5.366 8.05e-08 ***
edu.cat4early -0.1272049 0.0257165 -4.946 7.56e-07 ***
aspirin 0.0283460 0.0166421 1.703 0.08852 .
GenderMale -0.0408614 0.0178665 -2.287 0.02219 *
parac 0.0394382 0.0197491 1.997 0.04583 *
diclo 0.0005850 0.0629124 0.009 0.99258
M:diagn2EMCI 0.0012201 0.0003822 3.193 0.00141 **
M:diagn3LMCI -0.0033271 0.0002813 -11.828 < 2e-16 ***
M:diagn4AD -0.0115027 0.0009300 -12.369 < 2e-16 ***
M:APOE41 -0.0032495 0.0002669 -12.175 < 2e-16 ***
M:APOE42 -0.0066054 0.0004940 -13.372 < 2e-16 ***
M:GenderMale 0.0011680 0.0002565 4.553 5.28e-06 ***
M:edu.cat2tertiary 0.0014060 0.0003066 4.585 4.54e-06 ***
M:edu.cat3mid 0.0004144 0.0003439 1.205 0.22826
M:edu.cat4early 0.0002567 0.0003767 0.682 0.49549
M:parac -0.0005003 0.0002521 -1.985 0.04718 *
M:diclo 0.0014406 0.0006561 2.196 0.02811 *
---
Signif. codes: 0 '***' 0.001 '**' 0.01 '*' 0.05 '.' 0.1 ' ' 1

Correlation matrix not shown by default, as p = 26 > 12.
Use print(x, correlation=TRUE) or
 vcov(x) if you need it

convergence code: 0
Model failed to converge with max|grad| = 1.68004 (tol = 0.001, component 1)
Model is nearly unidentifiable: very large eigenvalue
 - Rescale variables?
failure to converge in 10000 evaluations

glmerP1.mmse<-glmer(neg.b.MMSE~M+AGE+ diagn+ APOE4+edu.cat + aspirin+ diagn*M +APOE4*M + Gender*M+ edu.cat*M +parac*M +diclo*M+(1|ID), family="poisson", data=MMSEdata)
summary(glmerP1.mmse)

Generalized linear mixed model fit by maximum likelihood (Laplace
 Approximation) [glmerMod]
 Family: poisson ( log )
Formula:
neg.b.MMSE ~ M + AGE + diagn + APOE4 + edu.cat + aspirin + diagn *
 M + APOE4 * M + Gender * M + edu.cat * M + parac * M + diclo *
 M + (1 | ID)
 Data: MMSEdata

 AIC BIC logLik deviance df.resid
 33945.3 34136.8 -16945.6 33891.3 8851

Scaled residuals:
 Min 1Q Median 3Q Max
-2.4160 -0.7380 -0.1472 0.4982 6.8113

Random effects:
 Groups Name Variance Std.Dev.
 ID (Intercept) 0.4199 0.648
Number of obs: 8878, groups: ID, 1619

Fixed effects:
 Estimate Std. Error z value Pr(>|z|)
(Intercept) -0.5843395 0.0622136 -9.392 < 2e-16 ***
M 0.0104785 0.0008291 12.638 < 2e-16 ***
AGE 0.0182338 0.0025949 7.027 2.11e-12 ***
diagn2EMCI 0.7032050 0.0648633 10.841 < 2e-16 ***
diagn3LMCI 1.2347291 0.0548653 22.505 < 2e-16 ***
diagn4AD 2.1017871 0.0615261 34.161 < 2e-16 ***
APOE41 0.1202217 0.0422239 2.847 0.004410 **
APOE42 0.2309916 0.0647525 3.567 0.000361 ***
edu.cat2tertiary 0.2009658 0.0492473 4.081 4.49e-05 ***
edu.cat3mid 0.3597684 0.0553364 6.501 7.95e-11 ***
edu.cat4early 0.3497061 0.0572422 6.109 1.00e-09 ***
aspirin -0.0794993 0.0370667 -2.145 0.031972 *
GenderMale 0.0969278 0.0402826 2.406 0.016120 *
parac -0.0956706 0.0448314 -2.134 0.032842 *
diclo 0.0201426 0.1455151 0.138 0.889906
M:diagn2EMCI -0.0032630 0.0010011 -3.259 0.001116 **
M:diagn3LMCI 0.0029143 0.0006970 4.181 2.90e-05 ***
M:diagn4AD 0.0087605 0.0013891 6.306 2.86e-10 ***
M:APOE41 0.0058592 0.0005962 9.828 < 2e-16 ***
M:APOE42 0.0077079 0.0009010 8.555 < 2e-16 ***
M:GenderMale -0.0017639 0.0005714 -3.087 0.002021 **
M:edu.cat2tertiary -0.0038352 0.0007163 -5.354 8.58e-08 ***
M:edu.cat3mid -0.0023327 0.0007840 -2.975 0.002927 **
M:edu.cat4early -0.0025846 0.0007902 -3.271 0.001072 **
M:parac 0.0013325 0.0005692 2.341 0.019227 *
M:diclo -0.0046960 0.0015571 -3.016 0.002563 **
---
Signif. codes: 0 '***' 0.001 '**' 0.01 '*' 0.05 '.' 0.1 ' ' 1

Correlation matrix not shown by default, as p = 26 > 12.
Use print(x, correlation=TRUE) or
 vcov(x) if you need it

convergence code: 0
Model failed to converge with max|grad| = 1.81792 (tol = 0.001, component 1)
Model is nearly unidentifiable: very large eigenvalue
 - Rescale variables?
failure to converge in 10000 evaluations

glmerP2.mmse<-glmer(neg.b.MMSE~M+AGE+ diagn+ APOE4+edu.cat + aspirin+ diagn*M +APOE4*M + Gender*M+ edu.cat*M +parac*M +diclo*M+(1|ID), family=poisson(link=sqrt), data=MMSEdata)
summary(glmerP2.mmse)

Generalized linear mixed model fit by maximum likelihood (Laplace
 Approximation) [glmerMod]
 Family: poisson ( sqrt )
Formula:
neg.b.MMSE ~ M + AGE + diagn + APOE4 + edu.cat + aspirin + diagn *
 M + APOE4 * M + Gender * M + edu.cat * M + parac * M + diclo *
 M + (1 | ID)
 Data: MMSEdata

 AIC BIC logLik deviance df.resid
 34526.4 34717.9 -17236.2 34472.4 8851

Scaled residuals:
 Min 1Q Median 3Q Max
-2.5222 -0.7037 -0.1507 0.5398 8.1956

Random effects:
 Groups Name Variance Std.Dev.
 ID (Intercept) 0.2783 0.5275
Number of obs: 8878, groups: ID, 1619

Fixed effects:
 Estimate Std. Error z value Pr(>|z|)
(Intercept) 0.7561636 0.0449700 16.815 < 2e-16 ***
M 0.0036989 0.0005856 6.317 2.67e-10 ***
AGE 0.0120439 0.0020975 5.742 9.36e-09 ***
diagn2EMCI 0.3869089 0.0470569 8.222 < 2e-16 ***
diagn3LMCI 0.7774911 0.0402885 19.298 < 2e-16 ***
diagn4AD 1.6545143 0.0494000 33.492 < 2e-16 ***
APOE41 0.0808192 0.0334751 2.414 0.01577 *
APOE42 0.0975871 0.0538635 1.812 0.07003 .
edu.cat2tertiary 0.1024872 0.0383281 2.674 0.00750 **
edu.cat3mid 0.2076190 0.0434798 4.775 1.80e-06 ***
edu.cat4early 0.2122161 0.0462316 4.590 4.43e-06 ***
aspirin -0.0468205 0.0297108 -1.576 0.11505
GenderMale 0.0579500 0.0318460 1.820 0.06881 .
parac -0.0684758 0.0351082 -1.950 0.05113 .
diclo -0.0014112 0.1105359 -0.013 0.98981
M:diagn2EMCI -0.0017666 0.0007131 -2.477 0.01324 *
M:diagn3LMCI 0.0062790 0.0005259 11.939 < 2e-16 ***
M:diagn4AD 0.0202745 0.0019231 10.542 < 2e-16 ***
M:APOE41 0.0055304 0.0005048 10.956 < 2e-16 ***
M:APOE42 0.0116775 0.0009721 12.012 < 2e-16 ***
M:GenderMale -0.0013402 0.0004786 -2.801 0.00510 **
M:edu.cat2tertiary -0.0016890 0.0005594 -3.019 0.00254 **
M:edu.cat3mid 0.0004038 0.0006456 0.625 0.53170
M:edu.cat4early 0.0004154 0.0007329 0.567 0.57081
M:parac 0.0009383 0.0004712 1.991 0.04644 *
M:diclo -0.0024562 0.0011967 -2.052 0.04012 *
---
Signif. codes: 0 '***' 0.001 '**' 0.01 '*' 0.05 '.' 0.1 ' ' 1

Correlation matrix not shown by default, as p = 26 > 12.
Use print(x, correlation=TRUE) or
 vcov(x) if you need it

convergence code: 0
Model failed to converge with max|grad| = 0.00891603 (tol = 0.001, component 1)
Model is nearly unidentifiable: very large eigenvalue
 - Rescale variables?

glmerB4.mmse<-glmer(cbind(success,fail)~M.Z+AGE+ diagn+ APOE4+edu.cat + aspirin+ APOE4*M.Z+edu.cat*M.Z+diagn*M.Z+ parac*M.Z+diclo*M.Z+(1|ID), family="binomial", data=MMSEdata)
summary(glmerB4.mmse)

Generalized linear mixed model fit by maximum likelihood (Laplace
 Approximation) [glmerMod]
 Family: binomial ( logit )
Formula: cbind(success, fail) ~ M.Z + AGE + diagn + APOE4 + edu.cat +
 aspirin + APOE4 * M.Z + edu.cat * M.Z + diagn * M.Z + parac *
 M.Z + diclo * M.Z + (1 | ID)
 Data: MMSEdata

 AIC BIC logLik deviance df.resid
 34421.1 34598.4 -17185.6 34371.1 8853

Scaled residuals:
 Min 1Q Median 3Q Max
-6.8589 -0.5523 0.1643 0.7536 3.0817

Random effects:
 Groups Name Variance Std.Dev.
 ID (Intercept) 0.592 0.7694
Number of obs: 8878, groups: ID, 1619

Fixed effects:
 Estimate Std. Error z value Pr(>|z|)
(Intercept) 3.678902 0.061378 59.938 < 2e-16 ***
M.Z -0.285888 0.022422 -12.750 < 2e-16 ***
AGE -0.020546 0.003009 -6.828 8.61e-12 ***
diagn2EMCI -0.655686 0.067766 -9.676 < 2e-16 ***
diagn3LMCI -1.472870 0.058049 -25.373 < 2e-16 ***
diagn4AD -2.872303 0.073108 -39.288 < 2e-16 ***
APOE41 -0.331552 0.046513 -7.128 1.02e-12 ***
APOE42 -0.536065 0.073410 -7.302 2.83e-13 ***
edu.cat2tertiary -0.098018 0.053741 -1.824 0.068168 .
edu.cat3mid -0.324750 0.059668 -5.443 5.25e-08 ***
edu.cat4early -0.308649 0.063115 -4.890 1.01e-06 ***
aspirin 0.083212 0.042858 1.942 0.052190 .
parac 0.081212 0.048933 1.660 0.096981 .
diclo 0.132478 0.158614 0.835 0.403592
M.Z:APOE41 -0.223431 0.018720 -11.935 < 2e-16 ***
M.Z:APOE42 -0.338140 0.030070 -11.245 < 2e-16 ***
M.Z:edu.cat2tertiary 0.141475 0.022434 6.306 2.86e-10 ***
M.Z:edu.cat3mid 0.061577 0.023968 2.569 0.010196 *
M.Z:edu.cat4early 0.061278 0.024947 2.456 0.014036 *
M.Z:diagn2EMCI 0.113832 0.029764 3.825 0.000131 ***
M.Z:diagn3LMCI -0.150333 0.021082 -7.131 9.98e-13 ***
M.Z:diagn4AD -0.498080 0.047735 -10.434 < 2e-16 ***
M.Z:parac -0.039911 0.017884 -2.232 0.025640 *
M.Z:diclo 0.156443 0.049214 3.179 0.001479 **
---
Signif. codes: 0 '***' 0.001 '**' 0.01 '*' 0.05 '.' 0.1 ' ' 1

Correlation matrix not shown by default, as p = 24 > 12.
Use print(x, correlation=TRUE) or
 vcov(x) if you need it

convergence code: 0
Model failed to converge with max|grad| = 0.0758989 (tol = 0.001, component 1)
failure to converge in 10000 evaluations

glmerB5.mmse<-glmer(cbind(success,fail)~M.Z+AGE+ diagn+ APOE4+edu.cat + aspirin+ APOE4*M.Z+edu.cat*M.Z+diagn*M.Z+ parac*M.Z+diclo*M.Z+(1|ID), family=binomial(link=probit), data=MMSEdata)
summary(glmerB5.mmse)

Generalized linear mixed model fit by maximum likelihood (Laplace
 Approximation) [glmerMod]
 Family: binomial ( probit )
Formula: cbind(success, fail) ~ M.Z + AGE + diagn + APOE4 + edu.cat +
 aspirin + APOE4 * M.Z + edu.cat * M.Z + diagn * M.Z + parac *
 M.Z + diclo * M.Z + (1 | ID)
 Data: MMSEdata

 AIC BIC logLik deviance df.resid
 34643.0 34820.3 -17296.5 34593.0 8853

Scaled residuals:
 Min 1Q Median 3Q Max
-7.2759 -0.5664 0.1701 0.7453 3.0662

Random effects:
 Groups Name Variance Std.Dev.
 ID (Intercept) 0.1515 0.3892
Number of obs: 8878, groups: ID, 1619

Fixed effects:
 Estimate Std. Error z value Pr(>|z|)
(Intercept) 1.9688096 0.0006489 3034.08 <2e-16 ***
M.Z -0.1155742 0.0006346 -182.11 <2e-16 ***
AGE -0.0096567 0.0005968 -16.18 <2e-16 ***
diagn2EMCI -0.2912915 0.0006478 -449.63 <2e-16 ***
diagn3LMCI -0.7147598 0.0006476 -1103.79 <2e-16 ***
diagn4AD -1.5077939 0.0006471 -2330.04 <2e-16 ***
APOE41 -0.1687045 0.0006477 -260.48 <2e-16 ***
APOE42 -0.2831016 0.0006473 -437.35 <2e-16 ***
edu.cat2tertiary -0.0430555 0.0006477 -66.47 <2e-16 ***
edu.cat3mid -0.1569556 0.0006476 -242.38 <2e-16 ***
edu.cat4early -0.1537171 0.0006473 -237.47 <2e-16 ***
aspirin 0.0419122 0.0006694 62.61 <2e-16 ***
parac 0.0395140 0.0006476 61.01 <2e-16 ***
diclo 0.0694188 0.0006474 107.23 <2e-16 ***
M.Z:APOE41 -0.1196262 0.0006422 -186.29 <2e-16 ***
M.Z:APOE42 -0.2033737 0.0006463 -314.65 <2e-16 ***
M.Z:edu.cat2tertiary 0.0634387 0.0006653 95.35 <2e-16 ***
M.Z:edu.cat3mid 0.0171000 0.0006665 25.66 <2e-16 ***
M.Z:edu.cat4early 0.0174339 0.0006452 27.02 <2e-16 ***
M.Z:diagn2EMCI 0.0529094 0.0006454 81.97 <2e-16 ***
M.Z:diagn3LMCI -0.0991587 0.0006628 -149.59 <2e-16 ***
M.Z:diagn4AD -0.3283573 0.0006468 -507.68 <2e-16 ***
M.Z:parac -0.0206157 0.0006423 -32.10 <2e-16 ***
M.Z:diclo 0.0705545 0.0006468 109.08 <2e-16 ***
---
Signif. codes: 0 '***' 0.001 '**' 0.01 '*' 0.05 '.' 0.1 ' ' 1

Correlation matrix not shown by default, as p = 24 > 12.
Use print(x, correlation=TRUE) or
 vcov(x) if you need it

convergence code: 0
Model failed to converge with max|grad| = 0.119649 (tol = 0.001, component 1)
Model is nearly unidentifiable: very large eigenvalue
 - Rescale variables?

glmerB6.mmse<-glmer(cbind(success,fail)~M.Z+AGE+ diagn+ APOE4+edu.cat + aspirin+ APOE4*M.Z+edu.cat*M.Z+diagn*M.Z+ parac*M.Z+diclo*M.Z+(1|ID), family=binomial(link=cloglog), data=MMSEdata)
summary(glmerB6.mmse)

Generalized linear mixed model fit by maximum likelihood (Laplace
 Approximation) [glmerMod]
 Family: binomial ( cloglog )
Formula: cbind(success, fail) ~ M.Z + AGE + diagn + APOE4 + edu.cat +
 aspirin + APOE4 * M.Z + edu.cat * M.Z + diagn * M.Z + parac *
 M.Z + diclo * M.Z + (1 | ID)
 Data: MMSEdata

 AIC BIC logLik deviance df.resid
 35061.0 35238.2 -17505.5 35011.0 8853

Scaled residuals:
 Min 1Q Median 3Q Max
-7.6918 -0.5716 0.1754 0.7351 3.0497

Random effects:
 Groups Name Variance Std.Dev.
 ID (Intercept) 0.08935 0.2989
Number of obs: 8878, groups: ID, 1619

Fixed effects:
 Estimate Std. Error z value Pr(>|z|)
(Intercept) 1.319e+00 2.283e-02 57.771 < 2e-16 ***
M.Z -6.394e-02 7.793e-03 -8.204 2.32e-16 ***
AGE -6.932e-03 1.164e-03 -5.954 2.62e-09 ***
diagn2EMCI -1.983e-01 2.528e-02 -7.845 4.31e-15 ***
diagn3LMCI -5.258e-01 2.178e-02 -24.146 < 2e-16 ***
diagn4AD -1.220e+00 3.138e-02 -38.870 < 2e-16 ***
APOE41 -1.286e-01 1.800e-02 -7.141 9.25e-13 ***
APOE42 -2.280e-01 2.906e-02 -7.846 4.31e-15 ***
edu.cat2tertiary -3.183e-02 2.063e-02 -1.543 0.122899
edu.cat3mid -1.193e-01 2.301e-02 -5.185 2.16e-07 ***
edu.cat4early -1.187e-01 2.461e-02 -4.824 1.41e-06 ***
aspirin 2.663e-02 1.650e-02 1.614 0.106528
parac 2.720e-02 1.880e-02 1.447 0.148006
diclo 4.783e-02 5.993e-02 0.798 0.424866
M.Z:APOE41 -9.088e-02 7.443e-03 -12.211 < 2e-16 ***
M.Z:APOE42 -1.825e-01 1.378e-02 -13.246 < 2e-16 ***
M.Z:edu.cat2tertiary 3.677e-02 8.543e-03 4.304 1.67e-05 ***
M.Z:edu.cat3mid 2.292e-04 9.279e-03 0.025 0.980291
M.Z:edu.cat4early 2.401e-05 1.039e-02 0.002 0.998156
M.Z:diagn2EMCI 3.573e-02 1.065e-02 3.354 0.000798 ***
M.Z:diagn3LMCI -8.866e-02 7.788e-03 -11.384 < 2e-16 ***
M.Z:diagn4AD -3.213e-01 2.594e-02 -12.387 < 2e-16 ***
M.Z:parac -1.640e-02 7.011e-03 -2.339 0.019322 *
M.Z:diclo 4.251e-02 1.830e-02 2.322 0.020222 *
---
Signif. codes: 0 '***' 0.001 '**' 0.01 '*' 0.05 '.' 0.1 ' ' 1

Correlation matrix not shown by default, as p = 24 > 12.
Use print(x, correlation=TRUE) or
 vcov(x) if you need it

convergence code: 0
Model failed to converge with max|grad| = 0.105304 (tol = 0.001, component 1)
Model is nearly unidentifiable: very large eigenvalue
 - Rescale variables?

glmerP3.mmse<-glmer(neg.b.MMSE~M.Z+AGE+ diagn+ APOE4+edu.cat + aspirin+ APOE4*M.Z+edu.cat*M.Z+diagn*M.Z+ parac*M.Z+diclo*M.Z+(1|ID), family="poisson", data=MMSEdata)
summary(glmerP3.mmse)

Generalized linear mixed model fit by maximum likelihood (Laplace
 Approximation) [glmerMod]
 Family: poisson ( log )
Formula: neg.b.MMSE ~ M.Z + AGE + diagn + APOE4 + edu.cat + aspirin +
 APOE4 * M.Z + edu.cat * M.Z + diagn * M.Z + parac * M.Z +
 diclo * M.Z + (1 | ID)
 Data: MMSEdata

 AIC BIC logLik deviance df.resid
 33952.6 34129.9 -16951.3 33902.6 8853

Scaled residuals:
 Min 1Q Median 3Q Max
-2.3556 -0.7372 -0.1470 0.4992 6.5483

Random effects:
 Groups Name Variance Std.Dev.
 ID (Intercept) 0.4215 0.6492
Number of obs: 8878, groups: ID, 1619

Fixed effects:
 Estimate Std. Error z value Pr(>|z|)
(Intercept) -0.266011 0.053394 -4.982 6.29e-07 ***
M.Z 0.261652 0.020692 12.645 < 2e-16 ***
AGE 0.018565 0.002579 7.198 6.13e-13 ***
diagn2EMCI 0.602959 0.058926 10.233 < 2e-16 ***
diagn3LMCI 1.311641 0.050321 26.066 < 2e-16 ***
diagn4AD 2.341503 0.061768 37.908 < 2e-16 ***
APOE41 0.285208 0.039880 7.152 8.58e-13 ***
APOE42 0.442039 0.062491 7.074 1.51e-12 ***
edu.cat2tertiary 0.094624 0.046191 2.049 0.040507 *
edu.cat3mid 0.287348 0.051203 5.612 2.00e-08 ***
edu.cat4early 0.273695 0.053998 5.069 4.01e-07 ***
aspirin -0.069264 0.036780 -1.883 0.059673 .
parac -0.063644 0.041987 -1.516 0.129568
diclo -0.127397 0.136759 -0.932 0.351575
M.Z:APOE41 0.164303 0.016594 9.901 < 2e-16 ***
M.Z:APOE42 0.211186 0.025137 8.401 < 2e-16 ***
M.Z:edu.cat2tertiary -0.106002 0.019982 -5.305 1.13e-07 ***
M.Z:edu.cat3mid -0.049266 0.021291 -2.314 0.020670 *
M.Z:edu.cat4early -0.059933 0.021612 -2.773 0.005551 **
M.Z:diagn2EMCI -0.096435 0.027902 -3.456 0.000548 ***
M.Z:diagn3LMCI 0.077277 0.019365 3.991 6.59e-05 ***
M.Z:diagn4AD 0.243934 0.038728 6.299 3.00e-10 ***
M.Z:parac 0.036374 0.015872 2.292 0.021919 *
M.Z:diclo -0.134572 0.043383 -3.102 0.001923 **
---
Signif. codes: 0 '***' 0.001 '**' 0.01 '*' 0.05 '.' 0.1 ' ' 1

Correlation matrix not shown by default, as p = 24 > 12.
Use print(x, correlation=TRUE) or
 vcov(x) if you need it

convergence code: 0
Model failed to converge with max|grad| = 0.32314 (tol = 0.001, component 1)
failure to converge in 10000 evaluations

glmerP4.mmse<-glmer(neg.b.MMSE~M.Z+AGE+ diagn+ APOE4+edu.cat + aspirin+ APOE4*M.Z+edu.cat*M.Z+diagn*M.Z+ parac*M.Z+diclo*M.Z+(1|ID), family=poisson(link=sqrt), data=MMSEdata)
summary(glmerP4.mmse)

Generalized linear mixed model fit by maximum likelihood (Laplace
 Approximation) [glmerMod]
 Family: poisson ( sqrt )
Formula: neg.b.MMSE ~ M.Z + AGE + diagn + APOE4 + edu.cat + aspirin +
 APOE4 * M.Z + edu.cat * M.Z + diagn * M.Z + parac * M.Z +
 diclo * M.Z + (1 | ID)
 Data: MMSEdata

 AIC BIC logLik deviance df.resid
 34523.4 34700.6 -17236.7 34473.4 8853

Scaled residuals:
 Min 1Q Median 3Q Max
-2.5553 -0.7035 -0.1523 0.5356 7.9587

Random effects:
 Groups Name Variance Std.Dev.
 ID (Intercept) 0.2759 0.5252
Number of obs: 8878, groups: ID, 1619

Fixed effects:
 Estimate Std. Error z value Pr(>|z|)
(Intercept) 0.867786 0.039902 21.748 < 2e-16 ***
M.Z 0.077963 0.013609 5.729 1.01e-08 ***
AGE 0.012370 0.002075 5.963 2.48e-09 ***
diagn2EMCI 0.336801 0.044333 7.597 3.03e-14 ***
diagn3LMCI 0.948660 0.038292 24.775 < 2e-16 ***
diagn4AD 2.212926 0.058943 37.544 < 2e-16 ***
APOE41 0.238021 0.032153 7.403 1.33e-13 ***
APOE42 0.422553 0.052432 8.059 7.69e-16 ***
edu.cat2tertiary 0.058226 0.036649 1.589 0.11211
edu.cat3mid 0.215115 0.040941 5.254 1.49e-07 ***
edu.cat4early 0.219827 0.044133 4.981 6.33e-07 ***
aspirin -0.041452 0.029324 -1.414 0.15749
parac -0.041226 0.033369 -1.235 0.21666
diclo -0.076505 0.105054 -0.728 0.46647
M.Z:APOE41 0.155192 0.014108 11.001 < 2e-16 ***
M.Z:APOE42 0.324422 0.027105 11.969 < 2e-16 ***
M.Z:edu.cat2tertiary -0.043559 0.015558 -2.800 0.00511 **
M.Z:edu.cat3mid 0.024040 0.017363 1.385 0.16619
M.Z:edu.cat4early 0.020095 0.020158 0.997 0.31881
M.Z:diagn2EMCI -0.051406 0.019847 -2.590 0.00960 **
M.Z:diagn3LMCI 0.169363 0.014533 11.654 < 2e-16 ***
M.Z:diagn4AD 0.563872 0.053635 10.513 < 2e-16 ***
M.Z:parac 0.029491 0.013104 2.250 0.02442 *
M.Z:diclo -0.071808 0.033401 -2.150 0.03157 *
---
Signif. codes: 0 '***' 0.001 '**' 0.01 '*' 0.05 '.' 0.1 ' ' 1

Correlation matrix not shown by default, as p = 24 > 12.
Use print(x, correlation=TRUE) or
 vcov(x) if you need it

lme1.mmse<-lmer(neg.b.MMSE~M.Z+AGE+ diagn+ APOE4+edu.cat + aspirin+ APOE4*M.Z+edu.cat*M.Z+diagn*M.Z+ parac*M.Z+diclo*M.Z+(1|ID), data=MMSEdata)
summary(lme1.mmse)

Linear mixed model fit by REML. t-tests use Satterthwaite's method [
lmerModLmerTest]
Formula: neg.b.MMSE ~ M.Z + AGE + diagn + APOE4 + edu.cat + aspirin +
 APOE4 * M.Z + edu.cat * M.Z + diagn * M.Z + parac * M.Z +
 diclo * M.Z + (1 | ID)
 Data: MMSEdata

REML criterion at convergence: 41677.5

Scaled residuals:
 Min 1Q Median 3Q Max
-4.0635 -0.4687 -0.0956 0.3871 8.3206

Random effects:
 Groups Name Variance Std.Dev.
 ID (Intercept) 4.831 2.198
 Residual 4.564 2.136
Number of obs: 8878, groups: ID, 1619

Fixed effects:
 Estimate Std. Error df t value Pr(>|t|)
(Intercept) 8.132e-01 1.671e-01 1.579e+03 4.867 1.24e-06 ***
M.Z 1.721e-01 5.700e-02 7.863e+03 3.020 0.002537 **
AGE 2.809e-02 8.704e-03 1.634e+03 3.227 0.001275 **
diagn2EMCI 8.443e-01 1.855e-01 1.573e+03 4.552 5.73e-06 ***
diagn3LMCI 3.245e+00 1.602e-01 1.543e+03 20.255 < 2e-16 ***
diagn4AD 1.013e+01 2.481e-01 3.961e+03 40.835 < 2e-16 ***
APOE41 8.820e-01 1.346e-01 1.635e+03 6.551 7.64e-11 ***
APOE42 1.684e+00 2.199e-01 1.748e+03 7.659 3.08e-14 ***
edu.cat2tertiary 8.471e-02 1.534e-01 1.613e+03 0.552 0.580928
edu.cat3mid 6.493e-01 1.715e-01 1.610e+03 3.786 0.000159 ***
edu.cat4early 6.617e-01 1.850e-01 1.679e+03 3.577 0.000357 ***
aspirin -1.197e-01 1.229e-01 1.607e+03 -0.974 0.330301
parac -2.118e-01 1.398e-01 1.552e+03 -1.515 0.129893
diclo -2.320e-01 4.399e-01 1.491e+03 -0.527 0.598052
M.Z:APOE41 7.561e-01 5.979e-02 7.947e+03 12.646 < 2e-16 ***
M.Z:APOE42 1.784e+00 1.159e-01 7.977e+03 15.395 < 2e-16 ***
M.Z:edu.cat2tertiary -1.995e-01 6.615e-02 7.946e+03 -3.016 0.002570 **
M.Z:edu.cat3mid 3.442e-02 7.337e-02 7.945e+03 0.469 0.639049
M.Z:edu.cat4early 9.701e-02 8.497e-02 7.982e+03 1.142 0.253584
M.Z:diagn2EMCI -1.219e-01 8.306e-02 7.712e+03 -1.468 0.142125
M.Z:diagn3LMCI 1.002e+00 6.148e-02 7.851e+03 16.296 < 2e-16 ***
M.Z:diagn4AD 4.249e+00 2.273e-01 7.808e+03 18.691 < 2e-16 ***
M.Z:parac 1.539e-01 5.582e-02 7.882e+03 2.756 0.005859 **
M.Z:diclo -2.442e-01 1.400e-01 7.800e+03 -1.744 0.081137 .
---
Signif. codes: 0 '***' 0.001 '**' 0.01 '*' 0.05 '.' 0.1 ' ' 1

Correlation matrix not shown by default, as p = 24 > 12.
Use print(x, correlation=TRUE) or
 vcov(x) if you need it

mcp.fnc(lme1.mmse)

Figure description: Residual plots demonstrating a lack of heteroskedasticity and normality.

lme2.mmse<-lmer(neg.b.MMSE~M+AGE+ diagn+ APOE4+edu.cat + aspirin+ diagn*M +APOE4*M + Gender*M+ edu.cat*M +parac*M +diclo*M+(1|ID), data=MMSEdata)
summary(lme2.mmse)

Linear mixed model fit by REML. t-tests use Satterthwaite's method [
lmerModLmerTest]
Formula:
neg.b.MMSE ~ M + AGE + diagn + APOE4 + edu.cat + aspirin + diagn *
 M + APOE4 * M + Gender * M + edu.cat * M + parac * M + diclo *
 M + (1 | ID)
 Data: MMSEdata

REML criterion at convergence: 41735.9

Scaled residuals:
 Min 1Q Median 3Q Max
-4.0107 -0.4738 -0.0929 0.3890 8.2097

Random effects:
 Groups Name Variance Std.Dev.
 ID (Intercept) 4.830 2.198
 Residual 4.549 2.133
Number of obs: 8878, groups: ID, 1619

Fixed effects:
 Estimate Std. Error df t value Pr(>|t|)
(Intercept) 5.328e-01 1.880e-01 1.880e+03 2.833 0.004654 **
M 1.309e-02 2.430e-03 7.869e+03 5.387 7.39e-08 ***
AGE 2.829e-02 8.766e-03 1.629e+03 3.227 0.001274 **
diagn2EMCI 9.588e-01 1.966e-01 1.991e+03 4.876 1.17e-06 ***
diagn3LMCI 2.229e+00 1.686e-01 1.883e+03 13.219 < 2e-16 ***
diagn4AD 5.916e+00 2.072e-01 2.300e+03 28.553 < 2e-16 ***
APOE41 1.355e-01 1.402e-01 1.959e+03 0.967 0.333887
APOE42 -8.658e-02 2.260e-01 1.998e+03 -0.383 0.701712
edu.cat2tertiary 2.952e-01 1.603e-01 1.944e+03 1.841 0.065774 .
edu.cat3mid 6.681e-01 1.819e-01 1.950e+03 3.672 0.000247 ***
edu.cat4early 5.988e-01 1.936e-01 1.969e+03 3.093 0.002006 **
aspirin -1.066e-01 1.240e-01 1.608e+03 -0.860 0.389901
GenderMale 1.548e-01 1.332e-01 1.948e+03 1.162 0.245274
parac -3.509e-01 1.471e-01 1.890e+03 -2.386 0.017112 *
diclo -2.837e-03 4.629e-01 1.833e+03 -0.006 0.995112
M:diagn2EMCI -3.832e-03 2.975e-03 7.710e+03 -1.288 0.197734
M:diagn3LMCI 3.747e-02 2.220e-03 7.848e+03 16.876 < 2e-16 ***
M:diagn4AD 1.527e-01 8.138e-03 7.805e+03 18.767 < 2e-16 ***
M:APOE41 2.702e-02 2.140e-03 7.944e+03 12.625 < 2e-16 ***
M:APOE42 6.444e-02 4.149e-03 7.974e+03 15.530 < 2e-16 ***
M:GenderMale -1.055e-02 2.013e-03 7.939e+03 -5.241 1.64e-07 ***
M:edu.cat2tertiary -8.055e-03 2.374e-03 7.944e+03 -3.393 0.000695 ***
M:edu.cat3mid -2.297e-03 2.711e-03 7.935e+03 -0.847 0.396877
M:edu.cat4early 1.247e-03 3.071e-03 7.976e+03 0.406 0.684792
M:parac 4.706e-03 2.004e-03 7.875e+03 2.348 0.018885 *
M:diclo -7.867e-03 5.013e-03 7.797e+03 -1.569 0.116642
---
Signif. codes: 0 '***' 0.001 '**' 0.01 '*' 0.05 '.' 0.1 ' ' 1

Correlation matrix not shown by default, as p = 26 > 12.
Use print(x, correlation=TRUE) or
 vcov(x) if you need it

mcp.fnc(lme2.mmse)

Figure description: Residual plots demonstrating a lack of heteroskedasticity and normality.

neg.binom.zero.mmse<-glmmadmb(neg.b.MMSE~M+AGE+ diagn+ APOE4+edu.cat + aspirin+ diagn*M +APOE4*M + Gender*M+ edu.cat*M +parac*M +diclo*M+(1|ID), family="nbinom1", data=MMSEdata, zeroInflation=TRUE)
anova(neg.binom.zero.mmse, neg.mmse)

Analysis of Deviance Table

Model 1: neg.b.MMSE ~ M + AGE + diagn + APOE4 + edu.cat + aspirin + diagn * M + APOE4 * M + Gender * M + edu.cat * M + parac * M + diclo * M
Model 2: neg.b.MMSE ~ M + AGE + diagn + APOE4 + edu.cat + aspirin + diagn * M + APOE4 * M + Gender * M + edu.cat * M + parac * M + diclo * M
 NoPar LogLik Df Deviance Pr(>Chi)
1 28 -16940
2 29 -16938 1 4 0.0455 *
---
Signif. codes: 0 '***' 0.001 '**' 0.01 '*' 0.05 '.' 0.1 ' ' 1

neg.binom.link.parameterisation.mmse<-glmmadmb(neg.b.MMSE~M+AGE+ diagn+ APOE4+edu.cat + aspirin+ diagn*M +APOE4*M + Gender*M+ edu.cat*M +parac*M +diclo*M+(1|ID), family="nbinom", data=MMSEdata)


AIC.MMSE<-c(
AIC(logLik(neg.mmse)),
AIC(logLik(glmerB1.mmse)),
AIC(logLik(glmerB2.mmse)),
AIC(logLik(glmerB3.mmse)),
AIC(logLik(glmerB4.mmse)),
AIC(logLik(glmerB5.mmse)),
AIC(logLik(glmerB6.mmse)),
AIC(logLik(glmerP1.mmse)),
AIC(logLik(glmerP2.mmse)),
AIC(logLik(glmerP3.mmse)),
AIC(logLik(glmerP4.mmse)),
AIC(logLik(lme1.mmse)),
AIC(logLik(lme2.mmse)),
AIC(logLik(neg.binom.zero.mmse)),
AIC(logLik(neg.binom.link.parameterisation.mmse)))

Model<-c("Negative Binomial", "Binomial (logit)", "Binomial (probit)","Binomial (Cloglog)","Binomial (logit, centered)", "Binomial (probit, centered)","Binomial (Cloglog, centered)","Poisson (log)","Poisson (sqrt)","Poisson (log, centered)","Poisson (sqrt, centered)","MLM (centered)","MLM","Negative binomial (zero inflated)","Negative binomial (classical parameterisation)")

data.frame(Model,AIC.MMSE)

Model AIC.MMSE
1 Negative Binomial 33936.00
2 Binomial (logit) 34398.91
3 Binomial (probit) 34621.30
4 Binomial (Cloglog) 35043.63
5 Binomial (logit, centered) 34421.14
6 Binomial (probit, centered) 34642.97
7 Binomial (Cloglog, centered) 35060.95
8 Poisson (log) 33945.30
9 Poisson (sqrt) 34526.45
10 Poisson (log, centered) 33952.64
11 Poisson (sqrt, centered) 34523.36
12 MLM (centered) 41729.48
13 MLM 41791.95
14 Negative binomial (zero inflated) 33934.00
15 Negative binomial (classical parameterisation) 33947.80

######

# Analysis of cognitive decline using the ADAS score

Alzheimer’s Disease Assessment Scale-Cog (ADAS) is a cognitive assessment with a focus on memory that is often used as the primary measure of Alzheimer’s disease progression in clinical trials. It is a score out of 90 with higher scores corresponding to worse cognitive performance.

## Dependent variable check

Those with no ADAS score were removed and variables were checked for correct categorization.

data<-read.csv("CleanedFinalData.csv", header=T)
data$ADAS<-data$ADAS13
ADASdata<-data[!is.na(data$ADAS),]
ADASdata$ID<-as.factor(ADASdata$ID)
ADASdata$APOE4<-as.factor(ADASdata$APOE4)

## Generation of dependent variables appropriate for different distributions

ADASdata$neg.b.ADAS<-round(3*ADASdata$ADAS13)
ADASdata$fail<-ADASdata$neg.b.ADAS
ADASdata$success<-270-ADASdata$fail
ADASdata$ADASscore<-rep(270,length(ADASdata$neg.b.ADAS))
ADASdata$proportion<-ADASdata$fail/ADASdata$ADASscore

## Transformation to obtain normal approximation

The total errors were then generated by constructing a linear model of with ADAS as the dependent variable and patient ID as the explanatory variable. It has been shown that if the total errors are homoscedastic and normal then the multi-level linear model errors will likely also be homoscedastic and normal distributed (Gurka *et al.* 2006). Therefore, total errors are an excellent starting point for model diagnostics. From this it was found that even with BoxCox optimized transformation the residuals while normally distributed fail to have even homoscedasticity. This is due to the categorical nature of the ADAS score and the high number of zero values. Therefore, a generalized linear model utilizing a non-gaussian distribution were performed.

qqp(ADASdata$ADAS[ADASdata$M==0], dist="norm")

Figure description: ADAS score plotted against quantiles with a normal distribution plotted in blue. At month 0.

[1] 1603 1154

m1<-lm((ADAS+1)~ID, data=ADASdata)
par(mfrow=c(2,2))
plot(m1)

Figure description: Residual distribution plots for evaluating the assumptions of the least-square methods.

boxcox<-boxcox(m1,lambda = seq(-5, 5, 1/100),plotit = TRUE )
selectedlambda<-boxcox$x[boxcox$y==max(boxcox$y)]
selectedlambda

[1] 0.26

ADASdata$tADAS<-(ADASdata$ADAS)^selectedlambda
m1.t<-lmer(tADAS~diagn+edu.cat+Gender+APOE4+M+(1|ID),data=ADASdata)
summary(m1.t)

Linear mixed model fit by REML. t-tests use Satterthwaite's method [
lmerModLmerTest]
Formula: tADAS ~ diagn + edu.cat + Gender + APOE4 + M + (1 | ID)
 Data: ADASdata

REML criterion at convergence: -3572.7

Scaled residuals:
 Min 1Q Median 3Q Max
-7.9140 -0.4878 0.0033 0.5021 7.3893

Random effects:
 Groups Name Variance Std.Dev.
 ID (Intercept) 0.05238 0.2289
 Residual 0.02475 0.1573
Number of obs: 8770, groups: ID, 1618

Fixed effects:
 Estimate Std. Error df t value Pr(>|t|)
(Intercept) 1.601e+00 1.632e-02 1.643e+03 98.082 < 2e-16 ***
diagn2EMCI 1.368e-01 1.824e-02 1.593e+03 7.496 1.08e-13 ***
diagn3LMCI 4.034e-01 1.602e-02 1.585e+03 25.182 < 2e-16 ***
diagn4AD 7.078e-01 1.894e-02 1.711e+03 37.364 < 2e-16 ***
edu.cat2tertiary 3.264e-02 1.521e-02 1.618e+03 2.146 0.032 *
edu.cat3mid 6.943e-02 1.723e-02 1.620e+03 4.029 5.85e-05 ***
edu.cat4early 9.608e-02 1.825e-02 1.642e+03 5.264 1.59e-07 ***
GenderMale 4.996e-02 1.240e-02 1.628e+03 4.030 5.84e-05 ***
APOE41 7.855e-02 1.326e-02 1.627e+03 5.925 3.80e-09 ***
APOE42 9.955e-02 2.107e-02 1.642e+03 4.726 2.49e-06 ***
M 2.837e-03 7.139e-05 7.484e+03 39.740 < 2e-16 ***
---
Signif. codes: 0 '***' 0.001 '**' 0.01 '*' 0.05 '.' 0.1 ' ' 1

Correlation of Fixed Effects:
 (Intr) d2EMCI d3LMCI dgn4AD ed.ct2 ed.ct3 ed.ct4 GndrMl APOE41
diagn2EMCI -0.421
diagn3LMCI -0.453 0.501
diagn4AD -0.351 0.436 0.531
ed.ct2trtry -0.418 0.011 -0.009 -0.043
edu.cat3mid -0.438 -0.027 0.024 -0.037 0.405
edu.cat4rly -0.348 -0.037 -0.069 -0.129 0.388 0.358
GenderMale -0.453 -0.044 -0.083 -0.048 0.050 0.174 0.142
APOE41 -0.192 -0.094 -0.178 -0.209 -0.030 -0.032 -0.047 0.002
APOE42 -0.072 -0.075 -0.177 -0.236 -0.045 -0.028 -0.019 -0.010 0.307
M -0.118 0.024 0.027 0.070 0.000 -0.002 0.003 -0.002 0.005
 APOE42
diagn2EMCI
diagn3LMCI
diagn4AD
ed.ct2trtry
edu.cat3mid
edu.cat4rly
GenderMale
APOE41
APOE42
M 0.005

mcp.fnc(m1.t)

Figure description: Box Cox plot of log-likelihood verse lambda showing the maximum is achieved at 0.26

Figure description: Residual distribution plots for evaluating the assumptions of the least-square methods. Normality close to being achieved, however, heteroskedasticity is still an issue due the discrete nature of the data.

qqp(ADASdata$tADAS[ADASdata$M==0], dist="norm")

Figure description: Plot of ADAS scores against quantile with expected normal distribution overlay (blue) . At month 0.

[1] 951 107

## Selecting non-gaussian model

The data was plotted against expected distributions including normal, Poisson, negative binomial, binomial, exponential or gamma distributions within each time point. Some transformations were required including converting the ADAS score to whole numbers (with a round (3xADAS) function) for the Poisson, negative binomial and exponential distributions. This is because 1/3 marks are received in the ADAS task. For the Poisson distribution there was over dispersion as seen by the vast difference in mean and variance, supporting the use of negative binomial models. This is also evident in the dependent variable not following a Poisson distribution. The binomial model seems appropriate theoretically as the ADAS score could be considered 270 trials with a proportion of failures occurring. However, the large number of zeros made logistic regression of the binomial distribution inappropriate. Furthermore, the exponential model was a poor fit. The negative binomial and gamma models appear to be the most appropriate models. However, given the categorical nature of the data the negative binomial model was chosen for analysis. This is an approximate method for distribution selection as it does not take into account the explanatory variables effects on the distribution. This approach was used as a starting point for model analysis. Distributions were compared once the final model was established and the negative binomial model proved to be the most appropriate. Furthermore, residual analysis of the final selected model confirmed the appropriateness of the negative binomial model.

hist(ADASdata$ADAS[ADASdata$M==0])

Figure description: Plot of the frequency of ADAS scores at month 0.

hist(ADASdata$ADAS[ADASdata$M==12])

Figure description: Plot of the frequency of ADAS scores at month 12.

hist(ADASdata$ADAS[ADASdata$M==24])

Figure description: Plot of the frequency of ADAS scores at month 24

hist(ADASdata$ADAS[ADASdata$M==48])

Figure description: Plot of the frequency of ADAS scores at month 48.

hist(ADASdata$ADAS[ADASdata$M==72])

Figure description: Plot of the frequency of ADAS scores at month 72

hist(ADASdata$ADAS[ADASdata$M==120])

Figure description: Plot of the frequency of ADAS scores at month 120

qqp(ADASdata$ADAS[ADASdata$M==0], "norm", main="Normal distribution model Month=0")

Figure description: Plot of ADAS scores against quantile with expected normal distribution overlay (blue) , month 0.

[1] 1603 1154

qqp(ADASdata$ADAS[ADASdata$M==12], "norm", main="Normal distribution model Month=12")

Figure description: Plot of ADAS scores against quantile with expected normal distribution overlay (blue) , month 12.

[1] 517 576

qqp(ADASdata$ADAS[ADASdata$M==24], "norm", main="Normal distribution model Month=24")

Figure description: Plot of ADAS scores against quantile with expected normal distribution overlay (blue) , month 24

[1] 546 159

qqp(ADASdata$ADAS[ADASdata$M==48], "norm", main="Normal distribution model Month=48")

Figure description: Plot of ADAS scores against quantile with expected normal distribution overlay (blue) , month 48.

[1] 220 453

qqp(ADASdata$ADAS[ADASdata$M==72], "norm", main="Normal distribution model Month=72")

Figure description: Plot of ADAS scores against quantile with expected normal distribution overlay (blue) , month 72.

[1] 120 106

qqp(ADASdata$ADAS[ADASdata$M==120], "norm", main="Normal distribution model Month=120")

Figure description: Plot of ADAS scores against quantile with expected normal distribution overlay (blue) , month 120.

Because ADAS was a score out of 90 that allowed 1/3 marks, and because the negative binomial models require whole values, the score was multiplied by 3 and modelled as a negative binomial.

[1] 41 35

hist(ADASdata$neg.b.ADAS[ADASdata$M==0])

Figure description: Histogram of ADAS (x3) frequencies demonstrating a negative binomial distribution at month 0.

hist(ADASdata$neg.b.ADAS[ADASdata$M==12])

Figure description: Histogram of ADAS (x3) frequencies demonstrating a negative binomial distribution at month 12.

hist(ADASdata$neg.b.ADAS[ADASdata$M==24])

Figure description: Histogram of ADAS (x3) frequencies demonstrating a negative binomial distribution at month 24.

hist(ADASdata$neg.b.ADAS[ADASdata$M==48])

Figure description: Histogram of ADAS (x3) frequencies demonstrating a negative binomial distribution at month 48.

hist(ADASdata$neg.b.ADAS[ADASdata$M==72])

Figure description: Histogram of ADAS (x3) frequencies demonstrating a negative binomial distribution at month 72.

hist(ADASdata$neg.b.ADAS[ADASdata$M==120])

Figure description: Histogram of ADAS (x3) frequencies demonstrating a negative binomial distribution at month 120.

nbinom<-fitdistr(ADASdata$neg.b.ADAS[ADASdata$M==0], "negative binomial")
qqp(ADASdata$neg.b.ADAS[ADASdata$M==0], "nbinom", size=nbinom$estimate[[1]], mu=nbinom$estimate[[2]], main="Negative binomial model Month=0")

Figure description: Plot of ADAS (x3) scores against negative binomial quantile with expected negative binomial distribution overlay (blue) , month 0.

[1] 1603 1154

nbinom<-fitdistr(ADASdata$neg.b.ADAS[ADASdata$M==12], "negative binomial")
qqp(ADASdata$neg.b.ADAS[ADASdata$M==12], "nbinom", size=nbinom$estimate[[1]], mu=nbinom$estimate[[2]], main="Negative binomial model Month=12")

Figure description: Plot of ADAS (x3) scores against negative binomial quantile with expected negative binomial distribution overlay (blue) , month 12.

[1] 517 576

nbinom<-fitdistr(ADASdata$neg.b.ADAS[ADASdata$M==24], "negative binomial")
qqp(ADASdata$neg.b.ADAS[ADASdata$M==24], "nbinom", size=nbinom$estimate[[1]], mu=nbinom$estimate[[2]], main="Negative binomial model Month=24")

Figure description: Plot of ADAS (x3) scores against negative binomial quantile with expected negative binomial distribution overlay (blue) , month 24.

[1] 546 159

nbinom<-fitdistr(ADASdata$neg.b.ADAS[ADASdata$M==48], "negative binomial")
qqp(ADASdata$neg.b.ADAS[ADASdata$M==48], "nbinom", size=nbinom$estimate[[1]], mu=nbinom$estimate[[2]], main="Negative binomial model Month=48")

Figure description: Plot of ADAS (x3) scores against negative binomial quantile with expected negative binomial distribution overlay (blue) , month 48.

[1] 220 453

nbinom<-fitdistr(ADASdata$neg.b.ADAS[ADASdata$M==72], "negative binomial")
qqp(ADASdata$neg.b.ADAS[ADASdata$M==72], "nbinom", size=nbinom$estimate[[1]], mu=nbinom$estimate[[2]], main="Negative binomial model Month=72")

Figure description: Plot of ADAS (x3) scores against negative binomial quantile with expected negative binomial distribution overlay (blue) , month 72.

[1] 120 106

nbinom<-fitdistr(ADASdata$neg.b.ADAS[ADASdata$M==120], "negative binomial")
qqp(ADASdata$neg.b.ADAS[ADASdata$M==120], "nbinom", size=nbinom$estimate[[1]], mu=nbinom$estimate[[2]], main="Negative binomial model Month=120")

Figure description: Plot of ADAS (x3) scores against negative binomial quantile with expected negative binomial distribution overlay (blue) , month 120.

[1] 41 35

poisson <- fitdistr(ADASdata$neg.b.ADAS[ADASdata$M==0]+1, "Poisson")
qqp(ADASdata$neg.b.ADAS, "pois", lambda=poisson$estimate, main="Poisson model Month=0")

Figure description: Plot of ADAS (x3) scores against Poisson quantile with expected Poisson distribution overlay (blue) , month 0.

[1] 6306 7138

poisson <- fitdistr(ADASdata$neg.b.ADAS[ADASdata$M==12], "Poisson")
qqp(ADASdata$neg.b.ADAS, "pois", lambda=poisson$estimate, main="Poisson model Month=12")

Figure description: Plot of ADAS (x3) scores against Poisson quantile with expected Poisson distribution overlay (blue) , month 12.

[1] 6306 7138

poisson <- fitdistr(ADASdata$neg.b.ADAS[ADASdata$M==24], "Poisson")
qqp(ADASdata$neg.b.ADAS, "pois", lambda=poisson$estimate, main="Poisson model Month=24")

Figure description: Plot of ADAS (x3) scores against Poisson quantile with expected Poisson distribution overlay (blue) , month 24.

[1] 6306 7138

poisson <- fitdistr(ADASdata$neg.b.ADAS[ADASdata$M==48], "Poisson")
qqp(ADASdata$neg.b.ADAS, "pois", lambda=poisson$estimate, main="Poisson model Month=48")

Figure description: Plot of ADAS (x3) scores against Poisson quantile with expected Poisson distribution overlay (blue) , month 48.

[1] 6306 7138

poisson <- fitdistr(ADASdata$neg.b.ADAS[ADASdata$M==72], "Poisson")
qqp(ADASdata$neg.b.ADAS, "pois", lambda=poisson$estimate, main="Poisson model Month=72")

Figure description: Plot of ADAS (x3) scores against Poisson quantile with expected Poisson distribution overlay (blue) , month 72.

[1] 6306 7138

poisson <- fitdistr(ADASdata$neg.b.ADAS[ADASdata$M==120], "Poisson")
qqp(ADASdata$neg.b.ADAS, "pois", lambda=poisson$estimate, main="Poisson model Month=120")

Figure description: Plot of ADAS (x3) scores against Poisson quantile with expected Poisson distribution overlay (blue) , month 120.

[1] 6306 7138

mean(ADASdata$neg.b.ADAS[ADASdata$M==0])

[1] 52.23211

var(ADASdata$neg.b.ADAS[ADASdata$M==0])

[1] 828.3178

mean(ADASdata$neg.b.ADAS[ADASdata$M==12])

[1] 53.92857

var(ADASdata$neg.b.ADAS[ADASdata$M==12])

[1] 1282.4

mean(ADASdata$neg.b.ADAS[ADASdata$M==24])

[1] 54.69595

var(ADASdata$neg.b.ADAS[ADASdata$M==24])

[1] 1483.316

mean(ADASdata$neg.b.ADAS[ADASdata$M==48])

[1] 49.02997

var(ADASdata$neg.b.ADAS[ADASdata$M==48])

[1] 1410.121

mean(ADASdata$neg.b.ADAS[ADASdata$M==72])

[1] 55.54958

var(ADASdata$neg.b.ADAS[ADASdata$M==72])

[1] 1629.714

mean(ADASdata$neg.b.ADAS[ADASdata$M==120])

[1] 55.09589

var(ADASdata$neg.b.ADAS[ADASdata$M==120])

[1] 1342.56

gamma <- fitdistr((ADASdata$neg.b.ADAS[ADASdata$M==0]+1), "gamma")
qqp(ADASdata$neg.b.ADAS[ADASdata$M==0]+1, "gamma", shape = gamma$estimate[[1]], rate = gamma$estimate[[2]], main="Gamma model Month=0")

Figure description: Plot of ADAS (x3) scores against gamma quantile with expected gamma distribution overlay (blue) , month 0.

[1] 1603 1154

gamma <- fitdistr((ADASdata$neg.b.ADAS[ADASdata$M==12]+1), "gamma")
qqp(ADASdata$neg.b.ADAS[ADASdata$M==12]+1, "gamma", shape = gamma$estimate[[1]], rate = gamma$estimate[[2]], main="Gamma model Month=12")

Figure description: Plot of ADAS (x3) scores against gamma quantile with expected gamma distribution overlay (blue) , month 12.

[1] 517 576

gamma <- fitdistr((ADASdata$neg.b.ADAS[ADASdata$M==24]+1), "gamma")
qqp(ADASdata$neg.b.ADAS[ADASdata$M==24]+1, "gamma", shape = gamma$estimate[[1]], rate = gamma$estimate[[2]], main="Gamma model Month=24")

Figure description: Plot of ADAS (x3) scores against gamma quantile with expected gamma distribution overlay (blue) , month 24.

[1] 546 159

gamma <- fitdistr((ADASdata$neg.b.ADAS[ADASdata$M==48]+1), "gamma")
qqp(ADASdata$neg.b.ADAS[ADASdata$M==48]+1, "gamma", shape = gamma$estimate[[1]], rate = gamma$estimate[[2]], main="Gamma model Month=48")

Figure description: Plot of ADAS (x3) scores against gamma quantile with expected gamma distribution overlay (blue) , month 48.

[1] 220 453

gamma <- fitdistr((ADASdata$neg.b.ADAS[ADASdata$M==72]+1), "gamma")
qqp(ADASdata$neg.b.ADAS[ADASdata$M==72]+1, "gamma", shape = gamma$estimate[[1]], rate = gamma$estimate[[2]], main="Gamma model Month=72")

Figure description: Plot of ADAS (x3) scores against gamma quantile with expected gamma distribution overlay (blue) , month 72.

[1] 120 106

gamma <- fitdistr((ADASdata$neg.b.ADAS[ADASdata$M==120]+1), "gamma")
qqp(ADASdata$neg.b.ADAS[ADASdata$M==120]+1, "gamma", shape = gamma$estimate[[1]], rate = gamma$estimate[[2]], main="Gamma model Month=120")

Figure description: Plot of ADAS (x3) scores against gamma quantile with expected gamma distribution overlay (blue) , month 120.

[1] 41 35

par(mar = c(3,3,3,3))
hist(ADASdata$proportion)

Figure description: Frequency plot of ADAS scores expressed as a proportion.

data<-ADASdata$proportion[ADASdata$M==0]
params<-fitdistr(data, "logistic")
qqp(data, dist="logis", params$estimate[[1]]);title(main="Logistic model Month=0", line=2)

Figure description: Plot of ADAS scores as a proportion against logistic quantiles with expected logistic distribution overlay (blue) , month 0.

data<-ADASdata$proportion[ADASdata$M==12]
params<-fitdistr(data, "logistic")
qqp(data, dist="logis", params$estimate[[1]]);title(main="Logistic model Month=12", line=2)

Figure description: Plot of ADAS scores as a proportion against logistic quantiles with expected logistic distribution overlay (blue) , month 12.

data<-ADASdata$proportion[ADASdata$M==24]
params<-fitdistr(data, "logistic")
qqp(data, dist="logis", params$estimate[[1]]);title(main="Logistic model Month=24", line=2)

Figure description: Plot of ADAS scores as a proportion against logistic quantiles with expected logistic distribution overlay (blue) , month 24.

data<-ADASdata$proportion[ADASdata$M==48]
params<-fitdistr(data, "logistic")
qqp(data, dist="logis", params$estimate[[1]]);title(main="Logistic model Month=48", line=2)

Figure description: Plot of ADAS scores as a proportion against logistic quantiles with expected logistic distribution overlay (blue) , month 48.

data<-ADASdata$proportion[ADASdata$M==72]
params<-fitdistr(data, "logistic")
qqp(data, dist="logis", params$estimate[[1]]);title(main="Logistic model Month=72", line=2)

Figure description: Plot of ADAS scores as a proportion against logistic quantiles with expected logistic distribution overlay (blue) , month 72.

data<-ADASdata$proportion[ADASdata$M==120]
params<-fitdistr(data, "logistic")
qqp(data, dist="logis",params$estimate[[1]]);title(main="Logistic model Month=120", line=2)

Figure description: Plot of ADAS scores as a proportion against logistic quantiles with expected logistic distribution overlay (blue) , month 120.

data<-ADASdata$neg.b.ADAS[ADASdata$M==0]
params<-fitdistr(data, "exponential")
qqp(data, "exp",rate = params$estimate, main="Exponential model Month=0")

Figure description: Plot of ADAS scores against exponential quantiles with expected exponential distribution overlay (blue) , month 0.

[1] 1603 1154

data<-ADASdata$neg.b.ADAS[ADASdata$M==12]
params<-fitdistr(data, "exponential")
qqp(data, "exp",rate = params$estimate, main="Exponential model Month=12")

Figure description: Plot of ADAS scores against exponential quantiles with expected exponential distribution overlay (blue) , month 12.

[1] 517 576

data<-ADASdata$neg.b.ADAS[ADASdata$M==24]
params<-fitdistr(data, "exponential")
qqp(data, "exp",rate = params$estimate, main="Exponential model Month=24")

Figure description: Plot of ADAS scores against exponential quantiles with expected exponential distribution overlay (blue) , month 24.

[1] 546 159

data<-ADASdata$neg.b.ADAS[ADASdata$M==48]
params<-fitdistr(data, "exponential")
qqp(data, "exp",rate = params$estimate, main="Exponential model Month=48")

Figure description: Plot of ADAS scores against exponential quantiles with expected exponential distribution overlay (blue) , month 48.

[1] 220 453

data<-ADASdata$neg.b.ADAS[ADASdata$M==72]
params<-fitdistr(data, "exponential")
qqp(data, "exp",rate = params$estimate, main="Exponential model Month=72")

Figure description: Plot of ADAS scores against exponential quantiles with expected exponential distribution overlay (blue) , month 72.

[1] 120 106

data<-ADASdata$neg.b.ADAS[ADASdata$M==120]
params<-fitdistr(data, "exponential")
qqp(data, "exp",rate = params$estimate, main="Exponential model Month=120")

Figure description: Plot of ADAS scores against exponential quantiles with expected exponential distribution overlay (blue) , month 120.

[1] 41 35

## Build base negative binomial model and comparing the different parameterization methods.

There are two commonly used parameterization methods used in the negative binomial model. To evaluate which to continue with all biological relevant main effect explanatory variables were include in the models and initial model diagnostic were performed. Using the AIC and log-likelihood statistics we can see that the Nbinom1 parameterization method is preferred. This uses a variance proportional to the mean method of parameterization.

neg.a.binom1 <- glmmadmb(neg.b.ADAS~AGE + APOE4 + M + Gender + edu.cat + diagn + vasc+diab+aspirin+parac+diclo+naprox+celex+Ibu+(1|ID), family="nbinom", data=ADASdata)

neg.a.binom2 <- glmmadmb(neg.b.ADAS~AGE + APOE4 + M + Gender + edu.cat + diagn + vasc+diab+aspirin+parac+diclo+naprox+celex+Ibu+ (1|ID), family="nbinom1", data=ADASdata)

AIC(logLik(neg.a.binom1))

[1] 73176.2

AIC(logLik(neg.a.binom2))

[1] 72520.6

logLik(neg.a.binom1)

'log Lik.' -36566.1 (df=22)

logLik(neg.a.binom2)

'log Lik.' -36238.3 (df=22)

## Observing the distribution of the residuals in for the initial model.

Residuals within each explanatory variable collectively and within each individual show no trends and are homoscedastic centred around zero. Therefore, this model is accurate and will be used to investigate the effects of the input variables (including pain medications) going forward.

augDat <- data.frame(ADASdata,resid=residuals(neg.a.binom2,type="pearson"),
fitted=fitted(neg.a.binom2))
ggplot(augDat,aes(x=Gender,y=resid,group=ID))+geom_boxplot()+coord_flip()

Figure description: Box Plots of the Pearson residuals per participant ID showing centred clustering around zero. Grouped by gender.

ggplot(augDat,aes(x=AGE,y=resid,group=ID))+geom_boxplot()+coord_flip()

Figure description: Box Plots of the Pearson residuals per participant ID showing centred clustering around zero. Plotted against Age.

ggplot(augDat,aes(x=edu.cat,y=resid,group=ID))+geom_boxplot()+coord_flip()

Figure description: Box Plots of the Pearson residuals per participant ID showing centred clustering around zero. Plotted against Education level.

ggplot(augDat,aes(x=diagn,y=resid,group=ID))+geom_boxplot()+coord_flip()

Figure description: Box Plots of the Pearson residuals per participant ID showing centred clustering around zero. Plotted against cognitive diagnosis.

ggplot(augDat,aes(x=APOE4,y=resid,group=ID))+geom_boxplot()+coord_flip()

Figure description: Box Plots of the Pearson residuals per participant ID showing centred clustering around zero. Plotted against ApoE4 status.

ggplot(augDat,aes(x=M,y=resid,group=ID))+geom_boxplot()+coord_flip()

Figure description: Box Plots of the Pearson residuals per participant ID showing centred clustering around zero. Plotted against time (months).

ggplot(augDat,aes(x=diclo,y=resid,group=ID))+geom_boxplot()+coord_flip()

Figure description: Box Plots of the Pearson residuals per participant ID showing centred clustering around zero. Plotted by diclofenac use.

ggplot(augDat,aes(x=vasc,y=resid,group=ID))+geom_boxplot()+coord_flip()

Figure description: Box Plots of the Pearson residuals per participant ID showing centred clustering around zero. Plotted by cardiovascular status.

ggplot(augDat,aes(x=Ibu,y=resid,group=ID))+geom_boxplot()+coord_flip()

Figure description: Box Plots of the Pearson residuals per participant ID showing centred clustering around zero. Plotted by ibuprofen use.

ggplot(augDat,aes(x=aspirin,y=resid,group=ID))+geom_boxplot()+coord_flip()

Figure description: Box Plots of the Pearson residuals per participant ID showing centred clustering around zero. Plotted by aspirin use.

ggplot(augDat,aes(x=diab,y=resid,group=ID))+geom_boxplot()+coord_flip()

Figure description: Box Plots of the Pearson residuals per participant ID showing centred clustering around zero. Plotted by diabetes status.

ggplot(augDat,aes(x=naprox,y=resid,group=ID))+geom_boxplot()+coord_flip()

group=ID))+geom_boxplot()+coord_flip()

Figure description: Box Plots of the Pearson residuals per participant ID showing centred clustering around zero. Plotted by naproxen use.

ggplot(augDat,aes(x=celex,y=resid,group=ID))+geom_boxplot()+coord_flip()

Figure description: Box Plots of the Pearson residuals per participant ID showing centred clustering around zero. Plotted by celecoxib use.

ggplot(augDat,aes(x=Gender,y=resid))+geom_boxplot()+coord_flip()

Figure description: Box Plots of the Pearson residuals at the participant level, showing centred clustering around zero. Plotted by gender.

ggplot(augDat,aes(x=AGE,y=resid))+geom_point()+coord_flip()+geom_smooth(method=lm)

Figure description: Box Plots of the Pearson residuals at the participant level, showing centred clustering around zero. Plotted by against age.

ggplot(augDat,aes(x=edu.cat,y=resid))+geom_boxplot()+coord_flip()

Figure description: Box Plots of the Pearson residuals at the participant level, showing centred clustering around zero. Plotted by education level.

ggplot(augDat,aes(x=diagn,y=resid))+geom_boxplot()+coord_flip()

Figure description: Box Plots of the Pearson residuals at the participant level, showing centred clustering around zero. Plotted by cognitive diagnosis.

ggplot(augDat,aes(x=as.factor(APOE4),y=resid))+geom_boxplot()+coord_flip()

Figure description: Box Plots of the Pearson residuals at the participant level, showing centred clustering around zero. Plotted by ApoE4 status.

ggplot(augDat,aes(x=as.factor(M),y=resid))+geom_boxplot()+coord_flip()

Figure description: Box Plots of the Pearson residuals at the participant level, showing centred clustering around zero. Plotted by time (month).

ggplot(augDat,aes(x=as.factor(diclo),y=resid))+geom_boxplot()+coord_flip()

Figure description: Box Plots of the Pearson residuals at the participant level, showing centred clustering around zero. Plotted by diclofenac use.

ggplot(augDat,aes(x=as.factor(vasc),y=resid))+geom_boxplot()+coord_flip()

Figure description: Box Plots of the Pearson residuals at the participant level, showing centred clustering around zero. Plotted by cardiovascular status.

ggplot(augDat,aes(x=as.factor(Ibu),y=resid))+geom_boxplot()+coord_flip()

Figure description: Box Plots of the Pearson residuals at the participant level, showing centred clustering around zero. Plotted by ibuprofen use.

ggplot(augDat,aes(x=as.factor(aspirin),y=resid))+geom_boxplot()+coord_flip()

Figure description: Box Plots of the Pearson residuals at the participant level, showing centred clustering around zero. Plotted by aspirin use.

ggplot(augDat,aes(x=as.factor(diab),y=resid))+geom_boxplot()+coord_flip()

Figure description: Box Plots of the Pearson residuals at the participant level, showing centred clustering around zero. Plotted by diabetes status.

ggplot(augDat,aes(x=as.factor(naprox),y=resid))+geom_boxplot()+coord_flip()

Figure description: Box Plots of the Pearson residuals at the participant level, showing centred clustering around zero. Plotted by naproxen use.

ggplot(augDat,aes(x=as.factor(celex),y=resid))+geom_boxplot()+coord_flip()

Figure description: Box Plots of the Pearson residuals at the participant level, showing centred clustering around zero. Plotted by celecoxib use.

## Analysing all variables in isolation

### Main effect of gender

Adding the main effect of gender to the model did significantly improve the model based on the log-likelihood statistic.

main.A.M<-glmmadmb(neg.b.ADAS~M+ (1|ID), family="nbinom1", data=ADASdata)
main.A.Gender <- glmmadmb(neg.b.ADAS~ M + Gender+ (1|ID), family="nbinom1", data=ADASdata)
summary(main.A.Gender)

Call:
glmmadmb(formula = neg.b.ADAS ~ M + Gender + (1 | ID), data = ADASdata,
 family = "nbinom1")

AIC: 73964.2

Coefficients:
 Estimate Std. Error z value Pr(>|z|)
(Intercept) 3.685405 0.025933 142.1 < 2e-16 ***
M 0.006653 0.000125 53.2 < 2e-16 ***
GenderMale 0.120365 0.034347 3.5 0.00046 ***
---
Signif. codes: 0 '***' 0.001 '**' 0.01 '*' 0.05 '.' 0.1 ' ' 1

Number of observations: total=8770, ID=1618
Random effect variance(s):
Group=ID
 Variance StdDev
(Intercept) 0.4516 0.672

Negative binomial dispersion parameter: 3.2926 (std. err.: 0.055721)

Log-likelihood: -36977.1

anova(main.A.M,main.A.Gender)

Analysis of Deviance Table

Model 1: neg.b.ADAS ~ M
Model 2: neg.b.ADAS ~ M + Gender
 NoPar LogLik Df Deviance Pr(>Chi)
1 4 -36983
2 5 -36977 1 12.2 0.0004779 ***
---
Signif. codes: 0 '***' 0.001 '**' 0.01 '*' 0.05 '.' 0.1 ' ' 1

### Main effect of age

Adding the main effect of age at the beginning of the study (AGE) to the model did improve the model as measured by the log-likelihood statistic.

main.A.AGE <- glmmadmb(neg.b.ADAS~ M + AGE+ (1|ID), family="nbinom1", data=ADASdata)
summary(main.A.AGE)

Call:
glmmadmb(formula = neg.b.ADAS ~ M + AGE + (1 | ID), data = ADASdata,
 family = "nbinom1")

AIC: 73924.6

Coefficients:
 Estimate Std. Error z value Pr(>|z|)
(Intercept) 3.751368 0.017080 219.64 < 2e-16 ***
M 0.006653 0.000125 53.22 < 2e-16 ***
AGE 0.016839 0.002326 7.24 4.5e-13 ***
---
Signif. codes: 0 '***' 0.001 '**' 0.01 '*' 0.05 '.' 0.1 ' ' 1

Number of observations: total=8770, ID=1618
Random effect variance(s):
Group=ID
 Variance StdDev
(Intercept) 0.4408 0.6639

Negative binomial dispersion parameter: 3.2922 (std. err.: 0.055705)

Log-likelihood: -36957.3

anova(main.A.M,main.A.AGE)

Analysis of Deviance Table

Model 1: neg.b.ADAS ~ M
Model 2: neg.b.ADAS ~ M + AGE
 NoPar LogLik Df Deviance Pr(>Chi)
1 4 -36983
2 5 -36957 1 51.8 6.145e-13 ***
---
Signif. codes: 0 '***' 0.001 '**' 0.01 '*' 0.05 '.' 0.1 ' ' 1

### Main effect of education level

Adding the main effect of education (edu.cat) to the model did improve the model as measured by the log-likelihood statistic.

main.A.edu.cat <- glmmadmb(neg.b.ADAS~ M + edu.cat+ (1|ID), family="nbinom1", data=ADASdata)
summary(main.A.edu.cat)

Call:
glmmadmb(formula = neg.b.ADAS ~ M + edu.cat + (1 | ID), data = ADASdata,
 family = "nbinom1")

AIC: 73922.6

Coefficients:
 Estimate Std. Error z value Pr(>|z|)
(Intercept) 3.619878 0.028653 126.34 < 2e-16 ***
M 0.006655 0.000125 53.24 < 2e-16 ***
edu.cat2tertiary 0.145944 0.042412 3.44 0.00058 ***
edu.cat3mid 0.138921 0.047354 2.93 0.00335 **
edu.cat4early 0.380343 0.049750 7.65 2.1e-14 ***
---
Signif. codes: 0 '***' 0.001 '**' 0.01 '*' 0.05 '.' 0.1 ' ' 1

Number of observations: total=8770, ID=1618
Random effect variance(s):
Group=ID
 Variance StdDev
(Intercept) 0.4384 0.6621

Negative binomial dispersion parameter: 3.2928 (std. err.: 0.055725)

Log-likelihood: -36954.3

anova(main.A.M,main.A.edu.cat)

Analysis of Deviance Table

Model 1: neg.b.ADAS ~ M
Model 2: neg.b.ADAS ~ M + edu.cat
 NoPar LogLik Df Deviance Pr(>Chi)
1 4 -36983
2 7 -36954 3 57.8 1.734e-12 ***
---
Signif. codes: 0 '***' 0.001 '**' 0.01 '*' 0.05 '.' 0.1 ' ' 1

### Main effect of diagnosis

Adding the main effect of initial diagnosis (diag) to the model did improve the model as measured by the log-likelihood statistic.

main.A.diagn <- glmmadmb(neg.b.ADAS~ M + diagn+ (1|ID), family="nbinom1", data=ADASdata)
summary(main.A.diagn)

Call:
glmmadmb(formula = neg.b.ADAS ~ M + diagn + (1 | ID), data = ADASdata,
 family = "nbinom1")

AIC: 72687.4

Coefficients:
 Estimate Std. Error z value Pr(>|z|)
(Intercept) 3.112011 0.023147 134.45 <2e-16 ***
M 0.006711 0.000124 53.93 <2e-16 ***
diagn2EMCI 0.321955 0.034833 9.24 <2e-16 ***
diagn3LMCI 0.839691 0.029676 28.30 <2e-16 ***
diagn4AD 1.401136 0.033763 41.50 <2e-16 ***
---
Signif. codes: 0 '***' 0.001 '**' 0.01 '*' 0.05 '.' 0.1 ' ' 1

Number of observations: total=8770, ID=1618
Random effect variance(s):
Group=ID
 Variance StdDev
(Intercept) 0.1907 0.4367

Negative binomial dispersion parameter: 3.3057 (std. err.: 0.05611)

Log-likelihood: -36336.7

anova(main.A.M,main.A.diagn)

Analysis of Deviance Table

Model 1: neg.b.ADAS ~ M
Model 2: neg.b.ADAS ~ M + diagn
 NoPar LogLik Df Deviance Pr(>Chi)
1 4 -36983
2 7 -36337 3 1293 < 2.2e-16 ***
---
Signif. codes: 0 '***' 0.001 '**' 0.01 '*' 0.05 '.' 0.1 ' ' 1

### Main effect of APOE status

main.A.APOE4<-glmmadmb(neg.b.ADAS~M+APOE4+ (1|ID), family="nbinom1", data=ADASdata)
main.A.Gender <- glmmadmb(neg.b.ADAS~ M + Gender+ (1|ID), family="nbinom1", data=ADASdata)
summary(main.A.APOE4)

Call:
glmmadmb(formula = neg.b.ADAS ~ M + APOE4 + (1 | ID), data = ADASdata,
 family = "nbinom1")

AIC: 73794.2

Coefficients:
 Estimate Std. Error z value Pr(>|z|)
(Intercept) 3.545538 0.022640 156.6 <2e-16 ***
M 0.006660 0.000125 53.3 <2e-16 ***
APOE41 0.385965 0.034578 11.2 <2e-16 ***
APOE42 0.601403 0.054310 11.1 <2e-16 ***
---
Signif. codes: 0 '***' 0.001 '**' 0.01 '*' 0.05 '.' 0.1 ' ' 1

Number of observations: total=8770, ID=1618
Random effect variance(s):
Group=ID
 Variance StdDev
(Intercept) 0.4031 0.6349

Negative binomial dispersion parameter: 3.2948 (std. err.: 0.055794)

Log-likelihood: -36891.1

anova(main.A.M,main.A.APOE4)

Analysis of Deviance Table

Model 1: neg.b.ADAS ~ M
Model 2: neg.b.ADAS ~ M + APOE4
 NoPar LogLik Df Deviance Pr(>Chi)
1 4 -36983
2 6 -36891 2 184.2 < 2.2e-16 ***
---
Signif. codes: 0 '***' 0.001 '**' 0.01 '*' 0.05 '.' 0.1 ' ' 1

### Main effect of cardiovascular pathology

Adding the main effect of cardiovascular co-morbidity (vasc) to the model did significantly improve the model as measured by the log-likelihood statistic.

main.A.vasc <- glmmadmb(neg.b.ADAS~ M + vasc+ (1|ID), family="nbinom1", data=ADASdata)
summary(main.A.vasc)

Call:
glmmadmb(formula = neg.b.ADAS ~ M + vasc + (1 | ID), data = ADASdata,
 family = "nbinom1")

AIC: 73976.4

Coefficients:
 Estimate Std. Error z value Pr(>|z|)
(Intercept) 3.751769 0.027713 135.38 <2e-16 ***
M 0.006656 0.000125 53.23 <2e-16 ***
vasc 0.002068 0.035144 0.06 0.95
---
Signif. codes: 0 '***' 0.001 '**' 0.01 '*' 0.05 '.' 0.1 ' ' 1

Number of observations: total=8770, ID=1618
Random effect variance(s):
Group=ID
 Variance StdDev
(Intercept) 0.4548 0.6744

Negative binomial dispersion parameter: 3.2929 (std. err.: 0.055733)

Log-likelihood: -36983.2

anova(main.A.M,main.A.vasc)

Analysis of Deviance Table

Model 1: neg.b.ADAS ~ M
Model 2: neg.b.ADAS ~ M + vasc
 NoPar LogLik Df Deviance Pr(>Chi)
1 4 -36983
2 5 -36983 1 0 1

### Main effect of diabetes

Adding the main effect of diabetes co-morbidity (diab) to the model did not improve the model as measured by the log-likelihood statistic.

main.A.diab <- glmmadmb(neg.b.ADAS~ M + diab+ (1|ID), family="nbinom1", data=ADASdata)
summary(main.A.diab)

Call:
glmmadmb(formula = neg.b.ADAS ~ M + diab + (1 | ID), data = ADASdata,
 family = "nbinom1")

AIC: 73976.4

Coefficients:
 Estimate Std. Error z value Pr(>|z|)
(Intercept) 3.754193 0.018169 206.63 <2e-16 ***
M 0.006656 0.000125 53.23 <2e-16 ***
diab -0.012424 0.058942 -0.21 0.83
---
Signif. codes: 0 '***' 0.001 '**' 0.01 '*' 0.05 '.' 0.1 ' ' 1

Number of observations: total=8770, ID=1618
Random effect variance(s):
Group=ID
 Variance StdDev
(Intercept) 0.4548 0.6744

Negative binomial dispersion parameter: 3.2929 (std. err.: 0.055733)

Log-likelihood: -36983.2

anova(main.A.M,main.A.diab)

Analysis of Deviance Table

Model 1: neg.b.ADAS ~ M
Model 2: neg.b.ADAS ~ M + diab
 NoPar LogLik Df Deviance Pr(>Chi)
1 4 -36983
2 5 -36983 1 0 1

### Main effect of smoking

Adding the main effect of smoking at the beginning of the study (AGE) to the model did improve the model as measured by the log-likelihood statistic.

main.A.smoke <- glmmadmb(neg.b.ADAS~ M + smoke+ (1|ID), family="nbinom1", data=ADASdata)
summary(main.A.smoke)

Call:
glmmadmb(formula = neg.b.ADAS ~ M + smoke + (1 | ID), data = ADASdata,
 family = "nbinom1")

AIC: 73976

Coefficients:
 Estimate Std. Error z value Pr(>|z|)
(Intercept) 3.746344 0.019997 187.35 <2e-16 ***
M 0.006656 0.000125 53.23 <2e-16 ***
smoke 0.026343 0.039232 0.67 0.5
---
Signif. codes: 0 '***' 0.001 '**' 0.01 '*' 0.05 '.' 0.1 ' ' 1

Number of observations: total=8770, ID=1618
Random effect variance(s):
Group=ID
 Variance StdDev
(Intercept) 0.4547 0.6743

Negative binomial dispersion parameter: 3.2929 (std. err.: 0.055733)

Log-likelihood: -36983

anova(main.A.M,main.A.smoke)

Analysis of Deviance Table

Model 1: neg.b.ADAS ~ M
Model 2: neg.b.ADAS ~ M + smoke
 NoPar LogLik Df Deviance Pr(>Chi)
1 4 -36983
2 5 -36983 1 0.4 0.5271

### Main effect of headache

Adding the main effect of headache at the beginning of the study (AGE) to the model did improve the model as measured by the log-likelihood statistic.

main.A.headache <- glmmadmb(neg.b.ADAS~ M + headache+ (1|ID), family="nbinom1", data=ADASdata)
summary(main.A.headache)

Call:
glmmadmb(formula = neg.b.ADAS ~ M + headache + (1 | ID), data = ADASdata,
 family = "nbinom1")

AIC: 73966

Coefficients:
 Estimate Std. Error z value Pr(>|z|)
(Intercept) 3.769872 0.018030 209.09 <2e-16 ***
M 0.006660 0.000125 53.27 <2e-16 ***
headache -0.197430 0.061015 -3.24 0.0012 **
---
Signif. codes: 0 '***' 0.001 '**' 0.01 '*' 0.05 '.' 0.1 ' ' 1

Number of observations: total=8770, ID=1618
Random effect variance(s):
Group=ID
 Variance StdDev
(Intercept) 0.4518 0.6722

Negative binomial dispersion parameter: 3.2927 (std. err.: 0.055726)

Log-likelihood: -36978

anova(main.A.M,main.A.headache)

Analysis of Deviance Table

Model 1: neg.b.ADAS ~ M
Model 2: neg.b.ADAS ~ M + headache
 NoPar LogLik Df Deviance Pr(>Chi)
1 4 -36983
2 5 -36978 1 10.4 0.00126 **
---
Signif. codes: 0 '***' 0.001 '**' 0.01 '*' 0.05 '.' 0.1 ' ' 1

### Main effect of arthritis

Adding the main effect of headache at the beginning of the study (AGE) to the model did improve the model as measured by the log-likelihood statistic.

main.A.arthrit <- glmmadmb(neg.b.ADAS~ M + arthrit+ (1|ID), family="nbinom1", data=ADASdata)
summary(main.A.arthrit)

Call:
glmmadmb(formula = neg.b.ADAS ~ M + arthrit + (1 | ID), data = ADASdata,
 family = "nbinom1")

AIC: 73967.8

Coefficients:
 Estimate Std. Error z value Pr(>|z|)
(Intercept) 3.792670 0.021942 172.85 <2e-16 ***
M 0.006659 0.000125 53.26 <2e-16 ***
arthrit -0.102313 0.034997 -2.92 0.0035 **
---
Signif. codes: 0 '***' 0.001 '**' 0.01 '*' 0.05 '.' 0.1 ' ' 1

Number of observations: total=8770, ID=1618
Random effect variance(s):
Group=ID
 Variance StdDev
(Intercept) 0.4522 0.6725

Negative binomial dispersion parameter: 3.2929 (std. err.: 0.055733)

Log-likelihood: -36978.9

anova(main.A.M,main.A.arthrit)

Analysis of Deviance Table

Model 1: neg.b.ADAS ~ M
Model 2: neg.b.ADAS ~ M + arthrit
 NoPar LogLik Df Deviance Pr(>Chi)
1 4 -36983
2 5 -36979 1 8.6 0.003362 **
---
Signif. codes: 0 '***' 0.001 '**' 0.01 '*' 0.05 '.' 0.1 ' ' 1

### Main effect of diclofenac

Adding the main effect of diclofenac to the model did not improve the model as measured by the log-likelihood statistic.

main.A.diclo <- glmmadmb(neg.b.ADAS~M+diclo+ (1|ID), family="nbinom1", data=ADASdata)
summary(main.A.diclo)

Call:
glmmadmb(formula = neg.b.ADAS ~ M + diclo + (1 | ID), data = ADASdata,
 family = "nbinom1")

AIC: 73963.4

Coefficients:
 Estimate Std. Error z value Pr(>|z|)
(Intercept) 3.761482 0.017415 216.0 < 2e-16 ***
M 0.006659 0.000125 53.3 < 2e-16 ***
diclo -0.455421 0.126530 -3.6 0.00032 ***
---
Signif. codes: 0 '***' 0.001 '**' 0.01 '*' 0.05 '.' 0.1 ' ' 1

Number of observations: total=8770, ID=1618
Random effect variance(s):
Group=ID
 Variance StdDev
(Intercept) 0.4511 0.6716

Negative binomial dispersion parameter: 3.2927 (std. err.: 0.055725)

Log-likelihood: -36976.7

anova(main.A.M,main.A.diclo)

Analysis of Deviance Table

Model 1: neg.b.ADAS ~ M
Model 2: neg.b.ADAS ~ M + diclo
 NoPar LogLik Df Deviance Pr(>Chi)
1 4 -36983
2 5 -36977 1 13 0.0003115 ***
---
Signif. codes: 0 '***' 0.001 '**' 0.01 '*' 0.05 '.' 0.1 ' ' 1

### Main effect of paracetamol

Adding the main effect of paracetamol to the model did improve the model as measured by the log-likelihood statistic.

main.A.parac <- glmmadmb(neg.b.ADAS~ M + parac+ (1|ID), family="nbinom1", data=ADASdata)
summary(main.A.parac)

Call:
glmmadmb(formula = neg.b.ADAS ~ M + parac + (1 | ID), data = ADASdata,
 family = "nbinom1")

AIC: 73967.8

Coefficients:
 Estimate Std. Error z value Pr(>|z|)
(Intercept) 3.781592 0.019841 190.59 <2e-16 ***
M 0.006663 0.000125 53.28 <2e-16 ***
parac -0.115191 0.039454 -2.92 0.0035 **
---
Signif. codes: 0 '***' 0.001 '**' 0.01 '*' 0.05 '.' 0.1 ' ' 1

Number of observations: total=8770, ID=1618
Random effect variance(s):
Group=ID
 Variance StdDev
(Intercept) 0.4523 0.6725

Negative binomial dispersion parameter: 3.2927 (std. err.: 0.055726)

Log-likelihood: -36978.9

anova(main.A.M,main.A.parac)

Analysis of Deviance Table

Model 1: neg.b.ADAS ~ M
Model 2: neg.b.ADAS ~ M + parac
 NoPar LogLik Df Deviance Pr(>Chi)
1 4 -36983
2 5 -36979 1 8.6 0.003362 **
---
Signif. codes: 0 '***' 0.001 '**' 0.01 '*' 0.05 '.' 0.1 ' ' 1

### Main effect celecoxib

Adding the main effect of celecoxib to the model did not improve the model as measured by the log-likelihood statistic.

main.A.celex <- glmmadmb(neg.b.ADAS~ M + celex+ (1|ID), family="nbinom1", data=ADASdata)
summary(main.A.celex)

Call:
glmmadmb(formula = neg.b.ADAS ~ M + celex + (1 | ID), data = ADASdata,
 family = "nbinom1")

AIC: 73975.2

Coefficients:
 Estimate Std. Error z value Pr(>|z|)
(Intercept) 3.756720 0.017664 212.68 <2e-16 ***
M 0.006657 0.000125 53.24 <2e-16 ***
celex -0.092952 0.087559 -1.06 0.29
---
Signif. codes: 0 '***' 0.001 '**' 0.01 '*' 0.05 '.' 0.1 ' ' 1

Number of observations: total=8770, ID=1618
Random effect variance(s):
Group=ID
 Variance StdDev
(Intercept) 0.4545 0.6741

Negative binomial dispersion parameter: 3.2929 (std. err.: 0.055733)

Log-likelihood: -36982.6

anova(main.A.M,main.A.celex)

Analysis of Deviance Table

Model 1: neg.b.ADAS ~ M
Model 2: neg.b.ADAS ~ M + celex
 NoPar LogLik Df Deviance Pr(>Chi)
1 4 -36983
2 5 -36983 1 1.2 0.2733

### Main effect of naproxen

Adding the main effect of naproxen to the model did significantly improve the model as measured by the log-likelihood statistic.

main.A.naprox <- glmmadmb(neg.b.ADAS~ M + naprox+ (1|ID), family="nbinom1", data=ADASdata)
summary(main.A.naprox)

Call:
glmmadmb(formula = neg.b.ADAS ~ M + naprox + (1 | ID), data = ADASdata,
 family = "nbinom1")

AIC: 73967.8

Coefficients:
 Estimate Std. Error z value Pr(>|z|)
(Intercept) 3.771189 0.018333 205.70 <2e-16 ***
M 0.006660 0.000125 53.26 <2e-16 ***
naprox -0.157431 0.053357 -2.95 0.0032 **
---
Signif. codes: 0 '***' 0.001 '**' 0.01 '*' 0.05 '.' 0.1 ' ' 1

Number of observations: total=8770, ID=1618
Random effect variance(s):
Group=ID
 Variance StdDev
(Intercept) 0.4522 0.6725

Negative binomial dispersion parameter: 3.2928 (std. err.: 0.055729)

Log-likelihood: -36978.9

anova(main.A.M,main.A.naprox)

Analysis of Deviance Table

Model 1: neg.b.ADAS ~ M
Model 2: neg.b.ADAS ~ M + naprox
 NoPar LogLik Df Deviance Pr(>Chi)
1 4 -36983
2 5 -36979 1 8.6 0.003362 **
---
Signif. codes: 0 '***' 0.001 '**' 0.01 '*' 0.05 '.' 0.1 ' ' 1

### Main effect of aspirin

Adding the main effect of aspirin use (aspirin) to the model did improve the model as measured by the log-likelihood statistic.

main.A.aspirin <- glmmadmb(neg.b.ADAS~ M + aspirin+ (1|ID), family="nbinom1", data=ADASdata)
summary(main.A.aspirin)

Call:
glmmadmb(formula = neg.b.ADAS ~ M + aspirin + (1 | ID), data = ADASdata,
 family = "nbinom1")

AIC: 73962.2

Coefficients:
 Estimate Std. Error z value Pr(>|z|)
(Intercept) 3.821872 0.025024 152.73 < 2e-16 ***
M 0.006666 0.000125 53.30 < 2e-16 ***
aspirin -0.129156 0.034112 -3.79 0.00015 ***
---
Signif. codes: 0 '***' 0.001 '**' 0.01 '*' 0.05 '.' 0.1 ' ' 1

Number of observations: total=8770, ID=1618
Random effect variance(s):
Group=ID
 Variance StdDev
(Intercept) 0.4502 0.671

Negative binomial dispersion parameter: 3.2933 (std. err.: 0.055742)

Log-likelihood: -36976.1

anova(main.A.M,main.A.aspirin)

Analysis of Deviance Table

Model 1: neg.b.ADAS ~ M
Model 2: neg.b.ADAS ~ M + aspirin
 NoPar LogLik Df Deviance Pr(>Chi)
1 4 -36983
2 5 -36976 1 14.2 0.0001644 ***
---
Signif. codes: 0 '***' 0.001 '**' 0.01 '*' 0.05 '.' 0.1 ' ' 1

### Main effect of ibuprofen

Adding the main effect of ibuprofen use (Ibu) to the model did improve the model as measured by the log-likelihood statistic.

main.A.Ibu <- glmmadmb(neg.b.ADAS~ M + Ibu+ (1|ID), family="nbinom1", data=ADASdata)
summary(main.A.Ibu)

Call:
glmmadmb(formula = neg.b.ADAS ~ M + Ibu + (1 | ID), data = ADASdata,
 family = "nbinom1")

AIC: 73946.2

Coefficients:
 Estimate Std. Error z value Pr(>|z|)
(Intercept) 3.793494 0.018651 203.39 < 2e-16 ***
M 0.006663 0.000125 53.30 < 2e-16 ***
Ibu -0.256593 0.046522 -5.52 3.5e-08 ***
---
Signif. codes: 0 '***' 0.001 '**' 0.01 '*' 0.05 '.' 0.1 ' ' 1

Number of observations: total=8770, ID=1618
Random effect variance(s):
Group=ID
 Variance StdDev
(Intercept) 0.4464 0.6681

Negative binomial dispersion parameter: 3.2923 (std. err.: 0.055716)

Log-likelihood: -36968.1

anova(main.A.M,main.A.Ibu)

Analysis of Deviance Table

Model 1: neg.b.ADAS ~ M
Model 2: neg.b.ADAS ~ M + Ibu
 NoPar LogLik Df Deviance Pr(>Chi)
1 4 -36983
2 5 -36968 1 30.2 3.897e-08 ***
---
Signif. codes: 0 '***' 0.001 '**' 0.01 '*' 0.05 '.' 0.1 ' ' 1

## Building combined main effect model

main.A.combined <- glmmadmb(neg.b.ADAS~M+AGE+ APOE4+Gender+ edu.cat + diagn+ headache+arthrit+ diclo+parac+naprox+aspirin+Ibu+ (1|ID), family="nbinom1", data=ADASdata)
summary(main.A.combined)

Call:
glmmadmb(formula = neg.b.ADAS ~ M + AGE + APOE4 + Gender + edu.cat +
 diagn + headache + arthrit + diclo + parac + naprox + aspirin +
 Ibu + (1 | ID), data = ADASdata, family = "nbinom1")

AIC: 72513.2

Coefficients:
 Estimate Std. Error z value Pr(>|z|)
(Intercept) 3.017811 0.034249 88.11 < 2e-16 ***
M 0.006727 0.000124 54.10 < 2e-16 ***
AGE 0.013673 0.001569 8.71 < 2e-16 ***
APOE41 0.163086 0.023681 6.89 5.7e-12 ***
APOE42 0.241815 0.037636 6.43 1.3e-10 ***
GenderMale 0.063162 0.022660 2.79 0.00531 **
edu.cat2tertiary 0.058493 0.027209 2.15 0.03157 *
edu.cat3mid 0.118337 0.030791 3.84 0.00012 ***
edu.cat4early 0.152681 0.032597 4.68 2.8e-06 ***
diagn2EMCI 0.325307 0.033546 9.70 < 2e-16 ***
diagn3LMCI 0.777334 0.028977 26.83 < 2e-16 ***
diagn4AD 1.274572 0.033966 37.52 < 2e-16 ***
headache -0.088027 0.038992 -2.26 0.02397 *
arthrit -0.017290 0.022850 -0.76 0.44924
diclo -0.220562 0.080433 -2.74 0.00610 **
parac -0.013643 0.025693 -0.53 0.59542
naprox -0.010708 0.034122 -0.31 0.75367
aspirin -0.018929 0.021981 -0.86 0.38915
Ibu -0.094950 0.029884 -3.18 0.00149 **
---
Signif. codes: 0 '***' 0.001 '**' 0.01 '*' 0.05 '.' 0.1 ' ' 1

Number of observations: total=8770, ID=1618
Random effect variance(s):
Group=ID
 Variance StdDev
(Intercept) 0.1672 0.4089

Negative binomial dispersion parameter: 3.3029 (std. err.: 0.055996)

Log-likelihood: -36235.6

anova(main.A.M,main.A.combined)

Analysis of Deviance Table

Model 1: neg.b.ADAS ~ M
Model 2: neg.b.ADAS ~ M + AGE + APOE4 + Gender + edu.cat + diagn + headache + arthrit + diclo + parac + naprox + aspirin + Ibu
 NoPar LogLik Df Deviance Pr(>Chi)
1 4 -36983
2 21 -36236 17 1495.2 < 2.2e-16 ***
---
Signif. codes: 0 '***' 0.001 '**' 0.01 '*' 0.05 '.' 0.1 ' ' 1

## Dropping non-significant terms

### Narpoxen

Dropping naproxen from the model did not significantly worsen the model

main.A.combined.drop.naprox <- glmmadmb(neg.b.ADAS~M+AGE+ APOE4+Gender+ edu.cat + diagn+ headache+arthrit+ diclo+parac+aspirin+Ibu+ (1|ID), family="nbinom1", data=ADASdata)
summary(main.A.combined.drop.naprox)

Call:
glmmadmb(formula = neg.b.ADAS ~ M + AGE + APOE4 + Gender + edu.cat +
 diagn + headache + arthrit + diclo + parac + aspirin + Ibu +
 (1 | ID), data = ADASdata, family = "nbinom1")

AIC: 72511.2

Coefficients:
 Estimate Std. Error z value Pr(>|z|)
(Intercept) 3.017192 0.034193 88.24 < 2e-16 ***
M 0.006727 0.000124 54.10 < 2e-16 ***
AGE 0.013697 0.001567 8.74 < 2e-16 ***
APOE41 0.163108 0.023681 6.89 5.7e-12 ***
APOE42 0.241901 0.037635 6.43 1.3e-10 ***
GenderMale 0.063369 0.022651 2.80 0.00515 **
edu.cat2tertiary 0.058214 0.027195 2.14 0.03230 *
edu.cat3mid 0.118172 0.030787 3.84 0.00012 ***
edu.cat4early 0.152481 0.032591 4.68 2.9e-06 ***
diagn2EMCI 0.325072 0.033538 9.69 < 2e-16 ***
diagn3LMCI 0.777288 0.028977 26.82 < 2e-16 ***
diagn4AD 1.275002 0.033939 37.57 < 2e-16 ***
headache -0.088009 0.038993 -2.26 0.02401 *
arthrit -0.017787 0.022796 -0.78 0.43524
diclo -0.220646 0.080433 -2.74 0.00608 **
parac -0.014713 0.025466 -0.58 0.56342
aspirin -0.019106 0.021974 -0.87 0.38459
Ibu -0.095481 0.029837 -3.20 0.00137 **
---
Signif. codes: 0 '***' 0.001 '**' 0.01 '*' 0.05 '.' 0.1 ' ' 1

Number of observations: total=8770, ID=1618
Random effect variance(s):
Group=ID
 Variance StdDev
(Intercept) 0.1672 0.4089

Negative binomial dispersion parameter: 3.3029 (std. err.: 0.055997)

Log-likelihood: -36235.6

anova(main.A.combined, main.A.combined.drop.naprox)

Analysis of Deviance Table

Model 1: neg.b.ADAS ~ M + AGE + APOE4 + Gender + edu.cat + diagn + headache + arthrit + diclo + parac + aspirin + Ibu
Model 2: neg.b.ADAS ~ M + AGE + APOE4 + Gender + edu.cat + diagn + headache + arthrit + diclo + parac + naprox + aspirin + Ibu
 NoPar LogLik Df Deviance Pr(>Chi)
1 20 -36236
2 21 -36236 1 0 1

### Paracetamol

Dropping paracetamol from the model did not significantly worsen the model

main.A.combined.drop.parac<- glmmadmb(neg.b.ADAS~M+AGE+ APOE4+Gender+ edu.cat + diagn+ headache+arthrit+ diclo+aspirin+Ibu+ (1|ID), family="nbinom1", data=ADASdata)
summary(main.A.combined.drop.parac)

Call:
glmmadmb(formula = neg.b.ADAS ~ M + AGE + APOE4 + Gender + edu.cat +
 diagn + headache + arthrit + diclo + aspirin + Ibu + (1 |
 ID), data = ADASdata, family = "nbinom1")

AIC: 72509.6

Coefficients:
 Estimate Std. Error z value Pr(>|z|)
(Intercept) 3.014473 0.033873 88.99 < 2e-16 ***
M 0.006725 0.000124 54.10 < 2e-16 ***
AGE 0.013664 0.001566 8.72 < 2e-16 ***
APOE41 0.163741 0.023659 6.92 4.5e-12 ***
APOE42 0.242298 0.037633 6.44 1.2e-10 ***
GenderMale 0.064281 0.022599 2.84 0.00445 **
edu.cat2tertiary 0.057853 0.027190 2.13 0.03336 *
edu.cat3mid 0.117554 0.030771 3.82 0.00013 ***
edu.cat4early 0.152769 0.032591 4.69 2.8e-06 ***
diagn2EMCI 0.325278 0.033540 9.70 < 2e-16 ***
diagn3LMCI 0.777241 0.028980 26.82 < 2e-16 ***
diagn4AD 1.275416 0.033935 37.58 < 2e-16 ***
headache -0.089581 0.038902 -2.30 0.02129 *
arthrit -0.019697 0.022557 -0.87 0.38256
diclo -0.221612 0.080423 -2.76 0.00586 **
aspirin -0.020072 0.021913 -0.92 0.35967
Ibu -0.097059 0.029715 -3.27 0.00109 **
---
Signif. codes: 0 '***' 0.001 '**' 0.01 '*' 0.05 '.' 0.1 ' ' 1

Number of observations: total=8770, ID=1618
Random effect variance(s):
Group=ID
 Variance StdDev
(Intercept) 0.1672 0.4089

Negative binomial dispersion parameter: 3.303 (std. err.: 0.056)

Log-likelihood: -36235.8

anova(main.A.combined.drop.naprox, main.A.combined.drop.parac)

Analysis of Deviance Table

Model 1: neg.b.ADAS ~ M + AGE + APOE4 + Gender + edu.cat + diagn + headache + arthrit + diclo + aspirin + Ibu
Model 2: neg.b.ADAS ~ M + AGE + APOE4 + Gender + edu.cat + diagn + headache + arthrit + diclo + parac + aspirin + Ibu
 NoPar LogLik Df Deviance Pr(>Chi)
1 19 -36236
2 20 -36236 1 0.4 0.5271

### Arthritis

Dropping arthritis from the model did not significantly worsen the model

main.A.combined.drop.arthritis<- glmmadmb(neg.b.ADAS~M+AGE+ APOE4+Gender+ edu.cat + diagn+ headache+ diclo+aspirin+Ibu+ (1|ID), family="nbinom1", data=ADASdata)
summary(main.A.combined.drop.arthritis)

Call:
glmmadmb(formula = neg.b.ADAS ~ M + AGE + APOE4 + Gender + edu.cat +
 diagn + headache + diclo + aspirin + Ibu + (1 | ID), data = ADASdata,
 family = "nbinom1")

AIC: 72508.2

Coefficients:
 Estimate Std. Error z value Pr(>|z|)
(Intercept) 3.006958 0.032775 91.75 < 2e-16 ***
M 0.006724 0.000124 54.10 < 2e-16 ***
AGE 0.013512 0.001557 8.68 < 2e-16 ***
APOE41 0.163040 0.023651 6.89 5.4e-12 ***
APOE42 0.242894 0.037637 6.45 1.1e-10 ***
GenderMale 0.065879 0.022531 2.92 0.00346 **
edu.cat2tertiary 0.056927 0.027177 2.09 0.03620 *
edu.cat3mid 0.116546 0.030758 3.79 0.00015 ***
edu.cat4early 0.151499 0.032567 4.65 3.3e-06 ***
diagn2EMCI 0.325251 0.033549 9.69 < 2e-16 ***
diagn3LMCI 0.778288 0.028963 26.87 < 2e-16 ***
diagn4AD 1.277357 0.033871 37.71 < 2e-16 ***
headache -0.092242 0.038792 -2.38 0.01741 *
diclo -0.228398 0.080070 -2.85 0.00434 **
aspirin -0.020381 0.021916 -0.93 0.35240
Ibu -0.099410 0.029601 -3.36 0.00078 ***
---
Signif. codes: 0 '***' 0.001 '**' 0.01 '*' 0.05 '.' 0.1 ' ' 1

Number of observations: total=8770, ID=1618
Random effect variance(s):
Group=ID
 Variance StdDev
(Intercept) 0.1673 0.4091

Negative binomial dispersion parameter: 3.303 (std. err.: 0.056)

Log-likelihood: -36236.1

anova(main.A.combined.drop.parac, main.A.combined.drop.arthritis)

Analysis of Deviance Table

Model 1: neg.b.ADAS ~ M + AGE + APOE4 + Gender + edu.cat + diagn + headache + diclo + aspirin + Ibu
Model 2: neg.b.ADAS ~ M + AGE + APOE4 + Gender + edu.cat + diagn + headache + arthrit + diclo + aspirin + Ibu
 NoPar LogLik Df Deviance Pr(>Chi)
1 18 -36236
2 19 -36236 1 0.6 0.4386

### Aspirin

Dropping aspirin from the model did not significantly worsen the model

main.A.combined.drop.aspirin<- glmmadmb(neg.b.ADAS~M+AGE+ APOE4+Gender+ edu.cat + diagn+ headache+ diclo+Ibu+ (1|ID), family="nbinom1", data=ADASdata)
summary(main.A.combined.drop.aspirin)

Call:
glmmadmb(formula = neg.b.ADAS ~ M + AGE + APOE4 + Gender + edu.cat +
 diagn + headache + diclo + Ibu + (1 | ID), data = ADASdata,
 family = "nbinom1")

AIC: 72507.2

Coefficients:
 Estimate Std. Error z value Pr(>|z|)
(Intercept) 2.996170 0.030673 97.68 < 2e-16 ***
M 0.006721 0.000124 54.10 < 2e-16 ***
AGE 0.013441 0.001556 8.64 < 2e-16 ***
APOE41 0.163353 0.023658 6.90 5.0e-12 ***
APOE42 0.242161 0.037644 6.43 1.3e-10 ***
GenderMale 0.063235 0.022359 2.83 0.00468 **
edu.cat2tertiary 0.057039 0.027187 2.10 0.03590 *
edu.cat3mid 0.116509 0.030770 3.79 0.00015 ***
edu.cat4early 0.152613 0.032558 4.69 2.8e-06 ***
diagn2EMCI 0.325540 0.033561 9.70 < 2e-16 ***
diagn3LMCI 0.779652 0.028938 26.94 < 2e-16 ***
diagn4AD 1.280977 0.033661 38.06 < 2e-16 ***
headache -0.091763 0.038804 -2.36 0.01804 *
diclo -0.232052 0.080003 -2.90 0.00373 **
Ibu -0.099927 0.029608 -3.38 0.00074 ***
---
Signif. codes: 0 '***' 0.001 '**' 0.01 '*' 0.05 '.' 0.1 ' ' 1

Number of observations: total=8770, ID=1618
Random effect variance(s):
Group=ID
 Variance StdDev
(Intercept) 0.1675 0.4092

Negative binomial dispersion parameter: 3.3029 (std. err.: 0.056)

Log-likelihood: -36236.6

anova(main.A.combined.drop.arthritis, main.A.combined.drop.aspirin)

Analysis of Deviance Table

Model 1: neg.b.ADAS ~ M + AGE + APOE4 + Gender + edu.cat + diagn + headache + diclo + Ibu
Model 2: neg.b.ADAS ~ M + AGE + APOE4 + Gender + edu.cat + diagn + headache + diclo + aspirin + Ibu
 NoPar LogLik Df Deviance Pr(>Chi)
1 17 -36237
2 18 -36236 1 1 0.3173

## Building combined main effect model

main.A.final<- glmmadmb(neg.b.ADAS~M+AGE+ APOE4+Gender+ edu.cat + diagn+ headache+ diclo+Ibu+ (1|ID), family="nbinom1", data=ADASdata)
summary(main.A.final)

Call:
glmmadmb(formula = neg.b.ADAS ~ M + AGE + APOE4 + Gender + edu.cat +
 diagn + headache + diclo + Ibu + (1 | ID), data = ADASdata,
 family = "nbinom1")

AIC: 72507.2

Coefficients:
 Estimate Std. Error z value Pr(>|z|)
(Intercept) 2.996170 0.030673 97.68 < 2e-16 ***
M 0.006721 0.000124 54.10 < 2e-16 ***
AGE 0.013441 0.001556 8.64 < 2e-16 ***
APOE41 0.163353 0.023658 6.90 5.0e-12 ***
APOE42 0.242161 0.037644 6.43 1.3e-10 ***
GenderMale 0.063235 0.022359 2.83 0.00468 **
edu.cat2tertiary 0.057039 0.027187 2.10 0.03590 *
edu.cat3mid 0.116509 0.030770 3.79 0.00015 ***
edu.cat4early 0.152613 0.032558 4.69 2.8e-06 ***
diagn2EMCI 0.325540 0.033561 9.70 < 2e-16 ***
diagn3LMCI 0.779652 0.028938 26.94 < 2e-16 ***
diagn4AD 1.280977 0.033661 38.06 < 2e-16 ***
headache -0.091763 0.038804 -2.36 0.01804 *
diclo -0.232052 0.080003 -2.90 0.00373 **
Ibu -0.099927 0.029608 -3.38 0.00074 ***
---
Signif. codes: 0 '***' 0.001 '**' 0.01 '*' 0.05 '.' 0.1 ' ' 1

Number of observations: total=8770, ID=1618
Random effect variance(s):
Group=ID
 Variance StdDev
(Intercept) 0.1675 0.4092

Negative binomial dispersion parameter: 3.3029 (std. err.: 0.056)

Log-likelihood: -36236.6

anova(main.A.combined,main.A.final)

Analysis of Deviance Table

Model 1: neg.b.ADAS ~ M + AGE + APOE4 + Gender + edu.cat + diagn + headache + diclo + Ibu
Model 2: neg.b.ADAS ~ M + AGE + APOE4 + Gender + edu.cat + diagn + headache + arthrit + diclo + parac + naprox + aspirin + Ibu
 NoPar LogLik Df Deviance Pr(>Chi)
1 17 -36237
2 21 -36236 4 2 0.7358

## Removing each explanitory variable in isolation

### Main effect of age at the start of the study

main.A.final.drop.AGE<- glmmadmb(neg.b.ADAS~M+ APOE4+Gender+ edu.cat + diagn+ headache+ diclo+Ibu+ (1|ID), family="nbinom1", data=ADASdata)
anova(main.A.final,main.A.final.drop.AGE)

Analysis of Deviance Table

Model 1: neg.b.ADAS ~ M + APOE4 + Gender + edu.cat + diagn + headache + diclo + Ibu
Model 2: neg.b.ADAS ~ M + AGE + APOE4 + Gender + edu.cat + diagn + headache + diclo + Ibu
 NoPar LogLik Df Deviance Pr(>Chi)
1 16 -36273
2 17 -36237 1 73.2 < 2.2e-16 ***
---
Signif. codes: 0 '***' 0.001 '**' 0.01 '*' 0.05 '.' 0.1 ' ' 1

### Main effect of APOE4 genotype

main.A.final.drop.APOE4<- glmmadmb(neg.b.ADAS~M+AGE+ Gender+ edu.cat + diagn+ headache+ diclo+Ibu+ (1|ID), family="nbinom1", data=ADASdata)
anova(main.A.final.drop.APOE4,main.A.final)

Analysis of Deviance Table

Model 1: neg.b.ADAS ~ M + AGE + Gender + edu.cat + diagn + headache + diclo + Ibu
Model 2: neg.b.ADAS ~ M + AGE + APOE4 + Gender + edu.cat + diagn + headache + diclo + Ibu
 NoPar LogLik Df Deviance Pr(>Chi)
1 15 -36270
2 17 -36237 2 66 4.663e-15 ***
---
Signif. codes: 0 '***' 0.001 '**' 0.01 '*' 0.05 '.' 0.1 ' ' 1

### Main effect of education level

main.A.final.drop.edu.cat<- glmmadmb(neg.b.ADAS~M+AGE+ APOE4+Gender+ diagn+ headache+ diclo+Ibu+ (1|ID), family="nbinom1", data=ADASdata)
anova(main.A.final.drop.edu.cat,main.A.final)

Analysis of Deviance Table

Model 1: neg.b.ADAS ~ M + AGE + APOE4 + Gender + diagn + headache + diclo + Ibu
Model 2: neg.b.ADAS ~ M + AGE + APOE4 + Gender + edu.cat + diagn + headache + diclo + Ibu
 NoPar LogLik Df Deviance Pr(>Chi)
1 14 -36250
2 17 -36237 3 27 5.887e-06 ***
---
Signif. codes: 0 '***' 0.001 '**' 0.01 '*' 0.05 '.' 0.1 ' ' 1

### Main effect of initial Alzhiemer’s diagnosis

main.A.final.drop.diagn<- glmmadmb(neg.b.ADAS~M+AGE+ APOE4+Gender+ edu.cat + headache+ diclo+Ibu+ (1|ID), family="nbinom1", data=ADASdata)
anova(main.A.final.drop.diagn,main.A.final)

Analysis of Deviance Table

Model 1: neg.b.ADAS ~ M + AGE + APOE4 + Gender + edu.cat + headache + diclo + Ibu
Model 2: neg.b.ADAS ~ M + AGE + APOE4 + Gender + edu.cat + diagn + headache + diclo + Ibu
 NoPar LogLik Df Deviance Pr(>Chi)
1 14 -36799
2 17 -36237 3 1124.8 < 2.2e-16 ***
---
Signif. codes: 0 '***' 0.001 '**' 0.01 '*' 0.05 '.' 0.1 ' ' 1

### Main effect of headaches

main.A.final.drop.headaches<- glmmadmb(neg.b.ADAS~M+AGE+ APOE4+Gender+ edu.cat + diagn+ diclo+Ibu+ (1|ID), family="nbinom1", data=ADASdata)
anova(main.A.final.drop.headaches,main.A.final)

Analysis of Deviance Table

Model 1: neg.b.ADAS ~ M + AGE + APOE4 + Gender + edu.cat + diagn + diclo + Ibu
Model 2: neg.b.ADAS ~ M + AGE + APOE4 + Gender + edu.cat + diagn + headache + diclo + Ibu
 NoPar LogLik Df Deviance Pr(>Chi)
1 16 -36239
2 17 -36237 1 5.6 0.01796 *
---
Signif. codes: 0 '***' 0.001 '**' 0.01 '*' 0.05 '.' 0.1 ' ' 1

### Main effect of Gender

main.A.final.drop.Gender<- glmmadmb(neg.b.ADAS~M+AGE+ APOE4+ edu.cat + diagn+ headache+ diclo+Ibu+ (1|ID), family="nbinom1", data=ADASdata)
anova(main.A.final.drop.Gender,main.A.final)

Analysis of Deviance Table

Model 1: neg.b.ADAS ~ M + AGE + APOE4 + edu.cat + diagn + headache + diclo + Ibu
Model 2: neg.b.ADAS ~ M + AGE + APOE4 + Gender + edu.cat + diagn + headache + diclo + Ibu
 NoPar LogLik Df Deviance Pr(>Chi)
1 16 -36241
2 17 -36237 1 8 0.004678 **
---
Signif. codes: 0 '***' 0.001 '**' 0.01 '*' 0.05 '.' 0.1 ' ' 1

### Main effect of Ibuprofen

main.A.final.drop.Ibuprofen<- glmmadmb(neg.b.ADAS~M+AGE+ APOE4+Gender+ edu.cat + diagn+ headache+ diclo+ (1|ID), family="nbinom1", data=ADASdata)
anova(main.A.final.drop.Ibuprofen,main.A.final)

Analysis of Deviance Table

Model 1: neg.b.ADAS ~ M + AGE + APOE4 + Gender + edu.cat + diagn + headache + diclo
Model 2: neg.b.ADAS ~ M + AGE + APOE4 + Gender + edu.cat + diagn + headache + diclo + Ibu
 NoPar LogLik Df Deviance Pr(>Chi)
1 16 -36242
2 17 -36237 1 11.4 0.0007344 ***
---
Signif. codes: 0 '***' 0.001 '**' 0.01 '*' 0.05 '.' 0.1 ' ' 1

### Main effect of Diclofenac

main.A.final.drop.Diclofenac<- glmmadmb(neg.b.ADAS~M+AGE+ APOE4+Gender+ edu.cat + diagn+ headache+ Ibu+ (1|ID), family="nbinom1", data=ADASdata)
anova(main.A.final.drop.Diclofenac,main.A.final)

Analysis of Deviance Table

Model 1: neg.b.ADAS ~ M + AGE + APOE4 + Gender + edu.cat + diagn + headache + Ibu
Model 2: neg.b.ADAS ~ M + AGE + APOE4 + Gender + edu.cat + diagn + headache + diclo + Ibu
 NoPar LogLik Df Deviance Pr(>Chi)
1 16 -36241
2 17 -36237 1 8.4 0.003752 **
---
Signif. codes: 0 '***' 0.001 '**' 0.01 '*' 0.05 '.' 0.1 ' ' 1

### AIC summary of main effect models

AIC(main.A.final,main.A.final.drop.AGE,main.A.final.drop.APOE4,main.A.final.drop.edu.cat,main.A.final.drop.diagn,main.A.final.drop.Diclofenac,main.A.final.drop.Ibuprofen,main.A.final.drop.Gender,main.A.final.drop.headaches)

df AIC
main.A.final 17 72507.2
main.A.final.drop.AGE 16 72578.4
main.A.final.drop.APOE4 15 72569.2
main.A.final.drop.edu.cat 14 72528.2
main.A.final.drop.diagn 14 73626.0
main.A.final.drop.Diclofenac 16 72513.6
main.A.final.drop.Ibuprofen 16 72516.6
main.A.final.drop.Gender 16 72513.2
main.A.final.drop.headaches 16 72510.8

## Investigating interaction terms

### The effects of diagnosis on cognitive decline progression

neg.a.interaction.diagn<- glmmadmb(neg.b.ADAS~M+AGE+ APOE4+Gender+ edu.cat + diagn+ headache+ diclo+Ibu+ diagn*M+(1|ID), family="nbinom1", data=ADASdata)
summary(neg.a.interaction.diagn)
anova(neg.a.interaction.diagn,main.A.final)

### The effects of smoking on cognitive decline progression

neg.a.interaction.smoke<- glmmadmb(neg.b.ADAS~M+AGE+ APOE4+Gender+ edu.cat + diagn+ headache+ diclo+Ibu+smoke*M+ (1|ID), family="nbinom1", data=ADASdata)
summary(neg.a.interaction.smoke)
anova(neg.a.interaction.smoke,main.A.final)

### The effects of arthritis on cognitive decline progression

neg.a.interaction.arthrit<- glmmadmb(neg.b.ADAS~M+AGE+ APOE4+Gender+ edu.cat + diagn+ headache+ diclo+Ibu+arthrit*M+ (1|ID), family="nbinom1", data=ADASdata)
summary(neg.a.interaction.arthrit)
anova(neg.a.interaction.arthrit, main.A.final)

### The effects of cardiovascular disease on cognitive decline progression

neg.a.interaction.vasc<- glmmadmb(neg.b.ADAS~M+AGE+ APOE4+Gender+ edu.cat + diagn+ headache+ diclo+Ibu+vasc*M+ (1|ID), family="nbinom1", data=ADASdata)
summary(neg.a.interaction.vasc)
anova(neg.a.interaction.vasc, main.A.final)

### The effect of headaches on cognitive decline progression

neg.a.interaction.headache<- glmmadmb(neg.b.ADAS~M+AGE+ APOE4+Gender+ edu.cat + diagn+ headache+ diclo+Ibu+headache*M+ (1|ID), family="nbinom1", data=ADASdata)
summary(neg.a.interaction.headache)

Call:
glmmadmb(formula = neg.b.ADAS ~ M + AGE + APOE4 + Gender + edu.cat +
 diagn + headache + diclo + Ibu + headache * M + (1 | ID),
 data = ADASdata, family = "nbinom1")

AIC: 72503.4

Coefficients:
 Estimate Std. Error z value Pr(>|z|)
(Intercept) 2.993245 0.030702 97.49 < 2e-16 ***
M 0.006837 0.000133 51.33 < 2e-16 ***
AGE 0.013457 0.001556 8.65 < 2e-16 ***
APOE41 0.163477 0.023662 6.91 4.9e-12 ***
APOE42 0.241777 0.037650 6.42 1.3e-10 ***
GenderMale 0.063623 0.022363 2.85 0.00444 **
edu.cat2tertiary 0.057155 0.027191 2.10 0.03556 *
edu.cat3mid 0.116559 0.030775 3.79 0.00015 ***
edu.cat4early 0.152558 0.032562 4.69 2.8e-06 ***
diagn2EMCI 0.325371 0.033565 9.69 < 2e-16 ***
diagn3LMCI 0.779868 0.028942 26.95 < 2e-16 ***
diagn4AD 1.281613 0.033667 38.07 < 2e-16 ***
headache -0.067821 0.040039 -1.69 0.09029 .
diclo -0.232237 0.080016 -2.90 0.00370 **
Ibu -0.100235 0.029613 -3.38 0.00071 ***
M:headache -0.000871 0.000363 -2.40 0.01628 *
---
Signif. codes: 0 '***' 0.001 '**' 0.01 '*' 0.05 '.' 0.1 ' ' 1

Number of observations: total=8770, ID=1618
Random effect variance(s):
Group=ID
 Variance StdDev
(Intercept) 0.1675 0.4093

Negative binomial dispersion parameter: 3.2997 (std. err.: 0.055949)

Log-likelihood: -36233.7

anova(neg.a.interaction.headache, main.A.final)

Analysis of Deviance Table

Model 1: neg.b.ADAS ~ M + AGE + APOE4 + Gender + edu.cat + diagn + headache + diclo + Ibu
Model 2: neg.b.ADAS ~ M + AGE + APOE4 + Gender + edu.cat + diagn + headache + diclo + Ibu + headache * M
 NoPar LogLik Df Deviance Pr(>Chi)
1 17 -36237
2 18 -36234 1 5.8 0.01603 *
---
Signif. codes: 0 '***' 0.001 '**' 0.01 '*' 0.05 '.' 0.1 ' ' 1

### The effect of diabetes on cognitive decline progression

neg.a.interaction.diabetes<- glmmadmb(neg.b.ADAS~M+AGE+ APOE4+Gender+ edu.cat + diagn+ headache+ diclo+Ibu+ diab*M+(1|ID), family="nbinom1", data=ADASdata)
summary(neg.a.interaction.headache)

Call:
glmmadmb(formula = neg.b.ADAS ~ M + AGE + APOE4 + Gender + edu.cat +
 diagn + headache + diclo + Ibu + headache * M + (1 | ID),
 data = ADASdata, family = "nbinom1")

AIC: 72503.4

Coefficients:
 Estimate Std. Error z value Pr(>|z|)
(Intercept) 2.993245 0.030702 97.49 < 2e-16 ***
M 0.006837 0.000133 51.33 < 2e-16 ***
AGE 0.013457 0.001556 8.65 < 2e-16 ***
APOE41 0.163477 0.023662 6.91 4.9e-12 ***
APOE42 0.241777 0.037650 6.42 1.3e-10 ***
GenderMale 0.063623 0.022363 2.85 0.00444 **
edu.cat2tertiary 0.057155 0.027191 2.10 0.03556 *
edu.cat3mid 0.116559 0.030775 3.79 0.00015 ***
edu.cat4early 0.152558 0.032562 4.69 2.8e-06 ***
diagn2EMCI 0.325371 0.033565 9.69 < 2e-16 ***
diagn3LMCI 0.779868 0.028942 26.95 < 2e-16 ***
diagn4AD 1.281613 0.033667 38.07 < 2e-16 ***
headache -0.067821 0.040039 -1.69 0.09029 .
diclo -0.232237 0.080016 -2.90 0.00370 **
Ibu -0.100235 0.029613 -3.38 0.00071 ***
M:headache -0.000871 0.000363 -2.40 0.01628 *
---
Signif. codes: 0 '***' 0.001 '**' 0.01 '*' 0.05 '.' 0.1 ' ' 1

Number of observations: total=8770, ID=1618
Random effect variance(s):
Group=ID
 Variance StdDev
(Intercept) 0.1675 0.4093

Negative binomial dispersion parameter: 3.2997 (std. err.: 0.055949)

Log-likelihood: -36233.7

anova(neg.a.interaction.diabetes, main.A.final)

Analysis of Deviance Table

Model 1: neg.b.ADAS ~ M + AGE + APOE4 + Gender + edu.cat + diagn + headache + diclo + Ibu
Model 2: neg.b.ADAS ~ M + AGE + APOE4 + Gender + edu.cat + diagn + headache + diclo + Ibu + diab * M
 NoPar LogLik Df Deviance Pr(>Chi)
1 17 -36237
2 19 -36236 2 1.8 0.4066

### The effect of AGE on cognitive decline progression

neg.a.interaction.AGE<- glmmadmb(neg.b.ADAS~M+AGE+ APOE4+Gender+ edu.cat + diagn+ headache+ diclo+Ibu+AGE*M+ (1|ID), family="nbinom1", data=ADASdata)
summary(neg.a.interaction.AGE)

Call:
glmmadmb(formula = neg.b.ADAS ~ M + AGE + APOE4 + Gender + edu.cat +
 diagn + headache + diclo + Ibu + AGE * M + (1 | ID), data = ADASdata,
 family = "nbinom1")

AIC: 72508.2

Coefficients:
 Estimate Std. Error z value Pr(>|z|)
(Intercept) 3.00e+00 3.07e-02 97.65 < 2e-16 ***
M 6.73e-03 1.24e-04 54.10 < 2e-16 ***
AGE 1.38e-02 1.60e-03 8.62 < 2e-16 ***
APOE41 1.63e-01 2.37e-02 6.90 5.1e-12 ***
APOE42 2.43e-01 3.77e-02 6.44 1.2e-10 ***
GenderMale 6.31e-02 2.24e-02 2.82 0.00479 **
edu.cat2tertiary 5.70e-02 2.72e-02 2.10 0.03595 *
edu.cat3mid 1.17e-01 3.08e-02 3.79 0.00015 ***
edu.cat4early 1.53e-01 3.26e-02 4.69 2.8e-06 ***
diagn2EMCI 3.25e-01 3.36e-02 9.68 < 2e-16 ***
diagn3LMCI 7.79e-01 2.90e-02 26.92 < 2e-16 ***
diagn4AD 1.28e+00 3.37e-02 38.03 < 2e-16 ***
headache -9.20e-02 3.88e-02 -2.37 0.01782 *
diclo -2.32e-01 8.00e-02 -2.90 0.00374 **
Ibu -9.98e-02 2.96e-02 -3.37 0.00075 ***
M:AGE -1.80e-05 1.88e-05 -0.96 0.33760
---
Signif. codes: 0 '***' 0.001 '**' 0.01 '*' 0.05 '.' 0.1 ' ' 1

Number of observations: total=8770, ID=1618
Random effect variance(s):
Group=ID
 Variance StdDev
(Intercept) 0.1676 0.4094

Negative binomial dispersion parameter: 3.3016 (std. err.: 0.055987)

Log-likelihood: -36236.1

anova(neg.a.interaction.AGE, main.A.final)

Analysis of Deviance Table

Model 1: neg.b.ADAS ~ M + AGE + APOE4 + Gender + edu.cat + diagn + headache + diclo + Ibu
Model 2: neg.b.ADAS ~ M + AGE + APOE4 + Gender + edu.cat + diagn + headache + diclo + Ibu + AGE * M
 NoPar LogLik Df Deviance Pr(>Chi)
1 17 -36237
2 18 -36236 1 1 0.3173

### The effect of APOE status on cognitive decline progression

neg.a.interaction.APOE4<- glmmadmb(neg.b.ADAS~M+AGE+ APOE4+Gender+ edu.cat + diagn+ headache+ diclo+Ibu+ APOE4*M+(1|ID), family="nbinom1", data=ADASdata)
summary(neg.a.interaction.APOE4)

Call:
glmmadmb(formula = neg.b.ADAS ~ M + AGE + APOE4 + Gender + edu.cat +
 diagn + headache + diclo + Ibu + APOE4 * M + (1 | ID), data = ADASdata,
 family = "nbinom1")

AIC: 72285.8

Coefficients:
 Estimate Std. Error z value Pr(>|z|)
(Intercept) 3.036500 0.030693 98.93 < 2e-16 ***
M 0.005142 0.000165 31.22 < 2e-16 ***
AGE 0.013391 0.001552 8.63 < 2e-16 ***
APOE41 0.093169 0.024278 3.84 0.00012 ***
APOE42 0.145675 0.038410 3.79 0.00015 ***
GenderMale 0.062943 0.022310 2.82 0.00478 **
edu.cat2tertiary 0.056311 0.027127 2.08 0.03791 *
edu.cat3mid 0.116544 0.030702 3.80 0.00015 ***
edu.cat4early 0.151799 0.032486 4.67 3e-06 ***
diagn2EMCI 0.318188 0.033479 9.50 < 2e-16 ***
diagn3LMCI 0.773176 0.028872 26.78 < 2e-16 ***
diagn4AD 1.284425 0.033580 38.25 < 2e-16 ***
headache -0.089277 0.038721 -2.31 0.02113 *
diclo -0.227894 0.079816 -2.86 0.00430 **
Ibu -0.095656 0.029545 -3.24 0.00121 **
M:APOE41 0.003174 0.000260 12.20 < 2e-16 ***
M:APOE42 0.005050 0.000437 11.56 < 2e-16 ***
---
Signif. codes: 0 '***' 0.001 '**' 0.01 '*' 0.05 '.' 0.1 ' ' 1

Number of observations: total=8770, ID=1618
Random effect variance(s):
Group=ID
 Variance StdDev
(Intercept) 0.1671 0.4088

Negative binomial dispersion parameter: 3.2043 (std. err.: 0.054319)

Log-likelihood: -36123.9

anova(neg.a.interaction.APOE4, main.A.final)

Analysis of Deviance Table

Model 1: neg.b.ADAS ~ M + AGE + APOE4 + Gender + edu.cat + diagn + headache + diclo + Ibu
Model 2: neg.b.ADAS ~ M + AGE + APOE4 + Gender + edu.cat + diagn + headache + diclo + Ibu + APOE4 * M
 NoPar LogLik Df Deviance Pr(>Chi)
1 17 -36237
2 19 -36124 2 225.4 < 2.2e-16 ***
---
Signif. codes: 0 '***' 0.001 '**' 0.01 '*' 0.05 '.' 0.1 ' ' 1

### The effect of education status on cognitive decline progression

neg.a.interaction.edu.cat<- glmmadmb(neg.b.ADAS~M+AGE+ APOE4+Gender+ edu.cat + diagn+ headache+ diclo+Ibu+edu.cat*M+ (1|ID), family="nbinom1", data=ADASdata)
summary(neg.a.interaction.edu.cat)

Call:
glmmadmb(formula = neg.b.ADAS ~ M + AGE + APOE4 + Gender + edu.cat +
 diagn + headache + diclo + Ibu + edu.cat * M + (1 | ID),
 data = ADASdata, family = "nbinom1")

AIC: 72478

Coefficients:
 Estimate Std. Error z value Pr(>|z|)
(Intercept) 2.978833 0.031011 96.06 < 2e-16 ***
M 0.007460 0.000211 35.31 < 2e-16 ***
AGE 0.013451 0.001559 8.63 < 2e-16 ***
APOE41 0.164254 0.023701 6.93 4.2e-12 ***
APOE42 0.241581 0.037713 6.41 1.5e-10 ***
GenderMale 0.062936 0.022400 2.81 0.00496 **
edu.cat2tertiary 0.098538 0.028138 3.50 0.00046 ***
edu.cat3mid 0.131083 0.031805 4.12 3.8e-05 ***
edu.cat4early 0.166818 0.033436 4.99 6.1e-07 ***
diagn2EMCI 0.325769 0.033618 9.69 < 2e-16 ***
diagn3LMCI 0.779189 0.028994 26.87 < 2e-16 ***
diagn4AD 1.279701 0.033719 37.95 < 2e-16 ***
headache -0.091994 0.038874 -2.37 0.01796 *
diclo -0.232956 0.080146 -2.91 0.00365 **
Ibu -0.099232 0.029661 -3.35 0.00082 ***
M:edu.cat2tertiary -0.001848 0.000315 -5.86 4.5e-09 ***
M:edu.cat3mid -0.000620 0.000342 -1.81 0.06958 .
M:edu.cat4early -0.000565 0.000358 -1.58 0.11476
---
Signif. codes: 0 '***' 0.001 '**' 0.01 '*' 0.05 '.' 0.1 ' ' 1

Number of observations: total=8770, ID=1618
Random effect variance(s):
Group=ID
 Variance StdDev
(Intercept) 0.1682 0.4101

Negative binomial dispersion parameter: 3.2834 (std. err.: 0.055665)

Log-likelihood: -36219

anova(neg.a.interaction.edu.cat, main.A.final)

Analysis of Deviance Table

Model 1: neg.b.ADAS ~ M + AGE + APOE4 + Gender + edu.cat + diagn + headache + diclo + Ibu
Model 2: neg.b.ADAS ~ M + AGE + APOE4 + Gender + edu.cat + diagn + headache + diclo + Ibu + edu.cat * M
 NoPar LogLik Df Deviance Pr(>Chi)
1 17 -36237
2 20 -36219 3 35.2 1.105e-07 ***
---
Signif. codes: 0 '***' 0.001 '**' 0.01 '*' 0.05 '.' 0.1 ' ' 1

### The effect of diabetes on cognitive decline progression

neg.a.interaction.diab<- glmmadmb(neg.b.ADAS~M+AGE+ APOE4+Gender+ edu.cat + diagn+ headache+ diclo+Ibu+diab*M+ (1|ID), family="nbinom1", data=ADASdata)
summary(neg.a.interaction.diab)

Call:
glmmadmb(formula = neg.b.ADAS ~ M + AGE + APOE4 + Gender + edu.cat +
 diagn + headache + diclo + Ibu + diab * M + (1 | ID), data = ADASdata,
 family = "nbinom1")

AIC: 72509.4

Coefficients:
 Estimate Std. Error z value Pr(>|z|)
(Intercept) 2.994485 0.030713 97.50 < 2e-16 ***
M 0.006769 0.000130 52.04 < 2e-16 ***
AGE 0.013466 0.001557 8.65 < 2e-16 ***
APOE41 0.163370 0.023661 6.90 5.0e-12 ***
APOE42 0.242948 0.037675 6.45 1.1e-10 ***
GenderMale 0.062491 0.022447 2.78 0.00537 **
edu.cat2tertiary 0.056087 0.027268 2.06 0.03970 *
edu.cat3mid 0.115475 0.030797 3.75 0.00018 ***
edu.cat4early 0.151322 0.032637 4.64 3.5e-06 ***
diagn2EMCI 0.325245 0.033583 9.68 < 2e-16 ***
diagn3LMCI 0.779731 0.028940 26.94 < 2e-16 ***
diagn4AD 1.281152 0.033664 38.06 < 2e-16 ***
headache -0.091288 0.038813 -2.35 0.01867 *
diclo -0.231672 0.080023 -2.90 0.00379 **
Ibu -0.100332 0.029612 -3.39 0.00070 ***
diab 0.029840 0.038418 0.78 0.43732
M:diab -0.000534 0.000431 -1.24 0.21544
---
Signif. codes: 0 '***' 0.001 '**' 0.01 '*' 0.05 '.' 0.1 ' ' 1

Number of observations: total=8770, ID=1618
Random effect variance(s):
Group=ID
 Variance StdDev
(Intercept) 0.1675 0.4093

Negative binomial dispersion parameter: 3.3019 (std. err.: 0.055983)

Log-likelihood: -36235.7

anova(neg.a.interaction.diab, main.A.final)

Analysis of Deviance Table

Model 1: neg.b.ADAS ~ M + AGE + APOE4 + Gender + edu.cat + diagn + headache + diclo + Ibu
Model 2: neg.b.ADAS ~ M + AGE + APOE4 + Gender + edu.cat + diagn + headache + diclo + Ibu + diab * M
 NoPar LogLik Df Deviance Pr(>Chi)
1 17 -36237
2 19 -36236 2 1.8 0.4066

### The effect of Gender on cognitive decline progression

neg.a.interaction.Gender<- glmmadmb(neg.b.ADAS~M+AGE+ APOE4+Gender+ edu.cat + diagn+ headache+ diclo+Ibu+Gender*M+ (1|ID), family="nbinom1", data=ADASdata)
summary(neg.a.interaction.Gender)

Call:
glmmadmb(formula = neg.b.ADAS ~ M + AGE + APOE4 + Gender + edu.cat +
 diagn + headache + diclo + Ibu + Gender * M + (1 | ID), data = ADASdata,
 family = "nbinom1")

AIC: 72452

Coefficients:
 Estimate Std. Error z value Pr(>|z|)
(Intercept) 2.970505 0.030931 96.04 < 2e-16 ***
M 0.007830 0.000191 41.03 < 2e-16 ***
AGE 0.013341 0.001559 8.56 < 2e-16 ***
APOE41 0.163579 0.023712 6.90 5.3e-12 ***
APOE42 0.242345 0.037731 6.42 1.3e-10 ***
GenderMale 0.104766 0.023072 4.54 5.6e-06 ***
edu.cat2tertiary 0.056269 0.027248 2.07 0.03892 *
edu.cat3mid 0.114572 0.030841 3.71 0.00020 ***
edu.cat4early 0.153623 0.032631 4.71 2.5e-06 ***
diagn2EMCI 0.326624 0.033633 9.71 < 2e-16 ***
diagn3LMCI 0.781496 0.029002 26.95 < 2e-16 ***
diagn4AD 1.283412 0.033736 38.04 < 2e-16 ***
headache -0.091613 0.038893 -2.36 0.01850 *
diclo -0.228988 0.080184 -2.86 0.00429 **
Ibu -0.101246 0.029676 -3.41 0.00065 ***
M:GenderMale -0.001884 0.000248 -7.58 3.3e-14 ***
---
Signif. codes: 0 '***' 0.001 '**' 0.01 '*' 0.05 '.' 0.1 ' ' 1

Number of observations: total=8770, ID=1618
Random effect variance(s):
Group=ID
 Variance StdDev
(Intercept) 0.1684 0.4104

Negative binomial dispersion parameter: 3.2728 (std. err.: 0.055469)

Log-likelihood: -36208

anova(neg.a.interaction.Gender, main.A.final)

Analysis of Deviance Table

Model 1: neg.b.ADAS ~ M + AGE + APOE4 + Gender + edu.cat + diagn + headache + diclo + Ibu
Model 2: neg.b.ADAS ~ M + AGE + APOE4 + Gender + edu.cat + diagn + headache + diclo + Ibu + Gender * M
 NoPar LogLik Df Deviance Pr(>Chi)
1 17 -36237
2 18 -36208 1 57.2 3.941e-14 ***
---
Signif. codes: 0 '***' 0.001 '**' 0.01 '*' 0.05 '.' 0.1 ' ' 1

### The effect of aspirin on cognitive decline progression

neg.a.interaction.aspirin<- glmmadmb(neg.b.ADAS~M+AGE+ APOE4+Gender+ edu.cat + diagn+ headache+ diclo+Ibu+aspirin*M+ (1|ID), family="nbinom1", data=ADASdata)
summary(neg.a.interaction.aspirin)

Call:
glmmadmb(formula = neg.b.ADAS ~ M + AGE + APOE4 + Gender + edu.cat +
 diagn + headache + diclo + Ibu + aspirin * M + (1 | ID),
 data = ADASdata, family = "nbinom1")

AIC: 72498.8

Coefficients:
 Estimate Std. Error z value Pr(>|z|)
(Intercept) 2.994179 0.033049 90.60 < 2e-16 ***
M 0.007378 0.000229 32.28 < 2e-16 ***
AGE 0.013553 0.001560 8.69 < 2e-16 ***
APOE41 0.162889 0.023693 6.87 6.2e-12 ***
APOE42 0.243206 0.037704 6.45 1.1e-10 ***
GenderMale 0.066751 0.022572 2.96 0.00310 **
edu.cat2tertiary 0.056277 0.027225 2.07 0.03872 *
edu.cat3mid 0.115801 0.030812 3.76 0.00017 ***
edu.cat4early 0.151793 0.032624 4.65 3.3e-06 ***
diagn2EMCI 0.324355 0.033607 9.65 < 2e-16 ***
diagn3LMCI 0.778754 0.029013 26.84 < 2e-16 ***
diagn4AD 1.280139 0.033940 37.72 < 2e-16 ***
headache -0.092640 0.038860 -2.38 0.01713 *
diclo -0.226726 0.080206 -2.83 0.00470 **
Ibu -0.099038 0.029653 -3.34 0.00084 ***
aspirin -0.001704 0.022635 -0.08 0.94001
M:aspirin -0.000919 0.000270 -3.40 0.00068 ***
---
Signif. codes: 0 '***' 0.001 '**' 0.01 '*' 0.05 '.' 0.1 ' ' 1

Number of observations: total=8770, ID=1618
Random effect variance(s):
Group=ID
 Variance StdDev
(Intercept) 0.168 0.4099

Negative binomial dispersion parameter: 3.2943 (std. err.: 0.055868)

Log-likelihood: -36230.4

anova(neg.a.interaction.aspirin, main.A.final)

Analysis of Deviance Table

Model 1: neg.b.ADAS ~ M + AGE + APOE4 + Gender + edu.cat + diagn + headache + diclo + Ibu
Model 2: neg.b.ADAS ~ M + AGE + APOE4 + Gender + edu.cat + diagn + headache + diclo + Ibu + aspirin * M
 NoPar LogLik Df Deviance Pr(>Chi)
1 17 -36237
2 19 -36230 2 12.4 0.002029 **
---
Signif. codes: 0 '***' 0.001 '**' 0.01 '*' 0.05 '.' 0.1 ' ' 1

### The effect of paracetamol on cognitive decline progression

neg.a.interaction.parac<- glmmadmb(neg.b.ADAS~M+AGE+ APOE4+Gender+ edu.cat + diagn+ headache+ diclo+Ibu+parac*M+ (1|ID), family="nbinom1", data=ADASdata)
summary(neg.a.interaction.parac)

Call:
glmmadmb(formula = neg.b.ADAS ~ M + AGE + APOE4 + Gender + edu.cat +
 diagn + headache + diclo + Ibu + parac * M + (1 | ID), data = ADASdata,
 family = "nbinom1")

AIC: 72502.6

Coefficients:
 Estimate Std. Error z value Pr(>|z|)
(Intercept) 3.007526 0.031457 95.61 < 2e-16 ***
M 0.006457 0.000156 41.32 < 2e-16 ***
AGE 0.013482 0.001557 8.66 < 2e-16 ***
APOE41 0.162151 0.023657 6.85 7.2e-12 ***
APOE42 0.240711 0.037615 6.40 1.6e-10 ***
GenderMale 0.061826 0.022395 2.76 0.00577 **
edu.cat2tertiary 0.057689 0.027172 2.12 0.03374 *
edu.cat3mid 0.116911 0.030766 3.80 0.00014 ***
edu.cat4early 0.151943 0.032529 4.67 3.0e-06 ***
diagn2EMCI 0.325530 0.033531 9.71 < 2e-16 ***
diagn3LMCI 0.779367 0.028911 26.96 < 2e-16 ***
diagn4AD 1.278976 0.033657 38.00 < 2e-16 ***
headache -0.089595 0.038890 -2.30 0.02123 *
diclo -0.231049 0.079991 -2.89 0.00387 **
Ibu -0.097793 0.029744 -3.29 0.00101 **
parac -0.037224 0.025918 -1.44 0.15094
M:parac 0.000717 0.000255 2.81 0.00489 **
---
Signif. codes: 0 '***' 0.001 '**' 0.01 '*' 0.05 '.' 0.1 ' ' 1

Number of observations: total=8770, ID=1618
Random effect variance(s):
Group=ID
 Variance StdDev
(Intercept) 0.1671 0.4088

Negative binomial dispersion parameter: 3.3002 (std. err.: 0.055953)

Log-likelihood: -36232.3

anova(neg.a.interaction.parac, main.A.final)

Analysis of Deviance Table

Model 1: neg.b.ADAS ~ M + AGE + APOE4 + Gender + edu.cat + diagn + headache + diclo + Ibu
Model 2: neg.b.ADAS ~ M + AGE + APOE4 + Gender + edu.cat + diagn + headache + diclo + Ibu + parac * M
 NoPar LogLik Df Deviance Pr(>Chi)
1 17 -36237
2 19 -36232 2 8.6 0.01357 *
---
Signif. codes: 0 '***' 0.001 '**' 0.01 '*' 0.05 '.' 0.1 ' ' 1

### The effect of diclofenac on cognitive decline progression

neg.a.interaction.diclo<- glmmadmb(neg.b.ADAS~M+AGE+ APOE4+Gender+ edu.cat + diagn+ headache+ diclo+Ibu+diclo*M+ (1|ID), family="nbinom1", data=ADASdata)
summary(neg.a.interaction.diclo)

Call:
glmmadmb(formula = neg.b.ADAS ~ M + AGE + APOE4 + Gender + edu.cat +
 diagn + headache + diclo + Ibu + diclo * M + (1 | ID), data = ADASdata,
 family = "nbinom1")

AIC: 72507.6

Coefficients:
 Estimate Std. Error z value Pr(>|z|)
(Intercept) 2.995474 0.030681 97.63 < 2e-16 ***
M 0.006750 0.000126 53.45 < 2e-16 ***
AGE 0.013436 0.001556 8.63 < 2e-16 ***
APOE41 0.163209 0.023661 6.90 5.3e-12 ***
APOE42 0.242229 0.037648 6.43 1.2e-10 ***
GenderMale 0.063397 0.022362 2.84 0.00458 **
edu.cat2tertiary 0.056963 0.027191 2.09 0.03618 *
edu.cat3mid 0.116438 0.030774 3.78 0.00015 ***
edu.cat4early 0.152731 0.032562 4.69 2.7e-06 ***
diagn2EMCI 0.325482 0.033565 9.70 < 2e-16 ***
diagn3LMCI 0.779719 0.028941 26.94 < 2e-16 ***
diagn4AD 1.281247 0.033666 38.06 < 2e-16 ***
headache -0.091821 0.038808 -2.37 0.01798 *
diclo -0.203363 0.083081 -2.45 0.01437 *
Ibu -0.100150 0.029612 -3.38 0.00072 ***
M:diclo -0.000876 0.000693 -1.26 0.20636
---
Signif. codes: 0 '***' 0.001 '**' 0.01 '*' 0.05 '.' 0.1 ' ' 1

Number of observations: total=8770, ID=1618
Random effect variance(s):
Group=ID
 Variance StdDev
(Intercept) 0.1675 0.4093

Negative binomial dispersion parameter: 3.302 (std. err.: 0.055985)

Log-likelihood: -36235.8

anova(neg.a.interaction.diclo, main.A.final)

Analysis of Deviance Table

Model 1: neg.b.ADAS ~ M + AGE + APOE4 + Gender + edu.cat + diagn + headache + diclo + Ibu
Model 2: neg.b.ADAS ~ M + AGE + APOE4 + Gender + edu.cat + diagn + headache + diclo + Ibu + diclo * M
 NoPar LogLik Df Deviance Pr(>Chi)
1 17 -36237
2 18 -36236 1 1.6 0.2059

### The effect of ibuprofen on cognitive decline progression

neg.a.interaction.Ibu<- glmmadmb(neg.b.ADAS~M+AGE+ APOE4+Gender+ edu.cat + diagn+ headache+ diclo+Ibu+Ibu*M+ (1|ID), family="nbinom1", data=ADASdata)
summary(neg.a.interaction.Ibu)

Call:
glmmadmb(formula = neg.b.ADAS ~ M + AGE + APOE4 + Gender + edu.cat +
 diagn + headache + diclo + Ibu + Ibu * M + (1 | ID), data = ADASdata,
 family = "nbinom1")

AIC: 72508.6

Coefficients:
 Estimate Std. Error z value Pr(>|z|)
(Intercept) 2.995124 0.030707 97.54 < 2e-16 ***
M 0.006771 0.000140 48.32 < 2e-16 ***
AGE 0.013451 0.001556 8.64 < 2e-16 ***
APOE41 0.163245 0.023662 6.90 5.2e-12 ***
APOE42 0.242099 0.037648 6.43 1.3e-10 ***
GenderMale 0.063190 0.022362 2.83 0.00472 **
edu.cat2tertiary 0.057114 0.027191 2.10 0.03569 *
edu.cat3mid 0.116645 0.030774 3.79 0.00015 ***
edu.cat4early 0.152618 0.032562 4.69 2.8e-06 ***
diagn2EMCI 0.325426 0.033565 9.70 < 2e-16 ***
diagn3LMCI 0.779600 0.028941 26.94 < 2e-16 ***
diagn4AD 1.281303 0.033668 38.06 < 2e-16 ***
headache -0.091849 0.038808 -2.37 0.01795 *
diclo -0.232437 0.080014 -2.90 0.00367 **
Ibu -0.093796 0.030663 -3.06 0.00222 **
M:Ibu -0.000230 0.000299 -0.77 0.44217
---
Signif. codes: 0 '***' 0.001 '**' 0.01 '*' 0.05 '.' 0.1 ' ' 1

Number of observations: total=8770, ID=1618
Random effect variance(s):
Group=ID
 Variance StdDev
(Intercept) 0.1675 0.4093

Negative binomial dispersion parameter: 3.3025 (std. err.: 0.055993)

Log-likelihood: -36236.3

anova(neg.a.interaction.Ibu, main.A.final)

Analysis of Deviance Table

Model 1: neg.b.ADAS ~ M + AGE + APOE4 + Gender + edu.cat + diagn + headache + diclo + Ibu
Model 2: neg.b.ADAS ~ M + AGE + APOE4 + Gender + edu.cat + diagn + headache + diclo + Ibu + Ibu * M
 NoPar LogLik Df Deviance Pr(>Chi)
1 17 -36237
2 18 -36236 1 0.6 0.4386

### The effect of naproxin on cognitive decline progression

neg.a.interaction.naprox<- glmmadmb(neg.b.ADAS~M+AGE+ APOE4+Gender+ edu.cat + diagn+ headache+ diclo+Ibu+naprox*M (1|ID), family="nbinom1", data=ADASdata)
summary(neg.a.interaction.naprox)

Call:
glmmadmb(formula = neg.b.ADAS ~ M + AGE + APOE4 + Gender + edu.cat +
 diagn + headache + diclo + Ibu + naprox * M(1 | ID), data = ADASdata,
 family = "nbinom1")

AIC: 72504.4

Coefficients:
 Estimate Std. Error z value Pr(>|z|)
(Intercept) 3.001528 0.030899 97.14 < 2e-16 ***
M 0.006575 0.000137 48.00 < 2e-16 ***
AGE 0.013424 0.001555 8.63 < 2e-16 ***
APOE41 0.163477 0.023635 6.92 4.6e-12 ***
APOE42 0.241305 0.037608 6.42 1.4e-10 ***
GenderMale 0.062568 0.022355 2.80 0.00513 **
edu.cat2tertiary 0.057595 0.027180 2.12 0.03409 *
edu.cat3mid 0.116517 0.030749 3.79 0.00015 ***
edu.cat4early 0.152924 0.032531 4.70 2.6e-06 ***
diagn2EMCI 0.326133 0.033534 9.73 < 2e-16 ***
diagn3LMCI 0.779605 0.028908 26.97 < 2e-16 ***
diagn4AD 1.278919 0.033677 37.98 < 2e-16 ***
headache -0.091607 0.038768 -2.36 0.01813 *
diclo -0.231299 0.079934 -2.89 0.00381 **
Ibu -0.098405 0.029666 -3.32 0.00091 ***
naprox -0.039186 0.034797 -1.13 0.26010
M:naprox 0.000824 0.000322 2.56 0.01038 *
---
Signif. codes: 0 '***' 0.001 '**' 0.01 '*' 0.05 '.' 0.1 ' ' 1

Number of observations: total=8770, ID=1618
Random effect variance(s):
Group=ID
 Variance StdDev
(Intercept) 0.1671 0.4088

Negative binomial dispersion parameter: 3.3011 (std. err.: 0.055973)

Log-likelihood: -36233.2

anova(neg.a.interaction.naprox, main.A.final)

Analysis of Deviance Table

Model 1: neg.b.ADAS ~ M + AGE + APOE4 + Gender + edu.cat + diagn + headache + diclo + Ibu
Model 2: neg.b.ADAS ~ M + AGE + APOE4 + Gender + edu.cat + diagn + headache + diclo + Ibu + naprox * M
 NoPar LogLik Df Deviance Pr(>Chi)
1 17 -36237
2 19 -36233 2 6.8 0.03337 *
---
Signif. codes: 0 '***' 0.001 '**' 0.01 '*' 0.05 '.' 0.1 ' ' 1

### The effect of celecoxib on cognitive decline progression

neg.a.interaction.celex<- glmmadmb(neg.b.ADAS~M+AGE+ APOE4+Gender+ edu.cat + diagn+ headache+ diclo+Ibu+celex*M (1|ID), family="nbinom1", data=ADASdata)
summary(neg.a.interaction.celex)

Call:
glmmadmb(formula = neg.b.ADAS ~ M + AGE + APOE4 + Gender + edu.cat +
 diagn + headache + diclo + Ibu + celex * M(1 | ID), data = ADASdata,
 family = "nbinom1")

AIC: 72510.8

Coefficients:
 Estimate Std. Error z value Pr(>|z|)
(Intercept) 2.995508 0.030797 97.27 < 2e-16 ***
M 0.006740 0.000128 52.66 < 2e-16 ***
AGE 0.013440 0.001556 8.64 < 2e-16 ***
APOE41 0.163354 0.023659 6.90 5.0e-12 ***
APOE42 0.242367 0.037648 6.44 1.2e-10 ***
GenderMale 0.063225 0.022361 2.83 0.00469 **
edu.cat2tertiary 0.056902 0.027190 2.09 0.03637 *
edu.cat3mid 0.116466 0.030771 3.78 0.00015 ***
edu.cat4early 0.152566 0.032572 4.68 2.8e-06 ***
diagn2EMCI 0.325535 0.033564 9.70 < 2e-16 ***
diagn3LMCI 0.779680 0.028940 26.94 < 2e-16 ***
diagn4AD 1.281279 0.033704 38.02 < 2e-16 ***
headache -0.091745 0.038882 -2.36 0.01830 *
diclo -0.232037 0.080047 -2.90 0.00375 **
Ibu -0.099851 0.029654 -3.37 0.00076 ***
celex 0.013661 0.056682 0.24 0.80955
M:celex -0.000327 0.000525 -0.62 0.53293
---
Signif. codes: 0 '***' 0.001 '**' 0.01 '*' 0.05 '.' 0.1 ' ' 1

Number of observations: total=8770, ID=1618
Random effect variance(s):
Group=ID
 Variance StdDev
(Intercept) 0.1675 0.4093

Negative binomial dispersion parameter: 3.3026 (std. err.: 0.055996)

Log-likelihood: -36236.4

anova(neg.a.interaction.celex, main.A.final)

Analysis of Deviance Table

Model 1: neg.b.ADAS ~ M + AGE + APOE4 + Gender + edu.cat + diagn + headache + diclo + Ibu
Model 2: neg.b.ADAS ~ M + AGE + APOE4 + Gender + edu.cat + diagn + headache + diclo + Ibu + celex * M
 NoPar LogLik Df Deviance Pr(>Chi)
1 17 -36237
2 19 -36236 2 0.4 0.8187

## Combined interaction model

neg.a.combined<- glmmadmb(neg.b.ADAS~M+AGE+ APOE4+Gender+ edu.cat + diagn+ headache+ diclo+Ibu+ edu.cat*M+ smoke*M+diagn*M+APOE4*M+Gender*M+parac*M+diclo*M+ (1|ID), family="nbinom1", data=ADASdata)
summary(neg.a.combined)

Call:
glmmadmb(formula = neg.b.ADAS ~ M + AGE + APOE4 + Gender + edu.cat +
 diagn + headache + diclo + Ibu + edu.cat * M + smoke * M +
 diagn * M + APOE4 * M + Gender * M + parac * M + diclo *
 M + (1 | ID), data = ADASdata, family = "nbinom1")

AIC: 72128.2

Coefficients:
 Estimate Std. Error z value Pr(>|z|)
(Intercept) 2.989645 0.032762 91.25 < 2e-16 ***
M 0.007308 0.000347 21.04 < 2e-16 ***
AGE 0.013325 0.001562 8.53 < 2e-16 ***
APOE41 0.095188 0.024426 3.90 9.7e-05 ***
APOE42 0.148257 0.038673 3.83 0.00013 ***
GenderMale 0.105252 0.023297 4.52 6.2e-06 ***
edu.cat2tertiary 0.104051 0.028158 3.70 0.00022 ***
edu.cat3mid 0.148367 0.031898 4.65 3.3e-06 ***
edu.cat4early 0.179550 0.033490 5.36 8.3e-08 ***
diagn2EMCI 0.358696 0.035137 10.21 < 2e-16 ***
diagn3LMCI 0.771943 0.030022 25.71 < 2e-16 ***
diagn4AD 1.248909 0.034818 35.87 < 2e-16 ***
headache -0.087011 0.039034 -2.23 0.02581 *
diclo -0.187060 0.083036 -2.25 0.02427 *
Ibu -0.094288 0.029861 -3.16 0.00159 **
smoke 0.007713 0.025548 0.30 0.76274
parac -0.036288 0.025980 -1.40 0.16249
M:edu.cat2tertiary -0.002151 0.000309 -6.96 3.4e-12 ***
M:edu.cat3mid -0.001548 0.000349 -4.44 9.0e-06 ***
M:edu.cat4early -0.001315 0.000362 -3.63 0.00028 ***
M:smoke 0.000325 0.000274 1.19 0.23532
M:diagn2EMCI -0.001647 0.000410 -4.02 5.9e-05 ***
M:diagn3LMCI 0.000199 0.000287 0.69 0.48877
M:diagn4AD 0.004435 0.000727 6.10 1.1e-09 ***
M:APOE41 0.002988 0.000263 11.34 < 2e-16 ***
M:APOE42 0.004855 0.000451 10.77 < 2e-16 ***
M:GenderMale -0.002095 0.000256 -8.19 2.6e-16 ***
M:parac 0.000598 0.000252 2.38 0.01748 *
M:diclo -0.001178 0.000681 -1.73 0.08382 .
---
Signif. codes: 0 '***' 0.001 '**' 0.01 '*' 0.05 '.' 0.1 ' ' 1

Number of observations: total=8770, ID=1618
Random effect variance(s):
Group=ID
 Variance StdDev
(Intercept) 0.1693 0.4114

Negative binomial dispersion parameter: 3.1173 (std. err.: 0.052809)

Log-likelihood: -36033.1

anova(neg.a.combined, main.A.final)

Analysis of Deviance Table

Model 1: neg.b.ADAS ~ M + AGE + APOE4 + Gender + edu.cat + diagn + headache + diclo + Ibu
Model 2: neg.b.ADAS ~ M + AGE + APOE4 + Gender + edu.cat + diagn + headache + diclo + Ibu + edu.cat * M + smoke * M + diagn * M + APOE4 * M + Gender * M + parac * M + diclo * M
 NoPar LogLik Df Deviance Pr(>Chi)
1 17 -36237
2 31 -36033 14 407 < 2.2e-16 ***
---
Signif. codes: 0 '***' 0.001 '**' 0.01 '*' 0.05 '.' 0.1 ' ' 1

### Dropping non-significant interactions

neg.a.combined.drop.smoke<- glmmadmb(neg.b.ADAS~M+AGE+ APOE4+Gender+ edu.cat + diagn+ headache+ diclo+Ibu+ edu.cat*M+ diagn*M+APOE4*M+Gender*M+parac*M+diclo*M+ (1|ID), family="nbinom1", data=ADASdata)
summary(neg.a.combined.drop.smoke)

Call:
glmmadmb(formula = neg.b.ADAS ~ M + AGE + APOE4 + Gender + edu.cat +
 diagn + headache + diclo + Ibu + edu.cat * M + diagn * M +
 APOE4 * M + Gender * M + parac * M + diclo * M + (1 | ID),
 data = ADASdata, family = "nbinom1")

AIC: 72126

Coefficients:
 Estimate Std. Error z value Pr(>|z|)
(Intercept) 2.990323 0.032490 92.04 < 2e-16 ***
M 0.007399 0.000339 21.82 < 2e-16 ***
AGE 0.013308 0.001562 8.52 < 2e-16 ***
APOE41 0.095546 0.024425 3.91 9.2e-05 ***
APOE42 0.147989 0.038648 3.83 0.00013 ***
GenderMale 0.106309 0.023148 4.59 4.4e-06 ***
edu.cat2tertiary 0.104703 0.028119 3.72 0.00020 ***
edu.cat3mid 0.148963 0.031845 4.68 2.9e-06 ***
edu.cat4early 0.180123 0.033464 5.38 7.3e-08 ***
diagn2EMCI 0.358418 0.035118 10.21 < 2e-16 ***
diagn3LMCI 0.772093 0.030023 25.72 < 2e-16 ***
diagn4AD 1.248836 0.034817 35.87 < 2e-16 ***
headache -0.087237 0.039035 -2.23 0.02543 *
diclo -0.186690 0.083038 -2.25 0.02456 *
Ibu -0.094001 0.029855 -3.15 0.00164 **
parac -0.035660 0.025975 -1.37 0.16980
M:edu.cat2tertiary -0.002141 0.000309 -6.93 4.3e-12 ***
M:edu.cat3mid -0.001521 0.000348 -4.37 1.2e-05 ***
M:edu.cat4early -0.001295 0.000362 -3.58 0.00034 ***
M:diagn2EMCI -0.001670 0.000410 -4.08 4.6e-05 ***
M:diagn3LMCI 0.000175 0.000286 0.61 0.54034
M:diagn4AD 0.004423 0.000727 6.08 1.2e-09 ***
M:APOE41 0.002978 0.000263 11.31 < 2e-16 ***
M:APOE42 0.004830 0.000450 10.72 < 2e-16 ***
M:GenderMale -0.002079 0.000255 -8.14 3.9e-16 ***
M:parac 0.000586 0.000252 2.33 0.01979 *
M:diclo -0.001185 0.000681 -1.74 0.08212 .
---
Signif. codes: 0 '***' 0.001 '**' 0.01 '*' 0.05 '.' 0.1 ' ' 1

Number of observations: total=8770, ID=1618
Random effect variance(s):
Group=ID
 Variance StdDev
(Intercept) 0.1693 0.4115

Negative binomial dispersion parameter: 3.1181 (std. err.: 0.05282)

Log-likelihood: -36034

anova(neg.a.combined, neg.a.combined.drop.smoke)

Analysis of Deviance Table

Model 1: neg.b.ADAS ~ M + AGE + APOE4 + Gender + edu.cat + diagn + headache + diclo + Ibu + edu.cat * M + diagn * M + APOE4 * M + Gender * M + parac * M + diclo * M
Model 2: neg.b.ADAS ~ M + AGE + APOE4 + Gender + edu.cat + diagn + headache + diclo + Ibu + edu.cat * M + diagn * M + APOE4 * M + Gender * M + parac * M + diclo * M
 NoPar LogLik Df Deviance Pr(>Chi)
1 28 -36036
2 29 -36033 1 3 0.08326

neg.a.combined.drop.diclo<- glmmadmb(neg.b.ADAS~M+AGE+ APOE4+Gender+ edu.cat + diagn+ headache+ diclo+Ibu+ edu.cat*M+ diagn*M+APOE4*M+Gender*M+parac*M+ (1|ID), family="nbinom1", data=ADASdata)
summary(neg.a.combined.drop.diclo)

Call:
glmmadmb(formula = neg.b.ADAS ~ M + AGE + APOE4 + Gender + edu.cat +
 diagn + headache + diclo + Ibu + edu.cat * M + diagn * M +
 APOE4 * M + Gender * M + parac * M + (1 | ID), data = ADASdata,
 family = "nbinom1")

AIC: 72127

Coefficients:
 Estimate Std. Error z value Pr(>|z|)
(Intercept) 2.991271 0.032483 92.09 < 2e-16 ***
M 0.007364 0.000339 21.75 < 2e-16 ***
AGE 0.013316 0.001562 8.52 < 2e-16 ***
APOE41 0.095729 0.024424 3.92 8.9e-05 ***
APOE42 0.149163 0.038638 3.86 0.00011 ***
GenderMale 0.106536 0.023147 4.60 4.2e-06 ***
edu.cat2tertiary 0.104722 0.028117 3.72 0.00020 ***
edu.cat3mid 0.148404 0.031842 4.66 3.2e-06 ***
edu.cat4early 0.180975 0.033458 5.41 6.3e-08 ***
diagn2EMCI 0.357465 0.035112 10.18 < 2e-16 ***
diagn3LMCI 0.771174 0.030017 25.69 < 2e-16 ***
diagn4AD 1.247515 0.034807 35.84 < 2e-16 ***
headache -0.087110 0.039032 -2.23 0.02563 *
diclo -0.224372 0.080266 -2.80 0.00518 **
Ibu -0.093702 0.029852 -3.14 0.00170 **
parac -0.035014 0.025971 -1.35 0.17759
M:edu.cat2tertiary -0.002137 0.000309 -6.91 4.7e-12 ***
M:edu.cat3mid -0.001490 0.000348 -4.29 1.8e-05 ***
M:edu.cat4early -0.001348 0.000361 -3.74 0.00019 ***
M:diagn2EMCI -0.001629 0.000409 -3.98 6.8e-05 ***
M:diagn3LMCI 0.000210 0.000286 0.74 0.46177
M:diagn4AD 0.004483 0.000726 6.17 6.8e-10 ***
M:APOE41 0.002980 0.000263 11.31 < 2e-16 ***
M:APOE42 0.004757 0.000448 10.61 < 2e-16 ***
M:GenderMale -0.002098 0.000255 -8.22 < 2e-16 ***
M:parac 0.000555 0.000251 2.21 0.02703 *
---
Signif. codes: 0 '***' 0.001 '**' 0.01 '*' 0.05 '.' 0.1 ' ' 1

Number of observations: total=8770, ID=1618
Random effect variance(s):
Group=ID
 Variance StdDev
(Intercept) 0.1693 0.4114

Negative binomial dispersion parameter: 3.1196 (std. err.: 0.052844)

Log-likelihood: -36035.5

anova(neg.a.combined, neg.a.combined.drop.diclo)

Analysis of Deviance Table

Model 1: neg.b.ADAS ~ M + AGE + APOE4 + Gender + edu.cat + diagn + headache + diclo + Ibu + edu.cat * M + diagn * M + APOE4 * M + Gender * M + parac * M
Model 2: neg.b.ADAS ~ M + AGE + APOE4 + Gender + edu.cat + diagn + headache + diclo + Ibu + edu.cat * M + smoke * M + diagn * M + APOE4 * M + Gender * M + parac * M + diclo * M
 NoPar LogLik Df Deviance Pr(>Chi)
1 28 -36036
2 31 -36033 3 4.8 0.187

## Final full model and plots of the coeffecients

The full model of all significant interaction terms is now created, and non-significant terms will be removed.

neg.ADAS<- glmmadmb(neg.b.ADAS~M+AGE+ APOE4+Gender+ edu.cat + diagn+ headache+ diclo+Ibu+ edu.cat*M+diagn*M+APOE4*M+Gender*M+parac*M+ (1|ID), family="nbinom1", data=ADASdata)

summary(neg.ADAS)

Call:
glmmadmb(formula = neg.b.ADAS ~ M + AGE + APOE4 + Gender + edu.cat +
 diagn + headache + diclo + Ibu + edu.cat * M + diagn * M +
 APOE4 * M + Gender * M + parac * M + (1 | ID), data = ADASdata,
 family = "nbinom1")

AIC: 72127

Coefficients:
 Estimate Std. Error z value Pr(>|z|)
(Intercept) 2.991271 0.032483 92.09 < 2e-16 ***
M 0.007364 0.000339 21.75 < 2e-16 ***
AGE 0.013316 0.001562 8.52 < 2e-16 ***
APOE41 0.095729 0.024424 3.92 8.9e-05 ***
APOE42 0.149163 0.038638 3.86 0.00011 ***
GenderMale 0.106536 0.023147 4.60 4.2e-06 ***
edu.cat2tertiary 0.104722 0.028117 3.72 0.00020 ***
edu.cat3mid 0.148404 0.031842 4.66 3.2e-06 ***
edu.cat4early 0.180975 0.033458 5.41 6.3e-08 ***
diagn2EMCI 0.357465 0.035112 10.18 < 2e-16 ***
diagn3LMCI 0.771174 0.030017 25.69 < 2e-16 ***
diagn4AD 1.247515 0.034807 35.84 < 2e-16 ***
headache -0.087110 0.039032 -2.23 0.02563 *
diclo -0.224372 0.080266 -2.80 0.00518 **
Ibu -0.093702 0.029852 -3.14 0.00170 **
parac -0.035014 0.025971 -1.35 0.17759
M:edu.cat2tertiary -0.002137 0.000309 -6.91 4.7e-12 ***
M:edu.cat3mid -0.001490 0.000348 -4.29 1.8e-05 ***
M:edu.cat4early -0.001348 0.000361 -3.74 0.00019 ***
M:diagn2EMCI -0.001629 0.000409 -3.98 6.8e-05 ***
M:diagn3LMCI 0.000210 0.000286 0.74 0.46177
M:diagn4AD 0.004483 0.000726 6.17 6.8e-10 ***
M:APOE41 0.002980 0.000263 11.31 < 2e-16 ***
M:APOE42 0.004757 0.000448 10.61 < 2e-16 ***
M:GenderMale -0.002098 0.000255 -8.22 < 2e-16 ***
M:parac 0.000555 0.000251 2.21 0.02703 *
---
Signif. codes: 0 '***' 0.001 '**' 0.01 '*' 0.05 '.' 0.1 ' ' 1

Number of observations: total=8770, ID=1618
Random effect variance(s):
Group=ID
 Variance StdDev
(Intercept) 0.1693 0.4114

Negative binomial dispersion parameter: 3.1196 (std. err.: 0.052844)

Log-likelihood: -36035.5

anova(neg.a.combined, neg.ADAS)

Analysis of Deviance Table

Model 1: neg.b.ADAS ~ M + AGE + APOE4 + Gender + edu.cat + diagn + headache + diclo + Ibu + edu.cat * M + diagn * M + APOE4 * M + Gender * M + parac * M
Model 2: neg.b.ADAS ~ M + AGE + APOE4 + Gender + edu.cat + diagn + headache + diclo + Ibu + edu.cat * M + smoke * M + diagn * M + APOE4 * M + Gender * M + parac * M + diclo * M
 NoPar LogLik Df Deviance Pr(>Chi)
1 28 -36036
2 31 -36033 3 4.8 0.187

### Coeffecient plot

coefplot(neg.ADAS)

Figure description: Plot of the coefficients with standard error (small lines) and 95% confidence intervals (larger lines), for the final selected model.

### Coeffecient plot of interaction terms

coefficients<-c("M:parac","M:APOE41","M:APOE42","M:edu.cat2tertiary","M:edu.cat3mid","M:edu.cat4early","M:diagn2EMCI","M:diagn3LMCI","M:diagn4AD","M")
coefplot(neg.ADAS,coefficients=coefficients,main="Interaction terms coeffecients")

Figure description: Plot of the coefficients of the interaction terms with standard error (small lines) and 95% confidence intervals (larger lines), for the final selected model.

## Dropping terms of the model to evaluate the significance of each variable in the ful model.

No.Age.ADAS<- glmmadmb(neg.b.ADAS~M+APOE4+Gender+ edu.cat + diagn+ headache+ diclo+Ibu+ edu.cat*M+ diagn*M+APOE4*M+Gender*M+parac*M+ (1|ID), family="nbinom1", data=ADASdata)


No.APOE4.ADAS<- glmmadmb(neg.b.ADAS~M+AGE+ Gender+ edu.cat + diagn+ headache+ diclo+Ibu+ edu.cat*M+ diagn*M+Gender*M+parac*M+ (1|ID), family="nbinom1", data=ADASdata)

APOE4.ADAS.Main<- glmmadmb(neg.b.ADAS~M+AGE+ APOE4+Gender+ edu.cat + diagn+ headache+ diclo+Ibu+ edu.cat*M+ diagn*M+Gender*M+parac*M+ (1|ID), family="nbinom1", data=ADASdata)

Education.ADAS.Main<- glmmadmb(neg.b.ADAS~M+AGE+ APOE4+Gender+ edu.cat + diagn+ headache+ diclo+Ibu+ diagn*M+APOE4*M+Gender*M+parac*M+ (1|ID), family="nbinom1", data=ADASdata)

No.Education.ADAS<- glmmadmb(neg.b.ADAS~M+AGE+ APOE4+Gender+ diagn+ headache+ diclo+Ibu+ diagn*M+APOE4*M+Gender*M+parac*M+ (1|ID), family="nbinom1", data=ADASdata)

Diagn.ADAS.Main<- glmmadmb(neg.b.ADAS~M+AGE+ APOE4+Gender+ edu.cat + diagn+ headache+ diclo+Ibu+ edu.cat*M+APOE4*M+Gender*M+parac*M+ (1|ID), family="nbinom1", data=ADASdata)

No.Diagn.ADAS<- glmmadmb(neg.b.ADAS~M+AGE+ APOE4+Gender+ edu.cat + headache+ diclo+Ibu+ edu.cat*M+APOE4*M+Gender*M+parac*M+ (1|ID), family="nbinom1", data=ADASdata)

Gender.ADAS.Main<- glmmadmb(neg.b.ADAS~M+AGE+ APOE4+Gender+ edu.cat + diagn+ headache+ diclo+Ibu+ edu.cat*M+ diagn*M+APOE4*M+parac*M+ (1|ID), family="nbinom1", data=ADASdata)

No.Gender.ADAS<- glmmadmb(neg.b.ADAS~M+AGE+ APOE4+ edu.cat + diagn+ headache+ diclo+Ibu+ edu.cat*M+ diagn*M+APOE4*M+parac*M+ (1|ID), family="nbinom1", data=ADASdata)

No.Paracet.ADAS<- glmmadmb(neg.b.ADAS~M+AGE+ APOE4+Gender+ edu.cat + diagn+ headache+ diclo+Ibu+ edu.cat*M+ diagn*M+APOE4*M+Gender*M+ (1|ID), family="nbinom1", data=ADASdata)

Paracet.ADAS.Main<- glmmadmb(neg.b.ADAS~parac+M+AGE+ APOE4+Gender+ edu.cat + diagn+ headache+ diclo+Ibu+ edu.cat*M+ diagn*M+APOE4*M+Gender*M+ (1|ID), family="nbinom1", data=ADASdata)

No.Diclofen.ADAS<- glmmadmb(neg.b.ADAS~M+AGE+ APOE4+Gender+ edu.cat + diagn+ headache+ Ibu+ edu.cat*M+ diagn*M+APOE4*M+Gender*M+parac*M+(1|ID), family="nbinom1", data=ADASdata)

No.Headache.ADAS<- glmmadmb(neg.b.ADAS~M+AGE+ APOE4+Gender+ edu.cat + diagn+ diclo+Ibu+ edu.cat*M+ diagn*M+APOE4*M+Gender*M+parac*M+ (1|ID), family="nbinom1", data=ADASdata)

No.Ibuprofen.ADAS<- glmmadmb(neg.b.ADAS~M+AGE+ APOE4+Gender+ edu.cat + diagn+ headache+ diclo+edu.cat*M+ diagn*M+APOE4*M+Gender*M+parac*M+ (1|ID), family="nbinom1", data=ADASdata)

summary(neg.ADAS)

Call:
glmmadmb(formula = neg.b.ADAS ~ M + AGE + APOE4 + Gender + edu.cat +
 diagn + headache + diclo + Ibu + edu.cat * M + diagn * M +
 APOE4 * M + Gender * M + parac * M + (1 | ID), data = ADASdata,
 family = "nbinom1")

AIC: 72127

Coefficients:
 Estimate Std. Error z value Pr(>|z|)
(Intercept) 2.991271 0.032483 92.09 < 2e-16 ***
M 0.007364 0.000339 21.75 < 2e-16 ***
AGE 0.013316 0.001562 8.52 < 2e-16 ***
APOE41 0.095729 0.024424 3.92 8.9e-05 ***
APOE42 0.149163 0.038638 3.86 0.00011 ***
GenderMale 0.106536 0.023147 4.60 4.2e-06 ***
edu.cat2tertiary 0.104722 0.028117 3.72 0.00020 ***
edu.cat3mid 0.148404 0.031842 4.66 3.2e-06 ***
edu.cat4early 0.180975 0.033458 5.41 6.3e-08 ***
diagn2EMCI 0.357465 0.035112 10.18 < 2e-16 ***
diagn3LMCI 0.771174 0.030017 25.69 < 2e-16 ***
diagn4AD 1.247515 0.034807 35.84 < 2e-16 ***
headache -0.087110 0.039032 -2.23 0.02563 *
diclo -0.224372 0.080266 -2.80 0.00518 **
Ibu -0.093702 0.029852 -3.14 0.00170 **
parac -0.035014 0.025971 -1.35 0.17759
M:edu.cat2tertiary -0.002137 0.000309 -6.91 4.7e-12 ***
M:edu.cat3mid -0.001490 0.000348 -4.29 1.8e-05 ***
M:edu.cat4early -0.001348 0.000361 -3.74 0.00019 ***
M:diagn2EMCI -0.001629 0.000409 -3.98 6.8e-05 ***
M:diagn3LMCI 0.000210 0.000286 0.74 0.46177
M:diagn4AD 0.004483 0.000726 6.17 6.8e-10 ***
M:APOE41 0.002980 0.000263 11.31 < 2e-16 ***
M:APOE42 0.004757 0.000448 10.61 < 2e-16 ***
M:GenderMale -0.002098 0.000255 -8.22 < 2e-16 ***
M:parac 0.000555 0.000251 2.21 0.02703 *
---
Signif. codes: 0 '***' 0.001 '**' 0.01 '*' 0.05 '.' 0.1 ' ' 1

Number of observations: total=8770, ID=1618
Random effect variance(s):
Group=ID
 Variance StdDev
(Intercept) 0.1693 0.4114

Negative binomial dispersion parameter: 3.1196 (std. err.: 0.052844)

Log-likelihood: -36035.5

Model<-c("Main effect of Age", "Main effect of APOE4", "Main effect of Education", "Main effect of Diagnosis","Main effect of Gender", "Main effect of Diclofenac","Main effect of Headache","Main effect of Ibuprofen","Main effect of Paracetamol","Interaction effect of Education","Interaction effect of Diagnosis","Interaction effect of APOE4","Interaction effect of Gender","Interaction effect of Paracetamol")
M.age<-anova(neg.ADAS, No.Age.ADAS)
M.APOE4<-anova(APOE4.ADAS.Main, No.APOE4.ADAS)
M.Education<-anova(No.Education.ADAS, Education.ADAS.Main)
M.Diagn<-anova(Diagn.ADAS.Main, No.Diagn.ADAS)
M.Gender<-anova(No.Gender.ADAS, Gender.ADAS.Main)
M.Diclofenac<-anova(neg.ADAS, No.Diclofen.ADAS)
M.Headache<-anova(neg.ADAS, No.Headache.ADAS)
M.Ibuprofen<-anova(neg.ADAS, No.Ibuprofen.ADAS)
M.Paracetamol<-anova(Paracet.ADAS.Main, No.Paracet.ADAS)


I.Education<-anova(neg.ADAS, Education.ADAS.Main)
I.Diagnosis<-anova(neg.ADAS, Diagn.ADAS.Main)
I.APOE4<-anova(neg.ADAS, APOE4.ADAS.Main)
I.Gender<-anova(neg.ADAS, Gender.ADAS.Main)
I.Paracetamol<-anova(neg.ADAS, Paracet.ADAS.Main)


ADAS.PValue<-rbind(M.age[2,3:5],M.APOE4[2,3:5],M.Education[2,3:5],M.Diagn[2,3:5],M.Gender[2,3:5],M.Diclofenac[2,3:5],M.Headache[2,3:5],M.Ibuprofen[2,3:5],M.Paracetamol[2,3:5],I.Education[2,3:5],I.Diagnosis[2,3:5],I.APOE4[2,3:5],I.Gender[2,3:5],I.Paracetamol[2,3:5])

ADAS.Final.model.dataframe<-data.frame(Model,ADAS.PValue)
ADAS.model.final<-neg.ADAS
print(ADAS.Final.model.dataframe)

Model Df Deviance Pr..Chi.
2 Main effect of Age 1 71.4 0.000000e+00
21 Main effect of APOE4 2 63.6 1.543210e-14
22 Main effect of Education 3 26.2 8.660640e-06
23 Main effect of Diagnosis 3 1124.4 0.000000e+00
24 Main effect of Gender 1 7.4 6.522388e-03
25 Main effect of Diclofenac 1 7.8 5.224623e-03
26 Main effect of Headache 1 5.0 2.534732e-02
27 Main effect of Ibuprofen 1 9.8 1.745119e-03
28 Main effect of Paracetamol 1 0.8 3.710934e-01
29 Interaction effect of Education 3 50.6 5.952605e-11
210 Interaction effect of Diagnosis 3 65.4 4.118927e-14
211 Interaction effect of APOE4 2 186.2 0.000000e+00
212 Interaction effect of Gender 1 67.0 2.220446e-16
213 Interaction effect of Paracetamol 1 4.8 2.845974e-02

print(summary(ADAS.model.final))

Call:
glmmadmb(formula = neg.b.ADAS ~ M + AGE + APOE4 + Gender + edu.cat +
 diagn + headache + diclo + Ibu + edu.cat * M + diagn * M +
 APOE4 * M + Gender * M + parac * M + (1 | ID), data = ADASdata,
 family = "nbinom1")

AIC: 72127

Coefficients:
 Estimate Std. Error z value Pr(>|z|)
(Intercept) 2.991271 0.032483 92.09 < 2e-16 ***
M 0.007364 0.000339 21.75 < 2e-16 ***
AGE 0.013316 0.001562 8.52 < 2e-16 ***
APOE41 0.095729 0.024424 3.92 8.9e-05 ***
APOE42 0.149163 0.038638 3.86 0.00011 ***
GenderMale 0.106536 0.023147 4.60 4.2e-06 ***
edu.cat2tertiary 0.104722 0.028117 3.72 0.00020 ***
edu.cat3mid 0.148404 0.031842 4.66 3.2e-06 ***
edu.cat4early 0.180975 0.033458 5.41 6.3e-08 ***
diagn2EMCI 0.357465 0.035112 10.18 < 2e-16 ***
diagn3LMCI 0.771174 0.030017 25.69 < 2e-16 ***
diagn4AD 1.247515 0.034807 35.84 < 2e-16 ***
headache -0.087110 0.039032 -2.23 0.02563 *
diclo -0.224372 0.080266 -2.80 0.00518 **
Ibu -0.093702 0.029852 -3.14 0.00170 **
parac -0.035014 0.025971 -1.35 0.17759
M:edu.cat2tertiary -0.002137 0.000309 -6.91 4.7e-12 ***
M:edu.cat3mid -0.001490 0.000348 -4.29 1.8e-05 ***
M:edu.cat4early -0.001348 0.000361 -3.74 0.00019 ***
M:diagn2EMCI -0.001629 0.000409 -3.98 6.8e-05 ***
M:diagn3LMCI 0.000210 0.000286 0.74 0.46177
M:diagn4AD 0.004483 0.000726 6.17 6.8e-10 ***
M:APOE41 0.002980 0.000263 11.31 < 2e-16 ***
M:APOE42 0.004757 0.000448 10.61 < 2e-16 ***
M:GenderMale -0.002098 0.000255 -8.22 < 2e-16 ***
M:parac 0.000555 0.000251 2.21 0.02703 *
---
Signif. codes: 0 '***' 0.001 '**' 0.01 '*' 0.05 '.' 0.1 ' ' 1

Number of observations: total=8770, ID=1618
Random effect variance(s):
Group=ID
 Variance StdDev
(Intercept) 0.1693 0.4114

Negative binomial dispersion parameter: 3.1196 (std. err.: 0.052844)

Log-likelihood: -36035.5

## Evaluating the progession and main-effects of each pain medication

neg.a.base<- glmmadmb(neg.b.ADAS~ AGE + APOE4 + M + edu.cat + diagn+ headache+ diagn*M +APOE4*M + Gender*M+ edu.cat*M + (1|ID), family="nbinom1", data=ADASdata)

neg.a.M.aspirin<- glmmadmb(neg.b.ADAS~ AGE + APOE4 + M + edu.cat + diagn+ headache+ diagn*M +APOE4*M + Gender*M+ edu.cat*M + aspirin+ (1|ID), family="nbinom1", data=ADASdata)
neg.a.M.celecoxib<- glmmadmb(neg.b.ADAS~ AGE + APOE4 + M + edu.cat + diagn+ headache+ diagn*M +APOE4*M + Gender*M+ edu.cat*M + celex+ (1|ID), family="nbinom1", data=ADASdata)
neg.a.M.diclofenac<- glmmadmb(neg.b.ADAS~ AGE + APOE4 + M + edu.cat + diagn+ headache+ diagn*M +APOE4*M + Gender*M+ edu.cat*M +diclo+ (1|ID), family="nbinom1", data=ADASdata)
neg.a.M.ibuprofen<- glmmadmb(neg.b.ADAS~ AGE + APOE4 + M + edu.cat + diagn+ headache+ diagn*M +APOE4*M + Gender*M+ edu.cat*M +Ibu+ (1|ID), family="nbinom1", data=ADASdata)
neg.a.M.naproxen<- glmmadmb(neg.b.ADAS~ AGE + APOE4 + M + edu.cat + diagn+ headache+ diagn*M +APOE4*M + Gender*M+ edu.cat*M +naprox+ (1|ID), family="nbinom1", data=ADASdata)
neg.a.M.paracetamol<- glmmadmb(neg.b.ADAS~ AGE + APOE4 + M + edu.cat + diagn+ headache+ diagn*M +APOE4*M + Gender*M+ edu.cat*M + parac+ (1|ID), family="nbinom1", data=ADASdata)
neg.a.I.aspirin<- glmmadmb(neg.b.ADAS~ AGE + APOE4 + M + edu.cat + diagn+ headache+ diagn*M +APOE4*M + Gender*M+ edu.cat*M +aspirin*M+ (1|ID), family="nbinom1", data=ADASdata)
neg.a.I.celecoxib<- glmmadmb(neg.b.ADAS~ AGE + APOE4 + M + edu.cat + diagn+ headache+ diagn*M +APOE4*M + Gender*M+ edu.cat*M +celex*M+ (1|ID), family="nbinom1", data=ADASdata)
[truncated: 154,519 more chars]
