# Supplementary material for: Anti-inflammatories in Alzheimer’s disease—potential therapy or spurious correlate?
Source: Brain Commun. 2020 Jul 24;2(2):fcaa109. doi: 10.1093/braincomms/fcaa109 (PMC7585697; doi:10.1093/braincomms/fcaa109)
Supplement: fcaa109_Supplementary_Data [file Original submission Decision Letter.pdf › Original submission Decision Letter.pdf]

Original submission Decision Letter

15-Apr-2020

Dear Dr. Rivers-Auty,

Thank you for your recent submission BRAINCOM-2020-049
entitled "Anti-inflammatories in Alzheimer's disease - potential therapy or spurious correlate?" to Brain Communications.

Your manuscript has been reviewed, and while the reviewers had largely positive comments, they have a few constructive critiques that I think you could address in a minor revision of the manuscript. Therefore, I invite you to respond to the reviewers' comments and revise your manuscript. The reviewers' comments are included at the bottom of this message. The editorial team agrees that your statistical approach is sound. Based on reviewer 2's comments, you may be able to clarify a few things for non-epidemiologist readers.

In addition to the reviewers' comments, please note our editorial comments added below to comply with our requirements for presentation of scientific data at Brain Communications.

Please also note that for the final accepted paper, we will require an abbreviated summary of no more than 50 words and a graphical abstract. Please include these in your revision.

Please format your revised manuscript according to our Instructions to Authors https://academic.oup.com/braincomms/pages/General_Instructions – a summary of key points is attached to help with this.

To revise your manuscript, click the link below or log into https://mc.manuscriptcentral.com/braincom and enter your Author Centre, where you will find your manuscript title listed under "Manuscripts with Decisions." Under "Actions," click on "Create a Revision." Your manuscript number has been appended to denote a revision.

*** PLEASE NOTE: This is a two-step process. After clicking on the link, you will be directed to a webpage to confirm. ***

https://mc.manuscriptcentral.com/braincom?URL_MASK=256ef6c8abf140129f5a92497d1efdcd

Please upload a revised manuscript with the changes you have made shown either with "track changes" or in coloured text.

When submitting your revised manuscript, you will be asked to respond to the reviewer(s) comments. Please be as specific as possible in your response to all of the comments.

The editorial team at Brain Communications also aims to increase transparency in scientific publishing and to demonstrate the value that peer review brings to the scientific process. Please therefore consider whether you are willing to have reviewer comments on your paper and your response published as supplementary data with the paper if it is accepted. This is entirely optional and will only be done if both the authors and reviewers agree. When submitting your revision you will have the opportunity to update the answer you entered previously.

IMPORTANT: Your original files are available to you when you upload your revised manuscript. Please delete any redundant files before completing the submission.

Because we are trying to facilitate timely publication of manuscripts submitted to Brain Communications, we would appreciate your revised manuscript as soon as possible. If it is not possible for you to resubmit within 3 months, the option to submit a revision will expire, and we would then consider your revised paper as a new submission. However, if you need a little more time, please get in touch and we can extend this deadline for you.

Once again, thank you for submitting your manuscript to Brain Communications and I look forward to receiving your revision.

Yours sincerely,
Prof. Karen Ritchie
Editor, Brain Communications

Reviewer(s)' Comments to Author:

Reviewer: 1

Comments to the Author
This manuscript describes a very important advance in our understanding of the role of inflammation in the risk of AD and in its progression. Previous epidemiological work had suggested that use of NSAIDS, or at least the need for and use of them by people with rheumatoid arthritis, led to a reduced risk of subsequently developing AD. However intervention studies in both AD and MCI failed to show any NSAID to significantly slow progression. The current study used innovative statistical methods and the ADNI database to assess whether use of NSAIDS or of paracetamol was associated with NC, AD or MCI diagnosis or with progression. The data are convincing that NSAIDS are associated with reduced prevalence but not reduced decline. The fact that these drugs have are structurally and functionally different suggested to the authors that this association may be due to confounding features of the populations (ie healthy user bias) and not to the anti-inflammatory effects of the drugs. Interestingly, this research identified diclofenac use was significantly associated with both reduced incidence and slower decline. In addition to its research findings, the study provides an in depth analysis of the literature on the anti-inflammatory drug approach to treating AD, which can inform future trials.

Reviewer: 2 Jie Yeap

Comments to the Author
This article describes a GLMM study using the ADNI case controlled longitudinal study dataset. The authors have carefully selected the most appropriate distribution for model construction, accounting for multiple common AD risk factors. This study is timely and important as some recent evidence suggest the potential of anti-inflammatory drugs at altering AD progression.

In the authors' negative binomial model using to investigate the progression of cognitive decline (based on MMSE and ADAS scores) between users and non-users of NSAID, the main finding of this study is that only diclofenac delayed cognitive decline, but not other commonly consumed NSAIDs. The authors also suggested that the lower AD prevalence amongst NSAID users could be due to healthy user bias. The results generated hypothesis for diclofenac mechanism/target studies. The study is interesting and can be improved if the following considerations are addressed:

1. It may be beneficial for readers to gain more information about the methods utilised within the abstract. The authors could give a brief outline of the GLMMs they have applied.

2. The authors should clarify whether SMC subjects recruited in ADNI2 were considered in this study.

3. It was mentioned that the dataset were downloaded in May 2018, and that the follow up duration of the subjects was up to 120 months depending on the recruitment stage. It is unclear whether t=120-month data were collected for all subjects as this may affect the n at later time points of the study and perhaps the power of the model.

4. Some results reporting seemed to be slightly contradictory.

For example, in Baseline Statistics, the authors described celecoxib, diclofenac, ibuprofen and naproxen users had substantially reduced AD prevalence; while aspirin and paracetamol users experienced moderately reduced prevalence. Later in Discussion, it was reported that all pain-relievers showed similar reductions in AD diagnosis. The authors should clarify whether there are statistically significant differences in AD prevalence between groups of NSAID users and the control, in addition to Chi-squared tests.

It is reported that diclofenac had a positive effect on MMSE & ADAS scores in the model as a main effect, however, the p-value of the statistics of inclusion for diclofenac in the MMSE table is p=0.317311.

The statistics for diclofenac in the ADAS table (interaction with time) was not shown.

5. It is unclear whether the authors implemented any correction for multiple testing. Running numerous analyses but just considering an uncorrected $p<0.05$ as significant is likely to introduce type 1 errors.

6. Readers might be interested to find out which significant explanatory variables and two-way interaction terms that were included in the final model and which were dropped.

7. It is interesting to see that diclofenac users showed the highest proportion of cognitively normal individuals. The authors could comment on this observation - whether this is spurious or a potential

prophylactic action of the drug.

8. With the in-depth description in Methods & Supplementary sections, the authors might consider including the final models in the main Methods section to make the both the analysis and results easier to interpret.

9. There are minor typos in Supplement section - particularly 'explanatory, diagnostic, corresponding, reliever', etc.

10. It would be good to give meaningful titles / very brief legends to the figures in Supplement section.

Editorial Office comments:
-Please supply a graphical abstract. A graphical abstract is a visual representation of the central finding or methodology of the paper. It will be displayed in the table of contents. Authors should strive to make the graphical abstract informative, interesting, visually appealing, and straightforward (no caption needed, no abbreviations except gene names). Please upload as a separate high resolution image file (tiff preferred). The technical requirements for the graphical abstracts are:

- size of the submitted image: 1200 pixels square at 300dpi
- font of the text: Arial 12-16 point (smaller fonts will not be legible online)

-Please supply an abbreviated summary of up to 50 words (no abbreviations except gene names). This will be used in the table of contents alongside the graphical abstract. Please upload as a separate word document.
- Please include a paragraph about statistical analysis in the 'Materials and Methods' section.
-The appropriate checklist must be included (see Reporting Guideline section within 'Instructions to authors').
