# Supplementary material for: Anti-inflammatories in Alzheimer’s disease—potential therapy or spurious correlate?
Source: Brain Commun. 2020 Jul 24;2(2):fcaa109. doi: 10.1093/braincomms/fcaa109 (PMC7585697; doi:10.1093/braincomms/fcaa109)
Supplement: fcaa109_Supplementary_Data [file Original_Submission_response_to_reviewer.pdf › Original_Submission_response_to_reviewer.pdf]

1. It may be beneficial for readers to gain more information about the methods utilised within the abstract. The authors could give a brief outline of the GLMMs they have applied.

   Response: This is an excellent suggestion given that these statistical methods are a novel approach to cognitive decline analyses for Alzheimer's research and are thus and important contribution to the field, we completely agree that these should be summarized in the abstract and have updated the abstract as such.

2. The authors should clarify whether SMC subjects recruited in ADNI2 were considered in this study.

Response: SMC subjects were removed due to this group likely being very variable because of the subjective nature of self-diagnosis. Thank you to the reviewer for picking up that this wasn't clear from our methods. We have updated our methods in the manuscript to make this explicitly clear.

3. It was mentioned that the dataset were downloaded in May 2018, and that the follow up duration of the subjects was up to 120 months depending on the recruitment stage. It is unclear whether t=120-month data were collected for all subjects as this may affect the n at later time points of the study and perhaps the power of the model.

Response: Thank you for this suggestion. We have now analysed drop-out and completeness of the data over time. From this we found that those who started the study on any of the drugs analysed were less likely to drop-out (due to very poor health, death or other). This again supports the healthy user bias conclusion, as healthier subjects were more likely to remain in the study. We repeated our progression analyses over 48 months, as over this time period there was limited drop-out. This analysis came up with very similar results to the main study. Supporting the conclusions of the study and suggesting that drop-out was not affecting the analyses (likely due to the use of maximum likelihood approaches which handle missing data very well, unlike least-squares methods).

4. Some results reporting seemed to be slightly contradictory.

For example, in Baseline Statistics, the authors described celecoxib, diclofenac, ibuprofen and naproxen users had substantially reduced AD prevalence; while aspirin and paracetamol users experienced moderately reduced prevalence. Later in Discussion, it was reported that all pain-relievers showed similar reductions in AD diagnosis. The authors should clarify whether there are statistically significant differences in AD prevalence between groups of NSAID users and the control, in addition to Chi-squared tests.

Thank you for this critique. We have completed a thorough prevalence analysis using logistic regression adjusting for nuisance variables, and found that there is no significant difference between pain relievers and that all pain relievers have lower prevalence than the no pain relief group (supplement 6.4.4.1 and 6.4.4.2). We have included these results and methods in the study and amended our language appropriately.

It is reported that diclofenac had a positive effect on MMSE & ADAS scores in the model as a main effect, however, the p-value of the statistics of inclusion for diclofenac in the MMSE table is p=0.317311.
This misunderstanding is due to our writing not being clear. When diclofenac was included in the model, **solely** as a main effect with no other explanatory variables, it was significant. This can be seen in supplement 7.8.11. However, once we included the explanatory variables and interaction term with time, this main effect became non-significant. Suggesting that diclofenac's predominate effect is on the decline (slope) and not as a main effect (although any potential main effect might be masked by the inclusion of the diagnosis variable and this would be picked up by changes in prevalence of each cognitive diagnosis). We completely understand that this was a confusing statement and we have amended our language to be more explicit.

The statistics for diclofenac in the ADAS table (interaction with time) was not shown.
This is because it did not reach significance and only our final model was shown. We have reported it in the supplement 8.17.

5. It is unclear whether the authors implemented any correction for multiple testing. Running numerous analyses but just considering an uncorrected $p<0.05$ as significant is likely to introduce type 1 errors.
Thank you for the suggestion. We have now described multiple comparison correction in the methods and mentioned the possibility of type 1 errors in the discussion.

6. Readers might be interested to find out which significant explanatory variables and two-way interaction terms that were included in the final model and which were dropped.
The tables reported are the final model. Therefore, all variables in the methods that are not in the final tables were dropped. This is discussed in the result section where we mention all the included and dropped variables. All the details of inclusion and exclusion with associated p-values are in the supplement.

7. It is interesting to see that diclofenac users showed the highest proportion of cognitively normal individuals. The authors could comment on this observation - whether this is spurious or a potential prophylactic action of the drug.
Thank you for the suggestion. While not statistically significantly different from the other NSAIDs. It is still worth mentioning. We have added this to the discussion.

8. With the in-depth description in Methods & Supplementary sections, the authors might consider including the final models in the main Methods section to make the both the analysis and results easier to interpret.
The tables reported are the final models with coefficients and p-values. This is stated in the title of the tables. We have now included a model equation in the methods to make this easier for the reader.

9. There are minor typos in Supplement section - particularly 'explanatory, diagnostic, corresponding, reliever', etc.
Thank you for this. We have gone through the supplement and address these typos.

10. It would be good to give meaningful titles / very brief legends to the figures in

Supplement section.

Thank you for this suggestion. We have gone through the supplement and improved the titles of each section to ensure readability and added figure descriptions.
