# Supplementary material for: Anti-inflammatories in Alzheimer’s disease—potential therapy or spurious correlate?
Source: Brain Commun. 2020 Jul 24;2(2):fcaa109. doi: 10.1093/braincomms/fcaa109 (PMC7585697; doi:10.1093/braincomms/fcaa109)
Supplement: fcaa109_Supplementary_Data [file Revision 1 Decision Letter.pdf › Revision 1 Decision Letter.pdf]

Revision 1 Decision Letter

23-Jun-2020

Dear Dr. Rivers-Auty,

It is a pleasure to accept your manuscript entitled "Anti-inflammatories in Alzheimer’s disease - potential therapy or spurious correlate?" for publication in Brain Communications. Any comments made by the reviewer(s) are included below.

The next step is for your paper to go through “First Look”. We will contact you again shortly if any final files or formatting changes are required for publication or if we have any queries.

If no changes are needed, your submission will be transferred to our Production Department and you will hear from the Production Editor in due course.

Thank you very much for your contribution. On behalf of the Editors of Brain Communications, we look forward to your future contributions to the Journal.

Yours sincerely,

Professor Karen Ritchie
Associate Editor
Brain Communications

Reviewer(s)' Comments to Author:

Reviewer: 2 Jie Yeap

Comments to the Author
The revision provides additional information which improves the clarity of the paper. The figures in Supplementary are nicely presented too.

Reviewer: 1

Comments to the Author
Substantial changes made in response the reviews have improved the manuscript greatly.
